# Traditional Herbal Medicine in Mesoamerica: Toward Its Evidence Base for Improving Universal Health Coverage

**DOI:** 10.3389/fphar.2020.01160

**Published:** 2020-07-31

**Authors:** Matthias S. Geck, Sol Cristians, Mónica Berger-González, Laura Casu, Michael Heinrich, Marco Leonti

**Affiliations:** ^1^ Department of Biomedical Sciences, University of Cagliari, Cagliari, Italy; ^2^ Biovision – Foundation for Ecological Development, Zurich, Switzerland; ^3^ Botanical Garden, Institute of Biology, Universidad Nacional Autónoma de México, Mexico City, Mexico; ^4^ Centro de Estudios en Salud, Universidad del Valle de Guatemala, Guatemala, Guatemala; ^5^ Department of Epidemiology and Public Heath, Swiss TPH, University of Basel, Basel, Switzerland; ^6^ Department of Life and Environmental Sciences, University of Cagliari, Cagliari, Italy; ^7^ Pharmacognosy and Phytotherapy, UCL School of Pharmacy, London, United Kingdom

**Keywords:** Mesoamerican traditional medicine, evidence-based phytotherapy, universal health coverage, medicinal plants, Mexico, Central America

## Abstract

The quality of health care in Mesoamerica is influenced by its rich cultural diversity and characterized by social inequalities. Especially indigenous and rural communities confront diverse barriers to accessing formal health services, leading to often conflicting plurimedical systems. Fostering integrative medicine is a fundamental pillar for achieving universal health coverage (UHC) for marginalized populations. Recent developments toward health sovereignty in the region are concerned with assessing the role of traditional medicines, and particularly herbal medicines, to foster accessible and culturally pertinent healthcare provision models. In Mesoamerica, as in most regions of the world, a wealth of information on traditional and complementary medicine has been recorded. Yet these data are often scattered, making it difficult for policy makers to regulate and integrate traditionally used botanical products into primary health care. This critical review is based on a quantitative analysis of 28 survey papers focusing on the traditional use of botanical drugs in Mesoamerica used for the compilation of the “Mesoamerican Medicinal Plant Database” (MAMPDB), which includes a total of 12,537 use-records for 2188 plant taxa. Our approach presents a fundamental step toward UHC by presenting a pharmacological and toxicological review of the cross-culturally salient plant taxa and associated botanical drugs used in traditional medicine in Mesoamerica. Especially for native herbal drugs, data about safety and effectiveness are limited. Commonly used cross-culturally salient botanical drugs, which are considered safe but for which data on effectiveness is lacking constitute ideal candidates for treatment outcome studies.

## Introduction

Access to adequate medical care is a basic human right (Article 25, Universal Declaration of Human Rights) and universal health coverage (UHC) is core to achieving Sustainable Development Goal three (SDG 3) of the UN Agenda 2030 (UN General Assembly, 1948; UN General Assembly, 2015). The World Health Organization (WHO, 2013) highlighted the need for integrating traditional and complementary medicine (T&CM) in national health systems in order to achieve UHC while respecting consumers’ choice. A comprehensive knowledge base is fundamental for establishing policies that allow people to “access T&CM in a safe, respectful, cost-efficient and effective manner” (WHO, 2013, p. 7). Lack of research data is seen as the number one challenge faced by member states for implementing the WHO’s T&CM strategy. The lack of systematic reviews of the available evidence on T&CM in Mesoamerica is reflected in insufficient policies and culturally sensitive health materials, representing critical barriers to care (Lozoya and Zolla, 1984; Nigenda et al., 2001; WHO, 2005; Caceres Guido et al., 2015; Mokdad et al., 2015). Recent emphases shifts in public health discussions stemming from debates around ‘Epistemologies of the South’ (De Sousa, 2011) propose that the route toward UHC in Mesoamerica is dependent on promoting “health sovereignty,” fostering a decolonial turn in favor of intercultural approaches that reflect the particular epidemiologies of the peoples (Basile, 2018). According to De Sousa (2010) and Laurell (2010) the neglected consideration of emic epistemologies in the shaping of public health policies should be contrasted with a turn toward an “ecology of knowledge-systems.” This perspective includes traditional medicine, particularly herbal medicine, which has been recognized as playing a key role toward providing culturally pertinent and accessible health coverage (Rocha-Buelvas, 2017) and is in line with the WHO’s guidelines, which pin-point acceptability as a factor fostering increased access to health provision services in diverse cultural settings (WHO, 2013).

**Figure 1:**
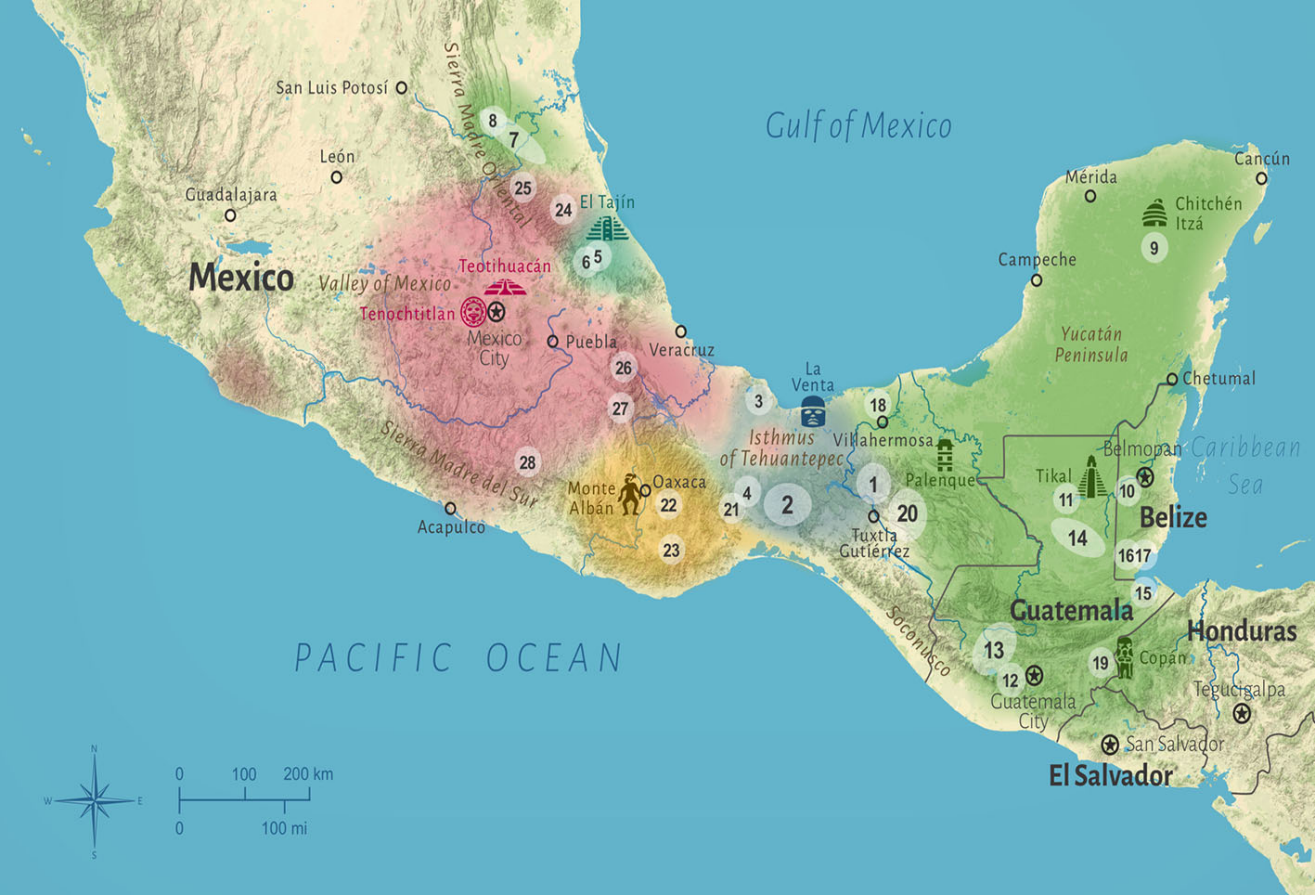
Map of Mesoamerica (MA). The colored areas mark the geographic extent of the major linguistic groups: Mayan (green) in eastern MA and the Huasteca, Mixe-Zoque (blue) in the Isthmus of Tehuantepec region, Nahuatl (red) in central and western MA, Totonac (turquoise) in Veracruz, and Zapotec (yellow) in Oaxaca. The numbers 1–28 refer to the study codes in **Table 1**; the extents of the respective study sites are highlighted in white.

Mesoamerica (from now on ‘MA’) is a term coined by Kirchoff (1943) and accepted by scholars to define a geographical region (**Figure 1**) inhabited by indigenous peoples that share several common cultural traits resulting from intense cultural interchange starting in the Early Preclassic period (Coe and Koontz, 2013). The advent of civilization in MA can be placed in the early second millennium BCE when the San Lorenzo Olmecs emerged in the region of today Veracruz (Mexico) and the Mokaya as the first socially stratified sedentary culture in the Soconusco region of Mexico and Guatemala on the Pacific coast (Clark, 1991, p. 13-26; Diehl, 2004, p. 129; Coe and Koontz, 2013). The common fundamental traits characterize the cultural area of MA and distinguish it from the rest of the Americas (Coe and Koontz, 2013, p. 9-10; Kirchhoff, 1943). Ranging from central Mexico to northern Central America, MA was home to several of the great civilizations of the Western Hemisphere, including the Olmecs, Maya, and Aztecs. Aztec medicine represented the culmination of a long cultural tradition uniting the different cultural groups of MA (Ortiz de Montellano, 1990). After the Conquest, the different health systems rapidly blended into a syncretic amalgamation combining indigenous and introduced elements (e.g., Ortiz de Montellano, 1975; Lozoya and Zolla, 1984; Ortiz de Montellano, 1990; Foster, 1994; Bye et al., 1995). Mirroring its outstanding cultural heritage, MA is also one of the world’s most biodiverse regions and among the most eminent centers of plant domestication (Vavilov, 1992; Smith, 1997; Myers et al., 2000; Ranere et al., 2009).

### Epidemiology, Health Systems, and Integrative Medicine in MA

Mexico and Central America are undergoing a rapid health transition resulting in a “double burden of disease” as poverty related diseases coexist with modern lifestyle diseases (Frenk, 2006; Stevens et al., 2008; Puig et al., 2009; Gómez-Dantés et al., 2016).

As a result of a higher life expectancy, after the 1940s Mexico experienced a great demographic expansion going hand in hand with economic growth (Cabrera, 1994). Toward the end of the 1960s, however, concerns were raised about the economic sustainability of the soaring population (Cabrera, 1994) and in 1973 a new population policy was implemented (Alba and Potter, 1986). The Mexican Ministry of Health and Welfare (SSA) together with the Institute of Social Security (Instituto Mexicano del Seguro Social; IMSS) started to provide family planning counseling and contraceptive services free of charge through their national networks in 1973 (Alba and Potter, 1986). During the 1970s and until 1982 the political administrations expanded the Mexican health sector by increasing the number of hospitals, clinics and medical staff. This initiative included the establishment of over 3,000 rural health posts, 71 rural hospitals and trainee programs for community health workers including traditional midwifes. As a consequence, the family planning services reached communities and individuals that were not formally considered by the social security system (Alba and Potter, 1986). These measures resulted in a reduced population growth that was more accentuated in urban compared to rural areas and overall reduced poverty (Cabrera, 1994; Allen, 2007). In 1983 the IMSS started to monitor systematically the epidemiology in the marginalized rural communities. This data was used as a baseline for launching programs aimed at reducing morbidity and mortality culminating in a decrease of digestive infections, less malnutrition and better assistance during gestation, birth and postpartum period (Flores Alvarado and Morán Zenteno, 1989). In Guatemala in particular and Central America in general, emerging civil armed conflicts at the end of the 1960s, lasting into the 1990s, prevented health programs to comprehensively address the needs and reach out to the rural population, aggravating health disparities (Braveman et al., 2000; Flores et al., 2009).

Today, reduced burdens of infectious diseases are partially offset by the need for health care assistance caused by interpersonal violence and chronic illnesses (Stevens et al., 2008; PAHO, 2009; Acosta et al., 2011; Becerril-Montekio and López-Dávila, 2011; Bermúdez-Madriz et al., 2011; Gómez-Dantés et al., 2011a; Gómez-Dantés et al., 2011b; Gómez-Dantés et al., 2016). Despite promising effects of recent health system reforms, pronounced regional disparities in regard to health indicators within and between the countries of MA persist (Frenk, 2006; Stevens et al., 2008; Mokdad et al., 2015; Gómez-Dantés et al., 2016). Health inequities imply that infectious diseases – particularly diarrheal disorders and infections of the lower respiratory tract – and reproductive health still account for a considerable disease burden, particularly among marginalized, often indigenous, populations. Meanwhile, depressive and chronic diseases are becoming major health concerns (Frenk, 2006; Stevens et al., 2008; Mokdad et al., 2015; Gómez-Dantés et al., 2016). Especially type II diabetes mellitus and chronic kidney disease of unknown cause (CKDu), now also called epidemic of chronic kidney disease of nontraditional origin (CKDnt) pose increasing challenges to the health systems in the region (Barcelo et al., 2003; Barcelo et al., 2012; Kierans et al., 2013; Gómez-Dantés et al., 2016; Johnson et al., 2019; Wesseling et al., 2020). The endemic form of CKDu occurring in MA was previously called Mesoamerican nephropathy (MeN) and affects above all young male workers of the agricultural sector (Wesseling et al., 2013; Wijkström et al., 2013). The origin of CKDu (also MeN and CKDnt) seems to be primarily driven by occupational heat stress linked to dehydration (Wijkström et al., 2013; Wesseling et al., 2020).

With the partial exception of Belize, the national health systems throughout MA have a similar structure and suffer from considerable degrees of fragmentation and segmentation (Homedes and Ugalde, 2009; Puig et al., 2009; Acosta et al., 2011; Becerril-Montekio and López-Dávila, 2011; Bermúdez-Madriz et al., 2011; Gómez-Dantés et al., 2011a; Kierans et al., 2013; Mokdad et al., 2015). Typically, public sectors in MA countries are composed of the respective ministries of health, social security institutes, and up to seven additional service providers. While formal employees benefit from social security, the majority of the populations rely on the – at least theoretically – free healthcare provided by the ministries of health. Notwithstanding the constitutional guarantee of affordable healthcare to all, a considerable proportion of the population of each country has no de facto access to healthcare provision from the public sector; a deficit partially compensated for by a multitude of civil society organizations operating in the most marginalized areas, but perhaps more importantly, by traditional healers embedded in long-standing ethnomedical systems.

In Guatemala it is estimated that public investments cover only 40% of the costs for accessing healthcare services, and that most of these funds are centralized in urban and peri-urban areas (World Bank, 2016; Gomez et al., 2017). This exemplifies how marginalized communities in rural settings have to rely on a plurimedical system, where practitioners of traditional Maya medicine play a key role in providing affordable services (Ceron, 2010; Berger-González et al., 2016b; CMMM, 2016). Comprehension of this situation prompted the development of the Model for Inclusive Health in 2004 (Fort et al., 2011). This model implements the parallel coordination of patients between specialists in traditional medicine such as *ajkum* (~ herbalists), *ajiyom* (~ midwives), *ajq’omaneel* (~ physicians) and biomedical staff in the first and second levels of attention those working at community health posts and district health centers. The Inclusive Health Model required the implementation of new protocols of attention including ‘cultural syndromes’ such as *susto* (fright) *ojeado* (evil eye) or *wuqub’ siwan* (disease of the seven ravines; Taquira et al., 2016), and an understanding of associated botanical drug based therapies, so that the medical staff could be trained in coordinating safe patient care with Maya health specialists. For example, in the Cuilco health district alone, located in the western highlands of Guatemala, 360 traditional medicine practitioners coordinated interventions with 78 medical health staff (ISIS, 2019). The Inclusive Health Model was strongly promoted by the Ministry of Public Health between 2016 and 2017, which led to the inclusion of 40 plants into the “Norms for the Attention of the First and Second Levels” of the Ministry of Health (MSPAS, 2018, p. 835-863). In spite of this initial effort, the lack of evidence about the safety and efficacy of medicinal plants and associated botanical drugs employed by practitioners of traditional medicine are a limiting factor in the translation of these intercultural protocols.

Finally, a growing for-profit private sector offers care to the urban socio-economic elites. Each of these service providers has its own infrastructure and, despite recent efforts to harmonize service provision, the coordination between different health institutions is limited. Within the respective national health systems, the Ministry of Public Health plays the stewardship role, including the formulation of T&CM policies as well as integrating and regulating T&CM products and practitioners in the formal health systems (WHO, 2005; Homedes and Ugalde, 2009; Puig et al., 2009; Acosta et al., 2011; Becerril-Montekio and López-Dávila, 2011; Bermúdez-Madriz et al., 2011; Gómez-Dantés et al., 2011a; Kierans et al., 2013; Mokdad et al., 2015). Inefficiency in the public sector as well as the lack of cultural competency results in unsatisfactory perceived quality of care (Puig et al., 2009; Mokdad et al., 2015). In Mexico, the health-care delivery clinics in rural areas are run by the IMSS and the Mexican Ministry of Health and Welfare (SSA). Usually they are staffed with recent medical graduates who spend their obligatory year of postgraduate social service as well as with community health workers. This situation is, however, not the best basis for achieving quality, consistency, cultural sensitivity, and ultimately, patients’ confidence (Berlin and Berlin, 1996, p. 6) and is one of the reasons why traditional medicine has retained its important role in rural areas. For marginalized people, experiencing excessive financial, physical, or cultural barriers to care, traditional medicine often presents the only accessible healthcare option. Meanwhile herbal products provide popular treatment alternatives for urban socioeconomic elites and Latin American migrant communities (Lozoya and Zolla, 1984; Taddei-Bringas et al., 1999; Waldstein, 2006; Loera et al., 2007; WHO, 2013; Ladas et al., 2014; Alonso-Castro et al., 2017a). However, integrative efforts and official recognition of Mesoamerican Traditional Medicine are limited. Several countries lack national policies and programs on T&CM and Mexico is the only country in MA with a national pharmacopeia (WHO, 2005; Caceres Guido, 2015). Yet, from the 129 herbal drugs listed in the Mexican Herbal Pharmacopoeia only around 36 are native (FEUM, 2013). With respect to Guatemala, the National Vademecum on Medicinal Plants (Cáceres, 2009) containing validated information on 101 herbal drugs of which 42 are native, got the endorsement of the University of San Carlos and the Ministry of Public Health and was later adopted as a reference by the Central American Technical Regulation.

The emphasis on introduced taxa in formal phytotherapy in Latin America is due to an often lacking evidence base for native botanical drugs (Caceres Guido et al., 2015; Valdivia-Correa et al., 2016; Alonso-Castro et al., 2017b) and the relative good documentation of effectiveness and safety issues of herbal drugs present in the European and the US Pharmacopoeia (Lozoya and Zolla, 1984; Caceres Guido et al., 2015; Martins et al., 2019).

Medical concepts and health beliefs regarding disease etiology, diagnosis, and treatment show striking similarities throughout MA, notwithstanding the uniqueness of each cultural group’s ethnomedical system and individual case to case variations (Lozoya and Zolla, 1984; Weller et al., 2002; Groark, 2005; Kleinman and Benson, 2006; Balick et al., 2008; Berger-González et al., 2016a; Geck et al., 2017). Efforts to integrating traditional practitioners and practices into the formal health system have been met with limited success [but see Chary et al. (2018) and Hitziger et al. (2017)], partially due to a limited understanding of ethnomedical concepts and rural medicine as well as an *a priori* disesteem toward traditional medicine by formal health institutions and physicians (Lozoya and Zolla, 1984; Nigenda et al., 2001; Bye and Linares, 2015; Colon-Gonzalez et al., 2015). Formal health professionals in Mexico regularly prescribe and use herbal products yet lament the lack of T&CM-specific education and training material (Taddei-Bringas et al., 1999; Romero-Cerecero and Tortoriello-García, 2007; Alonso-Castro et al., 2017a). Meanwhile traditional healers rarely find successors and acculturation is changing patterns in transmission of traditional and local knowledge (Comerford, 1996; Balick et al., 2008; García-Hernández et al., 2015; Geck et al., 2016). Consequently, written sources of knowledge as well as popular media are increasingly shaping the medical systems of local and indigenous communities (Leonti, 2011; Geck et al., 2016).

### Previous Cross-Cultural Comparisons and Compilations of Mesoamerican Herbal Medicine

Several national and regional compilations of medicinal plants exist in Mexico, covering over 3,000 botanical taxa (Argueta and Zolla, 1994; Bye et al., 1995). Unfortunately, these often demonstrate serious methodological deficits, particularly in regard to taxonomic identification and interpretation of ethnomedical data (Bye and Linares, 2015, p. 396-397). Further, the lacking quantification of traditional uses limit the utility of these compilations for the identification of cross-culturally salient taxa.

The first over-regional research program in the area aimed at the evaluation of the traditional use of botanical drugs in order to improve the quality of health care of marginalized populations was Tramil (Program of Applied Research to Popular Medicine in the Caribbean; Weniger, 1991; Boulogne et al., 2011). Tramil’s exclusive focus on the Caribbean implied that only very minor parts of MA were covered (Boulogne et al., 2011). Several studies compared the medicinal floras and ethnomedical concepts of closely related cultural groups within the same linguistic family (e.g., Berlin and Berlin, 1996; Leonti et al., 2003; Geck et al., 2016; Hitziger et al., 2016). Heinrich et al. (1998; 2014) conducted the most comprehensive cross-cultural analyses to date yet focused exclusively on gastrointestinal ailments. Additionally, several reviews exist on the treatment of emerging health concerns, specifically anxiety and depression, colorectal cancer, diabetes, and obesity (Alonso-Castro et al., 2015a; Cruz and Andrade-Cetto, 2015; Andrade-Cetto and Heinrich, 2016; Giovannini et al., 2016; Jacobo-Herrera et al., 2016; López-Rubalcava and Estrada-Camarena, 2016).

### Objectives of This Analysis

T&CM contributes significantly to the health coverage of the population of MA, particularly in poor and underserved indigenous communities (WHO, 2013). Similarly, to the situation in MA, in most regions of the world, a wealth of information on T&CM has been recorded. Yet these data are often scattered, making it difficult for policy makers to regulate and integrate herbal products into primary health care. Despite over 400 million estimated regular users of T&CM in Latin America, systematic approaches to integrate T&CM in formal health systems are widely lacking (Caceres Guido et al., 2015). Given the shared cultural history, harmonizing regulations between different nations of MA is recommended (WHO, 2013, p. 41). A lack of pharmacological and toxicological data on even the most commonly used herbal drugs is often considered the principal limitation to integrative medicine in MA (Caceres Guido et al., 2015; Alonso-Castro et al., 2017a). National and international efforts have been conducted in Guatemala in order to establish integrative medicine at academic and public health levels and although official acceptance is limited, national interest and expectations are high (Cáceres, 2019). Further, there is a strong need to integrate T&CM into formal health education (Romero-Cerecero and Tortoriello-García, 2007; Alonso-Castro et al., 2017a). Hence, creating quantitative regional databases based on internationally published literature can be an effective means for advancing the integration of evidence-based T&CM and therefore contribute to achieving UHC.

In accordance with the strategic objectives outlined in the Traditional Medicine Strategy of the World Health Organization (WHO, 2013), we aim at establishing a consensus-driven knowledge base on herbal drugs used in Mesoamerican traditional medicine. The focus is on plants used as medicine by traditional healers in rural indigenous communities. The quantitative nature of the review will allow for the prioritization of taxa for pharmacological and clinical studies. The pharmacological evidence for the safety and efficacy of the cross-culturally most salient taxa is reviewed and important knowledge gaps are indicated. The review is intended as a baseline of evidence for regulators, health professionals, and consumers for making informed decisions on herbal drugs and phytomedicines. Hence, this review and the MAMPDB is seen as an essential first step for an improved integration of traditional medicine into the national health systems of Mexico and Central America.

## Methods

The linguistic scope of this cross-cultural comparison is limited to the five groups that can be most closely linked to the cultural evolution of MA: Maya, Mixe-Zoque, Nahuatl, Totonac, and Zapotec (Kirchhoff, 1943; Campbell et al., 1986). Likewise, the geographic scope is limited to MA proper, excluding the frontier areas of northern Mexico and Central America, as these only temporally participated in MA (Coe and Houston, 2015: 13; Kirchhoff, 1943; **Figure 1**).

Published ethnobotanical and ethnopharmacological field studies related to the five linguistic groups were sought that met the minimum inclusion criteria of methodological transparency regarding data sampling, study location, population and taxonomic identification based on voucher specimens collected *in situ*. A comprehensive search on the online databases Medline (PubMed) and Scopus as well as the Swiss library network (swissbib) and the dissertations database ProQuest was conducted with the following search terms: ethnobotany OR ethnopharmacology OR “traditional medicine” OR “medicinal plants” OR “herbal medicine” AND Mesoamerica OR Mexico OR “Central America” Guatemala OR Belize OR “El Salvador” OR Honduras OR Maya OR Mixe OR Zoque OR Nahua OR Nahuatl OR Aztec OR Totonac OR Zapotec. Thus, 28 studies published between 1975 and 2016 were identified that met the geographic, linguistic, and methodological inclusion criteria to be considered by the MAMPDB (**Table 1**)^1^. Names and classification of linguistic groups follow glottolog 2.7 (Hammarström et al., 2016).

**Table 1:** Studies included in the Mesoamerican Medicinal Plant Database (MAMPDB).

Linguistic group (study codes)	Reference	Study site	Number of taxa (n = 2188)	Number of use-records (n = 12,537)	Notes
Mixe-Zoque (1-4)			974	3,900	
Zoque (1-3)			922	3,594	
Chiapas Zoque (1)	Geck et al., 2016	Mexico, Chiapas	411	1,200	
Chimalapa Zoque (2)	Geck et al., 2016	Mexico, Oaxaca	261	825	High degree of acculturation
Highland Popoluca (3)	Leonti et al., 2001; Leonti et al., 2013	Mexico, Veracruz	595	1,569	
Mixe					
Lowland Mixe (4)	Heinrich, 1989	Mexico, Oaxaca	208	306	In German
Totonacan (5-6)			193	504	
Totonac (5)	Martinez-Alfaro, 1984	Mexico, Puebla	100	223	Strong Nahua influences
Totonac (6)	Ugarte, 1997	Mexico, Puebla	128	281	Food-medicine continuum
Mayan (7-20)			1,418	5,824	
Huastecan (7-8)			539	1,508	
Huastec (7)	Alcorn, 1984	Mexico, San Luis Potosí/Veracruz	498	1,254	General ethnobotany
Huastec (8)	Alonso-Castro et al., 2012a	Mexico, San Luis Potosí	72	254	
Core Mayan (9-20)			1,142	4,316	
Yucatecan (9-11)			386	685	
Yucatec Maya (9)	Ankli et al., 1999; Ankli, 2000	Mexico, Yucatán	321	446	
Yucatec Maya (10)	Arnason et al., 1980	Belize, Cayo District	73	101	
Itzá (11)	Comerford, 1996	Guatemala, Petén	80	138	Only two informants
Quichean-Mamean (12-17)			771	2,727	
Kaqchikel (12)	Hitziger et al., 2016	Guatemalan highlands	335	1,371	Only four informants
K’iche’ (13)	Nicolas, 1997; Nicolas, 1999	Guatemalan highlands	214	586	In French
Kekchí (14)	Hitziger et al., 2016	Guatemala, Petén	296	623	Only five informants
Kekchí (15)	Michel et al., 2007; Michel et al., 2016	Guatemala, Izabal	31	52	Women’s health
Kekchí (16)	Bourbonnais-Spear, 2005; Bourbonnais-Spear et al., 2005	Belize, Toledo District	61	95	Mental and neurological
Kekchí (17)	Treyvaud-Amiguet et al., 2005	Belize, Toledo District	106	0	No uses disclosed
Western Mayan (18-20)			315	904	
Tabasco Chontal (18)	Magaña-Alejandro et al., 2010	Mexico, Tabasco	170	440	In Spanish
Chortí (19)	Kufer et al., 2005	Guatemala, Chiquimula	114	263	Diachronic comparison
Tzeltalan (20)	Berlin and Berlin, 1996	Mexico, Chiapas	100	201	Gastrointestinal ailments
Otomanguean					
Zapotec (21-23)			531	1,490	
Petapa Zapotec (21)	Frei, 1997; Frei et al., 1998	Mexico, Oaxaca	337	920	
Mitla Zapotec (22)	Messer, 1975	Mexico, Oaxaca	92	187	General ethnobotany
Miahuatlan Zapotec (23)	Hunn, 2008	Mexico, Oaxaca	210	383	General ethnobotany
Uto-Aztecan					
Eastern Nahuatl (24-28)			321	820	
Eastern Huasteca Nahuatl (24)	Smith-Oka, 2008	Mexico, Veracruz	27	36	Women’s health
Central Huasteca Nahuatl (25)	Andrade-Cetto, 2009	Mexico, Hidalgo	95	215	Few indigenous names
Zongolica Nahuatl (26)	Weimann and Heinrich, 1997; Weimann, 2000	Mexico, Veracruz	180	320	In German
Highland Puebla Nahuatl (27)	Canales et al., 2005	Mexico, Puebla	46	101	No indigenous names
Guerrero Nahuatl (28)	Juárez-Vázquez et al., 2013a	Mexico, Guerrero	66	148	

Names and classification of linguistic groups follow glottolog 2.7.

All plant taxa with medicinal uses mentioned in the 28 studies were incorporated into the MAMPDB after verifying their taxonomic identity with www.theplantlist.org (accessed 06.06.2016). Family affiliations of angiosperms follow the more up-to-date APG IV (The Angiosperm Phylogeny Group, 2016). In case several members of the same genus are used interchangeably under the same vernacular name for the same purpose, the taxon is denoted *Genus* sp. Infraspecific taxa are not specified.

The medicinal uses were classified according to the second edition of the International Classification of Primary Care (ICPC; **Table 2**). The ICPC allows for classification of ethnomedical uses into 17 symptom-based categories, not requiring detailed diagnostics (WICC, 2004; Staub et al., 2015). The only modification made to the ICPC system refers to toothache, which was classified as a neurological rather than a digestive system disorder.

**Table 2:** Overview of the distribution of taxa and use-records of the Mesoamerican Medicinal Plant Database in the 17 International Classification of Primary Care (ICPC) categories.

ICPC categories	No. of taxa	No. of use-recs.	Apparently safe and efficacious applications*	Native spp.*
**A:** General and unspecified	1,103	2,042	15	6
**B:** Blood, blood forming organs and immune mechanism	153	178	–	–
**D:** Digestive	1,082	2,180	40	22
**F:** Eye	154	196	–	–
**H:** Ear	105	130	–	–
**K:** Cardiovascular	249	312	–	–
**L:** Musculoskeletal	558	908	3	2
**N:** Neurological	553	800	2	1
**P:** Psychological	369	509	2	0
**R:** Respiratory	527	978	13	3
**S:** Skin	1,090	1,862	22	17
**T:** Endocrine/metabolic and nutritional	257	358	3	2
**U:** Urological	404	573	2	1
**W:** Pregnancy, childbearing, family planning	443	677	4	2
**X:** Female genital	494	724	1	1
**Y:** Male genital	94	103	–	–
**Z:** Social problems	7	7	–	–
**Total**	**2,188**	**12,537**	**107**	**57**

*Evaluation based on the 98 species (68 spp. are native and 30 spp. are exotic) assessed in **Table 3**.

For the species cited in at least nine studies (one third taken as an arbitrary threshold value) a comprehensive literature review of pharmacological data was conducted based on a literature search with the online databases Medline (PubMed), Scopus, and the Cochrane Library (**Table 3**). Preclinical and clinical data obtained with botanical drugs derived from the 98 medicinal plant species cited in at least nine studies are reported in correspondence to the predominant traditional uses (use-records). As an arbitrary threshold value predominant uses are defined here as those recorded in the same ICPC category by at least seven independent studies or alternatively, those most frequently recorded in an ICPC category in case no category was recorded in at least seven studies. A use-record is defined as a reported use per taxon and ICPC category in one study. Studies lacking methodological transparency or using doses unrealistically high from a therapeutic perspective were excluded from this review. We evaluated the available pharmacological and preclinical data in order to extrapolate on the safety and efficacy of clinical applications. We took the mode of application into account and considered the importance of cultural factors for the perceived effectiveness, which is to be distinguished from efficacy (Ortiz de Montellano, 1975; Last et al., 2001, p. 57-58; Moerman and Jonas, 2002; Witt, 2013). Therefore, we evaluated as potentially safe and effective also applications of herbal drugs for which no negative toxicological reports were available. Despite the human influence on the current distribution of plant taxa is not always exactly known we categorized the 98 species into natives and exotics, judging those species native which, due to their cultural importance, obtained a wide distribution range over South and MA prior to European conquest and colonization (e.g., *Bixa orellana* and *Petiveria alliacea*) as well as the pantropic species (e.g., *Cissampelos pareira* and *Cocos nucifera*).

**Table 3:** The cross-culturally most salient medicinal plant species in Mesoamerica (MA).

Taxon (Family) – English vernacular name – common names in MA	# of studies citing (# use-records)	Predominant uses (# of studies) and brief description	Pharmacological evidence on possible therapeutic benefits related to predominant uses	Toxicological evidence on the safety of traditional uses	Evaluation
***Dysphania ambrosioides*** (L.) Mosyakin & Clemants (syn.: *Chenopodium ambrosioides* L.); Amaranthaceae) – wormseed – *epazote* Native1	23 (62)	Digestive (22), particularly as anthelmintic; skin (10); Infusion of aromatic aerial parts used throughout MA against gastrointestinal parasites; added to bean dishes as a condiment. Aerial parts are topically applied to treat skin infections.	Antiascariasis: observational study with 60 children aged 3–14 with diagnosed ascariasis; 30 children received plant juice (1–2 ml/kg/d for 3 days) and 30 children received the standard anthelmintic drug albendazole (200-400 mg single dose). The qualitative efficacy, as measured in complete eradication of eggs in fecal samples, was similar in both groups (59.5% vs. 58.3%; López de Guimaraes et al., 2001). Nematocidal activity demonstrated in several preclinical studies (MacDonald et al., 2004). Anti-*Helicobacter pylori*: *in vitro* MIC of 16 μg/ml essential oil; 60% eradication of *H*. *pylori* in mice receiving essential oil (49.32 mg/kg/d PO for 28 days) was below the 70% eradication in mice receiving triple antibiotic treatment (lansoprazole, metronidazole, and clarithromycin) (Ye et al., 2015). Antileishmaniasis: prevention of lesion development in mice infected with *Leishmania amazonensis* receiving essential oil (30 mg/kg ID every 4 days for 14 days) was statistically superior to treatment with the standard leishmaniasis drug glucantime (28 mg/kg ID every 4 days for 14 days) (Monzote et al., 2014).	While infusions appear to be safe, toxicological issues persist in regard to the internal use of the essential oil, due to high concentrations of the terpene ascaridole (MacDonald et al., 2004). No significant signs of acute (0.3-3 mg/kg, IG) or subchronic (0.3-1 mg/kg/d, PO, 15 days) toxicity from aqueous leaf extracts in rats (da Silva et al., 2014); nor for subchronic (5–500 mg/kg/d PO for 15 days) toxicity from hydroalcoholic leaf extracts in mice was noted (Pereira et al., 2010).	Good evidence on therapeutic benefits and safe with reservations D1
***Psidium guajava*** L. (Myrtaceae) – guava – *guayaba* Native2	23 (66)	Digestive (23), particularly as antidiarrheal; skin (12); Infusion of astringent leaves used throughout MA against diarrheal disorders and maceration topically for inflammatory skin conditions; common fruit tree.	Antidiarrheal and antispasmodic: randomized, double blind, placebo-controlled clinical trial with 100 adult patients (61 f, 39 m), aged 20-59, diagnosed with acute diarrheic disease; treatment group (n = 50) received one capsule containing 500 mg leaf preparation standardized for concentration of the flavonoid quercetin (1 mg/capsule) every 8 h for 3 days; while there was no significant difference in five outcome measures (number of defecation/d, consistency of stool, presence of mucus in stool, presence of fever, episodes of vomiting), the remaining 2 (degree of abdominal pain, number of spasms/d) were significantly improved in the treatment group in comparison to placebo (Lozoya et al., 2002). ntidiarrheal and antirotavirus: observational study with 62 children aged 2–18 diagnosed with infantile rotaviral enteritis; improvement in all four outcome measures (recovery rate after 3 days, time of ceasing diarrhea, content of Na^+^ and glucose in stool, rate of negative conversion of human rotavirus antigen) was significantly superior in the treatment group (n=31) receiving aqueous leaf extract (10–20 g every 12 h for 3 days) in comparison to the control group (n = 31) receiving the Chinese compound drug *gegen qinlian* (33* g* every 12 h for 3 days; Wei et al., 2000). Antidiarrheal, spasmolytic, antiinflammatory, broad antibacterial (incl. gastrointestinal as well as dermatological pathogens), fungicidal, and antirotavirus activity of aqueous and alcoholic leaf and bark extracts at therapeutically realistic doses demonstrated in a number of *in vitro* and *in vivo* models, as extensively reviewed (Gutiérrez et al., 2008; Morais-Braga et al., 2016).	No adverse effects in clinical trial administering 500 mg leaves every 8 h for 3 days to 50 adult patients (Lozoya et al., 2002). Several *in vitro* and *in vivo* studies demonstrated no acute or subchronic toxicity at therapeutic doses, as extensively reviewed (Gutiérrez et al., 2008; Morais-Braga et al., 2016).	Good evidence on therapeutic benefits and safety D2, S1
***Persea americana*** Mill. (Lauraceae) – avocado – *aguacate* Native3	21 (88)	Digestive (13); respiratory (10); skin (10); musculoskeletal (8); pregnancy (8); Leaves, bark, and seeds common for diverse therapeutic uses throughout MA, apart from its culinary uses. For gastrointestinal and respiratory uses oral administration is most frequent, whereas bathes and topic applications predominate for dermatological, musculoskeletal, pregnancy-related disorders.	Antidiarrheal: significant, dose-dependent reduction in all 3 measures (water content in feces, frequency of defecation, enteropooling) of castor oil-induced diarrhea in rats after treatment with either chloroform or methanol leaf extract (100–200 mg/kg PO) (Christian et al., 2014). Anti-*Helicobacter pylori*: *in vitro* MIC of 7.5 μg/ml methanolic leaf extract (Castillo-Juárez et al., 2009). Antidermatophytosis: *in vitro* MIC of 31.3 μg/ml ethanolic leaf extract against *Trichophyton* spp. (Biasi-Garbin et al., 2016). Broad antimicrobial (incl. gastrointestinal, respiratory, dermatological, and gynecological pathogens): significant activity of chloroformic and ethanolic seed extracts (Jiménez-Arellanes et al., 2013), aqueous and ethanolic seed extracts (Raymond Chia and Dykes, 2010), aqueous and butanolic stem bark extract (Akinpelu et al., 2015), glycolic leaf extract (Jesus et al., 2015). Nutraceutical properties in relation to maternal health (Comerford et al, 2016), osteoarthritis (Christensen et al., 2008), and wound healing (Nayak et al., 2008).	No signs of acute toxicity in rats at therapeutic doses (10–2,600 mg/kg PO) of chloroform-methanol leaf extract (Christian et al., 2014). No signs of subchronic toxicity in rats at therapeutic doses (2.5–10 mg/kg/d PO for 28 days) of aqueous seed extract (Ozolua et al., 2009).	Safe but limited evidence for efficacy of external uses D3, S2
***Ricinuscommunis*** L. (Euphorbiaceae) – castor oil plant – *higuerilla* Exotic1	21 (65)	Digestive (17); general and unspecified (15); musculoskeletal (7); Introduced species, seeds (rather than castor oil) used as emetic and purgative, leaves topically against pain (mostly stomachache, rheumatic pain, and general body pain).	Purgative: there is little doubt on the efficacy of castor oil for colon cleansing, yet there are more effective alternatives with less side effects (e.g., Chen et al., 1999). Analgesic: methanolic leaf extracts (100–150 mg/kg IP) significantly reduced nociception in mice in the formalin, tail flick, and writhing tests similar to diclofenac (Taur et al., 2011). Antiosteoarthritis: randomized, double blind, comparative clinical trial with 100 adult patients (68 f, 32 m), aged 40–90, diagnosed with knee osteoarthritis; treatment group (n = 50) received one capsule containing 0.9 ml castor oil every 8 h for 28 days, the control group (n = 50) received diclofenac sodium (50 mg/8 h for 28 days); both groups reported similar reductions in symptoms, yet the control group reported more adverse side effects (Medhi et al., 2009).	The severe toxicity of castor seeds and particularly the lectin ricin has been extensively reviewed (e.g., Doan, 2004; Worbs et al., 2011). The hydrogenated castor oil is regarded as safe, as reviewed (Anonymous, 2007).	Good evidence on therapeutic benefits but highly toxic. Limited evidence on the safety and efficacy of the leaves
***Tagetes erecta*** L. (Asteraceae) – Aztec marigold – *cempoaxochitl* Native4	20 (75)	Digestive (15); general and unspecified (12), mostly for spiritual illnesses; respiratory (9); skin (9); Aerial parts of this common ornamental plant are used in infusions mainly for gastrointestinal pain and respiratory infections, macerations are topically applied for diverse skin conditions, and the flowers are important in healing rituals.	Nematocidal: acetonic flower extract produced 99% lethal activity *in vitro* against fourth larval stage of *Haemonchus contortus* after 24 h (Aguilar et al., 2008). Antimicrobial: aqueous and organic root extracts demonstrated significant activity *in vitro* against three Gram-positive and two Gram-negative bacteria and 2 fungal strains with MIC ranging 12.5–100 μg/ml (Gupta and Vasudeva, 2010). Analgesic: hydroalcoholic flower extract (100–300 mg/kg IP) induced significant, dose-dependent reduction in nociception in mice writhing test (Bashir and Gilani, 2008).	No signs of acute toxicity of aqueous flower extract (0.03–12 mg/kg IP) in mice (Martínez et al., 2009).	Limited evidence for safety and efficacy of traditional uses S3
***Zea mays*** L. (Poaceae) – maize – *maíz* Native5	20 (71)	Urological (17), as diuretic; digestive (13); general and unspecified (9); Infusions of corn silk are used throughout MA as diuretic, kernels of the carbohydrate staple in diverse preparations as antidiarrhoeic, bracts and kernels particularly of red varieties in ritual healing.	Diuretic: several studies demonstrated significantly increased urinary discharge as well as kaliuresis and natriuresis in rodents after oral administration of aqueous corn silk extracts, as reviewed (Hasanudin et al., 2012). A randomized, double blind, placebo-controlled study on 38 healthy adult male volunteers aged 18–27, however, showed no significant difference in urine output over a 24 h period between treatment group (n = 19) receiving corn silk decoction (16.7 g every 8 h for 1 day) and placebo group receiving a cane sugar solution (Du Dat et al., 1992). Antidiarrhea: the efficacy in reducing duration and symptoms of diarrhea of maize-based oral rehydration solutions has been demonstrated in several clinical studies (e.g., Yartey et al., 1995; Ramakrishna et al., 2008).	No signs of acute toxicity of aqueous extract up to 3200 mg/kg in mice after 48 h (Adedapo et al., 2016) and no signs of subchronic toxicity of aqueous corn silk extract 10 g/kg/d for 90 days) in rats (Wang et al., 2011). Inconsistent results found by Adedapo et al., 2016 in mice developing tachycardia at 200 and 400 mg/kg/d over 7 days but not at 800 mg/kg/d as well as moderate inflammation of the atrium and ventricle at 400 and 800 mg/kg/d after 7 days.	Possibly safe and effective, with reservations for diuretic activity D4, U1
***Cymbopogon citratus*** (DC.) Stapf (Poaceae) – lemongrass - *zacate limón, telimón, té de limón* Exotic2	18 (52)	Digestive (10); respiratory (10); general and unspecified (7); Infusion of aromatic leaves used throughout MA for stomachache, cough, common cold, and flu.	Broad antimicrobial: several preclinical studies demonstrated significant antimicrobial (incl. gastrointestinal and respiratory pathogens) activity attributed mostly to the essential oil fraction, as reviewed (Ekpenyong et al., 2015). Analgesic and antiinflammatory: several preclinical studies demonstrated significant activity in different rodent models, as reviewed (Ekpenyong et al., 2015).	No signs of acute or subchronic toxicity on 11 (5 f, 6 m) healthy volunteers aged 19–24 receiving aqueous leaf extract (4g/d for 14 days) (Leite et al., 1986).	Safe and effective A1, D5, R1
***Ocimumbasilicum*** L. (Lamiaceae) – basil – *albahaca* Exotic3	18 (67)	General and unspecified (14); digestive (11); neurological (9); Aromatic twigs used throughout MA for ritual healing, infusion of leaves drunk against gastrointestinal pain, maceration topically applied against headache.	Broad antimicrobial: a number of preclinical studies reported significant antibacterial, antiviral and fungicidal activities attributed to essential oil fractions, as reviewed (Suppakul et al., 2003). Anticolitis: essential oil (100–400 μl/kg PO) significantly protected rats from acetic acid-induced colitis (Rashidian et al., 2015). Analgesic: essential oil (50–200 mg/kg SC) significantly and dose-dependently reduced nociception in mice in the hot plate, formalin, and writhing tests (Venâncio et al., 2011).	No signs of acute or subchronic toxicity at therapeutic doses of essential oil (<1,000 mg/kg/d IG for 14 days) and hydroalcoholic leaf extract (50–500 mg/kg/d IG for 14 days) in rats (Fandohan et al., 2008; Rasekh et al., 2012).	Safe and effective A2, D6, N1
***Piper auritum*** Kunth (Piperaceae) – *hierba santa, santa maria, acuyo, momo* Native6	18 (61)	Digestive (9); skin (9); musculoskeletal (7); general and unspecified (7); Apart from culinary uses, the aromatic leaves are used throughout MA to treat diverse inflammatory conditions internally and topically.	Antiinflammatory: significant effect of ethanolic leaf extract (287–863 mg/kg IP) in carrageenan-induced rat paw edema model (Vega Montalvo and Lagarto Parra, 1999). Antileishmaniasis: essential oil showed *in vitro* IC_50_ of 22.3 μg/ml against *L*. *donovani* (Monzote et al., 2010).	No sign of acute dermal toxicity or irritation in rabbits and rats treated with aqueous leaf extract (López Barreiro et al., 2014). Safrole, making up around 70% of essential oil, has well studied hepatotoxic and hepatocarcinogenic properties (Gupta et al., 1985; Jin et al., 2011).	Limited data on efficacy and safety S4
***Byrsonima crassifolia*** (L.) Kunth (Malpighiaceae) – *nanche*, *nance* Native7	17 (50)	Digestive (13), particularly as antidiarrheal; skin (8); Decoction of astringent bark of fruit tree used in lowland MA for diarrhea and inflammatory skin conditions.	Antiinflammatory: significant, dose-dependent effect of lipophilic bark extract (30–300 μg/cm^2^ TOP) in croton oil-induced ear dermatitis in mice (Maldini et al., 2009). Antibacterial: moderate effects of root and stem extracts on several enteropathogenic strains in disk diffusion assay (Martínez-Vázquez et al., 1999). A 50% ethanolic bark extract showed activity against *Salmonella thyphi* and *Shigella flexneri* with inhibition zones ≥ 9 mm (Cáceres et al., 1990) Antifungal: significant effects (MIC 10–200 μg/ml) of aqueous and organic extracts from different organs on four dermatophytic strains (Cáceres et al., 1993b). Wound healing: significant effects hexane seed extracts in different wound healing models in diabetic rats (Pérez Gutiérrez and Muñiz Ramirez, 2013).	No signs of serious acute toxicity of organic extracts of aerial parts in mice (500–2,000 mg/kg PO) (Herrera-Ruiz et al., 2011).	Possibly effective and safe, yet no data on antidiarrheal activity D7, S5
***Phyla scaberrima*** (Juss. ex Pers.) Moldenke (syn.: *Lippia dulcis*) (Verbenaceae) – Aztec sweet herb – *hierba dulce*, *orozus* Native8	17 (40)	Respiratory (11); digestive (10); Infusions of sweet, aromatic leaves used throughout MA against stomachache and cough.	Spasmolytic: significant effect of essential oil on porcine bronchial segments at 100 μg/ml (Görnemann et al., 2008). Antibacterial: moderate effects on several enteropathogenic strains of organic leaf extracts in disk diffusion assay (Cáceres et al., 1993a). Antiinflammatory: ethanolic leaf extract (400 mg/kg PO; 0.5 mg/ear TOP) showed significant inhibition of carrageenan-induced rat paw edema and TPA-induced mice ear edema (Pérez et al., 2005).	Camphor, present at relatively high concentrations in the leaves and flowers, could be responsible for toxic symptoms – such as nausea, drowsiness and abortifacient properties – sometimes reported after ingestion of the plant (Compadre et al., 1986).	Possibly effective yet possibly toxic D8, R2
***Matricariachamomilla*** L. (Asteraceae) – chamomile – *manzanilla* Exotic4	16 (57)	Digestive (14); Infusions of this aromatic introduced herb are used throughout MA to alleviate gastrointestinal pain.	Spasms and inflammatory conditions of the digestive system: while internationally published clinical trials using only chamomile tea are lacking, the German Commission E has approved its internal use for spasms and inflammations of the gastrointestinal tract based on clinical trials (Wichtl, 2004; McKay and Blumberg, 2006). Further, several RCTs using combinations of chamomile and other species demonstrated significant improvement on infants and children suffering from colic or diarrhea, as comprehensively reviewed (McKay and Blumberg, 2006). Antidiarrheal, broad antimicrobial, antiinflammatory, and antiulcer activities: have been demonstrated in a considerable number of *in vitro* and *in vivo* models, as comprehensively reviewed (McKay and Blumberg, 2006).	Generally regarded as safe, although allergic reactions may occur (Wichtl, 2004; McKay and Blumberg, 2006).	Likely effective and possibly safe D9
***Rosmarinusofficinalis*** L. (Lamiaceae) – rosemary – *romero* Exotic5	16 (50)	General and unspecified (10); digestive (8); pregnancy (8); The aromatic twigs of this introduced species are used throughout MA in ritual healing, particularly as an alcoholic maceration; decoctions are drunk against stomachache and used in post-partum baths.	Dyspepsia: while internationally published clinical trials are lacking, the German Commission E has approved the use of rosemary leaves for dyspeptic conditions (Wichtl, 2004; Ulbricht et al., 2010). Improvement of mental state: some “methodologically weak” human trials showed significant effects of aromatherapy with rosemary essential oil, on cognitive performance and subjective well-being, as reviewed (Ulbricht et al., 2010). Antidepressant and anxiolytic: several studies showed significant effects in different rodent models, as reviewed (Ulbricht et al., 2010). Broad antimicrobial: a number of *in vitro* studies have demonstrated moderate antibacterial and antifungal effects of various rosemary extracts, as comprehensively reviewed (Ulbricht et al., 2010). Antiinflammatory: significant effects of various rosemary extracts have been demonstrated in several *in vitro* and *in vivo* models, as comprehensively reviewed (Ulbricht et al., 2010). Antinociceptive: significant effects of various rosemary extracts have been demonstrated in several *in vivo* models, as comprehensively reviewed (Ulbricht et al., 2010). Antiparasitic: 3 studies showed significant effects of different extracts on different protozoan parasites, as comprehensively reviewed (Ulbricht et al., 2010). Antiulcer: a crude hydroalcoholic extract significantly reduced ulcerative lesion in several rat models, as reviewed (Ulbricht et al., 2010). Antiviral: several studies report significant effects of various rosemary extracts on HI and herpes simplex virus, as comprehensively reviewed (Ulbricht et al., 2010). Spasmolytic: a single study demonstrated significant effects of an alcoholic leaf extract on isolated guinea pig ileum, as reviewed (Ulbricht et al., 2010).	Generally regarded as safe, although allergic reactions and interactions with other drugs may occur; unsafe for women who are pregnant or are trying to become pregnant (Wichtl, 2004; Ulbricht et al., 2010).	Possibly effective in certain conditions and likely safe with reservations A3, D10
***Sambucus canadensis*** L. (syn. *Sambucus nigra* supsp. *canadensis*) (Adoxaceae) – American elder – *saúco* Native9	16 (75)	Respiratory (14); skin (9); digestive (8); Infusions of the inflorescence are drunk against coughs; the leaves are used topically for skin infections and internally for gastrointestinal ailments.	Fungicidal: aqueous leaf extract was shown to be effective against several strains of dermatophytic fungi with tube dilution method (Cáceres et al., 1991) Common cold: the use of the flowers of the closely related *S. nigra* for colds has been approved by the German Commission E (Ulbricht et al., 2014). Antiviral: Several *in vitro* and *in vivo* studies reported significant antiinflammatory and antiviral (including several strains of influenza) effects of *S. nigra* fruit extracts, as comprehensively reviewed (Ulbricht et al., 2014).	Flowers from *S. canadensis* are generally recognized as safe by the U.S. Food and Drug Administration (FDA). Products derived from other plant parts are potentially toxic due to the presence of considerable quantities of the cyanogenic glycoside sambunigrin.	Flowers for respiratory conditions likely safe and effective; other uses likely unsafe R3, S6
***Tagetes lucida*** Cav. (Asteraceae) – sweet-scented marigold – *pericón* Native10	16 (55)	Digestive (12), mostly stomachache; pregnancy (8); general and unspecified (8), respiratory (7); Infusions of the anise-scented aerial parts are drunk against gastrointestinal pain and respiratory infections and used as washings after childbirth and in ritual healing.	Broad antimicrobial: significant effects against several bacterial and fungal strains (incl. pathogens of the digestive and respiratory tract) shown in several *in vitro* models (Céspedes et al., 2006; Castillo-Juárez et al., 2009; Leal et al., 2014). A 10% alcoholic leaf and flower extract showed good activity against *Vibrio cholerae* in the disk diffusion assay (50 μL/disc; unknown conc.) while the n-hexane extract (at 10, 30 and 50 μg) showed the best activity in the disk minimal inhibitory concentration method against different strains of the bacteria (Cáceres et al., 1993c). Antiinflammatory: induced nitric oxide and prostaglandin E2 production as well as the expression of COX-2 in macrophages was significantly inhibited by the essential oil (0.05–0.2 mg/ml) (Sepúlveda-Arias et al., 2013). Antidepressant and anxiolytic: aqueous and organic extracts (10–300 mg/kg IP or 5–200 mg/kg PO for 14 days) showed significant effects in various rodent models; the antidepressant-like effects were shown to be mediated *via* a serotonergic mechanism (Guadarrama-Cruz et al., 2008; Gabriela et al., 2012; Guadarrama-Cruz et al., 2012; Bonilla-Jaime et al., 2015; Pérez-Ortega et al., 2016).	Low acute toxicity in mice with LD_50_ values of 2,000 mg/kg (IP) and 970 mg/kg (IP) for an aqueous and ethanolic extract respectively; after administration the mice did not show signs of toxicity during a 14 days observation period (Pérez-Ortega et al., 2016).	Possibly safe and effective A4, D11, R4
***Aloe vera*** (L.) Burm.f. (Asphodelaceae) – *sábila* Exotic6	15 (81)	Skin (15); digestive (10); respiratory (10); musculoskeletal (9); general and unspecified (9); The leaves of this introduced ornamental are used internally and topically to treat diverse infectious and inflammatory conditions as well as pain and fever.	Antiinflammatory, analgesic, wound healing, antimicrobial, antiulcer and other pharmacological effects relevant to *A*. *vera*’s traditional uses have been demonstrated in a number of preclinical studies as reviewed (Akaberi et al., 2016). However, in most cases critical reviews find mixed, contradictory or insufficient evidence such as for wound healing (Dat et al., 2012) and ulcers (Borrelli and Izzo, 2000). On the other hand, a systematic review on clinical efficacy concludes that aloe gel might be effective in the treatment of genital herpes and psoriasis while several studies suggest topical antiinflammatory properties of aloe gel in mice (Vogler and Ernst, 1999).	Leaf juice and polysaccharides are safe as cosmetic ingredients with anthraquinone levels not exceeding 50 ppm. Aloe extracts for internal applications based on the laxative anthranoids are generally not regarded as safe (Andersen, 2007). Generally, ingestion of *A. vera* derived products is associated with diarrhea, electrolyte imbalance, kidney dysfunction and drug-drug interaction; further, contact dermatitis, erythema, and phototoxicity have been reported from topical applications (Boudreau and Beland, 2006).	Effective and safe for topical applications, likely unsafe upon ingestion S7
***Bryophyllum pinnatum*** (Lam.) Oken (syn. *Kalanchoe pinnata*) (Crassulaceae) – air plant, miracle leaf - *sanalotodo*, *belladonna*, *hoja de aire* Exotic7	15 (50)	Skin (11); neurological (9); musculoskeletal (7); general and unspecified (7); The succulent leaves of this introduced plant are applied topically for inflammatory skin disorders, fever and pain.	Antiinflammatory: upon administration of aqueous (400 mg/kg PO) as well as ethanolic extracts (0.1–1 mg/ear TOP) significantly reduced inflammatory response parameters in several rodent models (Chibli et al., 2014; F̈rer, et al., 2016). Antimicrobial: 2 studies showed significant effects on various bacterial and fungal strains *in vitro*, as reviewed (F̈rer, et al., 2016). Antileishmaniasis: an observational study with a single patient and a study with mice showed significant effects of aqueous leaf extracts (F̈rer, et al., 2016). Antinociceptive: various leaf extracts showed significant effects on rodents in the writhing and hot plate tests (F̈rer, et al., 2016). Wound healing: ethanolic leaf extract (0.25–1 mg/kg/d TOP for 14 days) significantly reduced the size of wound area in a rat wound excision model (Nayak et al., 2010). Neuro-depressive effects: aquatic leaf extract (50 to 200 mg/kg PO) showed sedative and muscle relaxant activities in rodents and an observational study found a subjective improvement of sleep quality in 49 pregnant women with sleeping disorders, as comprehensively reviewed (F̈rer, et al., 2016).	Therapeutic applications have been well tolerated in human trials. However, the herbal drug contains bufadienolides, which are potentially toxic upon ingestion (F̈rer, et al., 2016).	Topical applications possibly safe and effective; internal administration likely unsafe A5, S8
***Bursera simaruba*** (L.) Sarg. (Burseraceae) – gumbo-limbo – *palo mulato*, *chaca*, *jiote* Native11	15 (50)	General and unspecified (13), mainly as antipyretic; skin (7); Macerations of the bark are used throughout MA in washings against fever and burns.	Antiinflammatory: lipophilic bark extracts showed antiinflammatory activity (ID_50_ values of hexane and chloroform extracts of 221 and 143 μg/cm^2^ TOP, respectively) in an experimentally induced ear edema model in mice (Sosa et al., 2002). Hexane leaf extract (at an equivalent of 1.5 g/kg PO) significantly reduced carrageenan-induced rat paw edema (Carretero et al., 2008). Antimicrobial: aqueous, alcoholic and chloroform stem bark extracts showed moderate activity against *C. albicans* and *Streptococcus mutans* in the disc diffusion assay (Rahalison et al., 1993; Rosas-Piñón et al., 2012).	ND	Limited evidence on efficacy and lacking evidence on safety S9
***Carica papaya*** L. (Caricaceae) – papaya Native12	15 (27)	Digestive (10), mainly as anthelmintic; Seeds and sap of this fruit shrub are used in lowland MA against gastrointestinal parasites.	Nematocidal: latex was shown active against the intestinal mouse nematode *Heligmosomoides bakeri* (Luoga et al., 2015), while papaya seed extracts containing benzyl isothiocyanate were toxic for *Caenorhabditis elegans* (Kermanshai et al., 2001). A RCT confirmed the efficacy of cysteine proteinase obtained from papaya latex against *Trichuris suis* infection in pigs. A single oral dose of 450 μmol was effective against low and high infection with *T. suis* eggs (Levecke et al., 2014).	Papain, a bioactive protein present in *C. papaya* latex did not show mutagenic or toxic effects against *E. coli* strains (da Silva et al., 2010).	Possibly safe and therapeutically effective D12
***Citrus sinensis*** (L.) Osbeck (Rutaceae) – orange – *naranja* Exotic8	15 (64)	Digestive (11); respiratory (11); general and unspecified (10); psychological (8); Infusions of leaves, flowers and fruit peel of this introduced fruit tree are used throughout MA for stomachache, respiratory infections, anxiety-like disorders, and for ritual healing.	Broad antimicrobial: crude plant extracts, essential oils and isolated compounds demonstrated significant effects against a variety of fungal and bacterial strains incl. gastrointestinal and respiratory pathogens *in vitro*, as reviewed (Favela-Hernández et al., 2016). Anxiolytic and sedative: a limited number of clinical trials and rodent models showed significant tranquilizing effects of orange aroma and IP administration of organic extracts, as reviewed (Favela-Hernández et al., 2016).	No toxicological studies directly relevant to traditional uses have been published. Synephrine a component similar to the banned ephedrine and present in the leaves, peel and juice should not give rise to concerns (Arbo et al., 2008; Stohs et al., 2011; Ulbricht et al., 2013).	Possibly effective, yet limited evidence on safety A6, D13, P1, R5
***Hamelia patens*** Jacq. (Rubiaceae) – firebush – *chichipince*, *coralillo, chacloso* Native13	15 (50)	Skin (12); digestive (7); The leaves are used for wound healing and dermatological and gastrointestinal infections.	Antiinflammatory: lipophilic bark extracts showed antiinflammatory activity (ID_50_ value of the chloroform extract = 255 μg/cm^2^ TOP) in experimentally induced ear edema in mice (Sosa et al., 2002). The hexane extract of the leaves applied orally reduced carrageenan induced edema and inflammation in rats at 200 and more readily at 500 mg/kg (Jiménez-Suárez et al., 2016). Wound healing: ointment produced with 5% and 10% ethanolic extract of the aerial parts significantly augmented the healing process of experimentally induced wounds in rats (Gomez-Beloz et al., 2003). Antibacterial: hexane and methanol leaf extract showed moderate MIC (2.5 mg/ml) against *E. coli* (Camporese et al., 2003).	No signs of acute (14 days) or sub-acute (28 days) toxicity of ethanolic leaf extract in mice and rats, with LD_50_ determined at 2900 mg/kg IP and > 5000 mg/kg PO (Alonso-Castro et al., 2015b).	Possibly safe and effective S10
***Mangiferaindica*** L. (Anacardiaceae) – mango Exotic9	15 (38)	Digestive (10); respiratory (7); Decoctions of leaves and bark of this introduced fruit tree are used against diarrhea, gastrointestinal inflammations, and respiratory infections.	Antidiarrheal: aqueous leaf extract (25, 50 and 100 mg/kg PO) dose-dependently reduced the severity of diarrheal episodes in rats (Yakubu and Salimon, 2015); further, 2 mice studies showed antidiarrheal effects of seed extracts, as reviewed (Shah et al., 2010; Parvez, 2016). Antiinflammatory: a limited number of studies showed significant effects of seed and bark extracts in different *in vivo* and *in vitro* models, as reviewed (Shah et al., 2010; Parvez, 2016). Antimicrobial: several studies showed significant effects of aqueous and alcoholic leaf and bark extracts against a series of bacterial and fungal strains, incl. gastrointestinal and respiratory pathogens, in disk diffusion assays, as reviewed (Shah et al., 2010; Parvez, 2016). Gastroprotective: mangiferin, a xanthanoid abundant in the stem bark was shown to mediate antiinflammatory and gastroprotective effects (10 and 20mg/kg IP) in a gastric ulcer model with rats (Mahmoud-Awny et al., 2015). Mangiferin at 3, 10 and 30 mg/kg PO dose-dependently reduced gastric lesion induced by alcohol in rats (Carvalho et al., 2007).	No signs of acute toxicity of aqueous stem bark extracts (up to 2,000 mg/kg PO and TOP) in mice and rats (Garrido et al., 2009).	Possibly safe and effective D14, R6
***Nicotiana tabacum*** L. (Solanaceae) – tobacco – *tabaco* Native14	15 (50)	Skin (11), particularly against snakebites; respiratory (8); general and unspecified (8); Leaves are used topically against snakebites as well as skin and respiratory infections, tobacco is also important in ritual healing.	Broad antimicrobial: aqueous and organic leaf extracts as well as isolated polyphenols and nicotine significantly inhibited the growth of various strains of bacteria and fungi (incl. pathogens of the skin and respiratory system) in the disk diffusion and broth dilution methods (Akinpelu and Obuotor, 2000; Pavia et al., 2000; Wang et al., 2008; Bakht et al., 2012; Kalayou et al., 2012). Antisnake venom: as the neurotoxic effects of some snake venoms are mediated *via* nicotinic acetylcholine receptors (nAChR), nicotine may be effective in alleviating the symptoms of certain snakebites, as reviewed (Giovannini and Howes, 2017). Respiratory stimulation: in small doses nicotine can act as a respiratory stimulant. In larger doses, however, it causes respiratory depression (Dewick, 2002: 314)	Intoxication with nAChR agonists such as nicotine is biphasic and may lead to abdominal pain, hypertension, tachycardia, and tremors and successively give way to hypotension, bradycardia, and dyspnea while high doses may result in coma and respiratory failure (Schep et al., 2009).	Possibly effective yet probably unsafe
***Pimenta dioica*** (L.) Merr. (Myrtaceae) – allspice – *pimienta gorda* Native15	15 (55)	Digestive (12); female genital (8); pregnancy (8), particularly postpartum care; Leaves of this culinary spice are used in lowland MA as infusions for gastrointestinal ailments and as washings for reproductive health.	Broad antimicrobial: aqueous and organic leaf extracts, essential oil as well as isolated tannins and eugenol (the major compound of the essential oil) significantly inhibit the growth of various pathogenic bacterial and fungal strains (Shaival et al., 2001; Kamatou et al., 2012; Zhang and Lokeshwar, 2012; Bhat and Raveesha, 2016; Al-Harbi et al., 2018). Analgesic: leaf and fruit extracts as well as isolated eugenol have well demonstrated antinociceptive effects, as reviewed (Kamatou et al., 2012; Zhang and Lokeshwar, 2012). Antiinflammatory: aqueous leaf extracts, isolated eugenol and eugenol-rich extracts proved effective in various inflammation models, as reviewed (Kamatou et al., 2012; Zhang and Lokeshwar, 2012). Antidiarrheal: isolated eugenol significantly reduced various parameters of castor oil-induced diarrhea in rats (Kamatou et al., 2012). Antiulcer: aqueous leaf extracts and isolated eugenol significantly reduced the lesions from experimentally induced gastric ulcers in mice and rats, as reviewed (Kamatou et al., 2012; Zhang and Lokeshwar, 2012). Antispasmodic: isolated eugenol significantly reduced contractions in isolated ileum and tracheal muscles, as reviewed (Kamatou et al., 2012). Anticancer: eugenol’s antiproliferative and cytotoxic effects against different cancer cell lines were demonstrated in several *in vitro* and *in vivo* studies, as reviewed (Kamatou et al., 2012; Zhang and Lokeshwar, 2012).	Eugenol is generally recognized as safe by the US Food and Drug Administration (FDA). The safety of crude plant extracts has not been evaluated. (Kamatou et al., 2012; Zhang and Lokeshwar, 2012).	Possibly safe and effective D15, W1, X1
***Punicagranatum*** L. (Lythraceae) – pomegranate – *granada* Exotic10	15 (26)	Digestive (13); Infusions of the astringent fruit skin of this introduced tree are used throughout MA against oral infections and diarrhea.	Antidiarrheal: aqueous fruit peel extracts (100, 200, 300, 400 mg/kg IP) significantly and dose-dependently reduced gastrointestinal transit and castor oil-induced enteropooling in rats and contractions in isolated rat ileum (Qnais et al., 2007). Wound healing: TOP-applied gel, using methanolic fruit peel extract significantly accelerated the healing process in the wound excision model in rats (Murthy et al., 2004).	LD_50_ = 1321 mg/kg of aqueous fruit peel extract (IP) determined in mice (Qnais et al., 2007). Also, an acute toxicity study in mice with IP-administered hydroalcoholic whole fruit extract concluded that toxic effects occurred only at doses far higher than those used in T&CM (Vidal et al., 2003).	Possibly safe and effective D16
***Allium sativum*** L. (Amaryllidaceae) – garlic – *ajo* Exotic11	14 (63)	Digestive (11); general and unspecified (10); musculoskeletal (9); skin (8); respiratory (7); The raw bulbs of this introduced, cultivated plant are used internally against gastrointestinal parasites and respiratory infections, topically against musculoskeletal pain and fungal infections, and for ritual healing.	Anthelmintic: subchronic (7d) oral administration of garlic homogenate demonstrated more effective against *Aspiculuris tetraptera* in natural infected mice than the standard antiparasitic ivermectin (0.2 mg/kg IM) (Ayaz et al., 2008). Broad antimicrobial (incl. gastrointestinal, dermatological, and respiratory pathogens): a number of studies have demonstrated significant antibacterial, antiviral, and fungicidal effects of garlic extracts, as reviewed (Harris et al., 2001). Antiinflammatory and antiarthritis: topically administered isolated thiacremonone (1 μg/kg – 10 mg/kg) demonstrated significant activity in several rodent models (Ban et al., 2009).	No serious toxicological risks although allergic reactions can occur, as extensively reviewed (Aviello et al., 2009).	Safe and effective D17, L1, R7, S11
***Artemisia ludoviciana*** Nutt. (Asteraceae) – silver wormwood – *estafiate* Native16	14 (39)	Digestive (12); general and unspecified (7); Infusions of the aromatic and bitter aerial parts are drunk in Mexico for diverse gastrointestinal ailments.	Anti-*Helicobacter pylori*: *in vitro* MIC of 125 μg/ml aqueous extract (Castillo-Juárez et al., 2009). Anti-*Giardia lamblia* and *Entamoeba hystolitica*: *in vitro* IC_50_ of aqueous extracts > 1900 μg/ml; IC_50_ of hexane extracts < 140 μg/ml, for both pathogens (Said Fernández et al., 2005). Antinociceptive: essential oil (1–316 mg/kg) significantly and dose-dependently reduced the response of mice in the hot-plate and formalin tests, likely mediated *via* an opioid mechanism (Anaya-Eugenio et al., 2016). Not spasmolytic: organic extracts (0.97–1,000 μg/ml) showed no antispasmodic activity in isolated rat ileum (Estrada-Soto et al., 2012).	ND	Limited evidence on efficacy and no data on safety A7, D18
***Bixa orellana*** L. (Bixaceae) – achiote Native17	14 (45)	Skin (10); general and unspecified (9), mainly as antipyretic; Leaves and seeds are applied topically to treat skin infections and fever in lowland MA. Seeds also as coloring agent and culinary condiment.	Broad antimicrobial as well as antileishmaniasis: leaf extracts as well as seed powder (annatto) showed significant activity in a number of preclinical studies on a broad range of pathogens, as comprehensively reviewed (Ulbricht et al., 2012; Stohs, 2014). A 50% ethanolic leaf extract (90 ml/10 g powdered leaves) showed significant inhibition zones (17 mm) in the disk diffusion assay (50 μL/disk) against *Neisseria gonorrhoeae* (Cáceres et al., 1995). Antiinflammatory: significant activity of leaf extracts in a several rodent models, as reviewed (Ulbricht et al., 2012; Stohs, 2014).	No serious toxicological risks although allergic reactions can occur, as extensively reviewed (Ulbricht et al., 2012; Stohs, 2014).	Safe and effective with reservations; lacking evidence for antipyretic activity S12
***Hyptis verticillata*** Jacq. (Lamiaceae) – *hierba San Martín* Native18	14 (51)	General and unspecified (10); digestive (7); musculoskeletal (7); The aerial parts are used in lowland MA for ritual healing, infusions drunk for diverse gastrointestinal ailments, and alcoholic macerations are used for massaging aching bones.	Antimicrobial and antiinflammatory: few studies have been carried out and shown limited activity, as extensively reviewed (Picking et al., 2013).	Infusions and topic applications appear safe, some concern exists in regard to the use of alcoholic macerations due to elevated concentrations of cytotoxic lignans, as reviewed (Picking et al., 2013).	Lack of evidence on efficacy, safe with reservations
***Lantana camara*** L. (Verbenaceae) – *cinco negritos*, *conchita* Native19	14 (46)	Digestive (10); respiratory (7); Infusions of the aromatic leaves are drunk against gastrointestinal pain and respiratory infections.	Broad antimicrobial (incl. gastrointestinal and respiratory pathogens) and nematocidal: a number of preclinical studies demonstrated significant antibacterial, antiviral, fungicidal, and nematocidal activities of essential oil and leaf extracts, as extensively reviewed (Ghisalberti, 2000; Sharma et al., 2007). Antidiarrheal: methanolic leaf extract (125–1,000 mg/kg IP) induced significant dose-dependent reduction in fecal output in mice with castor oil-induced diarrhea (Sagar et al., 2005).	Severely hepatotoxic and phototoxic, attributed mostly to phenolics and triterpenoids, as comprehensively reviewed (Ghisalberti, 2000; Sharma et al., 2007).	Effective but unsafe
***Tecoma stans*** (L.) Juss. ex Kunth (Bignoniaceae) – yellow trumpetbush – *tronadora* Native20	14 (36)	Endocrine (10); mainly as antidiabetic; Decoctions of the aerial parts are drunk against hyperglycemia.	Antidiabetic: The decoction of the leaves and stem showed hypoglycemic effects in the glucose tolerance test in rabbits (Román-Ramos et al., 1991) and a leaf decoction antidiabetic activity in STZ-induced hyperglycemia in rats (Aguilar et al., 1993). Several studies reported antidiabetic effect of leaf extracts (Aguilar-Santamaría et al., 2009) while the aqueous leaf extract significantly reduced plasma cholesterol and triglycerides at 500 mg/kg/d for 21 days in STZ and healthy rats without affecting glycaemia (Aguilar-Santamaría et al., 2009). Moreover, the aqueous leaf and the methanolic bark extracts were shown to inhibit α-glucosidase *in vitro* (Aguilar-Santamaría et al., 2009; Evangeline et al., 2015).	Not signs of toxicity in *Drosophila melanogaster* larvae being fed aqueous extracts of aerial parts for 48 h (Sortibrán et al., 2011). A 50% alcoholic leaf extract showed no acute toxicity up to 5,000 mg/kg PO and no signs of chronic toxicity at 500 mg/kg/d over 28 days in mice (Larbie et al., 2019).	Effective with reservations, limited data on safety T1
***Cedrela odorata*** L. (Meliaceae) – *cedro* Native21	13 (43)	General and unspecified (7); Aqueous extracts of leaves, bark or wood of this important timber species are applied in a bath or orally as an antipyretic in lowland MA.	Antimalaria: *in vitro* IC_50_ of 1.1–9.3 μg/ml of ethanolic wood extracts from different populations against *Plasmodium falciparum* W2 and D6 clones, attributed to the limonoid gedunin (MacKinnon et al., 1997).	No serious toxicological risks: low acute (10–5,000 mg/kg IP) and subchronic (500 mg/kg/d PO) of hydroethanolic bark extract in mice even at elevated doses (Giordani et al., 2015).	Safe but limited data on efficacy
***Citrus* ×*aurantium*** L. (Rutaceae) – bitter orange – *naranja agria* Exotic12	13 (54)	General and unspecified (9); psychological (8); digestive (7); respiratory (7); Infusions of leaves, flowers, and zest of this introduced fruit tree are drunk for emotional and anxiety-like disorders, fever, respiratory infections, and stomachache and used for ritual healing.	Anxiolytic: clinical evidence for significant anxiolytic effects of flower distillate, as reviewed (Stohs et al., 2012; Sarris et al., 2013) and recently reaffirmed (Pimenta et al., 2016). Antimicrobial and antirotavirus: limited evidence from preclinical studies, as reviewed (Ulbricht et al., 2013)	No serious toxicological risks Synephrine a component similar to the banned ephedrine and present in the leaves, peel and juice should not give rise to concerns (Arbo et al., 2008; Stohs et al., 2011; Ulbricht et al., 2013).	Limited data on efficacy, save with reservations A8, D19, P2, R8
***Jatropha curcas*** L. (Euphorbiaceae) – purging nut – *piñon* Native22	13 (25)	Skin (12); The sap of this shrub, used for living fences, is applied to skin infections, particularly around the lips.	Antimicrobial: significant *in vitro* activity not including dermatological pathogens, as reviewed (Thomas et al., 2008). In a placebo-controlled study the milk sap was shown effective against common warts (Marroquin et al., 1997).	Severely acutely toxic upon oral ingestion, as reviewed (Thomas et al., 2008).	Unsafe upon ingestion and limited evidence for efficacy in topical applications
***Petiveria alliacea*** L. (Petiveriaceae) – *hierba de zorrillo, apacín* Native23	13 (44)	General and unspecified (9); digestive (7); musculoskeletal (7); Infusions of the entire plant are used in washings against fever and pain or drunk against stomachache in lowland MA.	Analgesic: significant antinociceptive effects of aqueous and organic extracts were demonstrated in several rodent models, as reviewed (Luz et al., 2016).	Low acute and subchronic toxicity of aqueous and organic extracts of different plant parts in several animal studies, as extensively reviewed (Luz et al., 2016).	Effective and safe with reservations A9, D20, L2
***Spondias purpurea*** L. (Anacardiaceae) – hog plum – *ciruela, jocote* Native24	13 (29)	Skin (8); digestive (7); Maceration of leaves of this lowland fruit tree are topically applied for inflammatory skin conditions and drunk for diverse gastrointestinal ailments.	Antibacterial: significant *in vitro* activity of hexane leaf extracts against several skin- and enteropathogens demonstrated with the disk diffusion method (Cáceres et al., 1993a; Miranda-Cruz et al., 2012). Antiulcer: hexane leaf extract (12.5–50 mg/kg PO) significantly and dose-dependently reduced area of induced GI ulcerative lesions in mice and rats (De Almeida et al., 2017).	ND	Limited evidence on efficacy and no data on safety D21, S13
***Tanacetumparthenium*** (L.) Sch.Bip. (Asteraceae) – feverfew – *Hierba Santa María* Exotic13	13 (53)	Digestive (12); pregnancy (8); general and unspecified (7); Infusions of aerial parts of this introduced aromatic herb are drunk against diverse gastrointestinal ailments and used in post-partum washing.	Analgesic: significant activity of ethanolic flower extracts (10–300 mg/kg PO) in several rodent models (Jain and Kulkarni, 1999; Mannelli et al., 2015) but insufficient evidence for significant prevention from migraine headache based on several double-blind RCTs against, as comprehensively reviewed (Pittler and Ernst, 2004). Antiinflammatory: significant activity of ethanolic flower extracts (10–300 mg/kg PO) in carrageenan-induced rat paw edema model (Jain and Kulkarni, 1999; Mannelli et al., 2015) as well as of the isolated sesquiterpene lactone parthenolide demonstrated in a number of studies, as reviewed (Mathema et al., 2012). Antibacterial (incl. several gastrointestinal pathogens): *in vitro* IC_50_ of 4–38 μg/ml of essential oil against 8 different Gram-positive and Gram-negative bacteria (Mohsenzadeh et al., 2011).	No major safety concerns, as only mild and transient adverse effects established in a number of clinical trials, as comprehensively reviewed (Pittler and Ernst, 2004).	Safe and effective with reservations A10, D22, W2
***Allium cepa*** L. (Amaryllidaceae) – onion – *cebolla* Exotic14	12 (44)	Respiratory (8); skin (7); The bulbs of this introduced food plant are used internally for respiratory infections and topically for diverse skin disorders.	Wound healing: a number of clinical trials evidenced efficacy of topically applied onion extracts for skin scarring, as comprehensively reviewed (Sidgwick et al., 2015). Antibacterial: significant *in vitro* activity against a number of Gram-positive strains, as reviewed (Corzo-Martínez et al., 2007; Sharifi-Rad et al., 2016). Antiasthmatic and antiinflammatory: limited evidence from *in vitro* models, as reviewed (Corzo-Martínez et al., 2007).	Generally regarded as safe, although some adverse effects may occur, as reviewed (Corzo-Martínez et al., 2007; Sidgwick et al., 2015).	Safe and effective with reservations R9, S14
***Citrus limon*** (L.) Osbeck (Rutaceae) – lemon – limón Exotic15	12 (53)	Digestive (10); respiratory (9); general and unspecified (7); The fruit juice, zest, and leaves of this introduced fruit tree are used internally for infections of the digestive and respiratory tract as well as for ritual healing.	Antiviral (incl. influenza virus) and broad antimicrobial (incl. enteropathogens): several *in vitro* studies demonstrated significant effects of essential oil, as reviewed (Goetz, 2014). Antiinflammatory specifically antibronchitis: a few *in vitro* and *in vivo* studies demonstrated significant activity, and attributed to flavonoids, as reviewed (Goetz, 2014).	Generally regarded as safe although irritation may occur, as reviewed (Goetz, 2014).	Effective and safe D23, R10
***Guazuma ulmifolia*** Lam. (Malvaceae) – West Indian elm – *caulote, guácimo* Native25	12 (34)	Digestive (11), mainly as antidiarrheal; Decoctions of the astringent bark and fruits are used in lowland MA against diarrhea.	Antimicrobial: significant *in vitro* activity against a number of enteropathogens (Cáceres et al., 1990; Camporese et al., 2003; Jacobo-Salcedo et al., 2011). Antidiarrheal: complete inhibition of cholera toxin-induced chloride secretion in rabbit distal colon and significant reduction of intestinal motility in mice upon administration of crude bark extract (Hör et al., 1995; Michelin et al., 2010).	Not acutely toxic in *Artemia salina* essay (aqueous leaf extract 10–1,000 ppm) (Navarro et al., 2003).	Likely effective but no evidence on safety of bark D24
***Justicia spicigera*** Schltdl. (Acanthaceae) – Mexican honeysuckle – *muicle, añil, hoja de tinta* Native26	12 (42)	General and unspecified (12); The aerial parts are used for dying and in ritual healing, including the oral administration of infusions.	Antidepressant: significant, dose-dependent effect of the isolated flavonoid kaempferitrin (1–20 mg/kg PO) in two mice models (Cassani et al., 2014). Immunostimulatory: ethanolic leaf extract as well as isolated kaempferitrin induced significant effects in several *in vitro* assays (Alonso-Castro et al., 2012b; Juárez-Vázquez et al., 2013b).	Not acutely toxic in mice, LD_50_ of ethanolic leaf extract > 5,000 mg/kg (IP and PO) (Alonso-Castro et al., 2012b).	Safe and possibly effective A11
***Lippia alba*** (Mill.) N.E.Br. ex Britton & P.Wilson (Verbenaceae) – bushy matgrass – *malvareal, salviareal* Native27	12 (35)	Digestive (9); Infusions of the aromatic leaves are applied internally for diverse gastrointestinal disorders.	Broad antimicrobial (incl. a number of enteropathogens): several *in vitro* studies have demonstrated significant activity of aqueous and organic extracts, as comprehensively reviewed (Hennebelle et al., 2008). Analgesic and antiinflammatory: a few studies showed significant effects of essential oil and organic extracts in different rodent models, as comprehensively reviewed (Hennebelle et al., 2008). In a longitudinal, prospective, phase 2, non-controlled cohort study with 21 women the hydroethanolic leaf extract (chemotype geranial-carvenone) was shown effective in controlling symptoms and the impact of migraine. Dose was one drop of a 70% alcoholic tincture made from 1L of solution and 200g of fresh leaves (macerated for 10 days) per kg body weight/d over 2 months (Carmona et al., 2013).	Not acutely toxic at therapeutic doses, as reviewed (Hennebelle et al., 2008).	Safe and effective D25
***Manilkara zapota*** (L.) P.Royen (Sapotaceae) – sapodilla – *chicozapote* Native28	12 (20)	Digestive (7); Infusions of the leaves of this fruit tree are used for diverse gastrointestinal disorders.	Antidiarrheal: ethanolic leaf extract (200–400 mg/kg PO) significantly reduced defecation in castor oil-induced diarrhea model on mice (Ganguly et al., 2016). Analgesic: ethanolic leaf extract (200–400 mg/kg PO) significantly reduced nociception in mice in the writhing and tail-flick tests (Ganguly et al., 2016). Antimicrobial: significant *in vitro* activity of aqueous and methanolic leaf extracts against a number of enteropathogens (Nair and Chanda, 2008).	No signs of acute toxicity in rats after administration of ethanolic leaf extract (200–3200 mg/kg PO) (Ganguly et al., 2016).	Safe and effective D26
***Mentha* ×*piperita*** L. (Lamiaceae) – peppermint – *hierbabuena, menta* Exotic16	12 (47)	Digestive (13); Infusions of the leaves of this introduced potherb are used throughout MA to treat stomachache.	Analgesic and antispasmodic (gastric and colonic): good scientific evidence from a number of clinical trials, as comprehensively reviewed (Keifer et al., 2007).	Safe at therapeutic doses, as comprehensively reviewed (Keifer et al., 2007).	Safe and effective D27
***Momordicacharantia*** L. (Cucurbitaceae) – bitter gourd – *cunduamor/cundeamor* Exotic17	12 (33)	Endocrine (8); Infusions of aerial parts of this introduced weed are used in lowland MA to treat diabetes.	Antidiabetic: while a number of preclinical and clinical studies suggest antidiabetic activity (Habicht et al., 2014), the four RCTs standing up to the quality criteria of a recent Cochrane review found limited efficacy in glycaemia control, the risk of bias being high in all four studies (Ooi et al., 2012). A newer systematic review and meta-analysis evaluating 10 clinical studies, however, concluded that different *M. charantia* preparations improved glycemic control in T2DM patients but based on low quality evidence for the primary outcomes (Peter et al., 2019) A placebo-controlled, double-blind trial with T2DM patients showed that the application of 2,000 mg dried fruit pulp/d over 3 months decreased a range of parameters associated with diabetes such as BW, BMI, waist circumference, fat percentage, glycated hemoglobin A1c, 2-h glucose in the oral glucose tolerance test and glucose in parallel to an increase in insulin secretion (Cortez-Navarrete et al., 2018).	No serious adverse effects in four clinical trials with a total of 479 adult patients but transient effects may occur, as comprehensively reviewed (Ooi et al., 2012) but more trials assessing safety are recommended (Peter et al., 2019).	T2
***Musa* × *paradisiaca*** L. (Musaceae) – banana, plantain - *plátano, guineo* Exotic18	12 (32)	Digestive (10); skin (7); The (unripe) fruits of this introduced food staple are used against diarrhea; the fruit skin, leaves and sap are topically applied for diverse skin disorders.	Antidiarrheal: randomized, controlled trial with 80 children (1–28 months of age) hospitalized for persistent diarrhea; all outcome measures (stool output and consistency, stool weight, diarrhea duration, daily gain in body weight) improved significantly in the treatment group (n = 40) receiving a diet based on green plantains (for 7 days) in comparison to and control (n = 40) receiving a yoghurt based diet (Alvarez-Acosta et al., 2009). Antibacterial (incl. gastrointestinal and dermatological pathogens): *in vitro* MIC of 15.6–125.0 μg/ml organic leaf extracts against nine bacterial strains (Karuppiah and Mustaffa, 2013). Wound healing: banana leaf wound dressings compared positively to paraffin gauze in 3 clinical trials, as reviewed (Benskin, 2013); the topically application of methanolic stem extract significantly improved wound healing in rats in comparison to control (Amutha and Selvakumari, 2016). Antileishmaniasis: significant *in vitro* activity of different fractions of fruit peel extracts against *L*. *infantum chagasi* promastigotes and amastigotes (Accioly et al., 2012; Silva et al., 2014).	No adverse effects were reported in clinical trials using internal or topical applications (Alvarez-Acosta et al., 2009; Benskin, 2013).	Safe and effective D28, S15
***Solanum americanum*** Mill. (Solanaceae) – American black nightshade – *hierbamora* Native29	12 (37)	Skin (10); The leaves of this common leaf vegetable are topically applied for diverse skin disorders.	Broad antimicrobial (incl. dermatologic pathogens): a number of studies reported significant activity of organic extracts on diverse bacteria and fungi, as reviewed (Chauhan et al., 2012). Antileishmaniasis: methanolic leaf extracts showed *in vitro* IC_50_ of 40 μg/ml against *L*. *amazonensis* (Braga et al., 2007). Antiinflammatory: ethanolic leaf extract (400 mg/kg IP) significantly protected chickens from histamine- and carrageenan-induced inflammatory dermal lesions (Lomash et al., 2010).	Toxic upon ingestion, yet no evidence on safety of topic applications, as reviewed (Jain et al., 2011).	Effective but lacking evidence for safety S16
***Tradescantia zebrina*** Bosse (syn. *Zebrina pendula*) (Commelinaceae) – inchplant – *hierba del pollo, siempreviva, madali* Native30	12 (35)	General and unspecified (7); Macerations of the aerial parts of this common weed and ornamental are used for washings against fever.	Antibacterial: methanolic leaf extracts showed MIC values of 5–10 mg/ml against 12 Gram-positive and -negative strains (Tan et al., 2014).	ND	Lacking evidence on efficacy and safety
***Argemone mexicana*** L. (Papaveraceae) – Mexican prickly poppy – *cardosanto*, *chicalote* Native31	11 (22)	Eye (6); The latex of this common weed is dripped into the eye to treat cataracts and glaucoma.	Antibacterial (incl. ophthalmological pathogens): several studies describe the significant *in vitro* activity of aqueous and organic extracts of the stems, leaves, roots and seeds as well as the essential oil, against Gram-positive and -negative bacteria, as reviewed (Brahmachari et al., 2013; Rubio-Piña and Vázquez-Flota, 2013). Anticholinergic: the presence of alkaloids in the aerial parts and seeds, e.g., berberine, capable of blocking acetylcholine, are prescribed for the treatment of glaucoma, as reviewed (Rubio-Piña and Vázquez-Flota, 2013).	Several reviews point out the whole plant, especially the seeds, as toxic. Effects reported include dermatitis, abortifacient and neurotoxicity, mainly related with the presence of alkaloids such as berberine and saguinarine (Paul and Seaforth, 2011; Alonso-Castro et al., 2017b). Acute toxicity of the plant extracts in mice (LD_50_ 400 mg/kg, IP); hepatotoxicity of sanguinarine in mice (10 mg/kg, IP); neuroparalysis and death of several people, were reviewed (Brahmachari et al., 2013; Rubio-Piña and Vázquez-Flota, 2013).	Solutions of berberine HCl are used as eye drops. Though applying the crude latex to the eyes is possibly unsafe. Limited evidence for efficacy
***Asclepias curassavica*** L. (Apocynaceae) – tropical milkweed, bloodflower – *quebramuela*, *flor de mariposa* Native32	11 (27)	Neurological (7); skin (7); The latex is dripped onto aching teeth and topically applied to treat skin infections and insect bites throughout lowland MA.	Wound healing: a latex enzyme fraction was shown to reduce plasma-clotting time with human blood *in vitro*, to hydrolyze fibrinogen and to induce the formation of fibrin clots in a dose-dependent manner (Shivaprasad et al., 2009). Antileishmaniasis: methanolic leaf extract showed moderate activity (IC_50_ = 99 μg/ml) against *Leishmania mexicana* promastigotes *in vitro* (Peraza-Sánchez et al., 2007).	The whole plant contains cardiotoxic cardenolides (Singh and Rastogi, 1969).	Limited evidence on therapeutic benefits and highly toxic if ingested
***Capsicum annuum*** L. (Solanaceae) – chili pepper – *chile* Native33	11 (23)	General and unspecified (9); Fresh and dried fruits of this commonly cultivated culinary spice are used for a variety of disorders, particularly in ritual healing.	ND related to possible effects in ritual healing; there is however a large range of health effects of the fruits and isolated capsaicin, as extensively reviewed (Sanati et al., 2018).	Generally recognized as safe but strong irritations may occur (Johnson, 2007).	Safe but therapeutic benefits difficult to evaluate
***Cinnamomum verum*** J.Presl (Lauraceae) – cinnamon – *canela* Exotic19	11 (44)	Pregnancy (9); respiratory (9); digestive (8); The bark of this introduced spice tree is used in infusions against labor pain, respiratory tract infections, and gastrointestinal pain.	Broad antimicrobial (incl. gastroenteral and respiratory pathogens: numerous studies reported significant *in vitro* and *in vivo* effects against a wide variety of bacteria and fungi, as comprehensively reviewed (Ranasinghe et al., 2013). Antinociceptive: ethanolic bark extracts (200–400 mg/kg PO) significantly and dose-dependently reduced the nociception of mice in the writhing and hot-plate tests (Atta and Alkofahi, 1998). Antidiarrheal: aqueous bark extract (100–200 mg/kg PO) significantly reduced the intensity of castor oil- and magnesium sulfate-induced diarrhea in mice (Rao and Lakshmi, 2012).	No serious adverse effects at therapeutic doses as comprehensively reviewed (Ranasinghe et al., 2013).	Likely effective and safe D29, R11, W3
***Coffea arabica*** L. (Rubiaceae) – coffee – *café* Exotic20	11 (29)	Skin (5); The roasted and ground seeds of this introduced cash crop are applied topically to facilitate wound healing in lowland MA.	Wound healing: a carbopol hydrogel of aqueous extract from green coffee beans residual press cake (10% v/v; 0.1 g per animal, TOP, 1 × day for 14 days) significantly reduced the wound area in the mice excision wound assay using animals 9 months old, similarly to the positive control allantoin (1% w/v) (Affonso et al., 2016). In 2-month-old animals the same study failed to show an effect of the coffee extract superior to the negative control.	No signs of acute or sub-chronic toxicity of whole fruit powder, aqueous and hydroalcoholic extracts (up to 4,000 mg/kg/d PO for up to 90 days) in rats. Further, no mutagenic effects of the extracts were found with *S. typhimurium* and *E. coli* strains and no genotoxicity with the micronucleus test using murine peripheral cells (Heimbach et al., 2010). No topic safety studies have been performed.	Limited evidence on therapeutic benefits and safety
***Crescentia cujete*** L. (Bignoniaceae) – calabash tree – *morro*, *jícara* Native34	11 (29)	Respiratory (8); Decoctions and syrups of the fruit are orally administered against cough throughout lowland MA.	Antibacterial: ethanolic and chloroform leaf and bark extracts (100–200 μg/disc) *in vitro* inhibited the growth of *Staphylococcus aureus* and *Escherichia coli* with zones of inhibition of 8–29 mm (Parvin et al., 2015). Antiinflammatory: ethanolic leaf and bark extracts (0.1–1 mg/ml) demonstrated significant and dose-dependent effects in the human red blood cell membrane stabilization test (Parvin et al., 2015).	ND	Limited evidence on therapeutic benefits and lack of evidence on safety
***Hibiscusrosa*-*sinensis*** L. (Malvaceae) – Chinese hibiscus – *tulipán* Exotic21	11 (24)	Respiratory (5); general and unspecified (5); The petals of this introduced ornamental are topically applied or drunk in infusions against coughs and fevers and used in ritual healing.	Antipyretic: ethanolic flower extract (125, 250, 500 mg/kg PO) dose-dependently reduced experimentally induced increases of rectal temperature in rats (Birari et al., 2009). Antiinflammatory: ethanolic flower extract (125, 250, 500 mg/kg PO) dose-dependently reduced carrageenan-induced rat paw edema. At 250 and 500 mg/kg the extract significantly reduced xylene-induced ear edema as well as cotton pellet-induced granuloma formation in rats (Birari et al., 2009). Analgesic: ethanolic flower extract (125, 250, 500 mg/kg PO) dose-dependently reduced acetic acid-induced writhing as well as formalin-induced paw licking in mice (Birari et al., 2009).	Ethanolic and hydroalcoholic extracts of the leaves and aerial parts showed LD_50_ values above 1,000 mg/kg (IP) in rodents, as reviewed (Jadhav et al., 2009). The safety of flowers and topic administration has not been evaluated.	Limited evidence of safety and efficacy A12
***Mimosapudica*** L. (Fabaceae) – sensitive plant, touch-me-not – *dormilona*, *vergonzosa* Native35	11 (28)	Psychological (8); This pantropical weed, well known for its rapid leaf movement, is used in washings, infusions and ritual healing to treat anxiety and insomnia in lowland MA.	Anxiolytic and antidepressant: aqueous and organic solvent leaf and root extracts in rel. high doses (200–2,000 mg/kg PO) showed significant, dose-dependent anxiolytic- and antidepressant-like effects in a number of rodent models (e.g., Ayissi Mbomo et al., 2011; Patro et al., 2016; Shashikumara et al., 2018).	The leaves petroleum ether extract shows no signs of acute toxicity in rat Lorke test (2,000 mg/kg PO) (Patro et al., 2016). Ethanolic root extract did not show acute toxicity in mice (5,000 mg/kg PO) (Shashikumara et al., 2018).	Possibly safe but limited evidence for therapeutic benefits
***Mirabilisjalapa*** L. (Nyctaginaceae) – four o’clock flower – *maravilla*, *flor de linda tarde* Native36	11 (30)	Skin (6); general and unspecified (6); The petals and leaves of this common weed and ornamental are topically applied to treat skin infections and fever and are used in ritual healing.	Antimicrobial (incl. dermatological pathogens): the methanolic extract of aerial parts showed low inhibitory activity (MIC 1 mg/ml) against Gram-positive and -negative bacteria (Zachariah et al., 2012). The ethanolic extract of the aerial parts displayed a slight activity against multi-drug resistant bacterial strains (6 – 18 mm inhibition diameter) (Oskay et al., 2009). Wound healing: aqueous and ethanolic extracts of aerial parts (25 and 50 µg/ml) significantly promoted keratinocyte growth in the MTT assay (Alerico et al., 2015). Antiinflammatory: ethanolic flower extract (100–200 mg/kg PO) significantly reduced CFA-induced rat paw edema (Augustine et al., 2013).	The ethanolic flower extract (2,000 mg/kg PO) did not show any signs of acute toxicity in rats (Augustine et al., 2013).	Possibly safe and effective for dermatological conditions; no evidence for antipyretic activity S17
***Neurolaenalobata*** (L.) R.Br. ex Cass. (Asteraceae) – *tres puntas*, *mano de lagarto* Native37	11 (19)	Skin (6); The leaves of this intensely bitter weed are topically applied to treat skin infections and insect bites.	Wound healing: significant improvements of various parameters in the rat wound excision model after treatment with ethanolic leaf extract (1:1 petroleum jelly, 100 mg/kg/d for 13 days) (Nayak et al., 2014). The sequiterpenelactones present in the leaves have shown potent antiinflammatory properties *in vitro* (reduced LPS stimulated TNF-α production) and in carrageenan-induced paw edema in rats receiving 60 mg/kg MeOH extract IP (Walshe-Roussel et al., 2013; McKinnon et al., 2014). Antimicrobial (incl. dermatological pathogens): several *in vitro* studies showed significant effects of ethanolic leaf extracts against various bacterial and fungal strains (Cáceres et al., 1998; Lentz et al., 1998; Nayak et al., 2014). Antileishmaniasis: significant *in vitro* activity of ethanolic extracts of different plant parts against promastigotes (Chinchilla-Carmona et al., 2014).	No signs of acute or sub-acute toxicity of ethanolic and hydroalcoholic leaf extracts in mice (1–5 g/kg PO; 500 mg/kg every second day for 21 days) (Cáceres et al., 1998; Gracioso et al., 1998)	Possibly safe and therapeutically effective S18
***Ocimumcampechianum*** Mill. (syn. *O*. *micranthum*) (Lamiaceae) – *albahaca cimarrón* Native38	11 (24)	Neurological (4); general and unspecified (4); The aerial parts of this aromatic weed are topically applied or drunk in infusions against headache and fatigue as well as used in ritual healing.	Analgesic: the essential oil (15–100 mg/kg PO) showed significant, dose-dependent antinociceptive activity in the acetic acid-induced writhing and formalin-induced licking tests in mice yet not in the hot plate test (De Pinho et al., 2012).	No signs of acute toxicity in mice receiving essential oil (1 – 5 g/kg PO) (De Pinho et al., 2012).	Possibly safe and effective A13, N2
***Parmentieraaculeata*** (Kunth) Seem. (syn. *P*. *edulis*) (Bignoniaceae) – *cuajilote* Native39	11 (38)	Respiratory (8): The fruits of this common shrub or small tree are split open, heated and topically applied to the chest or boiled and eaten to treat respiratory tract infections.	ND	ND	Lacking evidence for safety and efficacy
***Piperamalago*** L. (Piperaceae) – *cordoncillo* Native40	11 (24)	Skin (6); general and unspecified (6); The leaves of this and several other *Piper* spp. known as *cordoncillo* are topically applied to treat skin infections and inflammations and fever as well as being used in ritual healing.	Antimicrobial: methanolic extract of the leaves exhibit limited activity against *E. coli* (MIC 512 µg/ml) and *S. aureus* (MIC1024 µg/ml) (Yasunaka et al., 2005). The essential oil of the leaves did not show antifungal activity at evaluated conc. (0.48 – 1,000 µg/ml) (Giannetti et al., 2010). Antileishmaniasis: two amides isolated from the chloroform extract of the leaves showed significant activity (IC_50_ of 20±0.88 and 15±0.25 µM) against promastigotes *in vitro* (Carrara et al., 2013). Analgesic: ethanolic leaf extract (100 mg/kg PO) and two isolated pyrrolidine amides (1 mg/kg PO) showed significant antinociceptive activity on mice in several pain models (da Silva Arrigo et al., 2016). Anxiolytic: ethanolic leaf extract 8–75 mg/kg PO) showed significant, dose-dependent anxiolytic-like effects in several rodent models (Mullally et al., 2016).	ND	Limited evidence for therapeutic benefits and lacking evidence on safety A14, S19
***Rutachalepensis*** L. (Rutaceae) – fringed rue – *ruda* Exotic22	11 (58)	General and unspecified (10); digestive (9); neurological (9); The aerial parts of this introduced aromatic plant – as well as its close relative *R. graveolens* (together cited in 20 studies with 109 use-records) – are used throughout MA in infusions, topically applied macerations, washings and in ritual healing to alleviate fever, diarrhea, pain and general illness.	Antipyretic: ethanolic extract of the aerial parts (10–500 mg/kg IP) dose-dependently reduced yeast-induced hypothermia in mice (Al-Said et al., 1990) Analgesic: ethanolic extract of the aerial parts (3–300 mg/kg PO) failed to show antinociceptive activity in the hot plate and writhing tests yet showed significant, dose-dependent effects in the mouse formalin test (Al-Said et al., 1990; Gonzalez-Trujano et al., 2006). Antimicrobial (incl. enteropathogens): a number of *in vitro* studies demonstrated significant effects of aqueous and alcoholic extracts of the leaves and aerial parts as well as the essential oil on a broad range of pathogenic bacteria and fungi (e.g., Alzoreky and Nakahara, 2003; Alanís et al., 2005; Cho et al., 2005; Haddouchi et al., 2013; Ghazghazi et al., 2015; Kacem et al., 2015; Alotaibi et al., 2018). Antiprotozoal: ethanolic extract of aerial parts showed an IC_50_ of 62 μg/ml against *Entamoeba histolytica* and 38 μg/ml against *Giardia lamblia* while metronidazole an IC_50_ of 0.04 and 0.21 μg/ml, respectively (Calzada et al., 2006). Antidiarrheal: ethanolic extract of areal parts (300 mg/kg PO) significantly inhibited cholera toxin-induced intestinal secretion in the rat jejunal loops model (Velázquez et al., 2006). Antiinflammatory: ethanolic extract of the aerial parts (500 mg/kg PO) significantly reduced carrageenan-induced paw edema and cotton pellet-induced granuloma formation in rats (Al-Said et al., 1990).	Embryotoxic effects in mice were demonstrated with aqueous extracts (10–1,600 mg/kg/d), administered PO on days 1–14 post-coitus and IP days 9–17 post-coitus (Zeichen de Sa et al., 2000; Gonzales et al., 2007). The fresh plant and extracts cause contact dermatitis through phototoxic reactions (Brener and Friedman, 1985; Gonçalo et al., 1989). Extracts of the aerial parts in mice showed LD_50_ values > 2,500 mg/kg (methanolic IP) and > 5,000 mg/kg (ethanolic PO) (Aguilar-Santamaría and Tortoriello, 1996; Gonzalez-Trujano et al., 2006).	Possibly effective yet unsafe in pregnancy and for topic administration
***Sechium* edule** (Jacq.) Sw. (Cucurbitaceae) – *chayote*, *güisquil* Native41	11 (24)	Urological (6); The consumption of cooked fruits and young leaves of this common vegetable is recommended to alleviate urinary tract infections and kidney stones.	Antimicrobial: ethanolic leaf extracts exhibited significant activity on a large range of Gram-positive (MIC 4.16–8.32 µg/ml), Gram-negative (MIC 20–160 µg/ml) antibiotic-resistant bacteria and fungi (MIC 20 – 800 µg/ml), all of them related with urinary tract infections and renal complications (Ordoñez et al., 2003; Ordoñez et al., 2009). Nephroprotective: aqueous leaf extracts (200 mg/kg, PO) significantly protected rats against gentamicin- and potassium dichromate-induced nephrotoxicity and streptozotocin-induced diabetic nephropathy; showing a significant decrease of blood urea, blood urea nitrogen, serum uric acid and serum creatinine as well as improving the kidney histology (Mumtaz et al., 2013).	The methanolic fruit extract shows no signs of acute toxicity in mice Lorke test (<5,000 mg/kg PO) (Aguiñiga-Sánchez et al., 2017). No toxic effect of aqueous leaf and stem extracts when tested in bacterial and human lymphocyte systems and no genotoxic effect in Ames test (Yen et al., 2001; Ordoñez et al., 2009).	Possibly safe and effective U2
***Sennaoccidentalis*** (L.) Link (syn. *Cassia occidentalis*) (Fabaceae) – coffee senna – *frijolillo*, *hormiguillo* Native42	11 (29)	General and unspecified (7); Decoctions of the aerial parts of this common weed are used in washings to alleviate general fatigue and body pain.	The phytochemistry and the pharmacological data obtained with extracts was reviewed (Yadav et al., 2010) but none with a specificity associated to the main uses in MA.	The seeds are highly toxic (e.g., Barbosa-Ferreira et al., 2005). Hydroalcoholic extract of the aerial parts produced no signs of acute (5g/kg PO) or sub-acute (2.5 g/kg/d for 30 days PO) toxicity in rats (Silva et al., 2011). Topical toxicity has not been evaluated.	Lack of evidence on therapeutic benefits and concerns regarding safety
***Sidaacuta*** Burm.f. (Malvaceae) – common wireweed – *malva*, *escobillo* Native43	11 (36)	Digestive (7); skin (7); Decoctions of this weedy plant are drunk against gastrointestinal parasites and inflammations and used in washings against diverse skin infections.	Antimicrobial: a number of *in vitro* and *in vivo* studies have demonstrated significant activity against a broad spectrum of bacteria and fungi, including gastrointestinal pathogens, as comprehensively reviewed (Dinda et al., 2015). Antiulcer: two studies demonstrated significant effects in various GI rat models using ethanolic whole plant and leaf extracts, as comprehensively reviewed (Dinda et al., 2015).	Not acutely toxic at therapeutic doses of aqueous extract, LD_50_ of 3200 mg/kg (IP) in mice; no signs of subchronic toxicity in rats receiving aqueous extract (75–200 mg/kg/d for 28 days PO) (Konaté et al., 2012).	Possibly effective and likely safe D30, S20
***Solanumtorvum*** Sw. (Solanaceae) – prickly nightshade – *sosa*, *lavaplatos* Native44	11 (37)	Skin (6); Infusions and macerations of the leaves of this common weed are topically applied to treat skin infections and inflammations.	Antimicrobial (incl. dermatological pathogens): methanolic leaf extract showed moderate activity in disc diffusion assay at 1 mg extract per disc against several bacterial and fungal strains (Wiart et al., 2004). Antileishmaniasis: organic solvent leaf extract inhibited the proliferation of *Leishmania donovani* promastigotes *in vitro* (IC_50_ = 90 μg/ml) (Hubert et al., 2013). Antiinflammatory: aqueous leaf extract showed significant effects in several rodent models, as reviewed (Yousaf et al., 2013).	No signs of acute toxicity on liver and kidney in chickens receiving aqueous leaf extract (2 g/kg PO) (Hashemi et al., 2008).	Possibly effective and likely safe S21
***Tithoniadiversifolia*** (Hemsl.) A.Gray (Asteraceae) – Mexican sunflower, tree marigold – *árnica*, *girasol* Native45	11 (57)	Musculoskeletal (8); skin (8); general and unspecified (8); digestive (7); Decoctions of the bitter leaves of this common weed are applied orally, topically, and in washings for diverse inflammatory conditions throughout lowland MA.	Antiinflammatory: an ethanolic leaf extract (50–200 mg/kg PO) significantly and dose-dependently reduced carrageenan-induced paw edema in rats (Owoyele et al., 2004). Aqueous and acetonic leaf rinse extracts (10–150 mg/kg PO) significantly and dose-dependently reduced carrageenan-induced paw edema and croton oil-induced ear edema in mice (Chagas-Paula et al., 2011). Gastroprotective: methanolic and dichloromethanolic leaf extracts (10–100 mg/kg IG) significantly protected rats from ethanol-induced gastric ulcers (Sánchez-Mendoza et al., 2011). Leishmanicidal: an acetonic leaf rinse extract demonstrated an LD_50_ value of 1.5 μg/ml on promastigotes of *L*. *braziliensis* (de Toledo et al., 2014).	Moderately toxic; at doses below 100 mg/kg aqueous leaf extracts (10–100 mg/kg/d, IG for 90 days) induced only few alterations in hematological and biochemical parameters in rats. Acetone (10–100 mg/kg/d IG for 90 days) and ethanolic leaf extracts (400–1,600 mg/kg PO) proved toxic to liver and kidneys of rats. (Elufioye et al., 2009; Passoni et al., 2013)	Possibly effective but moderately toxic when applied orally L3, S22
***Zingiberofficinale*** Roscoe (Zingiberaceae) – ginger – *jengibre* Exotic23	11 (33)	Digestive (7); Decoctions of the rhizome of this introduced plant are drunk against stomach pain and gastrointestinal inflammations.	General gastrointestinal: Ginger is used globally and there is a large amount of clinical and preclinical evidence supporting its uses, especially against nausea, as comprehensively reviewed (Nikkhah-Bodagh et al., 2019). A number of clinical and preclinical trials have further demonstrated the analgesic and antiinflammatory activities of ginger, as comprehensively reviewed (Grzanna et al., 2005; Terry et al., 2011).	Generally regarded as safe, including during pregnancy; moderate to mild stomach upset, eructation, heartburn and nausea may occur rarely (Nikkhah-Bodagh et al., 2019).	Safe and effective D31
***Annona reticulata*** L. (Annonaceae) – custard apple, sweetsop – *anona* Native46	10 (26)	General and unspecified (8); The leaves of this common fruit tree are topically applied to reduce fever and alleviate general illness throughout lowland MA.	Antipyretic: aqueous and methanolic leaf extracts (100–200 mg/kg PO) significantly reduced brewer’s yeast-induced hyperpyrexia in rats (Jamkhande and Wattamwar, 2015; Kumar et al., 2015). Antiplasmodial: ethanolic bark extract was active against *Plasmodium falciparum* (IC_50_ = 0.29 µg/ml) but aqueous and leaf extracts were inactive (Yamthe et al., 2015).	No signs of acute toxicity of methanolic leaf extract (2,000 mg/kg PO) in rats (Kumar et al., 2015). Topical applications have not been evaluated.	Possibly safe but limited evidence for therapeutic benefits
***Baccharis inamoena*** Gardner (syn. *B*. *trinervis*) (Asteraceae) – *Santo Domingo* Native47	10 (34)	General and unspecified (7); Infusions of the aerial parts of this common weed are drunk to lower fever and used in washing and ritual healing to alleviate fatigue and general illness.	Antimicrobial and antiviral: several *in vitro* studies showed moderate activity of aqueous extracts of the aerial parts and the essential oil against pathogenic bacteria and fungi as well as against *Herpes simplex* and HI Virus strains, as reviewed (Jaramillo-García et al., 2018).	Cytotoxic and genotoxic effects of aqueous extract of the aerial parts and different organic solvent fractions were reported in different assays (Jaramillo-García et al., 2018).	Limited evidence on therapeutic benefits and possibly unsafe
***Bougainvillea glabra*** Choisy (Nyctaginaceae) – bougainvillea – *bugambilia* Exotic24	10 (22)	Respiratory (10); Most frequently cited species in this genus of common ornamental vines, the bracts of which are used to prepare antitussive infusions throughout MA.	Antimicrobial (incl. respiratory pathogens): a number of *in vitro* studies demonstrated significant but moderate activity of aqueous and organic solvent extracts of flowers and leaves of *B*. *glabra* and other *Bougainvillea* species against a range of bacterial and fungal species, as reviewed (Abarca-Vargas and Petricevich, 2018). Antiinflammatory: several studies showed significant activity of leave and flower extracts of *B*. *glabra* and other *Bougainvillea* species in mice and rats, as reviewed (Abarca-Vargas and Petricevich, 2018).	No signs of acute toxicity in rodents after oral administration of different extracts of flowers and leaves of *B*. *glabra* and other *Bougainvillea* species, as reviewed (Abarca-Vargas and Petricevich, 2018).	Limited evidence on therapeutic benefits but possibly safe R12
***Cecropia obtusifolia*** Bertol. (Urticaceae) – trumpet tree, snakewood – *guarumbo*, *guarumo*, *chancarro* Native48	10 (26)	Endocrine (7); Infusions or decoctions of the leaves of this common native tree – as well as of its close relative *C*. *peltata*, together accounting for 60 use-records in 17 studies – are drunk as an antidiabetic throughout MA.	Antidiabetic: a number of *in vitro* and *in vivo* studies reported significant effects (incl. hypoglycemic, increased glucose uptake by adipocytes, gluconeogenesis inhibition, improved glucose metabolism by liver glycogen accumulation and α-glucosidase inhibition) of aqueous and organic leaf extracts, as extensively reviewed (Andrade-Cetto and Heinrich, 2005; Alonso-Castro et al., 2008; Costa et al., 2011; Cadena-Zamudio et al., 2019; Fortis-Barrera et al., 2019). A non-controlled clinical trial (22 type 2 diabetics, 1g of dry leaf in boiling water 3 × day for 21 days PO) showed significant reductions in fasting blood glucose cholesterol and triglycerides values (Herrera-Arellano et al., 2004). As the patients also received glibenclamide at varying doses, the effects cannot be attributed to *C*. *obtusifolia* alone. An additional non-controlled clinical trial (12 type 2 diabetics, aqueous extract of 13.5 g dry leaves per day for 32 days PO) showed significant reductions in plasma glucose and glycosylated hemoglobin levels (Revilla-Monsalve et al., 2007).	Low toxicity, as an LD_50_ of 1450 mg/kg, IP for aqueous leaf extracts in mice was determined (Pérez-Guerrero et al., 2001). A daily dose of 13.5 g of dry leaves in water for 32 and 85 days in type 2 diabetics did not generate any genotoxic or cytotoxic effects in a human micronucleus assay in culture lymphocytes (Martínez-Toledo et al., 2008).	Possibly safe and effective T3
***Cissampelos pareira*** L. (Menispermaceae) – velvetleaf – *curalina*, *alcotán*, *bejuquillo*, *redondillo* Native49	10 (19)	Digestive (7); Decoctions of the intensely bitters root of this vine are drunk against stomach pain and diarrheal disorders in lowland MA.	Antidiarrheal: ethanolic root extract (25–100 mg/kg PO) significantly and dose-dependently reduced castor oil-induced diarrhea in mice, as reviewed (Semwal et al., 2014). Gastroprotective: several studies reported significant protective effects of organic solvent leaf and root extracts against experimentally-induced ulcer formation in rodents, as reviewed (Semwal et al., 2014). Antimicrobial (incl. gastrointestinal pathogens): a number of studies reported significant *in vitro* activity of aqueous and organic solvent extracts against a broad range of bacteria and fungi, as reviewed (Semwal et al., 2014). Analgesic: several studies demonstrated significant and dose-dependent effects of aqueous and hydroalcoholic leaf and root extracts in different rodent models, as reviewed and reaffirmed (Semwal et al., 2014; Singh et al., 2016). Antiinflammatory: several studies demonstrated significant and dose-dependent effects of aqueous and ethanolic leaf and root extracts in different rodent models, as reviewed (Semwal et al., 2014).	No signs of acute or chronic toxicity in mice receiving hydroalcoholic extract of the whole plant (1,000–2,000 mg/kg/d for 28 days, PO). However, the LD_50_ values determined in different studies with different extracts range between 282 and 8500 mg/kg (PO), with ethanolic root extract having the lowest value, as reviewed (Semwal et al., 2014).	Possibly safe and effective D32
***Citrus aurantiifolia*** (Christm.) Swingle (Rutaceae) – key lime – *lima*, *limón criollo* Exotic25	10 (54)	Respiratory (8); general and unspecified (8); digestive (7); The leaves and fruit skin of this commonly cultivated introduced fruit tree are prepared as infusions and macerations and drunk or used topically, in washings or in ritual healing against respiratory infections, fever, general illness, gastrointestinal pain and inflammations as well as diarrheal disorders.	Antimicrobial (incl. respiratory and gastrointestinal pathogens): several *in vitro* studies demonstrated significant activity of alcoholic and hexane extracts of the leaves and fruit peel as well as the essential oil and fruit juice on a variety of bacterial and fungal strains (Adeleye and Opiah, 2003; Meléndez and Capriles, 2006; Rahman et al., 2011; Pathan et al., 2012; Sandoval-Montemayor et al., 2012; Miller et al., 2015). Antiprotozoal: volatile components of the hexane fruit peel extract showed IC_50_ values of 34.2 -229.49 μg/ml against *Giardia lamblia*; in comparison to 0.52 μg/ml of metronidazole (Domínguez-Vigil et al., 2015). Antispasmodic: the essential oil (2–10 μg/ml) obtained from the fruit peel showed significant reduced contractions of smooth muscles from isolated rabbit jejunum and aorta (Spadaro et al., 2012).	In sensitive or allergic persons, the essential oil can cause contact dermatitis and phototoxic reactions (e.g., Swerdlin et al., 2010).	Possibly effective and safe with reservations A15, D33, R13
***Cocos nucifera*** L. (Arecaceae) – coconut – *coco* Native50	10 (22)	Digestive (9); Coconut water (the liquid endosperm of this pantropical multipurpose palm) is drunk throughout lowland MA against gastrointestinal parasites.	Anthelmintic: several studies demonstrated significant activity against intestinal nematodes in different mammals of aqueous and organic extracts of coconut husk (mesocarp) (Oliveira et al., 2009; Costa et al., 2010; Klimpel et al., 2011; Lima et al., 2015). Anthelmintic activity of coconut water has not been studied. Antibacterial: an *in vitro* study on *Streptococcus mutans* showed no zone of inhibition using both fresh and pasteurized coconut water (Rukmini et al., 2017). Gastroprotective: coconut water (2 ml/d PO for 40 days) reduced indomethacin-induced formation of gastric ulcers in rats by 39% (Nneli and Woyike, 2008).	No signs of acute toxicity in mice and rats receiving 3,000 mg/kg coconut water orally. Nor were there any signs of sub-chronic or chronic toxicity, as reviewed (Lima et al., 2015).	Likely safe and effective D34
***Coriandrum sativum*** L. (Apiaceae) – coriander, cilantro – *cilantro* Exotic26	10 (16)	Digestive (7); Infusions of the leaves or seeds of this very common introduced culinary herb are drunk against vomits, gastrointestinal pain or ulcers by speakers of Mayan and Totonac.	Antimicrobial (incl. gastrointestinal pathogens): a number of studies showed significant *in vitro* effects of essential oil, and isolated peptide as well as aqueous and organic solvent extracts of leaves and seeds against a broad range of bacteria and fungi, as comprehensively reviewed (Laribi et al., 2015; Wei et al., 2019). Anticolitis: hydroalcoholic seed extract (250–1,000 mg/kg PO) and essential oil (0.25–1 ml/kg PO) significantly and dose-dependently protected rats from acetic acid-induced colitis (Heidari et al., 2016). Anthelminthic: *in vitro* and *in vivo* studies showed moderate effects of aqueous and alcoholic extracts against *Haemonchus contortus* and *Hymenolepis nana* (Eguale et al., 2007; Hosseinzadeh et al., 2016). Analgesic and antiinflammatory: few studies showed moderate effects of aqueous and ethanolic (50–200 mg/kg PO) seed extracts in different rodent models, as reviewed (Laribi et al., 2015).	Generally regarded as safe, based on its long history of human consumption and a number of toxicity studies, as reviewed (Laribi et al., 2015; Wei et al., 2019). The no-observed-effect-level; (NOEL) for the essential oil has been reported as 160 mg/kg/day. The maternal no-observed-adverse-effect-level (NOAEL) is 250 mg/kg/day a compared to the developmental NOAEL was established as 500 mg/kg/day (Burdock and Carabin, 2009).	Possibly safe and effective D35
***Euphorbiatithymaloides*** L. (syn. *Pedilanthus pringlei* and *P*. *tithymaloides*) (Euphorbiaceae) – Christmas candle, devil’s backbone, red slipper spurge etc. – *mayorga* Native51	10 (23)	Skin (5); Infusions of the leaves or the latex of this common weed and ornamental plant are topically applied to treat sores, wounds and skin infections in MA close to the Atlantic Ocean.	Wound healing: ointments prepared with methanolic leaf extract (2.5 and 5% w/w TOP) and isolated compounds (0.25% w/w TOP) showed significant effects on a range of relevant parameters in different rat models (Ghosh et al., 2012). Antibacterial (incl. dermatological pathogens): ethanolic leaf extract showed moderate activity against some bacterial strains *in vitro* (Vidotti et al., 2006).	Taken orally it is highly toxic and known to irritate mucous membranes (Nellis, 1997). Safety of topic applications have not been assessed.	Possibly effective but toxic when ingested and lacking evidence of safety when applied on open wounds
***Gliricidia sepium*** (Jacq.) Walp. (Fabaceae) – *madrecacao*, *cocohuite* Native52	10 (26)	General and unspecified (9); In lowland MA the branches are commonly used in healing ceremonies and the macerations of the leaves are topically applied against fever.	Antimicrobial: ethanolic leaf extract showed moderate inhibition against Gram-positive *Bacillus subtilis* and the yeast *Candida albicans* in the agar well-diffusion method (Abdulaziz et al., 2019).	ND	Limited evidence for therapeutic benefits and lacking evidence on safety
***Lepidium virginicum*** L. (Brassicaceae) – least pepperwort, Virginia pepperweed – *lentejilla*, *jilipliege*, *mostaza* Native53	10 (28)	Digestive (6); Infusions of the root or the whole plant of this common weed are orally administered to alleviate gastrointestinal pain and diarrheal disorders.	Antiamoebic: benzyl glucosinolate was identified as the major antiamoebic compound from a methanolic root extract, which had an IC_50_ of 100 µg/ml against *Entamoeba histolytica* (Calzada et al., 2003).	ND	Lacking evidence on safety and limited evidence for therapeutic benefits D36
***Malvaviscusarboreus*** Cav. (Malvaceae) – Turkcap, wax mallow – *monacillo*, *tulipán de monte* Native54	10 (31)	Digestive (7); Decoctions or infusions of the root, leaves or fruits are drunk against different gastrointestinal disorders, incl. dysentery, diarrhea, constipation and pain.	Hepatoprotective: the ethyl acetate and dichloromethane fractions (300 mg/kg/d for 6 days, PO) of an ethanolic extract of the aerial parts significantly protected rats from carbon tetrachloride-induced liver injuries (Abdelhafez et al., 2018). Antibacterial (incl. gastrointestinal pathogens): aqueous leaf extract showed significant activity against six pathogenic bacteria (Rodríguez-García et al., 2019).	ND	Limited evidence on therapeutic benefits and lacking evidence on safety
***Mimosaalbida*** Willd. (Fabaceae) – *dormilona*, *uña de gato*, *vergonzosa*, *zarza* Native55	10 (30)	Skin (6); Macerations of the leaves or root of this spiny vine are topically applied against skin infections and for wound healing.	ND	No signs of acute toxicity in mice receiving aqueous root extract (3.2–400 mg/kg IP) (Rejón-Orantes et al., 2013). Safety of the leaves and topical applications have not been evaluated.	Limited evidence on safety and lack of evidence on therapeutic benefits
***Sidarhombifolia*** L. (Malvaceae) – arrowleaf sida, Indian hemp – *malva*, *escobillo* Native56	10 (35)	General and unspecified (7); Decoctions and macerations of this weedy plant are used in bathes and washings against fever.	Antiplasmodial: significant and dose-dependent effects against *P*. *berghei* in mice receiving methanolic leaf extract (200–600 mg/kg PO) (Akele, 2013).	No signs of acute toxicity upon oral administration of up to 6 g/kg as demonstrated in several studies with rodents, as comprehensively reviewed (Dinda et al., 2015).	Possibly safe but limited evidence on therapeutic benefits
***Solanumlycopersicum*** L. (Solanaceae) – tomato – *jitomate* Native57	10 (19)	General and unspecified (5); The leaves of this very common food plant are topically applied to alleviate fever, against mumps and folk illnesses of children.	Antiinflammatory: methanolic leaf extract significantly and dose-dependently inhibited prostaglandin E_2_ production and cyclooxygenase-2 gene expression *in vitro* (Amid et al., 2011).	No signs of acute toxicity in mice receiving ethanolic leaf extract (2 g/kg PO) (Shukla and Kumar, 2015). Dermatitis upon contact with the leaves has been described and the glycoalkaloid solanine, present in the leaves, gives rise to safety concerns (Germosén-Robineau, 2005).	Concerns regarding safety and very limited evidence on therapeutic benefits
***Tamarindusindica*** L. (Fabaceae) – tamarind – *tamarindo* Exotic27	10 (26)	General and unspecified (8); Macerations of the leaves of this introduced tree, commonly cultivated for its edible fruit pulp, are topically applied or used in washings to alleviate fever.	Antiplasmodial: aqueous stem bark extract (100 mg/kg/d for 4 days PO) reduced *P. berghei* parasitemia in mice by 30% (Mwangi et al., 2015). Febrifuge or antimalarial activities of the leaves have not been studied.	No signs of acute toxicity in rats receiving hydroalcoholic leaf extract (2,000 mg/ml PO) (Escalona-Arranz et al., 2016). Safety of topical administration has not been studied.	Limited evidence on safety and therapeutic benefits
***Artemisia absinthium*** L. (Asteraceae) – absinthe wormwood – *ajenjo* Exotic28	9 (23)	Digestive (9); Infusions of the leaves or aerial parts of this introduced aromatic plant are drunk to alleviate gastrointestinal pain, cramps and diarrheal disorders throughout MA.	General gastrointestinal: Some clinical evidence indicates that preparations from the aerial parts are superior to placebo in reducing symptoms of inflammatory bowel disease. There is also some clinical evidence that herbal extracts can be effective in the treatment of amoebiasis and increase biliary, gastric and intestinal excretion of. Further, several *in vivo* studies showed that aqueous and organic solvent extracts of the aerial parts had significant antiulcer, hepatoprotective effects and increased the excretion of bile and gastric juices, as comprehensively reviewed (EMA/HMPC, 2017). Broad antimicrobial (incl. gastrointestinal pathogens): several *in vitro* studies showed significant effects of the essential oil against a number of bacterial and fungal strains, as comprehensively reviewed (EMA/HMPC, 2017).	No toxic effects were observed in animal studies using herbal extracts. The isolated compound thujone, however is known to be toxic at higher doses and there is a lack of studies on reproductive and developmental toxicity. Thus, herbal products should not be used in larger amounts or extended periods and not at all during pregnancy and lactation or in children and adolescents, as comprehensively reviewed (EMA/HMPC, 2017).	Possibly effective and safe with reservations D37
***Begonia heracleifolia*** Cham. & Schltdl. (Begoniaceae) – star begonia – *caña agria*, *mano de león* Native58	9 (21)	Skin (5); The crushed rhizomes are topically applied to treat snakebites.	ND	ND	Lacking evidence on both safety and therapeutic benefits
***Brugmansia* ×*candida*** Pers. (Solanaceae) – angel’s trumpet – *flor de campana*, *floripondio* Exotic29	9 (22)	Skin (5); The most frequently cited of different *Brugmansia* spp., the leaves of which are topically applied to alleviate skin infections and inflammations.	Antibacterial (not incl. dermatological pathogens): an isolated peptide showed significant activity against Gram-positive and -negative bacteria *in vitro* (Kaewklom et al., 2018).	The whole plant contains highly toxic tropane alkaloids, which can result in severe poisoning and death after ingestion (e.g., Isbister et al., 2003; Kim et al., 2014). Topical toxicity has not been evaluated.	Toxic upon ingestion and very limited evidence on therapeutic benefits
***Cornutia pyramidata*** L. (syn. *C*. *grandiflora*) (Lamiaceae) – *carreto*, *piojillo*, *tabaquillo* Native59	9 (17)	General and unspecified (6); The leaves are used in ritual healing and macerations as washings to reduce fever.	Antiplasmodial: isolated diterpenoids from the leaves showed only marginal activity against two strains of *Plasmodium falciparum* (Jenett-Siems et al., 2003).	ND	Lack of evidence on safety and therapeutic benefits
***Datura stramonium*** L. (Solanaceae) – jimsonweed – *toloache* Native60	9 (23)	Skin (5); One of several *Datura* spp. (together cited in 13 studies with 35 use-records) the leaves of which are topically applied to treat infectious and inflammatory skin disorders.	Antiinflammatory: ethanolic leaf extract (200 mg/kg, PO) showed significantly reduced carrageenan-induced rat paw edema, as reviewed (Gaire and Subedi, 2013). Antibacterial (incl. dermatological pathogens): methanolic, ethanolic and aqueous extracts of the aerial parts significantly inhibited the growth of different Gram-positive and -negative bacteria, as reviewed (Gaire and Subedi, 2013).	Oral and systemic administration may lead to severe toxicity, characterized by anticholinergic symptoms, as comprehensively reviewed (Krenzelok, 2010; Gaire and Subedi, 2013); topic administrations have not been evaluated.	Toxic upon ingestion and very limited evidence on therapeutic benefits
***Dorstenia contrajerva*** L. (Moraceae) – snakewort – *contrayerba*, *cresta de gallo*, *hoja de sapo* Native61	9 (19)	Skin (5); The inflorescence, leaves or root are topically applied to treat warts and snakebites in lowland Zoquean and Mayan communities.	ND	ND, although the presence of furanocoumarins (Cáceres et al., 2001) might raise concerns of phytophototoxicity.	Lack of evidence on safety and therapeutic benefits
***Eryngium foetidum*** L. (Apiaceae) – Mexican coriander – *culantro*, *cilantro cimarrón*, *perejil* Native62	9 (31)	Digestive (5); Decoctions or infusions of the root or leaves of this common culinary herb are used to treat gastrointestinal pain and inflammations as well as diarrheal disorders.	Antibacterial (incl. gastrointestinal pathogens): a few *in vitro* studies demonstrated limited but significant activity of alcoholic leaf extracts against Gram-positive and –negative bacteria (Paul et al., 2011; Kouitcheu-Mabeku et al., 2016; Panda et al., 2016). Antiinflammatory: several studies showed significant effects of different leaf extracts in various *in vitro* and *in vivo* models (Paul et al., 2011; Mekhora et al., 2012; Dawilai et al., 2013). Anthelminthic: two *in vitro* studies showed significant effects of methanolic leaf extract and refined plant extract rich in eryngial against *Paramphistomum* sp. and *Strongyloides stercoralis* respectively (Paul et al., 2011; Swargiary et al., 2016).	No signs of serious chronic toxicity in mice receiving a diet supplemented with ground leaves (0.8%–3.2% for 24 weeks), although at elevated doses some adverse effects on kidney function were observed (Janwitthayanuchit et al., 2016).	Likely safe but limited evidence on therapeutic benefits D38
***Foeniculumvulgare*** Mill. (Apiaceae) – fennel – *hinojo* Exotic30	9 (24)	Digestive (8); Infusions of the aerial parts and fruits of this introduced aromatic plant are orally administered to treat gastrointestinal pain and vomiting.	General gastrointestinal: observational, *in vivo* and *in vitro* studies support the use of fennel fruit in the treatment of mild gastrointestinal complaints, especially spasmodic ailments and bloating. Further, several preclinical studies showed significant hepatoprotective, antiinflammatory and antinociceptive effects as well as relaxing effects on isolated smooth muscles, as comprehensively reviewed (EMA/HMPC, 2008; Badgujar et al., 2014). Broad antimicrobial (incl. gastrointestinal pathogens): a number of *in vitro* studies demonstrated significant effects of the essential oil and diverse extracts against a variety of bacterial and fungal strains, as comprehensively reviewed (EMA/HMPC, 2008; Badgujar et al., 2014). The reviewed studies mostly refer to the fruits; the leaves, which are frequently used in MA have received much less attention.	No signs of serious acute or chronic toxicity were recorded in several *in vivo* studies with doses up to 3g/kg (PO). Due to the lack of studies, however, the medicinal use of fennel should be avoided in children under 4 years of age, as comprehensively reviewed (EMA/HMPC, 2008; Badgujar et al., 2014).	Possibly safe and good evidence for therapeutic benefits D39
***Litseaglaucescens*** Kunth (Lauraceae) – Mexican bay leaf – *laurel* Native63	9 (26)	Digestive (6); general and unspecified (6); The leaves of this culinary condiment are used in ritual healing and infusions or decoctions are drunk to alleviate gastrointestinal pain and diarrheal disorders.	Antispasmodic: methanolic leaf extract exerted a slight inhibition (IC_50_ 885 ± 125 µg/ml) of isolated rabbit jejunum muscular contractility (Arroyo et al., 2004). Antiinflammatory: ethanolic leaf extract (3, 30, 100 mg/kg IP) showed significantly reduced carrageenan-induced rat paw edema in mice and was also active (30 mg/kg IP) in carrageenan induced pleurisy in mice (Simão da Silva et al., 2012). Antihyperalgesic: ethanolic leaf extract (30 mg/kg IP) exerted a significant preventive effect on mechanical hyperalgesia induced by partial sciatic nerve ligation in mice (Simão da Silva et al., 2012). Antibacterial (incl. enteropathogens): methanolic leaf extracts showed moderate activity (*S. aureus* MIC_50_ 400 µg/ml, *E. coli* MIC_50_ >1,000 µg/ml) (López-Romero et al., 2018). Antidepressant: essential oil (100 - 300 mg/kg IP) showed antidepressant-like activity in different mice models, as reviewed (Guzmán-Gutiérrez et al., 2014).	ND	Lacking evidence of safety and good evidence on therapeutic benefits D40
***Partheniumhysterophorus*** L. (Asteraceae) – Santa Maria feverfew – *hierba maestra*, *altamisa* Native64	9 (25)	General and unspecified (7); Infusions of the aerial parts of this common weed are orally administered to reduce fever.	Antiplasmodial: hydroalcoholic extract of the root showed partial activity (IC_50_ 45.2 µg/ml) against chloroquine-susceptible *Plasmodium falciparum* Ghana strain (Valdés et al., 2010).	Systemic toxic effects on grazing animals have been repeatedly reported, as reviewed (Patel, 2011). Genotoxicity of crude extracts and the isolated sesquiterpene lactone parthenin demonstrated in several studies (e.g., Ramos et al., 2001; Ramos et al., 2002). Known to cause contact dermatitis (e.g., Sharma and Sethuraman, 2007).	Unsafe and limited evidence on therapeutic benefits
***Plumeriarubra*** L. (Apocynaceae) – frangipani – *flor de mayo*, *cacalosúchil* Native65	9 (22)	Digestive (5); An infusion of the flowers or decoction of the bark of this common ornamental tree is orally administered to treat gastrointestinal pain, particularly the folk illness *empacho*.	Gastroprotective: the water-soluble protein fraction of the latex (0.5–50 mg/kg IV) significantly protected mice from ethanol-induced gastric lesions (de Alencar et al., 2015). Antimicrobial (not incl. gastrointestinal pathogens): all but 1 of 13 isolated compounds from the stem bark exhibited *in vitro* antibacterial, antifungal and/or antialgal activities (Kuigoua et al., 2010).	ND	Limited evidence on therapeutic benefits and lack of evidence on safety
***Pouteriasapota*** (Jacq.) H.E.Moore & Stearn (Sapotaceae) – mamey sapota – *mamey*, *zapote* (*colorado*) Native66	9 (17)	Skin (7); Macerations of the seed are topically applied to treat skin infections, dandruff and hair loss in lowland MA.	ND	No signs of dermal toxicity, irritation or edema in rabbits receiving aqueous or hydroalcoholic seed extract (200 mg/6 cm^2^ patch TOP). Both extracts (40 mg/eye) caused mild and reversible eye irritation in rabbits. Acute IG administration of seed extracts (300–2,000 mg/kg) to rats resulted in cyanosis, rectal bleeding and death (Dutok et al., 2015).	Possibly safe for topic administration yet lacking evidence on efficacy
***Tagetesfilifolia*** Lag. (Asteraceae) – liquorice herb, sweet Irish lace – *anís del monte* Native67	9 (17)	Digestive (8); Infusions of the aerial parts of this strongly scented weed are orally administered to alleviate gastrointestinal pains, cramps and diarrheal disorders.	Anthelminthic: methanolic leaf extract showed a significant effect against *Haemonchus contortus* larvae and eggs *in vitro* (Jasso Díaz et al., 2017). Antimicrobial: the 50% ethanol leaf extract inhibited moderately the growth of *Salmonella typhi* (≥ to 9mm) and *Shigella flexneri* (6 ≥ 8) in the disc diffusion assay (Cáceres et al., 1990).	ND	Very limited data on therapeutic benefits and lack of evidence on safety
***Theobromacacao*** L. (Malvaceae) – cacao, cocoa – *cacao* Native68	9 (13)	Pregnancy (4); general and unspecified (4); A hot drink prepared from the roasted seeds of this sacred and commonly cultivated fruit tree is drunk to induce and facilitate childbirth. The fruits and seeds are also important in ritual healing.	Pregnancy and childbirth: Through the increase of endogenous cyclic 3’,5’-AMP levels, caffeine is able to antagonize the response to oxytocin in rat uterine smooth muscle (Mitznegg et al., 1970). A prospective cohort study found that regular chocolate consumption (≥ 1–3 servings/week) during the first and third trimester of pregnancy is associated with significantly reduced risk of preeclampsia when compared to women who reported no regular chocolate consumption in these periods. Regular chocolate consumption, but only during the first trimester, was also associated with reduced odds gestational hypertension (Saftlas et al., 2010). Another prospective cohort study found umbilical cord serum theobromine concentrations negatively associated with preeclampsia and first and third trimester chocolate consumption associated with reduced preeclampsia risk (Triche et al., 2008)	A dietary survey monitoring the intake of theobromine, caffeine and theophylline were not found to have negative effects in pregnant women or on the fetus (Tebbutt et al., 1984).	Likely safe yet limited evidence on therapeutic benefits W4

The species are ranked in order of the number of studies citing and alphabetically sorted among species with the same number of citations. As predominant uses, all ICPC categories with at least seven citing studies are included or alternatively the most frequently cited category if no category is cited in at least seven studies. Abbreviations are as follows: day (d), female (f), male (m) inhibitory concentration (IC), intragastric (IG), intradermal (ID), intravenous (IV), oral (PO), maximal zones of inhibition (MZI), minimal inhibitory concentration (MIC), randomized controlled trial (RCT), topically (TOP). For ICPC key to A, D, L, N, P, R, S, T, U, W, and X (Column “Evaluation”) see **Table 2**. For references of use-records please see **Table 4.1** in the **Supplementary Material** and **Table 1**.

## Results and Discussion

### The Mesoamerican Medicinal Plant Database (MAMPDB)

The MAMPDB includes a total of 12,537 use-records for 2188 taxa (**Table 2**), including 1929 species and 259 taxa identified to the genus level only (**Table 4.1** in the **Supplementary Material**), 995 genera (**Table 4.2** in the **Supplementary Material**), and 185 families (**Table 4.3** in the S**upplementary Material**). For more than half of the species (1,100; 57%) no cross-cultural consensus does exist and 36% of the genera are only recorded in one of the 28 studies incorporated into the database.

In the different ICPC categories herbal medicine and pharmaceuticals are not considered to the same extent appropriate solutions for the treatment of the various health problems. Therefore, the number of taxa and use-records associated with these categories do not directly reflect the epidemiological situation in rural MA but can provide some information. Several categories are poorly recognized in ethnomedical systems such as “B” (blood, blood forming organs and immune mechanism), “K” (Cardiovascular) and “T” (endocrine/metabolic and nutritional) while “Z” (social problems) is unlikely to be treated with medicine. Typically, the broad categories “digestive” (D) and “skin” (S) are among those with the highest number of associated medicinal plants and use-records (**Table 2**). Musculoskeletal ailments (L) are often treated with massages by traditional healers called ‘*sobadores*’ or ‘*ajpamaj*’ (Ankli et al., 1999; Leonti et al., 2001; Berger-González et al., 2016b; Geck et al., 2016) while midwifes give massages for problems of the lower female organs (X) (Weimann and Heinrich, 1997; Ankli et al., 1999; Michel et al., 2016). Measures for family planning (W) are provided by the IMSS in collaboration with MEXFAM. For treating and preventing several ailments of the urological system (U) it is important to drink copiously and flush the urinary tract. This means that ailments and interventions with respect to the categories B, L, K, T, U, W, X, and Z are probably more frequent than suggested by the number of use-records related to botanical drugs.

The many species of Asteraceae (226), Fabaceae (194), Euphorbiaceae (85) and Lamiaceae (79) reported for medicinal purposes in the 28 studies (**Table 4.3** in the **Supplementary Material**) reflect the floristic representation of these plant families in the region (see Bye and Linares, 2015, p. 394). The use consensus of Asteraceae is on digestive (D; 54% spp./19.5% use-records) and inflammatory skin disorders (S; 47.8% spp./12.8% use-records) but the characteristic presence of cytotoxic sesquiterpene lactones in this family can lead to allergenic reactions (Siedle et al., 2004). The family with the highest share of species for skin disorders in the MAMPDB are the Solanaceae (66.2% spp./20.8% use-records) and the Euphorbiaceae (64.7% spp./24.7% use-records.), the latter notorious for their proinflammatory properties (Evans and Taylor, 1983). The family showing the highest share for A ‘unspecified’ are the Solanaceae (61.8% spp./18.6% use-records), Lamiaceae are most frequently used for digestive problems (D; 73.4% spp./20% use-records) and Fabaceae for skin disorders (S; 51% spp./15.6% use-records).

### Consensus Analysis

The consensus of those 98 species, for which a therapeutic use has been documented in at least nine (one third taken as an arbitrary threshold value) of the 28 independent studies is presented in **Table 3** together with a critical evaluation of the pharmacological and toxicological evidence based on existing literature. It highlights the most frequently cited taxa in the MAMPDB, which have roots in Olmec, Maya, Zapotec and Aztec as well as traditional Mediterranean medicine. Most of the 98 herbal drug species are either grown in home-gardens or thrive in the vegetation surrounding the villages and are thus easily available. Several are also sold on local markets for food purposes, including spices, herbs, vegetables and fruits. Of the 98 species 68 are native to MA (**Table 3**). Of those 22 considered effective and safe for skin problems (S) 17 are native to MA while of the 13 considered effective and safe for respiratory ailments (R) only 3 are native. For digestive (D), skin (S) and respiratory (R) problems as well as for the category ‘general and unspecified’ (A) among the 98 species a range of effective and safe herbal drug-based treatment options exist (**Table 3**).

Typically, many of the herbal drugs used against diarrhea (D) are rich in tannins and polyphenols (Heinrich, 1998), such as the bark of *Byrsonima crassifolia, Guazuma ulmifolia*, and *Mangifera* *indica*, leaves of *Psidium guajava* and *Mangifera* *indica*, unripe fruits of *Musa × paradisiaca* and fruit skin of *Punica* *granatum.* Those used altogether for gastrointestinal plain, cramps and diarrhea such as the leaves of *Eryngium foetidum* and *Litsea* *glaucescens*, the herb of *Artemisia absinthium*, the root of *Cissampelos pareira* and the zest of *Citrus aurantiifolia* are aromatic and/or bitter tasting drugs. Those herbal drugs effective and safe used for category ‘A’ fall under the sub-category of ‘general pain’, ‘weakness’ and ‘feeling ill’ (ICPC) and include those herbal species also used in ritual healing. These are often aromatic, essential oil-bearing plants such as *Ocimum basilicum*, *O. campechianum*, *Tagetes lucida* or *Piper amalago*. The ritual cleansing ceremony called “limpia” using aromatic herbs and lotions to brush away bad spirits from the patient is a typical therapeutic practice in MA (Zamora-Martínez and Pascual Pola, 1992). The leaves of *Ocimum* spp. are used either as an infusion or applied topically against headache (N) while the flowers and zest of *Citrus sinensis* and *C. aurantium* serve as infusions to treat anxiety and stress (P). The practice of using essential oil rich herbal drugs for treating the ICPC categories A, P and N remind of aromatherapy, which has been found effective in clinical trials focusing on stress and anxiety related disorders (Perry and Perry, 2006; Linck et al., 2010). There seems to be a lack of safe and effective diuretics as well as disinfectants of the urinary tract (only corn silk and fruits of *Sechium edule* (chayote) among **Table 3**). For muscular problems (L) nowadays often massages with commercialized balms are used and assistance during pregnancy, childbearing and family planning (W) is provided in Mexico by the IMSS in collaboration with trained local midwifes.

For several important medicinal species there is very limited evidence for either safety or efficacy or altogether, including but not limited to *Tagetes erecta*, *Piper auritum*, *Byrsonima crassifolia*, *Bursera simaruba*, *Artemisia ludoviciana*, *Hyptis verticillata*, *Spondias purpurea Tradescantia zebrina Crescentia cujete, Parmentiera aculeata, Piper amalago, Baccharis inamoena, Bougainvillea glabra, Lepidium virginicum, Malvaviscus arboreus, Mimosa albida, Solanum lycopersicum, Cornutia pyramidata, Dorstenia contrajerva, Tagetes filifolia* and *Jatropha curcas* (**Table 3**). This situation emphasizes the lacking knowledge base and the concerns regarding toxicity of native botanical drug species widely used in traditional medicine throughout MA (Caceres Guido et al., 2015; Valdivia-Correa et al., 2016; Alonso-Castro et al., 2017b).

According to the WHO guidelines for the assessment of herbal medicines (WHO, 1996, p. 181) a principle for the safety assessment of herbal medicines should be the traditional use of the product “without demonstrated harm” while “no specific restrictive regulatory action should be undertaken unless new evidence demands a revised risk-benefit assessment.” Yet even though a track record of traditional medical use can provide some evidence about the safety of herbal drugs and their applications it is necessary to point out that ‘natural’ is not to be confounded with safe, a common misconception among consumers of herbal drugs (WHO, 2013).

A well-studied case of toxic plant materials are aristolochic acids containing botanical drugs deriving from the *Aristolochiaceae* (Arlt et al., 2002; Michl et al., 2014). Aristolochic acids are nephrotoxic and carcinogenic and potentially contained in botanical drugs obtained from 14 different *Aristolochia* species present in the MAMPDB. The drugs obtained from *Aristolochia* spp. are often used for digestive problems and totaled 95 use-records in the MAMPDB (**Table 4.1** in the **Supplementary Material**). The time lag between the onset of chronic intoxication and the manifestation of eventually lethal kidney disorders is the reason why the resulting pathology is difficult for the general population to associate with the consumption of *Aristolochia* spp. Clearly safety concerns prevail above all with systemic applications. Also the use of castor oil plant seeds as an emetic and purgative is risky (Aplin and Eliseo, 1997) and due to the high content of the hepatotoxic and carcinogenic safrole the leaves of *Piper auritum*, which are also widely used as a condiment for pork tamales (wrapped maize dough cakes), should be used in low doses when applied orally. Anthranoid rich material (bitterness as a proxy) of *Aloe vera* is regarded as unsafe upon prolonged oral applications and particularly during pregnancy as the resulting increased blood flow to the uterus can induce abortion (Schulz et al., 2012, p. 250). The leaves of *Bryophyllum pinnatum* contain cardiotoxic bufadienolids, the aromatic leaves of *Lantana camara* hepatotoxic metabolites, the leaves of *Phyla scaberrima* high amounts of neurotoxic camphor, and the aerial parts of *Parthenium hysterophorus* toxic sesquiterpenelactones and are thus all unsafe depending on the dose ingested. Also applying the latex of *Argemone mexicana* to the eyes, the latex of *Asclepias curassavica* to aching teeth and using *Ruta chalepensis* during pregnancy are to be regarded unsafe.

The guidelines for the “Appropriate Use of Herbal Medicines” (WHO, 1998, p. 2) state that “it is necessary to make a systematic inventory and assessment (preclinical and clinical) of medicinal plants; to introduce measures on the regulation of herbal medicines to ensure quality control of herbal products by using modern techniques, applying suitable standards and good manufacturing practices; and to include herbal medicines in the national standard or pharmacopoeia.” A relatively cost-effective way for conducting clinical trials are retrospective treatment-outcome studies (RTO studies), which use questionnaires for the collection of information from a representative sample of the population (Graz et al., 2005; Graz et al., 2007). RTO studies assess retrospectively the effectiveness of herbal preparation and treatments for defined medical syndromes and clinical manifestations (Graz et al., 2005; Willcox et al., 2011). While the case of *Aristolochia* spp. derived products showcases that herbal drugs are not always safe, those with a traditional clinical track record and above all food items, usually considered safe, qualify for RTO studies (WHO, 1998; Willcox et al., 2011).

The quantitative approach and assessment of the 98 most frequently used medicinal taxa included in the MAMPDB highlights those herbal drugs with the highest intercultural acceptance as well as those applications potentially unsafe. The higher probability of exotic herbal drugs to be considered safe and efficacious (**Table 2**) is related to the better overall research situation of herbal drugs used in the European and the US Pharmacopoeia (Martins et al., 2019). However, **Table 3** includes several native herbal drugs uses as food such as spices and culinary herbs and constitutes potential starting points for RTO studies. While the intercultural consensus can give some indications about the safety and perceived effectiveness, intriguingly Graz et al. (2005) found that the treatment of malaria in Mali showed no significant correlation between cultural consensus and the best patient progress, underlining the strength of RTOs being complementary to ethnopharmacological field studies.

The list of medicinal species reviewed in **Table 3** constitutes a data collection that can be drawn on by the Mexican Herbal Pharmacopoeia Committee for increasing the Appendix VI (list of species with medical use in Mexico). Appendix VI is the prelude to the monographs of medicinal plants contained in the Pharmacopoeia and could be used to promote a better integration of native Mesoamerican species into that regulatory document. It should also be considered that knowledge about herbal remedies is increasingly shaped by literature and popular media, through which global trends in T&CM are introduced to local communities.

## Conclusions

Through the evaluation of the MAMPDB we have highlighted a group of locally available medicinal plants, yielding products with a high inter-cultural consensus of use and track record of traditional use. However, the results from preclinical *in vitro* or *in vivo* studies are only a proxy for medical efficacy in humans. Especially for native herbal drugs data about safety and effectiveness is limited. Commonly used cross-culturally salient botanical drugs, which are considered safe but for which data on effectiveness is lacking are ideal candidates for treatment outcome studies. These could be conducted at local health clinics and in collaboration with the respective ministries of health and social security institutes. Retrospective treatment outcome studies constitute a valid tool for a culturally sensitive evaluation of traditional medicines including the psychosocial dimension of healing. Collaborations between the medical staff of local health clinics with local health workers and practitioners of traditional medicine have the potential to close cultural gaps and medical misconceptions that preclude the implementation of intercultural models of healthcare attention. It would facilitate an enhanced acceptance and integration of different medical thoughts and foster communication between traditional health practitioners, patients and health professionals trained in Western biomedicine. A closer collaboration between practitioners of biomedical and traditional medical systems has the potential to increase affordability, accessibility and cultural acceptability of health care.

Similarly, to the situation in MA, in most regions of the world, a wealth of information on traditional and complementary medicine (T&CM) has been recorded. Yet these data are often scattered, making it difficult for policy makers to regulate and integrate traditionally used botanical products while existing compendia are often outdated. Creating quantitative regional databases that are based on internationally published literature can serve as effective means in the advancement of the integration of evidence-based T&CM and contribute to achieving UHC. It also constitutes a tool for responding to changing epidemiological landscapes and consumer preferences.

## Author Contributions

MG and ML designed the review. MG, MB-G, LC, SC, MH, and ML wrote the paper.

## Funding

This project has received funding from the European Union’s Seventh Framework Program for research, technological development and demonstration under grant agreement no. 606895.

## Conflict of Interest

The authors declare that the research was conducted in the absence of any commercial or financial relationships that could be construed as a potential conflict of interest.

## Footnotes

1 Four studies (no. 15, 16, 20, 24) focus on a specific disease category, whereas medicinal uses are not detailed in study no. 17, due to concerns about intellectual property rights.
