# Supplementary material for: Traditional Herbal Medicine in Mesoamerica: Toward Its Evidence Base for Improving Universal Health Coverage
Source: Front Pharmacol. 2020 Jul 31;11:1160. doi: 10.3389/fphar.2020.01160 (PMC7411306; doi:10.3389/fphar.2020.01160)
Supplement: Supplementary file 1 [file DataSheet_1.pdf]

**Table A4.1.**
Alphabetical list of the 2188 botanical taxa used medicinally in Mesoamerica. Abbreviations are as follows: Mixe-Zoque (MZ), Zoquean (Zoq), Totonac (Tot), pan-Mayan (Maya), Huastec (Hua), Core Mayan (CoreM), Yucatecan (Yuc), Quichean (Quich), Western Mayan (WesM), Zapotec (Zap), Nahuatl (Nah); a capital "C" stands for consonant and a capital "V" for vowel. A ">" B signifies that A is the probable donor and B the probable recipient, A "<>" B signifying that no conclusion can be drawn on the directionality of the language contact.

### *Abelmoschus esculentus* (L.) Moench (Malvaceae)

Spanish names: Café chino
Indigenous names: K'inal, Xaq K'inal, Ruhi Xul[14]
Used by (2*): Yucatecan Maya[09]; Quichean Maya[14]
Used for (2#): Skin (1)[09]; General and Unspecified (1)[14]
Cognates:
Language contact:

### *Abelmoschus moschatus* Medik. (Malvaceae)

Spanish names: Algalia, almiz, curalina, semilla de víbora, Santa Elena
Indigenous names: Almiz, almisy[01]; Tsa'ant ujts[04]; Kwinim ilaal[07]; Alkalia[20]
Used by (8*): Zoque[01, 02, 03]; Mixe[04]; Totonac[05]; Huastec[07]; Western Maya[19, 20]
Used for (18#): Digestive (5)[01, 04, 07, 19, 20]; Musculoskeletal (2)[03, 07]; Respiratory (2)[03, 07]; Skin (4)[01, 02, 04, 05]; Endocrine (1)[03]; Pregnancy (1)[04]; General and Unspecified (3)[01, 03, 07]
Cognates:
Language contact:

### *Abies guatemalensis* Rehder (Pinaceae)

Spanish names:
Indigenous names: Yàg-lgâzh[23]
Used by (1*): Zapotec[23]
Used for (1#): General and Unspecified (1)[23]
Cognates:
Language contact:

### *Abrus precatorius* L. (Fabaceae)

Spanish names:
Indigenous names: Oxo[09]
Used by (1*): Yucatecan Maya[09]
Used for (1#): Digestive (1)[09]
Cognates:
Language contact:

### *Abuta panamensis* (Standl.) Krukoff & Barneby (Menispermaceae)

Spanish names:
Indigenous names:
Used by (2*): Zoque[03]; Quichean Maya[17]
Used for (2#): Female genital (1)[03]; nd[17]
Cognates:
Language contact:

### *Abutilon hypoleucum* A.Gray (Malvaceae)

Spanish names:
Indigenous names: Hupchil ts'ohool, tsakam akich, tsakam kwinim ts'ohool, thak akich, thak ch'ohool[07]
Used by (1*): Huastec[07]
Used for (1#): Digestive (1)[07]
Cognates:
Language contact:

[01-28] refer to the study codes in Table 4.1. *Total number of studies citing this taxon #Total number of use-records

***Abutilon permolle* (Willd.) Sweet (Malvaceae)**

Spanish names:
Indigenous names: Sakmisbil, Sakpetmis[09]
Used by (1*): Yucatecan Maya[09]
Used for (1#): Skin (1)[09]
Cognates:
Language contact:

***Acacia angustissima* (Mill.) Kuntze (Fabaceae)**

Spanish names: Timbre
Indigenous names: Tzut 'tzuu'n[06]; Xixit, waakal mo'eel, chak thuk'[07]; Waxim[09]; Seq' Jaqarant Aq'om[12]; Me' ja' te', xaxib, xaxib pukuj, vax te'[20]; Yàg-læ̀[23]
Used by (7*): Totonac[06]; Huastec[07]; Yucatecan Maya[09]; Quichean Maya[12]; Western Maya[20]; Zapotec[23]; Nahua[26]
Used for (18#): Digestive (5)[06, 07, 12, 20, 26]; Eye (1)[20]; Musculoskeletal (4)[06, 07, 12, 26]; Neurological (1)[07]; Psychological (1)[26]; Respiratory (1)[20]; Skin (2)[09, 20]; Pregnancy (1)[23]; Female genital (1)[12]; Male genital (1)[12]
Cognates:
Language contact: Hua <> Tzeltalan <> Yuc

***Acacia collinsii* Saff. (Fabaceae)**

Spanish names: Espina de cacho, Cornisuelo
Indigenous names: Mesit apij[01]; Subinche'[09]
Used by (2*): Zoque[01]; Yucatecan Maya[09]
Used for (4#): Musculoskeletal (1)[01]; Skin (1)[09]; Pregnancy (1)[01]; General and Unspecified (1)[01]
Cognates:
Language contact:

***Acacia cookii* Saff. (Fabaceae)**

Spanish names:
Indigenous names: Xe' subin ut xaq, Sub'in[14]
Used by (1*): Quichean Maya[14]
Used for (1#): Skin (1)[14]
Cognates:
Language contact:

***Acacia cornigera* (L.) Willd. (Fabaceae)**

Spanish names: Cornisuelo, cacho del toro
Indigenous names: Juanapitx[03]; Juag aptx[04]; Thobem, thobem k'iith, theben, ist'am toro, toro k'iith[07]; Ajsubin[18]; Guedxebej[21]
Used by (6*): Zoque[03]; Mixe[04]; Huastec[07]; Western Maya[18]; Zapotec[21]; Nahua[26]
Used for (16#): Digestive (2)[03, 07]; Ear (1)[07]; Cardiovascular (1)[07]; Neurological (1)[07]; Respiratory (3)[07, 21, 26]; Skin (2)[04, 07]; Pregnancy (1)[07]; Female genital (4)[03, 18, 21, 26]; General and Unspecified (1)[07]
Cognates: MZ: aptx;
Language contact:

***Acacia farnesiana* (L.) Willd. (Fabaceae)**

Spanish names: Huizache, cacho de buey
Indigenous names: Thuhanoom, thujanum, thuhanib[07]; Sub'in[19]; Guish guiatzj[21]; Yàg-guièts-clâv-nquǐts, yàg-guièts-clâv-làs[23]; Huechachin[28]
Used by (7*): Zoque[01, 03]; Huastec[07]; Western Maya[19]; Zapotec[21, 23]; Nahua[28]
Used for (21#): Digestive (3)[03, 07, 28]; Eye (1)[03]; Musculoskeletal (2)[01, 07]; Neurological (3)[03, 07, 28]; Respiratory (3)[03, 19, 21]; Skin (1)[21]; Pregnancy (2)[03, 19]; Female genital (2)[19, 21]; General and Unspecified (4)[01, 03, 21, 23]
Cognates:
Language contact: Nah > Spanish

[01-28] refer to the study codes in Table 4.1. *Total number of studies citing this taxon #Total number of use-records

***Acacia hindsii* Benth. (Fabaceae)**

Spanish names: Cacho de buey, cachito de toro
Indigenous names: Ixcanal[19]
Used by (1*): Western Maya[19]
Used for (2#): Neurological (1)[19]; General and Unspecified (1)[19]
Cognates:
Language contact:

***Acacia macracantha* Willd. (Fabaceae)**

Spanish names: Cardo
Indigenous names:
Used by (1*): Zoque[01]
Used for (1#): General and Unspecified (1)[01]
Cognates:
Language contact:

***Acacia pennatula* (Schltdl. & Cham.) Benth. (Fabaceae)**

Spanish names:
Indigenous names: Yàg-guièts-yàaz,  yàg-guièts-dà, yàg-guièts-ngǒn[23]
Used by (1*): Zapotec[23]
Used for (1#): Digestive (1)[23]
Cognates:
Language contact:

***Acacia pringlei* Rose (Fabaceae)**

Spanish names:
Indigenous names: Uthuth te[07]
Used by (1*): Huastec[07]
Used for (2#): Neurological (1)[07]; General and Unspecified (1)[07]
Cognates:
Language contact:

***Acacia* sp. (Fabaceae)**

Spanish names:
Indigenous names: Ch'imay[09]
Used by (1*): Yucatecan Maya[09]
Used for (2#): Digestive (1)[09]; Skin (1)[09]
Cognates:
Language contact:

***Acaena elongata* L. (Rosaceae)**

Spanish names:
Indigenous names: Turumax[20]
Used by (1*): Western Maya[20]
Used for (1#): Digestive (1)[20]
Cognates:
Language contact:

***Acalypha alopecuroides* Jacq. (Euphorbiaceae)**

Spanish names: Cola de gato; Hierba del cancer
Indigenous names: Patsiit[04]; Xmisbil, Mehenmis[09]
Used by (3*): Mixe[04]; Yucatecan Maya[09, 11]
Used for (6#): Digestive (1)[09]; Neurological (1)[11]; Skin (2)[04, 11]; Endocrine (1)[09]; Urological (1)[11]
Cognates:
Language contact:

[01-28]refer to the study codes in Table 4.1.   *Total number of studies citing this taxon   #Total number of use-records

***Acalypha aristata*** **Kunth (Euphorbiaceae)**

Spanish names: Hierba de cancer
Indigenous names: Chi puk ay[03]; Čisšiši tuwan[05]; Tujuma'xanat[06]; Bohol ts'ohool, thuum ts'ohool, kakawate ts'ohool, xoko' ts'ohool, tsalam lat'em[07]; Kilik Q'ehen[14]; Contra ajtoy[18]
Used by (7*): Zoque[03]; Totonac[05, 06]; Huastec[07]; Quichean Maya[12, 14]; Western Maya[18]
Used for (21#): Blood (1)[12]; Digestive (4)[03, 06, 07, 12]; Eye (1)[03]; Skin (6)[03, 05, 06, 07, 12, 18]; Endocrine (1)[07]; Urological (2)[06, 12]; Pregnancy (1)[14]; Female genital (2)[12, 14]; Male genital (1)[12]; General and Unspecified (2)[07, 12]
Cognates: Toto: tu;
Language contact: Tot <> Hua

***Acalypha diversifolia*** **Jacq. (Euphorbiaceae)**

Spanish names: Corre chacalacca
Indigenous names: Ekchiñ poy cuy[03]
Used by (1*): Zoque[03]
Used for (2#): Skin (1)[03]; Endocrine (1)[03]
Cognates:
Language contact:

***Acalypha guatemalensis*** **Pax & K.Hoffm. (Euphorbiaceae)**

Spanish names: Hierba de Cancer Macho
Indigenous names: Ru xup Top, Sa'l Q'os[12]
Used by (1*): Quichean Maya[12]
Used for (3#): Musculoskeletal (1)[12]; Skin (1)[12]; General and Unspecified (1)[12]
Cognates:
Language contact:

***Acalypha leptopoda*** **Müll.Arg. (Euphorbiaceae)**

Spanish names: Corre chacalacca
Indigenous names: Ekchiñ poy cuy[03]; Ch'ilibtux il[09]
Used by (2*): Zoque[03]; Yucatecan Maya[09]
Used for (3#): Digestive (2)[03, 09]; Skin (1)[03]
Cognates:
Language contact:

***Acalypha monostachya*** **Cav. (Euphorbiaceae)**

Spanish names: Hierba del pastor
Indigenous names:
Used by (1*): Nahua[27]
Used for (3#): Skin (1)[27]; Pregnancy (1)[27]; General and Unspecified (1)[27]
Cognates:
Language contact:

***Acalypha mortoniana*** **Lundell (Euphorbiaceae)**

Spanish names:
Indigenous names: Xley Ipek[14]
Used by (1*): Quichean Maya[14]
Used for (2#): Respiratory (1)[14]; General and Unspecified (1)[14]
Cognates:
Language contact:

**_Acalypha phleoides_ Cav. (Euphorbiaceae)**

Spanish names:
Indigenous names: Tsak bohol ts'ohool, thuum ts'ohool tsakni'[07]
Used by (1*): Huastec[07]
Used for (1#): Digestive (1)[07]
Cognates:
Language contact:

**_Acalypha_ sp. (Euphorbiaceae)**

Spanish names: Hierba del cancer, hierba de mal de orín, hierba del gusano
Indigenous names: Tsuj mi ay[03]; Sak pasmar xiw[09]; Znajnoy[13]
Used by (3*): Zoque[03]; Yucatecan Maya[09]; Quichean Maya[13]
Used for (8#): Digestive (1)[13]; Musculoskeletal (1)[13]; Neurological (1)[03]; Skin (4)[03, 03, 09, 13]; Urological (1)[13]
Cognates:
Language contact:

**_Acalypha wilkesiana_ Müll.Arg. (Euphorbiaceae)**

Spanish names:
Indigenous names:
Used by (1*): Zoque[02]
Used for (1#): Skin (1)[02]
Cognates:
Language contact:

**_Acanthocereus tetragonus (_L.) Hummelinck (Cactaceae)**

Spanish names: Pitaya
Indigenous names: Xak'ub, tzatza[07]
Used by (2*): Huastec[07]; Quichean Maya[12]
Used for (2#): Psychological (1)[12]; Respiratory (1)[07]
Cognates:
Language contact:

**_Achillea millefolium_ L. (Asteraceae)**

Spanish names: Milenrama; Milenrama, achileo, milefolio, yerba de los carpinteros, ala de pollo; Plumajillo
Indigenous names: Milenrama[12]; Ushic ek, satul upa kaj, alhucema, sutul uxe kaj, sutil uwi kaj, uje kuk, tzilil up kaj, cumate q'aies, solb'al uwi kaj[13]
Used by (4*): Huastec[08]; Quichean Maya[12, 13]; Nahua[25]
Used for (21#): Digestive (3)[08, 12, 13]; Ear (2)[13, 25]; Cardiovascular (3)[08, 12, 13]; Musculoskeletal (2)[12, 13]; Psychological (1)[12]; Respiratory (1)[13]; Skin (2)[12, 13]; Endocrine (1)[13]; Urological (2)[12, 13]; Female genital (2)[12, 13]; General and Unspecified (2)[12, 13]
Cognates:
Language contact:

**_Achillea_ sp. (Asteraceae)**

Spanish names:
Indigenous names:
Used by (1*): Zoque[03]
Used for (1#): Musculoskeletal (1)[03]
Cognates:
Language contact:

***Achimenes erecta* (Lam.) H.P.Fuchs (Gesneriaceae)**

Spanish names:
Indigenous names: Baq'nel pim[14]
Used by (1*): Quichean Maya[14]
Used for (1#): General and Unspecified (1)[14]
Cognates:
Language contact:

***Achimenes grandiflora* (Schiede) DC. (Gesneriaceae)**

Spanish names:
Indigenous names:
Used by (1*): Zoque[03]
Used for (3#): Skin (1)[03]; Pregnancy (1)[03]; Female genital (1)[03]
Cognates:
Language contact:

***Achyranthes aspera* L. (Amaranthaceae)**

Spanish names:
Indigenous names: Ts'aah pathaam, lek'ab mitsu', tsakam pathaam akw'aal, itsik mitsu', itsaan an patham, akw'aalil pathaam, path ts'ohool[07]; Bayche'[09]
Used by (2*): Huastec[07]; Yucatecan Maya[09]
Used for (2#): Digestive (1)[09]; Skin (1)[07]
Cognates:
Language contact:

***Acmella oppositifolia* (Lam.) R.K.Jansen (Asteraceae)**

Spanish names: Orozus
Indigenous names:
Used by (1*): Yucatecan Maya[10]
Used for (1#): Respiratory (1)[10]
Cognates:
Language contact:

***Acmella radicans* (Jacq.) R. K. Jansen (Asteraceae)**

Spanish names:
Indigenous names: Ñiwi sotyi[03]
Used by (1*): Zoque[03]
Used for (1#): Neurological (1)[03]
Cognates:
Language contact:

***Acnistus arborescens* (L.) Schltdl. (Solanaceae)**

Spanish names:
Indigenous names: K'apaj, Ka'a pajl[14]
Used by (1*): Quichean Maya[14]
Used for (2#): Digestive (1)[14]; General and Unspecified (1)[14]
Cognates:
Language contact:

***Acosmium panamense* (Benth.) Yakovlev (Fabaceae)**

Spanish names: Guayacán
Indigenous names: Putsy takin[01]; Tzus kuy[02]; Sam cuy[03]; Nøøtxk[04]; K'a che'[17]; Guassi[21]
Used by (8*): Zoque[01, 02, 03]; Mixe[04]; Yucatecan Maya[10]; Quichean Maya[17]; Zapotec[21]; Nahua[26]
Used for (28#): Blood (2)[02, 03]; Digestive (5)[01, 02, 03, 04, 10]; Musculoskeletal (2)[03, 21]; Respiratory (3)[01, 03, 04]; Skin (1)[03]; Endocrine (3)[01, 02, 03]; Urological (1)[03]; Pregnancy (2)[02, 03]; Female genital (3)[03, 04, 21]; General and Unspecified (5)[02, 03, 04, 21, 26]; nd[17]
Cognates:
Language contact:

***Acourtia nudicaulis* (A.Gray) B.L.Turner (Asteraceae)**

Spanish names: Valeriana; Valeriana roja/de cerro
Indigenous names:
Used by (2*): Quichean Maya[12]; Western Maya[19]
Used for (7#): Musculoskeletal (1)[19]; Neurological (1)[19]; Psychological (2)[12, 19]; Pregnancy (1)[19]; Female genital (1)[19]; General and Unspecified (1)[19]
Cognates:
Language contact:

***Acourtia* sp. (Asteraceae)**

Spanish names:
Indigenous names: Guìzh-zhĭil-dán[23]
Used by (1*): Zapotec[23]
Used for (1#): Skin (1)[23]
Cognates:
Language contact:

***Acrocomia aculeata* (Jacq.) Lodd. ex Mart. (Arecaceae)**

Spanish names: Coyol
Indigenous names: Kuma[02]; Cuma[03]; Kum[04]; Maap[07]; Biga'aj[21]
Used by (6*): Zoque[01, 02, 03]; Mixe[04]; Huastec[07]; Zapotec[21]
Used for (9#): Blood (1)[03]; Digestive (4)[02, 03, 04, 21]; Respiratory (1)[07]; Endocrine (1)[01]; Pregnancy (1)[07]; General and Unspecified (1)[21]
Cognates: MZ: kum; Zoq: kuma;
Language contact:

***Adelobotrys adscendens* (Sw.) Triana (Melastomataceae)**

Spanish names:
Indigenous names: Chunac kejen[16]; Chunahak k'ejen[17]
Used by (2*): Quichean Maya[16, 17]
Used for (2#): Neurological (1)[16]; nd[17]
Cognates:
Language contact:

***Adenophyllum appendiculatum* (Lag.) Strother (Asteraceae)**

Spanish names: Flor de calandria
Indigenous names: Guibiguadajnii, zamposuche guesxi[21]
Used by (1*): Zapotec[21]
Used for (6#): Digestive (1)[21]; Eye (1)[21]; Psychological (1)[21]; Respiratory (1)[21]; Skin (1)[21]; General and Unspecified (1)[21]
Cognates:
Language contact:

***Adenophyllum aurantium*** **(L.) Strother (Asteraceae)**

Spanish names: Cempasúchil del monte/ ruda del monte
Indigenous names:
Used by (1*): Zoque[02]
Used for (3#): Digestive (1)[02]; Ear (1)[02]; Musculoskeletal (1)[02]
Cognates:
Language contact:

***Adiantopsis radiata*** **(L.) Fée (Pteridaceae)**

Spanish names:
Indigenous names: Kaxam[07]
Used by (1*): Huastec[07]
Used for (1#): General and Unspecified (1)[07]
Cognates:
Language contact:

***Adiantum andicola*** **Liebm. (Pteridaceae)**

Spanish names: Cilantrillo; Culantrillo; Culantrillo de pozo
Indigenous names: Culanto ch'o, coq' xip, uwi ja, uwi siwan, uklanto siwan[13]
Used by (3*): Zoque[01]; Quichean Maya[12, 13]
Used for (7#): Digestive (1)[12]; Neurological (1)[01]; Respiratory (1)[13]; Skin (1)[12]; Urological (1)[01]; Pregnancy (1)[13]; General and Unspecified (1)[13]
Cognates:
Language contact:

***Adiantum latifolium*** **Lam. (Pteridaceae)**

Spanish names:
Indigenous names:
Used by (1*): Quichean Maya[16]
Used for (3#): Neurological (1)[16]; Psychological (1)[16]; General and Unspecified (1)[16]
Cognates:
Language contact:

***Adiantum petiolatum*** **Desv. (Pteridaceae)**

Spanish names:
Indigenous names:
Used by (1*): Quichean Maya[17]
Used for (1#): nd[17]
Cognates:
Language contact:

***Adiantum poiretii*** **Wikstr. (Pteridaceae)**

Spanish names:
Indigenous names: Akan pich, akan ts'ok, akan hooh, chalam ha'[07]
Used by (1*): Huastec[07]
Used for (4#): Cardiovascular (1)[07]; Neurological (1)[07]; Pregnancy (1)[07]; General and Unspecified (1)[07]
Cognates:
Language contact:

***Adiantum princeps*** **T. Moore (Pteridaceae)**

Spanish names:
Indigenous names: Colantra pim[16]
Used by (2*): Zoque[03]; Quichean Maya[16]
Used for (4#): Digestive (1)[03]; Neurological (1)[16]; Female genital (1)[03]; General and Unspecified (1)[16]
Cognates:
Language contact:

[01-28]refer to the study codes in Table 4.1. *Total number of studies citing this taxon #Total number of use-records

**_Adiantum pulverulentum_ L. (Pteridaceae)**

Spanish names:
Indigenous names: Sis'bi pim[17]
Used by (1*): Quichean Maya[17]
Used for (1#): nd[17]
Cognates:
Language contact:

**_Adiantum_ sp. (Pteridaceae)**

Spanish names: Culantrillo; Pata de Clarinero
Indigenous names: Roq' Chi'wan[14]
Used by (2*): Quichean Maya[12, 14]
Used for (8#): Digestive (2)[12, 14]; Neurological (1)[14]; Psychological (1)[12]; Respiratory (1)[12]; Urological (1)[12]; Female genital (1)[12]; General and Unspecified (1)[12]
Cognates:
Language contact:

**_Adiantum tenerum_ Sw. (Pteridaceae)**

Spanish names: Colandrillo
Indigenous names: Tek piixi tek[04]; Akan pich, lapis ts'ohool[07]; Ecahuile[24]
Used by (4*): Mixe[04]; Huastec[07]; Zapotec[21]; Nahua[24]
Used for (4#): Urological (1)[21]; Pregnancy (1)[24]; Female genital (1)[04]; General and Unspecified (1)[07]
Cognates:
Language contact: Mixe <> Hua <> Nah

**_Adiantum tetraphyllum_ Humb. & Bonpl. ex Willd. (Pteridaceae)**

Spanish names:
Indigenous names: Roq chit cuan[16]
Used by (1*): Quichean Maya[16]
Used for (2#): Neurological (1)[16]; General and Unspecified (1)[16]
Cognates:
Language contact:

**_Adiantum tricholepis_ Fée (Pteridaceae)**

Spanish names:
Indigenous names: Akan pich[07]
Used by (1*): Huastec[07]
Used for (1#): General and Unspecified (1)[07]
Cognates:
Language contact:

**_Adiantum wilsonii_ Hook. (Pteridaceae)**

Spanish names:
Indigenous names: Ruj i rak'aj tza[16]; Ru'j i rak' aj tza[17]
Used by (2*): Quichean Maya[16, 17]
Used for (3#): Neurological (1)[16]; Psychological (1)[16]; nd[17]
Cognates:
Language contact:

***Aechmea bracteata* (Sw.) Griseb. (Bromeliaceae)**

Spanish names: Kardon
Indigenous names: K'ok'om, k'ok'om wits, ok'lom te'[07]; Ch'uk, Cinta k'uk'[09]
Used by (3*): Zoque[03]; Huastec[07]; Yucatecan Maya[09]
Used for (8#): Digestive (2)[03, 07]; Eye (1)[07]; Ear (1)[03]; Neurological (1)[07]; Urological (1)[03]; Pregnancy (1)[09]; General and Unspecified (1)[07]
Cognates: Mayan: k'ok/k'uk;
Language contact:

***Aechmea* sp. (Bromeliaceae)**

Spanish names: Ixtle
Indigenous names: Ñiauing[03]
Used by (1*): Zoque[03]
Used for (3#): Urological (1)[03]; Pregnancy (1)[03]; Female genital (1)[03]
Cognates:
Language contact:

***Aegiphila deppeana* Steud. (Lamiaceae)**

Spanish names:
Indigenous names: M¥a ay[03]
Used by (1*): Zoque[03]
Used for (2#): Musculoskeletal (1)[03]; Female genital (1)[03]
Cognates:
Language contact:

***Aegiphila monstrosa* Moldenke (Lamiaceae)**

Spanish names:
Indigenous names: Rok' xaa'n[17]
Used by (1*): Quichean Maya[17]
Used for (1#): nd[17]
Cognates:
Language contact:

***Aegiphila* sp. (Lamiaceae)**

Spanish names:
Indigenous names:
Used by (1*): Quichean Maya[16]
Used for (1#): Neurological (1)[16]
Cognates:
Language contact:

***Aeschynomene americana* L. (Fabaceae)**

Spanish names: Dormilona
Indigenous names:
Used by (1*): Zoque[01]
Used for (1#): General and Unspecified (1)[01]
Cognates:
Language contact:

***Aeschynomene fascicularis* Cham. & Schltdl. (Fabaceae)**

Spanish names:
Indigenous names: Salat-ik'[09]
Used by (1*): Yucatecan Maya[09]
Used for (1#): Skin (1)[09]
Cognates:
Language contact:

***Agarista mexicana*** **(Hemsl.) Judd (Ericaceae)**

Spanish names:
Indigenous names: Unuk tyiñcuy[03]
Used by (1*): Zoque[03]
Used for (3#): Respiratory (1)[03]; Skin (1)[03]; Female genital (1)[03]
Cognates:
Language contact:

***Agastache mexicana*** **(Kunth) Lint & Epling (Lamiaceae)**

Spanish names: Toronjil
Indigenous names:
Used by (3*): Totonac[06]; Nahua[25, 26]
Used for (6#): Digestive (1)[06]; Neurological (1)[25]; Psychological (1)[06]; Respiratory (1)[26]; Female genital (2)[06, 26]
Cognates:
Language contact:

***Agave americana*** **L. (Asparagaceae)**

Spanish names: Maguey
Indigenous names:
Used by (1*): Yucatecan Maya[09]
Used for (1#): Pregnancy (1)[09]
Cognates:
Language contact:

***Agave atrovirens*** **Karw. ex Salm-Dyck (Asparagaceae)**

Spanish names: Maguey
Indigenous names:
Used by (1*): Nahua[25]
Used for (6#): Cardiovascular (1)[25]; Musculoskeletal (1)[25]; Neurological (1)[25]; Skin (1)[25]; Pregnancy (1)[25]; General and Unspecified (1)[25]
Cognates:
Language contact:

***Agave kewensis*** **Jacobi (Asparagaceae)**

Spanish names: Maguey
Indigenous names: O[01]
Used by (1*): Zoque[01]
Used for (1#): Skin (1)[01]
Cognates:
Language contact:

***Agave marmorata*** **Roezl (Asparagaceae)**

Spanish names:
Indigenous names: Dòb-pcuêl, dòb-zân[23]
Used by (1*): Zapotec[23]
Used for (3#): Musculoskeletal (1)[23]; Skin (1)[23]; Pregnancy (1)[23]
Cognates:
Language contact:

[01-28]refer to the study codes in Table 4.1. *Total number of studies citing this taxon #Total number of use-records

***Agave* sp. (Asparagaceae)**

Spanish names: Maguey; Maguey del cerro
Indigenous names: Xa'[04]; Weey, tsi'iim, thak book[07]; Sukchij[19]; Dobala'a[21]; Dohb[22]
Used by (5*): Mixe[04]; Huastec[07]; Western Maya[19]; Zapotec[21, 22]
Used for (13#): Respiratory (2)[07, 21]; Skin (3)[07, 19, 21]; Pregnancy (4)[04, 07, 19, 22]; Female genital (1)[19]; General and Unspecified (3)[07, 21, 22]
Cognates: Zapo: dob;
Language contact: Hua <> Zap

***Agave stricta* Salm-Dyck (Asparagaceae)**

Spanish names: Magueicillo
Indigenous names:
Used by (1*): Nahua[27]
Used for (1#): Skin (1)[27]
Cognates:
Language contact:

***Agave tecta* Trel. (Asparagaceae)**

Spanish names: Maguey
Indigenous names: Sajkiy[12]
Used by (1*): Quichean Maya[12]
Used for (5#): Digestive (1)[12]; Psychological (1)[12]; Skin (1)[12]; Urological (1)[12]; General and Unspecified (1)[12]
Cognates:
Language contact:

***Agave vivipara* L. (Asparagaceae)**

Spanish names: Maguey
Indigenous names:
Used by (1*): Zoque[03]
Used for (1#): Skin (1)[03]
Cognates:
Language contact:

***Agdestis clematidea* Moc. & Sessé ex DC. (Phytolaccaceae)**

Spanish names: Suelda consuelda
Indigenous names: Yokya poj/ päksy[02]; Tuuwi', ix tuuwi', thak huchuk, thuyum[07]
Used by (2*): Zoque[02]; Huastec[07]
Used for (4#): Musculoskeletal (2)[02, 07]; Skin (1)[07]; General and Unspecified (1)[07]
Cognates:
Language contact:

***Ageratina espinosarum* (A.Gray) R.M.King & H.Rob. (Asteraceae)**

Spanish names: Yerba de culebra/ aigre
Indigenous names: Škwam bäl[22]
Used by (1*): Zapotec[22]
Used for (5#): Eye (1)[22]; Musculoskeletal (1)[22]; Neurological (1)[22]; Skin (1)[22]; General and Unspecified (1)[22]
Cognates:
Language contact:

***Ageratina ligustrina* (DC.) R.M.King & H.Rob. (Asteraceae)**

Spanish names:
Indigenous names: Sakil ch'a te', xuch'il vomol[20]
Used by (1*): Western Maya[20]
Used for (5#): Digestive (1)[20]; Respiratory (1)[20]; Skin (1)[20]; Pregnancy (1)[20]; General and Unspecified (1)[20]
Cognates:
Language contact:

***Ageratina mairetiana*** **(DC.) R.M.King & H.Rob. (Asteraceae)**

Spanish names:
Indigenous names: Yàg-bdìin, guìzh-bdìin, guìzh-zhwèe[23]
Used by (1*): Zapotec[23]
Used for (1#): General and Unspecified (1)[23]
Cognates:
Language contact:

***Ageratina petiolaris*** **(Moc. & Sessé ex DC.) R.M.King & H.Rob. (Asteraceae)**

Spanish names: Hierba de angerl
Indigenous names: K'anim mim[08]; Guìzh-rquiá-yàn[23]
Used by (2*): Huastec[08]; Zapotec[23]
Used for (3#): Neurological (1)[08]; Respiratory (1)[23]; General and Unspecified (1)[23]
Cognates:
Language contact:

***Ageratina pringlei*** **(B.L.Rob. & Greenm.) R.M.King & H.Rob. (Asteraceae)**

Spanish names:
Indigenous names: Pom ch'a te', ox yoket vomol[20]
Used by (1*): Western Maya[20]
Used for (4#): Digestive (1)[20]; Ear (1)[20]; Respiratory (1)[20]; General and Unspecified (1)[20]
Cognates:
Language contact:

***Ageratum conyzoides*** **(L.) L. (Asteraceae)**

Spanish names: Mejorana
Indigenous names: Taam ujts[04]; Xab'on Q'ehen[14]
Used by (4*): Mixe[04]; Quichean Maya[12, 13, 14]
Used for (9#): Digestive (2)[04, 12]; Neurological (1)[12]; Respiratory (3)[12, 13, 14]; Skin (2)[04, 13]; General and Unspecified (1)[12]
Cognates:
Language contact:

***Ageratum corymbosum*** **Zuccagni ex Pers. (Asteraceae)**

Spanish names: Hierba santa cimarrón; Mejorana, pericón morado
Indigenous names: Ya'atzan Pamaj Q'os[12]; Lok' ab' ixq[14]; Tlanecpaquelite[26]
Used by (3*): Quichean Maya[12, 14]; Nahua[26]
Used for (7#): Digestive (1)[12]; Neurological (1)[12]; Respiratory (1)[14]; Skin (2)[12, 26]; Endocrine (1)[12]; General and Unspecified (1)[12]
Cognates:
Language contact:

***Ageratum gaumeri*** **B.L.Rob. (Asteraceae)**

Spanish names:
Indigenous names: Xpasmar xiw[09]
Used by (1*): Yucatecan Maya[09]
Used for (1#): Digestive (1)[09]
Cognates:
Language contact:

***Ageratum houstonianum*** **Mill. (Asteraceae)**

Spanish names: Mostranzo
Indigenous names: Lok' ab' winq'[14]
Used by (2*): Quichean Maya[14]; Nahua[25]
Used for (5#): Digestive (1)[14]; Respiratory (1)[14]; Skin (1)[25]; Female genital (1)[14]; General and Unspecified (1)[14]
Cognates:
Language contact:

**_Agrimonia_ sp. (Rosaceae)**

Spanish names:
Indigenous names:
Used by (1*): Quichean Maya[13]
Used for (2#): Digestive (1)[13]; Skin (1)[13]
Cognates:
Language contact:

**_Albizia tomentosa_ (Micheli) Standl. (Fabaceae)**

Spanish names:
Indigenous names: Thukiim[07]
Used by (1*): Huastec[07]
Used for (1#): General and Unspecified (1)[07]
Cognates:
Language contact:

**_Alcea rosea_ L. (Malvaceae)**

Spanish names:
Indigenous names: Guièe-màrpôl-nquĭts[23]
Used by (1*): Zapotec[23]
Used for (1#): Respiratory (1)[23]
Cognates:
Language contact:

**_Alchemilla_ sp. (Rosaceae)**

Spanish names:
Indigenous names:
Used by (1*): Quichean Maya[13]
Used for (1#): Digestive (1)[13]
Cognates:
Language contact:

**_Alchornea latifolia_ Sw. (Euphorbiaceae)**

Spanish names: Marangola, cacahuatillo
Indigenous names: Tuwax'katat, liilhpatat[06]
Used by (1*): Totonac[06]
Used for (2#): Endocrine (1)[06]; General and Unspecified (1)[06]
Cognates:
Language contact:

**_Aldama dentata_ La Llave ex La Llave (Asteraceae)**

Spanish names: Escutumbul
Indigenous names: K'an wits, mataablom[07]; Escutumbul[18]
Used by (2*): Huastec[07]; Western Maya[18]
Used for (3#): Respiratory (1)[07]; Skin (1)[07]; Endocrine (1)[18]
Cognates:
Language contact: Hua <> Chontal

**_Alibertia edulis_ (Rich.) A.Rich. ex DC. (Rubiaceae)**

Spanish names: Catarita; Marimbola
Indigenous names: Wikpak[03]; Shbola bishulu'[21]
Used by (2*): Zoque[03]; Zapotec[21]
Used for (4#): Psychological (1)[03]; Respiratory (1)[03]; General and Unspecified (2)[03, 21]
Cognates:
Language contact:

[01-28] refer to the study codes in Table 4.1. *Total number of studies citing this taxon #Total number of use-records

***Allium cepa* L. (Amaryllidaceae)**

Spanish names: Cebolla
Indigenous names: Tzapaas ceullas/tsepolas[01]; Akat'sasna[06]; Thobollash dhoboyax[08]; Ceboy'[13]; Ceboy, cebul[21]; Žiht[22]; Nlěch, nsùch[23]
Used by (12*): Zoque[01, 02, 03]; Totonac[06]; Huastec[08]; Quichean Maya[12, 13, 14]; Western Maya[19]; Zapotec[21, 22, 23]
Used for (44#): Digestive (5)[01, 03, 08, 12, 21]; Eye (1)[02]; Ear (2)[06, 13]; Cardiovascular (1)[01]; Musculoskeletal (3)[08, 14, 21]; Neurological (4)[01, 08, 13, 21]; Psychological (2)[01, 03]; Respiratory (8)[01, 02, 03, 06, 08, 14, 19, 21]; Skin (7)[01, 03, 08, 13, 14, 19, 21]; Endocrine (2)[03, 08]; Pregnancy (2)[13, 22]; Male genital (2)[02, 03]; General and Unspecified (5)[01, 02, 12, 14, 23]
Cognates:
Language contact:

***Allium glandulosum* Link & Otto (Amaryllidaceae)**

Spanish names: Cebollín
Indigenous names: Antuxk[04]
Used by (2*): Zoque[01]; Mixe[04]
Used for (2#): General and Unspecified (2)[01, 04]
Cognates:
Language contact:

***Allium longifolium (*Kunth) Spreng. (Amaryllidaceae)**

Spanish names:
Indigenous names: Huun nakat, chuum nakat, xuun nakat[07]
Used by (1*): Huastec[07]
Used for (3#): Digestive (1)[07]; Skin (1)[07]; General and Unspecified (1)[07]
Cognates:
Language contact:

***Allium sativum* L. (Amaryllidaceae)**

Spanish names: Ajo
Indigenous names: Asyus[01]; Asyus[02]; A'xux[06]; Aaxux[07]; Axu'x[13]; Axux[20]; Aaxoj[21]
Used by (14*): Zoque[01, 02, 03]; Mixe[04]; Totonac[06]; Huastec[07]; Yucatecan Maya[09]; Quichean Maya[12, 13, 14]; Western Maya[19, 20]; Zapotec[21]; Nahua[25]
Used for (63#): Digestive (11)[01, 02, 03, 06, 07, 12, 13, 19, 19, 20, 21]; Ear (1)[04]; Cardiovascular (3)[12, 13, 19]; Musculoskeletal (9)[01, 02, 03, 06, 07, 13, 14, 21, 25]; Neurological (4)[01, 02, 09, 21]; Psychological (2)[06, 19]; Respiratory (7)[02, 03, 06, 12, 14, 19, 21]; Skin (8)[01, 02, 03, 04, 13, 14, 21, 25]; Endocrine (1)[03]; Urological (2)[02, 03]; Pregnancy (3)[03, 09, 13]; Female genital (2)[02, 09]; General and Unspecified (10)[01, 02, 06, 07, 12, 13, 14, 19, 20, 21]
Cognates:
Language contact:

***Allium schoenoprasum* L. (Amaryllidaceae)**

Spanish names: Cebollin
Indigenous names:
Used by (1*): Yucatecan Maya[09]
Used for (1#): Neurological (1)[09]
Cognates:
Language contact:

**Allium sp. (Amaryllidaceae)**

Spanish names: Cebollín
Indigenous names:
Used by (1*): Zapotec[21]
Used for (1#): Eye (1)[21]
Cognates:
Language contact:

[01-28]refer to the study codes in Table 4.1. *Total number of studies citing this taxon #Total number of use-records

***Alloispermum integrifolium* (DC.) H.Rob. (Asteraceae)**

Spanish names: Flor de estrella
Indigenous names: Ma'atza jäyä[01]
Used by (1*): Zoque[01]
Used for (1#): General and Unspecified (1)[01]
Cognates:
Language contact:

***Alloispermum* sp. (Asteraceae)**

Spanish names:
Indigenous names: K'oxox chij[20]
Used by (1*): Western Maya[20]
Used for (1#): Digestive (1)[20]
Cognates:
Language contact:

***Allophylus cominia* (L.) Sw. (Sapindaceae)**

Spanish names:
Indigenous names:
Used by (1*): Yucatecan Maya[09]
Used for (1#): Skin (1)[09]
Cognates:
Language contact:

***Allophylus* sp. (Sapindaceae)**

Spanish names:
Indigenous names: Katxu aay[03]
Used by (1*): Zoque[03]
Used for (1#): Skin (1)[03]
Cognates:
Language contact:

***Allosidastrum pyramidatum* (Desp. ex Cav.) Krapov., Fryxell & Bates (Malvaceae)**

Spanish names:
Indigenous names: T'unu' ix bek'em[07]
Used by (1*): Huastec[07]
Used for (1#): Pregnancy (1)[07]
Cognates:
Language contact:

***Alnus acuminata* Kunth (Betulaceae)**

Spanish names: Aliso; Avedul
Indigenous names: Lama'[12]; Lemob'[13]; Elite, ilite[26]
Used by (3*): Quichean Maya[12, 13]; Nahua[26]
Used for (9#): Digestive (1)[12]; Cardiovascular (1)[12]; Musculoskeletal (2)[12, 13]; Urological (1)[12]; Pregnancy (1)[13]; Female genital (1)[12]; General and Unspecified (2)[12, 26]
Cognates: Quich: lVmV;
Language contact:

***Alnus jorullensis* Kunth (Betulaceae)**

Spanish names: Avedul rojo
Indigenous names: Lama'[12]
Used by (1*): Quichean Maya[12]
Used for (1#): Cardiovascular (1)[12]
Cognates:
Language contact:

[01-28]refer to the study codes in Table 4.1. *Total number of studies citing this taxon #Total number of use-records

**_Alnus_ sp. (Betulaceae)**

Spanish names: Avedul
Indigenous names:
Used by (1*): Quichean Maya[12]
Used for (8#): Cardiovascular (1)[12]; Musculoskeletal (1)[12]; Skin (2)[12, 12]; Urological (1)[12]; Female genital (1)[12]; Male genital (1)[12]; General and Unspecified (1)[12]
Cognates:
Language contact:

**_Aloe_ sp. (Xanthorrhoeaceae)**

Spanish names: Sabila
Indigenous names: Xabila[07]
Used by (2*): Zoque[03]; Huastec[07]
Used for (8#): Digestive (1)[03]; Musculoskeletal (1)[03]; Respiratory (1)[03]; Skin (2)[03, 07]; Urological (1)[03]; Female genital (1)[03]; General and Unspecified (1)[03]
Cognates:
Language contact:

**_Aloe vera_ (L.) Burm.f. (Xanthorrhoeaceae)**

Spanish names: Sábila
Indigenous names: Petk'inki[09]; Pimki[13]; Tujtuj sukchij[19]; Dòb-xtîl[23]
Used by (15*): Zoque[01, 02]; Mixe[04]; Huastec[08]; Yucatecan Maya[09, 11]; Quichean Maya[12, 13, 14]; Western Maya[18, 19]; Zapotec[21, 23]; Nahua[25, 27]
Used for (81#): Blood (4)[01, 02, 12, 18]; Digestive (10)[01, 02, 08, 12, 13, 18, 19, 21, 23, 25]; Eye (2)[02, 27]; Cardiovascular (3)[01, 02, 21]; Musculoskeletal (9)[01, 02, 08, 12, 13, 18, 19, 21, 27]; Neurological (4)[08, 18, 19, 25]; Psychological (2)[11, 12]; Respiratory (10)[01, 02, 04, 09, 13, 18, 21, 23, 25, 27]; Skin (15)[01, 02, 04, 08, 09, 11, 12, 13, 14, 18, 19, 21, 23, 25, 27]; Endocrine (5)[08, 13, 18, 25, 27]; Urological (5)[01, 12, 13, 18, 23]; Female genital (2)[01, 12]; Male genital (1)[12]; General and Unspecified (9)[01, 08, 12, 13, 18, 19, 21, 23, 25]
Cognates:
Language contact:

**_Alophia drummondii_ (Graham) R.C.Foster (Iridaceae)**

Spanish names:
Indigenous names: Tsakam apats, tsak apats[07]
Used by (1*): Huastec[07]
Used for (1#): Digestive (1)[07]
Cognates:
Language contact:

**_Aloysia citriodora_ Palau (Verbenaceae)**

Spanish names: Cedron; Cedrón, Maria Luisa; Hierba luisa
Indigenous names: Bänälä luisa[18]
Used by (3*): Quichean Maya[12]; Western Maya[18]; Zapotec[22]
Used for (12#): Blood (1)[12]; Digestive (2)[12, 22]; Cardiovascular (1)[12]; Neurological (1)[12]; Psychological (2)[12, 22]; Respiratory (1)[12]; Skin (1)[12]; General and Unspecified (3)[12, 18, 22]
Cognates:
Language contact:

**_Alternanthera caracasana_ Kunth (Amaranthaceae)**

Spanish names: Golondrina
Indigenous names: Guìzh-guìdzdán, guìzh-yù, guìzh-guiět-nì , guìzh-zhàn-nì[23]
Used by (2*): Zapotec[23]; Nahua[26]
Used for (3#): Digestive (1)[23]; Skin (1)[26]; General and Unspecified (1)[23]
Cognates:
Language contact:

[01-28]refer to the study codes in Table 4.1. *Total number of studies citing this taxon #Total number of use-records

***Alternanthera laguroides* (Standl.) Standl. (Amaranthaceae)**

Spanish names:
Indigenous names: Tan wamal[20]
Used by (1*): Western Maya[20]
Used for (1#): Digestive (1)[20]
Cognates:
Language contact:

***Alternanthera philoxeroides* (Mart.) Griseb. (Amaranthaceae)**

Spanish names:
Indigenous names: Wiichab ts'ohool, wiichab maal, pulik wichab[07]
Used by (1*): Huastec[07]
Used for (3#): Digestive (1)[07]; Skin (1)[07]; General and Unspecified (1)[07]
Cognates:
Language contact:

***Alternanthera pungens* Kunth (Amaranthaceae)**

Spanish names:
Indigenous names: Tiangispepetl[26]
Used by (1*): Nahua[26]
Used for (4#): Digestive (1)[26]; Neurological (1)[26]; Respiratory (1)[26]; General and Unspecified (1)[26]
Cognates:
Language contact:

***Alternanthera sessilis (*L.) R.Br. ex DC. (Amaranthaceae)**

Spanish names:
Indigenous names: Tiyankix pepetal, ehtiil tsakam wichab[07]
Used by (1*): Huastec[07]
Used for (2#): Digestive (1)[07]; General and Unspecified (1)[07]
Cognates:
Language contact:

***Alvaradoa amorphoides* Liebm. (Picramniaceae)**

Spanish names: Palo de hormigas; Plumajillo
Indigenous names: Belsinikche'[09]; Tzutzula[11]; Chi'karar te'[19]
Used by (3*): Yucatecan Maya[09, 11]; Western Maya[19]
Used for (4#): Skin (3)[09, 11, 19]; General and Unspecified (1)[11]
Cognates:
Language contact:

***Amaranthus caudatus* L. (Amaranthaceae)**

Spanish names:
Indigenous names: Chith, lek'ab tooro[07]
Used by (1*): Huastec[07]
Used for (2#): Digestive (1)[07]; General and Unspecified (1)[07]
Cognates:
Language contact:

***Amaranthus hybridus* L. (Amaranthaceae)**

Spanish names: Bledo, Amaranto; Pie de paloma roja; Quintonil
Indigenous names: Chithal tooro, he'pan, chital ist'amaal, waho ts'ohool, alte' chith[07]; Nagucho'o[21]; Guìzbæ̀[23]
Used by (5*): Zoque[03]; Huastec[07]; Quichean Maya[12]; Zapotec[21, 23]
Used for (5#): Digestive (1)[23]; Cardiovascular (1)[12]; Skin (1)[07]; Female genital (1)[03]; General and Unspecified (1)[21]
Cognates:
Language contact:

***Amaranthus* sp. (Amaranthaceae)**

Spanish names: Bledo
Indigenous names: Tez[13]
Used by (1*): Quichean Maya[13]
Used for (1#): Blood (1)[13]
Cognates:
Language contact:

***Amaranthus spinosus* L. (Amaranthaceae)**

Spanish names: Espina; Espina de borrego; Quintonil espinoso
Indigenous names: Tu'kun calx'tunit[06]; Guedxe'e[21]
Used by (3*): Zoque[02]; Totonac[06]; Zapotec[21]
Used for (4#): Musculoskeletal (1)[02]; Urological (1)[06]; General and Unspecified (2)[06, 21]
Cognates:
Language contact:

***Amaranthus viridis* L. (Amaranthaceae)**

Spanish names: Bledo
Indigenous names:
Used by (1*): Zoque[01]
Used for (2#): Digestive (1)[01]; General and Unspecified (1)[01]
Cognates:
Language contact:

***Ambrosia artemisiifolia* L. (Asteraceae)**

Spanish names: Artemis; Hierba del perro, altamisa, hierba de las señoras
Indigenous names:
Used by (2*): Mixe[04]; Nahua[26]
Used for (2#): Musculoskeletal (1)[04]; Female genital (1)[26]
Cognates:
Language contact:

***Ambrosia peruviana* Willd. (Asteraceae)**

Spanish names: Altamís
Indigenous names:
Used by (2*): Western Maya[19, 20]
Used for (4#): Digestive (2)[19, 20]; Musculoskeletal (1)[19]; General and Unspecified (1)[19]
Cognates:
Language contact:

***Ampelocissus erdvendbergiana* Planch. (Vitaceae)**

Spanish names:
Indigenous names: Kamuuxy[04]
Used by (1*): Mixe[04]
Used for (1#): Eye (1)[04]
Cognates:
Language contact:

***Amphilophium crucigerum* (L.) L.G.Lohmann (Bignoniaceae)**

Spanish names:
Indigenous names: Lalab uthu', ch'ichaabil uthu, t'uu ch'aah[07]; Xache'ma'ax, Xache'xnuuk[09]
Used by (2*): Huastec[07]; Yucatecan Maya[09]
Used for (2#): Skin (2)[07, 09]
Cognates:
Language contact:

[01-28]refer to the study codes in Table 4.1. *Total number of studies citing this taxon #Total number of use-records

***Amphilophium paniculatum* (L.) Kunth (Bignoniaceae)**

Spanish names:
Indigenous names: Sit'macho, Petak'[09]; Soski-ak', Xdunt'-ak'[09]
Used by (1*): Yucatecan Maya[09]
Used for (3#): Digestive (2)[09, 09]; Skin (1)[09]
Cognates:
Language contact:

***Amphipterygium adstringens* (Schltdl.) Standl. (Anacardiaceae)**

Spanish names: Cuachalalate
Indigenous names: Kuy tupsy[02]; Ya'guiaj[21]
Used by (5*): Zoque[02, 03]; Zapotec[21, 22]; Nahua[27]
Used for (20#): Blood (1)[21]; Digestive (4)[02, 21, 22, 27]; Cardiovascular (1)[02]; Musculoskeletal (1)[21]; Skin (3)[02, 22, 27]; Endocrine (1)[27]; Urological (1)[27]; Pregnancy (2)[02, 03]; Female genital (3)[02, 03, 21]; General and Unspecified (3)[02, 21, 27]
Cognates:
Language contact:

***Amphitecna tuxtlensis* A.H.Gentry (Bignoniaceae)**

Spanish names: Jicara del duende
Indigenous names: Makti jeepe[03]; Cuacotecamatl, tecomate[26]
Used by (2*): Zoque[03]; Nahua[26]
Used for (3#): Psychological (1)[03]; Respiratory (1)[26]; Endocrine (1)[26]
Cognates:
Language contact: Highland Popoluca <-> Nah

***Anacardium occidentale* L. (Anacardiaceae)**

Spanish names: Jocote de marañon, anacardo, alcyoiba; Marañon
Indigenous names: Q'inóm[13]
Used by (3*): Mixe[04]; Quichean Maya[13]; Western Maya[19]
Used for (3#): Digestive (2)[13, 19]; Skin (1)[04]
Cognates:
Language contact:

***Anagallis arvensis* L. (Primulaceae)**

Spanish names:
Indigenous names: Yax chel[20]; Ncuàan-dzéb-còrâl[23]; Nequixanitl[26]
Used by (3*): Western Maya[20]; Zapotec[23]; Nahua[26]
Used for (4#): Digestive (1)[20]; Musculoskeletal (1)[26]; Skin (1)[26]; General and Unspecified (1)[23]
Cognates:
Language contact:

***Ananas comosus* (L.) Merr. (Bromeliaceae)**

Spanish names: Piña
Indigenous names: Tzik witz[01]; Uj'uj/tzikuy[02]; Uuju[03]; Aa'caa'xca[06]; Chabcham wits[07]
Used by (8*): Zoque[01, 02, 03]; Totonac[06]; Huastec[07]; Quichean Maya[12]; Western Maya[18]; Zapotec[21]
Used for (17#): Digestive (5)[02, 06, 07, 12, 21]; Musculoskeletal (1)[01]; Neurological (1)[18]; Endocrine (2)[06, 18]; Urological (4)[01, 02, 06, 12]; Female genital (2)[01, 02]; General and Unspecified (2)[03, 21]
Cognates:
Language contact:

[01-28] refer to the study codes in Table 4.1. *Total number of studies citing this taxon #Total number of use-records

***Andira galeottiana* Standl. (Fabaceae)**

Spanish names:
Indigenous names: Maka[03]; Iixy[04]
Used by (2*): Zoque[03]; Mixe[04]
Used for (2#): Skin (1)[03]; General and Unspecified (1)[04]
Cognates:
Language contact:

***Andira inermis* (Wright) DC. (Fabaceae)**

Spanish names: Lombricero
Indigenous names: Ma'aka kuy[02]; Tsukiñi cuy[03]; Ngaash[21]
Used by (3*): Zoque[02, 03]; Zapotec[21]
Used for (4#): Digestive (2)[02, 21]; Skin (1)[03]; General and Unspecified (1)[21]
Cognates:
Language contact:

***Anechites nerium* (Aubl.) Urb. (Apocynaceae)**

Spanish names: Lagartija
Indigenous names: Putzi ay[01]
Used by (1*): Zoque[01]
Used for (1#): Skin (1)[01]
Cognates:
Language contact:

***Anemia adiantifolia* (L.) Sw. (Anemiaceae)**

Spanish names:
Indigenous names: Huniil akam pich, chalam ha'[07]
Used by (1*): Huastec[07]
Used for (1#): General and Unspecified (1)[07]
Cognates:
Language contact:

***Anemia affinis* Baker (Anemiaceae)**

Spanish names:
Indigenous names: Nø' ømo pøh[04]
Used by (1*): Mixe[04]
Used for (1#): Female genital (1)[04]
Cognates:
Language contact:

***Anemia mexicana* Klotzsch (Anemiaceae)**

Spanish names:
Indigenous names: Boo'wat ts'ohool[07]
Used by (1*): Huastec[07]
Used for (1#): General and Unspecified (1)[07]
Cognates:
Language contact:

***Anemopaegma chrysanthum* Dugand (Bignoniaceae)**

Spanish names:
Indigenous names: Guatimal[03]
Used by (1*): Zoque[03]
Used for (1#): Female genital (1)[03]
Cognates:
Language contact:

***Anethum graveolens* L. (Apiaceae)**

Spanish names: Eneldo
Indigenous names: Neeld[21]; Guìzh-nêld[23]
Used by (8*): Zoque[01, 02, 03]; Mixe[04]; Quichean Maya[12, 13]; Zapotec[21, 23]
Used for (21#): Digestive (7)[01, 02, 03, 04, 12, 13, 21]; Cardiovascular (1)[12]; Musculoskeletal (1)[02]; Neurological (2)[12, 21]; Psychological (2)[02, 12]; Respiratory (1)[23]; Skin (1)[21]; Pregnancy (1)[03]; Female genital (3)[02, 03, 13]; General and Unspecified (2)[12, 21]
Cognates:
Language contact:

***Angelica mexicana* Vatke (Apiaceae)**

Spanish names: Angélica Macho/ hembra
Indigenous names: Tz'oloj B'aq Aqom[12]
Used by (1*): Quichean Maya[12]
Used for (5#): Digestive (1)[12]; Cardiovascular (1)[12]; Musculoskeletal (1)[12]; Psychological (1)[12]; General and Unspecified (1)[12]
Cognates:
Language contact:

***Anisacanthus quadrifidus* (Vahl) Nees (Acanthaceae)**

Spanish names:
Indigenous names: Gubedundh[22]
Used by (1*): Zapotec[22]
Used for (2#): Digestive (1)[22]; General and Unspecified (1)[22]
Cognates:
Language contact:

***Annona cherimola* Mill. (Annonaceae)**

Spanish names: Anona, chirimoya
Indigenous names: (Pakak) yati[01]; Kilpu'jaka, a'xit kiwi[05]; A'cchit[06]; Oop Čuhum[10]; Pek' Aq'om[12]; K'ewex[20]
Used by (7*): Zoque[01]; Totonac[05, 06]; Yucatecan Maya[10]; Quichean Maya[12]; Western Maya[20]; Nahua[25]
Used for (19#): Digestive (6)[01, 05, 06, 12, 20, 25]; Cardiovascular (1)[06]; Musculoskeletal (2)[01, 12]; Psychological (2)[01, 12]; Respiratory (2)[01, 12]; Skin (2)[06, 10]; Urological (1)[12]; General and Unspecified (3)[01, 10, 12]
Cognates: Toto: ax'it/a'chit;
Language contact: Zoq > Tot; Tot <> Tzeltalan

***Annona glabra* L. (Annonaceae)**

Spanish names: Anona blanca
Indigenous names: Tz'umuy[18]
Used by (1*): Western Maya[18]
Used for (1#): Digestive (1)[18]
Cognates:
Language contact:

**Annona globiflora Schltdl. (Annonaceae)**

Spanish names:
Indigenous names: Aanchuuch, kukayil an chuch, ts'een kukay, ts'aale an chuch, bekal tsuts, tsakam kukay[07]
Used by (1*): Huastec[07]
Used for (5#): Digestive (1)[07]; Respiratory (1)[07]; Skin (1)[07]; Social problems (1)[07]; General and Unspecified (1)[07]
Cognates:
Language contact:

***Annona muricata* L. (Annonaceae)**

Spanish names: Guanabana
Indigenous names: Apak yati/katzu yati[01]; Pasyte yati [02]; Katx yatyi[03]; Guayaban aay[04]; Ajpox[18]; Balagahuanabana'a[21]
Used by (7*): Zoque[01, 02, 03]; Mixe[04]; Yucatecan Maya[09]; Western Maya[18]; Zapotec[21]
Used for (29#): Blood (1)[02]; Digestive (6)[01, 02, 03, 04, 18, 21]; Cardiovascular (2)[01, 02]; Musculoskeletal (2)[01, 21]; Neurological (1)[02]; Psychological (1)[02]; Respiratory (4)[01, 02, 03, 09]; Skin (1)[21]; Endocrine (2)[01, 02]; Urological (2)[01, 02]; Pregnancy (1)[02]; Female genital (1)[01]; General and Unspecified (5)[01, 02, 03, 18, 21]
Cognates: MZ: a(t)i; Zoq: yati;
Language contact:

***Annona purpurea* Moc. & Sessé ex Dunal (Annonaceae)**

Spanish names: Candón; Condon, piñon anona; Ilama
Indigenous names: Kopak yati[02]; Kotsak yatyi[03]; Noajy aky[04]; Guele bajna'a[21]
Used by (4*): Zoque[02, 03]; Mixe[04]; Zapotec[21]
Used for (10#): Digestive (2)[03, 21]; Musculoskeletal (2)[04, 21]; Skin (2)[02, 03]; Pregnancy (1)[03]; Female genital (1)[03]; General and Unspecified (2)[04, 21]
Cognates: MZ: aCi; Zoq: koCak yati;
Language contact:

***Annona reticulata* L. (Annonaceae)**

Spanish names: Anona
Indigenous names: Yati[01]; Yati[02]; Jon yatyi[03]; Akčit kiwi[05]; Kukay, tsak kukay[07]; Oop[09]; Tz'umuy[18]; Guelebidxu'u[21]
Used by (10*): Zoque[01, 02, 03]; Mixe[04]; Totonac[05]; Huastec[07]; Yucatecan Maya[09, 11]; Western Maya[18]; Zapotec[21]
Used for (26#): Digestive (5)[01, 02, 03, 07, 21]; Musculoskeletal (3)[02, 11, 21]; Neurological (1)[02]; Psychological (1)[21]; Respiratory (2)[01, 02]; Skin (2)[05, 09]; Endocrine (1)[02]; Urological (1)[07]; Female genital (1)[21]; Male genital (1)[02]; General and Unspecified (8)[01, 02, 03, 04, 07, 09, 18, 21]
Cognates: Zoq: yati;
Language contact: Zoq > Tot

***Annona* sp. (Annonaceae)**

Spanish names: Anona
Indigenous names: K'uwex[13]; Holobob[17]
Used by (2*): Quichean Maya[13, 17]
Used for (7#): Digestive (1)[13]; Musculoskeletal (1)[13]; Neurological (1)[13]; Respiratory (1)[13]; Skin (1)[13]; General and Unspecified (1)[13]; nd[17]
Cognates:
Language contact:

***Annona squamosa* L. (Annonaceae)**

Spanish names: Caramuyo; Papause; Saramuyo
Indigenous names: Ahakte', kukay[07]; Ts'almuy[09]
Used by (4*): Huastec[07]; Yucatecan Maya[09, 11]; Zapotec[21]
Used for (7#): Digestive (1)[09]; Neurological (1)[11]; Psychological (1)[21]; Respiratory (1)[09]; General and Unspecified (3)[07, 11, 21]
Cognates:
Language contact:

***Anoda cristata* (L.) Schltdl. (Malvaceae)**

Spanish names: Halaches, flor morada, zoquilxochit; Malvavisca; Violea; Violeta, violete silvestre
Indigenous names: Bakan ts'ohool, tsakam bok'ool ts'ohool[07]; Muu ts'ojo[08]; Guìzh-quês, xín-mâlb[23]
Used by (6*): Totonac[06]; Huastec[07, 08]; Quichean Maya[13]; Zapotec[23]; Nahua[26]
Used for (17#): Digestive (2)[07, 08]; Neurological (1)[07]; Respiratory (2)[08, 26]; Skin (4)[07, 08, 13, 23]; Endocrine (1)[07]; Urological (2)[06, 08]; Pregnancy (1)[06]; Female genital (1)[06]; General and Unspecified (3)[07, 08, 13]
Cognates:
Language contact:

[01-28] refer to the study codes in Table 4.1. *Total number of studies citing this taxon #Total number of use-records

***Anoda* sp. (Malvaceae)**

Spanish names: Violeta
Indigenous names:
Used by (1*): Nahua[25]
Used for (2#): Digestive (1)[25]; Urological (1)[25]
Cognates:
Language contact:

***Anredera ramosa (*Moq.) Eliasson (Basellaceae)**

Spanish names: Suelda consuelda
Indigenous names: Gu'[21]
Used by (1*): Zapotec[21]
Used for (2#): Musculoskeletal (1)[21]; General and Unspecified (1)[21]
Cognates:
Language contact:

***Anredera* sp. (Basellaceae)**

Spanish names:
Indigenous names: Pak tyoopi[03]
Used by (1*): Zoque[03]
Used for (1#): Musculoskeletal (1)[03]
Cognates:
Language contact:

***Anredera vesicaria* (Lam.) C.F.Gaertn. (Basellaceae)**

Spanish names: Suelda consuelda
Indigenous names: Tza'a tzoy[01]; Omisal[06]; Ix tuyuum, thuyum[07]; Kaxichel[09]; Gu'u icu'uj[21]
Used by (7*): Zoque[01]; Totonac[06]; Huastec[07]; Yucatecan Maya[09, 11]; Quichean Maya[13]; Zapotec[21]
Used for (12#): Musculoskeletal (6)[06, 07, 09, 11, 13, 21]; Skin (3)[07, 09, 11]; General and Unspecified (3)[01, 07, 21]
Cognates:
Language contact: Hua <> Zap

***Anthemis* sp. (Asteraceae)**

Spanish names:
Indigenous names: Manzaniya[07]
Used by (1*): Huastec[07]
Used for (1#): General and Unspecified (1)[07]
Cognates:
Language contact:

***Anthurium crassinervium* (Jacq.) Schott (Araceae)**

Spanish names: Ojo de piedra, cola de faisan
Indigenous names:
Used by (1*): Yucatecan Maya[11]
Used for (2#): Neurological (1)[11]; General and Unspecified (1)[11]
Cognates:
Language contact:

***Anthurium pentaphyllum (*Aubl.) G. Don (Araceae)**

Spanish names:
Indigenous names: Tsa ay cinco hojas[03]
Used by (1*): Zoque[03]
Used for (1#): Musculoskeletal (1)[03]
Cognates:
Language contact:

[01-28]refer to the study codes in Table 4.1. *Total number of studies citing this taxon #Total number of use-records

***Anthurium scandens* (Aubl.) Engl. (Araceae)**

Spanish names:
Indigenous names: Kiñi ay[03]
Used by (1*): Zoque[03]
Used for (1#): Musculoskeletal (1)[03]
Cognates:
Language contact:

***Anthurium schlechtendalii* Kunth (Araceae)**

Spanish names: Cola de Pavo; Gañate de sope/ quebra piedras/ hoja de sope/ cabeza de guajolote; Hoja de piedra/Oreja de vaca/Riñonina/ lengua de vaca; Raiz de piedra
Indigenous names: Juki ay/ kanji kopak[01]; Tsa ay[03]; Tsak ujts, tsanakøxp'[04]; Bobtun[09]; Xye Pu'[14]
Used by (6*): Zoque[01, 02, 03]; Mixe[04]; Yucatecan Maya[09]; Quichean Maya[14]
Used for (16#): Digestive (3)[01, 03, 04]; Cardiovascular (1)[01]; Psychological (1)[14]; Skin (1)[03]; Endocrine (2)[01, 03]; Urological (3)[01, 02, 03]; Pregnancy (2)[02, 04]; Female genital (2)[04, 09]; General and Unspecified (1)[14]
Cognates: MZ: tsa;
Language contact:

***Anthurium* sp. (Araceae)**

Spanish names: Cola de Pavo
Indigenous names: Xye Pu'[14]; Xtye pu'u[16]
Used by (2*): Quichean Maya[14, 16]
Used for (3#): Cardiovascular (1)[14]; Neurological (1)[16]; Psychological (1)[16]
Cognates: Quich: xye pu';
Language contact:

***Anthurium willdenowii* Kunth (Araceae)**

Spanish names:
Indigenous names: Xchich maus[16]; X ch'cih ma'us[17]
Used by (2*): Quichean Maya[16, 17]
Used for (3#): Neurological (1)[16]; Psychological (1)[16]; nd[17]
Cognates:
Language contact:

***Antigonon flavescens* S.Watson (Polygonaceae)**

Spanish names: Camote de vibora
Indigenous names: Gulachiia[21]; Coacamalte, coamecatl[26]
Used by (2*): Zapotec[21]; Nahua[26]
Used for (3#): Digestive (1)[21]; Musculoskeletal (1)[26]; Skin (1)[26]
Cognates:
Language contact:

***Antigonon leptopus* Hook. & Arn. (Polygonaceae)**

Spanish names: San Pedro
Indigenous names: Rosario aats[04]; Ehtiil t'uthub[07]
Used by (3*): Mixe[04]; Huastec[07]; Yucatecan Maya[09]
Used for (3#): Respiratory (1)[09]; Pregnancy (1)[07]; Female genital (1)[04]
Cognates:
Language contact:

***Antrophyum cajenense* (Desv.) Spreng. (Pteridaceae)**

Spanish names: Costilla de Culebra
Indigenous names: Xcostill kanti, Xco'stii' Kanti[14]
Used by (1*): Quichean Maya[14]
Used for (1#): Skin (1)[14]
Cognates:
Language contact:

***Apeiba tibourbou* Aubl. (Malvaceae)**

Spanish names: Papachote
Indigenous names: Pujki[03]
Used by (1*): Zoque[03]
Used for (3#): Digestive (1)[03]; Skin (1)[03]; Female genital (1)[03]
Cognates:
Language contact:

***Aphelandra aurantiaca* (Scheidw.) Lindl. (Acanthaceae)**

Spanish names:
Indigenous names: Mecha mooya[03]
Used by (1*): Zoque[03]
Used for (1#): Skin (1)[03]
Cognates:
Language contact:

***Aphelandra heydeana* Donn.Sm. (Acanthaceae)**

Spanish names:
Indigenous names: Qani holom chakmut[14]
Used by (1*): Quichean Maya[14]
Used for (1#): Skin (1)[14]
Cognates:
Language contact:

***Aphelandra scabra* (Vahl) Sm. (Acanthaceae)**

Spanish names: Santa María
Indigenous names: Kiñi n¥pin mooya[03]; Xpere` Aj tzo', Xalam Q'ehen, Xjolom Chaqmut, Xbool, Chamat, Mai Q'ehen[14]; Sa'x jolom, chacmut, sita pim[17]
Used by (3*): Zoque[03]; Quichean Maya[14, 17]
Used for (11#): Cardiovascular (1)[14]; Musculoskeletal (1)[14]; Neurological (1)[14]; Skin (2)[03, 14]; Urological (1)[14]; Female genital (2)[03, 14]; Male genital (1)[14]; General and Unspecified (1)[14]; nd[17]
Cognates: Quich: chakmut;
Language contact:

***Aphelandra* sp. (Acanthaceae)**

Spanish names:
Indigenous names: Muuw te'[07]; Roq sosol[16]
Used by (2*): Huastec[07]; Quichean Maya[16]
Used for (3#): Neurological (1)[16]; Psychological (1)[16]; General and Unspecified (1)[07]
Cognates:
Language contact:

***Apium graveolens* L. (Apiaceae)**

Spanish names: Apio
Indigenous names:
Used by (4*): Zoque[01, 02]; Quichean Maya[12]; Nahua[25]
Used for (6#): Digestive (1)[25]; Cardiovascular (1)[02]; Musculoskeletal (1)[01]; Psychological (1)[12]; Respiratory (1)[25]; Endocrine (1)[12]
Cognates:
Language contact:

***Arachis hypogaea* L. (Fabaceae)**

Spanish names: Cacahuate
Indigenous names: Kacwa[03]; Cacau[06]
Used by (2*): Zoque[03]; Totonac[06]
Used for (3#): Psychological (1)[06]; Pregnancy (1)[06]; Female genital (1)[03]
Cognates:
Language contact:

***Arachniodes denticulata* (Sw.) Ching (Dryopteridaceae)**

Spanish names: Calahuala
Indigenous names:
Used by (1*): Quichean Maya[12]
Used for (4#): Blood (1)[12]; Skin (1)[12]; Urological (1)[12]; General and Unspecified (1)[12]
Cognates:
Language contact:

***Arachnothryx buddleioides* (Benth.) Planch. (Rubiaceae)**

Spanish names:
Indigenous names: Puchuch Re Tzuul[14]
Used by (1*): Quichean Maya[14]
Used for (1#): Neurological (1)[14]
Cognates:
Language contact:

***Arachnothryx leucophylla (*Kunth) Planch. (Rubiaceae)**

Spanish names: Huele de noche
Indigenous names:
Used by (1*): Zapotec[21]
Used for (2#): Psychological (1)[21]; General and Unspecified (1)[21]
Cognates:
Language contact:

***Arachnothryx stachyoidea* (Donn.Sm.) Borhidi (Rubiaceae)**

Spanish names:
Indigenous names: Kandel Che[15]
Used by (1*): Quichean Maya[15]
Used for (3#): Psychological (1)[15]; Pregnancy (1)[15]; General and Unspecified (1)[15]
Cognates:
Language contact:

***Arachnothryx villosa* (Hemsl.) Borhidi (Rubiaceae)**

Spanish names:
Indigenous names:
Used by (1*): Zoque[03]
Used for (2#): Skin (1)[03]; Female genital (1)[03]
Cognates:
Language contact:

[01-28]refer to the study codes in Table 4.1. *Total number of studies citing this taxon #Total number of use-records

***Arbutus xalapensis*** **Kunth (Ericaceae)**

Spanish names: Madrón; Madroña
Indigenous names: Uq'a[13]; On te'[20]; Yàg-yǎn[23]
Used by (3*): Quichean Maya[13]; Western Maya[20]; Zapotec[23]
Used for (3#): Digestive (1)[20]; Skin (1)[13]; General and Unspecified (1)[23]
Cognates:
Language contact: Tzeltalan <> Zap

***Archibaccharis auriculata (*****Hemsl.) G.L.Nesom (Asteraceae)**

Spanish names: Atlina
Indigenous names: Caxancapaxtli[28]
Used by (1*): Nahua[28]
Used for (1#): Pregnancy (1)[28]
Cognates:
Language contact:

***Arctostaphylos pungens*** **Kunth (Ericaceae)**

Spanish names: Pingüica; Pingüita
Indigenous names: Yàg-blæ̀æ[23]
Used by (2*): Zapotec[21, 23]
Used for (6#): Digestive (1)[21]; Cardiovascular (1)[21]; Musculoskeletal (1)[23]; Psychological (1)[23]; Skin (1)[23]; Urological (1)[23]
Cognates:
Language contact:

***Ardisia compressa*** **Kunth (Primulaceae)**

Spanish names: Chagalapoli
Indigenous names: Tsuk nok nok[03]
Used by (1*): Zoque[03]
Used for (5#): Digestive (1)[03]; Respiratory (1)[03]; Skin (1)[03]; Female genital (1)[03]; General and Unspecified (1)[03]
Cognates:
Language contact:

***Ardisia escallonioides*** **Schltdl. & Cham. (Primulaceae)**

Spanish names:
Indigenous names: Pelat puchun, wal puchun, pejte[07]
Used by (1*): Huastec[07]
Used for (1#): Digestive (1)[07]
Cognates:
Language contact:

***Ardisia nigrescens*** **Oerst. (Primulaceae)**

Spanish names:
Indigenous names: K'ak'al ilaal paktha'[07]
Used by (1*): Huastec[07]
Used for (1#): General and Unspecified (1)[07]
Cognates:
Language contact:

***Ardisia*** **sp. (Primulaceae)**

Spanish names:
Indigenous names: Xook'num[09]
Used by (1*): Yucatecan Maya[09]
Used for (1#): Respiratory (1)[09]
Cognates:
Language contact:

[01-28]refer to the study codes in Table 4.1. *Total number of studies citing this taxon #Total number of use-records

***Ardisia tuerckheimii* Donn.Sm. (Primulaceae)**

Spanish names:
Indigenous names: Petx uk cuy[03]
Used by (1*): Zoque[03]
Used for (2[#]): Digestive (1)[03]; Skin (1)[03]
Cognates:
Language contact:

***Argemone mexicana* L. (Papaveraceae)**

Spanish names: Cardosanto, chicalote
Indigenous names: Ko tzitzi[02]; San Pedro Agats[04]; Tsolich, [07]; Sac qix, Sak'ak'ix[12]; Ixmucur[13]; Guedxe buloj[21]; Guièts-nîz, guìzh-guièts-nîz[23]; Chicalotl[28]
Used by (11*): Zoque[01, 02]; Mixe[04]; Huastec[07]; Yucatecan Maya[09]; Quichean Maya[12, 13]; Zapotec[21, 23]; Nahua[27, 28]
Used for (22[#]): Digestive (2)[01, 23]; Eye (6)[07, 12, 13, 23, 27, 28]; Neurological (1)[12]; Respiratory (4)[02, 09, 13, 28]; Skin (3)[02, 21, 28]; Urological (2)[02, 09]; General and Unspecified (4)[04, 09, 12, 21]
Cognates: Mayan: ich/ix; Quich: ix;
Language contact: Maya <> Chimalapa Zoq <> Zap <> Nah > Spanish

***Aristolochia anguicida* Jacq. (Aristolochiaceae)**

Spanish names: Guaco
Indigenous names:
Used by (1*): Yucatecan Maya[09]
Used for (3[#]): Digestive (1)[09]; Pregnancy (1)[09]; Female genital (1)[09]
Cognates:
Language contact:

***Aristolochia arborea* Linden (Aristolochiaceae)**

Spanish names: Guaco de arbol
Indigenous names:
Used by (1*): Zoque[03]
Used for (3[#]): Digestive (1)[03]; Cardiovascular (1)[03]; Skin (1)[03]
Cognates:
Language contact:

***Aristolochia asclepiadifolia* Brandegee (Aristolochiaceae)**

Spanish names: Guaco de cochino, Guaco amarillo
Indigenous names:
Used by (1*): Zoque[03]
Used for (9[#]): Digestive (1)[03]; Cardiovascular (1)[03]; Musculoskeletal (1)[03]; Psychological (1)[03]; Skin (1)[03]; Urological (1)[03]; Pregnancy (1)[03]; Female genital (1)[03]; General and Unspecified (1)[03]
Cognates:
Language contact:

***Aristolochia grandiflora* Sw. (Aristolochiaceae)**

Spanish names: Guaco
Indigenous names: Patz pim[16]
Used by (2*): Zoque[03]; Quichean Maya[16]
Used for (5[#]): Digestive (1)[03]; Musculoskeletal (1)[03]; Neurological (1)[16]; Psychological (1)[16]; Skin (1)[03]
Cognates:
Language contact:

### *Aristolochia leuconeura* Linden (Aristolochiaceae)

Spanish names: Guaco; Hítamo real
Indigenous names:
Used by (2*): Zoque[01, 02]
Used for (7#): Digestive (2)[01, 02]; Respiratory (1)[02]; Skin (1)[02]; Urological (1)[02]; Pregnancy (1)[02]; Female genital (1)[02]
Cognates:
Language contact:

### *Aristolochia littoralis* Parodi (Aristolochiaceae)

Spanish names:
Indigenous names: Itsaan an ohob ilaal[07]
Used by (1*): Huastec[07]
Used for (1#): Respiratory (1)[07]
Cognates:
Language contact:

### *Aristolochia macrophylla* Lam. (Aristolochiaceae)

Spanish names: Guaco corriente
Indigenous names:
Used by (1*): Zapotec[21]
Used for (7#): Digestive (1)[21]; Cardiovascular (1)[21]; Musculoskeletal (1)[21]; Neurological (1)[21]; Skin (1)[21]; Pregnancy (1)[21]; General and Unspecified (1)[21]
Cognates:
Language contact:

### *Aristolochia maxima* Jacq. (Aristolochiaceae)

Spanish names: Guaco; Guaco castillo; Hitámo real
Indigenous names: Tzay jäyä/nä yutza[01]; Nojta wáko[19]
Used by (3*): Zoque[01]; Yucatecan Maya[09]; Western Maya[19]
Used for (7#): Digestive (3)[01, 09, 19]; Skin (1)[01]; Pregnancy (1)[09]; Female genital (2)[09, 19]
Cognates:
Language contact:

### *Aristolochia odoratissima* L. (Aristolochiaceae)

Spanish names: Guaco de castilla; Jocobal, cocobá
Indigenous names: Cocobá[18]
Used by (2*): Zoque[03]; Western Maya[18]
Used for (5#): Digestive (2)[03, 18]; Musculoskeletal (1)[03]; Respiratory (1)[18]; Skin (1)[03]
Cognates:
Language contact:

### *Aristolochia orbicularis* Duch. (Aristolochiaceae)

Spanish names:
Indigenous names: Ohob ilaal[07]
Used by (1*): Huastec[07]
Used for (2#): Musculoskeletal (1)[07]; Respiratory (1)[07]
Cognates:
Language contact:

[01-28]refer to the study codes in Table 4.1. *Total number of studies citing this taxon #Total number of use-records

***Aristolochia ovalifolia* Duch. (Aristolochiaceae)**

Spanish names: Guaco (fino); Guaco blanco
Indigenous names:
Used by (2*): Zoque[03]; Zapotec[21]
Used for (13#): Digestive (2)[03, 21]; Eye (1)[03]; Cardiovascular (1)[21]; Musculoskeletal (2)[03, 21]; Neurological (1)[21]; Skin (2)[03, 21]; Pregnancy (2)[03, 21]; Female genital (1)[03]; General and Unspecified (1)[21]
Cognates:
Language contact:

***Aristolochia pentandra* Jacq. (Aristolochiaceae)**

Spanish names: Camotillo; Guaco; Guaco castillo
Indigenous names: Paj m¥n[03]; Ajwacu'[18]
Used by (4*): Zoque[01, 03]; Yucatecan Maya[09]; Western Maya[18]
Used for (13#): Digestive (4)[01, 03, 09, 18]; Musculoskeletal (2)[01, 18]; Neurological (1)[18]; Skin (2)[03, 18]; Female genital (3)[01, 03, 18]; General and Unspecified (1)[01]
Cognates:
Language contact: Highland Popoluca <> Chortí

***Aristolochia pilosa* Kunth (Aristolochiaceae)**

Spanish names: Hierba de Sope
Indigenous names: So'osol Q'ehen [14]
Used by (1*): Quichean Maya[14]
Used for (1#): Psychological (1)[14]
Cognates:
Language contact:

***Aristolochia* sp. (Aristolochiaceae)**

Spanish names: Guaco
Indigenous names: Xjolom Kamenaq[14]; Patz pim[14]; Xa'ab maus[16]; Ncuàan-dzéb-zhòmbrêl[23]
Used by (5*): Zoque[03]; Quichean Maya[14, 16]; Zapotec[23]; Nahua[26]
Used for (18#): Digestive (3)[03, 23, 26]; Musculoskeletal (2)[03, 26]; Neurological (3)[03, 14, 26]; Psychological (1)[16]; Respiratory (1)[03]; Skin (3)[03, 14, 26]; Female genital (1)[03]; General and Unspecified (4)[03, 14, 23, 23]
Cognates:
Language contact:

***Aristolochia trilobata* L. (Aristolochiaceae)**

Spanish names: Contrebo
Indigenous names:
Used by (1*): Yucatecan Maya[10]
Used for (1#): Digestive (1)[10]
Cognates:
Language contact:

***Arnica montana* L. (Asteraceae)**

Spanish names: Arnica
Indigenous names: Maan huitz[08]
Used by (1*): Huastec[08]
Used for (5#): Digestive (1)[08]; Musculoskeletal (1)[08]; Respiratory (1)[08]; Skin (1)[08]; Endocrine (1)[08]
Cognates:
Language contact:

[01-28]refer to the study codes in Table 4.1. *Total number of studies citing this taxon #Total number of use-records

***Arracacia atropurpurea*** **(Lehm.) Benth. & Hook.f. ex Hemsl. (Apiaceae)**

Spanish names: Hierba del borrego
Indigenous names: Ts'ojolil an borrego[08]
Used by (1*): Huastec[08]
Used for (3#): Musculoskeletal (1)[08]; Respiratory (1)[08]; General and Unspecified (1)[08]
Cognates:
Language contact:

***Arracacia bracteata*** **J.M.Coult. & Rose (Apiaceae)**

Spanish names: Valeriana, Chichipate
Indigenous names:
Used by (1*): Quichean Maya[12]
Used for (3#): Cardiovascular (1)[12]; Neurological (1)[12]; Psychological (1)[12]
Cognates:
Language contact:

***Artemisia absinthium*** **L. (Asteraceae)**

Spanish names: Ajenjo
Indigenous names: Qa'yes, k'a q'ayes[13]; Guìzh-maêstr[23]
Used by (9*): Zoque[01]; Mixe[04]; Totonac[05]; Quichean Maya[12, 13]; Zapotec[21, 23]; Nahua[26, 27]
Used for (23#): Blood (1)[12]; Digestive (9)[01, 04, 05, 12, 13, 21, 23, 26, 27]; Cardiovascular (1)[12]; Neurological (2)[12, 23]; Psychological (1)[05]; Respiratory (1)[13]; Endocrine (2)[12, 13]; Pregnancy (3)[13, 21, 27]; Female genital (2)[12, 13]; General and Unspecified (1)[12]
Cognates:
Language contact:

***Artemisia ludoviciana*** **Nutt. (Asteraceae)**

Spanish names: Estafiate
Indigenous names: Uksuk[01]; Poma ay[03]; Teen wits, teen ts'ohool[07]; Si'isim[09]; Guìzh-guièe-rò[23]; Iztahuatl[26]; Iztauhyaitl[28]
Used by (14*): Zoque[01, 02, 03]; Mixe[04]; Totonac[05]; Huastec[07]; Yucatecan Maya[09]; Western Maya[18, 19]; Zapotec[21, 23]; Nahua[25, 26, 28]
Used for (39#): Digestive (12)[01, 02, 03, 04, 05, 07, 09, 21, 23, 25, 26, 28]; Ear (1)[07]; Musculoskeletal (1)[01]; Neurological (1)[25]; Psychological (3)[01, 18, 19]; Respiratory (3)[01, 03, 23]; Skin (4)[03, 05, 07, 28]; Endocrine (1)[02]; Pregnancy (2)[03, 05]; Female genital (4)[01, 02, 03, 05]; General and Unspecified (7)[01, 03, 05, 07, 19, 25, 28]
Cognates: Nahua: iztauhyatl;
Language contact:

***Artemisia mexicana*** **Willd. (Asteraceae)**

Spanish names: Estafiate; Incienso, Zi' zim
Indigenous names: Ten ts'ojol[08]; Tsintsin[11]; Pom[18]
Used by (3*): Huastec[08]; Yucatecan Maya[11]; Western Maya[18]
Used for (5#): Digestive (3)[08, 11, 18]; Musculoskeletal (1)[18]; General and Unspecified (1)[18]
Cognates:
Language contact: Hua <> Yuc

**Artemisia sp. (Asteraceae)**

Spanish names: Ajenjo; Ajenjo, alcanfor, ensencio, estafiate, incienso; Yerba maestra
Indigenous names: Sak sak, xak axaka, xaq' xaq'[13]
Used by (3*): Quichean Maya[12, 13]; Zapotec[22]
Used for (10#): Digestive (2)[13, 22]; Psychological (1)[22]; Skin (1)[22]; Endocrine (3)[12, 13, 22]; Pregnancy (1)[13]; Female genital (1)[13]; General and Unspecified (1)[22]
Cognates:
Language contact:

[01-28]refer to the study codes in Table 4.1. *Total number of studies citing this taxon #Total number of use-records

***Artemisia stelleriana* Besser (Asteraceae)**

Spanish names: Estafiate blanco
Indigenous names: Guichuch roob[21]
Used by (1*): Zapotec[21]
Used for (1#): Digestive (1)[21]
Cognates:
Language contact:

***Artemisia vulgaris* L. (Asteraceae)**

Spanish names: Ajenjo; Artemisa
Indigenous names: Tsakam ten huitz[08]; K'atab' Chaj[14]
Used by (4*): Huastec[08]; Quichean Maya[12, 14]; Nahua[25]
Used for (9#): Digestive (4)[08, 12, 14, 25]; Cardiovascular (1)[14]; Musculoskeletal (1)[12]; Neurological (1)[08]; Respiratory (2)[12, 14]
Cognates:
Language contact:

***Arthrostemma ciliatum* Pav. ex D. Don (Melastomataceae)**

Spanish names: Caña agria
Indigenous names: Katzu aksa/ katzu tane/ katzu rane[01]; Katxu kanapoki[03]; X'cut'ni[06]; Tzeleq' Ajsaq'[14]; Rok' za'ak[17]; Paj um'um wamal, pajal wamal, majk'al akan wamal, ik'al momol[20]; Nidaii[21]
Used by (7*): Zoque[01, 03]; Totonac[06]; Quichean Maya[14, 17]; Western Maya[20]; Zapotec[21]
Used for (23#): Digestive (4)[01, 06, 20, 21]; Eye (1)[01]; Cardiovascular (1)[01]; Musculoskeletal (1)[01]; Neurological (2)[01, 14]; Psychological (1)[01]; Respiratory (2)[01, 20]; Skin (3)[06, 14, 20]; Endocrine (1)[03]; Urological (3)[01, 03, 14]; Male genital (1)[14]; General and Unspecified (2)[01, 14]; nd[17]
Cognates: Zoq: katsu an; CoreM: ak; Quich: sak;
Language contact: Chiapas Zoq <> CoreM

***Arthrostemma parvifolium* Cogn. (Melastomataceae)**

Spanish names: Corona de Cristo
Indigenous names: Rixiiji Tz'i, Xoy Q'ehen, Kaq'i pim[14]
Used by (1*): Quichean Maya[14]
Used for (5#): Digestive (1)[14]; Musculoskeletal (1)[14]; Neurological (1)[14]; Endocrine (1)[14]; General and Unspecified (1)[14]
Cognates:
Language contact:

***Artocarpus altilis* (Parkinson ex F.A.Zorn) Fosberg (Moraceae)**

Spanish names: Castaña; Castaño
Indigenous names:
Used by (2*): Zoque[01]; Western Maya[18]
Used for (3#): Psychological (1)[01]; Skin (1)[01]; Female genital (1)[18]
Cognates:
Language contact:

***Arundinella deppeana* Nees (Poaceae)**

Spanish names: Cola de Caballo
Indigenous names: Xye' Kawaay[14]
Used by (1*): Quichean Maya[14]
Used for (2#): Endocrine (1)[14]; General and Unspecified (1)[14]
Cognates:
Language contact:

***Arundo donax* L. (Poaceae)**

Spanish names: Caña de Castillo; Carigo; Carizo; Carriz
Indigenous names: Kape[01]; Pakaab[07]
Used by (5*): Zoque[01]; Huastec[07]; Quichean Maya[12]; Nahua[24, 26]
Used for (8#): Musculoskeletal (1)[01]; Neurological (1)[01]; Psychological (1)[01]; Skin (2)[07, 26]; Pregnancy (1)[24]; General and Unspecified (2)[07, 12]
Cognates:
Language contact: Chiapas Zoq <> Hua

***Asclepias contrayerba* Sessé & Moc. (Apocynaceae)**

Spanish names: Jicaco
Indigenous names:
Used by (1*): Zoque[01]
Used for (3#): Digestive (1)[01]; Endocrine (1)[01]; General and Unspecified (1)[01]
Cognates:
Language contact:

***Asclepias curassavica* L. (Apocynaceae)**

Spanish names: Quebramuela
Indigenous names: Tusy put/witäm toya remedio /tusy kuy/ wenguy tätz [01]; Kiñi n¥pin mooya, Misi kuts¥k[03]; Paxebaa[04]; Papuyut tawuan cajli, pinatawuan[05]; Pinatawan[05]; Punchiix wits[07]; Anal, Ik'abal, Polkuts[09]; Ratzum' Pe'pem[14]; Guìzh-mĕy, guièe-mĕy, guìzh-yòob-lây, guìzh-lây, guièe-zìè[23]; Tacaxihuitl[26]
Used by (11*): Zoque[01, 02, 03]; Mixe[04]; Totonac[05]; Huastec[07]; Yucatecan Maya[09]; Quichean Maya[13, 14]; Zapotec[23]; Nahua[26]
Used for (27#): Digestive (5)[03, 04, 05, 07, 14]; Cardiovascular (3)[02, 05, 05]; Musculoskeletal (2)[01, 03]; Neurological (7)[01, 03, 05, 07, 09, 13, 23]; Skin (8)[01, 02, 03, 05, 05, 07, 14, 26]; General and Unspecified (2)[05, 07]
Cognates:
Language contact: Highland Popoluca <> Yuc

***Asclepias fournieri* Woodson (Apocynaceae)**

Spanish names:
Indigenous names: Guìzh-cònêf[23]
Used by (1*): Zapotec[23]
Used for (1#): Musculoskeletal (1)[23]
Cognates:
Language contact:

***Asclepias glaucescens* Kunth (Apocynaceae)**

Spanish names:
Indigenous names: Tyuuxi[03]
Used by (1*): Zoque[03]
Used for (1#): Digestive (1)[03]
Cognates:
Language contact:

***Asclepias linaria* Cav. (Apocynaceae)**

Spanish names: Romero de monte
Indigenous names:
Used by (1*): Nahua[27]
Used for (3#): Digestive (1)[27]; Pregnancy (1)[27]; General and Unspecified (1)[27]
Cognates:
Language contact:

[01-28]refer to the study codes in Table 4.1. *Total number of studies citing this taxon #Total number of use-records

***Asclepias similis* Hemsl. (Apocynaceae)**

Spanish names:
Indigenous names: Utu m'zat[13]
Used by (1*): Quichean Maya[13]
Used for (2#): Skin (1)[13]; Pregnancy (1)[13]
Cognates:
Language contact:

***Asclepias* sp. (Apocynaceae)**

Spanish names:
Indigenous names: Oiloxtli[26]
Used by (1*): Nahua[26]
Used for (1#): Skin (1)[26]
Cognates:
Language contact:

***Aspidosperma megalocarpon* Müll.Arg. (Apocynaceae)**

Spanish names: Malereo
Indigenous names: PiČmaŠ[10]
Used by (1*): Yucatecan Maya[10]
Used for (1#): Digestive (1)[10]
Cognates:
Language contact:

***Asplenium pumilum* Sw. (Aspleniaceae)**

Spanish names:
Indigenous names: Xapachik ts'ohool, koo' ha', bakan ts'ohool, ehtiil weew koxol[07]
Used by (1*): Huastec[07]
Used for (3#): Cardiovascular (1)[07]; Neurological (1)[07]; Endocrine (1)[07]
Cognates:
Language contact:

***Aster subulatus* (Michx.) Hort. ex Michx. (Asteraceae)**

Spanish names: Hierba del burro
Indigenous names: Jok poy tsus p¥k, Mok jipxi ay, Jok poy[03]
Used by (1*): Zoque[03]
Used for (3#): Digestive (1)[03]; Skin (1)[03]; Female genital (1)[03]
Cognates:
Language contact:

***Astraea lobata* (L.) Klotzsch (Euphorbiaceae)**

Spanish names:
Indigenous names:
Used by (1*): Huastec[07]
Used for (1#): Skin (1)[07]
Cognates:
Language contact:

***Astrocaryum mexicanum* Liebm. ex Mart. (Arecaceae)**

Spanish names: Coquito
Indigenous names: Tzytzyon kuy täp[01]
Used by (2*): Zoque[01, 02]
Used for (2#): Musculoskeletal (1)[02]; General and Unspecified (1)[01]
Cognates:
Language contact:

[01-28]refer to the study codes in Table 4.1. *Total number of studies citing this taxon #Total number of use-records

**_Astrolepis laevis_ (M. Martens & Galeotti) Mickel (Pteridaceae)**

Spanish names:
Indigenous names: Guìzh-zhĭil-wlâgw, guìzh-wlâgw, yàg-zhĭil-wlâgw, guìzh-zhĭil-dán[23]
Used by (1*): Zapotec[23]
Used for (1#): Skin (1)[23]
Cognates:
Language contact:

**_Astrolepis sinuata_ (Lag. ex Sw.) D.M. Benham & Windham (Pteridaceae)**

Spanish names:
Indigenous names: Ncuàan-dzéb-mæ̀cw[23]
Used by (1*): Zapotec[23]
Used for (1#): General and Unspecified (1)[23]
Cognates:
Language contact:

**_Astronium graveolens_ Jacq. (Anacardiaceae)**

Spanish names: Gateado; Jobillo; Tulin
Indigenous names: Putoki[03]
Used by (4*): Zoque[01, 03]; Yucatecan Maya[09, 11]
Used for (10#): Digestive (2)[01, 03]; Neurological (1)[03]; Respiratory (2)[03, 09]; Skin (2)[03, 11]; Female genital (1)[01]; General and Unspecified (2)[01, 03]
Cognates:
Language contact:

**_Ateleia albolutescens_ Mohlenbr. (Fabaceae)**

Spanish names: Palo de pozole
Indigenous names: Jam kuy[02]
Used by (1*): Zoque[02]
Used for (1#): Musculoskeletal (1)[02]
Cognates:
Language contact:

**_Ateleia_ sp. (Fabaceae)**

Spanish names:
Indigenous names: Ta'te', thak te', kax te'[07]
Used by (1*): Huastec[07]
Used for (1#): Skin (1)[07]
Cognates:
Language contact:

**_Attalea rostrata_ Oerst. (Arecaceae)**

Spanish names: Corozo, coquito
Indigenous names: Misi'yu[18]
Used by (1*): Western Maya[18]
Used for (3#): Skin (1)[18]; Endocrine (1)[18]; General and Unspecified (1)[18]
Cognates:
Language contact:

**_Augusta rivalis_ (Benth.) J.H.Kirkbr. (Rubiaceae)**

Spanish names:
Indigenous names: Pop n¥ang mooya[03]
Used by (1*): Zoque[03]
Used for (2#): Ear (1)[03]; General and Unspecified (1)[03]
Cognates:
Language contact:

[01-28] refer to the study codes in Table 4.1. *Total number of studies citing this taxon #Total number of use-records

***Avena sativa* L. (Poaceae)**

Spanish names: Avena
Indigenous names:
Used by (3*): Zoque[02]; Quichean Maya[12, 13]
Used for (5#): Blood (1)[13]; Digestive (1)[13]; Respiratory (1)[13]; Endocrine (1)[02]; General and Unspecified (1)[12]
Cognates:
Language contact:

***Averrhoa carambola* L. (Oxalidaceae)**

Spanish names: Carambola
Indigenous names:
Used by (3*): Zoque[01, 02]; Western Maya[18]
Used for (3#): Digestive (1)[02]; Urological (1)[18]; Male genital (1)[01]
Cognates:
Language contact:

***Axonopus compressus* (Sw.) P.Beauv. (Poaceae)**

Spanish names: Grama
Indigenous names: K'im[13]
Used by (1*): Quichean Maya[13]
Used for (2#): Digestive (1)[13]; Urological (1)[13]
Cognates:
Language contact:

***Azadirachta indica* A.Juss. (Meliaceae)**

Spanish names: Nim; Nin
Indigenous names:
Used by (3*): Zoque[01, 02]; Huastec[08]
Used for (10#): Blood (1)[02]; Cardiovascular (2)[02, 08]; Neurological (1)[02]; Skin (1)[02]; Endocrine (3)[01, 02, 08]; General and Unspecified (2)[01, 08]
Cognates:
Language contact:

***Aztecaster pyramidatus* (B.L.Rob. & Greenm.) G.L.Nesom (Asteraceae)**

Spanish names: Romero del campo
Indigenous names:
Used by (1*): Zapotec[22]
Used for (1#): General and Unspecified (1)[22]
Cognates:
Language contact:

***Baccharis conferta* Kunth (Asteraceae)**

Spanish names: Escoba, escobilla china
Indigenous names:
Used by (1*): Nahua[26]
Used for (4#): Digestive (1)[26]; Musculoskeletal (1)[26]; Skin (1)[26]; General and Unspecified (1)[26]
Cognates:
Language contact:

**_Baccharis inamoena_ Gardner (Asteraceae)**

Spanish names: Santo Domingo
Indigenous names: Ok momon tsyawit/ ak manhgu tzyay/ ak mon tzyay[01]; Tsay mayorga[03]; Thintsiil, thi'al tsiib, ts'itsiimbe ts'ohool, ts'itsin ts'ohool, li'ax wits, akaanom ts'ohool, pamchaa ts'ohool[07]; Raxkej Q'os[12]; Uje k'ik', toj q'us, toj quas[13]; Bisik Q'aham Q'ehen, Re' saq' tiqobl[14]; Tzelek zaâk[16]; Vara xik', valak xik, saki xijch[20]
Used by (10*): Zoque[01, 03]; Huastec[07]; Quichean Maya[12, 13, 14, 16, 17]; Western Maya[20]; Nahua[26]
Used for (34#): Blood (1)[07]; Digestive (3)[12, 20, 26]; Cardiovascular (2)[12, 14]; Musculoskeletal (4)[03, 07, 12, 13]; Neurological (2)[07, 16]; Psychological (2)[12, 14]; Respiratory (3)[12, 14, 20]; Skin (5)[03, 07, 12, 13, 14]; Endocrine (1)[13]; Pregnancy (3)[07, 12, 13]; General and Unspecified (7)[01, 03, 07, 12, 13, 14, 20]; nd[17]
Cognates: Zoq: tsay; Mayan: ak; CoreM: sak; Quich: q'os/q'us, sak;
Language contact: Chiapas Zoq <> Hua

**_Baccharis salicina_ Torr. & A.Gray (Asteraceae)**

Spanish names: Chamiso; Chamizo blanco; Chilca
Indigenous names: Näng pojä[02]; Badzu'umij, guajgu'u[21]; Yak šeh[22]; Yàg-yàaz-nquǐts, yàg-yàaz-ròbáa[23]
Used by (5*): Zoque[02]; Quichean Maya[12]; Zapotec[21, 22, 23]
Used for (13#): Digestive (2)[22, 23]; Musculoskeletal (1)[22]; Skin (2)[12, 21]; Pregnancy (2)[21, 22]; Female genital (2)[02, 21]; General and Unspecified (4)[02, 12, 21, 22]
Cognates:
Language contact:

**_Baccharis serraefolia_ DC. (Asteraceae)**

Spanish names: Té de Monte
Indigenous names: Vara xik', valak xik, vach' te' vomol, bak te'[20]
Used by (2*): Quichean Maya[12]; Western Maya[20]
Used for (6#): Digestive (1)[20]; Ear (1)[20]; Respiratory (1)[20]; Skin (1)[20]; General and Unspecified (2)[12, 20]
Cognates:
Language contact:

**_Baccharis_ sp. (Asteraceae)**

Spanish names: Chamizo; Té de monte
Indigenous names: Yàg-yàaz[23]; Yàg-yàaz-làs[23]
Used by (2*): Quichean Maya[13]; Zapotec[23]
Used for (8#): Digestive (2)[23, 23]; Ear (1)[23]; Respiratory (1)[23]; Skin (1)[13]; Female genital (1)[13]; General and Unspecified (2)[13, 23]
Cognates:
Language contact:

**_Baccharis vaccinioides_ Kunth (Asteraceae)**

Spanish names: Arayan, raijan
Indigenous names: K'ichob', ixchop[13]; Mes te'[20]
Used by (3*): Zoque[01]; Quichean Maya[13]; Western Maya[20]
Used for (11#): Digestive (2)[13, 20]; Musculoskeletal (1)[20]; Neurological (2)[01, 20]; Respiratory (2)[13, 20]; Pregnancy (1)[13]; Female genital (1)[13]; General and Unspecified (2)[13, 20]
Cognates:
Language contact:

**_Bacopa_ sp. (Plantaginaceae)**

Spanish names:
Indigenous names: Tsakam wiichab, wistiil a k'iichaa, ts'akat ti ichiich, chakam thutsub[07]
Used by (1*): Huastec[07]
Used for (3#): Psychological (1)[07]; Skin (1)[07]; General and Unspecified (1)[07]
Cognates:
Language contact:

[01-28] refer to the study codes in Table 4.1. *Total number of studies citing this taxon #Total number of use-records

***Bactris major* Jacq. (Arecaceae)**

Spanish names: Jahuacte, chiquiyul
Indigenous names: Chäkyu'[18]
Used by (1*): Western Maya[18]
Used for (1#): Respiratory (1)[18]
Cognates:
Language contact:

***Bambusa* sp. (Poaceae)**

Spanish names:
Indigenous names: Masi caña[03]
Used by (1*): Zoque[03]
Used for (1#): General and Unspecified (1)[03]
Cognates:
Language contact:

***Barkeria skinneri (*Bateman ex Lindl.) Paxton (Orchidaceae)**

Spanish names: Candelaria
Indigenous names:
Used by (1*): Quichean Maya[12]
Used for (1#): Urological (1)[12]
Cognates:
Language contact:

***Barkleyanthus salicifolius (*Kunth) H.Rob. & Brettell (Asteraceae)**

Spanish names: Chamizo amarillo
Indigenous names: Yàg-yàaz-nguĕts[23]
Used by (1*): Zapotec[23]
Used for (2#): Neurological (1)[23]; General and Unspecified (1)[23]
Cognates:
Language contact:

***Bartlettina* sp. (Asteraceae)**

Spanish names: Guaco
Indigenous names:
Used by (1*): Zoque[03]
Used for (1#): Digestive (1)[03]
Cognates:
Language contact:

***Bauhinia divaricata* L. (Fabaceae)**

Spanish names: Pata de vaca
Indigenous names: Xitiks[03]; šmakan siyan toro, makačupi wacaš[05]; Tatil bichim[07]; May vaca[09]; Xa'ab Wacax[14]
Used by (7*): Zoque[01, 03]; Totonac[05]; Huastec[07]; Yucatecan Maya[09, 11]; Quichean Maya[14]
Used for (21#): Digestive (4)[01, 03, 05, 07]; Musculoskeletal (1)[14]; Respiratory (2)[03, 09]; Skin (4)[03, 05, 07, 09]; Endocrine (2)[03, 09]; Urological (3)[03, 07, 09]; Female genital (1)[03]; General and Unspecified (4)[03, 07, 11, 14]
Cognates:
Language contact:

***Bauhinia glabra* Jacq. (Fabaceae)**

Spanish names: Mecate de Mico
Indigenous names: Xtab'imax[14]
Used by (1*): Quichean Maya[14]
Used for (1#): Endocrine (1)[14]
Cognates:
Language contact:

[01-28]refer to the study codes in Table 4.1. *Total number of studies citing this taxon #Total number of use-records

***Bauhinia herrerae*** **(Britton & Rose) Standl. & Stey (Fabaceae)**

Spanish names:
Indigenous names: Kibix, May vaca rojo, Ts'ulubtok'[09]
Used by (1*): Yucatecan Maya[09]
Used for (2#): Digestive (1)[09]; Respiratory (1)[09]
Cognates:
Language contact:

***Bauhinia ungulata*** **L. (Fabaceae)**

Spanish names: Pativaca roja
Indigenous names: Xitiks[03]
Used by (1*): Zoque[03]
Used for (2#): Digestive (1)[03]; General and Unspecified (1)[03]
Cognates:
Language contact:

***Bdallophytum americanum*** **(R.Br.) Eichler ex Solms (Cytinaceae)**

Spanish names:
Indigenous names: Boo'wat wits, chumil a tsakah[07]
Used by (1*): Huastec[07]
Used for (1#): Neurological (1)[07]
Cognates:
Language contact:

***Begonia fischeri*** **Schrank (Begoniaceae)**

Spanish names: Caña agria
Indigenous names:
Used by (1*): Zoque[03]
Used for (1#): Urological (1)[03]
Cognates:
Language contact:

***Begonia glabra*** **Aubl. (Begoniaceae)**

Spanish names:
Indigenous names: Kak'i pim, pa' ulul[17]
Used by (2*): Zoque[03]; Quichean Maya[17]
Used for (2#): Skin (1)[03]; nd[17]
Cognates:
Language contact:

***Begonia heracleifolia*** **Cham. & Schltdl. (Begoniaceae)**

Spanish names: Caña agria, mano de león
Indigenous names: Leon k¥, Katxu kanapoki[03]; Nøtem ujts[04]; Xocovole[05]; X'cut'ni[06]; Kaq'i Paulul, Paulul Q'ehen[14]; Xak' peck, pa' ulul[17]; Xoxoco[26]
Used by (9*): Zoque[01, 03]; Mixe[04]; Totonac[05, 06]; Quichean Maya[14, 17]; Zapotec[21]; Nahua[26]
Used for (21#): Digestive (3)[03, 05, 06]; Ear (1)[03]; Cardiovascular (1)[14]; Musculoskeletal (1)[21]; Psychological (1)[14]; Skin (5)[01, 03, 06, 14, 21]; Urological (3)[03, 14, 26]; Female genital (2)[04, 14]; Male genital (1)[14]; General and Unspecified (2)[14, 21]; nd[17]
Cognates: Toto: x(o)co/xcu; Quich: Cak, pa'ulul;
Language contact: Nah <> Tot <> Kekchí

[01-28]refer to the study codes in Table 4.1. *Total number of studies citing this taxon #Total number of use-records

**_Begonia incarnata_ Link & Otto (Begoniaceae)**

Spanish names: Chocoyul San Miguel del Angel
Indigenous names: X'cut'ni[06]
Used by (1*): Totonac[06]
Used for (1#): Skin (1)[06]
Cognates:
Language contact:

**_Begonia nelumbiifolia_ Cham. & Schltdl. (Begoniaceae)**

Spanish names: Begonia, xocoyol
Indigenous names: Kobakt¥k ay[03]; X'cut'ni[06]; Alte'begonia[08]; Saq'i Pa'ulul[14]; Pa' ulul[17]
Used by (7*): Zoque[02, 03]; Totonac[05, 06]; Huastec[08]; Quichean Maya[14, 17]
Used for (16#): Digestive (2)[05, 08]; Musculoskeletal (3)[03, 08, 14]; Neurological (2)[02, 08]; Skin (5)[02, 03, 05, 06, 14]; Endocrine (1)[08]; Female genital (1)[03]; General and Unspecified (1)[08]; nd[17]
Cognates: Quich: pa'ulul;
Language contact:

**_Begonia_ sp. (Begoniaceae)**

Spanish names: Hierba de purga
Indigenous names: Bok'ool uxkwe', bakaanil a iits'[07]; Paulul Q'ehen[14]
Used by (3*): Zoque[01]; Huastec[07]; Quichean Maya[14]
Used for (7#): Digestive (1)[01]; Eye (1)[07]; Skin (2)[01, 14]; Urological (2)[07, 14]; General and Unspecified (1)[07]
Cognates:
Language contact:

**_Begonia wallichiana_ Lehm. (Begoniaceae)**

Spanish names:
Indigenous names: Hiliyi ts'ohool, ts'amuxlaab ts'ohhol, tsakam ts'amuts k'animiim, huntal an pux lat'em[07]
Used by (1*): Huastec[07]
Used for (3#): Skin (1)[07]; Endocrine (1)[07]; General and Unspecified (1)[07]
Cognates:
Language contact:

**_Bernardia dodecandra_ (Sessé ex Cav.) Govaerts (Euphorbiaceae)**

Spanish names:
Indigenous names: Tsinat mahul, kwe' mahunal, liston te'[07]
Used by (1*): Huastec[07]
Used for (1#): Female genital (1)[07]
Cognates:
Language contact:

**_Beschorneria_ sp. (Asparagaceae)**

Spanish names:
Indigenous names: Tsakam tsi'iim[07]
Used by (1*): Huastec[07]
Used for (3#): Musculoskeletal (1)[07]; Skin (1)[07]; General and Unspecified (1)[07]
Cognates:
Language contact:

**_Besleria laxiflora_ Benth. (Gesneriaceae)**

Spanish names: Cabeza de Camarón
Indigenous names: Xjolom Ma'nzaan[14]; Ke' hal pim[17]
Used by (2*): Quichean Maya[14, 17]
Used for (2#): Skin (1)[14]; nd[17]
Cognates:
Language contact:

[01-28]refer to the study codes in Table 4.1. *Total number of studies citing this taxon #Total number of use-records

***Beta vulgaris* L. (Amaranthaceae)**

Spanish names: Acelga, remolacha; Betabel
Indigenous names:
Used by (3*): Zoque[01, 02]; Quichean Maya[12]
Used for (6#): Digestive (1)[01]; Cardiovascular (1)[12]; Psychological (1)[12]; Skin (1)[12]; General and Unspecified (2)[02, 12]
Cognates:
Language contact:

***Bidens aurea* (Aiton) Sherff (Asteraceae)**

Spanish names:
Indigenous names: Tzompiltecle[26]
Used by (1*): Nahua[26]
Used for (1#): Pregnancy (1)[26]
Cognates:
Language contact:

***Bidens pilosa* L. (Asteraceae)**

Spanish names: Mozote, aceitilla
Indigenous names: Uentex ay[03]; X'tiyu snapapap[06]; Kelem, kelem wits[07]; Xub'ay[14]; Guièe-tǐ-nquǐts[23]; Mózotl[25]; Mozotl[26]
Used by (8*): Zoque[03]; Totonac[05, 06]; Huastec[07]; Quichean Maya[14]; Zapotec[23]; Nahua[25, 26]
Used for (24#): Digestive (3)[05, 06, 25]; Cardiovascular (1)[05]; Musculoskeletal (2)[06, 14]; Neurological (2)[03, 14]; Psychological (3)[03, 14, 23]; Respiratory (3)[07, 25, 26]; Skin (3)[03, 07, 25]; Endocrine (1)[25]; Urological (2)[06, 26]; Female genital (1)[03]; General and Unspecified (3)[03, 06, 14]
Cognates: Nahua: mozotl;
Language contact: Nah > Spanish

***Bidens* sp. (Asteraceae)**

Spanish names:
Indigenous names: Ya'axk'an-ak'[09]; Sahun, Saksahun[09]; Job'on chokoj[11]; Guièe-tǐ[23]
Used by (3*): Yucatecan Maya[09, 11]; Zapotec[23]
Used for (7#): Digestive (1)[09]; Cardiovascular (1)[23]; Psychological (2)[23, 23]; Respiratory (1)[09]; General and Unspecified (2)[09, 11]
Cognates:
Language contact:

***Bidens squarrosa* Kunth (Asteraceae)**

Spanish names: Flor de muerto, , flor del alma
Indigenous names: Anima jäyä [01]; Kelem ts'aah[07]; Tuix momol[20]
Used by (3*): Zoque[01]; Huastec[07]; Western Maya[20]
Used for (5#): Digestive (2)[07, 20]; Skin (2)[01, 07]; General and Unspecified (1)[07]
Cognates:
Language contact:

***Bignonia binata* Thunb. (Bignoniaceae)**

Spanish names:
Indigenous names: Bichim ts'aah, pelat koox[07]; Puj Q'ehen[14]
Used by (2*): Huastec[07]; Quichean Maya[14]
Used for (5#): Digestive (1)[07]; Musculoskeletal (2)[07, 14]; Neurological (1)[14]; Urological (1)[07]
Cognates:
Language contact:

[01-28]refer to the study codes in Table 4.1. *Total number of studies citing this taxon #Total number of use-records

***Bignonia diversifolia* Kunth (Bignoniaceae)**

Spanish names:
Indigenous names: Ek'k'ixil, Soski-ak'[09]
Used by (1*): Yucatecan Maya[09]
Used for (1#): Skin (1)[09]
Cognates:
Language contact:

***Bignonia potosina* (K.Schum. & Loes.) L.G.Lohmann (Bignoniaceae)**

Spanish names: Bejuco de pimienta
Indigenous names: Moke poj[02]; Punath, punal, ataa ch'aah[07]; Cajlmecatl[24]
Used by (3*): Zoque[02]; Huastec[07]; Nahua[24]
Used for (4#): Digestive (1)[02]; Neurological (1)[07]; Pregnancy (1)[24]; General and Unspecified (1)[07]
Cognates:
Language contact:

***Biophytum dendroides* (Kunth) DC. (Oxalidaceae)**

Spanish names: Palmita
Indigenous names: Coco ay, Chuch suyat[03]; Tek xuuxy ujts[04]; Tlalhuaxe[26]
Used by (3*): Zoque[03]; Mixe[04]; Nahua[26]
Used for (6#): Digestive (2)[03, 26]; Psychological (2)[03, 04]; Pregnancy (1)[03]; General and Unspecified (1)[03]
Cognates:
Language contact: Mixe <> Nah

***Bixa orellana* L. (Bixaceae)**

Spanish names: Achiote
Indigenous names: Tzine ay/Zini[01]; Pukä[02]; Cuy puk[03]; Axut[04]; Ki'wi', K'uxub[09]; KuŠu[10]; Q'axob[13]; Xayaw[14]; Xaq xa yaw[16]; Jo'ox[18]; Kiwi'[19]; Mbeye'e[21]
Used by (14*): Zoque[01, 02, 03]; Mixe[04]; Totonac[06]; Yucatecan Maya[09, 10]; Quichean Maya[12, 13, 14, 16]; Western Maya[18, 19]; Zapotec[21]
Used for (45#): Digestive (6)[01, 09, 14, 18, 19, 21]; Ear (1)[01]; Cardiovascular (2)[01, 14]; Musculoskeletal (3)[01, 18, 21]; Neurological (2)[01, 03]; Psychological (1)[16]; Respiratory (2)[01, 10]; Skin (10)[01, 02, 03, 04, 09, 10, 12, 13, 18, 21]; Endocrine (1)[14]; Urological (2)[01, 03]; Pregnancy (2)[02, 03]; Female genital (3)[03, 14, 21]; Male genital (1)[01]; General and Unspecified (9)[01, 02, 03, 04, 06, 09, 10, 18, 21]
Cognates: Zoq: puk; CoreM: kCxCb, kiwi; Yuca: kuxu; Quich: xayaw;
Language contact:

***Blakea cuneata* Standl. (Melastomataceae)**

Spanish names:
Indigenous names: Oxlaju' ch'ajom[17]
Used by (1*): Quichean Maya[17]
Used for (1#): nd[17]
Cognates:
Language contact:

***Blakea* sp. (Melastomataceae)**

Spanish names:
Indigenous names: Yot' eq[14]
Used by (1*): Quichean Maya[14]
Used for (1#): Digestive (1)[14]
Cognates:
Language contact:

**_Blechnum gracile_ Kaulf. (Blechnaceae)**

Spanish names:
Indigenous names: Tsabats chimal[03]
Used by (1*): Zoque[03]
Used for (2#): Pregnancy (1)[03]; General and Unspecified (1)[03]
Cognates:
Language contact:

**_Blechum linnaei_ Nees (Acanthaceae)**

Spanish names:
Indigenous names: Ak'abxiw[09]
Used by (1*): Yucatecan Maya[09]
Used for (2#): Digestive (1)[09]; General and Unspecified (1)[09]
Cognates:
Language contact:

**_Blechum pyramidatum_ (Lam.) Urb. (Acanthaceae)**

Spanish names: Cancerillo; Hueso de rana
Indigenous names: Yexu' tsaakuy elul, xonnol palats verde, san miguel wits, itsaan an tsabal muuw, bohol ch'ohool[07]
Used by (4*): Zoque[03]; Huastec[07]; Quichean Maya[17]; Western Maya[18]
Used for (6#): Digestive (1)[07]; Skin (2)[07, 18]; General and Unspecified (2)[03, 07]; nd[17]
Cognates:
Language contact:

**_Blepharidium guatemalense_ Standl. (Rubiaceae)**

Spanish names: Girayol
Indigenous names: Yaxté[14]
Used by (1*): Quichean Maya[14]
Used for (2#): Skin (1)[14]; General and Unspecified (1)[14]
Cognates:
Language contact:

**_Blepharocalyx salicifolius_ (Kunth) O.Berg (Myrtaceae)**

Spanish names: Anacahuite
Indigenous names: Hkshi vit[08]
Used by (1*): Huastec[08]
Used for (2#): Skin (1)[08]; General and Unspecified (1)[08]
Cognates:
Language contact:

**_Bocconia arborea_ S.Watson (Papaveraceae)**

Spanish names: Arnica; Gordolobo (grande); Llorasangre
Indigenous names: Racan kaminaq'[12]; Xilcuahuitl[26]
Used by (3*): Quichean Maya[12]; Zapotec[23]; Nahua[26]
Used for (8#): Digestive (1)[26]; Musculoskeletal (1)[23]; Neurological (1)[12]; Psychological (1)[12]; Respiratory (1)[26]; Skin (2)[23, 26]; Female genital (1)[26]
Cognates:
Language contact:

**_Bocconia frutescens_ L. (Papaveraceae)**

Spanish names: Gordolobo; Hoja de toro; Llora sangre
Indigenous names: Susyui dane[01]; Ak¢i¢ileqs tuwan[05]; Tsîxte'[07]; Ts'ixte'[08]
Used by (6*): Zoque[01, 03]; Totonac[05]; Huastec[07, 08]; Nahua[25]
Used for (14#): Cardiovascular (1)[08]; Musculoskeletal (1)[08]; Neurological (2)[07, 08]; Respiratory (3)[03, 05, 08]; Skin (4)[01, 03, 05, 25]; Endocrine (1)[08]; Urological (1)[08]; General and Unspecified (1)[08]
Cognates: Huas: tsixte;
Language contact: Hua > Tot

**_Boerhavia diffusa_ L. (Nyctaginaceae)**

Spanish names: Hoja de azar embra
Indigenous names: Tza'a tzoy[01]
Used by (1*): Zoque[01]
Used for (2#): Skin (1)[01]; General and Unspecified (1)[01]
Cognates:
Language contact:

**_Boerhavia erecta_ L. (Nyctaginaceae)**

Spanish names: Yerba de arretes
Indigenous names:
Used by (1*): Zapotec[22]
Used for (1#): Digestive (1)[22]
Cognates:
Language contact:

**_Boerhavia scandens_ L. (Nyctaginaceae)**

Spanish names:
Indigenous names: Gurakw[22]
Used by (1*): Zapotec[22]
Used for (2#): Digestive (1)[22]; General and Unspecified (1)[22]
Cognates:
Language contact:

**_Boerhavia_ sp. (Nyctaginaceae)**

Spanish names: Vergonzosa, pega
Indigenous names: Chakle'[09]
Used by (2*): Yucatecan Maya[09]; Zapotec[21]
Used for (3#): Respiratory (1)[21]; Skin (2)[09, 21]
Cognates:
Language contact:

**_Bolbitis pergamentacea_ (Maxon) Ching (Dryopteridaceae)**

Spanish names:
Indigenous names: Quax kay pim[16]
Used by (2*): Quichean Maya[16, 17]
Used for (2#): Psychological (1)[16]; nd[17]
Cognates:
Language contact:

**_Bomarea acutifolia_ (Link & Otto) Herb. (Alstroemeriaceae)**

Spanish names: Lirio de Montaña
Indigenous names: Aq'om Q'enqoj Aq'om, Rawen K'echelaj Aq'om[12]; Lo'loin[20]
Used by (2*): Quichean Maya[12]; Western Maya[20]
Used for (3#): Digestive (2)[12, 20]; Urological (1)[12]
Cognates:
Language contact:

[01-28]refer to the study codes in Table 4.1.    *Total number of studies citing this taxon    #Total number of use-records

***Bomarea edulis* (Tussac) Herb. (Alstroemeriaceae)**

Spanish names: Campana
Indigenous names: San migeel wits, okoob pichich[07]; Tzujtzuj Q'ehen[14]; Lo'loin[20]
Used by (4*): Zoque[03]; Huastec[07]; Quichean Maya[14]; Western Maya[20]
Used for (9#): Digestive (3)[07, 14, 20]; Psychological (1)[07]; Skin (2)[07, 14]; Endocrine (1)[07]; Female genital (1)[03]; General and Unspecified (1)[14]
Cognates:
Language contact:

***Bonellia macrocarpa* (Cav.) B.Ståhl & Källersjö (Primulaceae)**

Spanish names:
Indigenous names: Korpus wits, k'iith wich[07]; Sink'inche'[09]
Used by (2*): Huastec[07]; Yucatecan Maya[09]
Used for (3#): Neurological (1)[07]; Respiratory (1)[07]; General and Unspecified (1)[09]
Cognates:
Language contact:

***Borago officinalis* L. (Boraginaceae)**

Spanish names: Borraja
Indigenous names:
Used by (6*): Zoque[01]; Quichean Maya[12]; Zapotec[21, 23]; Nahua[25, 27]
Used for (12#): Cardiovascular (1)[12]; Neurological (1)[12]; Respiratory (6)[01, 12, 21, 23, 25, 27]; Skin (1)[23]; Pregnancy (1)[12]; General and Unspecified (2)[12, 23]
Cognates:
Language contact:

***Bougainvillea buttiana* Holttum & Standl. (Nyctaginaceae)**

Spanish names: Bugambilia
Indigenous names:
Used by (1*): Western Maya[19]
Used for (1#): Respiratory (1)[19]
Cognates:
Language contact:

***Bougainvillea glabra* Choisy (Nyctaginaceae)**

Spanish names: Bugambilia
Indigenous names: Apit jäyä[01]; Shpupukuishonat[08]
Used by (10*): Zoque[01, 02]; Mixe[04]; Huastec[08]; Quichean Maya[12, 13]; Western Maya[18]; Zapotec[21]; Nahua[25, 26]
Used for (22#): Digestive (2)[02, 25]; Cardiovascular (1)[25]; Musculoskeletal (1)[08]; Psychological (1)[01]; Respiratory (10)[01, 02, 04, 08, 12, 13, 18, 21, 25, 26]; Skin (1)[01]; Endocrine (1)[08]; Urological (1)[08]; General and Unspecified (4)[08, 12, 21, 25]
Cognates:
Language contact:

***Bougainvillea* sp. (Nyctaginaceae)**

Spanish names: Bugambilia
Indigenous names:
Used by (1*): Zoque[03]
Used for (1#): Respiratory (1)[03]
Cognates:
Language contact:

***Bougainvillea spectabilis* Willd. (Nyctaginaceae)**

Spanish names: Bugambilia; Buganbilia
Indigenous names: Buganbiya[07]; Yàg-bùgàmbîl[23]
Used by (4*): Huastec[07]; Zapotec[23]; Nahua[27, 28]
Used for (6#): Psychological (1)[27]; Respiratory (2)[27, 28]; Pregnancy (1)[07]; General and Unspecified (2)[23, 28]
Cognates:
Language contact:

***Bourreria mollis* Standl. (Boraginaceae)**

Spanish names: Roble; Tres bono
Indigenous names: Oxib' boon'[14]
Used by (2*): Yucatecan Maya[11]; Quichean Maya[14]
Used for (3#): Psychological (1)[11]; Endocrine (1)[14]; General and Unspecified (1)[11]
Cognates:
Language contact:

***Bouvardia leiantha* Benth. (Rubiaceae)**

Spanish names: Pericón rojo
Indigenous names:
Used by (1*): Quichean Maya[12]
Used for (5#): Blood (1)[12]; Neurological (1)[12]; Skin (1)[12]; Endocrine (1)[12]; General and Unspecified (1)[12]
Cognates:
Language contact:

***Brachiaria mutica* (Forssk.) Stapf (Poaceae)**

Spanish names:
Indigenous names: Leh toom[07]
Used by (1*): Huastec[07]
Used for (1#): Skin (1)[07]
Cognates:
Language contact:

***Brachistus stramoniifolius* (Kunth) Miers (Solanaceae)**

Spanish names: Tomatillo
Indigenous names: Xcoya che'[12]
Used by (1*): Quichean Maya[12]
Used for (2#): Digestive (1)[12]; Endocrine (1)[12]
Cognates:
Language contact:

***Brahea dulcis* (Kunth) Mart. (Arecaceae)**

Spanish names:
Indigenous names: Yàg-zîn[23]
Used by (1*): Zapotec[23]
Used for (1#): General and Unspecified (1)[23]
Cognates:
Language contact:

***Brassica napus* L. (Brassicaceae)**

Spanish names: Napu
Indigenous names:
Used by (1*): Zoque[01]
Used for (1#): Skin (1)[01]
Cognates:
Language contact:

**_Brassica nigra_ (L.) K.Koch (Brassicaceae)**

Spanish names: Mostaza; Mostaza
Indigenous names: Mostas gihš[22]
Used by (3*): Zoque[02]; Western Maya[19]; Zapotec[22]
Used for (10#): Digestive (2)[02, 19]; Musculoskeletal (1)[22]; Neurological (2)[19, 22]; Endocrine (1)[02]; Female genital (1)[02]; General and Unspecified (3)[02, 19, 22]
Cognates:
Language contact:

**_Brassica oleracea_ L. (Brassicaceae)**

Spanish names: Repollo
Indigenous names: Pixla'q[12]; Ripoy[13]
Used by (3*): Zoque[01]; Quichean Maya[12, 13]
Used for (4#): Skin (2)[01, 13]; Urological (1)[12]; General and Unspecified (1)[12]
Cognates:
Language contact:

**_Brassica rapa_ L. (Brassicaceae)**

Spanish names: Mostaza
Indigenous names: Napx[12]
Used by (2*): Yucatecan Maya[11]; Quichean Maya[12]
Used for (7#): Digestive (1)[12]; Musculoskeletal (1)[11]; Neurological (1)[11]; Skin (2)[11, 12]; General and Unspecified (2)[11, 12]
Cognates:
Language contact:

**_Brassica_ sp. (Brassicaceae)**

Spanish names: Mostaza
Indigenous names: Mòxtâz[23]
Used by (2*): Zapotec[21, 23]
Used for (6#): Digestive (2)[21, 23]; Respiratory (1)[23]; Pregnancy (1)[21]; General and Unspecified (2)[21, 23]
Cognates:
Language contact:

**_Bravaisia berlandieriana_ (Nees) T.F.Daniel (Acanthaceae)**

Spanish names:
Indigenous names: Tz'ulub' Che'[14]
Used by (1*): Quichean Maya[14]
Used for (2#): Neurological (1)[14]; Social problems (1)[14]
Cognates:
Language contact:

**_Brickellia diffusa_ (Vahl) A.Gray (Asteraceae)**

Spanish names:
Indigenous names: Komino ts'ohool, alte yexu' ts'ohool[07]
Used by (1*): Huastec[07]
Used for (1#): General and Unspecified (1)[07]
Cognates:
Language contact:

**_Brickellia nutanticeps_ S.F.Blake (Asteraceae)**

Spanish names: Hojita larga
Indigenous names:
Used by (1*): Zoque[01]
Used for (1#): Digestive (1)[01]
Cognates:
Language contact:

***Brickellia paniculata* (Mill.) B.L.Rob. (Asteraceae)**

Spanish names:
Indigenous names: Ch'ail pox[20]
Used by (1*): Western Maya[20]
Used for (1#): Digestive (1)[20]
Cognates:
Language contact:

***Brickellia* sp. (Asteraceae)**

Spanish names:
Indigenous names: Ueji ay[03]
Used by (1*): Zoque[03]
Used for (1#): Psychological (1)[03]
Cognates:
Language contact:

***Brickellia veronicaefolia* (Kunth) A.Gray (Asteraceae)**

Spanish names:
Indigenous names: Guìzh-yòob-lây[23]
Used by (1*): Zapotec[23]
Used for (1#): Psychological (1)[23]
Cognates:
Language contact:

***Bromelia alsodes* H.St.John (Bromeliaceae)**

Spanish names:
Indigenous names: Patsikt[04]; Bathuch, bathuts[07]
Used by (2*): Mixe[04]; Huastec[07]
Used for (3#): Musculoskeletal (2)[04, 07]; Skin (1)[07]
Cognates:
Language contact: Mixe <> Hua

***Bromelia karatas* L. (Bromeliaceae)**

Spanish names: Muta, piñuela; Pinuela
Indigenous names: Chicuix[03]; Ch'om, Ch'am[09]; Tzetze'[19]
Used by (3*): Zoque[03]; Yucatecan Maya[09]; Western Maya[19]
Used for (4#): Ear (1)[19]; Skin (1)[03]; Pregnancy (1)[09]; Female genital (1)[09]
Cognates:
Language contact:

***Brongniartia papyracea* Dorado & D.M. Arias (Fabaceae)**

Spanish names:
Indigenous names: Guìzh-càpâr[23]
Used by (1*): Zapotec[23]
Used for (1#): General and Unspecified (1)[23]
Cognates:
Language contact:

***Bronwenia cornifolia* (Kunth) W.R.Anderson & C.Davis (Malpighiaceae)**

Spanish names: Hierba florentina
Indigenous names:
Used by (1*): Zoque[01]
Used for (1#): Musculoskeletal (1)[01]
Cognates:
Language contact:

**_Brosimum alicastrum_ Sw. (Moraceae)**

Spanish names: Ramón
Indigenous names: Ohox, ojox, ojx[07]; Ox[09]; Viguiiru'u[21]
Used by (3*): Huastec[07]; Yucatecan Maya[09]; Zapotec[21]
Used for (3#): Neurological (1)[07]; Respiratory (1)[09]; General and Unspecified (1)[21]
Cognates: Mayan: ox;
Language contact:

**_Brugmansia_ × _candida_ Pers. (Solanaceae)**

Spanish names: Flor de campana, floripondio
Indigenous names: Lokitsy/Kampentu/ kampantu jäya[01]; Kalapuš[05]; Kampaana wits[07]; Sach campana, asak[13]; Tzinläwe[18]; Guièe-pûnt, yàg-guièe-pûnt[23]
Used by (9*): Zoque[01]; Totonac[05]; Huastec[07]; Yucatecan Maya[10]; Quichean Maya[13]; Western Maya[18]; Zapotec[21, 23]; Nahua[25]
Used for (22#): Digestive (2)[01, 13]; Ear (1)[10]; Cardiovascular (2)[01, 21]; Musculoskeletal (2)[01, 18]; Neurological (3)[01, 13, 18]; Psychological (2)[05, 13]; Respiratory (2)[01, 25]; Skin (5)[01, 05, 07, 21, 25]; General and Unspecified (3)[01, 07, 23]
Cognates:
Language contact:

**_Brugmansia arborea_ (L.) Steud. (Solanaceae)**

Spanish names: Floripondio
Indigenous names:
Used by (1*): Nahua[26]
Used for (2#): Musculoskeletal (1)[26]; Skin (1)[26]
Cognates:
Language contact:

**_Brugmansia suaveolens_ (Humb. & Bonpl. ex Willd) Bercht. & J.Presl. (Solanaceae)**

Spanish names: Flor de campana; Hoja de campana, rosa
Indigenous names: Lokitsy/Kampentu/ kampantu jäya[01]; Nunak mooya[03]
Used by (3*): Zoque[01, 03]; Zapotec[21]
Used for (9#): Digestive (1)[03]; Cardiovascular (1)[21]; Musculoskeletal (2)[03, 21]; Neurological (1)[01]; Respiratory (1)[21]; Skin (1)[21]; General and Unspecified (2)[01, 21]
Cognates:
Language contact:

**_Bryophyllum delagoense_ (Eckl. & Zeyh.) Druce (Crassulaceae)**

Spanish names: Palito pinto
Indigenous names: Tzikin kuy[01]
Used by (1*): Zoque[01]
Used for (1#): Skin (1)[01]
Cognates:
Language contact:

**_Bryophyllum fedtschenkoi_ (Raym.-Hamet & H.Perrier) Lauz.-March (Crassulaceae)**

Spanish names: Beladona/sanalotodo
Indigenous names: Siwe tane[01]
Used by (1*): Zoque[01]
Used for (3#): Digestive (1)[01]; Musculoskeletal (1)[01]; Skin (1)[01]
Cognates:
Language contact:

[01-28]refer to the study codes in Table 4.1. *Total number of studies citing this taxon #Total number of use-records

***Bryophyllum pinnatum* (Lam.) Oken (Crassulaceae)**

Spanish names: Belladonna, sanalotodo, hoja de aire, maravilla
Indigenous names: Lakanyo/malva tane/ tini rane)/tok tzäksy/siwe tane/ tukun jäyä[01]; Tøts ujts[04]; Tkuya tuwan[05]; Tolow xekel, binage ts'ohool, lam ts'ohool, pahab eheenchx, pak'ak ts'ohool, xutsun buuru, pahab at'ax inik, pak'ak' xuts, toltha' ch'ohool[07]; Guish marauii, lu'ujtzanguana'aj[21]; Guìzh-cùchâr, rlăal-x-pǽæd-á, guièe-yùzh[23]; Campanaxochitl[26]
Used by (15*): Zoque[01, 02, 03]; Mixe[04]; Totonac[05]; Huastec[07]; Yucatecan Maya[09, 10, 11]; Quichean Maya[13, 16]; Western Maya[18]; Zapotec[21, 23]; Nahua[26]
Used for (50#): Digestive (5)[01, 02, 03, 07, 18]; Eye (2)[03, 05]; Ear (1)[03]; Cardiovascular (1)[07]; Musculoskeletal (7)[01, 02, 03, 10, 11, 18, 21]; Neurological (9)[01, 02, 03, 04, 05, 07, 16, 18, 26]; Psychological (1)[07]; Respiratory (3)[01, 03, 18]; Skin (11)[01, 02, 03, 07, 09, 10, 11, 13, 21, 23, 26]; Endocrine (1)[07]; Urological (1)[21]; General and Unspecified (7)[01, 03, 04, 07, 13, 21, 26]
Cognates:
Language contact:

***Buddleja americana* L. (Scrophulariaceae)**

Spanish names: Salvia santa, tepozan
Indigenous names: Xiapun ay[03]; Pajuik[04]; Pulik elte', thak te' hunta a anaamte'[07]; Salv' sant'[13]; Saq rix pim[16]; T'oxpe'[19]; Gush blaad[21]; Zayoliscan[28]
Used by (8*): Zoque[03]; Mixe[04]; Huastec[07]; Quichean Maya[13, 16]; Western Maya[19]; Zapotec[21]; Nahua[28]
Used for (28#): Digestive (3)[04, 19, 28]; Musculoskeletal (1)[19]; Neurological (2)[16, 19]; Psychological (2)[16, 19]; Respiratory (3)[03, 19, 21]; Skin (6)[03, 03, 07, 13, 21, 28]; Pregnancy (3)[13, 19, 21]; Female genital (1)[19]; General and Unspecified (7)[03, 07, 13, 19, 19, 21, 28]
Cognates:
Language contact:

***Buddleja cordata* Kunth (Scrophulariaceae)**

Spanish names: Huacalillo
Indigenous names: Nesahuashihuitl[25]; Tepozan[26]
Used by (2*): Nahua[25, 26]
Used for (5#): Digestive (1)[25]; Neurological (1)[26]; Skin (2)[25, 26]; Pregnancy (1)[26]
Cognates:
Language contact:

***Buddleja sessiliflora* Kunth (Scrophulariaceae)**

Spanish names: Lengua de vaca
Indigenous names: Bala giwi[22]; Blàg-wì, lùdz-ngŏn, yàg-chànêcw[23]
Used by (2*): Zapotec[22, 23]
Used for (5#): Digestive (2)[22, 23]; Musculoskeletal (1)[23]; General and Unspecified (2)[22, 23]
Cognates: Zapo: b(a)lag(i)wi;
Language contact:

***Buddleja* sp. (Scrophulariaceae)**

Spanish names:
Indigenous names: Sak yok te'[20]
Used by (1*): Western Maya[20]
Used for (1#): Digestive (1)[20]
Cognates:
Language contact:

***Bunchosia swartziana* Griseb. (Malpighiaceae)**

Spanish names:
Indigenous names: Sipche'[09]
Used by (1*): Yucatecan Maya[09]
Used for (2#): Digestive (1)[09]; Neurological (1)[09]
Cognates:
Language contact:

[01-28]refer to the study codes in Table 4.1. *Total number of studies citing this taxon #Total number of use-records

***Bursera arida* (Rose) Standl. (Burseraceae)**

Spanish names: Aceitillo
Indigenous names:
Used by (1*): Nahua[27]
Used for (2#): Digestive (1)[27]; Skin (1)[27]
Cognates:
Language contact:

***Bursera grandifolia* (Schltdl.) Engl. (Burseraceae)**

Spanish names: Palo mulato
Indigenous names: Yalajguettu'u[21]
Used by (1*): Zapotec[21]
Used for (4#): Digestive (1)[21]; Skin (1)[21]; Female genital (1)[21]; General and Unspecified (1)[21]
Cognates:
Language contact:

***Bursera graveolens* (Kunth) Triana & Planch. (Burseraceae)**

Spanish names: Azafrán; Sasafrás
Indigenous names: Putoki[03]; Kaxiy tsakah, tsamnek tsakah[07]
Used by (3*): Zoque[03]; Huastec[07]; Western Maya[18]
Used for (10#): Digestive (1)[03]; Cardiovascular (1)[03]; Musculoskeletal (2)[03, 18]; Neurological (1)[07]; Psychological (1)[07]; Skin (1)[03]; General and Unspecified (3)[03, 07, 18]
Cognates:
Language contact:

***Bursera ovalifolia* (Schltdl.) Engl. (Burseraceae)**

Spanish names: Mulato (rojo); Mulato rojo
Indigenous names: Tzaps kuy [01]; Tzäk/tzäksy[02]
Used by (2*): Zoque[01, 02]
Used for (10#): Digestive (2)[01, 02]; Skin (1)[02]; Endocrine (2)[01, 02]; Urological (1)[02]; Pregnancy (1)[02]; Female genital (1)[01]; Male genital (1)[01]; General and Unspecified (1)[02]
Cognates: Zoq: tza;
Language contact:

***Bursera simaruba* (L.) Sarg. (Burseraceae)**

Spanish names: Palo mulato, chaca, jiote
Indigenous names: Ts¥k[03]; Tsøk[04]; Tusun, chaca, tusuni[05]; Tsaka, tsakah, chaka[07]; Tsaka[08]; Chakah[09]; Čacah[10]; Chi'kaj (blanco/ morado)[11]; Sali Che'[12]; Che' Winq, Kaq'aj[14]; Kak'hajl che'[17]; Chäk' zulte'[18]; Chakajr[19]
Used by (15*): Zoque[03]; Mixe[04]; Totonac[05]; Huastec[07, 08]; Yucatecan Maya[09, 10, 11]; Quichean Maya[12, 14, 17]; Western Maya[18, 19]; Nahua[25, 26]
Used for (50#): Digestive (5)[03, 05, 07, 14, 26]; Cardiovascular (1)[14]; Musculoskeletal (5)[03, 07, 08, 12, 14]; Neurological (6)[05, 07, 08, 10, 14, 25]; Psychological (1)[07]; Respiratory (4)[07, 07, 12, 25]; Skin (7)[03, 05, 07, 08, 10, 14, 26]; Endocrine (1)[14]; Urological (5)[05, 12, 14, 18, 19]; Female genital (1)[03]; General and Unspecified (13)[03, 04, 05, 07, 08, 09, 10, 11, 12, 18, 19, 25, 26]; nd[17]
Cognates: MZ: tsäk; Mayan: tsaka/chaka; CoreM: chak/kak; Yuca: chakah; Quich: kak'a; WesM: chak;
Language contact: MZ > Tot and Maya

***Bursera* sp. (Burseraceae)**

Spanish names: Copal; Copalero; Hoja de sumerio; Mulato; Mulato negro
Indigenous names: Äkyui/uwäk kuy/tzatz kuy/tzapas kuy [01]; Bi Tzäksy/yäk tzäksy[02]; Ii'guiaj[21]; Guish yaguiyaj'[21]; Yahl[22]; Yàg-yàal, yàg-guín-quiè[23]
Used by (5*): Zoque[01, 02]; Zapotec[21, 22, 23]
Used for (19#): Digestive (2)[01, 21]; Musculoskeletal (3)[01, 21, 23]; Neurological (1)[22]; Respiratory (1)[21]; Skin (2)[01, 21]; Endocrine (1)[01]; Pregnancy (1)[21]; Female genital (1)[01]; Male genital (1)[01]; General and Unspecified (6)[01, 02, 21, 21, 22, 23]
Cognates: Zoq: tza; Zapo: yaal;
Language contact:

[01-28]refer to the study codes in Table 4.1. *Total number of studies citing this taxon #Total number of use-records

***Buxus sempervirens* L. (Buxaceae)**

Spanish names: Mirto Oloroso, Boj, Mirto, Lemonaria
Indigenous names:
Used by (1*): Quichean Maya[12]
Used for (3[#]): Neurological (1)[12]; Respiratory (1)[12]; General and Unspecified (1)[12]
Cognates:
Language contact:

***Byrsonima crassifolia* (L.) Kunth (Malpighiaceae)**

Spanish names: Nanche, nance
Indigenous names: Nansin[01]; Nansin/ tupsy täm[02]; Nanchiñ[03]; Tax[04]; Chi'[09]; Či[10]; Tapal[13]; Chi'[14]; Chi'[18]; Chi'[19]; Nantzin, lantzin, nantz chi'[20]; Mbatsi[21]; Bälwi[22]; Yàg-ngùd-guèy-pcàal, yàg-ngùd-pcàal, guìzh-pcàal, ngùd-guèy-nguěts[23]
Used by (17*): Zoque[01, 02, 03]; Mixe[04]; Totonac[06]; Yucatecan Maya[09, 10, 11]; Quichean Maya[12, 13, 14]; Western Maya[18, 19, 20]; Zapotec[21, 22, 23]
Used for (50[#]): Digestive (13)[01, 02, 03, 04, 06, 09, 10, 13, 18, 19, 20, 21, 22]; Eye (1)[02]; Cardiovascular (1)[02]; Musculoskeletal (1)[01]; Neurological (3)[01, 02, 19]; Psychological (1)[03]; Respiratory (4)[02, 12, 20, 21]; Skin (8)[01, 02, 03, 04, 09, 11, 20, 21]; Endocrine (2)[02, 14]; Urological (3)[01, 02, 03]; Pregnancy (1)[02]; Female genital (5)[01, 02, 03, 04, 21]; General and Unspecified (7)[02, 09, 12, 13, 20, 21, 23]
Cognates: Zoq: nansin; CoreM: chi; Yuca: chi; WesM: chi; Zapo: batsi/bälwi/pcaal;
Language contact: Nah <> Zoq <> CoreM; Zap > K'iche'

***Byttneria aculeata* Jacq. (Malvaceae)**

Spanish names: Uña de gato (blanco)/cola de iguana/cola de garrobo
Indigenous names: Misyu apits/ tzätzän tuts/ nu'tzi tutz[01]; Bolool othow, bolool uthu'[07]
Used by (2*): Zoque[01]; Huastec[07]
Used for (8[#]): Digestive (2)[01, 07]; Eye (1)[01]; Cardiovascular (1)[01]; Musculoskeletal (1)[01]; Skin (1)[01]; Pregnancy (1)[07]; General and Unspecified (1)[07]
Cognates:
Language contact:

***Cabomba haynesii* Wiersema (Cabombaceae)**

Spanish names:
Indigenous names: Weew bexe', hitil[07]
Used by (1*): Huastec[07]
Used for (2[#]): Neurological (1)[07]; General and Unspecified (1)[07]
Cognates:
Language contact:

***Caesalpinia bonduc* (L.) Roxb. (Fabaceae)**

Spanish names:
Indigenous names: Eelaa'[07]
Used by (1*): Huastec[07]
Used for (1[#]): Digestive (1)[07]
Cognates:
Language contact:

***Caesalpinia gaumeri* Greenm. (Fabaceae)**

Spanish names:
Indigenous names: Kitamche'[09]
Used by (1*): Yucatecan Maya[09]
Used for (1[#]): General and Unspecified (1)[09]
Cognates:
Language contact:

***Caesalpinia pulcherrima* (L.) Sw. (Fabaceae)**

Spanish names: Hoja de maravilla; Ortensia; Sinquin colorado
Indigenous names: Tsas mooya[03]; San husee te', san husee wits[07]; Sin k'in[11]
Used by (5*): Zoque[03]; Huastec[07]; Yucatecan Maya[10, 11]; Zapotec[21]
Used for (10#): Digestive (1)[21]; Musculoskeletal (2)[03, 21]; Neurological (1)[21]; Psychological (1)[07]; Respiratory (1)[03]; Skin (1)[03]; Female genital (1)[03]; General and Unspecified (2)[10, 11]
Cognates: Mayan: sin/san;
Language contact:

***Caesalpinia yucatanensis* Greenm. (Fabaceae)**

Spanish names:
Indigenous names: So'sol Q'ehen, Kanteel Maus[14]
Used by (1*): Quichean Maya[14]
Used for (3#): Neurological (1)[14]; Psychological (1)[14]; General and Unspecified (1)[14]
Cognates:
Language contact:

***Caladium bicolor* (Aiton) Vent. (Araceae)**

Spanish names:
Indigenous names: Tsabats txikx pixi[03]
Used by (1*): Zoque[03]
Used for (1#): Skin (1)[03]
Cognates:
Language contact:

***Calanthe calanthoides* (A.Rich. & Galeotti) Hamer & Garay (Orchidaceae)**

Spanish names: Cebollin
Indigenous names: Cebollin Aq'om[12]
Used by (1*): Quichean Maya[12]
Used for (4#): Digestive (1)[12]; Skin (1)[12]; Urological (1)[12]; General and Unspecified (1)[12]
Cognates:
Language contact:

***Calathea lutea (*Aubl.) E.Mey. ex Schult. (Marantaceae)**

Spanish names: Hoja blanca
Indigenous names: Pop' ay/popo ay[01]; Pob ay[03]; Yoco to'[18]
Used by (4*): Zoque[01, 03]; Western Maya[18]; Zapotec[21]
Used for (10#): Digestive (1)[01]; Eye (1)[03]; Skin (2)[01, 21]; Pregnancy (3)[01, 18, 21]; Female genital (2)[03, 18]; General and Unspecified (1)[21]
Cognates: Zoq: pop'ay;
Language contact:

***Calathea micans* (L.Mathieu) Körn. (Marantaceae)**

Spanish names:
Indigenous names: Moxpim[14]
Used by (1*): Quichean Maya[14]
Used for (1#): Pregnancy (1)[14]
Cognates:
Language contact:

[01-28]refer to the study codes in Table 4.1. *Total number of studies citing this taxon #Total number of use-records

***Calathea ovandensis* Matuda (Marantaceae)**

Spanish names: Hoja blanca
Indigenous names: Balagaquitzii[21]
Used by (1*): Zapotec[21]
Used for (3#): Skin (1)[21]; Pregnancy (1)[21]; General and Unspecified (1)[21]
Cognates:
Language contact:

***Calathea* sp. (Marantaceae)**

Spanish names:
Indigenous names: Kok' moch pim[14]; Koq mush[16]
Used by (2*): Quichean Maya[14, 16]
Used for (3#): Psychological (1)[16]; Pregnancy (1)[14]; Female genital (1)[14]
Cognates: Quich: koq moch;
Language contact:

***Calea integrifolia* (DC.) Hemsl. (Asteraceae)**

Spanish names:
Indigenous names: Axcaxihuitl[26]
Used by (1*): Nahua[26]
Used for (1#): Pregnancy (1)[26]
Cognates:
Language contact:

***Calea longipedicellata* B. L. Rob. et Greenm. (Asteraceae)**

Spanish names:
Indigenous names: Añi mutx ay[03]
Used by (1*): Zoque[03]
Used for (4#): Digestive (1)[03]; Musculoskeletal (1)[03]; Skin (1)[03]; General and Unspecified (1)[03]
Cognates:
Language contact:

***Calea* sp. (Asteraceae)**

Spanish names: Trabuco, hierba amarga
Indigenous names: Šuna tuwan[05]; Ich wou[14]; Ton ch'a te'[20]
Used by (3*): Totonac[05]; Quichean Maya[14]; Western Maya[20]
Used for (3#): Digestive (1)[20]; General and Unspecified (2)[05, 14]
Cognates:
Language contact:

***Calea urticifolia* (Mill.) DC. (Asteraceae)**

Spanish names: Prodigiosa, hierba del perro
Indigenous names: Tuwi ay/ takak tane[01]; Tsapt taam ujts[04]; Xka'xikin, Xikinkaax[09]; Sakil chi'xal wamal[20]
Used by (6*): Zoque[01, 03]; Mixe[04]; Yucatecan Maya[09]; Western Maya[20]; Zapotec[21]
Used for (12#): Digestive (5)[01, 04, 09, 20, 21]; Skin (3)[01, 03, 09]; Endocrine (1)[01]; Female genital (1)[03]; General and Unspecified (2)[01, 04]
Cognates: MZ: tam/k;
Language contact:

***Calea zacatechichi* Schltdl. (Asteraceae)**

Spanish names: Cacahuatón; Jaral; Mala hierba; Prodigiosa blanca
Indigenous names: Tam juñi[03]; Poop taam ujts[04]; Tsuleek' ethem, betse' ts'ohool, pun lat'em, xamxam, weew ethem, tsabaal tok'te', ehek witsiim[07]; Pux lat'em[08]; Škwam bižeh/ ghihb[22]; Ncuàan-zân[23]
Used by (7*): Zoque[03]; Mixe[04]; Huastec[07, 08]; Zapotec[21, 22, 23]
Used for (19#): Digestive (5)[03, 07, 08, 21, 22]; Cardiovascular (1)[22]; Musculoskeletal (2)[03, 22]; Psychological (1)[22]; Respiratory (2)[03, 07]; Skin (2)[03, 07]; Pregnancy (2)[22, 23]; Female genital (1)[03]; General and Unspecified (3)[03, 04, 07]
Cognates: MZ: tam; Huas: puC lat'em;
Language contact: MZ > Hua and Zap

***Calendula officinalis* L. (Asteraceae)**

Spanish names: Dormilón
Indigenous names: Guièe-dòrmìlôn, guièe-nguěts, guièe-gùts, guièe-nàad[23]
Used by (1*): Zapotec[23]
Used for (1#): Respiratory (1)[23]
Cognates:
Language contact:

***Calliandra houstoniana* (Mill.) Standl. (Fabaceae)**

Spanish names: Timbre
Indigenous names: Kine nhäpin [01]; Juix mooya[03]; Wiir'oot', xixit[07]; Ch'ich' ni'[20]
Used by (6*): Zoque[01, 03]; Huastec[07]; Quichean Maya[12]; Western Maya[20]; Nahua[26]
Used for (12#): Digestive (2)[12, 20]; Eye (1)[07]; Neurological (1)[07]; Respiratory (3)[01, 07, 20]; Skin (2)[07, 20]; Pregnancy (1)[26]; Female genital (1)[03]; General and Unspecified (1)[07]
Cognates:
Language contact: Highland Popoluca <> Hua <> Tzeltalan

***Calliandra juzepczukii* Standl. (Fabaceae)**

Spanish names: Palo sangre
Indigenous names: Kinä näpin [02]; Juix mooya[03]
Used by (2*): Zoque[02, 03]
Used for (5#): Digestive (1)[02]; Eye (1)[02]; Urological (1)[02]; Pregnancy (1)[03]; General and Unspecified (1)[03]
Cognates:
Language contact:

***Callicarpa acuminata* Kunth (Lamiaceae)**

Spanish names:
Indigenous names: Elte', thal te'[07]; Xpuk'in, Puk'im[09]
Used by (2*): Huastec[07]; Yucatecan Maya[09]
Used for (4#): Digestive (2)[07, 09]; Urological (1)[07]; Pregnancy (1)[07]
Cognates:
Language contact:

***Callisia fragrans* (Lindl.) Woodson (Commelinaceae)**

Spanish names:
Indigenous names: Uixpin[03]; Pulik utek', xutsun buuru[07]
Used by (2*): Zoque[03]; Huastec[07]
Used for (6#): Digestive (1)[03]; Ear (1)[07]; Cardiovascular (1)[07]; Psychological (1)[07]; Respiratory (1)[03]; Female genital (1)[03]
Cognates:
Language contact:

[01-28]refer to the study codes in Table 4.1. *Total number of studies citing this taxon #Total number of use-records

***Callisia* sp. (Commelinaceae)**

Spanish names:
Indigenous names: Utek' de bega[07]
Used by (1*): Huastec[07]
Used for (2#): Urological (1)[07]; General and Unspecified (1)[07]
Cognates:
Language contact:

***Callistemon citrinus* (Curtis) Skeels (Myrtaceae)**

Spanish names: Enebro
Indigenous names:
Used by (1*): Quichean Maya[12]
Used for (2#): Urological (1)[12]; General and Unspecified (1)[12]
Cognates:
Language contact:

***Calophyllum brasiliense* Cambess. (Clusiaceae)**

Spanish names: Lechemaria; Varín
Indigenous names: Ka' kuy/ kang kuy[02]; M¥anakcuy[03]
Used by (2*): Zoque[02, 03]
Used for (5#): Musculoskeletal (1)[02]; Neurological (1)[03]; Skin (2)[02, 03]; Female genital (1)[03]
Cognates:
Language contact:

***Calopogonium caeruleum* (Benth.) Sauvalle (Fabaceae)**

Spanish names: Zempantle
Indigenous names: Btuujtza[21]
Used by (1*): Zapotec[21]
Used for (3#): Neurological (1)[21]; Psychological (1)[21]; General and Unspecified (1)[21]
Cognates:
Language contact:

***Calyptocarpus vialis* Less. (Asteraceae)**

Spanish names: Huango/Hoja de azar
Indigenous names: Tza'a tzoy[01]
Used by (1*): Zoque[01]
Used for (1#): General and Unspecified (1)[01]
Cognates:
Language contact:

***Calyptocarpus wendlandii* Sch.Bip. (Asteraceae)**

Spanish names: Cabeza de zompopo, espina de gallina; Hierba Amarilla, cachito
Indigenous names: Xqa'ramaq'[12]
Used by (2*): Quichean Maya[12]; Western Maya[19]
Used for (4#): Digestive (1)[12]; Neurological (1)[12]; Psychological (1)[12]; Pregnancy (1)[19]
Cognates:
Language contact:

***Calyptranthes chytraculia* (L.) Sw. (Myrtaceae)**

Spanish names:
Indigenous names:
Used by (1*): Quichean Maya[17]
Used for (1#): nd[17]
Cognates:
Language contact:

[01-28]refer to the study codes in Table 4.1. *Total number of studies citing this taxon #Total number of use-records

***Camellia sinensis* (L.) Kuntze (Theaceae)**

Spanish names: Té Verde
Indigenous names:
Used by (1*): Quichean Maya[12]
Used for (3#): Blood (1)[12]; Cardiovascular (1)[12]; Musculoskeletal (1)[12]
Cognates:
Language contact:

***Campylocentrum micranthum* (Lindl.) Rolfe (Orchidaceae)**

Spanish names:
Indigenous names: Tz'ib'oyil Q'ehen[14]
Used by (1*): Quichean Maya[14]
Used for (1#): General and Unspecified (1)[14]
Cognates:
Language contact:

***Campyloneurum brevifolium* (Lodd. ex Link) Link (Polypodiaceae)**

Spanish names:
Indigenous names: Rix i xul[17]
Used by (1*): Quichean Maya[17]
Used for (1#): nd[17]
Cognates:
Language contact:

***Campyloneurum phyllitidis* (L.) C. Presl (Polypodiaceae)**

Spanish names: Helecho macho
Indigenous names: Cuy chimal[03]; wa'ub ik', wau, boo'wat, wayom ik, [07]
Used by (2*): Zoque[03]; Huastec[07]
Used for (5#): Digestive (1)[03]; Ear (1)[07]; Cardiovascular (1)[07]; Neurological (1)[07]; General and Unspecified (1)[03]
Cognates:
Language contact:

***Campyloneurum* sp. (Polypodiaceae)**

Spanish names: Cola de pavo
Indigenous names:
Used by (1*): Quichean Maya[15]
Used for (1#): Musculoskeletal (1)[15]
Cognates:
Language contact:

***Canavalia villosa* Benth. (Fabaceae)**

Spanish names: Sacramento
Indigenous names: Xagalamenta[03]; Koxol wits, koxoloow, pekeleexe wich[07]
Used by (2*): Zoque[03]; Huastec[07]
Used for (3#): Digestive (1)[03]; Skin (2)[03, 07]
Cognates:
Language contact:

**_Canna indica_ L. (Cannaceae)**

Spanish names: Papatla
Indigenous names: Nok ay[01]; ¥y¥ng ay[03]; Ciquichi, tzu'l kuat[05]; Chiquichi'[06]; K'uuwaap, tsak pik'o', tsabal wits, tabil[07]; Tsokon, maxan[13]
Used by (6*): Zoque[01, 03]; Totonac[05, 06]; Huastec[07]; Quichean Maya[13]
Used for (10#): Digestive (1)[07]; Ear (1)[03]; Musculoskeletal (1)[07]; Neurological (1)[07]; Skin (1)[01]; Pregnancy (3)[06, 07, 13]; General and Unspecified (2)[01, 05]
Cognates: Toto: ciquichi;
Language contact: Tot <> Hua <> Kiché

**_Cannabis sativa_ L. (Cannabaceae)**

Spanish names: Marihuana
Indigenous names: Jo'we ay' [02]; Ayuk juik[04]; Macutzín[28]
Used by (6*): Zoque[01, 02]; Mixe[04]; Zapotec[21, 22]; Nahua[28]
Used for (11#): Musculoskeletal (6)[01, 02, 04, 21, 22, 28]; Neurological (1)[02]; Urological (1)[01]; General and Unspecified (3)[01, 02, 21]
Cognates:
Language contact:

**_Capraria biflora_ L. (Scrophulariaceae)**

Spanish names: Esclaviosa, epazotillo
Indigenous names: Payau[04]; Sqeja lipajni[05]; Chokwil-xiw[09]; Pas mo Ši'iu[10]; Badxumij[21]
Used by (7*): Mixe[04]; Totonac[05]; Yucatecan Maya[09, 10, 11]; Western Maya[18]; Zapotec[21]
Used for (14#): Blood (1)[10]; Digestive (2)[09, 21]; Ear (1)[09]; Musculoskeletal (2)[05, 11]; Skin (3)[04, 18, 21]; Urological (2)[11, 18]; Female genital (1)[04]; General and Unspecified (2)[18, 21]
Cognates:
Language contact: Yuc <> Zap

**_Capraria mexicana_ Moric. ex Benth. (Scrophulariaceae)**

Spanish names: Epazotillo
Indigenous names: Kisawa ay[02]; Pulik ts'itsiimbe ts'ohool, weew bichim, tsamnek ts'ohool, [07]
Used by (2*): Zoque[02]; Huastec[07]
Used for (3#): Digestive (1)[07]; Neurological (1)[07]; Skin (1)[02]
Cognates:
Language contact:

**_Capraria saxifragifolia_ Schltdl. & Cham. (Scrophulariaceae)**

Spanish names:
Indigenous names: Akan t'ot, elul ts'ohool[07]
Used by (1*): Huastec[07]
Used for (2#): Cardiovascular (1)[07]; Female genital (1)[07]
Cognates:
Language contact:

**_Capsella bursa-pastoris_ (L.) Medik. (Brassicaceae)**

Spanish names: Bolsa de Pastor
Indigenous names:
Used by (1*): Quichean Maya[12]
Used for (4#): Skin (1)[12]; Urological (1)[12]; Female genital (1)[12]; General and Unspecified (1)[12]
Cognates:
Language contact:

[01-28]refer to the study codes in Table 4.1. *Total number of studies citing this taxon #Total number of use-records

***Capsicum annuum* L. (Solanaceae)**

Spanish names: Chile
Indigenous names: (Nama) niwi[01]; Niwi[02]; Niiy[04]; Pi'n[06]; Its, ich[07]; Iik[10]; Ik' Q'ehen, Xa'q Ik'[14]; Z'uts'ich[18]
Used by (11*): Zoque[01, 02]; Mixe[04]; Totonac[06]; Huastec[07]; Yucatecan Maya[10]; Quichean Maya[12, 14]; Western Maya[18]; Zapotec[21, 22]
Used for (23#): Digestive (1)[12]; Eye (3)[06, 07, 10]; Ear (1)[02]; Neurological (1)[18]; Psychological (2)[02, 04]; Skin (5)[01, 02, 06, 07, 12]; Endocrine (1)[12]; General and Unspecified (9)[01, 04, 06, 07, 12, 14, 18, 21, 22]
Cognates: MZ: ni(w)i; Zoq: niwi; Mayan: ich/ik;
Language contact: MZ <> Tot <> Maya

***Capsicum baccatum* L. (Solanaceae)**

Spanish names: Chile
Indigenous names: Balagaguina'a[21]
Used by (1*): Zapotec[21]
Used for (3#): Eye (1)[21]; Psychological (1)[21]; General and Unspecified (1)[21]
Cognates:
Language contact:

***Capsicum chinense* Jacq. (Solanaceae)**

Spanish names: Habanero
Indigenous names:
Used by (2*): Zoque[02]; Yucatecan Maya[09]
Used for (2#): Male genital (1)[02]; General and Unspecified (1)[09]
Cognates:
Language contact:

***Capsicum* sp. (Solanaceae)**

Spanish names: Chile; Chile
Indigenous names: Ñiwi[03]; Iq', ik'[13]; Ich[20]; Tlanxinal[25]
Used by (4*): Zoque[03]; Quichean Maya[13]; Western Maya[20]; Nahua[25]
Used for (7#): Digestive (1)[20]; Ear (1)[03]; Respiratory (1)[25]; Skin (1)[03]; Pregnancy (1)[13]; Male genital (1)[03]; General and Unspecified (1)[13]
Cognates: CoreM: ik / ich;
Language contact: Zoq <> Maya <> Nah

***Cardamine flaccida* Cham. & Schltdl. (Brassicaceae)**

Spanish names: Hoja de azar del rio
Indigenous names: Tza'a tzoy[01]; T'ik'om ichiich[07]
Used by (2*): Zoque[01]; Huastec[07]
Used for (2#): General and Unspecified (2)[01, 07]
Cognates:
Language contact:

***Cardiospermum halicacabum* L. (Sapindaceae)**

Spanish names: Hierba de pollo
Indigenous names:
Used by (1*): Nahua[28]
Used for (1#): Skin (1)[28]
Cognates:
Language contact:

[01-28] refer to the study codes in Table 4.1. *Total number of studies citing this taxon #Total number of use-records

***Carduus* sp. (Asteraceae)**

Spanish names:
Indigenous names: Ohob ilaal[07]
Used by (1*): Huastec[07]
Used for (2#): Musculoskeletal (1)[07]; Respiratory (1)[07]
Cognates:
Language contact:

***Carica papaya* L. (Caricaceae)**

Spanish names: Papaya
Indigenous names: Otzo[01]; Tuna[02]; Xala'cua[06]; Utsun[07]; Put[09]; ČiČ pu'ut[10]; Tu' Q'ehen [14]
Used by (15*): Zoque[01, 02, 03]; Mixe[04]; Totonac[06]; Huastec[07]; Yucatecan Maya[09, 10, 11]; Quichean Maya[12, 13, 14]; Western Maya[18]; Zapotec[21]; Nahua[26]
Used for (27#): Digestive (10)[01, 02, 03, 04, 06, 12, 13, 14, 18, 21]; Neurological (2)[02, 03]; Psychological (1)[02]; Respiratory (1)[06]; Skin (5)[03, 07, 09, 10, 11]; Endocrine (1)[18]; Urological (2)[02, 26]; Pregnancy (1)[07]; Female genital (1)[21]; General and Unspecified (3)[03, 12, 21]
Cognates: Yuca: put;
Language contact: Chiapas Zoq <> Hua

***Cascabela gaumeri* (Hemsl.) Lippold (Apocynaceae)**

Spanish names: Cojon de perro
Indigenous names: Akits[09]
Used by (1*): Yucatecan Maya[09]
Used for (1#): Skin (1)[09]
Cognates:
Language contact:

***Cascabela ovata* (Cav.) Lippold (Apocynaceae)**

Spanish names: Coyol de cochi; Flor de San Juan
Indigenous names: Yoyak puj [01]; Mbiigu' guexii[21]
Used by (3*): Zoque[01, 02]; Zapotec[21]
Used for (5#): Neurological (1)[02]; Respiratory (1)[21]; Skin (3)[01, 02, 21]
Cognates:
Language contact:

***Cascabela thevetia* (L.) Lippold (Apocynaceae)**

Spanish names: Chirrto
Indigenous names: Ts'een aanchuuch, antsuts[07]
Used by (2*): Huastec[07]; Yucatecan Maya[11]
Used for (4#): Cardiovascular (1)[07]; Neurological (1)[07]; Respiratory (1)[11]; Skin (1)[07]
Cognates:
Language contact:

***Cascabela thevetioides* (Kunth) Lippold (Apocynaceae)**

Spanish names: calaverita
Indigenous names: Mbiigu'[21]; Yoyotli[28]
Used by (2*): Zapotec[21]; Nahua[28]
Used for (6#): Digestive (1)[21]; Neurological (1)[28]; Respiratory (2)[21, 28]; Skin (1)[21]; General and Unspecified (1)[21]
Cognates:
Language contact:

***Casearia aculeata* Jacq. (Salicaceae)**

Spanish names:
Indigenous names: Chumak chul[07]
Used by (1*): Huastec[07]
Used for (2#): Respiratory (1)[07]; Skin (1)[07]
Cognates:
Language contact:

***Casearia corymbosa* Kunth (Salicaceae)**

Spanish names:
Indigenous names: ¥kx cuy, Tajuiñi ay[03]; Xi'mche'[09]
Used by (2*): Zoque[03]; Yucatecan Maya[09]
Used for (3#): Skin (2)[03, 09]; General and Unspecified (1)[09]
Cognates:
Language contact:

***Casearia sylvestris* Sw. (Salicaceae)**

Spanish names:
Indigenous names: Chumak chul[07]
Used by (1*): Huastec[07]
Used for (2#): Respiratory (1)[07]; Skin (1)[07]
Cognates:
Language contact:

***Casimiroa edulis* La Llave (Rutaceae)**

Spanish names: Matasano; Zapote blanco
Indigenous names: Ajachel[12]; Aja te'[20]; Yàg-ngùd-guèy[23]; Chipizapotl[28]
Used by (5*): Quichean Maya[12]; Western Maya[20]; Zapotec[23]; Nahua[25, 28]
Used for (5#): Blood (1)[12]; Digestive (1)[20]; Cardiovascular (2)[23, 25]; Pregnancy (1)[28]
Cognates: CoreM: aja;
Language contact:

***Casimiroa tetrameria* Millsp. (Rutaceae)**

Spanish names:
Indigenous names: Yuy, Sihun[09]
Used by (1*): Yucatecan Maya[09]
Used for (4#): Digestive (1)[09]; Musculoskeletal (1)[09]; Respiratory (1)[09]; General and Unspecified (1)[09]
Cognates:
Language contact:

***Cassia fistula* L. (Fabaceae)**

Spanish names: Caña fistula ; Cañafistola; Cañafistula
Indigenous names: Tsapt tsina'an[04]; Oj fistula[18]
Used by (4*): Zoque[01]; Mixe[04]; Western Maya[18, 19]
Used for (8#): Digestive (3)[01, 04, 18]; Respiratory (3)[01, 04, 18]; General and Unspecified (2)[04, 19]
Cognates:
Language contact:

***Cassia grandis* L.f. (Fabaceae)**

Spanish names: Cañafistula
Indigenous names: Bocut[10]
Used by (2*): Yucatecan Maya[10]; Zapotec[21]
Used for (4#): Blood (1)[10]; Respiratory (1)[21]; Skin (1)[10]; General and Unspecified (1)[21]
Cognates:
Language contact:

[01-28]refer to the study codes in Table 4.1. *Total number of studies citing this taxon #Total number of use-records

***Cassia moschata* Kunth (Fabaceae)**

Spanish names: Caña fista; Cañafistula
Indigenous names: Tzum kuy[02]
Used by (2*): Zoque[02, 03]
Used for (9#): Digestive (1)[02]; Musculoskeletal (1)[02]; Respiratory (2)[02, 03]; Urological (1)[02]; Pregnancy (1)[02]; Female genital (1)[02]; General and Unspecified (2)[02, 03]
Cognates:
Language contact:

***Cassia* sp. (Fabaceae)**

Spanish names: Citiso Laburno
Indigenous names: Q'en Q'echa Kineq' Aq'om[12]
Used by (1*): Quichean Maya[12]
Used for (8#): Blood (1)[12]; Cardiovascular (1)[12]; Musculoskeletal (1)[12]; Neurological (1)[12]; Psychological (1)[12]; Skin (1)[12]; Endocrine (1)[12]; Urological (1)[12]
Cognates:
Language contact:

***Castela tortuosa* Liebm. (Simaroubaceae)**

Spanish names: Chaparro amargoso; Venenillo
Indigenous names:
Used by (2*): Zoque[01]; Nahua[27]
Used for (4#): Digestive (1)[01]; Cardiovascular (1)[27]; Psychological (1)[27]; Endocrine (1)[27]
Cognates:
Language contact:

***Castilla elastica* Cerv. (Moraceae)**

Spanish names: Ule
Indigenous names: Peem[07]
Used by (2*): Huastec[07]; Yucatecan Maya[10]
Used for (4#): Musculoskeletal (2)[07, 10]; Neurological (1)[07]; Skin (1)[07]
Cognates:
Language contact:

***Castilleja scorzonerifolia* Kunth (Orobanchaceae)**

Spanish names:
Indigenous names: Elul ts'ohool, ehtiil hut'ut wuts, k'ak'al wits, akan t'ele' del monte, pulek bohol ch'ohool[07]
Used by (2*): Zoque[03]; Huastec[07]
Used for (5#): Digestive (2)[03, 07]; Neurological (1)[07]; Pregnancy (1)[07]; Female genital (1)[07]
Cognates:
Language contact:

***Catasetum integerrimum* Hook. (Orchidaceae)**

Spanish names:
Indigenous names: Ch'itku'uk[09]
Used by (1*): Yucatecan Maya[09]
Used for (2#): Musculoskeletal (1)[09]; Skin (1)[09]
Cognates:
Language contact:

***Catharanthus roseus* (L.) G.Don (Apocynaceae)**

Spanish names: Ninfa, vicaria, paraguita
Indigenous names: Ninfax[03]
Used by (7*): Zoque[01, 02, 03]; Yucatecan Maya[09, 11]; Western Maya[18]; Zapotec[21]
Used for (18[#]): Digestive (3)[02, 03, 21]; Eye (1)[18]; Musculoskeletal (1)[02]; Neurological (1)[03]; Respiratory (1)[18]; Skin (2)[18, 21]; Endocrine (1)[18]; Pregnancy (2)[03, 11]; Female genital (3)[02, 03, 09]; General and Unspecified (3)[01, 18, 21]
Cognates:
Language contact:

***Catopsis* sp. (Bromeliaceae)**

Spanish names: Piñita
Indigenous names:
Used by (1*): Zoque[03]
Used for (1[#]): Female genital (1)[03]
Cognates:
Language contact:

***Cayaponia racemosa* (Mill.) Cogn. (Cucurbitaceae)**

Spanish names: Hierba del soldado
Indigenous names: Wit po't[04]; Takeeyl[09]; Shuana'a soldadu[21]
Used by (3*): Mixe[04]; Yucatecan Maya[09]; Zapotec[21]
Used for (4[#]): Digestive (1)[21]; Skin (2)[04, 09]; General and Unspecified (1)[21]
Cognates:
Language contact:

***Ceanothus caeruleus* Lag. (Rhamnaceae)**

Spanish names: Charín
Indigenous names: Tatanatzin[28]
Used by (2*): Quichean Maya[12]; Nahua[28]
Used for (3[#]): Digestive (2)[12, 28]; Skin (1)[28]
Cognates:
Language contact:

***Cecropia obtusifolia* Bertol. (Urticaceae)**

Spanish names: Guarumbo, chancarro
Indigenous names: Mats[03]; Joot[04]; Akowa[05]; AA 'koo hua[06]; Tsulte'[07]; Xk'oochle'[09]; Aq'poo'jor, Po'jor[14]; Ajc'oloc'[18]
Used by (10*): Zoque[03]; Mixe[04]; Totonac[05, 06]; Huastec[07]; Yucatecan Maya[09]; Quichean Maya[14]; Western Maya[18]; Nahua[25, 26]
Used for (26[#]): Digestive (2)[03, 25]; Ear (1)[03]; Cardiovascular (1)[18]; Musculoskeletal (1)[04]; Psychological (2)[03, 18]; Respiratory (2)[03, 26]; Skin (3)[03, 07, 18]; Endocrine (7)[03, 04, 05, 06, 18, 25, 26]; Urological (2)[09, 26]; Pregnancy (1)[14]; Female genital (1)[03]; General and Unspecified (3)[03, 04, 14]
Cognates: Zoq: mats; Toto: akowa; CoreM: (a)koo;
Language contact: Mixe <> Tot <> CoreM

***Cecropia peltata* L. (Urticaceae)**

Spanish names: Guarumbo; Guarumo
Indigenous names: Ha'ql[14]; Chojb'[19]
Used by (4*): Yucatecan Maya[11]; Quichean Maya[14, 15]; Western Maya[19]
Used for (7[#]): Musculoskeletal (1)[11]; Psychological (1)[15]; Skin (1)[11]; Pregnancy (3)[14, 15, 19]; General and Unspecified (1)[14]
Cognates:
Language contact:

***Cecropia* sp. (Urticaceae)**

Spanish names: Guarrumbo; Guarumbo, chancarro
Indigenous names: Ma'atz kuy/ma'atza /maatsy kuy[01]; Watz, boron kuy, syi wangpo', shy wonhko[02]; Yagaba'aj[21]
Used by (3*): Zoque[01, 02]; Zapotec[21]
Used for (27#): Digestive (3)[01, 02, 21]; Ear (1)[01]; Cardiovascular (1)[02]; Musculoskeletal (3)[01, 02, 21]; Psychological (1)[01]; Respiratory (2)[01, 21]; Skin (3)[01, 02, 21]; Endocrine (3)[01, 02, 21]; Urological (2)[01, 02]; Pregnancy (1)[01]; Female genital (3)[01, 02, 21]; Male genital (1)[02]; General and Unspecified (3)[01, 02, 21]
Cognates: Zoq: atz;
Language contact: Zoq <> Zap

***Cedrela odorata* L. (Meliaceae)**

Spanish names: Cedro
Indigenous names: A' kuy/ aj kuy[01]; Aja kuy/masya kuy[02]; Acuy[03]; Ajk[04]; Lištankiwi, pušnankiwi[05]; Ik'te'[07]; Rix Yaw Che'[14]; Ch'ujte[18]; Yagadoo[21]; Yàg-sîdr[23]
Used by (13*): Zoque[01, 02, 03]; Mixe[04]; Totonac[05]; Huastec[07]; Yucatecan Maya[09, 10]; Quichean Maya[12, 14]; Western Maya[18]; Zapotec[21, 23]
Used for (43#): Blood (2)[01, 07]; Digestive (4)[01, 02, 03, 23]; Cardiovascular (1)[12]; Musculoskeletal (5)[01, 02, 03, 12, 21]; Neurological (4)[01, 02, 07, 12]; Psychological (2)[05, 07]; Respiratory (3)[01, 07, 21]; Skin (6)[01, 03, 07, 10, 14, 21]; Endocrine (2)[01, 02]; Urological (2)[01, 03]; Pregnancy (2)[03, 18]; Female genital (3)[03, 09, 12]; General and Unspecified (7)[01, 03, 04, 05, 07, 18, 21]
Cognates: MZ: Ajk; Zoq: a(j) kuy;
Language contact: MZ > Hua and Tot

***Cedrela salvadorensis* Standl. (Meliaceae)**

Spanish names:
Indigenous names: Nawal Che' Aq'om[12]
Used by (1*): Quichean Maya[12]
Used for (3#): Blood (1)[12]; Skin (1)[12]; General and Unspecified (1)[12]
Cognates:
Language contact:

***Ceiba acuminata* (S.Watson) Rose (Malvaceae)**

Spanish names: Ceiba
Indigenous names: Yaga shiene'e[21]
Used by (1*): Zapotec[21]
Used for (2#): Pregnancy (1)[21]; General and Unspecified (1)[21]
Cognates:
Language contact:

***Ceiba aesculifolia* (Kunth) Britten & Baker f. (Malvaceae)**

Spanish names: Pochote
Indigenous names: Pi'im[09]
Used by (2*): Yucatecan Maya[09]; Nahua[27]
Used for (7#): Digestive (1)[27]; Eye (1)[09]; Skin (2)[09, 27]; Endocrine (1)[27]; Urological (1)[27]; General and Unspecified (1)[27]
Cognates:
Language contact:

***Ceiba pentandra* (L.) Gaertn. (Malvaceae)**

Spanish names: Ceiba, pochote
Indigenous names: Pixtyiñ[03]; Pix ti'ink[04]; Unup[07]; Ya'axche'[09]; Ch'ixirte[18]; Biuug[21]
Used by (6*): Zoque[03]; Mixe[04]; Huastec[07]; Yucatecan Maya[09]; Western Maya[18]; Zapotec[21]
Used for (9#): Digestive (1)[21]; Musculoskeletal (1)[09]; Skin (4)[03, 04, 07, 21]; Endocrine (1)[18]; General and Unspecified (2)[07, 21]
Cognates: MZ: pixtin;
Language contact: MZ > Chontal

***Celosia argentea*** **L. (Amaranthaceae)**

Spanish names: Mano de leon / Cresta de gallo; Moco de chunto
Indigenous names: Cundauajo[03]; Sam' ak'ach'[14]
Used by (2*): Zoque[03]; Quichean Maya[14]
Used for (2#): Blood (1)[14]; Female genital (1)[03]
Cognates:
Language contact:

***Celtis iguanaea*** **(Jacq.) Sarg. (Cannabaceae)**

Spanish names:
Indigenous names: Thak loh[07]
Used by (1*): Huastec[07]
Used for (2#): Skin (1)[07]; General and Unspecified (1)[07]
Cognates:
Language contact:

***Cenchrus echinatus*** **L. (Poaceae)**

Spanish names:
Indigenous names: T'oyol k'iith, t'oyol toom[07]
Used by (1*): Huastec[07]
Used for (5#): Digestive (1)[07]; Psychological (1)[07]; Urological (1)[07]; Pregnancy (1)[07]; General and Unspecified (1)[07]
Cognates:
Language contact:

***Centratherum punctatum*** **Cass. (Asteraceae)**

Spanish names: Serpentina morada; Violeta de maceta
Indigenous names:
Used by (2*): Zoque[03]; Nahua[26]
Used for (3#): Respiratory (1)[26]; Skin (1)[26]; Pregnancy (1)[03]
Cognates:
Language contact:

***Centropogon cordifolius*** **Benth. (Campanulaceae)**

Spanish names:
Indigenous names: Ayuni/putpa' tane[01]
Used by (1*): Zoque[01]
Used for (1#): Skin (1)[01]
Cognates:
Language contact:

***Centrosema pubescens*** **Benth. (Fabaceae)**

Spanish names: Chureque
Indigenous names:
Used by (2*): Zoque[03]; Quichean Maya[12]
Used for (3#): Digestive (2)[03, 12]; Endocrine (1)[12]
Cognates:
Language contact:

***Centrosema sagittatum*** **(Willd.) L.Riley (Fabaceae)**

Spanish names:
Indigenous names: Hik'elom ts'olool, lantha xeklek[07]; Buy-ak'[09]
Used by (3*): Zoque[03]; Huastec[07]; Yucatecan Maya[09]
Used for (5#): Digestive (1)[03]; Eye (1)[09]; Psychological (1)[03]; Skin (1)[09]; General and Unspecified (1)[07]
Cognates:
Language contact:

[01-28] refer to the study codes in Table 4.1. *Total number of studies citing this taxon #Total number of use-records

***Cerastium guatemalense*** **Standl. (Caryophyllaceae)**

Spanish names: Hoja de azar
Indigenous names: Tza'a tzoy[01]
Used by (1*): Zoque[01]
Used for (1#): General and Unspecified (1)[01]
Cognates:
Language contact:

***Ceratozamia mexicana*** **Brongn. (Zamiaceae)**

Spanish names: Palmito
Indigenous names: Cuni[06]; Konlib[07]
Used by (2*): Totonac[06]; Huastec[07]
Used for (2#): Respiratory (1)[06]; Urological (1)[07]
Cognates:
Language contact:

***Cestrum aurantiacum*** **Lindl. (Solanaceae)**

Spanish names: Flor de goñon, huele de noche, jiquilete de monte, chilca de monte, sacate negro; Hierba de la Bilis, Hierba Morada
Indigenous names: Q'enqoj K'ay Q'os Aq'om[12]; Choanum, quispar chuq'ies, xapakpan[13]
Used by (2*): Quichean Maya[12, 13]
Used for (6#): Digestive (2)[12, 13]; Musculoskeletal (1)[13]; Respiratory (1)[13]; General and Unspecified (2)[12, 13]
Cognates:
Language contact:

***Cestrum dumetorum*** **Schltdl. (Solanaceae)**

Spanish names: Boton zhiwite; Matabuey; Orcajuda
Indigenous names: Tsabalte'[07]; Tsabalte'[08]; Patonšiwit[22]; Yàg-guièe-zhĭn, bòtòn-xìwît[23]
Used by (4*): Huastec[07, 08]; Zapotec[22, 23]
Used for (15#): Digestive (1)[22]; Musculoskeletal (1)[08]; Neurological (4)[07, 08, 22, 23]; Psychological (1)[07]; Respiratory (2)[07, 08]; Skin (1)[07]; Urological (1)[07]; Pregnancy (1)[07]; General and Unspecified (3)[07, 08, 23]
Cognates: Huas: tsabalte; Zapo: pVton xiwit;
Language contact:

***Cestrum fasciculatum*** **(Schltdl.) Miers (Solanaceae)**

Spanish names:
Indigenous names: Nixtamaxihuitl[25]
Used by (1*): Nahua[25]
Used for (3#): Neurological (1)[25]; Skin (1)[25]; General and Unspecified (1)[25]
Cognates:
Language contact:

***Cestrum nocturnum*** **L. (Solanaceae)**

Spanish names: Huele de noche
Indigenous names: Mok xoxay[03]; Tzisnutuwan, tzisni šanat[05]; Ehek tsabalte', it'iib to'ol[07]; Ejek tsabalte¿[08]; Xu Che'[14]
Used by (8*): Zoque[02, 03]; Totonac[05]; Huastec[07, 08]; Yucatecan Maya[09]; Quichean Maya[14]; Nahua[28]
Used for (18#): Digestive (1)[14]; Musculoskeletal (1)[08]; Neurological (1)[28]; Respiratory (1)[08]; Skin (3)[03, 09, 28]; Endocrine (1)[02]; Pregnancy (2)[03, 05]; Female genital (1)[03]; Male genital (1)[14]; General and Unspecified (6)[03, 05, 07, 08, 09, 14]
Cognates: Huas: eCek tsabalte;
Language contact:

***Cestrum schlechtendahlii* G.Don (Solanaceae)**

Spanish names:
Indigenous names: Rax ipaj, Xna ipaj[14]
Used by (1*): Quichean Maya[14]
Used for (2#): Endocrine (1)[14]; Male genital (1)[14]
Cognates:
Language contact:

***Cestrum tomentosum* L.f. (Solanaceae)**

Spanish names: Hierba del torro; Hoja helionanda
Indigenous names: Wujpa ay[01]; Ehepacxihuitl[26]
Used by (2*): Zoque[01]; Nahua[26]
Used for (2#): Neurological (1)[01]; General and Unspecified (1)[26]
Cognates:
Language contact:

***Chaetocalyx brasiliensis* (Vogel) Benth. (Fabaceae)**

Spanish names:
Indigenous names: M¥kstogay tsay[03]
Used by (1*): Zoque[03]
Used for (2#): Digestive (1)[03]; Skin (1)[03]
Cognates:
Language contact:

***Chamaecrista diphylla* (L.) Greene (Fabaceae)**

Spanish names:
Indigenous names:
Used by (1*): Zoque[03]
Used for (1#): Female genital (1)[03]
Cognates:
Language contact:

***Chamaecrista fagonioides* (Vogel) H.S.Irwin & Barneby (Fabaceae)**

Spanish names: Hoja sen
Indigenous names: Guixa'a sen[21]
Used by (1*): Zapotec[21]
Used for (5#): Digestive (1)[21]; Skin (1)[21]; Pregnancy (1)[21]; Female genital (1)[21]; General and Unspecified (1)[21]
Cognates:
Language contact:

***Chamaecrista flexuosa* (L.) Greene (Fabaceae)**

Spanish names: Ortiga
Indigenous names: Copa uaxiñ[03]
Used by (1*): Zoque[03]
Used for (3#): Digestive (1)[03]; Skin (1)[03]; Female genital (1)[03]
Cognates:
Language contact:

***Chamaecrista glandulosa (*L.) Greene (Fabaceae)**

Spanish names:
Indigenous names: Salatxiw, Salat-ik'[09]
Used by (1*): Yucatecan Maya[09]
Used for (1#): Eye (1)[09]
Cognates:
Language contact:

[01-28]refer to the study codes in Table 4.1. *Total number of studies citing this taxon #Total number of use-records

**_Chamaecrista hispidula_ (Vahl) H.S.Irwin & Barneby (Fabaceae)**

Spanish names: Hoja de burbua; Hoja sen
Indigenous names: Kojotzik ay / kotzäk ay[02]; Hitxi sotyi[03]; Sen ay[04]
Used by (3*): Zoque[02, 03]; Mixe[04]
Used for (7#): Digestive (1)[02]; Skin (1)[03]; Pregnancy (1)[02]; Female genital (3)[02, 03, 04]; General and Unspecified (1)[04]
Cognates:
Language contact:

**_Chamaecrista kunthiana_ (Schltdl. et Cham.) H. S. Irwin & Barneby (Fabaceae)**

Spanish names:
Indigenous names:
Used by (1*): Zoque[03]
Used for (2#): Eye (1)[03]; Urological (1)[03]
Cognates:
Language contact:

**_Chamaedorea elegans_ Mart. (Arecaceae)**

Spanish names:
Indigenous names: Weew bat'aw, thokob santu[07]
Used by (1*): Huastec[07]
Used for (2#): Psychological (1)[07]; Skin (1)[07]
Cognates:
Language contact:

**_Chamaedorea oblongata_ Mart. (Arecaceae)**

Spanish names: Tepejilote, tepexilot
Indigenous names: Liljtampan, litumpajna[05]
Used by (1*): Totonac[05]
Used for (1#): General and Unspecified (1)[05]
Cognates:
Language contact:

**_Chamaedorea pinnatifrons_ (Jacq.) Oerst. (Arecaceae)**

Spanish names: Tepejilote
Indigenous names: Pampi[03]
Used by (1*): Zoque[03]
Used for (1#): Respiratory (1)[03]
Cognates:
Language contact:

**_Chamaedorea_ sp. (Arecaceae)**

Spanish names: Chiquilote; Pacaya
Indigenous names: Chikx pampi[03]
Used by (2*): Zoque[03]; Quichean Maya[12]
Used for (9#): Blood (1)[12]; Digestive (1)[12]; Respiratory (1)[12]; Skin (1)[12]; Endocrine (2)[12, 12]; Pregnancy (1)[12]; General and Unspecified (2)[03, 12]
Cognates:
Language contact:

**_Chamaedorea tepejilote_ Liebm. (Arecaceae)**

Spanish names: Tepejilote
Indigenous names: Gue'etzu'u[21]; Tepechilote[26]
Used by (2*): Zapotec[21]; Nahua[26]
Used for (5#): Digestive (2)[21, 26]; Skin (2)[21, 26]; General and Unspecified (1)[21]
Cognates:
Language contact:

[01-28]refer to the study codes in Table 4.1. *Total number of studies citing this taxon #Total number of use-records

***Chaptalia nutans* (L.) Polák (Asteraceae)**

Spanish names: Diente de león; Hoja de dos colores; Lengua de chivo; Lengua de perro
Indigenous names: Tzyivu totz [01]; Ai'jäs poti[02]; Jeepe ay, Nuup kobak[03]; Yo'opky piig ujts[04]; Tsakam yehtsel, ehtiil i k'ubak kw'a, alte' lopeen[07]; Rujiraq'i Tz'i'[14]; Guish lay león[21]
Used by (7*): Zoque[01, 02, 03]; Mixe[04]; Huastec[07]; Quichean Maya[14]; Zapotec[21]
Used for (16#): Digestive (3)[03, 14, 21]; Eye (2)[02, 07]; Musculoskeletal (1)[01]; Neurological (1)[04]; Psychological (2)[03, 14]; Respiratory (1)[14]; Skin (2)[03, 04]; Urological (2)[03, 14]; Pregnancy (1)[03]; General and Unspecified (1)[07]
Cognates:
Language contact: Highland Popoluca <> Hua

***Cheilanthes angustifolia* Kunth (Pteridaceae)**

Spanish names: Culandrillo
Indigenous names:
Used by (1*): Quichean Maya[13]
Used for (1#): Neurological (1)[13]
Cognates:
Language contact:

***Chelonanthus alatus (*Aubl.) Pulle (Gentianaceae)**

Spanish names: Lengua de vaca
Indigenous names:
Used by (1*): Zoque[02]
Used for (1#): Skin (1)[02]
Cognates:
Language contact:

***Chenopodium berlandieri* Moq. (Amaranthaceae)**

Spanish names: Bledo, Amaranto
Indigenous names: Lob'i tzetz[12]
Used by (1*): Quichean Maya[12]
Used for (3#): Cardiovascular (1)[12]; Urological (1)[12]; General and Unspecified (1)[12]
Cognates:
Language contact:

***Chenopodium incisum* Poir. (Amaranthaceae)**

Spanish names: Epazote de zorillo; Hierba de zorrillo
Indigenous names: Sa 'kalhka'jna'[06]; Guìzh-mèt, ptiè-mèt[23]
Used by (2*): Totonac[06]; Zapotec[23]
Used for (3#): Digestive (2)[06, 23]; Female genital (1)[23]
Cognates:
Language contact:

***Chenopodium murale* L. (Amaranthaceae)**

Spanish names: Apacín; Chagaquelite; Yerba de guajilote
Indigenous names: Ka'i's par', par q'os[12]; Škwam behd[22]
Used by (3*): Quichean Maya[12]; Zapotec[22]; Nahua[27]
Used for (5#): Respiratory (1)[12]; Skin (2)[12, 22]; General and Unspecified (2)[12, 27]
Cognates:
Language contact:

[01-28]refer to the study codes in Table 4.1. *Total number of studies citing this taxon #Total number of use-records

***Chenopodium vulvaria* L. (Amaranthaceae)**

Spanish names: Apazote zorro; Epazote de monte, epazote de zorrillo
Indigenous names: Sik'aj par, uskaj par[13]
Used by (2*): Totonac[05]; Quichean Maya[13]
Used for (6#): Digestive (3)[05, 05, 13]; Pregnancy (1)[13]; Female genital (1)[13]; General and Unspecified (1)[13]
Cognates:
Language contact:

***Chimaphila maculata* (L.) Pursh (Ericaceae)**

Spanish names: Hierba del sapo
Indigenous names: Ka'che, p'jal q'ix[13]
Used by (1*): Quichean Maya[13]
Used for (1#): General and Unspecified (1)[13]
Cognates:
Language contact:

***Chiococca alba* (L.) Hitchc. (Rubiaceae)**

Spanish names: Moste cimarrón, sorrillo
Indigenous names: Majei muts[03]; Puut' ts'aah, te' laab te'[07]; Chimes-kas, Xiax-al'[09]; Ix ak'ä jauay[11]; Pahar i pim[17]; Xín-bèch-mbăr[23]
Used by (6*): Zoque[03]; Huastec[07]; Yucatecan Maya[09, 11]; Quichean Maya[17]; Zapotec[23]
Used for (14#): Digestive (2)[03, 11]; Musculoskeletal (2)[07, 11]; Neurological (1)[03]; Psychological (1)[03]; Respiratory (1)[03]; Skin (3)[03, 09, 23]; General and Unspecified (3)[03, 07, 11]; nd[17]
Cognates:
Language contact:

***Chiococca* sp. (Rubiaceae)**

Spanish names: Tamagás
Indigenous names:
Used by (1*): Western Maya[19]
Used for (3#): Digestive (1)[19]; Pregnancy (1)[19]; Female genital (1)[19]
Cognates:
Language contact:

***Chionolaena salicifolia* (Bertol.) G.L.Nesom (Asteraceae)**

Spanish names: Gordolobo hembra
Indigenous names: Saq' Mak'el Q'os[12]
Used by (1*): Quichean Maya[12]
Used for (2#): Respiratory (1)[12]; Skin (1)[12]
Cognates:
Language contact:

***Chiranthodendron pentadactylon* Larreat. (Malvaceae)**

Spanish names: Manita; Manita de león; Mano de león
Indigenous names: K'ubak k'wa'[08]; Q'anaq'[12]; Yàg-làz[23]
Used by (3*): Huastec[08]; Quichean Maya[12]; Zapotec[23]
Used for (8#): Digestive (1)[08]; Cardiovascular (2)[08, 12]; Musculoskeletal (1)[08]; Neurological (1)[08]; Psychological (1)[12]; Respiratory (1)[23]; Skin (1)[08]
Cognates:
Language contact: Hua <> Kaqchikel

***Chloroleucon mangense* (Jacq.) Britton & Rose (Fabaceae)**

Spanish names:
Indigenous names: Xiax-ek'[09]
Used by (1*): Yucatecan Maya[09]
Used for (1#): Skin (1)[09]
Cognates:
Language contact:

***Chromolaena collina* (DC.) R.M.King & H.Rob. (Asteraceae)**

Spanish names: Flor de gas, hoja petrolio
Indigenous names: Ehek witsiim, t'unu' witsiim[07]; Sakil sak ba te'[20]; Guish petrol[21]
Used by (3*): Huastec[07]; Western Maya[20]; Zapotec[21]
Used for (6#): Digestive (2)[07, 20]; Neurological (1)[07]; Skin (2)[07, 21]; General and Unspecified (1)[07]
Cognates:
Language contact:

***Chromolaena glaberrima* (DC.) R.M.King & H.Rob. (Asteraceae)**

Spanish names: Venadillo
Indigenous names:
Used by (1*): Western Maya[19]
Used for (1#): Digestive (1)[19]
Cognates:
Language contact:

***Chromolaena laevigata* (Lam.) R.M.King & H.Rob. (Asteraceae)**

Spanish names:
Indigenous names: Hatz[11]
Used by (1*): Yucatecan Maya[11]
Used for (2#): Musculoskeletal (1)[11]; General and Unspecified (1)[11]
Cognates:
Language contact:

***Chromolaena odorata* (L.) R.M.King & H.Rob. (Asteraceae)**

Spanish names: Prodigiosa, hoja de cruz
Indigenous names: Tam juñi[03]; Krus tok'te', tsamnek ts'ohool, thi'al t'eel[07]; Tok'aban[09]; Guish crush[21]
Used by (5*): Zoque[03]; Huastec[07]; Yucatecan Maya[09]; Quichean Maya[17]; Zapotec[21]
Used for (8#): Digestive (2)[03, 21]; Psychological (1)[07]; Respiratory (1)[07]; Endocrine (1)[09]; Urological (1)[09]; General and Unspecified (1)[07]; nd[17]
Cognates: Mayan: tok;
Language contact:

***Chrysobalanus icaco* L. (Chrysobalanaceae)**

Spanish names: Caco
Indigenous names:
Used by (1*): Zoque[02]
Used for (1#): Digestive (1)[02]
Cognates:
Language contact:

***Chrysophyllum mexicanum* Brandegee (Sapotaceae)**

Spanish names: Caimito silvestre
Indigenous names: Ajiya[03]; Chi'keeh[09]
Used by (2*): Zoque[03]; Yucatecan Maya[09]
Used for (4#): Digestive (2)[03, 09]; Skin (1)[03]; Female genital (1)[03]
Cognates:
Language contact:

[01-28]refer to the study codes in Table 4.1. *Total number of studies citing this taxon #Total number of use-records

***Chrysopogon zizanioides* (L.) Roberty (Poaceae)**

Spanish names: Valeriana
Indigenous names:
Used by (1*): Zoque[01]
Used for (1#): Digestive (1)[01]
Cognates:
Language contact:

***Cichorium intybus* L. (Asteraceae)**

Spanish names: Achicoria, Intibina
Indigenous names:
Used by (1*): Quichean Maya[12]
Used for (6#): Blood (1)[12]; Digestive (1)[12]; Skin (1)[12]; Endocrine (1)[12]; Urological (1)[12]; General and Unspecified (1)[12]
Cognates:
Language contact:

***Cinchona officinalis* L. (Rubiaceae)**

Spanish names: Quina
Indigenous names: Saq'i Che' / Saq Paau[14]
Used by (1*): Quichean Maya[14]
Used for (4#): Digestive (1)[14]; Endocrine (1)[14]; Female genital (1)[14]; General and Unspecified (1)[14]
Cognates:
Language contact:

***Cinnamomum brenesii (*Standl.) Kosterm (Lauraceae)**

Spanish names: Rosa negra
Indigenous names: Contra moko[03]
Used by (1*): Zoque[03]
Used for (3#): Digestive (1)[03]; Skin (1)[03]; General and Unspecified (1)[03]
Cognates:
Language contact:

***Cinnamomum* sp. (Lauraceae)**

Spanish names: Canela
Indigenous names:
Used by (3*): Quichean Maya[12, 14]; Nahua[26]
Used for (8#): Digestive (1)[12]; Musculoskeletal (1)[12]; Neurological (1)[12]; Psychological (1)[12]; Respiratory (2)[12, 26]; General and Unspecified (2)[12, 14]
Cognates:
Language contact:

***Cinnamomum tampicense* (Meisn.) Kosterm. (Lauraceae)**

Spanish names:
Indigenous names: Tsaayleel ohte'[07]
Used by (1*): Huastec[07]
Used for (1#): General and Unspecified (1)[07]
Cognates:
Language contact:

***Cinnamomum verum* J.Presl (Lauraceae)**

Spanish names: Canela
Indigenous names:
Used by (11*): Zoque[01, 02, 03]; Mixe[04]; Totonac[06]; Quichean Maya[13, 14]; Western Maya[18, 19]; Zapotec[21]; Nahua[25]
Used for (44#): Digestive (8)[01, 02, 03, 04, 06, 18, 19, 21]; Eye (1)[02]; Cardiovascular (1)[02]; Musculoskeletal (1)[02]; Neurological (1)[18]; Psychological (1)[02]; Respiratory (9)[01, 02, 03, 06, 13, 18, 19, 21, 25]; Skin (2)[18, 25]; Endocrine (1)[01]; Urological (1)[01]; Pregnancy (9)[01, 03, 06, 13, 14, 18, 19, 21, 25]; Female genital (5)[02, 04, 14, 18, 19]; General and Unspecified (4)[02, 13, 18, 21]
Cognates:
Language contact:

***Cionosicyos excisus* (Griseb.) C.Jeffrey (Cucurbitaceae)**

Spanish names:
Indigenous names: Kasam[09]
Used by (1*): Yucatecan Maya[09]
Used for (1#): Skin (1)[09]
Cognates:
Language contact:

***Cirsium ehrenbergii* Sch.Bip. (Asteraceae)**

Spanish names:
Indigenous names: Awin tsäpä[01]
Used by (1*): Zoque[01]
Used for (1#): Skin (1)[01]
Cognates:
Language contact:

***Cirsium jorullense* (Kunth) Spreng. (Asteraceae)**

Spanish names:
Indigenous names: Awin tsäpä[01]
Used by (1*): Zoque[01]
Used for (1#): Skin (1)[01]
Cognates:
Language contact:

***Cirsium mexicanum* DC. (Asteraceae)**

Spanish names: Alcachofa; Cardo santo; Cardosanto
Indigenous names: Kix' che', ukix che''[13]; Tepehuitzo[26]
Used by (5*): Zoque[03]; Quichean Maya[12, 13]; Nahua[25, 26]
Used for (13#): Blood (1)[12]; Digestive (3)[03, 12, 13]; Musculoskeletal (1)[13]; Respiratory (2)[03, 13]; Skin (1)[03]; Urological (2)[25, 26]; Pregnancy (1)[26]; General and Unspecified (2)[12, 25]
Cognates:
Language contact:

***Cirsium* sp. (Asteraceae)**

Spanish names:
Indigenous names: Cholich[07]
Used by (1*): Huastec[07]
Used for (3#): Digestive (1)[07]; Musculoskeletal (1)[07]; Respiratory (1)[07]
Cognates:
Language contact:

***Cirsium subcoriaceum* (Less.) Sch.Bip. ex Sch.Bip. (Asteraceae)**

Spanish names: Cardo Santo
Indigenous names: Sak' q'ix[12]
Used by (1*): Quichean Maya[12]
Used for (10#): Blood (1)[12]; Digestive (1)[12]; Cardiovascular (1)[12]; Musculoskeletal (1)[12]; Neurological (1)[12]; Psychological (1)[12]; Endocrine (1)[12]; Urological (1)[12]; Male genital (1)[12]; General and Unspecified (1)[12]
Cognates:
Language contact:

***Cissampelos owariensis* P.Beauv. ex DC. (Menispermaceae)**

Spanish names: Cintzo
Indigenous names: Cintzo[25]
Used by (1*): Nahua[25]
Used for (1#): General and Unspecified (1)[25]
Cognates:
Language contact:

***Cissampelos pareira* L. (Menispermaceae)**

Spanish names: Alcotán, bejucillo, curalina
Indigenous names: Taka ime/ tzana ay[01]; Tyiñi woyo[03]; Poop axtaam pikx[04]; K'on k'ach, walik ts'ohool, ichiichbe ts'ohool, uxum ichich, xuts ts'ohool, bok'ool ch'ohool[07]; Peteltun[09]; Ch'up i ai[17]; Chin ak' wamal, voy chij vomol, voy chij tz'i'lel, yaxal nixh vomol, tz'urupik' vomol, kurarina, pak chak, makmak chak', chin ak[20]
Used by (10*): Zoque[01, 03]; Mixe[04]; Totonac[05]; Huastec[07]; Yucatecan Maya[09, 11]; Quichean Maya[17]; Western Maya[19, 20]
Used for (19#): Digestive (7)[01, 03, 04, 07, 09, 19, 20]; Respiratory (1)[03]; Skin (4)[01, 03, 05, 20]; Endocrine (1)[01]; Pregnancy (1)[20]; Female genital (1)[03]; General and Unspecified (3)[05, 07, 11]; nd[17]
Cognates:
Language contact:

***Cissampelos* sp. (Menispermaceae)**

Spanish names: Bejuco zorrillo
Indigenous names: Sison' u'u[14]; Ajbejcuo a'uch[18]
Used by (2*): Quichean Maya[14]; Western Maya[18]
Used for (3#): Cardiovascular (1)[14]; General and Unspecified (2)[14, 18]
Cognates:
Language contact:

***Cissampelos tropaeolifolia* DC. (Menispermaceae)**

Spanish names: Bejuco de ombligo; Redondillo
Indigenous names: Tyiñi woyo[03]; Ch'up i ai[17]
Used by (3*): Zoque[03]; Quichean Maya[15, 17]
Used for (3#): Skin (1)[03]; Pregnancy (1)[15]; nd[17]
Cognates:
Language contact:

***Cissus erosa* Rich. (Vitaceae)**

Spanish names:
Indigenous names: Chikxtsay[03]
Used by (1*): Zoque[03]
Used for (1#): Skin (1)[03]
Cognates:
Language contact:

***Cissus microcarpa* Vahl (Vitaceae)**

Spanish names:
Indigenous names: Rok' ha'b'[17]
Used by (1*): Quichean Maya[17]
Used for (1#): nd[17]
Cognates:
Language contact:

***Cissus* sp. (Vitaceae)**

Spanish names: Pata de Hamaca
Indigenous names: Yax tsamnek [07]; Roq' Ab'[14]
Used by (2*): Huastec[07]; Quichean Maya[14]
Used for (2#): Skin (2)[07, 14]
Cognates:
Language contact:

***Cissus trifoliata* (L.) L. (Vitaceae)**

Spanish names:
Indigenous names: Cruz ojo xiw[09]
Used by (1*): Yucatecan Maya[09]
Used for (1#): Digestive (1)[09]
Cognates:
Language contact:

***Cissus verticillata* (L.) Nicolson & C.E.Jarvis (Vitaceae)**

Spanish names: Bejuco loco; Sanatodo; Uva cimarron, san julas
Indigenous names: Omisal[05]; Yax tsamnek, yax tsaah, pulik wako, k'apwal huuchul, [07]; Baladxi'i guexii, elbue sang'laash[21]; Blàg-pâsm, Ibæ̀-pâsm, guìzh-pâsm[23]
Used by (5*): Totonac[05]; Huastec[07]; Western Maya[18]; Zapotec[21, 23]
Used for (9#): Eye (1)[21]; Musculoskeletal (1)[05]; Skin (4)[05, 07, 18, 21]; General and Unspecified (3)[07, 21, 23]
Cognates:
Language contact:

***Citharexylum berlandieri* S.Watson (Verbenaceae)**

Spanish names:
Indigenous names: Wal to'ol te', ist'am te', ehtiil i tsak te', [07]
Used by (1*): Huastec[07]
Used for (1#): Musculoskeletal (1)[07]
Cognates:
Language contact:

***Citrullus lanatus* (Thunb.) Matsum. & Nakai (Cucurbitaceae)**

Spanish names: Sandía
Indigenous names: Mono[02]; Xandia[03]
Used by (4*): Zoque[02, 03]; Quichean Maya[12]; Western Maya[18]
Used for (5#): Digestive (1)[03]; General and Unspecified (4)[02, 03, 12, 18]
Cognates:
Language contact:

***Citrus* sp. (Rutaceae)**

Spanish names: Cajera
Indigenous names:
Used by (1*): Yucatecan Maya[09]
Used for (1#): Digestive (1)[09]
Cognates:
Language contact:

[01-28]refer to the study codes in Table 4.1. *Total number of studies citing this taxon #Total number of use-records

**_Citrus_ × _aurantium_ L. (Rutaceae)**

Spanish names: Naranja agría
Indigenous names: Katzu Tzyina/nanasya/ täme ay[01]; Anasa katzu/ansya katzu[02]; Tsootso[03]; Škeja lašuš[05]; Laaxux[06]; Thimallon lanaax, hiliy lanaax[07]; Pak'aal[09]; Pajäl aranxax[18]; Naraxa guayu'u[21]
Used by (13*): Zoque[01, 02, 03]; Totonac[05, 06]; Huastec[07]; Yucatecan Maya[09, 11]; Quichean Maya[12, 13, 15]; Western Maya[18]; Zapotec[21]
Used for (54#): Blood (1)[12]; Digestive (7)[01, 03, 06, 07, 09, 11, 18]; Cardiovascular (3)[03, 11, 12]; Musculoskeletal (5)[01, 03, 06, 12, 15]; Neurological (4)[03, 12, 13, 18]; Psychological (8)[01, 06, 07, 11, 12, 13, 15, 18]; Respiratory (7)[01, 02, 03, 07, 12, 18, 21]; Skin (4)[03, 07, 12, 21]; Endocrine (2)[01, 12]; Urological (1)[01]; Female genital (3)[01, 03, 12]; General and Unspecified (9)[01, 03, 05, 06, 07, 11, 15, 18, 21]
Cognates:
Language contact:

**_Citrus aurantiifolia (_Christm.) Swingle (Rutaceae)**

Spanish names: Lima, limón criollo
Indigenous names: Limones/katzu[01]; Imunisy/Tzunakat[02]; Hiliy limoon[07]; Limonix[12]
Used by (10*): Zoque[01, 02]; Totonac[06]; Huastec[07]; Yucatecan Maya[09, 10]; Quichean Maya[12, 14]; Zapotec[21]; Nahua[25]
Used for (54#): Blood (1)[12]; Digestive (7)[01, 02, 06, 07, 09, 12, 21]; Eye (2)[02, 06]; Ear (1)[01]; Cardiovascular (3)[01, 02, 12]; Musculoskeletal (3)[01, 07, 12]; Neurological (3)[01, 02, 12]; Psychological (3)[01, 02, 12]; Respiratory (8)[01, 02, 07, 09, 10, 12, 14, 25]; Skin (6)[01, 02, 07, 14, 21, 25]; Endocrine (2)[01, 12]; Urological (3)[01, 02, 06]; Pregnancy (2)[02, 14]; Female genital (1)[01]; Male genital (1)[02]; General and Unspecified (8)[01, 02, 06, 07, 12, 14, 25, 25]
Cognates:
Language contact:

**_Citrus grandis_ (L.) Osbeck (Rutaceae)**

Spanish names: Naranja grey
Indigenous names: Pajen grey[18]
Used by (1*): Western Maya[18]
Used for (1#): Endocrine (1)[18]
Cognates:
Language contact:

**_Citrus limon_ (L.) Osbeck (Rutaceae)**

Spanish names: Limón
Indigenous names: Apitx cuy[03]; Tsøpox[04]; Limuunix, xucut[06]; Chuuchu' liima[07]; Chuuchu lima)[08]; Re' li be'[14]; Paj'limon[18]; Cuanani'ij[21]
Used by (12*): Zoque[03]; Mixe[04]; Totonac[06]; Huastec[07, 08]; Yucatecan Maya[09]; Quichean Maya[14]; Western Maya[18, 19]; Zapotec[21, 22]; Nahua[28]
Used for (53#): Digestive (10)[03, 04, 06, 08, 09, 18, 19, 21, 22, 28]; Eye (3)[14, 21, 22]; Cardiovascular (2)[08, 28]; Musculoskeletal (1)[03]; Neurological (2)[18, 19]; Psychological (5)[03, 06, 18, 19, 21]; Respiratory (9)[03, 04, 06, 07, 08, 18, 19, 21, 28]; Skin (4)[03, 04, 19, 21]; Endocrine (1)[18]; Urological (2)[18, 19]; Pregnancy (4)[03, 04, 06, 19]; Female genital (2)[03, 19]; Male genital (1)[03]; General and Unspecified (7)[03, 08, 14, 18, 19, 21, 22]
Cognates:
Language contact:

**_Citrus maxima_ (Burm.) Merr. (Rutaceae)**

Spanish names: Pomela; Toronja
Indigenous names:
Used by (2*): Mixe[04]; Quichean Maya[12]
Used for (6#): Blood (1)[12]; Musculoskeletal (1)[12]; Respiratory (1)[04]; Endocrine (1)[12]; Urological (1)[12]; General and Unspecified (1)[12]
Cognates:
Language contact:

[01-28]refer to the study codes in Table 4.1. *Total number of studies citing this taxon #Total number of use-records

***Citrus medica* L. (Rutaceae)**

Spanish names: Lima, cidra
Indigenous names: Liima[07]; Tzon te lima[08]; Xaq i'chi'in[16]
Used by (8*): Zoque[01]; Mixe[04]; Totonac[06]; Huastec[07, 08]; Quichean Maya[13, 16]; Nahua[26]
Used for (18#): Digestive (2)[01, 08]; Eye (2)[01, 13]; Musculoskeletal (1)[01]; Psychological (4)[01, 06, 16, 26]; Respiratory (2)[07, 08]; Endocrine (1)[06]; Female genital (2)[01, 13]; General and Unspecified (4)[01, 04, 08, 26]
Cognates:
Language contact:

***Citrus paradisi* Macfad. (Rutaceae)**

Spanish names: Toronja
Indigenous names:
Used by (2*): Zoque[03]; Totonac[06]
Used for (4#): Digestive (2)[03, 06]; Psychological (1)[06]; Endocrine (1)[06]
Cognates:
Language contact:

***Citrus reticulata* Blanco (Rutaceae)**

Spanish names: Mandarina; Naranja cajera
Indigenous names: Pajen cajera[18]
Used by (5*): Zoque[01]; Totonac[06]; Yucatecan Maya[09]; Quichean Maya[12]; Western Maya[18]
Used for (5#): Digestive (2)[09, 18]; Cardiovascular (1)[06]; Respiratory (2)[01, 12]
Cognates:
Language contact:

***Citrus sinensis* (L.) Osbeck (Rutaceae)**

Spanish names: Naranja
Indigenous names: China/pos[01]; Anasa/anasya[02]; Tsuik[04]; Laaxux[06]; Lanash[08]; China[09]; Paàk'al[10]; Alan[12]; Alanxax[13]; Aranxax[18]; Naraxa[21]; Chilcoztic[28]
Used by (15*): Zoque[01, 02]; Mixe[04]; Totonac[06]; Huastec[08]; Yucatecan Maya[09, 10]; Quichean Maya[12, 13, 14]; Western Maya[18, 19]; Zapotec[21]; Nahua[25, 28]
Used for (64#): Blood (1)[12]; Digestive (11)[01, 02, 06, 08, 09, 12, 13, 18, 19, 25, 28]; Cardiovascular (4)[01, 02, 10, 18]; Musculoskeletal (4)[08, 12, 14, 19]; Neurological (4)[02, 18, 19, 21]; Psychological (8)[01, 02, 06, 08, 12, 13, 18, 19]; Respiratory (11)[01, 02, 06, 08, 12, 14, 18, 19, 21, 25, 28]; Skin (3)[02, 12, 21]; Endocrine (3)[02, 18, 25]; Urological (2)[13, 25]; Pregnancy (3)[02, 19, 21]; General and Unspecified (10)[01, 02, 04, 08, 10, 12, 13, 19, 21, 25]
Cognates:
Language contact:

***Citrus* sp. (Rutaceae)**

Spanish names: Lima; Limón; Limón agrio; Naranja
Indigenous names: Lamux[16]; Ermunex[20]; Narax[20]; Yàg-lîm[23]; Yàg-nàrânj[23]
Used by (7*): Quichean Maya[12, 13, 16]; Western Maya[20]; Zapotec[22, 23]; Nahua[26]
Used for (28#): Blood (1)[12]; Digestive (7)[13, 20, 20, 22, 23, 23, 26]; Eye (1)[12]; Cardiovascular (1)[12]; Musculoskeletal (2)[12, 23]; Psychological (3)[12, 16, 26]; Respiratory (1)[13]; Skin (1)[13]; Endocrine (2)[12, 23]; Urological (1)[13]; Pregnancy (3)[13, 22, 23]; Female genital (1)[13]; General and Unspecified (4)[13, 23, 26, 26]
Cognates:
Language contact:

***Cladocolea* sp. (Loranthaceae)**

Spanish names:
Indigenous names: Guièe-ló-yâg-guièts, guièe-ló-yâg-nlìbâd-tsò[23]
Used by (1*): Zapotec[23]
Used for (1#): Neurological (1)[23]
Cognates:
Language contact:

***Clematis dioica* L. (Ranunculaceae)**

Spanish names: Barbas de chivo, pestaña de tecolote
Indigenous names: Skaltzatzat momo, slarpitsitsit monksne[05]; Xmexmexib[09]; Ch'am pim[14]
Used by (3*): Totonac[05]; Yucatecan Maya[09]; Quichean Maya[14]
Used for (4#): Respiratory (1)[05]; Skin (2)[09, 14]; General and Unspecified (1)[14]
Cognates:
Language contact:

***Clematis grossa* Benth. (Ranunculaceae)**

Spanish names: Bejuco de barba viejo; Corraleña blanca
Indigenous names: Mäkshi jäyä[01]; Tsay ¥wix, Kunki kuts¥wi[03]; Ithim wahuts, ithim yehtsel, ithim an maam, ithim an pulik, ithim an pulek taata[07]
Used by (3*): Zoque[01, 03]; Huastec[07]
Used for (8#): Neurological (1)[07]; Respiratory (1)[07]; Skin (3)[01, 03, 07]; Pregnancy (1)[03]; Female genital (1)[03]; General and Unspecified (1)[03]
Cognates:
Language contact:

***Cleome aculeata* L. (Cleomaceae)**

Spanish names:
Indigenous names: Utsun ts'ohool[07]
Used by (1*): Huastec[07]
Used for (1#): Neurological (1)[07]
Cognates:
Language contact:

***Cleome viscosa* L. (Cleomaceae)**

Spanish names:
Indigenous names:
Used by (1*): Quichean Maya[12]
Used for (3#): Digestive (1)[12]; Male genital (1)[12]; General and Unspecified (1)[12]
Cognates:
Language contact:

***Cleoserrata serrata* (Jacq.) Iltis (Capparaceae)**

Spanish names:
Indigenous names: Huntal a puunchiix[07]
Used by (1*): Huastec[07]
Used for (1#): Eye (1)[07]
Cognates:
Language contact:

***Cleoserrata speciosa* (Raf.) Iltis (Capparaceae)**

Spanish names:
Indigenous names: Charamooya[03]
Used by (1*): Zoque[03]
Used for (1#): Skin (1)[03]
Cognates:
Language contact:

[01-28]refer to the study codes in Table 4.1. *Total number of studies citing this taxon #Total number of use-records

***Clerodendrum chinense*** **(Osbeck) Mabb. (Lamiaceae)**

Spanish names:
Indigenous names: Ts'een kwiniimte[07]
Used by (1*): Huastec[07]
Used for (1#): Skin (1)[07]
Cognates:
Language contact:

***Clethra occidentalis*** **(L.) Kuntze (Clethraceae)**

Spanish names:
Indigenous names:
Used by (1*): Zoque[03]
Used for (2#): Skin (1)[03]; Female genital (1)[03]
Cognates:
Language contact:

***Clethra suaveolens*** **Turcz. (Clethraceae)**

Spanish names:
Indigenous names: K'ajk'ete'[20]
Used by (1*): Western Maya[20]
Used for (1#): Digestive (1)[20]
Cognates:
Language contact:

***Clibadium arboreum*** **Donn.Sm. (Asteraceae)**

Spanish names:
Indigenous names: Tza'jil Che'[14]
Used by (1*): Quichean Maya[14]
Used for (1#): Respiratory (1)[14]
Cognates:
Language contact:

***Clibadium surinamense*** **L. (Asteraceae)**

Spanish names:
Indigenous names:
Used by (1*): Quichean Maya[14]
Used for (1#): Endocrine (1)[14]
Cognates:
Language contact:

***Clidemia capitellata*** **(Bonpl.) D. Don (Melastomataceae)**

Spanish names:
Indigenous names: Ik pim[17]
Used by (1*): Quichean Maya[17]
Used for (1#): nd[17]
Cognates:
Language contact:

***Clidemia petiolaris*** **(Schltdl. & Cham.) Schltdl. ex Triana (Melastomataceae)**

Spanish names: Tesuatillo de algodón
Indigenous names: Puki tesua[03]; Tsakam chikab ts'ohool, uxum ts'ohool, ehtiil ts'amuts' uxkwe, ehtiil puwaamte'[07]; Xa bol q'een[15]
Used by (3*): Zoque[03]; Huastec[07]; Quichean Maya[15]
Used for (6#): Digestive (1)[03]; Eye (1)[03]; Cardiovascular (1)[07]; Respiratory (1)[07]; Skin (1)[03]; Pregnancy (1)[15]
Cognates:
Language contact: Highland Popoluca <> Hua

[01-28]refer to the study codes in Table 4.1. *Total number of studies citing this taxon #Total number of use-records

***Clidemia setosa* (Triana) Gleason (Melastomataceae)**

Spanish names:
Indigenous names: Kaka rane [01]; Ixq Q'een[15]
Used by (2*): Zoque[01]; Quichean Maya[15]
Used for (3[#]): Pregnancy (1)[15]; Male genital (1)[15]; General and Unspecified (1)[01]
Cognates:
Language contact:

***Clinopodium brownei* (Sw.) Kuntze (Lamiaceae)**

Spanish names: Poleo, matalcín, hierba de espanto
Indigenous names: Jupi tane/tzämi tane/ jupi rane/ tzuk'u pu [01]; Piiquaa't tu 'huaa'n[06]; Ts'ots'on ts'ohool, ehtiil tsakam wiichab[07]; Tonalxihuitl[26]
Used by (6*): Zoque[01]; Totonac[05, 06]; Huastec[07]; Yucatecan Maya[09]; Nahua[26]
Used for (16[#]): Blood (1)[05]; Digestive (2)[01, 07]; Eye (1)[01]; Musculoskeletal (1)[26]; Neurological (3)[01, 07, 09]; Psychological (1)[26]; Respiratory (1)[01]; Pregnancy (1)[07]; General and Unspecified (5)[01, 05, 06, 07, 26]
Cognates:
Language contact: Chiapas Zoq <> Tot <> Hua

***Clinopodium macrostemum* (Moc. & Sessé ex Benth.) Kuntze (Lamiaceae)**

Spanish names: Poleo
Indigenous names: Yàg-wǎas, guìzh-wǎas[23]
Used by (1*): Zapotec[23]
Used for (3[#]): Digestive (1)[23]; Psychological (1)[23]; General and Unspecified (1)[23]
Cognates:
Language contact:

***Clinopodium mexicanum (*Benth.) Govaerts (Lamiaceae)**

Spanish names: Poleo
Indigenous names:
Used by (1*): Zapotec[22]
Used for (2[#]): Neurological (1)[22]; Respiratory (1)[22]
Cognates:
Language contact:

***Clitoria guianensis (*Aubl.) Benth. (Fabaceae)**

Spanish names:
Indigenous names: Jonwayi[03]
Used by (1*): Zoque[03]
Used for (2[#]): Neurological (1)[03]; Skin (1)[03]
Cognates:
Language contact:

***Clusia flava* Jacq. (Clusiaceae)**

Spanish names: Memela; Oreja de Cabro
Indigenous names: Upu kutkuy ay [01]; Xk'ik' yuk' hubulo'[14]
Used by (2*): Zoque[01]; Quichean Maya[14]
Used for (2[#]): Female genital (1)[01]; General and Unspecified (1)[14]
Cognates:
Language contact:

[01-28]refer to the study codes in Table 4.1. *Total number of studies citing this taxon [#]Total number of use-records

***Clusia salvinii* Donn.Sm. (Clusiaceae)**

Spanish names:
Indigenous names: Ch'iy'ak[12]
Used by (1*): Quichean Maya[12]
Used for (3#): Musculoskeletal (1)[12]; Psychological (1)[12]; General and Unspecified (1)[12]
Cognates:
Language contact:

***Cnidoscolus aconitifolius* (Mill.) I.M.Johnst. (Euphorbiaceae)**

Spanish names: Chaya, mala mujer
Indigenous names: Ata tsäpe/ata/ kenuk tsäpe[01]; Tzis kä'wang [02]; Chay[09]; Yop'ix'ek'[18]
Used by (8*): Zoque[01, 02, 03]; Yucatecan Maya[09]; Western Maya[18]; Nahua[25, 26, 27]
Used for (16#): Musculoskeletal (1)[01]; Neurological (1)[02]; Skin (1)[01]; Endocrine (4)[01, 18, 25, 27]; Urological (3)[02, 03, 26]; Pregnancy (2)[01, 18]; Female genital (1)[09]; Male genital (1)[02]; General and Unspecified (2)[09, 27]
Cognates:
Language contact:

***Cnidoscolus liebmannii* (Müll. Arg) Lundell (Euphorbiaceae)**

Spanish names: Chichicastle
Indigenous names: Kenuk[03]
Used by (1*): Zoque[03]
Used for (6#): Digestive (1)[03]; Neurological (1)[03]; Respiratory (1)[03]; Skin (1)[03]; Urological (1)[03]; Female genital (1)[03]
Cognates:
Language contact:

***Cnidoscolus multilobus* (Pax) I.M.Johnst. (Euphorbiaceae)**

Spanish names: Chichicastle; Hortiga, mala mujer; Mala mujer; Ortiga
Indigenous names: Kenuk[03]; Gahni, cahh'ne, kgajna, xaca'nat[05]; Kajni[06]; Ak', ma'iy ak'[07]; Yàg-làg[23]
Used by (5*): Zoque[03]; Totonac[05, 06]; Huastec[07]; Zapotec[23]
Used for (23#): Eye (2)[05, 06]; Musculoskeletal (2)[05, 06]; Neurological (5)[03, 05, 05, 06, 07]; Respiratory (1)[06]; Skin (4)[05, 06, 07, 23]; Endocrine (1)[06]; Urological (1)[07]; Pregnancy (2)[05, 07]; Female genital (4)[03, 05, 06, 07]; General and Unspecified (1)[06]
Cognates: Toto: kajnV;
Language contact: Zoq >Toto and Hua

***Cnidoscolus souzae* McVaugh (Euphorbiaceae)**

Spanish names: Chaya de monte
Indigenous names:
Used by (1*): Yucatecan Maya[09]
Used for (1#): Musculoskeletal (1)[09]
Cognates:
Language contact:

***Cnidoscolus tubulosus* (Müll.Arg.) I.M.Johnst. (Euphorbiaceae)**

Spanish names: Ejaj
Indigenous names: Kh'ajni[08]; Yàg-pcuà[23]
Used by (2*): Huastec[08]; Zapotec[23]
Used for (2#): Digestive (1)[08]; Skin (1)[23]
Cognates:
Language contact:

[01-28]refer to the study codes in Table 4.1. *Total number of studies citing this taxon #Total number of use-records

***Cnidoscolus urens* (L.) Arthur (Euphorbiaceae)**

Spanish names: Mala mujer
Indigenous names: Geč bahd[22]
Used by (1*): Zapotec[22]
Used for (1#): Musculoskeletal (1)[22]
Cognates:
Language contact:

***Coccocypselum hirsutum* Bartl. ex DC. (Rubiaceae)**

Spanish names: Hoja de cobertón
Indigenous names: Tsujmi ay[03]
Used by (1*): Zoque[03]
Used for (3#): Skin (1)[03]; Female genital (1)[03]; General and Unspecified (1)[03]
Cognates:
Language contact:

***Coccoloba barbadensis* Jacq. (Polygonaceae)**

Spanish names: Totopotzle, carnero negro/coyote, cinco negrito; Uvero
Indigenous names: Pakum[03]; Bidxuyej' shuga'a, shuubguijooj'[21]
Used by (2*): Zoque[03]; Zapotec[21]
Used for (8#): Digestive (1)[03]; Respiratory (1)[21]; Skin (2)[03, 21]; Female genital (2)[03, 21]; General and Unspecified (2)[03, 21]
Cognates:
Language contact:

***Coccoloba liebmannii* Lindau (Polygonaceae)**

Spanish names:
Indigenous names: Shuug[21]
Used by (1*): Zapotec[21]
Used for (4#): Respiratory (1)[21]; Skin (1)[21]; Female genital (1)[21]; General and Unspecified (1)[21]
Cognates:
Language contact:

***Coccoloba montana* Standl. (Polygonaceae)**

Spanish names: Uvero de montaña
Indigenous names: J¥mniom pakum[03]
Used by (1*): Zoque[03]
Used for (1#): Digestive (1)[03]
Cognates:
Language contact:

***Coccoloba* sp. (Polygonaceae)**

Spanish names: Carnero de coyote
Indigenous names: Shuug nguio'o[21]
Used by (1*): Zapotec[21]
Used for (5#): Digestive (1)[21]; Musculoskeletal (1)[21]; Respiratory (1)[21]; Skin (1)[21]; Female genital (1)[21]
Cognates:
Language contact:

***Coccoloba spicata* Lundell (Polygonaceae)**

Spanish names:
Indigenous names: Bob[09]
Used by (1*): Yucatecan Maya[09]
Used for (1#): Skin (1)[09]
Cognates:
Language contact:

**_Coccoloba uvifera_ (L.) L. (Polygonaceae)**

Spanish names: Uva de mar
Indigenous names:
Used by (1*): Yucatecan Maya[09]
Used for (1#): Urological (1)[09]
Cognates:
Language contact:

**_Cocculus diversifolius_ DC. (Menispermaceae)**

Spanish names:
Indigenous names: Lek'ab t'iim, ichich ts'ohool, kw'itool ichich, lek'ab tiiw, buk ichich[07]
Used by (1*): Huastec[07]
Used for (2#): Digestive (1)[07]; General and Unspecified (1)[07]
Cognates:
Language contact:

**_Cochlospermum vitifolium_ (Willd.) Spreng. (Bixaceae)**

Spanish names: Pochote, coquito
Indigenous names: Putz kuy[02]; Puts cuy[03]; Nø mu'und[04]; Te'aj pochote[18]; T'uyuy, mujrur te'[19]; Vapombu[21]
Used by (6*): Zoque[02, 03]; Mixe[04]; Western Maya[18, 19]; Zapotec[21]
Used for (16#): Digestive (4)[02, 03, 04, 21]; Neurological (1)[03]; Skin (3)[02, 03, 18]; Endocrine (2)[02, 03]; Pregnancy (1)[19]; Female genital (3)[02, 03, 21]; General and Unspecified (2)[03, 21]
Cognates: Zoq: puts kuy;
Language contact:

**_Cocos nucifera_ L. (Arecaceae)**

Spanish names: Coco
Indigenous names:
Used by (10*): Zoque[01, 02, 03]; Mixe[04]; Yucatecan Maya[09]; Quichean Maya[12]; Western Maya[18, 19]; Zapotec[21]; Nahua[28]
Used for (22#): Digestive (9)[01, 02, 03, 04, 12, 18, 19, 21, 28]; Neurological (1)[01]; Psychological (3)[01, 02, 18]; Endocrine (1)[18]; Urological (2)[01, 12]; Pregnancy (2)[09, 21]; Female genital (2)[03, 12]; Male genital (1)[12]; General and Unspecified (1)[21]
Cognates:
Language contact:

**_Codonanthe crassifolia_ (H.Focke) C.V.Morton (Gesneriaceae)**

Spanish names:
Indigenous names: Saq'kar pim[14]
Used by (1*): Quichean Maya[14]
Used for (2#): Respiratory (1)[14]; General and Unspecified (1)[14]
Cognates:
Language contact:

**_Coffea arabica_ L. (Rubiaceae)**

Spanish names: Café
Indigenous names: Capel[03]; Kafeey[04]; Capij[06]; Kapee[07]; Cape'[12]
Used by (11*): Zoque[01, 03]; Mixe[04]; Totonac[06]; Huastec[07]; Yucatecan Maya[09]; Quichean Maya[12, 14]; Western Maya[18, 19]; Nahua[26]
Used for (29#): Digestive (3)[03, 06, 26]; Cardiovascular (1)[01]; Musculoskeletal (3)[01, 06, 12]; Neurological (2)[12, 19]; Psychological (1)[26]; Respiratory (3)[01, 12, 19]; Skin (5)[01, 04, 06, 07, 14]; Urological (2)[01, 06]; Pregnancy (4)[01, 09, 12, 26]; Female genital (1)[12]; General and Unspecified (4)[03, 12, 18, 19]
Cognates:
Language contact:

***Coffea* sp. (Rubiaceae)**

Spanish names: Café
Indigenous names: Guis gue[21]
Used by (2*): Quichean Maya[13]; Zapotec[21]
Used for (7#): Digestive (1)[21]; Neurological (1)[13]; Skin (1)[21]; Pregnancy (2)[13, 21]; General and Unspecified (2)[13, 21]
Cognates:
Language contact:

***Cojoba arborea* (L.) Britton & Rose (Fabaceae)**

Spanish names: Carabina de chango
Indigenous names: Uuts tuj cuy[03]; Xlokok' hi ha'[14]
Used by (2*): Zoque[03]; Quichean Maya[14]
Used for (4#): Digestive (1)[03]; Skin (2)[03, 14]; General and Unspecified (1)[14]
Cognates:
Language contact:

***Colocasia esculenta* (L.) Schott (Araceae)**

Spanish names: Malanga
Indigenous names:
Used by (2*): Quichean Maya[12]; Zapotec[21]
Used for (2#): Endocrine (1)[12]; General and Unspecified (1)[21]
Cognates:
Language contact:

***Colubrina arborescens* (Mill.) Sarg. (Rhamnaceae)**

Spanish names: Toatán
Indigenous names: Jauté[18]
Used by (1*): Western Maya[18]
Used for (1#): General and Unspecified (1)[18]
Cognates:
Language contact:

***Colubrina greggii* S.Watson (Rhamnaceae)**

Spanish names:
Indigenous names: Ehtiil tsakam akich, itsaan an tsak look',[07]
Used by (1*): Huastec[07]
Used for (1#): Musculoskeletal (1)[07]
Cognates:
Language contact:

***Columnea schiedeana* Schltdl. (Gesneriaceae)**

Spanish names: Jala
Indigenous names: Hui'huat[06]
Used by (1*): Totonac[06]
Used for (2#): Digestive (1)[06]; Musculoskeletal (1)[06]
Cognates:
Language contact:

***Columnea* sp. (Gesneriaceae)**

Spanish names:
Indigenous names: Kaki pim[16]
Used by (1*): Quichean Maya[16]
Used for (1#): Neurological (1)[16]
Cognates:
Language contact:

***Columnea sulfurea* Donn.Sm. (Gesneriaceae)**

Spanish names:
Indigenous names: Xoy Q'ehen, Kaq'i pim[14]; Kak'i pim[17]
Used by (2*): Quichean Maya[14, 17]
Used for (5#): Digestive (1)[14]; Eye (1)[14]; Neurological (1)[14]; Respiratory (1)[14]; nd[17]
Cognates: Quich: kaki pim;
Language contact:

***Combretum fruticosum* (Loefl.) Stuntz (Combretaceae)**

Spanish names:
Indigenous names: Ka'an shan k'aham[17]
Used by (1*): Quichean Maya[17]
Used for (1#): nd[17]
Cognates:
Language contact:

***Combretum laxum* Jacq. (Combretaceae)**

Spanish names:
Indigenous names: Uouo tsay, Patan tsay[03]
Used by (1*): Zoque[03]
Used for (2#): Digestive (1)[03]; Skin (1)[03]
Cognates:
Language contact:

***Combretum* sp. (Combretaceae)**

Spanish names:
Indigenous names: Qa'xa'an caham[16]
Used by (1*): Quichean Maya[16]
Used for (1#): General and Unspecified (1)[16]
Cognates:
Language contact:

***Commelina coelestis* Willd. (Commelinaceae)**

Spanish names: Hierba de pollo
Indigenous names:
Used by (1*): Nahua[28]
Used for (3#): Digestive (1)[28]; Skin (1)[28]; Female genital (1)[28]
Cognates:
Language contact:

***Commelina diffusa* Burm.f. (Commelinaceae)**

Spanish names: Caterita/hoja de azar/ siempreviva; Matalin verde
Indigenous names: Tzima jäyä/tzuy/ tza'a ay[01]; Tsus uixpin[03]
Used by (2*): Zoque[01, 03]
Used for (7#): Eye (1)[01]; Cardiovascular (1)[01]; Neurological (1)[01]; Skin (1)[01]; Female genital (1)[01]; General and Unspecified (2)[01, 03]
Cognates:
Language contact:

***Commelina erecta*** **L. (Commelinaceae)**

Spanish names: Madali
Indigenous names: Po'otz ay/po'o tzoni[02]; Tsus uixpin[03]; Kasmalj[05]; Utek', mapk'ux ch'ohool[07]; U k'ak' ah ko'lebil, Ya'axha'xiw[09]; Madali[21]
Used by (6*): Zoque[02, 03]; Totonac[05]; Huastec[07]; Yucatecan Maya[09]; Zapotec[21]
Used for (13#): Digestive (2)[02, 07]; Eye (3)[02, 05, 07]; Neurological (1)[02]; Skin (3)[07, 09, 21]; Urological (1)[07]; Female genital (1)[07]; General and Unspecified (2)[03, 07]
Cognates:
Language contact: Hua <> Yuc

***Commelina obliqua*** **Vahl (Commelinaceae)**

Spanish names:
Indigenous names: Tz'i' Maaj Q'ehen[14]
Used by (1*): Quichean Maya[14]
Used for (2#): Cardiovascular (1)[14]; Endocrine (1)[14]
Cognates:
Language contact:

***Commelina*** **sp. (Commelinaceae)**

Spanish names: Hierba de pollo; Uña de Gato, Tripa de Gallina
Indigenous names: Karpar Coos[12]; Pitzijor[13]
Used by (2*): Quichean Maya[12, 13]
Used for (4#): Digestive (1)[13]; Skin (1)[13]; Urological (1)[13]; General and Unspecified (1)[12]
Cognates:
Language contact:

***Comocladia macrophylla*** **(Hook. & Arn.) L.Riley (Anacardiaceae)**

Spanish names: Hinchador, hinchahuevo
Indigenous names: Latz[21]
Used by (1*): Zapotec[21]
Used for (1#): Skin (1)[21]
Cognates:
Language contact:

***Conostegia icosandra*** **(Sw. ex Wilkstr.) Urb. (Melastomataceae)**

Spanish names:
Indigenous names: Jeepe[03]
Used by (1*): Zoque[03]
Used for (1#): Digestive (1)[03]
Cognates:
Language contact:

***Conostegia xalapensis*** **(Bonpl.) D. Don ex DC. (Melastomataceae)**

Spanish names: Capulin niua; Moradito del cerro
Indigenous names: Chuch jeepe[03]
Used by (2*): Zoque[03]; Zapotec[21]
Used for (5#): Digestive (1)[03]; Neurological (1)[03]; Pregnancy (1)[03]; Female genital (1)[03]; General and Unspecified (1)[21]
Cognates:
Language contact:

[01-28]refer to the study codes in Table 4.1. *Total number of studies citing this taxon #Total number of use-records

**_Convolvulus nodiflorus_ Desr. (Convolvulaceae)**

Spanish names: Flor de virgen chico
Indigenous names: Badooj buishii[21]
Used by (1*): Zapotec[21]
Used for (1#): General and Unspecified (1)[21]
Cognates:
Language contact:

**_Conyza gnaphalioides_ Kunth (Asteraceae)**

Spanish names: Simonilla
Indigenous names: Sìmònî[23]
Used by (1*): Zapotec[23]
Used for (2#): Digestive (1)[23]; Cardiovascular (1)[23]
Cognates:
Language contact:

**_Conyza laevigata_ (Rich.) Pruski (Asteraceae)**

Spanish names: Hoja de azar
Indigenous names: Tza'a tzoy[01]; K'atab' Chaj[14]
Used by (2*): Zoque[01]; Quichean Maya[14]
Used for (4#): Respiratory (1)[14]; Skin (1)[14]; General and Unspecified (2)[01, 14]
Cognates:
Language contact:

**_Conyza_ sp. (Asteraceae)**

Spanish names:
Indigenous names: Jok poy[03]
Used by (1*): Zoque[03]
Used for (3#): Skin (1)[03]; Urological (1)[03]; Female genital (1)[03]
Cognates:
Language contact:

**_Corchorus siliquosus_ L. (Malvaceae)**

Spanish names: Malvavisco/ malva macho
Indigenous names: Pehtsul kw'eet, pehtsul thipon, thipon kweet, loliy thipon[07]
Used by (2*): Zoque[01]; Huastec[07]
Used for (9#): Digestive (2)[01, 07]; Eye (1)[07]; Neurological (1)[07]; Skin (1)[07]; Urological (1)[07]; Pregnancy (1)[07]; General and Unspecified (2)[01, 07]
Cognates:
Language contact:

**_Corchorus_ sp. (Malvaceae)**

Spanish names:
Indigenous names: Ueji ay[03]
Used by (1*): Zoque[03]
Used for (1#): Psychological (1)[03]
Cognates:
Language contact:

**_Cordia alba_ (Jacq.) Roem. & Schult. (Boraginaceae)**

Spanish names: Gulabere; Gulaveri
Indigenous names: Syunuk[02]
Used by (2*): Zoque[02]; Zapotec[21]
Used for (5#): Eye (1)[02]; Musculoskeletal (1)[21]; Skin (1)[02]; General and Unspecified (2)[02, 21]
Cognates:
Language contact:

[01-28]refer to the study codes in Table 4.1. *Total number of studies citing this taxon #Total number of use-records

***Cordia alliodora*** **(Ruiz & Pav.) Oken (Boraginaceae)**

Spanish names: Solería
Indigenous names: Kiwa[03]; Wiixte'[07]
Used by (2*): Zoque[03]; Huastec[07]
Used for (2#): Eye (1)[07]; Psychological (1)[03]
Cognates:
Language contact:

***Cordia curassavica*** **(Jacq.) Roem. & Schult. (Boraginaceae)**

Spanish names: Escobillo, mais grande; Hoja de alacrán barraca; Xobarora
Indigenous names: Kaku'e ay[02]; Shubaruuba'a[21]; Gubenigw[22]
Used by (4*): Zoque[02]; Mixe[04]; Zapotec[21, 22]
Used for (8#): Digestive (2)[21, 22]; Respiratory (1)[04]; Female genital (2)[02, 04]; General and Unspecified (3)[04, 21, 22]
Cognates: Zapo: ubV;
Language contact: Zap > Chimalapa Zoq

***Cordia dodecandra*** **A.DC. (Boraginaceae)**

Spanish names:
Indigenous names: Cirricote, Kop'te'[09]
Used by (1*): Yucatecan Maya[09]
Used for (2#): Respiratory (1)[09]; General and Unspecified (1)[09]
Cognates:
Language contact:

***Cordia spinescens*** **L. (Boraginaceae)**

Spanish names: Vara negra
Indigenous names: Y¥k yom tsay[03]; Jolob te'[20]
Used by (2*): Zoque[03]; Western Maya[20]
Used for (4#): Digestive (2)[03, 20]; Skin (1)[03]; Female genital (1)[03]
Cognates:
Language contact:

***Cordyline fruticosa*** **(L.) A. Chev. (Asparagaceae)**

Spanish names: Cola de gallo; Palmita
Indigenous names:
Used by (2*): Zoque[01, 02]
Used for (3#): Musculoskeletal (1)[01]; Endocrine (1)[02]; Female genital (1)[02]
Cognates:
Language contact:

***Cordyline*** **sp. (Asparagaceae)**

Spanish names:
Indigenous names: Cuxtzi[16]
Used by (1*): Quichean Maya[16]
Used for (1#): Neurological (1)[16]
Cognates:
Language contact:

***Coreopsis mutica*** **DC. (Asteraceae)**

Spanish names:
Indigenous names: Bajk' al te'[20]; Ncuàan-bzhiân, guièe ngùzhánch[23]
Used by (2*): Western Maya[20]; Zapotec[23]
Used for (5#): Digestive (2)[20, 23]; Psychological (1)[23]; Endocrine (1)[23]; General and Unspecified (1)[23]
Cognates:
Language contact:

***Coriandrum sativum* L. (Apiaceae)**

Spanish names: Cilantro
Indigenous names: Cuulantu[06]; Kulaantu[07]; Culanto, uklanto´[13]; Sìlândr[23]
Used by (10*): Zoque[01]; Totonac[06]; Huastec[07]; Yucatecan Maya[09]; Quichean Maya[12, 13, 14]; Western Maya[18, 19]; Zapotec[23]
Used for (16[#]): Digestive (7)[06, 07, 09, 12, 13, 18, 19]; Cardiovascular (1)[13]; Musculoskeletal (1)[14]; Psychological (1)[12]; Respiratory (1)[14]; Endocrine (1)[12]; Urological (1)[01]; Pregnancy (2)[13, 23]; General and Unspecified (1)[12]
Cognates:
Language contact:

***Cornutia pyramidata* L. (Lamiaceae)**

Spanish names: Carreto, piojillo, tabaquillo
Indigenous names: M'yonh kuy/ monh'iun kuy[01]; Cana ay, Eexcuy[03]; Aškut kiwi[05]; Xolte'xnuk[09]; Loto'o che[11]; Sa'q Aatz'un[14]; Job lo te[16]; Yop'waúm'[18]; Yax e vomol[20]
Used by (9*): Zoque[01, 03]; Totonac[05]; Yucatecan Maya[09, 11]; Quichean Maya[14, 16]; Western Maya[18, 20]
Used for (17[#]): Digestive (1)[20]; Musculoskeletal (4)[01, 03, 09, 11]; Neurological (2)[16, 18]; Respiratory (1)[09]; Skin (2)[03, 14]; Pregnancy (1)[01]; General and Unspecified (6)[01, 03, 05, 09, 11, 18]
Cognates:
Language contact: Highland Popoluca <> Tot <> Yuc <> Kekchí; Kekchí <> Chontal

***Cortaderia selloana* (Schult. & Schult.f.) Asch. & Graebn. (Poaceae)**

Spanish names: Caña blanca
Indigenous names: Nida'a quitzii[21]
Used by (1*): Zapotec[21]
Used for (2[#]): Respiratory (1)[21]; General and Unspecified (1)[21]
Cognates:
Language contact:

***Cosmos caudatus* Kunth (Asteraceae)**

Spanish names:
Indigenous names: Molajtu sotyi[03]
Used by (1*): Zoque[03]
Used for (2[#]): Skin (1)[03]; General and Unspecified (1)[03]
Cognates:
Language contact:

***Cosmos crithmifolius* Kunth (Asteraceae)**

Spanish names: Copalio
Indigenous names:
Used by (1*): Zoque[01]
Used for (1[#]): Digestive (1)[01]
Cognates:
Language contact:

***Costus laevis* Ruiz & Pav. (Costaceae)**

Spanish names:
Indigenous names: Ch'uun te'[17]
Used by (1*): Quichean Maya[17]
Used for (1[#]): nd[17]
Cognates:
Language contact:

[01-28]refer to the study codes in Table 4.1. *Total number of studies citing this taxon [#]Total number of use-records

***Costus pictus* D.Don (Costaceae)**

Spanish names: Caña agria; Caña de cristo/caña agria/cañita
Indigenous names: Katzu aksa/baston katzu/katzu tane[01]; Chimpa tutu[03]
Used by (2*): Zoque[01, 03]
Used for (6#): Digestive (2)[01, 03]; Cardiovascular (1)[01]; Urological (2)[01, 03]; Female genital (1)[03]
Cognates:
Language contact:

***Costus pulverulentus* C.Presl (Costaceae)**

Spanish names: Caña agria, caña de cristo
Indigenous names: Katzu syitsi[02]; Chimpa tutu[03]; Pakaab olom[07]; Chu'un, Paguy t'e[14]; Nidaii[21]; Cuapitzoatl[25]
Used by (6*): Zoque[02, 03]; Huastec[07]; Quichean Maya[14]; Zapotec[21]; Nahua[25]
Used for (20#): Digestive (4)[02, 03, 07, 21]; Skin (1)[07]; Endocrine (1)[14]; Urological (6)[02, 03, 07, 14, 21, 25]; Pregnancy (1)[14]; Female genital (1)[03]; Male genital (1)[14]; Social problems (1)[14]; General and Unspecified (4)[07, 14, 21, 25]
Cognates:
Language contact:

***Costus* sp. (Costaceae)**

Spanish names: Caña de cristo
Indigenous names: Cha'ncat juki, cha'ncat pa'xni[06]; Tch'ama'jij[12]; Chu'un[14]; Tepeohuate[26]
Used by (5*): Zoque[01]; Totonac[06]; Quichean Maya[12, 14]; Nahua[26]
Used for (12#): Blood (1)[12]; Digestive (3)[01, 12, 26]; Musculoskeletal (1)[12]; Endocrine (2)[06, 12]; Urological (2)[06, 26]; Pregnancy (1)[14]; Male genital (1)[14]; General and Unspecified (1)[12]
Cognates:
Language contact:

***Costus spicatus* (Jacq.) Sw. (Costaceae)**

Spanish names: Caña agria; Caña de venado
Indigenous names: Chankat juki[05]; Oj paj[18]
Used by (2*): Totonac[05]; Western Maya[18]
Used for (3#): Urological (2)[05, 18]; General and Unspecified (1)[05]
Cognates:
Language contact:

***Couepia polyandra* (Kunth) Rose (Chrysobalanaceae)**

Spanish names: Olozapote
Indigenous names: Pillum[03]
Used by (1*): Zoque[03]
Used for (2#): Skin (1)[03]; Female genital (1)[03]
Cognates:
Language contact:

***Crassula ovata* (Mill.) Druce (Crassulaceae)**

Spanish names: Siempreviva
Indigenous names: Wiq bac, pix laq[13]
Used by (1*): Quichean Maya[13]
Used for (5#): Digestive (1)[13]; Eye (1)[13]; Musculoskeletal (1)[13]; Neurological (1)[13]; Skin (1)[13]
Cognates:
Language contact:

***Crataegus mexicana* Moç. & Sess ex DC. (Rosaceae)**

Spanish names: Manzanita
Indigenous names:
Used by (1*): Zoque[01]
Used for (1#): Respiratory (1)[01]
Cognates:
Language contact:

***Crataegus orientalis* Pall. ex M.Bieb. (Rosaceae)**

Spanish names:
Indigenous names: Ch'ix te', k'at'ix, k'achi'ix[20]
Used by (1*): Western Maya[20]
Used for (4#): Digestive (1)[20]; Neurological (1)[20]; Respiratory (1)[20]; Skin (1)[20]
Cognates:
Language contact:

***Crataegus* sp. (Rosaceae)**

Spanish names: Cardo; Manzanilla Arbol; Raíz de manzanita, tejocote
Indigenous names: Q'enum-che'[12]; Yàg-mànzànît[23]
Used by (3*): Zoque[01]; Quichean Maya[12]; Zapotec[23]
Used for (10#): Blood (1)[12]; Digestive (1)[12]; Musculoskeletal (2)[12, 23]; Neurological (1)[12]; Respiratory (1)[12]; Urological (1)[23]; Male genital (1)[12]; General and Unspecified (2)[01, 12]
Cognates:
Language contact:

***Crateva tapia* L. (Capparaceae)**

Spanish names: Coscorrón
Indigenous names: Tsine te', ts'olob ok, thak'chook' buuru, thak'chook' uut', thi'te', ist'am te', thak'chook' teneklaab[07]; Bajpam[18]
Used by (2*): Huastec[07]; Western Maya[18]
Used for (4#): Ear (1)[07]; Musculoskeletal (1)[18]; Neurological (1)[18]; Skin (1)[07]
Cognates:
Language contact:

***Crescentia alata* Kunth (Bignoniaceae)**

Spanish names: Morrito; Morro; Morro simarrón
Indigenous names: Tzima[02]; Thoot tima'[07]; Tzimaj[19]; Buru boj[21]
Used by (4*): Zoque[02]; Huastec[07]; Western Maya[19]; Zapotec[21]
Used for (10#): Digestive (1)[02]; Musculoskeletal (3)[02, 07, 21]; Respiratory (3)[02, 19, 21]; Skin (1)[21]; General and Unspecified (2)[07, 21]
Cognates:
Language contact: Zoq > Maya

***Crescentia cujete* L. (Bignoniaceae)**

Spanish names: Morro, jícara
Indigenous names: Tzima[01]; Tzima[02]; Jeepe[03]; Patsim[04]; Ra taxy[08]; Luch[09]; Tzi Max[12]; Rix'hijom[14]; T'ub[18]; Buru shiiga'a[21]; Yàg-zhìg[23]
Used by (11*): Zoque[01, 02, 03]; Mixe[04]; Huastec[08]; Yucatecan Maya[09]; Quichean Maya[12, 14]; Western Maya[18]; Zapotec[21, 23]
Used for (29#): Blood (1)[03]; Digestive (2)[04, 18]; Ear (1)[03]; Musculoskeletal (3)[02, 03, 21]; Neurological (2)[14, 18]; Respiratory (8)[02, 03, 04, 08, 09, 12, 18, 21]; Skin (3)[02, 12, 21]; Urological (1)[23]; Pregnancy (3)[03, 04, 12]; Male genital (1)[12]; General and Unspecified (4)[01, 12, 18, 21]
Cognates: MZ: tsim; Zoq: tsima; Zapo: zhig;
Language contact: MZ > Kaqchikel and Zap

***Crinum erubescens* L.f. ex Aiton (Amaryllidaceae)**

Spanish names:
Indigenous names: Xts'ulam[09]
Used by (1*): Yucatecan Maya[09]
Used for (2#): Respiratory (1)[09]; Skin (1)[09]
Cognates:
Language contact:

***Crinum* sp. (Amaryllidaceae)**

Spanish names: Lirio
Indigenous names: Pets'kini, Pets'kinil[09]
Used by (2*): Zoque[03]; Yucatecan Maya[09]
Used for (3#): Neurological (1)[09]; Skin (1)[03]; Endocrine (1)[03]
Cognates:
Language contact:

***Crinum* x *amabile* Sonn ex Ker Gawl. (Amaryllidaceae)**

Spanish names: Palenque, maguey blanco
Indigenous names:
Used by (1*): Zoque[01]
Used for (3#): Digestive (1)[01]; Neurological (1)[01]; General and Unspecified (1)[01]
Cognates:
Language contact:

***Critonia campechensis* (B.L.Rob.) R.M.King & H.Rob. (Asteraceae)**

Spanish names:
Indigenous names: Lok' ab' winq'[14]
Used by (1*): Quichean Maya[14]
Used for (1#): Respiratory (1)[14]
Cognates:
Language contact:

***Critonia daleoides* DC. (Asteraceae)**

Spanish names:
Indigenous names: Poma cuy[03]
Used by (1*): Zoque[03]
Used for (2#): Skin (1)[03]; General and Unspecified (1)[03]
Cognates:
Language contact:

***Critonia morifolia* (Mill.) R.M.King & H.Rob. (Asteraceae)**

Spanish names: Canotillo; Hoja de Agua; Hoja de contra-viento, hoja de viento, choplé; Lengua de vaca; San Nicolas; Vara negra
Indigenous names: Vaca totz[01]; Cortia ay[03]; Tsuxky ajuk tyujt[04]; T'unu' holol[07]; Yopo säc'[18]
Used by (8*): Zoque[01, 03]; Mixe[04]; Totonac[05]; Huastec[07]; Quichean Maya[12]; Western Maya[18]; Nahua[26]
Used for (14#): Digestive (2)[03, 07]; Musculoskeletal (3)[01, 03, 18]; Respiratory (2)[12, 26]; Skin (1)[05]; Urological (1)[07]; Pregnancy (1)[03]; Female genital (1)[04]; General and Unspecified (3)[03, 07, 18]
Cognates:
Language contact:

[01-28]refer to the study codes in Table 4.1. *Total number of studies citing this taxon #Total number of use-records

***Critonia quadrangularis (*DC.) R.M.King & H.Rob. (Asteraceae)**

Spanish names: Canotillo; Lengua de cierva; Lengua de vaca
Indigenous names: Wakas yenkuy/ wakas tots ay/wakasy tots[02]; Cortia ay[03]; Pajk teky, payøwa'ats[04]; Holol, alte may[07]; Guish lujtz yuss[21]
Used by (5*): Zoque[02, 03]; Mixe[04]; Huastec[07]; Zapotec[21]
Used for (19#): Digestive (2)[02, 21]; Musculoskeletal (5)[02, 03, 04, 07, 21]; Neurological (3)[02, 03, 07]; Skin (1)[07]; Urological (1)[07]; Pregnancy (1)[02]; Female genital (2)[02, 03]; General and Unspecified (4)[03, 04, 07, 21]
Cognates:
Language contact:

***Critonia spinaciifolia* (DC.) R.M.King & H.Rob. (Asteraceae)**

Spanish names:
Indigenous names: Tsamnek tok'te', animas ts'ohool[07]
Used by (1*): Huastec[07]
Used for (5#): Musculoskeletal (1)[07]; Neurological (1)[07]; Respiratory (1)[07]; Skin (1)[07]; General and Unspecified (1)[07]
Cognates:
Language contact:

***Crossopetalum filipes* (Sprague) Lundell (Celastraceae)**

Spanish names:
Indigenous names: Tzutzi tzaj käki[01]
Used by (1*): Zoque[01]
Used for (1#): Psychological (1)[01]
Cognates:
Language contact:

***Crossopetalum gaumeri* (Loes.) Lundell (Celastraceae)**

Spanish names: Viperol negro
Indigenous names:
Used by (1*): Yucatecan Maya[09]
Used for (2#): Digestive (1)[09]; Skin (1)[09]
Cognates:
Language contact:

***Crossopetalum parviflorum* (Hemsl.) Lundell (Celastraceae)**

Spanish names:
Indigenous names: Ra Mox[15]
Used by (1*): Quichean Maya[15]
Used for (2#): Musculoskeletal (1)[15]; General and Unspecified (1)[15]
Cognates:
Language contact:

***Crossopetalum uragoga* (Jacq.) Kuntze (Celastraceae)**

Spanish names:
Indigenous names: Ts'amuts' uxkwe', tsakam itsal koox, ts'amuts' uthu, tsatsa' ilaal[07]
Used by (1*): Huastec[07]
Used for (4#): Digestive (1)[07]; Eye (1)[07]; Skin (1)[07]; Female genital (1)[07]
Cognates:
Language contact:

[01-28]refer to the study codes in Table 4.1. *Total number of studies citing this taxon #Total number of use-records

***Crotalaria cajanifolia*** **Kunth (Fabaceae)**

Spanish names:
Indigenous names: Paxekuiny[04]
Used by (1*): Mixe[04]
Used for (1#): Psychological (1)[04]
Cognates:
Language contact:

***Crotalaria longirostrata*** **Hook. & Arn. (Fabaceae)**

Spanish names: Chipile; Chipilín; Chipillín
Indigenous names: Tza' tzäpä/ tsaj tzäpä[01]; Txipiñ ts¥p¥[03]; Much', B'uch[12]; Chepil[21]
Used by (4*): Zoque[01, 03]; Quichean Maya[12]; Zapotec[21]
Used for (11#): Blood (1)[01]; Digestive (3)[01, 03, 12]; Cardiovascular (1)[01]; Skin (2)[01, 21]; Pregnancy (1)[01]; Male genital (1)[01]; General and Unspecified (2)[01, 21]
Cognates:
Language contact:

***Crotalaria nitens*** **Kunth (Fabaceae)**

Spanish names: Sonaje de muerto
Indigenous names: Mak'xuxut niy[06]
Used by (1*): Totonac[06]
Used for (2#): Psychological (1)[06]; Urological (1)[06]
Cognates:
Language contact:

***Crotalaria sagittalis*** **L. (Fabaceae)**

Spanish names:
Indigenous names: Tsuts ts¥p¥[03]
Used by (1*): Zoque[03]
Used for (2#): Psychological (1)[03]; Skin (1)[03]
Cognates:
Language contact:

***Crotalaria*** **sp. (Fabaceae)**

Spanish names: Chepíl; Chipilin; Chipillín
Indigenous names: Much'[13]; Pxïizh, ncuàan-yè[23]
Used by (3*): Quichean Maya[12, 13]; Zapotec[23]
Used for (8#): Digestive (1)[23]; Musculoskeletal (1)[12]; Neurological (1)[12]; Psychological (2)[12, 13]; Skin (1)[23]; Female genital (1)[23]; General and Unspecified (1)[23]
Cognates:
Language contact:

***Crotalaria vitellina*** **Ker. Gawl. (Fabaceae)**

Spanish names: Chipillín
Indigenous names: Tsuts ts¥p¥[03]; Thootil tsan, pok' thoot[07]; Much'[12]
Used by (3*): Zoque[03]; Huastec[07]; Quichean Maya[12]
Used for (3#): Psychological (1)[12]; General and Unspecified (2)[03, 07]
Cognates:
Language contact:

[01-28]refer to the study codes in Table 4.1. *Total number of studies citing this taxon #Total number of use-records

**_Croton adspersus_ Benth. (Euphorbiaceae)**

Spanish names: Llora sangre
Indigenous names: Jäpya nhäpin[01]
Used by (1*): Zoque[01]
Used for (1#): Skin (1)[01]
Cognates:
Language contact:

**_Croton alamosanus_ Rose (Euphorbiaceae)**

Spanish names:
Indigenous names: Guaanashnash[21]
Used by (1*): Zapotec[21]
Used for (1#): Skin (1)[21]
Cognates:
Language contact:

**_Croton arboreus_ Millsp. (Euphorbiaceae)**

Spanish names: Copalchin
Indigenous names: Tapu ay[03]
Used by (2*): Zoque[03]; Zapotec[21]
Used for (3#): Skin (2)[03, 21]; Female genital (1)[21]
Cognates:
Language contact:

**_Croton billbergianus_ Müll.Arg. (Euphorbiaceae)**

Spanish names: Quina, ventoside
Indigenous names:
Used by (1*): Nahua[26]
Used for (3#): Neurological (1)[26]; Endocrine (1)[26]; General and Unspecified (1)[26]
Cognates:
Language contact:

**_Croton chichenensis_ Lundell (Euphorbiaceae)**

Spanish names:
Indigenous names: Xebalam, Butsumukuy, Xikinch'omak[09]
Used by (1*): Yucatecan Maya[09]
Used for (1#): Skin (1)[09]
Cognates:
Language contact:

**_Croton ciliatoglandulifer_ Ortega (Euphorbiaceae)**

Spanish names: Chiliue del monte; Mata pescado, hoja de mesquino
Indigenous names: Nä'wang niwi[02]; Xoliiman[07]; Guixaxunaashii[21]; Blàg-zhnâzh, guìzh-blàg-zhnâzh, guìzh-ngùdzìi, guìzh-ngùd-mzhiè-dán[23]
Used by (4*): Zoque[02]; Huastec[07]; Zapotec[21, 23]
Used for (7#): Eye (1)[23]; Musculoskeletal (1)[07]; Skin (3)[02, 21, 23]; General and Unspecified (2)[07, 23]
Cognates: Zapo: xunaax/zhnazh;
Language contact:

**_Croton cortesianus_ Kunth (Euphorbiaceae)**

Spanish names:
Indigenous names: Puthwal[07]
Used by (1*): Huastec[07]
Used for (4#): Digestive (1)[07]; Eye (1)[07]; Skin (1)[07]; Female genital (1)[07]
Cognates:
Language contact:

**_Croton draco_ Schltdl. (Euphorbiaceae)**

Spanish names: Sangregrado, llora sangre
Indigenous names: Nhäpin kuy/ po'a nhäpin/ kun nhäpin / nhäpin pi'owa/ jäpya nhäpin [01]; N¥piñi cuy[03]; Panii[04]; Pazlnankiwi, puklhni, puelnankiwi[05]; Xiixte', xits' te'[07]; Chi'ich' bot[20]; Yague riene[21]
Used by (7*): Zoque[01, 03]; Mixe[04]; Totonac[05]; Huastec[07]; Western Maya[20]; Zapotec[21]
Used for (20#): Digestive (4)[01, 03, 20, 21]; Musculoskeletal (1)[01]; Neurological (3)[01, 03, 07]; Respiratory (2)[05, 21]; Skin (5)[01, 03, 04, 05, 07]; Endocrine (1)[01]; Urological (1)[01]; Pregnancy (1)[05]; Female genital (1)[03]; General and Unspecified (1)[05]
Cognates: MZ: pVn; Zoq: näpin;
Language contact: MZ > Tot

**_Croton flavescens_ Greenm. (Euphorbiaceae)**

Spanish names:
Indigenous names: Guaanashnash[21]
Used by (1*): Zapotec[21]
Used for (1#): Skin (1)[21]
Cognates:
Language contact:

**_Croton glandulosus_ L. (Euphorbiaceae)**

Spanish names:
Indigenous names: Tuxoj tikts[04]
Used by (1*): Mixe[04]
Used for (1#): Digestive (1)[04]
Cognates:
Language contact:

**_Croton guatemalensis_ Lotsy (Euphorbiaceae)**

Spanish names: Hierba de Cancer Macho
Indigenous names: Ru xup Top, Sa'l Q'os[12]
Used by (1*): Quichean Maya[12]
Used for (3#): Neurological (1)[12]; Respiratory (1)[12]; Endocrine (1)[12]
Cognates:
Language contact:

**_Croton humilis_ L. (Euphorbiaceae)**

Spanish names:
Indigenous names: Ik-aban[09]
Used by (1*): Yucatecan Maya[09]
Used for (1#): Skin (1)[09]
Cognates:
Language contact:

**_Croton niveus_ Jacq. (Euphorbiaceae)**

Spanish names: Copachín; Copalchi; Copalchin
Indigenous names: Tin kuy/ponoj kuy[01]; Thak oliy[07]
Used by (4*): Zoque[01, 02]; Huastec[07]; Zapotec[21]
Used for (13#): Digestive (4)[01, 02, 07, 21]; Skin (3)[02, 07, 21]; Urological (1)[01]; Pregnancy (1)[02]; Female genital (2)[02, 21]; General and Unspecified (2)[01, 21]
Cognates:
Language contact:

[01-28]refer to the study codes in Table 4.1. *Total number of studies citing this taxon #Total number of use-records

***Croton oerstedianus* Müll.Arg. (Euphorbiaceae)**

Spanish names: Contra cimarrón
Indigenous names: Kokche'[09]
Used by (2*): Zoque[03]; Yucatecan Maya[09]
Used for (2#): Digestive (1)[03]; Respiratory (1)[09]
Cognates:
Language contact:

***Croton peraeruginosus* Croizat (Euphorbiaceae)**

Spanish names:
Indigenous names: Ek'balam, Xikm burro, Xikinch'omak[09]
Used by (1*): Yucatecan Maya[09]
Used for (1#): Skin (1)[09]
Cognates:
Language contact:

***Croton reflexifolius* Kunth (Euphorbiaceae)**

Spanish names:
Indigenous names: Oliy[07]; Pets'k'uts[09]
Used by (2*): Huastec[07]; Yucatecan Maya[09]
Used for (4#): Digestive (1)[07]; Neurological (1)[07]; Skin (2)[07, 09]
Cognates:
Language contact:

***Croton repens* Schltdl. (Euphorbiaceae)**

Spanish names: Agua dulce
Indigenous names: Copa n¥piñi cuy, Soj kobak, Soj muk[03]; Pop tsaxoj[04]
Used by (3*): Zoque[02, 03]; Mixe[04]
Used for (8#): Digestive (2)[03, 04]; Eye (1)[03]; Respiratory (1)[02]; Skin (1)[03]; Urological (1)[03]; Female genital (1)[03]; General and Unspecified (1)[03]
Cognates: MZ: soj;
Language contact:

***Croton schiedeanus* Schltdl. (Euphorbiaceae)**

Spanish names: Cascarilla
Indigenous names: Tam cuy[03]; Iri skutČ[10]; Copal chi'[17]
Used by (3*): Zoque[03]; Yucatecan Maya[10]; Quichean Maya[17]
Used for (9#): Blood (1)[10]; Digestive (1)[03]; Eye (1)[03]; Musculoskeletal (1)[03]; Neurological (1)[03]; Respiratory (1)[03]; Skin (1)[03]; Female genital (1)[03]; nd[17]
Cognates:
Language contact:

***Croton soliman* Cham. & Schltdl. (Euphorbiaceae)**

Spanish names: Mata pescado, hoja de mesquino
Indigenous names: Panii[04]; Luk, ist ts'ohool[07]
Used by (3*): Mixe[04]; Huastec[07]; Zapotec[21]
Used for (6#): Musculoskeletal (1)[07]; Neurological (1)[07]; Skin (3)[04, 07, 21]; General and Unspecified (1)[07]
Cognates:
Language contact:

[01-28]refer to the study codes in Table 4.1. *Total number of studies citing this taxon #Total number of use-records

***Croton* sp. (Euphorbiaceae)**

Spanish names: Contra
Indigenous names:
Used by (1*): Zoque[03]
Used for (1#): Digestive (1)[03]
Cognates:
Language contact:

***Croton trinitatis* Millsp. (Euphorbiaceae)**

Spanish names: Pata de paloma
Indigenous names:
Used by (1*): Zoque[02]
Used for (2#): Digestive (1)[02]; Urological (1)[02]
Cognates:
Language contact:

***Croton verapazensis* Donn.Sm. (Euphorbiaceae)**

Spanish names: Banquito
Indigenous names:
Used by (1*): Quichean Maya[13]
Used for (1#): Pregnancy (1)[13]
Cognates:
Language contact:

***Croton yucatanensis* Lundell (Euphorbiaceae)**

Spanish names:
Indigenous names: Sakpokche', Ik-haab[09]
Used by (1*): Yucatecan Maya[09]
Used for (1#): General and Unspecified (1)[09]
Cognates:
Language contact:

***Crusea calocephala* DC. (Rubiaceae)**

Spanish names:
Indigenous names: Uekx sotyi[03]
Used by (1*): Zoque[03]
Used for (1#): Skin (1)[03]
Cognates:
Language contact:

***Cryosophila stauracantha* (Heynh.) R.J.Evans (Arecaceae)**

Spanish names: Escoba
Indigenous names:
Used by (1*): Yucatecan Maya[10]
Used for (1#): Skin (1)[10]
Cognates:
Language contact:

***Ctenitis excelsa* (Desv.) Proctor (Dryopteridaceae)**

Spanish names: Cola de Piedra
Indigenous names: Q'uq'i Pek[14]
Used by (1*): Quichean Maya[14]
Used for (1#): General and Unspecified (1)[14]
Cognates:
Language contact:

***Ctenitis salvinii*** **(Baker) Stolze (Dryopteridaceae)**

Spanish names: Cola de Piedra
Indigenous names: Q'uq'i Pek[14]
Used by (1*): Quichean Maya[14]
Used for (1[#]): General and Unspecified (1)[14]
Cognates:
Language contact:

***Cucumis melo*** **L. (Cucurbitaceae)**

Spanish names: Melón
Indigenous names:
Used by (1*): Zoque[02]
Used for (3[#]): Cardiovascular (1)[02]; Endocrine (1)[02]; Urological (1)[02]
Cognates:
Language contact:

***Cucumis sativus*** **L. (Cucurbitaceae)**

Spanish names: Pepino
Indigenous names:
Used by (4*): Zoque[01, 02]; Quichean Maya[12]; Western Maya[18]
Used for (4[#]): Musculoskeletal (1)[01]; Skin (1)[18]; Urological (1)[02]; General and Unspecified (1)[12]
Cognates:
Language contact:

***Cucurbita argyrosperma*** **C.Huber (Cucurbitaceae)**

Spanish names: Ayote; Calabaza amarga
Indigenous names: Ratz'umi k'um [14]; Guedu laac[21]
Used by (2*): Quichean Maya[14]; Zapotec[21]
Used for (5[#]): Digestive (1)[14]; Skin (1)[21]; Urological (1)[21]; Male genital (1)[21]; General and Unspecified (1)[21]
Cognates:
Language contact:

***Cucurbita ficifolia*** **Bouché (Cucurbitaceae)**

Spanish names: Chilacayote
Indigenous names: Tsi'i[04]; Ka'ii[06]; Qo'q[13]; Mayil[20]
Used by (4*): Mixe[04]; Totonac[06]; Quichean Maya[13]; Western Maya[20]
Used for (4[#]): Digestive (1)[20]; Skin (1)[04]; Urological (1)[06]; Pregnancy (1)[13]
Cognates:
Language contact: Mixe <> Tot <> Tzeltalan <> Nah

***Cucurbita maxima*** **Duchesne (Cucurbitaceae)**

Spanish names: Calabaza
Indigenous names: Ch'um[18]
Used by (1*): Western Maya[18]
Used for (4[#]): Digestive (1)[18]; Skin (1)[18]; Endocrine (1)[18]; Pregnancy (1)[18]
Cognates:
Language contact:

***Cucurbita moschata*** **Duchesne (Cucurbitaceae)**

Spanish names: Ayote; Calabaza
Indigenous names: Ni'pxi[06]; Ch'um[19]
Used by (2*): Totonac[06]; Western Maya[19]
Used for (3[#]): Digestive (2)[06, 19]; General and Unspecified (1)[19]
Cognates:
Language contact:

[01-28]refer to the study codes in Table 4.1. *Total number of studies citing this taxon [#]Total number of use-records

***Cucurbita pepo* L. (Cucurbitaceae)**

Spanish names: Calabaza
Indigenous names: (Yama joma/nak) pa'sun[01]; Pa'so/une/ukum[02]; Maka ni'pxi[06]; Gueatu'u[21]; Giht[22]; Ayotli[28]
Used by (6*): Zoque[01, 02]; Totonac[06]; Zapotec[21, 22]; Nahua[28]
Used for (12#): Digestive (6)[01, 02, 06, 21, 22, 28]; Neurological (1)[02]; Skin (3)[01, 21, 28]; Endocrine (1)[01]; General and Unspecified (1)[21]
Cognates: Zoq: pa'su/pa'so;
Language contact:

***Cucurbita* sp. (Cucurbitaceae)**

Spanish names: Ayote; Calabaza
Indigenous names: Naspaasuñ[03]; Ts'oop[07]; Mukun[13]
Used by (3*): Zoque[03]; Huastec[07]; Quichean Maya[13]
Used for (4#): Blood (1)[13]; Digestive (1)[03]; Skin (1)[03]; General and Unspecified (1)[07]
Cognates:
Language contact:

***Cuminum cyminum* L. (Apiaceae)**

Spanish names: Comino
Indigenous names:
Used by (6*): Zoque[01, 02]; Totonac[06]; Quichean Maya[12, 13]; Zapotec[21]
Used for (10#): Digestive (3)[01, 12, 21]; Neurological (2)[06, 12]; Respiratory (2)[06, 21]; Pregnancy (2)[02, 13]; General and Unspecified (1)[13]
Cognates:
Language contact:

***Cunila polyantha* Benth. (Lamiaceae)**

Spanish names: Menta silvestre
Indigenous names: Cruz Q'os[12]
Used by (1*): Quichean Maya[12]
Used for (4#): Digestive (1)[12]; Psychological (1)[12]; Skin (1)[12]; General and Unspecified (1)[12]
Cognates:
Language contact:

***Cupania dentata* Moc. & Sessé ex DC. (Sapindaceae)**

Spanish names: Quebracho
Indigenous names: Akpak[03]
Used by (2*): Zoque[03]; Totonac[05]
Used for (4#): Digestive (1)[03]; Skin (1)[03]; Female genital (1)[03]; General and Unspecified (1)[05]
Cognates:
Language contact:

***Cuphea carthagenensis* (Jacq.) J.F.Macbr. (Lythraceae)**

Spanish names: Elotillo/malvarisco morado
Indigenous names: Mojko matsyi/ mojkuy mätzyik [01]; Q'anruhil Q'ehen[14]
Used by (2*): Zoque[01]; Quichean Maya[14]
Used for (6#): Blood (1)[01]; Digestive (1)[01]; Musculoskeletal (1)[01]; Psychological (1)[14]; Respiratory (1)[01]; Skin (1)[01]
Cognates:
Language contact:

***Cuphea decandra* Dryand. (Lythraceae)**

Spanish names:
Indigenous names: Arooz ts'ohool[07]
Used by (1*): Huastec[07]
Used for (1#): Skin (1)[07]
Cognates:
Language contact:

***Cuphea hyssopifolia* Kunth (Lythraceae)**

Spanish names: Escoba del rio/ de agua; Rosalito, kufeo, coralia
Indigenous names: Mesb'eel li ha', Xmes hi ha, Mes'uul ha'[14]
Used by (2*): Quichean Maya[13, 14]
Used for (6#): Blood (1)[14]; Digestive (2)[13, 14]; Psychological (1)[14]; Respiratory (1)[14]; General and Unspecified (1)[14]
Cognates:
Language contact:

***Cuphea nitidula* Kunth (Lythraceae)**

Spanish names: Hierba de venado
Indigenous names:
Used by (1*): Nahua[26]
Used for (1#): Digestive (1)[26]
Cognates:
Language contact:

***Cuphea parsonsia* (L.) R.Br. ex Steud. (Lythraceae)**

Spanish names: Comida de colibri
Indigenous names: Uwa' tzi'kin, uwa'tzunum, m'ax[13]
Used by (1*): Quichean Maya[13]
Used for (2#): Digestive (1)[13]; General and Unspecified (1)[13]
Cognates:
Language contact:

***Cuphea salicifolia* Schltdl. & Cham. (Lythraceae)**

Spanish names:
Indigenous names:
Used by (1*): Huastec[07]
Used for (1#): General and Unspecified (1)[07]
Cognates:
Language contact:

***Cuphea* sp. (Lythraceae)**

Spanish names: Mirto
Indigenous names:
Used by (1*): Nahua[26]
Used for (3#): Digestive (1)[26]; Skin (1)[26]; Female genital (1)[26]
Cognates:
Language contact:

***Cuphea tetrapetala* Koehne (Lythraceae)**

Spanish names:
Indigenous names: N¥ang s¥gay[03]
Used by (1*): Zoque[03]
Used for (3#): Neurological (1)[03]; Skin (1)[03]; General and Unspecified (1)[03]
Cognates:
Language contact:

***Cuphea utriculosa* Koehne (Lythraceae)**

Spanish names: Sanguinaria
Indigenous names:
Used by (1*): Zoque[01]
Used for (2#): Cardiovascular (1)[01]; Neurological (1)[01]
Cognates:
Language contact:

[01-28]refer to the study codes in Table 4.1. *Total number of studies citing this taxon #Total number of use-records

**_Cupressus lusitanica_ Mill. (Cupressaceae)**

Spanish names: Ciprés
Indigenous names: K'isis[12]; K'sis[13]; Xnaq' Cipres [14]
Used by (4*): Zoque[01]; Quichean Maya[12, 13, 14]
Used for (16#): Cardiovascular (1)[12]; Musculoskeletal (3)[01, 12, 13]; Neurological (3)[12, 13, 14]; Respiratory (2)[12, 13]; Skin (2)[01, 13]; Urological (1)[01]; Pregnancy (1)[12]; Female genital (1)[12]; General and Unspecified (2)[01, 12]
Cognates: Quich: k'(i)sis;
Language contact:

**_Curatella americana_ L. (Dilleniaceae)**

Spanish names: Lengua de vaca; Tachicón
Indigenous names: Wakas yenkuy[02]; Potcuy[03]; Xø popt lijar[04]; Balaga lujtza yussu'[21]
Used by (4*): Zoque[02, 03]; Mixe[04]; Zapotec[21]
Used for (8#): Digestive (1)[03]; Musculoskeletal (2)[02, 04]; Skin (2)[03, 04]; Female genital (1)[03]; General and Unspecified (2)[04, 21]
Cognates: MZ: pot;
Language contact:

**_Curcuma longa_ L. (Zingiberaceae)**

Spanish names: Curcuma
Indigenous names: Azafraan[07]
Used by (3*): Zoque[01]; Huastec[07]; Quichean Maya[12]
Used for (6#): Digestive (2)[01, 12]; Musculoskeletal (1)[07]; Psychological (1)[12]; Respiratory (1)[07]; Skin (1)[12]
Cognates:
Language contact:

**_Cuscuta corymbosa_ Ruiz & Pav. (Convolvulaceae)**

Spanish names: Barba del Diablo; Fideos, cordoncillo
Indigenous names: Q'en Sumachi' Aq'om[12]
Used by (2*): Quichean Maya[12]; Nahua[25]
Used for (4#): Endocrine (1)[25]; Urological (2)[12, 25]; General and Unspecified (1)[12]
Cognates:
Language contact:

**_Cuscuta_ sp. (Convolvulaceae)**

Spanish names: Cardenillo, cuernas del diablo; Tripa de gallina; Zacapal
Indigenous names: Puutx tsay[03]; Snuku[05]; Fideo ts'ohool, man ch'aah[07]
Used by (5*): Zoque[03]; Totonac[05]; Huastec[07]; Quichean Maya[13]; Nahua[26]
Used for (10#): Blood (1)[05]; Digestive (2)[03, 05]; Neurological (1)[07]; Skin (3)[07, 13, 26]; Urological (1)[03]; General and Unspecified (2)[03, 07]
Cognates:
Language contact:

**_Cuscuta tinctoria_ Mart. ex Engelm. (Convolvulaceae)**

Spanish names: Sopa de fideo, cardenillo
Indigenous names: Tza'a tane/ pu'utze [01]; Zacapactle[26]
Used by (2*): Zoque[01]; Nahua[26]
Used for (2#): Skin (1)[26]; General and Unspecified (1)[01]
Cognates:
Language contact:

***Cyathea costaricensis* (Mett. ex Kuhn) Domin (Cyatheaceae)**

Spanish names: Palo de vibora/mano de leon/cola de mono/helecho macho
Indigenous names: Kan nak'e /tzawi tutz/ tzimui[01]
Used by (1*): Zoque[01]
Used for (4#): Digestive (1)[01]; Endocrine (1)[01]; Urological (1)[01]; General and Unspecified (1)[01]
Cognates:
Language contact:

***Cyathea microdonta* (Desv.) Domin (Cyatheaceae)**

Spanish names: Palo de culebra
Indigenous names:
Used by (1*): Zoque[01]
Used for (1#): Endocrine (1)[01]
Cognates:
Language contact:

***Cyathea myosuroides* (Liebm.) Domin (Cyatheaceae)**

Spanish names: Cola de caballo; Colandrillo, cola de chango
Indigenous names: Kinä näpin/suyi suki pung [02]; Shgola miigu'u[21]
Used by (2*): Zoque[02]; Zapotec[21]
Used for (7#): Digestive (2)[02, 21]; Eye (1)[02]; Cardiovascular (1)[02]; Endocrine (1)[02]; Urological (1)[02]; General and Unspecified (1)[02]
Cognates:
Language contact:

***Cyclanthera dissecta* (Torr. & A.Gray) Arn. (Cucurbitaceae)**

Spanish names:
Indigenous names: Chunak Q'en[14]
Used by (1*): Quichean Maya[14]
Used for (1#): Skin (1)[14]
Cognates:
Language contact:

***Cyclospermum leptophyllum* (Pers.) Sprague (Apiaceae)**

Spanish names:
Indigenous names: Kulantoil an t'ot[07]
Used by (2*): Huastec[07]; Western Maya[20]
Used for (2#): Digestive (1)[20]; Respiratory (1)[07]
Cognates:
Language contact:

***Cydonia oblonga* Mill. (Rosaceae)**

Spanish names: Membrillo
Indigenous names: Yàg-mèmbrî[23]
Used by (3*): Quichean Maya[12, 13]; Zapotec[23]
Used for (8#): Digestive (2)[13, 23]; Cardiovascular (1)[12]; Musculoskeletal (1)[12]; Neurological (1)[12]; Skin (1)[12]; Urological (1)[12]; General and Unspecified (1)[12]
Cognates:
Language contact:

[01-28]refer to the study codes in Table 4.1. *Total number of studies citing this taxon #Total number of use-records

***Cymbopetalum penduliflorum* (Dunal) Baill. (Annonaceae)**

Spanish names: Orifela
Indigenous names:
Used by (1*): Zoque[02]
Used for (5#): Digestive (1)[02]; Ear (1)[02]; Neurological (1)[02]; Respiratory (1)[02]; Female genital (1)[02]
Cognates:
Language contact:

***Cymbopetalum* sp. (Annonaceae)**

Spanish names: Oreja de gato
Indigenous names: Yagamishu'u[21]
Used by (1*): Zapotec[21]
Used for (3#): Musculoskeletal (1)[21]; Neurological (1)[21]; Respiratory (1)[21]
Cognates:
Language contact:

***Cymbopetalum stenophyllum* Don.Sm. (Annonaceae)**

Spanish names: Orifela
Indigenous names:
Used by (1*): Zoque[01]
Used for (1#): Psychological (1)[01]
Cognates:
Language contact:

***Cymbopogon citratus* (DC.) Stapf (Poaceae)**

Spanish names: Zacate limón, telimón, té de limón
Indigenous names: Paja'k wäjpajk ay [02]; Limunmuk[03]; Limoon toom[07]; Q'is Q'im [14]; Bänälä limón[18]; Sansiwre wamal[20]; Te guisa[21]
Used by (18*): Zoque[01, 02, 03]; Mixe[04]; Totonac[05, 06]; Huastec[07]; Yucatecan Maya[09, 10, 11]; Quichean Maya[12, 13, 14]; Western Maya[18, 19, 20]; Zapotec[21]; Nahua[25]
Used for (52#): Blood (1)[12]; Digestive (10)[01, 03, 04, 05, 07, 09, 12, 18, 20, 21]; Cardiovascular (5)[02, 05, 12, 13, 14]; Musculoskeletal (2)[13, 18]; Neurological (2)[01, 13]; Psychological (5)[01, 02, 12, 18, 21]; Respiratory (10)[01, 02, 03, 09, 11, 12, 13, 18, 19, 25]; Endocrine (2)[02, 12]; Urological (2) [02, 07]; Pregnancy (3)[06, 13, 18]; Female genital (3)[01, 12, 13]; General and Unspecified (7)[01, 10, 11, 12, 13, 18, 21]
Cognates:
Language contact:

***Cyperus articulatus* L. (Cyperaceae)**

Spanish names: Chantulli; Chintul
Indigenous names: Tupux[09]; Shapandú[21]
Used by (3*): Mixe[04]; Yucatecan Maya[09]; Zapotec[21]
Used for (8#): Musculoskeletal (1)[21]; Neurological (2)[04, 21]; Respiratory (2)[04, 09]; Skin (2)[04, 21]; General and Unspecified (1)[21]
Cognates:
Language contact:

***Cyperus compressus* L. (Cyperaceae)**

Spanish names:
Indigenous names: Poopil Q'ehen[14]
Used by (1*): Quichean Maya[14]
Used for (1#): General and Unspecified (1)[14]
Cognates:
Language contact:

***Cyperus hermaphroditus* (Jacq.) Standl. (Cyperaceae)**

Spanish names: Cituli; Piomía
Indigenous names: Haluk'laab ts'ohool, tsakam tsaw, tathim toom[07]; Tzajal yisim be[20]
Used by (4*): Zoque[02]; Huastec[07]; Western Maya[20]; Nahua[27]
Used for (9#): Digestive (3)[07, 20, 27]; Respiratory (1)[02]; Skin (2)[02, 07]; Urological (1)[07]; General and Unspecified (2)[07, 27]
Cognates:
Language contact: Hua <> Tzeltalan

***Cyperus laxus* Lam. (Cyperaceae)**

Spanish names: Cintule
Indigenous names:
Used by (1*): Zoque[02]
Used for (1#): Skin (1)[02]
Cognates:
Language contact:

***Cyperus odoratus* L. (Cyperaceae)**

Spanish names:
Indigenous names: Pioninu, Puutx uixpin[03]
Used by (1*): Zoque[03]
Used for (1#): Psychological (1)[03]
Cognates:
Language contact:

***Cyperus* sp. (Cyperaceae)**

Spanish names: Grama; Grama Silvestre; Zacate
Indigenous names: Sabana Aq'om[12]; K'im[13]
Used by (3*): Quichean Maya[12, 13]; Zapotec[21]
Used for (7#): Blood (1)[12]; Digestive (1)[12]; Neurological (1)[12]; Urological (1)[12]; Pregnancy (2)[13, 21]; General and Unspecified (1)[21]
Cognates:
Language contact:

***Cyrtocarpa edulis* (Brandegee) Standl. (Anacardiaceae)**

Spanish names: Ciruelo
Indigenous names: K'inim te'[08]
Used by (1*): Huastec[08]
Used for (4#): Digestive (1)[08]; Neurological (1)[08]; Skin (1)[08]; General and Unspecified (1)[08]
Cognates:
Language contact:

***Cyrtocarpa procera* Kunth (Anacardiaceae)**

Spanish names: Chupandilla
Indigenous names:
Used by (1*): Nahua[27]
Used for (1#): Urological (1)[27]
Cognates:
Language contact:

***Cyrtocymura scorpioides* (Lam.) H.Rob. (Asteraceae)**

Spanish names: Tzitit
Indigenous names: Tsisykuy[01]
Used by (1*): Zoque[01]
Used for (2#): Neurological (1)[01]; Respiratory (1)[01]
Cognates:
Language contact:

***Dahlia imperialis*** **Roezl ex Ortgies (Asteraceae)**

Spanish names: Santa Catarina; Tunay
Indigenous names: Tney, tunay, oi[13]; Ch'olip, cho'liv, ch'olov[20]
Used by (3*): Quichean Maya[12, 13]; Western Maya[20]
Used for (18[#]): Digestive (3)[12, 13, 20]; Eye (1)[13]; Ear (2)[13, 20]; Cardiovascular (1)[12]; Neurological (2)[13, 20]; Psychological (1)[13]; Respiratory (1)[20]; Skin (2)[12, 13]; Urological (2)[12, 13]; General and Unspecified (3)[12, 13, 20]
Cognates:
Language contact: K'iche' <> Tzeltalan

***Dalbergia granadillo*** **Pittie (Fabaceae)**

Spanish names: Guayacan
Indigenous names:
Used by (1*): Zapotec[21]
Used for (3[#]): Musculoskeletal (1)[21]; Female genital (1)[21]; General and Unspecified (1)[21]
Cognates:
Language contact:

***Dalea carthagenensis (*****Jacq.) J.F.Macbr. (Fabaceae)**

Spanish names:
Indigenous names: Azúfre xiw, Suyk'ak'[09]
Used by (1*): Yucatecan Maya[09]
Used for (1[#]): Skin (1)[09]
Cognates:
Language contact:

***Dalea foliolosa*** **(Aiton) Barneby (Fabaceae)**

Spanish names: Toronjíl
Indigenous names: Nlít-quiè,  guìzh-nlít-quiè[23]
Used by (1*): Zapotec[23]
Used for (1[#]): Digestive (1)[23]
Cognates:
Language contact:

***Dalea lutea*** **(Cav.) Willd. (Fabaceae)**

Spanish names:
Indigenous names: Tsakam chilab[07]
Used by (1*): Huastec[07]
Used for (1[#]): General and Unspecified (1)[07]
Cognates:
Language contact:

***Dalea scandens*** **(Mill.) R.T.Clausen (Fabaceae)**

Spanish names:
Indigenous names: Tsun tsun[03]; Tsakam chilab[07]
Used by (2*): Zoque[03]; Huastec[07]
Used for (7[#]): Blood (1)[07]; Digestive (1)[03]; Ear (1)[07]; Musculoskeletal (1)[07]; Pregnancy (1)[03]; General and Unspecified (2)[03, 07]
Cognates:
Language contact:

***Dalea tomentosa*** **(Cav.) Willd. (Fabaceae)**

Spanish names: Quebrapiedra
Indigenous names:
Used by (1*): Zoque[01]
Used for (1[#]): Urological (1)[01]
Cognates:
Language contact:

[01-28]refer to the study codes in Table 4.1. *Total number of studies citing this taxon [#]Total number of use-records

***Dalechampia scandens* L. (Euphorbiaceae)**

Spanish names:
Indigenous names: Kw'aat', la'ix iits', tithelom ch'ohool[07]
Used by (1*): Huastec[07]
Used for (1#): Neurological (1)[07]
Cognates:
Language contact:

***Danaea* sp. (Marattiaceae)**

Spanish names:
Indigenous names:
Used by (1*): Quichean Maya[17]
Used for (1#): nd[17]
Cognates:
Language contact:

***Datura innoxia* Mill. (Solanaceae)**

Spanish names: Toloache
Indigenous names: Campana pøh[04]; Chaniko, Chamisa[09]
Used by (3*): Zoque[02]; Mixe[04]; Yucatecan Maya[09]
Used for (8#): Digestive (1)[02]; Eye (1)[02]; Musculoskeletal (2)[02, 04]; Skin (2)[02, 09]; Female genital (1)[02]; General and Unspecified (1)[04]
Cognates:
Language contact:

***Datura metel* L. (Solanaceae)**

Spanish names: Toloache morado
Indigenous names: Buuruj'hui moradu[21]
Used by (1*): Zapotec[21]
Used for (4#): Digestive (1)[21]; Musculoskeletal (1)[21]; Skin (1)[21]; General and Unspecified (1)[21]
Cognates:
Language contact:

***Datura stramonium* L. (Solanaceae)**

Spanish names: Toloache
Indigenous names: Matunu[03]; San pedro ujts[04]; Thanab[07]; Mbuuruj'hui[21]; Blàg-rzûdz, guìzh-rzûdz[23]
Used by (9*): Zoque[01, 03]; Mixe[04]; Huastec[07]; Yucatecan Maya[10]; Quichean Maya[13]; Western Maya[18]; Zapotec[21, 23]
Used for (23#): Digestive (4)[03, 13, 18, 21]; Cardiovascular (1)[18]; Musculoskeletal (2)[21, 23]; Neurological (1)[13]; Psychological (2)[13, 21]; Skin (5)[01, 04, 07, 21, 23]; Female genital (3)[03, 07, 21]; General and Unspecified (5)[03, 07, 10, 21, 23]
Cognates: Zapo: ru;
Language contact:

***Daucus carota* L. (Apiaceae)**

Spanish names: Zanahoria
Indigenous names:
Used by (2*): Zoque[02]; Quichean Maya[12]
Used for (7#): Digestive (1)[12]; Eye (1)[12]; Musculoskeletal (1)[12]; Psychological (1)[12]; Skin (1)[12]; Urological (1)[02]; General and Unspecified (1)[02]
Cognates:
Language contact:

***Daucus montanus*** **Humb. & Bonpl. ex Schult. (Apiaceae)**

Spanish names:
Indigenous names:
Used by (1*): Zoque[01]
Used for (1#): Digestive (1)[01]
Cognates:
Language contact:

***Davilla kunthii*** **A.St.-Hil. (Dilleniaceae)**

Spanish names:
Indigenous names: Potcuy tsay[03]; Kak'i k'aham[17]
Used by (2*): Zoque[03]; Quichean Maya[17]
Used for (2#): Digestive (1)[03]; nd[17]
Cognates:
Language contact:

***Decachaeta incompta*** **(DC.) R.M.King & H.Rob. (Asteraceae)**

Spanish names: Tabacco cimarrón
Indigenous names: Cimarrón ts¥ui[03]
Used by (1*): Zoque[03]
Used for (2#): Urological (1)[03]; Female genital (1)[03]
Cognates:
Language contact:

***Decatropis bicolor*** **(Zucc.) Radlk. (Rutaceae)**

Spanish names:
Indigenous names: Bichaam te'[07]
Used by (1*): Huastec[07]
Used for (3#): Digestive (1)[07]; Musculoskeletal (1)[07]; General and Unspecified (1)[07]
Cognates:
Language contact:

***Dendropanax arboreus*** **(L.) Decne. & Planch. (Araliaceae)**

Spanish names: Palo de agua
Indigenous names: Un cuy[03]; Multe'[07]; Cojl[17]
Used by (3*): Zoque[03]; Huastec[07]; Quichean Maya[17]
Used for (13#): Eye (1)[03]; Ear (1)[03]; Musculoskeletal (1)[07]; Neurological (2)[03, 07]; Psychological (2)[03, 07]; Respiratory (1)[07]; Skin (2)[03, 07]; General and Unspecified (2)[03, 07]; nd[17]
Cognates:
Language contact:

***Deppea*** **sp. (Rubiaceae)**

Spanish names:
Indigenous names:
Used by (1*): Zoque[03]
Used for (1#): General and Unspecified (1)[03]
Cognates:
Language contact:

***Desmanthus virgatus*** **(L.) Willd. (Fabaceae)**

Spanish names: Guajillo
Indigenous names: Salat-ik', Sibik'xiw, Sib-ik'', Sik'ink'ax[09]; Lya las[22]
Used by (2*): Yucatecan Maya[09]; Zapotec[22]
Used for (2#): Digestive (1)[22]; Eye (1)[09]
Cognates:
Language contact:

***Desmodium adscendens* (Sw.) DC. (Fabaceae)**

Spanish names:
Indigenous names: Ch'in pim[17]
Used by (1*): Quichean Maya[17]
Used for (1#): nd[17]
Cognates:
Language contact:

***Desmodium axillare* (Sw.) DC. (Fabaceae)**

Spanish names: Pegapega
Indigenous names: Ts'at' ts'ohool, t'apay thekw'eel[07]
Used by (2*): Zoque[02]; Huastec[07]
Used for (4#): Skin (2)[02, 07]; Urological (1)[02]; Pregnancy (1)[02]
Cognates:
Language contact:

***Desmodium incanum* DC. (Fabaceae)**

Spanish names: Cadillo rojo
Indigenous names: T¥ pitx nang tsang[03]; Ujts jayi pekpa[04]; T'apay thekw'eel, xutsun t'eel, bokoolil an t'eel, bakanil t'eel, anuch kw'a'[07]; Chim pim[14]
Used by (4*): Zoque[03]; Mixe[04]; Huastec[07]; Quichean Maya[14]
Used for (8#): Digestive (2)[03, 07]; Skin (2)[03, 07]; Urological (1)[04]; Pregnancy (2)[03, 14]; General and Unspecified (1)[14]
Cognates:
Language contact: Highland Popoluca <-> Hua

***Desmodium* sp. (Fabaceae)**

Spanish names:
Indigenous names: Pak'umpak'[09]; Chim pim[14]
Used by (2*): Yucatecan Maya[09]; Quichean Maya[14]
Used for (2#): Musculoskeletal (1)[14]; Skin (1)[09]
Cognates:
Language contact:

***Dialium guianense* (Aubl.) Sandwith (Fabaceae)**

Spanish names:
Indigenous names: T¥m paki[03]
Used by (1*): Zoque[03]
Used for (3#): Digestive (1)[03]; Pregnancy (1)[03]; General and Unspecified (1)[03]
Cognates:
Language contact:

***Dichondra* sp. (Convolvulaceae)**

Spanish names:
Indigenous names: Ahuaxmama[26]
Used by (1*): Nahua[26]
Used for (1#): Skin (1)[26]
Cognates:
Language contact:

***Dichorisandra hexandra* (Aubl.) Standl. (Commelinaceae)**

Spanish names:
Indigenous names: Tzima'j pim[17]
Used by (1*): Quichean Maya[17]
Used for (1#): nd[17]
Cognates:
Language contact:

[01-28]refer to the study codes in Table 4.1. *Total number of studies citing this taxon #Total number of use-records

***Dicliptera sexangularis* (L.) Juss. (Acanthaceae)**

Spanish names:
Indigenous names: Uxkwe' ts'ohool, wits paya'[07]
Used by (1*): Huastec[07]
Used for (2#): Female genital (1)[07]; General and Unspecified (1)[07]
Cognates:
Language contact:

***Dicliptera* sp. (Acanthaceae)**

Spanish names:
Indigenous names: Tza' Tzalun Mi' Ha'[14]
Used by (1*): Quichean Maya[14]
Used for (1#): General and Unspecified (1)[14]
Cognates:
Language contact:

***Dictyanthus yucatanensis* Standl. (Apocynaceae)**

Spanish names:
Indigenous names: Ensul, Emtsul[09]
Used by (1*): Yucatecan Maya[09]
Used for (1#): General and Unspecified (1)[09]
Cognates:
Language contact:

***Dictyoxiphium panamense* Hook. (Tectariaceae)**

Spanish names:
Indigenous names: Usi xul k'ejen[17]
Used by (1*): Quichean Maya[17]
Used for (1#): nd[17]
Cognates:
Language contact:

***Didymaea microphylla* L.O.Williams (Rubiaceae)**

Spanish names: Pegapega
Indigenous names: Ajak Ruk'amal Ibo'ch Aq'om[12]
Used by (1*): Quichean Maya[12]
Used for (4#): Digestive (1)[12]; Cardiovascular (1)[12]; Musculoskeletal (1)[12]; Neurological (1)[12]
Cognates:
Language contact:

***Digitalis purpurea* L. (Plantaginaceae)**

Spanish names: Digital
Indigenous names:
Used by (1*): Quichean Maya[12]
Used for (3#): Cardiovascular (1)[12]; Musculoskeletal (1)[12]; Endocrine (1)[12]
Cognates:
Language contact:

***Dioscorea alata* L. (Dioscoreaceae)**

Spanish names:
Indigenous names: Laab ith[07]
Used by (1*): Huastec[07]
Used for (1#): Urological (1)[07]
Cognates:
Language contact:

***Dioscorea bartlettii*** **C.V.Morton (Dioscoreaceae)**

Spanish names: Cocolmeca
Indigenous names: Xchup ichim qotz[15]
Used by (1*): Quichean Maya[15]
Used for (1#): Blood (1)[15]
Cognates:
Language contact:

***Dioscorea composita*** **Hemsl. (Dioscoreaceae)**

Spanish names: Barbasco; Barbasco, calabacilla; Ñame
Indigenous names: Manat[05]
Used by (4*): Mixe[04]; Totonac[05]; Western Maya[18]; Nahua[26]
Used for (6#): Digestive (1)[26]; Musculoskeletal (2)[04, 05]; Pregnancy (1)[26]; Female genital (1)[26]; General and Unspecified (1)[18]
Cognates:
Language contact:

***Dioscorea convolvulacea*** **Cham. & Schltdl. (Dioscoreaceae)**

Spanish names:
Indigenous names: P'ujk'[20]
Used by (1*): Western Maya[20]
Used for (1#): Digestive (1)[20]
Cognates:
Language contact:

***Dioscorea floribunda*** **M.Martens & Galeotti (Dioscoreaceae)**

Spanish names: Barbasco; Barbasco Amarillo
Indigenous names: Puutx naaku[03]; Ganabigujchii[21]
Used by (3*): Zoque[02, 03]; Zapotec[21]
Used for (6#): Musculoskeletal (2)[03, 21]; Skin (1)[02]; Female genital (1)[21]; General and Unspecified (2)[02, 21]
Cognates:
Language contact: Highland Popoluca <> Zap

***Dioscorea martiana*** **Griseb. (Dioscoreaceae)**

Spanish names: Barbasco
Indigenous names:
Used by (1*): Mixe[04]
Used for (1#): Musculoskeletal (1)[04]
Cognates:
Language contact:

***Dioscorea mexicana*** **Scheidw. (Dioscoreaceae)**

Spanish names: Tumba vaquero
Indigenous names: Tumi tätz[01]; Panil book, ix thuyum[07]
Used by (2*): Zoque[01]; Huastec[07]
Used for (6#): Cardiovascular (1)[01]; Musculoskeletal (1)[01]; Psychological (1)[07]; Urological (1)[01]; Pregnancy (1)[07]; Male genital (1)[01]
Cognates:
Language contact:

***Dioscorea*** **sp. (Dioscoreaceae)**

Spanish names: Cuculmeca
Indigenous names:
Used by (1*): Western Maya[19]
Used for (2#): Pregnancy (1)[19]; Female genital (1)[19]
Cognates:
Language contact:

[01-28] refer to the study codes in Table 4.1. *Total number of studies citing this taxon #Total number of use-records

***Dioscorea spiculiflora* Hemsl. (Dioscoreaceae)**

Spanish names:
Indigenous names: Cancer-ak', Wil-ak'[09]
Used by (1*): Yucatecan Maya[09]
Used for (1#): Skin (1)[09]
Cognates:
Language contact:

***Diospyros acapulcensis* Kunth (Ebenaceae)**

Spanish names: Tachona
Indigenous names: Ndxuuli'i[21]
Used by (1*): Zapotec[21]
Used for (1#): General and Unspecified (1)[21]
Cognates:
Language contact:

***Diospyros anisandra* S.F.Blake (Ebenaceae)**

Spanish names:
Indigenous names: Xkakalche'[09]
Used by (1*): Yucatecan Maya[09]
Used for (1#): Skin (1)[09]
Cognates:
Language contact:

***Diospyros nigra* (J.F.Gmel.) Perrier (Ebenaceae)**

Spanish names: Zapote negro
Indigenous names: Nuu[03]; Sawat[05]; Suua'l[06]; Munek'[07]; Bila'huaj[21]
Used by (5*): Zoque[03]; Totonac[05, 06]; Huastec[07]; Zapotec[21]
Used for (11#): Digestive (2)[05, 06]; Eye (1)[06]; Respiratory (1)[06]; Skin (2)[03, 21]; Female genital (1)[03]; General and Unspecified (4)[03, 06, 07, 21]
Cognates: Toto: sVwa;
Language contact: Tot <> Zap

***Diospyros tetrasperma* Sw. (Ebenaceae)**

Spanish names:
Indigenous names: Sibil[09]
Used by (1*): Yucatecan Maya[09]
Used for (1#): Skin (1)[09]
Cognates:
Language contact:

***Diphysa americana* (Mill.) M.Sousa (Fabaceae)**

Spanish names: Chipilcoite; Chipile; Guachipillín; Ruda cimarron
Indigenous names: Tzus kuy [01]; Tsus cuy[03]; Chichath, k'anaw te, tsitsab te'[07]; Ikuy[12]
Used by (5*): Zoque[01, 03]; Huastec[07]; Quichean Maya[12]; Zapotec[21]
Used for (16#): Blood (1)[07]; Digestive (2)[03, 07]; Musculoskeletal (2)[12, 21]; Neurological (1)[07]; Skin (2)[03, 07]; Endocrine (1)[12]; Urological (1)[07]; Pregnancy (1)[07]; General and Unspecified (5)[01, 03, 07, 12, 21]
Cognates: Zoq: tsuskuy;
Language contact: Zoq > Hua and Kaqchickel

***Diphysa carthagenensis* Jacq. (Fabaceae)**

Spanish names: Ruda de monte
Indigenous names: Tsuxp[04]; Susup, Ts'us'uk[09]; Ču Čuk[10]
Used by (3*): Mixe[04]; Yucatecan Maya[09, 10]
Used for (4#): Digestive (1)[09]; Skin (2)[04, 09]; General and Unspecified (1)[10]
Cognates:
Language contact: Mixe <> Yuc

***Diphysa floribunda* Peyr. (Fabaceae)**

Spanish names: Guachipillin
Indigenous names: Tzus kuy [01]; Chipilin Aq'om, Uku'j[12]
Used by (2*): Zoque[01]; Quichean Maya[12]
Used for (14#): Digestive (1)[01]; Ear (1)[01]; Cardiovascular (1)[12]; Musculoskeletal (2)[12, 12]; Neurological (2)[01, 12]; Psychological (1)[12]; Respiratory (1)[01]; Skin (1)[01]; Urological (1)[01]; Social problems (1)[01]; General and Unspecified (2)[01, 12]
Cognates:
Language contact:

***Disciphania calocarpa* Standl. (Menispermaceae)**

Spanish names: Bejuco real, Istamoreal
Indigenous names:
Used by (1*): Western Maya[18]
Used for (1#): Digestive (1)[18]
Cognates:
Language contact:

***Discocnide mexicana* (Liebm.) Chew (Urticaceae)**

Spanish names: Ortiga mayor
Indigenous names: Menuk/ kenuk[01]; Balagadena, lagui[21]
Used by (2*): Zoque[01]; Zapotec[21]
Used for (3#): Musculoskeletal (2)[01, 21]; Respiratory (1)[21]
Cognates:
Language contact:

***Disocactus martianus (*Zucc. ex Pfeiff.) Barthlott (Cactaceae)**

Spanish names:
Indigenous names: Brün-yăl-guièts, yàg-bdzì-lbæ̀, yàg-bdzì-làs[23]
Used by (1*): Zapotec[23]
Used for (1#): General and Unspecified (1)[23]
Cognates:
Language contact:

***Dodonaea viscosa* (L.) Jacq. (Sapindaceae)**

Spanish names: Jaras; Jarilla; Pimientillo
Indigenous names: Yahg žij[22]; Yàg-blàg-bîdz[23]
Used by (3*): Zoque[01]; Zapotec[22, 23]
Used for (8#): Digestive (1)[23]; Ear (1)[23]; Musculoskeletal (2)[22, 23]; Pregnancy (1)[01]; General and Unspecified (3)[01, 22, 23]
Cognates: Zapo: ich;
Language contact:

***Dolichandra quadrivalvis (*Jacq.) L.G.Lohmann (Bignoniaceae)**

Spanish names:
Indigenous names: Oohoox ts'aah. Itsik' ocho', kuxkum ts'aah[07]
Used by (1*): Huastec[07]
Used for (1#): Psychological (1)[07]
Cognates:
Language contact:

[01-28]refer to the study codes in Table 4.1. *Total number of studies citing this taxon #Total number of use-records

***Dolichandra unguis-cati* (L.) L.G.Lohmann (Bignoniaceae)**

Spanish names: Murciélago/bejuco de chinaco
Indigenous names: Täsi[01]
Used by (1*): Zoque[01]
Used for (2#): Cardiovascular (1)[01]; Musculoskeletal (1)[01]
Cognates:
Language contact:

***Doliocarpus dentatus* (Aubl.) Standl. (Dilleniaceae)**

Spanish names:
Indigenous names: Sun tsay[03]; Xø popt aats[04]
Used by (2*): Zoque[03]; Mixe[04]
Used for (5#): Digestive (1)[03]; Musculoskeletal (1)[04]; Skin (1)[04]; Female genital (1)[03]; General and Unspecified (1)[04]
Cognates:
Language contact:

***Dorstenia contrajerva* L. (Moraceae)**

Spanish names: Contrayerba, cresta de gallo, hoja de sapo
Indigenous names: Tzutz kin[01]; Tapu ay[02]; Nak ay[03]; K'ubak kw'a', itsal kw'a'[07]; Xkambalhaw[09]; K'opopo'il Q'ehen [14]; Ch'up i ai[17]
Used by (9*): Zoque[01, 02, 03]; Huastec[07]; Yucatecan Maya[09, 11]; Quichean Maya[14, 17]; Western Maya[18]
Used for (19#): Digestive (3)[07, 09, 11]; Ear (1)[03]; Musculoskeletal (2)[03, 07]; Neurological (1)[07]; Skin (5)[01, 02, 03, 14, 18]; Urological (1) [03]; Pregnancy (2)[07, 09]; Female genital (1)[03]; General and Unspecified (2)[07, 14]; nd[17]
Cognates:
Language contact:

***Dorstenia drakena* L. (Moraceae)**

Spanish names: Contrayerba
Indigenous names: Amtz'ak[19]; Yanayu'u[21]
Used by (2*): Western Maya[19]; Zapotec[21]
Used for (4#): Digestive (2)[19, 21]; Musculoskeletal (1)[21]; General and Unspecified (1)[21]
Cognates:
Language contact:

***Dorstenia lindeniana* Bureau (Moraceae)**

Spanish names: Mal de Sapo
Indigenous names: Xtulihom' K'opopo, K'urux Ixox[14]
Used by (1*): Quichean Maya[14]
Used for (2#): Skin (1)[14]; General and Unspecified (1)[14]
Cognates:
Language contact:

***Drimys winteri* J.R.Forst. & G.Forst. (Winteraceae)**

Spanish names:
Indigenous names: Canela y cuyo[09]
Used by (1*): Yucatecan Maya[09]
Used for (1#): Digestive (1)[09]
Cognates:
Language contact:

[01-28]refer to the study codes in Table 4.1. *Total number of studies citing this taxon #Total number of use-records

***Duranta erecta* L. (Verbenaceae)**

Spanish names:
Indigenous names: K'anpokolche'[09]
Used by (1*): Yucatecan Maya[09]
Used for (1#): Skin (1)[09]
Cognates:
Language contact:

***Dysphania ambrosioides* (L.) Mosyakin & Clemants (Amaranthaceae)**

Spanish names: Epazote
Indigenous names: Onyuk/Tzig tuna/ unyuk[01]; Kisawa/kisyawa[02]; Epazut[03]; Podeey[04]; Sa'kalhka'jna'[05]; Sa 'kha 'kalx 'na[06]; Tihtsan[07]; Tijson[08]; Lukumxiw[09]; Sik'ij[12]; Sik'aj[13]; Cha'jen binilaj[18]; Pasujt[19]; Kakan, koko'on, kokon', kaka'an, kajk'an[20]; Biajta'[21]; Bitia'[22]; Ptiè[23]; Epazote[25]; Epazotl[28]
Used by (23*): Zoque[01, 02, 03]; Mixe[04]; Totonac[05, 06]; Huastec[07, 08]; Yucatecan Maya[09, 10, 11]; Quichean Maya[12, 13, 14]; Western Maya[18, 19, 20]; Zapotec[21, 22, 23]; Nahua[25, 26, 28]
Used for (62#): Digestive (22)[01, 02, 03, 04, 05, 06, 07, 08, 09, 10, 11, 12, 13, 14, 18, 19, 20, 21, 23, 25, 26, 28]; Eye (1)[21]; Cardiovascular (1)[12]; Musculoskeletal (2)[01, 08]; Neurological (3)[02, 03, 12]; Psychological (2)[13, 22]; Respiratory (5)[02, 05, 12, 21, 28]; Skin (10)[01, 07, 11, 12, 13, 18, 19, 20, 21, 25]; Urological (1)[02]; Pregnancy (6)[05, 06, 07, 12, 13, 19]; Female genital (4)[05, 12, 14, 19]; General and Unspecified (5)[01, 05, 08, 12, 21]
Cognates: Toto: sa'kakaCna; Huas: tihtsan/tijson; CoreM: k'aj; Quich: sik'Vj; Zapo: biajta/bitia/ptie; Nahua: epazotl;
Language contact: Tot > CoreM; Quich > Chiapas Zoq; Zap > Chontal; Zap <> Nah and Hua; Nah > Spanish

***Dyssodia decipiens* (Bartl.) M.C.Johnst. ex M.C.Johnst. & B.L.Turner (Asteraceae)**

Spanish names: Flor de angelina
Indigenous names: Guibgui parloshaan[21]; Gi togol kwa'č[22]
Used by (2*): Zapotec[21, 22]
Used for (4#): Digestive (1)[21]; Psychological (1)[22]; Skin (1)[22]; General and Unspecified (1)[21]
Cognates:
Language contact:

***Echeandia reflexa* (Cav.) Rose (Asparagaceae)**

Spanish names:
Indigenous names: Eem ts'ohool, eem ilaal, eemil koy, thakpen thel, [07]
Used by (1*): Huastec[07]
Used for (4#): Digestive (1)[07]; Neurological (1)[07]; Skin (1)[07]; Pregnancy (1)[07]
Cognates:
Language contact:

***Echeveria* sp. (Crassulaceae)**

Spanish names: Siempreviva
Indigenous names: Guièe-yùzh[23]
Used by (2*): Zapotec[23]; Nahua[26]
Used for (2#): Skin (2)[23, 26]
Cognates:
Language contact:

***Echinopepon racemosus* (Steud.) C.Jeffrey (Cucurbitaceae)**

Spanish names:
Indigenous names: Tzuyo awit [01]
Used by (1*): Zoque[01]
Used for (1#): Urological (1)[01]
Cognates:
Language contact:

[01-28]refer to the study codes in Table 4.1. *Total number of studies citing this taxon #Total number of use-records

**_Echinopepon_ sp. (Cucurbitaceae)**

Spanish names: Hoja San pedro
Indigenous names: Yopo San Pedro[18]
Used by (1*): Western Maya[18]
Used for (1#): Neurological (1)[18]
Cognates:
Language contact:

**_Echites tuxtlensis_ Standl. (Apocynaceae)**

Spanish names:
Indigenous names: Tsank'ub ts'aah, thuuchum ts'ohool, , thuchuum ichiich, lek'ab choc', milim ch'ohool, ch'aahil otso[07]
Used by (1*): Huastec[07]
Used for (3#): Digestive (1)[07]; Respiratory (1)[07]; Skin (1)[07]
Cognates:
Language contact:

**_Echites yucatanensis_ Millsp. ex Standl. (Apocynaceae)**

Spanish names:
Indigenous names: Sak-viperol[09]
Used by (1*): Yucatecan Maya[09]
Used for (1#): Skin (1)[09]
Cognates:
Language contact:

**_Echium_ vulgare L. (Boraginaceae)**

Spanish names: hierba azul
Indigenous names: Tijtson ts'ojól[08]
Used by (1*): Huastec[08]
Used for (4#): Blood (1)[08]; Musculoskeletal (1)[08]; Skin (1)[08]; General and Unspecified (1)[08]
Cognates:
Language contact:

**_Ehretia anacua_ (Terán & Berland.) I.M.Johnst. (Boraginaceae)**

Spanish names:
Indigenous names: Thathub[07]
Used by (1*): Huastec[07]
Used for (5#): Digestive (1)[07]; Skin (1)[07]; Pregnancy (1)[07]; Female genital (1)[07]; General and Unspecified (1)[07]
Cognates:
Language contact:

**_Ehretia tinifolia_ L. (Boraginaceae)**

Spanish names: Lambimbo; Roble
Indigenous names: T'iiw te'[07]; Beeb, Xi'mche'[09]
Used by (3*): Huastec[07]; Yucatecan Maya[09]; Zapotec[21]
Used for (7#): Digestive (2)[07, 21]; Cardiovascular (1)[21]; Respiratory (1)[09]; Skin (1)[21]; General and Unspecified (2)[09, 21]
Cognates:
Language contact:

**_Eichhornia crassipes_ (Mart.) Solms (Pontederiaceae)**

Spanish names: Riñoncillo
Indigenous names:
Used by (1*): Zapotec[21]
Used for (2#): Digestive (1)[21]; General and Unspecified (1)[21]
Cognates:
Language contact:

[01-28]refer to the study codes in Table 4.1. *Total number of studies citing this taxon #Total number of use-records

***Elaphoglossum herminieri* (Bory ex Fée) T. Moore (Dryopteridaceae)**

Spanish names:
Indigenous names: Rubelsa i xul[17]
Used by (1*): Quichean Maya[17]
Used for (1#): nd[17]
Cognates:
Language contact:

***Elaphoglossum peltatum* (Sw.) Urb. (Dryopteridaceae)**

Spanish names:
Indigenous names: Culantro pim[17]
Used by (1*): Quichean Maya[17]
Used for (1#): nd[17]
Cognates:
Language contact:

***Elaphoglossum* sp. (Dryopteridaceae)**

Spanish names: Hierba Dura
Indigenous names: Achi'y Q'os Aqom[12]; Lùdz-mdzìn, guìzh-lùdz-mdzìn[23]
Used by (3*): Zoque[03]; Quichean Maya[12]; Zapotec[23]
Used for (7#): Digestive (3)[03, 12, 23]; Endocrine (1)[12]; Urological (1)[12]; Male genital (1)[12]; General and Unspecified (1)[12]
Cognates:
Language contact:

***Eleocharis elegans* (Kunth) Roem. & Schult. (Cyperaceae)**

Spanish names:
Indigenous names: Boo'wat toom[07]
Used by (1*): Huastec[07]
Used for (1#): General and Unspecified (1)[07]
Cognates:
Language contact:

***Eleocharis geniculata* (L.) Roem. & Schult. (Cyperaceae)**

Spanish names: Cola de borrego
Indigenous names:
Used by (1*): Nahua[26]
Used for (1#): Urological (1)[26]
Cognates:
Language contact:

***Eleocharis montana* (Kunth) Roem. & Schult. (Cyperaceae)**

Spanish names: Cintul
Indigenous names: Pap/tza'a tzoy[01]
Used by (1*): Zoque[01]
Used for (1#): General and Unspecified (1)[01]
Cognates:
Language contact:

***Elephantopus* sp. (Asteraceae)**

Spanish names: Tahuane sonjehuite
Indigenous names:
Used by (1*): Totonac[05]
Used for (1#): Skin (1)[05]
Cognates:
Language contact:

**_Eleutherine bulbosa_ (Mill.) Urb. (Iridaceae)**

Spanish names:
Indigenous names: Tsakam apats', chak apach'[07]
Used by (1*): Huastec[07]
Used for (1#): Digestive (1)[07]
Cognates:
Language contact:

**_Elytraria bromoides_ Oerst. (Acanthaceae)**

Spanish names:
Indigenous names: Xutsum bat'aw[07]
Used by (1*): Huastec[07]
Used for (1#): Ear (1)[07]
Cognates:
Language contact:

**_Elytraria imbricata_ (Vahl) Pers. (Acanthaceae)**

Spanish names: Un pie
Indigenous names: Was'an tzitz[01]; Kabalxa'an, Kambaxa'an[09]
Used by (2*): Zoque[01]; Yucatecan Maya[09]
Used for (3#): Digestive (1)[09]; Skin (1)[01]; Female genital (1)[09]
Cognates:
Language contact: Chiapas Zoq <> Yuc

**_Encyclia_ sp. (Orchidaceae)**

Spanish names:
Indigenous names: Xkananikte'[09]
Used by (1*): Yucatecan Maya[09]
Used for (1#): Skin (1)[09]
Cognates:
Language contact:

**_Entada rheedii_ Spreng. (Fabaceae)**

Spanish names: Dinero de judas, dinero del judío
Indigenous names:
Used by (1*): Totonac[05]
Used for (3#): Digestive (1)[05]; Musculoskeletal (1)[05]; Skin (1)[05]
Cognates:
Language contact:

**_Enterolobium cyclocarpum_ (Jacq.) Griseb. (Fabaceae)**

Spanish names: Guanacastle
Indigenous names: Tzang kuy [01]; Jipi[03]; Xøxy kepky[04]; Tiyow[07]; Pich[09]; Biguisha[21]
Used by (6*): Zoque[01, 03]; Mixe[04]; Huastec[07]; Yucatecan Maya[09]; Zapotec[21]
Used for (9#): Digestive (3)[01, 03, 09]; Respiratory (2)[01, 07]; Skin (2)[04, 21]; General and Unspecified (2)[09, 21]
Cognates:
Language contact:

**_Enterolobium schomburgkii_ (Benth.) Benth. (Fabaceae)**

Spanish names: Guanacastilla
Indigenous names:
Used by (1*): Mixe[04]
Used for (1#): Skin (1)[04]
Cognates:
Language contact:

[01-28]refer to the study codes in Table 4.1. *Total number of studies citing this taxon #Total number of use-records

***Epaltes mexicana* Less. (Asteraceae)**

Spanish names: Hierba de sapo, gurusapo; Manzanilla de tres lomos; Tabaquillo, sabañon
Indigenous names: Putax ay[03]; Piix[04]; Kúz ix much[18]; Badxuumiij[21]
Used by (5*): Zoque[02, 03]; Mixe[04]; Western Maya[18]; Zapotec[21]
Used for (18#): Digestive (1)[03]; Musculoskeletal (2)[03, 18]; Neurological (2)[04, 18]; Respiratory (2)[02, 18]; Skin (3)[03, 18, 21]; Pregnancy (1)[21]; Female genital (3)[02, 03, 21]; General and Unspecified (4)[03, 04, 18, 21]
Cognates: MZ: piix /putax;
Language contact: MZ > Chontal and Zap

***Epidendrum difforme* Jacq. (Orchidaceae)**

Spanish names:
Indigenous names: Tsooy pathum[07]
Used by (1*): Huastec[07]
Used for (2#): Neurological (1)[07]; General and Unspecified (1)[07]
Cognates:
Language contact:

***Epilobium* sp. (Onagraceae)**

Spanish names: Clavo del monte
Indigenous names: Guish claab[21]
Used by (1*): Zapotec[21]
Used for (3#): Respiratory (1)[21]; Skin (1)[21]; Female genital (1)[21]
Cognates:
Language contact:

***Epiphyllum crenatum* (Lindl.) Don (Cactaceae)**

Spanish names: Pitaya
Indigenous names: Nuchchi[03]
Used by (1*): Zoque[03]
Used for (4#): Digestive (1)[03]; Skin (1)[03]; Pregnancy (1)[03]; Female genital (1)[03]
Cognates:
Language contact:

***Epiphyllum hookeri* Haw. (Cactaceae)**

Spanish names:
Indigenous names: Ti'qol' bak'[14]
Used by (1*): Quichean Maya[14]
Used for (1#): Musculoskeletal (1)[14]
Cognates:
Language contact:

***Epiphyllum phyllanthus* (L.) Haw. (Cactaceae)**

Spanish names:
Indigenous names: Chik'ba'l b'ak[17]
Used by (1*): Quichean Maya[17]
Used for (1#): nd[17]
Cognates:
Language contact:

***Equisetum hyemale* L. (Equisetaceae)**

Spanish names: Cola da Caballo; Cola de iguana/ de caballo
Indigenous names: Rujey' kej, Xpumay Aq'om[12]; Xye' Kawaay [14]; Yok' es chan[20]
Used by (4*): Quichean Maya[12, 14]; Western Maya[20]; Nahua[27]
Used for (12#): Blood (1)[12]; Digestive (2)[12, 20]; Neurological (1)[12]; Skin (2)[12, 14]; Urological (2)[12, 27]; Female genital (1)[12]; Male genital (1)[12]; General and Unspecified (2)[12, 14]
Cognates: CoreM: kej / k'es;
Language contact:

***Equisetum myriochaetum* Schltdl. & Cham. (Equisetaceae)**

Spanish names: Cola de caballo
Indigenous names: Cayo tuty/sus tokdong/tu tane/caballo tutz muk [01]; U nej chj[19]; X-pàan ngùtsiěts, x-côl-càbâll, guìzh-zhìg[23]
Used by (6*): Zoque[01]; Western Maya[19]; Zapotec[23]; Nahua[25, 26, 28]
Used for (16#): Digestive (1)[01]; Cardiovascular (1)[01]; Musculoskeletal (2)[01, 23]; Skin (1)[28]; Endocrine (2)[01, 25]; Urological (6)[01, 19, 23, 25, 26, 28]; Female genital (1)[01]; Male genital (1)[01]; General and Unspecified (1)[28]
Cognates:
Language contact:

***Equisetum* sp. (Equisetaceae)**

Spanish names: Cola de caballo
Indigenous names: Tzoy muk [02]; Uje' kies, uje kej, tu aj, wiquab' q'ayes, rismachi koj, xul aj, tum[13]; Shcool cavaij[21]
Used by (4*): Zoque[02, 03]; Quichean Maya[13]; Zapotec[21]
Used for (15#): Blood (1)[21]; Digestive (2)[02, 21]; Musculoskeletal (1)[13]; Psychological (2)[13, 21]; Skin (1)[13]; Urological (3)[02, 13, 21]; Pregnancy (2)[02, 03]; Female genital (1)[13]; General and Unspecified (2)[13, 21]
Cognates:
Language contact:

***Erechtites hieracifolia* (L.) Raf. (Asteraceae)**

Spanish names:
Indigenous names: Malil koy, tsakam xobo' ts'ohool, akw'aalil koy[07]
Used by (1*): Huastec[07]
Used for (1#): General and Unspecified (1)[07]
Cognates:
Language contact:

***Eremosis leiocarpa* (DC.) Gleason (Asteraceae)**

Spanish names: Suquinay; Suquinay, Sal Andrews
Indigenous names: Suquinay[14]; Bakil vomol[20]
Used by (3*): Quichean Maya[12, 14]; Western Maya[20]
Used for (6#): Digestive (2)[12, 20]; Cardiovascular (1)[12]; Respiratory (1)[14]; Endocrine (1)[12]; General and Unspecified (1)[14]
Cognates:
Language contact:

***Erigeron karvinskianus* DC. (Asteraceae)**

Spanish names: Hierba del burro; Margarita silvestre
Indigenous names: Ts'ojolil an churi[08]; Sal b'ey, kotz'ij b'ey[12]; Tepemalin, tepemanzanilla[26]
Used by (4*): Zoque[01]; Huastec[08]; Quichean Maya[12]; Nahua[26]
Used for (13#): Digestive (3)[01, 08, 26]; Musculoskeletal (1)[08]; Neurological (2)[01, 08]; Respiratory (2)[08, 12]; Skin (3)[01, 08, 12]; Urological (1)[08]; General and Unspecified (1)[12]
Cognates:
Language contact:

***Eriobotrya japonica*** **(Thunb.) Lindl. (Rosaceae)**

Spanish names: Níspero
Indigenous names: Yàg-nîspèrò[23]
Used by (6*): Zoque[01]; Totonac[06]; Quichean Maya[12, 13]; Zapotec[23]; Nahua[25]
Used for (21#): Digestive (5)[01, 06, 12, 13, 23]; Cardiovascular (1)[06]; Musculoskeletal (2)[01, 06]; Respiratory (2)[12, 25]; Endocrine (2)[01, 12]; Urological (5)[01, 06, 12, 13, 25]; Female genital (2)[12, 13]; Male genital (1)[01]; General and Unspecified (1)[12]
Cognates:
Language contact:

***Eriosema crinitum*** **(Kunth) G. Don (Fabaceae)**

Spanish names:
Indigenous names:
Used by (1*): Zoque[03]
Used for (3#): Skin (1)[03]; Urological (1)[03]; Pregnancy (1)[03]
Cognates:
Language contact:

***Eriosema diffusum*** **(Kunth) G. Don (Fabaceae)**

Spanish names:
Indigenous names: Poja sotyi[03]
Used by (1*): Zoque[03]
Used for (3#): Digestive (1)[03]; Psychological (1)[03]; Skin (1)[03]
Cognates:
Language contact:

***Eryngium carlinae*** **F.Delaroche (Apiaceae)**

Spanish names: Cilandro ancho, culandro cimarron, culandro de monte, culandro indio, silentro de perro, espina de coche, espinabodoc, escorsoner
Indigenous names: Samat, uklanto tzé', to' k'ix[13]
Used by (1*): Quichean Maya[13]
Used for (4#): Ear (1)[13]; Musculoskeletal (1)[13]; Skin (1)[13]; Female genital (1)[13]
Cognates:
Language contact:

***Eryngium cymosum*** **F.Delaroche (Apiaceae)**

Spanish names: Cardo de Bosque; Espina blanca, escorsonera
Indigenous names: Q'ix juyu'[12]; Sak' k'ix[13]
Used by (2*): Quichean Maya[12, 13]
Used for (9#): Digestive (1)[13]; Cardiovascular (1)[12]; Musculoskeletal (2)[12, 13]; Neurological (1)[13]; Skin (2)[12, 13]; Female genital (1)[13]; General and Unspecified (1)[12]
Cognates: Quich: q'ix;
Language contact:

***Eryngium foetidum*** **L. (Apiaceae)**

Spanish names: Cilantro cimarrón, perejil
Indigenous names: Culando[01]; Kortesanto Kix Aq'om[12]; Samat[17]; Yujtz' ner via'r[19]
Used by (9*): Zoque[01, 03]; Mixe[04]; Quichean Maya[12, 17]; Western Maya[18, 19]; Zapotec[21]; Nahua[26]
Used for (31#): Digestive (5)[01, 03, 04, 18, 19]; Ear (1)[03]; Cardiovascular (1)[18]; Musculoskeletal (1)[21]; Neurological (2)[01, 26]; Psychological (1)[18]; Respiratory (3)[03, 18, 21]; Skin (3)[01, 18, 26]; Endocrine (1)[01]; Pregnancy (3)[01, 03, 18]; Female genital (2)[12, 18]; Male genital (3)[03, 12, 18]; General and Unspecified (4)[01, 12, 21, 26]; nd[17]
Cognates:
Language contact:

[01-28]refer to the study codes in Table 4.1. *Total number of studies citing this taxon #Total number of use-records

***Eryngium gracile* F.Delaroche (Apiaceae)**

Spanish names:
Indigenous names:
Used by (1*): Quichean Maya[13]
Used for (1#): Urological (1)[13]
Cognates:
Language contact:

***Eryngium longifolium* Cav. (Apiaceae)**

Spanish names: Piñuela
Indigenous names:
Used by (1*): Nahua[25]
Used for (2#): Endocrine (1)[25]; Urological (1)[25]
Cognates:
Language contact:

***Eryngium nasturtiifolium* Juss. ex F.Delaroche (Apiaceae)**

Spanish names:
Indigenous names: Ehtiil i laab kulaantu[07]
Used by (1*): Huastec[07]
Used for (1#): Respiratory (1)[07]
Cognates:
Language contact:

***Eryngium* sp. (Apiaceae)**

Spanish names:
Indigenous names: Tsakam bathuch[07]; Guièts-mél-lò, guìzh-guièts[23]
Used by (2*): Huastec[07]; Zapotec[23]
Used for (3#): Digestive (1)[23]; Respiratory (1)[07]; Urological (1)[23]
Cognates:
Language contact:

***Erythrina americana* Mill. (Fabaceae)**

Spanish names: Gasparo
Indigenous names: Ihahlni'[06]; Hutukuu', pemoch, pemuuts[07]
Used by (2*): Totonac[06]; Huastec[07]
Used for (5#): Psychological (1)[07]; Skin (1)[06]; Pregnancy (1)[07]; General and Unspecified (2)[06, 07]
Cognates:
Language contact:

***Erythrina berteroana* Urb. (Fabaceae)**

Spanish names: Hierbaboracha; Palo de Pito; Palo pito
Indigenous names: Monhg ay tzäpä[02]; Oxexy[04]; Tzité[12]; Mzi té[13]; Tzité[14]
Used by (5*): Zoque[02]; Mixe[04]; Quichean Maya[12, 13, 14]
Used for (22#): Blood (1)[12]; Digestive (3)[02, 12, 14]; Cardiovascular (1)[14]; Musculoskeletal (2)[12, 14]; Neurological (2)[12, 13]; Psychological (3)[02, 12, 14]; Respiratory (2)[04, 12]; Skin (3)[02, 12, 14]; Urological (1)[02]; Female genital (2)[12, 14]; General and Unspecified (2)[12, 12]
Cognates: Quich: tzite;
Language contact:

***Erythrina caribaea* Krukoff & Barneby (Fabaceae)**

Spanish names: Gasparo; Madrecacao/ Flor de pita/madre
Indigenous names: Tzentzen[01]; Ihahlni'[06]
Used by (2*): Zoque[01]; Totonac[06]
Used for (4#): Digestive (1)[01]; Skin (1)[06]; Urological (1)[01]; General and Unspecified (1)[06]
Cognates:
Language contact:

***Erythrina coralloides* DC. (Fabaceae)**

Spanish names: Colorín, gasparo
Indigenous names: Lagatin[05]; Ekimite[26]
Used by (2*): Totonac[05]; Nahua[26]
Used for (2#): Digestive (1)[26]; General and Unspecified (1)[05]
Cognates:
Language contact:

***Erythrina folkersii* Krukoff & Moldenke (Fabaceae)**

Spanish names: Colorin, zempantle
Indigenous names: Guixa'a mindu'u[21]
Used by (1*): Zapotec[21]
Used for (3#): Neurological (1)[21]; Psychological (1)[21]; General and Unspecified (1)[21]
Cognates:
Language contact:

***Erythrina herbacea* L. (Fabaceae)**

Spanish names:
Indigenous names: Copa tsen tsen[03]
Used by (1*): Zoque[03]
Used for (4#): Digestive (1)[03]; Skin (1)[03]; Pregnancy (1)[03]; Female genital (1)[03]
Cognates:
Language contact:

***Erythrina lanata* Rose (Fabaceae)**

Spanish names: Palo cuchillo
Indigenous names: Tzentzen[02]
Used by (1*): Zoque[02]
Used for (1#): Eye (1)[02]
Cognates:
Language contact:

***Erythrina rubrinervia* Kunth (Fabaceae)**

Spanish names: Pito
Indigenous names:
Used by (1*): Yucatecan Maya[10]
Used for (1#): General and Unspecified (1)[10]
Cognates:
Language contact:

***Erythrina* sp. (Fabaceae)**

Spanish names: Cosquelite, Colorín; Pito; Tzojantli
Indigenous names: N¥tung tsen tsen[03]; Mo'te'[19]; Tzompantl[28]
Used by (3*): Zoque[03]; Western Maya[19]; Nahua[28]
Used for (10#): Digestive (1)[28]; Neurological (1)[19]; Psychological (1)[19]; Skin (1)[03]; Pregnancy (2)[03, 19]; Female genital (2)[03, 19]; General and Unspecified (2)[03, 19]
Cognates:
Language contact:

[01-28]refer to the study codes in Table 4.1. *Total number of studies citing this taxon #Total number of use-records

***Erythrina standleyana* Krukoff (Fabaceae)**

Spanish names:
Indigenous names: Tsamnek hutukuu'[07]; Chakmolonche'[09]
Used by (2*): Huastec[07]; Yucatecan Maya[09]
Used for (6#): Eye (1)[09]; Neurological (1)[07]; Respiratory (2)[07, 09]; Urological (1)[09]; Pregnancy (1)[07]
Cognates:
Language contact:

***Erythroxylum rotundifolium* Lunan (Erythroxylaceae)**

Spanish names:
Indigenous names: Xik'iche'[09]
Used by (1*): Yucatecan Maya[09]
Used for (1#): Skin (1)[09]
Cognates:
Language contact:

***Esenbeckia berlandieri* Baill. (Rutaceae)**

Spanish names:
Indigenous names: Lanaax te'[07]
Used by (1*): Huastec[07]
Used for (2#): Digestive (1)[07]; Neurological (1)[07]
Cognates:
Language contact:

***Espejoa mexicana* DC. (Asteraceae)**

Spanish names: Maragrita del monte
Indigenous names:
Used by (1*): Zapotec[21]
Used for (1#): Skin (1)[21]
Cognates:
Language contact:

***Eucalyptus camaldulensis* Dehnh. & E. & E. sp. L'Hér. (Myrtaceae)**

Spanish names: Eukalypto
Indigenous names:
Used by (1*): Zoque[01]
Used for (1#): Respiratory (1)[01]
Cognates:
Language contact:

***Eucalyptus cinerea* F.Muell. ex Benth. (Myrtaceae)**

Spanish names: Eucalipto; Hoja de alcanfor
Indigenous names:
Used by (4*): Zoque[01, 02]; Quichean Maya[12, 14]
Used for (11#): Digestive (1)[12]; Musculoskeletal (2)[02, 12]; Psychological (1)[12]; Respiratory (4)[01, 02, 12, 14]; Skin (1)[12]; Female genital (1)[02]; General and Unspecified (1)[12]
Cognates:
Language contact:

[01-28]refer to the study codes in Table 4.1. *Total number of studies citing this taxon #Total number of use-records

***Eucalyptus globulus* Labill. (Myrtaceae)**

Spanish names: Eucalipto
Indigenous names: Ts'uj[08]; Ukal, awoliy che', ok'al che'[13]; Yàg-eùcàlîpt[23]
Used by (8*): Huastec[08]; Quichean Maya[13]; Western Maya[18]; Zapotec[22, 23]; Nahua[25, 26, 27]
Used for (15#): Digestive (1)[23]; Musculoskeletal (1)[13]; Neurological (2)[08, 13]; Respiratory (8)[08, 13, 18, 22, 23, 25, 26, 27]; Endocrine (1)[18]; General and Unspecified (2)[08, 18]
Cognates:
Language contact:

***Eucalyptus* sp. (Myrtaceae)**

Spanish names: Alcamfor; Eucalipto; Eucalito; Eucalyptus
Indigenous names: Gusha'a alcanfor, ventulatu[21]
Used by (7*): Zoque[02, 03]; Mixe[04]; Yucatecan Maya[09]; Western Maya[19]; Zapotec[21]; Nahua[26]
Used for (15#): Digestive (1)[21]; Musculoskeletal (2)[21, 26]; Respiratory (7)[02, 03, 04, 09, 19, 21, 26]; Endocrine (1)[03]; Pregnancy (1)[21]; Female genital (1)[03]; General and Unspecified (2)[03, 21]
Cognates:
Language contact:

***Eugenia acapulcensis* Steud. (Myrtaceae)**

Spanish names: Escobilla
Indigenous names: Petcuy[03]; Axtaam pikx[04]
Used by (2*): Zoque[03]; Mixe[04]
Used for (4#): Digestive (2)[03, 04]; Skin (1)[03]; Female genital (1)[03]
Cognates:
Language contact:

***Eugenia capuli* (Schltdl. & Cham.) Hook. & Arn. (Myrtaceae)**

Spanish names: Capulín; Cinco negrito
Indigenous names: Islakastápu tamak, aka'lasni[05]; Pehte', chuk ba'im[07]; Yanaj'[21]
Used by (3*): Totonac[05]; Huastec[07]; Zapotec[21]
Used for (11#): Digestive (3)[05, 07, 21]; Respiratory (2)[07, 21]; Skin (2)[07, 21]; Endocrine (1)[07]; Urological (1)[07]; Pregnancy (1)[21]; General and Unspecified (1)[21]
Cognates:
Language contact:

***Eugenia foetida* Pers. (Myrtaceae)**

Spanish names:
Indigenous names: Xhilnich', Sakloobche'[09]
Used by (1*): Yucatecan Maya[09]
Used for (1#): Skin (1)[09]
Cognates:
Language contact:

***Eugenia oerstediana* O.Berg (Myrtaceae)**

Spanish names:
Indigenous names: Ha' pehte', chuk ba'im, wal puchun[07]
Used by (1*): Huastec[07]
Used for (1#): Digestive (1)[07]
Cognates:
Language contact:

***Eugenia rhombea*** **(O.Berg) Krug & Urb. (Myrtaceae)**

Spanish names:
Indigenous names: Lamush pim[17]
Used by (1*): Quichean Maya[17]
Used for (1#): nd[17]
Cognates:
Language contact:

***Eugenia*** **sp. (Myrtaceae)**

Spanish names:
Indigenous names: Chaknii[09]
Used by (1*): Yucatecan Maya[09]
Used for (2#): Eye (1)[09]; Skin (1)[09]
Cognates:
Language contact:

***Eupatorium*** **sp. (Asteraceae)**

Spanish names: Hierba buenilla; Hierba de zopilote; Prodigiosa amarilla
Indigenous names: Š haà¢[10]; Ratz'un Q'ehen[14]
Used by (4*): Yucatecan Maya[10]; Quichean Maya[14]; Zapotec[21]; Nahua[26]
Used for (8#): Digestive (2)[21, 26]; Eye (1)[14]; Musculoskeletal (1)[10]; Neurological (1)[10]; Skin (2)[10, 26]; General and Unspecified (1)[10]
Cognates: CoreM: xaach / ratz;
Language contact:

***Euphorbia armourii*** **Millsp. (Euphorbiaceae)**

Spanish names:
Indigenous names: Sakchakah, Sibik'[09]
Used by (1*): Yucatecan Maya[09]
Used for (1#): Skin (1)[09]
Cognates:
Language contact:

***Euphorbia chamaesyce*** **L. (Euphorbiaceae)**

Spanish names: Hierba de la araña
Indigenous names:
Used by (1*): Nahua[26]
Used for (1#): Skin (1)[26]
Cognates:
Language contact:

***Euphorbia colletioides*** **Benth. (Euphorbiaceae)**

Spanish names:
Indigenous names: T'ot'oy ts'ohool, tsakam ts'uh[07]
Used by (1*): Huastec[07]
Used for (3#): Musculoskeletal (1)[07]; Skin (1)[07]; General and Unspecified (1)[07]
Cognates:
Language contact:

***Euphorbia cotinifolia*** **L. (Euphorbiaceae)**

Spanish names: Hierba Mala
Indigenous names: Orob[12]; Mansaana Q'ehen[14]
Used by (2*): Quichean Maya[12, 14]
Used for (3#): Digestive (1)[14]; Cardiovascular (1)[14]; Skin (1)[12]
Cognates:
Language contact:

[01-28]refer to the study codes in Table 4.1. *Total number of studies citing this taxon #Total number of use-records

***Euphorbia cyri*** **V.W.Steinm. (Euphorbiaceae)**

Spanish names: Gordobahn
Indigenous names: Bini[22]
Used by (1*): Zapotec[22]
Used for (1#): Digestive (1)[22]
Cognates:
Language contact:

***Euphorbia dioeca*** **Kunth (Euphorbiaceae)**

Spanish names: Hoja de azar
Indigenous names: Tza'a tzoy[01]
Used by (1*): Zoque[01]
Used for (1#): General and Unspecified (1)[01]
Cognates:
Language contact:

***Euphorbia dioscoreoides*** **Boiss. (Euphorbiaceae)**

Spanish names:
Indigenous names: Ehtiil mithith, bathuch ts'ohool, ik ts'ohool, thootil tsan pulik, ts'uleel thekw'eel, t'apay ts'ohool[07]
Used by (1*): Huastec[07]
Used for (1#): Skin (1)[07]
Cognates:
Language contact:

***Euphorbia heterophylla*** **L. (Euphorbiaceae)**

Spanish names: Hierba de la araña chica; Leche de Cabro, Quineo; Lecheria; Quebra muelas/ amarra hueso
Indigenous names: Hab ichiich, leetsa ts'ohool, puunchiix wits, alwa' akal ch'ohool, i'ixte, ch'awil ch'ohool[07]; X'lee'chi'yuk, Tul[14]
Used by (6*): Zoque[01, 03]; Huastec[07]; Quichean Maya[14]; Zapotec[21]; Nahua[26]
Used for (13#): Digestive (2)[01, 07]; Cardiovascular (1)[07]; Musculoskeletal (1)[01]; Neurological (1)[07]; Psychological (1)[07]; Respiratory (1)[07]; Skin (3)[14, 21, 26]; Pregnancy (1)[07]; Female genital (1)[03]; General and Unspecified (1)[07]
Cognates:
Language contact:

***Euphorbia hirta*** **L. (Euphorbiaceae)**

Spanish names: Golondrina
Indigenous names: Tza'a tzoy[01]; Paipai[03]; Xa' ts'ohool, xaa' uts'aal pulik, t'ithith i koy, pakw'[07]; Xanabmukuy[09]; Guish mbsia'a[21]
Used by (6*): Zoque[01, 03]; Huastec[07]; Yucatecan Maya[09]; Western Maya[18]; Zapotec[21]
Used for (11#): Digestive (2)[03, 21]; Eye (1)[03]; Respiratory (1)[03]; Skin (3)[07, 09, 18]; Urological (2)[03, 21]; Female genital (1)[03]; General and Unspecified (1)[01]
Cognates:
Language contact: Chiapas Zoq <> Hua <> Yuc <> Zap

***Euphorbia hypericifolia*** **L. (Euphorbiaceae)**

Spanish names:
Indigenous names: Leetsa ts'ohool tsakni', k'apwaal book', puxek' ts'ohool, [07]
Used by (1*): Huastec[07]
Used for (1#): Skin (1)[07]
Cognates:
Language contact:

[01-28]refer to the study codes in Table 4.1. *Total number of studies citing this taxon #Total number of use-records

***Euphorbia hyssopifolia* L. (Euphorbiaceae)**

Spanish names: Hierba de paño/hierba de antojo/hoja de azar
Indigenous names: Tza'a tzoy[01]; Yook mukuy[10]
Used by (3*): Zoque[01]; Yucatecan Maya[10]; Quichean Maya[17]
Used for (5#): Eye (1)[10]; Psychological (1)[01]; Skin (1)[01]; General and Unspecified (1)[01]; nd[17]
Cognates:
Language contact:

***Euphorbia lancifolia* Schltdl. (Euphorbiaceae)**

Spanish names: Lecheria
Indigenous names: M¥a xuxcuy[03]; Leetsa ts'ohool[07]; Ix but[11]; Ixbut[13]
Used by (5*): Zoque[03]; Huastec[07]; Yucatecan Maya[11]; Quichean Maya[13, 17]
Used for (10#): Digestive (1)[07]; Musculoskeletal (1)[07]; Skin (2)[03, 07]; Pregnancy (3)[07, 11, 13]; Male genital (1)[11]; General and Unspecified (1)[07]; nd[17]
Cognates: CoreM: ixbut;
Language contact:

***Euphorbia maculata* L. (Euphorbiaceae)**

Spanish names:
Indigenous names: Škwan gurak[22]
Used by (1*): Zapotec[22]
Used for (2#): Digestive (1)[22]; General and Unspecified (1)[22]
Cognates:
Language contact:

***Euphorbia micromera* Boiss. ex Engelm. (Euphorbiaceae)**

Spanish names: Zacate rojo
Indigenous names:
Used by (1*): Zapotec[21]
Used for (2#): Digestive (1)[21]; Skin (1)[21]
Cognates:
Language contact:

***Euphorbia milii* Des Moul. (Euphorbiaceae)**

Spanish names:
Indigenous names:
Used by (1*): Quichean Maya[14]
Used for (1#): Respiratory (1)[14]
Cognates:
Language contact:

***Euphorbia parviflora* L. (Euphorbiaceae)**

Spanish names: Hierba del gusano/ de la araña
Indigenous names:
Used by (1*): Nahua[26]
Used for (1#): Skin (1)[26]
Cognates:
Language contact:

***Euphorbia personata* (Croizat) V.W.Steinm. (Euphorbiaceae)**

Spanish names:
Indigenous names: Nabalche', Nahualte[09]
Used by (1*): Yucatecan Maya[09]
Used for (2#): Neurological (1)[09]; Skin (1)[09]
Cognates:
Language contact:

***Euphorbia prostrata* Aiton (Euphorbiaceae)**

Spanish names: Golondrina; Golondrina, riñonia; Golondrina/hoja de azar
Indigenous names: Tza'a tzoy[01]; Ixgolondrina[18]
Used by (3*): Zoque[01, 02]; Western Maya[18]
Used for (9#): Digestive (1)[02]; Cardiovascular (1)[02]; Respiratory (1)[02]; Endocrine (1)[02]; Urological (2)[02, 18]; Female genital (1)[02]; General and Unspecified (2)[01, 02]
Cognates:
Language contact:

***Euphorbia pteroneura* A.Berger (Euphorbiaceae)**

Spanish names:
Indigenous names: Much'kok[09]
Used by (1*): Yucatecan Maya[09]
Used for (1#): Respiratory (1)[09]
Cognates:
Language contact:

***Euphorbia pulcherrima* Willd. ex Klotzsch (Euphorbiaceae)**

Spanish names: Flor de noche buena; Noche buena, flor de pascua; Nochebuena
Indigenous names: Atxapts[04]; Oot' wits, k'alul wits[07]; Guie chien[21]; Custicpascua xochitl[28]
Used by (5*): Zoque[02]; Mixe[04]; Huastec[07]; Zapotec[21]; Nahua[28]
Used for (8#): Respiratory (1)[28]; Skin (3)[02, 07, 21]; Female genital (3)[04, 07, 28]; General and Unspecified (1)[21]
Cognates:
Language contact:

***Euphorbia serpens* Kunth (Euphorbiaceae)**

Spanish names:
Indigenous names: Xaa' uts'aal, tsakam nuk'ats ts'ohool[07]
Used by (1*): Huastec[07]
Used for (5#): Digestive (1)[07]; Ear (1)[07]; Respiratory (1)[07]; Skin (1)[07]; Pregnancy (1)[07]
Cognates:
Language contact:

***Euphorbia* sp. (Euphorbiaceae)**

Spanish names: Golndrina, china; Golondrina; Hierba de la golondrina
Indigenous names: Kambalchakah[09]; Hobon Čokoh[10]; Xtzin' iyuk[14]; Guìzh-biè, guìzh-xlŭtsí, guìzh-lìndrîn, guìzh-zhìp, zhìp-nquĭts[23]; Còrdòbân[23]
Used by (6*): Zoque[03]; Yucatecan Maya[09, 10]; Quichean Maya[13, 14]; Zapotec[23]
Used for (9#): Eye (3)[03, 23, 23]; Musculoskeletal (1)[23]; Neurological (1)[23]; Skin (2)[09, 13]; Female genital (1)[14]; General and Unspecified (1)[10]
Cognates:
Language contact:

***Euphorbia terracina* L. (Euphorbiaceae)**

Spanish names:
Indigenous names: Hobonk'ak'[09]
Used by (1*): Yucatecan Maya[09]
Used for (1#): Eye (1)[09]
Cognates:
Language contact:

[01-28]refer to the study codes in Table 4.1. *Total number of studies citing this taxon #Total number of use-records

***Euphorbia tithymaloides* L. (Euphorbiaceae)**

Spanish names: Mayorga
Indigenous names: Pak son kuy [01]; Majk tekøøk tsom[04]; Akan t'ele', chk'te', akan chakam, tsukte'[07]; Ya'axhalalche'[09]; Nia'badu'u[21]; Pilicxitl[24]
Used by (10*): Zoque[01, 03]; Mixe[04]; Huastec[07]; Yucatecan Maya[09]; Western Maya[18, 19]; Zapotec[21]; Nahua[24, 25]
Used for (23#): Digestive (1)[04]; Ear (1)[03]; Musculoskeletal (4)[01, 18, 19, 21]; Neurological (1)[21]; Skin (5)[03, 07, 09, 18, 25]; Endocrine (1)[25]; Pregnancy (3)[01, 07, 24]; Female genital (3)[01, 07, 25]; General and Unspecified (4)[03, 04, 21, 25]
Cognates:
Language contact:

***Euphorbia velleriflora* (Klotzsch & Garcke) Boiss. (Euphorbiaceae)**

Spanish names:
Indigenous names: Petx kukmuk[03]
Used by (1*): Zoque[03]
Used for (1#): Urological (1)[03]
Cognates:
Language contact:

***Eustoma exaltatum* (L.) Salisb. (Gentianaceae)**

Spanish names:
Indigenous names: Bioleta[07]; Ka'paj[14]
Used by (2*): Huastec[07]; Quichean Maya[14]
Used for (3#): Digestive (1)[14]; General and Unspecified (2)[07, 14]
Cognates:
Language contact:

***Evolvulus nummularius* (L.) L. (Convolvulaceae)**

Spanish names:
Indigenous names: Kok' centaviwil pim [14]
Used by (1*): Quichean Maya[14]
Used for (1#): General and Unspecified (1)[14]
Cognates:
Language contact:

***Exostema caribaeum* (Jacq.) Schult. (Rubiaceae)**

Spanish names: Chichipactli
Indigenous names: Tzapas kuy[02]
Used by (1*): Zoque[02]
Used for (3#): Digestive (1)[02]; Endocrine (1)[02]; Pregnancy (1)[02]
Cognates:
Language contact:

***Exostema mexicanum* A.Gray (Rubiaceae)**

Spanish names: Nazareno amargo; Quina; Quina, Hombre Grande
Indigenous names: Tam nazareno[03]; Saqi che'[14]
Used by (3*): Zoque[03]; Quichean Maya[12, 14]
Used for (7#): Blood (1)[03]; Digestive (1)[03]; Skin (1)[03]; Endocrine (1)[14]; Pregnancy (1)[03]; Female genital (1)[03]; General and Unspecified (1)[12]
Cognates:
Language contact:

***Exothea copalillo*** **(Schltdl.) Radlk. (Sapindaceae)**

Spanish names:
Indigenous names: T'il homte', t'il hom, bichaam te'[07]
Used by (1*): Huastec[07]
Used for (2#): Neurological (1)[07]; General and Unspecified (1)[07]
Cognates:
Language contact:

***Eysenhardtia adenostylis*** **Baill. (Fabaceae)**

Spanish names: Taray
Indigenous names: Much' Che'[12]
Used by (1*): Quichean Maya[12]
Used for (6#): Blood (1)[12]; Digestive (1)[12]; Endocrine (1)[12]; Urological (1)[12]; Female genital (1)[12]; General and Unspecified (1)[12]
Cognates:
Language contact:

***Eysenhardtia polystachya*** **(Ortega) Sarg. (Fabaceae)**

Spanish names: Taray
Indigenous names: Oku'ku piake[01]; Chilab, chakam wayal[07]
Used by (2*): Zoque[01]; Huastec[07]
Used for (8#): Digestive (1)[01]; Cardiovascular (1)[01]; Endocrine (1)[01]; Urological (2)[01, 07]; Female genital (1)[01]; Male genital (1) [01]; General and Unspecified (1)[07]
Cognates:
Language contact:

***Eysenhardtia*** **sp. (Fabaceae)**

Spanish names: Cuatle, jocotil
Indigenous names: Yàg-guièe-guiâ[23]
Used by (1*): Zapotec[23]
Used for (1#): Digestive (1)[23]
Cognates:
Language contact:

***Fernaldia pandurata*** **(A.DC.) Woodson (Apocynaceae)**

Spanish names:
Indigenous names: T'obs'i', t'obon, kuhuw akw'aal, topts'i'[07]
Used by (1*): Huastec[07]
Used for (3#): Digestive (1)[07]; Endocrine (1)[07]; General and Unspecified (1)[07]
Cognates:
Language contact:

***Ferocactus recurvus*** **(Mill.) Borg (Cactaceae)**

Spanish names: Biznaga
Indigenous names:
Used by (1*): Nahua[27]
Used for (1#): Urological (1)[27]
Cognates:
Language contact:

***Fevillea cordifolia* L. (Cucurbitaceae)**

Spanish names: Chichimora
Indigenous names:
Used by (1*): Western Maya[19]
Used for (1#): Digestive (1)[19]
Cognates:
Language contact:

***Ficus aurea* Nutt. (Moraceae)**

Spanish names: Amate
Indigenous names: Tzaman/jitsi[01]
Used by (1*): Zoque[01]
Used for (2#): Skin (2)[01, 01]
Cognates:
Language contact:

***Ficus carica* L. (Moraceae)**

Spanish names: Higo, Higuerillo / Higuera; Higuero
Indigenous names: Wix[12]
Used by (2*): Quichean Maya[12, 13]
Used for (5#): Cardiovascular (1)[12]; Musculoskeletal (2)[12, 13]; Respiratory (1)[12]; General and Unspecified (1)[12]
Cognates:
Language contact:

***Ficus cotinifolia* Kunth (Moraceae)**

Spanish names: Alamo
Indigenous names: Kopo'[09]
Used by (1*): Yucatecan Maya[09]
Used for (1#): Respiratory (1)[09]
Cognates:
Language contact:

***Ficus crassinervia* Desf. ex Willd. (Moraceae)**

Spanish names: Amate
Indigenous names:
Used by (1*): Zapotec[21]
Used for (4#): Digestive (1)[21]; Cardiovascular (1)[21]; Psychological (1)[21]; General and Unspecified (1)[21]
Cognates:
Language contact:

***Ficus elastica* Roxb. ex Hornem. (Moraceae)**

Spanish names:
Indigenous names: Oom[04]
Used by (1*): Mixe[04]
Used for (1#): Digestive (1)[04]
Cognates:
Language contact:

***Ficus insipida* Willd. (Moraceae)**

Spanish names: Amate
Indigenous names: Xq'oli hu [14]; Hu'u che'[17]
Used by (2*): Quichean Maya[14, 17]
Used for (4#): Neurological (1)[14]; Skin (1)[14]; General and Unspecified (1)[14]; nd[17]
Cognates:
Language contact:

[01-28]refer to the study codes in Table 4.1. *Total number of studies citing this taxon #Total number of use-records

***Ficus obtusifolia* Kunth (Moraceae)**

Spanish names:
Indigenous names: Noak[04]; Hopoy ts'uh[07]
Used by (2*): Mixe[04]; Huastec[07]
Used for (3#): Musculoskeletal (1)[07]; Neurological (1)[07]; General and Unspecified (1)[04]
Cognates:
Language contact:

***Ficus pertusa* L.f. (Moraceae)**

Spanish names:
Indigenous names: Tsui[03]; Tsakam ts'uh[07]
Used by (2*): Zoque[03]; Huastec[07]
Used for (6#): Musculoskeletal (1)[07]; Neurological (1)[07]; Respiratory (1)[07]; Skin (1)[07]; Female genital (1)[03]; General and Unspecified (1)[03]
Cognates:
Language contact: Highland Popoluca <> Hua

***Ficus* sp. (Moraceae)**

Spanish names: Amate grande/orejón; Higo
Indigenous names: Hopoy, ts'uh[07]; Jun, ma'n jun, chu jun[19]; Dxuumii[21]
Used by (3*): Huastec[07]; Western Maya[19]; Zapotec[21]
Used for (9#): Musculoskeletal (2)[07, 21]; Neurological (1)[07]; Respiratory (1)[21]; Skin (3)[07, 19, 21]; General and Unspecified (2)[07, 21]
Cognates:
Language contact: Maya <> Zap; maybe derived from MZ

***Flaveria* sp. (Asteraceae)**

Spanish names: Popote
Indigenous names:
Used by (1*): Nahua[27]
Used for (1#): Digestive (1)[27]
Cognates:
Language contact:

***Flaveria trinervia* (Spreng.) C.Mohr (Asteraceae)**

Spanish names: Hierba del sapo
Indigenous names:
Used by (1*): Nahua[27]
Used for (2#): Digestive (1)[27]; Skin (1)[27]
Cognates:
Language contact:

***Fleischmannia pycnocephala* (Less.) R.M.King & H.Rob. (Asteraceae)**

Spanish names: Flor de cristalina; Mejorana
Indigenous names:
Used by (2*): Zoque[01]; Quichean Maya[12]
Used for (9#): Digestive (1)[12]; Eye (1)[01]; Musculoskeletal (1)[12]; Neurological (1)[12]; Respiratory (1)[12]; Endocrine (1)[12]; Urological (1)[12]; Female genital (1)[12]; General and Unspecified (1)[12]
Cognates:
Language contact:

[01-28]refer to the study codes in Table 4.1. *Total number of studies citing this taxon #Total number of use-records

***Flourensia cernua* DC. (Asteraceae)**

Spanish names: Hoja sen
Indigenous names:
Used by (1*): Zapotec[22]
Used for (1#): Digestive (1)[22]
Cognates:
Language contact:

***Foeniculum vulgare* Mill. (Apiaceae)**

Spanish names: Hinojo
Indigenous names: Diojo xuitl[08]; Anix[13]; Inajo, inaja, injo[20]; Guìzh-hìnôj, mîlt-ró-yù[23]; Diojo xuitl[28]
Used by (9*): Zoque[01]; Huastec[08]; Quichean Maya[12, 13]; Western Maya[20]; Zapotec[23]; Nahua[25, 26, 28]
Used for (24#): Digestive (8)[01, 08, 12, 13, 20, 23, 26, 28]; Ear (1)[20]; Cardiovascular (1)[12]; Neurological (1)[20]; Psychological (2)[01, 13]; Respiratory (5)[01, 12, 20, 25, 28]; Skin (1)[08]; Endocrine (1)[12]; Pregnancy (2)[12, 13]; Male genital (1)[12]; General and Unspecified (1)[13]
Cognates:
Language contact:

***Forchhammeria trifoliata* Radlk. ex Millsp. (Capparaceae)**

Spanish names:
Indigenous names:
Used by (1*): Quichean Maya[14]
Used for (2#): Neurological (1)[14]; General and Unspecified (1)[14]
Cognates:
Language contact:

***Fosterella micrantha* (Lindl.) L. B. Sm. (Bromeliaceae)**

Spanish names:
Indigenous names:
Used by (1*): Zoque[03]
Used for (1#): General and Unspecified (1)[03]
Cognates:
Language contact:

***Fouquieria formosa* Kunth (Fouquieriaceae)**

Spanish names:
Indigenous names: Yàg-guièts-zhìg, yàg-guièe-zhìg[23]
Used by (1*): Zapotec[23]
Used for (1#): Skin (1)[23]
Cognates:
Language contact:

***Frangula purshiana* Cooper (Rhamnaceae)**

Spanish names: Cáscara sagrada
Indigenous names:
Used by (2*): Zoque[02]; Huastec[08]
Used for (3#): Digestive (2)[02, 08]; Skin (1)[08]
Cognates:
Language contact:

***Fraxinus americana* L. (Oleaceae)**

Spanish names:
Indigenous names: Yàg-frêsn[23]
Used by (1*): Zapotec[23]
Used for (2#): Digestive (1)[23]; General and Unspecified (1)[23]
Cognates:
Language contact:

[01-28]refer to the study codes in Table 4.1. *Total number of studies citing this taxon #Total number of use-records

***Fraxinus* sp. (Oleaceae)**

Spanish names: Fresno
Indigenous names:
Used by (1*): Zapotec[22]
Used for (4#): Digestive (1)[22]; Psychological (1)[22]; Skin (1)[22]; Pregnancy (1)[22]
Cognates:
Language contact:

***Fraxinus uhdei* (Wenz.) Lingelsh. (Oleaceae)**

Spanish names: Fresno
Indigenous names:
Used by (1*): Nahua[26]
Used for (2#): Endocrine (1)[26]; General and Unspecified (1)[26]
Cognates:
Language contact:

***Fridericia floribunda* (Kunth) L.G.Lohmann (Bignoniaceae)**

Spanish names:
Indigenous names: Sak-ak'[09]
Used by (1*): Yucatecan Maya[09]
Used for (1#): Skin (1)[09]
Cognates:
Language contact:

***Fridericia patellifera* (Schltdl.) L.G.Lohmann (Bignoniaceae)**

Spanish names:
Indigenous names: Ka'ax aats[04]
Used by (1*): Mixe[04]
Used for (1#): Skin (1)[04]
Cognates:
Language contact:

***Fridericia schumanniana* (Loes.) L.G.Lohmann (Bignoniaceae)**

Spanish names:
Indigenous names: Pobatsay[03]
Used by (1*): Zoque[03]
Used for (1#): Female genital (1)[03]
Cognates:
Language contact:

***Fuchsia hybrida* hort. ex Siebert & Voss (Onagraceae)**

Spanish names: Fuchsia
Indigenous names:
Used by (1*): Quichean Maya[12]
Used for (1#): General and Unspecified (1)[12]
Cognates:
Language contact:

***Fuchsia* sp. (Onagraceae)**

Spanish names:
Indigenous names: Patayuc[13]
Used by (1*): Quichean Maya[13]
Used for (1#): Skin (1)[13]
Cognates:
Language contact:

***Fuchsia splendens* Zucc. (Onagraceae)**

Spanish names:
Indigenous names: Sera nich vomol, tzajal kampana nichim, marabiya wamal, paj nich wamal, bik'tal rimon, batz' momol, , max te' momol, lobol ch'o, kenya jomol[20]
Used by (1*): Western Maya[20]
Used for (3#): Digestive (1)[20]; Respiratory (1)[20]; General and Unspecified (1)[20]
Cognates:
Language contact:

***Galactia striata* (Jacq.) Urb. (Fabaceae)**

Spanish names: Pegapega
Indigenous names:
Used by (1*): Yucatecan Maya[11]
Used for (1#): Urological (1)[11]
Cognates:
Language contact:

***Galeana pratensis* (Kunth) Rydb. (Asteraceae)**

Spanish names:
Indigenous names: Tsukt aay[04]
Used by (1*): Mixe[04]
Used for (1#): Pregnancy (1)[04]
Cognates:
Language contact:

***Galinsoga parviflora* Cav. (Asteraceae)**

Spanish names:
Indigenous names: Guìzh-guièe-lêch, guièe-bnîil-làs, guièe-sàntàmàrǐ-làs, xín-guièe-sàntàmàrǐ, guìzh-guièe-nquǐts[23]
Used by (1*): Zapotec[23]
Used for (1#): Digestive (1)[23]
Cognates:
Language contact:

***Galinsoga quadriradiata* Ruiz & Pav. (Asteraceae)**

Spanish names: San Nicolas
Indigenous names:
Used by (1*): Quichean Maya[12]
Used for (1#): Neurological (1)[12]
Cognates:
Language contact:

***Galium bermudense* L. (Rubiaceae)**

Spanish names:
Indigenous names:
Used by (1*): Quichean Maya[13]
Used for (1#): Neurological (1)[13]
Cognates:
Language contact:

***Galium mexicanum* Kunth (Rubiaceae)**

Spanish names: Pegarropa
Indigenous names: Guìzh-ngùdzìi,  guìzh-nàad[23]
Used by (2*): Zapotec[23]; Nahua[25]
Used for (2#): Ear (1)[23]; Pregnancy (1)[25]
Cognates:
Language contact:

[01-28]refer to the study codes in Table 4.1. *Total number of studies citing this taxon #Total number of use-records

***Galphimia glauca* Cav. (Malpighiaceae)**

Spanish names:
Indigenous names: Tsalaam kubi', it'il chuch[07]; Guìzh-ncuàan-bzhiân, ncuàan-bzhiân[23]
Used by (2*): Huastec[07]; Zapotec[23]
Used for (2#): Psychological (1)[23]; Skin (1)[07]
Cognates:
Language contact:

***Garcia nutans* Vahl ex Rohr (Euphorbiaceae)**

Spanish names:
Indigenous names: Thokb ot'el[07]
Used by (1*): Huastec[07]
Used for (1#): Psychological (1)[07]
Cognates:
Language contact:

***Garcinia intermedia* (Pittier) Hammel (Clusiaceae)**

Spanish names:
Indigenous names: Uouo[03]
Used by (1*): Zoque[03]
Used for (3#): Eye (1)[03]; Skin (1)[03]; Female genital (1)[03]
Cognates:
Language contact:

***Gardenia jasminoides* J.Ellis (Rubiaceae)**

Spanish names: Gardenia
Indigenous names:
Used by (1*): Zoque[02]
Used for (1#): Cardiovascular (1)[02]
Cognates:
Language contact:

***Gardenia* sp. (Rubiaceae)**

Spanish names: Gardenia
Indigenous names:
Used by (2*): Zoque[03]; Nahua[26]
Used for (3#): Digestive (1)[03]; Musculoskeletal (1)[03]; Respiratory (1)[26]
Cognates:
Language contact:

***Gaudichaudia albida* Schltdl. & Cham. (Malpighiaceae)**

Spanish names: Hoja de culebra
Indigenous names: Tzajin poj[02]
Used by (1*): Zoque[02]
Used for (1#): Musculoskeletal (1)[02]
Cognates:
Language contact:

***Gaultheria acuminata* Schltdl. & Cham. (Ericaceae)**

Spanish names: Axocopac
Indigenous names: Toka tzasa[01]; Tecolx'ma'palxch'ma[06]; Axocopa[26]
Used by (3*): Zoque[01]; Totonac[06]; Nahua[26]
Used for (6#): Musculoskeletal (1)[01]; Respiratory (1)[06]; Pregnancy (1)[01]; Female genital (1)[01]; General and Unspecified (2)[06, 26]
Cognates:
Language contact: Chiapas Zoq <> Tot

**_Gaultheria erecta_ Vent. (Ericaceae)**

Spanish names:
Indigenous names: Atres wamal[20]
Used by (1*): Western Maya[20]
Used for (1#): Digestive (1)[20]
Cognates:
Language contact:

**_Gaya calyptrata_ (Cav.) Kunth ex K.Schum. (Malvaceae)**

Spanish names:
Indigenous names: Xpupul-ik'[09]
Used by (1*): Yucatecan Maya[09]
Used for (2#): Digestive (1)[09]; Skin (1)[09]
Cognates:
Language contact:

**_Genipa americana_ L. (Rubiaceae)**

Spanish names: Maluku
Indigenous names: Nuk t¥m[03]
Used by (2*): Zoque[02, 03]
Used for (2#): Digestive (1)[02]; General and Unspecified (1)[03]
Cognates:
Language contact:

**_Geophila repens_ (L.) I.M.Johnst. (Rubiaceae)**

Spanish names:
Indigenous names: Ixim Q'en[14]
Used by (1*): Quichean Maya[14]
Used for (1#): Digestive (1)[14]
Cognates:
Language contact:

**_Geranium repens_ H.E.Moore (Geraniaceae)**

Spanish names: Sanícula
Indigenous names:
Used by (1*): Quichean Maya[12]
Used for (4#): Eye (1)[12]; Skin (1)[12]; Urological (1)[12]; General and Unspecified (1)[12]
Cognates:
Language contact:

**_Gibasis geniculata_ (Jacq.) Rohweder (Commelinaceae)**

Spanish names:
Indigenous names: Xwa ixul[14]
Used by (1*): Quichean Maya[14]
Used for (1#): Skin (1)[14]
Cognates:
Language contact:

**_Gibasis pellucida_ (M.Martens & Galeotti) D.R.Hunt (Commelinaceae)**

Spanish names:
Indigenous names: Madali[21]
Used by (1*): Zapotec[21]
Used for (1#): Skin (1)[21]
Cognates:
Language contact:

[01-28]refer to the study codes in Table 4.1. *Total number of studies citing this taxon #Total number of use-records

***Ginkgo biloba* L. (Ginkgoaceae)**

Spanish names: Gingko Biloba
Indigenous names:
Used by (1*): Quichean Maya[12]
Used for (4#): Cardiovascular (1)[12]; Musculoskeletal (1)[12]; Neurological (1)[12]; Psychological (1)[12]
Cognates:
Language contact:

***Gladiolus hortulanus* L.H. Bailey (Iridaceae)**

Spanish names: Gladiola
Indigenous names:
Used by (1*): Quichean Maya[12]
Used for (2#): Skin (1)[12]; General and Unspecified (1)[12]
Cognates:
Language contact:

***Glandularia bipinnatifida* (Schauer) Nutt. (Verbenaceae)**

Spanish names: Alfombrilla cimarrón
Indigenous names:
Used by (1*): Nahua[26]
Used for (1#): Skin (1)[26]
Cognates:
Language contact:

***Glandularia delticola* (Small ex Perry) Umber (Verbenaceae)**

Spanish names:
Indigenous names: Waleklaab ts'ohool, ehek walek, tsamnek ts'ohool, thuhal ts'ohool, thuhual ichich[07]
Used by (1*): Huastec[07]
Used for (2#): Skin (1)[07]; General and Unspecified (1)[07]
Cognates:
Language contact:

***Glandularia* sp. (Verbenaceae)**

Spanish names:
Indigenous names: Ncuàan-dzéb-cônch, guìzh-cônch, guìzh-ncuàan-x-cônch[23]
Used by (1*): Zapotec[23]
Used for (1#): General and Unspecified (1)[23]
Cognates:
Language contact:

***Gliricidia sepium* (Jacq.) Walp. (Fabaceae)**

Spanish names: Madrecacao, cocohuite
Indigenous names: Tzang kuy/oyo nuk/sawin kuy[01]; Tzawin kuy/ jäyä kuy[02]; Paaki[03]; Tsøøk[04]; Zakh'akh'a tasun[08]; Kante'[14]; Aj chänte'[18]; K'an te[19]; Guianixa'a[21]
Used by (10*): Zoque[01, 02, 03]; Mixe[04]; Totonac[05]; Huastec[08]; Quichean Maya[14]; Western Maya[18, 19]; Zapotec[21]
Used for (26#): Digestive (2)[01, 02]; Eye (1)[18]; Neurological (4)[01, 02, 03, 19]; Psychological (1)[21]; Respiratory (3)[05, 08, 18]; Skin (4)[02, 03, 19, 21]; Urological (2)[02, 21]; General and Unspecified (9)[01, 02, 03, 04, 05, 14, 18, 19, 21]
Cognates: MZ: tsVk; Zoq: sawin kuy; CoreM: kante;
Language contact: MZ > Hua

**_Gnaphalium americanum_ Mill. (Asteraceae)**

Spanish names: Gordolobo, Sanalotodo
Indigenous names: B'ojq'aos[12]
Used by (1*): Quichean Maya[12]
Used for (4#): Respiratory (1)[12]; Skin (1)[12]; Female genital (1)[12]; General and Unspecified (1)[12]
Cognates:
Language contact:

**_Gnaphalium attenuatum_ DC. (Asteraceae)**

Spanish names: Gordolobo; Gordolobo, Sanalotodo
Indigenous names: Poma ay cimarrón[03]; Wo'opt aay[04]
Used by (3*): Zoque[03]; Mixe[04]; Quichean Maya[12]
Used for (11#): Digestive (2)[04, 12]; Neurological (1)[12]; Respiratory (2)[03, 12]; Skin (2)[03, 12]; Pregnancy (1)[03]; Female genital (1)[12]; General and Unspecified (2)[03, 12]
Cognates:
Language contact:

**_Gnaphalium_ sp. (Asteraceae)**

Spanish names: Algodoncito; Gordolobo; Gordolobo, flor de seda, sanalotodo
Indigenous names: Uxkin imul, uxquin imul[13]; Tafiatu gueexii[21]; Ixcaxihuitl[26]
Used by (3*): Quichean Maya[13]; Zapotec[21]; Nahua[26]
Used for (8#): Digestive (1)[21]; Respiratory (2)[13, 21]; Skin (3)[13, 21, 26]; Pregnancy (1)[21]; General and Unspecified (1)[21]
Cognates:
Language contact: K'iche' <> Nah

**_Gnaphalium viscosum_ Kunth (Asteraceae)**

Spanish names: Gordolobo macho
Indigenous names: Saq' Mak'el Q'os[12]
Used by (1*): Quichean Maya[12]
Used for (2#): Respiratory (1)[12]; Skin (1)[12]
Cognates:
Language contact:

**_Godmania aesculifolia_ (Kunth) Standl. (Bignoniaceae)**

Spanish names:
Indigenous names: Xo'k'ab[09]
Used by (1*): Yucatecan Maya[09]
Used for (2#): Pregnancy (1)[09]; Female genital (1)[09]
Cognates:
Language contact:

**_Gomphrena diffusa_ (R.Br.) Spreng. (Amaranthaceae)**

Spanish names:
Indigenous names: Guìzh-guiĕt-nì[23]
Used by (1*): Zapotec[23]
Used for (2#): Digestive (1)[23]; Urological (1)[23]
Cognates:
Language contact:

**_Gomphrena globosa_ L. (Amaranthaceae)**

Spanish names: Amor Secco; Tinta roja
Indigenous names: J¥pak mooya[03]; T'oyol[07]; Pitunche[12]; Ku' qehen[14]
Used by (4*): Zoque[03]; Huastec[07]; Quichean Maya[12, 14]
Used for (7#): Digestive (2)[07, 14]; Psychological (1)[12]; Pregnancy (1)[12]; Female genital (1)[03]; General and Unspecified (2)[03, 12]
Cognates:
Language contact:

[01-28]refer to the study codes in Table 4.1. *Total number of studies citing this taxon #Total number of use-records

**_Gomphrena nitida_ Rothr. (Amaranthaceae)**

Spanish names:
Indigenous names: Gueyana[21]
Used by (1*): Zapotec[21]
Used for (2#): Digestive (1)[21]; General and Unspecified (1)[21]
Cognates:
Language contact:

**_Gomphrena serrata_ L. (Amaranthaceae)**

Spanish names:
Indigenous names:
Used by (1*): Zoque[03]
Used for (2#): Urological (1)[03]; General and Unspecified (1)[03]
Cognates:
Language contact:

**_Gomphrena_ sp. (Amaranthaceae)**

Spanish names:
Indigenous names:
Used by (1*): Zoque[03]
Used for (1#): Pregnancy (1)[03]
Cognates:
Language contact:

**_Goniophlebium furfuraceum_ (Schltdl. & Cham.) T. Moore (Polypodiaceae)**

Spanish names: Doradilla
Indigenous names:
Used by (1*): Zoque[01]
Used for (1#): General and Unspecified (1)[01]
Cognates:
Language contact:

**_Gonolobus barbatus_ Kunth (Apocynaceae)**

Spanish names:
Indigenous names: Kontua[02]
Used by (1*): Zoque[02]
Used for (2#): Female genital (1)[02]; General and Unspecified (1)[02]
Cognates:
Language contact:

**_Gonolobus niger_ (Cav.) Schult. (Apocynaceae)**

Spanish names: Yoyos
Indigenous names: U'ii[06]; Ooy[07]
Used by (2*): Totonac[06]; Huastec[07]
Used for (2#): Digestive (1)[06]; Skin (1)[07]
Cognates:
Language contact: Tot <> Hua

**_Gonolobus_ sp. (Apocynaceae)**

Spanish names: Candua
Indigenous names: Batuguexe'e[21]
Used by (1*): Zapotec[21]
Used for (1#): General and Unspecified (1)[21]
Cognates:
Language contact:

[01-28] refer to the study codes in Table 4.1. *Total number of studies citing this taxon #Total number of use-records

***Gonzalagunia panamensis* (Cav.) K.Schum. (Rubiaceae)**

Spanish names:
Indigenous names: Tzuul che'[17]
Used by (1*): Quichean Maya[17]
Used for (1#): nd[17]
Cognates:
Language contact:

***Gonzalagunia rosea* Standl. (Rubiaceae)**

Spanish names:
Indigenous names: Tzu'ul che[16]
Used by (1*): Quichean Maya[16]
Used for (1#): Neurological (1)[16]
Cognates:
Language contact:

***Gossypium barbadense* L. (Malvaceae)**

Spanish names: Algodón
Indigenous names:
Used by (1*): Zoque[01]
Used for (1#): Urological (1)[01]
Cognates:
Language contact:

***Gossypium hirsutum* L. (Malvaceae)**

Spanish names: Algodón
Indigenous names: Kwinim, tsokoy[07]; Taman[09]; Taman[10]; Noq'[14]; Shiila'a[21]; Yàg-zhĭil[23]
Used by (6*): Huastec[07]; Yucatecan Maya[09, 10]; Quichean Maya[14]; Zapotec[21, 23]
Used for (15#): Digestive (2)[07, 21]; Musculoskeletal (2)[07, 21]; Respiratory (4)[07, 09, 10, 14]; Skin (2)[07, 21]; Urological (2)[07, 21]; Pregnancy (1)[07]; General and Unspecified (2)[21, 23]
Cognates: Yuca: taman; Zapo: xiil;
Language contact:

***Gossypium* sp. (Malvaceae)**

Spanish names:
Indigenous names: Chuy-taman[09]
Used by (1*): Yucatecan Maya[09]
Used for (1#): Respiratory (1)[09]
Cognates:
Language contact:

***Gouania lupuloides* (L.) Urb. (Rhamnaceae)**

Spanish names: Jaboncillo liso
Indigenous names: Tzaku pu/Tzaj upu [01]; Xiapun tsay[03]; X-om-ak'[09]; Ch'jom k'aham[17]
Used by (4*): Zoque[01, 03]; Yucatecan Maya[09]; Quichean Maya[17]
Used for (4#): Skin (3)[01, 03, 09]; nd[17]
Cognates: Zoq: tsapu/xiapi; CoreM: xom ak/k'aham;
Language contact:

***Gouania polygama* (Jacq.) Urb. (Rhamnaceae)**

Spanish names: Jaboncillo liso
Indigenous names: Xiapun tsay[03]; Xajts oo'ts aats[04]; Thuhal ts'aah[07]
Used by (3*): Zoque[03]; Mixe[04]; Huastec[07]
Used for (7#): Musculoskeletal (1)[03]; Skin (3)[03, 04, 07]; Female genital (1)[03]; General and Unspecified (2)[03, 07]
Cognates:
Language contact:

[01-28]refer to the study codes in Table 4.1. *Total number of studies citing this taxon #Total number of use-records

**_Govenia alba_ A. Rich. & Galeotti (Orchidaceae)**

Spanish names: Flor blanca
Indigenous names: Popoj jäyä[01]
Used by (1*): Zoque[01]
Used for (1#): Digestive (1)[01]
Cognates:
Language contact:

**_Grevillea robusta_ A.Cunn. ex R.Br. (Proteaceae)**

Spanish names: Grabilea
Indigenous names:
Used by (1*): Quichean Maya[12]
Used for (1#): Psychological (1)[12]
Cognates:
Language contact:

**_Gronovia scandens_ L. (Loasaceae)**

Spanish names: Chaya
Indigenous names: Tzis kä wang [02]; Laalmuch[09]
Used by (2*): Zoque[02]; Yucatecan Maya[09]
Used for (2#): Musculoskeletal (1)[09]; Neurological (1)[02]
Cognates:
Language contact:

**_Guadua angustifolia_ Kunth (Poaceae)**

Spanish names:
Indigenous names: Tsahib, chahib[07]
Used by (1*): Huastec[07]
Used for (4#): Musculoskeletal (1)[07]; Skin (1)[07]; Pregnancy (1)[07]; General and Unspecified (1)[07]
Cognates:
Language contact:

**_Guaiacum coulteri_ A.Gray (Zygophyllaceae)**

Spanish names: Guayacan
Indigenous names: Hueycán[08]
Used by (1*): Huastec[08]
Used for (1#): Endocrine (1)[08]
Cognates:
Language contact:

**_Guaiacum sanctum_ L. (Zygophyllaceae)**

Spanish names: Balsamo
Indigenous names:
Used by (1*): Zapotec[21]
Used for (2#): Skin (1)[21]; General and Unspecified (1)[21]
Cognates:
Language contact:

**_Guarea guidonia_ (L.) Sleumer (Meliaceae)**

Spanish names:
Indigenous names: Bolb[16]
Used by (1*): Quichean Maya[16]
Used for (1#): General and Unspecified (1)[16]
Cognates:
Language contact:

***Guazuma ulmifolia*** **Lam. (Malvaceae)**

Spanish names: Caulote, guácimo
Indigenous names: Topaku/tzapas äkö/äkä[01]; Äkä[02]; ¥k¥[03]; Øøk[04]; Akgexta[06]; Akich[07]; Pixoy, Nohoch-pixoy[09]; Pixoy[11]; Tchabei[14]; Ajxuyuy[18]; Ch'ab'ay[19]; Yana'a[21]
Used by (12*): Zoque[01, 02, 03]; Mixe[04]; Totonac[06]; Huastec[07]; Yucatecan Maya[09, 11]; Quichean Maya[14]; Western Maya[18, 19]; Zapotec[21]
Used for (34#): Blood (1)[03]; Digestive (11)[01, 02, 03, 04, 06, 07, 11, 14, 18, 19, 21]; Cardiovascular (1)[18]; Neurological (1)[21]; Psychological (1)[18]; Respiratory (3)[02, 03, 07]; Skin (4)[02, 03, 07, 18]; Endocrine (1)[03]; Pregnancy (3)[03, 07, 09]; Female genital (3)[02, 03, 04]; Male genital (1)[01]; General and Unspecified (4)[02, 03, 07, 21]
Cognates: MZ: äk; Zoq: äkä; CoreM: chabay; Yuca: pixoy;
Language contact: MZ > Tot and Hua

***Gurania makoyana*** **(Lem.) Cogn. (Cucurbitaceae)**

Spanish names:
Indigenous names: Susum Sankh[14]; K'uum pim[17]
Used by (2*): Quichean Maya[14, 17]
Used for (2#): General and Unspecified (1)[14]nd[17]
Cognates: Quich: u(s)um;
Language contact:

***Gymnopodium floribundum*** **Rolfe (Polygonaceae)**

Spanish names:
Indigenous names: Ts'its'ilche'[09]
Used by (1*): Yucatecan Maya[09]
Used for (1#): Respiratory (1)[09]
Cognates:
Language contact:

***Gynerium sagittatum*** **(Aubl.) P.Beauv. (Poaceae)**

Spanish names: Caña brava
Indigenous names:
Used by (1*): Yucatecan Maya[10]
Used for (2#): Urological (1)[10]; Female genital (1)[10]
Cognates:
Language contact:

***Gypsacanthus nelsonii*** **E.J.Lott, V.Jaram. & Rzed. (Acanthaceae)**

Spanish names: Tiricia
Indigenous names:
Used by (1*): Nahua[27]
Used for (1#): General and Unspecified (1)[27]
Cognates:
Language contact:

***Gyrotaenia microcarpa*** **(Wess.) Fawc. & Rendle (Urticaceae)**

Spanish names: Mala mujer
Indigenous names: Si'in geno[01]
Used by (1*): Zoque[01]
Used for (1#): Skin (1)[01]
Cognates:
Language contact:

[01-28]refer to the study codes in Table 4.1. *Total number of studies citing this taxon #Total number of use-records

***Haematoxylum brasiletto* H.Karst. (Fabaceae)**

Spanish names: Brasil; Palo brasil
Indigenous names:
Used by (2*): Zoque[01]; Zapotec[21]
Used for (5#): Blood (1)[21]; Digestive (1)[21]; Endocrine (1)[01]; Urological (1)[01]; General and Unspecified (1)[21]
Cognates:
Language contact:

***Haematoxylum campechianum* L. (Fabaceae)**

Spanish names: Tinto
Indigenous names: Chäkte'[18]
Used by (1*): Western Maya[18]
Used for (2#): Digestive (1)[18]; General and Unspecified (1)[18]
Cognates:
Language contact:

***Hamelia axillaris* Sw. (Rubiaceae)**

Spanish names:
Indigenous names: Saq'ib Q'ehen[14]
Used by (1*): Quichean Maya[14]
Used for (1#): General and Unspecified (1)[14]
Cognates:
Language contact:

***Hamelia patens* Jacq. (Rubiaceae)**

Spanish names: Coralillo, chacloco
Indigenous names: Tondon ay/ tzan ay/ tzan jäyä[01]; Chochoday, Cangchocho, Cuma ay[03]; Maktantulonkis, akajetantulungush, tanchulukx kiui[05]; Tsak look', k'entsul te', chak ch'ohool, chak look'[07]; K'entsel te'[08]; Ele'kabi, K'anan[09]; Šk'anang[10]; Ik k'änän[11]; Ratzum Tz'unun, Ruk'a Tz'unun[14]; Chaj' mash[16]; Cacahuaxochitl[24]
Used by (15*): Zoque[01, 02, 03]; Totonac[05]; Huastec[07, 08]; Yucatecan Maya[09, 10, 11]; Quichean Maya[12, 14, 16]; Zapotec[21]; Nahua[24, 25]
Used for (50#): Blood (3)[05, 07, 08]; Digestive (7)[01, 05, 07, 08, 12, 24, 25]; Eye (1)[03]; Musculoskeletal (2)[01, 05]; Neurological (2)[05, 16]; Psychological (1)[12]; Respiratory (3)[01, 05, 14]; Skin (12)[01, 03, 05, 07, 08, 09, 10, 11, 14, 21, 24, 25]; Endocrine (4)[03, 05, 08, 12]; Urological (3)[02, 07, 25]; Pregnancy (1)[24]; Female genital (5)[03, 05, 14, 21, 24]; General and Unspecified (6)[01, 03, 05, 07, 12, 14]
Cognates: Mayan: kVn; Huas: kentsVl te; Yuca: k'anan;
Language contact: Hua > Tot and Highland Popoluca

***Hampea macrocarpa* Lundell (Malvaceae)**

Spanish names: Majahua
Indigenous names: Tsäkolte'[18]
Used by (1*): Western Maya[18]
Used for (3#): Digestive (1)[18]; Neurological (1)[18]; General and Unspecified (1)[18]
Cognates:
Language contact:

***Hanburia parviflora* Donn.Sm. (Cucurbitaceae)**

Spanish names:
Indigenous names: Wawi yomdak [01]
Used by (1*): Zoque[01]
Used for (1#): Skin (1)[01]
Cognates:
Language contact:

[01-28]refer to the study codes in Table 4.1. *Total number of studies citing this taxon #Total number of use-records

***Handroanthus chrysanthus* (Jacq.) S.O.Grose (Bignoniaceae)**

Spanish names: Guayacán
Indigenous names:
Used by (1*): Zoque[01]
Used for (1#): Endocrine (1)[01]
Cognates:
Language contact:

***Handroanthus guayacan* (Seem.) S.O.Grose (Bignoniaceae)**

Spanish names: Guayacán
Indigenous names: B'ajeer[14]; Uayakan[18]
Used by (2*): Quichean Maya[14]; Western Maya[18]
Used for (2#): Digestive (1)[14]; Endocrine (1)[18]
Cognates:
Language contact:

***Handroanthus impetiginosus* (Mart. ex DC.) Mattos (Bignoniaceae)**

Spanish names: Roble
Indigenous names:
Used by (1*): Zapotec[21]
Used for (2#): Musculoskeletal (1)[21]; Skin (1)[21]
Cognates:
Language contact:

***Harpalyce arborescens* A.Gray (Fabaceae)**

Spanish names:
Indigenous names: K'ante'[07]
Used by (1*): Huastec[07]
Used for (1#): Digestive (1)[07]
Cognates:
Language contact:

***Havardia albicans* (Kunth) Britton & Rose (Fabaceae)**

Spanish names:
Indigenous names: Chukum[09]
Used by (1*): Yucatecan Maya[09]
Used for (1#): Skin (1)[09]
Cognates:
Language contact:

***Hebanthe grandiflora* (Hook.) Borsch & Pedersen (Amaranthaceae)**

Spanish names:
Indigenous names: K¥kujuki ay[03]
Used by (1*): Zoque[03]
Used for (1#): Skin (1)[03]
Cognates:
Language contact:

***Hechtia podantha* Mez (Bromeliaceae)**

Spanish names: Lasolmic
Indigenous names: Laxomic[28]
Used by (1*): Nahua[28]
Used for (1#): Digestive (1)[28]
Cognates:
Language contact:

**_Hedeoma drummondii_ Benth. (Lamiaceae)**

Spanish names:
Indigenous names: Maal t'eel, tsakam polello[07]
Used by (1*): Huastec[07]
Used for (4#): Digestive (1)[07]; Respiratory (1)[07]; Pregnancy (1)[07]; General and Unspecified (1)[07]
Cognates:
Language contact:

**_Hedychium coronarium_ J.Koenig (Zingiberaceae)**

Spanish names: Mariposa
Indigenous names:
Used by (1*): Zoque[01]
Used for (3#): Musculoskeletal (1)[01]; Skin (1)[01]; General and Unspecified (1)[01]
Cognates:
Language contact:

**_Heimia salicifolia_ (Kunth) Link (Lythraceae)**

Spanish names: Sinicuichi; Sobadora
Indigenous names: Maan witsiil[07]; Ncuàan-yǎas[23]; Icxinicuiltzin[26]
Used by (4*): Huastec[07]; Zapotec[21, 23]; Nahua[26]
Used for (8#): Digestive (1)[26]; Musculoskeletal (3)[21, 23, 26]; Neurological (1)[26]; Skin (1)[23]; General and Unspecified (2)[07, 23]
Cognates:
Language contact: Nah <> Hua

**_Helenium mexicanum_ Kunth (Asteraceae)**

Spanish names: Floricilla
Indigenous names: Guièe-sàntàmàrǐ-mòntês, guièe-sàntàmàrǐ-dán, guièe-sàntàmàrǐ-nguěts, guièe-sàntàmàrǐ-gùts, guièe-sàntàmàrǐ-mòstêz[23]
Used by (2*): Quichean Maya[13]; Zapotec[23]
Used for (2#): Respiratory (2)[13, 23]
Cognates:
Language contact:

**_Helenium quadridentatum_ Labill. (Asteraceae)**

Spanish names:
Indigenous names: Hats'ix wits, hats'ix kw'eet, chemthak i ch'ak[07]
Used by (1*): Huastec[07]
Used for (1#): Skin (1)[07]
Cognates:
Language contact:

**_Helianthemum glomeratum_ (Lag.) Lag. ex Dunal (Cistaceae)**

Spanish names: Cenicero
Indigenous names: Xmasakh'e pak'a[08]; Vach' t'ul, tan vomol, tantan wamal, tan bak wamal, k'anal nich te', k'anal nich wach', vach' te' vomol, k'ujk'ul wamal[20]; Gbày-tæ̀, guìzh-òrêgànò-dán[23]
Used by (3*): Huastec[08]; Western Maya[20]; Zapotec[23]
Used for (12#): Digestive (2)[08, 20]; Eye (1)[20]; Ear (1)[20]; Musculoskeletal (2)[08, 20]; Respiratory (1)[20]; Skin (2)[20, 23]; Male genital (1)[20]; General and Unspecified (2)[20, 23]
Cognates: Mayan: pak;
Language contact:

[01-28]refer to the study codes in Table 4.1. *Total number of studies citing this taxon #Total number of use-records

***Heliconia latispatha* Benth. (Heliconiaceae)**

Spanish names: Platanillo
Indigenous names: Huahua[21]
Used by (1*): Zapotec[21]
Used for (1#): General and Unspecified (1)[21]
Cognates:
Language contact:

***Heliconia psittacorum* L.f. (Heliconiaceae)**

Spanish names: Calambre Negro
Indigenous names: Q'eq'i mox Kej [14]
Used by (1*): Quichean Maya[14]
Used for (2#): Digestive (1)[14]; Musculoskeletal (1)[14]
Cognates:
Language contact:

***Heliconia schiedeana* Klotzsch (Heliconiaceae)**

Spanish names:
Indigenous names: Lištampan[05]; Ts'umts'um, thulup[07]
Used by (2*): Totonac[05]; Huastec[07]
Used for (2#): Female genital (1)[07]; General and Unspecified (1)[05]
Cognates:
Language contact:

***Heliconia spissa* Griggs (Heliconiaceae)**

Spanish names:
Indigenous names: Tsabats ay[03]
Used by (1*): Zoque[03]
Used for (2#): Urological (1)[03]; Female genital (1)[03]
Cognates:
Language contact:

***Heliconia subulata* Ruiz & Pav. (Heliconiaceae)**

Spanish names:
Indigenous names: Q'eq'i mox Kej [14]
Used by (1*): Quichean Maya[14]
Used for (2#): Digestive (1)[14]; Musculoskeletal (1)[14]
Cognates:
Language contact:

***Helicteres baruensis* Jacq. (Malvaceae)**

Spanish names:
Indigenous names: Tsutup, Suput[09]
Used by (1*): Yucatecan Maya[09]
Used for (1#): Skin (1)[09]
Cognates:
Language contact:

***Helicteres guazumifolia* Kunth (Malvaceae)**

Spanish names:
Indigenous names: Ang Tzatz[02]; Piniaka[03]
Used by (2*): Zoque[02, 03]
Used for (4#): Neurological (1)[03]; Psychological (1)[02]; Pregnancy (1)[03]; Female genital (1)[03]
Cognates:
Language contact:

[01-28]refer to the study codes in Table 4.1. *Total number of studies citing this taxon #Total number of use-records

**_Heliocarpus americanus_ L. (Malvaceae)**

Spanish names: Jonote
Indigenous names: Po'a[02]; Panats[03]
Used by (2*): Zoque[02, 03]
Used for (5#): Digestive (1)[03]; Skin (2)[02, 03]; Urological (1)[03]; Pregnancy (1)[03]
Cognates:
Language contact:

**_Heliocarpus appendiculatus_ Turcz. (Malvaceae)**

Spanish names: Jonote; Palo de majagua
Indigenous names: Xunic[05]; Yaga lajsa'a[21]
Used by (2*): Totonac[05]; Zapotec[21]
Used for (5#): Digestive (1)[05]; Musculoskeletal (1)[21]; Skin (2)[05, 21]; Pregnancy (1)[05]
Cognates:
Language contact:

**_Heliocarpus donnellsmithii_ Rose (Malvaceae)**

Spanish names: Majagua; Majagua rojo
Indigenous names: (Tzapas) po'a[01]; Pa'ants[04]; Baat[07]; Lajtz[21]
Used by (4*): Zoque[01]; Mixe[04]; Huastec[07]; Zapotec[21]
Used for (9#): Digestive (1)[07]; Respiratory (1)[07]; Skin (4)[01, 04, 07, 21]; Pregnancy (1)[07]; General and Unspecified (2)[01, 07]
Cognates:
Language contact: MZ > Hua and Zap

**_Heliocarpus mexicanus_ (Turcz.) Sprague (Malvaceae)**

Spanish names:
Indigenous names: Ixhuaqué[24]
Used by (1*): Nahua[24]
Used for (1#): Pregnancy (1)[24]
Cognates:
Language contact:

**_Heliocarpus popayanensis_ Kunth (Malvaceae)**

Spanish names:
Indigenous names: Kaq'i B'ach[14]
Used by (1*): Quichean Maya[14]
Used for (3#): Urological (1)[14]; Pregnancy (1)[14]; General and Unspecified (1)[14]
Cognates:
Language contact:

**_Heliocarpus terebinthinaceus_ (DC.) Hochr. (Malvaceae)**

Spanish names: Majagua caballin
Indigenous names: Lajsa'a boogui[21]
Used by (1*): Zapotec[21]
Used for (3#): Digestive (1)[21]; Skin (1)[21]; General and Unspecified (1)[21]
Cognates:
Language contact:

**_Heliopsis buphthalmoides_ (Jacq.) Dunal (Asteraceae)**

Spanish names:
Indigenous names: Mansaana Q'ehen[14]
Used by (1*): Quichean Maya[14]
Used for (1#): General and Unspecified (1)[14]
Cognates:
Language contact:

***Heliotropium angiospermum* Murray (Boraginaceae)**

Spanish names: Cabeza de guajalote blanco; Rabomico
Indigenous names: Thiniy ts'ohool, weew thiniy[07]; Xnema'ax[09]; Guixamberu'u quitzii[21]
Used by (4*): Huastec[07]; Yucatecan Maya[09, 11]; Zapotec[21]
Used for (8#): Digestive (3)[09, 11, 21]; Neurological (1)[07]; Skin (2)[07, 21]; Pregnancy (1)[07]; Female genital (1)[21]
Cognates:
Language contact:

***Heliotropium fallax* I.M. Johnst. (Boraginaceae)**

Spanish names: Hoja de alacrán del monte
Indigenous names: Kaku'e ay[02]
Used by (1*): Zoque[02]
Used for (3#): Digestive (1)[02]; Cardiovascular (1)[02]; Female genital (1)[02]
Cognates:
Language contact:

***Heliotropium indicum* L. (Boraginaceae)**

Spanish names: Cola de alacrán
Indigenous names: Ma'ne' kinä/kaku'we ay[02]; Tunok kiñi[03]; No'ot ujts[04]; Nej ajpum[18]; Guixamberu'u [21]
Used by (6*): Zoque[02, 03]; Mixe[04]; Quichean Maya[14]; Western Maya[18]; Zapotec[21]
Used for (22#): Digestive (4)[02, 03, 18, 21]; Ear (1)[03]; Cardiovascular (1)[02]; Musculoskeletal (2)[02, 03]; Skin (4)[02, 03, 18, 21]; Urological (3)[02, 03, 14]; Pregnancy (1)[02]; Female genital (4)[02, 03, 04, 21]; Male genital (1)[02]; General and Unspecified (1)[21]
Cognates: Zoq: kinV;
Language contact:

***Heliotropium transalpinum* Vell. (Boraginaceae)**

Spanish names: Cola de alacrán/riñonina
Indigenous names: Kaku'e tuts [01]
Used by (1*): Zoque[01]
Used for (3#): Digestive (1)[01]; Skin (1)[01]; Urological (1)[01]
Cognates:
Language contact:

***Hemionitis palmata* L. (Pteridaceae)**

Spanish names: Hierba del gato
Indigenous names: Guish misht'daj'[21]
Used by (1*): Zapotec[21]
Used for (2#): Skin (1)[21]; General and Unspecified (1)[21]
Cognates:
Language contact:

***Henriettella cuneata* (Standl.) Gleason (Melastomataceae)**

Spanish names:
Indigenous names: Ixq Q'een[15]
Used by (1*): Quichean Maya[15]
Used for (1#): Pregnancy (1)[15]
Cognates:
Language contact:

***Herissantia crispa* (L.) Brizicky (Malvaceae)**

Spanish names:
Indigenous names:
Used by (1*): Huastec[07]
Used for (1#): Urological (1)[07]
Cognates:
Language contact:

[01-28]refer to the study codes in Table 4.1. *Total number of studies citing this taxon #Total number of use-records

**_Heterocentron subtriplinervium_ (Link & Otto) A. Braun & C.D. Bouché (Melastomataceae)**

Spanish names: Caña agria; Caña de Cristo
Indigenous names: Tzapas mankuy yajkuy[02]; Chäm ajij[12]
Used by (2*): Zoque[02]; Quichean Maya[12]
Used for (11#): Blood (1)[12]; Digestive (1)[12]; Cardiovascular (1)[12]; Musculoskeletal (1)[12]; Respiratory (1)[12]; Skin (1)[02]; Endocrine (2)[02, 12]; Urological (1)[02]; Female genital (1)[12]; General and Unspecified (1)[12]
Cognates:
Language contact:

**_Heteropterys laurifolia_ (L.) A. Juss. (Malpighiaceae)**

Spanish names:
Indigenous names: Patan tsay, Tsay uouo, T¥ts kunekne[03]
Used by (1*): Zoque[03]
Used for (3#): Digestive (1)[03]; Urological (1)[03]; Female genital (1)[03]
Cognates:
Language contact:

**_Heteropterys_ sp. (Malpighiaceae)**

Spanish names:
Indigenous names: Meeme ay[03]
Used by (1*): Zoque[03]
Used for (3#): Digestive (1)[03]; Urological (1)[03]; Female genital (1)[03]
Cognates:
Language contact:

**_Heterotheca inuloides_ Cass. (Asteraceae)**

Spanish names: Arnica
Indigenous names:
Used by (2*): Nahua[25, 26]
Used for (7#): Digestive (1)[25]; Musculoskeletal (2)[25, 26]; Skin (2)[25, 26]; Urological (1)[25]; General and Unspecified (1)[25]
Cognates:
Language contact:

**_Hevea brasiliensis_ Müll. Arg. (Euphorbiaceae)**

Spanish names: Pindule
Indigenous names: Juñi[03]
Used by (1*): Zoque[03]
Used for (1#): Skin (1)[03]
Cognates:
Language contact:

**_Hibiscus costatus_ A. Rich. (Malvaceae)**

Spanish names: Algodoncillo
Indigenous names: Tioch puk ay[03]
Used by (1*): Zoque[03]
Used for (1#): Digestive (1)[03]
Cognates:
Language contact:

**_Hibiscus lavateroides_ Moric. (Malvaceae)**

Spanish names:
Indigenous names: Thoot wits, manath ts'ohool, uxumlaab ts'ohool[07]
Used by (1*): Huastec[07]
Used for (2#): Pregnancy (1)[07]; Female genital (1)[07]
Cognates:
Language contact:

***Hibiscus rosa-sinensis* L. (Malvaceae)**

Spanish names: Tulipán
Indigenous names: Tulipaan[07]; Utz Uj'[15]
Used by (11*): Zoque[01, 02, 03]; Huastec[07]; Yucatecan Maya[09]; Quichean Maya[14, 15, 16]; Western Maya[18]; Zapotec[21]; Nahua[24]
Used for (24#): Digestive (2)[14, 18]; Neurological (2)[03, 16]; Psychological (3)[02, 15, 24]; Respiratory (5)[01, 02, 03, 09, 21]; Skin (1)[02]; Urological (1)[02]; Pregnancy (3)[14, 15, 24]; Female genital (2)[01, 03]; General and Unspecified (5)[01, 02, 03, 07, 21]
Cognates:
Language contact:

***Hibiscus sabdariffa* L. (Malvaceae)**

Spanish names: Flor de Jamaica, rosa de Jamaica
Indigenous names:
Used by (7*): Zoque[01, 02]; Totonac[06]; Quichean Maya[12, 13]; Western Maya[18]; Zapotec[21]
Used for (16#): Blood (2)[12, 21]; Digestive (2)[12, 21]; Cardiovascular (1)[01]; Musculoskeletal (1)[12]; Neurological (1)[12]; Endocrine (1)[12]; Urological (4)[01, 02, 12, 18]; General and Unspecified (4)[06, 12, 13, 21]
Cognates:
Language contact:

***Hibiscus uncinellus* Moc. & Sessé ex DC. (Malvaceae)**

Spanish names: Tulipan del cerro
Indigenous names:
Used by (1*): Zapotec[21]
Used for (1#): Respiratory (1)[21]
Cognates:
Language contact:

***Hidalgoa ternata* La Llave (Asteraceae)**

Spanish names: Cogollo de sarpullido
Indigenous names: Ting puy tzäpä[02]; Pitx ay[03]
Used by (2*): Zoque[02, 03]
Used for (3#): Eye (1)[03]; Skin (2)[02, 03]
Cognates:
Language contact:

***Hilaria ciliata* (Scribn.) Sohns (Poaceae)**

Spanish names:
Indigenous names:
Used by (1*): Huastec[07]
Used for (1#): Digestive (1)[07]
Cognates:
Language contact:

***Hintonia lumaeana* (Baill.) Bullock (Rubiaceae)**

Spanish names: Quina / Hombre Grande
Indigenous names: K'ailaj Che'[12]
Used by (1*): Quichean Maya[12]
Used for (4#): Digestive (1)[12]; Respiratory (1)[12]; Endocrine (1)[12]; General and Unspecified (1)[12]
Cognates:
Language contact:

[01-28]refer to the study codes in Table 4.1. *Total number of studies citing this taxon [#]Total number of use-records

***Hippobroma longiflora* (L.) G.Don (Campanulaceae)**

Spanish names: Acapa de monte
Indigenous names: Samat' pim[14]
Used by (2*): Quichean Maya[14, 17]
Used for (6#): Digestive (1)[14]; Musculoskeletal (1)[14]; Neurological (1)[14]; Skin (1)[14]; General and Unspecified (1)[14]; nd[17]
Cognates:
Language contact:

***Hirtella racemosa* Lam. (Chrysobalanaceae)**

Spanish names:
Indigenous names: Tsus pet cuy[03]
Used by (1*): Zoque[03]
Used for (1#): Digestive (1)[03]
Cognates:
Language contact:

***Hirtella triandra* Sw. (Chrysobalanaceae)**

Spanish names:
Indigenous names: T¥kchi cuy[03]
Used by (1*): Zoque[03]
Used for (1#): Female genital (1)[03]
Cognates:
Language contact:

***Hoffmannia ghiesbreghtii* (Lem.) Hemsl. (Rubiaceae)**

Spanish names: Hoja de rayo
Indigenous names: Mä tane, mä teksi[01]
Used by (1*): Zoque[01]
Used for (2#): Skin (1)[01]; General and Unspecified (1)[01]
Cognates:
Language contact:

***Hoffmannia nicotianifolia* (M. Martens et Galeotti) L. O. Williams (Rubiaceae)**

Spanish names:
Indigenous names: Masan ay, Ts¥¥b ay[03]
Used by (1*): Zoque[03]
Used for (1#): Skin (1)[03]
Cognates:
Language contact:

***Hoffmannia rotata* Donn.Sm. (Rubiaceae)**

Spanish names: Calechilla
Indigenous names:
Used by (1*): Nahua[25]
Used for (1#): Ear (1)[25]
Cognates:
Language contact:

***Hordeum vulgare* L. (Poaceae)**

Spanish names: Cebada
Indigenous names:
Used by (5*): Zoque[01, 02]; Quichean Maya[12, 13]; Western Maya[19]
Used for (10#): Digestive (2)[01, 13]; Neurological (1)[13]; Urological (2)[01, 12]; Male genital (1)[12]; General and Unspecified (4)[02, 12, 13, 19]
Cognates:
Language contact:

[01-28]refer to the study codes in Table 4.1. *Total number of studies citing this taxon #Total number of use-records

***Humulus lupulus* L. (Cannabaceae)**

Spanish names: Lúpulo
Indigenous names: Musuche' Aq'om[12]
Used by (1*): Quichean Maya[12]
Used for (5#): Digestive (1)[12]; Cardiovascular (1)[12]; Musculoskeletal (1)[12]; Psychological (1)[12]; Urological (1)[12]
Cognates:
Language contact:

***Hura polyandra* Baill. (Euphorbiaceae)**

Spanish names: Empurga; Jabilla
Indigenous names:
Used by (2*): Mixe[04]; Zapotec[21]
Used for (2#): Digestive (2)[04, 21]
Cognates:
Language contact:

***Hybanthus attenuatus* (Humb. & Bonpl. ex Schult.) Schulze-Menz (Violaceae)**

Spanish names: Hierba de San Antonio; Trébol; Yerba familia
Indigenous names:
Used by (3*): Quichean Maya[13]; Western Maya[18]; Nahua[24]
Used for (4#): Ear (1)[13]; Musculoskeletal (1)[13]; Pregnancy (1)[24]; General and Unspecified (1)[18]
Cognates:
Language contact:

***Hybanthus oppositifolius* (L.) Taub. (Violaceae)**

Spanish names:
Indigenous names: Nekx cuy[03]; T'ithith t'eel[07]
Used by (2*): Zoque[03]; Huastec[07]
Used for (3#): Digestive (1)[07]; Skin (1)[03]; General and Unspecified (1)[07]
Cognates:
Language contact:

***Hybanthus thiemei* (Donn. Sm.) C.V. Morton (Violaceae)**

Spanish names: Hoja de verruga
Indigenous names: Xpluxion xiw[09]
Used by (2*): Zoque[03]; Yucatecan Maya[09]
Used for (3#): Neurological (1)[09]; Skin (1)[03]; Pregnancy (1)[03]
Cognates:
Language contact:

***Hybanthus yucatanensis* Millsp. (Violaceae)**

Spanish names:
Indigenous names: Sakbakelkam[09]
Used by (1*): Yucatecan Maya[09]
Used for (1#): Skin (1)[09]
Cognates:
Language contact:

***Hydrangea macrophylla* (Thunb.) Ser. (Hydrangeaceae)**

Spanish names: Hortensia
Indigenous names:
Used by (1*): Quichean Maya[13]
Used for (1#): Neurological (1)[13]
Cognates:
Language contact:

***Hydrocotyle mexicana* Schltdl. & Cham. (Araliaceae)**

Spanish names:
Indigenous names: Tzyokolate tane[01]; Makmak nab wamal[20]
Used by (2*): Zoque[01]; Western Maya[20]
Used for (2#): Digestive (1)[20]; Skin (1)[01]
Cognates:
Language contact:

***Hygrophila* sp. (Acanthaceae)**

Spanish names:
Indigenous names: Re Kanteel[14]
Used by (1*): Quichean Maya[14]
Used for (1#): General and Unspecified (1)[14]
Cognates:
Language contact:

***Hylocereus minutiflorus* Britton & Rose (Cactaceae)**

Spanish names:
Indigenous names: Chik'ba'l b'ak[17]
Used by (1*): Quichean Maya[17]
Used for (1#): nd[17]
Cognates:
Language contact:

***Hylocereus undatus* (Haw.) Britton & Rose (Cactaceae)**

Spanish names: Pitahaya
Indigenous names: Tsatsa', chacha[07]; Tuna rexte' juyub'[13]
Used by (6*): Huastec[07]; Yucatecan Maya[09]; Quichean Maya[12, 13]; Nahua[24, 27]
Used for (10#): Digestive (2)[09, 13]; Psychological (1)[12]; Respiratory (1)[27]; Skin (2)[07, 13]; Endocrine (1)[12]; Pregnancy (2)[07, 24]; General and Unspecified (1)[12]
Cognates:
Language contact:

***Hymenaea courbaril* L. (Fabaceae)**

Spanish names: Guapinol; Guapinole
Indigenous names: Kuy po'te[02]; Payi[03]; Ak pej[04]; Pakay[19]; Biguu[21]
Used by (5*): Zoque[02, 03]; Mixe[04]; Western Maya[19]; Zapotec[21]
Used for (15#): Digestive (4)[02, 03, 04, 21]; Musculoskeletal (1)[21]; Respiratory (3)[03, 04, 21]; Skin (1)[04]; Endocrine (2)[02, 03]; Urological (1)[19]; Pregnancy (1)[19]; Female genital (1)[19]; General and Unspecified (1)[21]
Cognates: MZ: po'te/payi/pej;
Language contact: MZ > Chortí and Zap

***Hymenocallis* sp. (Amaryllidaceae)**

Spanish names:
Indigenous names: Lakuum[07]
Used by (1*): Huastec[07]
Used for (1#): Skin (1)[07]
Cognates:
Language contact:

[01-28]refer to the study codes in Table 4.1. *Total number of studies citing this taxon #Total number of use-records

***Hyperbaena mexicana* Miers (Menispermaceae)**

Spanish names: Huesillo; Zapote blanco
Indigenous names: Txa cuy[03]
Used by (2*): Zoque[02, 03]
Used for (4#): Cardiovascular (1)[02]; Skin (1)[03]; Endocrine (1)[02]; Urological (1)[03]
Cognates:
Language contact:

***Hypericum epigeium* R.Keller (Hypericaceae)**

Spanish names: Hipérico
Indigenous names:
Used by (1*): Quichean Maya[12]
Used for (1#): Psychological (1)[12]
Cognates:
Language contact:

***Hypericum moranense* Kunth (Hypericaceae)**

Spanish names:
Indigenous names: H¥xi ay[03]
Used by (1*): Zoque[03]
Used for (3#): Neurological (1)[03]; Psychological (1)[03]; Skin (1)[03]
Cognates:
Language contact:

***Hypericum perforatum* L. (Hypericaceae)**

Spanish names: Hipérico
Indigenous names:
Used by (1*): Quichean Maya[12]
Used for (1#): Psychological (1)[12]
Cognates:
Language contact:

***Hypericum silenoides* Juss. (Hypericaceae)**

Spanish names: Pericón del monte
Indigenous names:
Used by (1*): Zoque[02]
Used for (3#): Digestive (1)[02]; Pregnancy (1)[02]; General and Unspecified (1)[02]
Cognates:
Language contact:

***Hypericum thesiifolium* Kunth (Hypericaceae)**

Spanish names: Hierba del susto, hierba de San Antonio
Indigenous names:
Used by (1*): Quichean Maya[13]
Used for (2#): Digestive (1)[13]; General and Unspecified (1)[13]
Cognates:
Language contact:

***Hypoxis decumbens* L. (Hypoxidaceae)**

Spanish names:
Indigenous names: Ceboll pim, Seb'oyil pim, Cewoyil pim[14]
Used by (1*): Quichean Maya[14]
Used for (1#): Respiratory (1)[14]
Cognates:
Language contact:

**_Hyptis albida_ Kunth (Lamiaceae)**

Spanish names: Pie de tortola
Indigenous names: Gujcu'u, guichu'[21]
Used by (1*): Zapotec[21]
Used for (1[#]): Digestive (1)[21]
Cognates:
Language contact:

**_Hyptis capitata_ Jacq. (Lamiaceae)**

Spanish names:
Indigenous names: Tam juñi[03]; Tem Kik'[14]; Se' ru'j kaway[17]
Used by (3*): Zoque[03]; Quichean Maya[14, 17]
Used for (5[#]): Skin (1)[03]; Urological (1)[03]; Female genital (2)[03, 14]; nd[17]
Cognates:
Language contact: Highland Popoluca <> Kekchí

**_Hyptis mutabilis_ (Rich.) Briq. (Lamiaceae)**

Spanish names: Hierba de toro; Lavanda; Palo de menta
Indigenous names:
Used by (3*): Quichean Maya[12]; Zapotec[21]; Nahua[25]
Used for (6[#]): Digestive (1)[12]; Neurological (1)[12]; Skin (1)[25]; Female genital (1)[21]; General and Unspecified (2)[12, 25]
Cognates:
Language contact:

**_Hyptis pectinata_ (L.) Poit. (Lamiaceae)**

Spanish names: Cedrón; Miltomate
Indigenous names: Tsak maape'[07]
Used by (3*): Huastec[07]; Quichean Maya[12]; Nahua[26]
Used for (7[#]): Digestive (2)[12, 26]; Respiratory (1)[12]; Skin (2)[07, 12]; Female genital (1)[26]; General and Unspecified (1)[12]
Cognates:
Language contact:

**_Hyptis_ sp. (Lamiaceae)**

Spanish names: Menta
Indigenous names: Xta'ulum, Oregano KaX[09]; Xtye caway[16]
Used by (3*): Yucatecan Maya[09]; Quichean Maya[12, 16]
Used for (5[#]): Digestive (2)[09, 12]; Psychological (2)[12, 16]; Skin (1)[12]
Cognates: CoreM: xtV;
Language contact:

**_Hyptis suaveolens_ (L.) Poit. (Lamiaceae)**

Spanish names: Balsamo de campo/ balsamito; Hierba de toro; Hierba toro
Indigenous names: Naso'o mona[01]; Oyo ton/wakas ay[02]; Chan[12]
Used by (4*): Zoque[01, 02]; Quichean Maya[12]; Zapotec[21]
Used for (14[#]): Digestive (3)[01, 02, 21]; Musculoskeletal (3)[01, 02, 21]; Respiratory (2)[02, 21]; Skin (1)[21]; Urological (1)[12]; Pregnancy (1)[02]; General and Unspecified (3)[02, 12, 21]
Cognates: Zoq: o'o Con;
Language contact: Zoq > Kaqchikel

**_Hyptis tomentosa_ Poit. (Lamiaceae)**

Spanish names: La peludita
Indigenous names: Osyi ay[01]; Guichu'[21]
Used by (2*): Zoque[01]; Zapotec[21]
Used for (2[#]): Digestive (1)[21]; Urological (1)[01]
Cognates:
Language contact:

[01-28]refer to the study codes in Table 4.1. *Total number of studies citing this taxon [#]Total number of use-records

***Hyptis urticoides* Kunth (Lamiaceae)**

Spanish names: Alúcema blanca
Indigenous names:
Used by (1*): Quichean Maya[12]
Used for (3#): Neurological (1)[12]; Skin (1)[12]; General and Unspecified (1)[12]
Cognates:
Language contact:

***Hyptis verticillata* Jacq. (Lamiaceae)**

Spanish names: Hierba San Martín
Indigenous names: Tzitzirane/wekpa tane[01]; Tzapas pa'petkuy[02]; Tsutsbet cuy[03]; San Martin ujts[04]; Santalipa, sunalipaljni[05]; Tihtsan ts'ohool, pithomlaam ts'ohool[07]; Pitomlab ts'ojol[08]; Xkis Kawaay [14]; Chu pim[15]; Chu' pim[17]; Tsuk pimi[18]; Guixa'a[21]
Used by (14*): Zoque[01, 02, 03]; Mixe[04]; Totonac[05]; Huastec[07, 08]; Yucatecan Maya[11]; Quichean Maya[14, 15, 17]; Western Maya[18, 19]; Zapotec[21]
Used for (51#): Digestive (7)[02, 03, 04, 07, 08, 18, 21]; Musculoskeletal (7)[01, 03, 08, 14, 18, 19, 21]; Neurological (3)[01, 04, 18]; Psychological (3)[01, 02, 11]; Respiratory (5)[01, 02, 03, 08, 14]; Skin (5)[01, 02, 03, 04, 21]; Endocrine (1)[01]; Urological (1)[02]; Pregnancy (5)[01, 03, 07, 15, 21]; Female genital (3)[01, 02, 15]; General and Unspecified (10)[01, 02, 03, 05, 07, 11, 14, 18, 19, 21]; nd[17]
Cognates: Zoq: petkuy; Huas: pitomla(C) tsoCol; CoreM: chu/tsu pim; Quich: chu pim;
Language contact:

***Ibervillea millspaughii* (Cogn.) C. Jeffrey (Cucurbitaceae)**

Spanish names:
Indigenous names: K'umkanul[09]
Used by (1*): Yucatecan Maya[09]
Used for (2#): Musculoskeletal (1)[09]; Skin (1)[09]
Cognates:
Language contact:

***Ibervillea* sp. (Cucurbitaceae)**

Spanish names:
Indigenous names: Thokob ts'een[07]
Used by (1*): Huastec[07]
Used for (1#): Skin (1)[07]
Cognates:
Language contact:

***Illicium verum* Hook.f. (Schisandraceae)**

Spanish names: Anís de estrella
Indigenous names: Ptsìidz[23]
Used by (8*): Zoque[01, 02]; Mixe[04]; Yucatecan Maya[09]; Quichean Maya[12]; Zapotec[21, 22, 23]
Used for (12#): Digestive (6)[01, 02, 04, 09, 21, 22]; Cardiovascular (1)[12]; Psychological (2)[01, 12]; Respiratory (1)[01]; Pregnancy (1)[21]; General and Unspecified (1)[23]
Cognates:
Language contact:

***Impatiens balsamina* L. (Balsaminaceae)**

Spanish names: Maravilla
Indigenous names: Espiritu ujts[04]
Used by (2*): Zoque[03]; Mixe[04]
Used for (5#): Ear (1)[03]; Neurological (1)[04]; Respiratory (1)[03]; Skin (1)[03]; General and Unspecified (1)[04]
Cognates:
Language contact:

***Impatiens walleriana* Hook.f. (Balsaminaceae)**

Spanish names: Quinze añera
Indigenous names: China wits[07]
Used by (2*): Huastec[07]; Quichean Maya[13]
Used for (3#): Eye (1)[13]; Skin (1)[13]; Pregnancy (1)[07]
Cognates:
Language contact:

***Imperata brasiliensis* Trin. (Poaceae)**

Spanish names:
Indigenous names: Ataa toom, kubat toom, kulab toom[07]
Used by (1*): Huastec[07]
Used for (3#): Digestive (1)[07]; Urological (1)[07]; General and Unspecified (1)[07]
Cognates:
Language contact:

***Indigofera jamaicensis* Spreng. (Fabaceae)**

Spanish names:
Indigenous names: Xoxo-ak'[09]
Used by (1*): Yucatecan Maya[09]
Used for (1#): Digestive (1)[09]
Cognates:
Language contact:

***Indigofera lespedezioides* Kunth (Fabaceae)**

Spanish names:
Indigenous names: Chims¥k[03]
Used by (1*): Zoque[03]
Used for (3#): Digestive (1)[03]; Respiratory (1)[03]; Pregnancy (1)[03]
Cognates:
Language contact:

***Indigofera suffruticosa* Mill. (Fabaceae)**

Spanish names: Añil
Indigenous names: Tsakam yaax, tsakam chichath, , muklaab ts'ohool, manath ts'ohool, muuw[07]; Initiko[08]; Sujuxiw[09]
Used by (6*): Zoque[01]; Huastec[07, 08]; Yucatecan Maya[09]; Western Maya[18]; Zapotec[21]
Used for (18#): Blood (1)[07]; Digestive (4)[07, 08, 09, 18]; Musculoskeletal (1)[08]; Neurological (4)[07, 08, 18, 21]; Respiratory (2)[08, 21]; Skin (2)[08, 21]; Endocrine (1)[08]; Urological (1)[21]; General and Unspecified (2)[01, 21]
Cognates:
Language contact:

***Inga jinicuil* Schltdl. (Fabaceae)**

Spanish names: Cuincuil
Indigenous names: Bujte'[18]
Used by (1*): Western Maya[18]
Used for (2#): Digestive (1)[18]; Endocrine (1)[18]
Cognates:
Language contact:

***Inga micheliana* Harms (Fabaceae)**

Spanish names: Chelel
Indigenous names: Iki ay[01]
Used by (1*): Zoque[01]
Used for (1#): Female genital (1)[01]
Cognates:
Language contact:

[01-28]refer to the study codes in Table 4.1. *Total number of studies citing this taxon #Total number of use-records

***Inga punctata* Willd. (Fabaceae)**

Spanish names: Acotope, Vainilla
Indigenous names: Inki[03]
Used by (1*): Zoque[03]
Used for (3#): Digestive (1)[03]; Skin (1)[03]; Female genital (1)[03]
Cognates:
Language contact:

***Inga* sp. (Fabaceae)**

Spanish names: Carnequil
Indigenous names: Cahijnaquil[21]
Used by (1*): Zapotec[21]
Used for (1#): General and Unspecified (1)[21]
Cognates:
Language contact:

***Inga vera* Willd. (Fabaceae)**

Spanish names: Carniquil
Indigenous names: I'ki[02]; Inki[03]
Used by (2*): Zoque[02, 03]
Used for (3#): Neurological (1)[02]; Skin (1)[03]; Urological (1)[03]
Cognates: Zoq: iki;
Language contact:

***Iostephane heterophylla* (Cav.) Benth. (Asteraceae)**

Spanish names: Hierba del oso
Indigenous names: Ts'ojolil an oso[08]
Used by (1*): Huastec[08]
Used for (3#): Musculoskeletal (1)[08]; Respiratory (1)[08]; General and Unspecified (1)[08]
Cognates:
Language contact:

***Ipomoea alba* L. (Convolvulaceae)**

Spanish names: Guamol blanco/rojo
Indigenous names: Atooy aats[04]; Huchuk'[07]
Used by (3*): Mixe[04]; Huastec[07]; Zapotec[21]
Used for (9#): Digestive (3)[04, 07, 21]; Neurological (1)[07]; Skin (3)[04, 07, 21]; General and Unspecified (2)[04, 07]
Cognates:
Language contact:

***Ipomoea batatas* (L.) Lam. (Convolvulaceae)**

Spanish names: Camote
Indigenous names: Ith[07]; Ith[08]; Is[09]; Is[14]; Acum[18]
Used by (6*): Huastec[07, 08]; Yucatecan Maya[09]; Quichean Maya[14]; Western Maya[18]; Zapotec[21]
Used for (13#): Digestive (3)[08, 14, 18]; Musculoskeletal (3)[08, 18, 21]; Neurological (1)[14]; Skin (2)[07, 09]; Urological (1)[14]; Pregnancy (1)[18]; General and Unspecified (2)[14, 21]
Cognates: Mayan: ith/is; Huas: ith; CoreM: is;
Language contact:

***Ipomoea carnea* Jacq. (Convolvulaceae)**

Spanish names:
Indigenous names: Nunak mooya[03]
Used by (2*): Zoque[03]; Huastec[07]
Used for (3#): Skin (1)[03]; General and Unspecified (2)[03, 07]
Cognates:
Language contact:

[01-28]refer to the study codes in Table 4.1. *Total number of studies citing this taxon #Total number of use-records

**_Ipomoea dumosa_ (Benth.) L.O. Williams (Convolvulaceae)**

Spanish names: Manto
Indigenous names: Si'yu[06]; Thuuyu'[07]
Used by (2*): Totonac[06]; Huastec[07]
Used for (4#): Eye (1)[07]; Neurological (1)[07]; Pregnancy (2)[06, 07]
Cognates:
Language contact: Tot <> Hua

**_Ipomoea heterodoxa_ Standl. & Steyerm. (Convolvulaceae)**

Spanish names:
Indigenous names: Chiwohk'ax, Cancer xiw[09]
Used by (1*): Yucatecan Maya[09]
Used for (2#): Skin (1)[09]; General and Unspecified (1)[09]
Cognates:
Language contact:

**_Ipomoea indica_ (Burm.) Merr. (Convolvulaceae)**

Spanish names: Guamol
Indigenous names: Nak'atang poj[02]; Sayuk Q'ehen[14]
Used by (2*): Zoque[02]; Quichean Maya[14]
Used for (2#): Skin (1)[02]; Pregnancy (1)[14]
Cognates:
Language contact:

**_Ipomoea intrapilosa_ Rose (Convolvulaceae)**

Spanish names: Pájaro bobo
Indigenous names: Yàg-bnù, blàg-bnù, yàg-blàg-bnù[23]
Used by (1*): Zapotec[23]
Used for (2#): Neurological (1)[23]; General and Unspecified (1)[23]
Cognates:
Language contact:

**_Ipomoea pauciflora_ M. Martens & Galeotti (Convolvulaceae)**

Spanish names: Pajaro bobo
Indigenous names: Ya banu[22]
Used by (1*): Zapotec[22]
Used for (4#): Neurological (1)[22]; Respiratory (1)[22]; Skin (1)[22]; General and Unspecified (1)[22]
Cognates:
Language contact:

**_Ipomoea purga_ (Wender.) Hayne (Convolvulaceae)**

Spanish names: Jalapa
Indigenous names:
Used by (2*): Quichean Maya[12, 14]
Used for (2#): Psychological (1)[12]; General and Unspecified (1)[14]
Cognates:
Language contact:

**_Ipomoea purpurea_ (L.) Roth (Convolvulaceae)**

Spanish names: Guaco, Ipomea
Indigenous names: Paxin läq[12]
Used by (1*): Quichean Maya[12]
Used for (4#): Blood (1)[12]; Digestive (1)[12]; Musculoskeletal (1)[12]; Skin (1)[12]
Cognates:
Language contact:

[01-28]refer to the study codes in Table 4.1. *Total number of studies citing this taxon #Total number of use-records

### *Ipomoea silvicola* House (Convolvulaceae)

Spanish names:
Indigenous names: Nahuk Q'en[14]
Used by (1*): Quichean Maya[14]
Used for (1#): Ear (1)[14]
Cognates:
Language contact:

### *Ipomoea* sp. (Convolvulaceae)

Spanish names: Guaco, Ipomea
Indigenous names: Paxin läq[12]; Xoconob[14]
Used by (2*): Quichean Maya[12, 14]
Used for (6#): Blood (1)[12]; Digestive (2)[12, 14]; Musculoskeletal (1)[12]; Skin (1)[12]; General and Unspecified (1)[14]
Cognates:
Language contact:

### *Ipomoea tiliacea* (Willd.) Choisy (Convolvulaceae)

Spanish names:
Indigenous names: Guamol[21]
Used by (1*): Zapotec[21]
Used for (2#): Digestive (1)[21]; Skin (1)[21]
Cognates:
Language contact:

### *Ipomoea variabilis* (Schltdl. et Cham.) Choisy (Convolvulaceae)

Spanish names: Chonege
Indigenous names: Xonege[03]
Used by (1*): Zoque[03]
Used for (1#): Digestive (1)[03]
Cognates:
Language contact:

### *Iresine diffusa* Humb. & Bonpl. ex Willd. (Amaranthaceae)

Spanish names: Tlan cuaya
Indigenous names: Tsus tunuk koso[03]; Paloma jomol[20]; Tlancuaya[26]
Used by (3*): Zoque[03]; Western Maya[20]; Nahua[26]
Used for (8#): Blood (1)[03]; Digestive (2)[20, 26]; Musculoskeletal (1)[03]; Skin (2)[03, 26]; General and Unspecified (2)[03, 26]
Cognates:
Language contact:

### *Iresine nigra* Uline & W. L. Bray (Amaranthaceae)

Spanish names:
Indigenous names:
Used by (1*): Zoque[03]
Used for (1#): Skin (1)[03]
Cognates:
Language contact:

### *Iresine* sp. (Amaranthaceae)

Spanish names:
Indigenous names: Zhwǐs, guìzh guièe nquǐts, guièe-mîdz[23]
Used by (1*): Zapotec[23]
Used for (3#): Digestive (1)[23]; Psychological (1)[23]; General and Unspecified (1)[23]
Cognates:
Language contact:

[01-28]refer to the study codes in Table 4.1. *Total number of studies citing this taxon #Total number of use-records

***Isocarpha oppositifolia*** **(L.) Cass. (Asteraceae)**

Spanish names: Oreja de conejo, hierba de conejo
Indigenous names: U chikin t'ur[19]
Used by (1*): Western Maya[19]
Used for (1#): Digestive (1)[19]
Cognates:
Language contact:

***Isochilus linearis*** **(Jacq.) R.Br. (Orchidaceae)**

Spanish names:
Indigenous names: Toom[07]
Used by (1*): Huastec[07]
Used for (1#): General and Unspecified (1)[07]
Cognates:
Language contact:

***Jacaranda mimosifolia*** **D.Don (Bignoniaceae)**

Spanish names: Jacaranda
Indigenous names: Q'ojom che'[13]; Yàg-jàcàrân[23]
Used by (3*): Quichean Maya[12, 13]; Zapotec[23]
Used for (5#): Digestive (3)[12, 13, 23]; Psychological (1)[12]; General and Unspecified (1)[12]
Cognates:
Language contact:

***Jasminum sambac*** **(L.) Aiton (Oleaceae)**

Spanish names: Jazmín
Indigenous names:
Used by (1*): Zoque[02]
Used for (1#): Respiratory (1)[02]
Cognates:
Language contact:

***Jasminum*** **sp. (Oleaceae)**

Spanish names:
Indigenous names: Hasmiin[07]
Used by (1*): Huastec[07]
Used for (2#): Respiratory (1)[07]; General and Unspecified (1)[07]
Cognates:
Language contact:

***Jatropha curcas*** **L. (Euphorbiaceae)**

Spanish names: Piñon
Indigenous names: Ekis[02]; Cuyukum[03]; Kutsøkee[04]; Chuta[05]; Čuta[05]; Chut'a[06]; Thakpeen te', piiloch[07]; Siklite'[09]; Sakirte'[19]
Used by (13*): Zoque[01, 02, 03]; Mixe[04]; Totonac[05, 06]; Huastec[07]; Yucatecan Maya[09]; Quichean Maya[12, 13]; Western Maya[19]; Zapotec[21]; Nahua[25]
Used for (26#): Blood (1)[07]; Digestive (5)[01, 02, 05, 06, 07]; Musculoskeletal (1)[13]; Neurological (1)[19]; Respiratory (1)[05]; Skin (12)[02, 03, 04, 05, 05, 06, 07, 09, 12, 19, 21, 25]; Female genital (1)[03]; General and Unspecified (4)[02, 12, 19, 21]
Cognates: MZ: eki/uku/øke; Toto: chuta; CoreM: sVkite;
Language contact: MZ <> Tot; MZ > CoreM

[01-28]refer to the study codes in Table 4.1. *Total number of studies citing this taxon #Total number of use-records

***Jatropha dioica*** **Sessé (Euphorbiaceae)**

Spanish names: Suzí
Indigenous names:
Used by (1*): Zapotec[22]
Used for (4#): Digestive (1)[22]; Skin (1)[22]; Pregnancy (1)[22]; General and Unspecified (1)[22]
Cognates:
Language contact:

***Jatropha gaumeri*** **Greenm. (Euphorbiaceae)**

Spanish names: Pinon
Indigenous names: Pomolche'[09]
Used by (2*): Yucatecan Maya[09, 11]
Used for (3#): Musculoskeletal (1)[11]; Skin (2)[09, 11]
Cognates:
Language contact:

***Jatropha glauca*** **Vahl (Euphorbiaceae)**

Spanish names:
Indigenous names: Cruz ojo xiw[09]
Used by (1*): Yucatecan Maya[09]
Used for (1#): Digestive (1)[09]
Cognates:
Language contact:

***Jatropha gossypiifolia*** **L. (Euphorbiaceae)**

Spanish names: Mala mujer/toloache
Indigenous names: Tzis kä wang [02]
Used by (2*): Zoque[02, 03]
Used for (3#): Digestive (2)[02, 03]; Neurological (1)[02]
Cognates:
Language contact:

***Jatropha neopauciflora*** **Pax (Euphorbiaceae)**

Spanish names: Sangre de drago
Indigenous names:
Used by (1*): Nahua[27]
Used for (2#): Neurological (1)[27]; Skin (1)[27]
Cognates:
Language contact:

***Jatropha peltata*** **Sessé (Euphorbiaceae)**

Spanish names:
Indigenous names: Yàg-pcuà[23]
Used by (1*): Zapotec[23]
Used for (1#): Skin (1)[23]
Cognates:
Language contact:

***Jatropha*** **sp. (Euphorbiaceae)**

Spanish names: Ortiga
Indigenous names: Làal[10]
Used by (1*): Yucatecan Maya[10]
Used for (3#): Ear (1)[10]; Musculoskeletal (1)[10]; Neurological (1)[10]
Cognates:
Language contact:

***Juglans olanchana* Standl. & L.O.Williams (Juglandaceae)**

Spanish names: Nogal
Indigenous names:
Used by (1*): Quichean Maya[12]
Used for (6#): Blood (1)[12]; Digestive (1)[12]; Musculoskeletal (1)[12]; Respiratory (1)[12]; Endocrine (1)[12]; General and Unspecified (1)[12]
Cognates:
Language contact:

***Juglans regia* L. (Juglandaceae)**

Spanish names: Nogal
Indigenous names: Mak xu 'xut qui 'hui[06]
Used by (2*): Totonac[06]; Nahua[26]
Used for (4#): Digestive (1)[06]; Psychological (1)[26]; Skin (1)[06]; Urological (1)[26]
Cognates:
Language contact:

***Juglans* sp. (Juglandaceae)**

Spanish names: Nogal
Indigenous names:
Used by (1*): Zoque[03]
Used for (1#): Pregnancy (1)[03]
Cognates:
Language contact:

***Juniperus flaccida* Schltdl. (Cupressaceae)**

Spanish names: Cedro; Sabino, ahuehuete
Indigenous names: Yàg-guìzdòo, yàg-guìstòo[23]
Used by (2*): Zapotec[23]; Nahua[25]
Used for (3#): Neurological (1)[25]; Skin (1)[23]; General and Unspecified (1)[25]
Cognates:
Language contact:

***Justicia albobracteata* Leonard (Acanthaceae)**

Spanish names:
Indigenous names: Xna kejen[16]
Used by (1*): Quichean Maya[16]
Used for (1#): Neurological (1)[16]
Cognates:
Language contact:

***Justicia aurea* Schltdl. (Acanthaceae)**

Spanish names:
Indigenous names: Saxjolom chacmut[16]
Used by (1*): Quichean Maya[16]
Used for (2#): Neurological (1)[16]; Psychological (1)[16]
Cognates:
Language contact:

**_Justicia brandegeeana_ Wassh. & L.B.Sm. (Acanthaceae)**

Spanish names:
Indigenous names: Tsaakuy elul, xonol palats, nonool t'ot, witssil a eheenchix, ok t'ot, tsakam payab wits, uxkwe' ts'ohool, eheenchix wits, wots paya', bohol ch'ohool[07]
Used by (1*): Huastec[07]
Used for (7#): Digestive (1)[07]; Neurological (1)[07]; Psychological (1)[07]; Skin (1)[07]; Pregnancy (1)[07]; Female genital (1)[07]; General and Unspecified (1)[07]
Cognates:
Language contact:

**_Justicia breviflora_ (Nees) Rusby (Acanthaceae)**

Spanish names: Pote verde
Indigenous names: Majei chich[03]; Lumbresil pim[14]; Rax Pim[15]
Used by (3*): Zoque[03]; Quichean Maya[14, 15]
Used for (5#): Digestive (1)[14]; Skin (1)[03]; Pregnancy (1)[15]; Female genital (1)[15]; General and Unspecified (1)[03]
Cognates:
Language contact:

**_Justicia campechiana_ Standl. ex Lundell (Acanthaceae)**

Spanish names:
Indigenous names: Ixwaq[14]
Used by (1*): Quichean Maya[14]
Used for (2#): Digestive (1)[14]; General and Unspecified (1)[14]
Cognates:
Language contact:

**_Justicia candelariae_ (Oerst.) Leonard (Acanthaceae)**

Spanish names:
Indigenous names: Tutxti ay[03]
Used by (1*): Zoque[03]
Used for (3#): Digestive (1)[03]; Psychological (1)[03]; Female genital (1)[03]
Cognates:
Language contact:

**_Justicia comata_ (L.) Lam. (Acanthaceae)**

Spanish names: Trébol; Yolixpa
Indigenous names:
Used by (2*): Totonac[06]; Western Maya[18]
Used for (3#): Digestive (1)[06]; General and Unspecified (2)[06, 18]
Cognates:
Language contact:

**_Justicia fimbriata_ (Nees) V.A.W. Graham (Acanthaceae)**

Spanish names:
Indigenous names: Numay Pim[15]
Used by (1*): Quichean Maya[15]
Used for (2#): Psychological (1)[15]; General and Unspecified (1)[15]
Cognates:
Language contact:

[01-28]refer to the study codes in Table 4.1. *Total number of studies citing this taxon #Total number of use-records

**_Justicia fulvicoma_ Schltdl. & Cham. (Acanthaceae)**

Spanish names:
Indigenous names: Tsaakuy elul, xonol palats[07]
Used by (1*): Huastec[07]
Used for (1#): Pregnancy (1)[07]
Cognates:
Language contact:

**_Justicia macrantha_ Benth. (Acanthaceae)**

Spanish names: Camarón
Indigenous names:
Used by (1*): Quichean Maya[12]
Used for (1#): Skin (1)[12]
Cognates:
Language contact:

**_Justicia pectoralis_ Jacq. (Acanthaceae)**

Spanish names: Flor de tila, tilo
Indigenous names: Pithomlaab ts'ohool[07]; K'xuy i kok, Santa Maria kejen[16]; Santa Maria k'ejen[17]; U nich tila[18]
Used by (4*): Huastec[07]; Quichean Maya[16, 17]; Western Maya[18]
Used for (9#): Digestive (1)[07]; Ear (1)[18]; Neurological (2)[07, 16]; Psychological (1)[18]; Respiratory (1)[18]; Endocrine (1)[18]; General and Unspecified (1)[07]; nd[17]
Cognates:
Language contact:

**_Justicia_ sp. (Acanthaceae)**

Spanish names: Camarón de Montaña; Hierba del santuario
Indigenous names: Sa'x jolom chacmut[17]
Used by (3*): Quichean Maya[12, 17]; Nahua[26]
Used for (4#): Respiratory (1)[26]; Skin (1)[26]; Endocrine (1)[12]; nd[17]
Cognates:
Language contact:

**_Justicia spicigera_ Schltdl. (Acanthaceae)**

Spanish names: Añil, hoja de tinta, muicle
Indigenous names: Tzämi tane/tzitz[01]; Chich[03]; Limanin[05]; Muu, muuw[07]; Muu[08]; T'oxe ji'tn[18]; Mohuitl[25]; Muictli[28]
Used by (12*): Zoque[01, 03]; Totonac[05, 06]; Huastec[07, 08]; Western Maya[18, 19]; Zapotec[21]; Nahua[25, 26, 28]
Used for (42#): Blood (2)[06, 08]; Digestive (4)[01, 06, 25, 26]; Cardiovascular (3)[03, 08, 25]; Musculoskeletal (1)[25]; Neurological (5)[03, 07, 19, 21, 25]; Psychological (2)[08, 25]; Respiratory (3)[01, 07, 25]; Skin (3)[08, 25, 28]; Endocrine (1)[08]; Urological (2)[01, 18]; Pregnancy (2)[01, 07]; Female genital (2)[05, 07]; General and Unspecified (12)[01, 03, 03, 05, 06, 07, 08, 19, 21, 25, 26, 28]
Cognates: Zoq: tzitz/chich; Huas: muu; Nahua: muictli;
Language contact: Hua <> Nah > Spanish

**_Kalanchoe blossfeldiana_ Poelln. (Crassulaceae)**

Spanish names: Beladonna; Hüipil de monte
Indigenous names: Pooti'l qehen[14]
Used by (3*): Zoque[03]; Yucatecan Maya[09]; Quichean Maya[14]
Used for (5#): Digestive (1)[03]; Eye (1)[14]; Musculoskeletal (1)[14]; Skin (2)[03, 09]
Cognates:
Language contact:

[01-28]refer to the study codes in Table 4.1. *Total number of studies citing this taxon #Total number of use-records

***Kalanchoe calycinum*** **Salisb. (Crassulaceae)**

Spanish names: Maravilla real
Indigenous names: Majei maravilla[03]
Used by (1*): Zoque[03]
Used for (4#): Eye (1)[03]; Ear (1)[03]; Skin (1)[03]; General and Unspecified (1)[03]
Cognates:
Language contact:

***Kalanchoe daigremontiana*** **Raym.-Hamet & H. Perrier (Crassulaceae)**

Spanish names: Beladona (macho); sanalotodo/curalotodo/lengua de vecino
Indigenous names:
Used by (2*): Zoque[01, 02]
Used for (5#): Musculoskeletal (1)[02]; Neurological (1)[02]; Skin (1)[01]; General and Unspecified (2)[01, 02]
Cognates:
Language contact:

***Kalanchoe flammea*** **Stapf (Crassulaceae)**

Spanish names: Belladona
Indigenous names:
Used by (1*): Western Maya[18]
Used for (4#): Digestive (1)[18]; Musculoskeletal (1)[18]; Respiratory (1)[18]; Skin (1)[18]
Cognates:
Language contact:

***Kalanchoe integra*** **(Medik.) Kuntze (Crassulaceae)**

Spanish names: Beladonna
Indigenous names:
Used by (1*): Yucatecan Maya[09]
Used for (2#): Musculoskeletal (1)[09]; Skin (1)[09]
Cognates:
Language contact:

***Kalanchoe mortagei*** **Raym.-Hamet & H. Perrier (Crassulaceae)**

Spanish names: Beladonna gigante, mala madre
Indigenous names:
Used by (1*): Zoque[01]
Used for (4#): Digestive (1)[01]; Skin (1)[01]; Urological (1)[01]; Female genital (1)[01]
Cognates:
Language contact:

***Kallstroemia maxima*** **(L.) Hook. & Arn. (Zygophyllaceae)**

Spanish names: Campanilla/hoja de azar
Indigenous names: Tza'a tzoy[01]
Used by (1*): Zoque[01]
Used for (1#): General and Unspecified (1)[01]
Cognates:
Language contact:

***Karwinskia calderonii*** **Standl. (Rhamnaceae)**

Spanish names: Guiiguiste
Indigenous names: Ixim te'[19]
Used by (1*): Western Maya[19]
Used for (1#): Skin (1)[19]
Cognates:
Language contact:

***Kearnemalvastrum subtriflorum* (Lag.) D.M.Bates (Malvaceae)**

Spanish names: Malva real, Malvavisca
Indigenous names: Malma, Malva Aq'om[12]
Used by (1*): Quichean Maya[12]
Used for (5#): Blood (1)[12]; Cardiovascular (1)[12]; Psychological (1)[12]; Skin (1)[12]; General and Unspecified (1)[12]
Cognates:
Language contact:

***Kionophyton seminuda* (Schltr.) Garay (Orchidaceae)**

Spanish names:
Indigenous names: Kw'itool ts'ohool, k'ok'om it'ath ts'ohool[07]
Used by (1*): Huastec[07]
Used for (1#): Pregnancy (1)[07]
Cognates:
Language contact:

***Koanophyllon albicaule* (Sch.Bip. ex Klatt) R.M.King & H.Rob. (Asteraceae)**

Spanish names:
Indigenous names: Z¥¥y ay[03]
Used by (1*): Zoque[03]
Used for (2#): Skin (1)[03]; General and Unspecified (1)[03]
Cognates:
Language contact:

***Koanophyllon albicaulis* (Sch.Bip. ex Klatt) R.M.King & H.Rob. (Asteraceae)**

Spanish names: Hoja vishe
Indigenous names: Øgøin aay[04]; Tok'te', yaxal[07]
Used by (3*): Zoque[02]; Mixe[04]; Huastec[07]
Used for (8#): Digestive (1)[07]; Musculoskeletal (1)[07]; Neurological (1)[07]; Psychological (1)[02]; Respiratory (1)[07]; General and Unspecified (3)[02, 04, 07]
Cognates:
Language contact:

***Kohleria spicata* (Kunth) Oerst. (Gesneriaceae)**

Spanish names: Planta capulina
Indigenous names:
Used by (1*): Zoque[03]
Used for (1#): Skin (1)[03]
Cognates:
Language contact:

***Kosteletzkya tubiflora* (Moc. & Sessé ex DC.) O.J.Blanch. & McVaugh (Malvaceae)**

Spanish names:
Indigenous names: Bisil, Xcampana ka'ax[09]
Used by (1*): Yucatecan Maya[09]
Used for (1#): Digestive (1)[09]
Cognates:
Language contact:

***Krameria pauciflora* DC. (Krameriaceae)**

Spanish names: Romerito, hoja de disenteria
Indigenous names: Luu guiatzimbeer[21]
Used by (1*): Zapotec[21]
Used for (3#): Digestive (1)[21]; Pregnancy (1)[21]; Female genital (1)[21]
Cognates:
Language contact:

***Krugiodendron ferreum* (Vahl) Urb. (Rhamnaceae)**

Spanish names:
Indigenous names: Chintok'[09]
Used by (1*): Yucatecan Maya[09]
Used for (2#): Neurological (1)[09]; Urological (1)[09]
Cognates:
Language contact:

***Kyllinga brevifolia* Rottb. (Cyperaceae)**

Spanish names:
Indigenous names: Xmach tz'i' Q'ehen[14]
Used by (1*): Quichean Maya[14]
Used for (1#): Endocrine (1)[14]
Cognates:
Language contact:

***Lactuca sativa* L. (Asteraceae)**

Spanish names: Lechuga
Indigenous names:
Used by (3*): Zoque[02]; Totonac[06]; Quichean Maya[13]
Used for (4#): Psychological (1)[06]; Endocrine (1)[02]; Pregnancy (1)[13]; Female genital (1)[13]
Cognates:
Language contact:

***Laetia thamnia* L. (Salicaceae)**

Spanish names: Corrimiento
Indigenous names:
Used by (1*): Yucatecan Maya[11]
Used for (1#): General and Unspecified (1)[11]
Cognates:
Language contact:

***Lagascea* sp. (Asteraceae)**

Spanish names:
Indigenous names: Papan te'[20]
Used by (1*): Western Maya[20]
Used for (1#): Digestive (1)[20]
Cognates:
Language contact:

***Lagenaria siceraria* (Molina) Standl. (Cucurbitaceae)**

Spanish names: Chical pestle, pumpo, lipo
Indigenous names: Xomom, kweentu'[07]; Lek[09]; Lobej' quiasj'ga, beejru'u[21]
Used by (3*): Huastec[07]; Yucatecan Maya[09]; Zapotec[21]
Used for (6#): Digestive (1)[21]; Musculoskeletal (1)[09]; Respiratory (2)[07, 21]; Urological (1)[07]; General and Unspecified (1)[21]
Cognates:
Language contact: Yuc <> Zap

***Lagenaria* sp. (Cucurbitaceae)**

Spanish names: Tecomate
Indigenous names: Pok[03]
Used by (1*): Zoque[03]
Used for (2#): Digestive (1)[03]; Psychological (1)[03]
Cognates:
Language contact:

[01-28]refer to the study codes in Table 4.1. *Total number of studies citing this taxon #Total number of use-records

**_Lamourouxia_ sp. (Orobanchaceae)**

Spanish names:
Indigenous names: Guièe-dzǐng, guìzh-dzǐng[23]
Used by (1*): Zapotec[23]
Used for (2#): Skin (1)[23]; General and Unspecified (1)[23]
Cognates:
Language contact:

**_Lantana achyranthifolia_ Desf. (Verbenaceae)**

Spanish names: Riñonina
Indigenous names: Pajk jäyä[01]; Thak patelx, patel mantelx, kanil bakan, thak otoomal[07]
Used by (2*): Zoque[01]; Huastec[07]
Used for (4#): Digestive (1)[07]; Endocrine (1)[07]; Urological (1)[01]; General and Unspecified (1)[07]
Cognates:
Language contact: Chiapas Zoq <> Hua

**_Lantana camara_ L. (Verbenaceae)**

Spanish names: Cinco negritos, conchita, orozus
Indigenous names: Pajk jäyä[01]; Kan'muk[03]; Tøøts kumot[04]; Škaštajat štuki[05]; X'laca stap'u squii'ti[06]; Tsak patelax, witsiim i thayemlaab, tsakam maap, tsakam otoomal, wal thancha', pahatix wits[07]; Roq' Chiwan[12]; Saq'i Tulux[14]; Ch'ilvet, ch'ili wet, ch'ilch'il wajch[20]; Guxa'a riene'e[21]
Used by (14*): Zoque[01, 02, 03]; Mixe[04]; Totonac[05, 06]; Huastec[07]; Yucatecan Maya[09]; Quichean Maya[12, 14]; Western Maya[19, 20]; Zapotec[21]; Nahua[26]
Used for (46#): Digestive (10)[01, 02, 03, 06, 07, 09, 12, 14, 20, 21]; Cardiovascular (1)[12]; Musculoskeletal (2)[07, 12]; Neurological (6)[03, 04, 06, 07, 12, 14]; Psychological (1)[03]; Respiratory (7)[01, 03, 05, 06, 14, 20, 26]; Skin (3)[03, 07, 21]; Urological (4)[01, 07, 12, 20]; Pregnancy (4)[03, 07, 19, 20]; Female genital (2)[12, 19]; General and Unspecified (6)[02, 06, 07, 12, 20, 26]
Cognates: Mayan: sak tVlVx; CoreM: chi wa;
Language contact: Tot <> Hua

**_Lantana horrida_ Kunth (Verbenaceae)**

Spanish names: Cinco negritos, salvia silvestre
Indigenous names: Pak jäyä[01]; Ruwi amaj, corcoch, cor choch, utucán cumatz[13]; Ch'ilvet, ch'ili wet, ch'ilch'il wajch[20]
Used by (6*): Zoque[01]; Quichean Maya[12, 13, 14]; Western Maya[20]; Nahua[26]
Used for (24#): Digestive (3)[12, 13, 20]; Cardiovascular (1)[13]; Musculoskeletal (2)[12, 13]; Neurological (2)[13, 14]; Respiratory (3)[13, 20, 26]; Skin (2)[12, 13]; Urological (3)[01, 12, 20]; Pregnancy (1)[01]; Female genital (3)[01, 12, 13]; Male genital (1)[12]; General and Unspecified (3)[12, 13, 20]
Cognates:
Language contact:

**_Lantana involucrata_ L. (Verbenaceae)**

Spanish names:
Indigenous names: Baron[07]; Xkot' Kaway[14]
Used by (2*): Huastec[07]; Quichean Maya[14]
Used for (3#): Digestive (2)[07, 14]; General and Unspecified (1)[14]
Cognates:
Language contact:

**_Lantana_ sp. (Verbenaceae)**

Spanish names: Sapotilla
Indigenous names: Žob leh[22]
Used by (1*): Zapotec[22]
Used for (1#): Digestive (1)[22]
Cognates:
Language contact:

[01-28]refer to the study codes in Table 4.1. *Total number of studies citing this taxon #Total number of use-records

***Lantana trifolia* L. (Verbenaceae)**

Spanish names:
Indigenous names: Tøøts kumot[04]; Tulux Q'ehen[14]; Tulush pim[17]
Used by (4*): Zoque[03]; Mixe[04]; Quichean Maya[14, 17]
Used for (4#): Neurological (1)[04]; Respiratory (1)[03]; Endocrine (1)[14]; nd[17]
Cognates: Quich: tulux;
Language contact:

***Laportea aestuans* (L.) Chew (Urticaceae)**

Spanish names: Ortiga
Indigenous names: Làal[10]
Used by (1*): Yucatecan Maya[10]
Used for (3#): Ear (1)[10]; Musculoskeletal (1)[10]; Neurological (1)[10]
Cognates:
Language contact:

***Larrea tridentata* (Sessé & Moc. ex DC.) Coville (Zygophyllaceae)**

Spanish names: Gobernadora
Indigenous names:
Used by (1*): Zapotec[21]
Used for (1#): Digestive (1)[21]
Cognates:
Language contact:

***Lasiacis procerrima* (Hack.) Hitchc. ex Chase (Poaceae)**

Spanish names: Carisso; Carizo
Indigenous names: Am ay[02]; K¥kujuki ay[03]
Used by (2*): Zoque[02, 03]
Used for (2#): Skin (1)[03]; Urological (1)[02]
Cognates:
Language contact:

***Lasiacis ruscifolia* (Kunth) Hitchc. ex Chase (Poaceae)**

Spanish names: Carisso
Indigenous names: K¥kujuki ay[03]; Tseey kw'a', thimallon pakaab[07]; Siit[09]
Used by (3*): Zoque[03]; Huastec[07]; Yucatecan Maya[09]
Used for (6#): Digestive (1)[07]; Musculoskeletal (1)[07]; Skin (2)[03, 09]; Urological (1)[07]; General and Unspecified (1)[07]
Cognates:
Language contact:

***Lasiacis* sp. (Poaceae)**

Spanish names:
Indigenous names: Guìzh-gòob-guì,  guìzh-gòob[23]
Used by (1*): Zapotec[23]
Used for (1#): General and Unspecified (1)[23]
Cognates:
Language contact:

***Lasianthaea* sp. (Asteraceae)**

Spanish names:
Indigenous names: Q'aham tzaj[14]
Used by (1*): Quichean Maya[14]
Used for (1#): General and Unspecified (1)[14]
Cognates:
Language contact:

[01-28]refer to the study codes in Table 4.1. *Total number of studies citing this taxon #Total number of use-records

***Laurus nobilis* L. (Lauraceae)**

Spanish names: Laurel
Indigenous names:
Used by (1*): Zoque[03]
Used for (1#): General and Unspecified (1)[03]
Cognates:
Language contact:

***Lavandula* sp. (Lamiaceae)**

Spanish names: Alucema
Indigenous names:
Used by (3*): Zoque[01, 02]; Western Maya[19]
Used for (12#): Blood (1)[19]; Digestive (2)[01, 02]; Musculoskeletal (1)[02]; Respiratory (2)[01, 02]; Pregnancy (3)[01, 02, 19]; Female genital (2)[01, 02]; General and Unspecified (1)[01]
Cognates:
Language contact:

***Lawsonia inermis* L. (Lythraceae)**

Spanish names: Rosedad
Indigenous names: Rosedad[04]
Used by (2*): Mixe[04]; Zapotec[21]
Used for (2#): Skin (1)[04]; General and Unspecified (1)[21]
Cognates:
Language contact:

***Leonotis nepetifolia* (L.) R.Br. (Lamiaceae)**

Spanish names: Hierba del burro
Indigenous names:
Used by (2*): Yucatecan Maya[09]; Nahua[26]
Used for (2#): Digestive (1)[26]; Neurological (1)[09]
Cognates:
Language contact:

***Leonurus japonicus* Houtt. (Lamiaceae)**

Spanish names: Mariguana simarron/ Chiquisa; Marihuanilla
Indigenous names:
Used by (2*): Zoque[01, 03]
Used for (5#): Digestive (1)[03]; Musculoskeletal (2)[01, 03]; Skin (2)[01, 03]
Cognates:
Language contact:

***Leonurus sibiricus* L. (Lamiaceae)**

Spanish names:
Indigenous names:
Used by (1*): Mixe[04]
Used for (1#): Female genital (1)[04]
Cognates:
Language contact:

[01-28]refer to the study codes in Table 4.1. *Total number of studies citing this taxon #Total number of use-records

**_Lepechinia caulescens_ (Ortega) Epling (Lamiaceae)**

Spanish names: Bretónica; Bretonica, hierba tónica, arnica
Indigenous names: Qaj tajik q'ox[12]; Upek'tzi[13]
Used by (3*): Quichean Maya[12, 13]; Nahua[25]
Used for (10#): Digestive (1)[13]; Cardiovascular (1)[25]; Musculoskeletal (2)[12, 13]; Psychological (1)[13]; Respiratory (1)[25]; Skin (1)[13]; Endocrine (1)[25]; General and Unspecified (2)[12, 13]
Cognates:
Language contact:

**_Lepechinia schiedeana_ (Schltdl.) Vatke (Lamiaceae)**

Spanish names: Brétonica, hierba del sapo, chucharilla
Indigenous names: Cha vitz[13]; Tzotzil vomol, yaxal vomol, na p'ilix, chilchil tz'i'lel, poxil a'ch'ut[20]
Used by (2*): Quichean Maya[13]; Western Maya[20]
Used for (6#): Digestive (1)[20]; Neurological (1)[20]; Respiratory (1)[20]; Skin (1)[13]; General and Unspecified (2)[13, 20]
Cognates:
Language contact: K'iche' <> Tzeltalan

**_Lepidaploa canescens_ (Kunth) Cass. (Asteraceae)**

Spanish names:
Indigenous names: Semem Q'ehen[14]
Used by (1*): Quichean Maya[14]
Used for (1#): General and Unspecified (1)[14]
Cognates:
Language contact:

**_Lepidaploa tortuosa_ (L.) H.Rob. (Asteraceae)**

Spanish names:
Indigenous names: Sotopok tsay[03]
Used by (1*): Zoque[03]
Used for (4#): Digestive (1)[03]; Respiratory (1)[03]; Skin (1)[03]; General and Unspecified (1)[03]
Cognates:
Language contact:

**_Lepidium virginicum_ L. (Brassicaceae)**

Spanish names: Lentejilla
Indigenous names: Ma'tza yomo [01]; Tsakam utsun, utsun ts'ohool[07]; Rakän aqwäl, Saqil Kayis, Mesebäl Q'os Paxk'u'y[12]; Siquil be', skil q'ayes, ik q'ayes, masb'al uxekaj[13]; Pich t'uluk, pich' tz'i lel, sakil jomol, sak nich wamal, kajk'an wamal, anix te' wamal[20]; Pich t'uluk. Sakil jomol, sak nich wamal, kajk'an wamal, anix te' wamal[20]; Guish inguiedi[21]; Siak[22]
Used by (10*): Zoque[01]; Huastec[07]; Yucatecan Maya[11]; Quichean Maya[12, 13]; Western Maya[19, 20]; Zapotec[21, 22]; Nahua[26]
Used for (28#): Digestive (6)[01, 07, 12, 13, 19, 20]; Ear (1)[13]; Musculoskeletal (1)[12]; Neurological (2)[12, 13]; Psychological (2)[12, 13]; Respiratory (3)[12, 13, 26]; Skin (5)[11, 12, 20, 21, 22]; Endocrine (1)[12]; Urological (1)[13]; Pregnancy (1)[20]; Female genital (3)[11, 12, 21]; General and Unspecified (2)[12, 26]
Cognates: Mayan: sak; Quich: mesebäl/masb'al;
Language contact: Maya > Mitla Zap and Chiapas Zoq

**_Lessingianthus mollissimus_ (D.Don ex Hook. & Arn.) H.Rob. (Asteraceae)**

Spanish names:
Indigenous names: Semem[14]
Used by (1*): Quichean Maya[14]
Used for (1#): Digestive (1)[14]
Cognates:
Language contact:

[01-28]refer to the study codes in Table 4.1. *Total number of studies citing this taxon #Total number of use-records

**_Leucaena diversifolia_ (Schltdl.) Benth. (Fabaceae)**

Spanish names: Timbre
Indigenous names:
Used by (1*): Nahua[26]
Used for (1#): Digestive (1)[26]
Cognates:
Language contact:

**_Leucaena esculenta_ (DC.) Benth. (Fabaceae)**

Spanish names: Guaje
Indigenous names: Lya[22]
Used by (1*): Zapotec[22]
Used for (1#): Digestive (1)[22]
Cognates:
Language contact:

**_Leucaena lanceolata_ S.Watson (Fabaceae)**

Spanish names: Huaje de la peña/ de agua
Indigenous names: Laj'[21]
Used by (1*): Zapotec[21]
Used for (2#): Digestive (1)[21]; General and Unspecified (1)[21]
Cognates:
Language contact:

**_Leucaena leucocephala_ (Lam.) de Wit (Fabaceae)**

Spanish names: Guaxi; Huaje blanco; Huaje/Huash
Indigenous names: Pakapaka[01]; Li'li'ka, guamuxi[06]; Waxim[09]; Laj' tza gutzii[21]
Used by (4*): Zoque[01]; Totonac[06]; Yucatecan Maya[09]; Zapotec[21]
Used for (6#): Digestive (3)[01, 06, 21]; Skin (1)[09]; General and Unspecified (2)[01, 21]
Cognates:
Language contact: Tot <> Yuc

**_Leucaena pulverulenta_ (Schltdl.) Benth. (Fabaceae)**

Spanish names: Guaxi
Indigenous names: Li'li'ka, guamuxi[06]; Thuk'[07]
Used by (2*): Totonac[06]; Huastec[07]
Used for (2#): Digestive (1)[06]; General and Unspecified (1)[07]
Cognates:
Language contact:

**_Leucaena_ sp. (Fabaceae)**

Spanish names: Guaje
Indigenous names: Yàg-nlàbâd, yàg-nlìbâd, yàg-nlázhò, yàg-nlízhò[23]
Used by (1*): Zapotec[23]
Used for (2#): Digestive (1)[23]; Skin (1)[23]
Cognates:
Language contact:

**_Leucanthemum vulgare_ (Vaill.) Lam. (Asteraceae)**

Spanish names: Margarita comun
Indigenous names:
Used by (1*): Quichean Maya[12]
Used for (3#): Respiratory (1)[12]; Skin (1)[12]; General and Unspecified (1)[12]
Cognates:
Language contact:

[01-28] refer to the study codes in Table 4.1. *Total number of studies citing this taxon #Total number of use-records

***Liabum* sp. (Asteraceae)**

Spanish names:
Indigenous names: Saq sa'ab[16]
Used by (1*): Quichean Maya[16]
Used for (2#): Neurological (1)[16]; Psychological (1)[16]
Cognates:
Language contact:

***Licania platypus* (Hemsl.) Fritsch (Chrysobalanaceae)**

Spanish names: Mezon zapote; Zapote cabello; Zunso
Indigenous names: A'kchi xi't jaaca[06]; Moxpim, Jol'bob[14]; Jor b'oj[19]; Yàg-guiél-bêdz[23]
Used by (4*): Totonac[06]; Quichean Maya[14]; Western Maya[19]; Zapotec[23]
Used for (6#): Digestive (3)[06, 14, 19]; Skin (1)[23]; Pregnancy (1)[23]; General and Unspecified (1)[06]
Cognates:
Language contact: Chortí <> Zap

***Licaria capitata* (Schltdl. et Cham.) Kosterm. (Lauraceae)**

Spanish names: Laurel Negro
Indigenous names: Y¥k moko[03]
Used by (1*): Zoque[03]
Used for (4#): Digestive (1)[03]; Cardiovascular (1)[03]; Skin (1)[03]; Female genital (1)[03]
Cognates:
Language contact:

***Licaria peckii* (I. M. Johnst.) Kosterm. (Lauraceae)**

Spanish names: Laurel amarillo
Indigenous names: Puutx moko[03]
Used by (1*): Zoque[03]
Used for (2#): Digestive (1)[03]; Female genital (1)[03]
Cognates:
Language contact:

***Ligustrum lucidum* W.T.Aiton (Oleaceae)**

Spanish names: Trueno
Indigenous names: Yàg-truên[23]
Used by (1*): Zapotec[23]
Used for (1#): General and Unspecified (1)[23]
Cognates:
Language contact:

***Ligustrum sinense* Lour. (Oleaceae)**

Spanish names: Arrayana
Indigenous names:
Used by (1*): Zoque[01]
Used for (1#): Digestive (1)[01]
Cognates:
Language contact:

***Ligustrum vulgare* L. (Oleaceae)**

Spanish names:
Indigenous names:
Used by (1*): Quichean Maya[13]
Used for (1#): Skin (1)[13]
Cognates:
Language contact:

***Linum usitatissimum* L. (Linaceae)**

Spanish names: Linaza
Indigenous names:
Used by (4*): Zoque[01, 02]; Quichean Maya[12, 13]
Used for (10#): Digestive (3)[01, 12, 13]; Neurological (1)[12]; Respiratory (1)[01]; Skin (1)[12]; Urological (2)[01, 12]; General and Unspecified (2)[02, 12]
Cognates:
Language contact:

***Lippia alba* (Mill.) N.E.Br. ex Britton & P.Wilson (Verbenaceae)**

Spanish names: Malvareal, salviareal
Indigenous names: Salv' sant', uwi juyub q'ayes[13]; Tulux Q'ehen[14]; Alba noxi'na[18]; Tapontizana[26]
Used by (12*): Zoque[01, 02, 03]; Mixe[04]; Yucatecan Maya[09]; Quichean Maya[12, 13, 14]; Western Maya[18]; Zapotec[21, 22]; Nahua[26]
Used for (35#): Digestive (9)[02, 03, 04, 09, 12, 13, 18, 21, 26]; Cardiovascular (1)[12]; Musculoskeletal (3)[02, 03, 13]; Neurological (1)[22]; Psychological (2)[01, 13]; Respiratory (5)[01, 03, 12, 13, 14]; Skin (1)[26]; Pregnancy (4)[02, 04, 13, 21]; Female genital (3)[02, 03, 13]; Male genital (1)[02]; General and Unspecified (5)[03, 12, 13, 21, 22]
Cognates:
Language contact:

***Lippia graveolens* Kunth (Verbenaceae)**

Spanish names: Oregano
Indigenous names: Salb gohts[22]
Used by (6*): Huastec[07]; Yucatecan Maya[09]; Western Maya[19]; Zapotec[22]; Nahua[27, 28]
Used for (13#): Digestive (6)[07, 09, 19, 22, 27, 28]; Respiratory (1)[27]; Skin (1)[09]; Pregnancy (3)[09, 19, 27]; Female genital (2)[19, 28]
Cognates:
Language contact:

***Lippia myriocephala* Schltdl. & Cham. (Verbenaceae)**

Spanish names:
Indigenous names: Anaamte', thak te'[07]
Used by (1*): Huastec[07]
Used for (3#): Digestive (1)[07]; Skin (1)[07]; Urological (1)[07]
Cognates:
Language contact:

***Lippia* sp. (Verbenaceae)**

Spanish names: Sorosir
Indigenous names: Q'iil pim [14]; Tu'lush pim[16]
Used by (2*): Quichean Maya[14, 16]
Used for (2#): Neurological (1)[16]; Respiratory (1)[14]
Cognates:
Language contact:

***Lippia umbellata* Cav. (Verbenaceae)**

Spanish names: Palo de gusano; Salvia Sija
Indigenous names: Loq'oläj Chol Q'os[12]; Pisis nich vomol[20]; Yagangucha'a[21]
Used by (3*): Quichean Maya[12]; Western Maya[20]; Zapotec[21]
Used for (7#): Blood (1)[12]; Digestive (2)[20, 21]; Musculoskeletal (1)[12]; Neurological (1)[12]; Respiratory (1)[12]; General and Unspecified (1)[12]
Cognates:
Language contact:

[01-28]refer to the study codes in Table 4.1. *Total number of studies citing this taxon #Total number of use-records

***Liquidambar* sp. (Altingiaceae)**

Spanish names: Ocosote
Indigenous names: Bijtu'u[21]
Used by (1*): Zapotec[21]
Used for (2#): Digestive (1)[21]; General and Unspecified (1)[21]
Cognates:
Language contact:

***Liquidambar styraciflua* L. (Altingiaceae)**

Spanish names: Liquidambar, ocozote
Indigenous names: Täsy kuy[01]; T¥x cuy[03]; So'te'[20]; Suchiate[25]; Ocosotito[26]
Used by (6*): Zoque[01, 03]; Quichean Maya[13]; Western Maya[20]; Nahua[25, 26]
Used for (14#): Digestive (3)[01, 20, 25]; Musculoskeletal (2)[01, 13]; Neurological (1)[01]; Skin (3)[03, 13, 25]; Pregnancy (1)[01]; Female genital (1)[01]; General and Unspecified (3)[01, 13, 26]
Cognates: Zoq: täx;
Language contact:

***Lithachne pauciflora* (Sw.) P.Beauv. (Poaceae)**

Spanish names:
Indigenous names: Tsakam pakaab, tsakam tsahib, tsakam tseey kw'a', tsakam toom, pakaabil i kw'a'[07]
Used by (1*): Huastec[07]
Used for (2#): Neurological (1)[07]; Pregnancy (1)[07]
Cognates:
Language contact:

***Lithospermum mediale* I.M.Johnst. (Boraginaceae)**

Spanish names: Mitamorial
Indigenous names:
Used by (1*): Quichean Maya[13]
Used for (1#): Female genital (1)[13]
Cognates:
Language contact:

***Lithospermum* sp. (Boraginaceae)**

Spanish names: Té Moreal
Indigenous names:
Used by (1*): Quichean Maya[12]
Used for (2#): Digestive (1)[12]; Respiratory (1)[12]
Cognates:
Language contact:

***Litsea glaucescens* Kunth (Lauraceae)**

Spanish names: Laurel
Indigenous names: Toka tzajtza/toka tzasa/toka' ay[01]; Lawreel[07]; Ts'uj[08]; Roj Xwan[12]; Tziltzil ujch', tzis uch[20]; Guib diitz[21]
Used by (9*): Zoque[01, 02]; Huastec[07, 08]; Quichean Maya[12]; Western Maya[20]; Zapotec[21, 22, 23]
Used for (26#): Digestive (6)[01, 02, 08, 12, 20, 21]; Eye (1)[21]; Cardiovascular (1)[12]; Musculoskeletal (2)[01, 08]; Neurological (1)[08]; Psychological (2)[08, 12]; Endocrine (1)[08]; Pregnancy (4)[01, 21, 22, 23]; Female genital (2)[01, 02]; General and Unspecified (6)[01, 07, 08, 12, 21, 23]
Cognates:
Language contact: Chiapas Zoq <> Tzeltalan <> Zap

***Litsea* sp. (Lauraceae)**

Spanish names: Laurel
Indigenous names:
Used by (1*): Totonac[06]
Used for (5#): Digestive (1)[06]; Neurological (1)[06]; Psychological (1)[06]; Respiratory (1)[06]; General and Unspecified (1)[06]
Cognates:
Language contact:

***Lobelia cardinalis* L. (Campanulaceae)**

Spanish names:
Indigenous names: Guìzh-sàntàmàrĭ[23]
Used by (1*): Zapotec[23]
Used for (1#): Respiratory (1)[23]
Cognates:
Language contact:

***Lobelia laxiflora* Kunth (Campanulaceae)**

Spanish names: Hierba de Conejo, Mejorana Morada de Agua, Chilio; Hierba de la enferma, flor de chupa
Indigenous names: Sal' Keq Aq'om K'ik[12]; Tzajal nich wamal, paj nich te', turesna wamal, prima najk, pameyat[20]; Otzpacxihuitl[26]
Used by (3*): Quichean Maya[12]; Western Maya[20]; Nahua[26]
Used for (8#): Digestive (1)[20]; Cardiovascular (1)[12]; Musculoskeletal (2)[12, 20]; Psychological (1)[12]; Skin (1)[26]; Pregnancy (1)[20]; Female genital (1)[20]
Cognates:
Language contact:

***Lobelia* sp. (Campanulaceae)**

Spanish names:
Indigenous names: Guìzh-guièe-dzĭn̲g, guièe-dán, guièe-mĕets, guièe-nàrânj[23]
Used by (1*): Zapotec[23]
Used for (2#): Skin (1)[23]; General and Unspecified (1)[23]
Cognates:
Language contact:

***Loeselia ciliata* L. (Polemoniaceae)**

Spanish names: Jaboncillo, espinozillo de cerro
Indigenous names:
Used by (1*): Zapotec[21]
Used for (3#): Skin (1)[21]; Female genital (1)[21]; General and Unspecified (1)[21]
Cognates:
Language contact:

***Loeselia coerulea* (Cav.) G. Don (Polemoniaceae)**

Spanish names:
Indigenous names: Škwan jehb las[22]
Used by (1*): Zapotec[22]
Used for (1#): General and Unspecified (1)[22]
Cognates:
Language contact:

***Loeselia mexicana*** **(Lam.) Brand (Polemoniaceae)**

Spanish names: Espinosilla; Espinosillo; Espinosillo, yerba de espanto; Espinozillo
Indigenous names: Škwan jehb[22]; Huitzitziquitl[28]
Used by (4*): Zoque[02]; Zapotec[21, 22]; Nahua[28]
Used for (10#): Digestive (2)[21, 22]; Respiratory (1)[28]; Skin (1)[21]; Urological (1)[22]; Pregnancy (1)[22]; General and Unspecified (4)[02, 21, 22, 28]
Cognates:
Language contact:

***Loeselia*** **sp. (Polemoniaceae)**

Spanish names: Espinosilla
Indigenous names: Spìnòsî[23]
Used by (1*): Zapotec[23]
Used for (6#): Digestive (1)[23]; Neurological (1)[23]; Respiratory (1)[23]; Skin (1)[23]; Urological (1)[23]; General and Unspecified (1)[23]
Cognates:
Language contact:

***Lonchocarpus guatemalensis*** **Benth. (Fabaceae)**

Spanish names:
Indigenous names: Xuul[09]
Used by (1*): Yucatecan Maya[09]
Used for (3#): Musculoskeletal (1)[09]; Neurological (1)[09]; General and Unspecified (1)[09]
Cognates:
Language contact:

***Lonchocarpus punctatus*** **Kunth (Fabaceae)**

Spanish names:
Indigenous names: Balche'[09]
Used by (1*): Yucatecan Maya[09]
Used for (2#): Respiratory (1)[09]; General and Unspecified (1)[09]
Cognates:
Language contact:

***Lopezia racemosa*** **Cav. (Onagraceae)**

Spanish names: Hierba de la araña
Indigenous names: Xalu vomol[20]
Used by (2*): Western Maya[20]; Nahua[26]
Used for (3#): Digestive (1)[20]; Musculoskeletal (1)[26]; Skin (1)[26]
Cognates:
Language contact:

***Lophosoria quadripinnata*** **(J.F. Gmel.) C. Chr. (Dicksoniaceae)**

Spanish names: Zarzaparilla
Indigenous names:
Used by (1*): Nahua[25]
Used for (1#): Urological (1)[25]
Cognates:
Language contact:

***Louteridium donnell-smithii*** **S.Watson (Acanthaceae)**

Spanish names:
Indigenous names: Ojoj'[14]
Used by (1*): Quichean Maya[14]
Used for (2#): Digestive (1)[14]; General and Unspecified (1)[14]
Cognates:
Language contact:

[01-28]refer to the study codes in Table 4.1. *Total number of studies citing this taxon #Total number of use-records

**_Loxothysanus pedunculatus_ Rydb. (Asteraceae)**

Spanish names:
Indigenous names: Thak pux, chak pux[07]
Used by (1*): Huastec[07]
Used for (2#): Psychological (1)[07]; General and Unspecified (1)[07]
Cognates:
Language contact:

**_Loxothysanus sinuatus_ (Less.) B.L.Rob. (Asteraceae)**

Spanish names:
Indigenous names: W¥y¥ ay[03]; Kaax ujts[04]
Used by (2*): Zoque[03]; Mixe[04]
Used for (2#): Skin (2)[03, 04]
Cognates:
Language contact:

**_Ludwigia octovalvis_ (Jacq.) P.H.Raven (Onagraceae)**

Spanish names: Clavillo; Clavito
Indigenous names: Clavo sotyi[03]; Nø monda ujts[04]; Kla'uxa pim[14]
Used by (4*): Zoque[03]; Mixe[04]; Quichean Maya[14]; Nahua[26]
Used for (9#): Digestive (2)[03, 14]; Neurological (1)[03]; Respiratory (2)[03, 26]; Skin (2)[04, 26]; Female genital (1)[14]; General and Unspecified (1)[14]
Cognates:
Language contact:

**_Ludwigia_ sp. (Onagraceae)**

Spanish names: Clavillo
Indigenous names: Tzentlialeche[26]
Used by (1*): Nahua[26]
Used for (1#): Digestive (1)[26]
Cognates:
Language contact:

**_Luehea speciosa_ Willd. (Malvaceae)**

Spanish names: Tepecacao
Indigenous names: Cang cang pujki[03]; K'askat[09]
Used by (2*): Zoque[03]; Yucatecan Maya[09]
Used for (5#): Skin (2)[03, 09]; Urological (1)[03]; Female genital (1)[03]; General and Unspecified (1)[03]
Cognates:
Language contact: Highland Popoluca <> Yuc

**_Luffa cylindrica_ (L.) M.Roem. (Cucurbitaceae)**

Spanish names: Estropajo; Limpion
Indigenous names: Po't[04]
Used by (3*): Mixe[04]; Yucatecan Maya[09]; Zapotec[21]
Used for (4#): Digestive (1)[09]; Skin (2)[04, 21]; Urological (1)[09]
Cognates:
Language contact:

**_Lupinus mexicanus_ Lag. (Fabaceae)**

Spanish names: Alfalfa
Indigenous names: Much' Q'os[12]
Used by (1*): Quichean Maya[12]
Used for (2#): Blood (1)[12]; Pregnancy (1)[12]
Cognates:
Language contact:

[01-28]refer to the study codes in Table 4.1. *Total number of studies citing this taxon #Total number of use-records

***Lycianthes lenta*** **(Cav.) Bitter (Solanaceae)**

Spanish names: Majagua blanco
Indigenous names: Popo po'a[01]
Used by (1*): Zoque[01]
Used for (1#): Skin (1)[01]
Cognates:
Language contact:

***Lycianthes stephanocalyx*** **(Brandegee) Bitter (Solanaceae)**

Spanish names:
Indigenous names: Masan ay[03]
Used by (1*): Zoque[03]
Used for (1#): Skin (1)[03]
Cognates:
Language contact:

***Lycianthes synanthera*** **(Sendtn.) Bitter (Solanaceae)**

Spanish names:
Indigenous names: Roq' Xa'an[14]
Used by (1*): Quichean Maya[14]
Used for (1#): Skin (1)[14]
Cognates:
Language contact:

***Lycopodium clavatum*** **L. (Lycopodiaceae)**

Spanish names: Licopodio
Indigenous names:
Used by (1*): Zoque[01]
Used for (1#): Cardiovascular (1)[01]
Cognates:
Language contact:

***Lycoseris crocata*** **(Bertol.) S.F.Blake (Asteraceae)**

Spanish names: Santa María
Indigenous names:
Used by (1*): Quichean Maya[14]
Used for (1#): Skin (1)[14]
Cognates:
Language contact:

***Lygodium heterodoxum*** **Kunze (Lygodiaceae)**

Spanish names: Alambrilla; Atagota de bejuco
Indigenous names: Naxiui[03]; Rich' Mu' li' ba', Ruxb'i Kaaq'i[14]; Ruxb'i kaak[17]
Used by (3*): Zoque[03]; Quichean Maya[14, 17]
Used for (4#): Musculoskeletal (1)[14]; Neurological (1)[14]; Skin (1)[03]; nd[17]
Cognates: Quich: ruxb'i kaak;
Language contact:

***Lygodium*** **sp. (Lygodiaceae)**

Spanish names:
Indigenous names: Ruxb'i'kaak[16]
Used by (1*): Quichean Maya[16]
Used for (3#): Neurological (1)[16]; Psychological (1)[16]; General and Unspecified (1)[16]
Cognates:
Language contact:

***Lygodium venustum* Sw. (Lygodiaceae)**

Spanish names: Hoja de la vibora, curalina
Indigenous names: Zajin syingtzyi/o'osi ay[02]; Naxiui[03]; Kuti' iny ujts[04]; K'util papaam, paxlaab papaan[07]; Ruxb'i kaak[17]; Yop'te hacha[18]; Guixa'a mbala'a[21]
Used by (7*): Zoque[02, 03]; Mixe[04]; Huastec[07]; Quichean Maya[17]; Western Maya[18]; Zapotec[21]
Used for (16[#]): Digestive (2)[03, 07]; Psychological (2)[02, 07]; Skin (4)[02, 03, 04, 21]; Endocrine (1)[07]; Urological (3)[02, 03, 18]; Pregnancy (1)[07]; Female genital (1)[02]; General and Unspecified (1)[07]; nd[17]
Cognates:
Language contact: Mixe <> Hua; Hua <> Zap

***Lysiloma acapulcense* (Kunth) Benth. (Fabaceae)**

Spanish names: Tepehuaje, tehuaje
Indigenous names: Wayal[07]; Tehuaxi[28]
Used by (2*): Huastec[07]; Nahua[28]
Used for (8[#]): Digestive (2)[07, 28]; Neurological (1)[07]; Psychological (1)[07]; Respiratory (1)[28]; Skin (1)[07]; Female genital (1)[07]; General and Unspecified (1)[07]
Cognates:
Language contact:

***Lysiloma aurita* (Schltdl.) Benth. (Fabaceae)**

Spanish names: Songuavite
Indigenous names: Cana cuy[03]
Used by (1*): Zoque[03]
Used for (4[#]): Digestive (1)[03]; Skin (1)[03]; Endocrine (1)[03]; Female genital (1)[03]
Cognates:
Language contact:

***Lysiloma divaricatum* (Jacq.) J.F.Macbr. (Fabaceae)**

Spanish names: Guaje del campo; Timbre
Indigenous names: Lya[22]
Used by (2*): Zapotec[22]; Nahua[26]
Used for (2[#]): Digestive (2)[22, 26]
Cognates:
Language contact:

***Lysiloma latisiliquum* (L.) Benth. (Fabaceae)**

Spanish names:
Indigenous names: Tsalam[09]
Used by (1*): Yucatecan Maya[09]
Used for (1[#]): Neurological (1)[09]
Cognates:
Language contact:

***Lysiloma* sp. (Fabaceae)**

Spanish names: Quebracho
Indigenous names: Yaj[19]
Used by (1*): Western Maya[19]
Used for (2[#]): Digestive (1)[19]; Neurological (1)[19]
Cognates:
Language contact:

[01-28]refer to the study codes in Table 4.1. *Total number of studies citing this taxon [#]Total number of use-records

***Lythrum gracile* Benth. (Lythraceae)**

Spanish names:
Indigenous names: Lehem ts'ohool, tsab k'a'um, itsaan an maan witsiil[07]; Tlalhuayopactle[26]
Used by (2*): Huastec[07]; Nahua[26]
Used for (4#): Neurological (1)[26]; Respiratory (1)[26]; Skin (1)[26]; General and Unspecified (1)[07]
Cognates:
Language contact:

***Macadamia ternifolia* F.Muell. (Proteaceae)**

Spanish names: Macadamia
Indigenous names:
Used by (1*): Quichean Maya[12]
Used for (5#): Digestive (1)[12]; Neurological (1)[12]; Psychological (1)[12]; Skin (1)[12]; General and Unspecified (1)[12]
Cognates:
Language contact:

***Machaerium cirrhiferum* Pittier (Fabaceae)**

Spanish names:
Indigenous names: Lokoch k'ix[17]
Used by (1*): Quichean Maya[17]
Used for (1#): nd[17]
Cognates:
Language contact:

***Machaerium cobanense* Donn.Sm. (Fabaceae)**

Spanish names: Uña de gato; Uña de gato/sangre de cristo
Indigenous names: Misi k¥ts¥s[03]
Used by (2*): Zoque[01, 03]
Used for (3#): Skin (1)[03]; Endocrine (1)[03]; General and Unspecified (1)[01]
Cognates:
Language contact:

***Machaerium floribundum* Benth. (Fabaceae)**

Spanish names:
Indigenous names: N¥pin tsay[03]; Santo no'ot[04]
Used by (2*): Zoque[03]; Mixe[04]
Used for (5#): Digestive (2)[03, 04]; Skin (2)[03, 04]; Female genital (1)[03]
Cognates:
Language contact:

***Machaerium isadelphum* (E.Mey) Standl. (Fabaceae)**

Spanish names: Uña de gavilán
Indigenous names: Powui kä'tzi ma'syi[02]; N¥pin tsay[03]
Used by (2*): Zoque[02, 03]
Used for (4#): Digestive (1)[03]; Neurological (1)[02]; Skin (1)[03]; Female genital (1)[03]
Cognates:
Language contact:

***Machaerium salvadorense* (Donn.Sm.) Rudd (Fabaceae)**

Spanish names: Uña de gavilan
Indigenous names: N¥pin tsay[03]; Itsik' t'iiw[07]; Pur caham[16]
Used by (4*): Zoque[01, 03]; Huastec[07]; Quichean Maya[16]
Used for (5#): Neurological (1)[07]; Psychological (1)[16]; Skin (1)[03]; Female genital (1)[03]; General and Unspecified (1)[01]
Cognates:
Language contact:

[01-28]refer to the study codes in Table 4.1. *Total number of studies citing this taxon #Total number of use-records

***Maclura tinctoria* (L.) D.Don ex Steud. (Moraceae)**

Spanish names: Murra
Indigenous names: Tsitsiy[07]
Used by (2*): Zoque[03]; Huastec[07]
Used for (8#): Musculoskeletal (1)[07]; Neurological (2)[03, 07]; Respiratory (1)[07]; Skin (1)[07]; Urological (1)[07]; General and Unspecified (2)[03, 07]
Cognates:
Language contact:

***Macrothelypteris torresiana* (Gaudich.) Ching (Thelypteridaceae)**

Spanish names: Helecho silvestre/ macho
Indigenous names: Ocopetate[26]
Used by (1*): Nahua[26]
Used for (2#): Psychological (1)[26]; Urological (1)[26]
Cognates:
Language contact:

***Magnolia grandiflora* L. (Magnoliaceae)**

Spanish names: Magnolia
Indigenous names:
Used by (1*): Quichean Maya[12]
Used for (4#): Cardiovascular (1)[12]; Neurological (1)[12]; Psychological (1)[12]; General and Unspecified (1)[12]
Cognates:
Language contact:

***Magnolia mexicana* DC. (Magnoliaceae)**

Spanish names: Flor de corazón
Indigenous names: Tzoko jäyä/Tzapi kopak [01]; Pa' ju'us/tzokoy toya jäyä[02]; Mooyniakcuy[03]; Kuwi šanat[05]; Qui'huixan, qu'yu xanat[06]; Yagabedxii[21]
Used by (6*): Zoque[01, 02, 03]; Totonac[05, 06]; Zapotec[21]
Used for (18#): Digestive (5)[01, 02, 03, 05, 21]; Cardiovascular (4)[02, 05, 06, 21]; Psychological (3)[01, 05, 06]; Pregnancy (3)[03, 05, 06]; Female genital (1)[03]; General and Unspecified (2)[01, 05]
Cognates: Zoq: tzoko; Toto: kuwi xanat;
Language contact: Nah <> Zoq <> Tot

***Magnolia schiedeana* Schltl. (Magnoliaceae)**

Spanish names: Flor de corazón, magnolia
Indigenous names:
Used by (1*): Zapotec[21]
Used for (2#): Digestive (1)[21]; Cardiovascular (1)[21]
Cognates:
Language contact:

***Maianthemum flexuosum* (Bertol.) LaFrankie (Asparagaceae)**

Spanish names: Vara de Salomón
Indigenous names:
Used by (1*): Quichean Maya[12]
Used for (3#): Cardiovascular (1)[12]; Urological (1)[12]; General and Unspecified (1)[12]
Cognates:
Language contact:

***Malachra alceifolia* Jacq. (Malvaceae)**

Spanish names: Hierba de Cancer; Malva; Malva peluda
Indigenous names: Tza' Tzalun Mi' Ha'[14]
Used by (3*): Zoque[01]; Quichean Maya[14]; Western Maya[18]
Used for (4#): Digestive (1)[18]; Psychological (1)[01]; Urological (1)[01]; General and Unspecified (1)[14]
Cognates:
Language contact:

***Malachra capitata* (L.) L. (Malvaceae)**

Spanish names: Malva
Indigenous names: Pulik thipon, paktha' thipon[07]
Used by (2*): Zoque[01]; Huastec[07]
Used for (3#): Urological (1)[01]; General and Unspecified (2)[01, 07]
Cognates:
Language contact:

***Malouetia guatemalensis* (Müll.Arg.) Standl. (Apocynaceae)**

Spanish names: Mbiigu' moradu
Indigenous names:
Used by (1*): Zapotec[21]
Used for (1#): Skin (1)[21]
Cognates:
Language contact:

***Malpighia glabra* L. (Malpighiaceae)**

Spanish names:
Indigenous names: K'ak'al ilaal[07]
Used by (1*): Huastec[07]
Used for (3#): Neurological (1)[07]; Respiratory (1)[07]; General and Unspecified (1)[07]
Cognates:
Language contact:

***Malus domestica* Borkh. (Rosaceae)**

Spanish names: Manzana
Indigenous names: Yàg-mànzân[23]
Used by (1*): Zapotec[23]
Used for (1#): Respiratory (1)[23]
Cognates:
Language contact:

***Malus* sp. (Rosaceae)**

Spanish names: Manzana
Indigenous names:
Used by (1*): Quichean Maya[12]
Used for (4#): Digestive (1)[12]; Eye (1)[12]; Respiratory (1)[12]; General and Unspecified (1)[12]
Cognates:
Language contact:

***Malva parviflora* L. (Malvaceae)**

Spanish names: Malva
Indigenous names: Tankilkixit[08]; Cho'j, tzelej, tze'ek[13]; Gišlobeh [22]; Blàg-mêd, guìzh-blàg-mêd, mâlb[23]
Used by (8*): Zoque[01]; Huastec[08]; Quichean Maya[12, 13]; Zapotec[22, 23]; Nahua[25, 26]
Used for (35#): Blood (1)[12]; Digestive (4)[01, 12, 13, 22]; Eye (1)[13]; Cardiovascular (1)[12]; Musculoskeletal (1)[01]; Neurological (1)[12]; Psychological (2)[12, 13]; Respiratory (2)[13, 25]; Skin (6)[01, 08, 12, 13, 23, 25]; Urological (3)[12, 13, 22]; Pregnancy (2)[22, 26]; Female genital (5)[01, 12, 13, 23, 26]; General and Unspecified (6)[01, 12, 13, 22, 23, 25]
Cognates:
Language contact:

***Malva sylvestris* L. (Malvaceae)**

Spanish names: Malva
Indigenous names:
Used by (1*): Quichean Maya[13]
Used for (2#): Digestive (1)[13]; General and Unspecified (1)[13]
Cognates:
Language contact:

***Malvastrum americanum* (L.) Torr. (Malvaceae)**

Spanish names:
Indigenous names: Thipon, manath thipon[07]
Used by (1*): Huastec[07]
Used for (3#): Digestive (1)[07]; Skin (1)[07]; General and Unspecified (1)[07]
Cognates:
Language contact:

***Malvastrum coromandelianum* (L.) Garcke (Malvaceae)**

Spanish names: Malva
Indigenous names: Lantha thipon[07]
Used by (2*): Zoque[03]; Huastec[07]
Used for (2#): Psychological (1)[03]; Skin (1)[07]
Cognates:
Language contact:

***Malvaviscus penduliflorus* Moc. & Sessé ex DC. (Malvaceae)**

Spanish names: Orín de diablo/tulipán/Chavelita de jardín
Indigenous names: Kin nyäpin[01]
Used by (1*): Zoque[01]
Used for (4#): Digestive (1)[01]; Musculoskeletal (1)[01]; Respiratory (1)[01]; General and Unspecified (1)[01]
Cognates:
Language contact:

***Malvaviscus achanioides* (Turcz.) Fryxell (Malvaceae)**

Spanish names: Chavelita del monte
Indigenous names:
Used by (1*): Zoque[01]
Used for (2#): Musculoskeletal (1)[01]; General and Unspecified (1)[01]
Cognates:
Language contact:

***Malvaviscus arboreus* Cav. (Malvaceae)**

Spanish names: Monacillo, tulipán de monte
Indigenous names: Xoun pocuy[03]; Xuuxy aay[04]; Ix bek'em, thoot wits[07]; Bisil-che', Bisil, Holol[09]; Ru kotzij tzunum, Muxu'xkej, Räq' Q'os Aq'om[12]; Oxib Xbonol[14]; Yopo'aj ts'ibi[18]; Tulipan duendi[21]
Used by (10*): Zoque[03]; Mixe[04]; Huastec[07]; Yucatecan Maya[09]; Quichean Maya[12, 13, 14]; Western Maya[18]; Zapotec[21]; Nahua[26]
Used for (31#): Digestive (7)[03, 04, 07, 09, 12, 13, 18]; Eye (1)[18]; Musculoskeletal (1)[07]; Respiratory (4)[03, 07, 21, 26]; Skin (5)[03, 12, 18, 21, 26]; Endocrine (1)[14]; Urological (2)[03, 21]; Pregnancy (2)[04, 07]; Female genital (3)[03, 07, 12]; General and Unspecified (5)[03, 07, 12, 13, 21]
Cognates:
Language contact:

***Malvaviscus lanceolatus* Rose (Malvaceae)**

Spanish names: Tulipancito del rio
Indigenous names: Tzyi juts[02]
Used by (1*): Zoque[02]
Used for (2#): Digestive (1)[02]; Urological (1)[02]
Cognates:
Language contact:

***Mandevilla subsagittata* (Ruiz & Pav.) Woodson (Apocynaceae)**

Spanish names:
Indigenous names: Katx muk[03]
Used by (1*): Zoque[03]
Used for (1#): Skin (1)[03]
Cognates:
Language contact:

***Manfreda maculosa* (Hook.) Rose (Asparagaceae)**

Spanish names:
Indigenous names: Pets'k'im, Pets'k'inil[09]
Used by (1*): Yucatecan Maya[09]
Used for (1#): Neurological (1)[09]
Cognates:
Language contact:

***Manfreda pubescens* (Regel & Ortgies) Verh.-Will. ex Espejo & López-Ferr. (Asparagaceae)**

Spanish names: Asosena sabanera
Indigenous names: Copa asosena[03]
Used by (1*): Zoque[03]
Used for (2#): Skin (1)[03]; Pregnancy (1)[03]
Cognates:
Language contact:

***Manfreda variegata* (Jacobi) Rose (Asparagaceae)**

Spanish names:
Indigenous names: Pulik k'oyol, k'oyol ist'aamal, eemil kw'a'[07]
Used by (1*): Huastec[07]
Used for (3#): Neurological (1)[07]; Respiratory (1)[07]; Skin (1)[07]
Cognates:
Language contact:

[01-28]refer to the study codes in Table 4.1. *Total number of studies citing this taxon #Total number of use-records

***Mangifera indica* L. (Anacardiaceae)**

Spanish names: Mango
Indigenous names: Manku[03]; Mang aay[04]; Q'anatz'ub'[12]; Ma'nk'[14]; U pam[18]; Malak'[19]; Manko[20]; Yàg-mângw[23]; Maniltzapotl[28]
Used by (15*): Zoque[01, 02, 03]; Mixe[04]; Totonac[06]; Yucatecan Maya[09]; Quichean Maya[12, 13, 14]; Western Maya[18, 19, 20]; Zapotec[21, 23]; Nahua[28]
Used for (38#): Digestive (10)[01, 02, 03, 06, 12, 13, 18, 19, 20, 21]; Cardiovascular (1)[02]; Musculoskeletal (3)[01, 02, 06]; Neurological (3)[03, 18, 23]; Respiratory (7)[02, 03, 12, 14, 19, 21, 28]; Skin (3)[01, 03, 21]; Urological (1)[03]; Pregnancy (1)[09]; Female genital (3)[01, 02, 03]; General and Unspecified (6)[01, 02, 04, 09, 12, 21]
Cognates:
Language contact:

***Manihot esculenta* Crantz (Euphorbiaceae)**

Spanish names: Yucca
Indigenous names: Pisi[01]; T'inche'[07]; Ts'iim[09]; Tzín[13]; Tz'in[18]; Guyaajaca'a[21]
Used by (7*): Zoque[01, 03]; Huastec[07]; Yucatecan Maya[09]; Quichean Maya[13]; Western Maya[18]; Zapotec[21]
Used for (10#): Digestive (5)[01, 03, 07, 13, 18]; Skin (1)[09]; Female genital (1)[18]; General and Unspecified (3)[01, 09, 21]
Cognates: Mayan: t(s)'in; CoreM: tsin/tsim;
Language contact: Maya > Chiapas Zoq

***Manilkara chicle* (Pittier) Gilly (Sapotaceae)**

Spanish names:
Indigenous names: Jiya[03]
Used by (1*): Zoque[03]
Used for (1#): Female genital (1)[03]
Cognates:
Language contact:

***Manilkara zapota* (L.) P.Royen (Sapotaceae)**

Spanish names: Chicozapote
Indigenous names: Ji'ya[01]; Jiya[02]; Jiya[03]; Skalu jaka[05]; Ak'so sual[06]; Tsab it'ath[07]; Ya'[09]; Ya[10]; Chäbte'[18]; Wolja'as[18]; Guil ziji[21]
Used by (12*): Zoque[01, 02, 03]; Totonac[05, 06]; Huastec[07]; Yucatecan Maya[09, 10]; Quichean Maya[13]; Western Maya[18]; Zapotec[21]; Nahua[24]
Used for (20#): Digestive (8)[06, 07, 09, 10, 13, 18, 18, 21]; Cardiovascular (2)[01, 02]; Psychological (1)[02]; Skin (3)[01, 03, 05]; Endocrine (1)[18]; Urological (1)[03]; Female genital (2)[03, 24]; Male genital (1)[01]; General and Unspecified (1)[21]
Cognates: Zoq: jiya; Yuca: ya;
Language contact: Zoq > Tot and Yuc

***Mansoa alliacea* (Lam.) A.H.Gentry (Bignoniaceae)**

Spanish names: Hierba de ajo/hoja de ajo
Indigenous names: Asyus ay[01]; Aaaxux ts'aah[07]
Used by (2*): Zoque[01]; Huastec[07]
Used for (7#): Digestive (1)[07]; Musculoskeletal (2)[01, 07]; Respiratory (1)[01]; Skin (1)[07]; General and Unspecified (2)[01, 07]
Cognates:
Language contact: Chiapas Zoq <> Hua

***Maranta arundinacea* L. (Marantaceae)**

Spanish names: Hoja de Sahgún
Indigenous names: Uaja[03]; T'aaw', tsakam thulup[07]; Chaak[09]
Used by (4*): Zoque[03]; Totonac[06]; Huastec[07]; Yucatecan Maya[09]
Used for (9#): Digestive (3)[06, 07, 09]; Skin (1)[03]; Urological (2)[06, 07]; Female genital (1)[07]; General and Unspecified (2)[06, 07]
Cognates:
Language contact: Highland Popoluca <> Hua <> Yuc

[01-28]refer to the study codes in Table 4.1. *Total number of studies citing this taxon #Total number of use-records

***Maranta gibba* Sm. (Marantaceae)**

Spanish names:
Indigenous names: T'aaw' ok[07]
Used by (1*): Huastec[07]
Used for (1#): Digestive (1)[07]
Cognates:
Language contact:

***Marathrum* sp. (Podostemaceae)**

Spanish names:
Indigenous names: N¥muk, Niu muk[03]
Used by (1*): Zoque[03]
Used for (3#): Skin (1)[03]; Female genital (1)[03]; General and Unspecified (1)[03]
Cognates:
Language contact:

***Marchantia* sp. (Marchantiaceae)**

Spanish names:
Indigenous names: Miim ha'[07]
Used by (1*): Huastec[07]
Used for (2#): Skin (1)[07]; General and Unspecified (1)[07]
Cognates:
Language contact:

***Margaranthus solanaceous* Schltdl. (Solanaceae)**

Spanish names: Totomache
Indigenous names: Tuthaayil an t'iiw[07]
Used by (2*): Huastec[07]; Nahua[27]
Used for (4#): Digestive (2)[07, 27]; Psychological (1)[27]; Endocrine (1)[27]
Cognates:
Language contact:

***Margaritopsis microdon* (DC.) C.M.Taylor (Rubiaceae)**

Spanish names:
Indigenous names: Ketsu ts'ohool, wiichab ts'ohool, pulik puut' ts'aah[07]; Xbakalik'[09]
Used by (2*): Huastec[07]; Yucatecan Maya[09]
Used for (3#): Neurological (1)[09]; Skin (1)[07]; General and Unspecified (1)[07]
Cognates:
Language contact:

***Marina scopa* Barneby (Fabaceae)**

Spanish names:
Indigenous names: Tiith olom[07]
Used by (1*): Huastec[07]
Used for (1#): General and Unspecified (1)[07]
Cognates:
Language contact:

***Marrubium vulgare* L. (Lamiaceae)**

Spanish names: Marrubio
Indigenous names: Pition gihš[22]; Tzopiloshihuitl[28]
Used by (5*): Zapotec[22, 23]; Nahua[26, 27, 28]
Used for (14#): Digestive (4)[23, 26, 27, 28]; Musculoskeletal (1)[23]; Neurological (1)[28]; Psychological (2)[26, 27]; Skin (3)[22, 27, 28]; Urological (1)[28]; General and Unspecified (2)[27, 28]
Cognates:
Language contact:

***Marsdenia macrophylla*** **(Humb. & Bonpl. ex Schult.) E.Fourn. (Apocynaceae)**

Spanish names:
Indigenous names: Tan ooy[07]
Used by (1*): Huastec[07]
Used for (1#): Skin (1)[07]
Cognates:
Language contact:

***Marsdenia mexicana*** **Decne. (Apocynaceae)**

Spanish names: Yerba santa del campo
Indigenous names: Bala šoh gihš[22]
Used by (1*): Zapotec[22]
Used for (5#): Musculoskeletal (1)[22]; Neurological (1)[22]; Respiratory (1)[22]; Skin (1)[22]; General and Unspecified (1)[22]
Cognates:
Language contact:

***Martynia annua*** **L. (Martyniaceae)**

Spanish names: Uña de gato
Indigenous names: Joun k¥ts¥s[03]; Itsik' kuxkum[07]; Rixiij miss'[14]
Used by (6*): Zoque[01, 02, 03]; Huastec[07]; Yucatecan Maya[09]; Quichean Maya[14]
Used for (14#): Digestive (1)[14]; Eye (1)[02]; Cardiovascular (1)[02]; Musculoskeletal (1)[02]; Respiratory (1)[07]; Endocrine (2)[01, 02]; Urological (1)[09]; Female genital (2)[03, 14]; Male genital (1)[02]; General and Unspecified (3)[02, 03, 07]
Cognates:
Language contact:

***Matayba oppositifolia*** **(A.Rich.) Britton (Sapindaceae)**

Spanish names:
Indigenous names: Pix pix cuy[03]; Säk uayum[11]
Used by (2*): Zoque[03]; Yucatecan Maya[11]
Used for (3#): Digestive (1)[03]; Skin (1)[11]; Female genital (1)[03]
Cognates:
Language contact:

***Matelea denticulata*** **(Vahl) Fontella & E.A.Schwarz (Apocynaceae)**

Spanish names:
Indigenous names: Piin-k'ak', Kuyuch-ak', Xp'okini, Emtsul[09]
Used by (1*): Yucatecan Maya[09]
Used for (1#): Skin (1)[09]
Cognates:
Language contact:

***Matricaria chamomilla*** **L. (Asteraceae)**

Spanish names: Manzanilla
Indigenous names: Ix bek'em paktda'[08]; Mar'san la', mazne'y[13]; Mantzaniya[20]; Manzaniy neš[22]; Guièe-mànzànî[23]
Used by (16*): Mixe[04]; Totonac[05, 06]; Huastec[08]; Quichean Maya[12, 13, 14]; Western Maya[19, 20]; Zapotec[21, 22, 23]; Nahua[25, 26, 27, 28]
Used for (57#): Blood (1)[12]; Digestive (14)[04, 05, 06, 08, 12, 13, 20, 21, 22, 23, 25, 26, 27, 28]; Eye (4)[08, 13, 27, 28]; Ear (2)[04, 23]; Cardiovascular (2)[08, 12]; Musculoskeletal (3)[12, 13, 19]; Neurological (5)[12, 13, 19, 22, 28]; Psychological (2)[12, 13]; Respiratory (6)[08, 13, 14, 19, 25, 27]; Skin (3)[04, 21, 26]; Urological (2)[12, 22]; Pregnancy (4)[13, 19, 21, 22]; Female genital (3)[12, 13, 19]; Male genital (1)[12]; General and Unspecified (5)[08, 12, 19, 22, 27]
Cognates:
Language contact:

***Matricaria discoidea* DC. (Asteraceae)**

Spanish names: Manzanilla
Indigenous names:
Used by (2*): Zoque[01, 02]
Used for (17#): Digestive (2)[01, 02]; Eye (2)[01, 02]; Musculoskeletal (1)[01]; Neurological (1)[01]; Psychological (1)[02]; Respiratory (2)[01, 02]; Pregnancy (2)[01, 02]; Female genital (3)[01, 01, 02]; Male genital (1)[01]; General and Unspecified (2)[01, 02]
Cognates:
Language contact:

***Matricaria* sp. (Asteraceae)**

Spanish names: Manzanilla
Indigenous names: Manzaniya[07]
Used by (2*): Zoque[03]; Huastec[07]
Used for (5#): Digestive (2)[03, 07]; Musculoskeletal (1)[03]; Psychological (1)[03]; Pregnancy (1)[03]
Cognates:
Language contact:

***Maxillariella tenuifolia* (Lindl.) M.A.Blanco & Carnevali (Orchidaceae)**

Spanish names:
Indigenous names: Kowa nokcha[03]
Used by (1*): Zoque[03]
Used for (1#): Digestive (1)[03]
Cognates:
Language contact:

***Maytenus belizensis* Standl. (Celastraceae)**

Spanish names: Retamo
Indigenous names: Ñiwi cuy [03]
Used by (1*): Zoque[03]
Used for (6#): Digestive (1)[03]; Neurological (1)[03]; Skin (1)[03]; Urological (1)[03]; Pregnancy (1)[03]; Female genital (1)[03]
Cognates:
Language contact:

***Mecardonia procumbens* (Mill.) Small (Plantaginaceae)**

Spanish names: Chotete
Indigenous names: Xotete[03]; Ncuàan-dzéb-maêstr, ncuàan-dzéb-ròo, ncuàan-dzéb-guièel[23]; Tlaquexahuil[26]
Used by (3*): Zoque[03]; Zapotec[23]; Nahua[26]
Used for (5#): Blood (1)[03]; Skin (1)[26]; Female genital (1)[03]; General and Unspecified (2)[03, 23]
Cognates:
Language contact:

***Medicago sativa* L. (Fabaceae)**

Spanish names: Alfalfa
Indigenous names: Alfâlf, guìzh-frôw, guìzh-àlfâlf[23]
Used by (3*): Quichean Maya[13]; Zapotec[23]; Nahua[28]
Used for (4#): Psychological (1)[23]; Respiratory (1)[13]; Endocrine (1)[28]; Urological (1)[28]
Cognates:
Language contact:

***Melampodium divaricatum* (Rich. ex Rich.) DC. (Asteraceae)**

Spanish names: Acahuale amarillo; Azaján Criollo; Hoja de azar
Indigenous names: Tza'a tzoy[01]; Smucucu makatama[05]; R'u Wai kej[12]
Used by (3*): Zoque[01]; Totonac[05]; Quichean Maya[12]
Used for (5#): Digestive (1)[01]; Cardiovascular (1)[05]; Musculoskeletal (1)[12]; General and Unspecified (2)[01, 12]
Cognates:
Language contact:

***Melanthera nivea*** **(L.) Small (Asteraceae)**

Spanish names: Rosita
Indigenous names:
Used by (1*): Nahua[26]
Used for (1#): Respiratory (1)[26]
Cognates:
Language contact:

***Melia azedarach*** **L. (Meliaceae)**

Spanish names: Paraís; Paraíso; Piocha; Tarai
Indigenous names: Yàg-pàraîs[23]
Used by (5*): Zoque[03]; Totonac[05]; Western Maya[18, 19]; Zapotec[23]
Used for (18#): Blood (1)[03]; Digestive (2)[05, 23]; Ear (1)[03]; Musculoskeletal (1)[03]; Neurological (1)[19]; Skin (2)[03, 18]; Endocrine (1)[03]; Urological (1)[03]; Pregnancy (2)[03, 05]; Female genital (1)[05]; Male genital (1)[05]; General and Unspecified (4)[03, 03, 19, 23]
Cognates:
Language contact:

***Melicoccus oliviformis*** **Kunth (Sapindaceae)**

Spanish names: Guaya
Indigenous names:
Used by (1*): Western Maya[18]
Used for (1#): Digestive (1)[18]
Cognates:
Language contact:

***Melilotus indicus*** **(L.) All. (Fabaceae)**

Spanish names: Trebe
Indigenous names:
Used by (1*): Quichean Maya[12]
Used for (1#): Digestive (1)[12]
Cognates:
Language contact:

***Melilotus*** **sp. (Fabaceae)**

Spanish names:
Indigenous names: Alfâlf,  guìzh-frôw, guìzh-àlfâlf[23]
Used by (1*): Zapotec[23]
Used for (1#): Psychological (1)[23]
Cognates:
Language contact:

***Melissa officinalis*** **L. (Lamiaceae)**

Spanish names: Toronjil (de menta)
Indigenous names: Utz[18]
Used by (1*): Western Maya[18]
Used for (6#): Digestive (1)[18]; Neurological (1)[18]; Psychological (1)[18]; Respiratory (1)[18]; Endocrine (1)[18]; General and Unspecified (1)[18]
Cognates:
Language contact:

[01-28]refer to the study codes in Table 4.1. *Total number of studies citing this taxon #Total number of use-records

***Melochia nodiflora* Sw. (Malvaceae)**

Spanish names: Malva morada; Malva rosada
Indigenous names: Tsakam akich, tsabaal akich[07]; Lexuba'a moradu'u[21]
Used by (3*): Zoque[02]; Huastec[07]; Zapotec[21]
Used for (8#): Digestive (1)[07]; Skin (1)[21]; Urological (1)[07]; Pregnancy (1)[07]; Female genital (1)[21]; General and Unspecified (3)[02, 07, 21]
Cognates:
Language contact:

***Melochia pyramidata* L. (Malvaceae)**

Spanish names:
Indigenous names: Ehtiil i tsakam akich[07]
Used by (1*): Huastec[07]
Used for (1#): Digestive (1)[07]
Cognates:
Language contact:

***Melochia tomentosa* L. (Malvaceae)**

Spanish names: Malva rosada; Malvarisco morado
Indigenous names:
Used by (2*): Zoque[02]; Zapotec[21]
Used for (5#): Digestive (1)[02]; Skin (1)[21]; Endocrine (1)[02]; Female genital (1)[21]; Male genital (1)[02]
Cognates:
Language contact:

***Melothria pendula* L. (Cucurbitaceae)**

Spanish names: Hoja de carga
Indigenous names: Tzämi ay[01]; Tsaiñicut cuy, Xandia tsay[03]; Baleeyail an t'eel[07]; Sandia cho[16]; Sandi'a ch'o[17]
Used by (5*): Zoque[01, 03]; Huastec[07]; Quichean Maya[16, 17]
Used for (6#): Digestive (1)[07]; Neurological (1)[16]; Psychological (1)[03]; Urological (1)[03]; General and Unspecified (1)[01]; nd[17]
Cognates: Zoq: tsämi/tsaiñi;
Language contact:

***Mendoncia lindavii* Rusby (Acanthaceae)**

Spanish names:
Indigenous names:
Used by (1*): Quichean Maya[17]
Used for (1#): nd[17]
Cognates:
Language contact:

***Mentha × piperita* L. (Lamiaceae)**

Spanish names: Hierbabuena, yerbabuena, menta
Indigenous names: Yepena[01]; Ujts aay[04]; Elbenax[08]; Bänälä utz[18]; Utz[18]; Bedxestila'a[21]; Kallowena[28]
Used by (12*): Zoque[01, 02]; Mixe[04]; Huastec[08]; Yucatecan Maya[09, 11]; Quichean Maya[12]; Western Maya[18]; Zapotec[21]; Nahua[25, 27, 28]
Used for (47#): Digestive (13)[01, 02, 04, 08, 09, 11, 12, 18, 18, 21, 25, 27, 28]; Cardiovascular (3)[01, 08, 21]; Musculoskeletal (3)[01, 12, 28]; Neurological (6)[01, 09, 12, 18, 18, 28]; Psychological (3)[01, 08, 18]; Respiratory (6)[01, 02, 08, 12, 18, 27]; Urological (2)[01, 12]; Female genital (4)[01, 02, 12, 18]; Male genital (1)[01]; General and Unspecified (6)[01, 08, 12, 18, 18, 21]
Cognates:
Language contact:

***Mentha × rotundifolia*** **(L.) Huds. (Lamiaceae)**

Spanish names: Monstranza; Mostranza
Indigenous names: Tlanixalnilki[26]
Used by (2*): Zapotec[22]; Nahua[26]
Used for (3#): Digestive (1)[22]; Skin (1)[26]; General and Unspecified (1)[26]
Cognates:
Language contact:

***Mentha arvensis*** **L. (Lamiaceae)**

Spanish names: Hierbabuena
Indigenous names: Castalatka'jna[05]; Caxtalaalhka 'jna', ihpupuhui'na[06]
Used by (2*): Totonac[05, 06]
Used for (7#): Digestive (2)[05, 06]; Neurological (1)[06]; Respiratory (1)[05]; Pregnancy (1)[05]; Female genital (1)[06]; General and Unspecified (1)[05]
Cognates:
Language contact:

***Mentha pulegium*** **L. (Lamiaceae)**

Spanish names: Poleo ; Poleo, pompimi
Indigenous names: Xuiky[08]
Used by (2*): Huastec[08]; Western Maya[18]
Used for (7#): Digestive (2)[08, 18]; Musculoskeletal (1)[08]; Psychological (1)[08]; Respiratory (1)[08]; General and Unspecified (2)[08, 18]
Cognates:
Language contact:

***Mentha*** **sp. (Lamiaceae)**

Spanish names: Hierbabuena, menta
Indigenous names: Elbeenax[07]; Arvino, q'ebun, purxil', alwino, pärxil, esal guach pärxil[13]; Bäč štil[22]
Used by (7*): Zoque[03]; Huastec[07]; Yucatecan Maya[09]; Quichean Maya[12, 13, 14]; Zapotec[22]
Used for (24#): Digestive (8)[03, 07, 09, 09, 12, 13, 14, 22]; Cardiovascular (1)[13]; Musculoskeletal (1)[14]; Neurological (2)[09, 12]; Psychological (1)[12]; Respiratory (3)[12, 13, 14]; Urological (1)[13]; Pregnancy (3)[03, 07, 13]; Female genital (1)[13]; General and Unspecified (3)[03, 13, 14]
Cognates:
Language contact:

***Mentha spicata*** **L. (Lamiaceae)**

Spanish names: Hierbabuena, menta, toronjil
Indigenous names: Xuuikh[08]; Wena[20]
Used by (5*): Zoque[01]; Huastec[08]; Western Maya[19, 20]; Nahua[25]
Used for (13#): Digestive (5)[01, 08, 19, 20, 25]; Ear (1)[19]; Respiratory (2)[08, 19]; Pregnancy (1)[19]; Female genital (1)[19]; General and Unspecified (3)[01, 08, 25]
Cognates:
Language contact:

***Mentha suaveolens*** **Ehrh. (Lamiaceae)**

Spanish names:
Indigenous names: Guìzh-mònstânz[23]
Used by (1*): Zapotec[23]
Used for (2#): Respiratory (1)[23]; Pregnancy (1)[23]
Cognates:
Language contact:

[01-28]refer to the study codes in Table 4.1. *Total number of studies citing this taxon #Total number of use-records

***Mentzelia aspera* L. (Loasaceae)**

Spanish names: Mala mujer
Indigenous names:
Used by (1*): Nahua[24]
Used for (1[#]): Pregnancy (1)[24]
Cognates:
Language contact:

***Mentzelia conzattii* Greenm. (Loasaceae)**

Spanish names:
Indigenous names: Yàg-làa[23]
Used by (1*): Zapotec[23]
Used for (1[#]): Skin (1)[23]
Cognates:
Language contact:

***Mentzelia hispida* Willd. (Loasaceae)**

Spanish names:
Indigenous names: Thekw'em ch'ohool. Itsaan yakw'ab wahuts[07]; Guìzh-nàad, guièe-nàad[23]
Used by (2*): Huastec[07]; Zapotec[23]
Used for (3[#]): Psychological (1)[07]; Skin (1)[07]; Pregnancy (1)[23]
Cognates:
Language contact:

***Merremia dissecta* (Jacq.) Hallier f. (Convolvulaceae)**

Spanish names:
Indigenous names: Piith ts'aah[07]; Is k'aham[17]
Used by (2*): Huastec[07]; Quichean Maya[17]
Used for (3[#]): Skin (1)[07]; General and Unspecified (1)[07]; nd[17]
Cognates:
Language contact: Hua <> Kekchí

***Merremia tuberosa* (L.) Rendle (Convolvulaceae)**

Spanish names:
Indigenous names: Chuthat po'eel, man ch'aah[07]
Used by (2*): Huastec[07]; Quichean Maya[17]
Used for (2[#]): Neurological (1)[07]; nd[17]
Cognates:
Language contact:

***Merremia umbellata* (L.) Hallier f. (Convolvulaceae)**

Spanish names:
Indigenous names: San Diego ts'ohool, chuthat pok'eel[07]
Used by (1*): Huastec[07]
Used for (3[#]): Ear (1)[07]; Skin (1)[07]; General and Unspecified (1)[07]
Cognates:
Language contact:

***Metastelma schlechtendalii* Decne. (Apocynaceae)**

Spanish names:
Indigenous names: Xîum-ak'[09]
Used by (2*): Zoque[03]; Yucatecan Maya[09]
Used for (3[#]): Skin (1)[03]; Endocrine (1)[09]; Urological (1)[09]
Cognates:
Language contact:

***Miconia albicans*** **(Sw.) Steud. (Melastomataceae)**

Spanish names:
Indigenous names: Pak tesua[03]; Jama piits[04]
Used by (2*): Zoque[03]; Mixe[04]
Used for (6#): Digestive (1)[03]; Respiratory (1)[03]; Skin (2)[03, 04]; Urological (1)[03]; Female genital (1)[03]
Cognates:
Language contact:

***Miconia argentea*** **(Sw.) DC. (Melastomataceae)**

Spanish names: Tescuate
Indigenous names: Tesua[03]
Used by (1*): Zoque[03]
Used for (5#): Digestive (1)[03]; Respiratory (1)[03]; Urological (1)[03]; Pregnancy (1)[03]; Female genital (1)[03]
Cognates:
Language contact:

***Miconia calvescens*** **DC. (Melastomataceae)**

Spanish names:
Indigenous names: Xoy Q'ehen, Kaq'i pim[14]
Used by (1*): Quichean Maya[14]
Used for (3#): Neurological (1)[14]; Female genital (1)[14]; General and Unspecified (1)[14]
Cognates:
Language contact:

***Miconia glaberrima*** **(Schltdl.) Naudin (Melastomataceae)**

Spanish names:
Indigenous names:
Used by (1*): Zoque[03]
Used for (1#): Digestive (1)[03]
Cognates:
Language contact:

***Miconia ibaguensis*** **(Bonpl.) Triana (Melastomataceae)**

Spanish names:
Indigenous names: Chuk jeepe[03]
Used by (1*): Zoque[03]
Used for (1#): Digestive (1)[03]
Cognates:
Language contact:

***Miconia mexicana*** **(Bonpl.) Naudin (Melastomataceae)**

Spanish names: Cinco negritos del monte
Indigenous names:
Used by (1*): Zoque[01]
Used for (1#): Digestive (1)[01]
Cognates:
Language contact:

***Miconia minutiflora*** **(Bonpl.) DC. (Melastomataceae)**

Spanish names: Palo dulce
Indigenous names: Sa'ksi qui'hui[06]
Used by (1*): Totonac[06]
Used for (1#): Skin (1)[06]
Cognates:
Language contact:

***Miconia oinochrophylla* Donn. Sm. (Melastomataceae)**

Spanish names:
Indigenous names: Ixq'i Q'ehen [14]
Used by (2*): Quichean Maya[14, 17]
Used for (5#): Neurological (1)[14]; Pregnancy (1)[14]; Female genital (1)[14]; General and Unspecified (1)[14]; nd[17]
Cognates:
Language contact:

***Miconia schlechtendalii* Cogn. (Melastomataceae)**

Spanish names:
Indigenous names:
Used by (1*): Zoque[03]
Used for (2#): Digestive (1)[03]; Skin (1)[03]
Cognates:
Language contact:

***Miconia* sp. (Melastomataceae)**

Spanish names:
Indigenous names: Kux Sawi'i[14]; Kux Sawi'i, Tzib' Q'en[14]; Wa'bon'[14]
Used by (1*): Quichean Maya[14]
Used for (5#): Digestive (1)[14]; Neurological (1)[14]; Skin (1)[14]; Female genital (1)[14]; General and Unspecified (1)[14]
Cognates:
Language contact:

***Microgramma nitida* (J. Sm.) A.R. Sm. (Polypodiaceae)**

Spanish names:
Indigenous names: Canagual[03]; Chu'ub te', chu'ub ts'aah[07]; Tipte'-ak'[09]
Used by (3*): Zoque[03]; Huastec[07]; Yucatecan Maya[09]
Used for (4#): Digestive (1)[09]; Musculoskeletal (2)[03, 07]; General and Unspecified (1)[07]
Cognates:
Language contact:

***Micromeria douglasii* Benth. (Lamiaceae)**

Spanish names: Hierba buena
Indigenous names: Bìt-wàd, bìt-xtîl[23]
Used by (1*): Zapotec[23]
Used for (1#): Digestive (1)[23]
Cognates:
Language contact:

***Micropleura renifolia* Lag. (Apiaceae)**

Spanish names: Valeriana
Indigenous names: Valeriana[13]; Nich nab momol, nich nab tz'i' lel vomol, vomolal vo', makmak nab wamal, , bokol chuch wamal, bikil ch'o wamal[20]
Used by (2*): Quichean Maya[13]; Western Maya[20]
Used for (5#): Digestive (1)[20]; Neurological (2)[13, 20]; Pregnancy (1)[20]; Female genital (1)[20]
Cognates:
Language contact:

***Microsechium palmatum* (Ser.) Cogn. (Cucurbitaceae)**

Spanish names: Amole, tumba vaquero
Indigenous names: Bià-tòo, Ibæ̀-bià-tòo[23]
Used by (1*): Zapotec[23]
Used for (4#): Neurological (1)[23]; Psychological (1)[23]; Skin (1)[23]; General and Unspecified (1)[23]
Cognates:
Language contact:

### *Microsechium* sp. (Cucurbitaceae)

Spanish names: Yerba de raton, oreja de raton
Indigenous names: Škwam bizin[22]
Used by (1*): Zapotec[22]
Used for (1#): Skin (1)[22]
Cognates:
Language contact:

### *Mikania cordifolia* (L.f.) Willd. (Asteraceae)

Spanish names: Hoja de carga
Indigenous names: Tzämi tzoy tzämi ay[01]; Wako[07]
Used by (2*): Zoque[01]; Huastec[07]
Used for (3#): Digestive (1)[07]; Skin (1)[07]; General and Unspecified (1)[01]
Cognates:
Language contact:

### *Mikania micrantha* Kunth (Asteraceae)

Spanish names: Tabardillo
Indigenous names: Cha'onoob'[17]
Used by (3*): Zoque[03]; Quichean Maya[17]; Western Maya[19]
Used for (4#): Neurological (1)[19]; Female genital (1)[03]; General and Unspecified (1)[19]; nd[17]
Cognates:
Language contact:

### *Milla biflora* Cav. (Asparagaceae)

Spanish names: Lirio
Indigenous names:
Used by (1*): Quichean Maya[12]
Used for (2#): Neurological (1)[12]; Urological (1)[12]
Cognates:
Language contact:

### *Milleria quinqueflora* L. (Asteraceae)

Spanish names:
Indigenous names: Xø pøh[04]; Xontolok[09]
Used by (2*): Mixe[04]; Yucatecan Maya[09]
Used for (2#): Skin (2)[04, 09]
Cognates:
Language contact:

### *Mimosa aculeaticarpa* Ortega (Fabaceae)

Spanish names: Uña de gato
Indigenous names: Yàg-guièts-nàad[23]
Used by (1*): Zapotec[23]
Used for (1#): General and Unspecified (1)[23]
Cognates:
Language contact:

***Mimosa albida* Willd. (Fabaceae)**

Spanish names: Dormilona, vergonzosa, uña de gato, zarza
Indigenous names: Pik awit/ anhgen yäki/ äksy awit/ awit jäyä[01]; Nuk cuy[03]; Tančahuat[05]; Chobeem[07]; Xu'l Xpe Quej, Q'ix[12]; Xul q'ies, xul kiej, Cxal kij[13]; Guièts-làa-tó, guìzh-lòo-tó], guièts-guièe-tó[23]; Xohuoctihuistle[26]; Pinahuitz[28]
Used by (10*): Zoque[01, 03]; Totonac[05]; Huastec[07]; Quichean Maya[12, 13]; Zapotec[21, 23]; Nahua[26, 28]
Used for (30[#]): Digestive (5)[03, 05, 12, 23, 28]; Eye (1)[03]; Musculoskeletal (2)[01, 12]; Neurological (1)[12]; Psychological (3)[01, 12, 21]; Respiratory (1)[26]; Skin (6)[01, 03, 07, 12, 13, 28]; Endocrine (2)[01, 28]; Urological (1)[12]; Pregnancy (2)[12, 26]; Female genital (2)[03, 13]; General and Unspecified (4)[01, 12, 21, 28]
Cognates: Quich: q'ix/kij; Nahua: huistl;
Language contact: Nah <> Zap

***Mimosa bahamensis* Benth. (Fabaceae)**

Spanish names:
Indigenous names: Sak-katsim, Katsim[09]
Used by (1*): Yucatecan Maya[09]
Used for (1[#]): Respiratory (1)[09]
Cognates:
Language contact:

***Mimosa hondurana* Britton & Rose (Fabaceae)**

Spanish names: Uña de gato
Indigenous names: Misyu kä[01]
Used by (1*): Zoque[01]
Used for (2[#]): Digestive (1)[01]; Musculoskeletal (1)[01]
Cognates:
Language contact:

***Mimosa luisana* Brandegee (Fabaceae)**

Spanish names: Uña de gato
Indigenous names:
Used by (1*): Nahua[27]
Used for (1[#]): Endocrine (1)[27]
Cognates:
Language contact:

***Mimosa pigra* L. (Fabaceae)**

Spanish names:
Indigenous names: Tsobeem, wayma'[07]
Used by (1*): Huastec[07]
Used for (2[#]): Psychological (1)[07]; Pregnancy (1)[07]
Cognates:
Language contact:

***Mimosa polydactyla* Willd. (Fabaceae)**

Spanish names: Dormilona
Indigenous names: Ä' yäwi/ä' wewo tane/ok yäwi [01]
Used by (1*): Zoque[01]
Used for (3[#]): Psychological (1)[01]; Urological (1)[01]; General and Unspecified (1)[01]
Cognates:
Language contact:

[01-28]refer to the study codes in Table 4.1. *Total number of studies citing this taxon [#]Total number of use-records

***Mimosa pudica* L. (Fabaceae)**

Spanish names: Dormilona, vergonzosa
Indigenous names: Mo'ay sake ma'syi/ tzasyäkuy ay[02]; Mong mong ay[03]; Ma'ap ujts[04]; Šmu¢'ic[10]; Ix mutz[11]; Wara Q'ix[14]; Quare kix[16]; Ajwäye[18]; Guedzegumaj'alaj[21]
Used by (11*): Zoque[02, 03]; Mixe[04]; Yucatecan Maya[10, 11]; Quichean Maya[14, 15, 16]; Western Maya[18]; Zapotec[21]; Nahua[26]
Used for (28#): Digestive (3)[03, 21, 26]; Neurological (2)[02, 14]; Psychological (8)[02, 03, 04, 10, 14, 15, 16, 18]; Respiratory (3)[03, 18, 26]; Skin (2)[02, 21]; Urological (1)[21]; Pregnancy (2)[15, 26]; Female genital (1)[03]; General and Unspecified (6)[02, 03, 11, 14, 18, 21]
Cognates: MZ: mo'a/ma'a; Zoq: mo(ng) ay; Yuca: xmutz; Quich: arV kix;
Language contact: MZ > Zap

***Mimosa* sp. (Fabaceae)**

Spanish names: Tepehuaje cimarron
Indigenous names:
Used by (1*): Zapotec[21]
Used for (2#): Skin (1)[21]; Urological (1)[21]
Cognates:
Language contact:

***Mimosa tenuiflora* (Willd.) Poir. (Fabaceae)**

Spanish names: Tepesquehuite; Tepezcohuite; Tepezcohuite/Pezquehuite
Indigenous names: Gueedxe boog[21]
Used by (3*): Zoque[01, 02]; Zapotec[21]
Used for (7#): Digestive (2)[01, 21]; Skin (3)[01, 02, 21]; Urological (1)[21]; Pregnancy (1)[02]
Cognates:
Language contact:

***Mimosa velloziana* Mart. (Fabaceae)**

Spanish names: Zarza
Indigenous names: Jupupi[02]
Used by (2*): Zoque[01, 02]
Used for (3#): Digestive (1)[01]; Skin (1)[02]; General and Unspecified (1)[02]
Cognates:
Language contact:

***Mimulus glabratus* Kunth (Phrymaceae)**

Spanish names: Verdolaga
Indigenous names:
Used by (1*): Quichean Maya[13]
Used for (1#): General and Unspecified (1)[13]
Cognates:
Language contact:

***Mirabilis jalapa* L. (Nyctaginaceae)**

Spanish names: Maravilla, flor de linda tarde
Indigenous names: Tzuj jäyä[01]; Txuang mooya[03]; tsu pøh[04]; Ch'uyeem[07]; Guièe-màràvî, guièe-mòròvî[23]
Used by (11*): Zoque[01, 02, 03]; Mixe[04]; Huastec[07]; Yucatecan Maya[10, 11]; Quichean Maya[13]; Western Maya[18]; Zapotec[21, 23]
Used for (30#): Digestive (3)[02, 07, 23]; Eye (1)[02]; Musculoskeletal (4)[01, 02, 11, 21]; Neurological (2)[02, 18]; Psychological (3)[01, 03, 13]; Respiratory (4)[01, 02, 07, 21]; Skin (6)[01, 02, 04, 10, 18, 21]; Female genital (1)[02]; General and Unspecified (6)[01, 03, 07, 11, 18, 21]
Cognates: MZ: tsu; Zoq: tsu/txu;
Language contact: MZ > Hua

***Mirabilis longiflora* L. (Nyctaginaceae)**

Spanish names:
Indigenous names: Ch'uyeem[07]
Used by (1*): Huastec[07]
Used for (1#): General and Unspecified (1)[07]
Cognates:
Language contact:

***Mollinedia* sp. (Monimiaceae)**

Spanish names:
Indigenous names: Sakim pim[16]
Used by (1*): Quichean Maya[16]
Used for (1#): Neurological (1)[16]
Cognates:
Language contact:

***Mollinedia viridiflora* Tul. (Monimiaceae)**

Spanish names:
Indigenous names: Sak'i k'ejen[17]
Used by (1*): Quichean Maya[17]
Used for (1#): nd[17]
Cognates:
Language contact:

***Mollugo verticillata* L. (Molluginaceae)**

Spanish names:
Indigenous names: Billushít[22]
Used by (1*): Zapotec[22]
Used for (1#): Digestive (1)[22]
Cognates:
Language contact:

***Momordica charantia* L. (Cucurbitaceae)**

Spanish names: Cunduamor
Indigenous names: Paks ko'kinä[02]; Kundiamor[03]; Pa xandia[04]; Morax[09]; Sandiy'cho[14]; Ya'mor[17]; Kwnyamor[18]
Used by (12*): Zoque[01, 02, 03]; Mixe[04]; Yucatecan Maya[09, 10, 11]; Quichean Maya[14, 15, 17]; Western Maya[18]; Zapotec[21]
Used for (33#): Blood (1)[10]; Digestive (2)[02, 11]; Musculoskeletal (3)[02, 03, 14]; Neurological (1)[04]; Respiratory (2)[02, 04]; Skin (5)[02, 03, 09, 18, 21]; Endocrine (8)[01, 02, 03, 10, 11, 14, 18, 21]; Urological (2)[03, 11]; Pregnancy (1)[03]; Female genital (2)[02, 15]; Male genital (1)[18]; General and Unspecified (4)[02, 04, 11, 14]; nd[17]
Cognates:
Language contact:

***Monnina* sp. (Polygalaceae)**

Spanish names: San Benito, tintiamora
Indigenous names: Cha xu q'an[13]
Used by (1*): Quichean Maya[13]
Used for (1#): Eye (1)[13]
Cognates:
Language contact:

***Monochaetum deppeanum* (Schltdl. & Cham.) Naudin (Melastomataceae)**

Spanish names:
Indigenous names: Cacaloxihuitle[26]
Used by (1*): Nahua[26]
Used for (3#): Digestive (1)[26]; Respiratory (1)[26]; Female genital (1)[26]
Cognates:
Language contact:

***Monochaetum* sp. (Melastomataceae)**

Spanish names:
Indigenous names:
Used by (1*): Zoque[03]
Used for (2#): Skin (1)[03]; General and Unspecified (1)[03]
Cognates:
Language contact:

***Monstera acuminata* K. Koch (Araceae)**

Spanish names:
Indigenous names: Xunujti tsay[03]
Used by (1*): Zoque[03]
Used for (1#): Skin (1)[03]
Cognates:
Language contact:

***Monstera deliciosa* Liebm. (Araceae)**

Spanish names: Tripa de pollo
Indigenous names: Chibaba[21]
Used by (1*): Zapotec[21]
Used for (1#): General and Unspecified (1)[21]
Cognates:
Language contact:

***Monstera tuberculata* Lundell (Araceae)**

Spanish names: Hoja de corazón
Indigenous names: Anma ay[03]
Used by (1*): Zoque[03]
Used for (2#): Digestive (1)[03]; Skin (1)[03]
Cognates:
Language contact:

***Montanoa atriplicifolia* (Pers.) Sch.Bip. (Asteraceae)**

Spanish names:
Indigenous names: Xtankas- ak', Xuxtankas[09]
Used by (1*): Yucatecan Maya[09]
Used for (1#): General and Unspecified (1)[09]
Cognates:
Language contact:

***Montanoa grandiflora* (DC.) Sch.Bip. ex Hemsl. (Asteraceae)**

Spanish names: Flor de papera, flor de teresita; Penumbre
Indigenous names:
Used by (3*): Zoque[01, 03]; Zapotec[21]
Used for (6#): Digestive (2)[01, 21]; Musculoskeletal (1)[01]; Skin (2)[03, 21]; General and Unspecified (1)[21]
Cognates:
Language contact:

***Montanoa* sp. (Asteraceae)**

Spanish names:
Indigenous names: Yag tsun[22]
Used by (1*): Zapotec[22]
Used for (2#): Digestive (1)[22]; General and Unspecified (1)[22]
Cognates:
Language contact:

***Montanoa tomentosa* Cerv. (Asteraceae)**

Spanish names: Acahuite; Chamizo negro
Indigenous names: Yàg-yàaz-yâas, yàg-yàaz-ngǎs[23]
Used by (2*): Zapotec[23]; Nahua[27]
Used for (4#): Digestive (1)[23]; Skin (1)[23]; Pregnancy (1)[23]; General and Unspecified (1)[27]
Cognates:
Language contact:

***Morella cerifera* (L.) Small (Myricaceae)**

Spanish names: Arrayan
Indigenous names: Ot kamay/ jotomgay kamay [01]; Copa puso[03]; Pom che', uch'mix Tiox[13]; Sera te'[20]; Ahuaxochitl[25]; Xochicuahuitl[26]
Used by (6*): Zoque[01, 03]; Quichean Maya[13]; Western Maya[20]; Nahua[25, 26]
Used for (18#): Digestive (3)[01, 13, 20]; Cardiovascular (1)[25]; Musculoskeletal (2)[01, 13]; Neurological (1)[13]; Psychological (1)[01]; Respiratory (1)[03]; Pregnancy (2)[01, 13]; Female genital (2)[03, 13]; General and Unspecified (5)[01, 03, 13, 25, 26]
Cognates: Nahua: xochitl;
Language contact:

***Morinda citrifolia* L. (Rubiaceae)**

Spanish names: Noni; Noni/cancero; Nonis
Indigenous names: K'an i che'[17]
Used by (5*): Zoque[01, 02]; Quichean Maya[12, 14, 17]
Used for (11#): Digestive (1)[02]; Cardiovascular (1)[02]; Endocrine (2)[02, 14]; Urological (2)[01, 02]; Pregnancy (1)[01]; General and Unspecified (3)[01, 02, 12]; nd[17]
Cognates:
Language contact:

***Morinda royoc* L. (Rubiaceae)**

Spanish names: Piñita
Indigenous names: Piña ak', Piña kam[09]
Used by (2*): Yucatecan Maya[09, 11]
Used for (2#): Cardiovascular (1)[11]; Skin (1)[09]
Cognates:
Language contact:

***Moringa oleifera* Lam. (Moringaceae)**

Spanish names: Jacinto; Moringa; San Jacinto
Indigenous names:
Used by (4*): Zoque[02]; Quichean Maya[12, 14]; Zapotec[21]
Used for (8#): Digestive (1)[12]; Musculoskeletal (2)[02, 21]; Respiratory (1)[02]; Endocrine (2)[12, 14]; General and Unspecified (2)[12, 21]
Cognates:
Language contact:

***Mortoniodendron guatemalense*** **Standl. & Steyerm. (Malvaceae)**

Spanish names:
Indigenous names: Kaq'i B'ach[14]
Used by (1*): Quichean Maya[14]
Used for (2#): Pregnancy (1)[14]; Female genital (1)[14]
Cognates:
Language contact:

***Mosannona depressa*** **(Baill.) Chatrou (Annonaceae)**

Spanish names: Lemoy
Indigenous names: Elemuy[09]
Used by (2*): Yucatecan Maya[09, 11]
Used for (3#): Endocrine (1)[09]; Urological (1)[09]; General and Unspecified (1)[11]
Cognates:
Language contact:

***Mosquitoxylum jamaicense*** **Krug et Urb. (Anacardiaceae)**

Spanish names: Cedro nogal
Indigenous names: Se’mpe[03]
Used by (1*): Zoque[03]
Used for (2#): Female genital (1)[03]; General and Unspecified (1)[03]
Cognates:
Language contact:

***Mouriri*** **sp. (Melastomataceae)**

Spanish names:
Indigenous names: Chak B'olay Q'ehen[14]
Used by (1*): Quichean Maya[14]
Used for (2#): Digestive (1)[14]; Skin (1)[14]
Cognates:
Language contact:

***Moussonia deppeana*** **(Schltdl. & Cham.) Klotzsch ex Hanst. (Gesneriaceae)**

Spanish names: Cerbatanera; Hierba de zopilote
Indigenous names: Ajpu Q'ehen[14]; Cacahuatón[26]
Used by (2*): Quichean Maya[14]; Nahua[26]
Used for (5#): Digestive (1)[26]; Respiratory (1)[26]; Endocrine (1)[26]; Female genital (1)[26]; General and Unspecified (1)[14]
Cognates:
Language contact:

***Mucuna argyrophylla*** **Standl. (Fabaceae)**

Spanish names:
Indigenous names: Mooxoox[07]
Used by (1*): Huastec[07]
Used for (5#): Digestive (1)[07]; Neurological (1)[07]; Respiratory (1)[07]; Skin (1)[07]; General and Unspecified (1)[07]
Cognates:
Language contact:

***Mucuna pruriens*** **(L.) DC. (Fabaceae)**

Spanish names: Picapica, nescafé
Indigenous names: Kunkun[02]; Neskaape, tsanakw' thut'[07]; Xpica[09]
Used by (7*): Zoque[01, 02, 03]; Huastec[07]; Yucatecan Maya[09]; Quichean Maya[13]; Zapotec[21]
Used for (7#): Digestive (1)[09]; Eye (1)[13]; Cardiovascular (1)[01]; Neurological (1)[02]; Psychological (1)[03]; General and Unspecified (2)[07, 21]
Cognates:
Language contact:

[01-28]refer to the study codes in Table 4.1. *Total number of studies citing this taxon #Total number of use-records

***Muehlenbeckia platyclados* (F.Muell.) Meisn. (Polygonaceae)**

Spanish names: Cola de cabello
Indigenous names:
Used by (1*): Nahua[26]
Used for (2#): Musculoskeletal (1)[26]; Urological (1)[26]
Cognates:
Language contact:

***Muntingia calabura* L. (Muntingiaceae)**

Spanish names: Capulín
Indigenous names: Mukpe/juj kuy[01]; Mupe[02]; Capuli[03]; Mujt[04]; Tsakam puwaamte', puam[07]; Pujam[18]; Mbe'e ze'e[21]
Used by (7*): Zoque[01, 02, 03]; Mixe[04]; Huastec[07]; Western Maya[18]; Zapotec[21]
Used for (19#): Digestive (4)[02, 03, 18, 21]; Musculoskeletal (1)[02]; Respiratory (3)[01, 02, 21]; Skin (4)[03, 04, 18, 21]; Urological (1)[03]; Pregnancy (2)[01, 07]; Female genital (1)[03]; General and Unspecified (3)[02, 04, 21]
Cognates: MZ: muK; Zoq: mupe;
Language contact: MZ <> Maya

***Murraya paniculata* (L.) Jack (Rutaceae)**

Spanish names: Limonaria; Muraya
Indigenous names:
Used by (2*): Zoque[03]; Yucatecan Maya[09]
Used for (2#): Neurological (1)[03]; Respiratory (1)[09]
Cognates:
Language contact:

***Musa × paradisiaca* L. (Musaceae)**

Spanish names: Plátano, guineo
Indigenous names: Tsapuo [01]; Patanus/patanusy/kineya[02]; Ka'ak[04]; See'kna [06]; It'ath[07]; Costillon it'adh[08]; Inkiney, skul[13]; Xcol'itul, Ik B'olay Q'ehen [14]; Ja'as, Ajruatan[18]; Kene'[19]; Biduáj[21]
Used by (12*): Zoque[01, 02]; Mixe[04]; Totonac[06]; Huastec[07, 08]; Quichean Maya[12, 13, 14]; Western Maya[18, 19]; Zapotec[21]
Used for (32#): Digestive (10)[01, 02, 04, 06, 07, 13, 14, 18, 19, 21]; Psychological (2)[01, 18]; Respiratory (2)[06, 21]; Skin (7)[01, 02, 07, 13, 14, 18, 21]; Endocrine (1)[18]; Urological (1)[06]; Female genital (2)[01, 21]; Male genital (1)[01]; General and Unspecified (6)[01, 02, 08, 12, 14, 21]
Cognates:
Language contact:

***Musa acuminata* Colla (Musaceae)**

Spanish names: Platano cherimoya, platano pera, platano bolsa
Indigenous names: See'kna, tet'see'kann[06]
Used by (1*): Totonac[06]
Used for (2#): Digestive (1)[06]; Skin (1)[06]
Cognates:
Language contact:

***Musa* sp. (Musaceae)**

Spanish names: Platano; Platano rojo
Indigenous names: Hoko samñi[03]; Ha'as[09]
Used by (3*): Zoque[03]; Yucatecan Maya[09]; Nahua[24]
Used for (4#): Digestive (2)[03, 09]; Skin (1)[03]; Pregnancy (1)[24]
Cognates:
Language contact:

***Myrcianthes fragrans* (Sw.) Mc Vaugh (Myrtaceae)**

Spanish names:
Indigenous names: Cheks [03]
Used by (1*): Zoque[03]
Used for (1#): Digestive (1)[03]
Cognates:
Language contact:

***Myriocarpa heterospicata* Donn.Sm. (Urticaceae)**

Spanish names: Pegasoso/palo de barba/caracolillo/ la barbosa
Indigenous names: Tzoki 'an panatz/tzok yan panats[01]
Used by (1*): Zoque[01]
Used for (1#): Skin (1)[01]
Cognates:
Language contact:

***Myriocarpa longipes* Liebm. (Urticaceae)**

Spanish names: Chalagoge, panza de burro
Indigenous names: (Mo'a) siskuy[01]; Nä ninki[02]
Used by (2*): Zoque[01, 02]
Used for (4#): Digestive (1)[01]; Musculoskeletal (1)[02]; Urological (1)[01]; General and Unspecified (1)[01]
Cognates:
Language contact:

***Myristica fragrans* Houtt. (Myristicaceae)**

Spanish names: Nuez moscada; Nuez mozcada
Indigenous names:
Used by (3*): Zoque[01, 02, 03]
Used for (14#): Digestive (3)[01, 02, 03]; Musculoskeletal (1)[02]; Psychological (2)[01, 03]; Respiratory (1)[01]; Skin (2)[02, 03]; Pregnancy (2)[01, 03]; Female genital (1)[03]; General and Unspecified (2)[01, 03]
Cognates:
Language contact:

***Myroxylon balsamum* (L.) Harms (Fabaceae)**

Spanish names: Arbol de balsa; Balsamo de Peru; Bálsamo negro
Indigenous names: Balsa cuy[03]
Used by (3*): Zoque[01, 03]; Mixe[04]
Used for (6#): Cardiovascular (1)[03]; Musculoskeletal (2)[03, 04]; Skin (1)[03]; General and Unspecified (2)[01, 04]
Cognates:
Language contact:

***Myrrhis odorata* (L.) Scop. (Apiaceae)**

Spanish names: Mirrha
Indigenous names:
Used by (1*): Quichean Maya[12]
Used for (3#): Digestive (1)[12]; Respiratory (1)[12]; General and Unspecified (1)[12]
Cognates:
Language contact:

***Myrtus communis* L. (Myrtaceae)**

Spanish names: Mirto
Indigenous names: Uxum ts'ojol[08]
Used by (1*): Huastec[08]
Used for (4#): Digestive (1)[08]; Ear (1)[08]; Neurological (1)[08]; Skin (1)[08]
Cognates:
Language contact:

[01-28]refer to the study codes in Table 4.1. *Total number of studies citing this taxon #Total number of use-records

***Nasturtium officinale* R.Br. (Brassicaceae)**

Spanish names: Berro
Indigenous names: Uwi suk', chaj patre[13]
Used by (4*): Zoque[03]; Totonac[06]; Quichean Maya[13]; Zapotec[21]
Used for (11#): Blood (2)[06, 13]; Digestive (2)[06, 21]; Eye (1)[13]; Respiratory (2)[06, 13]; Skin (1)[03]; General and Unspecified (3)[06, 13, 21]
Cognates:
Language contact:

***Nectandra lineata* (Kunth) Rohwer (Lauraceae)**

Spanish names:
Indigenous names: Chajom Che'[14]
Used by (1*): Quichean Maya[14]
Used for (1#): Digestive (1)[14]
Cognates:
Language contact:

***Nectandra salicifolia* (Kunth) Nees (Lauraceae)**

Spanish names: Laurel
Indigenous names: Ohte'[07]
Used by (2*): Huastec[07]; Nahua[26]
Used for (3#): Digestive (1)[07]; Psychological (1)[26]; Skin (1)[07]
Cognates:
Language contact:

***Nectandra* sp. (Lauraceae)**

Spanish names:
Indigenous names: Ahuacachile[24]
Used by (1*): Nahua[24]
Used for (1#): Pregnancy (1)[24]
Cognates:
Language contact:

***Neea psychotrioides* Donn.Sm. (Nyctaginaceae)**

Spanish names:
Indigenous names: Tsabats masan ay[03]; K'ak'al xeklek, k'ak'al ilaal[07]; Xtatsim[09]
Used by (3*): Zoque[03]; Huastec[07]; Yucatecan Maya[09]
Used for (4#): Skin (2)[03, 09]; General and Unspecified (2)[03, 07]
Cognates:
Language contact:

***Neomillspaughia emarginata* (H. Gross) S.F. Blake (Polygonaceae)**

Spanish names:
Indigenous names: Sakitsa', Xtastabin[09]
Used by (1*): Yucatecan Maya[09]
Used for (2#): Respiratory (1)[09]; Skin (1)[09]
Cognates:
Language contact:

***Nephrolepis biserrata* (Sw.) Schott (Nephrolepidaceae)**

Spanish names:
Indigenous names: Xqu'uq mo'coch[16]; Y qu'q moco'ch[17]
Used by (2*): Quichean Maya[16, 17]
Used for (2#): Neurological (1)[16]; nd[17]
Cognates:
Language contact:

***Nephrolepis cordifolia*** **(L.) C. Presl (Nephrolepidaceae)**

Spanish names: Cola de Quetzal; Huechamacho
Indigenous names: Rix Ixul, Ch'upil Q'en[14]
Used by (2*): Quichean Maya[14]; Nahua[26]
Used for (4#): Digestive (1)[26]; Psychological (1)[14]; Skin (1)[14]; General and Unspecified (1)[14]
Cognates:
Language contact:

***Nephrolepis undulata*** **(Afzel. ex Sw.) J. Sm. (Nephrolepidaceae)**

Spanish names: Calahuala del Quetzal
Indigenous names: Kalaguala Ruje'y Maq'uq'[12]
Used by (1*): Quichean Maya[12]
Used for (2#): Digestive (1)[12]; General and Unspecified (1)[12]
Cognates:
Language contact:

***Nerium oleander*** **L. (Apocynaceae)**

Spanish names: Clabel; Laurel
Indigenous names: Pitx mooya[03]
Used by (3*): Zoque[03]; Zapotec[21]; Nahua[28]
Used for (7#): Musculoskeletal (1)[03]; Neurological (1)[03]; Respiratory (2)[21, 28]; Skin (1)[21]; Female genital (1)[21]; General and Unspecified (1)[28]
Cognates:
Language contact:

***Neurolaena lobata*** **(L.) R.Br. ex Cass. (Asteraceae)**

Spanish names: Tres puntas, mano de lagarto
Indigenous names: T'unu' ix bek'em[07]; Kayabim[10]; K'aman, c'a mank[13]; K'a' Mank'[14]; Kamank, gan mank[15]; K'a mank[17]; Ch'ajch'aj k'opot[19]
Used by (11*): Zoque[02, 03]; Huastec[07]; Yucatecan Maya[10, 11]; Quichean Maya[13, 14, 15, 17]; Western Maya[19]; Nahua[26]
Used for (19#): Digestive (2)[03, 14]; Musculoskeletal (1)[14]; Neurological (1)[19]; Skin (6)[02, 03, 10, 11, 14, 26]; Endocrine (1)[03]; Pregnancy (1)[07]; Female genital (1)[15]; General and Unspecified (5)[03, 11, 13, 14, 19]; nd[17]
Cognates: CoreM: kaCa; Quich: kamank;
Language contact:

***Nicandra physalodes*** **(L.) Gaertn. (Solanaceae)**

Spanish names: Tomate de Ratón
Indigenous names:
Used by (1*): Quichean Maya[12]
Used for (1#): Skin (1)[12]
Cognates:
Language contact:

***Nicotiana glauca*** **Graham (Solanaceae)**

Spanish names: Tabaco
Indigenous names: Mostas[22]; Yàg-brètâyn[23]; Nenestlichihuitl[28]
Used by (3*): Zapotec[22, 23]; Nahua[28]
Used for (10#): Ear (1)[23]; Musculoskeletal (1)[28]; Neurological (3)[22, 23, 28]; Skin (2)[22, 28]; General and Unspecified (3)[22, 23, 28]
Cognates:
Language contact:

***Nicotiana plumbaginifolia* Viv. (Solanaceae)**

Spanish names: Tabaquillo
Indigenous names: Guiass yeen[21]
Used by (1*): Zapotec[21]
Used for (4#): Musculoskeletal (1)[21]; Neurological (1)[21]; Psychological (1)[21]; General and Unspecified (1)[21]
Cognates:
Language contact:

***Nicotiana* sp. (Solanaceae)**

Spanish names: Tabaco
Indigenous names: Ts¥ui[03]
Used by (1*): Zoque[03]
Used for (2#): Ear (1)[03]; Skin (1)[03]
Cognates:
Language contact:

***Nicotiana tabacum* L. (Solanaceae)**

Spanish names: Tabaco
Indigenous names: (Tzuj/ tzushy) ozi[01]; Tzä'wi[02]; Juiky[04]; Maay[07]; K'uts[09]; K'uuČ[10]; Met', mee'[13]; Mai, Xa'q Mai[14]; C'utz[18]; K'ujtz[19]; Moy, may, bankilal[20]; Giasa'a[21]; Blàg-guièz, blàg-tàbâcw[23]
Used by (15*): Zoque[01, 02]; Mixe[04]; Huastec[07]; Yucatecan Maya[09, 10, 11]; Quichean Maya[13, 14]; Western Maya[18, 19, 20]; Zapotec[21, 23]; Nahua[26]
Used for (50#): Digestive (2)[01, 20]; Eye (1)[02]; Ear (2)[01, 02]; Cardiovascular (2)[07, 13]; Musculoskeletal (6)[01, 14, 18, 19, 21, 23]; Neurological (4)[02, 11, 18, 19]; Psychological (4)[01, 19, 20, 21]; Respiratory (8)[01, 02, 10, 11, 13, 20, 21, 23]; Skin (11)[01, 02, 04, 07, 09, 11, 13, 14, 18, 21, 26]; Pregnancy (2)[01, 13]; General and Unspecified (8)[01, 07, 13, 14, 14, 19, 20, 21]
Cognates: Mayan: k'uts/mai; Yuca: k'uts; Quich: mee/mai; WesM: k'uts; Zapo: gias/guiez;
Language contact: Zap <> Yuc and WesM

***Niphidium crassifolium* (L.) Lellinger (Polypodiaceae)**

Spanish names: Oreja de burro
Indigenous names: Burru tatzo [01]
Used by (1*): Zoque[01]
Used for (1#): Digestive (1)[01]
Cognates:
Language contact:

***Nissolia fruticosa* Jacq. (Fabaceae)**

Spanish names:
Indigenous names: Bo' xekel[07]; Xk'ant'uul[09]
Used by (2*): Huastec[07]; Yucatecan Maya[09]
Used for (4#): Neurological (1)[07]; Skin (2)[07, 09]; General and Unspecified (1)[07]
Cognates:
Language contact:

***Nopalea auberi* (Pfeiff.) Salm-Dyck (Cactaceae)**

Spanish names:
Indigenous names: Yàg-biǎa[23]
Used by (1*): Zapotec[23]
Used for (2#): Digestive (1)[23]; Skin (1)[23]
Cognates:
Language contact:

[01-28]refer to the study codes in Table 4.1. *Total number of studies citing this taxon #Total number of use-records

***Nopalea cochenillifera*** **(L.) Salm-Dyck (Cactaceae)**

Spanish names: Nopal
Indigenous names: Pak'ak[07]; Pak'ak[08]; Pak'am[09]
Used by (6*): Huastec[07, 08]; Yucatecan Maya[09]; Quichean Maya[12, 14]; Nahua[24]
Used for (18#): Digestive (3)[07, 12, 14]; Cardiovascular (1)[08]; Musculoskeletal (1)[08]; Respiratory (1)[09]; Skin (3)[07, 08, 14]; Endocrine (2)[08, 12]; Urological (2)[09, 12]; Pregnancy (2)[07, 24]; Female genital (1)[12]; General and Unspecified (2)[12, 14]
Cognates: Mayan: pak'aC; Huas: pak'ak;
Language contact:

***Nopalea guatemalensis*** **Roze (Cactaceae)**

Spanish names: Nopal
Indigenous names: Vaas pim[14]
Used by (1*): Quichean Maya[14]
Used for (1#): Musculoskeletal (1)[14]
Cognates:
Language contact:

***Nopalea*** **sp. (Cactaceae)**

Spanish names: Nopal
Indigenous names: Nakpat[01]; Kum pasyte[02]; Taat[04]; Aa'xilh[06]; Bia'aj[21]
Used by (7*): Zoque[01, 02, 03]; Mixe[04]; Totonac[06]; Zapotec[21]; Nahua[27]
Used for (26#): Blood (1)[03]; Digestive (2)[01, 21]; Cardiovascular (2)[01, 02]; Musculoskeletal (3)[01, 02, 21]; Neurological (1)[01]; Respiratory (3)[01, 06, 21]; Skin (2)[01, 02]; Endocrine (6)[01, 02, 02, 06, 21, 27]; Urological (2)[01, 02]; Pregnancy (1)[04]; Female genital (1)[01]; General and Unspecified (2)[03, 21]
Cognates: Zoq: pat/pax;
Language contact:

***Nymphaea ampla*** **(Salisb.) DC. (Nymphaeaceae)**

Spanish names: Lagua; Repollo
Indigenous names: Pulul[07]; Ch'oop Q'ehen[14]; Lagua[18]
Used by (3*): Huastec[07]; Quichean Maya[14]; Western Maya[18]
Used for (4#): Neurological (1)[07]; Skin (1)[18]; Pregnancy (1)[14]; General and Unspecified (1)[07]
Cognates:
Language contact:

***Ocimum basilicum*** **L. (Lamiaceae)**

Spanish names: Albahaca
Indigenous names: Ukä ay jäyä[02]; Xuuik[04]; Laab thekw'eel[07]; Lap thek'weel[08]; Albajaka[18]; Guiasharu'uj[21]; Talachía[24]; Sordoxihuitle[26]
Used by (18*): Zoque[02, 03]; Mixe[04]; Totonac[05]; Huastec[07, 08]; Yucatecan Maya[09]; Quichean Maya[12, 13, 14]; Western Maya[18]; Zapotec[21, 23]; Nahua[24, 25, 26, 27, 28]
Used for (67#): Digestive (11)[03, 05, 08, 12, 13, 18, 21, 25, 26, 27, 28]; Eye (2)[09, 18]; Ear (2)[04, 23]; Cardiovascular (2)[08, 25]; Musculoskeletal (5)[05, 08, 12, 18, 21]; Neurological (9)[02, 03, 07, 08, 09, 12, 18, 23, 25]; Psychological (3)[05, 12, 21]; Respiratory (5)[03, 08, 18, 27, 28]; Skin (4)[04, 08, 12, 26]; Urological (2)[02, 12]; Pregnancy (4)[03, 05, 08, 24]; Female genital (4)[03, 05, 12, 21]; General and Unspecified (14)[02, 03, 04, 05, 07, 08, 12, 13, 14, 18, 21, 23, 26, 27]
Cognates:
Language contact:

[01-28]refer to the study codes in Table 4.1. *Total number of studies citing this taxon #Total number of use-records

***Ocimum campechianum* Mill. (Lamiaceae)**

Spanish names: Albahaca cimarrón
Indigenous names: Nas moki pa'a/ moki une[01]; Paxuuik[04]; Tsin thekw'eel, chithan thekw'eel, thuutsub, [07]; Xkakaltun[09]; Xtem Qana'[14]; Obej', benq[15]; Xuch päm pimi[18]
Used by (11*): Zoque[01, 03]; Mixe[04]; Huastec[07]; Yucatecan Maya[09, 10, 11]; Quichean Maya[14, 15]; Western Maya[18]; Nahua[26]
Used for (24#): Digestive (3)[07, 09, 11]; Eye (3)[03, 10, 11]; Ear (2)[03, 14]; Musculoskeletal (1)[01]; Neurological (4)[01, 07, 11, 26]; Psychological (1)[07]; Respiratory (1)[01]; Skin (2)[01, 09]; Pregnancy (2)[03, 26]; Female genital (1)[15]; General and Unspecified (4)[01, 04, 07, 18]
Cognates: MZ: pa;
Language contact:

***Ocimum carnosum* (Spreng.) Link & Otto ex Benth. (Lamiaceae)**

Spanish names: Hoja de cólico/hierba santa marta/ hierba de cólico; Siempreviva; Tepocía
Indigenous names: Tung an petkuy/tzukspa' tane/näk tane/ tzukin jäyä/yäjkuy tane[01]; Paxcahujnuk[05]; Ik ts'ohool[07]; Chitam wamal, poxil ik', bakel chitam, tza'los wamal, san mikel wamal, ch'aal wamal, yax wamal, xulem te'[20]
Used by (5*): Zoque[01]; Totonac[05]; Huastec[07]; Western Maya[20]; Nahua[25]
Used for (11#): Digestive (3)[01, 05, 20]; Musculoskeletal (2)[01, 20]; Neurological (1)[07]; Psychological (1)[01]; Respiratory (2)[20, 25]; General and Unspecified (2)[01, 25]
Cognates:
Language contact: Chiapas Zoq <> Tot; Tot <> Tzeltalan; Hua <> Tzeltalan

***Ocimum gratissimum* L. (Lamiaceae)**

Spanish names: Oregano
Indigenous names:
Used by (1*): Zoque[01]
Used for (2#): Digestive (1)[01]; General and Unspecified (1)[01]
Cognates:
Language contact:

***Ocimum* sp. (Lamiaceae)**

Spanish names: Albahaca; Albahaca (cimarrona)
Indigenous names: Tzukspa'tane [01]; Ay jäyä[02]
Used by (4*): Zoque[01, 02]; Western Maya[19]; Zapotec[22]
Used for (30#): Blood (1)[01]; Digestive (3)[01, 02, 19]; Eye (3)[01, 02, 19]; Ear (3)[01, 02, 19]; Cardiovascular (2)[01, 02]; Musculoskeletal (2)[01, 02]; Neurological (2)[01, 02]; Psychological (1)[01]; Respiratory (3)[01, 01, 02]; Skin (1)[02]; Endocrine (1)[02]; Pregnancy (2)[02, 19]; Female genital (3)[01, 02, 19]; General and Unspecified (3)[01, 02, 22]
Cognates:
Language contact:

***Ocimum tenuiflorum* L. (Lamiaceae)**

Spanish names: Albahaca morada
Indigenous names:
Used by (1*): Quichean Maya[13]
Used for (3#): Digestive (1)[13]; Ear (1)[13]; Neurological (1)[13]
Cognates:
Language contact:

***Ocotea dendrodaphne* Mez (Lauraceae)**

Spanish names: Rosa negra
Indigenous names: Moko[03]
Used by (1*): Zoque[03]
Used for (1#): Digestive (1)[03]
Cognates:
Language contact:

[01-28]refer to the study codes in Table 4.1. *Total number of studies citing this taxon #Total number of use-records

***Ocotea heydeana*** **(Mez & Donn.Sm.) Bernardi (Lauraceae)**

Spanish names: Laurelillo
Indigenous names: Chuch moko[03]
Used by (1*): Zoque[03]
Used for (2#): Digestive (1)[03]; Cardiovascular (1)[03]
Cognates:
Language contact:

***Ocotea veraguensis*** **(Meissn.) Mez (Lauraceae)**

Spanish names: Laurel Negro
Indigenous names:
Used by (1*): Zoque[03]
Used for (3#): Digestive (1)[03]; Skin (1)[03]; Female genital (1)[03]
Cognates:
Language contact:

***Odontonema albiflorum*** **Leonard (Acanthaceae)**

Spanish names:
Indigenous names: Sam Ajtzo'[14]
Used by (1*): Quichean Maya[14]
Used for (2#): Musculoskeletal (1)[14]; Neurological (1)[14]
Cognates:
Language contact:

***Odontonema callistachyum*** **(Schltdl. & Cham.) Kuntze (Acanthaceae)**

Spanish names: Hoja de llanto; Hoja de paperón
Indigenous names: Naktam ay[03]; T'a'lom ts'ohool, k'alul ts'ohool[07]; Kux' Luq' Salaq' Baqel', Sam Ajtzo'[14]; Yopo uq'ue[18]
Used by (5*): Zoque[01, 03]; Huastec[07]; Quichean Maya[14]; Western Maya[18]
Used for (10#): Digestive (1)[01]; Ear (1)[03]; Musculoskeletal (2)[03, 14]; Neurological (1)[14]; Respiratory (1)[03]; Skin (1)[03]; General and Unspecified (3)[07, 14, 18]
Cognates:
Language contact:

***Odontonema tubaeforme*** **(Bertol.) Kuntze (Acanthaceae)**

Spanish names:
Indigenous names: T'a'lom ts'ohool, k'alul ts'ohool[07]
Used by (1*): Huastec[07]
Used for (1#): General and Unspecified (1)[07]
Cognates:
Language contact:

***Oeceoclades maculata*** **(Lindl.) Lindl. (Orchidaceae)**

Spanish names: Espalda de la Culebra, Curarina
Indigenous names: Rix li kanti, Xb'an Xmay Ixul[14]
Used by (1*): Quichean Maya[14]
Used for (2#): Skin (1)[14]; General and Unspecified (1)[14]
Cognates:
Language contact:

***Oecopetalum mexicanum*** **Greenm, & C.H. Thomps. (Icanicaceae)**

Spanish names: Cacate
Indigenous names: Kuk yaka/ kukyak kawak/ kokya kawa[01]
Used by (1*): Zoque[01]
Used for (3#): Digestive (1)[01]; Psychological (1)[01]; Endocrine (1)[01]
Cognates:
Language contact:

***Oenothera kunthiana* (Spach) Munz (Onagraceae)**

Spanish names:
Indigenous names: Waakal mo'eel[07]
Used by (1*): Huastec[07]
Used for (3#): Digestive (1)[07]; Psychological (1)[07]; General and Unspecified (1)[07]
Cognates:
Language contact:

***Oenothera pubescens* Willd. ex Spreng. (Onagraceae)**

Spanish names: Tomillo de Montaña
Indigenous names: Tomi'y Aq'om K'echelaj[12]
Used by (1*): Quichean Maya[12]
Used for (3#): Digestive (1)[12]; Respiratory (1)[12]; General and Unspecified (1)[12]
Cognates:
Language contact:

***Oenothera rosea* L'Hér. ex Aiton (Onagraceae)**

Spanish names: Hierba del golpe, hierba cólica, hoja de azar
Indigenous names: Tzate kä tane/ pu tzäpäsy häyä/ tz'a tat käyi tane[01]; Piiquaa't xanat[06]; Ts'een waakal mo'eel[07]; Ist'een wakal mo'el[08]; Guièe-zhàn̲-biăa[23]; Coxcatlacuache[26]; Cupachocotl[28]
Used by (8*): Zoque[01]; Totonac[06]; Huastec[07, 08]; Zapotec[23]; Nahua[25, 26, 28]
Used for (22#): Blood (1)[06]; Digestive (3)[25, 26, 28]; Musculoskeletal (2)[07, 08]; Neurological (2)[23, 25]; Respiratory (2)[23, 25]; Skin (5)[06, 08, 25, 26, 28]; Urological (1)[26]; Female genital (1)[26]; General and Unspecified (5)[01, 06, 08, 23, 25]
Cognates: Huas: t(s)'een wakal mo'el;
Language contact:

***Oenothera tetraptera* Cav. (Onagraceae)**

Spanish names:
Indigenous names: Waakal mo'eel, thamuul wich[07]
Used by (1*): Huastec[07]
Used for (3#): Digestive (1)[07]; Psychological (1)[07]; General and Unspecified (1)[07]
Cognates:
Language contact:

***Olea europaea* L. (Oleaceae)**

Spanish names: Aceituna; Olivo
Indigenous names:
Used by (2*): Quichean Maya[12, 13]
Used for (3#): Digestive (1)[12]; Pregnancy (1)[13]; General and Unspecified (1)[13]
Cognates:
Language contact:

***Olyra latifolia* L. (Poaceae)**

Spanish names:
Indigenous names: Xuwan Q'ehen[14]
Used by (1*): Quichean Maya[14]
Used for (1#): Female genital (1)[14]
Cognates:
Language contact:

[01-28] refer to the study codes in Table 4.1. *Total number of studies citing this taxon #Total number of use-records

***Oncidium* sp. (Orchidaceae)**

Spanish names: Cola de raton
Indigenous names: Tsuk i tyutsu[03]; Oxib Xjuruch[14]
Used by (2*): Zoque[03]; Quichean Maya[14]
Used for (5#): Ear (1)[03]; Skin (2)[03, 14]; Female genital (2)[03, 14]
Cognates:
Language contact:

***Oncidium sphacelatum* Lindl. (Orchidaceae)**

Spanish names: Orquidea
Indigenous names:
Used by (1*): Zoque[02]
Used for (1#): Respiratory (1)[02]
Cognates:
Language contact:

***Operculina pinnatifida* (Kunth) O'Donell (Convolvulaceae)**

Spanish names:
Indigenous names: Pok' laak, akan tsok'[07]; Rixihij li mis[14]
Used by (2*): Huastec[07]; Quichean Maya[14]
Used for (5#): Digestive (1)[07]; Musculoskeletal (1)[14]; Neurological (1)[07]; Skin (1)[07]; General and Unspecified (1)[07]
Cognates:
Language contact:

***Oplismenus compositus* (L.) P.Beauv. (Poaceae)**

Spanish names:
Indigenous names: Ts'aahil tsan, chukul bat'aw, lakab huchuul, ts'aahil kw'a', tsakam tsahib[07]
Used by (1*): Huastec[07]
Used for (1#): General and Unspecified (1)[07]
Cognates:
Language contact:

***Opuntia decumbens* Salm-Dyck (Cactaceae)**

Spanish names: Nopal
Indigenous names: Yop'nopal[18]
Used by (1*): Western Maya[18]
Used for (3#): Digestive (1)[18]; Respiratory (1)[18]; Endocrine (1)[18]
Cognates:
Language contact:

***Opuntia ficus-indica* (L.) Mill. (Cactaceae)**

Spanish names: Nopal
Indigenous names: Nach'te, nich'te, nachti[13]
Used by (1*): Quichean Maya[13]
Used for (3#): Respiratory (1)[13]; Skin (1)[13]; General and Unspecified (1)[13]
Cognates:
Language contact:

***Opuntia* sp. (Cactaceae)**

Spanish names:
Indigenous names: Pak'ak, k'aan k'iith, thiman pak'ak, bohol pak'ak[07]; Yàg-biǎa[23]
Used by (2*): Huastec[07]; Zapotec[23]
Used for (7#): Digestive (2)[07, 23]; Musculoskeletal (1)[07]; Respiratory (1)[07]; Skin (2)[07, 23]; General and Unspecified (1)[07]
Cognates:
Language contact:

[01-28]refer to the study codes in Table 4.1. *Total number of studies citing this taxon #Total number of use-records

***Opuntia streptacantha* Lem. (Cactaceae)**

Spanish names: Nopal
Indigenous names:
Used by (1*): Nahua[25]
Used for (2#): Respiratory (1)[25]; Endocrine (1)[25]
Cognates:
Language contact:

***Oreopanax peltatus* Linden ex Regel (Araliaceae)**

Spanish names: Palo de agua
Indigenous names:
Used by (1*): Nahua[26]
Used for (1#): Respiratory (1)[26]
Cognates:
Language contact:

***Origanum majorana* L. (Lamiaceae)**

Spanish names: Oregano, mejorana
Indigenous names:
Used by (1*): Nahua[26]
Used for (4#): Digestive (1)[26]; Psychological (1)[26]; Pregnancy (1)[26]; Female genital (1)[26]
Cognates:
Language contact:

***Origanum* sp. (Lamiaceae)**

Spanish names: Oregano; Oregano de comida
Indigenous names: Mehorana[07]
Used by (4*): Zoque[01, 02]; Huastec[07]; Zapotec[21]
Used for (7#): Digestive (2)[01, 02]; Neurological (1)[02]; Female genital (1)[02]; General and Unspecified (3)[01, 07, 21]
Cognates:
Language contact:

***Origanum vulgare* L. (Lamiaceae)**

Spanish names: Oregano, mejorana
Indigenous names: Tsiimal koy[08]
Used by (6*): Totonac[06]; Huastec[08]; Yucatecan Maya[09]; Quichean Maya[12]; Zapotec[22]; Nahua[25]
Used for (19#): Digestive (4)[06, 08, 12, 22]; Musculoskeletal (2)[08, 12]; Neurological (2)[08, 12]; Psychological (3)[06, 08, 12]; Respiratory (3)[06, 08, 12]; Urological (1)[12]; Pregnancy (1)[09]; Female genital (2)[06, 12]; General and Unspecified (1)[25]
Cognates:
Language contact:

***Ormosia isthmensis* Standl. (Fabaceae)**

Spanish names: Coral
Indigenous names: May cuy[03]
Used by (1*): Zoque[03]
Used for (2#): Pregnancy (1)[03]; Female genital (1)[03]
Cognates:
Language contact:

***Ornithocephalus inflexus* Lindl. (Orchidaceae)**

Spanish names:
Indigenous names: X-xi' Mukuy[14]
Used by (1*): Quichean Maya[14]
Used for (2#): Skin (1)[14]; General and Unspecified (1)[14]
Cognates:
Language contact:

[01-28]refer to the study codes in Table 4.1. *Total number of studies citing this taxon #Total number of use-records

***Orthrosanthus chimboracensis* (Kunth) Baker (Iridaceae)**

Spanish names: Palma Morada; Petate de ratón
Indigenous names: Aq'om Kotzij[12]; Pop chó[13]
Used by (2*): Quichean Maya[12, 13]
Used for (2#): Digestive (1)[12]; Female genital (1)[13]
Cognates:
Language contact:

***Orthrosanthus monadelphus* Ravenna (Iridaceae)**

Spanish names:
Indigenous names: Xmes hi' ha'[14]
Used by (1*): Quichean Maya[14]
Used for (1#): Respiratory (1)[14]
Cognates:
Language contact:

***Oryctanthus* sp. (Loranthaceae)**

Spanish names:
Indigenous names: Ne'ba pim[16]
Used by (1*): Quichean Maya[16]
Used for (1#): Psychological (1)[16]
Cognates:
Language contact:

***Oryza sativa* L. (Poaceae)**

Spanish names: Arroz
Indigenous names:
Used by (5*): Zoque[01, 02]; Mixe[04]; Quichean Maya[12]; Zapotec[21]
Used for (7#): Digestive (4)[01, 02, 04, 21]; Musculoskeletal (1)[01]; General and Unspecified (2)[12, 21]
Cognates:
Language contact:

***Osmunda regalis* L. (Osmundaceae)**

Spanish names: Helecho real
Indigenous names:
Used by (1*): Quichean Maya[13]
Used for (1#): Musculoskeletal (1)[13]
Cognates:
Language contact:

***Ostrya virginiana* (Mill.) K.Koch (Betulaceae)**

Spanish names: Encino niño/roble enano
Indigenous names: Une kamay/ tzut kamay[01]
Used by (1*): Zoque[01]
Used for (2#): Digestive (1)[01]; Respiratory (1)[01]
Cognates:
Language contact:

***Oxalis corniculata* L. (Oxalidaceae)**

Spanish names: Hierba de Chicha, Trebolillo amarillo; Hoja de azar; Trebol
Indigenous names: Tza'a tzoy[01]; Q'ais ch'am, Q'en Lotz Aq'om[12]; Kieq lotz[13]; Guìzh-bdiò-guìx, xín-guièe-bè, guìzh-bè-làs[23]
Used by (5*): Zoque[01, 03]; Quichean Maya[12, 13]; Zapotec[23]
Used for (18#): Blood (1)[12]; Digestive (3)[12, 13, 23]; Eye (1)[12]; Cardiovascular (1)[12]; Neurological (2)[01, 12]; Psychological (1)[12]; Skin (4)[01, 03, 12, 13]; Urological (1)[03]; Pregnancy (1)[03]; Female genital (1)[12]; General and Unspecified (2)[01, 12]
Cognates: Quich: lots;
Language contact:

[01-28]refer to the study codes in Table 4.1. *Total number of studies citing this taxon #Total number of use-records

***Oxalis dillenii* Jacq. (Oxalidaceae)**

Spanish names:
Indigenous names: Kital ichiich[07]
Used by (1*): Huastec[07]
Used for (1#): General and Unspecified (1)[07]
Cognates:
Language contact:

***Oxalis divergens* Benth. ex Lindl. (Oxalidaceae)**

Spanish names: Calzoncillo de Agua, Trebol silvestre; Trebol, acederill, aleluya, vinagrio
Indigenous names: Lost, Oxi' Ruxaq Q'os[12]; Lotz[13]
Used by (2*): Quichean Maya[12, 13]
Used for (11#): Blood (1)[12]; Digestive (2)[12, 13]; Eye (1)[12]; Musculoskeletal (1)[12]; Neurological (1)[12]; Psychological (1)[12]; Respiratory (1)[13]; Skin (2)[12, 13]; General and Unspecified (1)[12]
Cognates: Quich: lots;
Language contact:

***Oxalis frutescens* L. (Oxalidaceae)**

Spanish names: Capulin blanco; Hierba de sentimiento, hierba de pesar; Hoja de azar; Tamarindillo, monte de preñiz
Indigenous names: Tza'a tzoy[01]; Pixtic sotyi[03]; Ajsyan k'opot, pajpaj k'opot[19]
Used by (4*): Zoque[01, 03]; Yucatecan Maya[11]; Western Maya[19]
Used for (6#): Digestive (1)[19]; Skin (2)[03, 11]; General and Unspecified (3)[01, 03, 11]
Cognates:
Language contact:

***Oxalis latifolia* Kunth (Oxalidaceae)**

Spanish names:
Indigenous names: Hilil ts'ohool, otsom ichiich, t'i'kom ichiich[07]; Elel[09]
Used by (2*): Huastec[07]; Yucatecan Maya[09]
Used for (4#): Digestive (1)[07]; Skin (2)[07, 09]; General and Unspecified (1)[09]
Cognates:
Language contact: Hua <> Yuc

***Oxalis rhombifolia* Jacq. (Oxalidaceae)**

Spanish names: Capulin blanco
Indigenous names: Paxum[04]
Used by (2*): Zoque[03]; Mixe[04]
Used for (2#): Digestive (1)[04]; Psychological (1)[03]
Cognates:
Language contact:

***Oxyrhynchus volubilis* Brandegee (Fabaceae)**

Spanish names:
Indigenous names: Wal pooy[07]
Used by (1*): Huastec[07]
Used for (1#): Eye (1)[07]
Cognates:
Language contact:

***Pachira aquatica* Aubl. (Malvaceae)**

Spanish names: Zapote de agua
Indigenous names: Uwakta[01]; Uakta[03]; Ka'pa'[05]; K'unche', K'uyche'[09]; Sapote bobo[10]; Ajp'o tec[18]
Used by (6*): Zoque[01, 03]; Totonac[05]; Yucatecan Maya[09, 10]; Western Maya[18]
Used for (9#): Digestive (1)[18]; Eye (1)[18]; Skin (1)[05]; Endocrine (3)[01, 03, 18]; Urological (2)[03, 10]; General and Unspecified (1)[09]
Cognates: Zoq: uakta;
Language contact:

[01-28]refer to the study codes in Table 4.1. *Total number of studies citing this taxon #Total number of use-records

***Pachyrhizus erosus* (L.) Urb. (Fabaceae)**

Spanish names: Jicama
Indigenous names: Kobeem[07]; Kup[09]
Used by (2*): Huastec[07]; Yucatecan Maya[09]
Used for (2#): Respiratory (1)[09]; Skin (1)[07]
Cognates: Mayan: kob / kup;
Language contact:

***Panicum virgatum* L. (Poaceae)**

Spanish names:
Indigenous names: Itse' toom[07]
Used by (1*): Huastec[07]
Used for (1#): Respiratory (1)[07]
Cognates:
Language contact:

***Parathesis hondurensis* Standl. (Primulaceae)**

Spanish names:
Indigenous names: Tilil ja'[20]
Used by (1*): Western Maya[20]
Used for (1#): Digestive (1)[20]
Cognates:
Language contact:

***Parathesis neei* Lundell (Primulaceae)**

Spanish names:
Indigenous names: Chu cuxamñi[03]
Used by (1*): Zoque[03]
Used for (2#): Digestive (1)[03]; Female genital (1)[03]
Cognates:
Language contact:

***Parathesis serrulata* (Sw.) Mez (Primulaceae)**

Spanish names:
Indigenous names: Apulee', ebha'[07]
Used by (1*): Huastec[07]
Used for (2#): Digestive (1)[07]; General and Unspecified (1)[07]
Cognates:
Language contact:

***Parathesis* sp. (Primulaceae)**

Spanish names: Llorasangre, Corazón de la Selva
Indigenous names: Ruk'uch Juyu[12]
Used by (1*): Quichean Maya[12]
Used for (11#): Blood (1)[12]; Digestive (1)[12]; Cardiovascular (1)[12]; Musculoskeletal (2)[12, 12]; Psychological (1)[12]; Respiratory (2)[12, 12]; Endocrine (1)[12]; Urological (1)[12]; General and Unspecified (1)[12]
Cognates:
Language contact:

[01-28] refer to the study codes in Table 4.1. *Total number of studies citing this taxon #Total number of use-records

***Parmentiera aculeata* (Kunth) Seem. (Bignoniaceae)**

Spanish names: Cuajilote
Indigenous names: Apit kuy[01]; Paka'ak[04]; Pusni, puxni[05]; Pux'nit[06]; Tsoote'[07]; Kat[09]; Cho'te[18]; Biguaj guedxii[21]; Cuaxilotl[28]
Used by (11*): Zoque[01, 02, 03]; Mixe[04]; Totonac[05, 06]; Huastec[07]; Yucatecan Maya[09]; Western Maya[18]; Zapotec[21]; Nahua[28]
Used for (38#): Digestive (3)[01, 05, 07]; Ear (4)[05, 06, 07, 18]; Neurological (1)[05]; Respiratory (9)[01, 02, 03, 04, 05, 07, 18, 18, 21]; Skin (1)[07]; Endocrine (5)[05, 06, 09, 18, 28]; Urological (6)[05, 06, 07, 09, 18, 28]; Pregnancy (2)[03, 05]; Female genital (1)[03]; Male genital (1)[01]; General and Unspecified (5)[02, 05, 07, 18, 28]
Cognates: Toto: puxni; Mayan: tsoote/cho'te;
Language contact:

***Parmentiera millspaughiana* L.O.Williams (Bignoniaceae)**

Spanish names:
Indigenous names: Katche'[09]
Used by (1*): Yucatecan Maya[09]
Used for (1#): Urological (1)[09]
Cognates:
Language contact:

***Parthenium hysterophorus* L. (Asteraceae)**

Spanish names: Hierba maestra, altamisa
Indigenous names: Tzaj'u pu, ma'tza jäyä[01]; Pa artemis, artemis ujts[04]; Ts'a'il kw'eet, kaxiy kw'eet[07]; Cilantro Ši'u [10]; Corriente Ši'u [10]
Used by (9*): Zoque[01, 03]; Mixe[04]; Huastec[07]; Yucatecan Maya[09, 10, 11]; Western Maya[18]; Zapotec[21]
Used for (25#): Blood (1)[10]; Digestive (2)[07, 18]; Cardiovascular (1)[01]; Musculoskeletal (6)[01, 03, 04, 07, 18, 21]; Respiratory (1)[01]; Skin (3)[01, 07, 21]; Endocrine (2)[01, 03]; Pregnancy (1)[07]; Female genital (1)[09]; General and Unspecified (7)[01, 04, 07, 09, 10, 11, 21]
Cognates:
Language contact:

***Paspalum conjugatum* P.J.Bergius (Poaceae)**

Spanish names: Zacate grama
Indigenous names: So'ok[01]
Used by (2*): Zoque[01, 02]
Used for (3#): Psychological (1)[01]; Urological (1)[01]; General and Unspecified (1)[02]
Cognates:
Language contact:

***Passiflora ambigua* Hemsl. (Passifloraceae)**

Spanish names: Gagapachi
Indigenous names:
Used by (1*): Zoque[03]
Used for (3#): Eye (1)[03]; Psychological (1)[03]; Urological (1)[03]
Cognates:
Language contact:

***Passiflora biflora* Lam. (Passifloraceae)**

Spanish names: Ala de murcielago
Indigenous names: Sindi sprun, sandia spuun[06]
Used by (1*): Totonac[06]
Used for (1#): Urological (1)[06]
Cognates:
Language contact:

[01-28]refer to the study codes in Table 4.1. *Total number of studies citing this taxon #Total number of use-records

**_Passiflora ciliata_ Aiton (Passifloraceae)**

Spanish names: Pepe
Indigenous names:
Used by (1*): Zoque[02]
Used for (6[#]): Digestive (1)[02]; Cardiovascular (1)[02]; Psychological (1)[02]; Respiratory (1)[02]; Skin (1)[02]; Endocrine (1)[02]
Cognates:
Language contact:

**_Passiflora coriacea_ Juss. (Passifloraceae)**

Spanish names: Ala de murciélago
Indigenous names: Okoob thut'[07]; Xik'sots'[09]; Šik'soo¢[10]; Xik'isotz[14]
Used by (5*): Totonac[05]; Huastec[07]; Yucatecan Maya[09, 10]; Quichean Maya[14]
Used for (14[#]): Blood (1)[05]; Digestive (1)[07]; Ear (2)[07, 09]; Neurological (2)[07, 14]; Skin (2)[07, 14]; Urological (1)[05]; Pregnancy (1)[07]; Social problems (1)[14]; General and Unspecified (3)[07, 10, 14]
Cognates: CoreM: xik'sots; Yuca: xik'sots;
Language contact:

**_Passiflora edulis_ Sims (Passifloraceae)**

Spanish names: Granadilla; Maracuyá
Indigenous names: Tu' kej'[14]
Used by (3*): Zoque[02]; Quichean Maya[14]; Nahua[28]
Used for (8[#]): Digestive (1)[28]; Cardiovascular (1)[28]; Neurological (1)[28]; Psychological (1)[02]; Respiratory (1)[14]; Endocrine (1)[02]; Urological (1)[02]; General and Unspecified (1)[14]
Cognates:
Language contact:

**_Passiflora foetida_ L. (Passifloraceae)**

Spanish names: Jujito, top'o lac'; Monte flución, monte preñiz
Indigenous names: Xiu tiepo[03]; Tanto'xy[04]; Pok'pok', owel paat, tsakam hiliy[07]; Xpoch[09]; Juju'[18]; Pa'ch'em, julusyon k'opot[19]
Used by (6*): Zoque[03]; Mixe[04]; Huastec[07]; Yucatecan Maya[09]; Western Maya[18, 19]
Used for (17[#]): Digestive (1)[07]; Cardiovascular (1)[18]; Musculoskeletal (1)[19]; Neurological (2)[07, 19]; Psychological (2)[03, 18]; Skin (2)[03, 09]; Urological (2)[03, 04]; Pregnancy (1)[07]; Female genital (3)[03, 07, 18]; General and Unspecified (2)[18, 19]
Cognates:
Language contact: Hua <> Yuc <> Chortí

**_Passiflora hahnii_ (E.Fourn.) Mast. (Passifloraceae)**

Spanish names: Ala de Muerciélago
Indigenous names: Rismal ru' qanyaj, Xik'isotz[14]; Tu' kej[17]
Used by (2*): Quichean Maya[14, 17]
Used for (5[#]): Digestive (1)[14]; Neurological (1)[14]; Urological (1)[14]; Male genital (1)[14]; nd[17]
Cognates:
Language contact:

**_Passiflora ligularis_ Juss. (Passifloraceae)**

Spanish names: Granadilla, Passiflora
Indigenous names:
Used by (1*): Quichean Maya[12]
Used for (5[#]): Blood (1)[12]; Digestive (1)[12]; Musculoskeletal (1)[12]; Psychological (1)[12]; Respiratory (1)[12]
Cognates:
Language contact:

[01-28]refer to the study codes in Table 4.1. *Total number of studies citing this taxon [#]Total number of use-records

***Passiflora membranacea* Benth. (Passifloraceae)**

Spanish names: Granadilla de Culebra, Passiflora, Riñon de Montaña
Indigenous names: Cruz Q'an, Aq'om Q'ayis Riñon[12]
Used by (1*): Quichean Maya[12]
Used for (3#): Digestive (1)[12]; Psychological (1)[12]; Urological (1)[12]
Cognates:
Language contact:

***Passiflora oerstedii* Mast. (Passifloraceae)**

Spanish names:
Indigenous names: Tu' kej[17]
Used by (1*): Quichean Maya[17]
Used for (1#): nd[17]
Cognates:
Language contact:

***Passiflora ornithoura* Mast. (Passifloraceae)**

Spanish names: Cachito rastrero, calzoncillo
Indigenous names: Aq'om Kowil Q'os Ka'[12]
Used by (1*): Quichean Maya[12]
Used for (7#): Digestive (1)[12]; Musculoskeletal (1)[12]; Neurological (1)[12]; Endocrine (1)[12]; Urological (1)[12]; Male genital (1)[12]; General and Unspecified (1)[12]
Cognates:
Language contact:

***Passiflora palmeri* Rose (Passifloraceae)**

Spanish names: Passiflora
Indigenous names: Tzi pono [01]
Used by (1*): Zoque[01]
Used for (2#): Neurological (1)[01]; Psychological (1)[01]
Cognates:
Language contact:

***Passiflora serratifolia* L. (Passifloraceae)**

Spanish names:
Indigenous names: Xiu tiepo[03]
Used by (1*): Zoque[03]
Used for (3#): Eye (1)[03]; Skin (1)[03]; Urological (1)[03]
Cognates:
Language contact:

***Passiflora sexflora* Juss. (Passifloraceae)**

Spanish names: Guaco
Indigenous names:
Used by (1*): Quichean Maya[12]
Used for (2#): Skin (1)[12]; General and Unspecified (1)[12]
Cognates:
Language contact:

***Passiflora* sp. (Passifloraceae)**

Spanish names: Granadilla; Pepe, passiflora, granadita
Indigenous names: Tú' kej[14]; Granadillo y Cho' Ho'[14]; Roq' Maus aj' Winq[14]; Tu'kej[16]; Wapapa[21]; Ibæ̀-gràn̂âd, guìzh-gràn̂âd, gràn̂âd-dán[23]
Used by (4*): Quichean Maya[14, 16]; Zapotec[21, 23]
Used for (12#): Digestive (2)[14, 21]; Neurological (3)[14, 16, 23]; Psychological (1)[21]; Respiratory (1)[14]; Skin (1)[21]; Female genital (1)[21]; General and Unspecified (3)[14, 14, 21]
Cognates:
Language contact: Zap > Chimalapa Zoq via Spanish

***Passiflora subpeltata* Ortega (Passifloraceae)**

Spanish names:
Indigenous names: Choq'l pim[14]
Used by (1*): Quichean Maya[14]
Used for (1#): Respiratory (1)[14]
Cognates:
Language contact:

***Paullinia pinnata* L. (Sapindaceae)**

Spanish names:
Indigenous names: K¥kujuki ay[03]
Used by (1*): Zoque[03]
Used for (2#): Skin (1)[03]; Female genital (1)[03]
Cognates:
Language contact:

***Paullinia* sp. (Sapindaceae)**

Spanish names:
Indigenous names:
Used by (1*): Zoque[03]
Used for (4#): Digestive (1)[03]; Eye (1)[03]; Urological (1)[03]; Pregnancy (1)[03]
Cognates:
Language contact:

***Paullinia tomentosa* Jacq. (Sapindaceae)**

Spanish names:
Indigenous names: T'in kamab, tu kamaab[07]
Used by (1*): Huastec[07]
Used for (5#): Digestive (1)[07]; Skin (1)[07]; Endocrine (1)[07]; Pregnancy (1)[07]; Female genital (1)[07]
Cognates:
Language contact:

***Paullinia venosa* Radlk. (Sapindaceae)**

Spanish names:
Indigenous names:
Used by (1*): Zoque[03]
Used for (1#): Skin (1)[03]
Cognates:
Language contact:

***Pavonia rosea* Wall. ex Moris (Malvaceae)**

Spanish names: Lengua de perro; Lengua de vaca
Indigenous names: Kam dane[01]; Mul Tzi'[14]
Used by (3*): Zoque[01, 02]; Quichean Maya[14]
Used for (5[#]): Digestive (1)[01]; Cardiovascular (1)[01]; Neurological (1)[01]; Skin (2)[02, 14]
Cognates:
Language contact:

***Pavonia schiedeana* Steud. (Malvaceae)**

Spanish names: Cadillo
Indigenous names: Konko[03]; Uk tuukats[04]; Ts'ikiy uxum, liin ts'ohool[07]; Mul Tzi'[14]
Used by (4*): Zoque[03]; Mixe[04]; Huastec[07]; Quichean Maya[14]
Used for (14[#]): Digestive (2)[03, 07]; Musculoskeletal (1)[07]; Respiratory (1)[07]; Skin (2)[03, 07]; Urological (1)[07]; Pregnancy (4)[03, 03, 07, 14]; Female genital (1)[07]; General and Unspecified (2)[04, 14]
Cognates:
Language contact: Mixe <> Hua; Hua <> Kekchí

***Pecluma plumula* (Humb. & Bonpl. ex Willd.) M.G. Price (Polypodiaceae)**

Spanish names:
Indigenous names: Ehtiil weew koxol[07]
Used by (1*): Huastec[07]
Used for (1[#]): Neurological (1)[07]
Cognates:
Language contact:

***Pecluma* sp. (Polypodiaceae)**

Spanish names:
Indigenous names:
Used by (1*): Zoque[03]
Used for (2[#]): Digestive (1)[03]; General and Unspecified (1)[03]
Cognates:
Language contact:

***Pelargonium × hortorum* L.H. Bailey (Geraniaceae)**

Spanish names: Geranio; Geranio (rojo)
Indigenous names:
Used by (2*): Zoque[01]; Quichean Maya[12]
Used for (5[#]): Blood (1)[12]; Musculoskeletal (1)[01]; Neurological (2)[01, 12]; General and Unspecified (1)[12]
Cognates:
Language contact:

***Pelargonium graveolens* L'Hér. (Geraniaceae)**

Spanish names: Geranio (doméstico)
Indigenous names:
Used by (1*): Quichean Maya[12]
Used for (2[#]): Respiratory (1)[12]; Skin (1)[12]
Cognates:
Language contact:

***Pelargonium zonale* (L.) L'Hér. ex Aiton (Geraniaceae)**

Spanish names: Geranio; Geranio, solferino; Malva
Indigenous names: Guièe-jèrân[23]; Tlalalatl[28]
Used by (3*): Quichean Maya[13]; Zapotec[23]; Nahua[28]
Used for (3[#]): Digestive (1)[13]; Skin (1)[23]; Female genital (1)[28]
Cognates:
Language contact:

***Pellaea* sp. (Pteridaceae)**

Spanish names:
Indigenous names: Ncuàan-dzéb[23]
Used by (1*): Zapotec[23]
Used for (1[#]): General and Unspecified (1)[23]
Cognates:
Language contact:

***Pennisetum bambusiforme* (E.Fourn.) B.D.Jacks. (Poaceae)**

Spanish names:
Indigenous names: Pakaab[07]
Used by (1*): Huastec[07]
Used for (2[#]): Skin (1)[07]; General and Unspecified (1)[07]
Cognates:
Language contact:

***Pennisetum purpureum* Schumach. (Poaceae)**

Spanish names: Carizo
Indigenous names: Kape[01]
Used by (1*): Zoque[01]
Used for (2[#]): Skin (1)[01]; Urological (1)[01]
Cognates:
Language contact:

***Penstemon* sp. (Plantaginaceae)**

Spanish names:
Indigenous names: Guièe-dzĭng, guìzh-guièe-dzĭng, guìzh-dzĭng, guièe-mĕets, guièe-mìtsiĕ[23]
Used by (1*): Zapotec[23]
Used for (2[#]): Skin (1)[23]; General and Unspecified (1)[23]
Cognates:
Language contact:

***Pentalinon andrieuxii* (Müll.Arg.) B.F.Hansen & Wunderlin (Apocynaceae)**

Spanish names: Viperol verde
Indigenous names: Look' ts'aah[07]; Q'aru pim[14]
Used by (3*): Huastec[07]; Yucatecan Maya[09]; Quichean Maya[14]
Used for (3[#]): Skin (2)[07, 09]; Endocrine (1)[14]
Cognates:
Language contact:

***Peperomia asarifolia* Schltdl. (Piperaceae)**

Spanish names:
Indigenous names:
Used by (1*): Zoque[03]
Used for (2[#]): Musculoskeletal (1)[03]; Skin (1)[03]
Cognates:
Language contact:

***Peperomia blanda* (Jacq.) Kunth (Piperaceae)**

Spanish names:
Indigenous names: Kiñi ay[03]
Used by (1*): Zoque[03]
Used for (4[#]): Ear (1)[03]; Musculoskeletal (1)[03]; Skin (1)[03]; General and Unspecified (1)[03]
Cognates:
Language contact:

[01-28]refer to the study codes in Table 4.1. *Total number of studies citing this taxon [#]Total number of use-records

***Peperomia deppeana*** **Schltdl. & Cham. (Piperaceae)**

Spanish names:
Indigenous names: Conchuru ay, Epxi ay[03]
Used by (1*): Zoque[03]
Used for (4#): Ear (1)[03]; Musculoskeletal (1)[03]; Skin (1)[03]; General and Unspecified (1)[03]
Cognates:
Language contact:

***Peperomia elsana*** **Trel. & Yunck. (Piperaceae)**

Spanish names: Pata paloma
Indigenous names: Riqan plamux[13]
Used by (1*): Quichean Maya[13]
Used for (1#): Skin (1)[13]
Cognates:
Language contact:

***Peperomia glabella*** **(Sw.) A.Dietr. (Piperaceae)**

Spanish names:
Indigenous names: Tsakam ix tuyuum[07]; Chak B'olay Q'ehen, Mai pim, Leetzeb pim[14]
Used by (3*): Zoque[03]; Huastec[07]; Quichean Maya[14]
Used for (6#): Neurological (1)[14]; Skin (3)[03, 07, 14]; Female genital (1)[14]; General and Unspecified (1)[14]
Cognates:
Language contact:

***Peperomia granulosa*** **Trel. (Piperaceae)**

Spanish names:
Indigenous names: Kiñi ay[03]
Used by (1*): Zoque[03]
Used for (3#): Ear (1)[03]; Musculoskeletal (1)[03]; Skin (1)[03]
Cognates:
Language contact:

***Peperomia hispidula*** **(Sw.) A.Dietr. (Piperaceae)**

Spanish names:
Indigenous names: X cua'i xul[17]; Tzetzetzi[26]
Used by (2*): Quichean Maya[17]; Nahua[26]
Used for (2#): Skin (1)[26]; nd[17]
Cognates:
Language contact:

***Peperomia inaequalifolia*** **Ruiz & Pav. (Piperaceae)**

Spanish names: Pega huesos
Indigenous names: Wiq'baq'[13]
Used by (1*): Quichean Maya[13]
Used for (1#): Musculoskeletal (1)[13]
Cognates:
Language contact:

***Peperomia lancifolia*** **Hook. (Piperaceae)**

Spanish names:
Indigenous names:
Used by (1*): Zoque[03]
Used for (1#): Skin (1)[03]
Cognates:
Language contact:

***Peperomia macrostachya*** **(Vahl) A.Dietr. (Piperaceae)**

Spanish names: Xoyoquelite
Indigenous names: Ik B'olay Q'ehen[14]
Used by (2*): Totonac[05]; Quichean Maya[14]
Used for (3#): Skin (1)[14]; Pregnancy (1)[05]; General and Unspecified (1)[14]
Cognates:
Language contact:

***Peperomia maculosa*** **(L.) Hook. (Piperaceae)**

Spanish names:
Indigenous names: Chixchix ay[03]
Used by (1*): Zoque[03]
Used for (2#): Musculoskeletal (1)[03]; Skin (1)[03]
Cognates:
Language contact:

***Peperomia matlalucaensis*** **C.DC. (Piperaceae)**

Spanish names:
Indigenous names:
Used by (1*): Quichean Maya[17]
Used for (1#): nd[17]
Cognates:
Language contact:

***Peperomia obtusifolia*** **(L.) A.Dietr. (Piperaceae)**

Spanish names: Oreja de burro; Oreja de Cabro
Indigenous names: Kiñi ay[03]; Xwa Ixul, Xikyuk[14]
Used by (2*): Zoque[03]; Quichean Maya[14]
Used for (2#): Skin (2)[03, 14]
Cognates:
Language contact:

***Peperomia pellucida*** **(L.) Kunth (Piperaceae)**

Spanish names: Cristalillo; Hierba cristal; Ojo de Gato
Indigenous names: Maj'a rane[01]; Patsuxk juaxy[04]; Se'ru Mes[14]
Used by (5*): Zoque[01, 02]; Mixe[04]; Quichean Maya[14]; Western Maya[18]
Used for (6#): Neurological (1)[01]; Skin (5)[01, 02, 04, 14, 18]
Cognates:
Language contact:

***Peperomia pereskiifolia*** **(Jacq.) Kunth (Piperaceae)**

Spanish names:
Indigenous names: Chicu tats¥k[03]
Used by (1*): Zoque[03]
Used for (3#): Ear (1)[03]; Musculoskeletal (1)[03]; General and Unspecified (1)[03]
Cognates:
Language contact:

***Peperomia quadrifolia*** **(L.) Kunth (Piperaceae)**

Spanish names: Pega huesos
Indigenous names: Poj ujts[04]; Wiq'baq'[13]; Rubel xsa' ixul[14]
Used by (3*): Mixe[04]; Quichean Maya[13, 14]
Used for (5#): Digestive (1)[14]; Musculoskeletal (2)[13, 14]; General and Unspecified (2)[04, 14]
Cognates:
Language contact:

***Peperomia rotundifolia* (L.) Kunth (Piperaceae)**

Spanish names: Hoja de Pescado
Indigenous names: Se'ru Kar [14]
Used by (1*): Quichean Maya[14]
Used for (1#): Skin (1)[14]
Cognates:
Language contact:

***Peperomia* sp. (Piperaceae)**

Spanish names: Tarbatillo
Indigenous names: Boton ts'ohool, wiyab ts'ohool, homte' ts'ohool[07]; Chak B'olay Q'ehen, Ik B'olay pim, Xwa Ixul[14]; Xcua'aj aw chan[16]
Used by (4*): Huastec[07]; Quichean Maya[14, 16]; Nahua[25]
Used for (7#): Neurological (2)[07, 16]; Psychological (1)[16]; Respiratory (1)[25]; Skin (1)[14]; General and Unspecified (2)[07, 14]
Cognates: Quich: xua;
Language contact:

***Peperomia tetraphylla* (G. Fost.) Hook. & Arn. (Piperaceae)**

Spanish names:
Indigenous names:
Used by (1*): Zoque[03]
Used for (1#): Musculoskeletal (1)[03]
Cognates:
Language contact:

***Pereskia grandiflora* Pfeiff. (Cactaceae)**

Spanish names:
Indigenous names: Pulik kwi'inal[07]
Used by (1*): Huastec[07]
Used for (2#): Digestive (1)[07]; Musculoskeletal (1)[07]
Cognates:
Language contact:

***Pereskiopsis aquosa* (F.A.C.Weber) Britton & Rose (Cactaceae)**

Spanish names:
Indigenous names: Kweteem kwi'inal[07]
Used by (1*): Huastec[07]
Used for (3#): Digestive (1)[07]; Respiratory (1)[07]; General and Unspecified (1)[07]
Cognates:
Language contact:

***Persea americana* Mill. (Lauraceae)**

Spanish names: Aguacate
Indigenous names: Owi / Kuytäm / Kuytäp [01]; Kuy täm[02]; Cuy ty¥m[03]; Kuit[04]; Kuka'taj[05]; Cutacaj[06]; Uh, oh[07]; Uj[08]; On[09]; On[10]; Oj[12]; Oj[13]; Um[18]; Un[19]; On[20]; Yeexu'u[21]; Yàg-ngùd-guièx[23]; Aguacate[25]
Used by (21*): Zoque[01, 02, 03]; Mixe[04]; Totonac[05, 06]; Huastec[07, 08]; Yucatecan Maya[09, 10]; Quichean Maya[12, 13, 14]; Western Maya[18, 19, 20]; Zapotec[21, 23]; Nahua[24, 25, 26]
Used for (88#): Blood (2)[05, 12]; Digestive (13)[01, 02, 03, 04, 06, 07, 08, 13, 18, 19, 20, 21, 25]; Eye (1)[21]; Cardiovascular (5)[01, 02, 08, 18, 21]; Musculoskeletal (8)[01, 02, 03, 06, 08, 12, 13, 21]; Neurological (3)[03, 08, 12]; Psychological (3)[01, 12, 18]; Respiratory (10)[02, 05, 07, 08, 09, 10, 13, 19, 25, 26]; Skin (10)[01, 02, 03, 06, 07, 08, 13, 18, 21, 25]; Endocrine (5)[01, 03, 09, 12, 18]; Urological (3)[01, 09, 12]; Pregnancy (8)[02, 04, 12, 13, 14, 19, 21, 23]; Female genital (5)[01, 02, 03, 19, 24]; Male genital (3)[01, 03, 18]; General and Unspecified (9)[01, 05, 07, 08, 12, 14, 21, 25, 25]
Cognates: MZ: kuit; Zoq: kuitäm; Toto: kukataj; Mayan: on/un/oj/uj; Huas: uh/uj; Yuca: on; Quich: oj; WesM: un/on; Nahua: aguacatl;
Language contact: MZ > Tot; Maya > Chiapas Zoq; Nah > Spanish

**_Persea schiedeana_ Nees (Lauraceae)**

Spanish names: Pagua
Indigenous names: Xl'pu[06]
Used by (1*): Totonac[06]
Used for (3#): Cardiovascular (1)[06]; Psychological (1)[06]; Pregnancy (1)[06]
Cognates:
Language contact:

**_Persicaria glabra_ (Willd.) M.Gómez (Polygonaceae)**

Spanish names: Nueve Embarazo ; Pimienta de Agua
Indigenous names: Rukotz'ij chab'äk[12]; Beleeb' Q'ehen[14]
Used by (2*): Quichean Maya[12, 14]
Used for (6#): Digestive (1)[12]; Musculoskeletal (1)[14]; Urological (1)[12]; Pregnancy (1)[14]; General and Unspecified (2)[12, 14]
Cognates:
Language contact:

**_Persicaria hydropiperoides_ (Michx.) Small (Polygonaceae)**

Spanish names:
Indigenous names: Nø mo 'unts ujts[04]
Used by (1*): Mixe[04]
Used for (1#): Skin (1)[04]
Cognates:
Language contact:

**_Persicaria punctata_ (Elliot) Small (Polygonaceae)**

Spanish names: Hoja de azar
Indigenous names:
Used by (1*): Zoque[01]
Used for (1#): General and Unspecified (1)[01]
Cognates:
Language contact:

**_Peteravenia schultzii_ (Schnittsp.) R.M.King & H.Rob. (Asteraceae)**

Spanish names: Hierba de fiebre
Indigenous names: Nekx cuy[03]
Used by (2*): Zoque[01, 03]
Used for (5#): Digestive (1)[01]; Musculoskeletal (1)[01]; Skin (1)[03]; Female genital (1)[01]; General and Unspecified (1)[01]
Cognates:
Language contact:

**_Petiveria alliacea_ L. (Phytolaccaceae)**

Spanish names: Hierba de zorrillo, apacín
Indigenous names: Wujpa ay/pats rane/ pats ay[01]; Patz ay/paks ay/ pats a'watz/jaka kätzok[02]; Patscang ay[03]; Paj ujts[04]; Pathaam, path ts'ohool, pathaam uut'[07]; Pasim[11]; Paara Q'ehen[14]; Par'i'pim[16]; Tujen a'uch[18]; Bete'a[21]
Used by (13*): Zoque[01, 02, 03]; Mixe[04]; Totonac[05]; Huastec[07]; Yucatecan Maya[11]; Quichean Maya[12, 14, 16]; Western Maya[18, 19]; Zapotec[21]
Used for (44#): Digestive (7)[01, 02, 03, 11, 14, 19, 21]; Musculoskeletal (7)[01, 02, 03, 07, 14, 18, 21]; Neurological (5)[02, 07, 11, 14, 16]; Psychological (1)[16]; Respiratory (5)[01, 02, 04, 12, 21]; Skin (5)[03, 05, 07, 14, 21]; Endocrine (1)[07]; Urological (2)[01, 14]; Pregnancy (1)[19]; Female genital (1)[19]; General and Unspecified (9)[01, 02, 03, 05, 07, 12, 14, 18, 21]
Cognates: MZ: paC; Zoq: pats; Mayan: paC; Quich: par;
Language contact: MZ > Maya and Zap

***Petrea volubilis*** **L. (Verbenaceae)**

Spanish names: Comida de caballo; Flor de Nazareno; Raspasombrero
Indigenous names: Thathup tsâah, paaskwa wits, wayelom ts'ohool, kothow ch'aah[07]; Lat'ax xumplelab[08]; Yochop'tsimin[09]
Used by (4*): Huastec[07, 08]; Yucatecan Maya[09]; Quichean Maya[12]
Used for (13#): Digestive (2)[08, 09]; Musculoskeletal (1)[08]; Neurological (1)[07]; Psychological (1)[07]; Respiratory (1)[07]; Skin (2)[07, 12]; Urological (1)[08]; Pregnancy (1)[07]; Female genital (1)[07]; General and Unspecified (2)[07, 12]
Cognates:
Language contact:

***Petroselinum crispum*** **(Mill.) Fuss (Apiaceae)**

Spanish names: Perejil
Indigenous names: Parsil[13]
Used by (3*): Totonac[06]; Quichean Maya[13]; Zapotec[23]
Used for (9#): Digestive (1)[06]; Cardiovascular (1)[06]; Neurological (1)[23]; Respiratory (1)[23]; Pregnancy (1)[13]; Female genital (1)[06]; Male genital (1)[13]; General and Unspecified (2)[06, 23]
Cognates:
Language contact:

***Peumus boldus*** **Molina (Monimiaceae)**

Spanish names: Boldo
Indigenous names:
Used by (2*): Quichean Maya[12]; Zapotec[21]
Used for (7#): Blood (1)[12]; Digestive (2)[12, 21]; Psychological (1)[12]; Skin (1)[12]; Urological (1)[12]; General and Unspecified (1)[12]
Cognates:
Language contact:

***Phacelia platycarpa*** **(Cav.) Spreng. (Boraginaceae)**

Spanish names: Milenrama de Agua
Indigenous names: Aq'om Kumatzin Q'os ya'[12]
Used by (1*): Quichean Maya[12]
Used for (4#): Digestive (1)[12]; Cardiovascular (1)[12]; Musculoskeletal (1)[12]; Psychological (1)[12]
Cognates:
Language contact:

***Phalaris canariensis*** **L. (Poaceae)**

Spanish names: Alpiste
Indigenous names:
Used by (2*): Zoque[02]; Quichean Maya[12]
Used for (4#): Digestive (1)[12]; Cardiovascular (1)[02]; Skin (1)[12]; General and Unspecified (1)[12]
Cognates:
Language contact:

***Phaseolus*** **sp. (Fabaceae)**

Spanish names: Frijol
Indigenous names: S¥k[03]; Blǎ-dǐp, dzè-díp, bziàa-díp[23]
Used by (2*): Zoque[03]; Zapotec[23]
Used for (2#): Skin (1)[23]; Pregnancy (1)[03]
Cognates:
Language contact:

[01-28] refer to the study codes in Table 4.1. *Total number of studies citing this taxon #Total number of use-records

***Phaseolus vulgaris* L. (Fabaceae)**

Spanish names: Frijol; Frijol blanco; Frijol negro
Indigenous names: Popo säk[01]; Stapu[06]; Tsanakw', chanakw'[07]; Ik'ik bu'u[18]; Bizza'a[21]
Used by (5*): Zoque[01]; Totonac[06]; Huastec[07]; Western Maya[18]; Zapotec[21]
Used for (7[#]): Blood (2)[18, 21]; Digestive (1)[07]; Skin (2)[06, 21]; Pregnancy (1)[01]; General and Unspecified (1)[21]
Cognates:
Language contact: Tot <> Chontal; Chiapas Zoq <> Hua

***Phenax hirtus* (Sw.) Wedd. (Urticaceae)**

Spanish names: Gordoncillo
Indigenous names:
Used by (1*): Quichean Maya[13]
Used for (1[#]): Digestive (1)[13]
Cognates:
Language contact:

***Philodendron guttiferum* Kunth (Araceae)**

Spanish names: Bejuco Negro
Indigenous names: Saq'i Jolol[14]
Used by (1*): Quichean Maya[14]
Used for (4[#]): Cardiovascular (1)[14]; Musculoskeletal (1)[14]; Neurological (1)[14]; Skin (1)[14]
Cognates:
Language contact:

***Philodendron hederaceum* (Jacq.) Schott (Araceae)**

Spanish names: Chapiz
Indigenous names: Pasmuj ay[03]
Used by (3*): Zoque[03]; Totonac[05]; Yucatecan Maya[09]
Used for (4[#]): Musculoskeletal (1)[03]; Skin (3)[03, 05, 09]
Cognates:
Language contact:

***Philodendron inaequilaterum* Liebm. (Araceae)**

Spanish names:
Indigenous names: Marina ay[03]
Used by (1*): Zoque[03]
Used for (1[#]): Skin (1)[03]
Cognates:
Language contact:

***Philodendron radiatum* Schott (Araceae)**

Spanish names:
Indigenous names: Mututs[03]
Used by (1*): Zoque[03]
Used for (1[#]): Eye (1)[03]
Cognates:
Language contact:

***Philodendron sagittifolium* Liebm. (Araceae)**

Spanish names: Chapiz grande, malaste grande
Indigenous names: Tantai[05]
Used by (1*): Totonac[05]
Used for (1[#]): Eye (1)[05]
Cognates:
Language contact:

[01-28]refer to the study codes in Table 4.1. *Total number of studies citing this taxon [#]Total number of use-records

***Philodendron smithii* Engl. (Araceae)**

Spanish names:
Indigenous names: Toy'patek ay[02]
Used by (1*): Zoque[02]
Used for (1#): Eye (1)[02]
Cognates:
Language contact:

***Philodendron* sp. (Araceae)**

Spanish names:
Indigenous names: Ma'raq'[16]; Par'i'pim[16]; Rubelsa'i'xul[16]
Used by (1*): Quichean Maya[16]
Used for (4#): Neurological (3)[16, 16, 16]; Psychological (1)[16]
Cognates:
Language contact:

***Philodendron tripartitum* (Jacq.) Schott (Araceae)**

Spanish names:
Indigenous names:
Used by (1*): Zoque[03]
Used for (1#): Musculoskeletal (1)[03]
Cognates:
Language contact:

***Phlebodium areolatum* (Humb. & Bonpl. ex Willd.) J. Sm. (Polypodiaceae)**

Spanish names: Calagual, canaguala; Calaguala/Hierba de golpe
Indigenous names: Misyu mätzyik[01]; Bàz, guìzh-bàz[23]
Used by (2*): Zoque[01]; Zapotec[23]
Used for (9#): Digestive (2)[01, 23]; Musculoskeletal (1)[01]; Respiratory (2)[01, 23]; Endocrine (1)[01]; Female genital (1)[01]; Male genital (1)[01]; General and Unspecified (1)[23]
Cognates:
Language contact:

***Phlebodium aureum* (L.) J. Sm. (Polypodiaceae)**

Spanish names: Calaguala; Calahuala; Costilla de león; Lengua de ciervo
Indigenous names: Ts'een k'ubak koy, talab ik,bo' waak[07]; Tehualcachitihuitl[25]
Used by (5*): Totonac[05]; Huastec[07]; Quichean Maya[13]; Western Maya[18]; Nahua[25]
Used for (12#): Digestive (1)[13]; Cardiovascular (1)[25]; Musculoskeletal (4)[05, 13, 18, 25]; Respiratory (1)[07]; Skin (1)[18]; Endocrine (1)[13]; Pregnancy (1)[07]; General and Unspecified (2)[07, 13]
Cognates:
Language contact:

***Phlebodium decumanum* (Willd.) J. Sm. (Polypodiaceae)**

Spanish names:
Indigenous names: Ch'upil Q'en[14]
Used by (1*): Quichean Maya[14]
Used for (1#): Neurological (1)[14]
Cognates:
Language contact:

***Phlebodium pseudoaureum*** **(Cav.) Lellinger (Polypodiaceae)**

Spanish names: Calaguala; Calahuala
Indigenous names: Ruxe' Kalaguala Cha'at[12]; Lorom, u kab’ mis[19]
Used by (2*): Quichean Maya[12]; Western Maya[19]
Used for (4#): Digestive (1)[12]; Pregnancy (1)[19]; Female genital (1)[19]; General and Unspecified (1)[12]
Cognates:
Language contact:

***Phlebodium*** **sp. (Polypodiaceae)**

Spanish names:
Indigenous names: K'ubak koy, akan koy, t'ot ts'ohool, k'ubak mitsu', akan wahuts, k'ubak pathaam[07]
Used by (1*): Huastec[07]
Used for (4#): Respiratory (1)[07]; Skin (1)[07]; Pregnancy (1)[07]; General and Unspecified (1)[07]
Cognates:
Language contact:

***Phoradendron carneum*** **Urb. (Santalaceae)**

Spanish names: Mata palo
Indigenous names: Shaguii nagitzi'i[21]
Used by (1*): Zapotec[21]
Used for (3#): Musculoskeletal (1)[21]; Skin (1)[21]; General and Unspecified (1)[21]
Cognates:
Language contact:

***Phoradendron lanceolatum*** **Engelm. ex A.Gray (Santalaceae)**

Spanish names: Hierba amarilla
Indigenous names:
Used by (1*): Nahua[26]
Used for (1#): Endocrine (1)[26]
Cognates:
Language contact:

***Phoradendron piperoides*** **(Kunth) Trel. (Santalaceae)**

Spanish names:
Indigenous names: Palek aay[04]
Used by (1*): Mixe[04]
Used for (1#): Skin (1)[04]
Cognates:
Language contact:

***Phoradendron quadrangulare*** **(Kunth) Griseb. (Santalaceae)**

Spanish names: Cabellera; Muérdago; Muerdago, mata palo
Indigenous names: Cuyñukxi[03]; Ok'lom te' yexu'[07]; Tzara'[12]; Wik bak[13]
Used by (4*): Zoque[03]; Huastec[07]; Quichean Maya[12, 13]
Used for (7#): Digestive (1)[12]; Cardiovascular (1)[12]; Musculoskeletal (1)[13]; Neurological (1)[07]; Psychological (1)[12]; Skin (1)[03]; General and Unspecified (1)[03]
Cognates:
Language contact:

***Phoradendron robinsonii*** **Urb. (Santalaceae)**

Spanish names: Mundago/presta palo
Indigenous names: Kuy yakspa[01]
Used by (1*): Zoque[01]
Used for (1#): Cardiovascular (1)[01]
Cognates:
Language contact:

[01-28]refer to the study codes in Table 4.1. *Total number of studies citing this taxon #Total number of use-records

***Phoradendron tonduzii*** **Trel. (Santalaceae)**

Spanish names: Muérdago
Indigenous names: Tzara' geka[12]
Used by (1*): Quichean Maya[12]
Used for (3#): Cardiovascular (1)[12]; Skin (1)[12]; General and Unspecified (1)[12]
Cognates:
Language contact:

***Phragmites australis*** **(Cav.) Trin. ex Steud. (Poaceae)**

Spanish names: Caña de castilla
Indigenous names:
Used by (1*): Quichean Maya[13]
Used for (1#): Urological (1)[13]
Cognates:
Language contact:

***Phyla scaberrima*** **(Juss. ex Pers.) Moldenke (Verbenaceae)**

Spanish names: Hierba dulce, orozus
Indigenous names: Kanak pa'ak [01]; Cana ay[03]; Pa'ak ujts[04]; Sak'si'tuwan[05]; Tsi'iimal koy, chi'ik ch'ohool[07]; Orozús[13]; Q'iil pim [14]; Bänälä tzaj[18]; Guixa'a na'axii[21]
Used by (17*): Zoque[01, 02, 03]; Mixe[04]; Totonac[05]; Huastec[07]; Yucatecan Maya[09]; Quichean Maya[12, 13, 14, 17]; Western Maya[18, 19]; Zapotec[21, 22]; Nahua[25, 26]
Used for (40#): Digestive (10)[01, 02, 03, 05, 07, 09, 21, 22, 25, 26]; Neurological (2)[03, 13]; Respiratory (11)[01, 02, 03, 04, 05, 07, 12, 13, 18, 19, 21]; Skin (2)[07, 14]; Urological (2)[03, 12]; Pregnancy (2)[03, 26]; Female genital (3)[03, 05, 26]; Male genital (1)[12]; General and Unspecified (6)[07, 09, 12, 13, 14, 21]; nd[17]
Cognates: MZ: pa'ak; Zoq: kana;
Language contact:

***Phyla stoechadifolia*** **(L.) Small (Verbenaceae)**

Spanish names: Té de china
Indigenous names:
Used by (1*): Yucatecan Maya[09]
Used for (1#): Digestive (1)[09]
Cognates:
Language contact:

***Phyllanthus acuminatus*** **Vahl (Phyllanthaceae)**

Spanish names:
Indigenous names: Xulimil[09]
Used by (1*): Yucatecan Maya[09]
Used for (1#): Skin (1)[09]
Cognates:
Language contact:

***Phyllanthus adenodiscus*** **Müll.Arg. (Phyllanthaceae)**

Spanish names:
Indigenous names: Pok' thoot[07]; P'ix'ton-ak'[09]
Used by (2*): Huastec[07]; Yucatecan Maya[09]
Used for (5#): Eye (1)[07]; Neurological (1)[07]; Skin (1)[07]; Urological (1)[09]; General and Unspecified (1)[07]
Cognates:
Language contact:

***Phyllanthus graveolens* Kunth (Phyllanthaceae)**

Spanish names:
Indigenous names: Pets'k'mi, Kambaikiche'[09]
Used by (1*): Yucatecan Maya[09]
Used for (3#): Neurological (1)[09]; Skin (1)[09]; Female genital (1)[09]
Cognates:
Language contact:

***Phyllanthus hyssopifolioides* Kunth (Phyllanthaceae)**

Spanish names: Mal de ojo
Indigenous names:
Used by (1*): Nahua[26]
Used for (1#): Skin (1)[26]
Cognates:
Language contact:

***Phyllanthus niruri* L. (Phyllanthaceae)**

Spanish names: Hierba de piedrita/ quebra piedras/ rompepiedra; Hierba del gusano
Indigenous names: Poka rane [01]; Kux ichiich, kital ichiich, waleklaab ichiich[07]; Tleocuilpactle[26]
Used by (3*): Zoque[01]; Huastec[07]; Nahua[26]
Used for (6#): Digestive (1)[07]; Skin (3)[01, 07, 26]; Urological (1)[01]; General and Unspecified (1)[07]
Cognates:
Language contact: Chiapas Zoq <> Nah

***Phyllanthus* sp. (Phyllanthaceae)**

Spanish names: Zapote negro
Indigenous names: Antuñik¥ ay[03]; P'ix'tonche'[09]; Tliltzapotl[26]; Axocopa[26]
Used by (3*): Zoque[03]; Yucatecan Maya[09]; Nahua[26]
Used for (6#): Digestive (1)[03]; Psychological (1)[03]; Skin (2)[09, 26]; Pregnancy (1)[26]; General and Unspecified (1)[03]
Cognates:
Language contact:

***Phymosia umbellata* (Cav.) Kearney (Malvaceae)**

Spanish names:
Indigenous names: Tsak kwiniimte'[07]
Used by (1*): Huastec[07]
Used for (1#): Digestive (1)[07]
Cognates:
Language contact:

***Physalis coztomatl* Dunal (Solanaceae)**

Spanish names: Costomate
Indigenous names: Tudheyil t'ot[08]
Used by (1*): Huastec[08]
Used for (2#): Digestive (1)[08]; Respiratory (1)[08]
Cognates:
Language contact:

***Physalis gracilis* Miers (Solanaceae)**

Spanish names: Chap'ulul; Tomatillo
Indigenous names: Koya tzäpe/ täksy kuy ay/ toksy koyak [01]; Chap'ulul[06]; Tuthaayil an t'ot[07]
Used by (3*): Zoque[01]; Totonac[06]; Huastec[07]
Used for (8#): Digestive (3)[01, 06, 07]; Cardiovascular (1)[06]; Neurological (1)[01]; Respiratory (1)[01]; Skin (1)[07]; Urological (1)[06]
Cognates:
Language contact:

[01-28] refer to the study codes in Table 4.1. *Total number of studies citing this taxon #Total number of use-records

**_Physalis ixocarpa_ Brot. ex Hornem. (Solanaceae)**

Spanish names: Tomatillo
Indigenous names: Chap'ulul[06]
Used by (1*): Totonac[06]
Used for (3#): Digestive (1)[06]; Respiratory (1)[06]; Skin (1)[06]
Cognates:
Language contact:

**_Physalis melanocystis_ (B.L.Rob.) Bitter (Solanaceae)**

Spanish names:
Indigenous names: Akal k'ak'al ilaal, palat ichiich, tuthay te', walul ch'ohool[07]
Used by (1*): Huastec[07]
Used for (2#): Ear (1)[07]; General and Unspecified (1)[07]
Cognates:
Language contact:

**_Physalis philadelphica_ Lam. (Solanaceae)**

Spanish names: Miltomate
Indigenous names:
Used by (1*): Quichean Maya[12]
Used for (2#): Skin (1)[12]; General and Unspecified (1)[12]
Cognates:
Language contact:

**_Physalis pubescens_ L. (Solanaceae)**

Spanish names:
Indigenous names: Naka txipiñ[03]; Top tuČ[10]
Used by (2*): Zoque[03]; Yucatecan Maya[10]
Used for (3#): Respiratory (1)[03]; Skin (2)[03, 10]
Cognates:
Language contact:

**_Physalis_ sp. (Solanaceae)**

Spanish names: Miltomate; Tomate de lombriz; Tomate verde
Indigenous names: Joq pix[13]; Pe'ych k'opot[19]; Bityuš gihš[22]; Yàg-pchŭux-làs, pchŭux-làs[23]
Used by (5*): Quichean Maya[13]; Western Maya[19]; Zapotec[22, 23]; Nahua[27]
Used for (8#): Digestive (3)[13, 19, 22]; Respiratory (2)[13, 27]; Skin (2)[13, 23]; General and Unspecified (1)[22]
Cognates: CoreM: pix; Zapo: pxux;
Language contact: CoreM <> Zap

**_Physalis virginiana_ Mill. (Solanaceae)**

Spanish names:
Indigenous names: Tuthaayil an t'ot[07]
Used by (1*): Huastec[07]
Used for (2#): Digestive (1)[07]; Skin (1)[07]
Cognates:
Language contact:

**_Physalis viscosa_ L. (Solanaceae)**

Spanish names:
Indigenous names: Tuthaayil an t'ot[07]
Used by (1*): Huastec[07]
Used for (2#): Digestive (1)[07]; Skin (1)[07]
Cognates:
Language contact:

[01-28]refer to the study codes in Table 4.1. *Total number of studies citing this taxon #Total number of use-records

***Phytolacca icosandra* L. (Phytolaccaceae)**

Spanish names: Jaboncillo
Indigenous names: T'eikox[09]; Saltz'i' o Pek tz'i, Sa'l Tz'i Q'os[12]
Used by (2*): Yucatecan Maya[09]; Quichean Maya[12]
Used for (5[#]): Digestive (1)[12]; Skin (1)[12]; Urological (1)[12]; General and Unspecified (2)[09, 12]
Cognates:
Language contact:

***Phytolacca rivinoides* Kunth & C.D.Bouché (Phytolaccaceae)**

Spanish names: Hierba blanca, pie de palom tierno
Indigenous names: Tzakan[01]; Tzakanak[02]; Masan ay[03]
Used by (3*): Zoque[01, 02, 03]
Used for (4[#]): Skin (3)[01, 02, 03]; General and Unspecified (1)[02]
Cognates: Zoq: tzakan;
Language contact:

***Phytolacca* sp. (Phytolaccaceae)**

Spanish names: Jaboncillo
Indigenous names: Retz'e, raq'tze[13]
Used by (1*): Quichean Maya[13]
Used for (2[#]): Blood (1)[13]; Skin (1)[13]
Cognates:
Language contact:

***Picramnia antidesma* Sw. (Picramniaceae)**

Spanish names:
Indigenous names: Thal te', k'inim te', thak oliy, tsakam it'il[07]
Used by (2*): Zoque[03]; Huastec[07]
Used for (2[#]): Skin (2)[03, 07]
Cognates:
Language contact:

***Picramnia hirsuta* W. Thomas (Picramniaceae)**

Spanish names:
Indigenous names: J¥mniom petx maka[03]
Used by (1*): Zoque[03]
Used for (1[#]): Skin (1)[03]
Cognates:
Language contact:

***Picramnia teapensis* Tul. (Picramniaceae)**

Spanish names:
Indigenous names: Petx maka[03]
Used by (1*): Zoque[03]
Used for (1[#]): Skin (1)[03]
Cognates:
Language contact:

***Pilea hyalina* Fenzl (Urticaceae)**

Spanish names: Hoja de azar de hierbabuena
Indigenous names: Tza'a tzoy[01]; Txiñ txay sotyi[03]
Used by (2*): Zoque[01, 03]
Used for (2[#]): Skin (1)[03]; General and Unspecified (1)[01]
Cognates:
Language contact: Chiapas Zoq <> Highland Popoluca

[01-28]refer to the study codes in Table 4.1. *Total number of studies citing this taxon [#]Total number of use-records

***Pilea irrorata* Donn. Sm. (Urticaceae)**

Spanish names:
Indigenous names: Kiñi ay[03]
Used by (1*): Zoque[03]
Used for (2#): Ear (1)[03]; Musculoskeletal (1)[03]
Cognates:
Language contact:

***Pilea microphylla* (L.) Liebm. (Urticaceae)**

Spanish names: Espumilla; Hoja de azar
Indigenous names: Tza'a tzoy[01]; Ha'il tsan, tumiin ts'ohool, ohil tsan, pitsits wal[07]
Used by (4*): Zoque[01, 03]; Huastec[07]; Yucatecan Maya[11]
Used for (8#): Eye (1)[07]; Neurological (1)[07]; Respiratory (1)[11]; Skin (1)[07]; Urological (1)[11]; General and Unspecified (3)[01, 03, 07]
Cognates:
Language contact: Chiapas Zoq <> Hua

***Pilea pubescens* Liebm. (Urticaceae)**

Spanish names:
Indigenous names: Pux lat'em, kux laatem[07]; Ox eek'il Q'ehen[14]
Used by (2*): Huastec[07]; Quichean Maya[14]
Used for (6#): Eye (1)[07]; Neurological (1)[07]; Respiratory (1)[07]; Pregnancy (1)[07]; General and Unspecified (2)[07, 14]
Cognates:
Language contact:

***Pilea* sp. (Urticaceae)**

Spanish names:
Indigenous names: Tsakam tsahib[07]
Used by (1*): Huastec[07]
Used for (1#): General and Unspecified (1)[07]
Cognates:
Language contact:

***Pilocarpus racemosus* Vahl (Rutaceae)**

Spanish names:
Indigenous names: Tamkasche', Siische'[09]
Used by (1*): Yucatecan Maya[09]
Used for (3#): Digestive (1)[09]; Respiratory (1)[09]; General and Unspecified (1)[09]
Cognates:
Language contact:

***Pimenta dioica* (L.) Merr. (Myrtaceae)**

Spanish names: Pimienta gorda
Indigenous names: Moki[01]; Moke[02]; Uk suk[03]; U'cum[06]; Nohochpol[09]; Naba kook[10]; Peen's[14]; Xuxpat[18]; Pimient rooj[21]
Used by (15*): Zoque[01, 02, 03]; Mixe[04]; Totonac[05, 06]; Yucatecan Maya[09, 10, 11]; Quichean Maya[12, 13, 14]; Western Maya[18, 19]; Zapotec[21]
Used for (55#): Digestive (12)[01, 02, 03, 05, 06, 09, 10, 11, 12, 13, 18, 21]; Ear (1)[01]; Cardiovascular (1)[02]; Musculoskeletal (5)[02, 06, 12, 18, 21]; Neurological (3)[01, 02, 18]; Psychological (1)[02]; Respiratory (5)[01, 02, 03, 13, 18]; Skin (4)[01, 03, 18, 21]; Urological (1)[18]; Pregnancy (8)[01, 02, 06, 09, 13, 14, 19, 21]; Female genital (8)[01, 02, 03, 05, 09, 11, 19, 21]; General and Unspecified (6)[01, 02, 04, 12, 13, 21]
Cognates: Zoq: moke;
Language contact: Zoq > Tot

[01-28] refer to the study codes in Table 4.1. *Total number of studies citing this taxon #Total number of use-records

***Pimpinella anisum* L. (Apiaceae)**

Spanish names: Anís
Indigenous names: Anix[13]
Used by (6*): Zoque[02]; Totonac[06]; Yucatecan Maya[09]; Quichean Maya[12, 13]; Western Maya[19]
Used for (24#): Blood (1)[19]; Digestive (5)[02, 06, 09, 13, 19]; Musculoskeletal (2)[02, 12]; Neurological (1)[12]; Psychological (3)[02, 12, 13]; Endocrine (1)[02]; Urological (1)[12]; Pregnancy (5)[06, 09, 12, 13, 19]; Female genital (3)[02, 12, 13]; General and Unspecified (2)[12, 13]
Cognates:
Language contact:

***Pinaropappus roseus* (Less.) Less. (Asteraceae)**

Spanish names: Espule, espulga
Indigenous names: Guièe-mòrâd, guìzh-mòrâd, guìzh-nǐdz, ngùd-nǐdz,  guìzh-rziòob], guìzh-guiět-nì[23]
Used by (2*): Zapotec[22, 23]
Used for (3#): Eye (1)[23]; Skin (2)[22, 23]
Cognates:
Language contact:

***Pinguicula moranensis* Kunth (Lentibulariaceae)**

Spanish names: Trebol de Montaña
Indigenous names: Keq Treblo Aq'om[12]
Used by (1*): Quichean Maya[12]
Used for (7#): Blood (1)[12]; Digestive (1)[12]; Neurological (1)[12]; Psychological (1)[12]; Respiratory (1)[12]; Skin (1)[12]; General and Unspecified (1)[12]
Cognates:
Language contact:

***Pinguicula* sp. (Lentibulariaceae)**

Spanish names:
Indigenous names: Diàg-cûch,  guièe-ngùrùdz, guièe-yòob-chèn[23]
Used by (1*): Zapotec[23]
Used for (1#): Digestive (1)[23]
Cognates:
Language contact:

***Pinus oocarpa* Schiede (Pinaceae)**

Spanish names: Ocote; Pino
Indigenous names: Tzin[02]; Tyiñcuy[03]; Tsiin[04]; Tajte'[19]; Guiere'ej[21]
Used by (5*): Zoque[02, 03]; Mixe[04]; Western Maya[19]; Zapotec[21]
Used for (20#): Digestive (2)[02, 21]; Cardiovascular (1)[02]; Musculoskeletal (3)[02, 03, 21]; Neurological (2)[02, 19]; Respiratory (5)[02, 03, 04, 19, 21]; Skin (4)[02, 03, 04, 21]; Pregnancy (1)[21]; Female genital (1)[02]; General and Unspecified (1)[21]
Cognates: MZ: tsin;
Language contact: MZ <> Maya

***Pinus pseudostrobus* Lindl. (Pinaceae)**

Spanish names: Pino
Indigenous names: Chäj[12]
Used by (1*): Quichean Maya[12]
Used for (3#): Neurological (1)[12]; Respiratory (1)[12]; General and Unspecified (1)[12]
Cognates:
Language contact:

[01-28]refer to the study codes in Table 4.1. *Total number of studies citing this taxon #Total number of use-records

***Pinus* sp. (Pinaceae)**

Spanish names: Ocote; Ocote (colorado); Pino
Indigenous names: Tzin/tzit/ kon tzit/ tsapas koko'äng tzit[01]; Chäj[12]; Chaj'[13]; Taj, k'an toj[20]; Yàg-guièr[23]
Used by (5*): Zoque[01]; Quichean Maya[12, 13]; Western Maya[20]; Zapotec[23]
Used for (18#): Digestive (2)[01, 20]; Musculoskeletal (3)[01, 12, 13]; Neurological (1)[01]; Psychological (2)[01, 13]; Respiratory (3)[01, 12, 13]; Skin (2)[01, 13]; Pregnancy (1)[01]; General and Unspecified (4)[01, 12, 13, 23]
Cognates:
Language contact:

***Pinus teocote* Schied. ex Schltdl. & Cham. (Pinaceae)**

Spanish names:
Indigenous names: Pithomlaab[07]
Used by (1*): Huastec[07]
Used for (3#): Digestive (1)[07]; Neurological (1)[07]; General and Unspecified (1)[07]
Cognates:
Language contact:

***Piper aduncum* L. (Piperaceae)**

Spanish names: Cordoncillo; Velancitas
Indigenous names: Tunhkuy[01]; Tooso[03]; Thak kw'alal its'aamal[07]; Tdak kw'alal ist'amal[08]
Used by (5*): Zoque[01, 03]; Huastec[07, 08]; Nahua[26]
Used for (9#): Digestive (2)[01, 08]; Neurological (1)[07]; Skin (3)[03, 08, 26]; Pregnancy (1)[07]; General and Unspecified (2)[01, 08]
Cognates: Huas: tak kw'alal its'aamal;
Language contact:

***Piper aequale* Vahl (Piperaceae)**

Spanish names: Planta Barón
Indigenous names: Tooso[03]; Lima Q'ehen, Telom Q'ehen, Saq'i Puchuch [14]
Used by (2*): Zoque[03]; Quichean Maya[14]
Used for (4#): Musculoskeletal (1)[14]; Skin (1)[03]; Pregnancy (1)[14]; General and Unspecified (1)[14]
Cognates:
Language contact:

***Piper amalago* L. (Piperaceae)**

Spanish names: Cordoncillo
Indigenous names: Tsus tooso[03]; Yuk yo'on[04]; Kw'alal its'aamal, yaxal, kw'alal an kwathab, kw'alal i puthuch, yaxal[07]; Xpeheche'[09]; Seq' Ruk'amal Aq'om Itzel Yabil[12]; Q'eq'i Puchuch[14]; Kan pom, pu'chuch[16]; Tzirito' k'ejen[17]; Guiadajna'a[21]
Used by (11*): Zoque[03]; Mixe[04]; Huastec[07]; Yucatecan Maya[09, 10, 11]; Quichean Maya[12, 14, 16, 17]; Zapotec[21]
Used for (24#): Digestive (1)[21]; Cardiovascular (1)[21]; Musculoskeletal (2)[07, 21]; Neurological (4)[07, 10, 11, 16]; Respiratory (2)[07, 21]; Skin (6)[03, 07, 09, 10, 11, 21]; Pregnancy (1)[07]; General and Unspecified (6)[03, 04, 07, 12, 14, 21]; nd[17]
Cognates: Mayan: puCuch/peCech; Quich: puchuch;
Language contact:

***Piper auritum* Kunth (Piperaceae)**

Spanish names: Hierba santa, acuyo, momo, tequelite
Indigenous names: Jaku[01]; Toso/tin ay[02]; Aycuyo[03]; Woo[04]; Jinan[05]; Jina[06]; Xkub' sa' Qana', Hobel[14]; Ob'el[15]; U'bel[16]; Momo[18]; Hua'a[21]; Blàg-guiùu[23]; Tequelite[25]; Tlanecpaquelite[26]; Acuyo xuitl[28]
Used by (18*): Zoque[01, 02, 03]; Mixe[04]; Totonac[05, 06]; Yucatecan Maya[11]; Quichean Maya[12, 14, 15, 16]; Western Maya[18]; Zapotec[21, 23]; Nahua[25, 26, 27, 28]
Used for (61#): Blood (2)[05, 18]; Digestive (9)[01, 02, 05, 11, 18, 21, 23, 25, 28]; Cardiovascular (2)[02, 12]; Musculoskeletal (7)[01, 02, 03, 11, 14, 18, 21]; Neurological (4)[03, 11, 16, 28]; Respiratory (6)[01, 02, 03, 18, 26, 28]; Skin (9)[01, 02, 03, 04, 05, 11, 18, 21, 23]; Endocrine (1)[02]; Urological (3)[01, 02, 18]; Pregnancy (6)[01, 02, 05, 06, 15, 27]; Female genital (4)[02, 14, 15, 18]; Male genital (1)[21]; General and Unspecified (7)[01, 02, 03, 04, 05, 11, 21]
Cognates: Zoq: aku; Toto: jina; Quich: obel;
Language contact: Zoq <> Tot; Nah > Zoq

[01-28]refer to the study codes in Table 4.1. *Total number of studies citing this taxon #Total number of use-records

***Piper guazacapanense* Trel. & Standl. (Piperaceae)**

Spanish names: Cordoncillo oloroso
Indigenous names: Tooso[03]
Used by (1*): Zoque[03]
Used for (3[#]): Digestive (1)[03]; Skin (1)[03]; Female genital (1)[03]
Cognates:
Language contact:

***Piper hispidum* Sw. (Piperaceae)**

Spanish names: Cordoncillo
Indigenous names: Tooso[03]; Sok'ot, xalacuahuit[06]; Tiq'ual Q'ehen[14]; Puchuq[15]; K'an pom che'[17]
Used by (6*): Zoque[03]; Totonac[05, 06]; Quichean Maya[14, 15, 17]
Used for (11[#]): Musculoskeletal (2)[03, 15]; Neurological (1)[14]; Psychological (1)[14]; Skin (1)[03]; Pregnancy (2)[03, 06]; Female genital (1)[15]; General and Unspecified (2)[05, 06]; nd[17]
Cognates:
Language contact:

***Piper jacquemontianum* Kunth (Piperaceae)**

Spanish names: Tabaquillo
Indigenous names: Ruchek k'uch'[12]; Q'ampom, Re Tzuul[14]; Ampom[15]
Used by (3*): Quichean Maya[12, 14, 15]
Used for (11[#]): Blood (1)[15]; Digestive (2)[12, 14]; Musculoskeletal (1)[15]; Neurological (1)[14]; Pregnancy (1)[14]; Female genital (2)[12, 14]; Social problems (1)[14]; General and Unspecified (2)[12, 14]
Cognates: Quich: ampom;
Language contact:

***Piper marginatum* Jacq. (Piperaceae)**

Spanish names:
Indigenous names: Tooso[03]; Puchuch Q'ehen, Kuw Sawi'i, Kux Q'ehen[14]
Used by (2*): Zoque[03]; Quichean Maya[14]
Used for (2[#]): Skin (1)[03]; General and Unspecified (1)[14]
Cognates:
Language contact:

***Piper nigrum* L. (Piperaceae)**

Spanish names: Pimienta negra, pimienta de castilla
Indigenous names: Xuxpat ic'[18]; Ich pimyénta[19]
Used by (6*): Zoque[01, 03]; Western Maya[18, 19, 20]; Zapotec[22]
Used for (10[#]): Digestive (2)[01, 20]; Musculoskeletal (2)[01, 18]; Psychological (1)[03]; Skin (1)[18]; Pregnancy (1)[19]; Female genital (2)[03, 19]; General and Unspecified (1)[22]
Cognates:
Language contact:

***Piper obliquum* Ruiz & Pav. (Piperaceae)**

Spanish names:
Indigenous names: Tooso[03]
Used by (1*): Zoque[03]
Used for (2[#]): Musculoskeletal (1)[03]; Skin (1)[03]
Cognates:
Language contact:

[01-28]refer to the study codes in Table 4.1. *Total number of studies citing this taxon [#]Total number of use-records

***Piper peltatum*** **L. (Piperaceae)**

Spanish names: San Diego
Indigenous names: Yut'it'[14]; Tyut it pim[17]
Used by (3*): Yucatecan Maya[10]; Quichean Maya[14, 17]
Used for (9#): Digestive (2)[10, 14]; Musculoskeletal (1)[14]; Neurological (1)[10]; Skin (1)[14]; Urological (1)[14]; Female genital (1)[14]; General and Unspecified (1)[14]; nd[17]
Cognates: Quich: yut'it;
Language contact:

***Piper pseudofuligineum*** **C.DC. (Piperaceae)**

Spanish names:
Indigenous names: Tzuul, Puchuch Q'ehen[14]
Used by (1*): Quichean Maya[14]
Used for (2#): Neurological (1)[14]; General and Unspecified (1)[14]
Cognates:
Language contact:

***Piper sanctum*** **(Miq.) Schltdl. ex C.DC. (Piperaceae)**

Spanish names: Hoja santa
Indigenous names: Nin qui ru chaq' q'een[15]
Used by (3*): Totonac[05]; Quichean Maya[15]; Nahua[25]
Used for (4#): Digestive (1)[05]; Musculoskeletal (1)[15]; Skin (1)[25]; Pregnancy (1)[15]
Cognates:
Language contact:

***Piper schiedeanum*** **Steud. (Piperaceae)**

Spanish names:
Indigenous names: Paktha' yexal[07]
Used by (2*): Huastec[07]; Quichean Maya[17]
Used for (2#): Digestive (1)[07]; nd[17]
Cognates:
Language contact:

***Piper sempervirens*** **(Trel.) Lundell (Piperaceae)**

Spanish names: Cordoncillo hembra
Indigenous names:
Used by (1*): Yucatecan Maya[11]
Used for (1#): Skin (1)[11]
Cognates:
Language contact:

***Piper*** **sp. (Piperaceae)**

Spanish names: Cordoncillo; Hierba santilla; Hoja/ yerba santa; Momo sylvestre/ haku simarron/ hoja santa simarrona; Tamagás
Indigenous names: Pa' jaku/jiyen jaku/ jaku pa'an/ ji'ne haku[01]; Tunhkuy[01]; Suj[02]; Tooso[03]; Yo'on[04]; Yuk woo[04]; Tiiya', oh ts'ohool[07]; Q'eq'i Puchuch[14]; Puchuch[14]; Tiq'ual Q'ehen[14]; Cux sawi[16]; Pu'chuch[16]; Kan pom[16]; Mai pim[16]; Bala šoh[22]
Used by (11*): Zoque[01, 02, 03]; Mixe[04]; Huastec[07]; Quichean Maya[14, 16]; Western Maya[19]; Zapotec[21, 22]; Nahua[26]
Used for (46#): Digestive (6)[01, 07, 14, 19, 21, 26]; Musculoskeletal (6)[01, 03, 03, 14, 21, 22]; Neurological (5)[16, 16, 16, 16, 22]; Psychological (3)[16, 16, 16]; Respiratory (2)[01, 14]; Skin (7)[01, 03, 03, 03, 04, 22, 26]; Pregnancy (5)[01, 07, 14, 19, 22]; Female genital (3)[14, 19, 21]; General and Unspecified (9)[01, 01, 02, 03, 04, 04, 14, 16, 16]
Cognates:
Language contact:

***Piper tuberculatum* Jacq. (Piperaceae)**

Spanish names: Cordoncillo; Cordoncillo macho, cabeza de guajilote; Pie de pavito
Indigenous names: Ok aj ts'o[18]; Gui'iquimberu'u[21]
Used by (3*): Western Maya[18, 19]; Zapotec[21]
Used for (8#): Digestive (1)[21]; Musculoskeletal (1)[21]; Skin (2)[18, 21]; Pregnancy (1)[19]; Female genital (2)[19, 21]; General and Unspecified (1)[21]
Cognates:
Language contact:

***Piper tuerckheimii* C.DC. (Piperaceae)**

Spanish names: Caite de Diablo
Indigenous names: Kuxtin Q'ehen[14]; Cux sawi k'ejen[17]
Used by (3*): Quichean Maya[14, 15, 17]
Used for (12#): Digestive (1)[14]; Neurological (1)[14]; Psychological (1)[15]; Skin (1)[14]; Urological (1)[14]; Pregnancy (2)[14, 15]; Female genital (1)[14]; Social problems (1)[14]; General and Unspecified (2)[14, 15]; nd[17]
Cognates: Quich: kux keCen;
Language contact:

***Piper uhdei* C. DC. (Piperaceae)**

Spanish names: Cordoncillo
Indigenous names: Aq'om Q'aynaq' Cha'klaj[12]
Used by (1*): Quichean Maya[12]
Used for (4#): Blood (1)[12]; Respiratory (1)[12]; Skin (1)[12]; General and Unspecified (1)[12]
Cognates:
Language contact:

***Piper umbellatum* L. (Piperaceae)**

Spanish names: Acuyo / momo cimarrón
Indigenous names: Aycuyo cimarrón[03]; Bakaanil a iits', bok'ool uxkwe', pamta' an koy, bakanil an miimlaab, pakalaah, tiiya[07]; R'u xac y'a[12]; Momo ajma'[18]; Acoyo[24]
Used by (6*): Zoque[03]; Huastec[07]; Quichean Maya[12]; Western Maya[18]; Nahua[24, 26]
Used for (15#): Digestive (3)[07, 12, 18]; Musculoskeletal (3)[03, 07, 26]; Respiratory (2)[07, 18]; Skin (3)[03, 07, 26]; Pregnancy (2)[07, 24]; General and Unspecified (2)[07, 26]
Cognates:
Language contact:

***Piper unguiculatum* Ruiz & Pav. (Piperaceae)**

Spanish names: Cordoncillo
Indigenous names: Suj ay/syuj ay[02]
Used by (1*): Zoque[02]
Used for (5#): Digestive (1)[02]; Neurological (1)[02]; Psychological (1)[02]; Skin (1)[02]; General and Unspecified (1)[02]
Cognates:
Language contact:

***Piper veraguense* C.DC. (Piperaceae)**

Spanish names:
Indigenous names: Yut'it' puchuch[14]
Used by (1*): Quichean Maya[14]
Used for (5#): Blood (1)[14]; Musculoskeletal (1)[14]; Neurological (1)[14]; Female genital (1)[14]; General and Unspecified (1)[14]
Cognates:
Language contact:

***Piper yucatanense* C.DC. (Piperaceae)**

Spanish names:
Indigenous names: Tzulub pim[17]
Used by (1*): Quichean Maya[17]
Used for (1#): nd[17]
Cognates:
Language contact:

***Piper yzabalanum* C.DC. ex Donn.Sm. (Piperaceae)**

Spanish names: Cordoncillo grande
Indigenous names: Tz'y' Q'ehen[14]; Guiadajna'a rooj[21]
Used by (2*): Quichean Maya[14]; Zapotec[21]
Used for (3#): Musculoskeletal (1)[14]; Neurological (1)[14]; General and Unspecified (1)[21]
Cognates:
Language contact:

***Piptocarpha poeppigiana* (DC.) Baker (Asteraceae)**

Spanish names:
Indigenous names: Chunahak k'ejen[17]
Used by (1*): Quichean Maya[17]
Used for (1#): nd[17]
Cognates:
Language contact:

***Piqueria pilosa* Kunth (Asteraceae)**

Spanish names:
Indigenous names: Guìzh-làs[23]
Used by (1*): Zapotec[23]
Used for (2#): Musculoskeletal (1)[23]; Skin (1)[23]
Cognates:
Language contact:

***Piqueria trinervia* Cav. (Asteraceae)**

Spanish names:
Indigenous names: Bæ̌æl-dòo, guìzh-běæl-dòo, bæ̌æl-dǒ, guìzh-běæl-dǒ[23]
Used by (1*): Zapotec[23]
Used for (4#): Psychological (1)[23]; Skin (1)[23]; Endocrine (1)[23]; General and Unspecified (1)[23]
Cognates:
Language contact:

***Piscidia piscipula* (L.) Sarg. (Fabaceae)**

Spanish names:
Indigenous names: Ts'ihol, k'anaw te', chiihol[07]; Ha'abin[09]
Used by (2*): Huastec[07]; Yucatecan Maya[09]
Used for (7#): Blood (1)[07]; Digestive (2)[07, 09]; Neurological (1)[07]; Respiratory (1)[09]; Skin (1)[07]; General and Unspecified (1)[07]
Cognates:
Language contact:

***Pisonia aculeata* L. (Nyctaginaceae)**

Spanish names: Uña de gato
Indigenous names: Loh, itsik mitsu'[07]; Beeb[09]
Used by (2*): Huastec[07]; Yucatecan Maya[09]
Used for (3#): Skin (1)[07]; Pregnancy (1)[09]; General and Unspecified (1)[07]
Cognates:
Language contact:

[01-28]refer to the study codes in Table 4.1.   *Total number of studies citing this taxon   #Total number of use-records

***Pitcairnia punicea*** **Scheidw. (Bromeliaceae)**

Spanish names: Cebolla de Monte
Indigenous names: Ceboll pim, Seb'oyil pim, Cewoyil pim[14]
Used by (1*): Quichean Maya[14]
Used for (1#): Respiratory (1)[14]
Cognates:
Language contact:

***Pithecellobium albicaule*** **Britton & Rose (Fabaceae)**

Spanish names: Palo de pinolio
Indigenous names: Poposyutil[01]
Used by (1*): Zoque[01]
Used for (2#): Digestive (1)[01]; Neurological (1)[01]
Cognates:
Language contact:

***Pithecellobium dulce*** **(Roxb.) Benth. (Fabaceae)**

Spanish names: Guamúchil
Indigenous names: Umuw, umu[07]; Jumo[08]; Te'aj tuk'uy[18]; Coamochitl[28]
Used by (6*): Zoque[02]; Huastec[07, 08]; Western Maya[18]; Zapotec[21]; Nahua[28]
Used for (15#): Digestive (5)[02, 07, 08, 21, 28]; Musculoskeletal (1)[08]; Neurological (2)[07, 08]; Respiratory (1)[08]; Skin (2)[18, 28]; Female genital (1)[02]; Male genital (1)[02]; General and Unspecified (2)[07, 08]
Cognates: Huas: umu/umo;
Language contact: Hua <> Nah > Spanish

***Pithecellobium lanceolatum*** **(Willd.) Benth. (Fabaceae)**

Spanish names: Guamúchil
Indigenous names:
Used by (1*): Zoque[02]
Used for (5#): Digestive (1)[02]; Cardiovascular (1)[02]; Skin (1)[02]; Pregnancy (1)[02]; Female genital (1)[02]
Cognates:
Language contact:

***Pithecellobium pachypus*** **Pittier (Fabaceae)**

Spanish names:
Indigenous names: So'sol (Xkenq)[14]
Used by (1*): Quichean Maya[14]
Used for (2#): Digestive (1)[14]; General and Unspecified (1)[14]
Cognates:
Language contact:

***Pityopsis graminifolia*** **(Michx.) Nutt. (Asteraceae)**

Spanish names:
Indigenous names: Poja muk[03]
Used by (1*): Zoque[03]
Used for (1#): Skin (1)[03]
Cognates:
Language contact:

[01-28]refer to the study codes in Table 4.1. *Total number of studies citing this taxon #Total number of use-records

***Pityrogramma calomelanos*** **(L.) Link (Pteridaceae)**

Spanish names: Helecho calado
Indigenous names: Jab kingin[01]; Poja chimal [03]; Jam ujts[04]; Roq chit cuan[16]
Used by (4*): Zoque[01, 03]; Mixe[04]; Quichean Maya[16]
Used for (8#): Digestive (1)[01]; Neurological (1)[16]; Respiratory (1)[03]; Skin (1)[03]; Urological (1)[01]; Female genital (1)[04]; Male genital (1)[01]; General and Unspecified (1)[16]
Cognates: MZ: jaC;
Language contact:

***Plantago australis*** **Lam. (Plantaginaceae)**

Spanish names: Lengua de perro/ lengua de vaca; Llantén
Indigenous names: Tu' isy tojtz/ tuwi isy totz/ tu' totz[01]; Lentej ay[03]
Used by (4*): Zoque[01, 03]; Nahua[25, 26]
Used for (11#): Digestive (3)[01, 03, 25]; Eye (1)[01]; Musculoskeletal (1)[01]; Skin (2)[01, 26]; Urological (2)[01, 26]; Female genital (2)[01, 26]
Cognates:
Language contact:

***Plantago major*** **L. (Plantaginaceae)**

Spanish names: Llantén
Indigenous names: Tu' totz[01]; Ojalante'[18]
Used by (6*): Zoque[01]; Totonac[06]; Quichean Maya[12, 14]; Western Maya[18]; Zapotec[21]
Used for (23#): Blood (1)[12]; Digestive (4)[01, 12, 14, 18]; Eye (1)[21]; Cardiovascular (1)[12]; Musculoskeletal (2)[01, 12]; Psychological (1)[12]; Respiratory (2)[01, 18]; Skin (2)[01, 12]; Endocrine (1)[12]; Urological (2)[01, 12]; Female genital (2)[12, 18]; Male genital (1)[01]; General and Unspecified (3)[06, 12, 12]
Cognates:
Language contact:

***Plantago*** **sp. (Plantaginaceae)**

Spanish names: lanté; Llantén; Llantena, santena
Indigenous names: Uxkin imul, jal q'ayes[13]; Blàg-llàntên, blàg-sàntên, guìzh-gòdz, xín-guìzh-dòoz[23]
Used by (3*): Quichean Maya[13]; Zapotec[22, 23]
Used for (6#): Digestive (3)[13, 22, 23]; Eye (1)[13]; Musculoskeletal (1)[13]; Skin (1)[13]
Cognates:
Language contact:

***Platanus mexicana*** **Moric. (Platanaceae)**

Spanish names: Chote; Palo blanco
Indigenous names: Taktak[01]
Used by (2*): Zoque[01]; Nahua[25]
Used for (5#): Ear (1)[25]; Musculoskeletal (1)[01]; Endocrine (1)[25]; Urological (1)[25]; General and Unspecified (1)[01]
Cognates:
Language contact:

***Platycladus orientalis*** **(L.) Franco (Cupressaceae)**

Spanish names: Ciprés
Indigenous names:
Used by (1*): Yucatecan Maya[11]
Used for (1#): Respiratory (1)[11]
Cognates:
Language contact:

***Plectranthus amboinicus* (Lour.) Spreng. (Lamiaceae)**

Spanish names: Oregano; Oregano castillo, oregano chino; Oregano orejón; Oreganón
Indigenous names: Ts'kawe'e[18]
Used by (5*): Zoque[01, 02, 03]; Yucatecan Maya[09]; Western Maya[18]
Used for (18#): Digestive (3)[01, 02, 03]; Eye (1)[03]; Ear (4)[02, 03, 09, 18]; Neurological (2)[01, 18]; Respiratory (3)[01, 03, 18]; Endocrine (1)[02]; Pregnancy (2)[03, 18]; Female genital (1)[01]; General and Unspecified (1)[01]
Cognates:
Language contact:

***Plectranthus scutellarioides* (L.) R.Br. (Lamiaceae)**

Spanish names: Copa de rey; Flor de mantu; Mantu morado/ manto morado
Indigenous names:
Used by (4*): Zoque[01, 02, 03]; Nahua[25]
Used for (6#): Digestive (1)[25]; Ear (1)[03]; Neurological (1)[01]; Urological (1)[02]; General and Unspecified (2)[01, 02]
Cognates:
Language contact:

***Pleopeltis angusta* Humb. & Bonpl. ex Willd. (Polypodiaceae)**

Spanish names: Hierba de Palo con Mano, Calahuala
Indigenous names: K'amal Aqom Che' Qa'aj[12]
Used by (1*): Quichean Maya[12]
Used for (7#): Blood (1)[12]; Cardiovascular (1)[12]; Musculoskeletal (1)[12]; Psychological (1)[12]; Urological (1)[12]; Male genital (1)[12]; General and Unspecified (1)[12]
Cognates:
Language contact:

***Pluchea carolinensis* (Jacq.) D.Don (Asteraceae)**

Spanish names: Hoja Santa María, hoja de canela, siguapate
Indigenous names: Mä'a ome/ mä'a wa'a[01]; Salve ay[03]; Santa Maria aay[04]; Chalche'[09]; Musik witzir[19]; Balagasana, gui'xaan[21]; Chiquite[26]
Used by (8*): Zoque[01, 02, 03]; Mixe[04]; Yucatecan Maya[09]; Western Maya[19]; Zapotec[21]; Nahua[26]
Used for (35#): Digestive (5)[01, 02, 03, 04, 21]; Ear (1)[04]; Cardiovascular (1)[03]; Musculoskeletal (5)[01, 02, 03, 19, 21]; Neurological (2)[01, 19]; Respiratory (1)[03]; Skin (2)[02, 03]; Urological (2)[01, 03]; Pregnancy (6)[01, 02, 03, 09, 19, 21]; Female genital (4)[02, 03, 04, 19]; Male genital (1)[01]; General and Unspecified (5)[01, 02, 03, 19, 26]
Cognates:
Language contact:

***Pluchea odorata* (L.) Cass. (Asteraceae)**

Spanish names: Hierba de canela, siguapate
Indigenous names: K'aninmiin ts'ohool, hom ts'ohool,[07]; Čaal Če[10]; Chalache[11]; Kewuhj[12]; Siguapate[13]; Kwan zahn, kwan gusahn[22]; Guìzh-cànêl[23]
Used by (8*): Huastec[07]; Yucatecan Maya[10, 11]; Quichean Maya[12, 13, 17]; Zapotec[22, 23]
Used for (24#): Digestive (3)[07, 11, 22]; Musculoskeletal (4)[07, 11, 12, 13]; Neurological (4)[07, 11, 12, 13]; Respiratory (1)[07]; Skin (1)[11]; Urological (1)[12]; Pregnancy (5)[07, 10, 12, 22, 23]; General and Unspecified (4)[07, 10, 11, 12]; nd[17]
Cognates: Yuca: chal(a)che;
Language contact:

***Pluchea salicifolia* (Mill.) S.F.Blake (Asteraceae)**

Spanish names: Hoja de aire
Indigenous names: Syaw'ay[02]
Used by (1*): Zoque[02]
Used for (4#): Digestive (1)[02]; Musculoskeletal (1)[02]; Female genital (1)[02]; General and Unspecified (1)[02]
Cognates:
Language contact:

[01-28]refer to the study codes in Table 4.1. *Total number of studies citing this taxon #Total number of use-records

***Pluchea* sp. (Asteraceae)**

Spanish names: Canela del rio
Indigenous names: Mai pim[16]
Used by (2*): Quichean Maya[16]; Zapotec[21]
Used for (2#): Musculoskeletal (1)[21]; Neurological (1)[16]
Cognates:
Language contact:

***Plumbago zeylanica* L. (Plumbaginaceae)**

Spanish names: Hierba del pez; Hoja de chivato
Indigenous names: Hurika[07]; Hurika[08]; Guish chivat[21]; Guìzh-nàad, guìzh-mòràdító[23]
Used by (4*): Huastec[07, 08]; Zapotec[21, 23]
Used for (7#): Digestive (1)[23]; Musculoskeletal (1)[07]; Skin (3)[07, 08, 21]; General and Unspecified (2)[07, 08]
Cognates: Huas: hurika; Zapo: guish at;
Language contact:

***Plumeria rubra* L. (Apocynaceae)**

Spanish names: Flor de mayo
Indigenous names: (Tzama) popo jäyä[01]; Puutx mooya[03]; San Juan pøh[04]; Caxta xanat[06]; Ukul wits[07]; Guiatzatzii quitii[21]; Yàg-guièe-yăl[23]; Cacaloxochitl[24]; Cacahuaxóchitl[25]
Used by (9*): Zoque[01, 03]; Mixe[04]; Totonac[06]; Huastec[07]; Zapotec[21, 23]; Nahua[24, 25]
Used for (22#): Digestive (5)[03, 04, 06, 23, 25]; Eye (1)[23]; Musculoskeletal (3)[01, 04, 21]; Neurological (1)[03]; Respiratory (1)[21]; Skin (4)[01, 07, 21, 24]; Urological (1)[01]; Female genital (3)[01, 06, 21]; General and Unspecified (3)[03, 04, 21]
Cognates: Nahua: cacalo/cacahua xochitl;
Language contact: Tot <> Nah

***Plumeria* sp. (Apocynaceae)**

Spanish names: Flor de mayo
Indigenous names: Nikte'ch'om[09]
Used by (1*): Yucatecan Maya[09]
Used for (1#): Skin (1)[09]
Cognates:
Language contact:

***Pogostemon cablin* (Blanco) Benth. (Lamiaceae)**

Spanish names: Oregano grande; Patchuli
Indigenous names: Perfumen ujts[04]; Yop'aj pachulin[18]
Used by (3*): Mixe[04]; Western Maya[18]; Zapotec[21]
Used for (8#): Digestive (2)[18, 21]; Respiratory (1)[21]; Pregnancy (1)[21]; Female genital (1)[21]; General and Unspecified (3)[04, 18, 21]
Cognates:
Language contact:

***Poiretia punctata* (Willd.) Desv. (Fabaceae)**

Spanish names: Hierba malina; Hoja malina
Indigenous names: Poj jäjä[02]; Malin ujts[04]; Guixa'a[21]
Used by (3*): Zoque[02]; Mixe[04]; Zapotec[21]
Used for (7#): Digestive (1)[21]; Musculoskeletal (3)[02, 04, 21]; Skin (1)[21]; General and Unspecified (2)[04, 21]
Cognates:
Language contact:

[01-28]refer to the study codes in Table 4.1. *Total number of studies citing this taxon #Total number of use-records

***Polianthes tuberosa* L. (Asparagaceae)**

Spanish names: Azuzena
Indigenous names:
Used by (1*): Zoque[02]
Used for (2#): Respiratory (1)[02]; Pregnancy (1)[02]
Cognates:
Language contact:

***Polygala floribunda* Benth. (Polygalaceae)**

Spanish names: Lavapie/flor de esquipulas/ hierba de seiscientos
Indigenous names: Kä tzanhga / kä tzanhgä[01]
Used by (1*): Zoque[01]
Used for (1#): Skin (1)[01]
Cognates:
Language contact:

***Polygala nitida* Brandegee (Polygalaceae)**

Spanish names:
Indigenous names: Tsakam tsak mokok, ith ts'ohool[07]
Used by (1*): Huastec[07]
Used for (2#): Skin (1)[07]; Pregnancy (1)[07]
Cognates:
Language contact:

***Polygala paniculata* L. (Polygalaceae)**

Spanish names: Anis, reumatuluc
Indigenous names: Ueji ay[03]
Used by (2*): Zoque[03]; Nahua[26]
Used for (5#): Digestive (1)[03]; Musculoskeletal (1)[26]; Psychological (1)[03]; Skin (1)[03]; General and Unspecified (1)[03]
Cognates:
Language contact:

***Polygala trichosperma* L. (Polygalaceae)**

Spanish names:
Indigenous names: Møøy ics[04]
Used by (1*): Mixe[04]
Used for (1#): Digestive (1)[04]
Cognates:
Language contact:

***Polygonum mexicanum* Small (Polygonaceae)**

Spanish names:
Indigenous names: Ehtiil tok'oy[07]
Used by (1*): Huastec[07]
Used for (1#): General and Unspecified (1)[07]
Cognates:
Language contact:

***Polygonum* sp. (Polygonaceae)**

Spanish names: Lirio de Agua
Indigenous names: Aq'om Q'os ya' / Nuwulaj Abäj[12]
Used by (1*): Quichean Maya[12]
Used for (2#): Digestive (1)[12]; Urological (1)[12]
Cognates:
Language contact:

[01-28]refer to the study codes in Table 4.1. *Total number of studies citing this taxon #Total number of use-records

***Polypodium echinolepis*** **Fée (Polypodiaceae)**

Spanish names: Calahuala
Indigenous names:
Used by (1*): Quichean Maya[12]
Used for (8#): Digestive (1)[12]; Musculoskeletal (1)[12]; Psychological (1)[12]; Skin (1)[12]; Endocrine (1)[12]; Urological (1)[12]; Female genital (1)[12]; General and Unspecified (1)[12]
Cognates:
Language contact:

***Polypodium fraternum*** **Schltdl. & Cham. (Polypodiaceae)**

Spanish names: Calahuala
Indigenous names:
Used by (1*): Quichean Maya[13]
Used for (4#): Digestive (1)[13]; Musculoskeletal (1)[13]; Endocrine (1)[13]; General and Unspecified (1)[13]
Cognates:
Language contact:

***Polypodium polypodioides*** **(L.) Watt var. aciculare (Polypodiaceae)**

Spanish names: Helecho menor
Indigenous names: Tsa chimal[03]; Koo'te', tsooy ahaatik, kutsiil boo'waat, chuchim ts'ohool[07]; K'umatz Ruxe' Ibo'ch[12]
Used by (3*): Zoque[03]; Huastec[07]; Quichean Maya[12]
Used for (13#): Digestive (2)[03, 12]; Cardiovascular (2)[03, 12]; Musculoskeletal (1)[12]; Neurological (2)[07, 12]; Psychological (1)[12]; Respiratory (1)[03]; Skin (1)[12]; Endocrine (1)[03]; Urological (1)[03]; General and Unspecified (1)[07]
Cognates:
Language contact: Hua <> Kaqchikel

***Polypodium*** **sp. (Polypodiaceae)**

Spanish names: Calagual, canaguala
Indigenous names: Bàz, guìzh-bàz[23]
Used by (1*): Zapotec[23]
Used for (3#): Digestive (1)[23]; Respiratory (1)[23]; General and Unspecified (1)[23]
Cognates:
Language contact:

***Polypodium triseriale*** **Sw. (Polypodiaceae)**

Spanish names:
Indigenous names: Canagual[03]
Used by (1*): Zoque[03]
Used for (2#): Musculoskeletal (1)[03]; Skin (1)[03]
Cognates:
Language contact:

***Polypremum procumbens*** **L. (Tetrachondraceae)**

Spanish names:
Indigenous names: Billushít[22]
Used by (1*): Zapotec[22]
Used for (1#): Digestive (1)[22]
Cognates:
Language contact:

[01-28]refer to the study codes in Table 4.1. *Total number of studies citing this taxon #Total number of use-records

***Pontederia cordata* L. (Pontederiaceae)**

Spanish names:
Indigenous names: Xjolom Wakax[14]
Used by (1*): Quichean Maya[14]
Used for (4#): Digestive (1)[14]; Urological (1)[14]; Female genital (1)[14]; General and Unspecified (1)[14]
Cognates:
Language contact:

***Populus mexicana* Wesm. ex DC. (Salicaceae)**

Spanish names:
Indigenous names: Itsow[07]
Used by (1*): Huastec[07]
Used for (1#): Psychological (1)[07]
Cognates:
Language contact:

***Porophyllum punctatum* (Mill.) S.F.Blake (Asteraceae)**

Spanish names: Guapillo
Indigenous names: Xuk'ii[09]; So'sol Q'ehen (Macho)[14]
Used by (3*): Yucatecan Maya[09]; Quichean Maya[14]; Western Maya[19]
Used for (3#): Psychological (1)[14]; Pregnancy (1)[19]; General and Unspecified (1)[09]
Cognates:
Language contact:

***Porophyllum ruderale* (Jacq.) Cass. (Asteraceae)**

Spanish names: Pápaloquelite
Indigenous names: Comunk¥ ts¥p¥[03]; Tapahuelo[04]; Pucsnancac'a[06]; Mithith[07]; Sosol pim[16]
Used by (7*): Zoque[01, 03]; Mixe[04]; Totonac[06]; Huastec[07]; Quichean Maya[16]; Western Maya[18]
Used for (16#): Blood (1)[03]; Digestive (4)[03, 06, 07, 18]; Cardiovascular (1)[07]; Musculoskeletal (1)[18]; Neurological (2)[04, 16]; Psychological (1)[16]; Skin (2)[03, 18]; Urological (1)[01]; General and Unspecified (3)[03, 06, 18]
Cognates:
Language contact:

***Porophyllum* sp. (Asteraceae)**

Spanish names: Hoja de bruja/ de piojo
Indigenous names: Gixa'a mbeetzii[21]
Used by (1*): Zapotec[21]
Used for (3#): Digestive (1)[21]; Musculoskeletal (1)[21]; General and Unspecified (1)[21]
Cognates:
Language contact:

***Portulaca oleracea* L. (Portulacaceae)**

Spanish names: Verdolaga
Indigenous names: X'puhl cac'a[06]; Pitsits wal, he'pan paktha', tsakam ix thuyum[07]; Pixla[12]; Paxlac[13]; Mixquilit[28]
Used by (7*): Totonac[06]; Huastec[07]; Quichean Maya[12, 13]; Zapotec[21]; Nahua[26, 28]
Used for (15#): Blood (2)[06, 13]; Digestive (4)[07, 12, 13, 28]; Skin (1)[12]; Urological (2)[12, 26]; Male genital (1)[12]; General and Unspecified (5)[06, 07, 12, 21, 28]
Cognates: Mayan: pits/pix; Quich: pVxla;
Language contact: Maya <> Nah

[01-28]refer to the study codes in Table 4.1.  *Total number of studies citing this taxon  #Total number of use-records

***Portulaca pilosa* L. (Portulacaceae)**

Spanish names:
Indigenous names: Tsakam tsatsa, chak wits[07]
Used by (1*): Huastec[07]
Used for (3#): Respiratory (1)[07]; Skin (1)[07]; General and Unspecified (1)[07]
Cognates:
Language contact:

***Pouteria campechiana* (Kunth) Baehni (Sapotaceae)**

Spanish names: Zapote bianco
Indigenous names: Chu kuxamñi[03]; Sakya'[09]
Used by (2*): Zoque[03]; Yucatecan Maya[09]
Used for (3#): Digestive (2)[03, 09]; Female genital (1)[03]
Cognates:
Language contact:

***Pouteria glomerata* (Miq.) Radlk. (Sapotaceae)**

Spanish names:
Indigenous names: Tsatsootso[03]; Thokob[07]
Used by (2*): Zoque[03]; Huastec[07]
Used for (7#): Musculoskeletal (1)[03]; Psychological (1)[03]; Respiratory (2)[03, 07]; Skin (1)[07]; Pregnancy (1)[03]; Female genital (1)[03]
Cognates:
Language contact: Highland Popoluca <> Hua

***Pouteria sapota* (Jacq.) H.E.Moore & Stearn (Sapotaceae)**

Spanish names: Zapote (colorado), mamey
Indigenous names: Taki syapane/tzapas tzapane[01]; Tzapane/nui näjä/kawak[02]; Kuxamñi[03]; Jaka[05]; Jaaca[06]; Bolom it'ath[07]; La'adxiguelexuunu'uj[21]
Used by (9*): Zoque[01, 02, 03]; Totonac[05, 06]; Huastec[07]; Yucatecan Maya[09, 11]; Zapotec[21]
Used for (17#): Digestive (3)[03, 06, 09]; Cardiovascular (1)[07]; Musculoskeletal (1)[21]; Psychological (1)[05]; Respiratory (1)[03]; Skin (7)[01, 02, 03, 06, 07, 11, 21]; Female genital (1)[03]; General and Unspecified (2)[06, 21]
Cognates: Zoq: tzapane; Toto: jaka;
Language contact: MZ <> Toto; Zoq > Nah

***Pouzolzia occidentalis* (Liebm.) Wedd. (Urticaceae)**

Spanish names:
Indigenous names: Uxum ilaal, tsakam baat[07]
Used by (1*): Huastec[07]
Used for (5#): Digestive (1)[07]; Musculoskeletal (1)[07]; Respiratory (1)[07]; Pregnancy (1)[07]; Female genital (1)[07]
Cognates:
Language contact:

***Prestonia mexicana* A. DC. (Apocynaceae)**

Spanish names: Bejuco de jiote
Indigenous names: Kuts¥k[03]
Used by (1*): Zoque[03]
Used for (1#): Skin (1)[03]
Cognates:
Language contact:

[01-28]refer to the study codes in Table 4.1. *Total number of studies citing this taxon #Total number of use-records

***Prionosciadium nelsonii* J.M.Coult & Rose (Apiaceae)**

Spanish names: Eneldón
Indigenous names:
Used by (1*): Zoque[01]
Used for (1#): Pregnancy (1)[01]
Cognates:
Language contact:

***Prionosciadium thapsoides* (DC.) Mathias (Apiaceae)**

Spanish names: Eneldo, chilibia
Indigenous names: Tzi, tunai, buxnay[13]
Used by (1*): Quichean Maya[13]
Used for (2#): Digestive (1)[13]; General and Unspecified (1)[13]
Cognates:
Language contact:

***Priva lappulacea* (L.) Pers. (Verbenaceae)**

Spanish names: Cadillo de bolsa; Hoja de azar; Jehuite chicloso; Sogia de tusa
Indigenous names: Tza'a tzoy[01]; T¥pich nang tsang[03]; T'apay ts'ohool[07]; Tapay ts'ohool[08]; Xpak'umpak'[09]; Pax Pa'am[14]
Used by (8*): Zoque[01, 03]; Huastec[07, 08]; Yucatecan Maya[09]; Quichean Maya[14]; Western Maya[18]; Nahua[24]
Used for (11#): Digestive (3)[03, 07, 09]; Musculoskeletal (1)[08]; Neurological (1)[08]; Skin (2)[09, 18]; Pregnancy (1)[24]; Female genital (2)[03, 14]; General and Unspecified (1)[01]
Cognates: Zoq: tsa; Huas: tapay ts'ohool; CoreM: paC (um)paC;
Language contact: Hua > Highland Popoluca

***Prosopis laevigata* (Willd.) M.C.Johnst. (Fabaceae)**

Spanish names: Mesquite; Mezquite
Indigenous names: Ut'u[07]; Geč beh[22]; Mizquitl[28]
Used by (4*): Huastec[07]; Zapotec[22]; Nahua[27, 28]
Used for (8#): Digestive (3)[22, 27, 28]; Eye (4)[07, 22, 27, 28]; Pregnancy (1)[27]
Cognates:
Language contact:

***Prosopis* sp. (Fabaceae)**

Spanish names: Mesquite
Indigenous names:
Used by (1*): Zapotec[21]
Used for (4#): Digestive (1)[21]; Eye (1)[21]; Skin (1)[21]; General and Unspecified (1)[21]
Cognates:
Language contact:

***Prosthechea cochleata* (L.) W.E.Higgins (Orchidaceae)**

Spanish names:
Indigenous names: Ts'aak[07]
Used by (1*): Huastec[07]
Used for (1#): Endocrine (1)[07]
Cognates:
Language contact:

***Protium copal*** **(Schltdl. & Cham.) Engl. (Burseraceae)**

Spanish names: Copal; Copal de chichi; Copalillo
Indigenous names: Tzyutzyin bono/kutzyin bono/cochinbomo/Tzyitzyin bomo [01]; Puam, pum[05]; Homte', hom, ikob te'[07]; Pom[10]; Pom[11]
Used by (5*): Zoque[01]; Totonac[05]; Huastec[07]; Yucatecan Maya[10, 11]
Used for (14#): Digestive (2)[01, 07]; Ear (1)[07]; Musculoskeletal (2)[01, 11]; Neurological (3)[07, 10, 11]; Respiratory (1)[01]; Skin (1)[05]; General and Unspecified (4)[05, 07, 10, 11]
Cognates: Mayan: om; Yuca: pom;
Language contact: MZ > Tot and Maya

***Prunella*** **sp. (Lamiaceae)**

Spanish names:
Indigenous names: Xinahuate[26]
Used by (1*): Nahua[26]
Used for (1#): Skin (1)[26]
Cognates:
Language contact:

***Prunella vulgaris*** **L. (Lamiaceae)**

Spanish names:
Indigenous names:
Used by (1*): Quichean Maya[13]
Used for (1#): Skin (1)[13]
Cognates:
Language contact:

***Prunus armeniaca*** **L. (Rosaceae)**

Spanish names: Chabacano
Indigenous names:
Used by (1*): Totonac[06]
Used for (1#): Pregnancy (1)[06]
Cognates:
Language contact:

***Prunus domestica*** **L. (Rosaceae)**

Spanish names: Ciruela
Indigenous names:
Used by (1*): Quichean Maya[12]
Used for (1#): Digestive (1)[12]
Cognates:
Language contact:

***Prunus persica*** **(L.) Batsch (Rosaceae)**

Spanish names: Durazno
Indigenous names: Tunas[01]; Tras[13]; Turesna[20]; Yàg-drâz[23]
Used by (8*): Zoque[01]; Totonac[06]; Quichean Maya[12, 13]; Western Maya[20]; Zapotec[23]; Nahua[25, 26]
Used for (22#): Digestive (7)[01, 12, 13, 20, 23, 25, 26]; Cardiovascular (1)[01]; Musculoskeletal (1)[13]; Neurological (3)[01, 12, 26]; Respiratory (3)[01, 12, 13]; Skin (3)[06, 12, 25]; Female genital (1)[01]; General and Unspecified (3)[01, 12, 13]
Cognates:
Language contact:

[01-28] refer to the study codes in Table 4.1. *Total number of studies citing this taxon #Total number of use-records

***Prunus serotina* Ehrh. (Rosaceae)**

Spanish names: Cerezo
Indigenous names: K'ask'el[13]; Yàg-bziă[23]
Used by (2*): Quichean Maya[13]; Zapotec[23]
Used for (4#): Digestive (1)[23]; Musculoskeletal (1)[13]; Skin (1)[13]; General and Unspecified (1)[13]
Cognates:
Language contact:

***Pseudelephantopus spicatus* (Juss. ex Aubl.) Rohr (Asteraceae)**

Spanish names: Lengua de vaca
Indigenous names: Lek'ab paakax[07]
Used by (2*): Zoque[03]; Huastec[07]
Used for (5#): Digestive (1)[03]; Neurological (1)[07]; Respiratory (1)[03]; Skin (1)[03]; Urological (1)[03]
Cognates:
Language contact:

***Pseudobombax ellipticum* (Kunth) Dugand (Malvaceae)**

Spanish names: Amabola blanco; Clavillina; Solosoche
Indigenous names: Tzospo/ a'pompo[02]; Pop uakta[03]; Mokok[07]; Siklite, Xk'uxche'[09]; Xiloxoochitl[24]
Used by (5*): Zoque[02, 03]; Huastec[07]; Yucatecan Maya[09]; Nahua[24]
Used for (12#): Digestive (1)[07]; Psychological (1)[07]; Respiratory (1)[09]; Endocrine (1)[02]; Urological (2)[02, 03]; Pregnancy (3)[03, 07, 24]; Female genital (1)[03]; General and Unspecified (2)[03, 07]
Cognates:
Language contact: Yuc <> Nah

***Pseudognaphalium attenuatum* (DC.) Anderb. (Asteraceae)**

Spanish names: Gordolobo
Indigenous names: Popo 'uka tane/popo jäyä/pop tane/ akstäk tane[01]
Used by (2*): Zoque[01, 02]
Used for (4#): Digestive (1)[01]; Respiratory (2)[01, 02]; Skin (1)[01]
Cognates:
Language contact:

***Pseudognaphalium chartaceum* (Greenm.) Anderb. (Asteraceae)**

Spanish names: Simonilla
Indigenous names: Sìmònî[23]
Used by (1*): Zapotec[23]
Used for (2#): Blood (1)[23]; Digestive (1)[23]
Cognates:
Language contact:

***Pseudognaphalium ehrenbergianum* (Sch.Bip.ex Klatt) G.L.Nesom (Asteraceae)**

Spanish names: Gordolobo
Indigenous names:
Used by (1*): Nahua[26]
Used for (1#): Respiratory (1)[26]
Cognates:
Language contact:

***Pseudognaphalium oxyphyllum* (DC.) Kirp. (Asteraceae)**

Spanish names: Gordolobo
Indigenous names:
Used by (2*): Nahua[25, 28]
Used for (6#): Cardiovascular (1)[28]; Neurological (1)[25]; Respiratory (2)[25, 28]; Urological (1)[28]; General and Unspecified (1)[28]
Cognates:
Language contact:

[01-28]refer to the study codes in Table 4.1. *Total number of studies citing this taxon #Total number of use-records

***Pseudognaphalium* sp. (Asteraceae)**

Spanish names: Gordolobo
Indigenous names: Gòrdòlôb[23]
Used by (1*): Zapotec[23]
Used for (4#): Respiratory (1)[23]; Skin (1)[23]; Pregnancy (1)[23]; General and Unspecified (1)[23]
Cognates:
Language contact:

***Pseudogynoxys chenopodioides* (Kunth) Cabrera (Asteraceae)**

Spanish names:
Indigenous names: Uentex ay[03]; Te'te' wits, chuklaab ts'ohool[07]; Kanal xijch'[20]
Used by (3*): Zoque[03]; Huastec[07]; Western Maya[20]
Used for (3#): Digestive (1)[20]; Skin (1)[03]; General and Unspecified (1)[07]
Cognates:
Language contact:

***Pseudogynoxys haenkei* (DC.) Cabrera (Asteraceae)**

Spanish names: Hierba de carga
Indigenous names: Tzämi tzoy[01]
Used by (1*): Zoque[01]
Used for (1#): General and Unspecified (1)[01]
Cognates:
Language contact:

***Psidium friedrichsthalianum* (O. Berg) Nied. (Myrtaceae)**

Spanish names: Guajava agria
Indigenous names: Katsu patan[03]
Used by (1*): Zoque[03]
Used for (1#): Digestive (1)[03]
Cognates:
Language contact:

***Psidium guajava* L. (Myrtaceae)**

Spanish names: Guayaba
Indigenous names: Po'os/padan/paran/pataya[01]; Poks/po's/ pataya[02]; Patan[03]; Pox[04]; Asihuit, aci'huit, llasibit[05]; Aa'si'huiit[06]; Bek[07]; Bek[08]; Pichi'[09]; PiČi[10]; Keq'[12]; Pata'a[14]; Pata'[18]; Pataj[19]; Pata, potov, potoj, poto'[20]; Guisha nguetuj[21]; Bäwi[22]; Xalxocotl[24]; Xalxocotl[26]; Xalxócotl[28]
Used by (23*): Zoque[01, 02, 03]; Mixe[04]; Totonac[05, 06]; Huastec[07, 08]; Yucatecan Maya[09, 10, 11]; Quichean Maya[12, 14]; Western Maya[18, 19, 20]; Zapotec[21, 22]; Nahua[24, 25, 26, 27, 28]
Used for (66#): Blood (1)[12]; Digestive (23)[01, 02, 03, 04, 05, 06, 07, 08, 09, 10, 11, 12, 14, 18, 19, 20, 21, 22, 24, 25, 26, 27, 28]; Ear (1)[01]; Cardiovascular (2)[02, 12]; Musculoskeletal (2)[01, 12]; Neurological (1)[08]; Psychological (2)[01, 12]; Respiratory (5)[02, 03, 07, 19, 28]; Skin (12)[01, 02, 03, 05, 07, 08, 09, 10, 14, 18, 25, 26]; Urological (4)[01, 03, 07, 26]; Pregnancy (2)[02, 05]; Female genital (4)[01, 02, 03, 21]; General and Unspecified (7)[01, 02, 05, 09, 12, 21, 28]
Cognates: MZ: pos/pox; Zoq: pata; Toto: asiwit; Huas: bek; Yuca: pichi; WesM: pata; Nahua: xalxocotl;
Language contact: Zoq > WesM and Kekchí

***Psidium guineense* Sw. (Myrtaceae)**

Spanish names: Guayaba agria
Indigenous names: Katzu patan/katzu paran/ katsu 'ätz/ katzsy patan/ katzu wos[01]; Poks pataja/ katsu pataja/ toks pataja[02]; Copa patan[03]; Jokøønypapox tikts[04]; Bekil tooro, beekil an buuru[07]; Ixim pataj[19]; Paxchak', pajal pata/potoj, potov, poto'[20]
Used by (7*): Zoque[01, 02, 03]; Mixe[04]; Huastec[07]; Western Maya[19, 20]
Used for (21#): Digestive (6)[01, 02, 03, 04, 07, 20]; Eye (1)[20]; Musculoskeletal (1)[01]; Neurological (2)[01, 20]; Psychological (1)[01]; Respiratory (2)[01, 20]; Urological (2)[01, 02]; Pregnancy (2)[02, 19]; Female genital (3)[02, 03, 19]; General and Unspecified (1)[01]
Cognates: MZ: pos/pox; Zoq: pata; WesM: pata;
Language contact: Zoq > WesM

[01-28]refer to the study codes in Table 4.1. *Total number of studies citing this taxon #Total number of use-records

***Psidium hypoglaucum*** **Standl. (Myrtaceae)**

Spanish names: Guabillo
Indigenous names: Behuishxuba'a[21]
Used by (1*): Zapotec[21]
Used for (1#): Digestive (1)[21]
Cognates:
Language contact:

***Psidium salutare*** **(Kunth) O.Berg (Myrtaceae)**

Spanish names: Raiana; Rayanita, Rayana, Capulín de tierra
Indigenous names: Nas mupe[02]; Bihuishuba'aj[21]
Used by (2*): Zoque[02]; Zapotec[21]
Used for (7#): Digestive (2)[02, 21]; Cardiovascular (1)[02]; Neurological (1)[02]; Pregnancy (1)[02]; Female genital (1)[02]; General and Unspecified (1)[02]
Cognates:
Language contact:

***Psidium sartorianum*** **(O.Berg) Nied. (Myrtaceae)**

Spanish names:
Indigenous names: Pichiche'[09]
Used by (1*): Yucatecan Maya[09]
Used for (1#): Skin (1)[09]
Cognates:
Language contact:

***Psidium*** **sp. (Myrtaceae)**

Spanish names: Guayaba (dulce/agría); Guayaba cimmaron; Itamo real
Indigenous names: Aa'si'huiit[06]; (Kia/Chom) Kiej/keq'[13]
Used by (3*): Zoque[03]; Totonac[06]; Quichean Maya[13]
Used for (6#): Digestive (3)[03, 06, 13]; Pregnancy (1)[13]; Female genital (1)[13]; General and Unspecified (1)[13]
Cognates:
Language contact:

***Psiguria triphylla*** **(Miq.) J. Jeffrey (Cucurbitaceae)**

Spanish names:
Indigenous names:
Used by (1*): Zoque[03]
Used for (1#): Skin (1)[03]
Cognates:
Language contact:

***Psittacanthus calyculatus*** **(DC.) G.Don (Loranthaceae)**

Spanish names: Hoja de urraca, mata palo
Indigenous names: Guixa'a shaguii[21]
Used by (1*): Zapotec[21]
Used for (2#): Skin (1)[21]; General and Unspecified (1)[21]
Cognates:
Language contact:

***Psittacanthus schiedeanus*** **(Schltdl. & Cham.) G.Don (Loranthaceae)**

Spanish names:
Indigenous names: Ok'lom te' puulik[07]
Used by (1*): Huastec[07]
Used for (4#): Digestive (1)[07]; Neurological (1)[07]; Skin (1)[07]; General and Unspecified (1)[07]
Cognates:
Language contact:

[01-28]refer to the study codes in Table 4.1. *Total number of studies citing this taxon #Total number of use-records

***Psittacanthus* sp. (Loranthaceae)**

Spanish names:
Indigenous names: Guièe-ló-yâg-guièts, guièe-ló-yâg-nlìbâd-tsò[23]
Used by (1*): Zapotec[23]
Used for (1#): Neurological (1)[23]
Cognates:
Language contact:

***Psychotria biaristata* Bartl. ex DC. (Rubiaceae)**

Spanish names: Curamal
Indigenous names:
Used by (1*): Yucatecan Maya[11]
Used for (1#): Digestive (1)[11]
Cognates:
Language contact:

***Psychotria carthagenensis* Jacq. (Rubiaceae)**

Spanish names:
Indigenous names: Š Anal[10]
Used by (1*): Yucatecan Maya[10]
Used for (1#): Neurological (1)[10]
Cognates:
Language contact:

***Psychotria erythrocarpa* Schltdl. (Rubiaceae)**

Spanish names:
Indigenous names: Baina ts'ohool, wiichab ts'ohool, bekil papaam, k'animiin wits, mapux ts'ohool, bathex, ook' tse'tsem, paktha' wach'uy ch'ohool[07]
Used by (1*): Huastec[07]
Used for (3#): Digestive (1)[07]; Skin (1)[07]; General and Unspecified (1)[07]
Cognates:
Language contact:

***Psychotria flava* Oerst. ex Standl. (Rubiaceae)**

Spanish names:
Indigenous names: Tsus pitx cuy[03]; Xkoti yuk[14]
Used by (2*): Zoque[03]; Quichean Maya[14]
Used for (3#): Digestive (1)[14]; Respiratory (1)[14]; Skin (1)[03]
Cognates:
Language contact:

***Psychotria glomerulata* (Donn.Sm.) Steyerm. (Rubiaceae)**

Spanish names: Segunda Lengua del Perro
Indigenous names: Xkab' Rujiraq'i Tz'i'[14]
Used by (2*): Quichean Maya[14, 17]
Used for (3#): Neurological (1)[14]; General and Unspecified (1)[14]; nd[17]
Cognates:
Language contact:

***Psychotria hebeclada* DC. (Rubiaceae)**

Spanish names: Hoja morada
Indigenous names: Masan ay, Monch¥v ay[03]
Used by (1*): Zoque[03]
Used for (1#): Skin (1)[03]
Cognates:
Language contact:

***Psychotria nervosa* Sw. (Rubiaceae)**

Spanish names: Cafecillo
Indigenous names: Masan ay[03]
Used by (1*): Zoque[03]
Used for (2[#]): Digestive (1)[03]; Skin (1)[03]
Cognates:
Language contact:

***Psychotria panamensis* Standl. (Rubiaceae)**

Spanish names:
Indigenous names: Tsus pitx cuy[03]
Used by (1*): Zoque[03]
Used for (4[#]): Digestive (1)[03]; Musculoskeletal (1)[03]; Skin (1)[03]; Female genital (1)[03]
Cognates:
Language contact:

***Psychotria poeppigiana* Müll.Arg. (Rubiaceae)**

Spanish names:
Indigenous names: Ak Pere Tzo', peren pim[15]; Saxjolom chilan[16]
Used by (2*): Quichean Maya[15, 16]
Used for (2[#]): Neurological (1)[16]; Pregnancy (1)[15]
Cognates:
Language contact:

***Psychotria pubescens* Sw. (Rubiaceae)**

Spanish names:
Indigenous names: Tschul-keeh[09]; Chäk k'änan[11]
Used by (2*): Yucatecan Maya[09, 11]
Used for (3[#]): Neurological (1)[09]; General and Unspecified (2)[09, 11]
Cognates:
Language contact:

***Psychotria* sp. (Rubiaceae)**

Spanish names: Hierba de sabañon
Indigenous names: Tse'tsem ts'ohool, kapee ts'ohool, tsakam ts'abat, t'othooybe ts'ohool, baina ts'ohool, itsal kw'a, wats'ul, thuyum olom[07]; Tzuul Q'ehen re Ha'[14]; Na' Ichaj'[14]
Used by (3*): Huastec[07]; Quichean Maya[14]; Nahua[26]
Used for (8[#]): Digestive (1)[07]; Musculoskeletal (1)[14]; Neurological (1)[14]; Skin (2)[07, 26]; Female genital (1)[14]; General and Unspecified (2)[07, 14]
Cognates: Mayan: tsul;
Language contact:

***Psychotria tenuifolia* Sw. (Rubiaceae)**

Spanish names: Simonillo
Indigenous names: Tam txitx¥k[03]; Ix k'ä anal hembra[11]; Re Kanteel, Mai Tzuul[14]; Colaras[16]
Used by (4*): Zoque[03]; Yucatecan Maya[11]; Quichean Maya[14, 16]
Used for (7[#]): Digestive (1)[03]; Musculoskeletal (1)[14]; Neurological (2)[14, 16]; Skin (1)[03]; General and Unspecified (2)[11, 14]
Cognates:
Language contact: Yuc <> Kekchí

[01-28]refer to the study codes in Table 4.1. *Total number of studies citing this taxon [#]Total number of use-records

***Pteridium aquilinum* (L.) Kuhn (Dennstaedtiaceae)**

Spanish names: Costilludo/ tapa carbon; Helecho chispa; Ocopeta; Pesma
Indigenous names: Xip[13]
Used by (4*): Zoque[01, 03]; Quichean Maya[13]; Nahua[25]
Used for (7#): Musculoskeletal (1)[13]; Psychological (1)[03]; Respiratory (2)[03, 25]; Endocrine (1)[25]; Pregnancy (1)[01]; General and Unspecified (1)[13]
Cognates:
Language contact:

***Pteris chiapensis* A.R. Sm. (Pteridaceae)**

Spanish names: Helecho liso
Indigenous names: Taksy kingin/ pu'kuy tuks[01]
Used by (1*): Zoque[01]
Used for (5#): Digestive (1)[01]; Endocrine (1)[01]; Urological (1)[01]; Male genital (1)[01]; General and Unspecified (1)[01]
Cognates:
Language contact:

***Pteris grandifolia* L. (Pteridaceae)**

Spanish names: Helecho macho
Indigenous names: Mä'a kingin[01]
Used by (1*): Zoque[01]
Used for (1#): Digestive (1)[01]
Cognates:
Language contact:

***Pteris plumula* Desv. (Pteridaceae)**

Spanish names:
Indigenous names:
Used by (1*): Quichean Maya[16]
Used for (2#): Psychological (1)[16]; General and Unspecified (1)[16]
Cognates:
Language contact:

***Pterocarpus rohrii* Vahl (Fabaceae)**

Spanish names:
Indigenous names: Akxcuy[03]
Used by (1*): Zoque[03]
Used for (2#): Skin (1)[03]; Female genital (1)[03]
Cognates:
Language contact:

***Punica granatum* L. (Lythraceae)**

Spanish names: Granada
Indigenous names: Laab bek[07]; Žob štil[22]; Yàg-ngùd-guièe-ziè, yàg-grànâd[23]
Used by (15*): Zoque[01, 02, 03]; Mixe[04]; Huastec[07]; Yucatecan Maya[09, 10]; Quichean Maya[12, 13]; Western Maya[18, 19]; Zapotec[21, 22, 23]; Nahua[28]
Used for (26#): Blood (1)[02]; Digestive (13)[01, 02, 03, 04, 07, 09, 12, 18, 19, 21, 22, 23, 28]; Eye (1)[13]; Musculoskeletal (1)[21]; Neurological (2)[12, 28]; Psychological (1)[12]; Respiratory (1)[21]; Skin (3)[02, 09, 21]; Pregnancy (1)[10]; Female genital (1)[21]; General and Unspecified (1)[12]
Cognates:
Language contact:

[01-28]refer to the study codes in Table 4.1. *Total number of studies citing this taxon #Total number of use-records

***Quassia* sp. (Simaroubaceae)**

Spanish names: Cuassia
Indigenous names:
Used by (1*): Zapotec[21]
Used for (1#): Digestive (1)[21]
Cognates:
Language contact:

***Quercus candicans* Née (Fagaceae)**

Spanish names: Roble blanco
Indigenous names: Popo kamay[01]; Sak yok jij te'[20]
Used by (2*): Zoque[01]; Western Maya[20]
Used for (4#): Digestive (2)[01, 20]; Neurological (1)[01]; Endocrine (1)[01]
Cognates:
Language contact:

***Quercus conspersa* Benth. (Fagaceae)**

Spanish names: Encino rojo; Encino, Roble
Indigenous names: Cap soj / Tsabats soj[03]; Patän, Raxche'[12]
Used by (2*): Zoque[03]; Quichean Maya[12]
Used for (6#): Digestive (2)[03, 12]; Musculoskeletal (1)[12]; Neurological (1)[12]; Pregnancy (1)[03]; Female genital (1)[03]
Cognates:
Language contact:

***Quercus crassifolia* Bonpl. (Fagaceae)**

Spanish names: Roble
Indigenous names: Patän che', Räx che'[12]
Used by (1*): Quichean Maya[12]
Used for (2#): Skin (1)[12]; General and Unspecified (1)[12]
Cognates:
Language contact:

***Quercus elliptica* Née (Fagaceae)**

Spanish names: Encino nanche/encino rojo
Indigenous names: Pak soj/syoj[02]
Used by (1*): Zoque[02]
Used for (6#): Digestive (1)[02]; Musculoskeletal (1)[02]; Neurological (1)[02]; Respiratory (1)[02]; Skin (1)[02]; Female genital (1)[02]
Cognates:
Language contact:

***Quercus glaucescens* Bonpl. (Fagaceae)**

Spanish names: Encino amarillo; Encino blanco
Indigenous names: Popo soj[02]; Puutx soj[03]; Poop xoj[04]; Beshxii nagatzii[21]
Used by (4*): Zoque[02, 03]; Mixe[04]; Zapotec[21]
Used for (6#): Digestive (2)[03, 04]; Neurological (1)[02]; Female genital (3)[03, 04, 21]
Cognates: MZ: popo soj/xoj; Zoq: soj;
Language contact:

***Quercus oleoides* Schltdl. & Cham. (Fagaceae)**

Spanish names: Encino; Encino blanco/nanche
Indigenous names: Pop soj[03]; Jiny xoj[04]; Beshxii qutzii[21]
Used by (3*): Zoque[03]; Mixe[04]; Zapotec[21]
Used for (8#): Digestive (3)[03, 04, 21]; Neurological (1)[03]; Skin (1)[03]; Female genital (2)[03, 21]; General and Unspecified (1)[21]
Cognates: MZ: soj/xoj; Zoq: soj;
Language contact:

***Quercus peduncularis* Née (Fagaceae)**

Spanish names: Encino negro
Indigenous names: Y¥k soj[03]
Used by (1*): Zoque[03]
Used for (4#): Digestive (1)[03]; Skin (1)[03]; Pregnancy (1)[03]; Female genital (1)[03]
Cognates:
Language contact:

***Quercus sapotifolia* Liebm. (Fagaceae)**

Spanish names:
Indigenous names: Tsapt xoj, poop xoj[04]
Used by (1*): Mixe[04]
Used for (3#): Digestive (1)[04]; Skin (1)[04]; Female genital (1)[04]
Cognates:
Language contact:

***Quercus skinneri* Benth. (Fagaceae)**

Spanish names: Encino
Indigenous names: Pichik[12]
Used by (1*): Quichean Maya[12]
Used for (3#): Digestive (1)[12]; Cardiovascular (1)[12]; General and Unspecified (1)[12]
Cognates:
Language contact:

***Quercus* sp. (Fagaceae)**

Spanish names: Encino; Roble
Indigenous names: Soj[03]; Sk'el, squ'el, tuluk, tux[13]; Ch'oror[19]; K'an tulan[20]
Used by (4*): Zoque[03]; Quichean Maya[13]; Western Maya[19, 20]
Used for (10#): Blood (1)[03]; Digestive (4)[03, 13, 19, 20]; Neurological (2)[13, 19]; Skin (1)[13]; Female genital (1)[03]; General and Unspecified (1)[13]
Cognates: CoreM: tul;
Language contact:

***Quercus xalapensis* Bonpl. (Fagaceae)**

Spanish names:
Indigenous names: Ok soj[03]
Used by (1*): Zoque[03]
Used for (1#): Digestive (1)[03]
Cognates:
Language contact:

***Randia aculeata* L. (Rubiaceae)**

Spanish names: Morro
Indigenous names:
Used by (1*): Zoque[01]
Used for (1#): Digestive (1)[01]
Cognates:
Language contact:

***Randia laetevirens* Standl. (Rubiaceae)**

Spanish names:
Indigenous names: Tsotsoob olom, tsetsbaal i olom, kruus k'iith[07]
Used by (1*): Huastec[07]
Used for (2#): Digestive (1)[07]; Skin (1)[07]
Cognates:
Language contact:

***Randia longiloba* Hemsl. (Rubiaceae)**

Spanish names:
Indigenous names: K'ax[09]
Used by (1*): Yucatecan Maya[09]
Used for (1#): General and Unspecified (1)[09]
Cognates:
Language contact:

***Randia* sp. (Rubiaceae)**

Spanish names:
Indigenous names: Jeepe ay, Chuk wikpak[03]
Used by (1*): Zoque[03]
Used for (4#): Neurological (1)[03]; Psychological (1)[03]; Skin (1)[03]; General and Unspecified (1)[03]
Cognates:
Language contact:

***Ranunculus petiolaris* Humb., Bonpl. & Kunth ex DC. (Ranunculaceae)**

Spanish names: Pata de león, mano de león; Tijebete
Indigenous names: Xat uxaj'[13]
Used by (2*): Quichean Maya[13]; Nahua[26]
Used for (5#): Musculoskeletal (1)[26]; Skin (2)[13, 26]; Endocrine (1)[26]; General and Unspecified (1)[13]
Cognates:
Language contact:

***Raphanus raphanistrum* L. (Brassicaceae)**

Spanish names: Rabano
Indigenous names:
Used by (5*): Zoque[01]; Yucatecan Maya[09]; Quichean Maya[12, 13]; Zapotec[23]
Used for (12#): Digestive (2)[12, 13]; Cardiovascular (1)[12]; Musculoskeletal (1)[12]; Respiratory (3)[01, 09, 13]; Skin (1)[13]; Urological (1)[12]; Male genital (1)[12]; General and Unspecified (2)[12, 23]
Cognates:
Language contact:

***Rauvolfia tetraphylla* L. (Apocynaceae)**

Spanish names: Chalchupa; Veneno de perro
Indigenous names: Itsaan an k'ak'al ilaal[07]; Kambamuk[09]; Sisar k'opot[19]; Guanabajcu[21]
Used by (5*): Zoque[03]; Huastec[07]; Yucatecan Maya[09]; Western Maya[19]; Zapotec[21]
Used for (8#): Skin (3)[03, 09, 21]; Pregnancy (1)[19]; Female genital (3)[03, 19, 21]; General and Unspecified (1)[07]
Cognates:
Language contact:

***Renealmia alpinia* (Rottb.) Maas (Zingiberaceae)**

Spanish names:
Indigenous names: Jua[01]; Tz'i'[14]
Used by (2*): Zoque[01]; Quichean Maya[14]
Used for (2#): Musculoskeletal (1)[14]; General and Unspecified (1)[01]
Cognates:
Language contact:

***Renealmia mexicana* Klotzsch ex Petersen (Zingiberaceae)**

Spanish names:
Indigenous names: Jua'/tzuku' waja[02]
Used by (1*): Zoque[02]
Used for (2#): Musculoskeletal (1)[02]; Skin (1)[02]
Cognates:
Language contact:

[01-28]refer to the study codes in Table 4.1. *Total number of studies citing this taxon #Total number of use-records

***Rhamnus humboldtiana*** **Willd. ex Schult. (Rhamnaceae)**

Spanish names:
Indigenous names: Itsil, ichil[07]
Used by (1*): Huastec[07]
Used for (1#): General and Unspecified (1)[07]
Cognates:
Language contact:

***Rheum rhaponticum*** **L. (Polygonaceae)**

Spanish names: Ruibarbo
Indigenous names:
Used by (1*): Quichean Maya[12]
Used for (3#): Blood (1)[12]; Digestive (1)[12]; Cardiovascular (1)[12]
Cognates:
Language contact:

***Rhipidocladum pittieri*** **(Hack.) McClure (Poaceae)**

Spanish names: Carrizo
Indigenous names: Aj Che K'etchelaj[12]
Used by (1*): Quichean Maya[12]
Used for (2#): Musculoskeletal (1)[12]; Urological (1)[12]
Cognates:
Language contact:

***Rhipsalis baccifera*** **(J.S.Muell.) Stearn (Cactaceae)**

Spanish names: Injerto, solitaria, niguilla
Indigenous names: Paka hui'huat[06]; Xi'il boo'waat, xi'ixl uxum, weew i path uut', tsakam pak'ak', okôlom te', kwathnab ts'aah[07]; Roq Ak'ach Q'ehen[14]
Used by (3*): Totonac[06]; Huastec[07]; Quichean Maya[14]
Used for (10#): Cardiovascular (1)[07]; Musculoskeletal (1)[07]; Neurological (1)[07]; Psychological (1)[07]; Skin (2)[07, 14]; Endocrine (1)[06]; Urological (1)[07]; General and Unspecified (2)[07, 14]
Cognates: Mayan: ak'a;
Language contact: Tot <> Hua

***Rhododendron indicum*** **(L.) Sweet (Ericaceae)**

Spanish names: Azalia blanca
Indigenous names:
Used by (1*): Nahua[26]
Used for (1#): Respiratory (1)[26]
Cognates:
Language contact:

***Rhodosciadium*** **sp. (Apiaceae)**

Spanish names:
Indigenous names: Guiéer-ngüèets, xín-pèrèjîl[23]
Used by (1*): Zapotec[23]
Used for (1#): Urological (1)[23]
Cognates:
Language contact:

[01-28]refer to the study codes in Table 4.1. *Total number of studies citing this taxon #Total number of use-records

***Rhus pachyrrhachis* Hemsl. (Anacardiaceae)**

Spanish names: Zumaque
Indigenous names: Yàg-bèch-mbăr[23]
Used by (1*): Zapotec[23]
Used for (1#): Skin (1)[23]
Cognates:
Language contact:

***Rhus standleyi* F.A. Barkley (Anacardiaceae)**

Spanish names: Zumaque
Indigenous names: Xín-bèch-mbăr[23]
Used by (1*): Zapotec[23]
Used for (1#): Skin (1)[23]
Cognates:
Language contact:

***Rhus terebinthifolia* Schltdl. & Cham. (Anacardiaceae)**

Spanish names: Sal de venado, zumaque
Indigenous names: Katxu ay[03]; Rtz'am mzat/kiej[13]; B'iritaq[14]; Yàg-bèch-lsæ̀b[23]
Used by (6*): Zoque[01, 03]; Quichean Maya[12, 13, 14]; Zapotec[23]
Used for (13#): Digestive (2)[03, 14]; Cardiovascular (1)[12]; Musculoskeletal (2)[12, 13]; Neurological (1)[12]; Skin (3)[12, 13, 23]; Urological (1)[03]; Female genital (1)[03]; General and Unspecified (2)[01, 12]
Cognates:
Language contact:

***Rhynchosia erythrinoides* Schltdl. et Cham. (Fabaceae)**

Spanish names: Ojo de picho
Indigenous names: Sinchu ixcuy[03]
Used by (1*): Zoque[03]
Used for (1#): Skin (1)[03]
Cognates:
Language contact:

***Rhynchosia longeracemosa* M.Martens & Galeotti (Fabaceae)**

Spanish names:
Indigenous names: Tsanakw'iil t'eel[07]
Used by (1*): Huastec[07]
Used for (1#): Skin (1)[07]
Cognates:
Language contact:

***Rhynchosia pyramidalis* (Lam.) Urb. (Fabaceae)**

Spanish names: Ojo de picho
Indigenous names: Sinchu ixcuy[03]
Used by (1*): Zoque[03]
Used for (3#): Skin (1)[03]; Pregnancy (1)[03]; Female genital (1)[03]
Cognates:
Language contact:

***Rhynchospora radicans* (Schltdl. & Cham.) H.Pfeiff. (Cyperaceae)**

Spanish names:
Indigenous names: Tathiim toom[07]
Used by (1*): Huastec[07]
Used for (1#): Digestive (1)[07]
Cognates:
Language contact:

***Richardia scabra* L. (Rubiaceae)**

Spanish names: Cola de Alacrán
Indigenous names:
Used by (1*): Quichean Maya[12]
Used for (3[#]): Cardiovascular (1)[12]; Musculoskeletal (1)[12]; General and Unspecified (1)[12]
Cognates:
Language contact:

***Ricinus communis* L. (Euphorbiaceae)**

Spanish names: Higuerilla
Indigenous names: Kaslam tatki/kasy la wängi/kasyla wong [01]; Tätz kin/ tzäksy kin ay[02]; Nuku tsoy[03]; Tsak tsooy[04]; Kastalan quajne[05]; Thikeela'[07]; K'axtelenkh'et[08]; Xk'ooch[09]; ŠkotČ[10]; Ix k'o'och[11]; Azeta[12]; Acete[13]; Ch'apaky[18]; Yaga huegu'u[21]; Baláp[22]; Yàg-blàp[23]; Xepowiwtl[28]
Used by (21*): Zoque[01, 02, 03]; Mixe[04]; Totonac[05, 06]; Huastec[07, 08]; Yucatecan Maya[09, 10, 11]; Quichean Maya[12, 13]; Western Maya[18]; Zapotec[21, 22, 23]; Nahua[25, 26, 27, 28]
Used for (65[#]): Blood (1)[12]; Digestive (17)[01, 02, 04, 05, 06, 07, 08, 09, 12, 18, 21, 22, 23, 26, 26, 27, 28]; Eye (1)[13]; Cardiovascular (1)[02]; Musculoskeletal (7)[01, 02, 03, 12, 13, 23, 25]; Neurological (6)[07, 08, 09, 10, 13, 22]; Respiratory (5)[02, 07, 25, 26, 27]; Skin (6)[01, 02, 03, 07, 21, 22]; Urological (1)[02]; Pregnancy (3)[01, 07, 13]; Female genital (2)[01, 18]; General and Unspecified (15)[01, 03, 04, 05, 07, 09, 10, 11, 12, 13, 18, 22, 25, 27, 28]
Cognates: MZ: tat/k;
Language contact: MZ <> Yuc; Chiapas Zoq <> Hua and Tot

***Rivina humilis* L. (Phytolaccaceae)**

Spanish names: Chilio, colario
Indigenous names: Yawa niwi [01]; Masan ay[03]; Pinil štayat[05]; Taa' t'ele', ts'amuxlaab ts'ohool, luuk, tsakam tsak tuthay, tsakam k'ak'al xeklek[07]; Ikiche'[09]; Baq'nel pim[14]
Used by (6*): Zoque[01, 03]; Totonac[05]; Huastec[07]; Yucatecan Maya[09]; Quichean Maya[14]
Used for (19[#]): Digestive (4)[03, 05, 07, 14]; Cardiovascular (1)[14]; Musculoskeletal (2)[03, 14]; Neurological (1)[14]; Psychological (1)[14]; Respiratory (1)[14]; Skin (4)[03, 07, 09, 14]; Urological (1)[01]; Pregnancy (1)[01]; General and Unspecified (3)[05, 07, 14]
Cognates:
Language contact:

***Roldana oaxacana* (Hemsl.) H.Rob. & Brettell (Asteraceae)**

Spanish names:
Indigenous names: Tzyaptos[01]
Used by (1*): Zoque[01]
Used for (1[#]): Respiratory (1)[01]
Cognates:
Language contact:

***Roldana petasitis* (Sims) H.Rob. & Brettell (Asteraceae)**

Spanish names: Hierba / Hoja de Queso
Indigenous names: Sup[12]
Used by (1*): Quichean Maya[12]
Used for (2[#]): Respiratory (1)[12]; General and Unspecified (1)[12]
Cognates:
Language contact:

***Rollinia mucosa* (Jacq.) Baill. (Annonaceae)**

Spanish names: Anona
Indigenous names: Paj yatyi[03]
Used by (1*): Zoque[03]
Used for (1[#]): Digestive (1)[03]
Cognates:
Language contact:

***Ronabea latifolia* Aubl. (Rubiaceae)**

Spanish names:
Indigenous names: Ix k'ä anal macho[11]
Used by (1*): Yucatecan Maya[11]
Used for (1#): Digestive (1)[11]
Cognates:
Language contact:

***Rosa alba* L. (Rosaceae)**

Spanish names: Rosa blanca
Indigenous names: Nich i blanca[18]
Used by (1*): Western Maya[18]
Used for (1#): General and Unspecified (1)[18]
Cognates:
Language contact:

***Rosa centifolia* L. (Rosaceae)**

Spanish names: Isabelita; Rosa blanca/ de castilla; Rosa de castill; Rosa de castilla
Indigenous names:
Used by (4*): Western Maya[18]; Zapotec[21, 22]; Nahua[27]
Used for (11#): Digestive (3)[21, 22, 27]; Eye (3)[21, 22, 27]; Respiratory (1)[18]; Skin (1)[27]; Female genital (1)[21]; General and Unspecified (2)[21, 22]
Cognates:
Language contact:

***Rosa chinensis* Jacq. (Rosaceae)**

Spanish names: Rosa
Indigenous names: Tzapas jäyä, Popo jäyä[01]; Ran xux, arxux, anaxux[13]; Nich i roja[18]
Used by (6*): Zoque[01, 02]; Yucatecan Maya[09]; Quichean Maya[13]; Western Maya[18]; Nahua[26]
Used for (27#): Digestive (3)[01, 02, 26]; Eye (3)[01, 02, 13]; Ear (1)[01]; Musculoskeletal (2)[01, 02]; Psychological (2)[01, 18]; Respiratory (5)[01, 02, 09, 18, 26]; Skin (2)[02, 13]; Urological (1)[02]; Pregnancy (1)[01]; Female genital (1)[02]; Male genital (1)[02]; General and Unspecified (5)[01, 02, 13, 18, 26]
Cognates:
Language contact:

***Rosa gallica* L. (Rosaceae)**

Spanish names: Rosa de castill
Indigenous names: Nich i castilla[18]
Used by (1*): Western Maya[18]
Used for (1#): Eye (1)[18]
Cognates:
Language contact:

***Rosa moschata* Herrm. (Rosaceae)**

Spanish names: Flor de concha, rosa concha
Indigenous names: U nich pat[18]
Used by (1*): Western Maya[18]
Used for (5#): Eye (1)[18]; Neurological (1)[18]; Psychological (1)[18]; Respiratory (1)[18]; General and Unspecified (1)[18]
Cognates:
Language contact:

[01-28]refer to the study codes in Table 4.1. *Total number of studies citing this taxon #Total number of use-records

***Rosa* sp. (Rosaceae)**

Spanish names: Rosa Blanca; Rosa concha
Indigenous names: Gloorya wits, k'iith wits[07]; Guièe-rôs[23]
Used by (4*): Zoque[03]; Huastec[07]; Quichean Maya[12]; Zapotec[23]
Used for (18#): Digestive (2)[03, 23]; Eye (1)[03]; Neurological (3)[03, 03, 12]; Psychological (2)[03, 12]; Respiratory (2)[03, 07]; Skin (2)[03, 23]; Pregnancy (1)[23]; Female genital (1)[03]; General and Unspecified (4)[03, 07, 12, 23]
Cognates:
Language contact:

***Roseodendron donnell-smithii* (Rose) Miranda (Bignoniaceae)**

Spanish names: Primavera
Indigenous names:
Used by (1*): Zoque[01]
Used for (1#): Musculoskeletal (1)[01]
Cognates:
Language contact:

***Rosmarinus officinalis* L. (Lamiaceae)**

Spanish names: Romero
Indigenous names:
Used by (16*): Zoque[01, 02, 03]; Mixe[04]; Totonac[05]; Huastec[08]; Yucatecan Maya[09]; Quichean Maya[12, 13, 14]; Western Maya[19]; Zapotec[21, 22, 23]; Nahua[25, 28]
Used for (50#): Blood (1)[12]; Digestive (8)[01, 02, 08, 12, 21, 22, 25, 28]; Cardiovascular (1)[12]; Musculoskeletal (5)[02, 03, 08, 12, 23]; Neurological (1)[12]; Psychological (2)[03, 21]; Respiratory (5)[01, 02, 12, 25, 28]; Skin (4)[01, 08, 12, 25]; Urological (1)[12]; Pregnancy (8)[01, 02, 03, 09, 19, 21, 22, 23]; Female genital (4)[01, 02, 12, 19]; General and Unspecified (10)[01, 04, 05, 08, 12, 13, 14, 22, 23, 28]
Cognates:
Language contact:

***Roupala montana* Aubl. (Proteaceae)**

Spanish names: Palo de cucaracha
Indigenous names: T¥kchicuy[03]
Used by (1*): Zoque[03]
Used for (1#): Female genital (1)[03]
Cognates:
Language contact:

***Rourea glabra* Kunth (Connaraceae)**

Spanish names:
Indigenous names: It'iib chuch[07]
Used by (1*): Huastec[07]
Used for (4#): Musculoskeletal (1)[07]; Neurological (1)[07]; Skin (1)[07]; General and Unspecified (1)[07]
Cognates:
Language contact:

***Rubus adenotrichus* Schltdl. (Rosaceae)**

Spanish names: Mora; Mora simarron/mora agria
Indigenous names: (Katzu) watzan[01]; Tocan[12]; Makom, makum[20]
Used by (3*): Zoque[01]; Quichean Maya[12]; Western Maya[20]
Used for (9#): Digestive (2)[01, 20]; Musculoskeletal (1)[01]; Psychological (1)[01]; Respiratory (2)[12, 20]; General and Unspecified (3)[01, 12, 20]
Cognates:
Language contact:

***Rubus coriifolius* Liebm. (Rosaceae)**

Spanish names:
Indigenous names: Makom, makum[20]
Used by (1*): Western Maya[20]
Used for (3#): Digestive (1)[20]; Respiratory (1)[20]; General and Unspecified (1)[20]
Cognates:
Language contact:

***Rubus sapidus* Schltdl. (Rosaceae)**

Spanish names: Zarzamora
Indigenous names: Watzan[01]
Used by (1*): Zoque[01]
Used for (3#): Digestive (1)[01]; Urological (1)[01]; Male genital (1)[01]
Cognates:
Language contact:

***Rubus* sp. (Rosaceae)**

Spanish names: Sarsamora
Indigenous names: Tucán[13]
Used by (1*): Quichean Maya[13]
Used for (4#): Digestive (1)[13]; Eye (1)[13]; Respiratory (1)[13]; General and Unspecified (1)[13]
Cognates:
Language contact:

***Ruellia inundata* Kunth (Acanthaceae)**

Spanish names: Hierba de chivo/ barba de chivo
Indigenous names: Tzivo angbäk[01]
Used by (1*): Zoque[01]
Used for (4#): Musculoskeletal (1)[01]; Neurological (1)[01]; Respiratory (1)[01]; General and Unspecified (1)[01]
Cognates:
Language contact:

***Ruellia jussieuoides* Schltdl. (Acanthaceae)**

Spanish names:
Indigenous names: Tsus ay[03]
Used by (1*): Zoque[03]
Used for (1#): Digestive (1)[03]
Cognates:
Language contact:

***Ruellia megasphaera* Lindau (Acanthaceae)**

Spanish names:
Indigenous names: Poj ujts[04]
Used by (1*): Mixe[04]
Used for (1#): General and Unspecified (1)[04]
Cognates:
Language contact:

***Ruellia nudiflora* (Engelm. & A.Gray) Urb. (Acanthaceae)**

Spanish names:
Indigenous names: Kabalya'axnik[09]
Used by (1*): Yucatecan Maya[09]
Used for (2#): Eye (1)[09]; Urological (1)[09]
Cognates:
Language contact:

[01-28]refer to the study codes in Table 4.1. *Total number of studies citing this taxon #Total number of use-records

***Ruellia simplex* C.Wright (Acanthaceae)**

Spanish names:
Indigenous names: Tsab k'a'um, pithomlaab ts'ohool, tsakam tsahib, tok ts'ohool, ebchil k'a'um, ts'itsiimbe ts'ohool, bo' k'a'um[07]
Used by (1*): Huastec[07]
Used for (2#): Neurological (1)[07]; General and Unspecified (1)[07]
Cognates:
Language contact:

***Ruellia* sp. (Acanthaceae)**

Spanish names: Cuamaite
Indigenous names: Lisakan[05]; Tsamnek muu, pohoth ts'ohool, eem muuw, xutsun bat'aw, uxum kw'ahiilom[07]
Used by (3*): Zoque[03]; Totonac[05]; Huastec[07]
Used for (6#): Blood (1)[07]; Digestive (1)[03]; Neurological (1)[07]; Skin (1)[03]; Pregnancy (1)[05]; General and Unspecified (1)[07]
Cognates:
Language contact:

***Rumex crispus* L. (Polygonaceae)**

Spanish names: Lengua de vaca
Indigenous names: Paasii' ma kaa't[06]; Raqwax[12]
Used by (2*): Totonac[06]; Quichean Maya[12]
Used for (5#): Digestive (1)[06]; Skin (1)[12]; Urological (1)[12]; Female genital (1)[12]; General and Unspecified (1)[12]
Cognates:
Language contact:

***Rumex obtusifolius* L. (Polygonaceae)**

Spanish names: Lengua de vaca; Lengua de Vaca de Agua
Indigenous names: Aq'om Raq' Wakx[12]; Xocoquilitl[26]
Used by (2*): Quichean Maya[12]; Nahua[26]
Used for (4#): Digestive (1)[12]; Skin (2)[12, 26]; Urological (1)[26]
Cognates:
Language contact:

***Rumex* sp. (Polygonaceae)**

Spanish names: Lengua de vaca; Verdolaga
Indigenous names: Raq' wak'as[13]; Blàg-dòoz[23]
Used by (2*): Quichean Maya[13]; Zapotec[23]
Used for (6#): Blood (1)[13]; Musculoskeletal (1)[23]; Neurological (1)[23]; Skin (1)[23]; Pregnancy (1)[13]; General and Unspecified (1)[23]
Cognates:
Language contact:

***Russelia coccinea* (L.) Wettst. (Plantaginaceae)**

Spanish names: Barra de San Jose
Indigenous names:
Used by (1*): Zoque[01]
Used for (1#): Digestive (1)[01]
Cognates:
Language contact:

[01-28]refer to the study codes in Table 4.1. *Total number of studies citing this taxon #Total number of use-records

***Russelia equisetiformis* Schltdl. & Cham. (Plantaginaceae)**

Spanish names:
Indigenous names: Kutsiilte', kwayab an San Husee, thoot wits, toom wits, weeu koox[07]
Used by (1*): Huastec[07]
Used for (1#): General and Unspecified (1)[07]
Cognates:
Language contact:

***Russelia sarmentosa* Jacq. (Plantaginaceae)**

Spanish names: Hoja de cerilla; Tronadora
Indigenous names: Kiñi n¥pin mooya[03]; Aneeymats[04]; Kwayab ts'aale, kutsiilte', ook' ts'een ts'ohool[07]; Siik'xiw, Oxletk'ax[09]; guish crii[21]
Used by (5*): Zoque[03]; Mixe[04]; Huastec[07]; Yucatecan Maya[09]; Zapotec[21]
Used for (11#): Digestive (2)[03, 04]; Skin (3)[03, 09, 21]; Endocrine (1)[03]; Urological (1)[03]; Female genital (2)[03, 07]; General and Unspecified (2)[04, 07]
Cognates: Mayan: sii;
Language contact: Maya <> Zap

***Ruta chalepensis* L. (Rutaceae)**

Spanish names: Ruda
Indigenous names: Rurá[13]; Tujyan ixik[19]; Temalacatl[28]
Used by (11*): Totonac[06]; Yucatecan Maya[09]; Quichean Maya[12, 13]; Western Maya[18, 19]; Zapotec[21]; Nahua[25, 26, 27, 28]
Used for (58#): Blood (1)[12]; Digestive (9)[06, 09, 12, 13, 18, 25, 26, 27, 28]; Eye (1)[21]; Ear (1)[13]; Cardiovascular (5)[12, 13, 18, 21, 25]; Musculoskeletal (3)[18, 19, 28]; Neurological (9)[09, 12, 13, 18, 19, 21, 25, 27, 28]; Psychological (4)[13, 18, 19, 27]; Respiratory (4)[18, 19, 25, 27]; Skin (2)[13, 18]; Pregnancy (5)[06, 13, 18, 25, 26]; Female genital (4)[06, 12, 13, 18]; General and Unspecified (10)[06, 12, 13, 18, 19, 21, 25, 26, 27, 28]
Cognates:
Language contact:

***Ruta graveolens* L. (Rutaceae)**

Spanish names: Ruda
Indigenous names: Wits'ii te' ts'ojol[08]; Lula[20]
Used by (5*): Mixe[04]; Huastec[08]; Yucatecan Maya[11]; Western Maya[20]; Zapotec[22]
Used for (20#): Digestive (4)[04, 08, 20, 22]; Ear (2)[04, 08]; Musculoskeletal (2)[08, 22]; Neurological (2)[08, 22]; Psychological (2)[08, 22]; Respiratory (1)[08]; Skin (1)[08]; Pregnancy (2)[04, 22]; Female genital (1)[04]; General and Unspecified (3)[08, 11, 22]
Cognates:
Language contact:

***Ruta* sp. (Rutaceae)**

Spanish names: Ruda
Indigenous names: luta[01]; Rrûd[23]
Used by (4*): Zoque[01, 02, 03]; Zapotec[23]
Used for (31#): Blood (1)[01]; Digestive (4)[01, 02, 03, 23]; Eye (1)[01]; Ear (2)[01, 02]; Cardiovascular (3)[01, 02, 23]; Musculoskeletal (3)[01, 02, 03]; Neurological (1)[01]; Psychological (4)[01, 02, 03, 23]; Respiratory (1)[01]; Skin (4)[01, 02, 03, 23]; Urological (1)[02]; Pregnancy (2)[02, 03]; Female genital (1)[01]; General and Unspecified (3)[01, 03, 23]
Cognates:
Language contact:

***Rytidostylis gracilis* Hook. & Arn. (Cucurbitaceae)**

Spanish names: Hoja de carga
Indigenous names: Tzämi ay[01]
Used by (1*): Zoque[01]
Used for (1#): General and Unspecified (1)[01]
Cognates:
Language contact:

[01-28]refer to the study codes in Table 4.1. *Total number of studies citing this taxon #Total number of use-records

***Sabal mexicana* Mart. (Arecaceae)**

Spanish names: Cocobal; Palma real
Indigenous names: Jojo[02]; Yopo'jojobal[18]; Xi'inaaj[21]
Used by (3*): Zoque[02]; Western Maya[18]; Zapotec[21]
Used for (4#): Digestive (1)[18]; Musculoskeletal (1)[21]; Skin (1)[21]; Pregnancy (1)[02]
Cognates:
Language contact: Chimalapa Zoq <> Chontal

***Sabal* sp. (Arecaceae)**

Spanish names: Guano
Indigenous names: Ka'nal-xa'an[09]
Used by (1*): Yucatecan Maya[09]
Used for (2#): Pregnancy (1)[09]; Female genital (1)[09]
Cognates:
Language contact:

***Sabicea* sp. (Rubiaceae)**

Spanish names:
Indigenous names: Colaras[16]
Used by (1*): Quichean Maya[16]
Used for (1#): Neurological (1)[16]
Cognates:
Language contact:

***Sabicea villosa* Willd. ex Schult. (Rubiaceae)**

Spanish names:
Indigenous names: T'u zub k'aham[17]
Used by (1*): Quichean Maya[17]
Used for (1#): nd[17]
Cognates:
Language contact:

***Saccharum officinarum* L. (Poaceae)**

Spanish names: Caña de azúcar
Indigenous names: Cha'ncat[05]; Cha'ncat[06]; Pakab[07]
Used by (3*): Totonac[05, 06]; Huastec[07]
Used for (6#): Digestive (2)[06, 07]; Neurological (1)[05]; Respiratory (1)[05]; Skin (1)[05]; General and Unspecified (1)[06]
Cognates:
Language contact:

***Sageretia elegans* (Kunth) Brongn. (Rhamnaceae)**

Spanish names: Espina de corona
Indigenous names: Masan kobak apitx[03]
Used by (2*): Zoque[01, 03]
Used for (5#): Digestive (1)[03]; Endocrine (1)[01]; Urological (1)[03]; Female genital (1)[03]; General and Unspecified (1)[03]
Cognates:
Language contact:

***Salix humboldtiana* Willd. (Salicaceae)**

Spanish names: Sauce
Indigenous names: Weksya[02]; Nø aweey[04]; Tok'oy, hili te'[07]; Sk'os[13]; Te'aj sausal[18]
Used by (5*): Zoque[02]; Mixe[04]; Huastec[07]; Quichean Maya[13]; Western Maya[18]
Used for (9#): Neurological (1)[18]; Psychological (2)[02, 07]; Respiratory (1)[02]; Skin (1)[04]; Urological (1)[02]; General and Unspecified (3)[02, 07, 13]
Cognates:
Language contact:

[01-28]refer to the study codes in Table 4.1. *Total number of studies citing this taxon #Total number of use-records

***Salix* sp. (Salicaceae)**

Spanish names: Sauce
Indigenous names: Skab'ya'[12]; Yàg-zhguiès[23]
Used by (3*): Quichean Maya[12]; Zapotec[21, 23]
Used for (15#): Digestive (1)[21]; Cardiovascular (1)[12]; Musculoskeletal (3)[12, 21, 23]; Neurological (1)[12]; Psychological (1)[23]; Skin (2)[21, 23]; Endocrine (1)[23]; Pregnancy (1)[23]; Female genital (1)[12]; General and Unspecified (3)[12, 21, 23]
Cognates:
Language contact:

***Salix taxifolia* Kunth (Salicaceae)**

Spanish names: Taray
Indigenous names: Mak'astakat[06]
Used by (1*): Totonac[06]
Used for (1#): Urological (1)[06]
Cognates:
Language contact:

***Salmea oligocephala* Hemsl. (Asteraceae)**

Spanish names: Rayanita
Indigenous names: Nas mupe[02]
Used by (1*): Zoque[02]
Used for (1#): Digestive (1)[02]
Cognates:
Language contact:

***Salmea scandens* (L.) DC. (Asteraceae)**

Spanish names: Palo de chile
Indigenous names: Ix ts'aah, , it'iib to'ol, ix ch'aah[07]; Yàg-guìin, guìzh-yàg-guìin [23]
Used by (2*): Huastec[07]; Zapotec[23]
Used for (4#): Neurological (1)[07]; Respiratory (1)[07]; Skin (1)[07]; General and Unspecified (1)[23]
Cognates:
Language contact:

***Salmea* sp. (Asteraceae)**

Spanish names: Palo de chile
Indigenous names: Yagaguina'a[21]
Used by (1*): Zapotec[21]
Used for (3#): Respiratory (1)[21]; Skin (1)[21]; General and Unspecified (1)[21]
Cognates:
Language contact:

***Salpianthus arenarius* Bonpl. (Nyctaginaceae)**

Spanish names: Pie de paloma
Indigenous names:
Used by (1*): Zoque[02]
Used for (4#): Digestive (1)[02]; Musculoskeletal (1)[02]; Female genital (1)[02]; General and Unspecified (1)[02]
Cognates:
Language contact:

***Salpianthus macrodontus* Standl. (Nyctaginaceae)**

Spanish names: Pie de paloma
Indigenous names: Guish pileej[21]
Used by (1*): Zapotec[21]
Used for (1#): General and Unspecified (1)[21]
Cognates:
Language contact:

**_Salvia breviflora_ Moc. & Sessé ex Benth. (Lamiaceae)**

Spanish names:
Indigenous names: Ncuàan-zân-làs[23]
Used by (1*): Zapotec[23]
Used for (1#): Pregnancy (1)[23]
Cognates:
Language contact:

**_Salvia carnea_ Kunth (Lamiaceae)**

Spanish names:
Indigenous names: Tepechichia[26]
Used by (1*): Nahua[26]
Used for (1#): Digestive (1)[26]
Cognates:
Language contact:

**_Salvia cinnabarina_ M.Martens & Galeotti (Lamiaceae)**

Spanish names: Flor de gorrión, salvia
Indigenous names: Tatz'na'[13]
Used by (2*): Quichean Maya[12, 13]
Used for (4#): Musculoskeletal (2)[12, 13]; Skin (1)[13]; Female genital (1)[13]
Cognates:
Language contact:

**_Salvia circinnata_ Cav. (Lamiaceae)**

Spanish names: Verbena
Indigenous names: Zeh la[22]
Used by (1*): Zapotec[22]
Used for (3#): Psychological (1)[22]; Skin (1)[22]; Pregnancy (1)[22]
Cognates:
Language contact:

**_Salvia coccinea_ Buc'hoz ex Etl. (Lamiaceae)**

Spanish names: Mirto
Indigenous names: Hut'ut' wits, uxum ts'ohool, wayma', witsal a k'iitsaa[07]; Chaktsits[09]; Chäk ta pek'[11]
Used by (6*): Zoque[03]; Huastec[07]; Yucatecan Maya[09, 11]; Quichean Maya[12]; Nahua[25]
Used for (13#): Digestive (3)[07, 09, 25]; Eye (1)[07]; Psychological (1)[07]; Skin (1)[12]; Endocrine (1)[12]; Urological (1)[12]; Pregnancy (1)[07]; Female genital (1)[07]; General and Unspecified (3)[03, 11, 12]
Cognates: Yuca: chakt;
Language contact:

**_Salvia filipes_ Benth. (Lamiaceae)**

Spanish names:
Indigenous names: Tepechichia, ixcaxihuitl[26]
Used by (1*): Nahua[26]
Used for (2#): Digestive (1)[26]; General and Unspecified (1)[26]
Cognates:
Language contact:

**_Salvia herbacea_ Benth. (Lamiaceae)**

Spanish names:
Indigenous names: Møj ku'uk ujts[04]
Used by (1*): Mixe[04]
Used for (1#): Skin (1)[04]
Cognates:
Language contact:

[01-28]refer to the study codes in Table 4.1. *Total number of studies citing this taxon #Total number of use-records

***Salvia lavanduloides* Kunth (Lamiaceae)**

Spanish names: Alucema, hierba del ciervo, flor azul; Alusema
Indigenous names: Ajob q'yes[13]; Lùsêm[23]
Used by (2*): Quichean Maya[13]; Zapotec[23]
Used for (9#): Digestive (2)[13, 23]; Cardiovascular (1)[13]; Respiratory (1)[13]; Skin (1)[13]; Pregnancy (1)[13]; Female genital (1)[13]; General and Unspecified (2)[13, 23]
Cognates:
Language contact:

***Salvia leucantha* Cav. (Lamiaceae)**

Spanish names: Salvia morada
Indigenous names: Tochimixochitl[28]
Used by (1*): Nahua[28]
Used for (2#): Digestive (1)[28]; General and Unspecified (1)[28]
Cognates:
Language contact:

***Salvia mexicana* L. (Lamiaceae)**

Spanish names: Tlanchichinole
Indigenous names: Tlanchichinole[25]
Used by (1*): Nahua[25]
Used for (3#): Digestive (1)[25]; Skin (1)[25]; Urological (1)[25]
Cognates:
Language contact:

***Salvia microphylla* Kunth (Lamiaceae)**

Spanish names: Mirto
Indigenous names: Makakašahuat[05]
Used by (3*): Totonac[05]; Quichean Maya[13]; Zapotec[23]
Used for (13#): Digestive (3)[05, 13, 23]; Ear (1)[05]; Musculoskeletal (1)[13]; Respiratory (1)[05]; Pregnancy (3)[05, 13, 23]; Female genital (2)[13, 23]; General and Unspecified (2)[05, 23]
Cognates:
Language contact:

***Salvia polystachia* Cav. (Lamiaceae)**

Spanish names: Salvia
Indigenous names:
Used by (1*): Zoque[01]
Used for (1#): Digestive (1)[01]
Cognates:
Language contact:

***Salvia* sp. (Lamiaceae)**

Spanish names: Alúcema; Contrahierba; Limpia
Indigenous names: Xiax-k'ax, Chi-k'ak'[09]; Guièe-dzǐn̲g, gùìzh-dzǐn̲g, gùìzh-guièe-dzǐn̲g, guièe-měets[23]
Used by (4*): Yucatecan Maya[09]; Quichean Maya[12]; Zapotec[21, 23]
Used for (10#): Digestive (1)[12]; Neurological (1)[12]; Respiratory (2)[09, 12]; Skin (3)[09, 21, 23]; Female genital (1)[12]; General and Unspecified (2)[09, 23]
Cognates:
Language contact:

[01-28]refer to the study codes in Table 4.1. *Total number of studies citing this taxon #Total number of use-records

***Salvia tiliifolia* Vahl (Lamiaceae)**

Spanish names:
Indigenous names: Tzuji ay[01]; Blàg-guìtsiè, xín-lùzêm[23]
Used by (2*): Zoque[01]; Zapotec[23]
Used for (3#): Digestive (1)[01]; Psychological (1)[23]; Skin (1)[23]
Cognates:
Language contact:

***Salvia tuxtlensis* Ramamoorthy (Lamiaceae)**

Spanish names:
Indigenous names: Poja way cuy[03]
Used by (1*): Zoque[03]
Used for (4#): Digestive (1)[03]; Respiratory (1)[03]; Skin (1)[03]; Female genital (1)[03]
Cognates:
Language contact:

***Salvia urica* Epling (Lamiaceae)**

Spanish names: Albahaca silvestre
Indigenous names:
Used by (1*): Quichean Maya[12]
Used for (4#): Digestive (1)[12]; Female genital (1)[12]; Male genital (1)[12]; General and Unspecified (1)[12]
Cognates:
Language contact:

***Salvia xalapensis* Benth. (Lamiaceae)**

Spanish names:
Indigenous names: Tsus ay[03]; Pategen ujts[04]
Used by (2*): Zoque[03]; Mixe[04]
Used for (2#): Digestive (1)[03]; Skin (1)[04]
Cognates:
Language contact:

***Sambucus canadensis* L. (Adoxaceae)**

Spanish names: Saúco
Indigenous names: Okok yui/ok yui/ä'ju rane[01]; Toxeem[04]; Toxiba, tokxihua, toqsiwi, toquiwa[05]; Sawko[07]; Tunay' che', tzolo'j che', Tunayche' Aq'om[12]; Zoloji, shiij, tz'oloj che', xubam[13]; Yop'aj sauk'u[18]; Ch'ijil te'[20]; Yàg-saûz, yàzmîn[23]; Xometl[26]; Xometl[28]
Used by (16*): Zoque[01, 02, 03]; Mixe[04]; Totonac[05]; Huastec[07]; Quichean Maya[12, 13]; Western Maya[18, 20]; Zapotec[21, 22, 23]; Nahua[25, 26, 28]
Used for (75#): Blood (2)[01, 03]; Digestive (8)[01, 02, 03, 05, 13, 20, 25, 26]; Eye (3)[01, 21, 22]; Cardiovascular (2)[12, 25]; Musculoskeletal (6)[01, 02, 05, 12, 13, 21]; Neurological (5)[01, 02, 03, 12, 25]; Psychological (3)[01, 05, 18]; Respiratory (14)[01, 02, 03, 04, 05, 07, 12, 13, 18, 23, 25, 25, 26, 28]; Skin (9)[01, 02, 03, 05, 12, 13, 21, 22, 28]; Urological (3)[02, 12, 13]; Pregnancy (2)[05, 13]; Female genital (5)[02, 03, 12, 13, 21]; Male genital (2)[02, 12]; General and Unspecified (11)[01, 02, 03, 05, 12, 13, 18, 21, 25, 26, 28]
Cognates: CoreM: shiij/ch'ichil; Quich: tzoloj; Nahua: xometl;
Language contact:

***Samolus ebracteatus* Kunth (Primulaceae)**

Spanish names:
Indigenous names: Tsunya'hi[09]
Used by (1*): Yucatecan Maya[09]
Used for (3#): Musculoskeletal (1)[09]; Respiratory (1)[09]; Skin (1)[09]
Cognates:
Language contact:

[01-28]refer to the study codes in Table 4.1. *Total number of studies citing this taxon #Total number of use-records

***Samolus floribundus* Kunth (Primulaceae)**

Spanish names:
Indigenous names: Ncuàan-dzéb-nquĭts[23]
Used by (1*): Zapotec[23]
Used for (1#): General and Unspecified (1)[23]
Cognates:
Language contact:

***Samyda yucatanensis* Standl. (Salicaceae)**

Spanish names:
Indigenous names: Naranja che'[09]
Used by (1*): Yucatecan Maya[09]
Used for (2#): Skin (1)[09]; Endocrine (1)[09]
Cognates:
Language contact:

***Sanchezia parvibracteata* Sprague & Hutch. (Acanthaceae)**

Spanish names: Pavo Real
Indigenous names: Xjolom Chaqmut (Sak)[14]
Used by (2*): Quichean Maya[12, 14]
Used for (2#): Musculoskeletal (1)[12]; General and Unspecified (1)[14]
Cognates:
Language contact:

***Sansevieria hyacinthoides* (L.) Druce (Asparagaceae)**

Spanish names: Curarina
Indigenous names:
Used by (3*): Yucatecan Maya[11]; Quichean Maya[13]; Western Maya[19]
Used for (9#): Digestive (1)[13]; Cardiovascular (1)[13]; Neurological (2)[13, 19]; Skin (2)[13, 19]; General and Unspecified (3)[11, 13, 19]
Cognates:
Language contact:

***Sansevieria trifasciata* Prain (Asparagaceae)**

Spanish names: Curalina; Curarina
Indigenous names: Tzikin o [01]; Txikiña[03]
Used by (4*): Zoque[01, 02, 03]; Quichean Maya[12]
Used for (6#): Digestive (1)[01]; Skin (4)[01, 02, 03, 12]; General and Unspecified (1)[12]
Cognates: Zoq: tsikin a/o;
Language contact:

***Sansevieria zeylanica* (L.) Willd. (Asparagaceae)**

Spanish names: Cola de tigre
Indigenous names: Nej baläm[18]
Used by (1*): Western Maya[18]
Used for (1#): Musculoskeletal (1)[18]
Cognates:
Language contact:

***Sanvitalia procumbens* Lam. (Asteraceae)**

Spanish names: Hoja de azar
Indigenous names: Tza'a tzoy[01]
Used by (1*): Zoque[01]
Used for (1#): General and Unspecified (1)[01]
Cognates:
Language contact:

***Sapindus saponaria* L. (Sapindaceae)**

Spanish names: Jaboncillo
Indigenous names: Tsukma[03]; Walul[07]; Bijpiij[21]
Used by (3*): Zoque[03]; Huastec[07]; Zapotec[21]
Used for (5#): Digestive (1)[07]; Skin (2)[03, 21]; General and Unspecified (2)[07, 21]
Cognates:
Language contact:

***Sapranthus campechianus* (Kunth) Standl. (Annonaceae)**

Spanish names:
Indigenous names: Chuyu chajum,sakelemuy[09]
Used by (1*): Yucatecan Maya[09]
Used for (1#): Urological (1)[09]
Cognates:
Language contact:

***Sarcoglottis acaulis* (Sm.) Schltr. (Orchidaceae)**

Spanish names:
Indigenous names: Ts'ik'aach ts'ohool[07]
Used by (1*): Huastec[07]
Used for (1#): Pregnancy (1)[07]
Cognates:
Language contact:

***Saurauia oreophila* Hemsl. (Actinidiaceae)**

Spanish names: Llorasangre hembra
Indigenous names: Q'ayis Aq'om Kik' Ixoq[12]
Used by (1*): Quichean Maya[12]
Used for (8#): Digestive (1)[12]; Cardiovascular (1)[12]; Musculoskeletal (1)[12]; Neurological (1)[12]; Respiratory (1)[12]; Endocrine (1)[12]; Urological (1)[12]; General and Unspecified (1)[12]
Cognates:
Language contact:

***Schinus molle* L. (Anacardiaceae)**

Spanish names: Coabino, pirul; Pirú; Pirul
Indigenous names: Ya luj[22]; Yàg-pìrûl[23]; Peloncuáhuitl[28]
Used by (5*): Zoque[02]; Zapotec[22, 23]; Nahua[27, 28]
Used for (12#): Digestive (1)[22]; Eye (1)[22]; Musculoskeletal (1)[23]; Neurological (1)[22]; Respiratory (1)[28]; Skin (1)[22]; Pregnancy (2)[22, 27]; General and Unspecified (4)[02, 22, 27, 28]
Cognates:
Language contact:

***Schistocarpha eupatorioides* (Fenzl) Kuntze (Asteraceae)**

Spanish names:
Indigenous names: Pänpäm[01]; Sununkil Q'ehen, Jolam Pek Q'ehen[14]
Used by (3*): Zoque[01, 03]; Quichean Maya[14]
Used for (5#): Musculoskeletal (2)[01, 14]; Neurological (1)[14]; Respiratory (1)[14]; Skin (1)[03]
Cognates:
Language contact:

[01-28]refer to the study codes in Table 4.1.  *Total number of studies citing this taxon  #Total number of use-records

***Schkuhria pinnata*** **(Lam.) Kuntze ex Thell. (Asteraceae)**

Spanish names: Hierba del vendao
Indigenous names: Its'amal ts'ojol[08]; Gbày[23]
Used by (2*): Huastec[08]; Zapotec[23]
Used for (5#): Digestive (1)[08]; Musculoskeletal (1)[08]; Neurological (1)[08]; Skin (2)[08, 23]
Cognates:
Language contact:

***Schoenocaulon officinale*** **(Schltdl. & Cham.) A.Gray (Melanthiaceae)**

Spanish names:
Indigenous names: Tsuk tyiñ[03]; Sebadiya[07]
Used by (2*): Zoque[03]; Huastec[07]
Used for (2#): Skin (2)[03, 07]
Cognates:
Language contact:

***Schoepfia schreberi*** **J.F.Gmel. (Schoepfiaceae)**

Spanish names:
Indigenous names: Its'am te', yax ich'am te'[07]
Used by (1*): Huastec[07]
Used for (3#): Digestive (1)[07]; Neurological (1)[07]; Pregnancy (1)[07]
Cognates:
Language contact:

***Schrankia*** **sp. (Fabaceae)**

Spanish names:
Indigenous names: Tsakam tsobeem[07]
Used by (1*): Huastec[07]
Used for (2#): Neurological (1)[07]; Psychological (1)[07]
Cognates:
Language contact:

***Scindapsus pictus*** **Hassk. (Araceae)**

Spanish names:
Indigenous names: Kuax' Kay'[14]
Used by (1*): Quichean Maya[14]
Used for (3#): Neurological (1)[14]; Psychological (1)[14]; General and Unspecified (1)[14]
Cognates:
Language contact:

***Scleria bracteata*** **Cav. (Cyperaceae)**

Spanish names:
Indigenous names: Neiuk[03]
Used by (1*): Zoque[03]
Used for (2#): Digestive (1)[03]; Pregnancy (1)[03]
Cognates:
Language contact:

***Scleria distans*** **Poir. (Cyperaceae)**

Spanish names:
Indigenous names: Møøy[04]
Used by (1*): Mixe[04]
Used for (2#): Digestive (1)[04]; Female genital (1)[04]
Cognates:
Language contact:

[01-28]refer to the study codes in Table 4.1. *Total number of studies citing this taxon #Total number of use-records

***Scleria gaertneri* Raddi (Cyperaceae)**

Spanish names:
Indigenous names: T'oyol toom t'unu', kotox toom, tsakam k'iithath toom, tathiim toom[07]
Used by (1*): Huastec[07]
Used for (3#): Digestive (1)[07]; Skin (1)[07]; General and Unspecified (1)[07]
Cognates:
Language contact:

***Scleria lithosperma* (L.) Sw. (Cyperaceae)**

Spanish names:
Indigenous names: Xoknoon[09]
Used by (1*): Yucatecan Maya[09]
Used for (1#): Respiratory (1)[09]
Cognates:
Language contact:

***Scoparia dulcis* L. (Plantaginaceae)**

Spanish names: Cilandrillo; Cilantrillo; Escobillo
Indigenous names: Naax Podeey[04]; Kulantr pim[14]
Used by (5*): Zoque[03]; Mixe[04]; Quichean Maya[14, 15]; Western Maya[18]
Used for (9#): Digestive (3)[04, 14, 18]; Neurological (1)[03]; Skin (3)[03, 04, 14]; Pregnancy (1)[15]; Female genital (1)[03]
Cognates:
Language contact:

***Scutellaria seleriana* Loes. (Lamiaceae)**

Spanish names:
Indigenous names: Elbeenax ts'phool, waylom ts'ohool, chakam kuch, t'ot ich ch'ohool, chakam thoot[07]
Used by (1*): Huastec[07]
Used for (3#): Digestive (1)[07]; Cardiovascular (1)[07]; General and Unspecified (1)[07]
Cognates:
Language contact:

***Scutellaria* sp. (Lamiaceae)**

Spanish names: Orozuz
Indigenous names: Balsamo xiw[09]
Used by (1*): Yucatecan Maya[09]
Used for (1#): Digestive (1)[09]
Cognates:
Language contact:

***Sechium* edule (Jacq.) Sw. (Cucurbitaceae)**

Spanish names: Chayote, güisquil
Indigenous names: Awin pa'sun/apit pa'sun/ awit pa'sun/ pa'sun[01]; Apit[02]; Cuy pasun[03]; A'xaa[04]; Choyotesel, spup, malsh tu'kun[06]; Tsiw', chiw[07]; Pak'a Q'ix[12]; Q'ix[13]; Chijch'um[18]; Balagayaappa[21]; Chayote[25]
Used by (11*): Zoque[01, 02, 03]; Mixe[04]; Totonac[06]; Huastec[07]; Quichean Maya[12, 13]; Western Maya[18]; Zapotec[21]; Nahua[25]
Used for (24#): Digestive (3)[01, 04, 12]; Cardiovascular (3)[01, 06, 18]; Musculoskeletal (1)[03]; Psychological (1)[21]; Skin (4)[01, 02, 12, 13]; Endocrine (1)[25]; Urological (6)[01, 02, 03, 06, 07, 25]; Pregnancy (2)[01, 03]; General and Unspecified (3)[01, 03, 21]
Cognates: Zoq: aCit, pasun; Mayan: q'ix/chiw/chij; Quich: q'ix;
Language contact: Maya and Nah > Spanish

[01-28]refer to the study codes in Table 4.1. *Total number of studies citing this taxon #Total number of use-records

***Securidaca diversifolia*** **(L.) S.F.Blake (Polygalaceae)**

Spanish names: Balsamillo; Tripa de gallo
Indigenous names: Kipats ay[03]; Mamaal tsan, nuuk bichim, umuw tsaah, anuts bichim, , manil pet[07]; Se' ru' k'an tyaj; ch'up k'an tyaj[17]
Used by (4*): Zoque[02, 03]; Huastec[07]; Quichean Maya[17]
Used for (8#): Digestive (1)[03]; Musculoskeletal (1)[07]; Neurological (1)[07]; Respiratory (1)[02]; Skin (2)[03, 07]; General and Unspecified (1)[03]; nd[17]
Cognates:
Language contact:

***Sedum dendroideum*** **Moc. & Sessé ex DC. (Crassulaceae)**

Spanish names: Siempreviva
Indigenous names:
Used by (1*): Totonac[05]
Used for (1#): General and Unspecified (1)[05]
Cognates:
Language contact:

***Sedum morganianum*** **E.Walther (Crassulaceae)**

Spanish names: Cola de chivo
Indigenous names: Ujey chij[13]
Used by (1*): Quichean Maya[13]
Used for (1#): Eye (1)[13]
Cognates:
Language contact:

***Sedum praealtum*** **A.DC. (Crassulaceae)**

Spanish names:
Indigenous names: Guièe-yùzh[23]
Used by (1*): Zapotec[23]
Used for (1#): Skin (1)[23]
Cognates:
Language contact:

***Sedum*** **sp. (Crassulaceae)**

Spanish names:
Indigenous names: Yahualchit[25]
Used by (1*): Nahua[25]
Used for (3#): Skin (1)[25]; Female genital (1)[25]; General and Unspecified (1)[25]
Cognates:
Language contact:

***Selaginella convoluta*** **(Arn.) Spring (Selaginellaceae)**

Spanish names: Flor de piedra
Indigenous names: Mooch-tut[09]
Used by (1*): Yucatecan Maya[09]
Used for (1#): Urological (1)[09]
Cognates:
Language contact:

[01-28]refer to the study codes in Table 4.1. *Total number of studies citing this taxon #Total number of use-records

***Selaginella lepidophylla*** **(Hook. & Grev.) Spring (Selaginellaceae)**

Spanish names: Siempre viva, doradilla
Indigenous names:
Used by (1*): Nahua[27]
Used for (1#): Urological (1)[27]
Cognates:
Language contact:

***Selaginella pallescens*** **(C. Presl) Spring (Selaginellaceae)**

Spanish names: Hiedra de Piedra
Indigenous names: Kumatzin Q'os Abäj[12]
Used by (1*): Quichean Maya[12]
Used for (5#): Digestive (1)[12]; Skin (1)[12]; Female genital (1)[12]; Male genital (1)[12]; General and Unspecified (1)[12]
Cognates:
Language contact:

***Selaginella schizobasis*** **Baker (Selaginellaceae)**

Spanish names: Hoja vishu
Indigenous names:
Used by (1*): Zoque[02]
Used for (1#): Skin (1)[02]
Cognates:
Language contact:

***Selaginella*** **sp. (Selaginellaceae)**

Spanish names: Siempreviva, doradilla
Indigenous names: Chuklaab ts'ohool, tsakam ts'uh, muthlum ichiich, tsaakuy ts'ohool, ts'ohoolil k'aninmiin, yoxon, mul ichiich[07]; Rismal xilik [14]; Guìzh-yùzh[23]
Used by (8*): Zoque[02, 03]; Huastec[07]; Quichean Maya[14, 16, 17]; Zapotec[23]; Nahua[26]
Used for (13#): Neurological (2)[14, 16]; Psychological (2)[16, 23]; Skin (1)[07]; Endocrine (1)[26]; Pregnancy (1)[02]; Female genital (1)[03]; General and Unspecified (4)[03, 07, 16, 23]; nd[17]
Cognates:
Language contact:

***Selaginella umbrosa*** **Lem. ex Hieron. (Selaginellaceae)**

Spanish names:
Indigenous names:
Used by (1*): Quichean Maya[17]
Used for (1#): nd[17]
Cognates:
Language contact:

***Selenicereus donkelaarii*** **(Salm-Dyck) Britton & Rose (Cactaceae)**

Spanish names:
Indigenous names: Tsaran-ak'[09]
Used by (1*): Yucatecan Maya[09]
Used for (2#): Skin (1)[09]; Female genital (1)[09]
Cognates:
Language contact:

[01-28]refer to the study codes in Table 4.1. *Total number of studies citing this taxon #Total number of use-records

***Selenicereus* sp. (Cactaceae)**

Spanish names: Pitaya
Indigenous names: Bohol tsatsa'[07]
Used by (2*): Zoque[03]; Huastec[07]
Used for (4#): Neurological (1)[07]; Skin (1)[03]; Pregnancy (1)[07]; General and Unspecified (1)[07]
Cognates:
Language contact:

***Semialarium mexicanum* (Miers) Mennega (Celastraceae)**

Spanish names: Cancerina; Canserina
Indigenous names: Sak-bo'ob. Xooknom[09]
Used by (4*): Zoque[03]; Yucatecan Maya[09]; Quichean Maya[12]; Zapotec[21]
Used for (10#): Digestive (2)[12, 21]; Respiratory (1)[09]; Skin (1)[21]; Urological (2)[03, 12]; Female genital (3)[03, 12, 21]; General and Unspecified (1)[12]
Cognates:
Language contact:

***Senecio deppeanus* Hemsl. (Asteraceae)**

Spanish names:
Indigenous names: Ak monhgu tzyay/ ak manhgu tzyay[01]
Used by (1*): Zoque[01]
Used for (1#): General and Unspecified (1)[01]
Cognates:
Language contact:

***Senecio salignus* DC. (Asteraceae)**

Spanish names: Chilca
Indigenous names: Meteba'[12]; Chijob[13]
Used by (2*): Quichean Maya[12, 13]
Used for (15#): Blood (1)[12]; Digestive (2)[12, 13]; Cardiovascular (1)[12]; Musculoskeletal (2)[12, 13]; Neurological (1)[12]; Psychological (1)[12]; Respiratory (1)[12]; Skin (1)[13]; Urological (1)[12]; Pregnancy (1)[13]; Female genital (1)[13]; General and Unspecified (2)[12, 13]
Cognates:
Language contact:

***Senecio* sp. (Asteraceae)**

Spanish names: Azumiate; Hierba de borracho
Indigenous names: Guìzh-diâg-bûrr[23]; Tzompiltectle[26]; Axochitl[28]
Used by (3*): Zapotec[23]; Nahua[26, 28]
Used for (5#): Digestive (1)[26]; Skin (1)[28]; Female genital (1)[26]; General and Unspecified (2)[23, 28]
Cognates:
Language contact:

***Senna alata* (L.) Roxb. (Fabaceae)**

Spanish names: Taratana
Indigenous names: Kaxlan, Xche Kaxlan[14]; Bajero pim[17]; Taratana[18]
Used by (4*): Zoque[03]; Quichean Maya[14, 17]; Western Maya[18]
Used for (5#): Blood (1)[14]; Digestive (1)[14]; Skin (2)[03, 18]; nd[17]
Cognates: CoreM: aCan;
Language contact:

***Senna alexandrina* Mill. (Fabaceae)**

Spanish names: Hoja sen
Indigenous names: Yopo sen[18]
Used by (2*): Mixe[04]; Western Maya[18]
Used for (2#): Digestive (1)[18]; General and Unspecified (1)[04]
Cognates:
Language contact:

***Senna atomaria* (L.) H.S.Irwin & Barneby (Fabaceae)**

Spanish names: Frijolillo (arbol)
Indigenous names: Tu'ha'abin[09]
Used by (2*): Yucatecan Maya[09]; Zapotec[21]
Used for (5#): Digestive (1)[21]; Musculoskeletal (2)[09, 21]; Skin (1)[21]; General and Unspecified (1)[21]
Cognates:
Language contact:

***Senna bicapsularis* (L.) Roxb. (Fabaceae)**

Spanish names:
Indigenous names: Kaxiy ts'ihol[07]
Used by (1*): Huastec[07]
Used for (3#): Neurological (1)[07]; Psychological (1)[07]; General and Unspecified (1)[07]
Cognates:
Language contact:

***Senna cobanensis* (Britton & Rose) H.S.Irwin & Barneby (Fabaceae)**

Spanish names: Frijolillo del monte
Indigenous names: Bizandxa'a gueexii[21]
Used by (1*): Zapotec[21]
Used for (4#): Digestive (1)[21]; Musculoskeletal (1)[21]; Respiratory (1)[21]; General and Unspecified (1)[21]
Cognates:
Language contact:

***Senna foetidissima* (G.Don) H.S.Irwin & Barneby (Fabaceae)**

Spanish names: Hierba de zopilote
Indigenous names:
Used by (1*): Zoque[01]
Used for (1#): Skin (1)[01]
Cognates:
Language contact:

***Senna fruticosa* (Mill.) H.S.Irwin & Barneby (Fabaceae)**

Spanish names: Cerilla
Indigenous names: K'anchik'in-ak'[09]; Bizandxa'a[21]
Used by (2*): Yucatecan Maya[09]; Zapotec[21]
Used for (2#): Digestive (1)[09]; Skin (1)[21]
Cognates:
Language contact:

***Senna hayesiana* (Britton & Rose) H.S.Irwin & Ba (Fabaceae)**

Spanish names:
Indigenous names: Carabans i che'[17]
Used by (1*): Quichean Maya[17]
Used for (1#): nd[17]
Cognates:
Language contact:

[01-28]refer to the study codes in Table 4.1. *Total number of studies citing this taxon #Total number of use-records

***Senna hirsuta*** **(L.) H.S.Irwin & Barneby (Fabaceae)**

Spanish names:
Indigenous names: Chuch acuy[03]; Itsaan an bichaam[07]
Used by (2*): Zoque[03]; Huastec[07]
Used for (4#): Digestive (1)[03]; Musculoskeletal (2)[03, 07]; Skin (1)[03]
Cognates:
Language contact:

***Senna multijuga*** **(Rich.) H.S.Irwin & Barneby (Fabaceae)**

Spanish names: Palo Santiago
Indigenous names: Uaxiñ[03]
Used by (1*): Zoque[03]
Used for (3#): Digestive (1)[03]; Skin (1)[03]; Female genital (1)[03]
Cognates:
Language contact:

***Senna obtusifolia*** **(L.) H.S.Irwin & Barneby (Fabaceae)**

Spanish names:
Indigenous names: Mehenbu'ul-xiw[09]
Used by (1*): Yucatecan Maya[09]
Used for (2#): Digestive (1)[09]; Musculoskeletal (1)[09]
Cognates:
Language contact:

***Senna occidentalis*** **(L.) Link (Fabaceae)**

Spanish names: Hormiguillo, frijolillo
Indigenous names: Syäk mätzyi[02]; Chuch acuy[03]; Pa xuxk[04]; Bichaam ts'ohool, witsaap ch'ohool[07]; Linaq Q'ehen[14]; U mul xinich'[18]; B’u’r k’opot[19]; Higiacxihuitl[28]
Used by (11*): Zoque[02, 03]; Mixe[04]; Huastec[07]; Yucatecan Maya[10, 11]; Quichean Maya[14]; Western Maya[18, 19]; Zapotec[21]; Nahua[28]
Used for (29#): Blood (1)[14]; Digestive (5)[02, 03, 07, 18, 21]; Eye (1)[19]; Cardiovascular (1)[07]; Musculoskeletal (3)[03, 07, 21]; Neurological (1)[18]; Respiratory (4)[03, 18, 18, 21]; Skin (3)[11, 14, 28]; Pregnancy (1)[07]; Female genital (2)[02, 14]; General and Unspecified (7)[03, 04, 07, 10, 11, 19, 28]
Cognates: MZ: chuch/xux;
Language contact:

***Senna papillosa*** **(Britton et Rose) H. S. Irwin et Barneby (Fabaceae)**

Spanish names: Cachinbal
Indigenous names: Putx tsay[03]
Used by (1*): Zoque[03]
Used for (1#): Skin (1)[03]
Cognates:
Language contact:

***Senna pendula*** **(Willd.) H.S.Irwin & Barneby (Fabaceae)**

Spanish names:
Indigenous names: M¥k stogoy[03]; Tsuleek' ekwet, bichaam te', xamxam[07]
Used by (2*): Zoque[03]; Huastec[07]
Used for (7#): Digestive (1)[03]; Ear (1)[03]; Musculoskeletal (1)[07]; Neurological (1)[07]; Respiratory (1)[07]; Skin (2)[03, 07]
Cognates:
Language contact:

[01-28]refer to the study codes in Table 4.1. *Total number of studies citing this taxon #Total number of use-records

***Senna quinquangulata*** **(Rich.) H. S. Irwin et Barneby  (Fabaceae)**

Spanish names:
Indigenous names: Putx tsay [03]
Used by (1*): Zoque[03]
Used for (2#): Musculoskeletal (1)[03]; General and Unspecified (1)[03]
Cognates:
Language contact:

***Senna reticulata*** **(Willd.) H.S.Irwin & Barneby (Fabaceae)**

Spanish names: Barajo; Flor de abejón
Indigenous names: Guixa'a mbisundxi[21]
Used by (2*): Quichean Maya[15]; Zapotec[21]
Used for (4#): Musculoskeletal (1)[21]; Skin (1)[21]; Female genital (1)[15]; General and Unspecified (1)[21]
Cognates:
Language contact:

***Senna skinneri*** **(Benth.) H.S.Irwin & Barneby (Fabaceae)**

Spanish names:
Indigenous names: Pa xuxk[04]
Used by (1*): Mixe[04]
Used for (1#): General and Unspecified (1)[04]
Cognates:
Language contact:

***Senna*** **sp. (Fabaceae)**

Spanish names: Flor de San José
Indigenous names: Kara'bansi che[16]; Guièe-mzhòodz, guìzh-mzhòod, guièe-nguládz, guìzh-nguládz, x-guìzh-nguládz, guièe-sàn-wsé,  guièe-yòob-chèn, yàg-guièe-nguĕts[23]
Used by (2*): Quichean Maya[16]; Zapotec[23]
Used for (3#): Psychological (1)[16]; Skin (1)[23]; General and Unspecified (1)[23]
Cognates:
Language contact:

***Senna spectabilis*** **(DC.) H.S.Irwin & Barneby  (Fabaceae)**

Spanish names: Flor de todos santos; Todos los santos amarillo
Indigenous names: Ku'tzum kuy[02]; K¥ mooya[03]; Poop tsina'an[04]; Loolte'[07]
Used by (4*): Zoque[02, 03]; Mixe[04]; Huastec[07]
Used for (6#): Ear (1)[03]; Neurological (1)[03]; Respiratory (1)[04]; Skin (1)[03]; Female genital (1)[02]; General and Unspecified (1)[07]
Cognates:
Language contact: Chimalapa Zoq <> Mixe

***Senna villosa*** **(Mill.) H.S.Irwin & Barneby (Fabaceae)**

Spanish names:
Indigenous names: Saalche', Boxsaal[09]
Used by (1*): Yucatecan Maya[09]
Used for (1#): Skin (1)[09]
Cognates:
Language contact:

***Serjania goniocarpa*** **Radlk. (Sapindaceae)**

Spanish names: Tres costilla
Indigenous names: Tukas Tzayapak [01]
Used by (1*): Zoque[01]
Used for (4#): Digestive (1)[01]; Musculoskeletal (1)[01]; Urological (1)[01]; General and Unspecified (1)[01]
Cognates:
Language contact:

[01-28] refer to the study codes in Table 4.1.     *Total number of studies citing this taxon     #Total number of use-records

***Serjania mexicana* (L.) Willd. (Sapindaceae)**

Spanish names:
Indigenous names: Aki tiuts ay[03]
Used by (1*): Zoque[03]
Used for (4#): Digestive (1)[03]; Neurological (1)[03]; Skin (1)[03]; Female genital (1)[03]
Cognates:
Language contact:

***Serjania racemosa* Schumach. (Sapindaceae)**

Spanish names: Gulandrina
Indigenous names: Riagshinguish[21]
Used by (1*): Zapotec[21]
Used for (1#): Digestive (1)[21]
Cognates:
Language contact:

***Serjania* sp. (Sapindaceae)**

Spanish names:
Indigenous names: Tsank'ub ts'ohool, wal puchun[07]
Used by (1*): Huastec[07]
Used for (3#): Digestive (1)[07]; Neurological (1)[07]; Skin (1)[07]
Cognates:
Language contact:

***Serjania triquetra* Radlk. (Sapindaceae)**

Spanish names: Tres lomos
Indigenous names: Popo pujkuy poj [02]
Used by (1*): Zoque[02]
Used for (2#): Digestive (1)[02]; Male genital (1)[02]
Cognates:
Language contact:

***Sesamum indicum* L. (Pedaliaceae)**

Spanish names: Ajonjoli
Indigenous names: Talhtzi'nqui'hui, aa'juliim, cu'li'm, zin'qui'hui[06]
Used by (3*): Totonac[06]; Quichean Maya[12]; Zapotec[21]
Used for (6#): Digestive (1)[12]; Skin (1)[12]; Urological (1)[12]; Pregnancy (1)[06]; General and Unspecified (2)[12, 21]
Cognates:
Language contact:

***Setaria liebmannii* E.Fourn. (Poaceae)**

Spanish names:
Indigenous names: Weew ok[07]
Used by (1*): Huastec[07]
Used for (1#): Neurological (1)[07]
Cognates:
Language contact:

***Setaria parviflora* (Poir.) M.Kerguelen (Poaceae)**

Spanish names:
Indigenous names: Ch'upil Q'ehen[14]
Used by (1*): Quichean Maya[14]
Used for (1#): Female genital (1)[14]
Cognates:
Language contact:

[01-28]refer to the study codes in Table 4.1. *Total number of studies citing this taxon #Total number of use-records

***Sicydium tamnifolium* (Kunth) Cogn. (Cucurbitaceae)**

Spanish names:
Indigenous names: Chakmots-ak', Hoykep, Saloli-ak', Cbikimu-ik'[09]; Pak il[11]
Used by (2*): Yucatecan Maya[09, 11]
Used for (4#): Eye (1)[09]; Psychological (1)[11]; Skin (1)[09]; General and Unspecified (1)[11]
Cognates:
Language contact:

***Sicydium tuerckheimii* Donn.Sm. (Cucurbitaceae)**

Spanish names:
Indigenous names: Xsantii ajpaq, Santii' Aj Paq'[14]
Used by (1*): Quichean Maya[14]
Used for (1#): Cardiovascular (1)[14]
Cognates:
Language contact:

***Sida acuta* Burm.f. (Malvaceae)**

Spanish names: Malva, escobillo
Indigenous names: Kung pet kuy/tung'an petkuy/kan tane)/kan petkuy/ täm petkuy/pat petkuy/paj petkuy[01]; Yoya malva[03]; Poop tukats[04]; Thak thipon[07]; Chichibe[09]; Meseb'äl Q'os Aqom[12]; Mesb'eel, Mes'uul[14]; Mes' b'eel[17]; Lexuba'a nagatzii/ gutzii[21]
Used by (11*): Zoque[01, 02, 03]; Mixe[04]; Huastec[07]; Yucatecan Maya[09]; Quichean Maya[12, 14, 17]; Western Maya[18]; Zapotec[21]
Used for (36#): Digestive (7)[01, 02, 07, 09, 12, 14, 18]; Musculoskeletal (2)[01, 21]; Neurological (1)[04]; Respiratory (2)[01, 18]; Skin (7)[01, 04, 07, 09, 12, 14, 21]; Endocrine (1)[02]; Urological (3)[01, 03, 12]; Pregnancy (3)[01, 14, 21]; Female genital (3)[03, 12, 21]; Male genital (1)[02]; General and Unspecified (5)[01, 02, 02, 04, 21]; nd[17]
Cognates: Quich: mesb'el;
Language contact:

***Sida rhombifolia* L. (Malvaceae)**

Spanish names: Malva, escobillo
Indigenous names: Ta'petkuy[02]; Yoya malva[03]; Tsap tukaats[04]; Pasmar xiw[09]; Mezbel q'ais[12]; Mesb'al[13]; Mesbel[15]; Tzatzal mes[20]; Màlbàrîscw[23]; Tlanexahuiltzi[26]
Used by (10*): Zoque[02, 03]; Mixe[04]; Yucatecan Maya[09]; Quichean Maya[12, 13, 15]; Western Maya[20]; Zapotec[23]; Nahua[26]
Used for (35#): Blood (1)[12]; Digestive (6)[02, 03, 09, 12, 20, 26]; Musculoskeletal (2)[02, 26]; Neurological (2)[02, 04]; Respiratory (2)[12, 23]; Skin (5)[02, 04, 12, 13, 26]; Endocrine (1)[02]; Urological (2)[03, 15]; Pregnancy (4)[02, 13, 15, 23]; Female genital (2)[02, 03]; Male genital (1)[02]; General and Unspecified (7)[02, 04, 09, 12, 13, 23, 26]
Cognates: CoreM: mes(b'el);
Language contact:

***Sida* sp. (Malvaceae)**

Spanish names: Malva de cochino
Indigenous names: Yoya malva[03]
Used by (1*): Zoque[03]
Used for (7#): Digestive (1)[03]; Musculoskeletal (1)[03]; Psychological (1)[03]; Respiratory (1)[03]; Skin (1)[03]; Female genital (1)[03]; General and Unspecified (1)[03]
Cognates:
Language contact:

***Sidastrum paniculatum* (L.) Fryxell (Malvaceae)**

Spanish names:
Indigenous names:
Used by (1*): Zoque[03]
Used for (1#): Digestive (1)[03]
Cognates:
Language contact:

***Sideroxylon capiri* (A.DC.) Pittier (Sapotaceae)**

Spanish names: Tempisque
Indigenous names:
Used by (1*): Quichean Maya[12]
Used for (2#): Cardiovascular (1)[12]; General and Unspecified (1)[12]
Cognates:
Language contact:

***Silybum marianum* (L.) Gaertn. (Asteraceae)**

Spanish names: Cardo Maria, Cardo Silvestre
Indigenous names:
Used by (1*): Quichean Maya[12]
Used for (7#): Blood (1)[12]; Digestive (1)[12]; Musculoskeletal (1)[12]; Neurological (1)[12]; Psychological (1)[12]; Endocrine (1)[12]; Urological (1)[12]
Cognates:
Language contact:

***Simaba cedron* Planch. (Simaroubaceae)**

Spanish names: Cedrón
Indigenous names:
Used by (5*): Zoque[01, 02, 03]; Western Maya[19]; Zapotec[21]
Used for (10#): Digestive (3)[01, 03, 19]; Musculoskeletal (1)[21]; Skin (4)[01, 02, 03, 21]; General and Unspecified (2)[01, 03]
Cognates:
Language contact:

***Simarouba amara* Aubl. (Simaroubaceae)**

Spanish names: Aceituno; Negrito
Indigenous names: Passac[10]; Pa'sa'ak'[11]
Used by (3*): Yucatecan Maya[10, 11]; Western Maya[19]
Used for (3#): Digestive (3)[10, 11, 19]
Cognates: Yuca: pasak;
Language contact:

***Simira mexicana* (Bullock) Steyerm. (Rubiaceae)**

Spanish names: Nazareno rojo
Indigenous names:
Used by (1*): Zoque[03]
Used for (5#): Blood (1)[03]; Digestive (1)[03]; Musculoskeletal (1)[03]; Skin (1)[03]; Female genital (1)[03]
Cognates:
Language contact:

***Sinapis* sp. (Brassicaceae)**

Spanish names: Mostaza
Indigenous names:
Used by (1*): Zoque[03]
Used for (1#): Psychological (1)[03]
Cognates:
Language contact:

***Sinclairia discolor* Hook. & Arn. (Asteraceae)**

Spanish names:
Indigenous names: Luisa ay[03]
Used by (1*): Zoque[03]
Used for (2#): Respiratory (1)[03]; Skin (1)[03]
Cognates:
Language contact:

[01-28]refer to the study codes in Table 4.1. *Total number of studies citing this taxon #Total number of use-records

***Sinningia incarnata*** **(Aubl.) D.L.Denham (Gesneriaceae)**

Spanish names:
Indigenous names: Ujts køjk[04]
Used by (1*): Mixe[04]
Used for (2#): Digestive (1)[04]; Pregnancy (1)[04]
Cognates:
Language contact:

***Siparuna gesnerioides*** **(Kunth) A.DC. (Siparunaceae)**

Spanish names: Hoja de tigre
Indigenous names: Tzantzan tuj kuy[02]
Used by (1*): Zoque[02]
Used for (2#): Eye (1)[02]; Musculoskeletal (1)[02]
Cognates:
Language contact:

***Siparuna*** **sp. (Siparunaceae)**

Spanish names:
Indigenous names: Roq xa'an, co moj che[16]
Used by (1*): Quichean Maya[16]
Used for (1#): Neurological (1)[16]
Cognates:
Language contact:

***Siparuna thecaphora*** **(Poepp. & Endl.) A.DC. (Siparunaceae)**

Spanish names: Hoja de zopilote, hoje mixe, negra; Hoja de zorro/hoja de zorrillo/ hoja de tigre
Indigenous names: Kun tzyantzyan/kun tzantzan/ ku tzantzan/ wekpa tane[01]; Tsan tsan ay[03]; Atsømtsi'ixy[04]; Chu' Che'[14]; Balagamixii[21]
Used by (5*): Zoque[01, 03]; Mixe[04]; Quichean Maya[14]; Zapotec[21]
Used for (26#): Blood (2)[03, 14]; Digestive (1)[03]; Eye (1)[14]; Cardiovascular (1)[01]; Musculoskeletal (4)[01, 03, 14, 21]; Neurological (3)[01, 03, 14]; Psychological (1)[01]; Respiratory (3)[01, 04, 14]; Skin (2)[03, 21]; Pregnancy (2)[01, 03]; Female genital (1)[21]; General and Unspecified (5)[01, 03, 04, 14, 21]
Cognates: MZ: tsan/tsäm; Zoq: tsantsan;
Language contact:

***Smallanthus maculatus*** **(Cav.) H.Rob. (Asteraceae)**

Spanish names: Flor de margarita
Indigenous names: K'ail, p'ilix momol, balam k'in[20]
Used by (3*): Zoque[01]; Western Maya[20]; Nahua[25]
Used for (5#): Digestive (2)[20, 25]; Skin (1)[20]; Female genital (1)[20]; General and Unspecified (1)[01]
Cognates:
Language contact:

***Smallanthus oaxacanus*** **(Sch.Bip. ex Klatt) H.Rob. (Asteraceae)**

Spanish names: Mano de león
Indigenous names: Ko'son[01]
Used by (1*): Zoque[01]
Used for (1#): Skin (1)[01]
Cognates:
Language contact:

[01-28]refer to the study codes in Table 4.1. *Total number of studies citing this taxon #Total number of use-records

***Smilax aristolochiifolia* Mill. (Smilacaceae)**

Spanish names: Cocolmeca (de hoja larga); Diente de perro; Sarsa
Indigenous names: Tuwi tätz[01]; Tzujus poj[02]
Used by (3*): Zoque[01, 02, 03]
Used for (7#): Digestive (1)[02]; Cardiovascular (1)[01]; Musculoskeletal (1)[01]; Skin (1)[03]; Urological (2)[01, 02]; Male genital (1)[01]
Cognates:
Language contact:

***Smilax bona-nox* L. (Smilacaceae)**

Spanish names: Diente de perro/zarzaparilla
Indigenous names: Tuwi tätz[01]
Used by (1*): Zoque[01]
Used for (6#): Cardiovascular (1)[01]; Musculoskeletal (1)[01]; Skin (1)[01]; Urological (1)[01]; Male genital (1)[01]; General and Unspecified (1)[01]
Cognates:
Language contact:

***Smilax domingensis* Willd. (Smilacaceae)**

Spanish names: Axquiote, Guatotole; Cocolmeca; Zarzaparilla
Indigenous names: Mom[03]; Q'ix Q'ul, Qaynäq Chaklaj Aq'om[12]; Chub Ixim[15]; Cocolmecate[26]
Used by (4*): Zoque[03]; Quichean Maya[12, 15]; Nahua[26]
Used for (24#): Blood (5)[03, 12, 12, 12, 26]; Digestive (1)[03]; Cardiovascular (2)[12, 12]; Musculoskeletal (2)[12, 12]; Skin (2)[12, 12]; Endocrine (2)[03, 12]; Urological (2)[03, 26]; Pregnancy (1)[03]; Female genital (3)[03, 12, 12]; Male genital (1)[03]; General and Unspecified (3)[12, 12, 15]
Cognates:
Language contact:

***Smilax laurifolia* L. (Smilacaceae)**

Spanish names:
Indigenous names: Tuutk kopk aats[04]
Used by (1*): Mixe[04]
Used for (2#): Digestive (1)[04]; Female genital (1)[04]
Cognates:
Language contact:

***Smilax moranensis* M.Martens & Galeotti (Smilacaceae)**

Spanish names: Bigotes de cozol; Zarzaparilla hembra
Indigenous names: Can'ci'lil[06]; Salcha'ak Aq'om[12]
Used by (2*): Totonac[06]; Quichean Maya[12]
Used for (7#): Blood (1)[12]; Digestive (1)[06]; Cardiovascular (1)[12]; Musculoskeletal (1)[12]; Skin (1)[12]; Female genital (1)[12]; General and Unspecified (1)[12]
Cognates:
Language contact:

***Smilax* sp. (Smilacaceae)**

Spanish names: Zarzaparilla, diente de perro, cocolmeca
Indigenous names: Ajni mayak[05]; Kantzilil[05]; Can'ci'lil[06]; Weew uut', kwayab uut', uut' ts'aah[07]; K'ul[13]; Q'ix pim[14]
Used by (7*): Totonac[05, 06]; Huastec[07]; Quichean Maya[12, 13, 14]; Zapotec[21]
Used for (15#): Blood (2)[13, 14]; Digestive (5)[05, 05, 06, 07, 13]; Skin (4)[07, 13, 14, 21]; Urological (1)[07]; Male genital (1)[05]; General and Unspecified (2)[07, 12]
Cognates: Toto: kantzilil; Quich: kul;
Language contact:

[01-28]refer to the study codes in Table 4.1. *Total number of studies citing this taxon #Total number of use-records

***Smilax spinosa* Mill. (Smilacaceae)**

Spanish names: Diente de perro; Zarzaparilla
Indigenous names: Mäm ma'si[02]; Q'ul[12]
Used by (2*): Zoque[02]; Quichean Maya[12]
Used for (6#): Blood (1)[12]; Musculoskeletal (1)[02]; Neurological (1)[02]; Skin (1)[12]; Urological (1)[02]; General and Unspecified (1)[12]
Cognates:
Language contact:

***Smilax velutina* Killip & C.V.Morton (Smilacaceae)**

Spanish names: Cocolmeca (de hoja chica); Tumba vaquero
Indigenous names: Tuwi tätz[01]
Used by (2*): Zoque[01, 02]
Used for (6#): Digestive (1)[02]; Eye (1)[02]; Cardiovascular (1)[01]; Musculoskeletal (1)[01]; Urological (1)[01]; Male genital (1)[01]
Cognates:
Language contact:

***Solandra maxima* (Moc. & Sessé ex Dunal) P.S.Green (Solanaceae)**

Spanish names:
Indigenous names: Tima' wits[07]
Used by (1*): Huastec[07]
Used for (2#): Eye (1)[07]; General and Unspecified (1)[07]
Cognates:
Language contact:

***Solanum acerifolium* Dunal (Solanaceae)**

Spanish names: Sosa
Indigenous names: Täptäp kuy awit[01]
Used by (1*): Zoque[01]
Used for (3#): Cardiovascular (1)[01]; Musculoskeletal (1)[01]; Pregnancy (1)[01]
Cognates:
Language contact:

***Solanum americanum* Mill. (Solanaceae)**

Spanish names: Hierbamora
Indigenous names: Mun Tzäbä[01]; Mä'a witöm ay / tzäpä[02]; Ts¥p¥ [03]; Muu stu'luut[06]; Wal ts'ok, wal piich, wal piita', ich ch'ohool[07]; Macuy[15]; Majk'ui[19]; Ledxuxii[21]; Bityuš bä'kw[22]; Yàg-pchǔux-yâas, pxât[23]
Used by (12*): Zoque[01, 02, 03]; Totonac[06]; Huastec[07]; Yucatecan Maya[09]; Quichean Maya[15]; Western Maya[19]; Zapotec[21, 22, 23]; Nahua[26]
Used for (37#): Blood (2)[15, 26]; Digestive (6)[01, 06, 07, 22, 23, 26]; Eye (1)[01]; Ear (1)[07]; Cardiovascular (1)[02]; Musculoskeletal (2)[02, 26]; Psychological (2)[02, 23]; Skin (10)[01, 03, 06, 07, 09, 19, 21, 22, 23, 26]; Endocrine (3)[01, 03, 06]; Urological (1)[06]; Pregnancy (2)[01, 07]; Female genital (2)[01, 15]; General and Unspecified (4)[01, 06, 21, 22]
Cognates: Zoq: tsäpä; CoreM: ma(j)kuy; Zapo: chux;
Language contact: Zoq <> Toto; Zoq > Zap

***Solanum candidum* Lindl. (Solanaceae)**

Spanish names: Papera
Indigenous names: Ts¥n hon kiñi[03]
Used by (2*): Zoque[03]; Yucatecan Maya[09]
Used for (3#): Skin (1)[09]; Female genital (1)[03]; Male genital (1)[03]
Cognates:
Language contact:

***Solanum chiapasense* K.E. Roes (Solanaceae)**

Spanish names: Tabardilla/ tabardillo
Indigenous names:
Used by (1*): Zoque[01]
Used for (2#): Digestive (1)[01]; General and Unspecified (1)[01]
Cognates:
Language contact:

***Solanum chrysotrichum* Schltdl. (Solanaceae)**

Spanish names:
Indigenous names: K'ux peul, tujkulum ch'ix[20]
Used by (1*): Western Maya[20]
Used for (1#): Digestive (1)[20]
Cognates:
Language contact:

***Solanum diphyllum* L. (Solanaceae)**

Spanish names:
Indigenous names: Tsakam tsabalte', tsakam wheelom, t'othoy ts'ohool, chakam walul[07]
Used by (2*): Zoque[03]; Huastec[07]
Used for (9#): Digestive (1)[07]; Ear (1)[07]; Musculoskeletal (2)[03, 07]; Neurological (1)[07]; Psychological (1)[03]; Respiratory (1)[07]; Skin (1)[07]; General and Unspecified (1)[07]
Cognates:
Language contact:

***Solanum erianthum* D. Don (Solanaceae)**

Spanish names: Cajancli, casancli; Lavaplatos blanco; Temalabar
Indigenous names: Xpahhux, Ukuch kax[09]; OkutČ[10]; Kajanki, kaxajqui[28]
Used by (6*): Zoque[01, 02, 03]; Yucatecan Maya[09, 10]; Nahua[28]
Used for (12#): Digestive (1)[02]; Cardiovascular (1)[02]; Musculoskeletal (1)[02]; Skin (4)[02, 03, 09, 10]; Urological (1)[01]; Pregnancy (2)[01, 28]; Female genital (1)[02]; General and Unspecified (1)[03]
Cognates: Yuca: ukuch/okutch;
Language contact:

***Solanum hartwegii* Benth. (Solanaceae)**

Spanish names: Tomatillo del Monte
Indigenous names: Xkoya' Q'os[12]
Used by (1*): Quichean Maya[12]
Used for (5#): Musculoskeletal (2)[12, 12]; Psychological (1)[12]; Female genital (1)[12]; General and Unspecified (1)[12]
Cognates:
Language contact:

***Solanum hirtum* Vahl (Solanaceae)**

Spanish names: Lavaplato blanco; Planta de chuchu
Indigenous names: Thak'chook' uut', thak'chook' an teneklaab, ehtiil i muuthuuts'[07]; Putbalam[09]; Guedxe baladu'u[21]
Used by (4*): Huastec[07]; Yucatecan Maya[09, 11]; Zapotec[21]
Used for (7#): Psychological (1)[07]; Skin (3)[07, 09, 21]; Female genital (2)[11, 21]; General and Unspecified (1)[21]
Cognates:
Language contact: Yuc <> Zap

[01-28]refer to the study codes in Table 4.1.  *Total number of studies citing this taxon  #Total number of use-records

***Solanum lanceifolium* Jacq. (Solanaceae)**

Spanish names:
Indigenous names: Itsik' mitsu', ehtiil muuthuuts'[07]; K'ux peul, tujkulum ch'ix[20]
Used by (2*): Huastec[07]; Western Maya[20]
Used for (2#): Digestive (1)[20]; Pregnancy (1)[07]
Cognates:
Language contact:

***Solanum lanceolatum* Cav. (Solanaceae)**

Spanish names: Lavaplato morado/ espina; Sosa, berenjena
Indigenous names: K'ux peul, tujkulum ch'ix[20]; Guedxe baladu'u[21]; Yàg-guièdz-zân[23]
Used by (3*): Western Maya[20]; Zapotec[21, 23]
Used for (6#): Digestive (1)[20]; Skin (2)[21, 23]; Pregnancy (1)[23]; General and Unspecified (2)[21, 23]
Cognates:
Language contact:

***Solanum lycopersicum* L. (Solanaceae)**

Spanish names: Jitomate
Indigenous names: Koya[01]; Txipiñ[03]; Palcha[05]; Paklcha[06]; Tuthay[07]; P'ak'[09]; Yäyäx tomate[18]; Jitomate[25]
Used by (10*): Zoque[01, 03]; Totonac[05, 06]; Huastec[07]; Yucatecan Maya[09]; Quichean Maya[12, 14]; Western Maya[18]; Nahua[25]
Used for (19#): Blood (1)[18]; Digestive (3)[03, 06, 12]; Neurological (1)[25]; Respiratory (4)[06, 07, 18, 25]; Skin (4)[01, 06, 07, 09]; Urological (1)[06]; General and Unspecified (5)[01, 03, 05, 06, 14]
Cognates: Toto: pa(k)lcha;
Language contact: Tot <> Yuc

***Solanum mammosum* L. (Solanaceae)**

Spanish names:
Indigenous names: ČuČu[10]
Used by (1*): Yucatecan Maya[10]
Used for (1#): Skin (1)[10]
Cognates:
Language contact:

***Solanum myriacanthum* Dunal (Solanaceae)**

Spanish names:
Indigenous names: Yomo tzutzi [01]; Chirion[04]
Used by (2*): Zoque[01]; Mixe[04]
Used for (2#): Digestive (1)[01]; Skin (1)[04]
Cognates:
Language contact:

***Solanum nigrescens* M. Martens & Galeotti (Solanaceae)**

Spanish names: Hierba Mora, Quilete, Macuy
Indigenous names: Majk'uy[12]; Tomatillo[25]
Used by (2*): Quichean Maya[12]; Nahua[25]
Used for (11#): Blood (1)[12]; Cardiovascular (1)[12]; Musculoskeletal (1)[12]; Neurological (1)[12]; Psychological (1)[12]; Respiratory (1)[12]; Skin (2)[12, 25]; Endocrine (1)[12]; Female genital (1)[12]; General and Unspecified (1)[12]
Cognates:
Language contact:

[01-28]refer to the study codes in Table 4.1. *Total number of studies citing this taxon #Total number of use-records

***Solanum nigricans*** **M. Martens & Galeotti (Solanaceae)**

Spanish names: Macuy, Hierba Amarga, Hierba Mora de Montaña
Indigenous names: K'e'y aqom ki'k'[12]
Used by (1*): Quichean Maya[12]
Used for (4#): Blood (1)[12]; Digestive (1)[12]; Neurological (1)[12]; Endocrine (1)[12]
Cognates:
Language contact:

***Solanum nudum*** **Dunal (Solanaceae)**

Spanish names: Hoja de azar/huele de noche
Indigenous names: Yä kosyoj/pop matsy kuy/ bola kuy[01]; Poa nanchiñ[03]; Saq' yool[14]; Yahuajcuahuitl[24]
Used by (4*): Zoque[01, 03]; Quichean Maya[14]; Nahua[24]
Used for (9#): Digestive (1)[03]; Musculoskeletal (1)[03]; Psychological (1)[24]; Skin (1)[14]; Pregnancy (1)[24]; Female genital (1)[24]; General and Unspecified (3)[01, 03, 14]
Cognates:
Language contact: Nah <> Chiapas Zoq

***Solanum pseudocapsicum*** **L. (Solanaceae)**

Spanish names: Cordilliera
Indigenous names: Chacuaquillo[21]
Used by (1*): Zapotec[21]
Used for (2#): Skin (1)[21]; General and Unspecified (1)[21]
Cognates:
Language contact:

***Solanum pubigerum*** **Dunal (Solanaceae)**

Spanish names:
Indigenous names: Ixiquelite, chichiquelite[26]
Used by (1*): Nahua[26]
Used for (2#): Digestive (1)[26]; General and Unspecified (1)[26]
Cognates:
Language contact:

***Solanum rostratum*** **Dunal (Solanaceae)**

Spanish names: Pata de cabron
Indigenous names: Geč bigin[22]
Used by (1*): Zapotec[22]
Used for (1#): Skin (1)[22]
Cognates:
Language contact:

***Solanum rudepannum*** **Dunal (Solanaceae)**

Spanish names:
Indigenous names: Xsikli-much[09]
Used by (1*): Yucatecan Maya[09]
Used for (1#): Skin (1)[09]
Cognates:
Language contact:

***Solanum schlechtendalianum*** **Walp. (Solanaceae)**

Spanish names: Hoja de balsamo
Indigenous names: Nuup cuy[03]
Used by (2*): Zoque[03]; Mixe[04]
Used for (5#): Digestive (1)[03]; Neurological (1)[04]; Skin (1)[03]; General and Unspecified (2)[03, 04]
Cognates:
Language contact:

[01-28]refer to the study codes in Table 4.1. *Total number of studies citing this taxon #Total number of use-records

***Solanum seaforthianum* Andrews (Solanaceae)**

Spanish names: Bejuco de iguana
Indigenous names: Quequelito[21]
Used by (1*): Zapotec[21]
Used for (2#): Digestive (1)[21]; General and Unspecified (1)[21]
Cognates:
Language contact:

***Solanum* sp. (Solanaceae)**

Spanish names: Berenjena; Diente de coche, gastabodoc; Gigandilla; Hierba mora, quilete, macuy, quilete de monte; Lavaplatos de espina/ barraco; San Cayetano
Indigenous names: Paga kuy/ poti ay[02]; Muutsei cuy[03]; Lacaxat, laca šanat [05]; Xsikli-much[09]; Arepa Ši'iu[10]; Imut'[13]; Tijol q'ix, uwarc'ak[13]; Rax Ik Che[14]; Ik, kejen, sajom te[16]; Geč gusohp[22]; Chichiquelite[26]; Atlapa[26]
Used by (10*): Zoque[02, 03]; Totonac[05]; Yucatecan Maya[09, 10]; Quichean Maya[13, 14, 16]; Zapotec[22]; Nahua[26]
Used for (30#): Blood (1)[13]; Digestive (2)[02, 03]; Cardiovascular (1)[02]; Musculoskeletal (4)[02, 03, 13, 26]; Neurological (4)[03, 13, 16, 22]; Psychological (2)[13, 16]; Respiratory (2)[02, 13]; Skin (6)[02, 03, 05, 09, 13, 14]; Endocrine (1)[02]; Urological (1)[02]; Pregnancy (2)[03, 10]; Female genital (1)[02]; General and Unspecified (3)[13, 14, 26]
Cognates:
Language contact:

***Solanum torvum* Sw. (Solanaceae)**

Spanish names: Sosa, lavaplato
Indigenous names: Täptäp kuy awit/tawis tane/awin täptäp kuy/ täm tujkuy/ awit kuy/täptäjkuy awit/ tätsy koya awit/ täktäk kuy awit[01]; Kamatøøts[04]; Muuthuuts', pothots, t'akaab plato[07]; Tôom pa'ap[10]; Pak'a Q'ix[12]; Paja quix', pajal q'ix', tzajal q'ix'[13]; Pajl' Q'ehen, Pajl Q'ix[14]; Quitzii[21]
Used by (11*): Zoque[01]; Mixe[04]; Huastec[07]; Yucatecan Maya[10]; Quichean Maya[12, 13, 14]; Western Maya[18, 19]; Zapotec[21]; Nahua[26]
Used for (37#): Digestive (4)[01, 07, 12, 18]; Ear (2)[01, 13]; Musculoskeletal (5)[01, 07, 12, 13, 26]; Neurological (3)[01, 04, 18]; Psychological (3)[01, 07, 14]; Respiratory (1)[13]; Skin (6)[01, 07, 10, 12, 13, 14]; Endocrine (1)[01]; Urological (2)[01, 14]; Pregnancy (2)[01, 19]; Female genital (2)[01, 19]; Male genital (1)[01]; General and Unspecified (5)[01, 04, 12, 14, 21]
Cognates: MZ: täts; CoreM: pa(C)'a; Quich: paC(a) qix;
Language contact: MZ > Hua and Yuc

***Solanum tuberosum* L. (Solanaceae)**

Spanish names: Papa
Indigenous names: Saq wach[13]
Used by (5*): Zoque[03]; Totonac[06]; Quichean Maya[12, 13, 14]
Used for (10#): Digestive (4)[03, 12, 13, 14]; Neurological (1)[06]; Skin (3)[06, 12, 13]; General and Unspecified (2)[06, 12]
Cognates:
Language contact:

***Solanum tuerckheimii* Greenm. (Solanaceae)**

Spanish names:
Indigenous names: Xk'a Waka'x[14]
Used by (1*): Quichean Maya[14]
Used for (2#): Digestive (1)[14]; Female genital (1)[14]
Cognates:
Language contact:

***Solanum umbellatum* Mill. (Solanaceae)**

Spanish names:
Indigenous names: Chi tiuts[03]; Maayte', eheenchix ts'ohool[07]
Used by (2*): Zoque[03]; Huastec[07]
Used for (5#): Musculoskeletal (2)[03, 07]; Skin (1)[03]; General and Unspecified (2)[03, 07]
Cognates:
Language contact:

***Solanum wendlandii* Hook. f. (Solanaceae)**

Spanish names:
Indigenous names: Necaxancuamecatl[24]
Used by (1*): Nahua[24]
Used for (4#): Musculoskeletal (1)[24]; Endocrine (1)[24]; Pregnancy (1)[24]; General and Unspecified (1)[24]
Cognates:
Language contact:

***Solidago stricta* Aiton (Asteraceae)**

Spanish names: Monta bilis, bara de oro
Indigenous names:
Used by (1*): Quichean Maya[13]
Used for (1#): Digestive (1)[13]
Cognates:
Language contact:

***Sonchus oleraceus* (L.) L. (Asteraceae)**

Spanish names: Amargón, Lechuguilla, Diente de León; Chicorea; Lechiguilla
Indigenous names: Met kiej[13]; Chikaryo, kulix pimil, tzepen[20]
Used by (4*): Zoque[01]; Quichean Maya[12, 13]; Western Maya[20]
Used for (13#): Blood (1)[12]; Digestive (3)[01, 12, 20]; Musculoskeletal (1)[01]; Neurological (1)[12]; Respiratory (1)[20]; Skin (2)[12, 20]; Urological (2)[01, 13]; Pregnancy (2)[12, 13]
Cognates:
Language contact:

***Sorghum bicolor* (L.) Moench (Poaceae)**

Spanish names:
Indigenous names: Laab eem[07]
Used by (1*): Huastec[07]
Used for (2#): Digestive (1)[07]; Endocrine (1)[07]
Cognates:
Language contact:

***Souroubea gilgii* V.A.Richt. (Marcgraviaceae)**

Spanish names:
Indigenous names: Hub'ub'[17]
Used by (1*): Quichean Maya[17]
Used for (1#): nd[17]
Cognates:
Language contact:

***Spananthe* sp. (Apiaceae)**

Spanish names: Cañuela
Indigenous names:
Used by (1*): Nahua[26]
Used for (1#): Skin (1)[26]
Cognates:
Language contact:

***Spathiphyllum blandum* Schott (Araceae)**

Spanish names:
Indigenous names: Yuk Q'ehen[14]
Used by (1*): Quichean Maya[14]
Used for (2#): Musculoskeletal (1)[14]; Female genital (1)[14]
Cognates:
Language contact:

***Spathiphyllum* sp. (Araceae)**

Spanish names:
Indigenous names:
Used by (1*): Zoque[03]
Used for (1#): General and Unspecified (1)[03]
Cognates:
Language contact:

***Spermacoce alata* Aubl. (Rubiaceae)**

Spanish names: Riñonina
Indigenous names:
Used by (1*): Zoque[03]
Used for (4#): Musculoskeletal (1)[03]; Skin (1)[03]; Urological (1)[03]; Female genital (1)[03]
Cognates:
Language contact:

***Spermacoce densiflora* (DC.) Alain (Rubiaceae)**

Spanish names:
Indigenous names:
Used by (1*): Zoque[03]
Used for (1#): Urological (1)[03]
Cognates:
Language contact:

***Spermacoce laevis* Lam. (Rubiaceae)**

Spanish names: Hierba cresta/tecabezón
Indigenous names: Jutut sotyi[03]; Wayel ts'ohool, pakaab ch'ohool[07]; Voton vomol, buluk sit, ve'el buluk sat vomol, k'uxbal buluk sit wamal, vol nich vomol, ni' chitom[20]
Used by (4*): Zoque[01, 03]; Huastec[07]; Western Maya[20]
Used for (8#): Digestive (4)[01, 03, 07, 20]; Respiratory (1)[20]; Skin (1)[20]; Urological (1)[03]; Female genital (1)[03]
Cognates:
Language contact:

***Spermacoce ocymifolia* Willd. ex Roem. & Schult. (Rubiaceae)**

Spanish names: Crucero
Indigenous names:
Used by (1*): Quichean Maya[13]
Used for (1#): Skin (1)[13]
Cognates:
Language contact:

***Spermacoce ocymoides* Burm.f. (Rubiaceae)**

Spanish names: Corte Santito blanco; Paletaria, sanalotodo, hierba del sapo
Indigenous names: Seq' Q'os ixoq'[12]; Tzilij, zlij, tzelej[13]; B'iritaqil Q'ehen, Josq'il Q'ehen[14]
Used by (3*): Quichean Maya[12, 13, 14]
Used for (6#): Digestive (2)[13, 14]; Respiratory (1)[13]; Skin (1)[13]; Pregnancy (1)[12]; Female genital (1)[12]
Cognates:
Language contact: Kaqchikel <> Kekchí

***Spermacoce* sp. (Rubiaceae)**

Spanish names:
Indigenous names: Haway[09]
Used by (1*): Yucatecan Maya[09]
Used for (1#): Skin (1)[09]
Cognates:
Language contact:

***Spermacoce suaveolens* (G.Mey.) Kuntze (Rubiaceae)**

Spanish names:
Indigenous names: Butun sotyi[03]
Used by (1*): Zoque[03]
Used for (1#): Skin (1)[03]
Cognates:
Language contact:

***Spermacoce tenuior* L. (Rubiaceae)**

Spanish names:
Indigenous names:
Used by (1*): Quichean Maya[17]
Used for (1#): nd[17]
Cognates:
Language contact:

***Spermacoce verticillata* L. (Rubiaceae)**

Spanish names:
Indigenous names: Haway, Haway-k'ak'[09]
Used by (1*): Yucatecan Maya[09]
Used for (1#): Skin (1)[09]
Cognates:
Language contact:

***Sphaeralcea angustifolia* (Cav.) G.Don (Malvaceae)**

Spanish names:
Indigenous names: Hierba del negro[25]
Used by (1*): Nahua[25]
Used for (2#): Musculoskeletal (1)[25]; Skin (1)[25]
Cognates:
Language contact:

***Sphagneticola trilobata (*L.) Pruski (Asteraceae)**

Spanish names:
Indigenous names: Samatil Q'ehen[14]
Used by (1*): Quichean Maya[14]
Used for (2#): Cardiovascular (1)[14]; Psychological (1)[14]
Cognates:
Language contact:

***Spigelia* sp. (Loganiaceae)**

Spanish names: Lombricera; Lombrizero
Indigenous names:
Used by (2*): Zoque[03]; Yucatecan Maya[09]
Used for (2#): Digestive (2)[03, 09]
Cognates:
Language contact:

***Spiranthes* sp. (Orchidaceae)**

Spanish names:
Indigenous names: Chiwohk'aak'[09]
Used by (1*): Yucatecan Maya[09]
Used for (1#): Skin (1)[09]
Cognates:
Language contact:

***Spondias mombin* L. (Anacardiaceae)**

Spanish names: Ciruela; Jobo
Indigenous names: K'inim[07]; Ajpoc'[18]; Biadxi[21]
Used by (3*): Huastec[07]; Western Maya[18]; Zapotec[21]
Used for (5#): Respiratory (1)[21]; Skin (2)[18, 21]; Female genital (1)[21]; General and Unspecified (1)[07]
Cognates:
Language contact:

***Spondias purpurea* L. (Anacardiaceae)**

Spanish names: Ciruela, jocote, jobo
Indigenous names: Katzsu witze[01]; Tuni[02]; Pitx cuy[03]; Tun[04]; Skatin[05]; Teen[07]; Abal[09]; Kening[10]; Abän[18]; Biadxiu'u[21]
Used by (13*): Zoque[01, 02, 03]; Mixe[04]; Totonac[05]; Huastec[07]; Yucatecan Maya[09, 10, 11]; Quichean Maya[12]; Western Maya[18]; Zapotec[21]; Nahua[26]
Used for (29#): Digestive (7)[02, 03, 04, 05, 07, 12, 18]; Musculoskeletal (1)[12]; Psychological (1)[12]; Respiratory (3)[03, 21, 26]; Skin (8)[01, 02, 03, 07, 11, 12, 18, 21]; Female genital (2)[10, 21]; Male genital (1)[02]; General and Unspecified (6)[02, 03, 09, 12, 18, 21]
Cognates: MZ: tun; Zoq: its/itx; CoreM: abaC;
Language contact: MZ > Tot and Hua

***Spondias radlkoferi* Donn.Sm. (Anacardiaceae)**

Spanish names: Jobo
Indigenous names: Jumu[02]
Used by (1*): Zoque[02]
Used for (1#): Digestive (1)[02]
Cognates:
Language contact:

***Spondias* sp. (Anacardiaceae)**

Spanish names: Jocote clavo/macho/agrio/acido/cimarrón
Indigenous names: Paré[19]
Used by (1*): Western Maya[19]
Used for (1#): General and Unspecified (1)[19]
Cognates:
Language contact:

***Sporobolus indicus* (L.) R.Br. (Poaceae)**

Spanish names:
Indigenous names: Zacate[26]
Used by (1*): Nahua[26]
Used for (1#): Pregnancy (1)[26]
Cognates:
Language contact:

***Stachys coccinea* Ortega (Lamiaceae)**

Spanish names: Muiña
Indigenous names: Guìzh-zhwĭn[23]
Used by (1*): Zapotec[23]
Used for (1#): Psychological (1)[23]
Cognates:
Language contact:

[01-28]refer to the study codes in Table 4.1. *Total number of studies citing this taxon #Total number of use-records

***Stachys* sp. (Lamiaceae)**

Spanish names: Hierba de quemadura
Indigenous names: Tlacchinoxihuitl[26]
Used by (1*): Nahua[26]
Used for (1#): Skin (1)[26]
Cognates:
Language contact:

***Stachytarpheta cayennensis* (Rich.) Vahl (Verbenaceae)**

Spanish names: Cola de Armadillo, Armado; Vervena
Indigenous names: Xye' ajwech [14]
Used by (2*): Yucatecan Maya[11]; Quichean Maya[14]
Used for (9#): Digestive (1)[14]; Eye (1)[14]; Neurological (1)[11]; Skin (1)[14]; Urological (2)[11, 14]; Female genital (1)[14]; General and Unspecified (2)[11, 14]
Cognates:
Language contact:

***Stachytarpheta frantzii* Pol. (Verbenaceae)**

Spanish names: Cola de alacrán/verbena embra
Indigenous names: Kaku'e tuts [01]; Xtye aj pak[16]
Used by (2*): Zoque[01]; Quichean Maya[16]
Used for (5#): Digestive (1)[01]; Cardiovascular (1)[01]; Neurological (2)[01, 16]; Skin (1)[01]
Cognates:
Language contact:

***Stachytarpheta jamaicensis* (L.) Vahl (Verbenaceae)**

Spanish names: Verbena
Indigenous names: Chi tiuts[03]; Pacer ujts[04]; Iben-xiw[09]; Tye aj pak[17]
Used by (6*): Zoque[03]; Mixe[04]; Yucatecan Maya[09]; Quichean Maya[17]; Western Maya[18]; Zapotec[21]
Used for (14#): Digestive (3)[04, 18, 21]; Musculoskeletal (1)[09]; Neurological (2)[03, 18]; Skin (1)[03]; Urological (1)[03]; Female genital (2)[03, 09]; Male genital (1)[18]; General and Unspecified (2)[18, 21]; nd[17]
Cognates:
Language contact:

***Stegnosperma cubense* A.Rich. (Stegnospermataceae)**

Spanish names: Niño que no se puede levantar
Indigenous names:
Used by (1*): Zapotec[21]
Used for (2#): Musculoskeletal (1)[21]; Neurological (1)[21]
Cognates:
Language contact:

***Stellaria cuspidata* Willd. ex Schltdl. (Caryophyllaceae)**

Spanish names: Cadillo ovalado; Hierba de rosillo; Hoja de azar
Indigenous names: Tza'a tzoy[01]; Ahuaxmama[26]
Used by (3*): Zoque[01, 03]; Nahua[26]
Used for (4#): Urological (1)[03]; Female genital (1)[03]; General and Unspecified (2)[01, 26]
Cognates:
Language contact:

***Stellaria ovata*** **Willd. ex Schltdl. (Caryophyllaceae)**

Spanish names: Trebolillo rojo
Indigenous names: Thuuyuu'il t'eel, ehtiil utek'[07]; Keq Lotz Aq'om[12]
Used by (2*): Huastec[07]; Quichean Maya[12]
Used for (8#): Eye (1)[12]; Cardiovascular (2)[07, 12]; Musculoskeletal (1)[12]; Neurological (1)[12]; Psychological (1)[12]; Skin (1)[12]; General and Unspecified (1)[12]
Cognates:
Language contact:

***Stemodia macrantha*** **B.L. Rob. (Plantaginaceae)**

Spanish names:
Indigenous names: Tlaquexaniltzi[26]
Used by (1*): Nahua[26]
Used for (1#): Skin (1)[26]
Cognates:
Language contact:

***Stenocereus*** **sp. (Cactaceae)**

Spanish names: Cactus
Indigenous names: Tzap kom[01]
Used by (1*): Zoque[01]
Used for (1#): Skin (1)[01]
Cognates:
Language contact:

***Stevia lucida*** **Lag. (Asteraceae)**

Spanish names: Yerba de borracho
Indigenous names: Gi lahk[22]
Used by (1*): Zapotec[22]
Used for (1#): Digestive (1)[22]
Cognates:
Language contact:

***Stevia ovata*** **Willd. (Asteraceae)**

Spanish names: Hierba blanca de muerto; Ocaotera
Indigenous names: Winyo wajo' jop ay[02]; Ch'aj te' nichim, ch'aal sakil nich wamal, ch'aal wamal, ch'a momol, nak' obal us[20]
Used by (3*): Zoque[01, 02]; Western Maya[20]
Used for (5#): Digestive (1)[20]; Musculoskeletal (1)[20]; Skin (3)[01, 02, 20]
Cognates:
Language contact:

***Stevia polycephala*** **Bertol. (Asteraceae)**

Spanish names: Pericón blanco
Indigenous names: Sak'a eya'[12]
Used by (1*): Quichean Maya[12]
Used for (6#): Blood (1)[12]; Digestive (1)[12]; Neurological (1)[12]; Skin (1)[12]; Endocrine (1)[12]; General and Unspecified (1)[12]
Cognates:
Language contact:

***Stevia serrata*** **Cav. (Asteraceae)**

Spanish names: Cabeza de hongo
Indigenous names: Ujlom ikox[13]
Used by (1*): Quichean Maya[13]
Used for (1#): Digestive (1)[13]
Cognates:
Language contact:

***Stevia* sp. (Asteraceae)**

Spanish names:
Indigenous names: Tzajal nak'obal us[20]
Used by (1*): Western Maya[20]
Used for (1#): Digestive (1)[20]
Cognates:
Language contact:

***Stevia suaveolens* Lag. (Asteraceae)**

Spanish names: Pericón blanco
Indigenous names: Sak'a eya'[12]
Used by (1*): Quichean Maya[12]
Used for (2#): Neurological (1)[12]; General and Unspecified (1)[12]
Cognates:
Language contact:

***Stigmaphyllon ellipticum* (Kunth) Juss. (Malpighiaceae)**

Spanish names:
Indigenous names: Samñi tsay[03]
Used by (1*): Zoque[03]
Used for (2#): Digestive (1)[03]; Female genital (1)[03]
Cognates:
Language contact:

***Stigmatophyllon* sp. (Malpighiaceae)**

Spanish names:
Indigenous names: Samñi tsay[03]
Used by (1*): Zoque[03]
Used for (1#): Female genital (1)[03]
Cognates:
Language contact:

***Stillingia acutifolia* (Benth.) Benth. & Hook.f. ex Hemsl. (Euphorbiaceae)**

Spanish names: Hierba mala
Indigenous names: Puj Q'ayis[12]; Etzel k'ayes[13]
Used by (2*): Quichean Maya[12, 13]
Used for (3#): Skin (2)[12, 13]; General and Unspecified (1)[12]
Cognates: Quich: k'ayis;
Language contact:

***Stizophyllum riparium* (Kunth) Sandwith (Bignoniaceae)**

Spanish names:
Indigenous names: Xa'bach[09]
Used by (1*): Yucatecan Maya[09]
Used for (1#): Respiratory (1)[09]
Cognates:
Language contact:

***Stromanthe jacquinii* (Roem. & Schult.) H.A.Kenn. & Nicolson (Marantaceae)**

Spanish names:
Indigenous names: Xmox So'sol[14]
Used by (1*): Quichean Maya[14]
Used for (1#): Female genital (1)[14]
Cognates:
Language contact:

[01-28]refer to the study codes in Table 4.1. *Total number of studies citing this taxon #Total number of use-records

***Stromanthe macrochlamys*** **(Woodson & Standl.) H.A.Kenn & Nicolson (Marantaceae)**

Spanish names: Hoja de piedra
Indigenous names: Tza ay[01]
Used by (1*): Zoque[01]
Used for (2#): Digestive (1)[01]; Urological (1)[01]
Cognates:
Language contact:

***Struthanthus cassythoides*** **Millsp. ex Standl. (Loranthaceae)**

Spanish names: Caballera; Cabellera
Indigenous names: Cuyñukxi[03]; Tak'ä zte'[18]
Used by (2*): Zoque[03]; Western Maya[18]
Used for (3#): Skin (2)[03, 18]; Pregnancy (1)[18]
Cognates:
Language contact:

***Struthanthus crassipes*** **(Oliv.) Eichler (Loranthaceae)**

Spanish names:
Indigenous names: Ok'lom te'[07]
Used by (1*): Huastec[07]
Used for (1#): Digestive (1)[07]
Cognates:
Language contact:

***Struthanthus quercicola*** **(Schltdl. & Cham.) D.Don (Loranthaceae)**

Spanish names: Mata palo; Seca palo
Indigenous names: Eju katzakatza[01]; Mak'tahila[08]
Used by (2*): Zoque[01]; Huastec[08]
Used for (4#): Digestive (1)[01]; Skin (1)[08]; Endocrine (1)[08]; Female genital (1)[01]
Cognates:
Language contact:

***Struthanthus*** **sp. (Loranthaceae)**

Spanish names: Cabellera; Muérdago
Indigenous names: Cuyñukxi[03]; Ok'lom te'[07]; Tzara' Aq'om[12]; Raximai Q'en, Mayil Q'en[14]
Used by (4*): Zoque[03]; Huastec[07]; Quichean Maya[12, 14]
Used for (14#): Blood (1)[12]; Digestive (1)[12]; Cardiovascular (1)[07]; Musculoskeletal (1)[12]; Neurological (2)[07, 12]; Psychological (3)[07, 12, 12]; Skin (3)[03, 12, 14]; General and Unspecified (2)[12, 14]
Cognates:
Language contact: Hua <> Kaqchikel

***Struthanthus tacanensis*** **Lundell (Loranthaceae)**

Spanish names: Muérdago
Indigenous names: Tzara' [12]
Used by (1*): Quichean Maya[12]
Used for (4#): Blood (1)[12]; Cardiovascular (1)[12]; Skin (1)[12]; General and Unspecified (1)[12]
Cognates:
Language contact:

***Strychnos panamensis*** **Seem. (Loganiaceae)**

Spanish names: Chicoloro
Indigenous names: Curux k'ix[17]
Used by (2*): Yucatecan Maya[10]; Quichean Maya[17]
Used for (3#): Digestive (1)[10]; Skin (1)[10]; nd[17]
Cognates:
Language contact:

[01-28]refer to the study codes in Table 4.1. *Total number of studies citing this taxon #Total number of use-records

***Stylosanthes guianensis* (Aubl.) Sw. (Fabaceae)**

Spanish names:
Indigenous names:
Used by (1*): Zoque[03]
Used for (1#): Skin (1)[03]
Cognates:
Language contact:

***Stylosanthes humilis* Kunth (Fabaceae)**

Spanish names: Hoja de azar
Indigenous names: Tza'a tzoy[01]
Used by (1*): Zoque[01]
Used for (1#): General and Unspecified (1)[01]
Cognates:
Language contact:

***Stylotrichium corymbosum* (DC.) Mattf. (Asteraceae)**

Spanish names: Hierba de dulce, sacate dulce
Indigenous names: Loc'ab[13]
Used by (1*): Quichean Maya[13]
Used for (1#): Skin (1)[13]
Cognates:
Language contact:

***Swietenia humilis* Zucc. (Meliaceae)**

Spanish names: Caoba
Indigenous names: Tzapats kuy[02]; Gueleyexi'i[21]
Used by (3*): Zoque[01, 02]; Zapotec[21]
Used for (8#): Digestive (1)[21]; Skin (1)[21]; Endocrine (2)[01, 02]; Urological (1)[01]; Pregnancy (1)[21]; General and Unspecified (2)[01, 21]
Cognates:
Language contact:

***Swietenia* sp. (Meliaceae)**

Spanish names: Caoba
Indigenous names:
Used by (1*): Zoque[03]
Used for (3#): Digestive (1)[03]; Musculoskeletal (1)[03]; Female genital (1)[03]
Cognates:
Language contact:

***Symphyotrichum moranense* (Kunth) G.L. Nesom (Asteraceae)**

Spanish names: Tomillo silvestre
Indigenous names:
Used by (1*): Zoque[01]
Used for (1#): General and Unspecified (1)[01]
Cognates:
Language contact:

***Symphyotrichum bullatum* (Klatt) G.L.Nesom (Asteraceae)**

Spanish names: Pericon de agua
Indigenous names:
Used by (1*): Zoque[03]
Used for (1#): General and Unspecified (1)[03]
Cognates:
Language contact:

***Syngonium angustatum* Schott (Araceae)**

Spanish names:
Indigenous names: Pa-uk tiinxy[04]
Used by (1*): Mixe[04]
Used for (1#): Skin (1)[04]
Cognates:
Language contact:

***Syngonium podophyllum* Schott (Araceae)**

Spanish names: Mano de Diablo ; Pegapega; Teleconte
Indigenous names: Mu' katzu/ Mä'k katzu[02]; Kwaath[07]; Ruq' Maus aj' Winq[14]
Used by (4*): Zoque[02]; Huastec[07]; Yucatecan Maya[11]; Quichean Maya[14]
Used for (7#): Digestive (2)[02, 07]; Musculoskeletal (1)[11]; Neurological (1)[14]; Skin (2)[02, 14]; General and Unspecified (1)[14]
Cognates:
Language contact:

***Syngonium* sp. (Araceae)**

Spanish names: Palma de Diablo; Platano del monte
Indigenous names: Ruq' Maus aj' Winq[14]; Ruj i ruk'aj tza, ruk maus[16]; Buduaj gueexii[21]
Used by (3*): Quichean Maya[14, 16]; Zapotec[21]
Used for (4#): Neurological (2)[14, 16]; Psychological (1)[16]; General and Unspecified (1)[21]
Cognates: Quich: ruq maus;
Language contact:

***Syzygium aromaticum* (L.) Merr. & L.M.Perry (Myrtaceae)**

Spanish names: Clavo de olor
Indigenous names: Cuonagueleraajeu'u[21]
Used by (6*): Zoque[01, 02]; Quichean Maya[13]; Zapotec[21, 22, 23]
Used for (10#): Digestive (1)[01]; Ear (1)[01]; Musculoskeletal (1)[21]; Neurological (1)[02]; Respiratory (2)[01, 21]; Skin (1)[23]; Pregnancy (1)[13]; General and Unspecified (2)[21, 22]
Cognates:
Language contact:

***Syzygium jambos* (L.) Alston (Myrtaceae)**

Spanish names: Pomarosa
Indigenous names:
Used by (2*): Zoque[03]; Totonac[06]
Used for (3#): Digestive (1)[06]; Psychological (1)[03]; Respiratory (1)[03]
Cognates:
Language contact:

***Tabebuia rosea* (Bertol.) Bertero ex A.DC. (Bignoniaceae)**

Spanish names: Macuilis, macuilisguate, roble
Indigenous names: Tzan kuy [01]; Emkuioxk[04]; K'uul, wakats wich[07]; Makulis[14]; Känkän aj maculis[18]
Used by (7*): Zoque[01, 02, 03]; Mixe[04]; Huastec[07]; Quichean Maya[14]; Western Maya[18]
Used for (21#): Digestive (4)[02, 07, 14, 18]; Musculoskeletal (2)[01, 03]; Neurological (1)[18]; Skin (6)[01, 02, 03, 04, 07, 18]; Endocrine (1)[18]; Pregnancy (1)[03]; Female genital (3)[03, 07, 14]; General and Unspecified (3)[07, 14, 18]
Cognates: CoreM: makulis;
Language contact:

[01-28]refer to the study codes in Table 4.1. *Total number of studies citing this taxon #Total number of use-records

***Tabernaemontana alba* Mill. (Apocynaceae)**

Spanish names: Coyol de burro; Huevo de venado
Indigenous names: Burro puj[02]; Na'a cuy[03]; No'og waaky[04]; T'abat'[07]
Used by (4*): Zoque[02, 03]; Mixe[04]; Huastec[07]
Used for (7#): Digestive (1)[03]; Neurological (3)[03, 04, 07]; Skin (3)[02, 03, 07]
Cognates: MZ: na'a / no'o;
Language contact:

***Tabernaemontana amygdalifolia* Jacq. (Apocynaceae)**

Spanish names:
Indigenous names: Uts'upek'[09]
Used by (1*): Yucatecan Maya[09]
Used for (1#): Skin (1)[09]
Cognates:
Language contact:

***Tabernaemontana donnell-smithii* Rose ex J.D.Sm. (Apocynaceae)**

Spanish names: Huevo caballo
Indigenous names: Chac le king[10]
Used by (1*): Yucatecan Maya[10]
Used for (1#): Skin (1)[10]
Cognates:
Language contact:

***Tabernaemontana* sp. (Apocynaceae)**

Spanish names:
Indigenous names: Mbiigu' del monte[21]
Used by (1*): Zapotec[21]
Used for (2#): Respiratory (1)[21]; Skin (1)[21]
Cognates:
Language contact:

***Tagetes erecta* L. (Asteraceae)**

Spanish names: Cempoaxochitl
Indigenous names: Postin jäyä/putsy jäyä/putzin jäyä[01]; Tasi jäyä[02]; Tsuts mooya[03]; Pikøn'ak pøh[04]; Kaltpu'xa'm[05]; Santoorom wits, k'uchuth wits, k'ochith wits, kaxiy wits, ts'a'il wits, pamta wich[07]; K'uchid huitz[08]; Stupuh amarillo[10]; Ix tupuj[11]; Tu'z, Tutz, Tutz' Tatz'un Kamenaq[14]; Tiskok[18]; Sampwer[19]; Guibigua[21]; Gi bigu štil, gi togol[22]; Guièe-cŏb-mzhĭg, guièe-cŏb-yâg[23]; Sempoalxochitl[24]; Cempasúchitl[25]; Cempoalxochitl[26]; Cempoalxóchitl[28]
Used by (20*): Zoque[01, 02, 03]; Mixe[04]; Totonac[05]; Huastec[07, 08]; Yucatecan Maya[10, 11]; Quichean Maya[12, 14]; Western Maya[18, 19]; Zapotec[21, 22, 23]; Nahua[24, 25, 26, 28]
Used for (75#): Blood (1)[12]; Digestive (15)[01, 02, 03, 05, 07, 08, 11, 12, 14, 21, 22, 23, 25, 26, 28]; Eye (1)[19]; Ear (3)[01, 19, 23]; Cardiovascular (2)[01, 08]; Musculoskeletal (6)[02, 03, 07, 08, 11, 18]; Neurological (4)[07, 12, 14, 18]; Psychological (4)[01, 03, 05, 19]; Respiratory (9)[01, 07, 08, 14, 18, 21, 23, 25, 28]; Skin (9)[01, 03, 05, 07, 08, 12, 18, 21, 25]; Endocrine (1)[07]; Pregnancy (4)[07, 21, 22, 24]; Female genital (4)[02, 05, 12, 21]; General and Unspecified (12)[01, 03, 04, 05, 07, 08, 10, 12, 14, 18, 19, 21]
Cognates: Zoq: Cuts ; Huas: k'ochith wits; CoreM: tu; Yuca: tupu; Zapo: g(u)ibigiu; Nahua: cempoalxochitl;
Language contact: Zoq > Tot and Kaqchikel; Nah > Spanish

***Tagetes filifolia* Lag. (Asteraceae)**

Spanish names: Anís del monte
Indigenous names: Estrella pok[01]; Anis ujts[04]; Anix tzi'[13]; Mantzaniya ch'o, kulentu jos, inajo antíwo, injo wamal, tzitz, tzitz olol[20]; Guìzh-nìzh, guièe-nìzh[23]
Used by (9*): Zoque[01, 03]; Mixe[04]; Totonac[06]; Quichean Maya[13]; Western Maya[19, 20]; Zapotec[21, 23]
Used for (17#): Digestive (8)[01, 03, 06, 13, 19, 20, 21, 23]; Psychological (1)[13]; Respiratory (3)[01, 20, 21]; Urological (1)[13]; Pregnancy (1)[13]; General and Unspecified (3)[04, 13, 20]
Cognates:
Language contact: K'iche' <> Tzeltalan <> Zap

[01-28] refer to the study codes in Table 4.1. *Total number of studies citing this taxon #Total number of use-records

***Tagetes lucida* Cav. (Asteraceae)**

Spanish names: Pericón
Indigenous names: Tzitzimuk[01]; Kotzok ay [02]; Ohoom[07]; Eva' / Eya'[12]; Illa', iya'[13]; Yop'aj melicon[18]; Tzitz wamal, tzitz uch, sansiwre wamal, k'anal nich wamal, pimento wamal[20]; Guiahuajsa'ac[21]; Guièe-dzùu, guièe-cŏb-dzùu[23]; Yauhtli[28]
Used by (16*): Zoque[01, 02, 03]; Mixe[04]; Huastec[07]; Quichean Maya[12, 13, 14]; Western Maya[18, 19, 20]; Zapotec[21, 22, 23]; Nahua[26, 28]
Used for (55#): Blood (1)[12]; Digestive (12)[01, 02, 03, 04, 12, 13, 18, 20, 21, 22, 23, 28]; Musculoskeletal (3)[12, 20, 26]; Neurological (3)[12, 18, 28]; Psychological (3)[01, 12, 20]; Respiratory (7)[01, 03, 12, 13, 14, 20, 21]; Skin (3)[18, 21, 28]; Urological (1)[12]; Pregnancy (8)[02, 03, 13, 19, 21, 22, 23, 26]; Female genital (6)[01, 03, 12, 13, 19, 21]; General and Unspecified (8)[02, 07, 12, 13, 18, 20, 21, 28]
Cognates: Quich: eya/iya;
Language contact: Chiapas Zoq <> Tzeltalan

***Tagetes micrantha* Cav. (Asteraceae)**

Spanish names: Anís; Anisillo
Indigenous names:
Used by (3*): Nahua[25, 26, 28]
Used for (4#): Digestive (3)[25, 26, 28]; Pregnancy (1)[26]
Cognates:
Language contact:

***Tagetes nelsonii* Greenm. (Asteraceae)**

Spanish names:
Indigenous names: Yama chauk[20]
Used by (1*): Western Maya[20]
Used for (1#): Digestive (1)[20]
Cognates:
Language contact:

***Tagetes* sp. (Asteraceae)**

Spanish names: Anís del camo; Flor de muerto
Indigenous names: Tempula[09]; Prutz', pretz', ukutz'ij kaminaq[13]; Anís gihš[22]
Used by (3*): Yucatecan Maya[09]; Quichean Maya[13]; Zapotec[22]
Used for (4#): Digestive (2)[13, 22]; Pregnancy (1)[13]; Female genital (1)[09]
Cognates:
Language contact:

***Tagetes tenuifolia* Cav. (Asteraceae)**

Spanish names: Todos santos
Indigenous names: Anima jäyä [01]
Used by (1*): Zoque[01]
Used for (4#): Digestive (1)[01]; Respiratory (1)[01]; Skin (1)[01]; Urological (1)[01]
Cognates:
Language contact:

***Talinum fruticosum* (L.) Juss. (Talinaceae)**

Spanish names:
Indigenous names: Thak akw'aal, t'uhub akw'aal[07]
Used by (1*): Huastec[07]
Used for (3#): Digestive (1)[07]; Skin (1)[07]; Urological (1)[07]
Cognates:
Language contact:

***Talinum paniculatum* (Jacq.) Gaertn. (Talinaceae)**

Spanish names: Hierba del riñon
Indigenous names: Masan ay[03]; Pitsits wal pktha', tsakam ix thuyum[07]; Ncuàan-dzéb-ròo[23]
Used by (4*): Zoque[01, 03]; Huastec[07]; Zapotec[23]
Used for (7#): Digestive (1)[03]; Musculoskeletal (1)[03]; Skin (1)[03]; Urological (1)[01]; Female genital (1)[01]; General and Unspecified (2)[07, 23]
Cognates:
Language contact:

***Tamarindus indica* L. (Fabaceae)**

Spanish names: Tamarindo
Indigenous names: Tamariindu, tam malin[07]; Gubshnii'[21]
Used by (10*): Zoque[02, 03]; Mixe[04]; Huastec[07]; Yucatecan Maya[09]; Quichean Maya[12]; Western Maya[18, 19]; Zapotec[21, 23]
Used for (26#): Digestive (6)[02, 03, 09, 12, 18, 23]; Musculoskeletal (1)[02]; Neurological (1)[02]; Psychological (2)[07, 21]; Respiratory (2)[02, 03]; Skin (2)[02, 18]; Endocrine (2)[02, 12]; Urological (2)[03, 18]; General and Unspecified (8)[02, 03, 04, 07, 12, 18, 19, 21]
Cognates:
Language contact:

***Tamonea curassavica* (L.) Pers. (Verbenaceae)**

Spanish names:
Indigenous names: Ook' t'ithith[07]
Used by (1*): Huastec[07]
Used for (2#): Digestive (1)[07]; Urological (1)[07]
Cognates:
Language contact:

***Tanacetum parthenium* (L.) Sch.Bip. (Asteraceae)**

Spanish names: Hierba Santa María
Indigenous names: Artamiza/artamisy/altamiza[01]; Santa Maria aay[04]; K'anim mim María ts'ojol[08]; Altamix[12]; Altamix, altimix, altamixá[13]; Guixnash[21]; Manzaniy la[22]; Guièe-sàntàmàrĭ, guièe-sàntàmàrĭ-nquĭts[23]; Caltemesha[28]
Used by (13*): Zoque[01, 02]; Mixe[04]; Huastec[08]; Quichean Maya[12, 13]; Zapotec[21, 22, 23]; Nahua[25, 26, 27, 28]
Used for (53#): Blood (1)[12]; Digestive (12)[01, 02, 04, 08, 12, 13, 21, 22, 23, 25, 26, 27]; Ear (2)[04, 23]; Cardiovascular (1)[23]; Musculoskeletal (4)[01, 12, 13, 23]; Neurological (3)[08, 12, 13]; Psychological (3)[13, 22, 27]; Respiratory (2)[12, 23]; Skin (1)[12]; Urological (3)[02, 12, 13]; Pregnancy (8)[01, 02, 12, 13, 21, 23, 25, 27]; Female genital (6)[01, 02, 04, 12, 13, 23]; General and Unspecified (7)[01, 08, 12, 13, 22, 25, 28]
Cognates:
Language contact:

***Tanaecium pyramidatum* (Rich.) L.G.Lohmann (Bignoniaceae)**

Spanish names:
Indigenous names: Pobatsay[03]
Used by (1*): Zoque[03]
Used for (2#): Skin (1)[03]; Female genital (1)[03]
Cognates:
Language contact:

***Tanaecium tetragonolobum* (Jacq.) L.G.Lohmann (Bignoniaceae)**

Spanish names:
Indigenous names: Bak chiwoh[09]; Ški'iŠ[10]
Used by (2*): Yucatecan Maya[09, 10]
Used for (2#): Respiratory (1)[09]; Skin (1)[10]
Cognates:
Language contact:

[01-28]refer to the study codes in Table 4.1. *Total number of studies citing this taxon #Total number of use-records

### *Tapirira mexicana* March. (Anacardiaceae)

Spanish names: Ocholillo
Indigenous names: Ocholi nuumpi[03]
Used by (1*): Zoque[03]
Used for (2#): Musculoskeletal (1)[03]; Psychological (1)[03]
Cognates:
Language contact:

### *Taraxacum campylodes* G.E.Haglund (Asteraceae)

Spanish names: Amargón, Lechuguilla, Diente de León, Lechuga criollo; Diente de león; Diente de león, amargón, scorzonera amarga; Diente de león, lechogilla
Indigenous names: Q'uqun Q'än Q'os[12]; Kur kur[13]
Used by (5*): Zoque[01]; Quichean Maya[12, 13]; Nahua[25, 26]
Used for (17#): Blood (1)[13]; Digestive (3)[01, 12, 13]; Cardiovascular (1)[25]; Psychological (1)[12]; Respiratory (2)[13, 25]; Endocrine (2)[12, 26]; Urological (5)[01, 12, 13, 25, 26]; Pregnancy (1)[13]; General and Unspecified (1)[12]
Cognates:
Language contact:

### *Taxodium huegelii* C.Lawson (Cupressaceae)

Spanish names: Sabino, ahuehuete
Indigenous names: Chuuche'[07]; Yàg-guìzdòo, yàg-guìstòo[23]
Used by (2*): Huastec[07]; Zapotec[23]
Used for (3#): Skin (1)[23]; Pregnancy (1)[07]; Female genital (1)[07]
Cognates:
Language contact:

### *Tecoma stans* (L.) Juss. ex Kunth (Bignoniaceae)

Spanish names: Tronadora
Indigenous names: Kan yoks[01]; Totzkä jäyä[02]; Puutx mooya[03]; San pedro wits[07]; K'anlol[09]; Timbuk'[13]; Ch'aj te[19]; Dze'ing[21]; Guièe-tùts, guìzh-tùts, guìzh-guièe-tùts, yàg-guièe-tùts, yàg-nlibâd-tùts[23]; Nixtamalxochitl[28]
Used by (14*): Zoque[01, 02, 03]; Mixe[04]; Huastec[07]; Yucatecan Maya[09]; Quichean Maya[12, 13]; Western Maya[18, 19]; Zapotec[21, 22, 23]; Nahua[28]
Used for (36#): Digestive (5)[02, 03, 21, 22, 28]; Cardiovascular (1)[12]; Musculoskeletal (4)[01, 02, 19, 21]; Neurological (2)[12, 19]; Respiratory (4)[02, 03, 19, 23]; Skin (2)[01, 19]; Endocrine (10)[01, 02, 03, 04, 07, 09, 12, 13, 18, 28]; Urological (1)[01]; Pregnancy (1)[04]; Female genital (1)[21]; General and Unspecified (5)[01, 03, 04, 19, 21]
Cognates:
Language contact: Chiapas Zoq <> Yuca <> Chortí; Chiapas Zoq <> Chimalapa Zoq <> Zap

### *Tectaria heracleifolia* (Willd.) Underw. (Tectariaceae)

Spanish names: Hierba del monte; Lengua de ciervo; Pesmita; Pijul; Siempreviva
Indigenous names: Weew koxol, akan hooh, k'ubak k'aninmiin, akan tspk, pok' ts'ohool, pux lat'em, lek'aab torro[07]; Kubak ajatik[08]; Roq' Chi'wan[14]
Used by (6*): Zoque[03]; Totonac[05]; Huastec[07, 08]; Quichean Maya[14]; Nahua[25]
Used for (12#): Blood (1)[07]; Digestive (1)[07]; Cardiovascular (1)[07]; Neurological (2)[07, 14]; Psychological (1)[03]; Skin (2)[05, 08]; Endocrine (1)[08]; Pregnancy (1)[07]; General and Unspecified (2)[07, 25]
Cognates: Huas: kubak;
Language contact:

### *Tectaria* sp. (Tectariaceae)

Spanish names:
Indigenous names: Matlaxcalxihuitl[24]
Used by (1*): Nahua[24]
Used for (1#): Pregnancy (1)[24]
Cognates:
Language contact:

[01-28]refer to the study codes in Table 4.1. *Total number of studies citing this taxon #Total number of use-records

***Telanthophora arborescens* (Steetz) H.Rob. & Brettell (Asteraceae)**

Spanish names: Mano de León
Indigenous names:
Used by (1*): Quichean Maya[12]
Used for (3#): Psychological (1)[12]; Urological (1)[12]; General and Unspecified (1)[12]
Cognates:
Language contact:

***Telanthophora grandifolia* (Less.) H.Rob. & Brettell (Asteraceae)**

Spanish names:
Indigenous names: Nag ay[03]
Used by (1*): Zoque[03]
Used for (2#): Skin (1)[03]; Pregnancy (1)[03]
Cognates:
Language contact:

***Tephrosia lanata* M. Martens et Galeotti (Fabaceae)**

Spanish names:
Indigenous names: Mokoy[03]
Used by (1*): Zoque[03]
Used for (1#): Skin (1)[03]
Cognates:
Language contact:

***Tephrosia multifolia* Rose (Fabaceae)**

Spanish names:
Indigenous names: Mokoy[03]; Ch'alam[17]
Used by (2*): Zoque[03]; Quichean Maya[17]
Used for (2#): Skin (1)[03]; nd[17]
Cognates:
Language contact:

***Terminalia catappa* L. (Combretaceae)**

Spanish names: Almendro
Indigenous names: Bek almendra[18]
Used by (7*): Zoque[01, 02, 03]; Yucatecan Maya[09]; Western Maya[18, 19]; Zapotec[21]
Used for (21#): Digestive (6)[01, 02, 03, 09, 18, 21]; Neurological (1)[18]; Respiratory (2)[01, 19]; Skin (3)[01, 02, 18]; Endocrine (3)[01, 02, 03]; Urological (2)[01, 09]; Pregnancy (1)[03]; Female genital (1)[03]; General and Unspecified (2)[01, 21]
Cognates:
Language contact:

***Ternstroemia lineata* DC. (Pentaphylacaceae)**

Spanish names: Té de tila
Indigenous names:
Used by (1*): Zapotec[21]
Used for (3#): Digestive (1)[21]; Cardiovascular (1)[21]; Psychological (1)[21]
Cognates:
Language contact:

***Ternstroemia* sp. (Pentaphylacaceae)**

Spanish names: Flor de tila
Indigenous names:
Used by (2*): Zoque[02, 03]
Used for (5#): Digestive (1)[03]; Cardiovascular (1)[03]; Psychological (2)[02, 03]; Pregnancy (1)[03]
Cognates:
Language contact:

***Ternstroemia sylvatica* Schltdl. et Cham. (Pentaphylacaceae)**

Spanish names: Flor de tila
Indigenous names: M¥a nanchiñ[03]
Used by (1*): Zoque[03]
Used for (2#): Digestive (1)[03]; Psychological (1)[03]
Cognates:
Language contact:

***Ternstroemia tepezapote* Cham. & Schldtl. (Pentaphylacaceae)**

Spanish names: Trompito/flor de tila
Indigenous names:
Used by (1*): Zoque[01]
Used for (4#): Digestive (1)[01]; Cardiovascular (1)[01]; Psychological (1)[01]; General and Unspecified (1)[01]
Cognates:
Language contact:

***Tetrachyron manicatum* Schltdl. (Asteraceae)**

Spanish names: Flor de enjambre
Indigenous names: Cerreroxochitl[26]
Used by (1*): Nahua[26]
Used for (1#): Musculoskeletal (1)[26]
Cognates:
Language contact:

***Tetramerium* sp. (Acanthaceae)**

Spanish names: Hoja de vergüenza
Indigenous names: J¥xi ay[03]
Used by (1*): Zoque[03]
Used for (1#): Psychological (1)[03]
Cognates:
Language contact:

***Teucrium cubense* Jacq. (Lamiaceae)**

Spanish names:
Indigenous names: Tihtsan kw'eet, weew ethem, t'othoy ts'ohool, thak pux, thak ch'a'ik ch'ohool[07]
Used by (1*): Huastec[07]
Used for (4#): Blood (1)[07]; Cardiovascular (1)[07]; Skin (1)[07]; General and Unspecified (1)[07]
Cognates:
Language contact:

***Thalictrum hernandezii* Tausch ex J. Presl (Ranunculaceae)**

Spanish names: Flor de San Antonio
Indigenous names:
Used by (1*): Quichean Maya[13]
Used for (2#): Neurological (1)[13]; General and Unspecified (1)[13]
Cognates:
Language contact:

***Thalictrum* sp. (Ranunculaceae)**

Spanish names:
Indigenous names: Guìzh-mèt,  guìzh-crùz-nguǎts, guìzh-tûb, guièer-ngüèts, ncuàan-dzéb-ròo[23]
Used by (1*): Zapotec[23]
Used for (1#): General and Unspecified (1)[23]
Cognates:
Language contact:

[01-28]refer to the study codes in Table 4.1. *Total number of studies citing this taxon #Total number of use-records

***Thalictrum standleyi* Steyerm. (Ranunculaceae)**

Spanish names: Hoja de azar
Indigenous names:
Used by (1*): Zoque[01]
Used for (1#): General and Unspecified (1)[01]
Cognates:
Language contact:

***Thelypteris* sp. (Thelypteridaceae)**

Spanish names: Colandrillo
Indigenous names:
Used by (1*): Zapotec[21]
Used for (1#): Urological (1)[21]
Cognates:
Language contact:

***Thelypteris tetragona* (Sw.) Small (Thelypteridaceae)**

Spanish names: Helecho
Indigenous names:
Used by (2*): Western Maya[18]; Nahua[24]
Used for (2#): Respiratory (1)[18]; Pregnancy (1)[24]
Cognates:
Language contact:

***Theobroma cacao* L. (Malvaceae)**

Spanish names: Cacao
Indigenous names: Kakawa[01]; Kakawa[02]; Caco[13]; Cacao, X'aq Cacao[14]; Cäcäw[18]; Yàg-bzèy[23]
Used by (9*): Zoque[01, 02]; Quichean Maya[12, 13, 14]; Western Maya[18]; Zapotec[21, 22, 23]
Used for (13#): Blood (1)[12]; Digestive (2)[01, 18]; Skin (1)[18]; Endocrine (1)[12]; Pregnancy (4)[01, 02, 13, 23]; General and Unspecified (4)[12, 14, 21, 22]
Cognates: Zoq: kakawa;
Language contact: MZ > CoreM and Spanish

***Thevetia ahouai* (L.) A.DC. (Apocynaceae)**

Spanish names: Coyal de burro
Indigenous names: Yoyak puj [01]; Puti kuy, burro puj, jatiks kumku[02]; Cha'lawaka, M¥aaktsa[03]; Atsømtu'ty[04]; Ch'ich'iyan, Naq'irit i' mis[14]; Ch'ich i jak[17]
Used by (6*): Zoque[01, 02, 03]; Mixe[04]; Quichean Maya[14, 17]
Used for (11#): Digestive (1)[14]; Musculoskeletal (1)[03]; Neurological (1)[04]; Skin (4)[01, 02, 03, 14]; Pregnancy (1)[03]; Female genital (1)[03]; General and Unspecified (1)[14]; nd[17]
Cognates: Zoq: puj; Quich: ch'ich'i ya/ja;
Language contact: CoreM > Chiapas Zoq

***Thevetia* sp. (Apocynaceae)**

Spanish names:
Indigenous names: Yoyotle[26]
Used by (1*): Nahua[26]
Used for (1#): General and Unspecified (1)[26]
Cognates:
Language contact:

***Thunbergia alata* Bojer ex Sims (Acanthaceae)**

Spanish names: Hierba del espanto
Indigenous names: Makti mooya[03]; Paxtama'tahua, pastak'natahuan[05]; Tsaayleel ts'ohool[07]
Used by (3*): Zoque[03]; Totonac[05]; Huastec[07]
Used for (5#): Digestive (1)[03]; Ear (1)[03]; Neurological (1)[07]; Skin (1)[03]; General and Unspecified (1)[05]
Cognates:
Language contact: Highland Popoluca <> Tot

***Thunbergia fragrans* Roxb. (Acanthaceae)**

Spanish names:
Indigenous names: Yommooya[03]; Ik' ts'ohool[07]
Used by (2*): Zoque[03]; Huastec[07]
Used for (3#): Ear (1)[03]; Neurological (1)[07]; General and Unspecified (1)[03]
Cognates:
Language contact:

***Thunbergia mysorensis* (Wight) T.Anderson (Acanthaceae)**

Spanish names: Tumbergia
Indigenous names: Rukama'l B'ochil Kotzij[12]
Used by (1*): Quichean Maya[12]
Used for (5#): Cardiovascular (1)[12]; Musculoskeletal (1)[12]; Psychological (1)[12]; Skin (1)[12]; General and Unspecified (1)[12]
Cognates:
Language contact:

***Thymus serpyllum* L. (Lamiaceae)**

Spanish names: Toronjil
Indigenous names:
Used by (1*): Quichean Maya[13]
Used for (1#): Respiratory (1)[13]
Cognates:
Language contact:

***Thymus vulgaris* L. (Lamiaceae)**

Spanish names: Tomillo
Indigenous names:
Used by (5*): Zoque[01]; Totonac[06]; Quichean Maya[12, 13]; Western Maya[19]
Used for (14#): Digestive (2)[06, 12]; Musculoskeletal (1)[12]; Neurological (1)[12]; Psychological (1)[12]; Respiratory (4)[01, 12, 13, 19]; Urological (1)[12]; Pregnancy (1)[12]; Female genital (1)[12]; General and Unspecified (2)[01, 12]
Cognates:
Language contact:

***Tibouchina longifolia* (Vahl) Baill. (Melastomataceae)**

Spanish names: Tesuatillo
Indigenous names: Cacaloxihuitle[26]
Used by (2*): Zoque[03]; Nahua[26]
Used for (4#): Digestive (1)[26]; Eye (1)[03]; Respiratory (1)[26]; Skin (1)[03]
Cognates:
Language contact:

***Tilia americana* L. (Malvaceae)**

Spanish names: Flor de tila; Tila
Indigenous names: Guièe-tîl[23]
Used by (3*): Huastec[08]; Zapotec[23]; Nahua[25]
Used for (7#): Digestive (2)[23, 25]; Eye (1)[08]; Cardiovascular (2)[08, 25]; Psychological (2)[08, 25]
Cognates:
Language contact:

[01-28]refer to the study codes in Table 4.1. *Total number of studies citing this taxon #Total number of use-records

***Tilia platyphyllos* Scop. (Malvaceae)**

Spanish names: Tilo
Indigenous names:
Used by (1*): Quichean Maya[12]
Used for (10#): Blood (1)[12]; Digestive (1)[12]; Cardiovascular (1)[12]; Musculoskeletal (1)[12]; Neurological (1)[12]; Psychological (2)[12, 12]; Urological (1)[12]; Female genital (1)[12]; General and Unspecified (1)[12]
Cognates:
Language contact:

***Tillandsia balbisiana* Schult. & Schult.f. (Bromeliaceae)**

Spanish names:
Indigenous names: Ch'u[09]
Used by (1*): Yucatecan Maya[09]
Used for (1#): Pregnancy (1)[09]
Cognates:
Language contact:

***Tillandsia brachycaulos* Schltdl. (Bromeliaceae)**

Spanish names:
Indigenous names: Ch'u[09]
Used by (1*): Yucatecan Maya[09]
Used for (1#): Female genital (1)[09]
Cognates:
Language contact:

***Tillandsia elongata* Kunth (Bromeliaceae)**

Spanish names:
Indigenous names: Ch'u[09]
Used by (1*): Yucatecan Maya[09]
Used for (1#): Female genital (1)[09]
Cognates:
Language contact:

***Tillandsia recurvata* (L.) L. (Bromeliaceae)**

Spanish names:
Indigenous names: Cuamimisi[24]
Used by (1*): Nahua[24]
Used for (1#): Female genital (1)[24]
Cognates:
Language contact:

***Tillandsia schiedeana* Steud. (Bromeliaceae)**

Spanish names:
Indigenous names: Tsakam k'ok'om[07]; Ch'u[09]
Used by (2*): Huastec[07]; Yucatecan Maya[09]
Used for (3#): Neurological (1)[07]; Pregnancy (1)[09]; General and Unspecified (1)[07]
Cognates:
Language contact:

***Tillandsia* sp. (Bromeliaceae)**

Spanish names: Pie de Gallo
Indigenous names: Ch'u[09]; Roq Aj Tzo'[14]
Used by (2*): Yucatecan Maya[09]; Quichean Maya[14]
Used for (2#): Skin (1)[14]; Female genital (1)[09]
Cognates:
Language contact: Yuc <> Kekchí

[01-28]refer to the study codes in Table 4.1. *Total number of studies citing this taxon #Total number of use-records

***Tillandsia usneoides*** **(L.) L. (Bromeliaceae)**

Spanish names: Paxte, pashtio
Indigenous names: K'uthay[07]; Q'ux[13]
Used by (2*): Huastec[07]; Quichean Maya[13]
Used for (6#): Musculoskeletal (1)[13]; Respiratory (1)[07]; Skin (1)[07]; Pregnancy (1)[07]; General and Unspecified (2)[07, 13]
Cognates:
Language contact: Hua <> K'iche'

***Tinantia erecta*** **(Jacq.) Fenzl (Commelinaceae)**

Spanish names: Pata de pollo, pata de gallo; Siemprevive blanca
Indigenous names: Tzui[01]; Quix'tac[06]
Used by (2*): Zoque[01]; Totonac[06]
Used for (7#): Digestive (1)[06]; Psychological (1)[01]; Skin (2)[01, 06]; Urological (1)[06]; General and Unspecified (2)[01, 06]
Cognates:
Language contact:

***Tithonia diversifolia*** **(Hemsl.) A.Gray (Asteraceae)**

Spanish names: Arnica, girasol
Indigenous names: Tapungäsy jäyä/tapkuy ay/tat kuy/tan tzitzi/tab kuy/tam tzitzi[01]; Tam tzyi/ tam tzyitz/ tam ay[02]; Tam chich[03]; Nø tammtsy[04]; Chaksu'um[09]; S'um Aq'om[12]; Ch'aj[18]; Ch'ajkil, neek ch'a te', k'ail, p'ilix[20]; Ru'ulá[21]; Bnîil-zhĭil, guièe-bnîil-zhĭil, guìzh-bnîil-zhĭil, guièe-bnîil-yòx, blàg-bnîil, guièe-chòg-zhĭil[23]
Used by (11*): Zoque[01, 02, 03]; Mixe[04]; Yucatecan Maya[09]; Quichean Maya[12]; Western Maya[18, 20]; Zapotec[21, 23]; Nahua[26]
Used for (57#): Blood (2)[02, 12]; Digestive (7)[01, 02, 03, 12, 18, 20, 21]; Eye (1)[02]; Cardiovascular (2)[02, 02]; Musculoskeletal (8)[01, 02, 03, 04, 09, 12, 21, 23]; Neurological (1)[18]; Psychological (2)[02, 23]; Respiratory (4)[01, 02, 03, 21]; Skin (8)[01, 02, 03, 04, 18, 20, 21, 26]; Endocrine (3)[01, 02, 03]; Urological (1)[02]; Pregnancy (4)[01, 02, 03, 21]; Female genital (5)[01, 02, 03, 18, 21]; Male genital (1)[02]; General and Unspecified (8)[01, 02, 03, 04, 12, 18, 20, 21]
Cognates: MZ: tamtsi; Zoq: tamtsits; CoreM: chaj/chak, s'um; WesM: ch'aj;
Language contact: MZ > Zap

***Tithonia longiradiata*** **(Bertol.) S.F.Blake (Asteraceae)**

Spanish names: Arnica
Indigenous names: Kaj Tajiq'os[12]
Used by (1*): Quichean Maya[12]
Used for (4#): Digestive (1)[12]; Cardiovascular (1)[12]; Musculoskeletal (1)[12]; Skin (1)[12]
Cognates:
Language contact:

***Tithonia tubaeformis*** **(Jacq.) Cass. (Asteraceae)**

Spanish names: Mirasol
Indigenous names: Bârr-dòo-lă[23]; Acahuali[28]
Used by (2*): Zapotec[23]; Nahua[28]
Used for (6#): Digestive (2)[23, 28]; Cardiovascular (1)[23]; Musculoskeletal (1)[28]; Psychological (1)[23]; Skin (1)[23]
Cognates:
Language contact:

***Tonduzia stenophylla*** **(Donn.Sm.) Pittier (Apocynaceae)**

Spanish names:
Indigenous names: Akab' K'elem[14]
Used by (1*): Quichean Maya[14]
Used for (3#): Digestive (1)[14]; Skin (1)[14]; General and Unspecified (1)[14]
Cognates:
Language contact:

[01-28] refer to the study codes in Table 4.1. *Total number of studies citing this taxon #Total number of use-records

***Topobea watsonii* Cogn. (Melastomataceae)**

Spanish names:
Indigenous names: Oxlaju Ch'ajom, Kaq'i Ch'ajom, Yot' eq [14]
Used by (1*): Quichean Maya[14]
Used for (1#): Digestive (1)[14]
Cognates:
Language contact:

***Tournefortia bicolor* Sw. (Boraginaceae)**

Spanish names: Hoja de aire
Indigenous names:
Used by (1*): Zoque[01]
Used for (2#): Musculoskeletal (1)[01]; Neurological (1)[01]
Cognates:
Language contact:

***Tournefortia densiflora* M.Martens & Galeotti (Boraginaceae)**

Spanish names: Hoja de cancer
Indigenous names: Biajtu mshtajala[21]
Used by (1*): Zapotec[21]
Used for (5#): Digestive (1)[21]; Eye (1)[21]; Musculoskeletal (1)[21]; Skin (1)[21]; Female genital (1)[21]
Cognates:
Language contact:

***Tournefortia glabra* L. (Boraginaceae)**

Spanish names: Hoja de cancer
Indigenous names:
Used by (1*): Mixe[04]
Used for (1#): Skin (1)[04]
Cognates:
Language contact:

***Tournefortia hirsutissima* L. (Boraginaceae)**

Spanish names:
Indigenous names: Mach'much[07]
Used by (1*): Huastec[07]
Used for (2#): Skin (1)[07]; General and Unspecified (1)[07]
Cognates:
Language contact:

***Tournefortia mutabilis* Vent. (Boraginaceae)**

Spanish names: Yerba del cancer
Indigenous names:
Used by (1*): Zapotec[22]
Used for (1#): Skin (1)[22]
Cognates:
Language contact:

***Tournefortia* sp. (Boraginaceae)**

Spanish names: Hoja de cáncer
Indigenous names: Waylom te'[07]; Kann'bolie kejen[16]; Blàg-chòg, guìzh-blàg-chòg, guìzh-chòg, guìzh-cânzr[23]
Used by (3*): Huastec[07]; Quichean Maya[16]; Zapotec[23]
Used for (4#): Neurological (1)[16]; Respiratory (1)[23]; Skin (1)[23]; General and Unspecified (1)[07]
Cognates:
Language contact:

***Tournefortia volubilis* L. (Boraginaceae)**

Spanish names:
Indigenous names: Xulk'ini, Sal[09]
Used by (1*): Yucatecan Maya[09]
Used for (1#): Skin (1)[09]
Cognates:
Language contact:

***Toxicodendron radicans* (L.) Kuntze (Anacardiaceae)**

Spanish names:
Indigenous names: Ja'yi[01]
Used by (1*): Zoque[01]
Used for (1#): Skin (1)[01]
Cognates:
Language contact:

***Tradescantia commelinoides* Schult. & Schult.f. (Commelinaceae)**

Spanish names: Uña de Gato, Tripa de Gallina
Indigenous names: Loch'och[12]
Used by (1*): Quichean Maya[12]
Used for (3#): Respiratory (1)[12]; Skin (1)[12]; General and Unspecified (1)[12]
Cognates:
Language contact:

***Tradescantia guatemalensis* C.B.Clarke ex J.D.Sm. (Commelinaceae)**

Spanish names: Hierba de pollo; Siemprevive menuda/ hierba de pollo
Indigenous names: (Nama) tzui[01]; Pitzijor[13]
Used by (2*): Zoque[01]; Quichean Maya[13]
Used for (7#): Digestive (1)[13]; Psychological (1)[01]; Respiratory (1)[01]; Skin (1)[13]; Urological (1)[13]; Female genital (1)[01]; General and Unspecified (1)[01]
Cognates:
Language contact:

***Tradescantia pallida* (Rose) D.R.Hunt (Commelinaceae)**

Spanish names: Moradito
Indigenous names:
Used by (2*): Zoque[03]; Zapotec[21]
Used for (4#): Digestive (1)[21]; Skin (1)[21]; Female genital (1)[03]; General and Unspecified (1)[21]
Cognates:
Language contact:

***Tradescantia schippii* D.R.Hunt (Commelinaceae)**

Spanish names:
Indigenous names: Ka'paj[14]
Used by (1*): Quichean Maya[14]
Used for (1#): General and Unspecified (1)[14]
Cognates:
Language contact:

[01-28]refer to the study codes in Table 4.1. *Total number of studies citing this taxon #Total number of use-records

### *Tradescantia* sp. (Commelinaceae)

Spanish names: Hierba de Pollo
Indigenous names: Ru Guaij a'k[12]; X-tòoz-pěch, guìzh-pěch , guìzh-guièe-pěch, blàg-pěch, x-tòoz-ngă[23]
Used by (2*): Quichean Maya[12]; Zapotec[23]
Used for (6[#]): Digestive (1)[12]; Neurological (1)[12]; Respiratory (1)[12]; Skin (1)[12]; Pregnancy (1)[23]; General and Unspecified (1)[12]
Cognates:
Language contact:

### *Tradescantia spathacea* Sw. (Commelinaceae)

Spanish names: Maguey morado, sábila morada
Indigenous names: Tzukin u /katzu o /katzu ay/tzapas o[01]; Jam'ay[02]; Chakts'am[09]; Asuseen itzuul[14]; Chächäkpimi[18]
Used by (8*): Zoque[01, 02, 03]; Yucatecan Maya[09, 10]; Quichean Maya[14]; Western Maya[18]; Zapotec[21]
Used for (40[#]): Digestive (5)[01, 02, 03, 18, 21]; Ear (1)[01]; Cardiovascular (3)[02, 03, 18]; Musculoskeletal (4)[01, 02, 10, 21]; Neurological (2)[14, 18]; Psychological (1)[18]; Respiratory (4)[01, 02, 03, 18]; Skin (6)[01, 02, 09, 14, 18, 21]; Endocrine (1)[02]; Urological (3)[01, 03, 18]; Pregnancy (1)[02]; Female genital (5)[01, 02, 03, 18, 21]; General and Unspecified (4)[01, 02, 18, 21]
Cognates:
Language contact: Chiapas Zoq <> Kekchí <> Yuc <> Chontal <> Chimalapa Zoq

### *Tradescantia standleyi* Steyerm. (Commelinaceae)

Spanish names:
Indigenous names: Uky ujts[04]
Used by (1*): Mixe[04]
Used for (1[#]): Urological (1)[04]
Cognates:
Language contact:

### *Tradescantia zanonia* (L.) Sw. (Commelinaceae)

Spanish names: Hoja de azar/tinta china
Indigenous names: Tza'a tzoy[01]; Paktha' utek', tsakam k'oyol, uto, yax tsamnek[07]; Saqi' choq'l, Tz'i' Maaj Q'ehen[14]
Used by (3*): Zoque[01]; Huastec[07]; Quichean Maya[14]
Used for (6[#]): Ear (1)[07]; Cardiovascular (1)[14]; Neurological (1)[14]; Skin (1)[14]; Endocrine (1)[14]; General and Unspecified (1)[01]
Cognates:
Language contact:

### *Tradescantia zebrina* Bosse (Commelinaceae)

Spanish names: Hierba del pollo, siempreviva, madali
Indigenous names: (Tzapas) tzui[01]; Jäs'ukä potznä[02]; Tsabats uixpin[03]; Tsak utek', xutstun pathaam, mili' ch'ohool[07]; Ru Guaij a'k[12]; Rak'eq'[13]; Cha cha[15]; Yop'ixpätz[18]; Ak'ach k'opot[19]; Madali[21]; Matlali[25]; Matlale[26]
Used by (12*): Zoque[01, 02, 03]; Huastec[07]; Quichean Maya[12, 13, 15]; Western Maya[18, 19]; Zapotec[21]; Nahua[25, 26]
Used for (35[#]): Blood (1)[01]; Digestive (5)[01, 02, 07, 18, 26]; Eye (2)[01, 13]; Cardiovascular (1)[26]; Musculoskeletal (1)[21]; Neurological (3)[07, 12, 18]; Skin (6)[01, 02, 12, 13, 21, 26]; Endocrine (2)[01, 18]; Urological (3)[01, 18, 26]; Pregnancy (1)[13]; Female genital (3)[01, 03, 15]; General and Unspecified (7)[01, 07, 12, 19, 21, 25, 26]
Cognates: Mayan: ak; CoreM: ak'V; Nahua: matlali;
Language contact: Chimalapa Zoq <> Chontal; Nah > Spanish

### *Tragia mexicana* Müll.Arg. (Euphorbiaceae)

Spanish names:
Indigenous names: Tiplay', thinlay', chalam tiya[07]
Used by (1*): Huastec[07]
Used for (2[#]): Musculoskeletal (1)[07]; General and Unspecified (1)[07]
Cognates:
Language contact:

[01-28]refer to the study codes in Table 4.1. *Total number of studies citing this taxon [#]Total number of use-records

***Tragia* sp. (Euphorbiaceae)**

Spanish names:
Indigenous names: Tsay kenuk[03]; P'op'ox[09]
Used by (2*): Zoque[03]; Yucatecan Maya[09]
Used for (2#): Musculoskeletal (1)[09]; Urological (1)[03]
Cognates:
Language contact:

***Tragia yucatanensis* Millsp. (Euphorbiaceae)**

Spanish names:
Indigenous names: P'ŏop'oŠ[10]
Used by (1*): Yucatecan Maya[10]
Used for (2#): Musculoskeletal (1)[10]; Skin (1)[10]
Cognates:
Language contact:

***Trema micrantha* (L.) Blume (Cannabaceae)**

Spanish names: Capulín; Majagua mixe, jonote
Indigenous names: Tzay kuy [01]; Puwaamte[07]; Lajsa'a baagui'[21]
Used by (3*): Zoque[01]; Huastec[07]; Zapotec[21]
Used for (3#): Skin (1)[21]; Urological (1)[01]; General and Unspecified (1)[07]
Cognates:
Language contact:

***Trichilia glabra* L. (Meliaceae)**

Spanish names:
Indigenous names: Čabon Če[10]
Used by (1*): Yucatecan Maya[10]
Used for (1#): Digestive (1)[10]
Cognates:
Language contact:

***Trichilia havanensis* Jacq. (Meliaceae)**

Spanish names: Cualimón, limoncillo; Palo cuchara; Tinajillo
Indigenous names: Yooni cuy[03]; Thokob saantu, k'olol te', thiblab[07]
Used by (4*): Zoque[03]; Totonac[05]; Huastec[07]; Quichean Maya[13]
Used for (12#): Blood (2)[03, 07]; Neurological (2)[03, 07]; Psychological (1)[07]; Respiratory (2)[03, 07]; Pregnancy (1)[03]; General and Unspecified (4)[03, 05, 07, 13]
Cognates:
Language contact:

***Trichocentrum ascendens* (Lindl.) M.W.Chase & N.H.Williams (Orchidaceae)**

Spanish names:
Indigenous names: Puts'ubche', Bac chivo, Puts'maskab[09]
Used by (1*): Yucatecan Maya[09]
Used for (1#): Skin (1)[09]
Cognates:
Language contact:

***Trichocentrum carthagenense* (Jacq.) M.W.Chase & N.H.Williams (Orchidaceae)**

Spanish names:
Indigenous names: Xutsun buuru, ko'lom te'[07]; U'tsumpek[09]
Used by (2*): Huastec[07]; Yucatecan Maya[09]
Used for (2#): Neurological (1)[07]; Pregnancy (1)[09]
Cognates:
Language contact:

***Trichomanes pinnatum* Hedw. (Hymenophyllacae)**

Spanish names:
Indigenous names: Chimal[03]
Used by (1*): Zoque[03]
Used for (1#): Neurological (1)[03]
Cognates:
Language contact:

***Tridax procumbens* (L.) L. (Asteraceae)**

Spanish names: Hierba del toro; Hoja de azar
Indigenous names: Tza'a tzoy[01]; Wakax k'opot[19]
Used by (2*): Zoque[01]; Western Maya[19]
Used for (5#): Blood (1)[19]; Musculoskeletal (1)[19]; Neurological (1)[19]; General and Unspecified (2)[01, 19]
Cognates:
Language contact:

***Trifolium amabile* Kunth (Fabaceae)**

Spanish names:
Indigenous names: Dĭp-rên,  guièe-frôw[23]
Used by (1*): Zapotec[23]
Used for (1#): General and Unspecified (1)[23]
Cognates:
Language contact:

***Trifolium repens* L. (Fabaceae)**

Spanish names: Trebol
Indigenous names:
Used by (1*): Nahua[26]
Used for (1#): Cardiovascular (1)[26]
Cognates:
Language contact:

***Trigonella foenum-graecum* L. (Fabaceae)**

Spanish names: Fenogreco
Indigenous names:
Used by (1*): Quichean Maya[12]
Used for (4#): Blood (1)[12]; Digestive (1)[12]; Skin (1)[12]; General and Unspecified (1)[12]
Cognates:
Language contact:

***Tripogandra grandiflora* (Donn.Sm.) Woodson (Commelinaceae)**

Spanish names:
Indigenous names: Ik B'olay Q'ehen[14]; Tzima'j k'ejen[17]
Used by (2*): Quichean Maya[14, 17]
Used for (4#): Musculoskeletal (1)[14]; Skin (1)[14]; General and Unspecified (1)[14]; nd[17]
Cognates:
Language contact:

[01-28]refer to the study codes in Table 4.1.  *Total number of studies citing this taxon  #Total number of use-records

***Tripogandra serrulata* (Vahl) Handlos (Commelinaceae)**

Spanish names: Matalillo, siempreviva
Indigenous names: Tzapas tzui[01]; Tsus uixpin[03]; Kasmal[05]; A'kasman[06]; Utek witssil thakni', tsakam tsahib[07]; Matlale morado[26]
Used by (7*): Zoque[01, 03]; Totonac[05, 06]; Huastec[07]; Zapotec[21]; Nahua[26]
Used for (12#): Digestive (3)[05, 06, 07]; Cardiovascular (1)[26]; Psychological (1)[01]; Skin (3)[01, 07, 21]; Endocrine (1)[07]; Urological (1)[26]; General and Unspecified (2)[03, 07]
Cognates: Toto: kasmaC;
Language contact:

***Tripogandra* sp. (Commelinaceae)**

Spanish names:
Indigenous names: X-tòoz-pěch, guìzh-pěch , guìzh-guièe-pěch, blàg-pěch, x-tòoz-ngă[23]
Used by (1*): Zapotec[23]
Used for (1#): Pregnancy (1)[23]
Cognates:
Language contact:

***Triumfetta bogotensis* DC. (Malvaceae)**

Spanish names:
Indigenous names: Woyo'o pim[14]
Used by (2*): Quichean Maya[14]; Western Maya[20]
Used for (4#): Digestive (2)[14, 20]; Pregnancy (1)[14]; Female genital (1)[14]
Cognates:
Language contact:

***Triumfetta polyandra* Sessé & Moc. ex DC. (Malvaceae)**

Spanish names:
Indigenous names: Ueji ay[03]
Used by (1*): Zoque[03]
Used for (2#): Skin (1)[03]; General and Unspecified (1)[03]
Cognates:
Language contact:

***Triumfetta semitriloba* Jacq. (Malvaceae)**

Spanish names: Cadillo; Mozote
Indigenous names: Konko[03]; Thipaxi', pitaxe'[07]; Mul-och[09]; Cuoyo'[17]
Used by (5*): Zoque[01, 03]; Huastec[07]; Yucatecan Maya[09]; Quichean Maya[17]
Used for (11#): Digestive (3)[01, 07, 09]; Musculoskeletal (1)[07]; Respiratory (1)[07]; Skin (1)[03]; Urological (2)[03, 07]; Pregnancy (2)[07, 09]; nd[17]
Cognates: Mayan: ax/ox/oyo;
Language contact:

***Triumfetta* sp. (Malvaceae)**

Spanish names:
Indigenous names: Kambapixoy[09]
Used by (1*): Yucatecan Maya[09]
Used for (2#): Pregnancy (1)[09]; Female genital (1)[09]
Cognates:
Language contact:

[01-28]refer to the study codes in Table 4.1. *Total number of studies citing this taxon #Total number of use-records

***Triumfetta speciosa* Seem. (Malvaceae)**

Spanish names: Majagua blanca; Mozote
Indigenous names: Tzo kon[13]; Yag lass[21]
Used by (2*): Quichean Maya[13]; Zapotec[21]
Used for (2#): Digestive (1)[13]; Skin (1)[21]
Cognates:
Language contact:

***Trixis inula* Crantz (Asteraceae)**

Spanish names: Contrimbruja
Indigenous names: Pub kw'ahiil, , thak ts'aah[07]; Fluxion xiw, Xtankas-ak'[09]
Used by (4*): Zoque[03]; Huastec[07]; Yucatecan Maya[09]; Zapotec[21]
Used for (9#): Digestive (2)[07, 21]; Neurological (1)[09]; Respiratory (1)[07]; Skin (2)[03, 07]; Pregnancy (1)[07]; General and Unspecified (2)[03, 07]
Cognates: Mayan: thak ts'aah / tankas;
Language contact:

***Tropaeolum majus* L. (Tropaeolaceae)**

Spanish names: Montwerz
Indigenous names: Màltuêrs[23]
Used by (2*): Zapotec[22, 23]
Used for (5#): Neurological (2)[22, 23]; Respiratory (1)[23]; General and Unspecified (2)[22, 23]
Cognates:
Language contact:

***Trophis racemosa* (L.) Urb. (Moraceae)**

Spanish names:
Indigenous names: Tsumte', chumte'[07]
Used by (1*): Huastec[07]
Used for (2#): Neurological (1)[07]; General and Unspecified (1)[07]
Cognates:
Language contact:

***Turbina corymbosa* (L.) Raf. (Convolvulaceae)**

Spanish names: Bejuco de viento; Flor de virgen ; Piule
Indigenous names: Atsay[03]; Pamaxuunk[04]; Xtabentun[09]; Badooj[21]; Zhì-ncuàan-ná-zhnâzh[23]
Used by (5*): Zoque[03]; Mixe[04]; Yucatecan Maya[09]; Zapotec[21, 23]
Used for (10#): Digestive (1)[03]; Neurological (1)[09]; Psychological (2)[03, 23]; Respiratory (1)[09]; Pregnancy (1)[09]; General and Unspecified (4)[03, 04, 21, 23]
Cognates:
Language contact:

***Turnera diffusa* Willd. ex Schult. (Passifloraceae)**

Spanish names: Hoja de fiebre; Itamo real; Oregano de monte; Salvia blanca
Indigenous names: Oregano k'ax[09]; Guish fiebre[21]; Salb nol[22]
Used by (4*): Yucatecan Maya[09]; Zapotec[21, 22]; Nahua[27]
Used for (4#): Digestive (2)[22, 27]; Respiratory (1)[09]; General and Unspecified (1)[21]
Cognates:
Language contact:

***Turnera pumilea* L. (Passifloraceae)**

Spanish names: Flor de linda tarde
Indigenous names:
Used by (1*): Zoque[02]
Used for (1#): Skin (1)[02]
Cognates:
Language contact:

***Turnera ulmifolia* L. (Passifloraceae)**

Spanish names: Malva blanca
Indigenous names: Lexuuba'a quitzii[21]
Used by (2*): Zoque[03]; Zapotec[21]
Used for (5#): Digestive (1)[21]; Skin (2)[03, 21]; Pregnancy (1)[21]; General and Unspecified (1)[03]
Cognates:
Language contact:

***Ulmus mexicana* (Liebm.) Planch. ex DC (Ulmaceae)**

Spanish names: Palo cuero
Indigenous names: Pagaxniakcuy[03]
Used by (1*): Zoque[03]
Used for (1#): Pregnancy (1)[03]
Cognates:
Language contact:

***Urera baccifera* (L.) Gaudich. ex Wedd. (Urticaceae)**

Spanish names: Ortiga
Indigenous names:
Used by (1*): Yucatecan Maya[11]
Used for (2#): Musculoskeletal (1)[11]; General and Unspecified (1)[11]
Cognates:
Language contact:

***Urera caracasana* (Jacq.) Gaudich. ex Griseb. (Urticaceae)**

Spanish names: Chichicaste de Caballo; Ortiga real; Ortiga, pica-pica
Indigenous names: Aaamlay', pulik pux lat'em[07]; Laal[09]; Lej', Läjy, Yel[12]
Used by (5*): Zoque[03]; Huastec[07]; Yucatecan Maya[09]; Quichean Maya[12]; Nahua[25]
Used for (12#): Digestive (1)[09]; Eye (1)[07]; Musculoskeletal (4)[03, 09, 12, 25]; Neurological (1)[09]; Respiratory (1)[25]; Skin (1)[03]; Urological (1)[12]; General and Unspecified (2)[07, 12]
Cognates: Mayan: lay/lal/laj;
Language contact:

***Urera elata* (Sw.) Griseb. (Urticaceae)**

Spanish names: Ortiga, chichicaste
Indigenous names: Ley'[12]; Oqob' Q'ehen[14]
Used by (2*): Quichean Maya[12, 14]
Used for (3#): Respiratory (1)[14]; Urological (1)[12]; General and Unspecified (1)[12]
Cognates:
Language contact:

***Urera* sp. (Urticaceae)**

Spanish names: Chichicaste
Indigenous names: Tsooklay', choklay'[07]; La'[13]
Used by (2*): Huastec[07]; Quichean Maya[13]
Used for (5#): Musculoskeletal (2)[07, 13]; Psychological (1)[07]; Respiratory (1)[07]; General and Unspecified (1)[07]
Cognates: Mayan: la;
Language contact:

[01-28]refer to the study codes in Table 4.1. *Total number of studies citing this taxon #Total number of use-records

***Urtica chamaedryoides* Pursh (Urticaceae)**

Spanish names: Ortiga menor/ bejucillo
Indigenous names: Menuk/ kenuk[01]; La'ix iits', tsakam tsooklay'[07]
Used by (2*): Zoque[01]; Huastec[07]
Used for (4#): Musculoskeletal (2)[01, 07]; Urological (1)[01]; Female genital (1)[01]
Cognates:
Language contact:

***Urtica dioica* L. (Urticaceae)**

Spanish names: Ortiga
Indigenous names:
Used by (1*): Quichean Maya[12]
Used for (2#): Urological (1)[12]; General and Unspecified (1)[12]
Cognates:
Language contact:

***Urtica urens* L. (Urticaceae)**

Spanish names: Ortiga roja
Indigenous names: Ley' pi'y[12]
Used by (1*): Quichean Maya[12]
Used for (6#): Blood (1)[12]; Cardiovascular (1)[12]; Neurological (1)[12]; Psychological (1)[12]; Skin (1)[12]; General and Unspecified (1)[12]
Cognates:
Language contact:

***Urvillea ulmacea* Kunth (Sapindaceae)**

Spanish names:
Indigenous names: Tsaayleelaab ts'ohool, itsaan an t'in kamaab, tsank'ub ts'aah[07]
Used by (1*): Huastec[07]
Used for (2#): Neurological (1)[07]; General and Unspecified (1)[07]
Cognates:
Language contact:

***Valeriana officinalis* L. (Caprifoliaceae)**

Spanish names: Valeriana
Indigenous names:
Used by (1*): Quichean Maya[12]
Used for (7#): Digestive (1)[12]; Musculoskeletal (1)[12]; Neurological (1)[12]; Psychological (1)[12]; Urological (1)[12]; Female genital (1)[12]; General and Unspecified (1)[12]
Cognates:
Language contact:

***Valeriana scandens* L. (Caprifoliaceae)**

Spanish names: Bejuco silvestre, hierba de perrito; Hierba azar de guía/gua blanca
Indigenous names: Po'o sudan/ poye rane / Nakta[01]; Puua ay, Puutx ay[03]; Tetziltzi, tlacote[26]
Used by (3*): Zoque[01, 03]; Nahua[26]
Used for (8#): Digestive (1)[01]; Musculoskeletal (1)[01]; Psychological (1)[01]; Skin (3)[01, 03, 26]; General and Unspecified (2)[01, 03]
Cognates: Zoq: po'o/puua;
Language contact:

[01-28]refer to the study codes in Table 4.1. *Total number of studies citing this taxon #Total number of use-records

***Valeriana sorbifolia*** **Kunth (Caprifoliaceae)**

Spanish names: Valeriana Silvestre
Indigenous names:
Used by (1*): Quichean Maya[12]
Used for (3#): Cardiovascular (1)[12]; Neurological (1)[12]; Psychological (1)[12]
Cognates:
Language contact:

***Valeriana*** **sp. (Caprifoliaceae)**

Spanish names:
Indigenous names: Guìzh-ngùd-lèn, ngùd-lèn, gù-vàlèriân[23]
Used by (1*): Zapotec[23]
Used for (5#): Digestive (1)[23]; Musculoskeletal (1)[23]; Endocrine (1)[23]; Female genital (1)[23]; General and Unspecified (1)[23]
Cognates:
Language contact:

***Valeriana urticifolia*** **Kunth (Caprifoliaceae)**

Spanish names: Valeriana
Indigenous names: Uxe' ulew[13]
Used by (1*): Quichean Maya[13]
Used for (4#): Neurological (1)[13]; Psychological (1)[13]; Skin (1)[13]; General and Unspecified (1)[13]
Cognates:
Language contact:

***Vanilla planifolia*** **Jacks. ex Andrews (Orchidaceae)**

Spanish names: Vainilla
Indigenous names: Suumii'xa'nat[06]
Used by (3*): Mixe[04]; Totonac[06]; Zapotec[21]
Used for (4#): Skin (1)[06]; Pregnancy (1)[06]; General and Unspecified (2)[04, 21]
Cognates:
Language contact:

***Vasconcellea cauliflora*** **(Jacq.) A.DC. (Caricaceae)**

Spanish names: Papaya cimarrona; Papaya de Montaña; Papaya simarron
Indigenous names: Otzo'[01]; Mäki mama[02]; Tutun 'chich, tatan chich[06]; Q'ilx Q'en[14]
Used by (4*): Zoque[01, 02]; Totonac[06]; Quichean Maya[14]
Used for (7#): Digestive (4)[01, 02, 06, 14]; Cardiovascular (1)[14]; Neurological (1)[02]; Respiratory (1)[06]
Cognates:
Language contact:

***Vatairea lundellii*** **(Standl.) Killip. ex Record (Fabaceae)**

Spanish names: Palo picho
Indigenous names: Y¥k cuy[03]
Used by (1*): Zoque[03]
Used for (1#): Digestive (1)[03]
Cognates:
Language contact:

***Vellozia caudata*** **Mello-Silva (Velloziaceae)**

Spanish names:
Indigenous names: Susm kik[14]
Used by (1*): Quichean Maya[14]
Used for (1#): Neurological (1)[14]
Cognates:
Language contact:

[01-28]refer to the study codes in Table 4.1. *Total number of studies citing this taxon #Total number of use-records

***Verbascum thapsus* L. (Scrophulariaceae)**

Spanish names: Oreja de Burro, Hoja Santa
Indigenous names:
Used by (1*): Quichean Maya[12]
Used for (9#): Blood (1)[12]; Digestive (2)[12, 12]; Neurological (1)[12]; Respiratory (1)[12]; Skin (1)[12]; Urological (1)[12]; Female genital (1)[12]; General and Unspecified (1)[12]
Cognates:
Language contact:

***Verbena carolina* L. (Verbenaceae)**

Spanish names: Verbena
Indigenous names: Pem k'ulub, yakan k'ulub wamal, yaxal tob tz'i'lel, yax uran nich momol[20]; Ptiôn-dán, guìzh-ptiôn, guièe-ptiôn, bít-wĩ[23]; Ndinich[28]
Used by (4*): Quichean Maya[12]; Western Maya[20]; Zapotec[23]; Nahua[28]
Used for (7#): Digestive (2)[20, 28]; Psychological (1)[23]; Respiratory (1)[20]; General and Unspecified (3)[12, 20, 28]
Cognates:
Language contact: Nah <> Tzeltalan

***Verbena litoralis* Kunth (Verbenaceae)**

Spanish names: Verbena
Indigenous names: Ejtil i kwayab ts'aale[08]; Chachal' b'ey[12]; Xkis Kawaay [14]; Pem k'ulub, yakan k'ulub wamal[20]
Used by (7*): Totonac[05]; Huastec[08]; Quichean Maya[12, 14]; Western Maya[18, 19, 20]
Used for (26#): Blood (2)[05, 12]; Digestive (6)[05, 08, 12, 18, 19, 20]; Musculoskeletal (3)[12, 14, 19]; Neurological (2)[12, 14]; Psychological (1) [12]; Respiratory (3)[12, 18, 20]; Skin (1)[14]; Urological (1)[12]; Female genital (2)[12, 14]; General and Unspecified (5)[12, 14, 18, 19, 20]
Cognates:
Language contact: Hua <> Kekchí

***Verbena menthifolia* Benth. (Verbenaceae)**

Spanish names: Verbena
Indigenous names: Ohpanchichic[26]; Ndinich[28]
Used by (2*): Nahua[26, 28]
Used for (2#): Digestive (1)[26]; General and Unspecified (1)[28]
Cognates:
Language contact:

***Verbena officinalis* L. (Verbenaceae)**

Spanish names: Pitiona
Indigenous names: Ptiôn[23]
Used by (1*): Zapotec[23]
Used for (1#): Digestive (1)[23]
Cognates:
Language contact:

***Verbena recta* Kunth (Verbenaceae)**

Spanish names: Verbena
Indigenous names:
Used by (1*): Zapotec[21]
Used for (1#): Digestive (1)[21]
Cognates:
Language contact:

***Verbena* sp. (Verbenaceae)**

Spanish names: Diente de ratón; Hierba buenilla, moradita, ; Verbena
Indigenous names: Tung'an petkuy/kan petkuy/tak'an petkuy/tzitzirane/ takak rane/ tuk tane [01]; Uchachal be', werwen, chachal be'[13]; Clanquimiche[26]
Used by (3*): Zoque[01]; Quichean Maya[13]; Nahua[26]
Used for (19#): Digestive (4)[01, 13, 26, 26]; Musculoskeletal (3)[01, 13, 26]; Neurological (1)[13]; Psychological (1)[01]; Respiratory (1)[13]; Skin (1)[01]; Endocrine (1)[01]; Urological (1)[01]; Pregnancy (3)[01, 13, 26]; General and Unspecified (3)[01, 13, 26]
Cognates:
Language contact:

***Verbesina crocata* (Cav.) Less. (Asteraceae)**

Spanish names: Arnica
Indigenous names: Axocopa[26]
Used by (2*): Nahua[26, 27]
Used for (5#): Respiratory (1)[27]; Skin (1)[27]; Pregnancy (2)[26, 27]; General and Unspecified (1)[27]
Cognates:
Language contact:

***Verbesina fastigiata* B.L.Rob. & Greenm. (Asteraceae)**

Spanish names: Bordón de vieja; Chimpfloque
Indigenous names: Oko kaku[02]
Used by (2*): Zoque[01, 02]
Used for (4#): Digestive (1)[01]; Musculoskeletal (1)[01]; Skin (2)[01, 02]
Cognates:
Language contact:

***Verbesina fraseri* Hemsl. (Asteraceae)**

Spanish names: Capitanec, Capitaneja
Indigenous names:
Used by (1*): Quichean Maya[12]
Used for (3#): Digestive (1)[12]; Musculoskeletal (1)[12]; General and Unspecified (1)[12]
Cognates:
Language contact:

***Verbesina gigantea* Jacq. (Asteraceae)**

Spanish names:
Indigenous names: Chulkeeh[09]
Used by (1*): Yucatecan Maya[09]
Used for (1#): Respiratory (1)[09]
Cognates:
Language contact:

***Verbesina persicifolia* DC. (Asteraceae)**

Spanish names: Huichim; Huixin; Machichili
Indigenous names: Witsiim, wichin[07]; Taxiwua[08]
Used by (4*): Totonac[05, 06]; Huastec[07, 08]
Used for (17#): Digestive (3)[05, 06, 07]; Ear (1)[07]; Musculoskeletal (2)[05, 07]; Neurological (1)[07]; Skin (3)[05, 06, 07]; Endocrine (2)[05, 06]; Urological (1)[05]; Pregnancy (1)[06]; General and Unspecified (3)[05, 07, 08]
Cognates:
Language contact:

[01-28]refer to the study codes in Table 4.1. *Total number of studies citing this taxon #Total number of use-records

***Verbesina robinsonii* (Klatt) Fernald ex B.L.Rob. & Greenm. (Asteraceae)**

Spanish names:
Indigenous names:
Used by (1*): Zoque[03]
Used for (1#): Digestive (1)[03]
Cognates:
Language contact:

***Verbesina tetraptera* (Ortega) A.Gray (Asteraceae)**

Spanish names: Paletaria
Indigenous names:
Used by (1*): Nahua[26]
Used for (1#): Skin (1)[26]
Cognates:
Language contact:

***Verbesina turbacensis* Kunth (Asteraceae)**

Spanish names:
Indigenous names: Copanteñicuy[03]
Used by (1*): Zoque[03]
Used for (1#): Skin (1)[03]
Cognates:
Language contact:

***Vernonanthura deppeana* (Less.) H.Rob. (Asteraceae)**

Spanish names: Hoja de canela
Indigenous names: Ux cuy[03]; Suquinay[13]; Holobob te'[17]; Gui'xaan[21]
Used by (4*): Zoque[03]; Quichean Maya[13, 17]; Zapotec[21]
Used for (10#): Digestive (2)[13, 21]; Eye (1)[03]; Ear (1)[03]; Musculoskeletal (2)[13, 21]; Pregnancy (1)[21]; Female genital (2)[03, 13]; nd[17]
Cognates:
Language contact: Highland Popoluca <> K'iche' and Zap

***Vernonanthura patens* (Kunth) H.Rob. (Asteraceae)**

Spanish names:
Indigenous names: Ti'tzin'[13]; Bik'tal sitit[20]
Used by (2*): Quichean Maya[13]; Western Maya[20]
Used for (2#): Digestive (1)[20]; Female genital (1)[13]
Cognates:
Language contact: K'iche' <> Tzeltalan

***Vernonanthura* sp. (Asteraceae)**

Spanish names: Suquinay
Indigenous names: Suquinay[14]
Used by (1*): Quichean Maya[14]
Used for (1#): General and Unspecified (1)[14]
Cognates:
Language contact:

***Vernonia* sp. (Asteraceae)**

Spanish names:
Indigenous names: Xihuatahua[05]; Suquinay, sucunay, ub'i te[19]
Used by (3*): Totonac[05]; Quichean Maya[14]; Western Maya[19]
Used for (3#): Digestive (1)[19]; Respiratory (1)[05]; General and Unspecified (1)[14]
Cognates:
Language contact:

***Veronica acinifolia* L. (Plantaginaceae)**

Spanish names: Trebolillo azul
Indigenous names: Lotz Q'eq'oj Aq'om[12]
Used by (1*): Quichean Maya[12]
Used for (9#): Blood (1)[12]; Digestive (1)[12]; Eye (1)[12]; Musculoskeletal (1)[12]; Neurological (1)[12]; Psychological (1)[12]; Respiratory (1)[12]; Skin (1)[12]; General and Unspecified (1)[12]
Cognates:
Language contact:

***Viburnum hartwegii* Benth. (Adoxaceae)**

Spanish names: Hoja verde
Indigenous names:
Used by (1*): Zoque[01]
Used for (1#): Digestive (1)[01]
Cognates:
Language contact:

***Viburnum* sp. (Adoxaceae)**

Spanish names:
Indigenous names:
Used by (1*): Quichean Maya[13]
Used for (1#): Skin (1)[13]
Cognates:
Language contact:

***Vicia villosa* Roth (Fabaceae)**

Spanish names: Visia Belluda
Indigenous names:
Used by (1*): Quichean Maya[12]
Used for (3#): Blood (1)[12]; Urological (1)[12]; General and Unspecified (1)[12]
Cognates:
Language contact:

***Vigna speciosa* (Kunth) Verdc. (Fabaceae)**

Spanish names: Flor de guajolote
Indigenous names:
Used by (1*): Zapotec[21]
Used for (1#): General and Unspecified (1)[21]
Cognates:
Language contact:

***Vigna unguiculata* (L.) Walp. (Fabaceae)**

Spanish names: Frijol torito, frijol tripa de tuza
Indigenous names: X'pal'lihua s'aca[06]
Used by (1*): Totonac[06]
Used for (1#): Skin (1)[06]
Cognates:
Language contact:

***Viguiera cordata* (Hook. & Arn.) D'Arcy (Asteraceae)**

Spanish names: Azaján
Indigenous names: Thapil bichim[07]
Used by (2*): Huastec[07]; Quichean Maya[12]
Used for (2#): Musculoskeletal (1)[12]; Skin (1)[07]
Cognates:
Language contact:

***Viguiera dentata*** **(Cav.) Spreng. (Asteraceae)**

Spanish names: Chimalacate
Indigenous names: Tah[10]; Qän Kewoj Achin[12]
Used by (3*): Yucatecan Maya[10]; Quichean Maya[12]; Nahua[27]
Used for (7#): Skin (2)[10, 27]; Urological (1)[12]; Pregnancy (1)[27]; Male genital (1)[12]; General and Unspecified (2)[10, 12]
Cognates:
Language contact:

***Vinca major*** **L. (Apocynaceae)**

Spanish names: Vincapervinca
Indigenous names:
Used by (1*): Quichean Maya[12]
Used for (3#): Cardiovascular (1)[12]; Urological (1)[12]; General and Unspecified (1)[12]
Cognates:
Language contact:

***Viola guatemalensis*** **W.Becker (Violaceae)**

Spanish names: Trebol real
Indigenous names: Trebo'l Aq'om[12]
Used by (1*): Quichean Maya[12]
Used for (7#): Blood (1)[12]; Digestive (1)[12]; Neurological (1)[12]; Psychological (1)[12]; Respiratory (1)[12]; Skin (1)[12]; General and Unspecified (1)[12]
Cognates:
Language contact:

***Virola guatemalensis*** **(Hemsl.) Warb. (Myristicaceae)**

Spanish names: Cedrillo
Indigenous names: J¥mniom cas[03]
Used by (1*): Zoque[03]
Used for (2#): Musculoskeletal (1)[03]; Skin (1)[03]
Cognates:
Language contact:

***Vismia baccifera*** **(L.) Planch. & Triana (Hypericaceae)**

Spanish names: Cayamita
Indigenous names: K'anparaquay che'[17]
Used by (2*): Zoque[03]; Quichean Maya[17]
Used for (5#): Digestive (1)[03]; Skin (1)[03]; Urological (1)[03]; Female genital (1)[03]; nd[17]
Cognates:
Language contact:

***Vismia camparaguey*** **Sprague & L.Riley (Hypericaceae)**

Spanish names:
Indigenous names: Q'an parwuay, Qan' paraway[14]
Used by (1*): Quichean Maya[14]
Used for (1#): Digestive (1)[14]
Cognates:
Language contact:

***Vismia*** **sp. (Hypericaceae)**

Spanish names: Huacalilla, lecherilla
Indigenous names:
Used by (1*): Nahua[26]
Used for (1#): Skin (1)[26]
Cognates:
Language contact:

[01-28]refer to the study codes in Table 4.1. *Total number of studies citing this taxon #Total number of use-records

***Vitex gaumeri* Greenm. (Lamiaceae)**

Spanish names:
Indigenous names: Ya'axnik[09]; YaŠnik[10]
Used by (2*): Yucatecan Maya[09, 10]
Used for (2#): Skin (1)[10]; General and Unspecified (1)[09]
Cognates: Yuca: yaxnik;
Language contact:

***Vitex mollis* Kunth (Lamiaceae)**

Spanish names: Beo; Ceresa negra; Coyotomate
Indigenous names: Jäk'yi'äye[02]; Boyajsa'a[21]; Cuahuilotl[28]
Used by (3*): Zoque[02]; Zapotec[21]; Nahua[28]
Used for (6#): Digestive (1)[02]; Musculoskeletal (1)[02]; Respiratory (3)[02, 21, 28]; Skin (1)[28]
Cognates:
Language contact:

***Vitis bourgaeana* Planch. (Vitaceae)**

Spanish names:
Indigenous names: Tu'sub' Kaham'[14]
Used by (1*): Quichean Maya[14]
Used for (1#): Pregnancy (1)[14]
Cognates:
Language contact:

***Vitis popenoei* J.L. Fennell (Vitaceae)**

Spanish names: Totoloche
Indigenous names: Mopst¥m[03]
Used by (1*): Zoque[03]
Used for (2#): Digestive (1)[03]; Eye (1)[03]
Cognates:
Language contact:

***Vitis* sp. (Vitaceae)**

Spanish names: Uva
Indigenous names: T'uthub[07]
Used by (2*): Huastec[07]; Quichean Maya[12]
Used for (7#): Eye (2)[07, 12]; Neurological (1)[07]; Endocrine (1)[12]; Pregnancy (1)[07]; General and Unspecified (2)[07, 12]
Cognates:
Language contact:

***Vitis tiliifolia* Humb. & Bonpl. ex Schult. (Vitaceae)**

Spanish names: Uva silvestre
Indigenous names: ku'untu/ku'untatz ay[01]; Po'on tzu'u[02]; Y¥k ty¥m tsay[03]; Snuun'gut[06]; Tusúp'[13]; Tu'sub' Kaham'[14]; T'u zub k'aham[17]; Chuyur ch'a'n[19]
Used by (8*): Zoque[01, 02, 03]; Totonac[06]; Quichean Maya[13, 14, 17]; Western Maya[19]
Used for (18#): Digestive (3)[02, 03, 13]; Eye (4)[01, 02, 03, 19]; Ear (1)[01]; Cardiovascular (1)[06]; Respiratory (1)[03]; Skin (1)[03]; Endocrine (1)[01]; Urological (1)[03]; Female genital (1)[03]; Male genital (2)[01, 14]; General and Unspecified (1)[01]; nd[17]
Cognates: Quich: tusup (kaham);
Language contact: Zoq <> Tot <> Quich

[01-28]refer to the study codes in Table 4.1. *Total number of studies citing this taxon #Total number of use-records

***Vittaria lineata* (L.) Sm. (Pteridaceae)**

Spanish names:
Indigenous names: Rubel xsa' kanti[14]
Used by (1*): Quichean Maya[14]
Used for (1#): Skin (1)[14]
Cognates:
Language contact:

***Volkameria ligustrina* Jacq. (Lamiaceae)**

Spanish names: Moste
Indigenous names: Muts[03]
Used by (1*): Zoque[03]
Used for (3#): Neurological (1)[03]; Psychological (1)[03]; General and Unspecified (1)[03]
Cognates:
Language contact:

***Waltheria glomerata* C. Persl (Malvaceae)**

Spanish names:
Indigenous names: Piniaka[03]
Used by (1*): Zoque[03]
Used for (1#): Urological (1)[03]
Cognates:
Language contact:

***Waltheria indica* L. (Malvaceae)**

Spanish names:
Indigenous names: Pun¥g ay[03]
Used by (1*): Zoque[03]
Used for (3#): Digestive (1)[03]; Psychological (1)[03]; Female genital (1)[03]
Cognates:
Language contact:

***Waltheria* sp. (Malvaceae)**

Spanish names: Malvarisco amarillo
Indigenous names: Alshoob nagatzi'[21]
Used by (1*): Zapotec[21]
Used for (3#): Digestive (1)[21]; Skin (1)[21]; Pregnancy (1)[21]
Cognates:
Language contact:

***Wedelia acapulcensis* Kunth (Asteraceae)**

Spanish names: Orozus
Indigenous names:
Used by (1*): Yucatecan Maya[10]
Used for (1#): Neurological (1)[10]
Cognates:
Language contact:

***Wedelia fertilis* McVaugh (Asteraceae)**

Spanish names:
Indigenous names: Sahun[09]
Used by (1*): Yucatecan Maya[09]
Used for (1#): Skin (1)[09]
Cognates:
Language contact:

***Wigandia urens* (Ruiz & Pav.) Kunth (Boraginaceae)**

Spanish names: Chichicaste, Chacom, Chocon; Chocón; Hoja de San Pablo
Indigenous names: Blàg-wê[23]
Used by (3*): Quichean Maya[12, 13]; Zapotec[23]
Used for (11#): Digestive (1)[13]; Cardiovascular (1)[12]; Neurological (1)[12]; Psychological (1)[12]; Respiratory (2)[13, 23]; Skin (2)[12, 23]; Pregnancy (1)[12]; General and Unspecified (2)[12, 23]
Cognates: Quich: chacom/chocon;
Language contact:

***Wimmeria bartlettii* Lundell (Celastraceae)**

Spanish names: Canserina
Indigenous names:
Used by (1*): Zoque[03]
Used for (3#): Digestive (1)[03]; Pregnancy (1)[03]; Female genital (1)[03]
Cognates:
Language contact:

***Wissadula amplissima* (L.) R. E. Fr. (Malvaceae)**

Spanish names:
Indigenous names: Puk ay[03]
Used by (1*): Zoque[03]
Used for (1#): General and Unspecified (1)[03]
Cognates:
Language contact:

***Wissadula excelsior* (Cav.) C. Persl (Malvaceae)**

Spanish names:
Indigenous names: J¥mniom cuy puk[03]
Used by (1*): Zoque[03]
Used for (1#): Skin (1)[03]
Cognates:
Language contact:

***Witheringia meiantha* (Don.Sm.) Hunz. (Solanaceae)**

Spanish names:
Indigenous names: Kunya[01]
Used by (1*): Zoque[01]
Used for (1#): Endocrine (1)[01]
Cognates:
Language contact:

***Witheringia solanacea* L'Hér (Solanaceae)**

Spanish names: Hoja de sapo
Indigenous names: Naj ka bokste/tziski boni rane[01]
Used by (1*): Zoque[01]
Used for (3#): Musculoskeletal (1)[01]; Skin (1)[01]; General and Unspecified (1)[01]
Cognates:
Language contact:

[01-28]refer to the study codes in Table 4.1. *Total number of studies citing this taxon #Total number of use-records

***Xanthosoma robustum* Schott (Araceae)**

Spanish names: Quequeste, malanga silvestre
Indigenous names: Poko[01]; Pix ay[03]; Tujk aay[04]; Paxni'cac[06]; Ts'ikiy luum[07]; Xtup'[13]; Marak [14]; Biu'ulú[21]
Used by (8*): Zoque[01, 03]; Mixe[04]; Totonac[06]; Huastec[07]; Quichean Maya[13, 14]; Zapotec[21]
Used for (15#): Blood (1)[06]; Musculoskeletal (2)[01, 21]; Respiratory (1)[01]; Skin (8)[01, 03, 04, 06, 07, 13, 14, 21]; Pregnancy (1)[13]; General and Unspecified (2)[14, 21]
Cognates:
Language contact: Chiapas Zoq <> Highland Popoluca <> Tot

***Xanthosoma sagittifolium* (L.) Schott (Araceae)**

Spanish names: Mafafa morada
Indigenous names: Paxni'cac[06]; Luum[07]
Used by (2*): Totonac[06]; Huastec[07]
Used for (3#): Skin (1)[07]; Pregnancy (1)[06]; General and Unspecified (1)[07]
Cognates:
Language contact:

***Ximenia americana* L. (Olacaceae)**

Spanish names:
Indigenous names: Paja pitx cuy[03]; Ma'ap ujts[04]; Nabche', Tsu'tsup[09]
Used by (3*): Zoque[03]; Mixe[04]; Yucatecan Maya[09]
Used for (4#): Digestive (2)[03, 09]; Female genital (1)[04]; General and Unspecified (1)[03]
Cognates:
Language contact: Mixe <> Yuc

***Xiphidium caeruleum* Aubl. (Haemodoraceae)**

Spanish names:
Indigenous names: X cua' li k'uch[17]
Used by (1*): Quichean Maya[17]
Used for (1#): nd[17]
Cognates:
Language contact:

***Xylosma flexuosa* (Kunth) Hemsl. (Salicaceae)**

Spanish names: Espina de brujo/espina de cruz
Indigenous names: Yatzi apit/cruz apit[01]; Jaman juix apitx[03]; Tsak k'iith, wi' chunun[07]; Puts'ukche', Xchaknif[09]
Used by (4*): Zoque[01, 03]; Huastec[07]; Yucatecan Maya[09]
Used for (10#): Digestive (1)[07]; Musculoskeletal (2)[01, 07]; Respiratory (1)[07]; Skin (2)[07, 09]; Female genital (1)[03]; General and Unspecified (3)[01, 03, 07]
Cognates: Zoq: apit; Mayan: tsak'ith/chaknit;
Language contact: Zoq <> Maya

***Xylosma panamensis* Turcz. (Salicaceae)**

Spanish names: Chatay
Indigenous names: Jaman juix apitx[03]
Used by (3*): Zoque[03]; Totonac[05, 06]
Used for (7#): Digestive (1)[06]; Respiratory (2)[05, 06]; Skin (1)[03]; Endocrine (1)[06]; Urological (1)[06]; General and Unspecified (1)[03]
Cognates:
Language contact:

[01-28] refer to the study codes in Table 4.1. *Total number of studies citing this taxon #Total number of use-records

***Yucca aloifolia*** **L. (Asparagaceae)**

Spanish names: Equizote, yucca
Indigenous names: A'ka'lokot[06]
Used by (1*): Totonac[06]
Used for (1#): Ear (1)[06]
Cognates:
Language contact:

***Yucca gigantea*** **Lem. (Asparagaceae)**

Spanish names: Izote
Indigenous names: Tsamnek k'oyol, chemnek k'oyol[07]; Tuk[09]; Oq'ki[13]; Sit'a'[19]
Used by (7*): Zoque[01, 03]; Huastec[07]; Yucatecan Maya[09]; Quichean Maya[12, 13]; Western Maya[19]
Used for (20#): Ear (4)[07, 12, 13, 19]; Cardiovascular (1)[01]; Musculoskeletal (1)[12]; Neurological (3)[12, 13, 19]; Psychological (1)[09]; Respiratory (3)[12, 13, 19]; Endocrine (2)[03, 12]; Pregnancy (1)[07]; Male genital (1)[12]; General and Unspecified (3)[07, 12, 19]
Cognates:
Language contact:

***Yucca*** **sp. (Asparagaceae)**

Spanish names:
Indigenous names: K'oyol[07]; Ikzote[26]
Used by (2*): Huastec[07]; Nahua[26]
Used for (4#): Ear (1)[26]; Skin (1)[26]; Pregnancy (1)[07]; General and Unspecified (1)[07]
Cognates:
Language contact:

***Zaluzania triloba*** **(Ortega) Pers. (Asteraceae)**

Spanish names: Hierba amarga
Indigenous names: Chichitzihuatl[25]
Used by (1*): Nahua[25]
Used for (2#): Digestive (1)[25]; Pregnancy (1)[25]
Cognates:
Language contact:

***Zamia loddigesii*** **Miq. (Zamiaceae)**

Spanish names: Mais Viejo
Indigenous names: Pekmuk kobak[03]; Møj mook[04]; Q'ixtab[14]
Used by (3*): Zoque[03]; Mixe[04]; Quichean Maya[14]
Used for (6#): Digestive (2)[03, 14]; Skin (1)[03]; Female genital (1)[03]; Male genital (1)[03]; General and Unspecified (1)[04]
Cognates: MZ: muk/mook;
Language contact:

***Zamia muricata*** **Willd. (Zamiaceae)**

Spanish names:
Indigenous names: Ox Q'en[14]
Used by (1*): Quichean Maya[14]
Used for (3#): Blood (1)[14]; Skin (1)[14]; General and Unspecified (1)[14]
Cognates:
Language contact:

***Zamia*** **sp. (Zamiaceae)**

Spanish names:
Indigenous names: J¥mniom pekmuk[03]; Tsalam thipaak, tsakam way', ahaatik a eem[07]
Used by (2*): Zoque[03]; Huastec[07]
Used for (6#): Digestive (1)[07]; Musculoskeletal (1)[07]; Endocrine (1)[07]; Urological (2)[03, 07]; General and Unspecified (1)[07]
Cognates:
Language contact:

***Zamia variegata* Warsz. (Zamiaceae)**

Spanish names:
Indigenous names: Sikad[17]
Used by (1*): Quichean Maya[17]
Used for (1#): nd[17]
Cognates:
Language contact:

***Zanthoxylum caribaeum* Lam. (Rutaceae)**

Spanish names: Mata de escorpión; Tachuelillo; Zorrillo cimarrón, zorrillo bravo, zorrillo de castilla, sasafrás de espina
Indigenous names: Apitx cuy[03]; Sinanche'[09]; Sina'an ché[11]; Tujen a'uch cimarrón[18]
Used by (4*): Zoque[03]; Yucatecan Maya[09, 11]; Western Maya[18]
Used for (12#): Digestive (1)[03]; Musculoskeletal (2)[11, 18]; Neurological (1)[09]; Respiratory (1)[03]; Skin (2)[03, 18]; Urological (1)[03]; Female genital (1)[03]; General and Unspecified (3)[09, 11, 18]
Cognates: Yuca: sinanche;
Language contact:

***Zanthoxylum fagara* (L.) Sarg. (Rutaceae)**

Spanish names:
Indigenous names: Wi'puuy[07]
Used by (1*): Huastec[07]
Used for (6#): Digestive (1)[07]; Musculoskeletal (1)[07]; Neurological (1)[07]; Respiratory (1)[07]; Pregnancy (1)[07]; General and Unspecified (1)[07]
Cognates:
Language contact:

***Zanthoxylum foliolosum* Donn.Sm. (Rutaceae)**

Spanish names:
Indigenous names: Leben sotz'[20]
Used by (1*): Western Maya[20]
Used for (1#): Digestive (1)[20]
Cognates:
Language contact:

***Zanthoxylum petenense* Lundell (Rutaceae)**

Spanish names:
Indigenous names:
Used by (1*): Quichean Maya[17]
Used for (1#): nd[17]
Cognates:
Language contact:

***Zanthoxylum* sp. (Rutaceae)**

Spanish names: Limoncillo
Indigenous names: Copixpix[03]
Used by (1*): Zoque[03]
Used for (1#): Blood (1)[03]
Cognates:
Language contact:

[01-28]refer to the study codes in Table 4.1. *Total number of studies citing this taxon #Total number of use-records

### *Zapoteca lambertiana* (G. Don) H. M. Hern. (Fabaceae)

Spanish names:
Indigenous names:
Used by (1*): Zoque[03]
Used for (1#): Skin (1)[03]
Cognates:
Language contact:

### *Zapoteca* sp. (Fabaceae)

Spanish names: Barba de chivo
Indigenous names: Tenzonhuaxi[26]
Used by (1*): Nahua[26]
Used for (1#): General and Unspecified (1)[26]
Cognates:
Language contact:

### *Zea mays* L. (Poaceae)

Spanish names: Maíz
Indigenous names: Mok owai/ japinjoke/ mojkuy su yaj/ tzapas tokok/ tzapas oksi/ tzapas äksi[01]; Mok/u'kä japo, äksi, mok angwa'y/mok puj[02]; Mok[03]; Mook, mook kuay, [04]; Cuxi[05]; Cuxi[06]; Eem, ithith[07]; Eem[08]; IŠim[10]; Ixim[12]; Ixim, jal, wi jal, uwijal, tzmi'y[13]; Ixim[18]; Ixim, nar[19]; Xhuba'a, bacuejlu'u moradu'u[21]; Žob[22]; Zhŏb, dòz, wgà, pcuêl[23]; Tlaoli[28]
Used by (20*): Zoque[01, 02, 03]; Mixe[04]; Totonac[05, 06]; Huastec[07, 08]; Yucatecan Maya[09, 10]; Quichean Maya[12, 13, 14]; Western Maya[18, 19]; Zapotec[21, 22, 23]; Nahua[25, 28]
Used for (71#): Digestive (13)[01, 02, 04, 05, 07, 08, 09, 12, 13, 14, 18, 21, 28]; Eye (1)[06]; Musculoskeletal (4)[01, 07, 08, 18]; Neurological (1)[12]; Psychological (1)[07]; Respiratory (4)[02, 07, 23, 28]; Skin (5)[01, 02, 03, 07, 21]; Endocrine (6)[01, 07, 08, 09, 12, 18]; Urological (17)[01, 02, 03, 04, 06, 07, 08, 09, 10, 12, 13, 18, 18, 19, 21, 25, 28]; Pregnancy (4)[01, 03, 22, 23]; Female genital (3)[02, 03, 10]; Male genital (3)[01, 12, 13]; General and Unspecified (9)[01, 02, 07, 08, 12, 13, 21, 22, 28]
Cognates: MZ: mok; Zoq: mok; Toto: cuxi; Mayan: iCi; Huas: eem; CoreM: ixim; Quich: ixim; WesM: ixim; Zapo: chob;
Language contact:

### *Zephyranthes brevipes* Standl. (Amaryllidaceae)

Spanish names: Cebolla de Zopilote
Indigenous names: Xnaket Q'os[12]
Used by (1*): Quichean Maya[12]
Used for (3#): Endocrine (1)[12]; Urological (1)[12]; General and Unspecified (1)[12]
Cognates:
Language contact:

### *Zephyranthes lindleyana* Herb. (Amaryllidaceae)

Spanish names: Brujita
Indigenous names: Ajtz'a 'taya[18]
Used by (1*): Western Maya[18]
Used for (1#): Ear (1)[18]
Cognates:
Language contact:

### *Zexmenia serrata* La Llave (Asteraceae)

Spanish names:
Indigenous names:
Used by (1*): Zoque[03]
Used for (1#): Skin (1)[03]
Cognates:
Language contact:

***Zingiber officinale* Roscoe (Zingiberaceae)**

Spanish names: Jengibre
Indigenous names: Kaxtxan ñiwi[03]; Caxta'lam pin[06]; Laab ist[07]; Xan Xir[14]; Xinxibeer[15]
Used by (11*): Zoque[01, 03]; Totonac[06]; Huastec[07]; Yucatecan Maya[09]; Quichean Maya[12, 13, 14, 15]; Western Maya[19]; Zapotec[21]
Used for (33#): Digestive (7)[01, 03, 06, 07, 09, 12, 14]; Musculoskeletal (5)[03, 06, 07, 13, 21]; Neurological (2)[06, 12]; Psychological (2)[06, 12]; Respiratory (4)[01, 07, 14, 21]; Skin (3)[03, 12, 21]; Endocrine (1)[01]; Pregnancy (2)[06, 19]; Female genital (4)[01, 03, 15, 19]; General and Unspecified (3)[12, 15, 21]
Cognates:
Language contact:

***Zingiber* sp. (Zingiberaceae)**

Spanish names: Ajenjible
Indigenous names: Chilcamotle[26]
Used by (1*): Nahua[26]
Used for (2#): Neurological (1)[26]; General and Unspecified (1)[26]
Cognates:
Language contact:

***Zinnia elegans* Jacq. (Asteraceae)**

Spanish names: Clavenia
Indigenous names:
Used by (1*): Zoque[03]
Used for (1#): Neurological (1)[03]
Cognates:
Language contact:

***Zinnia peruviana* (L.) L. (Asteraceae)**

Spanish names: Molinillo
Indigenous names:
Used by (1*): Quichean Maya[12]
Used for (1#): Psychological (1)[12]
Cognates:
Language contact:

***Ziziphus amole* (Sessé & Moc.) M.C. Johnst. (Rhamnaceae)**

Spanish names: Pendeno
Indigenous names:
Used by (1*): Zapotec[21]
Used for (1#): Female genital (1)[21]
Cognates:
Language contact:

***Zornia* sp. (Fabaceae)**

Spanish names:
Indigenous names: Tsaiñiay[03]
Used by (1*): Zoque[03]
Used for (1#): Skin (1)[03]
Cognates:
Language contact:

### *Zornia thymifolia* Kunth (Fabaceae)

Spanish names:
Indigenous names: Naax wiin aats[04]
Used by (1*): Mixe[04]
Used for (1#): Digestive (1)[04]
Cognates:
Language contact:

### *Zuelania guidonia* (Sw.) Britton & Millsp. (Salicaceae)

Spanish names:
Indigenous names: Bolantin, tatham te'[07]; Tamay, Bot'ox[09]
Used by (2*): Huastec[07]; Yucatecan Maya[09]
Used for (3#): Eye (1)[07]; Neurological (1)[07]; Female genital (1)[09]
Cognates:
Language contact:
