# Supplementary material for: Traditional Herbal Medicine in Mesoamerica: Toward Its Evidence Base for Improving Universal Health Coverage
Source: Front Pharmacol. 2020 Jul 31;11:1160. doi: 10.3389/fphar.2020.01160 (PMC7411306; doi:10.3389/fphar.2020.01160)
Supplement: Supplementary file 2 [file DataSheet_2.pdf]

Table A4.2.
Alphabetical list of the 995 botanical genera used medicinally in Mesoamerica. Abbreviations are as follows: Mixe-Zoque (MZ), Zoquean (Zoq), Totonac (Tot), pan-Mayan (Maya), Huastec (Hua), Core Mayan (CoreM), Yucatecan (Yuc), Quichean (Quich), Western Mayan (WesM), Zapotec (Zap), Nahuatl (Nah); a capital "C" stands for consonant and a capital "V" for vowel. A ">" B signifies that A is the probable donor and B the probable recipient, A "<>" B signifying that no conclusion can be drawn on the directionality of the language contact.

***Abelmoschus* (Malvaceae)**

Spanish names: Algalia; Arcalia, doña alpite, curalina; Boraja; Café chino; Curalina; Santa elena, semilla de vibora
Indigenous names: Almiz, almisy[01]; Tsa'ant ujts[04]; Kwinim ilaal[07]; K'inal, Xaq K'inal, Ruhi Xul[14]; Alkalia[20]
Used by (10*): Zoque[01, 02, 03]; Mixe[04]; Totonac[05]; Huastec[07]; Yucatecan Maya[09]; Quichean Maya[14]; Western Maya[19, 20]
Used for (20#): Digestive[01, 04, 07, 19, 20]; Musculoskeletal[03, 07]; Respiratory[03, 07]; Skin[01, 02, 04, 05, 09]; Endocrine[03]; Pregnancy[04]; General and Unspecified[01, 03, 07, 14]
Cognates:
Language contact:

***Abies* (Pinaceae)**

Spanish names:
Indigenous names: Yàg-Igâzh[23]
Used by (1*): Zapotec[23]
Used for (1#): General and Unspecified[23]
Cognates:
Language contact:

***Abrus* (Fabaceae)**

Spanish names:
Indigenous names: Oxo[09]
Used by (1*): Yucatecan Maya[09]
Used for (1#): Digestive[09]
Cognates:
Language contact:

***Abuta* (Menispermaceae)**

Spanish names:
Indigenous names:
Used by (2*): Zoque[03]; Quichean Maya[17]
Used for (2#): Female genital[03]; nd[17]
Cognates:
Language contact:

***Abutilon* (Malvaceae)**

Spanish names:
Indigenous names: Hupchil ts'ohool, tsakam akich, tsakam kwinim ts'ohool, thak akich, thak ch'ohool[07]; Sakmisbil, Sakpetmis[09]
Used by (2*): Huastec[07]; Yucatecan Maya[09]
Used for (2#): Digestive[07]; Skin[09]
Cognates:
Language contact:

Acacia (Fabaceae)

Spanish names: Alcornoque; Cacho de buey, cachito de toro; cacho de toro, cacho de buey, huizache; Cacho del toro; Cardo; Cornezuelo; Cornisuelo; Cornisuelo, cuerno; Espina de cacho, Cornisuelo; Espino blanco; Huisachi, espina blanca; Huizache; Jacarandillo blanco silvestre; Maroma; Timbre, guaje; Timbrillo blanco
Indigenous names: Mesit apij[01]; Juanapitx[03]; Juag aptx[04]; Tzut 'tzuu'n[06]; Xixit, waakal mo'eel, chak thuk'[07]; Thobem, thobem k'iith, theben, ist'am toro, toro k'iith[07]; Thuhanoom, thujanum, thuhanib[07]; Uthuth te[07]; Ch'imay[09]; Waxim[09]; Subinche'[09]; Seq' Jaqarant Aq'om[12]; Xe' subin ut xaq, Sub'in[14]; Ajsubin[18]; Sub'in[19]; Ixcanal[19]; Me' ja' te', xaxib, xaxib pukuj, vax te'[20]; Guedxebej[21]; Guish guiatzj[21]; Yàg-guièts-yàaz, yàg-guièts-dà, yàg-guièts-ngǒn[23]; Yàg-læ̀[23]; Yàg-guièts-clâv-nquǐts, yàg-guièts-clâv-làs[23]; Huechachin[28]
Used by (15*): Zoque[01, 03]; Mixe[04]; Totonac[06]; Huastec[07]; Yucatecan Maya[09]; Quichean Maya[12, 14]; Western Maya[18, 19, 20]; Zapotec[21, 23]; Nahua[26, 28]
Used for (68#): Digestive[03, 06, 07, 09, 12, 20, 23, 26, 28]; Eye[03, 20]; Ear[07]; Cardiovascular[07]; Musculoskeletal[01, 06, 07, 12, 26]; Neurological[03, 07, 19, 28]; Psychological[26]; Respiratory[03, 07, 19, 20, 21, 26]; Skin[04, 07, 09, 14, 20, 21]; Pregnancy[01, 03, 07, 19, 23]; Female genital[03, 12, 18, 19, 21, 26]; Male genital[12]; General and Unspecified[01, 03, 07, 19, 21, 23]
Cognates: MZ: JuaC apitx; Zoq: apitx; CoreM: sub'in; WesM: sub'in;
Language contact: MZ > Zapo; Tot <> Hua; Huas <> Yuc <> Tzeltalan

Acaena (Rosaceae)

Spanish names:
Indigenous names: Turumax[20]
Used by (1*): Western Maya[20]
Used for (1#): Digestive[20]
Cognates:
Language contact:

Acalypha (Euphorbiaceae)

Spanish names: Cola de gato; Contra araña; Corre chacalacca; Hierba de Cancer; Hierba de Cancer Hembra; Hierba de Cancer Macho; Hierba del cáncer; Hierba del cancer, hierba de mal de orín, hierba del gusano; Hierba del pastor; Tapón de burro
Indigenous names: Chi puk ay[03]; Ekchiñ poy cuy[03]; Tsuj mi ay[03]; Patsiit[04]; Čisšiši tuwan[05]; Tujuma'xanat[06]; Bohol ts'ohool, thuum ts'ohool, kakawate ts'ohool, xoko' ts'ohool, tsalam lat'em[07]; Tsak bohol ts'ohool, thuum ts'ohool tsakni'[07]; Xmisbil, Mehenmis[09]; Ch'ilibtux il[09]; Sak pasmar xiw[09]; Ru xup Top, Sa'l Q'os[12]; Znajnoy[13]; Kilik Q'ehen[14]; Xley Ipek[14]; Contra ajtoy[18]
Used by (13*): Zoque[02, 03]; Mixe[04]; Totonac[05, 06]; Huastec[07]; Yucatecan Maya[09, 11]; Quichean Maya[12, 13, 14]; Western Maya[18]; Nahua[27]
Used for (50#): Blood[12]; Digestive[03, 06, 07, 09, 12, 13]; Eye[03]; Musculoskeletal[12, 13]; Neurological[03, 11]; Respiratory[14]; Skin[02, 03, 04, 05, 06, 07, 09, 11, 12, 13, 18, 27]; Endocrine[03, 07, 09]; Urological[06, 11, 12, 13]; Pregnancy[14, 27]; Female genital[12, 14]; Male genital[12]; General and Unspecified[07, 12, 14, 27]
Cognates:
Language contact:

Acanthocereus (Cactaceae)

Spanish names: Pitaya
Indigenous names: Xak'ub, tzatza[07]
Used by (2*): Huastec[07]; Quichean Maya[12]
Used for (2#): Psychological[12]; Respiratory[07]
Cognates:
Language contact:

Achillea (Asteraceae)

Spanish names: Milenrama; Milenrama, achileo, milefolio, yerba de los carpinteros, ala de pollo; Plumajillo
Indigenous names: Milenrama[12]; Ushic ek, satul upa kaj, alhucema, sutul uxe kaj, sutil uwi kaj, uje kuk, tzilil up kaj, cumate q'aies, solb'al uwi kaj[13]
Used by (5*): Zoque[03]; Huastec[08]; Quichean Maya[12, 13]; Nahua[25]
Used for (22#): Digestive[08, 12, 13]; Ear[13, 25]; Cardiovascular[08, 12, 13]; Musculoskeletal[03, 12, 13]; Psychological[12]; Respiratory[13]; Skin[12, 13]; Endocrine[13]; Urological[12, 13]; Female genital[12, 13]; General and Unspecified[12, 13]
Cognates:
Language contact:

Achimenes **(Gesneriaceae)**

Spanish names:
Indigenous names: Baq'nel pim[14]
Used by (2*): Zoque[03]; Quichean Maya[14]
Used for (4#): Skin[03]; Pregnancy[03]; Female genital[03]; General and Unspecified[14]
Cognates:
Language contact:

Achyranthes **(Amaranthaceae)**

Spanish names:
Indigenous names: Ts'aah pathaam, lek'ab mitsu', tsakam pathaam akw'aal, itsik mitsu', itsaan an patham, akw'aalil pathaam, path ts'ohool[07]; Bayche'[09]
Used by (2*): Huastec[07]; Yucatecan Maya[09]
Used for (2#): Digestive[09]; Skin[07]
Cognates:
Language contact:

Acmella **(Asteraceae)**

Spanish names: Orozus
Indigenous names: Ñiwi sotyi[03]
Used by (2*): Zoque[03]; Yucatecan Maya[10]
Used for (2#): Neurological[03]; Respiratory[10]
Cognates:
Language contact:

Acnistus **(Solanaceae)**

Spanish names:
Indigenous names: K'apaj, Ka'a pajl[14]
Used by (1*): Quichean Maya[14]
Used for (2#): Digestive[14]; General and Unspecified[14]
Cognates:
Language contact:

Acosmium **(Fabaceae)**

Spanish names: Guayacán; Huayacan
Indigenous names: Putsy takin[01]; Tzus kuy[02]; Sam cuy[03]; Nøøtxk[04]; K'a che'[17]; Guassi[21]
Used by (8*): Zoque[01, 02, 03]; Mixe[04]; Yucatecan Maya[10]; Quichean Maya[17]; Zapotec[21]; Nahua[26]
Used for (28#): Blood[02, 03]; Digestive[01, 02, 03, 04, 10]; Musculoskeletal[03, 21]; Respiratory[01, 03, 04]; Skin[03]; Endocrine[01, 02, 03]; Urological[03]; Pregnancy[02, 03]; Female genital[03, 04, 21]; General and Unspecified[02, 03, 04, 21, 26]; nd[17]
Cognates:
Language contact:

Acourtia **(Asteraceae)**

Spanish names: Valeriana; Valeriana roja/de cerro
Indigenous names: Guìzh-zhĭil-dán[23]
Used by (3*): Quichean Maya[12]; Western Maya[19]; Zapotec[23]
Used for (8#): Musculoskeletal[19]; Neurological[19]; Psychological[12, 19]; Skin[23]; Pregnancy[19]; Female genital[19]; General and Unspecified[19]
Cognates:
Language contact:

Acrocomia (Arecaceae)

Spanish names: Cocoyol; Coyol; Coyole
Indigenous names: Kuma[02]; Cuma[03]; Kum[04]; Maap[07]; Biga'aj[21]
Used by (6*): Zoque[01, 02, 03]; Mixe[04]; Huastec[07]; Zapotec[21]
Used for (9#): Blood[03]; Digestive[02, 03, 04, 21]; Respiratory[07]; Endocrine[01]; Pregnancy[07]; General and Unspecified[21]
Cognates: MZ: kum; Zoq: kuma;
Language contact:

Adelobotrys (Melastomataceae)

Spanish names:
Indigenous names: Chunac kejen[16]; Chunahak k'ejen[17]
Used by (2*): Quichean Maya[16, 17]
Used for (2#): Neurological[16]; nd[17]
Cognates:
Language contact:

Adenophyllum (Asteraceae)

Spanish names: Cempasúchil del monte/ ruda del monte; Flor de calandria
Indigenous names: Guibiguadajnii, zamposuche guesxi[21]
Used by (2*): Zoque[02]; Zapotec[21]
Used for (9#): Digestive[02, 21]; Eye[21]; Ear[02]; Musculoskeletal[02]; Psychological[21]; Respiratory[21]; Skin[21]; General and Unspecified[21]
Cognates:
Language contact:

Adiantopsis (Pteridaceae)

Spanish names:
Indigenous names: Kaxam[07]
Used by (1*): Huastec[07]
Used for (1#): General and Unspecified[07]
Cognates:
Language contact:

Adiantum (Pteridaceae)

Spanish names: Cilantrillo; Colandrillo; Culantrillo; Culantrillo de pozo; Pata de Clarinero
Indigenous names: Tek piixi tek[04]; Akan pich, akan ts'ok, akan hooh, chalam ha'[07]; Akan pich, lapis ts'ohool[07]; Akan pich[07]; Culanto ch'o, coq' xip, uwi ja, uwi siwan, uklanto siwan[13]; Roq' Chi'wan[14]; Colantra pim[16]; Roq chit cuan[16]; Ruj i rak'aj tza[16]; Sis'bi pim[17]; Ru'j i rak' aj tza[17]; Ecahuile[24]
Used by (11*): Zoque[01, 03]; Mixe[04]; Huastec[07]; Quichean Maya[12, 13, 14, 16, 17]; Zapotec[21]; Nahua[24]
Used for (38#): Digestive[03, 12, 14]; Cardiovascular[07]; Neurological[01, 07, 14, 16]; Psychological[12, 16]; Respiratory[12, 13]; Skin[12]; Urological[01, 12, 21]; Pregnancy[07, 13, 24]; Female genital[03, 04, 12]; General and Unspecified[07, 12, 13, 16]; nd[17]
Cognates: Quich: (roq) siwan / chiwan / chitcuan;
Language contact: Mixe <> Hua

Aechmea (Bromeliaceae)

Spanish names: Ixtle; Kardon
Indigenous names: Ñiauing[03]; K'ok'om, k'ok'om wits, ok'lom te'[07]; Ch'uk, Cinta k'uk'[09]
Used by (3*): Zoque[03]; Huastec[07]; Yucatecan Maya[09]
Used for (11#): Digestive[03, 07]; Eye[07]; Ear[03]; Neurological[07]; Urological[03]; Pregnancy[03, 09]; Female genital[03]; General and Unspecified[07]
Cognates: Maya: k'ok/k'uk;
Language contact:

***Aegiphila* (Lamiaceae)**

Spanish names:
Indigenous names: M¥a ay[03]; Rok' xaa'n[17]
Used by (3*): Zoque[03]; Quichean Maya[16, 17]
Used for (4#): Musculoskeletal[03]; Neurological[16]; Female genital[03]; nd[17]
Cognates:
Language contact:

***Aeschynomene* (Fabaceae)**

Spanish names: Dormilona
Indigenous names: Salat-ik'[09]
Used by (2*): Zoque[01]; Yucatecan Maya[09]
Used for (2#): Skin[09]; General and Unspecified[01]
Cognates:
Language contact:

***Agarista* (Ericaceae)**

Spanish names:
Indigenous names: Unuk tyiñcuy[03]
Used by (1*): Zoque[03]
Used for (3#): Respiratory[03]; Skin[03]; Female genital[03]
Cognates:
Language contact:

***Agastache* (Lamiaceae)**

Spanish names: Toronjil
Indigenous names:
Used by (3*): Totonac[06]; Nahua[25, 26]
Used for (6#): Digestive[06]; Neurological[25]; Psychological[06]; Respiratory[26]; Female genital[06, 26]
Cognates:
Language contact:

***Agave* (Asparagaceae)**

Spanish names: Magueicillo; Maguey; Maguey del cerro
Indigenous names: O[01]; Xa'[04]; Weey, tsi'iim, thak book[07]; Sajkiy[12]; Sukchij[19]; Dobala'a[21]; Dohb[22]; Dòb-pcuêl, dòb-zân[23]
Used by (12*): Zoque[01, 03]; Mixe[04]; Huastec[07]; Yucatecan Maya[09]; Quichean Maya[12]; Western Maya[19]; Zapotec[21, 22, 23]; Nahua[25, 27]
Used for (31#): Digestive[12]; Cardiovascular[25]; Musculoskeletal[23, 25]; Neurological[25]; Psychological[12]; Respiratory[07, 21]; Skin[01, 03, 07, 12, 19, 21, 23, 25, 27]; Urological[12]; Pregnancy[04, 07, 09, 19, 22, 23, 25]; Female genital[19]; General and Unspecified[07, 12, 21, 22, 25]
Cognates: CoreM: sajkiy/sukchij; Zap: dob;
Language contact:

***Agdestis* (Phytolaccaceae)**

Spanish names: Suelda consuelda
Indigenous names: Yokya poj/ päksy[02]; Tuuwi', ix tuuwi', thak huchuk, thuyum[07]
Used by (2*): Zoque[02]; Huastec[07]
Used for (4#): Musculoskeletal[02, 07]; Skin[07]; General and Unspecified[07]
Cognates:
Language contact:

***Ageratina* (Asteraceae)**

Spanish names: Hierba de angerl; Yerba de culebra/ aigre
Indigenous names: K'anim mim[08]; Sakil ch'a te', xuch'il vomol[20]; Pom ch'a te', ox yoket vomol[20]; Škwam bäl[22]; Yàg-bdìin, guìzh-bdìin, guìzh-zhwèe[23]; Guìzh-rquiá-yàn[23]
Used by (4*): Huastec[08]; Western Maya[20]; Zapotec[22, 23]
Used for (18#): Digestive[20]; Eye[22]; Ear[20]; Musculoskeletal[22]; Neurological[08, 22]; Respiratory[20, 23]; Skin[20, 22]; Pregnancy[20]; General and Unspecified[20, 22, 23]
Cognates:
Language contact:

***Ageratum* (Asteraceae)**

Spanish names: Hierba santa cimarrón; Mejorana; Mejorana, pericón morado; Mostranzo
Indigenous names: Taam ujts[04]; Xpasmar xiw[09]; Ya'atzan Pamaj Q'os[12]; Lok' ab' winq'[14]; Xab'on Q'ehen[14]; Lok' ab' ixq[14]; Tlanecpaquelite[26]
Used by (7*): Mixe[04]; Yucatecan Maya[09]; Quichean Maya[12, 13, 14]; Nahua[25, 26]
Used for (22#): Digestive[04, 09, 12, 14]; Neurological[12]; Respiratory[12, 13, 14]; Skin[04, 12, 13, 25, 26]; Endocrine[12]; Female genital[14]; General and Unspecified[12, 14]
Cognates: CoreM: pa(s)ma;
Language contact:

***Agrimonia* (Rosaceae)**

Spanish names:
Indigenous names:
Used by (1*): Quichean Maya[13]
Used for (2#): Digestive[13]; Skin[13]
Cognates:
Language contact:

***Albizia* (Fabaceae)**

Spanish names:
Indigenous names: Thukiim[07]
Used by (1*): Huastec[07]
Used for (1#): General and Unspecified[07]
Cognates:
Language contact:

***Alcea* (Malvaceae)**

Spanish names:
Indigenous names: Guièe-màrpôl-nquǐts[23]
Used by (1*): Zapotec[23]
Used for (1#): Respiratory[23]
Cognates:
Language contact:

***Alchemilla* (Rosaceae)**

Spanish names:
Indigenous names:
Used by (1*): Quichean Maya[13]
Used for (1#): Digestive[13]
Cognates:
Language contact:

Alchornea (Euphorbiaceae)

Spanish names: Marangola, cacahuatillo
Indigenous names: Tuwax'katat, liilhpatat[06]
Used by (1*): Totonac[06]
Used for (2#): Endocrine[06]; General and Unspecified[06]
Cognates:
Language contact:

Aldama (Asteraceae)

Spanish names: Escutumbul
Indigenous names: K'an wits, mataablom[07]; Escutumbul[18]
Used by (2*): Huastec[07]; Western Maya[18]
Used for (3#): Respiratory[07]; Skin[07]; Endocrine[18]
Cognates:
Language contact:

Alibertia (Rubiaceae)

Spanish names: Catarita; Marimbola
Indigenous names: Wikpak[03]; Shbola bishulu'[21]
Used by (2*): Zoque[03]; Zapotec[21]
Used for (4#): Psychological[03]; Respiratory[03]; General and Unspecified[03, 21]
Cognates:
Language contact:

Allium (Amaryllidaceae)

Spanish names: Ajo; Cebolla; Cebollín
Indigenous names: Tzapaas ceullas/tsepolas[01]; Asyus[01]; Asyus[02]; Antuxk[04]; A'xux[06]; Akat'sasna[06]; Huun nakat, chuum nakat, xuun nakat[07]; Aaxux[07]; Thobollash dhoboyax[08]; Axu'x[13]; Ceboy'[13]; Axux[20]; Ceboy, cebul[21]; Aaxoj[21]; Žiht[22]; Nlěch, nsùch[23]
Used by (17*): Zoque[01, 02, 03]; Mixe[04]; Totonac[06]; Huastec[07, 08]; Yucatecan Maya[09]; Quichean Maya[12, 13, 14]; Western Maya[19, 20]; Zapotec[21, 22, 23]; Nahua[25]
Used for (114#): Digestive[01, 02, 03, 06, 07, 08, 12, 13, 19, 20, 21]; Eye[02, 21]; Ear[04, 06, 13]; Cardiovascular[01, 12, 13, 19]; Musculoskeletal[01, 02, 03, 06, 07, 08, 13, 14, 21, 25]; Neurological[01, 02, 08, 09, 13, 21]; Psychological[01, 03, 06, 19]; Respiratory[01, 02, 03, 06, 08, 12, 14, 19, 21]; Skin[01, 02, 03, 04, 07, 08, 13, 14, 19, 21, 25]; Endocrine[03, 08]; Urological[02, 03]; Pregnancy[03, 09, 13, 22]; Female genital[02, 09]; Male genital[02, 03]; General and Unspecified[01, 02, 04, 06, 07, 12, 13, 14, 19, 20, 21, 23]
Cognates:
Language contact:

Alloispermum (Asteraceae)

Spanish names: Flor de estrella
Indigenous names: Ma'atza jäyä[01]; K'oxox chij[20]
Used by (2*): Zoque[01]; Western Maya[20]
Used for (2#): Digestive[20]; General and Unspecified[01]
Cognates:
Language contact:

Allophylus (Sapindaceae)

Spanish names:
Indigenous names: Katxu aay[03]
Used by (2*): Zoque[03]; Yucatecan Maya[09]
Used for (2#): Skin[03, 09]
Cognates:
Language contact:

***Allosidastrum* (Malvaceae)**

Spanish names:
Indigenous names: T'unu' ix bek'em[07]
Used by (1*): Huastec[07]
Used for (1#): Pregnancy[07]
Cognates:
Language contact:

***Alnus* (Betulaceae)**

Spanish names: Aliso; Avedul; Avedul rojo
Indigenous names: Lama'[12]; Lemob'[13]; Elite, ilite[26]
Used by (3*): Quichean Maya[12, 13]; Nahua[26]
Used for (18#): Digestive[12]; Cardiovascular[12]; Musculoskeletal[12, 13]; Skin[12]; Urological[12]; Pregnancy[13]; Female genital[12]; Male genital[12]; General and Unspecified[12, 26]
Cognates: Quich: lama/lemob;
Language contact:

***Aloe* (Xanthorrhoeaceae)**

Spanish names: Sábila; Sávila; Zabila
Indigenous names: Xabila[07]; Petk'inki[09]; Pimki[13]; Tujtuj sukchij[19]; Dòb-xtîl[23]
Used by (17*): Zoque[01, 02, 03]; Mixe[04]; Huastec[07, 08]; Yucatecan Maya[09, 11]; Quichean Maya[12, 13, 14]; Western Maya[18, 19]; Zapotec[21, 23]; Nahua[25, 27]
Used for (89#): Blood[01, 02, 12, 18]; Digestive[01, 02, 03, 08, 12, 13, 18, 19, 21, 23, 25]; Eye[02, 27]; Cardiovascular[01, 02, 21]; Musculoskeletal[01, 02, 03, 08, 12, 13, 18, 19, 21, 27]; Neurological[08, 18, 19, 25]; Psychological[11, 12]; Respiratory[01, 02, 03, 04, 09, 13, 18, 21, 23, 25, 27]; Skin[01, 02, 03, 04, 07, 08, 09, 11, 12, 13, 14, 18, 19, 21, 23, 25, 27]; Endocrine[08, 13, 18, 25, 27]; Urological[01, 03, 12, 13, 18, 23]; Female genital[01, 03, 12]; Male genital[12]; General and Unspecified[01, 03, 08, 12, 13, 18, 19, 21, 23, 25]
Cognates:
Language contact:

***Alophia* (Iridaceae)**

Spanish names:
Indigenous names: Tsakam apats, tsak apats[07]
Used by (1*): Huastec[07]
Used for (1#): Digestive[07]
Cognates:
Language contact:

***Aloysia* (Verbenaceae)**

Spanish names: Cedron; Cedrón, Maria Luisa; Hierba luisa
Indigenous names: Bänälä luisa[18]
Used by (3*): Quichean Maya[12]; Western Maya[18]; Zapotec[22]
Used for (12#): Blood[12]; Digestive[12, 22]; Cardiovascular[12]; Neurological[12]; Psychological[12, 22]; Respiratory[12]; Skin[12]; General and Unspecified[12, 18, 22]
Cognates:
Language contact:

***Alternanthera* (Amaranthaceae)**

Spanish names: Golondrina
Indigenous names: Wiichab ts'ohool, wiichab maal, pulik wichab[07]; Tiyankix pepetal, ehtiil tsakam wichab[07]; Tan wamal[20]; Guìzh-guìdzdán, guìzh-yù, guìzh-guiĕt-nì , guìzh-zhàn̲-nì[23]; Tiangispepetl[26]
Used by (4*): Huastec[07]; Western Maya[20]; Zapotec[23]; Nahua[26]
Used for (13#): Digestive[07, 20, 23, 26]; Neurological[26]; Respiratory[26]; Skin[07, 26]; General and Unspecified[07, 23, 26]
Cognates:
Language contact:

***Alvaradoa* (Picramniaceae)**

Spanish names: Palo de hormigas; Plumajillo
Indigenous names: Belsinikche'[09]; Tzutzula[11]; Chi'karar te'[19]
Used by (3*): Yucatecan Maya[09, 11]; Western Maya[19]
Used for (4#): Skin[09, 11, 19]; General and Unspecified[11]
Cognates:
Language contact:

***Amaranthus* (Amaranthaceae)**

Spanish names: Bledo; Bledo, Amaranto; Espina; Espina de borrego; Pie de paloma roja; Quintonil; Quintonil espinoso
Indigenous names: Tu'kun calx'tunit[06]; Chith, lek'ab tooro[07]; Chithal tooro, he'pan, chital ist'amaal, waho ts'ohool, alte' chith[07]; Tez[13]; Nagucho'o[21]; Guedxe'e[21]; Guìzbæ̀[23]
Used by (9*): Zoque[01, 02, 03]; Totonac[06]; Huastec[07]; Quichean Maya[12, 13]; Zapotec[21, 23]
Used for (14#): Blood[13]; Digestive[01, 07, 23]; Cardiovascular[12]; Musculoskeletal[02]; Skin[07]; Urological[06]; Female genital[03]; General and Unspecified[01, 06, 07, 21]
Cognates:
Language contact:

***Ambrosia* (Asteraceae)**

Spanish names: Altamís; Artemis; Hierba del perro, altamisa, hierba de las señoras
Indigenous names:
Used by (4*): Mixe[04]; Western Maya[19, 20]; Nahua[26]
Used for (6#): Digestive[19, 20]; Musculoskeletal[04, 19]; Female genital[26]; General and Unspecified[19]
Cognates:
Language contact:

***Ampelocissus* (Vitaceae)**

Spanish names:
Indigenous names: Kamuuxy[04]
Used by (1*): Mixe[04]
Used for (1#): Eye[04]
Cognates:
Language contact:

***Amphilophium* (Bignoniaceae)**

Spanish names:
Indigenous names: Lalab uthu', ch'ichaabil uthu, t'uu ch'aah[07]; Sit'macho, Petak'[09]; Soski-ak', Xdunt'-ak'[09]; Xache'ma'ax, Xache'xnuuk[09]
Used by (2*): Huastec[07]; Yucatecan Maya[09]
Used for (5#): Digestive[09]; Skin[07, 09]
Cognates:
Language contact:

***Amphipterygium* (Anacardiaceae)**

Spanish names: Cuachalala; Cuachalalate; Cuachinala; Guachalalate
Indigenous names: Kuy tupsy[02]; Ya'guiaj[21]
Used by (5*): Zoque[02, 03]; Zapotec[21, 22]; Nahua[27]
Used for (20#): Blood[21]; Digestive[02, 21, 22, 27]; Cardiovascular[02]; Musculoskeletal[21]; Skin[02, 22, 27]; Endocrine[27]; Urological[27]; Pregnancy[02, 03]; Female genital[02, 03, 21]; General and Unspecified[02, 21, 27]
Cognates:
Language contact:

***Amphitecna* (Bignoniaceae)**

Spanish names: Jicara del duende
Indigenous names: Makti jeepe[03]; Cuacotecamatl, tecomate[26]
Used by (2*): Zoque[03]; Nahua[26]
Used for (3#): Psychological[03]; Respiratory[26]; Endocrine[26]
Cognates:
Language contact:

***Anacardium* (Anacardiaceae)**

Spanish names: Jocote de marañon, anacardo, alcyoiba; Marañon
Indigenous names: Q'inóm[13]
Used by (3*): Mixe[04]; Quichean Maya[13]; Western Maya[19]
Used for (3#): Digestive[13, 19]; Skin[04]
Cognates:
Language contact:

***Anagallis* (Primulaceae)**

Spanish names:
Indigenous names: Yax chel[20]; Ncuàan-dzéb-còrâl[23]; Nequixanitl[26]
Used by (3*): Western Maya[20]; Zapotec[23]; Nahua[26]
Used for (4#): Digestive[20]; Musculoskeletal[26]; Skin[26]; General and Unspecified[23]
Cognates:
Language contact:

***Ananas* (Bromeliaceae)**

Spanish names: Piña
Indigenous names: Tzik witz[01]; Uj'uj/tzikuy[02]; Uuju[03]; Aa'caa'xca[06]; Chabcham wits[07]
Used by (8*): Zoque[01, 02, 03]; Totonac[06]; Huastec[07]; Quichean Maya[12]; Western Maya[18]; Zapotec[21]
Used for (17#): Digestive[02, 06, 07, 12, 21]; Musculoskeletal[01]; Neurological[18]; Endocrine[06, 18]; Urological[01, 02, 06, 12]; Female genital[01, 02]; General and Unspecified[03, 21]
Cognates: Zoq: tzik/uju;
Language contact:

***Andira* (Fabaceae)**

Spanish names: Lombricero
Indigenous names: Ma'aka kuy[02]; Maka[03]; Tsukiñi cuy[03]; Iixy[04]; Ngaash[21]
Used by (4*): Zoque[02, 03]; Mixe[04]; Zapotec[21]
Used for (6#): Digestive[02, 21]; Skin[03]; General and Unspecified[04, 21]
Cognates: Zoq: maka;
Language contact:

***Anechites* (Apocynaceae)**

Spanish names: Lagartija
Indigenous names: Putzi ay[01]
Used by (1*): Zoque[01]
Used for (1#): Skin[01]
Cognates:
Language contact:

***Anemia* (Anemiaceae)**

Spanish names:
Indigenous names: Nø' ømo pøh[04]; Huniil akam pich, chalam ha'[07]; Boo'wat ts'ohool[07]
Used by (2*): Mixe[04]; Huastec[07]
Used for (3#): Female genital[04]; General and Unspecified[07]
Cognates:
Language contact:

***Anemopaegma* (Bignoniaceae)**

Spanish names:
Indigenous names: Guatimal[03]
Used by (1*): Zoque[03]
Used for (1#): Female genital[03]
Cognates:
Language contact:

***Anethum* (Apiaceae)**

Spanish names: Eneldito/ eneldo; Eneldo
Indigenous names: Neeld[21]; Guìzh-nêld[23]
Used by (8*): Zoque[01, 02, 03]; Mixe[04]; Quichean Maya[12, 13]; Zapotec[21, 23]
Used for (21#): Digestive[01, 02, 03, 04, 12, 13, 21]; Cardiovascular[12]; Musculoskeletal[02]; Neurological[12, 21]; Psychological[02, 12]; Respiratory[23]; Skin[21]; Pregnancy[03]; Female genital[02, 03, 13]; General and Unspecified[12, 21]
Cognates:
Language contact:

***Angelica* (Apiaceae)**

Spanish names: Angélica Macho/ hembra
Indigenous names: Tz'oloj B'aq Aqom[12]
Used by (1*): Quichean Maya[12]
Used for (5#): Digestive[12]; Cardiovascular[12]; Musculoskeletal[12]; Psychological[12]; General and Unspecified[12]
Cognates:
Language contact:

***Anisacanthus* (Acanthaceae)**

Spanish names:
Indigenous names: Gubedundh[22]
Used by (1*): Zapotec[22]
Used for (2#): Digestive[22]; General and Unspecified[22]
Cognates:
Language contact:

***Annona* (Annonaceae)**

Spanish names: Anona; Anona ; Anona (fría); Anona blanca; Candón; Caramuyo; Chirimoya; Condon, piñon anona; Guanabana; Ilama; Papause; Saramuyo
Indigenous names: (Pakak) yati[01]; Apak yati/katzu yati[01]; Yati[01]; Pasyte yati [02]; Yati[02]; Kopak yati[02]; Katx yatyi[03]; Kotsak yatyi[03]; Jon yatyi[03]; Guayaban aay[04]; Noajy aky[04]; Kilpu'jaka, a'xit kiwi[05]; Akčit kiwi[05]; A'cchit[06]; Aanchuuch, kukayil an chuch, ts'een kukay, ts'aale an chuch, bekal tsuts, tsakam kukay[07]; Kukay, tsak kukay[07]; Ahakte', kukay[07]; Ts'almuy[09]; Oop[09]; Oop Čuhum[10]; Pek' Aq'om[12]; K'uwex[13]; Holobob[17]; Tz'umuy[18]; Ajpox[18]; K'ewex[20]; Balagahuanabana'a[21]; Guele bajna'a[21]; Guelebidxu'u[21]
Used by (17*): Zoque[01, 02, 03]; Mixe[04]; Totonac[05, 06]; Huastec[07]; Yucatecan Maya[09, 10, 11]; Quichean Maya[12, 13, 17]; Western Maya[18, 20]; Zapotec[21]; Nahua[25]
Used for (104#): Blood[02]; Digestive[01, 02, 03, 04, 05, 06, 07, 09, 12, 13, 18, 20, 21, 25]; Cardiovascular[01, 02, 06]; Musculoskeletal[01, 02, 04, 11, 12, 13, 21]; Neurological[02, 11, 13]; Psychological[01, 02, 12, 21]; Respiratory[01, 02, 03, 07, 09, 12, 13]; Skin[02, 03, 05, 06, 07, 09, 10, 13, 21]; Endocrine[01, 02]; Urological[01, 02, 07, 12]; Pregnancy[02, 03]; Female genital[01, 03, 21]; Male genital[02]; Social problems[07]; General and Unspecified[01, 02, 03, 04, 07, 09, 10, 11, 12, 13, 18, 21]; nd[17]
Cognates: MZ: aCi; Zoq: yati; Tot: akchit; CoreM: k'Cwex; Yuc: oop;
Language contact: MZ > Tot <> Hua; Tot <> CoreM; Yuc > Kekchí

***Anoda* (Malvaceae)**

Spanish names: Halaches, flor morada, zoquilxochit; Malvavisca; Violea; Violeta; Violeta, violete silvestre
Indigenous names: Bakan ts'ohool, tsakam bok'ool ts'ohool[07]; Muu ts'ojo[08]; Guìzh-quês, xín-mâlb[23]
Used by (7*): Totonac[06]; Huastec[07, 08]; Quichean Maya[13]; Zapotec[23]; Nahua[25, 26]
Used for (19#): Digestive[07, 08, 25]; Neurological[07]; Respiratory[08, 26]; Skin[07, 08, 13, 23]; Endocrine[07]; Urological[06, 08, 25]; Pregnancy[06]; Female genital[06]; General and Unspecified[07, 08, 13]
Cognates:
Language contact:

***Anredera* (Basellaceae)**

Spanish names: Hoja de azar hembra; Iedra; Omisal; Suela con suela; Suelda consuelda; Suelda consuelda, camote que huele feo
Indigenous names: Tza'a tzoy[01]; Pak tyoopi[03]; Omisal[06]; Ix tuyuum, thuyum[07]; Kaxichel[09]; Gu'[21]; Gu'u icu'uj[21]
Used by (8*): Zoque[01, 03]; Totonac[06]; Huastec[07]; Yucatecan Maya[09, 11]; Quichean Maya[13]; Zapotec[21]
Used for (15#): Musculoskeletal[03, 06, 07, 09, 11, 13, 21]; Skin[07, 09, 11]; General and Unspecified[01, 07, 21]
Cognates:
Language contact:

***Anthemis* (Asteraceae)**

Spanish names:
Indigenous names: Manzaniya[07]
Used by (1*): Huastec[07]
Used for (1#): General and Unspecified[07]
Cognates:
Language contact:

***Anthurium* (Araceae)**

Spanish names: Cola de Pavo; Gañate de sope/ quebra piedras/ hoja de sope/ cabeza de guajolote; Hoja de piedra/Oreja de vaca/Riñonina/ lengua de vaca; Ojo de piedra, cola de faisan; Raiz de piedra
Indigenous names: Juki ay/ kanji kopak[01]; Tsa ay[03]; Tsa ay cinco hojas[03]; Kiñi ay[03]; Tsak ujts, tsanakøxp'[04]; Bobtun[09]; Xye Pu'[14]; Xtye pu'u[16]; Xchich maus[16]; X ch'cih ma'us[17]
Used by (9*): Zoque[01, 02, 03]; Mixe[04]; Yucatecan Maya[09, 11]; Quichean Maya[14, 16, 17]
Used for (26#): Digestive[01, 03, 04]; Cardiovascular[01, 14]; Musculoskeletal[03]; Neurological[11, 16]; Psychological[14, 16]; Skin[03]; Endocrine[01, 03]; Urological[01, 02, 03]; Pregnancy[02, 04]; Female genital[04, 09]; General and Unspecified[11, 14]; nd[17]
Cognates: MZ: tsa; Quich: xye pu/ xchich maus;
Language contact:

01-28 refer to the studies described in Table 4.1 * Total number of studies recording this taxon #Total number of use-records

***Antigonon* (Polygonaceae)**

Spanish names: Camote de vibora; San Pedro
Indigenous names: Rosario aats[04]; Ehtiil t'uthub[07]; Gulachiia[21]; Coacamalte, coamecatl[26]
Used by (5*): Mixe[04]; Huastec[07]; Yucatecan Maya[09]; Zapotec[21]; Nahua[26]
Used for (6#): Digestive[21]; Musculoskeletal[26]; Respiratory[09]; Skin[26]; Pregnancy[07]; Female genital[04]
Cognates:
Language contact:

***Antrophyum* (Pteridaceae)**

Spanish names: Costilla de Culebra
Indigenous names: Xcostill kanti, Xco'stii' Kanti[14]
Used by (1*): Quichean Maya[14]
Used for (1#): Skin[14]
Cognates:
Language contact:

***Apeiba* (Malvaceae)**

Spanish names: Papachote
Indigenous names: Pujki[03]
Used by (1*): Zoque[03]
Used for (3#): Digestive[03]; Skin[03]; Female genital[03]
Cognates:
Language contact:

***Aphelandra* (Acanthaceae)**

Spanish names: Santa María
Indigenous names: Mecha mooya[03]; Kiñi n¥pin mooya[03]; Muuw te'[07]; Qani holom chakmut[14]; Xpere` Aj tzo', Xalam Q'ehen, Xjolom Chaqmut, Xbool, Chamat, Mai Q'ehen[14]; Roq sosol[16]; Sa'x jolom, chacmut, sita pim[17]
Used by (5*): Zoque[03]; Huastec[07]; Quichean Maya[14, 16, 17]
Used for (16#): Cardiovascular[14]; Musculoskeletal[14]; Neurological[14, 16]; Psychological[16]; Skin[03, 14]; Urological[14]; Female genital[03, 14]; Male genital[14]; General and Unspecified[07, 14]; nd[17]
Cognates: Maya: mu; Quich: chakmut;
Language contact:

***Apium* (Apiaceae)**

Spanish names: Apio
Indigenous names:
Used by (4*): Zoque[01, 02]; Quichean Maya[12]; Nahua[25]
Used for (6#): Digestive[25]; Cardiovascular[02]; Musculoskeletal[01]; Psychological[12]; Respiratory[25]; Endocrine[12]
Cognates:
Language contact:

***Arachis* (Fabaceae)**

Spanish names: Cacahuate
Indigenous names: Kacwa[03]; Cacau[06]
Used by (2*): Zoque[03]; Totonac[06]
Used for (3#): Psychological[06]; Pregnancy[06]; Female genital[03]
Cognates:
Language contact:

Arachniodes (Dryopteridaceae)

Spanish names: Calahuala
Indigenous names:
Used by (1*): Quichean Maya[12]
Used for (4#): Blood[12]; Skin[12]; Urological[12]; General and Unspecified[12]
Cognates:
Language contact:

Arachnothryx (Rubiaceae)

Spanish names: Huele de noche
Indigenous names: Puchuch Re Tzuul[14]; Kandel Che[15]
Used by (4*): Zoque[03]; Quichean Maya[14, 15]; Zapotec[21]
Used for (8#): Neurological[14]; Psychological[15, 21]; Skin[03]; Pregnancy[15]; Female genital[03]; General and Unspecified[15, 21]
Cognates:
Language contact:

Arbutus (Ericaceae)

Spanish names: Madrón; Madroña
Indigenous names: Uq'a[13]; On te'[20]; Yàg-yǎn[23]
Used by (3*): Quichean Maya[13]; Western Maya[20]; Zapotec[23]
Used for (3#): Digestive[20]; Skin[13]; General and Unspecified[23]
Cognates:
Language contact:

Archibaccharis (Asteraceae)

Spanish names: Atlina
Indigenous names: Caxancapaxtli[28]
Used by (1*): Nahua[28]
Used for (1#): Pregnancy[28]
Cognates:
Language contact:

Arctostaphylos (Ericaceae)

Spanish names: Pingüica; Pingüita
Indigenous names: Yàg-blæ̀æ[23]
Used by (2*): Zapotec[21, 23]
Used for (6#): Digestive[21]; Cardiovascular[21]; Musculoskeletal[23]; Psychological[23]; Skin[23]; Urological[23]
Cognates:
Language contact:

Ardisia (Primulaceae)

Spanish names: Chagalapoli
Indigenous names: Tsuk nok nok[03]; Petx uk cuy[03]; Pelat puchun, wal puchun, pejte[07]; K'ak'al ilaal paktha'[07]; Xook'num[09]
Used by (3*): Zoque[03]; Huastec[07]; Yucatecan Maya[09]
Used for (10#): Digestive[03, 07]; Respiratory[03, 09]; Skin[03]; Female genital[03]; General and Unspecified[03, 07]
Cognates:
Language contact: Highland Popoluca <> Hua

Argemone **(Papaveraceae)**

Spanish names: Cardosanto; Carmesanta; Chacalote; Chicale; Chicalote; Chicalote, cardo santo; Chicalote, Espina blanca; Magueycito/huichache
Indigenous names: Ko tzitzi[02]; San Pedro Agats[04]; Tsolich, [07]; Sac qix, Sak'ak'ix[12]; Ixmucur[13]; Guedxe buloj[21]; Guièts-nîz, guìzh-guièts-nîz[23]; Chicalotl[28]
Used by (11*): Zoque[01, 02]; Mixe[04]; Huastec[07]; Yucatecan Maya[09]; Quichean Maya[12, 13]; Zapotec[21, 23]; Nahua[27, 28]
Used for (22#): Digestive[01, 23]; Eye[07, 12, 13, 23, 27, 28]; Neurological[12]; Respiratory[02, 09, 13, 28]; Skin[02, 21, 28]; Urological[02, 09]; General and Unspecified[04, 09, 12, 21]
Cognates:
Language contact:

Aristolochia **(Aristolochiaceae)**

Spanish names: Camotillo; Contrebo; Guaco; Guaco (fino); Guaco blanco; Guaco castillo; Guaco corriente; Guaco de arbol; Guaco de castilla; Guaco de cochino, Guaco amarillo; Hierba de Sope; Hítamo real; Jocobal, cocobá; Pato de Monte
Indigenous names: Tzay jäyä/nä yutza[01]; Paj m¥n[03]; Itsaan an ohob ilaal[07]; Ohob ilaal[07]; So'osol Q'ehen [14]; Xjolom Kamenaq[14]; Patz pim[14]; Patz pim[16]; Xa'ab maus[16]; Cocobá[18]; Ajwacu'[18]; Nojta wáko[19]; Ncuàan-dzéb-zhòmbrêl[23]
Used by (13*): Zoque[01, 02, 03]; Huastec[07]; Yucatecan Maya[09, 10]; Quichean Maya[14, 16]; Western Maya[18, 19]; Zapotec[21, 23]; Nahua[26]
Used for (95#): Digestive[01, 02, 03, 09, 10, 18, 19, 21, 23, 26]; Eye[03]; Cardiovascular[03, 21]; Musculoskeletal[01, 03, 07, 18, 21, 26]; Neurological[03, 14, 16, 18, 21, 26]; Psychological[03, 14, 16]; Respiratory[02, 03, 07, 18]; Skin[01, 02, 03, 14, 18, 21, 26]; Urological[02, 03]; Pregnancy[02, 03, 09, 21]; Female genital[01, 02, 03, 09, 18, 19]; General and Unspecified[01, 03, 14, 21, 23]
Cognates: Quich: patz pim;
Language contact:

Arnica **(Asteraceae)**

Spanish names: Arnica
Indigenous names: Maan huitz[08]
Used by (1*): Huastec[08]
Used for (5#): Digestive[08]; Musculoskeletal[08]; Respiratory[08]; Skin[08]; Endocrine[08]
Cognates:
Language contact:

Arracacia **(Apiaceae)**

Spanish names: Hierba del borrego; Valeriana, Chichipate
Indigenous names: Ts'ojolil an borrego[08]
Used by (2*): Huastec[08]; Quichean Maya[12]
Used for (6#): Cardiovascular[12]; Musculoskeletal[08]; Neurological[12]; Psychological[12]; Respiratory[08]; General and Unspecified[08]
Cognates:
Language contact:

Artemisia **(Asteraceae)**

Spanish names: Ajenjo; Ajenjo, alcanfor, ensencio, estafiate, incienso; Ajenjo, altamsa, artemisia amarga, encenso, ensenscio, té ruso, hierba santa; Altamisa, ajenjo; Artemisa; Artemisia; Estafiate; Estafiate blanco; Estafiate/estofiate; Hierba de incienso, esencia; Hierba de maestra, ajenjo; Hierba maestra; Hierba maestra, ajenjo; Incienso, Zi' zim; Yerba maestra
Indigenous names: Uksuk[01]; Poma ay[03]; Teen wits, teen ts'ohool[07]; Ten ts'ojol[08]; Tsakam ten huitz[08]; Si'isim[09]; Tsintsin[11]; Qa'yes, k'a q'ayes[13]; Sak sak, xak axaka, xaq' xaq'[13]; K'atab' Chaj[14]; Pom[18]; Guichuch roob[21]; Guìzh-maêstr[23]; Guìzh-guièe-rò[23]; Iztahuatl[26]; Iztauhyaitl[28]
Used by (21*): Zoque[01, 02, 03]; Mixe[04]; Totonac[05]; Huastec[07, 08]; Yucatecan Maya[09, 11]; Quichean Maya[12, 13, 14]; Western Maya[18, 19]; Zapotec[21, 22, 23]; Nahua[25, 26, 27, 28]
Used for (87#): Blood[12]; Digestive[01, 02, 03, 04, 05, 07, 08, 09, 11, 12, 13, 14, 18, 21, 22, 23, 25, 26, 27, 28]; Ear[07]; Cardiovascular[12, 14]; Musculoskeletal[01, 12, 18]; Neurological[08, 12, 23, 25]; Psychological[01, 05, 18, 19, 22]; Respiratory[01, 03, 12, 13, 14, 23]; Skin[03, 05, 07, 22, 28]; Endocrine[02, 12, 13, 22]; Pregnancy[03, 05, 13, 21, 27]; Female genital[01, 02, 03, 05, 12, 13]; General and Unspecified[01, 03, 05, 07, 12, 18, 19, 22, 25, 28]
Cognates: Hua: ten tsohol; Yuc: si'sim/tsintsin; Quich: katab' chaj / xakaxak; Zap: guizh ro; Nah: iztauhyatl;
Language contact:

***Arthrostemma* (Melastomataceae)**

Spanish names: Caña agria; Caña agria quadrata; Corona de Cristo; Xocoyuli
Indigenous names: Katzu aksa/ katzu tane/ katzu rane[01]; Katxu kanapoki[03]; X'cut'ni[06]; Rixiiji Tz'i, Xoy Q'ehen, Kaq'i pim[14]; Tzeleq' Ajsaq'[14]; Rok' za'ak[17]; Paj um'um wamal, pajal wamal, majk'al akan wamal, ik'al momol[20]; Nidaii[21]
Used by (7*): Zoque[01, 03]; Totonac[06]; Quichean Maya[14, 17]; Western Maya[20]; Zapotec[21]
Used for (28#): Digestive[01, 06, 14, 20, 21]; Eye[01]; Cardiovascular[01]; Musculoskeletal[01, 14]; Neurological[01, 14]; Psychological[01]; Respiratory[01, 20]; Skin[06, 14, 20]; Endocrine[03, 14]; Urological[01, 03, 14]; Male genital[14]; General and Unspecified[01, 14]; nd[17]
Cognates: Zoq: katzu anV; CoreM: ak; Quich: sak/kak;
Language contact:

***Artocarpus* (Moraceae)**

Spanish names: Castaña; Castaño
Indigenous names:
Used by (2*): Zoque[01]; Western Maya[18]
Used for (3#): Psychological[01]; Skin[01]; Female genital[18]
Cognates:
Language contact:

***Arundinella* (Poaceae)**

Spanish names: Cola de Caballo
Indigenous names: Xye' Kawaay[14]
Used by (1*): Quichean Maya[14]
Used for (2#): Endocrine[14]; General and Unspecified[14]
Cognates:
Language contact:

***Arundo* (Poaceae)**

Spanish names: Caña de Castillo; Carigo; Carizo; Carriz
Indigenous names: Kape[01]; Pakaab[07]
Used by (5*): Zoque[01]; Huastec[07]; Quichean Maya[12]; Nahua[24, 26]
Used for (8#): Musculoskeletal[01]; Neurological[01]; Psychological[01]; Skin[07, 26]; Pregnancy[24]; General and Unspecified[07, 12]
Cognates:
Language contact:

***Asclepias* (Apocynaceae)**

Spanish names: Chilillo; Flor de Mariposa; Jicaco; Marejilla; Mata caballo; Quebra muelas/ flor de serrillo; Quebramuela; Romero de monte
Indigenous names: Tusy put/witäm toya remedio /tusy kuy/ wenguy tätz [01]; Kiñi n¥pin mooya, Misi kuts¥k[03]; Tyuuxi[03]; Paxebaa[04]; Papuyut tawuan cajli, pinatawuan[05]; Pinatawan[05]; Punchiix wits[07]; Anal, Ik'abal, Polkuts[09]; Utu m'zat[13]; Ratzum' Pe'pem[14]; Guìzh-měy, guièe-měy, guìzh-yòob-lây, guìzh-lây, guièe-ziè[23]; Guìzh-cònêf[23]; Tacaxihuitl[26]; Oiloxtli[26]
Used by (12*): Zoque[01, 02, 03]; Mixe[04]; Totonac[05]; Huastec[07]; Yucatecan Maya[09]; Quichean Maya[13, 14]; Zapotec[23]; Nahua[26, 27]
Used for (38#): Digestive[01, 03, 04, 05, 07, 14, 27]; Cardiovascular[02, 05]; Musculoskeletal[01, 03, 23]; Neurological[01, 03, 05, 07, 09, 13, 23]; Skin[01, 02, 03, 05, 07, 13, 14, 26]; Endocrine[01]; Pregnancy[13, 27]; General and Unspecified[01, 05, 07, 27]
Cognates: Zoq: t(y)ux;
Language contact:

***Aspidosperma* (Apocynaceae)**

Spanish names: Malereo
Indigenous names: PiČmaŠ[10]
Used by (1*): Yucatecan Maya[10]
Used for (1#): Digestive[10]
Cognates:
Language contact:

***Asplenium* (Aspleniaceae)**

Spanish names:
Indigenous names: Xapachik ts'ohool, koo' ha', bakan ts'ohool, ehtiil weew koxol[07]
Used by (1*): Huastec[07]
Used for (3#): Cardiovascular[07]; Neurological[07]; Endocrine[07]
Cognates:
Language contact:

***Aster* (Asteraceae)**

Spanish names: Hierba del burro
Indigenous names: Jok poy tsus p¥k, Mok jipxi ay, Jok poy[03]
Used by (1*): Zoque[03]
Used for (3#): Digestive[03]; Skin[03]; Female genital[03]
Cognates:
Language contact:

***Astraea* (Euphorbiaceae)**

Spanish names:
Indigenous names:
Used by (1*): Huastec[07]
Used for (1#): Skin[07]
Cognates:
Language contact:

***Astrocaryum* (Arecaceae)**

Spanish names: Coquito
Indigenous names: Tzytzyon kuy täp[01]
Used by (2*): Zoque[01, 02]
Used for (2#): Musculoskeletal[02]; General and Unspecified[01]
Cognates:
Language contact:

***Astrolepis* (Pteridaceae)**

Spanish names:
Indigenous names: Guìzh-zhĭil-wlâgw, guìzh-wlâgw, yàg-zhĭil-wlâgw, guìzh-zhĭil-dán[23]; Ncuàan-dzéb-mæ̀cw[23]
Used by (1*): Zapotec[23]
Used for (2#): Skin[23]; General and Unspecified[23]
Cognates:
Language contact:

***Astronium* (Anacardiaceae)**

Spanish names: Gateado; Jobillo; Tulin
Indigenous names: Putoki[03]
Used by (4*): Zoque[01, 03]; Yucatecan Maya[09, 11]
Used for (10#): Digestive[01, 03]; Neurological[03]; Respiratory[03, 09]; Skin[03, 11]; Female genital[01]; General and Unspecified[01, 03]
Cognates:
Language contact:

Ateleia (Fabaceae)

Spanish names: Palo de pozole
Indigenous names: Jam kuy[02]; Ta'te', thak te', kax te'[07]
Used by (2*): Zoque[02]; Huastec[07]
Used for (2#): Musculoskeletal[02]; Skin[07]
Cognates:
Language contact:

Attalea (Arecaceae)

Spanish names: Corozo, coquito
Indigenous names: Misi'yu[18]
Used by (1*): Western Maya[18]
Used for (3#): Skin[18]; Endocrine[18]; General and Unspecified[18]
Cognates:
Language contact:

Augusta (Rubiaceae)

Spanish names:
Indigenous names: Pop n¥ang mooya[03]
Used by (1*): Zoque[03]
Used for (2#): Ear[03]; General and Unspecified[03]
Cognates:
Language contact:

Avena (Poaceae)

Spanish names: Avena
Indigenous names:
Used by (3*): Zoque[02]; Quichean Maya[12, 13]
Used for (5#): Blood[13]; Digestive[13]; Respiratory[13]; Endocrine[02]; General and Unspecified[12]
Cognates:
Language contact:

Averrhoa (Oxalidaceae)

Spanish names: Carambola
Indigenous names:
Used by (3*): Zoque[01, 02]; Western Maya[18]
Used for (3#): Digestive[02]; Urological[18]; Male genital[01]
Cognates:
Language contact:

Axonopus (Poaceae)

Spanish names: Grama
Indigenous names: K'im[13]
Used by (1*): Quichean Maya[13]
Used for (2#): Digestive[13]; Urological[13]
Cognates:
Language contact:

***Azadirachta* (Meliaceae)**

Spanish names: Nim; Nin
Indigenous names:
Used by (3*): Zoque[01, 02]; Huastec[08]
Used for (10#): Blood[02]; Cardiovascular[02, 08]; Neurological[02]; Skin[02]; Endocrine[01, 02, 08]; General and Unspecified[01, 08]
Cognates:
Language contact:

***Aztecaster* (Asteraceae)**

Spanish names: Romero del campo
Indigenous names:
Used by (1*): Zapotec[22]
Used for (1#): General and Unspecified[22]
Cognates:
Language contact:

***Baccharis* (Asteraceae)**

Spanish names: Arayan, raijan; Chamiso; Chamizo; Chamizo blanco; Chilca; Escoba, escobilla china; Lengua de pajarito; Sant Domingo, té de monte; Santo Domingo; Té de Monte
Indigenous names: Ok momon tsyawit/ ak manhgu tzyay/ ak mon tzyay[01]; Näng pojä[02]; Tsay mayorga[03]; Thintsiil, thi'al tsiib, ts'itsiimbe ts'ohool, ts'itsin ts'ohool, li'ax wits, akaanom ts'ohool, pamchaa ts'ohool[07]; Raxkej Q'os[12]; K'ichob', ixchop[13]; Uje k'ik', toj q'us, toj quas[13]; Bisik Q'aham Q'ehen, Re' saq' tiqobl[14]; Tzelek zaâk[16]; Vara xik', valak xik, saki xijch[20]; Vara xik', valak xik, vach' te' vomol, bak te'[20]; Mes te'[20]; Badzu'umij, guajgu'u[21]; Yak šeh[22]; Yàg-yàaz-nquĭts, yàg-yàaz-ròbáa[23]; Yàg-yàaz[23]; Yàg-yàaz-làs[23]
Used by (14*): Zoque[01, 02, 03]; Huastec[07]; Quichean Maya[12, 13, 14, 16, 17]; Western Maya[20]; Zapotec[21, 22, 23]; Nahua[26]
Used for (76#): Blood[07]; Digestive[12, 13, 20, 22, 23, 26]; Ear[20, 23]; Cardiovascular[12, 14]; Musculoskeletal[03, 07, 12, 13, 20, 22, 26]; Neurological[01, 07, 16, 20]; Psychological[12, 14]; Respiratory[12, 13, 14, 20, 23]; Skin[03, 07, 12, 13, 14, 20, 21, 26]; Endocrine[13]; Pregnancy[07, 12, 13, 21, 22]; Female genital[02, 13, 21]; General and Unspecified[01, 02, 03, 07, 12, 13, 14, 20, 21, 22, 23, 26]; nd[17]
Cognates: Zoq: tsay; Quich: iqob/ichob; sak; q'os/q'us;
Language contact: Chiapas Zoq <> Hua; Kekchí <> Tzeltalan

***Bacopa* (Plantaginaceae)**

Spanish names:
Indigenous names: Tsakam wiichab, wistiil a k'iichaa, ts'akat ti ichiich, chakam thutsub[07]
Used by (1*): Huastec[07]
Used for (3#): Psychological[07]; Skin[07]; General and Unspecified[07]
Cognates:
Language contact:

***Bactris* (Arecaceae)**

Spanish names: Jahuacte, chiquiyul
Indigenous names: Chäkyu'[18]
Used by (1*): Western Maya[18]
Used for (1#): Respiratory[18]
Cognates:
Language contact:

***Bambusa* (Poaceae)**

Spanish names:
Indigenous names: Masi caña[03]
Used by (1*): Zoque[03]
Used for (1#): General and Unspecified[03]
Cognates:
Language contact:

***Barkeria* (Orchidaceae)**

Spanish names: Candelaria
Indigenous names:
Used by (1*): Quichean Maya[12]
Used for (1#): Urological[12]
Cognates:
Language contact:

***Barkleyanthus* (Asteraceae)**

Spanish names: Chamizo amarillo
Indigenous names: Yàg-yàaz-nguěts[23]
Used by (1*): Zapotec[23]
Used for (2#): Neurological[23]; General and Unspecified[23]
Cognates:
Language contact:

***Bartlettina* (Asteraceae)**

Spanish names: Guaco
Indigenous names:
Used by (1*): Zoque[03]
Used for (1#): Digestive[03]
Cognates:
Language contact:

***Bauhinia* (Fabaceae)**

Spanish names: Casco de vaca; Mecate de Mico; Pata de cochi; Pata de vaca; Pativaca blanca; Pativaca roja
Indigenous names: Xitiks[03]; šmakan siyan toro, makačupi wacaš[05]; Tatil bichim[07]; Kibix, May vaca rojo, Ts'ulubtok'[09]; May vaca[09]; Xa'ab Wacax[14]; Xtab'imax[14]
Used by (7*): Zoque[01, 03]; Totonac[05]; Huastec[07]; Yucatecan Maya[09, 11]; Quichean Maya[14]
Used for (26#): Digestive[01, 03, 05, 07, 09]; Musculoskeletal[14]; Respiratory[03, 09]; Skin[03, 05, 07, 09]; Endocrine[03, 09, 14]; Urological[03, 07, 09]; Female genital[03]; General and Unspecified[03, 07, 11, 14]
Cognates:
Language contact:

***Bdallophytum* (Cytinaceae)**

Spanish names:
Indigenous names: Boo'wat wits, chumil a tsakah[07]
Used by (1*): Huastec[07]
Used for (1#): Neurological[07]
Cognates:
Language contact:

***Begonia* (Begoniaceae)**

Spanish names: Begonia del monte; Caña agria; Cañita agria; Chocoyul San Miguel del Angel; Hierba de purga; Mano de león, Caña agria; San Nicolas; Xocoyole fino/blanco; Xocoyul; Xocoyul blanco
Indigenous names: Leon k¥, Katxu kanapoki[03]; Kobakt¥k ay[03]; Nøtem ujts[04]; Xocovole[05]; X'cut'ni[06]; Bok'ool uxkwe', bakaanil a iits'[07]; Hiliyi ts'ohool, ts'amuxlaab ts'ohhol, tsakam ts'amuts k'animiim, huntal an pux lat'em[07]; Alte'begonia[08]; Kaq'i Paulul, Paulul Q'ehen[14]; Saq'i Pa'ulul[14]; Paulul Q'ehen[14]; Kak'i pim, pa' ulul[17]; Xak' peck, pa' ulul[17]; Pa' ulul[17]; Xoxoco[26]
Used by (12*): Zoque[01, 02, 03]; Mixe[04]; Totonac[05, 06]; Huastec[07, 08]; Quichean Maya[14, 17]; Zapotec[21]; Nahua[26]
Used for (51#): Digestive[01, 03, 05, 06, 08]; Eye[07]; Ear[03]; Cardiovascular[14]; Musculoskeletal[03, 08, 14, 21]; Neurological[02, 08]; Psychological[14]; Skin[01, 02, 03, 05, 06, 07, 14, 21]; Endocrine[07, 08]; Urological[03, 07, 14, 26]; Female genital[03, 04, 14]; Male genital[14]; General and Unspecified[07, 08, 14, 21]; nd[17]
Cognates: Quich: aki pa'ulul;
Language contact: Tot <> Nah

01-28 refer to the studies described in Table 4.1 * Total number of studies recording this taxon #Total number of use-records

***Bernardia* (Euphorbiaceae)**

Spanish names:
Indigenous names: Tsinat mahul, kwe' mahunal, liston te'[07]
Used by (1*): Huastec[07]
Used for (1#): Female genital[07]
Cognates:
Language contact:

***Beschorneria* (Asparagaceae)**

Spanish names:
Indigenous names: Tsakam tsi'iim[07]
Used by (1*): Huastec[07]
Used for (3#): Musculoskeletal[07]; Skin[07]; General and Unspecified[07]
Cognates:
Language contact:

***Besleria* (Gesneriaceae)**

Spanish names: Cabeza de Camarón
Indigenous names: Xjolom Ma'nzaan[14]; Ke' hal pim[17]
Used by (2*): Quichean Maya[14, 17]
Used for (2#): Skin[14]; nd[17]
Cognates:
Language contact:

***Beta* (Amaranthaceae)**

Spanish names: Acelga, remolacha; Betabel
Indigenous names:
Used by (3*): Zoque[01, 02]; Quichean Maya[12]
Used for (6#): Digestive[01]; Cardiovascular[12]; Psychological[12]; Skin[12]; General and Unspecified[02, 12]
Cognates:
Language contact:

***Bidens* (Asteraceae)**

Spanish names: Aceitilla; Amozo; Flor de muerto, , flor del alma; Mozote blanco, Aceitilla; Mozote planco; Mozote, acahuale blanco
Indigenous names: Anima jäyä [01]; Uentex ay[03]; X'tiyu snapapap[06]; Kelem ts'aah[07]; Kelem, kelem wits[07]; Ya'axk'an-ak'[09]; Sahun, Saksahun[09]; Job'on chokoj[11]; Xub'ay[14]; Tuix momol[20]; Guièe-tĭ-nquĭts[23]; Guièe-tĭ[23]; Mózotl[25]; Tzompiltecle[26]; Mozotl[26]
Used by (12*): Zoque[01, 03]; Totonac[05, 06]; Huastec[07]; Yucatecan Maya[09, 11]; Quichean Maya[14]; Western Maya[20]; Zapotec[23]; Nahua[25, 26]
Used for (37#): Digestive[05, 06, 07, 09, 20, 25]; Cardiovascular[05, 23]; Musculoskeletal[06, 14]; Neurological[03, 14]; Psychological[03, 14, 23]; Respiratory[07, 09, 25, 26]; Skin[01, 03, 07, 25]; Endocrine[25]; Urological[06, 26]; Pregnancy[26]; Female genital[03]; General and Unspecified[03, 06, 07, 09, 11, 14]
Cognates: Nah: mozotl;
Language contact:

***Bignonia* (Bignoniaceae)**

Spanish names: Bejuco de pimienta
Indigenous names: Moke poj[02]; Bichim ts'aah, pelat koox[07]; Punath, punal, ataa ch'aah[07]; Ek'k'ixil, Soski-ak'[09]; Puj Q'ehen[14]; Cajlmecatl[24]
Used by (5*): Zoque[02]; Huastec[07]; Yucatecan Maya[09]; Quichean Maya[14]; Nahua[24]
Used for (10#): Digestive[02, 07]; Musculoskeletal[07, 14]; Neurological[07, 14]; Skin[09]; Urological[07]; Pregnancy[24]; General and Unspecified[07]
Cognates:
Language contact:

***Biophytum* (Oxalidaceae)**

Spanish names: Palmita
Indigenous names: Coco ay, Chuch suyat[03]; Tek xuuxy ujts[04]; Tlalhuaxe[26]
Used by (3*): Zoque[03]; Mixe[04]; Nahua[26]
Used for (6#): Digestive[03, 26]; Psychological[03, 04]; Pregnancy[03]; General and Unspecified[03]
Cognates:
Language contact:

***Bixa* (Bixaceae)**

Spanish names: Achiote
Indigenous names: Tzine ay/Zini[01]; Pukä[02]; Cuy puk[03]; Axut[04]; Ki'wi', K'uxub[09]; KuŠu[10]; Q'axob[13]; Xayaw[14]; Xaq xa yaw[16]; Jo'ox[18]; Kiwi'[19]; Mbeye'e[21]
Used by (14*): Zoque[01, 02, 03]; Mixe[04]; Totonac[06]; Yucatecan Maya[09, 10]; Quichean Maya[12, 13, 14, 16]; Western Maya[18, 19]; Zapotec[21]
Used for (45#): Digestive[01, 09, 14, 18, 19, 21]; Ear[01]; Cardiovascular[01, 14]; Musculoskeletal[01, 18, 21]; Neurological[01, 03]; Psychological[16]; Respiratory[01, 10]; Skin[01, 02, 03, 04, 09, 10, 12, 13, 18, 21]; Endocrine[14]; Urological[01, 03]; Pregnancy[02, 03]; Female genital[03, 14, 21]; Male genital[01]; General and Unspecified[01, 02, 03, 04, 06, 09, 10, 18, 21]
Cognates: Zoq: puk; Yuc: kuxu; Quich: xayaw;
Language contact: Yuc <> K'iche' & Chortí

***Blakea* (Melastomataceae)**

Spanish names:
Indigenous names: Yot' eq[14]; Oxlaju' ch'ajom[17]
Used by (2*): Quichean Maya[14, 17]
Used for (2#): Digestive[14]; nd[17]
Cognates:
Language contact:

***Blechnum* (Blechnaceae)**

Spanish names:
Indigenous names: Tsabats chimal[03]
Used by (1*): Zoque[03]
Used for (2#): Pregnancy[03]; General and Unspecified[03]
Cognates:
Language contact:

***Blechum* (Acanthaceae)**

Spanish names: Cancerillo; Hueso de rana
Indigenous names: Yexu' tsaakuy elul, xonnol palats verde, san miguel wits, itsaan an tsabal muuw, bohol ch'ohool[07]; Ak'abxiw[09]
Used by (5*): Zoque[03]; Huastec[07]; Yucatecan Maya[09]; Quichean Maya[17]; Western Maya[18]
Used for (8#): Digestive[07, 09]; Skin[07, 18]; General and Unspecified[03, 07, 09]; nd[17]
Cognates:
Language contact:

***Blepharidium* (Rubiaceae)**

Spanish names: Girayol
Indigenous names: Yaxté[14]
Used by (1*): Quichean Maya[14]
Used for (2#): Skin[14]; General and Unspecified[14]
Cognates:
Language contact:

01-28 refer to the studies described in Table 4.1 * Total number of studies recording this taxon #Total number of use-records

***Blepharocalyx* (Myrtaceae)**

Spanish names: Anacahuite
Indigenous names: Hkshi vit[08]
Used by (1*): Huastec[08]
Used for (2#): Skin[08]; General and Unspecified[08]
Cognates:
Language contact:

***Bocconia* (Papaveraceae)**

Spanish names: Arnica; Gordolobo; Gordolobo (grande); Hoja de toro; Llora sangre; Llorasangre
Indigenous names: Susyui dane[01]; Ak¢i¢ileqs tuwan[05]; Tsîxte'[07]; Ts'ixte'[08]; Racan kaminaq'[12]; Xilcuahuitl[26]
Used by (9*): Zoque[01, 03]; Totonac[05]; Huastec[07, 08]; Quichean Maya[12]; Zapotec[23]; Nahua[25, 26]
Used for (22#): Digestive[26]; Cardiovascular[08]; Musculoskeletal[08, 23]; Neurological[07, 08, 12]; Psychological[12]; Respiratory[03, 05, 08, 26]; Skin[01, 03, 05, 23, 25, 26]; Endocrine[08]; Urological[08]; Female genital[26]; General and Unspecified[08]
Cognates: Hua: tsixte;
Language contact: Hua > Tot

***Boerhavia* (Nyctaginaceae)**

Spanish names: Hoja de azar embra; Vergonzosa, pega; Yerba de arretes
Indigenous names: Tza'a tzoy[01]; Chakle'[09]; Gurakw[22]
Used by (4*): Zoque[01]; Yucatecan Maya[09]; Zapotec[21, 22]
Used for (8#): Digestive[22]; Respiratory[21]; Skin[01, 09, 21]; General and Unspecified[01, 22]
Cognates:
Language contact:

***Bolbitis* (Dryopteridaceae)**

Spanish names:
Indigenous names: Quax kay pim[16]
Used by (2*): Quichean Maya[16, 17]
Used for (2#): Psychological[16]; nd[17]
Cognates:
Language contact:

***Bomarea* (Alstroemeriaceae)**

Spanish names: Campana; Lirio de Montaña
Indigenous names: San migeel wits, okoob pichich[07]; Aq'om Q'enqoj Aq'om, Rawen K'echelaj Aq'om[12]; Tzujtzuj Q'ehen[14]; Lo'loin[20]
Used by (5*): Zoque[03]; Huastec[07]; Quichean Maya[12, 14]; Western Maya[20]
Used for (12#): Digestive[07, 12, 14, 20]; Psychological[07]; Skin[07, 14]; Endocrine[07]; Urological[12]; Female genital[03]; General and Unspecified[14]
Cognates:
Language contact:

***Bonellia* (Primulaceae)**

Spanish names:
Indigenous names: Korpus wits, k'iith wich[07]; Sink'inche'[09]
Used by (2*): Huastec[07]; Yucatecan Maya[09]
Used for (3#): Neurological[07]; Respiratory[07]; General and Unspecified[09]
Cognates:
Language contact:

***Borago* (Boraginaceae)**

Spanish names: Borragia; Borraja; Borrajas
Indigenous names:
Used by (6*): Zoque[01]; Quichean Maya[12]; Zapotec[21, 23]; Nahua[25, 27]
Used for (12#): Cardiovascular[12]; Neurological[12]; Respiratory[01, 12, 21, 23, 25, 27]; Skin[23]; Pregnancy[12]; General and Unspecified[12, 23]
Cognates:
Language contact:

***Bougainvillea* (Nyctaginaceae)**

Spanish names: Baugmvilla; Bugambilia; Bugambilia morada; Buganbilia
Indigenous names: Apit jäyä[01]; Buganbiya[07]; Shpupukuishonat[08]; Yàg-bùgàmbîl[23]
Used by (16*): Zoque[01, 02, 03]; Mixe[04]; Huastec[07, 08]; Quichean Maya[12, 13]; Western Maya[18, 19]; Zapotec[21, 23]; Nahua[25, 26, 27, 28]
Used for (30#): Digestive[02, 25]; Cardiovascular[25]; Musculoskeletal[08]; Psychological[01, 27]; Respiratory[01, 02, 03, 04, 08, 12, 13, 18, 19, 21, 25, 26, 27, 28]; Skin[01]; Endocrine[08]; Urological[08]; Pregnancy[07]; General and Unspecified[08, 12, 21, 23, 25, 28]
Cognates:
Language contact:

***Bourreria* (Boraginaceae)**

Spanish names: Roble; Tres bono
Indigenous names: Oxib' boon'[14]
Used by (2*): Yucatecan Maya[11]; Quichean Maya[14]
Used for (3#): Psychological[11]; Endocrine[14]; General and Unspecified[11]
Cognates:
Language contact:

***Bouvardia* (Rubiaceae)**

Spanish names: Pericón rojo
Indigenous names:
Used by (1*): Quichean Maya[12]
Used for (5#): Blood[12]; Neurological[12]; Skin[12]; Endocrine[12]; General and Unspecified[12]
Cognates:
Language contact:

***Brachiaria* (Poaceae)**

Spanish names:
Indigenous names: Leh toom[07]
Used by (1*): Huastec[07]
Used for (1#): Skin[07]
Cognates:
Language contact:

***Brachistus* (Solanaceae)**

Spanish names: Tomatillo
Indigenous names: Xcoya che'[12]
Used by (1*): Quichean Maya[12]
Used for (2#): Digestive[12]; Endocrine[12]
Cognates:
Language contact:

***Brahea* (Arecaceae)**

Spanish names:
Indigenous names: Yàg-zîn[23]
Used by (1*): Zapotec[23]
Used for (1#): General and Unspecified[23]
Cognates:
Language contact:

***Brassica* (Brassicaceae)**

Spanish names: Mostaza; Mostaza ; Napu; Repollo
Indigenous names: Napx[12]; Pixla'q[12]; Ripoy[13]; Mostas gihš[22]; Mòxtâz[23]
Used by (9*): Zoque[01, 02]; Yucatecan Maya[11]; Quichean Maya[12, 13]; Western Maya[19]; Zapotec[21, 22, 23]
Used for (28#): Digestive[02, 12, 19, 21, 23]; Musculoskeletal[11, 22]; Neurological[11, 19, 22]; Respiratory[23]; Skin[01, 11, 12, 13]; Endocrine[02]; Urological[12]; Pregnancy[21]; Female genital[02]; General and Unspecified[02, 11, 12, 19, 21, 22, 23]
Cognates:
Language contact:

***Bravaisia* (Acanthaceae)**

Spanish names:
Indigenous names: Tz'ulub' Che'[14]
Used by (1*): Quichean Maya[14]
Used for (2#): Neurological[14]; Social problems[14]
Cognates:
Language contact:

***Brickellia* (Asteraceae)**

Spanish names: Hojita larga
Indigenous names: Ueji ay[03]; Komino ts'ohool, alte yexu' ts'ohool[07]; Ch'ail pox[20]; Guìzh-yòob-lây[23]
Used by (5*): Zoque[01, 03]; Huastec[07]; Western Maya[20]; Zapotec[23]
Used for (5#): Digestive[01, 20]; Psychological[03, 23]; General and Unspecified[07]
Cognates:
Language contact:

***Bromelia* (Bromeliaceae)**

Spanish names: Muta, piñuela; Pinuela
Indigenous names: Chicuix[03]; Patsikt[04]; Bathuch, bathuts[07]; Ch'om, Ch'am[09]; Tzetze'[19]
Used by (5*): Zoque[03]; Mixe[04]; Huastec[07]; Yucatecan Maya[09]; Western Maya[19]
Used for (7#): Ear[19]; Musculoskeletal[04, 07]; Skin[03, 07]; Pregnancy[09]; Female genital[09]
Cognates: MZ: chik/tsik;
Language contact:

***Brongniartia* (Fabaceae)**

Spanish names:
Indigenous names: Guìzh-càpâr[23]
Used by (1*): Zapotec[23]
Used for (1#): General and Unspecified[23]
Cognates:
Language contact:

***Bronwenia* (Malpighiaceae)**

Spanish names: Hierba florentina
Indigenous names:
Used by (1*): Zoque[01]
Used for (1#): Musculoskeletal[01]
Cognates:
Language contact:

***Brosimum* (Moraceae)**

Spanish names: Ramón
Indigenous names: Ohox, ojox, ojx[07]; Ox[09]; Viguiiru'u[21]
Used by (3*): Huastec[07]; Yucatecan Maya[09]; Zapotec[21]
Used for (3#): Neurological[07]; Respiratory[09]; General and Unspecified[21]
Cognates: Maya: ox;
Language contact:

***Brugmansia* (Solanaceae)**

Spanish names: Campana; Chanico; Flor de campana; Florifundia, flor de campana; Florifundio, flor de campana; Floripondio; Floripondio, flor de campana blanca; Hoja de campana, rosa
Indigenous names: Lokitsy/Kampentu/ kampantu jäya[01]; Nunak mooya[03]; Kalapuš[05]; Kampaana wits[07]; Sach campana, asak[13]; Tzinläwe[18]; Guièe-pûnt, yàg-guièe-pûnt[23]
Used by (11*): Zoque[01, 03]; Totonac[05]; Huastec[07]; Yucatecan Maya[10]; Quichean Maya[13]; Western Maya[18]; Zapotec[21, 23]; Nahua[25, 26]
Used for (33#): Digestive[01, 03, 13]; Ear[10]; Cardiovascular[01, 21]; Musculoskeletal[01, 03, 18, 21, 26]; Neurological[01, 13, 18]; Psychological[05, 13]; Respiratory[01, 21, 25]; Skin[01, 05, 07, 21, 25, 26]; General and Unspecified[01, 07, 21, 23]
Cognates:
Language contact:

***Bryophyllum* (Crassulaceae)**

Spanish names: Beladona; Beladona/sanalotodo; Belladona, sanalotodo, pericón; Hoja de aire; Maravilla; Palito pinto; Sanalotodo/Beladona/malva/hoja de paperón; Siempre viva; Siempreviva; Siempreviva, hoja fresca; Siempreviva, maravillosa, lengua de mujer
Indigenous names: Siwe tane[01]; Lakanyo/malva tane/ tini rane)/tok tzäksy/siwe tane/ tukun jäyä[01]; Tzikin kuy[01]; Tøts ujts[04]; Tkuya tuwan[05]; Tolow xekel, binage ts'ohool, lam ts'ohool, pahab eheenchx, pak'ak ts'ohool, xutsun buuru, pahab at'ax inik, pak'ak' xuts, toltha' ch'ohool[07]; Guish marauii, lu'ujtzanguana'aj[21]; Guìzh-cùchâr, rlăal̲-x-pǽæd-á, guièe-yùzh[23]; Campanaxochitl[26]
Used by (15*): Zoque[01, 02, 03]; Mixe[04]; Totonac[05]; Huastec[07]; Yucatecan Maya[09, 10, 11]; Quichean Maya[13, 16]; Western Maya[18]; Zapotec[21, 23]; Nahua[26]
Used for (54#): Digestive[01, 02, 03, 07, 18]; Eye[03, 05]; Ear[03]; Cardiovascular[07]; Musculoskeletal[01, 02, 03, 10, 11, 18, 21]; Neurological[01, 02, 03, 04, 05, 07, 16, 18, 26]; Psychological[07]; Respiratory[01, 03, 18]; Skin[01, 02, 03, 07, 09, 10, 11, 13, 21, 23, 26]; Endocrine[07]; Urological[21]; General and Unspecified[01, 03, 04, 07, 13, 21, 26]
Cognates:
Language contact:

***Buddleja* (Scrophulariaceae)**

Spanish names: Cacho de venado, salvia santa; Huacalillo; Lavatraste; Lengua de vaca; Salve real; Salvia santa, hoja blanca; Teposan
Indigenous names: Xiapun ay[03]; Pajuik[04]; Pulik elte', thak te' hunta a anaamte'[07]; Salv' sant'[13]; Saq rix pim[16]; T'oxpe'[19]; Sak yok te'[20]; Gush blaad[21]; Bala giwi[22]; Blàg-wì, lùdz-ngǒn, yàg-chànêcw[23]; Nesahuashihuitl[25]; Tepozan[26]; Zayoliscan[28]
Used by (13*): Zoque[03]; Mixe[04]; Huastec[07]; Quichean Maya[13, 16]; Western Maya[19, 20]; Zapotec[21, 22, 23]; Nahua[25, 26, 28]
Used for (39#): Digestive[04, 19, 20, 22, 23, 25, 28]; Musculoskeletal[19, 23]; Neurological[16, 19, 26]; Psychological[16, 19]; Respiratory[03, 19, 21]; Skin[03, 07, 13, 21, 25, 26, 28]; Pregnancy[13, 19, 21, 26]; Female genital[19]; General and Unspecified[03, 07, 13, 19, 21, 22, 23, 28]
Cognates: CoreM: sak; Nah: b(a)lag(i)wi;
Language contact:

Bunchosia (Malpighiaceae)

Spanish names:
Indigenous names: Sipche'[09]
Used by (1*): Yucatecan Maya[09]
Used for (2#): Digestive[09]; Neurological[09]
Cognates:
Language contact:

Bursera (Burseraceae)

Spanish names: Aceitillo; Azafrán; Chaca; Chacah blanco/ morado; Chaká; Copal; Copalero; Hoja de sumerio; Jiote, fiote, palo chino; Mulato; Mulato (rojo); Mulato negro; Mulato rojo; Palo de Hombre, Palo de Jiote; Palo de Jiote; Palo mulato; Sasafrás
Indigenous names: Tzaps kuy [01]; Äkyui/uwäk kuy/tzatz kuy/tzapas kuy [01]; Tzäk/tzäksy[02]; Bi Tzäksy/yäk tzäksy[02]; Putoki[03]; Ts¥k[03]; Tsøk[04]; Tusun, chaca, tusuni[05]; Tsaka, tsakah, chaka[07]; Kaxiy tsakah, tsamnek tsakah[07]; Tsaka[08]; Chakah[09]; Čacah[10]; Chi'kaj (blanco/ morado)[11]; Sali Che'[12]; Che' Winq, Kaq'aj[14]; Kak'hajl che'[17]; Chäk' zulte'[18]; Chakajr[19]; Yalajguettu'u[21]; Ii'guiaj[21]; Guish yaguiyaj'[21]; Yahl[22]; Yàg-yàal, yàg-guín-quiè[23]
Used by (21*): Zoque[01, 02, 03]; Mixe[04]; Totonac[05]; Huastec[07, 08]; Yucatecan Maya[09, 10, 11]; Quichean Maya[12, 14, 17]; Western Maya[18, 19]; Zapotec[21, 22, 23]; Nahua[25, 26, 27]
Used for (95#): Digestive[01, 02, 03, 05, 07, 14, 21, 26, 27]; Cardiovascular[03, 14]; Musculoskeletal[01, 03, 07, 08, 12, 14, 18, 21, 23]; Neurological[05, 07, 08, 10, 14, 22, 25]; Psychological[07]; Respiratory[07, 12, 21, 25]; Skin[01, 02, 03, 05, 07, 08, 10, 14, 21, 26, 27]; Endocrine[01, 02, 14]; Urological[02, 05, 12, 14, 18, 19]; Pregnancy[02, 21]; Female genital[01, 03, 21]; Male genital[01]; General and Unspecified[01, 02, 03, 04, 05, 07, 08, 09, 10, 11, 12, 18, 19, 21, 22, 23, 25, 26]; nd[17]
Cognates: MZ: tsäk; Zoq: tsäk; Maya: tsaka / chaka; Hua: tsaka; CoreM: chakaj; Yuc: chaka; Quich: kakaj; WesM: chäk / chak; Zap: yal;
Language contact: MZ > Maya

Buxus (Buxaceae)

Spanish names: Mirto Oloroso, Boj, Mirto, Lemonaria
Indigenous names:
Used by (1*): Quichean Maya[12]
Used for (3#): Neurological[12]; Respiratory[12]; General and Unspecified[12]
Cognates:
Language contact:

Byrsonima (Malpighiaceae)

Spanish names: Nance; Nance dulce; Nanche; Nanche/ nance; Nanchi; Tomatillo, tamaquilpa
Indigenous names: Nansin[01]; Nansin/ tupsy täm[02]; Nanchiñ[03]; Tax[04]; Chi'[09]; Či[10]; Tapal[13]; Chi'[14]; Chi'[18]; Chi'[19]; Nantzin, lantzin, nantz chi'[20]; Mbatsi[21]; Bälwi[22]; Yàg-ngùd-guèy-pcàal, yàg-ngùd-pcàal, guìzh-pcàal, ngùd-guèy-nguĕts[23]
Used by (17*): Zoque[01, 02, 03]; Mixe[04]; Totonac[06]; Yucatecan Maya[09, 10, 11]; Quichean Maya[12, 13, 14]; Western Maya[18, 19, 20]; Zapotec[21, 22, 23]
Used for (50#): Digestive[01, 02, 03, 04, 06, 09, 10, 13, 18, 19, 20, 21, 22]; Eye[02]; Cardiovascular[02]; Musculoskeletal[01]; Neurological[01, 02, 19]; Psychological[03]; Respiratory[02, 12, 20, 21]; Skin[01, 02, 03, 04, 09, 11, 20, 21]; Endocrine[02, 14]; Urological[01, 02, 03]; Pregnancy[02]; Female genital[01, 02, 03, 04, 21]; General and Unspecified[02, 09, 12, 13, 20, 21, 23]
Cognates: Zoq: nansin/nanchin; Maya: chi;
Language contact: Zoq <> Maya; Mixe <> K'iche'

Byttneria (Malvaceae)

Spanish names: Uña de gato (blanco)/cola de iguana/cola de garrobo
Indigenous names: Misyu apits/ tzätzän tuts/ nu'tzi tutz[01]; Bolool othow, bolool uthu'[07]
Used by (2*): Zoque[01]; Huastec[07]
Used for (8#): Digestive[01, 07]; Eye[01]; Cardiovascular[01]; Musculoskeletal[01]; Skin[01]; Pregnancy[07]; General and Unspecified[07]
Cognates:
Language contact:

01-28 refer to the studies described in Table 4.1 * Total number of studies recording this taxon #Total number of use-records

***Cabomba* (Cabombaceae)**

Spanish names:
Indigenous names: Weew bexe', hitil[07]
Used by (1*): Huastec[07]
Used for (2#): Neurological[07]; General and Unspecified[07]
Cognates:
Language contact:

***Caesalpinia* (Fabaceae)**

Spanish names: Hoja de maravilla; Ortensia; Sinquin colorado
Indigenous names: Tsas mooya[03]; Eelaa'[07]; San husee te', san husee wits[07]; Kitamche'[09]; Sin k'in[11]; So'sol Q'ehen, Kanteel Maus[14]
Used by (7*): Zoque[03]; Huastec[07]; Yucatecan Maya[09, 10, 11]; Quichean Maya[14]; Zapotec[21]
Used for (15#): Digestive[07, 21]; Musculoskeletal[03, 21]; Neurological[14, 21]; Psychological[07, 14]; Respiratory[03]; Skin[03]; Female genital[03]; General and Unspecified[09, 10, 11, 14]
Cognates:
Language contact:

***Caladium* (Araceae)**

Spanish names:
Indigenous names: Tsabats txikx pixi[03]
Used by (1*): Zoque[03]
Used for (1#): Skin[03]
Cognates:
Language contact:

***Calanthe* (Orchidaceae)**

Spanish names: Cebollin
Indigenous names: Cebollin Aq'om[12]
Used by (1*): Quichean Maya[12]
Used for (4#): Digestive[12]; Skin[12]; Urological[12]; General and Unspecified[12]
Cognates:
Language contact:

***Calathea* (Marantaceae)**

Spanish names: Hoja blanca
Indigenous names: Pop' ay/popo ay[01]; Pob ay[03]; Moxpim[14]; Kok' moch pim[14]; Koq mush[16]; Yoco to'[18]; Balagaquitzii[21]
Used by (6*): Zoque[01, 03]; Quichean Maya[14, 16]; Western Maya[18]; Zapotec[21]
Used for (17#): Digestive[01]; Eye[03]; Psychological[16]; Skin[01, 21]; Pregnancy[01, 14, 18, 21]; Female genital[03, 14, 18]; General and Unspecified[21]
Cognates: Zoq: pop ay ; Quich: kok moch/mux;
Language contact:

***Calea* (Asteraceae)**

Spanish names: Cacahuatón; Chinche; Hierba del perro/prodegiosa/ chinina/ lengua de borrego; Jaral; Mala hierba; Prodigiosa blanca; Prolijiosa/ prodigiosa (amarilla); Trabuco, hierba amarga
Indigenous names: Tuwi ay/ takak tane[01]; Añi mutx ay[03]; Tam juñi[03]; Tsapt taam ujts[04]; Poop taam ujts[04]; Šuna tuwan[05]; Tsuleek' ethem, betse' ts'ohool, pun lat'em, xamxam, weew ethem, tsabaal tok'te', ehek witsiim[07]; Pux lat'em[08]; Xka'xikin, Xikinkaax[09]; Ich wou[14]; Ton ch'a te'[20]; Sakil chi'xal wamal[20]; Škwam bižeh/ ghihb[22]; Ncuàan-zân[23]; Axcaxihuitl[26]
Used by (13*): Zoque[01, 03]; Mixe[04]; Totonac[05]; Huastec[07, 08]; Yucatecan Maya[09]; Quichean Maya[14]; Western Maya[20]; Zapotec[21, 22, 23]; Nahua[26]
Used for (39#): Digestive[01, 03, 04, 07, 08, 09, 20, 21, 22]; Cardiovascular[22]; Musculoskeletal[03, 22]; Psychological[22]; Respiratory[03, 07]; Skin[01, 03, 07, 09]; Endocrine[01]; Pregnancy[22, 23, 26]; Female genital[03]; General and Unspecified[01, 03, 04, 05, 07, 14]
Cognates: MZ: tam; Zoq: taC; Hua: puC lat'em;
Language contact:

***Calendula* (Asteraceae)**

Spanish names: Dormilón
Indigenous names: Guièe-dòrmìlôn, guièe-nguĕts, guièe-gùts, guièe-nàad[23]
Used by (1*): Zapotec[23]
Used for (1#): Respiratory[23]
Cognates:
Language contact:

***Calliandra* (Fabaceae)**

Spanish names: Barba del Rey; Palo sangre; Timbre
Indigenous names: Kine nhäpin [01]; Kinä näpin [02]; Juix mooya[03]; Wiir'oot', xixit[07]; Ch'ich' ni'[20]
Used by (7*): Zoque[01, 02, 03]; Huastec[07]; Quichean Maya[12]; Western Maya[20]; Nahua[26]
Used for (17#): Digestive[02, 12, 20]; Eye[02, 07]; Neurological[07]; Respiratory[01, 07, 20]; Skin[07, 20]; Urological[02]; Pregnancy[03, 26]; Female genital[03]; General and Unspecified[03, 07]
Cognates: Zoq: kine näpin; Maya: xix/chich;
Language contact: Maya > Highland Popoluca

***Callicarpa* (Lamiaceae)**

Spanish names:
Indigenous names: Elte', thal te'[07]; Xpuk'in, Puk'im[09]
Used by (2*): Huastec[07]; Yucatecan Maya[09]
Used for (4#): Digestive[07, 09]; Urological[07]; Pregnancy[07]
Cognates:
Language contact:

***Callisia* (Commelinaceae)**

Spanish names:
Indigenous names: Uixpin[03]; Pulik utek', xutsun buuru[07]; Utek' de bega[07]
Used by (2*): Zoque[03]; Huastec[07]
Used for (8#): Digestive[03]; Ear[07]; Cardiovascular[07]; Psychological[07]; Respiratory[03]; Urological[07]; Female genital[03]; General and Unspecified[07]
Cognates:
Language contact:

***Callistemon* (Myrtaceae)**

Spanish names: Enebro
Indigenous names:
Used by (1*): Quichean Maya[12]
Used for (2#): Urological[12]; General and Unspecified[12]
Cognates:
Language contact:

***Calophyllum* (Clusiaceae)**

Spanish names: Lechemaria; Varín
Indigenous names: Ka' kuy/ kang kuy[02]; M¥anakcuy[03]
Used by (2*): Zoque[02, 03]
Used for (5#): Musculoskeletal[02]; Neurological[03]; Skin[02, 03]; Female genital[03]
Cognates:
Language contact: Chimalapa Zoq <> Highland Popoluca

01-28 refer to the studies described in Table 4.1 * Total number of studies recording this taxon #Total number of use-records

***Calopogonium* (Fabaceae)**

Spanish names: Zempantle
Indigenous names: Btuujtza[21]
Used by (1*): Zapotec[21]
Used for (3#): Neurological[21]; Psychological[21]; General and Unspecified[21]
Cognates:
Language contact:

***Calyptocarpus* (Asteraceae)**

Spanish names: Cabeza de zompopo, espina de gallina; Hierba Amarilla, cachito; Huango/Hoja de azar
Indigenous names: Tza'a tzoy[01]; Xqa'ramaq'[12]
Used by (3*): Zoque[01]; Quichean Maya[12]; Western Maya[19]
Used for (5#): Digestive[12]; Neurological[12]; Psychological[12]; Pregnancy[19]; General and Unspecified[01]
Cognates:
Language contact:

***Calyptranthes* (Myrtaceae)**

Spanish names:
Indigenous names:
Used by (1*): Quichean Maya[17]
Used for (1#): nd[17]
Cognates:
Language contact:

***Camellia* (Theaceae)**

Spanish names: Té Verde
Indigenous names:
Used by (1*): Quichean Maya[12]
Used for (3#): Blood[12]; Cardiovascular[12]; Musculoskeletal[12]
Cognates:
Language contact:

***Campylocentrum* (Orchidaceae)**

Spanish names:
Indigenous names: Tz'ib'oyil Q'ehen[14]
Used by (1*): Quichean Maya[14]
Used for (1#): General and Unspecified[14]
Cognates:
Language contact:

***Campyloneurum* (Polypodiaceae)**

Spanish names: Cola de pavo; Helecho macho
Indigenous names: Cuy chimal[03]; wa'ub ik', wau, boo'wat, wayom ik, [07]; Rix i xul[17]
Used by (4*): Zoque[03]; Huastec[07]; Quichean Maya[15, 17]
Used for (7#): Digestive[03]; Ear[07]; Cardiovascular[07]; Musculoskeletal[15]; Neurological[07]; General and Unspecified[03]; nd[17]
Cognates:
Language contact:

***Canavalia* (Fabaceae)**

Spanish names: Sacramento
Indigenous names: Xagalamenta[03]; Koxol wits, koxoloow, pekeleexe wich[07]
Used by (2*): Zoque[03]; Huastec[07]
Used for (3#): Digestive[03]; Skin[03, 07]
Cognates:
Language contact:

***Canna* (Cannaceae)**

Spanish names: Cucuyus; Papatla; Papatla amarilla; Platanillo
Indigenous names: Nok ay[01]; ¥y¥ng ay[03]; Ciquichi, tzu'l kuat[05]; Chiquichi'[06]; K'uuwaap, tsak pik'o', tsabal wits, tabil[07]; Tsokon, maxan[13]
Used by (6*): Zoque[01, 03]; Totonac[05, 06]; Huastec[07]; Quichean Maya[13]
Used for (10#): Digestive[07]; Ear[03]; Musculoskeletal[07]; Neurological[07]; Skin[01]; Pregnancy[06, 07, 13]; General and Unspecified[01, 05]
Cognates: Tot: ciquichi;
Language contact:

***Cannabis* (Cannabaceae)**

Spanish names: Mariguana; Marihuana; Marijuana
Indigenous names: Jo'we ay' [02]; Ayuk juik[04]; Macutzín[28]
Used by (6*): Zoque[01, 02]; Mixe[04]; Zapotec[21, 22]; Nahua[28]
Used for (11#): Musculoskeletal[01, 02, 04, 21, 22, 28]; Neurological[02]; Urological[01]; General and Unspecified[01, 02, 21]
Cognates:
Language contact:

***Capraria* (Scrophulariaceae)**

Spanish names: Apazote de zorrillo, sabañon; Claviosa; Epazotillo; Esclaviosa; Pasmo, claviosa
Indigenous names: Kisawa ay[02]; Payau[04]; Sqeja lipajni[05]; Pulik ts'itsiimbe ts'ohool, weew bichim, tsamnek ts'ohool, [07]; Akan t'ot, elul ts'ohool[07]; Chokwil-xiw[09]; Pas mo Ši'iu[10]; Badxumij[21]
Used by (9*): Zoque[02]; Mixe[04]; Totonac[05]; Huastec[07]; Yucatecan Maya[09, 10, 11]; Western Maya[18]; Zapotec[21]
Used for (19#): Blood[10]; Digestive[07, 09, 21]; Ear[09]; Cardiovascular[07]; Musculoskeletal[05, 11]; Neurological[07]; Skin[02, 04, 18, 21]; Urological[11, 18]; Female genital[04, 07]; General and Unspecified[18, 21]
Cognates: Yuc: xiw/xi'iu;
Language contact:

***Capsella* (Brassicaceae)**

Spanish names: Bolsa de Pastor
Indigenous names:
Used by (1*): Quichean Maya[12]
Used for (4#): Skin[12]; Urological[12]; Female genital[12]; General and Unspecified[12]
Cognates:
Language contact:

***Capsicum* (Solanaceae)**

Spanish names: Chile; Chile ; Chile (menudo); Chile amashito; Chili; Chiltepe; Habanero
Indigenous names: (Nama) niwi[01]; Niwi[02]; Ñiwi[03]; Niiy[04]; Pi'n[06]; Its, ich[07]; Iik[10]; Iq', ik'[13]; Ik' Q'ehen, Xa'q Ik'[14]; Z'uts'ich[18]; Ich[20]; Balagaguina'a[21]; Tlanxinal[25]
Used by (16*): Zoque[01, 02, 03]; Mixe[04]; Totonac[06]; Huastec[07]; Yucatecan Maya[09, 10]; Quichean Maya[12, 13, 14]; Western Maya[18, 20]; Zapotec[21, 22]; Nahua[25]
Used for (35#): Digestive[12, 20]; Eye[06, 07, 10, 21]; Ear[02, 03]; Neurological[18]; Psychological[02, 04, 21]; Respiratory[25]; Skin[01, 02, 03, 06, 07, 12]; Endocrine[12]; Pregnancy[13]; Male genital[02, 03]; General and Unspecified[01, 04, 06, 07, 09, 12, 13, 14, 18, 21, 22]
Cognates: MZ: ni(w)i; Zoq: niwi; Maya: ich/ik; Quich: ik; WesM: ich;
Language contact: MZ <> Maya <> Tot <> Zap <> Nah

01-28 refer to the studies described in Table 4.1 * Total number of studies recording this taxon #Total number of use-records

Cardamine **(Brassicaceae)**

Spanish names: Hoja de azar del rio
Indigenous names: Tza'a tzoy[01]; T'ik'om ichiich[07]
Used by (2*): Zoque[01]; Huastec[07]
Used for (2#): General and Unspecified[01, 07]
Cognates:
Language contact:

Cardiospermum **(Sapindaceae)**

Spanish names: Hierba de pollo
Indigenous names:
Used by (1*): Nahua[28]
Used for (1#): Skin[28]
Cognates:
Language contact:

Carduus **(Asteraceae)**

Spanish names:
Indigenous names: Ohob ilaal[07]
Used by (1*): Huastec[07]
Used for (2#): Musculoskeletal[07]; Respiratory[07]
Cognates:
Language contact:

Carica **(Caricaceae)**

Spanish names: Papaya
Indigenous names: Otzo[01]; Tuna[02]; Xala'cua[06]; Utsun[07]; Put[09]; ČiČ pu'ut[10]; Tu' Q'ehen [14]
Used by (15*): Zoque[01, 02, 03]; Mixe[04]; Totonac[06]; Huastec[07]; Yucatecan Maya[09, 10, 11]; Quichean Maya[12, 13, 14]; Western Maya[18]; Zapotec[21]; Nahua[26]
Used for (27#): Digestive[01, 02, 03, 04, 06, 12, 13, 14, 18, 21]; Neurological[02, 03]; Psychological[02]; Respiratory[06]; Skin[03, 07, 09, 10, 11]; Endocrine[18]; Urological[02, 26]; Pregnancy[07]; Female genital[21]; General and Unspecified[03, 12, 21]
Cognates: Yuc: put;
Language contact: Chiapas Zoq > Hua

Cascabela **(Apocynaceae)**

Spanish names: calaverita; Chirrto; Cojon de perro; Coyol de cochi; Flor de San Juan
Indigenous names: Yoyak puj [01]; Ts'een aanchuuch, antsuts[07]; Akits[09]; Mbiigu'[21]; Mbiigu' guexii[21]; Yoyotli[28]
Used by (7*): Zoque[01, 02]; Huastec[07]; Yucatecan Maya[09, 11]; Zapotec[21]; Nahua[28]
Used for (16#): Digestive[21]; Cardiovascular[07]; Neurological[02, 07, 28]; Respiratory[11, 21, 28]; Skin[01, 02, 07, 09, 21]; General and Unspecified[21]
Cognates:
Language contact: Chiapas Zoq <> Nah; Hua <> Yuc

Casearia **(Salicaceae)**

Spanish names:
Indigenous names: ¥kx cuy, Tajuiñi ay[03]; Chumak chul[07]; Xi'mche'[09]
Used by (3*): Zoque[03]; Huastec[07]; Yucatecan Maya[09]
Used for (7#): Respiratory[07]; Skin[03, 07, 09]; General and Unspecified[09]
Cognates:
Language contact: Hua <> Yuc

***Casimiroa* (Rutaceae)**

Spanish names: Matasano; Zapote blanco
Indigenous names: Yuy, Sihun[09]; Ajachel[12]; Aja te'[20]; Yàg-ngùd-guèy[23]; Chipizapotl[28]
Used by (6*): Yucatecan Maya[09]; Quichean Maya[12]; Western Maya[20]; Zapotec[23]; Nahua[25, 28]
Used for (9#): Blood[12]; Digestive[09, 20]; Cardiovascular[23, 25]; Musculoskeletal[09]; Respiratory[09]; Pregnancy[28]; General and Unspecified[09]
Cognates: CoreM: aja;
Language contact:

***Cassia* (Fabaceae)**

Spanish names: Caña fista; Caña fistula ; Cañafistola; Cañafistula; Citiso Laburno
Indigenous names: Tzum kuy[02]; Tsapt tsina'an[04]; Bocut[10]; Q'en Q'echa Kineq' Aq'om[12]; Oj fistula[18]
Used by (9*): Zoque[01, 02, 03]; Mixe[04]; Yucatecan Maya[10]; Quichean Maya[12]; Western Maya[18, 19]; Zapotec[21]
Used for (29#): Blood[10, 12]; Digestive[01, 02, 04, 18]; Cardiovascular[12]; Musculoskeletal[02, 12]; Neurological[12]; Psychological[12]; Respiratory[01, 02, 03, 04, 18, 21]; Skin[10, 12]; Endocrine[12]; Urological[02, 12]; Pregnancy[02]; Female genital[02]; General and Unspecified[02, 03, 04, 19, 21]
Cognates:
Language contact:

***Castela* (Simaroubaceae)**

Spanish names: Chaparro amargoso; Venenillo
Indigenous names:
Used by (2*): Zoque[01]; Nahua[27]
Used for (4#): Digestive[01]; Cardiovascular[27]; Psychological[27]; Endocrine[27]
Cognates:
Language contact:

***Castilla* (Moraceae)**

Spanish names: Ule
Indigenous names: Peem[07]
Used by (2*): Huastec[07]; Yucatecan Maya[10]
Used for (4#): Musculoskeletal[07, 10]; Neurological[07]; Skin[07]
Cognates:
Language contact:

***Castilleja* (Orobanchaceae)**

Spanish names:
Indigenous names: Elul ts'ohool, ehtiil hut'ut wuts, k'ak'al wits, akan t'ele' del monte, pulek bohol ch'ohool[07]
Used by (2*): Zoque[03]; Huastec[07]
Used for (5#): Digestive[03, 07]; Neurological[07]; Pregnancy[07]; Female genital[07]
Cognates:
Language contact:

***Catasetum* (Orchidaceae)**

Spanish names:
Indigenous names: Ch'itku'uk[09]
Used by (1*): Yucatecan Maya[09]
Used for (2#): Musculoskeletal[09]; Skin[09]
Cognates:
Language contact:

Catharanthus (Apocynaceae)

Spanish names: Chatilla; Ninfa; Paraguaita; Paraguita; Vicaria
Indigenous names: Ninfax[03]
Used by (7*): Zoque[01, 02, 03]; Yucatecan Maya[09, 11]; Western Maya[18]; Zapotec[21]
Used for (18#): Digestive[02, 03, 21]; Eye[18]; Musculoskeletal[02]; Neurological[03]; Respiratory[18]; Skin[18, 21]; Endocrine[18]; Pregnancy[03, 11]; Female genital[02, 03, 09]; General and Unspecified[01, 18, 21]
Cognates:
Language contact:

Catopsis (Bromeliaceae)

Spanish names: Piñita
Indigenous names:
Used by (1*): Zoque[03]
Used for (1#): Female genital[03]
Cognates:
Language contact:

Cayaponia (Cucurbitaceae)

Spanish names: Hierba del soldado
Indigenous names: Wit po't[04]; Takeeyl[09]; Shuana'a soldadu[21]
Used by (3*): Mixe[04]; Yucatecan Maya[09]; Zapotec[21]
Used for (4#): Digestive[21]; Skin[04, 09]; General and Unspecified[21]
Cognates:
Language contact:

Ceanothus (Rhamnaceae)

Spanish names: Charín
Indigenous names: Tatanatzin[28]
Used by (2*): Quichean Maya[12]; Nahua[28]
Used for (3#): Digestive[12, 28]; Skin[28]
Cognates:
Language contact:

Cecropia (Urticaceae)

Spanish names: Chancarro; Guarrumbo; Guarumbo; Guarumbo, chancarro; Guarumo; Hormiguillo; Palo chiflón
Indigenous names: Ma'atz kuy/ma'atza /maatsy kuy[01]; Watz, boron kuy, syi wangpo', shy wonhko[02]; Mats[03]; Joot[04]; Akowa[05]; AA 'koo hua[06]; Tsulte'[07]; Xk'oochle'[09]; Aq'poo'jor, Po'jor[14]; Ha'ql[14]; Ajc'oloc'[18]; Chojb'[19]; Yagaba'aj[21]
Used by (16*): Zoque[01, 02, 03]; Mixe[04]; Totonac[05, 06]; Huastec[07]; Yucatecan Maya[09, 11]; Quichean Maya[14, 15]; Western Maya[18, 19]; Zapotec[21]; Nahua[25, 26]
Used for (60#): Digestive[01, 02, 03, 21, 25]; Ear[01, 03]; Cardiovascular[02, 18]; Musculoskeletal[01, 02, 04, 11, 21]; Psychological[01, 03, 15, 18]; Respiratory[01, 03, 21, 26]; Skin[01, 02, 03, 07, 11, 18, 21]; Endocrine[01, 02, 03, 04, 05, 06, 18, 21, 25, 26]; Urological[01, 02, 09, 26]; Pregnancy[01, 14, 15, 19]; Female genital[01, 02, 03, 21]; Male genital[02]; General and Unspecified[01, 02, 03, 04, 14, 21]
Cognates: Zoq: mats; Tot: akowa;
Language contact: Tot <> Tabasco Chontal & Kekchí & Yuc

Cedrela (Meliaceae)

Spanish names: Cedrillo, Cedro; Cedro; Cedro hembra
Indigenous names: A' kuy/ aj kuy[01]; Aja kuy/masya kuy[02]; Acuy[03]; Ajk[04]; Lištankiwi, pušnankiwi[05]; Ik'te'[07]; Nawal Che' Aq'om[12]; Rix Yaw Che'[14]; Ch'ujte[18]; Yagadoo[21]; Yàg-sîdr[23]
Used by (13*): Zoque[01, 02, 03]; Mixe[04]; Totonac[05]; Huastec[07]; Yucatecan Maya[09, 10]; Quichean Maya[12, 14]; Western Maya[18]; Zapotec[21, 23]
Used for (46#): Blood[01, 07, 12]; Digestive[01, 02, 03, 23]; Cardiovascular[12]; Musculoskeletal[01, 02, 03, 12, 21]; Neurological[01, 02, 07, 12]; Psychological[05, 07]; Respiratory[01, 07, 21]; Skin[01, 03, 07, 10, 12, 14, 21]; Endocrine[01, 02]; Urological[01, 03]; Pregnancy[03, 18]; Female genital[03, 09, 12]; General and Unspecified[01, 03, 04, 05, 07, 12, 18, 21]
Cognates: MZ: ajk; Zoq: a(j) kuy;
Language contact: MZ > Tot & Hua

01-28 refer to the studies described in Table 4.1 * Total number of studies recording this taxon #Total number of use-records

Ceiba (Malvaceae)

Spanish names: Ceiba; Pochote
Indigenous names: Pixtyiñ[03]; Pix ti'ink[04]; Unup[07]; Pi'im[09]; Ya'axche'[09]; Ch'ixirte[18]; Biuug[21]; Yaga shiene'e[21]
Used by (7*): Zoque[03]; Mixe[04]; Huastec[07]; Yucatecan Maya[09]; Western Maya[18]; Zapotec[21]; Nahua[27]
Used for (18#): Digestive[21, 27]; Eye[09]; Musculoskeletal[09]; Skin[03, 04, 07, 09, 21, 27]; Endocrine[18, 27]; Urological[27]; Pregnancy[21]; General and Unspecified[07, 21, 27]
Cognates: MZ: pixtin;
Language contact: MZ <> Yuc

Celosia (Amaranthaceae)

Spanish names: Mano de leon / Cresta de gallo; Moco de chunto
Indigenous names: Cundauajo[03]; Sam' ak'ach'[14]
Used by (2*): Zoque[03]; Quichean Maya[14]
Used for (2#): Blood[14]; Female genital[03]
Cognates:
Language contact:

Celtis (Cannabaceae)

Spanish names:
Indigenous names: Thak loh[07]
Used by (1*): Huastec[07]
Used for (2#): Skin[07]; General and Unspecified[07]
Cognates:
Language contact:

Cenchrus (Poaceae)

Spanish names:
Indigenous names: T'oyol k'iith, t'oyol toom[07]
Used by (1*): Huastec[07]
Used for (5#): Digestive[07]; Psychological[07]; Urological[07]; Pregnancy[07]; General and Unspecified[07]
Cognates:
Language contact:

Centratherum (Asteraceae)

Spanish names: Serpentina morada; Violeta de maceta
Indigenous names:
Used by (2*): Zoque[03]; Nahua[26]
Used for (3#): Respiratory[26]; Skin[26]; Pregnancy[03]
Cognates:
Language contact:

Centropogon (Campanulaceae)

Spanish names:
Indigenous names: Ayuni/putpa' tane[01]
Used by (1*): Zoque[01]
Used for (1#): Skin[01]
Cognates:
Language contact:

01-28 refer to the studies described in Table 4.1   * Total number of studies recording this taxon   #Total number of use-records

***Centrosema* (Fabaceae)**

Spanish names: Chureque
Indigenous names: Hik'elom ts'olool, lantha xeklek[07]; Buy-ak'[09]
Used by (4*): Zoque[03]; Huastec[07]; Yucatecan Maya[09]; Quichean Maya[12]
Used for (8#): Digestive[03, 12]; Eye[09]; Psychological[03]; Skin[09]; Endocrine[12]; General and Unspecified[07]
Cognates:
Language contact:

***Cerastium* (Caryophyllaceae)**

Spanish names: Hoja de azar
Indigenous names: Tza'a tzoy[01]
Used by (1*): Zoque[01]
Used for (1#): General and Unspecified[01]
Cognates:
Language contact:

***Ceratozamia* (Zamiaceae)**

Spanish names: Palmito
Indigenous names: Cuni[06]; Konlib[07]
Used by (2*): Totonac[06]; Huastec[07]
Used for (2#): Respiratory[06]; Urological[07]
Cognates:
Language contact: Tot <> Hua

***Cestrum* (Solanaceae)**

Spanish names: Boton zhiwite; Flor de goñon, huele de noche, jiquilete de monte, chilca de monte, sacate negro; Hierba de la Bilis, Hierba Morada; Hierba del torro; Hoja helionanda; Huele de noche; Huele noche; Juan de noche; Matabuey; Orcajuda; Orcajuda negra
Indigenous names: Wujpa ay[01]; Mok xoxay[03]; Tzisnutuwan, tzisni šanat[05]; Tsabalte'[07]; Ehek tsabalte', it'iib to'ol[07]; Tsabalte'[08]; Ejek tsabalte¿[08]; Q'enqoj K'ay Q'os Aq'om[12]; Choanum, quispar chuq'ies, xapakpan[13]; Xu Che'[14]; Rax ipaj, Xna ipaj[14]; Patonšiwit[22]; Yàg-guièe-zhǐn, bòtòn-xìwît[23]; Nixtamaxihuitl[25]; Ehepacxihuitl[26]
Used by (15*): Zoque[01, 02, 03]; Totonac[05]; Huastec[07, 08]; Yucatecan Maya[09]; Quichean Maya[12, 13, 14]; Zapotec[22, 23]; Nahua[25, 26, 28]
Used for (46#): Digestive[12, 13, 14, 22]; Musculoskeletal[08, 13]; Neurological[01, 07, 08, 22, 23, 25, 28]; Psychological[07]; Respiratory[07, 08, 13]; Skin[03, 07, 09, 25, 28]; Endocrine[02, 14]; Urological[07]; Pregnancy[03, 05, 07]; Female genital[03]; Male genital[14]; General and Unspecified[03, 05, 07, 08, 09, 12, 13, 14, 23, 25, 26]
Cognates: Hua: ehek tsabalte; Zap: pVtonxiwit; Nah: axihuitl;
Language contact:

***Chaetocalyx* (Fabaceae)**

Spanish names:
Indigenous names: M¥kstogay tsay[03]
Used by (1*): Zoque[03]
Used for (2#): Digestive[03]; Skin[03]
Cognates:
Language contact:

***Chamaecrista* (Fabaceae)**

Spanish names: Hoja de burbua; Hoja sen; Ortiga
Indigenous names: Kojotzik ay / kotzäk ay[02]; Copa uaxiñ[03]; Hitxi sotyi[03]; Sen ay[04]; Salatxiw, Salat-ik'[09]; Guixa'a sen[21]
Used by (5*): Zoque[02, 03]; Mixe[04]; Yucatecan Maya[09]; Zapotec[21]
Used for (19#): Digestive[02, 03, 21]; Eye[03, 09]; Skin[03, 21]; Urological[03]; Pregnancy[02, 21]; Female genital[02, 03, 04, 21]; General and Unspecified[04, 21]
Cognates:
Language contact:

01-28 refer to the studies described in Table 4.1 * Total number of studies recording this taxon #Total number of use-records

***Chamaedorea* (Arecaceae)**

Spanish names: Chiquilote; Pacaya; Tepejilote; Tepejilote, tepexilot
Indigenous names: Pampi[03]; Chikx pampi[03]; Liljtampan, litumpajna[05]; Weew bat'aw, thokob santu[07]; Gue'etzu'u[21]; Tepechilote[26]
Used by (6*): Zoque[03]; Totonac[05]; Huastec[07]; Quichean Maya[12]; Zapotec[21]; Nahua[26]
Used for (18#): Blood[12]; Digestive[12, 21, 26]; Psychological[07]; Respiratory[03, 12]; Skin[07, 12, 21, 26]; Endocrine[12]; Pregnancy[12]; General and Unspecified[03, 05, 12, 21]
Cognates:
Language contact:

***Chaptalia* (Asteraceae)**

Spanish names: Diente de león; Hoja de dos colores; Lengua de chivo; Lengua de perro
Indigenous names: Tzyivu totz [01]; Ai'jäs poti[02]; Jeepe ay, Nuup kobak[03]; Yo'opky piig ujts[04]; Tsakam yehtsel, ehtiil i k'ubak kw'a, alte' lopeen[07]; Rujiraq'i Tz'i'[14]; Guish lay león[21]
Used by (7*): Zoque[01, 02, 03]; Mixe[04]; Huastec[07]; Quichean Maya[14]; Zapotec[21]
Used for (16#): Digestive[03, 14, 21]; Eye[02, 07]; Musculoskeletal[01]; Neurological[04]; Psychological[03, 14]; Respiratory[14]; Skin[03, 04]; Urological[03, 14]; Pregnancy[03]; General and Unspecified[07]
Cognates: MZ: yo'op/jeepe/jäspo;
Language contact: MZ > Hua

***Cheilanthes* (Pteridaceae)**

Spanish names: Culandrillo
Indigenous names:
Used by (1*): Quichean Maya[13]
Used for (1#): Neurological[13]
Cognates:
Language contact:

***Chelonanthus* (Gentianaceae)**

Spanish names: Lengua de vaca
Indigenous names:
Used by (1*): Zoque[02]
Used for (1#): Skin[02]
Cognates:
Language contact:

***Chenopodium* (Amaranthaceae)**

Spanish names: Apacín; Apazote zorro; Bledo, Amaranto; Chagaquelite; Epazote de monte, epazote de zorrillo; Epazote de zorillo; Hierba de zorrillo; Yerba de guajilote
Indigenous names: Sa 'kalhka'jna'[06]; Lob'i tzetz[12]; Ka'i's par', par q'os[12]; Sik'aj par, uskaj par[13]; Škwam behd[22]; Guìzh-mèt, ptiè-mèt[23]
Used by (7*): Totonac[05, 06]; Quichean Maya[12, 13]; Zapotec[22, 23]; Nahua[27]
Used for (17#): Digestive[05, 06, 13, 23]; Cardiovascular[12]; Respiratory[12]; Skin[12, 22]; Urological[12]; Pregnancy[13]; Female genital[13, 23]; General and Unspecified[12, 13, 27]
Cognates: Quich: ka'i/kaj par; Zap: Cet;
Language contact: Tot <> Quich

***Chimaphila* (Ericaceae)**

Spanish names: Hierba del sapo
Indigenous names: Ka'che, p'jal q'ix[13]
Used by (1*): Quichean Maya[13]
Used for (1#): General and Unspecified[13]
Cognates:
Language contact:

01-28 refer to the studies described in Table 4.1 * Total number of studies recording this taxon #Total number of use-records

***Chiococca* (Rubiaceae)**

Spanish names: Moste cimarrón; Sorrillo; Tamagás
Indigenous names: Majei muts[03]; Puut' ts'aah, te' laab te'[07]; Chimes-kas, Xiax-al'[09]; Ix ak'ä jauay[11]; Pahar i pim[17]; Xín-bèch-mbăr[23]
Used by (7*): Zoque[03]; Huastec[07]; Yucatecan Maya[09, 11]; Quichean Maya[17]; Western Maya[19]; Zapotec[23]
Used for (17#): Digestive[03, 11, 19]; Musculoskeletal[07, 11]; Neurological[03]; Psychological[03]; Respiratory[03]; Skin[03, 09, 23]; Pregnancy[19]; Female genital[19]; General and Unspecified[03, 07, 11]; nd[17]
Cognates: Yuc: i(a)xa;
Language contact:

***Chionolaena* (Asteraceae)**

Spanish names: Gordolobo hembra
Indigenous names: Saq' Mak'el Q'os[12]
Used by (1*): Quichean Maya[12]
Used for (2#): Respiratory[12]; Skin[12]
Cognates:
Language contact:

***Chiranthodendron* (Malvaceae)**

Spanish names: Manita; Manita de león; Mano de león
Indigenous names: K'ubak k'wa'[08]; Q'anaq'[12]; Yàg-làz[23]
Used by (3*): Huastec[08]; Quichean Maya[12]; Zapotec[23]
Used for (8#): Digestive[08]; Cardiovascular[08, 12]; Musculoskeletal[08]; Neurological[08]; Psychological[12]; Respiratory[23]; Skin[08]
Cognates:
Language contact:

***Chloroleucon* (Fabaceae)**

Spanish names:
Indigenous names: Xiax-ek'[09]
Used by (1*): Yucatecan Maya[09]
Used for (1#): Skin[09]
Cognates:
Language contact:

***Chromolaena* (Asteraceae)**

Spanish names: Flor de gas, hoja petrolio; Prodigiosa, hoja de cruz; Venadillo
Indigenous names: Tam juñi[03]; Ehek witsiim, t'unu' witsiim[07]; Krus tok'te', tsamnek ts'ohool, thi'al t'eel[07]; Tok'aban[09]; Hatz[11]; Sakil sak ba te'[20]; Guish crush[21]; Guish petrol[21]
Used by (8*): Zoque[03]; Huastec[07]; Yucatecan Maya[09, 11]; Quichean Maya[17]; Western Maya[19, 20]; Zapotec[21]
Used for (17#): Digestive[03, 07, 19, 20, 21]; Musculoskeletal[11]; Neurological[07]; Psychological[07]; Respiratory[07]; Skin[07, 21]; Endocrine[09]; Urological[09]; General and Unspecified[07, 11]; nd[17]
Cognates:
Language contact:

***Chrysobalanus* (Chrysobalanaceae)**

Spanish names: Caco
Indigenous names:
Used by (1*): Zoque[02]
Used for (1#): Digestive[02]
Cognates:
Language contact:

Chrysophyllum (Sapotaceae)

Spanish names: Caimito silvestre
Indigenous names: Ajiya[03]; Chi'keeh[09]
Used by (2*): Zoque[03]; Yucatecan Maya[09]
Used for (4#): Digestive[03, 09]; Skin[03]; Female genital[03]
Cognates:
Language contact:

Chrysopogon (Poaceae)

Spanish names: Valeriana
Indigenous names:
Used by (1*): Zoque[01]
Used for (1#): Digestive[01]
Cognates:
Language contact:

Cichorium (Asteraceae)

Spanish names: Achicoria, Intibina
Indigenous names:
Used by (1*): Quichean Maya[12]
Used for (6#): Blood[12]; Digestive[12]; Skin[12]; Endocrine[12]; Urological[12]; General and Unspecified[12]
Cognates:
Language contact:

Cinchona (Rubiaceae)

Spanish names: Quina
Indigenous names: Saq'i Che' / Saq Paau[14]
Used by (1*): Quichean Maya[14]
Used for (4#): Digestive[14]; Endocrine[14]; Female genital[14]; General and Unspecified[14]
Cognates:
Language contact:

Cinnamomum (Lauraceae)

Spanish names: Canela; Rosa negra
Indigenous names: Contra moko[03]; Tsaayleel ohte'[07]
Used by (14*): Zoque[01, 02, 03]; Mixe[04]; Totonac[06]; Huastec[07]; Quichean Maya[12, 13, 14]; Western Maya[18, 19]; Zapotec[21]; Nahua[25, 26]
Used for (56#): Digestive[01, 02, 03, 04, 06, 12, 18, 19, 21]; Eye[02]; Cardiovascular[02]; Musculoskeletal[02, 12]; Neurological[12, 18]; Psychological[02, 12]; Respiratory[01, 02, 03, 06, 12, 13, 18, 19, 21, 25, 26]; Skin[03, 18, 25]; Endocrine[01]; Urological[01]; Pregnancy[01, 03, 06, 13, 14, 18, 19, 21, 25]; Female genital[02, 04, 14, 18, 19]; General and Unspecified[02, 03, 07, 12, 13, 14, 18, 21]
Cognates:
Language contact:

Cionosicyos (Cucurbitaceae)

Spanish names:
Indigenous names: Kasam[09]
Used by (1*): Yucatecan Maya[09]
Used for (1#): Skin[09]
Cognates:
Language contact:

***Cirsium* (Asteraceae)**

Spanish names: Alcachofa; Cardo santo; Cardosanto
Indigenous names: Awin tsäpä[01]; Cholich[07]; Sak' q'ix[12]; Kix' che', ukix che'[13]; Tepehuitzo[26]
Used by (7*): Zoque[01, 03]; Huastec[07]; Quichean Maya[12, 13]; Nahua[25, 26]
Used for (28#): Blood[12]; Digestive[03, 07, 12, 13]; Cardiovascular[12]; Musculoskeletal[07, 12, 13]; Neurological[12]; Psychological[12]; Respiratory[03, 07, 13]; Skin[01, 03]; Endocrine[12]; Urological[12, 25, 26]; Pregnancy[26]; Male genital[12]; General and Unspecified[12, 25]
Cognates: Maya: ich/ix; Quich: kix;
Language contact:

***Cissampelos* (Menispermaceae)**

Spanish names: Alcotán; Bejuco de ombligo; Bejuco zorrillo; Bejuquillo; Cintzo; Curalina ; Nonacal; Oreja de ratön; Redondillo
Indigenous names: Taka ime/ tzana ay[01]; Tyiñi woyo[03]; Poop axtaam pikx[04]; K'on k'ach, walik ts'ohool, ichiichbe ts'ohool, uxum ichich, xuts ts'ohool, bok'ool ch'ohool[07]; Peteltun[09]; Sison' u'u[14]; Ch'up i ai[17]; Ajbejcuo a'uch[18]; Chin ak' wamal, voy chij vomol, voy chij tz'i'lel, yaxal nixh vomol, tz'urupik' vomol, kurarina, pak chak, makmak chak', chin ak[20]; Cintzo[25]
Used by (14*): Zoque[01, 03]; Mixe[04]; Totonac[05]; Huastec[07]; Yucatecan Maya[09, 11]; Quichean Maya[14, 15, 17]; Western Maya[18, 19, 20]; Nahua[25]
Used for (26#): Digestive[01, 03, 04, 07, 09, 19, 20]; Cardiovascular[14]; Respiratory[03]; Skin[01, 03, 05, 20]; Endocrine[01]; Pregnancy[15, 20]; Female genital[03]; General and Unspecified[05, 07, 11, 14, 18, 25]; nd[17]
Cognates:
Language contact:

***Cissus* (Vitaceae)**

Spanish names: Bejuco loco; Pata de Hamaca; Sanatodo; Uva cimarron, san julas
Indigenous names: Chikxtsay[03]; Omisal[05]; Yax tsamnek [07]; Yax tsamnek, yax tsaah, pulik wako, k'apwal huuchul, [07]; Cruz ojo xiw[09]; Roq' Ab'[14]; Rok' ha'b'[17]; Baladxi'i guexii, elbue sang'laash[21]; Blàg-pâsm, Ibæ̀-pâsm, guìzh-pâsm[23]
Used by (9*): Zoque[03]; Totonac[05]; Huastec[07]; Yucatecan Maya[09]; Quichean Maya[14, 17]; Western Maya[18]; Zapotec[21, 23]
Used for (14#): Digestive[09]; Eye[21]; Musculoskeletal[05]; Skin[03, 05, 07, 14, 18, 21]; General and Unspecified[07, 21, 23]; nd[17]
Cognates: Maya: kap; Quich: rok'ab;
Language contact:

***Citharexylum* (Verbenaceae)**

Spanish names:
Indigenous names: Wal to'ol te', ist'am te', ehtiil i tsak te', [07]
Used by (1*): Huastec[07]
Used for (1#): Musculoskeletal[07]
Cognates:
Language contact:

***Citrullus* (Cucurbitaceae)**

Spanish names: Sandía
Indigenous names: Mono[02]; Xandia[03]
Used by (4*): Zoque[02, 03]; Quichean Maya[12]; Western Maya[18]
Used for (5#): Digestive[03]; General and Unspecified[02, 03, 12, 18]
Cognates:
Language contact:

Citrus **(Rutaceae)**

Spanish names: Cajera; Cidra; Lima; Lima, cedra; Limón; Limón agria; Limón agrio; Limón criollo; Limoncillo; Mandarina; Naranja; Naranja agria; Naranja agria/cuxa; Naranja cajera; Naranja dulce; Naranja grey; Nranja agría; Pomela; Sidra; Toronja
Indigenous names: Katzu Tzyina/nanasya/ täme ay[01]; Limones/katzu[01]; China/pos[01]; Imunisy/Tzunakat[02]; Anasa/anasya[02]; Anasa katzu/ansya katzu[02]; Tsootso[03]; Apitx cuy[03]; Tsøpox[04]; Tsuik[04]; Škeja lašuš[05]; Laaxux[06]; Limuunix, xucut[06]; Thimallon lanaax, hiliy lanaax[07]; Hiliy limoon[07]; Chuuchu' liima[07]; Liima[07]; Chuuchu lima)[08]; Tzon te lima[08]; Lanash[08]; Pak'aal[09]; China[09]; Paàk'al[10]; Limonix[12]; Alan[12]; Alanxax[13]; Re' li be'[14]; Xaq i'chi'in[16]; Lamux[16]; Pajäl aranxax[18]; Paj'limon[18]; Pajen cajera[18]; Aranxax[18]; Pajen grey[18]; Ermunex[20]; Narax[20]; Cuanani'ij[21]; Naraxa guayu'u[21]; Naraxa[21]; Yàg-lîm[23]; Yàg-nàrânj[23]; Chilcoztic[28]
Used by (25*): Zoque[01, 02, 03]; Mixe[04]; Totonac[05, 06]; Huastec[07, 08]; Yucatecan Maya[09, 10, 11]; Quichean Maya[12, 13, 14, 15, 16]; Western Maya[18, 19, 20]; Zapotec[21, 22, 23]; Nahua[25, 26, 28]
Used for (288#): Blood[12]; Digestive[01, 02, 03, 04, 06, 07, 08, 09, 11, 12, 13, 18, 19, 20, 21, 22, 23, 25, 26, 28]; Eye[01, 02, 06, 12, 13, 14, 21, 22]; Ear[01]; Cardiovascular[01, 02, 03, 06, 08, 10, 11, 12, 18, 28]; Musculoskeletal[01, 03, 06, 07, 08, 12, 14, 15, 19, 23]; Neurological[01, 02, 03, 12, 13, 18, 19, 21]; Psychological[01, 02, 03, 06, 07, 08, 11, 12, 13, 15, 16, 18, 19, 21, 26]; Respiratory[01, 02, 03, 04, 06, 07, 08, 09, 10, 12, 13, 14, 18, 19, 21, 25, 28]; Skin[01, 02, 03, 04, 07, 12, 13, 14, 19, 21, 25]; Endocrine[01, 02, 06, 12, 18, 23, 25]; Urological[01, 02, 06, 12, 13, 18, 19, 25]; Pregnancy[02, 03, 04, 06, 13, 14, 19, 21, 22, 23]; Female genital[01, 03, 12, 13, 19]; Male genital[02, 03]; General and Unspecified[01, 02, 03, 04, 05, 06, 07, 08, 10, 11, 12, 13, 14, 15, 18, 19, 21, 22, 23, 25, 26]
Cognates:
Language contact:

Cladocolea **(Loranthaceae)**

Spanish names:
Indigenous names: Guièe-ló-yâg-guièts, guièe-ló-yâg-nlìbâd-tsò[23]
Used by (1*): Zapotec[23]
Used for (1#): Neurological[23]
Cognates:
Language contact:

Clematis **(Ranunculaceae)**

Spanish names: Barbas de chivo, pestaña de tecolote; Bejuco de barba viejo; Corraleña blanca
Indigenous names: Mäkshi jäyä[01]; Tsay ¥wix, Kunki kuts¥wi[03]; Skaltzatzat momo, slarpitsitsit monksne[05]; Ithim wahuts, ithim yehtsel, ithim an maam, ithim an pulik, ithim an pulek taata[07]; Xmexmexib[09]; Ch'am pim[14]
Used by (6*): Zoque[01, 03]; Totonac[05]; Huastec[07]; Yucatecan Maya[09]; Quichean Maya[14]
Used for (12#): Neurological[07]; Respiratory[05, 07]; Skin[01, 03, 07, 09, 14]; Pregnancy[03]; Female genital[03]; General and Unspecified[03, 14]
Cognates:
Language contact: Tot <> Hua

Cleome **(Cleomaceae)**

Spanish names:
Indigenous names: Utsun ts'ohool[07]
Used by (2*): Huastec[07]; Quichean Maya[12]
Used for (4#): Digestive[12]; Neurological[07]; Male genital[12]; General and Unspecified[12]
Cognates:
Language contact:

Cleoserrata **(Capparaceae)**

Spanish names:
Indigenous names: Charamooya[03]; Huntal a puunchiix[07]
Used by (2*): Zoque[03]; Huastec[07]
Used for (2#): Eye[07]; Skin[03]
Cognates:
Language contact:

***Clerodendrum* (Lamiaceae)**

Spanish names:
Indigenous names: Ts'een kwiniimte[07]
Used by (1*): Huastec[07]
Used for (1#): Skin[07]
Cognates:
Language contact:

***Clethra* (Clethraceae)**

Spanish names:
Indigenous names: K'ajk'ete'[20]
Used by (2*): Zoque[03]; Western Maya[20]
Used for (3#): Digestive[20]; Skin[03]; Female genital[03]
Cognates:
Language contact:

***Clibadium* (Asteraceae)**

Spanish names:
Indigenous names: Tza'jil Che'[14]
Used by (1*): Quichean Maya[14]
Used for (2#): Respiratory[14]; Endocrine[14]
Cognates:
Language contact:

***Clidemia* (Melastomataceae)**

Spanish names: Tesuatillo de algodón
Indigenous names: Kaka rane [01]; Puki tesua[03]; Tsakam chikab ts'ohool, uxum ts'ohool, ehtiil ts'amuts' uxkwe, ehtiil puwaamte'[07]; Xa bol q'een[15]; Ixq Q'een[15]; Ik pim[17]
Used by (5*): Zoque[01, 03]; Huastec[07]; Quichean Maya[15, 17]
Used for (10#): Digestive[03]; Eye[03]; Cardiovascular[07]; Respiratory[07]; Skin[03]; Pregnancy[15]; Male genital[15]; General and Unspecified[01]; nd[17]
Cognates: Quich: i(x)k;
Language contact:

***Clinopodium* (Lamiaceae)**

Spanish names: Hierba de espanto; Matalzín; Matanci; Poleo; Tripa de rata/raton
Indigenous names: Jupi tane/tzämi tane/ jupi rane/ tzuk'u pu [01]; Piiquaa't tu 'huaa'n[06]; Ts'ots'on ts'ohool, ehtiil tsakam wiichab[07]; Yàg-wăas, guìzh-wăas[23]; Tonalxihuitl[26]
Used by (8*): Zoque[01]; Totonac[05, 06]; Huastec[07]; Yucatecan Maya[09]; Zapotec[22, 23]; Nahua[26]
Used for (21#): Blood[05]; Digestive[01, 07, 23]; Eye[01]; Musculoskeletal[26]; Neurological[01, 07, 09, 22]; Psychological[23, 26]; Respiratory[01, 22]; Pregnancy[07]; General and Unspecified[01, 05, 06, 07, 23, 26]
Cognates:
Language contact:

***Clitoria* (Fabaceae)**

Spanish names:
Indigenous names: Jonwayi[03]
Used by (1*): Zoque[03]
Used for (2#): Neurological[03]; Skin[03]
Cognates:
Language contact:

Clusia **(Clusiaceae)**

Spanish names: Memela; Oreja de Cabro
Indigenous names: Upu kutkuy ay [01]; Ch'iy'ak[12]; Xk'ik' yuk' hubulo'[14]
Used by (3*): Zoque[01]; Quichean Maya[12, 14]
Used for (5#): Musculoskeletal[12]; Psychological[12]; Female genital[01]; General and Unspecified[12, 14]
Cognates: Quich: iyak/ikyuk;
Language contact:

Cnidoscolus **(Euphorbiaceae)**

Spanish names: Chaya; Chaya de monte; Chaya que pica/mala mujer; Chayamansa; Chayo; Chichicastle; Ejaj; Hortiga, mala mujer; Mala mujer; Ortiga
Indigenous names: Ata tsäpe/ata/ kenuk tsäpe[01]; Tzis kä'wang [02]; Kenuk[03]; Gahni, cahh'ne, kgajna, xaca'nat[05]; Kajni[06]; Ak', ma'iy ak'[07]; Kh'ajni[08]; Chay[09]; Yop'ix'ek'[18]; Geč bahd[22]; Yàg-làg[23]; Yàg-pcuà[23]
Used by (14*): Zoque[01, 02, 03]; Totonac[05, 06]; Huastec[07, 08]; Yucatecan Maya[09]; Western Maya[18]; Zapotec[22, 23]; Nahua[25, 26, 27]
Used for (49#): Digestive[03, 08]; Eye[05, 06]; Musculoskeletal[01, 05, 06, 09, 22]; Neurological[02, 03, 05, 06, 07]; Respiratory[03, 06]; Skin[01, 03, 05, 06, 07, 23]; Endocrine[01, 06, 18, 25, 27]; Urological[02, 03, 07, 26]; Pregnancy[01, 05, 07, 18]; Female genital[03, 05, 06, 07, 09]; Male genital[02]; General and Unspecified[06, 09, 27]
Cognates: Zoq: kenuk; Tot: kajni;
Language contact: Zoq > Tot & Hua

Coccocypselum **(Rubiaceae)**

Spanish names: Hoja de cobertón
Indigenous names: Tsujmi ay[03]
Used by (1*): Zoque[03]
Used for (3#): Skin[03]; Female genital[03]; General and Unspecified[03]
Cognates:
Language contact:

Coccoloba **(Polygonaceae)**

Spanish names: Carnero de coyote; Totopotzle, carnero negro/coyote, cinco negrito; Uva de mar; Uvero; Uvero de montaña
Indigenous names: Pakum[03]; J¥mniom pakum[03]; Bob[09]; Shuug nguio'o[21]; Bidxuyej' shuga'a, shuubguijooj'[21]; Shuug[21]
Used by (3*): Zoque[03]; Yucatecan Maya[09]; Zapotec[21]
Used for (20#): Digestive[03, 21]; Musculoskeletal[21]; Respiratory[21]; Skin[03, 09, 21]; Urological[09]; Female genital[03, 21]; General and Unspecified[03, 21]
Cognates:
Language contact:

Cocculus **(Menispermaceae)**

Spanish names:
Indigenous names: Lek'ab t'iim, ichich ts'ohool, kw'itool ichich, lek'ab tiiw, buk ichich[07]
Used by (1*): Huastec[07]
Used for (2#): Digestive[07]; General and Unspecified[07]
Cognates:
Language contact:

Cochlospermum **(Bixaceae)**

Spanish names: Coquito; Pochote; Pongolote; Tecomasuchi
Indigenous names: Putz kuy[02]; Puts cuy[03]; Nø mu'und[04]; Te'aj pochote[18]; T'uyuy, mujrur te'[19]; Vapombu[21]
Used by (6*): Zoque[02, 03]; Mixe[04]; Western Maya[18, 19]; Zapotec[21]
Used for (16#): Digestive[02, 03, 04, 21]; Neurological[03]; Skin[02, 03, 18]; Endocrine[02, 03]; Pregnancy[19]; Female genital[02, 03, 21]; General and Unspecified[03, 21]
Cognates: Zoq: puts kuy;
Language contact:

01-28 refer to the studies described in Table 4.1 * Total number of studies recording this taxon #Total number of use-records

***Cocos* (Arecaceae)**

Spanish names: Coco
Indigenous names:
Used by (10*): Zoque[01, 02, 03]; Mixe[04]; Yucatecan Maya[09]; Quichean Maya[12]; Western Maya[18, 19]; Zapotec[21]; Nahua[28]
Used for (22#): Digestive[01, 02, 03, 04, 12, 18, 19, 21, 28]; Neurological[01]; Psychological[01, 02, 18]; Endocrine[18]; Urological[01, 12]; Pregnancy[09, 21]; Female genital[03, 12]; Male genital[12]; General and Unspecified[21]
Cognates:
Language contact:

***Codonanthe* (Gesneriaceae)**

Spanish names:
Indigenous names: Saq'kar pim[14]
Used by (1*): Quichean Maya[14]
Used for (2#): Respiratory[14]; General and Unspecified[14]
Cognates:
Language contact:

***Coffea* (Rubiaceae)**

Spanish names: Café
Indigenous names: Capel[03]; Kafeey[04]; Capij[06]; Kapee[07]; Cape'[12]; Guis gue[21]
Used by (13*): Zoque[01, 03]; Mixe[04]; Totonac[06]; Huastec[07]; Yucatecan Maya[09]; Quichean Maya[12, 13, 14]; Western Maya[18, 19]; Zapotec[21]; Nahua[26]
Used for (36#): Digestive[03, 06, 21, 26]; Cardiovascular[01]; Musculoskeletal[01, 06, 12]; Neurological[12, 13, 19]; Psychological[26]; Respiratory[01, 12, 19]; Skin[01, 04, 06, 07, 14, 21]; Urological[01, 06]; Pregnancy[01, 09, 12, 13, 21, 26]; Female genital[12]; General and Unspecified[03, 12, 13, 18, 19, 21]
Cognates:
Language contact:

***Cojoba* (Fabaceae)**

Spanish names: Carabina de chango
Indigenous names: Uuts tuj cuy[03]; Xlokok' hi ha'[14]
Used by (2*): Zoque[03]; Quichean Maya[14]
Used for (4#): Digestive[03]; Skin[03, 14]; General and Unspecified[14]
Cognates:
Language contact:

***Colocasia* (Araceae)**

Spanish names: Malanga
Indigenous names:
Used by (2*): Quichean Maya[12]; Zapotec[21]
Used for (2#): Endocrine[12]; General and Unspecified[21]
Cognates:
Language contact:

***Colubrina* (Rhamnaceae)**

Spanish names: Toatán
Indigenous names: Ehtiil tsakam akich, itsaan an tsak look',[07]; Jauté[18]
Used by (2*): Huastec[07]; Western Maya[18]
Used for (2#): Musculoskeletal[07]; General and Unspecified[18]
Cognates:
Language contact:

Columnea **(Gesneriaceae)**

Spanish names: Jala
Indigenous names: Hui'huat[06]; Xoy Q'ehen, Kaq'i pim[14]; Kaki pim[16]; Kak'i pim[17]
Used by (4*): Totonac[06]; Quichean Maya[14, 16, 17]
Used for (8#): Digestive[06, 14]; Eye[14]; Musculoskeletal[06]; Neurological[14, 16]; Respiratory[14]; nd[17]
Cognates: Quich: kaki pim;
Language contact:

Combretum **(Combretaceae)**

Spanish names:
Indigenous names: Uouo tsay, Patan tsay[03]; Qa'xa'an caham[16]; Ka'an shan k'aham[17]
Used by (3*): Zoque[03]; Quichean Maya[16, 17]
Used for (4#): Digestive[03]; Skin[03]; General and Unspecified[16]; nd[17]
Cognates:
Language contact:

Commelina **(Commelinaceae)**

Spanish names: Caterita/hoja de azar/ siempreviva; Hierba de pollo; Madali; Matalín; Matalin verde; Uña de Gato, Tripa de Gallina
Indigenous names: Tzima jäyä/tzuy/ tza'a ay[01]; Po'otz ay/po'o tzoni[02]; Tsus uixpin[03]; Kasmalj[05]; Utek', mapk'ux ch'ohool[07]; U k'ak' ah ko'lebil, Ya'axha'xiw[09]; Karpar Coos[12]; Pitzijor[13]; Tz'i' Maaj Q'ehen[14]; Madali[21]
Used by (11*): Zoque[01, 02, 03]; Totonac[05]; Huastec[07]; Yucatecan Maya[09]; Quichean Maya[12, 13, 14]; Zapotec[21]; Nahua[28]
Used for (29#): Digestive[02, 07, 13, 28]; Eye[01, 02, 05, 07]; Cardiovascular[01, 14]; Neurological[01, 02]; Skin[01, 07, 09, 13, 21, 28]; Endocrine[14]; Urological[07, 13]; Female genital[01, 07, 28]; General and Unspecified[01, 03, 07, 12]
Cognates: Zoq: tsu;
Language contact:

Comocladia **(Anacardiaceae)**

Spanish names: Hinchador, hinchahuevo
Indigenous names: Latz[21]
Used by (1*): Zapotec[21]
Used for (1#): Skin[21]
Cognates:
Language contact:

Conostegia **(Melastomataceae)**

Spanish names: Capulin niua; Moradito del cerro
Indigenous names: Jeepe[03]; Chuch jeepe[03]
Used by (2*): Zoque[03]; Zapotec[21]
Used for (6#): Digestive[03]; Neurological[03]; Pregnancy[03]; Female genital[03]; General and Unspecified[21]
Cognates:
Language contact:

Convolvulus **(Convolvulaceae)**

Spanish names: Flor de virgen chico
Indigenous names: Badooj buishii[21]
Used by (1*): Zapotec[21]
Used for (1#): General and Unspecified[21]
Cognates:
Language contact:

***Conyza* (Asteraceae)**

Spanish names: Hoja de azar; Simonilla
Indigenous names: Tza'a tzoy[01]; Jok poy[03]; K'atab' Chaj[14]; Sìmònî[23]
Used by (4*): Zoque[01, 03]; Quichean Maya[14]; Zapotec[23]
Used for (9#): Digestive[23]; Cardiovascular[23]; Respiratory[14]; Skin[03, 14]; Urological[03]; Female genital[03]; General and Unspecified[01, 14]
Cognates:
Language contact:

***Corchorus* (Malvaceae)**

Spanish names: Malvavisco/ malva macho
Indigenous names: Ueji ay[03]; Pehtsul kw'eet, pehtsul thipon, thipon kweet, loliy thipon[07]
Used by (3*): Zoque[01, 03]; Huastec[07]
Used for (10#): Digestive[01, 07]; Eye[07]; Neurological[07]; Psychological[03]; Skin[07]; Urological[07]; Pregnancy[07]; General and Unspecified[01, 07]
Cognates:
Language contact:

***Cordia* (Boraginaceae)**

Spanish names: Escobillo, mais grande; Gulabere; Gulaveri; Hoja de alacrán barraca; Solería; Vara negra; Xobarora
Indigenous names: Syunuk[02]; Kaku'e ay[02]; Y¥k yom tsay[03]; Kiwa[03]; Wiixte'[07]; Cirricote, Kop'te'[09]; Jolob te'[20]; Shubaruuba'a[21]; Gubenigw[22]
Used by (8*): Zoque[02, 03]; Mixe[04]; Huastec[07]; Yucatecan Maya[09]; Western Maya[20]; Zapotec[21, 22]
Used for (21#): Digestive[03, 20, 21, 22]; Eye[02, 07]; Musculoskeletal[21]; Psychological[03]; Respiratory[04, 09]; Skin[02, 03]; Female genital[02, 03, 04]; General and Unspecified[02, 04, 09, 21, 22]
Cognates: CoreM: op'te;
Language contact:

***Cordyline* (Asparagaceae)**

Spanish names: Cola de gallo; Palmita
Indigenous names: Cuxtzi[16]
Used by (3*): Zoque[01, 02]; Quichean Maya[16]
Used for (4#): Musculoskeletal[01]; Neurological[16]; Endocrine[02]; Female genital[02]
Cognates:
Language contact:

***Coreopsis* (Asteraceae)**

Spanish names:
Indigenous names: Bajk' al te'[20]; Ncuàan-bzhiân, guièe ngùzhánch[23]
Used by (2*): Western Maya[20]; Zapotec[23]
Used for (5#): Digestive[20, 23]; Psychological[23]; Endocrine[23]; General and Unspecified[23]
Cognates:
Language contact:

***Coriandrum* (Apiaceae)**

Spanish names: Cilandro; Cilantro; Culantro
Indigenous names: Cuulantu[06]; Kulaantu[07]; Culanto, uklanto´[13]; Sìlândr[23]
Used by (10*): Zoque[01]; Totonac[06]; Huastec[07]; Yucatecan Maya[09]; Quichean Maya[12, 13, 14]; Western Maya[18, 19]; Zapotec[23]
Used for (16#): Digestive[06, 07, 09, 12, 13, 18, 19]; Cardiovascular[13]; Musculoskeletal[14]; Psychological[12]; Respiratory[14]; Endocrine[12]; Urological[01]; Pregnancy[13, 23]; General and Unspecified[12]
Cognates:
Language contact:

***Cornutia* (Lamiaceae)**

Spanish names: Carreto; Piojillo/pangajey; Tabaquillo
Indigenous names: M'yonh kuy/ monh'iun kuy[01]; Cana ay, Eexcuy[03]; Aškut kiwi[05]; Xolte'xnuk[09]; Loto'o che[11]; Sa'q Aatz'un[14]; Job lo te[16]; Yop'waúm'[18]; Yax e vomol[20]
Used by (9*): Zoque[01, 03]; Totonac[05]; Yucatecan Maya[09, 11]; Quichean Maya[14, 16]; Western Maya[18, 20]
Used for (17#): Digestive[20]; Musculoskeletal[01, 03, 09, 11]; Neurological[16, 18]; Respiratory[09]; Skin[03, 14]; Pregnancy[01]; General and Unspecified[01, 03, 05, 09, 11, 18]
Cognates: CoreM: lotV;
Language contact: Highland Popoluca <> Tot <> Yuc <> Kaqchikel

***Cortaderia* (Poaceae)**

Spanish names: Caña blanca
Indigenous names: Nida'a quitzii[21]
Used by (1*): Zapotec[21]
Used for (2#): Respiratory[21]; General and Unspecified[21]
Cognates:
Language contact:

***Cosmos* (Asteraceae)**

Spanish names: Copalio
Indigenous names: Molajtu sotyi[03]
Used by (2*): Zoque[01, 03]
Used for (3#): Digestive[01]; Skin[03]; General and Unspecified[03]
Cognates:
Language contact:

***Costus* (Costaceae)**

Spanish names: Caña agria; Caña de Cristo; Caña de Cristo ; Caña de cristo/caña agria/cañita; Caña de jabalí; Caña de venado; Caña de venado, caña de jabalí; Cañita; Cañuela
Indigenous names: Katzu aksa/baston katzu/katzu tane[01]; Katzu syitsi[02]; Chimpa tutu[03]; Chankat juki[05]; Cha'ncat juki, cha'ncat pa'xni[06]; Pakaab olom[07]; Tch'ama'jij[12]; Chu'un, Paguy t'e[14]; Chu'un[14]; Ch'uun te'[17]; Oj paj[18]; Nidaii[21]; Cuapitzoatl[25]; Tepeohuate[26]
Used by (13*): Zoque[01, 02, 03]; Totonac[05, 06]; Huastec[07]; Quichean Maya[12, 14, 17]; Western Maya[18]; Zapotec[21]; Nahua[25, 26]
Used for (42#): Blood[12]; Digestive[01, 02, 03, 07, 12, 21, 26]; Cardiovascular[01]; Musculoskeletal[12]; Skin[07]; Endocrine[06, 12, 14]; Urological[01, 02, 03, 05, 06, 07, 14, 18, 21, 25, 26]; Pregnancy[14]; Female genital[03]; Male genital[14]; Social problems[14]; General and Unspecified[05, 07, 12, 14, 21, 25]; nd[17]
Cognates: Zoq: katzu; Tot: chancat juki; Quich: chu'un;
Language contact:

***Couepia* (Chrysobalanaceae)**

Spanish names: Olozapote
Indigenous names: Pillum[03]
Used by (1*): Zoque[03]
Used for (2#): Skin[03]; Female genital[03]
Cognates:
Language contact:

***Crassula* (Crassulaceae)**

Spanish names: Siempreviva
Indigenous names: Wiq bac, pix laq[13]
Used by (1*): Quichean Maya[13]
Used for (5#): Digestive[13]; Eye[13]; Musculoskeletal[13]; Neurological[13]; Skin[13]
Cognates:
Language contact:

***Crataegus* (Rosaceae)**

Spanish names: Cardo; Manzanilla Arbol; Manzanita; Raíz de manzanita, tejocote
Indigenous names: Q'enum-che'[12]; Ch'ix te', k'at'ix, k'achi'ix[20]; Yàg-mànzànît[23]
Used by (4*): Zoque[01]; Quichean Maya[12]; Western Maya[20]; Zapotec[23]
Used for (15#): Blood[12]; Digestive[12, 20]; Musculoskeletal[12, 23]; Neurological[12, 20]; Respiratory[01, 12, 20]; Skin[20]; Urological[23]; Male genital[12]; General and Unspecified[01, 12]
Cognates:
Language contact:

***Crateva* (Capparaceae)**

Spanish names: Coscorrón
Indigenous names: Tsine te', ts'olob ok, thak'chook' buuru, thak'chook' uut', thi'te', ist'am te', thak'chook' teneklaab[07]; Bajpam[18]
Used by (2*): Huastec[07]; Western Maya[18]
Used for (4#): Ear[07]; Musculoskeletal[18]; Neurological[18]; Skin[07]
Cognates:
Language contact:

***Crescentia* (Bignoniaceae)**

Spanish names: Jícara; Jícara, Morro; Morrito; Morro; Morro simarrón; Sycamorro/jícara
Indigenous names: Tzima[01]; Tzima[02]; Jeepe[03]; Patsim[04]; Thoot tima'[07]; Ra taxy[08]; Luch[09]; Tzi Max[12]; Rix'hijom[14]; T'ub[18]; Tzimaj[19]; Buru boj[21]; Buru shiiga'a[21]; Yàg-zhìg[23]
Used by (13*): Zoque[01, 02, 03]; Mixe[04]; Huastec[07, 08]; Yucatecan Maya[09]; Quichean Maya[12, 14]; Western Maya[18, 19]; Zapotec[21, 23]
Used for (39#): Blood[03]; Digestive[02, 04, 18]; Ear[03]; Musculoskeletal[02, 03, 07, 21]; Neurological[14, 18]; Respiratory[02, 03, 04, 08, 09, 12, 18, 19, 21]; Skin[02, 12, 21]; Urological[23]; Pregnancy[03, 04, 12]; Male genital[12]; General and Unspecified[01, 07, 12, 18, 21]
Cognates: MZ: tsim; Zoq: tsima; Maya: tsima/tima; CoreM: tsima; Zap: zhig;
Language contact: MZ > Maya & Zap

***Crinum* (Amaryllidaceae)**

Spanish names: Lirio; Palenque, maguey blanco
Indigenous names: Xts'ulam[09]; Pets'kini, Pets'kinil[09]
Used by (3*): Zoque[01, 03]; Yucatecan Maya[09]
Used for (8#): Digestive[01]; Neurological[01, 09]; Respiratory[09]; Skin[03, 09]; Endocrine[03]; General and Unspecified[01]
Cognates:
Language contact:

***Critonia* (Asteraceae)**

Spanish names: Canotillo; Hoja de Agua; Hoja de contra-viento, hoja de viento, choplé; Lengua de cierva; Lengua de vaca; San Nicolas; Vara negra
Indigenous names: Vaca totz[01]; Wakas yenkuy/ wakas tots ay/wakasy tots[02]; Cortia ay[03]; Poma cuy[03]; Tsuxky ajuk tyujt[04]; Pajk teky, payøwa'ats[04]; T'unu' holol[07]; Holol, alte may[07]; Tsamnek tok'te', animas ts'ohool[07]; Lok' ab' winq'[14]; Yopo säc'[18]; Guish lujtz yuss[21]
Used by (11*): Zoque[01, 02, 03]; Mixe[04]; Totonac[05]; Huastec[07]; Quichean Maya[12, 14]; Western Maya[18]; Zapotec[21]; Nahua[26]
Used for (41#): Digestive[02, 03, 07, 21]; Musculoskeletal[01, 02, 03, 04, 07, 18, 21]; Neurological[02, 03, 07]; Respiratory[07, 12, 14, 26]; Skin[03, 05, 07]; Urological[07]; Pregnancy[02, 03]; Female genital[02, 03, 04]; General and Unspecified[03, 04, 07, 18, 21]
Cognates:
Language contact:

***Crossopetalum* (Celastraceae)**

Spanish names: Viperol negro
Indigenous names: Tzutzi tzaj käki[01]; Ts'amuts' uxkwe', tsakam itsal koox, ts'amuts' uthu, tsatsa' ilaal[07]; Ra Mox[15]
Used by (4*): Zoque[01]; Huastec[07]; Yucatecan Maya[09]; Quichean Maya[15]
Used for (9#): Digestive[07, 09]; Eye[07]; Musculoskeletal[15]; Psychological[01]; Skin[07, 09]; Female genital[07]; General and Unspecified[15]
Cognates:
Language contact:

***Crotalaria* (Fabaceae)**

Spanish names: Chepíl; Chipile; Chipilín; Chipillín; Sonaje de muerto
Indigenous names: Tza' tzäpä/ tsaj tzäpä[01]; Txipiñ ts¥p¥[03]; Tsuts ts¥p¥[03]; Paxekuiny[04]; Mak'xuxut niy[06]; Thootil tsan, pok' thoot[07]; Much', B'uch[12]; Much'[12]; Much'[13]; Chepil[21]; Pxĭizh, ncuàan-yè[23]
Used by (9*): Zoque[01, 03]; Mixe[04]; Totonac[06]; Huastec[07]; Quichean Maya[12, 13]; Zapotec[21, 23]
Used for (27[#]): Blood[01]; Digestive[01, 03, 12, 23]; Cardiovascular[01]; Musculoskeletal[12]; Neurological[12]; Psychological[03, 04, 06, 12, 13]; Skin[01, 03, 21, 23]; Urological[06]; Pregnancy[01]; Female genital[23]; Male genital[01]; General and Unspecified[01, 03, 07, 21, 23]
Cognates: Zoq: tsaj/tsuts tsäpä; Quich: much;
Language contact: Chiapas Zoq <> Hua; Tot <> Quich

***Croton* (Euphorbiaceae)**

Spanish names: Agua dulce; Banquito; Cascarilla; Chiliue del monte; Contra; Contra cimarrón; Copachín; Copalchi; Copalchin; Hierba de Cancer Macho; Llora sangre; Llora sangre/palo de sangre; Mata pescado, hoja de mesquino; Pata de paloma; Quina, ventoside; Sangregado; Sangregrado
Indigenous names: Nhäpin kuy/ po'a nhäpin/ kun nhäpin / nhäpin pi'owa/ jäpya nhäpin [01]; Tin kuy/ponoj kuy[01]; Jäpya nhäpin[01]; Nä'wang niwi[02]; N¥piñi cuy[03]; Copa n¥piñi cuy, Soj kobak, Soj muk[03]; Tam cuy[03]; Tapu ay[03]; Tuxoj tikts[04]; Pop tsaxoj[04]; Panii[04]; Pazlnankiwi, puklhni, puelnankiwi[05]; Puthwal[07]; Thak oliy[07]; Oliy[07]; Xoliiman[07]; Xiixte', xits' te'[07]; Luk, ist ts'ohool[07]; Xebalam, Butsumukuy, Xikinch'omak[09]; Ik-aban[09]; Kokche'[09]; Ek'balam, Xikm burro, Xikinch'omak[09]; Pets'k'uts[09]; Sakpokche', Ik-haab[09]; Iri skutČ[10]; Ru xup Top, Sa'l Q'os[12]; Copal chi'[17]; Chi'ich' bot[20]; Yague riene[21]; Guaanashnash[21]; Guixaxunaashii[21]; Blàg-zhnâzh, guìzh-blàg-zhnâzh, guìzh-ngùdzìi, guìzh-ngùd-mzhiè-dán[23]
Used by (15*): Zoque[01, 02, 03]; Mixe[04]; Totonac[05]; Huastec[07]; Yucatecan Maya[09, 10]; Quichean Maya[12, 13, 17]; Western Maya[20]; Zapotec[21, 23]; Nahua[26]
Used for (94[#]): Blood[10]; Digestive[01, 02, 03, 04, 07, 20, 21]; Eye[03, 07, 23]; Musculoskeletal[01, 03, 07]; Neurological[01, 03, 07, 12, 26]; Respiratory[02, 03, 05, 09, 12, 21]; Skin[01, 02, 03, 04, 05, 07, 09, 21, 23]; Endocrine[01, 12, 26]; Urological[01, 02, 03]; Pregnancy[02, 05, 13]; Female genital[02, 03, 07, 21]; General and Unspecified[01, 03, 05, 07, 09, 21, 23, 26]; nd[17]
Cognates: MZ: soj; Zoq: näpin; Yuc: skuts; Zap: xnax;
Language contact:

***Crusea* (Rubiaceae)**

Spanish names:
Indigenous names: Uekx sotyi[03]
Used by (1*): Zoque[03]
Used for (1[#]): Skin[03]
Cognates:
Language contact:

***Cryosophila* (Arecaceae)**

Spanish names: Escoba
Indigenous names:
Used by (1*): Yucatecan Maya[10]
Used for (1[#]): Skin[10]
Cognates:
Language contact:

***Ctenitis* (Dryopteridaceae)**

Spanish names: Cola de Piedra
Indigenous names: Q'uq'i Pek[14]
Used by (1*): Quichean Maya[14]
Used for (2[#]): General and Unspecified[14]
Cognates:
Language contact:

Cucumis **(Cucurbitaceae)**

Spanish names: Melón; Pepino
Indigenous names:
Used by (4*): Zoque[01, 02]; Quichean Maya[12]; Western Maya[18]
Used for (7#): Cardiovascular[02]; Musculoskeletal[01]; Skin[18]; Endocrine[02]; Urological[02]; General and Unspecified[12]
Cognates:
Language contact:

Cucurbita **(Cucurbitaceae)**

Spanish names: Ayote; Calabaza; Calabaza amarga; Calabaza de castilla; Chilacayote
Indigenous names: (Yama joma/nak) pa'sun[01]; Pa'so/une/ukum[02]; Naspaasuñ[03]; Tsi'i[04]; Ni'pxi[06]; Maka ni'pxi[06]; Ka'ii[06]; Ts'oop[07]; Qo'q[13]; Mukun[13]; Ratz'umi k'um [14]; Ch'um[18]; Ch'um[19]; Mayil[20]; Gueatu'u[21]; Guedu laac[21]; Giht[22]; Ayotli[28]
Used by (14*): Zoque[01, 02, 03]; Mixe[04]; Totonac[06]; Huastec[07]; Quichean Maya[13, 14]; Western Maya[18, 19, 20]; Zapotec[21, 22]; Nahua[28]
Used for (32#): Blood[13]; Digestive[01, 02, 03, 06, 14, 18, 19, 20, 21, 22, 28]; Neurological[02]; Skin[01, 03, 04, 18, 21, 28]; Endocrine[01, 18]; Urological[06, 21]; Pregnancy[13, 18]; Male genital[21]; General and Unspecified[07, 19, 21]
Cognates: Zoq: pasun; CoreM: k'um / ch'um;
Language contact: Quich > Chimalapa Zoq

Cuminum **(Apiaceae)**

Spanish names: Comino
Indigenous names:
Used by (6*): Zoque[01, 02]; Totonac[06]; Quichean Maya[12, 13]; Zapotec[21]
Used for (10#): Digestive[01, 12, 21]; Neurological[06, 12]; Respiratory[06, 21]; Pregnancy[02, 13]; General and Unspecified[13]
Cognates:
Language contact:

Cunila **(Lamiaceae)**

Spanish names: Menta silvestre
Indigenous names: Cruz Q'os[12]
Used by (1*): Quichean Maya[12]
Used for (4#): Digestive[12]; Psychological[12]; Skin[12]; General and Unspecified[12]
Cognates:
Language contact:

Cupania **(Sapindaceae)**

Spanish names: Quebracho
Indigenous names: Akpak[03]
Used by (2*): Zoque[03]; Totonac[05]
Used for (4#): Digestive[03]; Skin[03]; Female genital[03]; General and Unspecified[05]
Cognates:
Language contact:

Cuphea **(Lythraceae)**

Spanish names: Comida de colibri; Elotillo/malvarisco morado; Escoba del rio/ de agua; Hierba de venado; Mirto; Rosalito, kufeo, coralia; Sanguinaria
Indigenous names: Mojko matsyi/ mojkuy mätzyik [01]; N¥ang s¥gay[03]; Arooz ts'ohool[07]; Uwa' tzi'kin, uwa'tzunum, m'ax[13]; Mesb'eel li ha', Xmes hi ha, Mes'uul ha'[14]; Q'anruhil Q'ehen[14]
Used by (6*): Zoque[01, 03]; Huastec[07]; Quichean Maya[13, 14]; Nahua[26]
Used for (25#): Blood[01, 14]; Digestive[01, 13, 14, 26]; Cardiovascular[01]; Musculoskeletal[01]; Neurological[01, 03]; Psychological[14]; Respiratory[01, 14]; Skin[01, 03, 07, 26]; Female genital[26]; General and Unspecified[03, 07, 13, 14]
Cognates:
Language contact:

***Cupressus* (Cupressaceae)**

Spanish names: Ciprés
Indigenous names: K'isis[12]; K'sis[13]; Xnaq' Cipres [14]
Used by (4*): Zoque[01]; Quichean Maya[12, 13, 14]
Used for (16#): Cardiovascular[12]; Musculoskeletal[01, 12, 13]; Neurological[12, 13, 14]; Respiratory[12, 13]; Skin[01, 13]; Urological[01]; Pregnancy[12]; Female genital[12]; General and Unspecified[01, 12]
Cognates: Quich: kisis;
Language contact:

***Curatella* (Dilleniaceae)**

Spanish names: Lengua de vaca; Tachicón
Indigenous names: Wakas yenkuy[02]; Potcuy[03]; Xø popt lijar[04]; Balaga lujtza yussu'[21]
Used by (4*): Zoque[02, 03]; Mixe[04]; Zapotec[21]
Used for (8#): Digestive[03]; Musculoskeletal[02, 04]; Skin[03, 04]; Female genital[03]; General and Unspecified[04, 21]
Cognates:
Language contact:

***Curcuma* (Zingiberaceae)**

Spanish names: Curcuma
Indigenous names: Azafraan[07]
Used by (3*): Zoque[01]; Huastec[07]; Quichean Maya[12]
Used for (6#): Digestive[01, 12]; Musculoskeletal[07]; Psychological[12]; Respiratory[07]; Skin[12]
Cognates:
Language contact:

***Cuscuta* (Convolvulaceae)**

Spanish names: Barba del Diablo; Cardenillo, cuernas del diablo; Fideos, cordoncillo; Sopa de fideo, cardenillo; Tripa de gallina; Zacapal
Indigenous names: Tza'a tane/ pu'utze [01]; Puutx tsay[03]; Snuku[05]; Fideo ts'ohool, man ch'aah[07]; Q'en Sumachi' Aq'om[12]; Zacapactle[26]
Used by (8*): Zoque[01, 03]; Totonac[05]; Huastec[07]; Quichean Maya[12, 13]; Nahua[25, 26]
Used for (16#): Blood[05]; Digestive[03, 05]; Neurological[07]; Skin[07, 13, 26]; Endocrine[25]; Urological[03, 12, 25]; General and Unspecified[01, 03, 07, 12]
Cognates: Zoq: puts/putx;
Language contact:

***Cyathea* (Cyatheaceae)**

Spanish names: Cola de caballo; Colandrillo, cola de chango; Palo de culebra; Palo de vibora/mano de leon/cola de mono/helecho macho
Indigenous names: Kan nak'e /tzawi tutz/ tzimui[01]; Kinä näpin/suyi suki pung [02]; Shgola miigu'u[21]
Used by (3*): Zoque[01, 02]; Zapotec[21]
Used for (12#): Digestive[01, 02, 21]; Eye[02]; Cardiovascular[02]; Endocrine[01, 02]; Urological[01, 02]; General and Unspecified[01, 02]
Cognates:
Language contact:

***Cyclanthera* (Cucurbitaceae)**

Spanish names:
Indigenous names: Chunak Q'en[14]
Used by (1*): Quichean Maya[14]
Used for (1#): Skin[14]
Cognates:
Language contact:

***Cyclospermum* (Apiaceae)**

Spanish names:
Indigenous names: Kulantoil an t'ot[07]
Used by (2*): Huastec[07]; Western Maya[20]
Used for (2#): Digestive[20]; Respiratory[07]
Cognates:
Language contact:

***Cydonia* (Rosaceae)**

Spanish names: Membrillo
Indigenous names: Yàg-mèmbrî[23]
Used by (3*): Quichean Maya[12, 13]; Zapotec[23]
Used for (8#): Digestive[13, 23]; Cardiovascular[12]; Musculoskeletal[12]; Neurological[12]; Skin[12]; Urological[12]; General and Unspecified[12]
Cognates:
Language contact:

***Cymbopetalum* (Annonaceae)**

Spanish names: Oreja de gato; Orifela
Indigenous names: Yagamishu'u[21]
Used by (3*): Zoque[01, 02]; Zapotec[21]
Used for (9#): Digestive[02]; Ear[02]; Musculoskeletal[21]; Neurological[02, 21]; Psychological[01]; Respiratory[02, 21]; Female genital[02]
Cognates:
Language contact:

***Cymbopogon* (Poaceae)**

Spanish names: Té de Limón; Te de limón, zacate limón; Té limón; Telimon; Telimon, zacate limón; Telimón/ te de zacate; Zacate de limon; Zacate limón
Indigenous names: Paja'k wäjpajk ay [02]; Limunmuk[03]; Limoon toom[07]; Q'is Q'im [14]; Bänälä limón[18]; Sansiwre wamal[20]; Te guisa[21]
Used by (18*): Zoque[01, 02, 03]; Mixe[04]; Totonac[05, 06]; Huastec[07]; Yucatecan Maya[09, 10, 11]; Quichean Maya[12, 13, 14]; Western Maya[18, 19, 20]; Zapotec[21]; Nahua[25]
Used for (52#): Blood[12]; Digestive[01, 03, 04, 05, 07, 09, 12, 18, 20, 21]; Cardiovascular[02, 05, 12, 13, 14]; Musculoskeletal[13, 18]; Neurological[01, 13]; Psychological[01, 02, 12, 18, 21]; Respiratory[01, 02, 03, 09, 11, 12, 13, 18, 19, 25]; Endocrine[02, 12]; Urological[02, 07]; Pregnancy[06, 13, 18]; Female genital[01, 12, 13]; General and Unspecified[01, 10, 11, 12, 13, 18, 21]
Cognates:
Language contact:

***Cyperus* (Cyperaceae)**

Spanish names: Chantulli; Chintul; Cintule; Cituli; Grama; Grama Silvestre; Piomía; Zacate
Indigenous names: Pioninu, Puutx uixpin[03]; Haluk'laab ts'ohool, tsakam tsaw, tathim toom[07]; Tupux[09]; Sabana Aq'om[12]; K'im[13]; Poopil Q'ehen[14]; Tzajal yisim be[20]; Shapandú[21]
Used by (11*): Zoque[02, 03]; Mixe[04]; Huastec[07]; Yucatecan Maya[09]; Quichean Maya[12, 13, 14]; Western Maya[20]; Zapotec[21]; Nahua[27]
Used for (27#): Blood[12]; Digestive[07, 12, 20, 27]; Musculoskeletal[21]; Neurological[04, 12, 21]; Psychological[03]; Respiratory[02, 04, 09]; Skin[02, 04, 07, 21]; Urological[07, 12]; Pregnancy[13, 21]; General and Unspecified[07, 14, 21, 27]
Cognates:
Language contact: Highland Popoluca <> Yuc

***Cyrtocarpa* (Anacardiaceae)**

Spanish names: Chupandilla; Ciruelo
Indigenous names: K'inim te'[08]
Used by (2*): Huastec[08]; Nahua[27]
Used for (5#): Digestive[08]; Neurological[08]; Skin[08]; Urological[27]; General and Unspecified[08]
Cognates:
Language contact:

***Cyrtocymura* (Asteraceae)**

Spanish names: Tzitit
Indigenous names: Tsisykuy[01]
Used by (1*): Zoque[01]
Used for (2#): Neurological[01]; Respiratory[01]
Cognates:
Language contact:

***Dahlia* (Asteraceae)**

Spanish names: Santa Catarina; Tunay
Indigenous names: Tney, tunay, oi[13]; Ch'olip, cho'liv, ch'olov[20]
Used by (3*): Quichean Maya[12, 13]; Western Maya[20]
Used for (18#): Digestive[12, 13, 20]; Eye[13]; Ear[13, 20]; Cardiovascular[12]; Neurological[13, 20]; Psychological[13]; Respiratory[20]; Skin[12, 13]; Urological[12, 13]; General and Unspecified[12, 13, 20]
Cognates: CoreM: o(l)i;
Language contact:

***Dalbergia* (Fabaceae)**

Spanish names: Guayacan
Indigenous names:
Used by (1*): Zapotec[21]
Used for (3#): Musculoskeletal[21]; Female genital[21]; General and Unspecified[21]
Cognates:
Language contact:

***Dalea* (Fabaceae)**

Spanish names: Quebrapiedra; Toronjíl
Indigenous names: Tsun tsun[03]; Tsakam chilab[07]; Azúfre xiw, Suyk'ak'[09]; Nlít-quiè, guìzh-nlít-quiè[23]
Used by (5*): Zoque[01, 03]; Huastec[07]; Yucatecan Maya[09]; Zapotec[23]
Used for (11#): Blood[07]; Digestive[03, 23]; Ear[07]; Musculoskeletal[07]; Skin[09]; Urological[01]; Pregnancy[03]; General and Unspecified[03, 07]
Cognates:
Language contact:

***Dalechampia* (Euphorbiaceae)**

Spanish names:
Indigenous names: Kw'aat', la'ix iits', tithelom ch'ohool[07]
Used by (1*): Huastec[07]
Used for (1#): Neurological[07]
Cognates:
Language contact:

***Danaea* (Marattiaceae)**

Spanish names:
Indigenous names:
Used by (1*): Quichean Maya[17]
Used for (1#): nd[17]
Cognates:
Language contact:

***Datura* (Solanaceae)**

Spanish names: Chanico; Contra brujo; Toloache; Toloache blanco; Toloache morado; Toloache, reina de la noche; Toloache/ hierba del diablo
Indigenous names: Matunu[03]; Campana pøh[04]; San pedro ujts[04]; Thanab[07]; Chaniko, Chamisa[09]; Buuruj'hui moradu[21]; Mbuuruj'hui[21]; Blàg-rzûdz, guìzh-rzûdz[23]
Used by (11*): Zoque[01, 02, 03]; Mixe[04]; Huastec[07]; Yucatecan Maya[09, 10]; Quichean Maya[13]; Western Maya[18]; Zapotec[21, 23]
Used for (35#): Digestive[02, 03, 13, 18, 21]; Eye[02]; Cardiovascular[18]; Musculoskeletal[02, 04, 21, 23]; Neurological[13]; Psychological[13, 21]; Skin[01, 02, 04, 07, 09, 21, 23]; Female genital[02, 03, 07, 21]; General and Unspecified[03, 04, 07, 10, 21, 23]
Cognates: Zap: ruj;
Language contact:

***Daucus* (Apiaceae)**

Spanish names: Zanahoria
Indigenous names:
Used by (3*): Zoque[01, 02]; Quichean Maya[12]
Used for (8#): Digestive[01, 12]; Eye[12]; Musculoskeletal[12]; Psychological[12]; Skin[12]; Urological[02]; General and Unspecified[02]
Cognates:
Language contact:

***Davilla* (Dilleniaceae)**

Spanish names:
Indigenous names: Potcuy tsay[03]; Kak'i k'aham[17]
Used by (2*): Zoque[03]; Quichean Maya[17]
Used for (2#): Digestive[03]; nd[17]
Cognates:
Language contact:

***Decachaeta* (Asteraceae)**

Spanish names: Tabacco cimarrón
Indigenous names: Cimarrón ts¥ui[03]
Used by (1*): Zoque[03]
Used for (2#): Urological[03]; Female genital[03]
Cognates:
Language contact:

***Decatropis* (Rutaceae)**

Spanish names:
Indigenous names: Bichaam te'[07]
Used by (1*): Huastec[07]
Used for (3#): Digestive[07]; Musculoskeletal[07]; General and Unspecified[07]
Cognates:
Language contact:

***Dendropanax* (Araliaceae)**

Spanish names: Palo de agua
Indigenous names: Un cuy[03]; Multe'[07]; Cojl[17]
Used by (3*): Zoque[03]; Huastec[07]; Quichean Maya[17]
Used for (13#): Eye[03]; Ear[03]; Musculoskeletal[07]; Neurological[03, 07]; Psychological[03, 07]; Respiratory[07]; Skin[03, 07]; General and Unspecified[03, 07]; nd[17]
Cognates:
Language contact:

***Deppea* (Rubiaceae)**

Spanish names:
Indigenous names:
Used by (1*): Zoque[03]
Used for (1#): General and Unspecified[03]
Cognates:
Language contact:

***Desmanthus* (Fabaceae)**

Spanish names: Guajillo
Indigenous names: Salat-ik', Sibik'xiw, Sib-ik'', Sik'ink'ax[09]; Lya las[22]
Used by (2*): Yucatecan Maya[09]; Zapotec[22]
Used for (2#): Digestive[22]; Eye[09]
Cognates:
Language contact:

***Desmodium* (Fabaceae)**

Spanish names: Cadillo rojo; Pegapega
Indigenous names: T¥ pitx nang tsang[03]; Ujts jayi pekpa[04]; T'apay thekw'eel, xutsun t'eel, bokoolil an t'eel, bakanil t'eel, anuch kw'a'[07]; Ts'at' ts'ohool, t'apay thekw'eel[07]; Pak'umpak'[09]; Chim pim[14]; Ch'in pim[17]
Used by (7*): Zoque[02, 03]; Mixe[04]; Huastec[07]; Yucatecan Maya[09]; Quichean Maya[14, 17]
Used for (15#): Digestive[03, 07]; Musculoskeletal[14]; Skin[02, 03, 07, 09]; Urological[02, 04]; Pregnancy[02, 03, 14]; General and Unspecified[14]; nd[17]
Cognates:
Language contact: Highland Popoluca <> Hua; Mixe <> Yuc

***Dialium* (Fabaceae)**

Spanish names:
Indigenous names: T¥m paki[03]
Used by (1*): Zoque[03]
Used for (3#): Digestive[03]; Pregnancy[03]; General and Unspecified[03]
Cognates:
Language contact:

***Dichondra* (Convolvulaceae)**

Spanish names:
Indigenous names: Ahuaxmama[26]
Used by (1*): Nahua[26]
Used for (1#): Skin[26]
Cognates:
Language contact:

***Dichorisandra* (Commelinaceae)**

Spanish names:
Indigenous names: Tzima'j pim[17]
Used by (1*): Quichean Maya[17]
Used for (1#): nd[17]
Cognates:
Language contact:

01-28 refer to the studies described in Table 4.1 * Total number of studies recording this taxon #Total number of use-records

Dicliptera **(Acanthaceae)**

Spanish names:
Indigenous names: Uxkwe' ts'ohool, wits paya'[07]; Tza' Tzalun Mi' Ha'[14]
Used by (2*): Huastec[07]; Quichean Maya[14]
Used for (3#): Female genital[07]; General and Unspecified[07, 14]
Cognates:
Language contact:

Dictyanthus **(Apocynaceae)**

Spanish names:
Indigenous names: Ensul, Emtsul[09]
Used by (1*): Yucatecan Maya[09]
Used for (1#): General and Unspecified[09]
Cognates:
Language contact:

Dictyoxiphium **(Tectariaceae)**

Spanish names:
Indigenous names: Usi xul k'ejen[17]
Used by (1*): Quichean Maya[17]
Used for (1#): nd[17]
Cognates:
Language contact:

Didymaea **(Rubiaceae)**

Spanish names: Pegapega
Indigenous names: Ajak Ruk'amal Ibo'ch Aq'om[12]
Used by (1*): Quichean Maya[12]
Used for (4#): Digestive[12]; Cardiovascular[12]; Musculoskeletal[12]; Neurological[12]
Cognates:
Language contact:

Digitalis **(Plantaginaceae)**

Spanish names: Digital
Indigenous names:
Used by (1*): Quichean Maya[12]
Used for (3#): Cardiovascular[12]; Musculoskeletal[12]; Endocrine[12]
Cognates:
Language contact:

Dioscorea **(Dioscoreaceae)**

Spanish names: Barbasco; Barbasco Amarillo; Barbasco, calabacilla; Cocolmeca; Cuculmeca; Ñame; Tumba vaquero
Indigenous names: Tumi tätz[01]; Puutx naaku[03]; Manat[05]; Laab ith[07]; Panil book, ix thuyum[07]; Cancer-ak', Wil-ak'[09]; Xchup ichim qotz[15]; P'ujk'[20]; Ganabigujchii[21]
Used by (13*): Zoque[01, 02, 03]; Mixe[04]; Totonac[05]; Huastec[07]; Yucatecan Maya[09]; Quichean Maya[15]; Western Maya[18, 19, 20]; Zapotec[21]; Nahua[26]
Used for (25#): Blood[15]; Digestive[20, 26]; Cardiovascular[01]; Musculoskeletal[01, 03, 04, 05, 21]; Psychological[07]; Skin[02, 09]; Urological[01, 07]; Pregnancy[07, 19, 26]; Female genital[19, 21, 26]; Male genital[01]; General and Unspecified[02, 18, 21]
Cognates:
Language contact:

Diospyros **(Ebenaceae)**

Spanish names: Tachona; Zapote negro
Indigenous names: Nuu[03]; Sawat[05]; Suua'l[06]; Munek'[07]; Xkakalche'[09]; Sibil[09]; Ndxuuli'i[21]; Bila'huaj[21]
Used by (6*): Zoque[03]; Totonac[05, 06]; Huastec[07]; Yucatecan Maya[09]; Zapotec[21]
Used for (14#): Digestive[05, 06]; Eye[06]; Respiratory[06]; Skin[03, 09, 21]; Female genital[03]; General and Unspecified[03, 06, 07, 21]
Cognates: Tot: sawat/suua'l;
Language contact:

Diphysa **(Fabaceae)**

Spanish names: Chipilcoite; Chipile; Guachipillin; Ruda cimarron; Ruda de monte
Indigenous names: Tzus kuy [01]; Tsus cuy[03]; Tsuxp[04]; Chichath, k'anaw te, tsitsab te'[07]; Susup, Ts'us'uk[09]; Ču Čuk[10]; Ikuy[12]; Chipilin Aq'om, Uku'j[12]
Used by (8*): Zoque[01, 03]; Mixe[04]; Huastec[07]; Yucatecan Maya[09, 10]; Quichean Maya[12]; Zapotec[21]
Used for (34#): Blood[07]; Digestive[01, 03, 07, 09]; Ear[01]; Cardiovascular[12]; Musculoskeletal[12, 21]; Neurological[01, 07, 12]; Psychological[12]; Respiratory[01]; Skin[01, 03, 04, 07, 09]; Endocrine[12]; Urological[01, 07]; Pregnancy[07]; Social problems[01]; General and Unspecified[01, 03, 07, 10, 12, 21]
Cognates: MZ: tsus/tsux; Zoq: tsuskuy; Yuc: ts'us'uk/tzutzuk;
Language contact: MZ > Yuc

Disciphania **(Menispermaceae)**

Spanish names: Bejuco real, Istamoreal
Indigenous names:
Used by (1*): Western Maya[18]
Used for (1#): Digestive[18]
Cognates:
Language contact:

Discocnide **(Urticaceae)**

Spanish names: Ortiga mayor
Indigenous names: Menuk/ kenuk[01]; Balagadena, lagui[21]
Used by (2*): Zoque[01]; Zapotec[21]
Used for (3#): Musculoskeletal[01, 21]; Respiratory[21]
Cognates:
Language contact:

Disocactus **(Cactaceae)**

Spanish names:
Indigenous names: Brün-yǎl-guièts, yàg-bdzì-lbæ̀, yàg-bdzì-làs[23]
Used by (1*): Zapotec[23]
Used for (1#): General and Unspecified[23]
Cognates:
Language contact:

Dodonaea **(Sapindaceae)**

Spanish names: Jaras; Jarilla; Pimientillo
Indigenous names: Yahg žij[22]; Yàg-blàg-bîdz[23]
Used by (3*): Zoque[01]; Zapotec[22, 23]
Used for (8#): Digestive[23]; Ear[23]; Musculoskeletal[22, 23]; Pregnancy[01]; General and Unspecified[01, 22, 23]
Cognates:
Language contact:

***Dolichandra* (Bignoniaceae)**

Spanish names: Murciélago/bejuco de chinaco
Indigenous names: Täsi[01]; Oohoox ts'aah. Itsik' ocho', kuxkum ts'aah[07]
Used by (2*): Zoque[01]; Huastec[07]
Used for (3#): Cardiovascular[01]; Musculoskeletal[01]; Psychological[07]
Cognates:
Language contact:

***Doliocarpus* (Dilleniaceae)**

Spanish names:
Indigenous names: Sun tsay[03]; Xø popt aats[04]
Used by (2*): Zoque[03]; Mixe[04]
Used for (5#): Digestive[03]; Musculoskeletal[04]; Skin[04]; Female genital[03]; General and Unspecified[04]
Cognates:
Language contact:

***Dorstenia* (Moraceae)**

Spanish names: Contra hierba; Contrayerba; Cresta de gallo; Cresto de gallo; Hoja de sapo/mano de sapo/hoja de mizquino; Mal de Sapo
Indigenous names: Tzutz kin[01]; Tapu ay[02]; Nak ay[03]; K'ubak kw'a', itsal kw'a'[07]; Xkambalhaw[09]; K'opopo'il Q'ehen [14]; Xtulihom' K'opopo, K'urux Ixox[14]; Ch'up i ai[17]; Amtz'ak[19]; Yanayu'u[21]
Used by (11*): Zoque[01, 02, 03]; Huastec[07]; Yucatecan Maya[09, 11]; Quichean Maya[14, 17]; Western Maya[18, 19]; Zapotec[21]
Used for (25#): Digestive[07, 09, 11, 19, 21]; Ear[03]; Musculoskeletal[03, 07, 21]; Neurological[07]; Skin[01, 02, 03, 14, 18]; Urological[03]; Pregnancy[07, 09]; Female genital[03]; General and Unspecified[07, 14, 21]; nd[17]
Cognates:
Language contact:

***Drimys* (Winteraceae)**

Spanish names:
Indigenous names: Canela y cuyo[09]
Used by (1*): Yucatecan Maya[09]
Used for (1#): Digestive[09]
Cognates:
Language contact:

***Duranta* (Verbenaceae)**

Spanish names:
Indigenous names: K'anpokolche'[09]
Used by (1*): Yucatecan Maya[09]
Used for (1#): Skin[09]
Cognates:
Language contact:

***Dysphania* (Amaranthaceae)**

Spanish names: Apasote; Apazote de comida; Epasote; Epazote
Indigenous names: Onyuk/Tzig tuna/ unyuk[01]; Kisawa/kisyawa[02]; Epazut[03]; Podeey[04]; Sa'kalhka'jna'[05]; Sa 'kha 'kalx 'na[06]; Tihtsan[07]; Tijson[08]; Lukumxiw[09]; Sik'ij[12]; Sik'aj[13]; Cha'jen binilaj[18]; Pasujt[19]; Kakan, koko'on, kokon', kaka'an, kajk'an[20]; Biajta'[21]; Bitia'[22]; Ptiè[23]; Epazote[25]; Epazotl[28]
Used by (23*): Zoque[01, 02, 03]; Mixe[04]; Totonac[05, 06]; Huastec[07, 08]; Yucatecan Maya[09, 10, 11]; Quichean Maya[12, 13, 14]; Western Maya[18, 19, 20]; Zapotec[21, 22, 23]; Nahua[25, 26, 28]
Used for (62#): Digestive[01, 02, 03, 04, 05, 06, 07, 08, 09, 10, 11, 12, 13, 14, 18, 19, 20, 21, 23, 25, 26, 28]; Eye[21]; Cardiovascular[12]; Musculoskeletal[01, 08]; Neurological[02, 03, 12]; Psychological[13, 22]; Respiratory[02, 05, 12, 21, 28]; Skin[01, 07, 11, 12, 13, 18, 19, 20, 21, 25]; Urological[02]; Pregnancy[05, 06, 07, 12, 13, 19]; Female genital[05, 12, 14, 19]; General and Unspecified[01, 05, 08, 12, 21]
Cognates: Tot: sa'khalka'xna; Hua: ti'sVn; CoreM: k'aj; Quich: sik'Vj; Zap: bi'tia; Nah: epazotl;
Language contact: Tot > CoreM; Quich > Chiapas Zoq; Zap > Tabasco Chontal

***Dyssodia* (Asteraceae)**

Spanish names: Flor de angelina
Indigenous names: Guibgui parloshaan[21]; Gi togol kwa'č[22]
Used by (2*): Zapotec[21, 22]
Used for (4#): Digestive[21]; Psychological[22]; Skin[22]; General and Unspecified[21]
Cognates:
Language contact:

***Echeandia* (Asparagaceae)**

Spanish names:
Indigenous names: Eem ts'ohool, eem ilaal, eemil koy, thakpen thel, [07]
Used by (1*): Huastec[07]
Used for (4#): Digestive[07]; Neurological[07]; Skin[07]; Pregnancy[07]
Cognates:
Language contact:

***Echeveria* (Crassulaceae)**

Spanish names: Siempreviva
Indigenous names: Guièe-yùzh[23]
Used by (2*): Zapotec[23]; Nahua[26]
Used for (2#): Skin[23, 26]
Cognates:
Language contact:

***Echinopepon* (Cucurbitaceae)**

Spanish names: Hoja San pedro
Indigenous names: Tzuyo awit [01]; Yopo San Pedro[18]
Used by (2*): Zoque[01]; Western Maya[18]
Used for (2#): Neurological[18]; Urological[01]
Cognates:
Language contact:

***Echites* (Apocynaceae)**

Spanish names:
Indigenous names: Tsank'ub ts'aah, thuuchum ts'ohool, , thuchuum ichiich, lek'ab choc', milim ch'ohool, ch'aahil otso[07]; Sak-viperol[09]
Used by (2*): Huastec[07]; Yucatecan Maya[09]
Used for (4#): Digestive[07]; Respiratory[07]; Skin[07, 09]
Cognates:
Language contact:

***Echium* (Boraginaceae)**

Spanish names: hierba azul
Indigenous names: Tijtson ts'ojól[08]
Used by (1*): Huastec[08]
Used for (4#): Blood[08]; Musculoskeletal[08]; Skin[08]; General and Unspecified[08]
Cognates:
Language contact:

***Ehretia* (Boraginaceae)**

Spanish names: Lambimbo; Roble
Indigenous names: Thathub[07]; T'iiw te'[07]; Beeb, Xi'mche'[09]
Used by (3*): Huastec[07]; Yucatecan Maya[09]; Zapotec[21]
Used for (12#): Digestive[07, 21]; Cardiovascular[21]; Respiratory[09]; Skin[07, 21]; Pregnancy[07]; Female genital[07]; General and Unspecified[07, 09, 21]
Cognates:
Language contact:

***Eichhornia* (Pontederiaceae)**

Spanish names: Riñoncillo
Indigenous names:
Used by (1*): Zapotec[21]
Used for (2#): Digestive[21]; General and Unspecified[21]
Cognates:
Language contact:

***Elaphoglossum* (Dryopteridaceae)**

Spanish names: Hierba Dura
Indigenous names: Achi'y Q'os Aqom[12]; Rubelsa i xul[17]; Culantro pim[17]; Lùdz-mdzìn, guìzh-lùdz-mdzìn[23]
Used by (4*): Zoque[03]; Quichean Maya[12, 17]; Zapotec[23]
Used for (9#): Digestive[03, 12, 23]; Endocrine[12]; Urological[12]; Male genital[12]; General and Unspecified[12]; nd[17]
Cognates:
Language contact:

***Eleocharis* (Cyperaceae)**

Spanish names: Cintul; Cola de borrego
Indigenous names: Pap/tza'a tzoy[01]; Boo'wat toom[07]
Used by (3*): Zoque[01]; Huastec[07]; Nahua[26]
Used for (3#): Urological[26]; General and Unspecified[01, 07]
Cognates:
Language contact:

***Elephantopus* (Asteraceae)**

Spanish names: Tahuane sonjehuite
Indigenous names:
Used by (1*): Totonac[05]
Used for (1#): Skin[05]
Cognates:
Language contact:

***Eleutherine* (Iridaceae)**

Spanish names:
Indigenous names: Tsakam apats', chak apach'[07]
Used by (1*): Huastec[07]
Used for (1#): Digestive[07]
Cognates:
Language contact:

***Elytraria* (Acanthaceae)**

Spanish names: Un pie
Indigenous names: Was'an tzitz[01]; Xutsum bat'aw[07]; Kabalxa'an, Kambaxa'an[09]
Used by (3*): Zoque[01]; Huastec[07]; Yucatecan Maya[09]
Used for (4#): Digestive[09]; Ear[07]; Skin[01]; Female genital[09]
Cognates: Maya: baCa;
Language contact: Yuc > Chiapas Zoq

***Encyclia* (Orchidaceae)**

Spanish names:
Indigenous names: Xkananikte'[09]
Used by (1*): Yucatecan Maya[09]
Used for (1#): Skin[09]
Cognates:
Language contact:

***Entada* (Fabaceae)**

Spanish names: Dinero de judas, dinero del judío
Indigenous names:
Used by (1*): Totonac[05]
Used for (3#): Digestive[05]; Musculoskeletal[05]; Skin[05]
Cognates:
Language contact:

***Enterolobium* (Fabaceae)**

Spanish names: Guanacastilla; Guanacastle; Nacastle
Indigenous names: Tzang kuy [01]; Jipi[03]; Xøxy kepky[04]; Tiyow[07]; Pich[09]; Biguisha[21]
Used by (6*): Zoque[01, 03]; Mixe[04]; Huastec[07]; Yucatecan Maya[09]; Zapotec[21]
Used for (10#): Digestive[01, 03, 09]; Respiratory[01, 07]; Skin[04, 21]; General and Unspecified[09, 21]
Cognates:
Language contact:

***Epaltes* (Asteraceae)**

Spanish names: Hierba de sapo, gurusapo; Manzanilla de tres lomos; Tabaquillo, sabañon
Indigenous names: Putax ay[03]; Piix[04]; Kúz ix much[18]; Badxuumiij[21]
Used by (5*): Zoque[02, 03]; Mixe[04]; Western Maya[18]; Zapotec[21]
Used for (18#): Digestive[03]; Musculoskeletal[03, 18]; Neurological[04, 18]; Respiratory[02, 18]; Skin[03, 18, 21]; Pregnancy[21]; Female genital[02, 03, 21]; General and Unspecified[03, 04, 18, 21]
Cognates:
Language contact:

***Epidendrum* (Orchidaceae)**

Spanish names:
Indigenous names: Tsooy pathum[07]
Used by (1*): Huastec[07]
Used for (2#): Neurological[07]; General and Unspecified[07]
Cognates:
Language contact:

***Epilobium* (Onagraceae)**

Spanish names: Clavo del monte
Indigenous names: Guish claab[21]
Used by (1*): Zapotec[21]
Used for (3#): Respiratory[21]; Skin[21]; Female genital[21]
Cognates:
Language contact:

***Epiphyllum* (Cactaceae)**

Spanish names: Pitaya
Indigenous names: Nuchchi[03]; Ti'qol' bak'[14]; Chik'ba'l b'ak[17]
Used by (3*): Zoque[03]; Quichean Maya[14, 17]
Used for (6#): Digestive[03]; Musculoskeletal[14]; Skin[03]; Pregnancy[03]; Female genital[03]; nd[17]
Cognates: Quich: ik bak;
Language contact:

***Equisetum* (Equisetaceae)**

Spanish names: Cola da Caballo; Cola de caballo; Cola de caballo, boldo; Cola de caballo/cola de macho; Cola de iguana/ de caballo
Indigenous names: Cayo tuty/sus tokdong/tu tane/caballo tutz muk [01]; Tzoy muk [02]; Rujey' kej, Xpumay Aq'om[12]; Uje' kies, uje kej, tu aj, wiquab' q'ayes, rismachi koj, xul aj, tum[13]; Xye' Kawaay [14]; U nej chj[19]; Yok' es chan[20]; Shcool cavaij[21]; X-pàan ngùtsiĕts, x-côl-càbâll, guìzh-zhìg[23]
Used by (14*): Zoque[01, 02, 03]; Quichean Maya[12, 13, 14]; Western Maya[19, 20]; Zapotec[21, 23]; Nahua[25, 26, 27, 28]
Used for (43#): Blood[12, 21]; Digestive[01, 02, 12, 20, 21]; Cardiovascular[01]; Musculoskeletal[01, 13, 23]; Neurological[12]; Psychological[13, 21]; Skin[12, 13, 14, 28]; Endocrine[01, 25]; Urological[01, 02, 12, 13, 19, 21, 23, 25, 26, 27, 28]; Pregnancy[02, 03]; Female genital[01, 12, 13]; Male genital[01, 12]; General and Unspecified[12, 13, 14, 21, 28]
Cognates: Zoq: muk; Quich: uje kej;
Language contact: Quich <> Chortí

***Erechtites* (Asteraceae)**

Spanish names:
Indigenous names: Malil koy, tsakam xobo' ts'ohool, akw'aalil koy[07]
Used by (1*): Huastec[07]
Used for (1#): General and Unspecified[07]
Cognates:
Language contact:

***Eremosis* (Asteraceae)**

Spanish names: Suquinay; Suquinay, Sal Andrews
Indigenous names: Suquinay[14]; Bakil vomol[20]
Used by (3*): Quichean Maya[12, 14]; Western Maya[20]
Used for (6#): Digestive[12, 20]; Cardiovascular[12]; Respiratory[14]; Endocrine[12]; General and Unspecified[14]
Cognates:
Language contact:

***Erigeron* (Asteraceae)**

Spanish names: Hierba del burro; Margarita silvestre
Indigenous names: Ts'ojolil an churi[08]; Sal b'ey, kotz'ij b'ey[12]; Tepemalin, tepemanzanilla[26]
Used by (4*): Zoque[01]; Huastec[08]; Quichean Maya[12]; Nahua[26]
Used for (13#): Digestive[01, 08, 26]; Musculoskeletal[08]; Neurological[01, 08]; Respiratory[08, 12]; Skin[01, 08, 12]; Urological[08]; General and Unspecified[12]
Cognates:
Language contact:

01-28 refer to the studies described in Table 4.1 * Total number of studies recording this taxon #Total number of use-records

***Eriobotrya* (Rosaceae)**

Spanish names: Mispero; Nispero
Indigenous names: Yàg-nîspèrò[23]
Used by (6*): Zoque[01]; Totonac[06]; Quichean Maya[12, 13]; Zapotec[23]; Nahua[25]
Used for (21#): Digestive[01, 06, 12, 13, 23]; Cardiovascular[06]; Musculoskeletal[01, 06]; Respiratory[12, 25]; Endocrine[01, 12]; Urological[01, 06, 12, 13, 25]; Female genital[12, 13]; Male genital[01]; General and Unspecified[12]
Cognates:
Language contact:

***Eriosema* (Fabaceae)**

Spanish names:
Indigenous names: Poja sotyi[03]
Used by (1*): Zoque[03]
Used for (6#): Digestive[03]; Psychological[03]; Skin[03]; Urological[03]; Pregnancy[03]
Cognates:
Language contact:

***Eryngium* (Apiaceae)**

Spanish names: Cardo de Bosque; Cilandro ancho, culandro cimarron, culandro de monte, culandro indio, silentro de perro, espina de coche, espinabodoc, escorsoner; Cilandro cimarron; Cilandro de castill, vanero; Cilantro cimarron; Corte Santo; Culantro cimarrón; Espina blanca, escorsonera; Perejil; Perenjil, Cilantro cimarrón; Piñuela
Indigenous names: Culando[01]; Ehtiil i laab kulaantu[07]; Tsakam bathuch[07]; Q'ix juyu'[12]; Kortesanto Kix Aq'om[12]; Sak' k'ix[13]; Samat, uklanto tzé', to' k'ix[13]; Samat[17]; Yujtz' ner via'r[19]; Guièts-mél-lò, gùizh-guièts[23]
Used by (13*): Zoque[01, 03]; Mixe[04]; Huastec[07]; Quichean Maya[12, 13, 17]; Western Maya[18, 19]; Zapotec[21, 23]; Nahua[25, 26]
Used for (51#): Digestive[01, 03, 04, 13, 18, 19, 23]; Ear[03, 13]; Cardiovascular[12, 18]; Musculoskeletal[12, 13, 21]; Neurological[01, 13, 26]; Psychological[18]; Respiratory[03, 07, 18, 21]; Skin[01, 12, 13, 18, 26]; Endocrine[01, 25]; Urological[13, 23, 25]; Pregnancy[01, 03, 18]; Female genital[12, 13, 18]; Male genital[03, 12, 18]; General and Unspecified[01, 12, 21, 26]; nd[17]
Cognates: Quich: k'ix;
Language contact:

***Erythrina* (Fabaceae)**

Spanish names: Colorín, gasparo; Colorin, zempantle; Cosquelite, Colorín; Gasparo; Hierbaboracha; Madrecacao/ Flor de pita/madre; Palo cuchillo; Palo de Pito; Palo pito; Pito; Tzojantli
Indigenous names: Tzentzen[01]; Monhg ay tzäpä[02]; Tzentzen[02]; Copa tsen tsen[03]; N¥tung tsen tsen[03]; Oxexy[04]; Lagatin[05]; Ihahlni'[06]; Hutukuu', pemoch, pemuuts[07]; Tsamnek hutukuu'[07]; Chakmolonche'[09]; Tzité[12]; Mzi té[13]; Tzité[14]; Mo'te'[19]; Guixa'a mindu'u[21]; Ekimite[26]; Tzompantl[28]
Used by (16*): Zoque[01, 02, 03]; Mixe[04]; Totonac[05, 06]; Huastec[07]; Yucatecan Maya[09, 10]; Quichean Maya[12, 13, 14]; Western Maya[19]; Zapotec[21]; Nahua[26, 28]
Used for (58#): Blood[12]; Digestive[01, 02, 03, 12, 14, 26, 28]; Eye[02, 09]; Cardiovascular[14]; Musculoskeletal[12, 14]; Neurological[07, 12, 13, 19, 21]; Psychological[02, 07, 12, 14, 19, 21]; Respiratory[04, 07, 09, 12]; Skin[02, 03, 06, 12, 14]; Urological[01, 02, 09]; Pregnancy[03, 07, 19]; Female genital[03, 12, 14, 19]; General and Unspecified[03, 05, 06, 07, 10, 12, 19, 21]
Cognates: Zoq: tzentzen; Quich: tzite;
Language contact: Zoq > Quich

***Erythroxylum* (Erythroxylaceae)**

Spanish names:
Indigenous names: Xik'iche'[09]
Used by (1*): Yucatecan Maya[09]
Used for (1#): Skin[09]
Cognates:
Language contact:

Esenbeckia **(Rutaceae)**

Spanish names:
Indigenous names: Lanaax te'[07]
Used by (1*): Huastec[07]
Used for (2#): Digestive[07]; Neurological[07]
Cognates:
Language contact:

Espejoa **(Asteraceae)**

Spanish names: Maragrita del monte
Indigenous names:
Used by (1*): Zapotec[21]
Used for (1#): Skin[21]
Cognates:
Language contact:

Eucalyptus **(Myrtaceae)**

Spanish names: Alcamfor; Eucalipto; Eucalito; Eucalyptus; Euclipto; Eukalypto; Hoja de alcanfor
Indigenous names: Ts'uj[08]; Ukal, awoliy che', ok'al che'[13]; Gusha'a alcanfor, ventulatu[21]; Yàg-eùcàlîpt[23]
Used by (17*): Zoque[01, 02, 03]; Mixe[04]; Huastec[08]; Yucatecan Maya[09]; Quichean Maya[12, 13, 14]; Western Maya[18, 19]; Zapotec[21, 22, 23]; Nahua[25, 26, 27]
Used for (42#): Digestive[12, 21, 23]; Musculoskeletal[02, 12, 13, 21, 26]; Neurological[08, 13]; Psychological[12]; Respiratory[01, 02, 03, 04, 08, 09, 12, 13, 14, 18, 19, 21, 22, 23, 25, 26, 27]; Skin[12]; Endocrine[03, 18]; Pregnancy[21]; Female genital[02, 03]; General and Unspecified[03, 08, 12, 18, 21]
Cognates:
Language contact:

Eugenia **(Myrtaceae)**

Spanish names: Capulín; Cinco negrito; Escobilla
Indigenous names: Petcuy[03]; Axtaam pikx[04]; Islakastápu tamak, aka'lasni[05]; Pehte', chuk ba'im[07]; Ha' pehte', chuk ba'im, wal puchun[07]; Xhilnich', Sakloobche'[09]; Chaknii[09]; Lamush pim[17]; Yanaj'[21]
Used by (7*): Zoque[03]; Mixe[04]; Totonac[05]; Huastec[07]; Yucatecan Maya[09]; Quichean Maya[17]; Zapotec[21]
Used for (20#): Digestive[03, 04, 05, 07, 21]; Eye[09]; Respiratory[07, 21]; Skin[03, 07, 09, 21]; Endocrine[07]; Urological[07]; Pregnancy[21]; Female genital[03]; General and Unspecified[21]; nd[17]
Cognates:
Language contact: Highland Popoluca <> Hua; Mixe <> Tot

Eupatorium **(Asteraceae)**

Spanish names: Hierba buenilla; Hierba de zopilote; Prodigiosa amarilla
Indigenous names: Š haà¢[10]; Ratz'un Q'ehen[14]
Used by (4*): Yucatecan Maya[10]; Quichean Maya[14]; Zapotec[21]; Nahua[26]
Used for (8#): Digestive[21, 26]; Eye[14]; Musculoskeletal[10]; Neurological[10]; Skin[10, 26]; General and Unspecified[10]
Cognates:
Language contact:

Euphorbia (Euphorbiaceae)

Spanish names: Añadir hueso; Flor de noche buena; Fraile; Golndrina, china; Golondrina; Golondrina, riñonia; Golondrina/hoja de azar; Gordobahn; Hierba de la araña; Hierba de la araña chica; Hierba de la golondrina; Hierba de paño/hierba de antojo/hoja de azar; Hierba del gusano/ de la araña; Hierba Mala; Hoja de azar; Leche de Cabro, Quineo; Lecheria; Lechillo; Mayorga; Noche buena, flor de pascua; Nochebuena; Pie de niño; Quebra muelas/ amarra hueso; Suelda consuelda/mayorga/ hoja de elda; Tamaulipas; Zacate rojo
Indigenous names: Tza'a tzoy[01]; Pak son kuy [01]; Paipai[03]; M¥a xuxcuy[03]; Petx kukmuk[03]; Majk tekøøk tsom[04]; Atxapts[04]; Hab ichiich, leetsa ts'ohool, puunchiix wits, alwa' akal ch'ohool, i'ixte, ch'awil ch'ohool[07]; Leetsa ts'ohool[07]; Xaa' uts'aal, tsakam nuk'ats ts'ohool[07]; T'ot'oy ts'ohool, tsakam ts'uh[07]; Ehtiil mithith, bathuch ts'ohool, ik ts'ohool, thootil tsan pulik, ts'uleel thekw'eel, t'apay ts'ohool[07]; Xa' ts'ohool, xaa' uts'aal pulik, t'ithith i koy, pakw'[07]; Leetsa ts'ohool tsakni', k'apwaal book', puxek' ts'ohool, [07]; Oot' wits, k'alul wits[07]; Akan t'ele', chk'te', akan chakam, tsukte'[07]; Sakchakah, Sibik'[09]; Xanabmukuy[09]; Nabalche', Nahualte[09]; Much'kok[09]; Kambalchakah[09]; Hobonk'ak'[09]; Ya'axhalalche'[09]; Yook mukuy[10]; Hobon Čokoh[10]; Ix but[11]; Orob[12]; Ixbut[13]; Mansaana Q'ehen[14]; X'lee'chi'yuk, Tul[14]; Xtzin' iyuk[14]; Ixgolondrina[18]; Guish mbsia'a[21]; Guie chien[21]; Nia'badu'u[21]; Bini[22]; Škwan gurak[22]; Guìzh-biè, guìzh-xlǚtsí, guìzh-lìndrîn, guìzh-zhìp, zhìp-nquǐts[23]; Còrdòbân[23]; Pilicxitl[24]; Custicpascua xochitl[28]
Used by (21*): Zoque[01, 02, 03]; Mixe[04]; Huastec[07]; Yucatecan Maya[09, 10, 11]; Quichean Maya[12, 13, 14, 17]; Western Maya[18, 19]; Zapotec[21, 22, 23]; Nahua[24, 25, 26, 28]
Used for (116#): Digestive[01, 02, 03, 04, 07, 14, 21, 22]; Eye[03, 09, 10, 23]; Ear[03, 07]; Cardiovascular[02, 07, 14]; Musculoskeletal[01, 07, 18, 19, 21, 23]; Neurological[07, 09, 21, 23]; Psychological[01, 07]; Respiratory[02, 03, 07, 09, 14, 28]; Skin[01, 02, 03, 07, 09, 12, 13, 14, 18, 21, 25, 26]; Endocrine[02, 25]; Urological[02, 03, 18, 21]; Pregnancy[01, 07, 11, 13, 24]; Female genital[01, 02, 03, 04, 07, 14, 25, 28]; Male genital[11]; General and Unspecified[01, 02, 03, 04, 07, 10, 21, 22, 25]; nd[17]
Cognates: Yuc: mukuy, hobon k'ak/cok; Zap: bini/bie/chien?;
Language contact: Hua <> Yuc; Yuc <> Quich; Hua > Highland Popoluca; Chiapas Zoq <> Hua t

Eustoma (Gentianaceae)

Spanish names:
Indigenous names: Bioleta[07]; Ka'paj[14]
Used by (2*): Huastec[07]; Quichean Maya[14]
Used for (3#): Digestive[14]; General and Unspecified[07, 14]
Cognates:
Language contact:

Evolvulus sp. (Convolvulaceae)

Spanish names:
Indigenous names: Kok' centaviwil pim [14]
Used by (1*): Quichean Maya[14]
Used for (1#): General and Unspecified[14]
Cognates:
Language contact:

Exostema (Rubiaceae)

Spanish names: Chichipactli; Nazareno amargo; Quina; Quina, Hombre Grande
Indigenous names: Tzapas kuy[02]; Tam nazareno[03]; Saqi che'[14]
Used by (4*): Zoque[02, 03]; Quichean Maya[12, 14]
Used for (10#): Blood[03]; Digestive[02, 03]; Skin[03]; Endocrine[02, 14]; Pregnancy[02, 03]; Female genital[03]; General and Unspecified[12]
Cognates:
Language contact:

Exothea (Sapindaceae)

Spanish names:
Indigenous names: T'il homte', t'il hom, bichaam te'[07]
Used by (1*): Huastec[07]
Used for (2#): Neurological[07]; General and Unspecified[07]
Cognates:
Language contact:

Eysenhardtia **(Fabaceae)**

Spanish names: Cuatle, jocotil; Taray
Indigenous names: Oku'ku piake[01]; Chilab, chakam wayal[07]; Much' Che'[12]; Yàg-guièe-guiâ[23]
Used by (4*): Zoque[01]; Huastec[07]; Quichean Maya[12]; Zapotec[23]
Used for (15#): Blood[12]; Digestive[01, 12, 23]; Cardiovascular[01]; Endocrine[01, 12]; Urological[01, 07, 12]; Female genital[01, 12]; Male genital[01]; General and Unspecified[07, 12]
Cognates:
Language contact:

Fernaldia **(Apocynaceae)**

Spanish names:
Indigenous names: T'obs'i', t'obon, kuhuw akw'aal, topts'i'[07]
Used by (1*): Huastec[07]
Used for (3#): Digestive[07]; Endocrine[07]; General and Unspecified[07]
Cognates:
Language contact:

Ferocactus **(Cactaceae)**

Spanish names: Biznaga
Indigenous names:
Used by (1*): Nahua[27]
Used for (1#): Urological[27]
Cognates:
Language contact:

Fevillea **(Cucurbitaceae)**

Spanish names: Chichimora
Indigenous names:
Used by (1*): Western Maya[19]
Used for (1#): Digestive[19]
Cognates:
Language contact:

Ficus **(Moraceae)**

Spanish names: Alamo; Amate; Amate grande/orejón; Higo; Higo, Higuerillo / Higuera; Higuero
Indigenous names: Tzaman/jitsi[01]; Tsui[03]; Oom[04]; Noak[04]; Hopoy ts'uh[07]; Tsakam ts'uh[07]; Hopoy, ts'uh[07]; Kopo'[09]; Wix[12]; Xq'oli hu [14]; Hu'u che'[17]; Jun, ma'n jun, chu jun[19]; Dxuumii[21]
Used by (11*): Zoque[01, 03]; Mixe[04]; Huastec[07]; Yucatecan Maya[09]; Quichean Maya[12, 13, 14, 17]; Western Maya[19]; Zapotec[21]
Used for (35#): Digestive[04, 21]; Cardiovascular[12, 21]; Musculoskeletal[07, 12, 13, 21]; Neurological[07, 14]; Psychological[21]; Respiratory[07, 09, 12, 21]; Skin[01, 07, 14, 19, 21]; Female genital[03]; General and Unspecified[03, 04, 07, 12, 14, 21]; nd[17]
Cognates: Quich: hu;
Language contact: Highalnd Popoluca <> Hua & Zap; Quich <> Chortí

Flaveria **(Asteraceae)**

Spanish names: Hierba del sapo; Popote
Indigenous names:
Used by (1*): Nahua[27]
Used for (3#): Digestive[27]; Skin[27]
Cognates:
Language contact:

01-28 refer to the studies described in Table 4.1 * Total number of studies recording this taxon #Total number of use-records

***Fleischmannia* (Asteraceae)**

Spanish names: Flor de cristalina; Mejorana
Indigenous names:
Used by (2*): Zoque[01]; Quichean Maya[12]
Used for (9#): Digestive[12]; Eye[01]; Musculoskeletal[12]; Neurological[12]; Respiratory[12]; Endocrine[12]; Urological[12]; Female genital[12]; General and Unspecified[12]
Cognates:
Language contact:

***Flourensia* (Asteraceae)**

Spanish names: Hoja sen
Indigenous names:
Used by (1*): Zapotec[22]
Used for (1#): Digestive[22]
Cognates:
Language contact:

***Foeniculum* (Apiaceae)**

Spanish names: Hinojo
Indigenous names: Diojo xuitl[08]; Anix[13]; Inajo, inaja, injo[20]; Guìzh-hìnôj, mîlt-ró-yù[23]; Diojo xuitl[28]
Used by (9*): Zoque[01]; Huastec[08]; Quichean Maya[12, 13]; Western Maya[20]; Zapotec[23]; Nahua[25, 26, 28]
Used for (24#): Digestive[01, 08, 12, 13, 20, 23, 26, 28]; Ear[20]; Cardiovascular[12]; Neurological[20]; Psychological[01, 13]; Respiratory[01, 12, 20, 25, 28]; Skin[08]; Endocrine[12]; Pregnancy[12, 13]; Male genital[12]; General and Unspecified[13]
Cognates:
Language contact:

***Forchhammeria* (Capparaceae)**

Spanish names:
Indigenous names:
Used by (1*): Quichean Maya[14]
Used for (2#): Neurological[14]; General and Unspecified[14]
Cognates:
Language contact:

***Fosterella* (Bromeliaceae)**

Spanish names:
Indigenous names:
Used by (1*): Zoque[03]
Used for (1#): General and Unspecified[03]
Cognates:
Language contact:

***Fouquieria* (Fouquieriaceae)**

Spanish names:
Indigenous names: Yàg-guièts-zhìg, yàg-guièe-zhìg[23]
Used by (1*): Zapotec[23]
Used for (1#): Skin[23]
Cognates:
Language contact:

Frangula **(Rhamnaceae)**

Spanish names: Cáscara sagrada
Indigenous names:
Used by (2*): Zoque[02]; Huastec[08]
Used for (3#): Digestive[02, 08]; Skin[08]
Cognates:
Language contact:

Fraxinus **(Oleaceae)**

Spanish names: Fresno
Indigenous names: Yàg-frêsn[23]
Used by (3*): Zapotec[22, 23]; Nahua[26]
Used for (8#): Digestive[22, 23]; Psychological[22]; Skin[22]; Endocrine[26]; Pregnancy[22]; General and Unspecified[23, 26]
Cognates:
Language contact:

Fridericia **(Bignoniaceae)**

Spanish names:
Indigenous names: Pobatsay[03]; Ka'ax aats[04]; Sak-ak'[09]
Used by (3*): Zoque[03]; Mixe[04]; Yucatecan Maya[09]
Used for (3#): Skin[04, 09]; Female genital[03]
Cognates: MZ: ats;
Language contact:

Fuchsia **(Onagraceae)**

Spanish names: Fuchsia
Indigenous names: Patayuc[13]; Sera nich vomol, tzajal kampana nichim, marabiya wamal, paj nich wamal, bik'tal rimon, batz' momol, , max te' momol, lobol ch'o, kenya jomol[20]
Used by (3*): Quichean Maya[12, 13]; Western Maya[20]
Used for (5#): Digestive[20]; Respiratory[20]; Skin[13]; General and Unspecified[12, 20]
Cognates:
Language contact:

Galactia **(Fabaceae)**

Spanish names: Pegapega
Indigenous names:
Used by (1*): Yucatecan Maya[11]
Used for (1#): Urological[11]
Cognates:
Language contact:

Galeana **(Asteraceae)**

Spanish names:
Indigenous names: Tsukt aay[04]
Used by (1*): Mixe[04]
Used for (1#): Pregnancy[04]
Cognates:
Language contact:

***Galinsoga* (Asteraceae)**

Spanish names: San Nicolas
Indigenous names: Guìzh-guièe-lêch, guièe-bnîil-làs, guièe-sàntàmàrǐ-làs, xín-guièe-sàntàmàrǐ, guìzh-guièe-nquǐts[23]
Used by (2*): Quichean Maya[12]; Zapotec[23]
Used for (2#): Digestive[23]; Neurological[12]
Cognates:
Language contact:

***Galium* (Rubiaceae)**

Spanish names: Pegarropa
Indigenous names: Guìzh-ngùdzìi, guìzh-nàad[23]
Used by (3*): Quichean Maya[13]; Zapotec[23]; Nahua[25]
Used for (3#): Ear[23]; Neurological[13]; Pregnancy[25]
Cognates:
Language contact:

***Galphimia* (Malpighiaceae)**

Spanish names:
Indigenous names: Tsalaam kubi', it'il chuch[07]; Guìzh-ncuàan-bzhiân, ncuàan-bzhiân[23]
Used by (2*): Huastec[07]; Zapotec[23]
Used for (2#): Psychological[23]; Skin[07]
Cognates:
Language contact:

***Garcia* (Euphorbiaceae)**

Spanish names:
Indigenous names: Thokb ot'el[07]
Used by (1*): Huastec[07]
Used for (1#): Psychological[07]
Cognates:
Language contact:

***Garcinia* (Clusiaceae)**

Spanish names:
Indigenous names: Uouo[03]
Used by (1*): Zoque[03]
Used for (3#): Eye[03]; Skin[03]; Female genital[03]
Cognates:
Language contact:

***Gardenia* (Rubiaceae)**

Spanish names: Gardenia
Indigenous names:
Used by (3*): Zoque[02, 03]; Nahua[26]
Used for (4#): Digestive[03]; Cardiovascular[02]; Musculoskeletal[03]; Respiratory[26]
Cognates:
Language contact:

***Gaudichaudia* (Malpighiaceae)**

Spanish names: Hoja de culebra
Indigenous names: Tzajin poj[02]
Used by (1*): Zoque[02]
Used for (1#): Musculoskeletal[02]
Cognates:
Language contact:

***Gaultheria* (Ericaceae)**

Spanish names: Axocopac
Indigenous names: Toka tzasa[01]; Tecolx'ma'palxch'ma[06]; Atres wamal[20]; Axocopa[26]
Used by (4*): Zoque[01]; Totonac[06]; Western Maya[20]; Nahua[26]
Used for (7#): Digestive[20]; Musculoskeletal[01]; Respiratory[06]; Pregnancy[01]; Female genital[01]; General and Unspecified[06, 26]
Cognates:
Language contact:

***Gaya* (Malvaceae)**

Spanish names:
Indigenous names: Xpupul-ik'[09]
Used by (1*): Yucatecan Maya[09]
Used for (2#): Digestive[09]; Skin[09]
Cognates:
Language contact:

***Genipa* (Rubiaceae)**

Spanish names: Maluku
Indigenous names: Nuk t¥m[03]
Used by (2*): Zoque[02, 03]
Used for (2#): Digestive[02]; General and Unspecified[03]
Cognates:
Language contact:

***Geophila* (Rubiaceae)**

Spanish names:
Indigenous names: Ixim Q'en[14]
Used by (1*): Quichean Maya[14]
Used for (1#): Digestive[14]
Cognates:
Language contact:

***Geranium* (Geraniaceae)**

Spanish names: Sanícula
Indigenous names:
Used by (1*): Quichean Maya[12]
Used for (4#): Eye[12]; Skin[12]; Urological[12]; General and Unspecified[12]
Cognates:
Language contact:

***Gibasis* (Commelinaceae)**

Spanish names:
Indigenous names: Xwa ixul[14]; Madali[21]
Used by (2*): Quichean Maya[14]; Zapotec[21]
Used for (2#): Skin[14, 21]
Cognates:
Language contact:

***Ginkgo* (Ginkgoaceae)**

Spanish names: Gingko Biloba
Indigenous names:
Used by (1*): Quichean Maya[12]
Used for (4#): Cardiovascular[12]; Musculoskeletal[12]; Neurological[12]; Psychological[12]
Cognates:
Language contact:

***Gladiolus* (Iridaceae)**

Spanish names: Gladiola
Indigenous names:
Used by (1*): Quichean Maya[12]
Used for (2#): Skin[12]; General and Unspecified[12]
Cognates:
Language contact:

***Glandularia* (Verbenaceae)**

Spanish names: Alfombrilla cimarrón
Indigenous names: Waleklaab ts'ohool, ehek walek, tsamnek ts'ohool, thuhal ts'ohool, thuhual ichich[07]; Ncuàan-dzéb-cônch, guìzh-cônch, guìzh-ncuàan-x-cônch[23]
Used by (3*): Huastec[07]; Zapotec[23]; Nahua[26]
Used for (4#): Skin[07, 26]; General and Unspecified[07, 23]
Cognates:
Language contact:

***Gliricidia* (Fabaceae)**

Spanish names: Cacao de nance, madrecacao; Cocohuite; Cocohuite/mataratón; Cocoite; Cocuite; Madre de Cacao; Madrecacao; Sacahuite
Indigenous names: Tzang kuy/oyo nuk/sawin kuy[01]; Tzawin kuy/ jäyä kuy[02]; Paaki[03]; Tsøøk[04]; Zakh'akh'a tasun[08]; Kante'[14]; Aj chänte'[18]; K'an te[19]; Guianixa'a[21]
Used by (10*): Zoque[01, 02, 03]; Mixe[04]; Totonac[05]; Huastec[08]; Quichean Maya[14]; Western Maya[18, 19]; Zapotec[21]
Used for (26#): Digestive[01, 02]; Eye[18]; Neurological[01, 02, 03, 19]; Psychological[21]; Respiratory[05, 08, 18]; Skin[02, 03, 19, 21]; Urological[02, 21]; General and Unspecified[01, 02, 03, 04, 05, 14, 18, 19, 21]
Cognates: Zoq: sawin kuy; CoreM: kante;
Language contact:

***Gnaphalium* (Asteraceae)**

Spanish names: Algodoncito; Gordolobo; Gordolobo macho; Gordolobo, flor de seda, sanalotodo; Gordolobo, Sanalotodo
Indigenous names: Poma ay cimarrón[03]; Wo'opt aay[04]; B'ojq'aos[12]; Saq' Mak'el Q'os[12]; Uxkin imul, uxquin imul[13]; Tafiatu gueexii[21]; Ixcaxihuitl[26]
Used by (6*): Zoque[03]; Mixe[04]; Quichean Maya[12, 13]; Zapotec[21]; Nahua[26]
Used for (25#): Digestive[04, 12, 21]; Neurological[12]; Respiratory[03, 12, 13, 21]; Skin[03, 12, 13, 21, 26]; Pregnancy[03, 21]; Female genital[12]; General and Unspecified[03, 12, 21]
Cognates:
Language contact:

***Godmania* (Bignoniaceae)**

Spanish names:
Indigenous names: Xo'k'ab[09]
Used by (1*): Yucatecan Maya[09]
Used for (2#): Pregnancy[09]; Female genital[09]
Cognates:
Language contact:

***Gomphrena* (Amaranthaceae)**

Spanish names: Amor Secco; Tinta roja
Indigenous names: J¥pak mooya[03]; T'oyol[07]; Pitunche[12]; Ku' qehen[14]; Gueyana[21]; Guìzh-guiĕt-nì[23]
Used by (6*): Zoque[03]; Huastec[07]; Quichean Maya[12, 14]; Zapotec[21, 23]
Used for (14#): Digestive[07, 14, 21, 23]; Psychological[12]; Urological[03, 23]; Pregnancy[03, 12]; Female genital[03]; General and Unspecified[03, 12, 21]
Cognates:
Language contact:

***Goniophlebium* (Polypodiaceae)**

Spanish names: Doradilla
Indigenous names:
Used by (1*): Zoque[01]
Used for (1#): General and Unspecified[01]
Cognates:
Language contact:

***Gonolobus* (Apocynaceae)**

Spanish names: Candua; Yoyos
Indigenous names: Kontua[02]; U'ii[06]; Ooy[07]; Batuguexe'e[21]
Used by (4*): Zoque[02]; Totonac[06]; Huastec[07]; Zapotec[21]
Used for (5#): Digestive[06]; Skin[07]; Female genital[02]; General and Unspecified[02, 21]
Cognates:
Language contact: Tot <> Hua

***Gonzalagunia* (Rubiaceae)**

Spanish names:
Indigenous names: Tzu'ul che[16]; Tzuul che'[17]
Used by (2*): Quichean Maya[16, 17]
Used for (2#): Neurological[16]; nd[17]
Cognates:
Language contact:

***Gossypium* (Malvaceae)**

Spanish names: Algodón; Algodon gris
Indigenous names: Kwinim, tsokoy[07]; Taman[09]; Chuy-taman[09]; Taman[10]; Noq'[14]; Shiila'a[21]; Yàg-zhĭil[23]
Used by (7*): Zoque[01]; Huastec[07]; Yucatecan Maya[09, 10]; Quichean Maya[14]; Zapotec[21, 23]
Used for (17#): Digestive[07, 21]; Musculoskeletal[07, 21]; Respiratory[07, 09, 10, 14]; Skin[07, 21]; Urological[01, 07, 21]; Pregnancy[07]; General and Unspecified[21, 23]
Cognates: Yuc: taman; Zap: xiil;
Language contact:

Gouania **(Rhamnaceae)**

Spanish names: Jaboncillo liso
Indigenous names: Tzaku pu/Tzaj upu [01]; Xiapun tsay[03]; Xajts oo'ts aats[04]; Thuhal ts'aah[07]; X-om-ak'[09]; Ch'jom k'aham[17]
Used by (6*): Zoque[01, 03]; Mixe[04]; Huastec[07]; Yucatecan Maya[09]; Quichean Maya[17]
Used for (11#): Musculoskeletal[03]; Skin[01, 03, 04, 07, 09]; Female genital[03]; General and Unspecified[03, 07]; nd[17]
Cognates: MZ: tsaj/tsats;
Language contact: MZ > Hua

Govenia **(Orchidaceae)**

Spanish names: Flor blanca
Indigenous names: Popoj jäyä[01]
Used by (1*): Zoque[01]
Used for (1#): Digestive[01]
Cognates:
Language contact:

Grevillea **(Proteaceae)**

Spanish names: Grabilea
Indigenous names:
Used by (1*): Quichean Maya[12]
Used for (1#): Psychological[12]
Cognates:
Language contact:

Gronovia **(Loasaceae)**

Spanish names: Chaya
Indigenous names: Tzis kä wang [02]; Laalmuch[09]
Used by (2*): Zoque[02]; Yucatecan Maya[09]
Used for (2#): Musculoskeletal[09]; Neurological[02]
Cognates:
Language contact:

Guadua **(Poaceae)**

Spanish names:
Indigenous names: Tsahib, chahib[07]
Used by (1*): Huastec[07]
Used for (4#): Musculoskeletal[07]; Skin[07]; Pregnancy[07]; General and Unspecified[07]
Cognates:
Language contact:

Guaiacum **(Zygophyllaceae)**

Spanish names: Balsamo; Guayacan
Indigenous names: Hueycán[08]
Used by (2*): Huastec[08]; Zapotec[21]
Used for (3#): Skin[21]; Endocrine[08]; General and Unspecified[21]
Cognates:
Language contact:

Guarea **(Meliaceae)**

Spanish names:
Indigenous names: Bolb[16]
Used by (1*): Quichean Maya[16]
Used for (1#): General and Unspecified[16]
Cognates:
Language contact:

Guazuma **(Malvaceae)**

Spanish names: Caulote; Caulote/tapaculo; Guacima; Guácimo; Guazimo
Indigenous names: Topaku/tzapas äkö/äkä[01]; Äkä[02]; ¥k¥[03]; Øøk[04]; Akgexta[06]; Akich[07]; Pixoy, Nohoch-pixoy[09]; Pixoy[11]; Tchabei[14]; Ajxuyuy[18]; Ch'ab'ay[19]; Yana'a[21]
Used by (12*): Zoque[01, 02, 03]; Mixe[04]; Totonac[06]; Huastec[07]; Yucatecan Maya[09, 11]; Quichean Maya[14]; Western Maya[18, 19]; Zapotec[21]
Used for (34#): Blood[03]; Digestive[01, 02, 03, 04, 06, 07, 11, 14, 18, 19, 21]; Cardiovascular[18]; Neurological[21]; Psychological[18]; Respiratory[02, 03, 07]; Skin[02, 03, 07, 18]; Endocrine[03]; Pregnancy[03, 07, 09]; Female genital[02, 03, 04]; Male genital[01]; General and Unspecified[02, 03, 07, 21]
Cognates: MZ: äk(ä); Zoq: äkä; CoreM: chabai; Yuc: pixoy;
Language contact: MZ > Tot & Hua

Gurania **(Cucurbitaceae)**

Spanish names:
Indigenous names: Susum Sankh[14]; K'uum pim[17]
Used by (2*): Quichean Maya[14, 17]
Used for (2#): General and Unspecified[14]nd[17]
Cognates:
Language contact:

Gymnopodium **(Polygonaceae)**

Spanish names:
Indigenous names: Ts'its'ilche'[09]
Used by (1*): Yucatecan Maya[09]
Used for (1#): Respiratory[09]
Cognates:
Language contact:

Gynerium **(Poaceae)**

Spanish names: Caña brava
Indigenous names:
Used by (1*): Yucatecan Maya[10]
Used for (2#): Urological[10]; Female genital[10]
Cognates:
Language contact:

Gypsacanthus **(Acanthaceae)**

Spanish names: Tiricia
Indigenous names:
Used by (1*): Nahua[27]
Used for (1#): General and Unspecified[27]
Cognates:
Language contact:

01-28 refer to the studies described in Table 4.1 * Total number of studies recording this taxon #Total number of use-records

***Gyrotaenia* (Urticaceae)**

Spanish names: Mala mujer
Indigenous names: Si'in geno[01]
Used by (1*): Zoque[01]
Used for (1#): Skin[01]
Cognates:
Language contact:

***Haematoxylum* (Fabaceae)**

Spanish names: Brasil; Palo brasil; Tinto
Indigenous names: Chäkte'[18]
Used by (3*): Zoque[01]; Western Maya[18]; Zapotec[21]
Used for (7#): Blood[21]; Digestive[18, 21]; Endocrine[01]; Urological[01]; General and Unspecified[18, 21]
Cognates:
Language contact:

***Hamelia* (Rubiaceae)**

Spanish names: Baletilla; Canserina del monte; Chacloco; Chichipín; Coralio; Coyolillo, Cosocoyole, Chupacoyole; Madura zapote
Indigenous names: Tondon ay/ tzan ay/ tzan jäyä[01]; Chochoday, Cangchocho, Cuma ay[03]; Maktantulonkis, akajetantulungush, tanchulukx kiui[05]; Tsak look', k'entsul te', chak ch'ohool, chak look'[07]; K'entsel te'[08]; Ele'kabi, K'anan[09]; Šk'anang[10]; Ik k'änän[11]; Saq'ib Q'ehen[14]; Ratzum Tz'unun, Ruk'a Tz'unun[14]; Chaj' mash[16]; Cacahuaxochitl[24]
Used by (15*): Zoque[01, 02, 03]; Totonac[05]; Huastec[07, 08]; Yucatecan Maya[09, 10, 11]; Quichean Maya[12, 14, 16]; Zapotec[21]; Nahua[24, 25]
Used for (51#): Blood[05, 07, 08]; Digestive[01, 05, 07, 08, 12, 24, 25]; Eye[03]; Musculoskeletal[01, 05]; Neurological[05, 16]; Psychological[12]; Respiratory[01, 05, 14]; Skin[01, 03, 05, 07, 08, 09, 10, 11, 14, 21, 24, 25]; Endocrine[03, 05, 08, 12]; Urological[02, 07, 25]; Pregnancy[24]; Female genital[03, 05, 14, 21, 24]; General and Unspecified[01, 03, 05, 07, 12, 14]
Cognates: Hua: kentsVl; Yuc: k'anan;
Language contact: Chiapas Zoq <> Hua <> Kekchí

***Hampea* (Malvaceae)**

Spanish names: Majahua
Indigenous names: Tsäkolte'[18]
Used by (1*): Western Maya[18]
Used for (3#): Digestive[18]; Neurological[18]; General and Unspecified[18]
Cognates:
Language contact:

***Hanburia* (Cucurbitaceae)**

Spanish names:
Indigenous names: Wawi yomdak [01]
Used by (1*): Zoque[01]
Used for (1#): Skin[01]
Cognates:
Language contact:

***Handroanthus* (Bignoniaceae)**

Spanish names: Guayacán; Roble
Indigenous names: B'ajeer[14]; Uayakan[18]
Used by (4*): Zoque[01]; Quichean Maya[14]; Western Maya[18]; Zapotec[21]
Used for (5#): Digestive[14]; Musculoskeletal[21]; Skin[21]; Endocrine[01, 18]
Cognates:
Language contact:

***Harpalyce* (Fabaceae)**

Spanish names:
Indigenous names: K'ante'[07]
Used by (1*): Huastec[07]
Used for (1#): Digestive[07]
Cognates:
Language contact:

***Havardia* (Fabaceae)**

Spanish names:
Indigenous names: Chukum[09]
Used by (1*): Yucatecan Maya[09]
Used for (1#): Skin[09]
Cognates:
Language contact:

***Hebanthe* (Amaranthaceae)**

Spanish names:
Indigenous names: K¥kujuki ay[03]
Used by (1*): Zoque[03]
Used for (1#): Skin[03]
Cognates:
Language contact:

***Hechtia* (Bromeliaceae)**

Spanish names: Lasolmic
Indigenous names: Laxomic[28]
Used by (1*): Nahua[28]
Used for (1#): Digestive[28]
Cognates:
Language contact:

***Hedeoma* (Lamiaceae)**

Spanish names:
Indigenous names: Maal t'eel, tsakam polello[07]
Used by (1*): Huastec[07]
Used for (4#): Digestive[07]; Respiratory[07]; Pregnancy[07]; General and Unspecified[07]
Cognates:
Language contact:

***Hedychium* (Zingiberaceae)**

Spanish names: Mariposa
Indigenous names:
Used by (1*): Zoque[01]
Used for (3#): Musculoskeletal[01]; Skin[01]; General and Unspecified[01]
Cognates:
Language contact:

***Heimia* (Lythraceae)**

Spanish names: Sinicuichi; Sobadora
Indigenous names: Maan witsiil[07]; Ncuàan-yǎas[23]; Icxinicuiltzin[26]
Used by (4*): Huastec[07]; Zapotec[21, 23]; Nahua[26]
Used for (8#): Digestive[26]; Musculoskeletal[21, 23, 26]; Neurological[26]; Skin[23]; General and Unspecified[07, 23]
Cognates:
Language contact:

***Helenium* (Asteraceae)**

Spanish names: Floricilla
Indigenous names: Hats'ix wits, hats'ix kw'eet, chemthak i ch'ak[07]; Guièe-sàntàmàrǐ-mòntês, guièe-sàntàmàrǐ-dán, guièe-sàntàmàrǐ-nguěts, guièe-sàntàmàrǐ-gùts, guièe-sàntàmàrǐ-mòstêz[23]
Used by (3*): Huastec[07]; Quichean Maya[13]; Zapotec[23]
Used for (3#): Respiratory[13, 23]; Skin[07]
Cognates:
Language contact:

***Helianthemum* (Cistaceae)**

Spanish names: Cenicero
Indigenous names: Xmasakh'e pak'a[08]; Vach' t'ul, tan vomol, tantan wamal, tan bak wamal, k'anal nich te', k'anal nich wach', vach' te' vomol, k'ujk'ul wamal[20]; Gbày-tǽ, guìzh-òrêgànò-dán[23]
Used by (3*): Huastec[08]; Western Maya[20]; Zapotec[23]
Used for (12#): Digestive[08, 20]; Eye[20]; Ear[20]; Musculoskeletal[08, 20]; Respiratory[20]; Skin[20, 23]; Male genital[20]; General and Unspecified[20, 23]
Cognates:
Language contact:

***Heliconia* (Heliconiaceae)**

Spanish names: Calambre Negro ; Platanillo
Indigenous names: Tsabats ay[03]; Lištampan[05]; Ts'umts'um, thulup[07]; Q'eq'i mox Kej [14]; Huahua[21]
Used by (5*): Zoque[03]; Totonac[05]; Huastec[07]; Quichean Maya[14]; Zapotec[21]
Used for (9#): Digestive[14]; Musculoskeletal[14]; Urological[03]; Female genital[03, 07]; General and Unspecified[05, 21]
Cognates:
Language contact:

***Helicteres* (Malvaceae)**

Spanish names:
Indigenous names: Ang Tzatz[02]; Piniaka[03]; Tsutup, Suput[09]
Used by (3*): Zoque[02, 03]; Yucatecan Maya[09]
Used for (5#): Neurological[03]; Psychological[02]; Skin[09]; Pregnancy[03]; Female genital[03]
Cognates:
Language contact:

***Heliocarpus* (Malvaceae)**

Spanish names: Jonote; Majagua; Majagua caballin; Majagua rojo; Palo de majagua
Indigenous names: (Tzapas) po'a[01]; Po'a[02]; Panats[03]; Pa'ants[04]; Xunic[05]; Baat[07]; Kaq'i B'ach[14]; Lajsa'a boogui[21]; Yaga lajsa'a[21]; Lajtz[21]; Ixhuaqué[24]
Used by (9*): Zoque[01, 02, 03]; Mixe[04]; Totonac[05]; Huastec[07]; Quichean Maya[14]; Zapotec[21]; Nahua[24]
Used for (26#): Digestive[03, 05, 07, 21]; Musculoskeletal[21]; Respiratory[07]; Skin[01, 02, 03, 04, 05, 07, 21]; Urological[03, 14]; Pregnancy[03, 05, 07, 14, 24]; General and Unspecified[01, 07, 14, 21]
Cognates: MZ: panats/pa'ants; Zoq: po'a; Maya: baat/b'ach;
Language contact: MZ <> Maya

Heliopsis (Asteraceae)

Spanish names:
Indigenous names: Mansaana Q'ehen[14]
Used by (1*): Quichean Maya[14]
Used for (1#): General and Unspecified[14]
Cognates:
Language contact:

Heliotropium (Boraginaceae)

Spanish names: Cabeza de guajalote blanco; Cola de alacrán; Cola de alacran, hoja de guajalote; Cola de alacrán/riñonina; Hoja de alacrán del monte; Hoja de alacrán/cola de alacran; Rabo de mico; Rabomico
Indigenous names: Kaku'e tuts [01]; Kaku'e ay[02]; Ma'ne' kinä/kaku'we ay[02]; Tunok kiñi[03]; No'ot ujts[04]; Thiniy ts'ohool, weew thiniy[07]; Xnema'ax[09]; Nej ajpum[18]; Guixamberu'u quitzii[21]; Guixamberu'u [21]
Used by (10*): Zoque[01, 02, 03]; Mixe[04]; Huastec[07]; Yucatecan Maya[09, 11]; Quichean Maya[14]; Western Maya[18]; Zapotec[21]
Used for (36#): Digestive[01, 02, 03, 09, 11, 18, 21]; Ear[03]; Cardiovascular[02]; Musculoskeletal[02, 03]; Neurological[07]; Skin[01, 02, 03, 07, 18, 21]; Urological[01, 02, 03, 14]; Pregnancy[02, 07]; Female genital[02, 03, 04, 21]; Male genital[02]; General and Unspecified[21]
Cognates: Zoq: kinV, kaku'e;
Language contact: Zoq > Hua

Hemionitis (Pteridaceae)

Spanish names: Hierba del gato
Indigenous names: Guish misht'daj'[21]
Used by (1*): Zapotec[21]
Used for (2#): Skin[21]; General and Unspecified[21]
Cognates:
Language contact:

Henriettella (Melastomataceae)

Spanish names:
Indigenous names: Ixq Q'een[15]
Used by (1*): Quichean Maya[15]
Used for (1#): Pregnancy[15]
Cognates:
Language contact:

Herissantia (Malvaceae)

Spanish names:
Indigenous names:
Used by (1*): Huastec[07]
Used for (1#): Urological[07]
Cognates:
Language contact:

Heterocentron (Melastomataceae)

Spanish names: Caña agria; Caña de Cristo
Indigenous names: Tzapas mankuy yajkuy[02]; Chäm ajij[12]
Used by (2*): Zoque[02]; Quichean Maya[12]
Used for (11#): Blood[12]; Digestive[12]; Cardiovascular[12]; Musculoskeletal[12]; Respiratory[12]; Skin[02]; Endocrine[02, 12]; Urological[02]; Female genital[12]; General and Unspecified[12]
Cognates:
Language contact:

***Heteropterys* (Malpighiaceae)**

Spanish names:
Indigenous names: Patan tsay, Tsay uouo, T¥ts kunekne[03]; Meeme ay[03]
Used by (1*): Zoque[03]
Used for (6#): Digestive[03]; Urological[03]; Female genital[03]
Cognates:
Language contact:

***Heterotheca* (Asteraceae)**

Spanish names: Arnica
Indigenous names:
Used by (2*): Nahua[25, 26]
Used for (7#): Digestive[25]; Musculoskeletal[25, 26]; Skin[25, 26]; Urological[25]; General and Unspecified[25]
Cognates:
Language contact:

***Hevea* (Euphorbiaceae)**

Spanish names: Pindule
Indigenous names: Juñi[03]
Used by (1*): Zoque[03]
Used for (1#): Skin[03]
Cognates:
Language contact:

***Hibiscus* (Malvaceae)**

Spanish names: Algodoncillo; Clavel; Flor de jamaica; Jamaica; Rosa de Jamaica; Tulipán; Tulipan del cerro; Tulipán rojo
Indigenous names: Tioch puk ay[03]; Thoot wits, manath ts'ohool, uxumlaab ts'ohool[07]; Tulipaan[07]; Utz Uj'[15]
Used by (14*): Zoque[01, 02, 03]; Totonac[06]; Huastec[07]; Yucatecan Maya[09]; Quichean Maya[12, 13, 14, 15, 16]; Western Maya[18]; Zapotec[21]; Nahua[24]
Used for (44#): Blood[12, 21]; Digestive[03, 12, 14, 18, 21]; Cardiovascular[01]; Musculoskeletal[12]; Neurological[03, 12, 16]; Psychological[02, 15, 24]; Respiratory[01, 02, 03, 09, 21]; Skin[02]; Endocrine[12]; Urological[01, 02, 12, 18]; Pregnancy[07, 14, 15, 24]; Female genital[01, 03, 07]; General and Unspecified[01, 02, 03, 06, 07, 12, 13, 21]
Cognates:
Language contact:

***Hidalgoa* (Asteraceae)**

Spanish names: Cogollo de sarpullido
Indigenous names: Ting puy tzäpä[02]; Pitx ay[03]
Used by (2*): Zoque[02, 03]
Used for (3#): Eye[03]; Skin[02, 03]
Cognates:
Language contact:

***Hilaria* (Poaceae)**

Spanish names:
Indigenous names:
Used by (1*): Huastec[07]
Used for (1#): Digestive[07]
Cognates:
Language contact:

Hintonia **(Rubiaceae)**

Spanish names: Quina / Hombre Grande
Indigenous names: K'ailaj Che'[12]
Used by (1*): Quichean Maya[12]
Used for (4#): Digestive[12]; Respiratory[12]; Endocrine[12]; General and Unspecified[12]
Cognates:
Language contact:

Hippobroma **(Campanulaceae)**

Spanish names: Acapa de monte
Indigenous names: Samat' pim[14]
Used by (2*): Quichean Maya[14, 17]
Used for (6#): Digestive[14]; Musculoskeletal[14]; Neurological[14]; Skin[14]; General and Unspecified[14]; nd[17]
Cognates:
Language contact:

Hirtella **(Chrysobalanaceae)**

Spanish names:
Indigenous names: Tsus pet cuy[03]; T¥kchi cuy[03]
Used by (1*): Zoque[03]
Used for (2#): Digestive[03]; Female genital[03]
Cognates:
Language contact:

Hoffmannia **(Rubiaceae)**

Spanish names: Calechilla; Hoja de rayo
Indigenous names: Mä tane, mä teksi[01]; Masan ay, Ts¥¥b ay[03]
Used by (3*): Zoque[01, 03]; Nahua[25]
Used for (4#): Ear[25]; Skin[01, 03]; General and Unspecified[01]
Cognates:
Language contact:

Hordeum **(Poaceae)**

Spanish names: Cebada
Indigenous names:
Used by (5*): Zoque[01, 02]; Quichean Maya[12, 13]; Western Maya[19]
Used for (10#): Digestive[01, 13]; Neurological[13]; Urological[01, 12]; Male genital[12]; General and Unspecified[02, 12, 13, 19]
Cognates:
Language contact:

Humulus **(Cannabaceae)**

Spanish names: Lúpulo
Indigenous names: Musuche' Aq'om[12]
Used by (1*): Quichean Maya[12]
Used for (5#): Digestive[12]; Cardiovascular[12]; Musculoskeletal[12]; Psychological[12]; Urological[12]
Cognates:
Language contact:

***Hura* (Euphorbiaceae)**

Spanish names: Empurga; Jabilla
Indigenous names:
Used by (2*): Mixe[04]; Zapotec[21]
Used for (2#): Digestive[04, 21]
Cognates:
Language contact:

***Hybanthus* (Violaceae)**

Spanish names: Hierba de San Antonio; Hoja de verruga; Trébol; Yerba familia
Indigenous names: Nekx cuy[03]; T'ithith t'eel[07]; Xpluxion xiw[09]; Sakbakelkam[09]
Used by (6*): Zoque[03]; Huastec[07]; Yucatecan Maya[09]; Quichean Maya[13]; Western Maya[18]; Nahua[24]
Used for (11#): Digestive[07]; Ear[13]; Musculoskeletal[13]; Neurological[09]; Skin[03, 09]; Pregnancy[03, 24]; General and Unspecified[07, 18]
Cognates:
Language contact:

***Hydrangea* (Hydrangeaceae)**

Spanish names: Hortensia
Indigenous names:
Used by (1*): Quichean Maya[13]
Used for (1#): Neurological[13]
Cognates:
Language contact:

***Hydrocotyle* (Araliaceae)**

Spanish names:
Indigenous names: Tzyokolate tane[01]; Makmak nab wamal[20]
Used by (2*): Zoque[01]; Western Maya[20]
Used for (2#): Digestive[20]; Skin[01]
Cognates:
Language contact:

***Hygrophila* (Acanthaceae)**

Spanish names:
Indigenous names: Re Kanteel[14]
Used by (1*): Quichean Maya[14]
Used for (1#): General and Unspecified[14]
Cognates:
Language contact:

***Hylocereus* (Cactaceae)**

Spanish names: Pitahaya; Pitaya
Indigenous names: Tsatsa', chacha[07]; Tuna rexte' juyub'[13]; Chik'ba'l b'ak[17]
Used by (7*): Huastec[07]; Yucatecan Maya[09]; Quichean Maya[12, 13, 17]; Nahua[24, 27]
Used for (11#): Digestive[09, 13]; Psychological[12]; Respiratory[27]; Skin[07, 13]; Endocrine[12]; Pregnancy[07, 24]; General and Unspecified[12]; nd[17]
Cognates:
Language contact:

Hymenaea **(Fabaceae)**

Spanish names: Guapinol; Guapinole
Indigenous names: Kuy po'te[02]; Payi[03]; Ak pej[04]; Pakay[19]; Biguu[21]
Used by (5*): Zoque[02, 03]; Mixe[04]; Western Maya[19]; Zapotec[21]
Used for (15#): Digestive[02, 03, 04, 21]; Musculoskeletal[21]; Respiratory[03, 04, 21]; Skin[04]; Endocrine[02, 03]; Urological[19]; Pregnancy[19]; Female genital[19]; General and Unspecified[21]
Cognates: MZ: po'te/payi/pej; Zoq: po'te/payi;
Language contact: MZ > Chortí

Hymenocallis **(Amaryllidaceae)**

Spanish names:
Indigenous names: Lakuum[07]
Used by (1*): Huastec[07]
Used for (1#): Skin[07]
Cognates:
Language contact:

Hyperbaena **(Menispermaceae)**

Spanish names: Huesillo; Zapote blanco
Indigenous names: Txa cuy[03]
Used by (2*): Zoque[02, 03]
Used for (4#): Cardiovascular[02]; Skin[03]; Endocrine[02]; Urological[03]
Cognates:
Language contact:

Hypericum **(Hypericaceae)**

Spanish names: Hierba del susto, hierba de San Antonio; Hipérico; Pericón del monte
Indigenous names: H¥xi ay[03]
Used by (4*): Zoque[02, 03]; Quichean Maya[12, 13]
Used for (10#): Digestive[02, 13]; Neurological[03]; Psychological[03, 12]; Skin[03]; Pregnancy[02]; General and Unspecified[02, 13]
Cognates:
Language contact:

Hypoxis **(Hypoxidaceae)**

Spanish names:
Indigenous names: Ceboll pim, Seb'oyil pim, Cewoyil pim[14]
Used by (1*): Quichean Maya[14]
Used for (1#): Respiratory[14]
Cognates:
Language contact:

Hyptis **(Lamiaceae)**

Spanish names: Alúcema blanca; Balsamo de campo/ balsamito; Canelón, verbena; Cedrón; Hierba de (san) martín/verbena; Hierba de martina; Hierba de toro; Hierba martín; Hierba martina/ hoja de martina; hierba negra; Hierba San Martín; Hierba toro; La peludita; Lavanda; Menta; Miltomate; Palo de menta; Pie de tortola; San martin; Verbena
Indigenous names: Naso'o mona[01]; Osyi ay[01]; Tzitzirane/wekpa tane[01]; Oyo ton/wakas ay[02]; Tzapas pa'petkuy[02]; Tsutsbet cuy[03]; Tam juñi[03]; San Martin ujts[04]; Santalipa, sunalipaljni[05]; Tihtsan ts'ohool, pithomlaam ts'ohool[07]; Tsak maape'[07]; Pitomlab ts'ojol[08]; Xta'ulum, Oregano KaX[09]; Chan[12]; Tem Kik'[14]; Xkis Kawaay [14]; Chu pim[15]; Xtye caway[16]; Se' ru'j kaway[17]; Chu' pim[17]; Tsuk pimi[18]; Gujcu'u, guichu'[21]; Guichu'[21]; Guixa'a[21]
Used by (19*): Zoque[01, 02, 03]; Mixe[04]; Totonac[05]; Huastec[07, 08]; Yucatecan Maya[09, 11]; Quichean Maya[12, 14, 15, 16, 17]; Western Maya[18, 19]; Zapotec[21]; Nahua[25, 26]
Used for (94#): Digestive[01, 02, 03, 04, 07, 08, 09, 12, 18, 21, 26]; Musculoskeletal[01, 02, 03, 08, 14, 18, 19, 21]; Neurological[01, 04, 12, 18]; Psychological[01, 02, 11, 12, 16]; Respiratory[01, 02, 03, 08, 12, 14, 21]; Skin[01, 02, 03, 04, 07, 12, 21, 25]; Endocrine[01]; Urological[01, 02, 03, 12]; Pregnancy[01, 02, 03, 07, 15, 21]; Female genital[01, 02, 03, 14, 15, 21, 26]; General and Unspecified[01, 02, 03, 05, 07, 11, 12, 14, 18, 19, 21, 25]; nd[17]
Cognates: Hua: pitomlaC tsoCol; CoreM: chu(k) pim; Quich: chu pim;
Language contact:

***Ibervillea* (Cucurbitaceae)**

Spanish names:
Indigenous names: Thokob ts'een[07]; K'umkanul[09]
Used by (2*): Huastec[07]; Yucatecan Maya[09]
Used for (3#): Musculoskeletal[09]; Skin[07, 09]
Cognates:
Language contact:

***Illicium* (Schisandraceae)**

Spanish names: Anís de estrella; Anis estrella; Anis estrellado, Anis chino, Bardana
Indigenous names: Ptsìidz[23]
Used by (8*): Zoque[01, 02]; Mixe[04]; Yucatecan Maya[09]; Quichean Maya[12]; Zapotec[21, 22, 23]
Used for (12#): Digestive[01, 02, 04, 09, 21, 22]; Cardiovascular[12]; Psychological[01, 12]; Respiratory[01]; Pregnancy[21]; General and Unspecified[23]
Cognates:
Language contact:

***Impatiens* (Balsaminaceae)**

Spanish names: Maravilla; Quinze añera
Indigenous names: Espiritu ujts[04]; China wits[07]
Used by (4*): Zoque[03]; Mixe[04]; Huastec[07]; Quichean Maya[13]
Used for (8#): Eye[13]; Ear[03]; Neurological[04]; Respiratory[03]; Skin[03, 13]; Pregnancy[07]; General and Unspecified[04]
Cognates:
Language contact:

***Imperata* (Poaceae)**

Spanish names:
Indigenous names: Ataa toom, kubat toom, kulab toom[07]
Used by (1*): Huastec[07]
Used for (3#): Digestive[07]; Urological[07]; General and Unspecified[07]
Cognates:
Language contact:

***Indigofera* (Fabaceae)**

Spanish names: Añil; Añil cimarron; Añil cimarron, hoja de tinta; Añil de Tinta
Indigenous names: Chims¥k[03]; Tsakam yaax, tsakam chichath, , muklaab ts'ohool, manath ts'ohool, muuw[07]; Initiko[08]; Xoxo-ak'[09]; Sujuxiw[09]
Used by (7*): Zoque[01, 03]; Huastec[07, 08]; Yucatecan Maya[09]; Western Maya[18]; Zapotec[21]
Used for (22#): Blood[07]; Digestive[03, 07, 08, 09, 18]; Musculoskeletal[08]; Neurological[07, 08, 18, 21]; Respiratory[03, 08, 21]; Skin[08, 21]; Endocrine[08]; Urological[21]; Pregnancy[03]; General and Unspecified[01, 21]
Cognates:
Language contact:

***Inga* (Fabaceae)**

Spanish names: Acotope, Vainilla; Carnequil; Carniquil; Chelel; Cuincuil
Indigenous names: Iki ay[01]; I'ki[02]; Inki[03]; Bujte'[18]; Cahijnaquil[21]
Used by (5*): Zoque[01, 02, 03]; Western Maya[18]; Zapotec[21]
Used for (10#): Digestive[03, 18]; Neurological[02]; Skin[03]; Endocrine[18]; Urological[03]; Female genital[01, 03]; General and Unspecified[21]
Cognates: Zoq: I(n)ki;
Language contact: Zoq > Nah

***Iostephane* (Asteraceae)**

Spanish names: Hierba del oso
Indigenous names: Ts'ojolil an oso[08]
Used by (1*): Huastec[08]
Used for (3#): Musculoskeletal[08]; Respiratory[08]; General and Unspecified[08]
Cognates:
Language contact:

***Ipomoea* (Convolvulaceae)**

Spanish names: Camote; Camote tahua; Chonege; Escamote; Guaco, Ipomea; Guamol; Guamol blanco/rojo; Jalapa; Manto; Pajaro bobo
Indigenous names: Nak'atang poj[02]; Xonege[03]; Nunak mooya[03]; Atooy aats[04]; Si'yu[06]; Huchuk'[07]; Ith[07]; Thuuyu'[07]; Ith[08]; Is[09]; Chiwohk'ax, Cancer xiw[09]; Paxin läq[12]; Is[14]; Xoconob[14]; Sayuk Q'ehen[14]; Nahuk Q'en[14]; Acum[18]; Guamol[21]; Ya banu[22]; Yàg-bnù, blàg-bnù, yàg-blàg-bnù[23]
Used by (13*): Zoque[02, 03]; Mixe[04]; Totonac[06]; Huastec[07, 08]; Yucatecan Maya[09]; Quichean Maya[12, 14]; Western Maya[18]; Zapotec[21, 22, 23]
Used for (55#): Blood[12]; Digestive[03, 04, 07, 08, 12, 14, 18, 21]; Eye[07]; Ear[14]; Musculoskeletal[08, 12, 18, 21]; Neurological[07, 14, 22, 23]; Psychological[12]; Respiratory[22]; Skin[02, 03, 04, 07, 09, 12, 21, 22]; Urological[14]; Pregnancy[06, 07, 14, 18]; General and Unspecified[03, 04, 07, 09, 14, 21, 22, 23]
Cognates: Zoq: nak; Maya: ith/is; Hua: ith; Zap: b(a)nu;
Language contact: Tot > Kekchí

***Iresine* (Amaranthaceae)**

Spanish names: Tlan cuaya
Indigenous names: Tsus tunuk koso[03]; Paloma jomol[20]; Zhwĭs, guìzh guièe nquĭts, guièe-mîdz[23]; Tlancuaya[26]
Used by (4*): Zoque[03]; Western Maya[20]; Zapotec[23]; Nahua[26]
Used for (12#): Blood[03]; Digestive[20, 23, 26]; Musculoskeletal[03]; Psychological[23]; Skin[03, 26]; General and Unspecified[03, 23, 26]
Cognates:
Language contact:

***Isocarpha* (Asteraceae)**

Spanish names: Oreja de conejo, hierba de conejo
Indigenous names: U chikin t'ur[19]
Used by (1*): Western Maya[19]
Used for (1#): Digestive[19]
Cognates:
Language contact:

***Isochilus* (Orchidaceae)**

Spanish names:
Indigenous names: Toom[07]
Used by (1*): Huastec[07]
Used for (1#): General and Unspecified[07]
Cognates:
Language contact:

***Jacaranda* (Bignoniaceae)**

Spanish names: Jacaranda
Indigenous names: Q'ojom che'[13]; Yàg-jàcàrân[23]
Used by (3*): Quichean Maya[12, 13]; Zapotec[23]
Used for (5#): Digestive[12, 13, 23]; Psychological[12]; General and Unspecified[12]
Cognates:
Language contact:

***Jasminum* (Oleaceae)**

Spanish names: Jazmín
Indigenous names: Hasmiin[07]
Used by (2*): Zoque[02]; Huastec[07]
Used for (3#): Respiratory[02, 07]; General and Unspecified[07]
Cognates:
Language contact:

***Jatropha* (Euphorbiaceae)**

Spanish names: Mala mujer/toloache; Ortiga; Piñon; Piñon, botja; Piñoncillo; Sangre de drago; Suzí
Indigenous names: Ekis[02]; Tzis kä wang [02]; Cuyukum[03]; Kutsøkee[04]; Chuta[05]; Čuta[05]; Chut'a[06]; Thakpeen te', piiloch[07]; Cruz ojo xiw[09]; Siklite'[09]; Pomolche'[09]; Làal[10]; Sakirte'[19]; Yàg-pcuà[23]
Used by (18*): Zoque[01, 02, 03]; Mixe[04]; Totonac[05, 06]; Huastec[07]; Yucatecan Maya[09, 10, 11]; Quichean Maya[12, 13]; Western Maya[19]; Zapotec[21, 22, 23]; Nahua[25, 27]
Used for (43#): Blood[07]; Digestive[01, 02, 03, 05, 06, 07, 09, 22]; Ear[10]; Musculoskeletal[10, 11, 13]; Neurological[02, 10, 19, 27]; Respiratory[05]; Skin[02, 03, 04, 05, 06, 07, 09, 11, 12, 19, 21, 22, 23, 25, 27]; Pregnancy[22]; Female genital[03]; General and Unspecified[02, 12, 19, 21, 22]
Cognates: Tot: chuta; CoreM: siklite / sakirte;
Language contact: Tot <> Highland Popoluca <> Mixe

***Juglans* (Juglandaceae)**

Spanish names: Nogal
Indigenous names: Mak xu 'xut qui 'hui[06]
Used by (4*): Zoque[03]; Totonac[06]; Quichean Maya[12]; Nahua[26]
Used for (11#): Blood[12]; Digestive[06, 12]; Musculoskeletal[12]; Psychological[26]; Respiratory[12]; Skin[06]; Endocrine[12]; Urological[26]; Pregnancy[03]; General and Unspecified[12]
Cognates:
Language contact:

***Juniperus* (Cupressaceae)**

Spanish names: Cedro; Sabino, ahuehuete
Indigenous names: Yàg-guìzdòo, yàg-guìstòo[23]
Used by (2*): Zapotec[23]; Nahua[25]
Used for (3#): Neurological[25]; Skin[23]; General and Unspecified[25]
Cognates:
Language contact:

***Justicia* (Acanthaceae)**

Spanish names: Añil/tinta chiapaneca/ hoja de tinta, Ciencodo/codito ; Camarón; Camarón de Montaña; Flor de tila, tilo; Hierba azul; Hierba del santuario; Hoje de tinta, añil; Mohuite; Moitle, muicle; Muicle; Muitle; Pote de la tiñadora, Añil; Pote verde; Rompe piedra; Tinta; Trébol; Yolixpa
Indigenous names: Tzämi tane/tzitz[01]; Tutxti ay[03]; Majei chich[03]; Chich[03]; Limanin[05]; Tsaakuy elul, xonol palats, nonool t'ot, witssil a eheenchix, ok t'ot, tsakam payab wits, uxkwe' ts'ohool, eheenchix wits, wots paya', bohol ch'ohool[07]; Pithomlaab ts'ohool[07]; Tsaakuy elul, xonol palats[07]; Muu, muuw[07]; Muu[08]; Lumbresil pim[14]; Ixwaq[14]; Rax Pim[15]; Numay Pim[15]; Xna kejen[16]; Saxjolom chacmut[16]; K'xuy i kok, Santa Maria kejen[16]; Santa Maria k'ejen[17]; Sa'x jolom chacmut[17]; U nich tila[18]; T'oxe ji'tn[18]; Mohuitl[25]; Muictli[28]
Used by (17*): Zoque[01, 03]; Totonac[05, 06]; Huastec[07, 08]; Quichean Maya[12, 14, 15, 16, 17]; Western Maya[18, 19]; Zapotec[21]; Nahua[25, 26, 28]
Used for (82#): Blood[06, 08]; Digestive[01, 03, 06, 07, 14, 25, 26]; Ear[18]; Cardiovascular[03, 08, 25]; Musculoskeletal[25]; Neurological[03, 07, 16, 19, 21, 25]; Psychological[03, 07, 08, 15, 16, 18, 25]; Respiratory[01, 07, 18, 25, 26]; Skin[03, 07, 08, 12, 25, 26, 28]; Endocrine[08, 12, 18]; Urological[01, 18]; Pregnancy[01, 07, 15]; Female genital[03, 05, 07, 15]; General and Unspecified[01, 03, 05, 06, 07, 08, 14, 15, 18, 19, 21, 25, 26, 28]; nd[17]
Cognates: Zoq: tzitz / chich; Hua: muu; Nah: muictli;
Language contact: Hua <> Quich <> Nah

01-28 refer to the studies described in Table 4.1 * Total number of studies recording this taxon #Total number of use-records

Kalanchoe (Crassulaceae)

Spanish names: Beladona (macho); Beladonna; Beladonna gigante, mala madre; Belladona; Hüipil de monte; Maravilla real; sanalotodo/curalotodo/lengua de vecino
Indigenous names: Majei maravilla[03]; Pooti'l qehen[14]
Used by (6*): Zoque[01, 02, 03]; Yucatecan Maya[09]; Quichean Maya[14]; Western Maya[18]
Used for (24#): Digestive[01, 03, 18]; Eye[03, 14]; Ear[03]; Musculoskeletal[02, 09, 14, 18]; Neurological[02]; Respiratory[18]; Skin[01, 03, 09, 18]; Urological[01]; Female genital[01]; General and Unspecified[01, 02, 03]
Cognates:
Language contact:

Kallstroemia (Zygophyllaceae)

Spanish names: Campanilla/hoja de azar
Indigenous names: Tza'a tzoy[01]
Used by (1*): Zoque[01]
Used for (1#): General and Unspecified[01]
Cognates:
Language contact:

Karwinskia (Rhamnaceae)

Spanish names: Guiiguiste
Indigenous names: Ixim te'[19]
Used by (1*): Western Maya[19]
Used for (1#): Skin[19]
Cognates:
Language contact:

Kearnemalvastrum (Malvaceae)

Spanish names: Malva real, Malvavisca
Indigenous names: Malma, Malva Aq'om[12]
Used by (1*): Quichean Maya[12]
Used for (5#): Blood[12]; Cardiovascular[12]; Psychological[12]; Skin[12]; General and Unspecified[12]
Cognates:
Language contact:

Kionophyton (Orchidaceae)

Spanish names:
Indigenous names: Kw'itool ts'ohool, k'ok'om it'ath ts'ohool[07]
Used by (1*): Huastec[07]
Used for (1#): Pregnancy[07]
Cognates:
Language contact:

Koanophyllon (Asteraceae)

Spanish names: Hoja vishe
Indigenous names: Z¥¥y ay[03]; Øgøin aay[04]; Tok'te', yaxal[07]
Used by (4*): Zoque[02, 03]; Mixe[04]; Huastec[07]
Used for (10#): Digestive[07]; Musculoskeletal[07]; Neurological[07]; Psychological[02]; Respiratory[07]; Skin[03]; General and Unspecified[02, 03, 04, 07]
Cognates:
Language contact:

Kohleria **(Gesneriaceae)**

Spanish names: Planta capulina
Indigenous names:
Used by (1*): Zoque[03]
Used for (1#): Skin[03]
Cognates:
Language contact:

Kosteletzkya **(Malvaceae)**

Spanish names:
Indigenous names: Bisil, Xcampana ka'ax[09]
Used by (1*): Yucatecan Maya[09]
Used for (1#): Digestive[09]
Cognates:
Language contact:

Krameria **(Krameriaceae)**

Spanish names: Romerito, hoja de disenteria
Indigenous names: Luu guiatzimbeer[21]
Used by (1*): Zapotec[21]
Used for (3#): Digestive[21]; Pregnancy[21]; Female genital[21]
Cognates:
Language contact:

Krugiodendron **(Rhamnaceae)**

Spanish names:
Indigenous names: Chintok'[09]
Used by (1*): Yucatecan Maya[09]
Used for (2#): Neurological[09]; Urological[09]
Cognates:
Language contact:

Kyllinga . **(Cyperaceae)**

Spanish names:
Indigenous names: Xmach tz'i' Q'ehen[14]
Used by (1*): Quichean Maya[14]
Used for (1#): Endocrine[14]
Cognates:
Language contact:

Lactuca **(Asteraceae)**

Spanish names: Lechuga
Indigenous names:
Used by (3*): Zoque[02]; Totonac[06]; Quichean Maya[13]
Used for (4#): Psychological[06]; Endocrine[02]; Pregnancy[13]; Female genital[13]
Cognates:
Language contact:

Laetia (Salicaceae)

Spanish names: Corrimiento
Indigenous names:
Used by (1*): Yucatecan Maya[11]
Used for (1#): General and Unspecified[11]
Cognates:
Language contact:

Lagascea (Asteraceae)

Spanish names:
Indigenous names: Papan te'[20]
Used by (1*): Western Maya[20]
Used for (1#): Digestive[20]
Cognates:
Language contact:

Lagenaria (Cucurbitaceae)

Spanish names: Chical pestle, pumpo, lipo; Tecomate
Indigenous names: Pok[03]; Xomom, kweentu'[07]; Lek[09]; Lobej' quiasj'ga, beejru'u[21]
Used by (4*): Zoque[03]; Huastec[07]; Yucatecan Maya[09]; Zapotec[21]
Used for (8#): Digestive[03, 21]; Musculoskeletal[09]; Psychological[03]; Respiratory[07, 21]; Urological[07]; General and Unspecified[21]
Cognates:
Language contact:

Lamourouxia (Orobanchaceae)

Spanish names:
Indigenous names: Guièe-dzĭṉg, guìzh-dzĭṉg[23]
Used by (1*): Zapotec[23]
Used for (2#): Skin[23]; General and Unspecified[23]
Cognates:
Language contact:

Lantana (Verbenaceae)

Spanish names: Cinco negritos; Cinco negritos (blanco); Cinco negritos, salvia de monte, salvia silvestre, oregano; Conchita/conchinita/conchulita/chubaroba/chibaloba/hoja de conchudo; Flor de sangre; Ojo de pescado, orozuz; Orozuz; Riñonina; Salvareal/riñosan; Sapotilla; Siete Negritos; Té de monte
Indigenous names: Pajk jäyä[01]; Pak jäyä[01]; Kan'muk[03]; Tøøts kumot[04]; Škaštajat štuki[05]; X'laca stap'u squii'ti[06]; Thak patelx, patel mantelx, kanil bakan, thak otoomal[07]; Tsak patelax, witsiim i thayemlaab, tsakam maap, tsakam otoomal, wal thancha', pahatix wits[07]; Baron[07]; Roq' Chiwan[12]; Ruwi amaj, corcoch, cor choch, utucán cumatz[13]; Saq'i Tulux[14]; Xkot' Kaway[14]; Tulux Q'ehen[14]; Tulush pim[17]; Ch'ilvet, ch'ili wet, ch'ilch'il wajch[20]; Guxa'a riene'e[21]; Žob leh[22]
Used by (17*): Zoque[01, 02, 03]; Mixe[04]; Totonac[05, 06]; Huastec[07]; Yucatecan Maya[09]; Quichean Maya[12, 13, 14, 17]; Western Maya[19, 20]; Zapotec[21, 22]; Nahua[26]
Used for (82#): Digestive[01, 02, 03, 06, 07, 09, 12, 13, 14, 20, 21, 22]; Cardiovascular[12, 13]; Musculoskeletal[07, 12, 13]; Neurological[03, 04, 06, 07, 12, 13, 14]; Psychological[03]; Respiratory[01, 03, 05, 06, 13, 14, 20, 26]; Skin[03, 07, 12, 13, 21]; Endocrine[07, 14]; Urological[01, 07, 12, 20]; Pregnancy[01, 03, 07, 19, 20]; Female genital[01, 12, 13, 19]; Male genital[12]; General and Unspecified[02, 06, 07, 12, 13, 14, 20, 26]; nd[17]
Cognates: CoreM: chiwan; Quich: tulux;
Language contact: Chiapas Zoq <> Hua <> Tot

Laportea (Urticaceae)

Spanish names: Ortiga
Indigenous names: Làal[10]
Used by (1*): Yucatecan Maya[10]
Used for (3#): Ear[10]; Musculoskeletal[10]; Neurological[10]
Cognates:
Language contact:

01-28 refer to the studies described in Table 4.1 * Total number of studies recording this taxon #Total number of use-records

***Larrea* (Zygophyllaceae)**

Spanish names: Gobernadora
Indigenous names:
Used by (1*): Zapotec[21]
Used for (1#): Digestive[21]
Cognates:
Language contact:

***Lasiacis* (Poaceae)**

Spanish names: Carisso; Carizo
Indigenous names: Am ay[02]; K¥kujuki ay[03]; Tseey kw'a', thimallon pakaab[07]; Siit[09]; Guìzh-gòob-guì, guìzh-gòob[23]
Used by (5*): Zoque[02, 03]; Huastec[07]; Yucatecan Maya[09]; Zapotec[23]
Used for (9#): Digestive[07]; Musculoskeletal[07]; Skin[03, 09]; Urological[02, 07]; General and Unspecified[07, 23]
Cognates:
Language contact:

***Lasianthaea* (Asteraceae)**

Spanish names:
Indigenous names: Q'aham tzaj[14]
Used by (1*): Quichean Maya[14]
Used for (1#): General and Unspecified[14]
Cognates:
Language contact:

***Laurus* (Lauraceae)**

Spanish names: Laurel
Indigenous names:
Used by (1*): Zoque[03]
Used for (1#): General and Unspecified[03]
Cognates:
Language contact:

***Lavandula* (Lamiaceae)**

Spanish names: Alucema
Indigenous names:
Used by (3*): Zoque[01, 02]; Western Maya[19]
Used for (12#): Blood[19]; Digestive[01, 02]; Musculoskeletal[02]; Respiratory[01, 02]; Pregnancy[01, 02, 19]; Female genital[01, 02]; General and Unspecified[01]
Cognates:
Language contact:

***Lawsonia* (Lythraceae)**

Spanish names: Rosedad
Indigenous names: Rosedad[04]
Used by (2*): Mixe[04]; Zapotec[21]
Used for (2#): Skin[04]; General and Unspecified[21]
Cognates:
Language contact:

***Leonotis* (Lamiaceae)**

Spanish names: Hierba del burro
Indigenous names:
Used by (2*): Yucatecan Maya[09]; Nahua[26]
Used for (2#): Digestive[26]; Neurological[09]
Cognates:
Language contact:

***Leonurus* (Lamiaceae)**

Spanish names: Mariguana simarron/ Chiquisa; Marihuanilla
Indigenous names:
Used by (3*): Zoque[01, 03]; Mixe[04]
Used for (6#): Digestive[03]; Musculoskeletal[01, 03]; Skin[01, 03]; Female genital[04]
Cognates:
Language contact:

***Lepechinia* (Lamiaceae)**

Spanish names: Bretónica; Brétonica, hierba del sapo, chucharilla; Bretonica, hierba tónica, arnica
Indigenous names: Qaj tajik q'ox[12]; Upek'tzi[13]; Cha vitz[13]; Tzotzil vomol, yaxal vomol, na p'ilix, chilchil tz'i'lel, poxil a'ch'ut[20]
Used by (4*): Quichean Maya[12, 13]; Western Maya[20]; Nahua[25]
Used for (16#): Digestive[13, 20]; Cardiovascular[25]; Musculoskeletal[12, 13]; Neurological[20]; Psychological[13]; Respiratory[20, 25]; Skin[13]; Endocrine[25]; General and Unspecified[12, 13, 20]
Cognates:
Language contact:

***Lepidaploa* (Asteraceae)**

Spanish names:
Indigenous names: Sotopok tsay[03]; Semem Q'ehen[14]
Used by (2*): Zoque[03]; Quichean Maya[14]
Used for (5#): Digestive[03]; Respiratory[03]; Skin[03]; General and Unspecified[03, 14]
Cognates:
Language contact:

***Lepidium* (Brassicaceae)**

Spanish names: Altanisa; Hierba de pollo; Jilipliege, antemelia; Jilipliege, Mostaza; Maltuerce; Mermejita, estrella hembra; Pañalita; Pierna vieja
Indigenous names: Ma'tza yomo [01]; Tsakam utsun, utsun ts'ohool[07]; Rakän aqwäl, Saqil Kayis, Mesebäl Q'os Paxk'u'y[12]; Siquil be', skil q'ayes, ik q'ayes, masb'al uxekaj[13]; Pich t'uluk, pich' tz'i lel, sakil jomol, sak nich wamal, kajk'an wamal, anix te' wamal[20]; Pich t'uluk. Sakil jomol, sak nich wamal, kajk'an wamal, anix te' wamal[20]; Guish inguiedi[21]; Siak[22]
Used by (10*): Zoque[01]; Huastec[07]; Yucatecan Maya[11]; Quichean Maya[12, 13]; Western Maya[19, 20]; Zapotec[21, 22]; Nahua[26]
Used for (28#): Digestive[01, 07, 12, 13, 19, 20]; Ear[13]; Musculoskeletal[12]; Neurological[12, 13]; Psychological[12, 13]; Respiratory[12, 13, 26]; Skin[11, 12, 20, 21, 22]; Endocrine[12]; Urological[13]; Pregnancy[20]; Female genital[11, 12, 21]; General and Unspecified[12, 26]
Cognates: CoreM: sakil; Quich: s(a)kil kayis, mesbel;
Language contact: Chiapas Zoq <> Hua

***Lessingianthus* (Asteraceae)**

Spanish names:
Indigenous names: Semem[14]
Used by (1*): Quichean Maya[14]
Used for (1#): Digestive[14]
Cognates:
Language contact:

***Leucaena* (Fabaceae)**

Spanish names: Guaje; Guaxi; Huaje blanco; Huaje de la peña/ de agua; Huaje/Huash; Timbre
Indigenous names: Pakapaka[01]; Li'li'ka, guamuxi[06]; Thuk'[07]; Waxim[09]; Laj'[21]; Laj' tza gutzii[21]; Lya[22]; Yàg-nlàbâd, yàg-nlìbâd, yàg-nlázhò, yàg-nlízhò[23]
Used by (8*): Zoque[01]; Totonac[06]; Huastec[07]; Yucatecan Maya[09]; Zapotec[21, 22, 23]; Nahua[26]
Used for (14#): Digestive[01, 06, 21, 22, 23, 26]; Skin[09, 23]; General and Unspecified[01, 07, 21]
Cognates: Zap: laj/lya/lazh;
Language contact: Zap > Tot

***Leucanthemum* (Asteraceae)**

Spanish names: Margarita comun
Indigenous names:
Used by (1*): Quichean Maya[12]
Used for (3#): Respiratory[12]; Skin[12]; General and Unspecified[12]
Cognates:
Language contact:

***Liabum* (Asteraceae)**

Spanish names:
Indigenous names: Saq sa'ab[16]
Used by (1*): Quichean Maya[16]
Used for (2#): Neurological[16]; Psychological[16]
Cognates:
Language contact:

***Licania* (Chrysobalanaceae)**

Spanish names: Mezon zapote; Zapote cabello; Zunso
Indigenous names: A'kchi xi't jaaca[06]; Moxpim, Jol'bob[14]; Jor b'oj[19]; Yàg-guiél-bêdz[23]
Used by (4*): Totonac[06]; Quichean Maya[14]; Western Maya[19]; Zapotec[23]
Used for (6#): Digestive[06, 14, 19]; Skin[23]; Pregnancy[23]; General and Unspecified[06]
Cognates: CoreM: jol'bob/jorb'oj;
Language contact:

***Licaria* (Lauraceae)**

Spanish names: Laurel amarillo; Laurel Negro
Indigenous names: Y¥k moko[03]; Puutx moko[03]
Used by (1*): Zoque[03]
Used for (6#): Digestive[03]; Cardiovascular[03]; Skin[03]; Female genital[03]
Cognates:
Language contact:

***Ligustrum* (Oleaceae)**

Spanish names: Arrayana; Trueno
Indigenous names: Yàg-truên[23]
Used by (3*): Zoque[01]; Quichean Maya[13]; Zapotec[23]
Used for (3#): Digestive[01]; Skin[13]; General and Unspecified[23]
Cognates:
Language contact:

***Linum* (Linaceae)**

Spanish names: Linaza
Indigenous names:
Used by (4*): Zoque[01, 02]; Quichean Maya[12, 13]
Used for (10#): Digestive[01, 12, 13]; Neurological[12]; Respiratory[01]; Skin[12]; Urological[01, 12]; General and Unspecified[02, 12]
Cognates:
Language contact:

***Lippia* (Verbenaceae)**

Spanish names: Malvareal/salvareal/malva de castilla/hierbabuena; Oregano; Orégano ; Oregano silvestre/ de campo; Palo de gusano; Pitiona; Salvareal; Salvia amarilla; Salvia de castilla; Salvia real, malva real; Salvia Santa; Salvia Sija; Salvia sija, salvia santa, salvia morada, juanilana; Sorosir; Tapón; Te de china/orozus; Té de la abuela; Té de limón
Indigenous names: Anaamte', thak te'[07]; Loq'oläj Chol Q'os[12]; Salv' sant', uwi juyub q'ayes[13]; Tulux Q'ehen[14]; Q'iil pim [14]; Tu'lush pim[16]; Alba noxi'na[18]; Pisis nich vomol[20]; Yagangucha'a[21]; Salb gohts[22]; Tapontizana[26]
Used by (18*): Zoque[01, 02, 03]; Mixe[04]; Huastec[07]; Yucatecan Maya[09]; Quichean Maya[12, 13, 14, 16]; Western Maya[18, 19, 20]; Zapotec[21, 22]; Nahua[26, 27, 28]
Used for (60#): Blood[12]; Digestive[02, 03, 04, 07, 09, 12, 13, 18, 19, 20, 21, 22, 26, 27, 28]; Cardiovascular[12]; Musculoskeletal[02, 03, 12, 13]; Neurological[12, 16, 22]; Psychological[01, 13]; Respiratory[01, 03, 12, 13, 14, 27]; Skin[07, 09, 26]; Urological[07]; Pregnancy[02, 04, 09, 13, 19, 21, 27]; Female genital[02, 03, 13, 19, 28]; Male genital[02]; General and Unspecified[03, 12, 13, 21, 22]
Cognates: Quich: tulux;
Language contact:

***Liquidambar* (Altingiaceae)**

Spanish names: Liquidambar; Ocosote; Ocozote; Suchiate
Indigenous names: Täsy kuy[01]; T¥x cuy[03]; So'te'[20]; Bijtu'u[21]; Suchiate[25]; Ocosotito[26]
Used by (7*): Zoque[01, 03]; Quichean Maya[13]; Western Maya[20]; Zapotec[21]; Nahua[25, 26]
Used for (16#): Digestive[01, 20, 21, 25]; Musculoskeletal[01, 13]; Neurological[01]; Skin[03, 13, 25]; Pregnancy[01]; Female genital[01]; General and Unspecified[01, 13, 21, 26]
Cognates: Zoq: täx kuy;
Language contact: Nah <> Tzeltalan

***Lithachne* (Poaceae)**

Spanish names:
Indigenous names: Tsakam pakaab, tsakam tsahib, tsakam tseey kw'a', tsakam toom, pakaabil i kw'a'[07]
Used by (1*): Huastec[07]
Used for (2#): Neurological[07]; Pregnancy[07]
Cognates:
Language contact:

***Lithospermum* (Boraginaceae)**

Spanish names: Mitamorial; Té Moreal
Indigenous names:
Used by (2*): Quichean Maya[12, 13]
Used for (3#): Digestive[12]; Respiratory[12]; Female genital[13]
Cognates:
Language contact:

***Litsea* (Lauraceae)**

Spanish names: Laurel
Indigenous names: Toka tzajtza/toka tzasa/toka' ay[01]; Lawreel[07]; Ts'uj[08]; Roj Xwan[12]; Tziltzil ujch', tzis uch[20]; Guib diitz[21]
Used by (10*): Zoque[01, 02]; Totonac[06]; Huastec[07, 08]; Quichean Maya[12]; Western Maya[20]; Zapotec[21, 22, 23]
Used for (31#): Digestive[01, 02, 06, 08, 12, 20, 21]; Eye[21]; Cardiovascular[12]; Musculoskeletal[01, 08]; Neurological[06, 08]; Psychological[06, 08, 12]; Respiratory[06]; Endocrine[08]; Pregnancy[01, 21, 22, 23]; Female genital[01, 02]; General and Unspecified[01, 06, 07, 08, 12, 21, 23]
Cognates:
Language contact: Chiapas Zoq <> Hua <> Tzeltalan <> Zap

01-28 refer to the studies described in Table 4.1 * Total number of studies recording this taxon #Total number of use-records

Lobelia **(Campanulaceae)**

Spanish names: Hierba de Conejo, Mejorana Morada de Agua, Chilio; Hierba de la enferma, flor de chupa
Indigenous names: Sal' Keq Aq'om K'ik[12]; Tzajal nich wamal, paj nich te', turesna wamal, prima najk, pameyat[20]; Guìzh-sàntàmàrǐ[23]; Guìzh-guièe-dzǐng, guièe-dán, guièe-měets, guièe-nàrânj[23]; Otzpacxihuitl[26]
Used by (4*): Quichean Maya[12]; Western Maya[20]; Zapotec[23]; Nahua[26]
Used for (11#): Digestive[20]; Cardiovascular[12]; Musculoskeletal[12, 20]; Psychological[12]; Respiratory[23]; Skin[23, 26]; Pregnancy[20]; Female genital[20]; General and Unspecified[23]
Cognates:
Language contact:

Loeselia **(Polemoniaceae)**

Spanish names: Espinosilla; Espinosillo; Espinosillo, yerba de espanto; Espinozillo; Jaboncillo, espinozillo de cerro
Indigenous names: Škwan jehb[22]; Škwan jehb las[22]; Spìnòsî[23]; Huitzitziquitl[28]
Used by (5*): Zoque[02]; Zapotec[21, 22, 23]; Nahua[28]
Used for (20#): Digestive[21, 22, 23]; Neurological[23]; Respiratory[23, 28]; Skin[21, 23]; Urological[22, 23]; Pregnancy[22]; Female genital[21]; General and Unspecified[02, 21, 22, 23, 28]
Cognates:
Language contact:

Lonchocarpus **(Fabaceae)**

Spanish names:
Indigenous names: Xuul[09]; Balche'[09]
Used by (1*): Yucatecan Maya[09]
Used for (5#): Musculoskeletal[09]; Neurological[09]; Respiratory[09]; General and Unspecified[09]
Cognates:
Language contact:

Lopezia **(Onagraceae)**

Spanish names: Hierba de la araña
Indigenous names: Xalu vomol[20]
Used by (2*): Western Maya[20]; Nahua[26]
Used for (3#): Digestive[20]; Musculoskeletal[26]; Skin[26]
Cognates:
Language contact:

Lophosoria **(Dicksoniaceae)**

Spanish names: Zarzaparilla
Indigenous names:
Used by (1*): Nahua[25]
Used for (1#): Urological[25]
Cognates:
Language contact:

Louteridium **(Acanthaceae)**

Spanish names:
Indigenous names: Ojoj'[14]
Used by (1*): Quichean Maya[14]
Used for (2#): Digestive[14]; General and Unspecified[14]
Cognates:
Language contact:

***Loxothysanus* (Asteraceae)**

Spanish names:
Indigenous names: W¥y¥ ay[03]; Kaax ujts[04]; Thak pux, chak pux[07]
Used by (3*): Zoque[03]; Mixe[04]; Huastec[07]
Used for (4#): Psychological[07]; Skin[03, 04]; General and Unspecified[07]
Cognates:
Language contact:

***Ludwigia* (Onagraceae)**

Spanish names: Clavillo; Clavito
Indigenous names: Clavo sotyi[03]; Nø monda ujts[04]; Kla'uxa pim[14]; Tzentlialeche[26]
Used by (4*): Zoque[03]; Mixe[04]; Quichean Maya[14]; Nahua[26]
Used for (10#): Digestive[03, 14, 26]; Neurological[03]; Respiratory[03, 26]; Skin[04, 26]; Female genital[14]; General and Unspecified[14]
Cognates:
Language contact:

***Luehea* (Malvaceae)**

Spanish names: Tepecacao
Indigenous names: Cang cang pujki[03]; K'askat[09]
Used by (2*): Zoque[03]; Yucatecan Maya[09]
Used for (5#): Skin[03, 09]; Urological[03]; Female genital[03]; General and Unspecified[03]
Cognates:
Language contact:

***Luffa* (Cucurbitaceae)**

Spanish names: Estropajo; Limpion
Indigenous names: Po't[04]
Used by (3*): Mixe[04]; Yucatecan Maya[09]; Zapotec[21]
Used for (4#): Digestive[09]; Skin[04, 21]; Urological[09]
Cognates:
Language contact:

***Lupinus* (Fabaceae)**

Spanish names: Alfalfa
Indigenous names: Much' Q'os[12]
Used by (1*): Quichean Maya[12]
Used for (2#): Blood[12]; Pregnancy[12]
Cognates:
Language contact:

***Lycianthes* (Solanaceae)**

Spanish names: Majagua blanco
Indigenous names: Popo po'a[01]; Masan ay[03]; Roq' Xa'an[14]
Used by (3*): Zoque[01, 03]; Quichean Maya[14]
Used for (3#): Skin[01, 03, 14]
Cognates:
Language contact:

***Lycopodium* (Lycopodiaceae)**

Spanish names: Licopodio
Indigenous names:
Used by (1*): Zoque[01]
Used for (1#): Cardiovascular[01]
Cognates:
Language contact:

***Lycoseris* (Asteraceae)**

Spanish names: Santa María
Indigenous names:
Used by (1*): Quichean Maya[14]
Used for (1#): Skin[14]
Cognates:
Language contact:

***Lygodium* (Lygodiaceae)**

Spanish names: Alambrilla; Atagota de bejuco; Atagota de bejuco, Bejuco de lambri; Curalina/hoja vishu; Doradilla, quebrahaca; Hoja de la vibora
Indigenous names: Zajin syingtzyi/o'osi ay[02]; Naxiui[03]; Kuti' iny ujts[04]; K'util papaam, paxlaab papaan[07]; Rich' Mu' li' ba', Ruxb'i Kaaq'i[14]; Ruxb'i'kaak[16]; Ruxb'i kaak[17]; Yop'te hacha[18]; Guixa'a mbala'a[21]
Used by (9*): Zoque[02, 03]; Mixe[04]; Huastec[07]; Quichean Maya[14, 16, 17]; Western Maya[18]; Zapotec[21]
Used for (23#): Digestive[03, 07]; Musculoskeletal[14]; Neurological[14, 16]; Psychological[02, 07, 16]; Skin[02, 03, 04, 21]; Endocrine[07]; Urological[02, 03, 18]; Pregnancy[07]; Female genital[02]; General and Unspecified[07, 16]; nd[17]
Cognates: Quich: ruxb'i kaak;
Language contact: Mixe <> Hua; Hua <> Zap

***Lysiloma* (Fabaceae)**

Spanish names: Guaje del campo; Quebracho; Songuavite; Tepehuaje, tehuaje; Timbre
Indigenous names: Cana cuy[03]; Wayal[07]; Tsalam[09]; Yaj[19]; Lya[22]; Tehuaxi[28]
Used by (7*): Zoque[03]; Huastec[07]; Yucatecan Maya[09]; Western Maya[19]; Zapotec[22]; Nahua[26, 28]
Used for (17#): Digestive[03, 07, 19, 22, 26, 28]; Neurological[07, 09, 19]; Psychological[07]; Respiratory[28]; Skin[03, 07]; Endocrine[03]; Female genital[03, 07]; General and Unspecified[07]
Cognates:
Language contact: Hua <> Chortí <> Zap <> Nah

***Lythrum* (Lythraceae)**

Spanish names:
Indigenous names: Lehem ts'ohool, tsab k'a'um, itsaan an maan witsiil[07]; Tlalhuayopactle[26]
Used by (2*): Huastec[07]; Nahua[26]
Used for (4#): Neurological[26]; Respiratory[26]; Skin[26]; General and Unspecified[07]
Cognates:
Language contact:

***Macadamia* (Proteaceae)**

Spanish names: Macadamia
Indigenous names:
Used by (1*): Quichean Maya[12]
Used for (5#): Digestive[12]; Neurological[12]; Psychological[12]; Skin[12]; General and Unspecified[12]
Cognates:
Language contact:

***Machaerium* (Fabaceae)**

Spanish names: Uña de gato; Uña de gato/sangre de cristo; Uña de gavilan
Indigenous names: Powui kä'tzi ma'syi[02]; N¥pin tsay[03]; Misi k¥ts¥s[03]; Santo no'ot[04]; Itsik' t'iiw[07]; Pur caham[16]; Lokoch k'ix[17]
Used by (7*): Zoque[01, 02, 03]; Mixe[04]; Huastec[07]; Quichean Maya[16, 17]
Used for (18#): Digestive[03, 04]; Neurological[02, 07]; Psychological[16]; Skin[03, 04]; Endocrine[03]; Female genital[03]; General and Unspecified[01]; nd[17]
Cognates:
Language contact:

***Maclura* (Moraceae)**

Spanish names: Murra
Indigenous names: Tsitsiy[07]
Used by (2*): Zoque[03]; Huastec[07]
Used for (8#): Musculoskeletal[07]; Neurological[03, 07]; Respiratory[07]; Skin[07]; Urological[07]; General and Unspecified[03, 07]
Cognates:
Language contact:

***Macrothelypteris* (Thelypteridaceae)**

Spanish names: Helecho silvestre/ macho
Indigenous names: Ocopetate[26]
Used by (1*): Nahua[26]
Used for (2#): Psychological[26]; Urological[26]
Cognates:
Language contact:

***Magnolia* (Magnoliaceae)**

Spanish names: Flor de corazón; Flor de corazón, magnolia; Magnolia; Yololxochitl; Yoloxóchitl
Indigenous names: Tzoko jäyä/Tzapi kopak [01]; Pa' ju'us/tzokoy toya jäyä[02]; Mooyniakcuy[03]; Kuwi šanat[05]; Qui'huixan, qu'yu xanat[06]; Yagabedxii[21]
Used by (7*): Zoque[01, 02, 03]; Totonac[05, 06]; Quichean Maya[12]; Zapotec[21]
Used for (24#): Digestive[01, 02, 03, 05, 21]; Cardiovascular[02, 05, 06, 12, 21]; Neurological[12]; Psychological[01, 05, 06, 12]; Pregnancy[03, 05, 06]; Female genital[03]; General and Unspecified[01, 05, 12]
Cognates: Zoq: tzoko jäyä; Tot: kuwi xanat;
Language contact:

***Maianthemum* (Asparagaceae)**

Spanish names: Vara de Salomón
Indigenous names:
Used by (1*): Quichean Maya[12]
Used for (3#): Cardiovascular[12]; Urological[12]; General and Unspecified[12]
Cognates:
Language contact:

***Malachra* (Malvaceae)**

Spanish names: Hierba de Cancer; Malva; Malva peluda
Indigenous names: Pulik thipon, paktha' thipon[07]; Tza' Tzalun Mi' Ha'[14]
Used by (4*): Zoque[01]; Huastec[07]; Quichean Maya[14]; Western Maya[18]
Used for (7#): Digestive[18]; Psychological[01]; Urological[01]; General and Unspecified[01, 07, 14]
Cognates:
Language contact:

Malouetia (Apocynaceae)

Spanish names: Mbiigu' moradu
Indigenous names:
Used by (1*): Zapotec[21]
Used for (1#): Skin[21]
Cognates:
Language contact:

Malpighia (Malpighiaceae)

Spanish names:
Indigenous names: K'ak'al ilaal[07]
Used by (1*): Huastec[07]
Used for (3#): Neurological[07]; Respiratory[07]; General and Unspecified[07]
Cognates:
Language contact:

Malus (Rosaceae)

Spanish names: Manzana
Indigenous names: Yàg-mànzân[23]
Used by (2*): Quichean Maya[12]; Zapotec[23]
Used for (5#): Digestive[12]; Eye[12]; Respiratory[12, 23]; General and Unspecified[12]
Cognates:
Language contact:

Malva (Malvaceae)

Spanish names: Hierba del puerco; Malba; Malva
Indigenous names: Tankilkixit[08]; Cho'j, tzelej, tze'ek[13]; Gišlobeh [22]; Blàg-mêd, guìzh-blàg-mêd, mâlb[23]
Used by (8*): Zoque[01]; Huastec[08]; Quichean Maya[12, 13]; Zapotec[22, 23]; Nahua[25, 26]
Used for (37#): Blood[12]; Digestive[01, 12, 13, 22]; Eye[13]; Cardiovascular[12]; Musculoskeletal[01]; Neurological[12]; Psychological[12, 13]; Respiratory[13, 25]; Skin[01, 08, 12, 13, 23, 25]; Urological[12, 13, 22]; Pregnancy[22, 26]; Female genital[01, 12, 13, 23, 26]; General and Unspecified[01, 12, 13, 22, 23, 25]
Cognates:
Language contact:

Malvastrum (Malvaceae)

Spanish names: Malva
Indigenous names: Thipon, manath thipon[07]; Lantha thipon[07]
Used by (2*): Zoque[03]; Huastec[07]
Used for (5#): Digestive[07]; Psychological[03]; Skin[07]; General and Unspecified[07]
Cognates:
Language contact:

Malvaviscus (Malvaceae)

Spanish names: Amapola; Chavelita del monte; Monacillo; Monasillo, Ombligo de Caballo, Hierba Lengua; Orín de diablo/tulipán/Chavelita de jardín; Rompe olla; Sibí, sibil; Tres colores; Tulipancito del rio
Indigenous names: Kin nyäpin[01]; Tzyi juts[02]; Xoun pocuy[03]; Xuuxy aay[04]; Ix bek'em, thoot wits[07]; Bisil-che', Bisil, Holol[09]; Ru kotzij tzunum, Muxu'xkej, Räq' Q'os Aq'om[12]; Oxib Xbonol[14]; Yopo'aj ts'ibi[18]; Tulipan duendi[21]
Used by (12*): Zoque[01, 02, 03]; Mixe[04]; Huastec[07]; Yucatecan Maya[09]; Quichean Maya[12, 13, 14]; Western Maya[18]; Zapotec[21]; Nahua[26]
Used for (39#): Digestive[01, 02, 03, 04, 07, 09, 12, 13, 18]; Eye[18]; Musculoskeletal[01, 07]; Respiratory[01, 03, 07, 21, 26]; Skin[03, 12, 18, 21, 26]; Endocrine[14]; Urological[02, 03, 21]; Pregnancy[04, 07]; Female genital[03, 07, 12]; General and Unspecified[01, 03, 07, 12, 13, 21]
Cognates:
Language contact:

***Mandevilla* (Apocynaceae)**

Spanish names:
Indigenous names: Katx muk[03]
Used by (1*): Zoque[03]
Used for (1#): Skin[03]
Cognates:
Language contact:

***Manfreda* (Asparagaceae)**

Spanish names: Asosena sabanera
Indigenous names: Copa asosena[03]; Pulik k'oyol, k'oyol ist'aamal, eemil kw'a'[07]; Pets'k'im, Pets'k'inil[09]
Used by (3*): Zoque[03]; Huastec[07]; Yucatecan Maya[09]
Used for (6#): Neurological[07, 09]; Respiratory[07]; Skin[03, 07]; Pregnancy[03]
Cognates:
Language contact:

***Mangifera* (Anacardiaceae)**

Spanish names: Mango
Indigenous names: Manku[03]; Mang aay[04]; Q'anatz'ub'[12]; Ma'nk'[14]; U pam[18]; Malak'[19]; Manko[20]; Yàg-mângw[23]; Maniltzapotl[28]
Used by (15*): Zoque[01, 02, 03]; Mixe[04]; Totonac[06]; Yucatecan Maya[09]; Quichean Maya[12, 13, 14]; Western Maya[18, 19, 20]; Zapotec[21, 23]; Nahua[28]
Used for (38#): Digestive[01, 02, 03, 06, 12, 13, 18, 19, 20, 21]; Cardiovascular[02]; Musculoskeletal[01, 02, 06]; Neurological[03, 18, 23]; Respiratory[02, 03, 12, 14, 19, 21, 28]; Skin[01, 03, 21]; Urological[03]; Pregnancy[09]; Female genital[01, 02, 03]; General and Unspecified[01, 02, 04, 09, 12, 21]
Cognates:
Language contact:

***Manihot* (Euphorbiaceae)**

Spanish names: Yuca; Yucca
Indigenous names: Pisi[01]; T'inche'[07]; Ts'iim[09]; Tzín[13]; Tz'in[18]; Guyaajaca'a[21]
Used by (7*): Zoque[01, 03]; Huastec[07]; Yucatecan Maya[09]; Quichean Maya[13]; Western Maya[18]; Zapotec[21]
Used for (10#): Digestive[01, 03, 07, 13, 18]; Skin[09]; Female genital[18]; General and Unspecified[01, 09, 21]
Cognates: Maya: tin/tzin/tzim; CoreM: tzin/tzim;
Language contact: Zoq <> Maya

***Manilkara* (Sapotaceae)**

Spanish names: Chicosapote; Chicozapote; Sunzapote; Zapote; Zapote, Chicle-zapote
Indigenous names: Ji'ya[01]; Jiya[02]; Jiya[03]; Skalu jaka[05]; Ak'so sual[06]; Tsab it'ath[07]; Ya'[09]; Ya[10]; Chäbte'[18]; Wolja'as[18]; Guil ziji[21]
Used by (12*): Zoque[01, 02, 03]; Totonac[05, 06]; Huastec[07]; Yucatecan Maya[09, 10]; Quichean Maya[13]; Western Maya[18]; Zapotec[21]; Nahua[24]
Used for (21#): Digestive[06, 07, 09, 10, 13, 18, 21]; Cardiovascular[01, 02]; Psychological[02]; Skin[01, 03, 05]; Endocrine[18]; Urological[03]; Female genital[03, 24]; Male genital[01]; General and Unspecified[21]
Cognates: Zoq: jiya; Yuc: ya;
Language contact: Zoq > Tot & Hua & Tabasco Chontal & Yuc & Zap

***Mansoa* (Bignoniaceae)**

Spanish names: Hierba de ajo/hoja de ajo
Indigenous names: Asyus ay[01]; Aaaxux ts'aah[07]
Used by (2*): Zoque[01]; Huastec[07]
Used for (7#): Digestive[07]; Musculoskeletal[01, 07]; Respiratory[01]; Skin[07]; General and Unspecified[01, 07]
Cognates:
Language contact:

***Maranta* (Marantaceae)**

Spanish names: Hoja de Sahgún
Indigenous names: Uaja[03]; T'aaw', tsakam thulup[07]; T'aaw' ok[07]; Chaak[09]
Used by (4*): Zoque[03]; Totonac[06]; Huastec[07]; Yucatecan Maya[09]
Used for (10#): Digestive[06, 07, 09]; Skin[03]; Urological[06, 07]; Female genital[07]; General and Unspecified[06, 07]
Cognates:
Language contact:

***Marathrum* (Podostemaceae)**

Spanish names:
Indigenous names: N¥muk, Niu muk[03]
Used by (1*): Zoque[03]
Used for (3#): Skin[03]; Female genital[03]; General and Unspecified[03]
Cognates:
Language contact:

***Marchantia* (Marchantiaceae)**

Spanish names:
Indigenous names: Miim ha'[07]
Used by (1*): Huastec[07]
Used for (2#): Skin[07]; General and Unspecified[07]
Cognates:
Language contact:

***Margaranthus* (Solanaceae)**

Spanish names: Totomache
Indigenous names: Tuthaayil an t'iiw[07]
Used by (2*): Huastec[07]; Nahua[27]
Used for (4#): Digestive[07, 27]; Psychological[27]; Endocrine[27]
Cognates:
Language contact:

***Margaritopsis* (Rubiaceae)**

Spanish names:
Indigenous names: Ketsu ts'ohool, wiichab ts'ohool, pulik puut' ts'aah[07]; Xbakalik'[09]
Used by (2*): Huastec[07]; Yucatecan Maya[09]
Used for (3#): Neurological[09]; Skin[07]; General and Unspecified[07]
Cognates:
Language contact:

***Marina* (Fabaceae)**

Spanish names:
Indigenous names: Tiith olom[07]
Used by (1*): Huastec[07]
Used for (1#): General and Unspecified[07]
Cognates:
Language contact:

Marrubium **(Lamiaceae)**

Spanish names: Marrubia; Marrubio
Indigenous names: Pition gihš[22]; Tzopiloshihuitl[28]
Used by (5*): Zapotec[22, 23]; Nahua[26, 27, 28]
Used for (14#): Digestive[23, 26, 27, 28]; Musculoskeletal[23]; Neurological[28]; Psychological[26, 27]; Skin[22, 27, 28]; Urological[28]; General and Unspecified[27, 28]
Cognates:
Language contact:

Marsdenia **(Apocynaceae)**

Spanish names: Yerba santa del campo
Indigenous names: Tan ooy[07]; Bala šoh gihš[22]
Used by (2*): Huastec[07]; Zapotec[22]
Used for (6#): Musculoskeletal[22]; Neurological[22]; Respiratory[22]; Skin[07, 22]; General and Unspecified[22]
Cognates:
Language contact:

Martynia **(Martyniaceae)**

Spanish names: Uña de gato; Uña de gato, Carnavalia, Sarsaparilla
Indigenous names: Joun k¥ts¥s[03]; Itsik' kuxkum[07]; Rixiij miss'[14]
Used by (6*): Zoque[01, 02, 03]; Huastec[07]; Yucatecan Maya[09]; Quichean Maya[14]
Used for (14#): Digestive[14]; Eye[02]; Cardiovascular[02]; Musculoskeletal[02]; Respiratory[07]; Endocrine[01, 02]; Urological[09]; Female genital[03, 14]; Male genital[02]; General and Unspecified[02, 03, 07]
Cognates:
Language contact:

Matayba **(Sapindaceae)**

Spanish names:
Indigenous names: Pix pix cuy[03]; Säk uayum[11]
Used by (2*): Zoque[03]; Yucatecan Maya[11]
Used for (3#): Digestive[03]; Skin[11]; Female genital[03]
Cognates:
Language contact:

Matelea **(Apocynaceae)**

Spanish names:
Indigenous names: Piin-k'ak', Kuyuch-ak', Xp'okini, Emtsul[09]
Used by (1*): Yucatecan Maya[09]
Used for (1#): Skin[09]
Cognates:
Language contact:

Matricaria **(Asteraceae)**

Spanish names: Manzanilla; Manzanilla (dulce); Manzanilla dulce; Manzanilla, camomilla
Indigenous names: Manzaniya[07]; Ix bek'em paktda'[08]; Mar'san la', mazne'y[13]; Mantzaniya[20]; Manzaniy neš[22]; Guièe-mànzànî[23]
Used by (20*): Zoque[01, 02, 03]; Mixe[04]; Totonac[05, 06]; Huastec[07, 08]; Quichean Maya[12, 13, 14]; Western Maya[19, 20]; Zapotec[21, 22, 23]; Nahua[25, 26, 27, 28]
Used for (79#): Blood[12]; Digestive[01, 02, 03, 04, 05, 06, 07, 08, 12, 13, 20, 21, 22, 23, 25, 26, 27, 28]; Eye[01, 02, 08, 13, 27, 28]; Ear[04, 23]; Cardiovascular[08, 12]; Musculoskeletal[01, 03, 12, 13, 19]; Neurological[01, 12, 13, 19, 22, 28]; Psychological[02, 03, 12, 13]; Respiratory[01, 02, 08, 13, 14, 19, 25, 27]; Skin[04, 21, 26]; Urological[12, 22]; Pregnancy[01, 02, 03, 13, 19, 21, 22]; Female genital[01, 02, 12, 13, 19]; Male genital[01, 12]; General and Unspecified[01, 02, 08, 12, 19, 22, 27]
Cognates:
Language contact:

***Maxillariella* (Orchidaceae)**

Spanish names:
Indigenous names: Kowa nokcha[03]
Used by (1*): Zoque[03]
Used for (1#): Digestive[03]
Cognates:
Language contact:

***Maytenus* (Celastraceae)**

Spanish names: Retamo
Indigenous names: Ñiwi cuy [03]
Used by (1*): Zoque[03]
Used for (6#): Digestive[03]; Neurological[03]; Skin[03]; Urological[03]; Pregnancy[03]; Female genital[03]
Cognates:
Language contact:

***Mecardonia* (Plantaginaceae)**

Spanish names: Chotete
Indigenous names: Xotete[03]; Ncuàan-dzéb-maêstr, ncuàan-dzéb-ròo, ncuàan-dzéb-guièel[23]; Tlaquexahuil[26]
Used by (3*): Zoque[03]; Zapotec[23]; Nahua[26]
Used for (5#): Blood[03]; Skin[26]; Female genital[03]; General and Unspecified[03, 23]
Cognates:
Language contact:

***Medicago* (Fabaceae)**

Spanish names: Alfalfa
Indigenous names: Alfâlf, guìzh-frôw, guìzh-àlfâlf[23]
Used by (3*): Quichean Maya[13]; Zapotec[23]; Nahua[28]
Used for (4#): Psychological[23]; Respiratory[13]; Endocrine[28]; Urological[28]
Cognates:
Language contact:

***Melampodium* (Asteraceae)**

Spanish names: Acahuale amarillo; Azaján Criollo; Hoja de azar
Indigenous names: Tza'a tzoy[01]; Smucucu makatama[05]; R'u Wai kej[12]
Used by (3*): Zoque[01]; Totonac[05]; Quichean Maya[12]
Used for (5#): Digestive[01]; Cardiovascular[05]; Musculoskeletal[12]; General and Unspecified[01, 12]
Cognates:
Language contact:

***Melanthera* (Asteraceae)**

Spanish names: Rosita
Indigenous names:
Used by (1*): Nahua[26]
Used for (1#): Respiratory[26]
Cognates:
Language contact:

Melia **(Meliaceae)**

Spanish names: Paraís; Paraíso; Piocha; Tarai
Indigenous names: Yàg-pàraîs[23]
Used by (5*): Zoque[03]; Totonac[05]; Western Maya[18, 19]; Zapotec[23]
Used for (18#): Blood[03]; Digestive[05, 23]; Ear[03]; Musculoskeletal[03]; Neurological[19]; Skin[03, 18]; Endocrine[03]; Urological[03]; Pregnancy[03, 05]; Female genital[05]; Male genital[05]; General and Unspecified[03, 19, 23]
Cognates:
Language contact:

Melicoccus **(Sapindaceae)**

Spanish names: Guaya
Indigenous names:
Used by (1*): Western Maya[18]
Used for (1#): Digestive[18]
Cognates:
Language contact:

Melilotus **(Fabaceae)**

Spanish names: Trebe
Indigenous names: Alfâlf, guìzh-frôw, guìzh-àlfâlf[23]
Used by (2*): Quichean Maya[12]; Zapotec[23]
Used for (2#): Digestive[12]; Psychological[23]
Cognates:
Language contact:

Melissa **(Lamiaceae)**

Spanish names: Toronjil (de menta)
Indigenous names: Utz[18]
Used by (1*): Western Maya[18]
Used for (6#): Digestive[18]; Neurological[18]; Psychological[18]; Respiratory[18]; Endocrine[18]; General and Unspecified[18]
Cognates:
Language contact:

Melochia **(Malvaceae)**

Spanish names: Malva morada; Malva rosada; Malvarisco morado
Indigenous names: Tsakam akich, tsabaal akich[07]; Ehtiil i tsakam akich[07]; Lexuba'a moradu'u[21]
Used by (3*): Zoque[02]; Huastec[07]; Zapotec[21]
Used for (14#): Digestive[02, 07]; Skin[21]; Endocrine[02]; Urological[07]; Pregnancy[07]; Female genital[21]; Male genital[02]; General and Unspecified[02, 07, 21]
Cognates:
Language contact:

Melothria **(Cucurbitaceae)**

Spanish names: Hoja de carga
Indigenous names: Tzämi ay[01]; Tsaiñicut cuy, Xandia tsay[03]; Baleeyail an t'eel[07]; Sandia cho[16]; Sandi'a ch'o[17]
Used by (5*): Zoque[01, 03]; Huastec[07]; Quichean Maya[16, 17]
Used for (6#): Digestive[07]; Neurological[16]; Psychological[03]; Urological[03]; General and Unspecified[01]; nd[17]
Cognates:
Language contact:

***Mendoncia* (Acanthaceae)**

Spanish names:
Indigenous names:
Used by (1*): Quichean Maya[17]
Used for (1#): nd[17]
Cognates:
Language contact:

***Mentha* (Lamiaceae)**

Spanish names: Hierba buena; Hierbabuena; Menta; Menta, Balsamo; Monstranza; Mostranza; Poleo ; Poleo, pompimi; Toronjil criollo; Toronjil, Balsamo; Toronjil/hierbabuena de mentha; Yerba buena; Yerbabuena
Indigenous names: Yepena[01]; Ujts aay[04]; Castalatka'jna[05]; Caxtalaalhka 'jna', ihpupuhui'na[06]; Elbeenax[07]; Elbenax[08]; Xuiky[08]; Xuuikh[08]; Arvino, q'ebun, purxil', alwino, pärxil, esal guach pärxil[13]; Bänälä utz[18]; Utz[18]; Wena[20]; Bedxestila'a[21]; Bäč štil[22]; Guìzh-mònstânz[23]; Tlanixalnilki[26]; Kallowena[28]
Used by (23*): Zoque[01, 02, 03]; Mixe[04]; Totonac[05, 06]; Huastec[07, 08]; Yucatecan Maya[09, 11]; Quichean Maya[12, 13, 14]; Western Maya[18, 19, 20]; Zapotec[21, 22, 23]; Nahua[25, 26, 27, 28]
Used for (103#): Digestive[01, 02, 03, 04, 05, 06, 07, 08, 09, 11, 12, 13, 14, 18, 19, 20, 21, 22, 25, 27, 28]; Ear[19]; Cardiovascular[01, 08, 13, 21]; Musculoskeletal[01, 08, 12, 14, 28]; Neurological[01, 06, 09, 12, 18, 28]; Psychological[01, 08, 12, 18]; Respiratory[01, 02, 05, 08, 12, 13, 14, 18, 19, 23, 27]; Skin[26]; Urological[01, 12, 13]; Pregnancy[03, 05, 07, 13, 19, 23]; Female genital[01, 02, 06, 12, 13, 18, 19]; Male genital[01]; General and Unspecified[01, 03, 05, 08, 12, 13, 14, 18, 21, 25, 26]
Cognates:
Language contact:

***Mentzelia* (Loasaceae)**

Spanish names: Mala mujer
Indigenous names: Thekw'em ch'ohool. Itsaan yakw'ab wahuts[07]; Yàg-làa[23]; Guìzh-nàad, guièe-nàad[23]
Used by (3*): Huastec[07]; Zapotec[23]; Nahua[24]
Used for (5#): Psychological[07]; Skin[07, 23]; Pregnancy[23, 24]
Cognates:
Language contact:

***Merremia* (Convolvulaceae)**

Spanish names:
Indigenous names: Piith ts'aah[07]; Chuthat po'eel, man ch'aah[07]; San Diego ts'ohool, chuthat pok'eel[07]; Is k'aham[17]
Used by (2*): Huastec[07]; Quichean Maya[17]
Used for (8#): Ear[07]; Neurological[07]; Skin[07]; General and Unspecified[07]; nd[17]
Cognates:
Language contact: Hua <> Kekchí

***Metastelma* (Apocynaceae)**

Spanish names:
Indigenous names: Xîum-ak'[09]
Used by (2*): Zoque[03]; Yucatecan Maya[09]
Used for (3#): Skin[03]; Endocrine[09]; Urological[09]
Cognates:
Language contact:

***Miconia* (Melastomataceae)**

Spanish names: Cinco negritos del monte; Palo dulce; Tescuate
Indigenous names: Pak tesua[03]; Tesua[03]; Chuk jeepe[03]; Jama piits[04]; Sa'ksi qui'hui[06]; Kux Sawi'i[14]; Xoy Q'ehen, Kaq'i pim[14]; Ixq'i Q'ehen [14]; Kux Sawi'i, Tzib' Q'en[14]; Wa'bon'[14]
Used by (6*): Zoque[01, 03]; Mixe[04]; Totonac[06]; Quichean Maya[14, 17]
Used for (30#): Digestive[01, 03, 14]; Neurological[14]; Respiratory[03]; Skin[03, 04, 06, 14]; Urological[03]; Pregnancy[03, 14]; Female genital[03, 14]; General and Unspecified[14]; nd[17]
Cognates:
Language contact: Tot <> Kekchí

***Microgramma* (Polypodiaceae)**

Spanish names:
Indigenous names: Canagual[03]; Chu'ub te', chu'ub ts'aah[07]; Tipte'-ak'[09]
Used by (3*): Zoque[03]; Huastec[07]; Yucatecan Maya[09]
Used for (4#): Digestive[09]; Musculoskeletal[03, 07]; General and Unspecified[07]
Cognates:
Language contact:

***Micromeria* (Lamiaceae)**

Spanish names: Hierba buena
Indigenous names: Bìt-wàd, bìt-xtîl[23]
Used by (1*): Zapotec[23]
Used for (1#): Digestive[23]
Cognates:
Language contact:

***Micropleura* (Apiaceae)**

Spanish names: Valeriana
Indigenous names: Valeriana[13]; Nich nab momol, nich nab tz'i' lel vomol, vomolal vo', makmak nab wamal, , bokol chuch wamal, bikil ch'o wamal[20]
Used by (2*): Quichean Maya[13]; Western Maya[20]
Used for (5#): Digestive[20]; Neurological[13, 20]; Pregnancy[20]; Female genital[20]
Cognates:
Language contact:

***Microsechium* (Cucurbitaceae)**

Spanish names: Amole, tumba vaquero; Yerba de raton, oreja de raton
Indigenous names: Škwam bizin[22]; Bià-tòo, Ibæ̀-bià-tòo[23]
Used by (2*): Zapotec[22, 23]
Used for (5#): Neurological[23]; Psychological[23]; Skin[22, 23]; General and Unspecified[23]
Cognates:
Language contact:

***Mikania* (Asteraceae)**

Spanish names: Hoja de carga; Tabardillo
Indigenous names: Tzämi tzoy tzämi ay[01]; Wako[07]; Cha'onoob'[17]
Used by (5*): Zoque[01, 03]; Huastec[07]; Quichean Maya[17]; Western Maya[19]
Used for (7#): Digestive[07]; Neurological[19]; Skin[07]; Female genital[03]; General and Unspecified[01, 19]; nd[17]
Cognates:
Language contact:

***Milla* (Asparagaceae)**

Spanish names: Lirio
Indigenous names:
Used by (1*): Quichean Maya[12]
Used for (2#): Neurological[12]; Urological[12]
Cognates:
Language contact:

***Milleria* (Asteraceae)**

Spanish names:
Indigenous names: Xø pøh[04]; Xontolok[09]
Used by (2*): Mixe[04]; Yucatecan Maya[09]
Used for (2#): Skin[04, 09]
Cognates:
Language contact: Mixe <> Yuc

***Mimosa* (Fabaceae)**

Spanish names: Adormidero, Zarza Dormilona; Corona de cristo, dormidillo; Dormilona; Duermidillo; Espina dormilona, hierba dormilona; Huaje rosadito, la vergonzosa; La vergonzosa/dormilona; Pinahuixtle; Sarsa sabanera; Tepehuaje cimarron; Tepesquehuite; Tepezcohuite; Tepezcohuite/Pezquehuite; Uña de gato; Uña de gato/espina dormilona/dormilona; Vergonzosa; Vergonzosa grande; Vergonzosa, meramera; Zarza; Zarza Dormilona
Indigenous names: Misyu kä[01]; Pik awit/ anhgen yäki/ äksy awit/ awit jäyä[01]; Ä' yäwi/ä' wewo tane/ok yäwi [01]; Mo'ay sake ma'syi/ tzasyäkuy ay[02]; Jupupi[02]; Nuk cuy[03]; Mong mong ay[03]; Ma'ap ujts[04]; Tančahuat[05]; Chobeem[07]; Tsobeem, wayma'[07]; Sak-katsim, Katsim[09]; Šmu¢'ic[10]; Ix mutz[11]; Xu'l Xpe Quej, Q'ix[12]; Xul q'ies, xul kiej, Cxal kij[13]; Wara Q'ix[14]; Quare kix[16]; Ajwäye[18]; Guedzegumaj'alaj[21]; Gueedxe boog[21]; Guièts-làa-tó, guìzh-lòo-tó], guièts-guièe-tó[23]; Yàg-guièts-nàad[23]; Xohuoctihuistle[26]; Pinahuitz[28]
Used by (20*): Zoque[01, 02, 03]; Mixe[04]; Totonac[05]; Huastec[07]; Yucatecan Maya[09, 10, 11]; Quichean Maya[12, 13, 14, 15, 16]; Western Maya[18]; Zapotec[21, 23]; Nahua[26, 27, 28]
Used for (80#): Digestive[01, 03, 05, 12, 21, 23, 26, 28]; Eye[03]; Musculoskeletal[01, 12]; Neurological[02, 12, 14]; Psychological[01, 02, 03, 04, 07, 10, 12, 14, 15, 16, 18, 21]; Respiratory[03, 09, 18, 26]; Skin[01, 02, 03, 07, 12, 13, 21, 28]; Endocrine[01, 27, 28]; Urological[01, 12, 21]; Pregnancy[02, 07, 12, 15, 26]; Female genital[03, 13]; General and Unspecified[01, 02, 03, 11, 12, 14, 18, 21, 23, 28]
Cognates: Zoq: mo' ay / mong ay; Yuc: xmutz; Quich: xul kej, (q'ix);
Language contact: Chiapas Zoq > Tabasco Chontal

***Mimulus* (Phrymaceae)**

Spanish names: Verdolaga
Indigenous names:
Used by (1*): Quichean Maya[13]
Used for (1#): General and Unspecified[13]
Cognates:
Language contact:

***Mirabilis* (Nyctaginaceae)**

Spanish names: Flor de linda tarde; Hoja de linda tarde, flor de china; Maravilla; Maravilla/ mañanita/ flor de noche
Indigenous names: Tzuj jäyä[01]; Txuang mooya[03]; tsu pøh[04]; Ch'uyeem[07]; Guièe-màràvî, guièe-mòròvî[23]
Used by (11*): Zoque[01, 02, 03]; Mixe[04]; Huastec[07]; Yucatecan Maya[10, 11]; Quichean Maya[13]; Western Maya[18]; Zapotec[21, 23]
Used for (31#): Digestive[02, 07, 23]; Eye[02]; Musculoskeletal[01, 02, 11, 21]; Neurological[02, 18]; Psychological[01, 03, 13]; Respiratory[01, 02, 07, 21]; Skin[01, 02, 04, 10, 18, 21]; Female genital[02]; General and Unspecified[01, 03, 07, 11, 18, 21]
Cognates: MZ: tsu/txu;
Language contact: MZ > Hua

***Mollinedia* (Monimiaceae)**

Spanish names:
Indigenous names: Sakim pim[16]; Sak'i k'ejen[17]
Used by (2*): Quichean Maya[16, 17]
Used for (2#): Neurological[16]; nd[17]
Cognates:
Language contact:

***Mollugo* (Molluginaceae)**

Spanish names:
Indigenous names: Billushít[22]
Used by (1*): Zapotec[22]
Used for (1#): Digestive[22]
Cognates:
Language contact:

***Momordica* (Cucurbitaceae)**

Spanish names: Condiamor; Cu'undu amor; Cundeamor; Cundiamor; Cunduamor; Manzanina; Manzanita, flor de chino; Sandía de Ratón, Jaime; Sorosi
Indigenous names: Paks ko'kinä[02]; Kundiamor[03]; Pa xandia[04]; Morax[09]; Sandiy'cho[14]; Ya'mor[17]; Kwnyamor[18]
Used by (12*): Zoque[01, 02, 03]; Mixe[04]; Yucatecan Maya[09, 10, 11]; Quichean Maya[14, 15, 17]; Western Maya[18]; Zapotec[21]
Used for (33#): Blood[10]; Digestive[02, 11]; Musculoskeletal[02, 03, 14]; Neurological[04]; Respiratory[02, 04]; Skin[02, 03, 09, 18, 21]; Endocrine[01, 02, 03, 10, 11, 14, 18, 21]; Urological[03, 11]; Pregnancy[03]; Female genital[02, 15]; Male genital[18]; General and Unspecified[02, 04, 11, 14]; nd[17]
Cognates:
Language contact:

***Monnina* (Polygalaceae)**

Spanish names: San Benito, tintiamora
Indigenous names: Cha xu q'an[13]
Used by (1*): Quichean Maya[13]
Used for (1#): Eye[13]
Cognates:
Language contact:

***Monochaetum* (Melastomataceae)**

Spanish names:
Indigenous names: Cacaloxihuitle[26]
Used by (2*): Zoque[03]; Nahua[26]
Used for (5#): Digestive[26]; Respiratory[26]; Skin[03]; Female genital[26]; General and Unspecified[03]
Cognates:
Language contact:

***Monstera* (Araceae)**

Spanish names: Hoja de corazón; Tripa de pollo
Indigenous names: Anma ay[03]; Xunujti tsay[03]; Chibaba[21]
Used by (2*): Zoque[03]; Zapotec[21]
Used for (4#): Digestive[03]; Skin[03]; General and Unspecified[21]
Cognates:
Language contact:

***Montanoa* (Asteraceae)**

Spanish names: Acahuite; Chamizo negro; Flor de papera, flor de teresita; Penumbre
Indigenous names: Xtankas- ak', Xuxtankas[09]; Yag tsun[22]; Yàg-yàaz-yâas, yàg-yàaz-ngǎs[23]
Used by (7*): Zoque[01, 03]; Yucatecan Maya[09]; Zapotec[21, 22, 23]; Nahua[27]
Used for (13#): Digestive[01, 21, 22, 23]; Musculoskeletal[01]; Skin[03, 21, 23]; Pregnancy[23]; General and Unspecified[09, 21, 22, 27]
Cognates:
Language contact: Yuc <> Zap

Morella **(Myricaceae)**

Spanish names: Arrayana/laurel simarron; Garrote de viejito, raijan, arrayan
Indigenous names: Ot kamay/ jotomgay kamay [01]; Copa puso[03]; Pom che', uch'mix Tiox[13]; Sera te'[20]; Ahuaxochitl[25]; Xochicuahuitl[26]
Used by (6*): Zoque[01, 03]; Quichean Maya[13]; Western Maya[20]; Nahua[25, 26]
Used for (18#): Digestive[01, 13, 20]; Cardiovascular[25]; Musculoskeletal[01, 13]; Neurological[13]; Psychological[01]; Respiratory[03]; Pregnancy[01, 13]; Female genital[03, 13]; General and Unspecified[01, 03, 13, 25, 26]
Cognates:
Language contact:

Morinda **(Rubiaceae)**

Spanish names: Noni; Noni/cancero; Nonis; Piñita
Indigenous names: Piña ak', Piña kam[09]; K'an i che'[17]
Used by (7*): Zoque[01, 02]; Yucatecan Maya[09, 11]; Quichean Maya[12, 14, 17]
Used for (13#): Digestive[02]; Cardiovascular[02, 11]; Skin[09]; Endocrine[02, 14]; Urological[01, 02]; Pregnancy[01]; General and Unspecified[01, 02, 12]; nd[17]
Cognates:
Language contact:

Moringa **(Moringaceae)**

Spanish names: Jacinto; Moringa; San Jacinto
Indigenous names:
Used by (4*): Zoque[02]; Quichean Maya[12, 14]; Zapotec[21]
Used for (8#): Digestive[12]; Musculoskeletal[02, 21]; Respiratory[02]; Endocrine[12, 14]; General and Unspecified[12, 21]
Cognates:
Language contact:

Mortoniodendron **(Malvaceae)**

Spanish names:
Indigenous names: Kaq'i B'ach[14]
Used by (1*): Quichean Maya[14]
Used for (2#): Pregnancy[14]; Female genital[14]
Cognates:
Language contact:

Mosannona **(Annonaceae)**

Spanish names: Lemoy
Indigenous names: Elemuy[09]
Used by (2*): Yucatecan Maya[09, 11]
Used for (3#): Endocrine[09]; Urological[09]; General and Unspecified[11]
Cognates:
Language contact:

Mosquitoxylum **(Anacardiaceae)**

Spanish names: Cedro nogal
Indigenous names: Se'mpe[03]
Used by (1*): Zoque[03]
Used for (2#): Female genital[03]; General and Unspecified[03]
Cognates:
Language contact:

Mouriri **(Melastomataceae)**

Spanish names:
Indigenous names: Chak B'olay Q'ehen[14]
Used by (1*): Quichean Maya[14]
Used for (2#): Digestive[14]; Skin[14]
Cognates:
Language contact:

Moussonia **(Gesneriaceae)**

Spanish names: Cerbatanera; Hierba de zopilote
Indigenous names: Ajpu Q'ehen[14]; Cacahuatón[26]
Used by (2*): Quichean Maya[14]; Nahua[26]
Used for (5#): Digestive[26]; Respiratory[26]; Endocrine[26]; Female genital[26]; General and Unspecified[14]
Cognates:
Language contact:

Mucuna **(Fabaceae)**

Spanish names: Nescafé; Nescafé, picapica; Ojo de venado; Pica pica
Indigenous names: Kunkun[02]; Mooxoox[07]; Neskaape, tsanakw' thut'[07]; Xpica[09]
Used by (7*): Zoque[01, 02, 03]; Huastec[07]; Yucatecan Maya[09]; Quichean Maya[13]; Zapotec[21]
Used for (12#): Digestive[07, 09]; Eye[13]; Cardiovascular[01]; Neurological[02, 07]; Psychological[03]; Respiratory[07]; Skin[07]; General and Unspecified[07, 21]
Cognates:
Language contact:

Muehlenbeckia **(Polygonaceae)**

Spanish names: Cola de cabello
Indigenous names:
Used by (1*): Nahua[26]
Used for (2#): Musculoskeletal[26]; Urological[26]
Cognates:
Language contact:

Muntingia **(Muntingiaceae)**

Spanish names: Capulín
Indigenous names: Mukpe/juj kuy[01]; Mupe[02]; Capuli[03]; Mujt[04]; Tsakam puwaamte', puam[07]; Pujam[18]; Mbe'e ze'e[21]
Used by (7*): Zoque[01, 02, 03]; Mixe[04]; Huastec[07]; Western Maya[18]; Zapotec[21]
Used for (19#): Digestive[02, 03, 18, 21]; Musculoskeletal[02]; Respiratory[01, 02, 21]; Skin[03, 04, 18, 21]; Urological[03]; Pregnancy[01, 07]; Female genital[03]; General and Unspecified[02, 04, 21]
Cognates: MZ: mu; Zoq: mu(k)pe; Maya: pu(j)am;
Language contact:

Murraya **(Rutaceae)**

Spanish names: Limonaria; Muraya
Indigenous names:
Used by (2*): Zoque[03]; Yucatecan Maya[09]
Used for (2#): Neurological[03]; Respiratory[09]
Cognates:
Language contact:

***Musa* (Musaceae)**

Spanish names: Guineo; Muide; Platano; Plátano (macho), yopin tzup, Plátano roatán; Platano blanco, guineo, platano manzano, platano keny, platano roatan; Platano cherimoya, platano pera, platano bolsa; Platano rojo; Platano, guineo, banano; Platano/guineo
Indigenous names: Tsapuo [01]; Patanus/patanusy/kineya[02]; Hoko samñi[03]; Ka'ak[04]; See'kna [06]; See'kna, tet'see'kann[06]; It'ath[07]; Costillon it'adh[08]; Ha'as[09]; Inkiney, skul[13]; Xcol'itul, Ik B'olay Q'ehen [14]; Ja'as, Ajruatan[18]; Kene'[19]; Biduáj[21]
Used by (15*): Zoque[01, 02, 03]; Mixe[04]; Totonac[06]; Huastec[07, 08]; Yucatecan Maya[09]; Quichean Maya[12, 13, 14]; Western Maya[18, 19]; Zapotec[21]; Nahua[24]
Used for (38#): Digestive[01, 02, 03, 04, 06, 07, 09, 13, 14, 18, 19, 21]; Psychological[01, 18]; Respiratory[06, 21]; Skin[01, 02, 03, 06, 07, 13, 14, 18, 21]; Endocrine[18]; Urological[06]; Pregnancy[24]; Female genital[01, 21]; Male genital[01]; General and Unspecified[01, 02, 08, 12, 14, 21]
Cognates:
Language contact:

***Myrcianthes* (Myrtaceae)**

Spanish names:
Indigenous names: Cheks [03]
Used by (1*): Zoque[03]
Used for (1#): Digestive[03]
Cognates:
Language contact:

***Myriocarpa* (Urticaceae)**

Spanish names: Chalagoge, panza de burro; Pegasoso/palo de barba/caracolillo/ la barbosa
Indigenous names: (Mo'a) siskuy[01]; Tzoki 'an panatz/tzok yan panats[01]; Nä ninki[02]
Used by (2*): Zoque[01, 02]
Used for (5#): Digestive[01]; Musculoskeletal[02]; Skin[01]; Urological[01]; General and Unspecified[01]
Cognates:
Language contact:

***Myristica* (Myristicaceae)**

Spanish names: Nuez moscada; Nuez mozcada
Indigenous names:
Used by (3*): Zoque[01, 02, 03]
Used for (14#): Digestive[01, 02, 03]; Musculoskeletal[02]; Psychological[01, 03]; Respiratory[01]; Skin[02, 03]; Pregnancy[01, 03]; Female genital[03]; General and Unspecified[01, 03]
Cognates:
Language contact:

***Myroxylon* (Apiaceae)**

Spanish names: Arbol de balsa; Balsamo de Peru; Bálsamo negro; Mirrha; Mirto
Indigenous names: Balsa cuy[03]; Uxum ts'ojol[08]
Used by (5*): Zoque[01, 03]; Mixe[04]; Huastec[08]; Quichean Maya[12]
Used for (13#): Digestive[08, 12]; Ear[08]; Cardiovascular[03]; Musculoskeletal[03, 04]; Neurological[08]; Respiratory[12]; Skin[03, 08]; General and Unspecified[01, 04, 12]
Cognates:
Language contact:

***Nasturtium* (Brassicaceae)**

Spanish names: Berro
Indigenous names: Uwi suk', chaj patre[13]
Used by (4*): Zoque[03]; Totonac[06]; Quichean Maya[13]; Zapotec[21]
Used for (11#): Blood[06, 13]; Digestive[06, 21]; Eye[13]; Respiratory[06, 13]; Skin[03]; General and Unspecified[06, 13, 21]
Cognates:
Language contact:

***Nectandra* (Lauraceae)**

Spanish names: Laurel
Indigenous names: Ohte'[07]; Chajom Che'[14]; Ahuacachile[24]
Used by (4*): Huastec[07]; Quichean Maya[14]; Nahua[24, 26]
Used for (5#): Digestive[07, 14]; Psychological[26]; Skin[07]; Pregnancy[24]
Cognates:
Language contact:

***Neea* (Nyctaginaceae)**

Spanish names:
Indigenous names: Tsabats masan ay[03]; K'ak'al xeklek, k'ak'al ilaal[07]; Xtatsim[09]
Used by (3*): Zoque[03]; Huastec[07]; Yucatecan Maya[09]
Used for (4#): Skin[03, 09]; General and Unspecified[03, 07]
Cognates:
Language contact:

***Neomillspaughia* (Polygonaceae)**

Spanish names:
Indigenous names: Sakitsa', Xtastabin[09]
Used by (1*): Yucatecan Maya[09]
Used for (2#): Respiratory[09]; Skin[09]
Cognates:
Language contact:

***Nephrolepis* (Nephrolepidaceae)**

Spanish names: Calahuala del Quetzal; Cola de Quetzal; Huechamacho
Indigenous names: Kalaguala Ruje'y Maq'uq'[12]; Rix Ixul, Ch'upil Q'en[14]; Xqu'uq mo'coch[16]; Y qu'q moco'ch[17]
Used by (5*): Quichean Maya[12, 14, 16, 17]; Nahua[26]
Used for (8#): Digestive[12, 26]; Neurological[16]; Psychological[14]; Skin[14]; General and Unspecified[12, 14]; nd[17]
Cognates:
Language contact:

***Nerium* (Apocynaceae)**

Spanish names: Clabel; Laurel
Indigenous names: Pitx mooya[03]
Used by (3*): Zoque[03]; Zapotec[21]; Nahua[28]
Used for (7#): Musculoskeletal[03]; Neurological[03]; Respiratory[21, 28]; Skin[21]; Female genital[21]; General and Unspecified[28]
Cognates:
Language contact:

***Neurolaena* (Asteraceae)**

Spanish names: Cola de faisán; Crementina; Mano lagarto; Tres puntas; Tres puntas, mano de lagarto
Indigenous names: T'unu' ix bek'em[07]; Kayabim[10]; K'aman, c'a mank[13]; K'a' Mank'[14]; Kamank, gan mank[15]; K'a mank[17]; Ch'ajch'aj k'opot[19]
Used by (11*): Zoque[02, 03]; Huastec[07]; Yucatecan Maya[10, 11]; Quichean Maya[13, 14, 15, 17]; Western Maya[19]; Nahua[26]
Used for (19#): Digestive[03, 14]; Musculoskeletal[14]; Neurological[19]; Skin[02, 03, 10, 11, 14, 26]; Endocrine[03]; Pregnancy[07]; Female genital[15]; General and Unspecified[03, 11, 13, 14, 19]; nd[17]
Cognates: Quich: kamank;
Language contact: Quich <> Yuc

01-28 refer to the studies described in Table 4.1 * Total number of studies recording this taxon #Total number of use-records

***Nicandra* (Solanaceae)**

Spanish names: Tomate de Ratón
Indigenous names:
Used by (1*): Quichean Maya[12]
Used for (1#): Skin[12]
Cognates:
Language contact:

***Nicotiana* (Solanaceae)**

Spanish names: Puro; Tabaco; Tabaco (bobo/blanco); Tabaco (verde); Tabaco cimarron; Tabaquillo
Indigenous names: (Tzuj/ tzushy) ozi[01]; Tzä'wi[02]; Ts¥ui[03]; Juiky[04]; Maay[07]; K'uts[09]; K'uuČ[10]; Met', mee'[13]; Mai, Xa'q Mai[14]; C'utz[18]; K'ujtz[19]; Moy, may, bankilal[20]; Guiass yeen[21]; Giasa'a[21]; Mostas[22]; Yàg-brètâyn[23]; Blàg-guièz, blàg-tàbâcw[23]; Nenestlichihuitl[28]
Used by (18*): Zoque[01, 02, 03]; Mixe[04]; Huastec[07]; Yucatecan Maya[09, 10, 11]; Quichean Maya[13, 14]; Western Maya[18, 19, 20]; Zapotec[21, 22, 23]; Nahua[26, 28]
Used for (66#): Digestive[01, 20]; Eye[02]; Ear[01, 02, 03, 23]; Cardiovascular[07, 13]; Musculoskeletal[01, 14, 18, 19, 21, 23, 28]; Neurological[02, 11, 18, 19, 21, 22, 23, 28]; Psychological[01, 19, 20, 21]; Respiratory[01, 02, 10, 11, 13, 20, 21, 23]; Skin[01, 02, 03, 04, 07, 09, 11, 13, 14, 18, 21, 22, 26, 28]; Pregnancy[01, 13]; General and Unspecified[01, 07, 13, 14, 19, 20, 21, 22, 23, 28]
Cognates: Zoq: tsäwi; Maya: k'uts/mai; Yuc: k'uts; Quich: mai/me; WesM: k'uts; Zap: gias;
Language contact:

***Niphidium* (Polypodiaceae)**

Spanish names: Oreja de burro
Indigenous names: Burru tatzo [01]
Used by (1*): Zoque[01]
Used for (1#): Digestive[01]
Cognates:
Language contact:

***Nissolia* (Fabaceae)**

Spanish names:
Indigenous names: Bo' xekel[07]; Xk'ant'uul[09]
Used by (2*): Huastec[07]; Yucatecan Maya[09]
Used for (4#): Neurological[07]; Skin[07, 09]; General and Unspecified[07]
Cognates:
Language contact:

***Nopalea* (Cactaceae)**

Spanish names: Nopal; Nopal, Tuna; Nopallilo
Indigenous names: Nakpat[01]; Kum pasyte[02]; Taat[04]; Aa'xilh[06]; Pak'ak[07]; Pak'ak[08]; Pak'am[09]; Vaas pim[14]; Bia'aj[21]; Yàg-biăa[23]
Used by (14*): Zoque[01, 02, 03]; Mixe[04]; Totonac[06]; Huastec[07, 08]; Yucatecan Maya[09]; Quichean Maya[12, 14]; Zapotec[21, 23]; Nahua[24, 27]
Used for (47#): Blood[03]; Digestive[01, 07, 12, 14, 21, 23]; Cardiovascular[01, 02, 08]; Musculoskeletal[01, 02, 08, 14, 21]; Neurological[01]; Respiratory[01, 06, 09, 21]; Skin[01, 02, 07, 08, 14, 23]; Endocrine[01, 02, 06, 08, 12, 21, 27]; Urological[01, 02, 09, 12]; Pregnancy[04, 07, 24]; Female genital[01, 12]; General and Unspecified[03, 12, 14, 21]
Cognates: MZ: at; Zoq: pat/paxt; Maya: pak; Hua: pak'ak; Zap: bia'a;
Language contact: Zoq <> Maya

***Nymphaea* (Nymphaeaceae)**

Spanish names: Lagua; Repollo
Indigenous names: Pulul[07]; Ch'oop Q'ehen[14]; Lagua[18]
Used by (3*): Huastec[07]; Quichean Maya[14]; Western Maya[18]
Used for (4#): Neurological[07]; Skin[18]; Pregnancy[14]; General and Unspecified[07]
Cognates:
Language contact:

01-28 refer to the studies described in Table 4.1 * Total number of studies recording this taxon #Total number of use-records

***Ocimum* (Lamiaceae)**

Spanish names: Albahaca; Albahaca (cimarrona); Albahaca de monte; Albahaca de tierra; Albahaca morada; albahaca negra/morada; Albahaca simaron; Albahaca simarron/pimentillo; Albahaca, basilica, calamento, hierba real; Albahacar; Especie cimarrón; Hoja de cólico/hierba santa marta/ hierba de cólico; Oregano; Oregano de castill; Oreja, Orégano Castillo; Siempreviva; Tepocía
Indigenous names: Tung an petkuy/tzukspa' tane/näk tane/ tzukin jäyä/yäjkuy tane[01]; Tzukspa'tane [01]; Nas moki pa'a/ moki une[01]; Ay jäyä[02]; Ukä ay jäyä[02]; Xuuik[04]; Paxuuik[04]; Paxcahujnuk[05]; Tsin thekw'eel, chithan thekw'eel, thuutsub, [07]; Laab thekw'eel[07]; Ik ts'ohool[07]; Lap thek'weel[08]; Xkakaltun[09]; Xtem Qana'[14]; Obej', benq[15]; Albajaka[18]; Xuch päm pimi[18]; Chitam wamal, poxil ik', bakel chitam, tza'los wamal, san mikel wamal, ch'aal wamal, yax wamal, xulem te'[20]; Guiasharu'uj[21]; Talachía[24]; Sordoxihuitle[26]
Used by (25*): Zoque[01, 02, 03]; Mixe[04]; Totonac[05]; Huastec[07, 08]; Yucatecan Maya[09, 10, 11]; Quichean Maya[12, 13, 14, 15]; Western Maya[18, 19, 20]; Zapotec[21, 22, 23]; Nahua[24, 25, 26, 27, 28]
Used for (137#): Blood[01]; Digestive[01, 02, 03, 05, 07, 08, 09, 11, 12, 13, 18, 19, 20, 21, 25, 26, 27, 28]; Eye[01, 02, 03, 09, 10, 11, 18, 19]; Ear[01, 02, 03, 04, 13, 14, 19, 23]; Cardiovascular[01, 02, 08, 25]; Musculoskeletal[01, 02, 05, 08, 12, 18, 20, 21]; Neurological[01, 02, 03, 07, 08, 09, 11, 12, 13, 18, 23, 25, 26]; Psychological[01, 05, 07, 12, 21]; Respiratory[01, 02, 03, 08, 18, 20, 25, 27, 28]; Skin[01, 02, 04, 08, 09, 12, 26]; Endocrine[02]; Urological[02, 12]; Pregnancy[02, 03, 05, 08, 19, 24, 26]; Female genital[01, 02, 03, 05, 12, 15, 19, 21]; General and Unspecified[01, 02, 03, 04, 05, 07, 08, 12, 13, 14, 18, 21, 22, 23, 25, 26, 27]
Cognates: Hua: thekw'eel;
Language contact: Chiapas Zoq <> Tot; Mixe <> Tot <> Yuc; Tot <> Tzeltalan

***Ocotea* (Lauraceae)**

Spanish names: Laurel Negro; Laurelillo; Rosa negra
Indigenous names: Moko[03]; Chuch moko[03]
Used by (1*): Zoque[03]
Used for (6#): Digestive[03]; Cardiovascular[03]; Skin[03]; Female genital[03]
Cognates:
Language contact:

***Odontonema* (Acanthaceae)**

Spanish names: Hoja de llanto; Hoja de paperón
Indigenous names: Naktam ay[03]; T'a'lom ts'ohool, k'alul ts'ohool[07]; Sam Ajtzo'[14]; Kux' Luq' Salaq' Baqel', Sam Ajtzo'[14]; Yopo uq'ue[18]
Used by (5*): Zoque[01, 03]; Huastec[07]; Quichean Maya[14]; Western Maya[18]
Used for (13#): Digestive[01]; Ear[03]; Musculoskeletal[03, 14]; Neurological[14]; Respiratory[03]; Skin[03]; General and Unspecified[07, 14, 18]
Cognates:
Language contact:

***Oeceoclades* (Orchidaceae)**

Spanish names: Espalda de la Culebra, Curarina
Indigenous names: Rix li kanti, Xb'an Xmay Ixul[14]
Used by (1*): Quichean Maya[14]
Used for (2#): Skin[14]; General and Unspecified[14]
Cognates:
Language contact:

***Oecopetalum* (Icanicaceae)**

Spanish names: Cacate
Indigenous names: Kuk yaka/ kukyak kawak/ kokya kawa[01]
Used by (1*): Zoque[01]
Used for (3#): Digestive[01]; Psychological[01]; Endocrine[01]
Cognates:
Language contact:

01-28 refer to the studies described in Table 4.1 * Total number of studies recording this taxon #Total number of use-records

***Oenothera* (Onagraceae)**

Spanish names: Hierba azar de mata/hierba de antojo/ hoja de azar fría; Hierba cólica; Hierba del golpe; Hierbita de pollo; Saramella; Tomillo de Montaña
Indigenous names: Tzate kä tane/ pu tzäpäsy häyä/ tz'a tat käyi tane[01]; Piiquaa't xanat[06]; Waakal mo'eel[07]; Waakal mo'eel, thamuul wich[07]; Ts'een waakal mo'eel[07]; Ist'een wakal mo'el[08]; Tomi'y Aq'om K'echelaj[12]; Guièe-zhàn̲-biǎa[23]; Coxcatlacuache[26]; Cupachocotl[28]
Used by (9*): Zoque[01]; Totonac[06]; Huastec[07, 08]; Quichean Maya[12]; Zapotec[23]; Nahua[25, 26, 28]
Used for (31#): Blood[06]; Digestive[07, 12, 25, 26, 28]; Musculoskeletal[07, 08]; Neurological[23, 25]; Psychological[07]; Respiratory[12, 23, 25]; Skin[06, 08, 25, 26, 28]; Urological[26]; Female genital[26]; General and Unspecified[01, 06, 07, 08, 12, 23, 25]
Cognates:
Language contact:

***Olea* (Oleaceae)**

Spanish names: Aceituna; Olivo
Indigenous names:
Used by (2*): Quichean Maya[12, 13]
Used for (3#): Digestive[12]; Pregnancy[13]; General and Unspecified[13]
Cognates:
Language contact:

***Olyra .* (Poaceae)**

Spanish names:
Indigenous names: Xuwan Q'ehen[14]
Used by (1*): Quichean Maya[14]
Used for (1#): Female genital[14]
Cognates:
Language contact:

***Oncidium* (Orchidaceae)**

Spanish names: Cola de raton; Orquidea
Indigenous names: Tsuk i tyutsu[03]; Oxib Xjuruch[14]
Used by (3*): Zoque[02, 03]; Quichean Maya[14]
Used for (6#): Ear[03]; Respiratory[02]; Skin[03, 14]; Female genital[03, 14]
Cognates:
Language contact:

***Operculina* (Convolvulaceae)**

Spanish names:
Indigenous names: Pok' laak, akan tsok'[07]; Rixihij li mis[14]
Used by (2*): Huastec[07]; Quichean Maya[14]
Used for (5#): Digestive[07]; Musculoskeletal[14]; Neurological[07]; Skin[07]; General and Unspecified[07]
Cognates:
Language contact:

***Oplismenus* (Poaceae)**

Spanish names:
Indigenous names: Ts'aahil tsan, chukul bat'aw, lakab huchuul, ts'aahil kw'a', tsakam tsahib[07]
Used by (1*): Huastec[07]
Used for (1#): General and Unspecified[07]
Cognates:
Language contact:

Opuntia **(Cactaceae)**

Spanish names: Nopal
Indigenous names: Pak'ak, k'aan k'iith, thiman pak'ak, bohol pak'ak[07]; Nach'te, nich'te, nachti[13]; Yop'nopal[18]; Yàg-biăa[23]
Used by (5*): Huastec[07]; Quichean Maya[13]; Western Maya[18]; Zapotec[23]; Nahua[25]
Used for (15#): Digestive[07, 18, 23]; Musculoskeletal[07]; Respiratory[07, 13, 18, 25]; Skin[07, 13, 23]; Endocrine[18, 25]; General and Unspecified[07, 13]
Cognates:
Language contact:

Oreopanax **(Araliaceae)**

Spanish names: Palo de agua
Indigenous names:
Used by (1*): Nahua[26]
Used for (1#): Respiratory[26]
Cognates:
Language contact:

Origanum **(Lamiaceae)**

Spanish names: Oregano
Indigenous names:
Used by (1*): Zapotec[21]
Used for (1#): General and Unspecified[21]
Cognates:
Language contact:

Origanum **(Lamiaceae)**

Spanish names: Mejorana; Oregano; Oregano de comida; Oregano, mejorana
Indigenous names: Mehorana[07]; Tsiimal koy[08]
Used by (10*): Zoque[01, 02]; Totonac[06]; Huastec[07, 08]; Yucatecan Maya[09]; Quichean Maya[12]; Zapotec[22]; Nahua[25, 26]
Used for (29#): Digestive[01, 02, 06, 08, 12, 22, 26]; Musculoskeletal[08, 12]; Neurological[02, 08, 12]; Psychological[06, 08, 12, 26]; Respiratory[06, 08, 12]; Urological[12]; Pregnancy[09, 26]; Female genital[02, 06, 12, 26]; General and Unspecified[01, 07, 25]
Cognates:
Language contact:

Ormosia **(Fabaceae)**

Spanish names: Coral
Indigenous names: May cuy[03]
Used by (1*): Zoque[03]
Used for (2#): Pregnancy[03]; Female genital[03]
Cognates:
Language contact:

Ornithocephalus **(Orchidaceae)**

Spanish names:
Indigenous names: X-xi' Mukuy[14]
Used by (1*): Quichean Maya[14]
Used for (2#): Skin[14]; General and Unspecified[14]
Cognates:
Language contact:

***Orthrosanthus* (Iridaceae)**

Spanish names: Palma Morada; Petate de ratón
Indigenous names: Aq'om Kotzij[12]; Pop chó[13]; Xmes hi' ha'[14]
Used by (3*): Quichean Maya[12, 13, 14]
Used for (3#): Digestive[12]; Respiratory[14]; Female genital[13]
Cognates:
Language contact:

***Oryctanthus* (Loranthaceae)**

Spanish names:
Indigenous names: Ne'ba pim[16]
Used by (1*): Quichean Maya[16]
Used for (1#): Psychological[16]
Cognates:
Language contact:

***Oryza* (Poaceae)**

Spanish names: Arroz
Indigenous names:
Used by (5*): Zoque[01, 02]; Mixe[04]; Quichean Maya[12]; Zapotec[21]
Used for (7#): Digestive[01, 02, 04, 21]; Musculoskeletal[01]; General and Unspecified[12, 21]
Cognates:
Language contact:

***Osmunda* (Osmundaceae)**

Spanish names: Helecho real
Indigenous names:
Used by (1*): Quichean Maya[13]
Used for (1#): Musculoskeletal[13]
Cognates:
Language contact:

***Ostrya* (Betulaceae)**

Spanish names: Encino niño/roble enano
Indigenous names: Une kamay/ tzut kamay[01]
Used by (1*): Zoque[01]
Used for (2#): Digestive[01]; Respiratory[01]
Cognates:
Language contact:

***Oxalis* (Oxalidaceae)**

Spanish names: Calzoncillo de Agua, Trebol silvestre; Capulin blanco; Hierba de Chicha, Trebolillo amarillo; Hierba de sentimiento, hierba de pesar; Hoja de azar; Tamarindillo, monte de preñiz; Trebol; Trebol, acederill, aleluya, vinagrio
Indigenous names: Tza'a tzoy[01]; Pixtic sotyi[03]; Paxum[04]; Hilil ts'ohool, otsom ichiich, t'i'kom ichiich[07]; Kital ichiich[07]; Elel[09]; Q'ais ch'am, Q'en Lotz Aq'om[12]; Lost, Oxi' Ruxaq Q'os[12]; Kieq lotz[13]; Lotz[13]; Ajsyan k'opot, pajpaj k'opot[19]; Guìzh-bdiò-guìx, xín-guièe-bè, guìzh-bè-làs[23]
Used by (10*): Zoque[01, 03]; Mixe[04]; Huastec[07]; Yucatecan Maya[09, 11]; Quichean Maya[12, 13]; Western Maya[19]; Zapotec[23]
Used for (42#): Blood[12]; Digestive[04, 07, 12, 13, 19, 23]; Eye[12]; Cardiovascular[12]; Musculoskeletal[12]; Neurological[01, 12]; Psychological[03, 12]; Respiratory[13]; Skin[01, 03, 07, 09, 11, 12, 13]; Urological[03]; Pregnancy[03]; Female genital[12]; General and Unspecified[01, 03, 07, 09, 11, 12]
Cognates: Quich: lotz;
Language contact: Hua <> Yuc

***Oxyrhynchus* (Fabaceae)**

Spanish names:
Indigenous names: Wal pooy[07]
Used by (1*): Huastec[07]
Used for (1#): Eye[07]
Cognates:
Language contact:

***Pachira* (Malvaceae)**

Spanish names: Apompo; Bonete; Palo de agua; Santa Domingo; Zapote de agua; Zapote de laguna
Indigenous names: Uwakta[01]; Uakta[03]; Ka'pa'[05]; K'unche', K'uyche'[09]; Sapote bobo[10]; Ajp'o tec[18]
Used by (6*): Zoque[01, 03]; Totonac[05]; Yucatecan Maya[09, 10]; Western Maya[18]
Used for (9#): Digestive[18]; Eye[18]; Skin[05]; Endocrine[01, 03, 18]; Urological[03, 10]; General and Unspecified[09]
Cognates: Zoq: u(w)akta;
Language contact:

***Pachyrhizus* (Fabaceae)**

Spanish names: Jicama
Indigenous names: Kobeem[07]; Kup[09]
Used by (2*): Huastec[07]; Yucatecan Maya[09]
Used for (2#): Respiratory[09]; Skin[07]
Cognates: Maya: kob/kup;
Language contact:

***Panicum* (Poaceae)**

Spanish names:
Indigenous names: Itse' toom[07]
Used by (1*): Huastec[07]
Used for (1#): Respiratory[07]
Cognates:
Language contact:

***Parathesis* (Primulaceae)**

Spanish names: Llorasangre, Corazón de la Selva
Indigenous names: Chu cuxamñi[03]; Apulee', ebha'[07]; Ruk'uch Juyu[12]; Tilil ja'[20]
Used by (4*): Zoque[03]; Huastec[07]; Quichean Maya[12]; Western Maya[20]
Used for (16#): Blood[12]; Digestive[03, 07, 12, 20]; Cardiovascular[12]; Musculoskeletal[12]; Psychological[12]; Respiratory[12]; Endocrine[12]; Urological[12]; Female genital[03]; General and Unspecified[07, 12]
Cognates:
Language contact:

***Parmentiera* (Bignoniaceae)**

Spanish names: Chote, cuajilote; Cuajilote; Guajilote; Pepino cat; Platano espina, uña de tigre
Indigenous names: Apit kuy[01]; Paka'ak[04]; Pusni, puxni[05]; Pux'nit[06]; Tsoote'[07]; Kat[09]; Katche'[09]; Cho'te[18]; Biguaj guedxii[21]; Cuaxilotl[28]
Used by (11*): Zoque[01, 02, 03]; Mixe[04]; Totonac[05, 06]; Huastec[07]; Yucatecan Maya[09]; Western Maya[18]; Zapotec[21]; Nahua[28]
Used for (39#): Digestive[01, 05, 07]; Ear[05, 06, 07, 18]; Neurological[05]; Respiratory[01, 02, 03, 04, 05, 07, 18, 21]; Skin[07]; Endocrine[05, 06, 09, 18, 28]; Urological[05, 06, 07, 09, 18, 28]; Pregnancy[03, 05]; Female genital[03]; Male genital[01]; General and Unspecified[02, 05, 07, 18, 28]
Cognates: Tot: puxni; Maya: tsoote/choote;
Language contact:

***Parthenium* (Asteraceae)**

Spanish names: Altamisa; Altamisa, cilantrillo; Hierba maestra/velo de reina/ estrella; Marijuana cimarron; Sicutía
Indigenous names: Tzaj'u pu, ma'tza jäyä[01]; Pa artemis, artemis ujts[04]; Ts'a'il kw'eet, kaxiy kw'eet[07]; Cilantro Ši'u [10]; Corriente Ši'u [10]
Used by (9*): Zoque[01, 03]; Mixe[04]; Huastec[07]; Yucatecan Maya[09, 10, 11]; Western Maya[18]; Zapotec[21]
Used for (25#): Blood[10]; Digestive[07, 18]; Cardiovascular[01]; Musculoskeletal[01, 03, 04, 07, 18, 21]; Respiratory[01]; Skin[01, 07, 21]; Endocrine[01, 03]; Pregnancy[07]; Female genital[09]; General and Unspecified[01, 04, 07, 09, 10, 11, 21]
Cognates:
Language contact:

***Paspalum* (Poaceae)**

Spanish names: Zacate grama
Indigenous names: So'ok[01]
Used by (2*): Zoque[01, 02]
Used for (3#): Psychological[01]; Urological[01]; General and Unspecified[02]
Cognates:
Language contact:

***Passiflora* (Passifloraceae)**

Spanish names: Ala de Muerciélago ; Ala de murciélago; Cachito rastrero, calzoncillo; Gagapachi; Granadilla; Granadilla de Culebra, Passiflora, Riñon de Montaña; Granadilla, Passiflora; Guaco; Jujito, top'o lac'; Maracuyá; Monte flución, monte preñiz; Passiflora; Pepe; Pepe, passiflora, granadita
Indigenous names: Tzi pono [01]; Xiu tiepo[03]; Tanto'xy[04]; Sindi sprun, sandia spuun[06]; Okoob thut'[07]; Pok'pok', owel paat, tsakam hiliy[07]; Xik'sots'[09]; Xpoch[09]; Šik'soo¢[10]; Cruz Q'an, Aq'om Q'ayis Riñon[12]; Aq'om Kowil Q'os Ka'[12]; Rismal ru' qanyaj, Xik'isotz[14]; Tú' kej[14]; Xik'isotz[14]; Tu' kej'[14]; Granadillo y Cho' Ho'[14]; Roq' Maus aj' Winq[14]; Choq'l pim[14]; Tu'kej[16]; Tu' kej[17]; Juju'[18]; Pa'ch'em, julusyon k'opot[19]; Wapapa[21]; Ibæ̀-grànâd, guìzh-grànâd, grànâd-dán[23]
Used by (18*): Zoque[01, 02, 03]; Mixe[04]; Totonac[05, 06]; Huastec[07]; Yucatecan Maya[09, 10]; Quichean Maya[12, 14, 16, 17]; Western Maya[18, 19]; Zapotec[21, 23]; Nahua[28]
Used for (90#): Blood[05, 12]; Digestive[02, 07, 12, 14, 21, 28]; Eye[03]; Ear[07, 09]; Cardiovascular[02, 18, 28]; Musculoskeletal[12, 19]; Neurological[01, 07, 12, 14, 16, 19, 23, 28]; Psychological[01, 02, 03, 12, 18, 21]; Respiratory[02, 12, 14]; Skin[02, 03, 07, 09, 12, 14, 21]; Endocrine[02, 12]; Urological[02, 03, 04, 05, 06, 12, 14]; Pregnancy[07]; Female genital[03, 07, 18, 21]; Male genital[12, 14]; Social problems[14]; General and Unspecified[07, 10, 12, 14, 18, 19, 21]; nd[17]
Cognates: CoreM: xik'sots; Yuc: xik'sots; Quich: tu'kej;
Language contact: Zap > Chimalapa Zoq via Spanish

***Paullinia* (Sapindaceae)**

Spanish names:
Indigenous names: K¥kujuki ay[03]; T'in kamab, tu kamaab[07]
Used by (2*): Zoque[03]; Huastec[07]
Used for (12#): Digestive[03, 07]; Eye[03]; Skin[03, 07]; Endocrine[07]; Urological[03]; Pregnancy[03, 07]; Female genital[03, 07]
Cognates:
Language contact:

***Pavonia* (Malvaceae)**

Spanish names: Cadillo; Lengua de perro; Lengua de vaca
Indigenous names: Kam dane[01]; Konko[03]; Uk tuukats[04]; Ts'ikiy uxum, liin ts'ohool[07]; Mul Tzi'[14]
Used by (6*): Zoque[01, 02, 03]; Mixe[04]; Huastec[07]; Quichean Maya[14]
Used for (19#): Digestive[01, 03, 07]; Cardiovascular[01]; Musculoskeletal[07]; Neurological[01]; Respiratory[07]; Skin[02, 03, 07, 14]; Urological[07]; Pregnancy[03, 07, 14]; Female genital[07]; General and Unspecified[04, 14]
Cognates:
Language contact:

01-28 refer to the studies described in Table 4.1 * Total number of studies recording this taxon #Total number of use-records

***Pecluma* (Polypodiaceae)**

Spanish names:
Indigenous names: Ehtiil weew koxol[07]
Used by (2*): Zoque[03]; Huastec[07]
Used for (3[#]): Digestive[03]; Neurological[07]; General and Unspecified[03]
Cognates:
Language contact:

***Pelargonium* (Geraniaceae)**

Spanish names: Geranio; Geranio (doméstico); Geranio (rojo); Geranio, solferino; Malva
Indigenous names: Guiѐe-jèrân[23]; Tlalalatl[28]
Used by (5*): Zoque[01]; Quichean Maya[12, 13]; Zapotec[23]; Nahua[28]
Used for (10[#]): Blood[12]; Digestive[13]; Musculoskeletal[01]; Neurological[01, 12]; Respiratory[12]; Skin[12, 23]; Female genital[28]; General and Unspecified[12]
Cognates:
Language contact:

***Pellaea* (Pteridaceae)**

Spanish names:
Indigenous names: Ncuàan-dzéb[23]
Used by (1*): Zapotec[23]
Used for (1[#]): General and Unspecified[23]
Cognates:
Language contact:

***Pennisetum* (Poaceae)**

Spanish names: Carizo
Indigenous names: Kape[01]; Pakaab[07]
Used by (2*): Zoque[01]; Huastec[07]
Used for (4[#]): Skin[01, 07]; Urological[01]; General and Unspecified[07]
Cognates:
Language contact: Chiapas Zoq <> Hua

***Penstemon* (Plantaginaceae)**

Spanish names:
Indigenous names: Guièe-dzǐn̲g̲, guìzh-guièe-dzǐn̲g̲, guìzh-dzǐn̲g̲, guièe-mĕets, guièe-mìtsiĕ[23]
Used by (1*): Zapotec[23]
Used for (2[#]): Skin[23]; General and Unspecified[23]
Cognates:
Language contact:

***Pentalinon* (Apocynaceae)**

Spanish names: Viperol verde
Indigenous names: Look' ts'aah[07]; Q'aru pim[14]
Used by (3*): Huastec[07]; Yucatecan Maya[09]; Quichean Maya[14]
Used for (3[#]): Skin[07, 09]; Endocrine[14]
Cognates:
Language contact:

***Peperomia* (Piperaceae)**

Spanish names: Cristalillo; Hierba cristal; Hoja de Pescado; Ojo de Gato; Oreja de burro; Oreja de Cabro; Pata paloma; Pega huesos; Tarbatillo; Xoyoquelite
Indigenous names: Maj'a rane[01]; Kiñi ay[03]; Conchuru ay, Epxi ay[03]; Chixchix ay[03]; Chicu tats¥k[03]; Patsuxk juaxy[04]; Poj ujts[04]; Tsakam ix tuyuum[07]; Boton ts'ohool, wiyab ts'ohool, homte' ts'ohool[07]; Riqan plamux[13]; Wiq'baq'[13]; Rubel xsa' ixul[14]; Chak B'olay Q'ehen, Mai pim, Leetzeb pim[14]; Ik B'olay Q'ehen[14]; Xwa Ixul, Xikyuk[14]; Se'ru Mes[14]; Se'ru Kar [14]; Chak B'olay Q'ehen, Ik B'olay pim, Xwa Ixul[14]; Xcua'aj aw chan[16]; X cua'i xul[17]; Tzetzetzi[26]
Used by (13*): Zoque[01, 02, 03]; Mixe[04]; Totonac[05]; Huastec[07]; Quichean Maya[13, 14, 16, 17]; Western Maya[18]; Nahua[25, 26]
Used for (55#): Digestive[14]; Ear[03]; Musculoskeletal[03, 13, 14]; Neurological[01, 07, 14, 16]; Psychological[16]; Respiratory[25]; Skin[01, 02, 03, 04, 07, 13, 14, 18, 26]; Pregnancy[05]; Female genital[14]; General and Unspecified[03, 04, 07, 14]; nd[17]
Cognates: Quich: x(c)ua ixul;
Language contact:

***Pereskia* (Cactaceae)**

Spanish names:
Indigenous names: Pulik kwi'inal[07]
Used by (1*): Huastec[07]
Used for (2#): Digestive[07]; Musculoskeletal[07]
Cognates:
Language contact:

***Pereskiopsis* (Cactaceae)**

Spanish names:
Indigenous names: Kweteem kwi'inal[07]
Used by (1*): Huastec[07]
Used for (3#): Digestive[07]; Respiratory[07]; General and Unspecified[07]
Cognates:
Language contact:

***Persea* (Lauraceae)**

Spanish names: Aguacate; Aguacate oloroso; Pagua
Indigenous names: Owi / Kuytäm / Kuytäp [01]; Kuy täm[02]; Cuy ty¥m[03]; Kuit[04]; Kuka'taj[05]; Cutacaj[06]; Xl'pu[06]; Uh, oh[07]; Uj[08]; On[09]; On[10]; Oj[12]; Oj[13]; Um[18]; Un[19]; On[20]; Yeexu'u[21]; Yàg-ngùd-guièx[23]; Aguacate[25]
Used by (21*): Zoque[01, 02, 03]; Mixe[04]; Totonac[05, 06]; Huastec[07, 08]; Yucatecan Maya[09, 10]; Quichean Maya[12, 13, 14]; Western Maya[18, 19, 20]; Zapotec[21, 23]; Nahua[24, 25, 26]
Used for (91#): Blood[05, 12]; Digestive[01, 02, 03, 04, 06, 07, 08, 13, 18, 19, 20, 21, 25]; Eye[21]; Cardiovascular[01, 02, 06, 08, 18, 21]; Musculoskeletal[01, 02, 03, 06, 08, 12, 13, 21]; Neurological[03, 08, 12]; Psychological[01, 06, 12, 18]; Respiratory[02, 05, 07, 08, 09, 10, 13, 19, 25, 26]; Skin[01, 02, 03, 06, 07, 08, 13, 18, 21, 25]; Endocrine[01, 03, 09, 12, 18]; Urological[01, 09, 12]; Pregnancy[02, 04, 06, 12, 13, 14, 19, 21, 23]; Female genital[01, 02, 03, 19, 24]; Male genital[01, 03, 18]; General and Unspecified[01, 05, 07, 08, 12, 14, 21, 25]
Cognates: MZ: kuit; Zoq: kuitäm; Tot: kukataj; Maya: oj/on; Hua: oh/uh/uj; Yuc: on; Quich: oj; WesM: um/un/on; Nah: aguacatl;
Language contact: MZ > Tot; Mayan > Chiapas Zoq

***Persicaria* (Polygonaceae)**

Spanish names: Hoja de azar; Nueve Embarazo ; Pimienta de Agua
Indigenous names: Nø mo 'unts ujts[04]; Rukotz'ij chab'äk[12]; Beleeb' Q'ehen[14]
Used by (4*): Zoque[01]; Mixe[04]; Quichean Maya[12, 14]
Used for (8#): Digestive[12]; Musculoskeletal[14]; Skin[04]; Urological[12]; Pregnancy[14]; General and Unspecified[01, 12, 14]
Cognates:
Language contact:

Peteravenia (Asteraceae)

Spanish names: Hierba de fiebre
Indigenous names: Nekx cuy[03]
Used by (2*): Zoque[01, 03]
Used for (5#): Digestive[01]; Musculoskeletal[01]; Skin[03]; Female genital[01]; General and Unspecified[01]
Cognates:
Language contact:

Petiveria (Phytolaccaceae)

Spanish names: Apacín; Apacina; Hierba del zorrillo; Hierba del zorro/hoja de zorro/ hierba de zorrillo; Hoja de zorillo; Hoja de zorrillo/ hoja del zorro; Zorrillo, hoja de mal viento
Indigenous names: Wujpa ay/pats rane/ pats ay[01]; Patz ay/paks ay/ pats a'watz/jaka kätzok[02]; Patscang ay[03]; Paj ujts[04]; Pathaam, path ts'ohool, pathaam uut'[07]; Pasim[11]; Paara Q'ehen[14]; Par'i'pim[16]; Tujen a'uch[18]; Bete'a[21]
Used by (13*): Zoque[01, 02, 03]; Mixe[04]; Totonac[05]; Huastec[07]; Yucatecan Maya[11]; Quichean Maya[12, 14, 16]; Western Maya[18, 19]; Zapotec[21]
Used for (44#): Digestive[01, 02, 03, 11, 14, 19, 21]; Musculoskeletal[01, 02, 03, 07, 14, 18, 21]; Neurological[02, 07, 11, 14, 16]; Psychological[16]; Respiratory[01, 02, 04, 12, 21]; Skin[03, 05, 07, 14, 21]; Endocrine[07]; Urological[01, 14]; Pregnancy[19]; Female genital[19]; General and Unspecified[01, 02, 03, 05, 07, 12, 14, 18, 21]
Cognates: MZ: paj/pats; Zoq: pats; Maya: path/pas/par; Quich: par;
Language contact: MZ > Maya

Petrea (Verbenaceae)

Spanish names: Comida de caballo; Flor de Nazareno; Raspasombrero
Indigenous names: Thathup tsâah, paaskwa wits, wayelom ts'ohool, kothow ch'aah[07]; Lat'ax xumplelab[08]; Yochop'tsimin[09]
Used by (4*): Huastec[07, 08]; Yucatecan Maya[09]; Quichean Maya[12]
Used for (13#): Digestive[08, 09]; Musculoskeletal[08]; Neurological[07]; Psychological[07]; Respiratory[07]; Skin[07, 12]; Urological[08]; Pregnancy[07]; Female genital[07]; General and Unspecified[07, 12]
Cognates:
Language contact:

Petroselinum (Apiaceae)

Spanish names: Perejil
Indigenous names: Parsil[13]
Used by (3*): Totonac[06]; Quichean Maya[13]; Zapotec[23]
Used for (9#): Digestive[06]; Cardiovascular[06]; Neurological[23]; Respiratory[23]; Pregnancy[13]; Female genital[06]; Male genital[13]; General and Unspecified[06, 23]
Cognates:
Language contact:

Peumus (Monimiaceae)

Spanish names: Boldo
Indigenous names:
Used by (2*): Quichean Maya[12]; Zapotec[21]
Used for (7#): Blood[12]; Digestive[12, 21]; Psychological[12]; Skin[12]; Urological[12]; General and Unspecified[12]
Cognates:
Language contact:

Phacelia (Boraginaceae)

Spanish names: Milenrama de Agua
Indigenous names: Aq'om Kumatzin Q'os ya'[12]
Used by (1*): Quichean Maya[12]
Used for (4#): Digestive[12]; Cardiovascular[12]; Musculoskeletal[12]; Psychological[12]
Cognates:
Language contact:

***Phalaris* (Poaceae)**

Spanish names: Alpiste
Indigenous names:
Used by (2*): Zoque[02]; Quichean Maya[12]
Used for (4#): Digestive[12]; Cardiovascular[02]; Skin[12]; General and Unspecified[12]
Cognates:
Language contact:

***Phaseolus* (Fabaceae)**

Spanish names: Frijol; Frijol blanco; Frijol negro
Indigenous names: Popo säk[01]; S¥k[03]; Stapu[06]; Tsanakw', chanakw'[07]; Ik'ik bu'u[18]; Bizza'a[21]; Blă-dĭp, dzè-díp, bziàa-díp[23]
Used by (7*): Zoque[01, 03]; Totonac[06]; Huastec[07]; Western Maya[18]; Zapotec[21, 23]
Used for (9#): Blood[18, 21]; Digestive[07]; Skin[06, 21, 23]; Pregnancy[01, 03]; General and Unspecified[21]
Cognates: Zoq: säk; Zap: bizza'a/bziaa;
Language contact: Zoq > Hua

***Phenax* (Urticaceae)**

Spanish names: Gordoncillo
Indigenous names:
Used by (1*): Quichean Maya[13]
Used for (1#): Digestive[13]
Cognates:
Language contact:

***Philodendron* (Araceae)**

Spanish names: Bejuco Negro; Chapiz; Chapiz grande, malaste grande
Indigenous names: Toy'patek ay[02]; Pasmuj ay[03]; Marina ay[03]; Mututs[03]; Tantai[05]; Saq'i Jolol[14]; Ma'raq'[16]; Par'i'pim[16]; Rubelsa'i'xul[16]
Used by (6*): Zoque[01, 02, 03]; Totonac[05]; Huastec[07]; Yucatecan Maya[09]; Quichean Maya[12, 13, 14, 16]; Western Maya[18, 19]; Zapotec[23]; Nahua[25]
Used for (17#): Eye[02, 03, 05]; Cardiovascular[14]; Musculoskeletal[03, 14]; Neurological[14, 16]; Psychological[16]; Skin[03, 05, 09, 14]
Cognates:
Language contact:

***Phlebodium* (Polypodiaceae)**

Spanish names: Calagual, canaguala; Calaguala; Calaguala/Hierba de golpe; Calahuala; Costilla de león; Lengua de ciervo
Indigenous names: Misyu mätzyik[01]; Ts'een k'ubak koy, talab ik,bo' waak[07]; K'ubak koy, akan koy, t'ot ts'ohool, k'ubak mitsu', akan wahuts, k'ubak pathaam[07]; Ruxe' Kalaguala Cha'at[12]; Ch'upil Q'en[14]; Lorom, u kab' mis[19]; Bàz, guìzh-bàz[23]; Tehualcachitihuitl[25]
Used by (10*): Totonac[05]
Used for (30#): Digestive[01, 12, 13, 23]; Cardiovascular[25]; Musculoskeletal[01, 05, 13, 18, 25]; Neurological[14]; Respiratory[01, 07, 23]; Skin[07, 18]; Endocrine[01, 13]; Pregnancy[07, 19]; Female genital[01, 19]; Male genital[01]; General and Unspecified[07, 12, 13, 23]
Cognates:
Language contact: Chiapas Zoq <> Hua <> Chortí

***Phoradendron* (Santalaceae)**

Spanish names: Cabellera; Hierba amarilla; Mata palo; Muérdago; Muerdago, mata palo; Mundago/presta palo
Indigenous names: Kuy yakspa[01]; Cuyñukxi[03]; Palek aay[04]; Ok'lom te' yexu'[07]; Tzara'[12]; Tzara' geka[12]; Wik bak[13]; Shaguii nagitzi'i[21]
Used by (8*): Zoque[01, 03]; Mixe[04]; Huastec[07]; Quichean Maya[12, 13]; Zapotec[21]; Nahua[26]
Used for (16#): Digestive[12]; Cardiovascular[01, 12]; Musculoskeletal[13, 21]; Neurological[07]; Psychological[12]; Skin[03, 04, 12, 21]; Endocrine[26]; General and Unspecified[03, 12, 21]
Cognates:
Language contact:

***Phragmites* (Poaceae)**

Spanish names: Caña de castilla
Indigenous names:
Used by (1*): Quichean Maya[13]
Used for (1#): Urological[13]
Cognates:
Language contact:

***Phyla* (Verbenaceae)**

Spanish names: Hierba dulce; Hierba dulce, orozol, orozul, orozús, salvia santa; Hierba dulce, orozuz; Hierba dulce, té de abuela; Orozul/hierba dulce; Orozus; Orozus/santa lucia/verbena; Orozuz; Sorosir; Té de china; Yerba dulce
Indigenous names: Kanak pa'ak [01]; Cana ay[03]; Pa'ak ujts[04]; Sak'si'tuwan[05]; Tsi'iimal koy, chi'ik ch'ohool[07]; Orozús[13]; Q'iil pim [14]; Bänälä tzaj[18]; Guixa'a na'axii[21]
Used by (17*): Zoque[01, 02, 03]; Mixe[04]; Totonac[05]; Huastec[07]; Yucatecan Maya[09]; Quichean Maya[12, 13, 14, 17]; Western Maya[18, 19]; Zapotec[21, 22]; Nahua[25, 26]
Used for (41#): Digestive[01, 02, 03, 05, 07, 09, 21, 22, 25, 26]; Neurological[03, 13]; Respiratory[01, 02, 03, 04, 05, 07, 12, 13, 18, 19, 21]; Skin[07, 14]; Urological[03, 12]; Pregnancy[03, 26]; Female genital[03, 05, 26]; Male genital[12]; General and Unspecified[07, 09, 12, 13, 14, 21]; nd[17]
Cognates: MZ: pa'ak; Zoq: kana;
Language contact:

***Phyllanthus* (Phyllanthaceae)**

Spanish names: Hierba de piedrita/ quebra piedras/ rompepiedra; Hierba del gusano; Mal de ojo; Zapote negro
Indigenous names: Poka rane [01]; Antuñik¥ ay[03]; Kux ichiich, kital ichiich, waleklaab ichiich[07]; Pok' thoot[07]; Xulimil[09]; P'ix'ton-ak'[09]; Pets'k'mi, Kambaikiche'[09]; P'ix'tonche'[09]; Tleocuilpactle[26]; Tliltzapotl[26]; Axocopa[26]
Used by (5*): Zoque[01, 03]; Huastec[07]; Yucatecan Maya[09]; Nahua[26]
Used for (22#): Digestive[03, 07]; Eye[07]; Neurological[07, 09]; Psychological[03]; Skin[01, 07, 09, 26]; Urological[01, 09]; Pregnancy[26]; Female genital[09]; General and Unspecified[03, 07]
Cognates:
Language contact: Chiapas Zoq <> Hua <> Yuc

***Phymosia* (Malvaceae)**

Spanish names:
Indigenous names: Tsak kwiniimte'[07]
Used by (1*): Huastec[07]
Used for (1#): Digestive[07]
Cognates:
Language contact:

***Physalis* (Solanaceae)**

Spanish names: Chap'ulul; Costomate; Miltomate; Tomate de lombriz; Tomate verde; Tomatillo
Indigenous names: Koya tzäpe/ täksy kuy ay/ toksy koyak [01]; Naka txipiñ[03]; Chap'ulul[06]; Tuthaayil an t'ot[07]; Akal k'ak'al ilaal, palat ichiich, tuthay te', walul ch'ohool[07]; Tudheyil t'ot[08]; Top tuČ[10]; Joq pix[13]; Pe'ych k'opot[19]; Bityuš gihš[22]; Yàg-pchŭux-làs, pchŭux-làs[23]
Used by (12*): Zoque[01, 03]; Totonac[06]; Huastec[07, 08]; Yucatecan Maya[10]; Quichean Maya[12, 13]; Western Maya[19]; Zapotec[22, 23]; Nahua[27]
Used for (32#): Digestive[01, 06, 07, 08, 13, 19, 22]; Ear[07]; Cardiovascular[06]; Neurological[01]; Respiratory[01, 03, 06, 08, 13, 27]; Skin[03, 06, 07, 10, 12, 13, 23]; Urological[06]; General and Unspecified[07, 12, 22]
Cognates: Hua: thuthVyil t'ot; Zap: pchux;
Language contact: Tot <> Hua; Yuc <> Chortí

***Phytolacca* (Phytolaccaceae)**

Spanish names: Hierba blanca, pie de palom tierno; Jaboncillo
Indigenous names: Tzakan[01]; Tzakanak[02]; Masan ay[03]; T'eikox[09]; Saltz'i' o Pek tz'i, Sa'l Tz'i Q'os[12]; Retz'e, raq'tze[13]
Used by (6*): Zoque[01, 02, 03]; Yucatecan Maya[09]; Quichean Maya[12, 13]
Used for (11#): Blood[13]; Digestive[12]; Skin[01, 02, 03, 12, 13]; Urological[12]; General and Unspecified[02, 09, 12]
Cognates: Zoq: tzakan; CoreM: t'eikox/tz'i q'os;
Language contact:

***Picramnia* (Picramniaceae)**

Spanish names:
Indigenous names: J¥mniom petx maka[03]; Petx maka[03]; Thal te', k'inim te', thak oliy, tsakam it'il[07]
Used by (2*): Zoque[03]; Huastec[07]
Used for (4#): Skin[03, 07]
Cognates:
Language contact:

***Pilea* (Urticaceae)**

Spanish names: Espumilla; Hoja de azar; Hoja de azar de hierbabuena
Indigenous names: Tza'a tzoy[01]; Txiñ txay sotyi[03]; Kiñi ay[03]; Ha'il tsan, tumiin ts'ohool, ohil tsan, pitsits wal[07]; Pux lat'em, kux laatem[07]; Tsakam tsahib[07]; Ox eek'il Q'ehen[14]
Used by (5*): Zoque[01, 03]; Huastec[07]; Yucatecan Maya[11]; Quichean Maya[14]
Used for (19#): Eye[07]; Ear[03]; Musculoskeletal[03]; Neurological[07]; Respiratory[07, 11]; Skin[03, 07]; Urological[11]; Pregnancy[07]; General and Unspecified[01, 03, 07, 14]
Cognates: Zoq: tsa/txa;
Language contact: Zoq > Hua

***Pilocarpus* (Rutaceae)**

Spanish names:
Indigenous names: Tamkasche', Siische'[09]
Used by (1*): Yucatecan Maya[09]
Used for (3#): Digestive[09]; Respiratory[09]; General and Unspecified[09]
Cognates:
Language contact:

***Pimenta* (Myrtaceae)**

Spanish names: Patololote, Pimienta gorda; Pimenton; Pimienta; Pimienta castilla; Pimienta de tabasco; Pimienta gorda; Pimienta grande
Indigenous names: Moki[01]; Moke[02]; Uk suk[03]; U'cum[06]; Nohochpol[09]; Naba kook[10]; Peen's[14]; Xuxpat[18]; Pimient rooj[21]
Used by (15*): Zoque[01, 02, 03]; Mixe[04]; Totonac[05, 06]; Yucatecan Maya[09, 10, 11]; Quichean Maya[12, 13, 14]; Western Maya[18, 19]; Zapotec[21]
Used for (55#): Digestive[01, 02, 03, 05, 06, 09, 10, 11, 12, 13, 18, 21]; Ear[01]; Cardiovascular[02]; Musculoskeletal[02, 06, 12, 18, 21]; Neurological[01, 02, 18]; Psychological[02]; Respiratory[01, 02, 03, 13, 18]; Skin[01, 03, 18, 21]; Urological[18]; Pregnancy[01, 02, 06, 09, 13, 14, 19, 21]; Female genital[01, 02, 03, 05, 09, 11, 19, 21]; General and Unspecified[01, 02, 04, 12, 13, 21]
Cognates: Zoq: moki/e;
Language contact: Zoq > Tot

***Pimpinella* (Apiaceae)**

Spanish names: Anís
Indigenous names: Anix[13]
Used by (6*): Zoque[02]; Totonac[06]; Yucatecan Maya[09]; Quichean Maya[12, 13]; Western Maya[19]
Used for (24#): Blood[19]; Digestive[02, 06, 09, 13, 19]; Musculoskeletal[02, 12]; Neurological[12]; Psychological[02, 12, 13]; Endocrine[02]; Urological[12]; Pregnancy[06, 09, 12, 13, 19]; Female genital[02, 12, 13]; General and Unspecified[12, 13]
Cognates:
Language contact:

01-28 refer to the studies described in Table 4.1 * Total number of studies recording this taxon #Total number of use-records

***Pinaropappus* (Asteraceae)**

Spanish names: Espule, espulga
Indigenous names: Guièe-mòrâd, guìzh-mòrâd, guìzh-nĭdz, ngùd-nĭdz, guìzh-rziòob], guìzh-guiĕt-nì[23]
Used by (2*): Zapotec[22, 23]
Used for (3[#]): Eye[23]; Skin[22, 23]
Cognates:
Language contact:

***Pinguicula* (Lentibulariaceae)**

Spanish names: Trebol de Montaña
Indigenous names: Keq Treblo Aq'om[12]; Diàg-cûch, guièe-ngùrùdz, guièe-yòob-chèn[23]
Used by (2*): Quichean Maya[12]; Zapotec[23]
Used for (8[#]): Blood[12]; Digestive[12, 23]; Neurological[12]; Psychological[12]; Respiratory[12]; Skin[12]; General and Unspecified[12]
Cognates:
Language contact:

***Pinus* (Pinaceae)**

Spanish names: Ocote; Ocote (colorado); Pino
Indigenous names: Tzin/tzit/ kon tzit/ tsapas koko'äng tzit[01]; Tzin[02]; Tyiñcuy[03]; Tsiin[04]; Pithomlaab[07]; Chäj[12]; Chaj'[13]; Tajte'[19]; Taj, k'an toj[20]; Guiere'ej[21]; Yàg-guièr[23]
Used by (11*): Zoque[01, 02, 03]; Mixe[04]; Huastec[07, 08]; Quichean Maya[12, 13, 14]; Western Maya[19, 20]; Zapotec[21, 23]; Nahua[26]
Used for (44[#]): Digestive[01, 02, 07, 20, 21]; Cardiovascular[02]; Musculoskeletal[01, 02, 03, 12, 13, 21]; Neurological[01, 02, 07, 12, 19]; Psychological[01, 13]; Respiratory[01, 02, 03, 04, 12, 13, 19, 21]; Skin[01, 02, 03, 04, 13, 21]; Pregnancy[01, 21]; Female genital[02]; General and Unspecified[01, 07, 12, 13, 21, 23]
Cognates: MZ: tzin; Zoq: tzin; CoreM: aj; Quich: chaj; WesM: taj;
Language contact:

***Piper* (Piperaceae)**

Spanish names: Acuyo; Acuyo cimarrón; Bella dona; Caite de Diablo; Cordoncillo; Cordoncillo de Montaña; Cordoncillo grande; Cordoncillo hembra; Cordoncillo macho, cabeza de guajilote; Cordoncillo oloroso; Cordoncillo verde; Cordonsillo; Hierba santa; Hierba santa/ (momo)/ hoja santa; Hierba santilla; Hoja de Jute; Hoja de Río; Hoja santa; Hoja/ yerba santa; Ixpiakok, Santa María ; Momo; Momo de chombo, momo de zopilote; Momo sylvestre/ haku simarron/ hoja santa simarrona; Obel; Omequelite; Omoquelite; Pata de vaca; Pie de pavito; Pimienta de castilla; Pimienta de castilla/pimienta chica; Pimienta negra; Planta Barón; San Diego; Tabaquillo; Tamagás; Tequelite; Velancitas
Indigenous names: Tunhkuy[01]; Jaku[01]; Pa' jaku/jiyen jaku/ jaku pa'an/ ji'ne haku[01]; Toso/tin ay[02]; Suj ay/syuj ay[02]; Suj[02]; Tooso[03]; Tsus tooso[03]; Aycuyo[03]; Aycuyo cimarrón[03]; Yuk yo'on[04]; Woo[04]; Yo'on[04]; Yuk woo[04]; Jinan[05]; Jina[06]; Sok'ot, xalacuahuit[06]; Paktha' yexal[07]; Tiiya', oh ts'ohool[07]; Bakaanil a iits', bok'ool uxkwe', pamta' an koy, bakanil an miimlaab, pakalaah, tiiya[07]; Thak kw'alal its'aamal[07]; Kw'alal its'aamal, yaxal, kw'alal an kwathab, kw'alal i puthuch, yaxal[07]; Tdak kw'alal ist'amal[08]; Xpeheche'[09]; Ruchek k'uch'[12]; R'u xac y'a[12]; Seq' Ruk'amal Aq'om Itzel Yabil[12]; Aq'om Q'aynaq' Cha'klaj[12]; Q'ampom, Re Tzuul[14]; Yut'it'[14]; Q'eq'i Puchuch[14]; Kuxtin Q'ehen[14]; Lima Q'ehen, Telom Q'ehen, Saq'i Puchuch [14]; Xkub' sa' Qana', Hobel[14]; Tiq'ual Q'ehen[14]; Puchuch Q'ehen, Kuw Sawi'i, Kux Q'ehen[14]; Tzuul, Puchuch Q'ehen[14]; Puchuch[14]; Yut'it' puchuch[14]; Tz'y' Q'ehen[14]; Ob'el[15]; Puchuq[15]; Ampom[15]; Nin qui ru chaq' q'een[15]; Kan pom, pu'chuch[16]; U'bel[16]; Cux sawi[16]; Pu'chuch[16]; Kan pom[16]; Mai pim[16]; Tzirito' k'ejen[17]; K'an pom che'[17]; Tyut it pim[17]; Cux sawi k'ejen[17]; Tzulub pim[17]; Momo[18]; Momo ajma'[18]; Xuxpat ic'[18]; Ok aj ts'o[18]; Ich pimyénta[19]; Guiadajna'a[21]; Hua'a[21]; Gui'iquimberu'u[21]; Guiadajna'a rooj[21]; Bala šoh[22]; Blàg-guiùu[23]; Acoyo[24]; Tequelite[25]; Tlanecpaquelite[26]; Acuyo xuitl[28]
Used by (27*): Zoque[01, 02, 03]; Mixe[04]; Totonac[05, 06]; Huastec[07]; Yucatecan Maya[09, 10, 11]; Quichean Maya[12, 14, 15, 16, 17]; Western Maya[18, 19, 20]; Zapotec[21, 22, 23]; Nahua[24, 25, 26, 27, 28]
Used for (254[#]): Blood[05, 12, 14, 15, 18]; Digestive[01, 02, 03, 05, 07, 08, 10, 11, 12, 14, 18, 19, 20, 21, 23, 25, 26, 28]; Cardiovascular[02, 12, 21]; Musculoskeletal[01, 02, 03, 07, 11, 14, 15, 18, 21, 22, 26]; Neurological[02, 03, 07, 10, 11, 14, 16, 22, 28]; Psychological[02, 03, 14, 15, 16]; Respiratory[01, 02, 03, 07, 12, 14, 18, 21, 26, 28]; Skin[01, 02, 03, 04, 05, 07, 08, 09, 10, 11, 12, 14, 18, 21, 22, 23, 25, 26]; Endocrine[02]; Urological[01, 02, 14, 18]; Pregnancy[01, 02, 03, 05, 06, 07, 14, 15, 19, 22, 24, 27]; Female genital[02, 03, 12, 14, 15, 18, 19, 21]; Male genital[21]; Social problems[14]; General and Unspecified[01, 02, 03, 04, 05, 06, 07, 08, 11, 12, 14, 15, 16, 21, 22, 26]; nd[17]
Cognates: Zoq: tVn, tooso, aku; Tot: jina; Maya: puCuch; Hua: tiiya, yaxal, tak kw'alal its'aamal; Quich: obel, q'ehen, puchuch, ampom, yut'it, kux; Nah: acoyo;
Language contact: Nah > Zoq; Zoq <> Tot & Hua

***Piptocarpha* (Asteraceae)**

Spanish names:
Indigenous names: Chunahak k'ejen[17]
Used by (1*): Quichean Maya[17]
Used for (1#): nd[17]
Cognates:
Language contact:

***Piqueria* (Asteraceae)**

Spanish names:
Indigenous names: Guìzh-làs[23]; Bæ̌æl-dòo, guìzh-běæl-dòo, bæ̌æl-dǒ, guìzh-běæl-dǒ[23]
Used by (1*): Zapotec[23]
Used for (6#): Musculoskeletal[23]; Psychological[23]; Skin[23]; Endocrine[23]; General and Unspecified[23]
Cognates:
Language contact:

***Piscidia* (Fabaceae)**

Spanish names:
Indigenous names: Ts'ihol, k'anaw te', chiihol[07]; Ha'abin[09]
Used by (2*): Huastec[07]; Yucatecan Maya[09]
Used for (7#): Blood[07]; Digestive[07, 09]; Neurological[07]; Respiratory[09]; Skin[07]; General and Unspecified[07]
Cognates:
Language contact:

***Pisonia* (Nyctaginaceae)**

Spanish names: Uña de gato
Indigenous names: Loh, itsik mitsu'[07]; Beeb[09]
Used by (2*): Huastec[07]; Yucatecan Maya[09]
Used for (3#): Skin[07]; Pregnancy[09]; General and Unspecified[07]
Cognates:
Language contact:

***Pitcairnia* (Bromeliaceae)**

Spanish names: Cebolla de Monte
Indigenous names: Ceboll pim, Seb'oyil pim, Cewoyil pim[14]
Used by (1*): Quichean Maya[14]
Used for (1#): Respiratory[14]
Cognates:
Language contact:

***Pithecellobium* (Fabaceae)**

Spanish names: Guamuchi; Guamúchil; Huamuchil; Palo de pinolio; Tucuy
Indigenous names: Poposyutil[01]; Umuw, umu[07]; Jumo[08]; So'sol (Xkenq)[14]; Te'aj tuk'uy[18]; Coamochitl[28]
Used by (8*): Zoque[01, 02]; Huastec[07, 08]; Quichean Maya[14]; Western Maya[18]; Zapotec[21]; Nahua[28]
Used for (24#): Digestive[01, 02, 07, 08, 14, 21, 28]; Cardiovascular[02]; Musculoskeletal[08]; Neurological[01, 07, 08]; Respiratory[08]; Skin[02, 18, 28]; Pregnancy[02]; Female genital[02]; Male genital[02]; General and Unspecified[07, 08, 14]
Cognates: Hua: umo/umu;
Language contact: Hua <> Nah

***Pityopsis* (Asteraceae)**

Spanish names:
Indigenous names: Poja muk[03]
Used by (1*): Zoque[03]
Used for (1#): Skin[03]
Cognates:
Language contact:

***Pityrogramma* (Pteridaceae)**

Spanish names: Helecho calado
Indigenous names: Jab kingin[01]; Poja chimal [03]; Jam ujts[04]; Roq chit cuan[16]
Used by (4*): Zoque[01, 03]; Mixe[04]; Quichean Maya[16]
Used for (8#): Digestive[01]; Neurological[16]; Respiratory[03]; Skin[03]; Urological[01]; Female genital[04]; Male genital[01]; General and Unspecified[16]
Cognates: MZ: jaC; Zoq: ja;
Language contact:

***Plantago* (Plantaginaceae)**

Spanish names: lanté; Lengua de perro/ lengua de vaca; Llanté; Llanté/lantera/lanter/lengua de vaca/ lengua de perro; Llanten; Llantena, santena
Indigenous names: Tu' isy tojtz/ tuwi isy totz/ tu' totz[01]; Tu' totz[01]; Lentej ay[03]; Uxkin imul, jal q'ayes[13]; Ojalante'[18]; Blàg-llàntên, blàg-sàntên, guìzh-gòdz, xín-guìzh-dòoz[23]
Used by (12*): Zoque[01, 03]; Totonac[06]; Quichean Maya[12, 13, 14]; Western Maya[18]; Zapotec[21, 22, 23]; Nahua[25, 26]
Used for (40#): Blood[12]; Digestive[01, 03, 12, 13, 14, 18, 22, 23, 25]; Eye[01, 13, 21]; Cardiovascular[12]; Musculoskeletal[01, 12, 13]; Psychological[12]; Respiratory[01, 18]; Skin[01, 12, 13, 26]; Endocrine[12]; Urological[01, 12, 26]; Female genital[01, 12, 18, 26]; Male genital[01]; General and Unspecified[06, 12]
Cognates: Zoq: tu'(wi) totz;
Language contact: Zoq <> Zap

***Platanus* (Platanaceae)**

Spanish names: Chote; Palo blanco
Indigenous names: Taktak[01]
Used by (2*): Zoque[01]; Nahua[25]
Used for (5#): Ear[25]; Musculoskeletal[01]; Endocrine[25]; Urological[25]; General and Unspecified[01]
Cognates:
Language contact:

***Platycladus* (Cupressaceae)**

Spanish names: Ciprés
Indigenous names:
Used by (1*): Yucatecan Maya[11]
Used for (1#): Respiratory[11]
Cognates:
Language contact:

***Plectranthus* (Lamiaceae)**

Spanish names: Copa de rey; Flor de mantu; Mantu morado/ manto morado; Oregano; Oregano castillo, oregano chino; Oregano orejón; Oreganón
Indigenous names: Ts'kawe'e[18]
Used by (6*): Zoque[01, 02, 03]; Yucatecan Maya[09]; Western Maya[18]; Nahua[25]
Used for (24#): Digestive[01, 02, 03, 25]; Eye[03]; Ear[02, 03, 09, 18]; Neurological[01, 18]; Respiratory[01, 03, 18]; Endocrine[02]; Urological[02]; Pregnancy[03, 18]; Female genital[01]; General and Unspecified[01, 02]
Cognates:
Language contact:

***Pleopeltis* (Polypodiaceae)**

Spanish names: Hierba de Palo con Mano, Calahuala
Indigenous names: K'amal Aqom Che' Qa'aj[12]
Used by (1*): Quichean Maya[12]
Used for (7#): Blood[12]; Cardiovascular[12]; Musculoskeletal[12]; Psychological[12]; Urological[12]; Male genital[12]; General and Unspecified[12]
Cognates:
Language contact:

***Pluchea* (Asteraceae)**

Spanish names: Canela del rio; Ciguapate; Flor de Santa Maria, canela del rio, hoja de canela, ; Hierba de canela; Hierba de venado/ ojo de venado/ sivipake/ hierba la playa; Hoja de aire; Hoja de cancer; Salvia; Santa Maria; Siguapate; Yerba de canela
Indigenous names: Mä'a ome/ mä'a wa'a[01]; Syaw'ay[02]; Salve ay[03]; Santa Maria aay[04]; K'aninmiin ts'ohool, hom ts'ohool,[07]; Chalche'[09]; Čaal Če[10]; Chalache[11]; Kewuhj[12]; Siguapate[13]; Mai pim[16]; Musik witzir[19]; Balagasana, gui'xaan[21]; Kwan zahn, kwan gusahn[22]; Guìzh-cànêl[23]; Chiquite[26]
Used by (17*): Zoque[01, 02, 03]; Mixe[04]; Huastec[07]; Yucatecan Maya[09, 10, 11]; Quichean Maya[12, 13, 16, 17]; Western Maya[19]; Zapotec[21, 22, 23]; Nahua[26]
Used for (65#): Digestive[01, 02, 03, 04, 07, 11, 21, 22]; Ear[04]; Cardiovascular[03]; Musculoskeletal[01, 02, 03, 07, 11, 12, 13, 19, 21]; Neurological[01, 07, 11, 12, 13, 16, 19]; Respiratory[03, 07]; Skin[02, 03, 11]; Urological[01, 03, 12]; Pregnancy[01, 02, 03, 07, 09, 10, 12, 19, 21, 22, 23]; Female genital[02, 03, 04, 19]; Male genital[01]; General and Unspecified[01, 02, 03, 07, 10, 11, 12, 19, 26]; nd[17]
Cognates: Yuc: chaalche; Zap: san;
Language contact:

***Plumbago* (Plumbaginaceae)**

Spanish names: Hierba del pez; Hoja de chivato
Indigenous names: Hurika[07]; Hurika[08]; Guish chivat[21]; Guìzh-nàad, guìzh-mòràdítò[23]
Used by (4*): Huastec[07, 08]; Zapotec[21, 23]
Used for (7#): Digestive[23]; Musculoskeletal[07]; Skin[07, 08, 21]; General and Unspecified[07, 08]
Cognates: Hua: hurik;
Language contact:

***Plumeria* (Apocynaceae)**

Spanish names: Cacalosúchitl, flor de mayo; Flor de cal; Flor de mayo; Flor de mayo simarron; Palo loco
Indigenous names: (Tzama) popo jäyä[01]; Puutx mooya[03]; San Juan pøh[04]; Caxta xanat[06]; Ukul wits[07]; Nikte'ch'om[09]; Guiatzatzii quitii[21]; Yàg-guièe-yăl[23]; Cacaloxochitl[24]; Cacahuaxóchitl[25]
Used by (10*): Zoque[01, 03]; Mixe[04]; Totonac[06]; Huastec[07]; Yucatecan Maya[09]; Zapotec[21, 23]; Nahua[24, 25]
Used for (23#): Digestive[03, 04, 06, 23, 25]; Eye[23]; Musculoskeletal[01, 04, 21]; Neurological[03]; Respiratory[21]; Skin[01, 07, 09, 21, 24]; Urological[01]; Female genital[01, 06, 21]; General and Unspecified[03, 04, 21]
Cognates: Nah: cacahuaxochitl;
Language contact:

***Pogostemon* (Lamiaceae)**

Spanish names: Oregano grande; Patchuli
Indigenous names: Perfumen ujts[04]; Yop'aj pachulin[18]
Used by (3*): Mixe[04]; Western Maya[18]; Zapotec[21]
Used for (8#): Digestive[18, 21]; Respiratory[21]; Pregnancy[21]; Female genital[21]; General and Unspecified[04, 18, 21]
Cognates:
Language contact:

***Poiretia* (Fabaceae)**

Spanish names: Hierba malina; Hoja malina
Indigenous names: Poj jäjä[02]; Malin ujts[04]; Guixa'a[21]
Used by (3*): Zoque[02]; Mixe[04]; Zapotec[21]
Used for (7#): Digestive[21]; Musculoskeletal[02, 04, 21]; Skin[21]; General and Unspecified[04, 21]
Cognates:
Language contact:

***Polianthes* (Asparagaceae)**

Spanish names: Azuzena
Indigenous names:
Used by (1*): Zoque[02]
Used for (2#): Respiratory[02]; Pregnancy[02]
Cognates:
Language contact:

***Polygala* (Polygalaceae)**

Spanish names: Anis, reumatuluc; Lavapie/flor de esquipulas/ hierba de seiscientos
Indigenous names: Kä tzanhga / kä tzanhgä[01]; Ueji ay[03]; Møøy ics[04]; Tsakam tsak mokok, ith ts'ohool[07]
Used by (5*): Zoque[01, 03]; Mixe[04]; Huastec[07]; Nahua[26]
Used for (9#): Digestive[03, 04]; Musculoskeletal[26]; Psychological[03]; Skin[01, 03, 07]; Pregnancy[07]; General and Unspecified[03]
Cognates:
Language contact:

***Polygonum* (Polygonaceae)**

Spanish names: Lirio de Agua
Indigenous names: Ehtiil tok'oy[07]; Aq'om Q'os ya' / Nuwulaj Abäj[12]
Used by (2*): Huastec[07]; Quichean Maya[12]
Used for (3#): Digestive[12]; Urological[12]; General and Unspecified[07]
Cognates:
Language contact:

***Polypodium* (Polypodiaceae)**

Spanish names: Calagual, canaguala; Calahuala; Helecho menor
Indigenous names: Tsa chimal[03]; Canagual[03]; Koo'te', tsooy ahaatik, kutsiil boo'waat, chuchim ts'ohool[07]; K'umatz Ruxe' Ibo'ch[12]; Bàz, guìzh-bàz[23]
Used by (5*): Zoque[03]; Huastec[07]; Quichean Maya[12, 13]; Zapotec[23]
Used for (30#): Digestive[03, 12, 13, 23]; Cardiovascular[03, 12]; Musculoskeletal[03, 12, 13]; Neurological[07, 12]; Psychological[12]; Respiratory[03, 23]; Skin[03, 12]; Endocrine[03, 12, 13]; Urological[03, 12]; Female genital[12]; General and Unspecified[07, 12, 13, 23]
Cognates:
Language contact:

***Polypremum* (Tetrachondraceae)**

Spanish names:
Indigenous names: Billushít[22]
Used by (1*): Zapotec[22]
Used for (1#): Digestive[22]
Cognates:
Language contact:

***Pontederia .* (Pontederiaceae)**

Spanish names:
Indigenous names: Xjolom Wakax[14]
Used by (1*): Quichean Maya[14]
Used for (4[#]): Digestive[14]; Urological[14]; Female genital[14]; General and Unspecified[14]
Cognates:
Language contact:

***Populus* (Salicaceae)**

Spanish names:
Indigenous names: Itsow[07]
Used by (1*): Huastec[07]
Used for (1[#]): Psychological[07]
Cognates:
Language contact:

***Porophyllum* (Asteraceae)**

Spanish names: Guapillo; Hoja de bruja/ de piojo; Hoja de orín; Papaloquelite; Papaloquelite, hoja quelite
Indigenous names: Comunk¥ ts¥p¥[03]; Tapahuelo[04]; Pucsnancac'a[06]; Mithith[07]; Xuk'ii[09]; So'sol Q'ehen (Macho)[14]; Sosol pim[16]; Gixa'a mbeetzii[21]
Used by (11*): Zoque[01, 03]; Mixe[04]; Totonac[06]; Huastec[07]; Yucatecan Maya[09]; Quichean Maya[14, 16]; Western Maya[18, 19]; Zapotec[21]
Used for (22[#]): Blood[03]; Digestive[03, 06, 07, 18, 21]; Cardiovascular[07]; Musculoskeletal[18, 21]; Neurological[04, 16]; Psychological[14, 16]; Skin[03, 18]; Urological[01]; Pregnancy[19]; General and Unspecified[03, 06, 09, 18, 21]
Cognates: Quich: sosol;
Language contact:

***Portulaca* (Portulacaceae)**

Spanish names: Verdolaga
Indigenous names: X'puhl cac'a[06]; Pitsits wal, he'pan paktha', tsakam ix thuyum[07]; Tsakam tsatsa, chak wits[07]; Pixla[12]; Paxlac[13]; Mixquilit[28]
Used by (7*): Totonac[06]; Huastec[07]; Quichean Maya[12, 13]; Zapotec[21]; Nahua[26, 28]
Used for (18[#]): Blood[06, 13]; Digestive[07, 12, 13, 28]; Respiratory[07]; Skin[07, 12]; Urological[12, 26]; Male genital[12]; General and Unspecified[06, 07, 12, 21, 28]
Cognates: Maya: pits/pix/pax; Quich: pix/pax;
Language contact:

***Pouteria* (Sapotaceae)**

Spanish names: Mamey; Mamey/zapote; Mamey/zapote (colorado); Zapote bianco; Zapote mamey
Indigenous names: Taki syapane/tzapas tzapane[01]; Tzapane/nui näjä/kawak[02]; Chu kuxamñi[03]; Kuxamñi[03]; Tsatsootso[03]; Jaka[05]; Jaaca[06]; Thokob[07]; Bolom it'ath[07]; Sakya'[09]; La'adxiguelexuunu'uj[21]
Used by (9*): Zoque[01, 02, 03]; Totonac[05, 06]; Huastec[07]; Yucatecan Maya[09, 11]; Zapotec[21]
Used for (27[#]): Digestive[03, 06, 09]; Cardiovascular[07]; Musculoskeletal[03, 21]; Psychological[03, 05]; Respiratory[03, 07]; Skin[01, 02, 03, 06, 07, 11, 21]; Pregnancy[03]; Female genital[03]; General and Unspecified[06, 21]
Cognates: Zoq: tsapane;
Language contact: Tot <> Yuc

***Pouzolzia* (Urticaceae)**

Spanish names:
Indigenous names: Uxum ilaal, tsakam baat[07]
Used by (1*): Huastec[07]
Used for (5[#]): Digestive[07]; Musculoskeletal[07]; Respiratory[07]; Pregnancy[07]; Female genital[07]
Cognates:
Language contact:

01-28 refer to the studies described in Table 4.1 * Total number of studies recording this taxon #Total number of use-records

***Prestonia* (Apocynaceae)**

Spanish names: Bejuco de jiote
Indigenous names: Kuts¥k[03]
Used by (1*): Zoque[03]
Used for (1#): Skin[03]
Cognates:
Language contact:

***Prionosciadium* (Apiaceae)**

Spanish names: Eneldo, chilibia; Eneldón
Indigenous names: Tzi, tunai, buxnay[13]
Used by (2*): Zoque[01]; Quichean Maya[13]
Used for (3#): Digestive[13]; Pregnancy[01]; General and Unspecified[13]
Cognates:
Language contact:

***Priva* (Verbenaceae)**

Spanish names: Cadillo de bolsa; Hoja de azar; Jehuite chicloso; Sogia de tusa
Indigenous names: Tza'a tzoy[01]; T¥pich nang tsang[03]; T'apay ts'ohool[07]; Tapay ts'ohool[08]; Xpak'umpak'[09]; Pax Pa'am[14]
Used by (8*): Zoque[01, 03]; Huastec[07, 08]; Yucatecan Maya[09]; Quichean Maya[14]; Western Maya[18]; Nahua[24]
Used for (11#): Digestive[03, 07, 09]; Musculoskeletal[08]; Neurological[08]; Skin[09, 18]; Pregnancy[24]; Female genital[03, 14]; General and Unspecified[01]
Cognates: Zoq: tsa; Maya: paC; Hua: tapay ts'ohool;
Language contact: Hua > Highland Popoluca

***Prosopis* (Fabaceae)**

Spanish names: Mesquite; Mezquite
Indigenous names: Ut'u[07]; Geč beh[22]; Mizquitl[28]
Used by (5*): Huastec[07]; Zapotec[21, 22]; Nahua[27, 28]
Used for (12#): Digestive[21, 22, 27, 28]; Eye[07, 21, 22, 27, 28]; Skin[21]; Pregnancy[27]; General and Unspecified[21]
Cognates:
Language contact:

***Prosthechea* (Orchidaceae)**

Spanish names:
Indigenous names: Ts'aak[07]
Used by (1*): Huastec[07]
Used for (1#): Endocrine[07]
Cognates:
Language contact:

***Protium* (Burseraceae)**

Spanish names: Copal; Copal de chichi; Copalillo
Indigenous names: Tzyutzyin bono/kutzyin bono/cochinbomo/Tzyitzyin bomo [01]; Puam, pum[05]; Homte', hom, ikob te'[07]; Pom[10]; Pom[11]
Used by (5*): Zoque[01]; Totonac[05]; Huastec[07]; Yucatecan Maya[10, 11]
Used for (14#): Digestive[01, 07]; Ear[07]; Musculoskeletal[01, 11]; Neurological[07, 10, 11]; Respiratory[01]; Skin[05]; General and Unspecified[05, 07, 10, 11]
Cognates: Maya: om; Yuc: pom;
Language contact: MZ > Tot & Maya

***Prunella* (Lamiaceae)**

Spanish names:
Indigenous names: Xinahuate[26]
Used by (2*): Quichean Maya[13]; Nahua[26]
Used for (2#): Skin[13, 26]
Cognates:
Language contact:

***Prunus* (Rosaceae)**

Spanish names: Cerezo; Chabacano; Ciruela; Durazno
Indigenous names: Tunas[01]; Tras[13]; K'ask'el[13]; Turesna[20]; Yàg-drâz[23]; Yàg-bziă[23]
Used by (8*): Zoque[01]; Totonac[06]; Quichean Maya[12, 13]; Western Maya[20]; Zapotec[23]; Nahua[25, 26]
Used for (28#): Digestive[01, 12, 13, 20, 23, 25, 26]; Cardiovascular[01]; Musculoskeletal[13]; Neurological[01, 12, 26]; Respiratory[01, 12, 13]; Skin[06, 12, 13, 25]; Pregnancy[06]; Female genital[01]; General and Unspecified[01, 12, 13]
Cognates:
Language contact:

***Pseudelephantopus* (Asteraceae)**

Spanish names: Lengua de vaca
Indigenous names: Lek'ab paakax[07]
Used by (2*): Zoque[03]; Huastec[07]
Used for (5#): Digestive[03]; Neurological[07]; Respiratory[03]; Skin[03]; Urological[03]
Cognates:
Language contact:

***Pseudobombax* (Malvaceae)**

Spanish names: Amabola blanco; Clavillina; Solosoche
Indigenous names: Tzospo/ a'pompo[02]; Pop uakta[03]; Mokok[07]; Siklite, Xk'uxche'[09]; Xiloxoochitl[24]
Used by (5*): Zoque[02, 03]; Huastec[07]; Yucatecan Maya[09]; Nahua[24]
Used for (12#): Digestive[07]; Psychological[07]; Respiratory[09]; Endocrine[02]; Urological[02, 03]; Pregnancy[03, 07, 24]; Female genital[03]; General and Unspecified[03, 07]
Cognates: Zoq: po(m)p;
Language contact:

***Pseudognaphalium* (Asteraceae)**

Spanish names: Gordolobo; Simonilla
Indigenous names: Popo 'uka tane/popo jäyä/pop tane/ akstäk tane[01]; Sìmònî[23]; Gòrdòlôb[23]
Used by (6*): Zoque[01, 02]; Zapotec[23]; Nahua[25, 26, 28]
Used for (17#): Blood[23]; Digestive[01, 23]; Cardiovascular[28]; Neurological[25]; Respiratory[01, 02, 23, 25, 26, 28]; Skin[01, 23]; Urological[28]; Pregnancy[23]; General and Unspecified[23, 28]
Cognates:
Language contact:

***Pseudogynoxys* (Asteraceae)**

Spanish names: Hierba de carga
Indigenous names: Tzämi tzoy[01]; Uentex ay[03]; Te'te' wits, chuklaab ts'ohool[07]; Kanal xijch'[20]
Used by (4*): Zoque[01, 03]; Huastec[07]; Western Maya[20]
Used for (4#): Digestive[20]; Skin[03]; General and Unspecified[01, 07]
Cognates:
Language contact:

***Psidium* (Myrtaceae)**

Spanish names: Guabillo; Guajaba sabanera; Guajava agria; Guayaba; Guayaba (dulce/agría); Guayaba agria; Guayaba cimmaron; Guayaba dulce; Guayabita/guayaba agria; Guayabo; Guayabo moreno; Guayava; Itamo real; Raiana; Rayana, guayaba arrayán; Rayanita, Rayana, Capulín de tierra
Indigenous names: Po'os/padan/paran/pataya[01]; Katzu patan/katzu paran/ katsu 'ätz/ katzsy patan/ katzu wos[01]; Poks/po's/ pataya[02]; Poks pataja/ katsu pataja/ toks pataja[02]; Nas mupe[02]; Katsu patan[03]; Patan[03]; Copa patan[03]; Pox[04]; Jokøønypapox tikts[04]; Asihuit, aci'huit, llasibit[05]; Aa'si'huiit[06]; Bek[07]; Bekil tooro, beekil an buuru[07]; Bek[08]; Pichi'[09]; Pichiche'[09]; PiČi[10]; Keq'[12]; (Kia/Chom) Kiej/keq'[13]; Pata'a[14]; Pata'[18]; Pataj[19]; Ixim pataj[19]; Pata, potov, potoj, poto'[20]; Paxchak', pajal pata/potoj, potov, poto'[20]; Guisha nguetuj[21]; Behuishxuba'a[21]; Bihuishuba'aj[21]; Bäwi[22]; Xalxocotl[24]; Xalxocotl[26]; Xalxócotl[28]
Used by (24*): Zoque[01, 02, 03]; Mixe[04]; Totonac[05, 06]; Huastec[07, 08]; Yucatecan Maya[09, 10, 11]; Quichean Maya[12, 13, 14]; Western Maya[18, 19, 20]; Zapotec[21, 22]; Nahua[24, 25, 26, 27, 28]
Used for (103#): Blood[12]; Digestive[01, 02, 03, 04, 05, 06, 07, 08, 09, 10, 11, 12, 13, 14, 18, 19, 20, 21, 22, 24, 25, 26, 27, 28]; Eye[20]; Ear[01]; Cardiovascular[02, 12]; Musculoskeletal[01, 12]; Neurological[01, 02, 08, 20]; Psychological[01, 12]; Respiratory[01, 02, 03, 07, 19, 20, 28]; Skin[01, 02, 03, 05, 07, 08, 09, 10, 14, 18, 25, 26]; Urological[01, 02, 03, 07, 26]; Pregnancy[02, 05, 13, 19]; Female genital[01, 02, 03, 13, 19, 21]; General and Unspecified[01, 02, 05, 09, 12, 13, 21, 28]
Cognates: MZ: pos / pox; Zoq: patan / pataja; Tot: asihuit; Maya: bek / keq; Hua: bek; Yuc: pichi; Quich: keq; WesM: pata; Zap: bewi; Nah: xalxocotl;
Language contact: Zoq > Kekchi & WesM

***Psiguria* (Cucurbitaceae)**

Spanish names:
Indigenous names:
Used by (1*): Zoque[03]
Used for (1#): Skin[03]
Cognates:
Language contact:

***Psittacanthus* (Loranthaceae)**

Spanish names: Hoja de urraca, mata palo
Indigenous names: Ok'lom te' puulik[07]; Guixa'a shaguii[21]; Guièe-ló-yâg-guièts, guièe-ló-yâg-nlìbâd-tsò[23]
Used by (3*): Huastec[07]; Zapotec[21, 23]
Used for (7#): Digestive[07]; Neurological[07, 23]; Skin[07, 21]; General and Unspecified[07, 21]
Cognates:
Language contact:

***Psychotria* (Rubiaceae)**

Spanish names: Cafecillo; Curamal; Hierba de sabañon; Hoja morada; Segunda Lengua del Perro; Simonillo
Indigenous names: Masan ay[03]; Tsus pitx cuy[03]; Tam txitx¥k[03]; Masan ay, Monch¥v ay[03]; Baina ts'ohool, wiichab ts'ohool, bekil papaam, k'animiin wits, mapux ts'ohool, bathex, ook' tse'tsem, paktha' wach'uy ch'ohool[07]; Tse'tsem ts'ohool, kapee ts'ohool, tsakam ts'abat, t'othooybe ts'ohool, baina ts'ohool, itsal kw'a, wats'ul, thuyum olom[07]; Tschul-keeh[09]; Š Anal[10]; Chäk k'änan[11]; Ix k'ä anal hembra[11]; Xkoti yuk[14]; Xkab' Rujiraq'i Tz'i'[14]; Tzuul Q'ehen re Ha'[14]; Na' Ichaj'[14]; Re Kanteel, Mai Tzuul[14]; Ak Pere Tzo', peren pim[15]; Saxjolom chilan[16]; Colaras[16]
Used by (10*): Zoque[03]; Huastec[07]; Yucatecan Maya[09, 10, 11]; Quichean Maya[14, 15, 16, 17]; Nahua[26]
Used for (38#): Digestive[03, 07, 11, 14]; Musculoskeletal[03, 14]; Neurological[09, 10, 14, 16]; Respiratory[14]; Skin[03, 07, 26]; Pregnancy[15]; Female genital[03, 14]; General and Unspecified[07, 09, 11, 14]; nd[17]
Cognates: Yuc: anal;
Language contact:

***Pteridium* (Dennstaedtiaceae)**

Spanish names: Costillludo/ tapa carbon; Helecho chispa; Ocopeta; Pesma
Indigenous names: Xip[13]
Used by (4*): Zoque[01, 03]; Quichean Maya[13]; Nahua[25]
Used for (7#): Musculoskeletal[13]; Psychological[03]; Respiratory[03, 25]; Endocrine[25]; Pregnancy[01]; General and Unspecified[13]
Cognates:
Language contact:

01-28 refer to the studies described in Table 4.1 * Total number of studies recording this taxon #Total number of use-records

Pteris **(Pteridaceae)**

Spanish names: Helecho liso; Helecho macho
Indigenous names: Taksy kingin/ pu'kuy tuks[01]; Mä'a kingin[01]
Used by (2*): Zoque[01]; Quichean Maya[16]
Used for (8#): Digestive[01]; Psychological[16]; Endocrine[01]; Urological[01]; Male genital[01]; General and Unspecified[01, 16]
Cognates:
Language contact:

Pterocarpus **(Fabaceae)**

Spanish names:
Indigenous names: Akxcuy[03]
Used by (1*): Zoque[03]
Used for (2#): Skin[03]; Female genital[03]
Cognates:
Language contact:

Punica **(Lythraceae)**

Spanish names: Granada
Indigenous names:
Used by (1*): Western Maya[19]
Used for (1#): Digestive[19]
Cognates:
Language contact:

Punica **(Lythraceae)**

Spanish names: Granada; Granado; Grenadillo
Indigenous names: Laab bek[07]; Žob štil[22]; Yàg-ngùd-guièe-ziè, yàg-grànâd[23]
Used by (14*): Zoque[01, 02, 03]; Mixe[04]; Huastec[07]; Yucatecan Maya[09, 10]; Quichean Maya[12, 13]; Western Maya[18]; Zapotec[21, 22, 23]; Nahua[28]
Used for (25#): Blood[02]; Digestive[01, 02, 03, 04, 07, 09, 12, 18, 21, 22, 23, 28]; Eye[13]; Musculoskeletal[21]; Neurological[12, 28]; Psychological[12]; Respiratory[21]; Skin[02, 09, 21]; Pregnancy[10]; Female genital[21]; General and Unspecified[12]
Cognates:
Language contact:

Quassia **(Simaroubaceae)**

Spanish names: Cuassia
Indigenous names:
Used by (1*): Zapotec[21]
Used for (1#): Digestive[21]
Cognates:
Language contact:

Quercus **(Fagaceae)**

Spanish names: Encino; Encino amarillo; Encino blanco; Encino blanco/nanche; Encino nanche/encino rojo; Encino negro; Encino rojo; Encino, Roble; Roble; Roble blanco
Indigenous names: Popo kamay[01]; Pak soj/syoj[02]; Popo soj[02]; Cap soj / Tsabats soj[03]; Puutx soj[03]; Pop soj[03]; Y¥k soj[03]; Soj[03]; Ok soj[03]; Poop xoj[04]; Jiny xoj[04]; Tsapt xoj, poop xoj[04]; Patän, Raxche'[12]; Pichik[12]; Patän che', Räx che'[12]; Sk'el, squ'el, tuluk, tux[13]; Ch'oror[19]; Sak yok jij te'[20]; K'an tulan[20]; Beshxii qutzii[21]; Beshxii nagatzii[21]
Used by (9*): Zoque[01, 02, 03]; Mixe[04]; Quichean Maya[12, 13]; Western Maya[19, 20]; Zapotec[21]
Used for (53#): Blood[03]; Digestive[01, 02, 03, 04, 12, 13, 19, 20, 21]; Cardiovascular[12]; Musculoskeletal[02, 12]; Neurological[01, 02, 03, 12, 13, 19]; Respiratory[02]; Skin[02, 03, 04, 12, 13]; Endocrine[01]; Pregnancy[03]; Female genital[02, 03, 04, 21]; General and Unspecified[12, 13, 21]
Cognates: MZ: soj/xoj; CoreM: tulan/tuluk;
Language contact:

01-28 refer to the studies described in Table 4.1 * Total number of studies recording this taxon #Total number of use-records

Randia **(Rubiaceae)**

Spanish names: Morro
Indigenous names: Jeepe ay, Chuk wikpak[03]; Tsotsoob olom, tsetsbaal i olom, kruus k'iith[07]; K'ax[09]
Used by (4*): Zoque[01, 03]; Huastec[07]; Yucatecan Maya[09]
Used for (8#): Digestive[01, 07]; Neurological[03]; Psychological[03]; Skin[03, 07]; General and Unspecified[03, 09]
Cognates:
Language contact:

Ranunculus **(Ranunculaceae)**

Spanish names: Pata de león, mano de león; Tijebete
Indigenous names: Xat uxaj'[13]
Used by (2*): Quichean Maya[13]; Nahua[26]
Used for (5#): Musculoskeletal[26]; Skin[13, 26]; Endocrine[26]; General and Unspecified[13]
Cognates:
Language contact:

Raphanus **(Brassicaceae)**

Spanish names: Rabano
Indigenous names:
Used by (5*): Zoque[01]; Yucatecan Maya[09]; Quichean Maya[12, 13]; Zapotec[23]
Used for (12#): Digestive[12, 13]; Cardiovascular[12]; Musculoskeletal[12]; Respiratory[01, 09, 13]; Skin[13]; Urological[12]; Male genital[12]; General and Unspecified[12, 23]
Cognates:
Language contact:

Rauvolfia **(Apocynaceae)**

Spanish names: Chalchupa; Veneno de perro
Indigenous names: Itsaan an k'ak'al ilaal[07]; Kambamuk[09]; Sisar k'opot[19]; Guanabajcu[21]
Used by (5*): Zoque[03]; Huastec[07]; Yucatecan Maya[09]; Western Maya[19]; Zapotec[21]
Used for (8#): Skin[03, 09, 21]; Pregnancy[19]; Female genital[03, 19, 21]; General and Unspecified[07]
Cognates:
Language contact:

Renealmia **(Zingiberaceae)**

Spanish names:
Indigenous names: Jua[01]; Jua'/tzuku' waja[02]; Tz'i'[14]
Used by (3*): Zoque[01, 02]; Quichean Maya[14]
Used for (4#): Musculoskeletal[02, 14]; Skin[02]; General and Unspecified[01]
Cognates: Zoq: jua;
Language contact:

Rhamnus **(Rhamnaceae)**

Spanish names:
Indigenous names: Itsil, ichil[07]
Used by (1*): Huastec[07]
Used for (1#): General and Unspecified[07]
Cognates:
Language contact:

***Rheum* (Polygonaceae)**

Spanish names: Ruibarbo
Indigenous names:
Used by (1*): Quichean Maya[12]
Used for (3#): Blood[12]; Digestive[12]; Cardiovascular[12]
Cognates:
Language contact:

***Rhipidocladum* (Poaceae)**

Spanish names: Carrizo
Indigenous names: Aj Che K'etchelaj[12]
Used by (1*): Quichean Maya[12]
Used for (2#): Musculoskeletal[12]; Urological[12]
Cognates:
Language contact:

***Rhipsalis* (Cactaceae)**

Spanish names: Injerto, solitaria, niguilla
Indigenous names: Paka hui'huat[06]; Xi'il boo'waat, xi'ixl uxum, weew i path uut', tsakam pak'ak', okôlom te', kwathnab ts'aah[07]; Roq Ak'ach Q'ehen[14]
Used by (3*): Totonac[06]; Huastec[07]; Quichean Maya[14]
Used for (10#): Cardiovascular[07]; Musculoskeletal[07]; Neurological[07]; Psychological[07]; Skin[07, 14]; Endocrine[06]; Urological[07]; General and Unspecified[07, 14]
Cognates: Maya: ak'a;
Language contact: Tot <> Hua

***Rhododendron* (Ericaceae)**

Spanish names: Azalia blanca
Indigenous names:
Used by (1*): Nahua[26]
Used for (1#): Respiratory[26]
Cognates:
Language contact:

***Rhodosciadium* (Apiaceae)**

Spanish names:
Indigenous names: Guiéer-ngüèets, xín-pèrèjîl[23]
Used by (1*): Zapotec[23]
Used for (1#): Urological[23]
Cognates:
Language contact:

***Rhus* (Anacardiaceae)**

Spanish names: Sal de venado; Zumaque
Indigenous names: Katxu ay[03]; Rtz'am mzat/kiej[13]; B'iritaq[14]; Yàg-bèch-mbăr[23]; Xín-bèch-mbăr[23]; Yàg-bèch-lsæ̀b[23]
Used by (6*): Zoque[01, 03]; Quichean Maya[12, 13, 14]; Zapotec[23]
Used for (15#): Digestive[03, 14]; Cardiovascular[12]; Musculoskeletal[12, 13]; Neurological[12]; Skin[12, 13, 23]; Urological[03]; Female genital[03]; General and Unspecified[01, 12]
Cognates:
Language contact:

01-28 refer to the studies described in Table 4.1 * Total number of studies recording this taxon #Total number of use-records

***Rhynchosia* (Fabaceae)**

Spanish names: Ojo de picho
Indigenous names: Sinchu ixcuy[03]; Tsanakw'iil t'eel[07]
Used by (2*): Zoque[03]; Huastec[07]
Used for (5#): Skin[03, 07]; Pregnancy[03]; Female genital[03]
Cognates:
Language contact:

***Rhynchospora* (Cyperaceae)**

Spanish names:
Indigenous names: Tathiim toom[07]
Used by (1*): Huastec[07]
Used for (1#): Digestive[07]
Cognates:
Language contact:

***Richardia* (Rubiaceae)**

Spanish names: Cola de Alacrán
Indigenous names:
Used by (1*): Quichean Maya[12]
Used for (3#): Cardiovascular[12]; Musculoskeletal[12]; General and Unspecified[12]
Cognates:
Language contact:

***Ricinus* (Euphorbiaceae)**

Spanish names: Grilla, higuerilla; Higuera; Higuerilla; Higuerilla/higuera; Higuerrilla; Higuerrillo; Huerilla; Huigerilla; Rizino
Indigenous names: Kaslam tatki/kasy la wängi/kasyla wong [01]; Tätz kin/ tzäksy kin ay[02]; Nuku tsoy[03]; Tsak tsooy[04]; Kastalan quajne[05]; Thikeela'[07]; K'axtelenkh'et[08]; Xk'ooch[09]; ŠkotČ[10]; Ix k'o'och[11]; Azeta[12]; Acete[13]; Ch'apaky[18]; Yaga huegu'u[21]; Baláp[22]; Yàg-blàp[23]; Xepowiwtl[28]
Used by (21*): Zoque[01, 02, 03]; Mixe[04]; Totonac[05, 06]; Huastec[07, 08]; Yucatecan Maya[09, 10, 11]; Quichean Maya[12, 13]; Western Maya[18]; Zapotec[21, 22, 23]; Nahua[25, 26, 27, 28]
Used for (65#): Blood[12]; Digestive[01, 02, 04, 05, 06, 07, 08, 09, 12, 18, 21, 22, 23, 26, 27, 28]; Eye[13]; Cardiovascular[02]; Musculoskeletal[01, 02, 03, 12, 13, 23, 25]; Neurological[07, 08, 09, 10, 13, 22]; Respiratory[02, 07, 25, 26, 27]; Skin[01, 02, 03, 07, 21, 22]; Urological[02]; Pregnancy[01, 07, 13]; Female genital[01, 18]; General and Unspecified[01, 03, 04, 05, 07, 09, 10, 11, 12, 13, 18, 22, 25, 27, 28]
Cognates:
Language contact:

***Rivina* (Phytolaccaceae)**

Spanish names: Baja tripa; Chilio; Colario
Indigenous names: Yawa niwi [01]; Masan ay[03]; Pinil štayat[05]; Taa' t'ele', ts'amuxlaab ts'ohool, luuk, tsakam tsak tuthay, tsakam k'ak'al xeklek[07]; Ikiche'[09]; Baq'nel pim[14]
Used by (6*): Zoque[01, 03]; Totonac[05]; Huastec[07]; Yucatecan Maya[09]; Quichean Maya[14]
Used for (19#): Digestive[03, 05, 07, 14]; Cardiovascular[14]; Musculoskeletal[03, 14]; Neurological[14]; Psychological[14]; Respiratory[14]; Skin[03, 07, 09, 14]; Urological[01]; Pregnancy[01]; General and Unspecified[05, 07, 14]
Cognates:
Language contact:

***Roldana* (Asteraceae)**

Spanish names: Hierba / Hoja de Queso
Indigenous names: Tzyaptos[01]; Sup[12]
Used by (2*): Zoque[01]; Quichean Maya[12]
Used for (3#): Respiratory[01, 12]; General and Unspecified[12]
Cognates:
Language contact:

01-28 refer to the studies described in Table 4.1 * Total number of studies recording this taxon #Total number of use-records

Rollinia **(Annonaceae)**

Spanish names: Anona
Indigenous names: Paj yatyi[03]
Used by (1*): Zoque[03]
Used for (1#): Digestive[03]
Cognates:
Language contact:

Ronabea **(Rubiaceae)**

Spanish names:
Indigenous names: Ix k'ä anal macho[11]
Used by (1*): Yucatecan Maya[11]
Used for (1#): Digestive[11]
Cognates:
Language contact:

Rosa **(Rosaceae)**

Spanish names: Flor de concha, rosa concha; Flor roja/rosa/rosa de castilla/ rosa china, Rosa blanca/ rosa de concha; Isabelita; Rosa; Rosa Blanca; Rosa blanca/ de castilla; Rosa china; Rosa concha; Rosa de castill; Rosa de castilla; Rosa roja
Indigenous names: Tzapas jäyä, Popo jäyä[01]; Gloorya wits, k'iith wits[07]; Ran xux, arxux, anaxux[13]; Nich i blanca[18]; Nich i roja[18]; Nich i castilla[18]; U nich pat[18]; Guièe-rôs[23]
Used by (13*): Zoque[01, 02, 03]; Huastec[07]; Yucatecan Maya[09]; Quichean Maya[12, 13]; Western Maya[18]; Zapotec[21, 22, 23]; Nahua[26, 27]
Used for (63#): Digestive[01, 02, 03, 21, 22, 23, 26, 27]; Eye[01, 02, 03, 13, 18, 21, 22, 27]; Ear[01]; Musculoskeletal[01, 02]; Neurological[03, 12, 18]; Psychological[01, 03, 12, 18]; Respiratory[01, 02, 03, 07, 09, 18, 26]; Skin[02, 03, 13, 23, 27]; Urological[02]; Pregnancy[01, 23]; Female genital[02, 03, 21]; Male genital[02]; General and Unspecified[01, 02, 03, 07, 12, 13, 18, 21, 22, 23, 26]
Cognates:
Language contact:

Roseodendron **(Bignoniaceae)**

Spanish names: Primavera
Indigenous names:
Used by (1*): Zoque[01]
Used for (1#): Musculoskeletal[01]
Cognates:
Language contact:

Rosmarinus **(Lamiaceae)**

Spanish names: Romero
Indigenous names:
Used by (16*): Zoque[01, 02, 03]; Mixe[04]; Totonac[05]; Huastec[08]; Yucatecan Maya[09]; Quichean Maya[12, 13, 14]; Western Maya[19]; Zapotec[21, 22, 23]; Nahua[25, 28]
Used for (50#): Blood[12]; Digestive[01, 02, 08, 12, 21, 22, 25, 28]; Cardiovascular[12]; Musculoskeletal[02, 03, 08, 12, 23]; Neurological[12]; Psychological[03, 21]; Respiratory[01, 02, 12, 25, 28]; Skin[01, 08, 12, 25]; Urological[12]; Pregnancy[01, 02, 03, 09, 19, 21, 22, 23]; Female genital[01, 02, 12, 19]; General and Unspecified[01, 04, 05, 08, 12, 13, 14, 22, 23, 28]
Cognates:
Language contact:

Roupala **(Proteaceae)**

Spanish names: Palo de cucaracha
Indigenous names: T¥kchicuy[03]
Used by (1*): Zoque[03]
Used for (1#): Female genital[03]
Cognates:
Language contact:

***Rourea* (Connaraceae)**

Spanish names:
Indigenous names: It'iib chuch[07]
Used by (1*): Huastec[07]
Used for (4#): Musculoskeletal[07]; Neurological[07]; Skin[07]; General and Unspecified[07]
Cognates:
Language contact:

***Rubus* (Rosaceae)**

Spanish names: Mora; Mora simarron/mora agria; Sarsamora; Zarzamora
Indigenous names: (Katzu) watzan[01]; Watzan[01]; Tocan[12]; Tucán[13]; Makom, makum[20]
Used by (4*): Zoque[01]; Quichean Maya[12, 13]; Western Maya[20]
Used for (19#): Digestive[01, 13, 20]; Eye[13]; Musculoskeletal[01]; Psychological[01]; Respiratory[12, 13, 20]; Urological[01]; Male genital[01]; General and Unspecified[01, 12, 13, 20]
Cognates: Quich: tokan/tukan;
Language contact:

***Ruellia* (Acanthaceae)**

Spanish names: Cuamaite; Hierba de chivo/ barba de chivo
Indigenous names: Tzivo angbäk[01]; Tsus ay[03]; Poj ujts[04]; Lisakan[05]; Tsab k'a'um, pithomlaab ts'ohool, tsakam tsahib, tok ts'ohool, ebchil k'a'um, ts'itsiimbe ts'ohool, bo' k'a'um[07]; Tsamnek muu, pohoth ts'ohool, eem muuw, xutsun bat'aw, uxum kw'ahiilom[07]; Kabalya'axnik[09]
Used by (6*): Zoque[01, 03]; Mixe[04]; Totonac[05]; Huastec[07]; Yucatecan Maya[09]
Used for (16#): Blood[07]; Digestive[03]; Eye[09]; Musculoskeletal[01]; Neurological[01, 07]; Respiratory[01]; Skin[03]; Urological[09]; Pregnancy[05]; General and Unspecified[01, 04, 07]
Cognates:
Language contact:

***Rumex* (Polygonaceae)**

Spanish names: Lengua de vaca; Lengua de Vaca de Agua; Verdolaga
Indigenous names: Paasii' ma kaa't[06]; Aq'om Raq' Wakx[12]; Raqwax[12]; Raq' wak'as[13]; Blàg-dòoz[23]; Xocoquilitl[26]
Used by (5*): Totonac[06]; Quichean Maya[12, 13]; Zapotec[23]; Nahua[26]
Used for (15#): Blood[13]; Digestive[06, 12]; Musculoskeletal[23]; Neurological[23]; Skin[12, 23, 26]; Urological[12, 26]; Pregnancy[13]; Female genital[12]; General and Unspecified[12, 23]
Cognates:
Language contact:

***Russelia* (Plantaginaceae)**

Spanish names: Barra de San Jose; Hoja de cerilla; Tronadora
Indigenous names: Kiñi n¥pin mooya[03]; Aneeymats[04]; Kutsiilte', kwayab an San Husee, thoot wits, toom wits, weeu koox[07]; Kwayab ts'aale, kutsiilte', ook' ts'een ts'ohool[07]; Siik'xiw, Oxletk'ax[09]; guish crii[21]
Used by (6*): Zoque[01, 03]; Mixe[04]; Huastec[07]; Yucatecan Maya[09]; Zapotec[21]
Used for (13#): Digestive[01, 03, 04]; Skin[03, 09, 21]; Endocrine[03]; Urological[03]; Female genital[03, 07]; General and Unspecified[04, 07]
Cognates:
Language contact:

Ruta **(Rutaceae)**

Spanish names: Ruda
Indigenous names: luta[01]; Wits'ii te' ts'ojol[08]; Rurá[13]; Tujyan ixik[19]; Lula[20]; Rrûd[23]; Temalacatl[28]
Used by (20*): Zoque[01, 02, 03]; Mixe[04]; Totonac[06]; Huastec[08]; Yucatecan Maya[09, 11]; Quichean Maya[12, 13]; Western Maya[18, 19, 20]; Zapotec[21, 22, 23]; Nahua[25, 26, 27, 28]
Used for (109#): Blood[01, 12]; Digestive[01, 02, 03, 04, 06, 08, 09, 12, 13, 18, 20, 22, 23, 25, 26, 27, 28]; Eye[01, 21]; Ear[01, 02, 04, 08, 13]; Cardiovascular[01, 02, 12, 13, 18, 21, 23, 25]; Musculoskeletal[01, 02, 03, 08, 18, 19, 22, 28]; Neurological[01, 08, 09, 12, 13, 18, 19, 21, 22, 25, 27, 28]; Psychological[01, 02, 03, 08, 13, 18, 19, 22, 23, 27]; Respiratory[01, 08, 18, 19, 25, 27]; Skin[01, 02, 03, 08, 13, 18, 23]; Urological[02]; Pregnancy[02, 03, 04, 06, 13, 18, 22, 25, 26]; Female genital[01, 04, 06, 12, 13, 18]; General and Unspecified[01, 03, 06, 08, 11, 12, 13, 18, 19, 21, 22, 23, 25, 26, 27, 28]
Cognates:
Language contact:

Rytidostylis **(Cucurbitaceae)**

Spanish names: Hoja de carga
Indigenous names: Tzämi ay[01]
Used by (1*): Zoque[01]
Used for (1#): General and Unspecified[01]
Cognates:
Language contact:

Sabal **(Arecaceae)**

Spanish names: Cocobal; Guano; Palma real
Indigenous names: Jojo[02]; Ka'nal-xa'an[09]; Yopo'jojobal[18]; Xi'inaaj[21]
Used by (4*): Zoque[02]; Yucatecan Maya[09]; Western Maya[18]; Zapotec[21]
Used for (6#): Digestive[18]; Musculoskeletal[21]; Skin[21]; Pregnancy[02, 09]; Female genital[09]
Cognates:
Language contact: Chimalapa Zoq <> Tabasco Chontal

Sabicea **(Rubiaceae)**

Spanish names:
Indigenous names: Colaras[16]; T'u zub k'aham[17]
Used by (2*): Quichean Maya[16, 17]
Used for (2#): Neurological[16]; nd[17]
Cognates:
Language contact:

Saccharum **(Poaceae)**

Spanish names: Caña de azúcar
Indigenous names: Cha'ncat[05]; Cha'ncat[06]; Pakab[07]
Used by (3*): Totonac[05, 06]; Huastec[07]
Used for (6#): Digestive[06, 07]; Neurological[05]; Respiratory[05]; Skin[05]; General and Unspecified[06]
Cognates:
Language contact:

Sageretia **(Rhamnaceae)**

Spanish names: Espina de corona
Indigenous names: Masan kobak apitx[03]
Used by (2*): Zoque[01, 03]
Used for (5#): Digestive[03]; Endocrine[01]; Urological[03]; Female genital[03]; General and Unspecified[03]
Cognates:
Language contact:

***Salix* (Salicaceae)**

Spanish names: Sauce; Taray
Indigenous names: Weksya[02]; Nø aweey[04]; Mak'astakat[06]; Tok'oy, hili te'[07]; Skab'ya'[12]; Sk'os[13]; Te'aj sausal[18]; Yàg-zhguiès[23]
Used by (9*): Zoque[02]; Mixe[04]; Totonac[06]; Huastec[07]; Quichean Maya[12, 13]; Western Maya[18]; Zapotec[21, 23]
Used for (25#): Digestive[21]; Cardiovascular[12]; Musculoskeletal[12, 21, 23]; Neurological[12, 18]; Psychological[02, 07, 23]; Respiratory[02]; Skin[04, 21, 23]; Endocrine[23]; Urological[02, 06]; Pregnancy[23]; Female genital[12]; General and Unspecified[02, 07, 12, 13, 21, 23]
Cognates: MZ: we; Quich: skV;
Language contact:

***Salmea* (Asteraceae)**

Spanish names: Palo de chile; Rayanita
Indigenous names: Nas mupe[02]; Ix ts'aah, , it'iib to'ol, ix ch'aah[07]; Yagaguina'a[21]; Yàg-guìin, guìzh-yàg-guìin [23]
Used by (4*): Zoque[02]; Huastec[07]; Zapotec[21, 23]
Used for (8#): Digestive[02]; Neurological[07]; Respiratory[07, 21]; Skin[07, 21]; General and Unspecified[21, 23]
Cognates: Zap: guin;
Language contact:

***Salpianthus* (Nyctaginaceae)**

Spanish names: Pie de paloma
Indigenous names: Guish pileej[21]
Used by (2*): Zoque[02]; Zapotec[21]
Used for (5#): Digestive[02]; Musculoskeletal[02]; Female genital[02]; General and Unspecified[02, 21]
Cognates:
Language contact:

***Salvia* (Lamiaceae)**

Spanish names: Albahaca silvestre; Alúcema; Alucema, hierba del ciervo, flor azul; Alusema; Contrahierba; Flor de gorrión, salvia; Flor de Jericó; Limpia; Mirto; Pezuña de caballo, Uñas de caballo; Salvia; Salvia morada; Tlanchichinole; Verbena
Indigenous names: Tzuji ay[01]; Poja way cuy[03]; Tsus ay[03]; Møj ku'uk ujts[04]; Pategen ujts[04]; Makakašahuat[05]; Hut'ut' wits, uxum ts'ohool, wayma', witsal a k'iitsaa[07]; Chaktsits[09]; Xiax-k'ax, Chi-k'ak'[09]; Chäk ta pek'[11]; Ajob q'yes[13]; Tatz'na'[13]; Zeh la[22]; Lùsêm[23]; Ncuàan-zân-làs[23]; Guièe-dzǐng, guìzh-dzǐng, guìzh-guièe-dzǐng, guièe-měets[23]; Blàg-guìtsiè, xín-lùzêm[23]; Tlanchichinole[25]; Tepechichia[26]; Tepechichia, ixcaxihuitl[26]; Tochimixochitl[28]
Used by (15*): Zoque[01, 03]; Mixe[04]; Totonac[05]; Huastec[07]; Yucatecan Maya[09, 11]; Quichean Maya[12, 13]; Zapotec[21, 22, 23]; Nahua[25, 26, 28]
Used for (76#): Digestive[01, 03, 05, 07, 09, 12, 13, 23, 25, 26, 28]; Eye[07]; Ear[05]; Cardiovascular[13]; Musculoskeletal[12, 13]; Neurological[12]; Psychological[07, 22, 23]; Respiratory[03, 05, 09, 12, 13]; Skin[03, 04, 09, 12, 13, 21, 22, 23, 25]; Endocrine[12]; Urological[12, 25]; Pregnancy[05, 07, 13, 22, 23]; Female genital[03, 07, 12, 13, 23]; Male genital[12]; General and Unspecified[03, 05, 09, 11, 12, 13, 23, 26, 28]
Cognates: Yuc: chäk/chak; Zap: zeh la / zan la; Nah: chichi;
Language contact:

***Sambucus* (Adoxaceae)**

Spanish names: Flor de sacuo; Flor de sauco; Sauco
Indigenous names: Okok yui/ok yui/ä'ju rane[01]; Toxeem[04]; Toxiba, tokxihua, toqsiwi, toquiwa[05]; Sawko[07]; Tunay' che', tzolo'j che', Tunayche' Aq'om[12]; Zoloji, shiij, tz'oloj che', xubam[13]; Yop'aj sauk'u[18]; Ch'ijil te'[20]; Yàg-saûz, yàzmîn[23]; Xometl[26]; Xometl[28]
Used by (16*): Zoque[01, 02, 03]; Mixe[04]; Totonac[05]; Huastec[07]; Quichean Maya[12, 13]; Western Maya[18, 20]; Zapotec[21, 22, 23]; Nahua[25, 26, 28]
Used for (75#): Blood[01, 03]; Digestive[01, 02, 03, 05, 13, 20, 25, 26]; Eye[01, 21, 22]; Cardiovascular[12, 25]; Musculoskeletal[01, 02, 05, 12, 13, 21]; Neurological[01, 02, 03, 12, 25]; Psychological[01, 05, 18]; Respiratory[01, 02, 03, 04, 05, 07, 12, 13, 18, 23, 25, 26, 28]; Skin[01, 02, 03, 05, 12, 13, 21, 22, 28]; Urological[02, 12, 13]; Pregnancy[05, 13]; Female genital[02, 03, 12, 13, 21]; Male genital[02, 12]; General and Unspecified[01, 02, 03, 05, 12, 13, 18, 21, 25, 26, 28]
Cognates: Nah: xometl;
Language contact: Mixe <>Tot <> K'iche'

Samolus (Primulaceae)

Spanish names:
Indigenous names: Tsunya'hi[09]; Ncuàan-dzéb-nquĭts[23]
Used by (2*): Yucatecan Maya[09]; Zapotec[23]
Used for (4#): Musculoskeletal[09]; Respiratory[09]; Skin[09]; General and Unspecified[23]
Cognates:
Language contact:

Samyda (Salicaceae)

Spanish names:
Indigenous names: Naranja che'[09]
Used by (1*): Yucatecan Maya[09]
Used for (2#): Skin[09]; Endocrine[09]
Cognates:
Language contact:

Sanchezia (Acanthaceae)

Spanish names: Pavo Real
Indigenous names: Xjolom Chaqmut (Sak)[14]
Used by (2*): Quichean Maya[12, 14]
Used for (2#): Musculoskeletal[12]; General and Unspecified[14]
Cognates:
Language contact:

Sansevieria (Asparagaceae)

Spanish names: Cola de tigre; Curalina; Curarina
Indigenous names: Tzikin o [01]; Txikiña[03]; Nej baläm[18]
Used by (8*): Zoque[01, 02, 03]; Yucatecan Maya[11]; Quichean Maya[12, 13]; Western Maya[18, 19]
Used for (16#): Digestive[01, 13]; Cardiovascular[13]; Musculoskeletal[18]; Neurological[13, 19]; Skin[01, 02, 03, 12, 13, 19]; General and Unspecified[11, 12, 13, 19]
Cognates:
Language contact:

Sanvitalia (Asteraceae)

Spanish names: Hoja de azar
Indigenous names: Tza'a tzoy[01]
Used by (1*): Zoque[01]
Used for (1#): General and Unspecified[01]
Cognates:
Language contact:

Sapindus (Sapindaceae)

Spanish names: Jaboncillo
Indigenous names: Tsukma[03]; Walul[07]; Bijpiij[21]
Used by (3*): Zoque[03]; Huastec[07]; Zapotec[21]
Used for (5#): Digestive[07]; Skin[03, 21]; General and Unspecified[07, 21]
Cognates:
Language contact:

***Sapranthus* (Annonaceae)**

Spanish names:
Indigenous names: Chuyu chajum,sakelemuy[09]
Used by (1*): Yucatecan Maya[09]
Used for (1#): Urological[09]
Cognates:
Language contact:

***Sarcoglottis* (Orchidaceae)**

Spanish names:
Indigenous names: Ts'ik'aach ts'ohool[07]
Used by (1*): Huastec[07]
Used for (1#): Pregnancy[07]
Cognates:
Language contact:

***Saurauia* (Actinidiaceae)**

Spanish names: Llorasangre hembra
Indigenous names: Q'ayis Aq'om Kik' Ixoq[12]
Used by (1*): Quichean Maya[12]
Used for (8#): Digestive[12]; Cardiovascular[12]; Musculoskeletal[12]; Neurological[12]; Respiratory[12]; Endocrine[12]; Urological[12]; General and Unspecified[12]
Cognates:
Language contact:

***Schinus* (Anacardiaceae)**

Spanish names: Coabino, pirul; Pirú; Pirul
Indigenous names: Ya luj[22]; Yàg-pìrûl[23]; Peloncuáhuitl[28]
Used by (4*): Zapotec[22, 23]; Nahua[27, 28]
Used for (11#): Digestive[22]; Eye[22]; Musculoskeletal[23]; Neurological[22]; Respiratory[28]; Skin[22]; Pregnancy[22, 27]; General and Unspecified[22, 27, 28]
Cognates:
Language contact:

***Schinus* (Anacardiaceae)**

Spanish names: Pirul
Indigenous names:
Used by (1*): Zoque[02]
Used for (1#): General and Unspecified[02]
Cognates:
Language contact:

***Schistocarpha* (Asteraceae)**

Spanish names:
Indigenous names: Pänpäm[01]; Sununkil Q'ehen, Jolam Pek Q'ehen[14]
Used by (3*): Zoque[01, 03]; Quichean Maya[14]
Used for (5#): Musculoskeletal[01, 14]; Neurological[14]; Respiratory[14]; Skin[03]
Cognates:
Language contact:

Schkuhria (Asteraceae)

Spanish names: Hierba del vendao
Indigenous names: Its'amal ts'ojol[08]; Gbày[23]
Used by (2*): Huastec[08]; Zapotec[23]
Used for (5#): Digestive[08]; Musculoskeletal[08]; Neurological[08]; Skin[08, 23]
Cognates:
Language contact:

Schoenocaulon (Melanthiaceae)

Spanish names:
Indigenous names: Tsuk tyiñ[03]; Sebadiya[07]
Used by (2*): Zoque[03]; Huastec[07]
Used for (2#): Skin[03, 07]
Cognates:
Language contact:

Schoepfia (Schoepfiaceae)

Spanish names:
Indigenous names: Its'am te', yax ich'am te'[07]
Used by (1*): Huastec[07]
Used for (3#): Digestive[07]; Neurological[07]; Pregnancy[07]
Cognates:
Language contact:

Schrankia (Fabaceae)

Spanish names:
Indigenous names: Tsakam tsobeem[07]
Used by (1*): Huastec[07]
Used for (2#): Neurological[07]; Psychological[07]
Cognates:
Language contact:

Scindapsus (Araceae)

Spanish names:
Indigenous names: Kuax' Kay'[14]
Used by (1*): Quichean Maya[14]
Used for (3#): Neurological[14]; Psychological[14]; General and Unspecified[14]
Cognates:
Language contact:

Scleria (Cyperaceae)

Spanish names:
Indigenous names: Neiuk[03]; Møøy[04]; T'oyol toom t'unu', kotox toom, tsakam k'iithath toom, tathiim toom[07]; Xoknoon[09]
Used by (4*): Zoque[03]; Mixe[04]; Huastec[07]; Yucatecan Maya[09]
Used for (8#): Digestive[03, 04, 07]; Respiratory[09]; Skin[07]; Pregnancy[03]; Female genital[04]; General and Unspecified[07]
Cognates:
Language contact:

Scoparia **(Plantaginaceae)**

Spanish names: Cilandrillo; Cilantrillo; Escobillo
Indigenous names: Naax Podeey[04]; Kulantr pim[14]
Used by (5*): Zoque[03]; Mixe[04]; Quichean Maya[14, 15]; Western Maya[18]
Used for (9#): Digestive[04, 14, 18]; Neurological[03]; Skin[03, 04, 14]; Pregnancy[15]; Female genital[03]
Cognates:
Language contact:

Scutellaria **(Lamiaceae)**

Spanish names: Orozuz
Indigenous names: Elbeenax ts'phool, waylom ts'ohool, chakam kuch, t'ot ich ch'ohool, chakam thoot[07]; Balsamo xiw[09]
Used by (2*): Huastec[07]; Yucatecan Maya[09]
Used for (4#): Digestive[07, 09]; Cardiovascular[07]; General and Unspecified[07]
Cognates:
Language contact:

Sechium **(Cucurbitaceae)**

Spanish names: Chayote; Espino, chayote; Guisquil; Huisquil
Indigenous names: Awin pa'sun/apit pa'sun/ awit pa'sun/ pa'sun[01]; Apit[02]; Cuy pasun[03]; A'xaa[04]; Choyotesel, spup, malsh tu'kun[06]; Tsiw', chiw[07]; Pak'a Q'ix[12]; Q'ix[13]; Chijch'um[18]; Balagayaappa[21]; Chayote[25]
Used by (11*): Zoque[01, 02, 03]; Mixe[04]; Totonac[06]; Huastec[07]; Quichean Maya[12, 13]; Western Maya[18]; Zapotec[21]; Nahua[25]
Used for (24#): Digestive[01, 04, 12]; Cardiovascular[01, 06, 18]; Musculoskeletal[03]; Psychological[21]; Skin[01, 02, 12, 13]; Endocrine[25]; Urological[01, 02, 03, 06, 07, 25]; Pregnancy[01, 03]; General and Unspecified[01, 03, 21]
Cognates: MZ: awit/apit/a'xaa; Zoq: awit/apit, pasun; Maya: q'ix/chiw/chij; Quich: q'ix;
Language contact:

Securidaca **(Polygalaceae)**

Spanish names: Balsamillo; Tripa de gallo
Indigenous names: Kipats ay[03]; Mamaal tsan, nuuk bichim, umuw tsaah, anuts bichim, , manil pet[07]; Se' ru' k'an tyaj; ch'up k'an tyaj[17]
Used by (4*): Zoque[02, 03]; Huastec[07]; Quichean Maya[17]
Used for (8#): Digestive[03]; Musculoskeletal[07]; Neurological[07]; Respiratory[02]; Skin[03, 07]; General and Unspecified[03]; nd[17]
Cognates:
Language contact:

Sedum **(Crassulaceae)**

Spanish names: Cola de chivo; Siempreviva
Indigenous names: Ujey chij[13]; Guièe-yùzh[23]; Yahualchit[25]
Used by (4*): Totonac[05]; Quichean Maya[13]; Zapotec[23]; Nahua[25]
Used for (6#): Eye[13]; Skin[23, 25]; Female genital[25]; General and Unspecified[05, 25]
Cognates:
Language contact:

Selaginella **(Selaginellaceae)**

Spanish names: Doradilla; Flor de piedra; Hiedra de Piedra; Hoja vishu; Pelo de Hombre primitivo; Siempre viva; Siempre viva, doradilla; Siemprevivo
Indigenous names: Chuklaab ts'ohool, tsakam ts'uh, muthlum ichiich, tsaakuy ts'ohool, ts'ohoolil k'aninmiin, yoxon, mul ichiich[07]; Mooch-tut[09]; Kumatzin Q'os Abäj[12]; Rismal xilik [14]; Guìzh-yùzh[23]
Used by (11*): Zoque[02, 03]; Huastec[07]; Yucatecan Maya[09]; Quichean Maya[12, 14, 16, 17]; Zapotec[23]; Nahua[26, 27]
Used for (22#): Digestive[12]; Neurological[14, 16]; Psychological[16, 23]; Skin[02, 07, 12]; Endocrine[26]; Urological[09, 27]; Pregnancy[02]; Female genital[03, 12]; Male genital[12]; General and Unspecified[03, 07, 12, 16, 23]; nd[17]
Cognates:
Language contact:

***Selenicereus* (Cactaceae)**

Spanish names: Pitaya
Indigenous names: Bohol tsatsa'[07]; Tsaran-ak'[09]
Used by (3*): Zoque[03]; Huastec[07]; Yucatecan Maya[09]
Used for (6#): Neurological[07]; Skin[03, 09]; Pregnancy[07]; Female genital[09]; General and Unspecified[07]
Cognates:
Language contact:

***Semialarium* (Celastraceae)**

Spanish names: Cancerina; Canserina
Indigenous names: Sak-bo'ob. Xooknom[09]
Used by (4*): Zoque[03]; Yucatecan Maya[09]; Quichean Maya[12]; Zapotec[21]
Used for (10#): Digestive[12, 21]; Respiratory[09]; Skin[21]; Urological[03, 12]; Female genital[03, 12, 21]; General and Unspecified[12]
Cognates:
Language contact:

***Senecio* (Asteraceae)**

Spanish names: Azumiate; Chilca; Hierba de borracho
Indigenous names: Ak monhgu tzyay/ ak manhgu tzyay[01]; Meteba'[12]; Chijob[13]; Guìzh-diâg-bûrr[23]; Tzompiltectle[26]; Axochitl[28]
Used by (6*): Zoque[01]; Quichean Maya[12, 13]; Zapotec[23]; Nahua[26, 28]
Used for (21#): Blood[12]; Digestive[12, 13, 26]; Cardiovascular[12]; Musculoskeletal[12, 13]; Neurological[12]; Psychological[12]; Respiratory[12]; Skin[13, 28]; Urological[12]; Pregnancy[13]; Female genital[13, 26]; General and Unspecified[01, 12, 13, 23, 28]
Cognates:
Language contact:

***Senna* (Fabaceae)**

Spanish names: Barajo; Cachinbal; Cerilla; Flor de abejón; Flor de San José; Flor de todos santos; Frijolillo; Frijolillo (arbol); Frijolillo del monte; Hierba de zopilote; Hoja sen; Hormiguera, pico de pájaro, hormiguillo; Palo Santiago; Taratana; Todos los santos amarillo
Indigenous names: Syäk mätzyi[02]; Ku'tzum kuy[02]; Chuch acuy[03]; Uaxiñ[03]; M¥k stogoy[03]; Putx tsay[03]; Putx tsay [03]; K¥ mooya[03]; Pa xuxk[04]; Poop tsina'an[04]; Bichaam ts'ohool, witsaap ch'ohool[07]; Kaxiy ts'ihol[07]; Itsaan an bichaam[07]; Tsuleek' ekwet, bichaam te', xamxam[07]; Loolte'[07]; K'anchik'in-ak'[09]; Mehenbu'ul-xiw[09]; Tu'ha'abin[09]; Saalche', Boxsaal[09]; Kaxlan, Xche Kaxlan[14]; Linaq Q'ehen[14]; Kara'bansi che[16]; Bajero pim[17]; Carabans i che'[17]; Yopo sen[18]; U mul xinich'[18]; Taratana[18]; B'u'r k'opot[19]; Bizandxa'a gueexii[21]; Bizandxa'a[21]; Guixa'a mbisundxi[21]; Guièe-mzhòodz, guìzh-mzhòod, guièe-nguládz, guìzh-nguládz, x-guìzh-nguládz, guièe-sàn-wsé, guièe-yòob-chèn, yàg-guièe-nguĕts[23]; Higiacxihuitl[28]
Used by (17*): Zoque[01, 02, 03]; Mixe[04]; Huastec[07]; Yucatecan Maya[09, 10, 11]; Quichean Maya[14, 15, 16, 17]; Western Maya[18, 19]; Zapotec[21, 23]; Nahua[28]
Used for (86#): Blood[14]; Digestive[02, 03, 07, 09, 14, 18, 21]; Eye[19]; Ear[03]; Cardiovascular[07]; Musculoskeletal[03, 07, 09, 21]; Neurological[03, 07, 18]; Psychological[07, 16]; Respiratory[03, 04, 07, 18, 21]; Skin[01, 03, 07, 09, 11, 14, 18, 21, 23, 28]; Pregnancy[07]; Female genital[02, 03, 14, 15]; General and Unspecified[03, 04, 07, 10, 11, 19, 21, 23, 28]; nd[17]
Cognates:
Language contact:

***Serjania* (Sapindaceae)**

Spanish names: Gulandrina; Tres costilla; Tres lomos
Indigenous names: Tukas Tzayapak [01]; Popo pujkuy poj [02]; Aki tiuts ay[03]; Tsank'ub ts'ohool, wal puchun[07]; Riagshinguish[21]
Used by (5*): Zoque[01, 02, 03]; Huastec[07]; Zapotec[21]
Used for (14#): Digestive[01, 02, 03, 07, 21]; Musculoskeletal[01]; Neurological[03, 07]; Skin[03, 07]; Urological[01]; Female genital[03]; Male genital[02]; General and Unspecified[01]
Cognates:
Language contact: Chimalapa Zoq <> Hua

01-28 refer to the studies described in Table 4.1 * Total number of studies recording this taxon #Total number of use-records

Sesamum (Pedaliaceae)

Spanish names: Ajonjoli
Indigenous names: Talhtzi'nqui'hui, aa'juliim, cu'li'm, zin'qui'hui[06]
Used by (3*): Totonac[06]; Quichean Maya[12]; Zapotec[21]
Used for (6#): Digestive[12]; Skin[12]; Urological[12]; Pregnancy[06]; General and Unspecified[12, 21]
Cognates:
Language contact:

Setaria (Poaceae)

Spanish names:
Indigenous names: Weew ok[07]; Ch'upil Q'ehen[14]
Used by (2*): Huastec[07]; Quichean Maya[14]
Used for (2#): Neurological[07]; Female genital[14]
Cognates:
Language contact:

Sicydium (Cucurbitaceae)

Spanish names:
Indigenous names: Chakmots-ak', Hoykep, Saloli-ak', Cbikimu-ik'[09]; Pak il[11]; Xsantii ajpaq, Santii' Aj Paq'[14]
Used by (3*): Yucatecan Maya[09, 11]; Quichean Maya[14]
Used for (5#): Eye[09]; Cardiovascular[14]; Psychological[11]; Skin[09]; General and Unspecified[11]
Cognates: CoreM: pak;
Language contact:

Sida (Malvaceae)

Spanish names: Escoba; Escobillo; Escobillo Hembra; Escobillo Macho; Malva (chica); Malva (de puerco); Malva blanca; Malva blanca/ amarilla; Malva de cochino; Malva negra; Malvarisco/ malva hembra; Malvavisco
Indigenous names: Kung pet kuy/tung'an petkuy/kan tane)/kan petkuy/ täm petkuy/pat petkuy/paj petkuy[01]; Ta'petkuy[02]; Yoya malva[03]; Poop tukats[04]; Tsap tukaats[04]; Thak thipon[07]; Chichibe[09]; Pasmar xiw[09]; Meseb'äl Q'os Aqom[12]; Mezbel q'ais[12]; Mesb'al[13]; Mesb'eel, Mes'uul[14]; Mesbel[15]; Mes' b'eel[17]; Tzatzal mes[20]; Lexuba'a nagatzii/ gutzii[21]; Màlbàrîscw[23]; Tlanexahuiltzi[26]
Used by (16*): Zoque[01, 02, 03]; Mixe[04]; Huastec[07]; Yucatecan Maya[09]; Quichean Maya[12, 13, 14, 15, 17]; Western Maya[18, 20]; Zapotec[21, 23]; Nahua[26]
Used for (78#): Blood[12]; Digestive[01, 02, 03, 07, 09, 12, 14, 18, 20, 26]; Musculoskeletal[01, 02, 03, 21, 26]; Neurological[02, 04]; Psychological[03]; Respiratory[01, 03, 12, 18, 23]; Skin[01, 02, 03, 04, 07, 09, 12, 13, 14, 21, 26]; Endocrine[02]; Urological[01, 03, 12, 15]; Pregnancy[01, 02, 13, 14, 15, 21, 23]; Female genital[02, 03, 12, 21]; Male genital[02]; General and Unspecified[01, 02, 03, 04, 09, 12, 13, 21, 23, 26]; nd[17]
Cognates: Zoq: petkuy; CoreM: mes; Quich: mesbel;
Language contact:

Sidastrum (Malvaceae)

Spanish names:
Indigenous names:
Used by (1*): Zoque[03]
Used for (1#): Digestive[03]
Cognates:
Language contact:

Sideroxylon (Sapotaceae)

Spanish names: Tempisque
Indigenous names:
Used by (1*): Quichean Maya[12]
Used for (2#): Cardiovascular[12]; General and Unspecified[12]
Cognates:
Language contact:

***Silybum* (Asteraceae)**

Spanish names: Cardo Maria, Cardo Silvestre
Indigenous names:
Used by (1*): Quichean Maya[12]
Used for (7#): Blood[12]; Digestive[12]; Musculoskeletal[12]; Neurological[12]; Psychological[12]; Endocrine[12]; Urological[12]
Cognates:
Language contact:

***Simaba* (Simaroubaceae)**

Spanish names: Cedrón
Indigenous names:
Used by (5*): Zoque[01, 02, 03]; Western Maya[19]; Zapotec[21]
Used for (10#): Digestive[01, 03, 19]; Musculoskeletal[21]; Skin[01, 02, 03, 21]; General and Unspecified[01, 03]
Cognates:
Language contact:

***Simarouba* (Simaroubaceae)**

Spanish names: Aceituno; Negrito
Indigenous names: Passac[10]; Pa'sa'ak'[11]
Used by (3*): Yucatecan Maya[10, 11]; Western Maya[19]
Used for (3#): Digestive[10, 11, 19]
Cognates: Yuc: pasak;
Language contact:

***Simira* (Rubiaceae)**

Spanish names: Nazareno rojo
Indigenous names:
Used by (1*): Zoque[03]
Used for (5#): Blood[03]; Digestive[03]; Musculoskeletal[03]; Skin[03]; Female genital[03]
Cognates:
Language contact:

***Sinapis* (Brassicaceae)**

Spanish names: Mostaza
Indigenous names:
Used by (1*): Zoque[03]
Used for (1#): Psychological[03]
Cognates:
Language contact:

***Sinclairia* (Asteraceae)**

Spanish names:
Indigenous names: Luisa ay[03]
Used by (1*): Zoque[03]
Used for (2#): Respiratory[03]; Skin[03]
Cognates:
Language contact:

***Sinningia* (Gesneriaceae)**

Spanish names:
Indigenous names: Ujts køjk[04]
Used by (1*): Mixe[04]
Used for (2#): Digestive[04]; Pregnancy[04]
Cognates:
Language contact:

***Siparuna* (Siparunaceae)**

Spanish names: Hoja de tigre; Hoja de zopilote, hoje mixe, negra; Hoja de zorro/hoja de zorrillo/ hoja de tigre
Indigenous names: Kun tzyantzyan/kun tzantzan/ ku tzantzan/ wekpa tane[01]; Tzantzan tuj kuy[02]; Tsan tsan ay[03]; Atsømtsi'ixy[04]; Chu' Che'[14]; Roq xa'an, co moj che[16]; Balagamixii[21]
Used by (7*): Zoque[01, 02, 03]; Mixe[04]; Quichean Maya[14, 16]; Zapotec[21]
Used for (29#): Blood[03, 14]; Digestive[03]; Eye[02, 14]; Cardiovascular[01]; Musculoskeletal[01, 02, 03, 14, 21]; Neurological[01, 03, 14, 16]; Psychological[01]; Respiratory[01, 04, 14]; Skin[03, 21]; Pregnancy[01, 03]; Female genital[21]; General and Unspecified[01, 03, 04, 14, 21]
Cognates: MZ: tsan/tsäm; Zoq: tsantsan;
Language contact:

***Smallanthus* (Asteraceae)**

Spanish names: Flor de margarita; Mano de león
Indigenous names: Ko'son[01]; K'ail, p'ilix momol, balam k'in[20]
Used by (3*): Zoque[01]; Western Maya[20]; Nahua[25]
Used for (6#): Digestive[20, 25]; Skin[01, 20]; Female genital[20]; General and Unspecified[01]
Cognates:
Language contact:

***Smilax* (Smilacaceae)**

Spanish names: Axquiote, Guatotole; Bigotes de cozol; Cocolmeca; Cocolmeca (de hoja chica); Cocolmeca (de hoja larga); Diente de chucho, sarzaparilla, bejuco de vida; Diente de perro; Diente de perro/zarzaparilla; Sarsa; Tumba vaquero; Zarzaparilla; Zarzaparilla hembra; Zarzaparilla, barbasco
Indigenous names: Tuwi tätz[01]; Tzujus poj[02]; Mäm ma'si[02]; Mom[03]; Tuutk kopk aats[04]; Ajni mayak[05]; Kantzilil[05]; Can'ci'lil[06]; Weew uut', kwayab uut', uut' ts'aah[07]; Q'ix Q'ul, Qaynäq Chaklaj Aq'om[12]; Salcha'ak Aq'om[12]; Q'ul[12]; K'ul[13]; Q'ix pim[14]; Chub Ixim[15]; Cocolmecate[26]
Used by (13*): Zoque[01, 02, 03]; Mixe[04]; Totonac[05, 06]; Huastec[07]; Quichean Maya[12, 13, 14, 15]; Zapotec[21]; Nahua[26]
Used for (73#): Blood[03, 12, 13, 14, 26]; Digestive[02, 03, 04, 05, 06, 07, 13]; Eye[02]; Cardiovascular[01, 12]; Musculoskeletal[01, 02, 12]; Neurological[02]; Skin[01, 03, 07, 12, 13, 14, 21]; Endocrine[03, 12]; Urological[01, 02, 03, 07, 26]; Pregnancy[03]; Female genital[03, 04, 12]; Male genital[01, 03, 05]; General and Unspecified[01, 07, 12, 15]
Cognates: Zoq: mäm/mom; Tot: kantzilil; Quich: k'ul;
Language contact: Quich <> Nah

***Solandra* (Solanaceae)**

Spanish names:
Indigenous names: Tima' wits[07]
Used by (1*): Huastec[07]
Used for (2#): Eye[07]; General and Unspecified[07]
Cognates:
Language contact:

Solanum (Solanaceae)

Spanish names: Bejuco de iguana; Berenjena; Cajancli, casancli; Cordilliera; Diente de coche, gastabodoc; Gigandilla; Hierba mora; Hierba mora, quelite; Hierba Mora, Quilete, Macuy; Hierba mora, quilete, macuy, quilete de monte; Hierbamora; Hoja de azar/huele de noche; Hoja de balsamo; Jitomate; Lavaplato; Lavaplato blanco; Lavaplato morado/ espina; Lavaplatos blanco; Lavaplatos de espina/ barraco; Macuy, Hierba Amarga, Hierba Mora de Montaña; Merenchena silvestre; Papa; Papera; Pata de cabron; Planta de chuchu; Quelite; Riega platos; San Cayetano; Sosa; Sosa, berenjena; Tabardilla/ tabardillo; Temalabar; Tomate; Tomatillo del Monte; Yerba mora
Indigenous names: Mun Tzäbä[01]; Yomo tzutzi [01]; Täptäp kuy awit/tawis tane/awin täptäp kuy/ täm tujkuy/ awit kuy/täptäjkuy awit/ tätsy koya awit/ täktäk kuy awit[01]; Täptäp kuy awit[01]; Koya[01]; Yä kosyoj/pop matsy kuy/ bola kuy[01]; Paga kuy/ poti ay[02]; Mä'a witöm ay / tzäpä[02]; Txipiñ[03]; Poa nanchiñ[03]; Nuup cuy[03]; Muutsei cuy[03]; Ts¥p¥ [03]; Ts¥n hon kiñi[03]; Chi tiuts[03]; Chirion[04]; Kamatøøts[04]; Palcha[05]; Lacaxat, laca šanat [05]; Muu stu'luut[06]; Paklcha[06]; Wal ts'ok, wal piich, wal piita', ich ch'ohool[07]; Tsakam tsabalte', tsakam wheelom, t'othoy ts'ohool, chakam walul[07]; Muuthuuts', pothots, t'akaab plato[07]; Thak'chook' uut', thak'chook' an teneklaab, ehtiil i muuthuuts'[07]; Itsik' mitsu', ehtiil muuthuuts'[07]; Tuthay[07]; Maayte', eheenchix ts'ohool[07]; Xpahhux, Ukuch kax[09]; Putbalam[09]; P'ak'[09]; Xsikli-much[09]; OkutČ[10]; ČuČu[10]; Arepa Ši'iu[10]; Tôom pa'ap[10]; K'e'y aqom ki'k'[12]; Pak'a Q'ix[12]; Xkoya' Q'os[12]; Majk'uy[12]; Saq wach[13]; Imut'[13]; Tijol q'ix, uwarc'ak[13]; Paja quix', pajal q'ix', tzajal q'ix'[13]; Xk'a Waka'x[14]; Saq' yool[14]; Rax Ik Che[14]; Pajl' Q'ehen, Pajl Q'ix[14]; Macuy[15]; Ik, kejen, sajom te[16]; Yäyäx tomate[18]; Majk'ui[19]; K'ux peul, tujkulum ch'ix[20]; Quequelito[21]; Ledxuxii[21]; Guedxe baladu'u[21]; Chacuaquillo[21]; Quitzii[21]; Bityuš bä'kw[22]; Geč bigin[22]; Geč gusohp[22]; Yàg-pchŭux-yâas, pxât[23]; Yàg-guièdz-zân[23]; Yahuajcuahuitl[24]; Necaxancuamecatl[24]; Jitomate[25]; Tomatillo[25]; Ixiquelite, chichiquelite[26]; Chichiquelite[26]; Atlapa[26]; Kajanki, kaxajqui[28]
Used by (25*): Zoque[01, 02, 03]; Mixe[04]; Totonac[05, 06]; Huastec[07]; Yucatecan Maya[09, 10, 11]; Quichean Maya[12, 13, 14, 15, 16]; Western Maya[18, 19, 20]; Zapotec[21, 22, 23]; Nahua[24, 25, 26, 28]
Used for (234#): Blood[12, 13, 15, 18, 26]; Digestive[01, 02, 03, 06, 07, 12, 13, 14, 18, 20, 21, 22, 23, 26]; Eye[01]; Ear[01, 07, 13]; Cardiovascular[01, 02, 12]; Musculoskeletal[01, 02, 03, 07, 12, 13, 24, 26]; Neurological[01, 03, 04, 06, 07, 12, 13, 16, 18, 22, 25]; Psychological[01, 02, 03, 07, 12, 13, 14, 16, 23, 24]; Respiratory[02, 06, 07, 12, 13, 18, 25]; Skin[01, 02, 03, 04, 05, 06, 07, 09, 10, 12, 13, 14, 19, 21, 22, 23, 25, 26]; Endocrine[01, 02, 03, 06, 12, 24]; Urological[01, 02, 06, 14]; Pregnancy[01, 03, 07, 10, 19, 23, 24, 28]; Female genital[01, 02, 03, 11, 12, 14, 15, 19, 21, 24]; Male genital[01, 03]; General and Unspecified[01, 03, 04, 05, 06, 07, 12, 13, 14, 21, 22, 23, 24, 26]
Cognates: MZ: täts, chi; Zoq: mats/muts kuy, tsäpä, täm; Tot: palcha; CoreM: majkuy, pak; Yuc: ukuch / okuch; Quich: makuy / majkuy; paCa q'ix; Zap: pchux / xux; Nah: kaxa;
Language contact: Zoq > CoreM; Zoq <> Tot & Hua & Yuc; Zoq > Kaqchikel; Tot <> Hua & Nah; Zoq > Yuc; Zap <> Yuc & Tzeltalan; Tot <> Yuc & Quich

Solidago (Asteraceae)

Spanish names: Monta bilis, bara de oro
Indigenous names:
Used by (1*): Quichean Maya[13]
Used for (1#): Digestive[13]
Cognates:
Language contact:

Sonchus (Asteraceae)

Spanish names: Amargón, Lechuguilla, Diente de León; Chicorea; Lechiguilla
Indigenous names: Met kiej[13]; Chikaryo, kulix pimil, tzepen[20]
Used by (4*): Zoque[01]; Quichean Maya[12, 13]; Western Maya[20]
Used for (13#): Blood[12]; Digestive[01, 12, 20]; Musculoskeletal[01]; Neurological[12]; Respiratory[20]; Skin[12, 20]; Urological[01, 13]; Pregnancy[12, 13]
Cognates:
Language contact:

Sorghum (Poaceae)

Spanish names:
Indigenous names: Laab eem[07]
Used by (1*): Huastec[07]
Used for (2#): Digestive[07]; Endocrine[07]
Cognates:
Language contact:

01-28 refer to the studies described in Table 4.1 * Total number of studies recording this taxon #Total number of use-records

Souroubea **(Marcgraviaceae)**

Spanish names:
Indigenous names: Hub'ub'[17]
Used by (1*): Quichean Maya[17]
Used for (1#): nd[17]
Cognates:
Language contact:

Spananthe **(Apiaceae)**

Spanish names: Cañuela
Indigenous names:
Used by (1*): Nahua[26]
Used for (1#): Skin[26]
Cognates:
Language contact:

Spathiphyllum **(Araceae)**

Spanish names:
Indigenous names: Yuk Q'ehen[14]
Used by (2*): Zoque[03]; Quichean Maya[14]
Used for (3#): Musculoskeletal[14]; Female genital[14]; General and Unspecified[03]
Cognates:
Language contact:

Spermacoce **(Rubiaceae)**

Spanish names: Corte Santito blanco; Crucero; Hierba cresta/tecabezón; Paletaria, sanalotodo, hierba del sapo; Riñonina
Indigenous names: Jutut sotyi[03]; Butun sotyi[03]; Wayel ts'ohool, pakaab ch'ohool[07]; Haway[09]; Haway, Haway-k'ak'[09]; Seq' Q'os ixoq'[12]; Tzilij, zlij, tzelej[13]; B'iritaqil Q'ehen, Josq'il Q'ehen[14]; Voton vomol, buluk sit, ve'el buluk sat vomol, k'uxbal buluk sit wamal, vol nich vomol, ni' chitom[20]
Used by (9*): Zoque[01, 03]; Huastec[07]; Yucatecan Maya[09]; Quichean Maya[12, 13, 14, 17]; Western Maya[20]
Used for (24#): Digestive[01, 03, 07, 13, 14, 20]; Musculoskeletal[03]; Respiratory[13, 20]; Skin[03, 09, 13, 20]; Urological[03]; Pregnancy[12]; Female genital[03, 12]; nd[17]
Cognates: Maya: way;
Language contact:

Sphaeralcea **(Malvaceae)**

Spanish names:
Indigenous names: Hierba del negro[25]
Used by (1*): Nahua[25]
Used for (2#): Musculoskeletal[25]; Skin[25]
Cognates:
Language contact:

Sphagneticola **(Asteraceae)**

Spanish names:
Indigenous names: Samatil Q'ehen[14]
Used by (1*): Quichean Maya[14]
Used for (2#): Cardiovascular[14]; Psychological[14]
Cognates:
Language contact:

***Spigelia* (Loganiaceae)**

Spanish names: Lombricera; Lombrizero
Indigenous names:
Used by (2*): Zoque[03]; Yucatecan Maya[09]
Used for (2#): Digestive[03, 09]
Cognates:
Language contact:

***Spiranthes* (Orchidaceae)**

Spanish names:
Indigenous names: Chiwohk'aak'[09]
Used by (1*): Yucatecan Maya[09]
Used for (1#): Skin[09]
Cognates:
Language contact:

***Spondias* (Anacardiaceae)**

Spanish names: Ciruela; Jobo; Jobo, jocote; Jocote; Jocote agrio; Jocote clavo/macho/agrio/acido/cimarrón; Jovo cimarrón
Indigenous names: Katzsu witze[01]; Tuni[02]; Jumu[02]; Pitx cuy[03]; Tun[04]; Skatin[05]; Teen[07]; K'inim[07]; Abal[09]; Kening[10]; Abän[18]; Ajpoc'[18]; Paré[19]; Biadxi[21]; Biadxiu'u[21]
Used by (14*): Zoque[01, 02, 03]; Mixe[04]; Totonac[05]; Huastec[07]; Yucatecan Maya[09, 10, 11]; Quichean Maya[12]; Western Maya[18, 19]; Zapotec[21]; Nahua[26]
Used for (36#): Digestive[02, 03, 04, 05, 07, 12, 18]; Musculoskeletal[12]; Psychological[12]; Respiratory[03, 21, 26]; Skin[01, 02, 03, 07, 11, 12, 18, 21]; Female genital[10, 21]; Male genital[02]; General and Unspecified[02, 03, 07, 09, 12, 18, 19, 21]
Cognates: MZ: tun; Zoq: witz / pitx;
Language contact: MZ > Tot & Hua; Hua <> Yuc; Yuc <> Tabsco Chontal

***Sporobolus* (Poaceae)**

Spanish names:
Indigenous names: Zacate[26]
Used by (1*): Nahua[26]
Used for (1#): Pregnancy[26]
Cognates:
Language contact:

***Stachys* (Lamiaceae)**

Spanish names: Hierba de quemadura; Muiña
Indigenous names: Guìzh-zhwǐn[23]; Tlacchinoxihuitl[26]
Used by (2*): Zapotec[23]; Nahua[26]
Used for (2#): Psychological[23]; Skin[26]
Cognates:
Language contact: Zap <> Nah

***Stachytarpheta* (Verbenaceae)**

Spanish names: Cola de alacrán/verbena embra; Cola de Armadillo, Armado; Verbena; Vervena
Indigenous names: Kaku'e tuts [01]; Chi tiuts[03]; Pacer ujts[04]; Iben-xiw[09]; Xye' ajwech [14]; Xtye aj pak[16]; Tye aj pak[17]
Used by (10*): Zoque[01, 03]; Mixe[04]; Yucatecan Maya[09, 11]; Quichean Maya[14, 16, 17]; Western Maya[18]; Zapotec[21]
Used for (28#): Digestive[01, 04, 14, 18, 21]; Eye[14]; Cardiovascular[01]; Musculoskeletal[09]; Neurological[01, 03, 11, 16, 18]; Skin[01, 03, 14]; Urological[03, 11, 14]; Female genital[03, 09, 14]; Male genital[18]; General and Unspecified[11, 14, 18, 21]; nd[17]
Cognates: Quich: ye aj ;
Language contact:

01-28 refer to the studies described in Table 4.1   * Total number of studies recording this taxon   #Total number of use-records

***Stegnosperma* (Stegnospermataceae)**

Spanish names: Niño que no se puede levantar
Indigenous names:
Used by (1*): Zapotec[21]
Used for (2#): Musculoskeletal[21]; Neurological[21]
Cognates:
Language contact:

***Stellaria* (Caryophyllaceae)**

Spanish names: Cadillo ovalado; Hierba de rosillo; Hoja de azar; Trebolillo rojo
Indigenous names: Tza'a tzoy[01]; Thuuyuu'il t'eel, ehtiil utek'[07]; Keq Lotz Aq'om[12]; Ahuaxmama[26]
Used by (5*): Zoque[01, 03]; Huastec[07]; Quichean Maya[12]; Nahua[26]
Used for (12#): Eye[12]; Cardiovascular[07, 12]; Musculoskeletal[12]; Neurological[12]; Psychological[12]; Skin[12]; Urological[03]; Female genital[03]; General and Unspecified[01, 12, 26]
Cognates:
Language contact:

***Stemodia* (Plantaginaceae)**

Spanish names:
Indigenous names: Tlaquexaniltzi[26]
Used by (1*): Nahua[26]
Used for (1#): Skin[26]
Cognates:
Language contact:

***Stenocereus* (Cactaceae)**

Spanish names: Cactus
Indigenous names: Tzap kom[01]
Used by (1*): Zoque[01]
Used for (1#): Skin[01]
Cognates:
Language contact:

***Stevia* (Asteraceae)**

Spanish names: Cabeza de hongo; Hierba blanca de muerto; Ocaotera; Pericón blanco; Yerba de borracho
Indigenous names: Winyo wajo' jop ay[02]; Sak'a eya'[12]; Ujlom ikox[13]; Ch'aj te' nichim, ch'aal sakil nich wamal, ch'aal wamal, ch'a momol, nak' obal us[20]; Tzajal nak'obal us[20]; Gi lahk[22]
Used by (6*): Zoque[01, 02]; Quichean Maya[12, 13]; Western Maya[20]; Zapotec[22]
Used for (16#): Blood[12]; Digestive[12, 13, 20, 22]; Musculoskeletal[20]; Neurological[12]; Skin[01, 02, 12, 20]; Endocrine[12]; General and Unspecified[12]
Cognates:
Language contact:

***Stigmaphyllon* (Malpighiaceae)**

Spanish names:
Indigenous names: Samñi tsay[03]
Used by (1*): Zoque[03]
Used for (3#): Digestive[03]; Female genital[03]
Cognates:
Language contact:

Stillingia **(Euphorbiaceae)**

Spanish names: Hierba mala
Indigenous names: Puj Q'ayis[12]; Etzel k'ayes[13]
Used by (2*): Quichean Maya[12, 13]
Used for (3#): Skin[12, 13]; General and Unspecified[12]
Cognates: Quich: k'ayis;
Language contact:

Stizophyllum **(Bignoniaceae)**

Spanish names:
Indigenous names: Xa'bach[09]
Used by (1*): Yucatecan Maya[09]
Used for (1#): Respiratory[09]
Cognates:
Language contact:

Stromanthe **(Marantaceae)**

Spanish names: Hoja de piedra
Indigenous names: Tza ay[01]; Xmox So'sol[14]
Used by (2*): Zoque[01]; Quichean Maya[14]
Used for (3#): Digestive[01]; Urological[01]; Female genital[14]
Cognates:
Language contact:

Struthanthus **(Loranthaceae)**

Spanish names: Caballera; Cabellera; Mata palo; Muérdago; Seca palo
Indigenous names: Eju katzakatza[01]; Cuyñukxi[03]; Ok'lom te'[07]; Mak'tahila[08]; Tzara' Aq'om[12]; Tzara' [12]; Raximai Q'en, Mayil Q'en[14]; Tak'ä zte'[18]
Used by (7*): Zoque[01, 03]; Huastec[07, 08]; Quichean Maya[12, 14]; Western Maya[18]
Used for (26#): Blood[12]; Digestive[01, 07, 12]; Cardiovascular[07, 12]; Musculoskeletal[12]; Neurological[07, 12]; Psychological[07, 12]; Skin[03, 08, 12, 14, 18]; Endocrine[08]; Pregnancy[18]; Female genital[01]; General and Unspecified[12, 14]
Cognates:
Language contact:

Strychnos **(Loganiaceae)**

Spanish names: Chicoloro
Indigenous names: Curux k'ix[17]
Used by (2*): Yucatecan Maya[10]; Quichean Maya[17]
Used for (3#): Digestive[10]; Skin[10]; nd[17]
Cognates:
Language contact:

Stylosanthes **(Fabaceae)**

Spanish names: Hoja de azar
Indigenous names: Tza'a tzoy[01]
Used by (2*): Zoque[01, 03]
Used for (2#): Skin[03]; General and Unspecified[01]
Cognates:
Language contact:

***Stylotrichium* (Asteraceae)**

Spanish names: Hierba de dulce, sacate dulce
Indigenous names: Loc'ab[13]
Used by (1*): Quichean Maya[13]
Used for (1#): Skin[13]
Cognates:
Language contact:

***Swietenia* (Meliaceae)**

Spanish names: Caoba
Indigenous names: Tzapats kuy[02]; Gueleyexi'i[21]
Used by (4*): Zoque[01, 02, 03]; Zapotec[21]
Used for (11#): Digestive[03, 21]; Musculoskeletal[03]; Skin[21]; Endocrine[01, 02]; Urological[01]; Pregnancy[21]; Female genital[03]; General and Unspecified[01, 21]
Cognates:
Language contact:

***Symphyotrichum* (Asteraceae)**

Spanish names: Pericon de agua; Tomillo silvestre
Indigenous names:
Used by (2*): Zoque[01, 03]
Used for (2#): General and Unspecified[01, 03]
Cognates:
Language contact:

***Syngonium* (Araceae)**

Spanish names: Mano de Diablo ; Palma de Diablo; Pegapega; Platano del monte; Teleconte
Indigenous names: Mu' katzu/ Mä'k katzu[02]; Pa-uk tiinxy[04]; Kwaath[07]; Ruq' Maus aj' Winq[14]; Ruj i ruk'aj tza, ruk maus[16]; Buduaj gueexii[21]
Used by (7*): Zoque[02]; Mixe[04]; Huastec[07]; Yucatecan Maya[11]; Quichean Maya[14, 16]; Zapotec[21]
Used for (12#): Digestive[02, 07]; Musculoskeletal[11]; Neurological[14, 16]; Psychological[16]; Skin[02, 04, 14]; General and Unspecified[14, 21]
Cognates: Quich: ruk maus;
Language contact:

***Syzygium* (Myrtaceae)**

Spanish names: Clavo; Clavo de olor; Clavo de olor/ clavo de comida; Pomarosa
Indigenous names: Cuonagueleraajeu'u[21]
Used by (8*): Zoque[01, 02, 03]; Totonac[06]; Quichean Maya[13]; Zapotec[21, 22, 23]
Used for (13#): Digestive[01, 06]; Ear[01]; Musculoskeletal[21]; Neurological[02]; Psychological[03]; Respiratory[01, 03, 21]; Skin[23]; Pregnancy[13]; General and Unspecified[21, 22]
Cognates:
Language contact:

***Tabebuia* (Bignoniaceae)**

Spanish names: Macuiliz, macuiliz amarillo; Makulis / Matiliguate ; Maquilishuat/maquilisguate; Roble
Indigenous names: Tzan kuy [01]; Emkuioxk[04]; K'uul, wakats wich[07]; Makulis[14]; Känkän aj maculis[18]
Used by (7*): Zoque[01, 02, 03]; Mixe[04]; Huastec[07]; Quichean Maya[14]; Western Maya[18]
Used for (21#): Digestive[02, 07, 14, 18]; Musculoskeletal[01, 03]; Neurological[18]; Skin[01, 02, 03, 04, 07, 18]; Endocrine[18]; Pregnancy[03]; Female genital[03, 07, 14]; General and Unspecified[07, 14, 18]
Cognates: CoreM: makulis;
Language contact:

***Tabernaemontana* (Apocynaceae)**

Spanish names: Coyol de burro; Huevo caballo; Huevo de venado
Indigenous names: Burro puj[02]; Na'a cuy[03]; No'og waaky[04]; T'abat'[07]; Uts'upek'[09]; Chac le king[10]; Mbiigu' del monte[21]
Used by (7*): Zoque[02, 03]; Mixe[04]; Huastec[07]; Yucatecan Maya[09, 10]; Zapotec[21]
Used for (11#): Digestive[03]; Neurological[03, 04, 07]; Respiratory[21]; Skin[02, 03, 07, 09, 10, 21]
Cognates: MZ: na'a/no'o;
Language contact:

***Tagetes* (Asteraceae)**

Spanish names: Anicillo, anís de monte; Anís; Anis de chucho/ monte / del campo; Anis de estrella, Melicón; Anís del camo; Anis del monte; Anisillo; Anisillo/anis/anis de estrella; Cempasúchil; Cempasuchil, flor de muerto; Cempasúchitl; Cempazúchitl; Cempoalxótchitl; Flor de anís; Flor de Muerte; Flor de Muerto; Flor de muerto, tixcoque; Flor de perico; Flor del alma, flor de muerto, tsampoala, cempasúchil, flor de difunto/ sempoala; Hierba anis; Pericón; Pericón amarillo ; Pericón, hierba de anís, hierba de San Juan, hierba de Santa Maria; Sempolsuchet, togoles; Todos santos; Xempasochil; Zampozuche
Indigenous names: Postin jäyä/putsy jäyä/putzin jäyä[01]; Estrella pok[01]; Tzitzimuk[01]; Anima jäyä [01]; Tasi jäyä[02]; Kotzok ay [02]; Tsuts mooya[03]; Pikøn'ak pøh[04]; Anis ujts[04]; Kaltpu'xa'm[05]; Santoorom wits, k'uchuth wits, k'ochith wits, kaxiy wits, ts'a'il wits, pamta wich[07]; Ohoom[07]; K'uchid huitz[08]; Tempula[09]; Stupuh amarillo[10]; Ix tupuj[11]; Eva' / Eya'[12]; Anix tzi'[13]; Illa', iya'[13]; Prutz', pretz', ukutz'ij kaminaq[13]; Tu'z, Tutz, Tutz' Tatz'un Kamenaq[14]; Yop'aj melicon[18]; Tiskok[18]; Sampwer[19]; Mantzaniya ch'o, kulentu jos, inajo antíwo, injo wamal, tzitz, tzitz olol[20]; Tzitz wamal, tzitz uch, sansiwre wamal, k'anal nich wamal, pimento wamal[20]; Yama chauk[20]; Guibigua[21]; Guiahuajsa'ac[21]; Gi bigu štil, gi togol[22]; Anís gihš[22]; Guièe-cŏb-mzhĭg, guièe-cŏb-yâg[23]; Guìzh-nìzh, guièe-nìzh[23]; Guièe-dzùu, guièe-cŏb-dzùu[23]; Sempoalxochitl[24]; Cempasúchitl[25]; Cempoalxochitl[26]; Cempoalxóchitl[28]; Yauhtli[28]
Used by (24*): Zoque[01, 02, 03]; Mixe[04]; Totonac[05, 06]; Huastec[07, 08]; Yucatecan Maya[09, 10, 11]; Quichean Maya[12, 13, 14]; Western Maya[18, 19, 20]; Zapotec[21, 22, 23]; Nahua[24, 25, 26, 28]
Used for (160#): Blood[12]; Digestive[01, 02, 03, 04, 05, 06, 07, 08, 11, 12, 13, 14, 18, 19, 20, 21, 22, 23, 25, 26, 28]; Eye[19]; Ear[01, 19, 23]; Cardiovascular[01, 08]; Musculoskeletal[02, 03, 07, 08, 11, 12, 18, 20, 26]; Neurological[07, 12, 14, 18, 28]; Psychological[01, 03, 05, 12, 13, 19, 20]; Respiratory[01, 03, 07, 08, 12, 13, 14, 18, 20, 21, 23, 25, 28]; Skin[01, 03, 05, 07, 08, 12, 18, 21, 25, 28]; Endocrine[07]; Urological[01, 12, 13]; Pregnancy[02, 03, 07, 13, 19, 21, 22, 23, 24, 26]; Female genital[01, 02, 03, 05, 09, 12, 13, 19, 21]; General and Unspecified[01, 02, 03, 04, 05, 07, 08, 10, 12, 13, 14, 18, 19, 20, 21, 28]
Cognates: Zoq: Cuts mooya/jäyä; Hua: k'ochith wits; Yuc: tupu; Quich: eya/iya, utz; Zap: gibigu; Nah: cempoalxochitl;
Language contact: Zoq <> Tot & Hua & Yuc & Quich; WesM > Chiapas Zoq (tzitz)

***Talinum* (Talinaceae)**

Spanish names: Hierba del riñon
Indigenous names: Masan ay[03]; Thak akw'aal, t'uhub akw'aal[07]; Pitsits wal pktha', tsakam ix thuyum[07]; Ncuàan-dzéb-ròo[23]
Used by (4*): Zoque[01, 03]; Huastec[07]; Zapotec[23]
Used for (10#): Digestive[03, 07]; Musculoskeletal[03]; Skin[03, 07]; Urological[01, 07]; Female genital[01]; General and Unspecified[07, 23]
Cognates:
Language contact:

***Tamarindus* (Fabaceae)**

Spanish names: Tamarindo
Indigenous names: Tamariindu, tam malin[07]; Gubshnii'[21]
Used by (10*): Zoque[02, 03]; Mixe[04]; Huastec[07]; Yucatecan Maya[09]; Quichean Maya[12]; Western Maya[18, 19]; Zapotec[21, 23]
Used for (26#): Digestive[02, 03, 09, 12, 18, 23]; Musculoskeletal[02]; Neurological[02]; Psychological[07, 21]; Respiratory[02, 03]; Skin[02, 18]; Endocrine[02, 12]; Urological[03, 18]; General and Unspecified[02, 03, 04, 07, 12, 18, 19, 21]
Cognates:
Language contact:

***Tamonea* (Verbenaceae)**

Spanish names:
Indigenous names: Ook' t'ithith[07]
Used by (1*): Huastec[07]
Used for (2#): Digestive[07]; Urological[07]
Cognates:
Language contact:

01-28 refer to the studies described in Table 4.1 * Total number of studies recording this taxon #Total number of use-records

***Tanacetum* (Asteraceae)**

Spanish names: Altamisa; Altamisa, artemisa, margarita; Flor de Santa Maria; Hierba de Santa Maria; Hierba santa maria; Hierbasanta; Manzanilla amarga, Santa María; Santa María; Santa Maria, manzanilla amarga; Santamaría
Indigenous names: Artamiza/artamisy/altamiza[01]; Santa Maria aay[04]; K'anim mim María ts'ojol[08]; Altamix[12]; Altamix, altimix, altamixá[13]; Guixnash[21]; Manzaniy la[22]; Guièe-sàntàmàrǐ, guièe-sàntàmàrǐ-nquǐts[23]; Caltemesha[28]
Used by (13*): Zoque[01, 02]; Mixe[04]; Huastec[08]; Quichean Maya[12, 13]; Zapotec[21, 22, 23]; Nahua[25, 26, 27, 28]
Used for (53#): Blood[12]; Digestive[01, 02, 04, 08, 12, 13, 21, 22, 23, 25, 26, 27]; Ear[04, 23]; Cardiovascular[23]; Musculoskeletal[01, 12, 13, 23]; Neurological[08, 12, 13]; Psychological[13, 22, 27]; Respiratory[12, 23]; Skin[12]; Urological[02, 12, 13]; Pregnancy[01, 02, 12, 13, 21, 23, 25, 27]; Female genital[01, 02, 04, 12, 13, 23]; General and Unspecified[01, 08, 12, 13, 22, 25, 28]
Cognates:
Language contact:

***Tanaecium* (Bignoniaceae)**

Spanish names:
Indigenous names: Pobatsay[03]; Bak chiwoh[09]; Ški'iŠ[10]
Used by (3*): Zoque[03]; Yucatecan Maya[09, 10]
Used for (4#): Respiratory[09]; Skin[03, 10]; Female genital[03]
Cognates:
Language contact:

***Tapirira* (Anacardiaceae)**

Spanish names: Ocholillo
Indigenous names: Ocholi nuumpi[03]
Used by (1*): Zoque[03]
Used for (2#): Musculoskeletal[03]; Psychological[03]
Cognates:
Language contact:

***Taraxacum* (Asteraceae)**

Spanish names: Amargón, Lechuguilla, Diente de León, Lechuga criollo; Diente de león; Diente de león, amargón, scorzonera amarga; Diente de león, lechogilla
Indigenous names: Q'uqun Q'än Q'os[12]; Kur kur[13]
Used by (5*): Zoque[01]; Quichean Maya[12, 13]; Nahua[25, 26]
Used for (17#): Blood[13]; Digestive[01, 12, 13]; Cardiovascular[25]; Psychological[12]; Respiratory[13, 25]; Endocrine[12, 26]; Urological[01, 12, 13, 25, 26]; Pregnancy[13]; General and Unspecified[12]
Cognates:
Language contact:

***Taxodium* (Cupressaceae)**

Spanish names: Sabino, ahuehuete
Indigenous names: Chuuche'[07]; Yàg-guìzdòo, yàg-guìstòo[23]
Used by (2*): Huastec[07]; Zapotec[23]
Used for (3#): Skin[23]; Pregnancy[07]; Female genital[07]
Cognates:
Language contact:

***Tecoma* (Bignoniaceae)**

Spanish names: Argel; Flor amarilla, chinche; Hoja de San Pedro, tronadora; Timboque; Trobadora, yerba de empacho; Tronadora; Tronadora, flor de arco
Indigenous names: Kan yoks[01]; Totzkä jäyä[02]; Puutx mooya[03]; San pedro wits[07]; K'anlol[09]; Timbuk'[13]; Ch'aj te[19]; Dze'ing[21]; Guièe-tùts, guìzh-tùts, guìzh-guièe-tùts, yàg-guièe-tùts, yàg-nlibâd-tùts[23]; Nixtamalxochitl[28]
Used by (14*): Zoque[01, 02, 03]; Mixe[04]; Huastec[07]; Yucatecan Maya[09]; Quichean Maya[12, 13]; Western Maya[18, 19]; Zapotec[21, 22, 23]; Nahua[28]
Used for (36#): Digestive[02, 03, 21, 22, 28]; Cardiovascular[12]; Musculoskeletal[01, 02, 19, 21]; Neurological[12, 19]; Respiratory[02, 03, 19, 23]; Skin[01, 19]; Endocrine[01, 02, 03, 04, 07, 09, 12, 13, 18, 28]; Urological[01]; Pregnancy[04]; Female genital[21]; General and Unspecified[01, 03, 04, 19, 21]
Cognates:
Language contact: Chimalapa Zoq <> Zap; Yuc > Chiapas Zoq

***Tectaria* (Tectariaceae)**

Spanish names: Hierba del monte; Lengua de ciervo; Pesmita; Pijul; Siempreviva
Indigenous names: Weew koxol, akan hooh, k'ubak k'aninmiin, akan tspk, pok' ts'ohool, pux lat'em, lek'aab torro[07]; Kubak ajatik[08]; Roq' Chi'wan[14]; Matlaxcalxihuitl[24]
Used by (7*): Zoque[03]; Totonac[05]; Huastec[07, 08]; Quichean Maya[14]; Nahua[24, 25]
Used for (13#): Blood[07]; Digestive[07]; Cardiovascular[07]; Neurological[07, 14]; Psychological[03]; Skin[05, 08]; Endocrine[08]; Pregnancy[07, 24]; General and Unspecified[07, 25]
Cognates: Hua: kubak;
Language contact:

***Telanthophora* (Asteraceae)**

Spanish names: Mano de León
Indigenous names: Nag ay[03]
Used by (2*): Zoque[03]; Quichean Maya[12]
Used for (5#): Psychological[12]; Skin[03]; Urological[12]; Pregnancy[03]; General and Unspecified[12]
Cognates:
Language contact:

***Tephrosia* (Fabaceae)**

Spanish names:
Indigenous names: Mokoy[03]; Ch'alam[17]
Used by (2*): Zoque[03]; Quichean Maya[17]
Used for (3#): Skin[03]; nd[17]
Cognates:
Language contact:

***Terminalia* (Combretaceae)**

Spanish names: Almendra; Almendro
Indigenous names: Bek almendra[18]
Used by (7*): Zoque[01, 02, 03]; Yucatecan Maya[09]; Western Maya[18, 19]; Zapotec[21]
Used for (21#): Digestive[01, 02, 03, 09, 18, 21]; Neurological[18]; Respiratory[01, 19]; Skin[01, 02, 18]; Endocrine[01, 02, 03]; Urological[01, 09]; Pregnancy[03]; Female genital[03]; General and Unspecified[01, 21]
Cognates:
Language contact:

***Ternstroemia* (Pentaphylacaceae)**

Spanish names: Flor de tila; Té de tila; Trompito/flor de tila
Indigenous names: M¥a nanchiñ[03]
Used by (4*): Zoque[01, 02, 03]; Zapotec[21]
Used for (14#): Digestive[01, 03, 21]; Cardiovascular[01, 03, 21]; Psychological[01, 02, 03, 21]; Pregnancy[03]; General and Unspecified[01]
Cognates:
Language contact:

Tetrachyron **(Asteraceae)**

Spanish names: Flor de enjambre
Indigenous names: Cerreroxochitl[26]
Used by (1*): Nahua[26]
Used for (1#): Musculoskeletal[26]
Cognates:
Language contact:

Tetramerium **(Acanthaceae)**

Spanish names: Hoja de vergüenza
Indigenous names: J¥xi ay[03]
Used by (1*): Zoque[03]
Used for (1#): Psychological[03]
Cognates:
Language contact:

Teucrium **(Lamiaceae)**

Spanish names:
Indigenous names: Tihtsan kw'eet, weew ethem, t'othoy ts'ohool, thak pux, thak ch'a'ik ch'ohool[07]
Used by (1*): Huastec[07]
Used for (4#): Blood[07]; Cardiovascular[07]; Skin[07]; General and Unspecified[07]
Cognates:
Language contact:

Thalictrum **(Ranunculaceae)**

Spanish names: Flor de San Antonio; Hoja de azar
Indigenous names: Guìzh-mèt, guìzh-crùz-nguǎts, guìzh-tûb, guièer-ngüèts, ncuàan-dzéb-ròo[23]
Used by (3*): Zoque[01]; Quichean Maya[13]; Zapotec[23]
Used for (4#): Neurological[13]; General and Unspecified[01, 13, 23]
Cognates:
Language contact:

Thelypteris **(Thelypteridaceae)**

Spanish names: Colandrillo; Helecho
Indigenous names:
Used by (3*): Western Maya[18]; Zapotec[21]; Nahua[24]
Used for (3#): Respiratory[18]; Urological[21]; Pregnancy[24]
Cognates:
Language contact:

Theobroma **(Malvaceae)**

Spanish names: Cacao
Indigenous names: Kakawa[01]; Kakawa[02]; Caco[13]; Cacao, X'aq Cacao[14]; Cäcäw[18]; Yàg-bzèy[23]
Used by (9*): Zoque[01, 02]; Quichean Maya[12, 13, 14]; Western Maya[18]; Zapotec[21, 22, 23]
Used for (13#): Blood[12]; Digestive[01, 18]; Skin[18]; Endocrine[12]; Pregnancy[01, 02, 13, 23]; General and Unspecified[12, 14, 21, 22]
Cognates: Zoq: kakawa; CoreM: kakV;
Language contact: Zoq > Mesoamerican

***Thevetia* (Apocynaceae)**

Spanish names: Corcocho; Coyol de burro/coyol de gato; Huevo de gato
Indigenous names: Yoyak puj [01]; Puti kuy, burro puj, jatiks kumku[02]; Cha'lawaka, M¥aaktsa[03]; Atsømtu'ty[04]; Ch'ich'iyan, Naq'irit i' mis[14]; Ch'ich i jak[17]; Yoyotle[26]
Used by (7*): Zoque[01, 02, 03]; Mixe[04]; Quichean Maya[14, 17]; Nahua[26]
Used for (12#): Digestive[14]; Musculoskeletal[03]; Neurological[04]; Skin[01, 02, 03, 14]; Pregnancy[03]; Female genital[03]; General and Unspecified[14, 26]; nd[17]
Cognates: Zoq: puj; Quich: chichi yan/jak;
Language contact: Nah > Chiapas Zoq

***Thunbergia* (Acanthaceae)**

Spanish names: Hierba del espanto; Tumbergia
Indigenous names: Makti mooya[03]; Yommooya[03]; Paxtama'tahua, pastak'natahuan[05]; Tsaayleel ts'ohool[07]; Ik' ts'ohool[07]; Rukama'l B'ochil Kotzij[12]
Used by (4*): Zoque[03]; Totonac[05]; Huastec[07]; Quichean Maya[12]
Used for (13#): Digestive[03]; Ear[03]; Cardiovascular[12]; Musculoskeletal[12]; Neurological[07]; Psychological[12]; Skin[03, 12]; General and Unspecified[03, 05, 12]
Cognates:
Language contact: Highland Popoluca <> Tot

***Thymus* (Lamiaceae)**

Spanish names: Tomillo; Toronjil
Indigenous names:
Used by (5*): Zoque[01]; Totonac[06]; Quichean Maya[12, 13]; Western Maya[19]
Used for (15#): Digestive[06, 12]; Musculoskeletal[12]; Neurological[12]; Psychological[12]; Respiratory[01, 12, 13, 19]; Urological[12]; Pregnancy[12]; Female genital[12]; General and Unspecified[01, 12]
Cognates:
Language contact:

***Tibouchina* (Melastomataceae)**

Spanish names: Tesuatillo
Indigenous names: Cacaloxihuitle[26]
Used by (2*): Zoque[03]; Nahua[26]
Used for (4#): Digestive[26]; Eye[03]; Respiratory[26]; Skin[03]
Cognates:
Language contact:

***Tilia* (Malvaceae)**

Spanish names: Flor de tila; Tila; Tilo
Indigenous names: Guiède-tîl[23]
Used by (4*): Huastec[08]; Quichean Maya[12]; Zapotec[23]; Nahua[25]
Used for (17#): Blood[12]; Digestive[12, 23, 25]; Eye[08]; Cardiovascular[08, 12, 25]; Musculoskeletal[12]; Neurological[12]; Psychological[08, 12, 25]; Urological[12]; Female genital[12]; General and Unspecified[12]
Cognates:
Language contact:

***Tillandsia* (Bromeliaceae)**

Spanish names: Paxte, pashtio; Pie de Gallo
Indigenous names: Tsakam k'ok'om[07]; K'uthay[07]; Ch'u[09]; Q'ux[13]; Roq Aj Tzo'[14]; Cuamimisi[24]
Used by (5*): Huastec[07]; Yucatecan Maya[09]; Quichean Maya[13, 14]; Nahua[24]
Used for (15#): Musculoskeletal[13]; Neurological[07]; Respiratory[07]; Skin[07, 14]; Pregnancy[07, 09]; Female genital[09, 24]; General and Unspecified[07, 13]
Cognates: Maya: k'u/ch'u;
Language contact:

01-28 refer to the studies described in Table 4.1 * Total number of studies recording this taxon #Total number of use-records

Tinantia **(Commelinaceae)**

Spanish names: Pata de pollo, pata de gallo; Siemprevive blanca
Indigenous names: Tzui[01]; Quix'tac[06]
Used by (2*): Zoque[01]; Totonac[06]
Used for (7#): Digestive[06]; Psychological[01]; Skin[01, 06]; Urological[06]; General and Unspecified[01, 06]
Cognates:
Language contact:

Tithonia **(Asteraceae)**

Spanish names: Amargoso, Arnica; Arnica; Arnica/Girasol; Gigantón, girasol; Girayol silvestre; Mirasol
Indigenous names: Tapungäsy jäyä/tapkuy ay/tat kuy/tan tzitzi/tab kuy/tam tzitzi[01]; Tam tzyi/ tam tzyitz/ tam ay[02]; Tam chich[03]; Nø tammtsy[04]; Chaksu'um[09]; S'um Aq'om[12]; Kaj Tajiq'os[12]; Ch'aj[18]; Ch'ajkil, neek ch'a te', k'ail, p'ilix[20]; Ru'ulá[21]; Bârr-dòo-lă[23]; Bnîil-zhĭil, guièe-bnîil-zhĭil, guìzh-bnîil-zhĭil, guièe-bnîil-yòx, blàg-bnîil, guièe-chòg-zhĭil[23]; Acahuali[28]
Used by (12*): Zoque[01, 02, 03]; Mixe[04]; Yucatecan Maya[09]; Quichean Maya[12]; Western Maya[18, 20]; Zapotec[21, 23]; Nahua[26, 28]
Used for (67#): Blood[02, 12]; Digestive[01, 02, 03, 12, 18, 20, 21, 23, 28]; Eye[02]; Cardiovascular[02, 12, 23]; Musculoskeletal[01, 02, 03, 04, 09, 12, 21, 23, 28]; Neurological[18]; Psychological[02, 23]; Respiratory[01, 02, 03, 21]; Skin[01, 02, 03, 04, 12, 18, 20, 21, 23, 26]; Endocrine[01, 02, 03]; Urological[02]; Pregnancy[01, 02, 03, 21]; Female genital[01, 02, 03, 18, 21]; Male genital[02]; General and Unspecified[01, 02, 03, 04, 12, 18, 20, 21]
Cognates: MZ: Tam chi; Zoq: tam chitz; CoreM: s'um, chaj/kaj;
Language contact: MZ > Zap

Tonduzia **(Apocynaceae)**

Spanish names:
Indigenous names: Akab' K'elem[14]
Used by (1*): Quichean Maya[14]
Used for (3#): Digestive[14]; Skin[14]; General and Unspecified[14]
Cognates:
Language contact:

Topobea **(Melastomataceae)**

Spanish names:
Indigenous names: Oxlaju Ch'ajom, Kaq'i Ch'ajom, Yot' eq [14]
Used by (1*): Quichean Maya[14]
Used for (1#): Digestive[14]
Cognates:
Language contact:

Tournefortia **(Boraginaceae)**

Spanish names: Hoja de aire; Hoja de cancer; Yerba del cancer
Indigenous names: Mach'much[07]; Waylom te'[07]; Xulk'ini, Sal[09]; Kann'bolie kejen[16]; Biajtu mshtajala[21]; Blàg-chòg, guìzh-blàg-chòg, guìzh-chòg, guìzh-cânzr[23]
Used by (8*): Zoque[01]; Mixe[04]; Huastec[07]; Yucatecan Maya[09]; Quichean Maya[16]; Zapotec[21, 22, 23]
Used for (16#): Digestive[21]; Eye[21]; Musculoskeletal[01, 21]; Neurological[01, 16]; Respiratory[23]; Skin[04, 07, 09, 21, 22, 23]; Female genital[21]; General and Unspecified[07]
Cognates:
Language contact:

Toxicodendron **(Anacardiaceae)**

Spanish names:
Indigenous names: Ja'yi[01]
Used by (1*): Zoque[01]
Used for (1#): Skin[01]
Cognates:
Language contact:

01-28 refer to the studies described in Table 4.1 * Total number of studies recording this taxon #Total number of use-records

***Tradescantia* (Commelinaceae)**

Spanish names: Hierba de Pollo; Hierba de pollo/siemprevive/natali/ siemprevive morado/madali; Hierba del pollo; Hoja de azar/tinta china; Lengua de pollo, tripa de pollo, siempre viva; Madali morado; Maguey morado; Maguey morado/ rojo; Matalí; Matlali; Moradito; Pabana; Siemprevive menuda/ hierba de pollo; Uña de Gato, Tripa de Gallina; Zabila morada
Indigenous names: Tzukin u /katzu o /katzu ay/tzapas o[01]; (Tzapas) tzui[01]; (Nama) tzui[01]; Tza'a tzoy[01]; Jam'ay[02]; Jäs'ukä potznä[02]; Tsabats uixpin[03]; Uky ujts[04]; Tsak utek', xutstun pathaam, mili' ch'ohool[07]; Paktha' utek', tsakam k'oyol, uto, yax tsamnek[07]; Chakts'am[09]; Ru Guaij a'k[12]; Loch'och[12]; Pitzijor[13]; Rak'eq'[13]; Ka'paj[14]; Asuseen itzuul[14]; Saqi' choq'l, Tz'i' Maaj Q'ehen[14]; Cha cha[15]; Chächäkpimi[18]; Yop'ixpätz[18]; Ak'ach k'opot[19]; Madali[21]; X-tòoz-pĕch, guìzh-pĕch , guìzh-guièe-pĕch, blàg-pĕch, x-tòoz-ngă[23]; Matlali[25]; Matlale[26]
Used by (17*): Zoque[01, 02, 03]; Mixe[04]; Huastec[07]; Yucatecan Maya[09, 10]; Quichean Maya[12, 13, 14, 15]; Western Maya[18, 19]; Zapotec[21, 23]; Nahua[25, 26]
Used for (103#): Blood[01]; Digestive[01, 02, 03, 07, 12, 13, 18, 21, 26]; Eye[01, 13]; Ear[01, 07]; Cardiovascular[02, 03, 14, 18, 26]; Musculoskeletal[01, 02, 10, 21]; Neurological[07, 12, 14, 18]; Psychological[01, 18]; Respiratory[01, 02, 03, 12, 18]; Skin[01, 02, 09, 12, 13, 14, 18, 21, 26]; Endocrine[01, 02, 14, 18]; Urological[01, 03, 04, 13, 18, 26]; Pregnancy[02, 13, 23]; Female genital[01, 02, 03, 15, 18, 21]; General and Unspecified[01, 02, 07, 12, 14, 18, 19, 21, 25, 26]
Cognates: MZ: uki/ukä; Zoq: uki/ukä; CoreM: cha/chak/chäk; Nah: matlali;
Language contact: MZ <> Maya; Chiapas Zoq <> Kekchí; Chimalapa Zoq <> Tabasco Chontal

***Tragia* (Euphorbiaceae)**

Spanish names:
Indigenous names: Tsay kenuk[03]; Tiplay', thinlay', chalam tiya[07]; P'op'ox[09]; P'ŏop'oŠ[10]
Used by (4*): Zoque[03]; Huastec[07]; Yucatecan Maya[09, 10]
Used for (6#): Musculoskeletal[07, 09, 10]; Skin[10]; Urological[03]; General and Unspecified[07]
Cognates: Yuc: p'op'ox;
Language contact:

***Trema* (Cannabaceae)**

Spanish names: Capulín; Majagua mixe, jonote
Indigenous names: Tzay kuy [01]; Puwaamte[07]; Lajsa'a baagui'[21]
Used by (3*): Zoque[01]; Huastec[07]; Zapotec[21]
Used for (3#): Skin[21]; Urological[01]; General and Unspecified[07]
Cognates:
Language contact:

***Trichilia* (Meliaceae)**

Spanish names: Cualimón, limoncillo; Palo cuchara; Tinajillo
Indigenous names: Yooni cuy[03]; Thokob saantu, k'olol te', thiblab[07]; Čabon Če[10]
Used by (5*): Zoque[03]; Totonac[05]; Huastec[07]; Yucatecan Maya[10]; Quichean Maya[13]
Used for (13#): Blood[03, 07]; Digestive[10]; Neurological[03, 07]; Psychological[07]; Respiratory[03, 07]; Pregnancy[03]; General and Unspecified[03, 05, 07, 13]
Cognates:
Language contact:

***Trichocentrum* (Orchidaceae)**

Spanish names:
Indigenous names: Xutsun buuru, ko'lom te'[07]; Puts'ubche', Bac chivo, Puts'maskab[09]; U'tsumpek[09]
Used by (2*): Huastec[07]; Yucatecan Maya[09]
Used for (3#): Neurological[07]; Skin[09]; Pregnancy[09]
Cognates: Maya: utsum/n;
Language contact:

***Trichomanes* (Hymenophyllacae)**

Spanish names:
Indigenous names: Chimal[03]
Used by (1*): Zoque[03]
Used for (1#): Neurological[03]
Cognates:
Language contact:

***Tridax* (Asteraceae)**

Spanish names: Hierba del toro; Hoja de azar
Indigenous names: Tza'a tzoy[01]; Wakax k'opot[19]
Used by (2*): Zoque[01]; Western Maya[19]
Used for (5#): Blood[19]; Musculoskeletal[19]; Neurological[19]; General and Unspecified[01, 19]
Cognates:
Language contact:

***Trifolium* (Fabaceae)**

Spanish names: Trebol
Indigenous names: Dĭp-rên, guiѐe-frôw[23]
Used by (2*): Zapotec[23]; Nahua[26]
Used for (2#): Cardiovascular[26]; General and Unspecified[23]
Cognates:
Language contact:

***Trigonella* (Fabaceae)**

Spanish names: Fenogreco
Indigenous names:
Used by (1*): Quichean Maya[12]
Used for (4#): Blood[12]; Digestive[12]; Skin[12]; General and Unspecified[12]
Cognates:
Language contact:

***Tripogandra* (Commelinaceae)**

Spanish names: Madali blanco; Matalillo; Matalin verde; Siemprevive roja; Tripa de tuza
Indigenous names: Tzapas tzui[01]; Tsus uixpin[03]; Kasmal[05]; A'kasman[06]; Utek witssil thakni', tsakam tsahib[07]; Ik B'olay Q'ehen[14]; Tzima'j k'ejen[17]; X-tòoz-pĕch, guìzh-pĕch , guìzh-guièe-pĕch, blàg-pĕch, x-tòoz-ngă[23]; Matlale morado[26]
Used by (10*): Zoque[01, 03]; Totonac[05, 06]; Huastec[07]; Quichean Maya[14, 17]; Zapotec[21, 23]; Nahua[26]
Used for (17#): Digestive[05, 06, 07]; Cardiovascular[26]; Musculoskeletal[14]; Psychological[01]; Skin[01, 07, 14, 21]; Endocrine[07]; Urological[26]; Pregnancy[23]; General and Unspecified[03, 07, 14]; nd[17]
Cognates: Tot: kasma;
Language contact:

***Triumfetta* (Malvaceae)**

Spanish names: Cadillo; Majagua blanca; Mozote
Indigenous names: Ueji ay[03]; Konko[03]; Thipaxi', pitaxe'[07]; Mul-och[09]; Kambapixoy[09]; Tzo kon[13]; Woyo'o pim[14]; Cuoyo'[17]; Yag lass[21]
Used by (9*): Zoque[01, 03]; Huastec[07]; Yucatecan Maya[09]; Quichean Maya[13, 14, 17]; Western Maya[20]; Zapotec[21]
Used for (21#): Digestive[01, 07, 09, 13, 14, 20]; Musculoskeletal[07]; Respiratory[07]; Skin[03, 21]; Urological[03, 07]; Pregnancy[07, 09, 14]; Female genital[09, 14]; General and Unspecified[03]; nd[17]
Cognates: Quich: woyo/cuoyo;
Language contact: Hua <> Yuc

Trixis **(Asteraceae)**

Spanish names: Contrimbruja
Indigenous names: Pub kw'ahiil, , thak ts'aah[07]; Fluxion xiw, Xtankas-ak'[09]
Used by (4*): Zoque[03]; Huastec[07]; Yucatecan Maya[09]; Zapotec[21]
Used for (9#): Digestive[07, 21]; Neurological[09]; Respiratory[07]; Skin[03, 07]; Pregnancy[07]; General and Unspecified[03, 07]
Cognates:
Language contact:

Tropaeolum **(Tropaeolaceae)**

Spanish names: Montwerz
Indigenous names: Màltuêrs[23]
Used by (2*): Zapotec[22, 23]
Used for (5#): Neurological[22, 23]; Respiratory[23]; General and Unspecified[22, 23]
Cognates:
Language contact:

Trophis **(Moraceae)**

Spanish names:
Indigenous names: Tsumte', chumte'[07]
Used by (1*): Huastec[07]
Used for (2#): Neurological[07]; General and Unspecified[07]
Cognates:
Language contact:

Turbina **(Convolvulaceae)**

Spanish names: Bejuco de viento; Flor de virgen ; Piule
Indigenous names: Atsay[03]; Pamaxuunk[04]; Xtabentun[09]; Badooj[21]; Zhì-ncuàan-ná-zhnâzh[23]
Used by (5*): Zoque[03]; Mixe[04]; Yucatecan Maya[09]; Zapotec[21, 23]
Used for (10#): Digestive[03]; Neurological[09]; Psychological[03, 23]; Respiratory[09]; Pregnancy[09]; General and Unspecified[03, 04, 21, 23]
Cognates:
Language contact:

Turnera **(Passifloraceae)**

Spanish names: Flor de linda tarde; Hoja de fiebre; Itamo real; Malva blanca; Oregano de monte; Salvia blanca
Indigenous names: Oregano k'ax[09]; Lexuuba'a quitzii[21]; Guish fiebre[21]; Salb nol[22]
Used by (6*): Zoque[02, 03]; Yucatecan Maya[09]; Zapotec[21, 22]; Nahua[27]
Used for (10#): Digestive[21, 22, 27]; Respiratory[09]; Skin[02, 03, 21]; Pregnancy[21]; General and Unspecified[03, 21]
Cognates:
Language contact:

Ulmus **(Ulmaceae)**

Spanish names: Palo cuero
Indigenous names: Pagaxniakcuy[03]
Used by (1*): Zoque[03]
Used for (1#): Pregnancy[03]
Cognates:
Language contact:

***Urera* (Urticaceae)**

Spanish names: Chichicaste; Chichicaste de Caballo; Ortiga; Ortiga real; Ortiga, chichicaste; Ortiga, pica-pica
Indigenous names: Aaamlay', pulik pux lat'em[07]; Tsooklay', choklay'[07]; Laal[09]; Lej', Läjy, Yel[12]; Ley'[12]; La'[13]; Oqob' Q'ehen[14]
Used by (8*): Zoque[03]; Huastec[07]; Yucatecan Maya[09, 11]; Quichean Maya[12, 13, 14]; Nahua[25]
Used for (22#): Digestive[09]; Eye[07]; Musculoskeletal[03, 07, 09, 11, 12, 13, 25]; Neurological[09]; Psychological[07]; Respiratory[07, 14, 25]; Skin[03]; Urological[12]; General and Unspecified[07, 11, 12]
Cognates: Maya: la/lay/ley;
Language contact:

***Urtica* (Urticaceae)**

Spanish names: Ortiga; Ortiga menor/ bejucillo; Ortiga roja
Indigenous names: Menuk/ kenuk[01]; La'ix iits', tsakam tsooklay'[07]; Ley' pi'y[12]
Used by (3*): Zoque[01]; Huastec[07]; Quichean Maya[12]
Used for (12#): Blood[12]; Cardiovascular[12]; Musculoskeletal[01, 07]; Neurological[12]; Psychological[12]; Skin[12]; Urological[01, 12]; Female genital[01]; General and Unspecified[12]
Cognates: Maya: lai/ley;
Language contact:

***Urvillea* (Sapindaceae)**

Spanish names:
Indigenous names: Tsaayleelaab ts'ohool, itsaan an t'in kamaab, tsank'ub ts'aah[07]
Used by (1*): Huastec[07]
Used for (2#): Neurological[07]; General and Unspecified[07]
Cognates:
Language contact:

***Valeriana* (Caprifoliaceae)**

Spanish names: Bejuco silvestre, hierba de perrito; Hierba azar de guía/gua blanca; Valeriana; Valeriana Silvestre
Indigenous names: Po'o sudan/ poye rane / Nakta[01]; Puua ay, Puutx ay[03]; Uxe' ulew[13]; Guìzh-ngùd-lèn̲, ngùd-lèn̲, gù-vàlèriân[23]; Tetziltzi, tlacote[26]
Used by (6*): Zoque[01, 03]; Quichean Maya[12, 13]; Zapotec[23]; Nahua[26]
Used for (27#): Digestive[01, 12, 23]; Cardiovascular[12]; Musculoskeletal[01, 12, 23]; Neurological[12, 13]; Psychological[01, 12, 13]; Skin[01, 03, 13, 26]; Endocrine[23]; Urological[12]; Female genital[12, 23]; General and Unspecified[01, 03, 12, 13, 23]
Cognates: Zoq: po'o/puua;
Language contact:

***Vanilla* (Orchidaceae)**

Spanish names: Vainilla
Indigenous names: Suumii'xa'nat[06]
Used by (3*): Mixe[04]; Totonac[06]; Zapotec[21]
Used for (4#): Skin[06]; Pregnancy[06]; General and Unspecified[04, 21]
Cognates:
Language contact:

***Vasconcellea* (Caricaceae)**

Spanish names: Papaya cimarrona; Papaya de Montaña; Papaya simarron
Indigenous names: Otzo'[01]; Mäki mama[02]; Tutun 'chich, tatan chich[06]; Q'ilx Q'en[14]
Used by (4*): Zoque[01, 02]; Totonac[06]; Quichean Maya[14]
Used for (7#): Digestive[01, 02, 06, 14]; Cardiovascular[14]; Neurological[02]; Respiratory[06]
Cognates:
Language contact:

01-28 refer to the studies described in Table 4.1 * Total number of studies recording this taxon #Total number of use-records

***Vatairea* (Fabaceae)**

Spanish names: Palo picho
Indigenous names: Y¥k cuy[03]
Used by (1*): Zoque[03]
Used for (1#): Digestive[03]
Cognates:
Language contact:

***Vellozia* (Velloziaceae)**

Spanish names:
Indigenous names: Susm kik[14]
Used by (1*): Quichean Maya[14]
Used for (1#): Neurological[14]
Cognates:
Language contact:

***Verbascum* (Scrophulariaceae)**

Spanish names: Oreja de Burro, Hoja Santa
Indigenous names:
Used by (1*): Quichean Maya[12]
Used for (9#): Blood[12]; Digestive[12]; Neurological[12]; Respiratory[12]; Skin[12]; Urological[12]; Female genital[12]; General and Unspecified[12]
Cognates:
Language contact:

***Verbena* (Verbenaceae)**

Spanish names: Diente de ratón; Hierba buenilla, moradita, ; Pitiona; Verbena
Indigenous names: Tung'an petkuy/kan petkuy/tak'an petkuy/tzitzirane/ takak rane/ tuk tane [01]; Ejtil i kwayab ts'aale[08]; Chachal' b'ey[12]; Uchachal be', werwen, chachal be'[13]; Xkis Kawaay [14]; Pem k'ulub, yakan k'ulub wamal, yaxal tob tz'i'lel, yax uran nich momol[20]; Pem k'ulub, yakan k'ulub wamal[20]; Ptiôn[23]; Ptiôn-dán, guìzh-ptiôn, guièe-ptiôn, bít-wĩ[23]; Ohpanchichic[26]; Clanquimiche[26]; Ndinich[28]
Used by (13*): Zoque[01]; Totonac[05]; Huastec[08]; Quichean Maya[12, 13, 14]; Western Maya[18, 19, 20]; Zapotec[21, 23]; Nahua[26, 28]
Used for (56#): Blood[05, 12]; Digestive[01, 05, 08, 12, 13, 18, 19, 20, 21, 23, 26, 28]; Musculoskeletal[01, 12, 13, 14, 19, 26]; Neurological[12, 13, 14]; Psychological[01, 12, 23]; Respiratory[12, 13, 18, 20]; Skin[01, 14]; Endocrine[01]; Urological[01, 12]; Pregnancy[01, 13, 26]; Female genital[12, 14]; General and Unspecified[01, 12, 13, 14, 18, 19, 20, 26, 28]
Cognates: Quich: chachal be;
Language contact:

***Verbesina* (Asteraceae)**

Spanish names: Arnica; Bordón de vieja; Capitanec, Capitaneja; Chimpfloque; Huichim; Huixin; Machichili; Paletaria
Indigenous names: Oko kaku[02]; Copanteñicuy[03]; Witsiim, wichin[07]; Taxiwua[08]; Chulkeeh[09]; Axocopa[26]
Used by (11*): Zoque[01, 02, 03]; Totonac[05, 06]; Huastec[07, 08]; Yucatecan Maya[09]; Quichean Maya[12]; Nahua[26, 27]
Used for (33#): Digestive[01, 03, 05, 06, 07, 12]; Ear[07]; Musculoskeletal[01, 05, 07, 12]; Neurological[07]; Respiratory[09, 27]; Skin[01, 02, 03, 05, 06, 07, 26, 27]; Endocrine[05, 06]; Urological[05]; Pregnancy[06, 26, 27]; General and Unspecified[05, 07, 08, 12, 27]
Cognates:
Language contact:

***Vernonanthura* (Asteraceae)**

Spanish names: Hoja de canela; Suquinay
Indigenous names: Ux cuy[03]; Suquinay[13]; Ti'tzin'[13]; Suquinay[14]; Holobob te'[17]; Bik'tal sitit[20]; Gui'xaan[21]
Used by (6*): Zoque[03]; Quichean Maya[13, 14, 17]; Western Maya[20]; Zapotec[21]
Used for (13#): Digestive[13, 20, 21]; Eye[03]; Ear[03]; Musculoskeletal[13, 21]; Pregnancy[21]; Female genital[03, 13]; General and Unspecified[14]; nd[17]
Cognates:
Language contact:

***Vernonia* (Asteraceae)**

Spanish names:
Indigenous names: Xihuatahua[05]; Suquinay, sucunay, ub'i te[19]
Used by (3*): Totonac[05]; Quichean Maya[14]; Western Maya[19]
Used for (3#): Digestive[19]; Respiratory[05]; General and Unspecified[14]
Cognates:
Language contact:

***Veronica* (Plantaginaceae)**

Spanish names: Trebolillo azul
Indigenous names: Lotz Q'eq'oj Aq'om[12]
Used by (1*): Quichean Maya[12]
Used for (9#): Blood[12]; Digestive[12]; Eye[12]; Musculoskeletal[12]; Neurological[12]; Psychological[12]; Respiratory[12]; Skin[12]; General and Unspecified[12]
Cognates:
Language contact:

***Viburnum* (Adoxaceae)**

Spanish names: Hoja verde
Indigenous names:
Used by (2*): Zoque[01]; Quichean Maya[13]
Used for (2#): Digestive[01]; Skin[13]
Cognates:
Language contact:

***Vicia* (Fabaceae)**

Spanish names: Visia Belluda
Indigenous names:
Used by (1*): Quichean Maya[12]
Used for (3#): Blood[12]; Urological[12]; General and Unspecified[12]
Cognates:
Language contact:

***Vigna* (Fabaceae)**

Spanish names: Flor de guajolote; Frijol torito, frijol tripa de tuza
Indigenous names: X'pal'lihua s'aca[06]
Used by (2*): Totonac[06]; Zapotec[21]
Used for (2#): Skin[06]; General and Unspecified[21]
Cognates:
Language contact:

***Viguiera* (Asteraceae)**

Spanish names: Azaján; Chimalacate
Indigenous names: Thapil bichim[07]; Tah[10]; Qän Kewoj Achin[12]
Used by (4*): Huastec[07]; Yucatecan Maya[10]; Quichean Maya[12]; Nahua[27]
Used for (9#): Musculoskeletal[12]; Skin[07, 10, 27]; Urological[12]; Pregnancy[27]; Male genital[12]; General and Unspecified[10, 12]
Cognates: Maya: tha/tah;
Language contact:

Vinca **(Apocynaceae)**

Spanish names: Vincapervinca
Indigenous names:
Used by (1*): Quichean Maya[12]
Used for (3#): Cardiovascular[12]; Urological[12]; General and Unspecified[12]
Cognates:
Language contact:

Viola **(Violaceae)**

Spanish names: Trebol real
Indigenous names: Trebo'l Aq'om[12]
Used by (1*): Quichean Maya[12]
Used for (7#): Blood[12]; Digestive[12]; Neurological[12]; Psychological[12]; Respiratory[12]; Skin[12]; General and Unspecified[12]
Cognates:
Language contact:

Virola **(Myristicaceae)**

Spanish names: Cedrillo
Indigenous names: J¥mniom cas[03]
Used by (1*): Zoque[03]
Used for (2#): Musculoskeletal[03]; Skin[03]
Cognates:
Language contact:

Vismia **(Hypericaceae)**

Spanish names: Cayamita; Huacalilla, lecherilla
Indigenous names: Q'an parwuay, Qan' paraway[14]; K'anparaquay che'[17]
Used by (4*): Zoque[03]; Quichean Maya[14, 17]; Nahua[26]
Used for (7#): Digestive[03, 14]; Skin[03, 26]; Urological[03]; Female genital[03]; nd[17]
Cognates: Quich: para(q)way;
Language contact:

Vitex **(Lamiaceae)**

Spanish names: Beo; Ceresa negra; Coyotomate
Indigenous names: Jäk'yi'äye[02]; Ya'axnik[09]; YaŠnik[10]; Boyajsa'a[21]; Cuahuilotl[28]
Used by (5*): Zoque[02]; Yucatecan Maya[09, 10]; Zapotec[21]; Nahua[28]
Used for (8#): Digestive[02]; Musculoskeletal[02]; Respiratory[02, 21, 28]; Skin[10, 28]; General and Unspecified[09]
Cognates: Yuc: yaxnik;
Language contact: Yuc > Chimalapa Zoq & Zap

Vitis **(Vitaceae)**

Spanish names: Agras; Bejuco de uva; Siete corazon, parra; Totoloche; Uva; Uva de Montaña ; Uva silvestre; Uva/bejuco negro/uva silvestre/ corraleña
Indigenous names: ku'untu/ku'untatz ay[01]; Po'on tzu'u[02]; Mopst¥m[03]; Y¥k ty¥m tsay[03]; Snuun'gut[06]; T'uthub[07]; Tusúp'[13]; Tu'sub' Kaham'[14]; T'u zub k'aham[17]; Chuyur ch'a'n[19]
Used by (10*): Zoque[01, 02, 03]; Totonac[06]; Huastec[07]; Quichean Maya[12, 13, 14, 17]; Western Maya[19]
Used for (28#): Digestive[02, 03, 13]; Eye[01, 02, 03, 07, 12, 19]; Ear[01]; Cardiovascular[06]; Neurological[07]; Respiratory[03]; Skin[03]; Endocrine[01, 12]; Urological[03]; Pregnancy[07, 14]; Female genital[03]; Male genital[01, 14]; General and Unspecified[01, 07, 12]; nd[17]
Cognates: Zoq: u'untu/o'ontzu; Maya: t'uthub/t'usub; Quich: tusup (kaham);
Language contact: Mayan <> Tot

***Vittaria* (Pteridaceae)**

Spanish names:
Indigenous names: Rubel xsa' kanti[14]
Used by (1*): Quichean Maya[14]
Used for (1#): Skin[14]
Cognates:
Language contact:

***Volkameria* (Lamiaceae)**

Spanish names: Moste
Indigenous names: Muts[03]
Used by (1*): Zoque[03]
Used for (3#): Neurological[03]; Psychological[03]; General and Unspecified[03]
Cognates:
Language contact:

***Waltheria* (Malvaceae)**

Spanish names: Malvarisco amarillo
Indigenous names: Pun¥g ay[03]; Piniaka[03]; Alshoob nagatzi'[21]
Used by (2*): Zoque[03]; Zapotec[21]
Used for (7#): Digestive[03, 21]; Psychological[03]; Skin[21]; Urological[03]; Pregnancy[21]; Female genital[03]
Cognates:
Language contact:

***Wedelia* (Asteraceae)**

Spanish names: Orozus
Indigenous names: Sahun[09]
Used by (2*): Yucatecan Maya[09, 10]
Used for (2#): Neurological[10]; Skin[09]
Cognates:
Language contact:

***Wigandia* (Boraginaceae)**

Spanish names: Chichicaste, Chacom, Chocon; Chocón; Hoja de San Pablo
Indigenous names: Blàg-wê[23]
Used by (3*): Quichean Maya[12, 13]; Zapotec[23]
Used for (11#): Digestive[13]; Cardiovascular[12]; Neurological[12]; Psychological[12]; Respiratory[13, 23]; Skin[12, 23]; Pregnancy[12]; General and Unspecified[12, 23]
Cognates:
Language contact:

***Wimmeria* (Celastraceae)**

Spanish names: Canserina
Indigenous names:
Used by (1*): Zoque[03]
Used for (3#): Digestive[03]; Pregnancy[03]; Female genital[03]
Cognates:
Language contact:

***Wissadula* (Malvaceae)**

Spanish names:
Indigenous names: Puk ay[03]; J¥mniom cuy puk[03]
Used by (1*): Zoque[03]
Used for (2#): Skin[03]; General and Unspecified[03]
Cognates:
Language contact:

***Witheringia* (Solanaceae)**

Spanish names: Hoja de sapo
Indigenous names: Kunya[01]; Naj ka bokste/tziski boni rane[01]
Used by (1*): Zoque[01]
Used for (4#): Musculoskeletal[01]; Skin[01]; Endocrine[01]; General and Unspecified[01]
Cognates:
Language contact:

***Xanthosoma* (Araceae)**

Spanish names: Mafafa blanca; Mafafa morada; Malanga Silvestre; Quequeste; Tepesquite
Indigenous names: Poko[01]; Pix ay[03]; Tujk aay[04]; Paxni'cac[06]; Ts'ikiy luum[07]; Luum[07]; Xtup'[13]; Marak [14]; Biu'ulú[21]
Used by (8*): Zoque[01, 03]; Mixe[04]; Totonac[06]; Huastec[07]; Quichean Maya[13, 14]; Zapotec[21]
Used for (18#): Blood[06]; Musculoskeletal[01, 21]; Respiratory[01]; Skin[01, 03, 04, 06, 07, 13, 14, 21]; Pregnancy[06, 13]; General and Unspecified[07, 14, 21]
Cognates:
Language contact: Zoq <> Tot

***Ximenia* (Olacaceae)**

Spanish names:
Indigenous names: Paja pitx cuy[03]; Ma'ap ujts[04]; Nabche', Tsu'tsup[09]
Used by (3*): Zoque[03]; Mixe[04]; Yucatecan Maya[09]
Used for (4#): Digestive[03, 09]; Female genital[04]; General and Unspecified[03]
Cognates:
Language contact: Mixe <> Yuc

***Xiphidium* (Haemodoraceae)**

Spanish names:
Indigenous names: X cua' li k'uch[17]
Used by (1*): Quichean Maya[17]
Used for (1#): nd[17]
Cognates:
Language contact:

***Xylosma* (Salicaceae)**

Spanish names: Chatay; Espina de brujo/espina de cruz
Indigenous names: Yatzi apit/cruz apit[01]; Jaman juix apitx[03]; Tsak k'iith, wi' chunun[07]; Puts'ukche', Xchaknif[09]
Used by (6*): Zoque[01, 03]; Totonac[05, 06]; Huastec[07]; Yucatecan Maya[09]
Used for (17#): Digestive[06, 07]; Musculoskeletal[01, 07]; Respiratory[05, 06, 07]; Skin[03, 07, 09]; Endocrine[06]; Urological[06]; Female genital[03]; General and Unspecified[01, 03, 07]
Cognates: Zoq: apit(x);
Language contact:

Yucca **(Asparagaceae)**

Spanish names: Equizote, yucca; Izote; Maguey silvestre
Indigenous names: A'ka'lokot[06]; Tsamnek k'oyol, chemnek k'oyol[07]; K'oyol[07]; Tuk[09]; Oq'ki[13]; Sit'a'[19]; Ikzote[26]
Used by (9*): Zoque[01, 03]; Totonac[06]; Huastec[07]; Yucatecan Maya[09]; Quichean Maya[12, 13]; Western Maya[19]; Nahua[26]
Used for (25#): Ear[06, 07, 12, 13, 19, 26]; Cardiovascular[01]; Musculoskeletal[12]; Neurological[12, 13, 19]; Psychological[09]; Respiratory[12, 13, 19]; Skin[26]; Endocrine[03, 12]; Pregnancy[07]; Male genital[12]; General and Unspecified[07, 12, 19]
Cognates:
Language contact:

Zaluzania **(Asteraceae)**

Spanish names: Hierba amarga
Indigenous names: Chichitzihuatl[25]
Used by (1*): Nahua[25]
Used for (2#): Digestive[25]; Pregnancy[25]
Cognates:
Language contact:

Zamia **(Zamiaceae)**

Spanish names: Mais Viejo
Indigenous names: Pekmuk kobak[03]; J¥mniom pekmuk[03]; Møj mook[04]; Tsalam thipaak, tsakam way', ahaatik a eem[07]; Q'ixtab[14]; Ox Q'en[14]; Sikad[17]
Used by (5*): Zoque[03]; Mixe[04]; Huastec[07]; Quichean Maya[14, 17]
Used for (16#): Blood[14]; Digestive[03, 07, 14]; Musculoskeletal[07]; Skin[03, 14]; Endocrine[07]; Urological[03, 07]; Female genital[03]; Male genital[03]; General and Unspecified[04, 07, 14]; nd[17]
Cognates: MZ: mook/muk;
Language contact:

Zanthoxylum **(Rutaceae)**

Spanish names: Limoncillo; Mata de escorpión; Tachuelillo; Zorrillo cimarrón, zorrillo bravo, zorrillo de castilla, sasafrás de espina
Indigenous names: Apitx cuy[03]; Copixpix[03]; Wi'puuy[07]; Sinanche'[09]; Sina'an ché[11]; Tujen a'uch cimarrón[18]; Leben sotz'[20]
Used by (7*): Zoque[03]; Huastec[07]; Yucatecan Maya[09, 11]; Quichean Maya[17]; Western Maya[18, 20]
Used for (21#): Blood[03]; Digestive[03, 07, 20]; Musculoskeletal[07, 11, 18]; Neurological[07, 09]; Respiratory[03, 07]; Skin[03, 18]; Urological[03]; Pregnancy[07]; Female genital[03]; General and Unspecified[07, 09, 11, 18]; nd[17]
Cognates: Yuc: sinanche;
Language contact:

Zapoteca **(Fabaceae)**

Spanish names: Barba de chivo
Indigenous names: Tenzonhuaxi[26]
Used by (2*): Zoque[03]; Nahua[26]
Used for (2#): Skin[03]; General and Unspecified[26]
Cognates:
Language contact:

***Zea* (Poaceae)**

Spanish names: Cabello de elote; Maís; Maïs, Cabello de elote, Atole, Pinole; Maiz; Maíz, barba de maíz, totomosle morado; Maíz/tortomosle morado/granos de maíz/pelo de elote; Pelo de elote/elote morado/maíz morado/cabello de maz/ colochi morado
Indigenous names: Mok owai/ japinjoke/ mojkuy su yaj/ tzapas tokok/ tzapas oksi/ tzapas äksi[01]; Mok/u'kä japo, äksi, mok angwa'y/mok puj[02]; Mok[03]; Mook, mook kuay, [04]; Cuxi[05]; Cuxi[06]; Eem, ithith[07]; Eem[08]; IŠim[10]; Ixim[12]; Ixim, jal, wi jal, uwijal, tzmi'y[13]; Ixim[18]; Ixim, nar[19]; Xhuba'a, bacuejlu'u moradu'u[21]; Žob[22]; Zhǒb, dòz, wgà, pcuêl[23]; Tlaoli[28]
Used by (20*): Zoque[01, 02, 03]; Mixe[04]; Totonac[05, 06]; Huastec[07, 08]; Yucatecan Maya[09, 10]; Quichean Maya[12, 13, 14]; Western Maya[18, 19]; Zapotec[21, 22, 23]; Nahua[25, 28]
Used for (71#): Digestive[01, 02, 04, 05, 07, 08, 09, 12, 13, 14, 18, 21, 28]; Eye[06]; Musculoskeletal[01, 07, 08, 18]; Neurological[12]; Psychological[07]; Respiratory[02, 07, 23, 28]; Skin[01, 02, 03, 07, 21]; Endocrine[01, 07, 08, 09, 12, 18]; Urological[01, 02, 03, 04, 06, 07, 08, 09, 10, 12, 13, 18, 19, 21, 25, 28]; Pregnancy[01, 03, 22, 23]; Female genital[02, 03, 10]; Male genital[01, 12, 13]; General and Unspecified[01, 02, 07, 08, 12, 13, 21, 22, 28]
Cognates: MZ: mok, mok wai; Zoq: mok, äksi; Tot: cuxi; Maya: ixim/ithith; Hua: eem; CoreM: ixim; Quich: ixim; Zap: zhob;
Language contact:

***Zephyranthes* (Amaryllidaceae)**

Spanish names: Brujita; Cebolla de Zopilote
Indigenous names: Xnaket Q'os[12]; Ajtz'a 'taya[18]
Used by (2*): Quichean Maya[12]; Western Maya[18]
Used for (4#): Ear[18]; Endocrine[12]; Urological[12]; General and Unspecified[12]
Cognates:
Language contact:

***Zexmenia* (Asteraceae)**

Spanish names:
Indigenous names:
Used by (1*): Zoque[03]
Used for (1#): Skin[03]
Cognates:
Language contact:

***Zingiber* (Zingiberaceae)**

Spanish names: Ajenjible; Gengibre; Genjibre; Jengibre; Jenjibre
Indigenous names: Kaxtxan ñiwi[03]; Caxta'lam pin[06]; Laab ist[07]; Xan Xir[14]; Xinxibeer[15]; Chilcamotle[26]
Used by (12*): Zoque[01, 03]; Totonac[06]; Huastec[07]; Yucatecan Maya[09]; Quichean Maya[12, 13, 14, 15]; Western Maya[19]; Zapotec[21]; Nahua[26]
Used for (35#): Digestive[01, 03, 06, 07, 09, 12, 14]; Musculoskeletal[03, 06, 07, 13, 21]; Neurological[06, 12, 26]; Psychological[06, 12]; Respiratory[01, 07, 14, 21]; Skin[03, 12, 21]; Endocrine[01]; Pregnancy[06, 19]; Female genital[01, 03, 15, 19]; General and Unspecified[12, 15, 21, 26]
Cognates:
Language contact:

***Zinnia* (Asteraceae)**

Spanish names: Clavenia; Molinillo
Indigenous names:
Used by (2*): Zoque[03]; Quichean Maya[12]
Used for (2#): Neurological[03]; Psychological[12]
Cognates:
Language contact:

***Ziziphus* (Rhamnaceae)**

Spanish names: Pendeno
Indigenous names:
Used by (1*): Zapotec[21]
Used for (1#): Female genital[21]
Cognates:
Language contact:

01-28 refer to the studies described in Table 4.1   * Total number of studies recording this taxon   #Total number of use-records

***Zornia* (Fabaceae)**

Spanish names:
Indigenous names: Tsaiñiay[03]; Naax wiin aats[04]
Used by (2*): Zoque[03]; Mixe[04]
Used for (2#): Digestive[04]; Skin[03]
Cognates:
Language contact:

***Zuelania* (Salicaceae)**

Spanish names:
Indigenous names: Bolantin, tatham te'[07]; Tamay, Bot'ox[09]
Used by (2*): Huastec[07]; Yucatecan Maya[09]
Used for (3#): Eye[07]; Neurological[07]; Female genital[09]
Cognates:
Language contact:

01-28 refer to the studies described in Table 4.1 * Total number of studies recording this taxon #Total number of use-records
