# Supplementary material for: Traditional Herbal Medicine in Mesoamerica: Toward Its Evidence Base for Improving Universal Health Coverage
Source: Front Pharmacol. 2020 Jul 31;11:1160. doi: 10.3389/fphar.2020.01160 (PMC7411306; doi:10.3389/fphar.2020.01160)
Supplement: Supplementary file 3 [file DataSheet_3.pdf]

Table A4.3.
List of the 185 botanical families used medicinally in Mesoamerica. The families are rank-ordered on number of use-records.

Families	Number of use-records	Number of studies citing	Number of species	Number of genera
**Asteraceae**	266	28	226	117
**Lamiaceae**	216	28	79	31
**Fabaceae**	201	26	194	74
**Rutaceae**	195	27	25	8
**Solanaceae**	193	27	68	14
**Euphorbiaceae**	180	26	85	16
**Malvaceae**	164	27	76	39
**Verbenaceae**	137	26	31	12
**Rubiaceae**	125	25	66	30
**Lauraceae**	125	22	17	7
**Piperaceae**	124	28	43	2
**Myrtaceae**	122	26	24	9
**Poaceae**	118	24	35	31
**Bignoniaceae**	115	21	28	17
**Apiaceae**	107	20	28	17
**Acanthaceae**	95	21	43	16
**Cucurbitaceae**	94	23	31	20
**Apocynaceae**	94	24	41	23
**Urticaceae**	94	19	22	10
**Rosaceae**	92	16	24	10
**Commelinaceae**	91	21	23	7
**Amaryllidaceae**	89	18	12	4
**Asphodelaceae**	88	17	2	1
**Anacardiaceae**	87	23	18	13
**Amaranthaceae**	85	24	27	10
**Burseraceae**	77	21	7	2
**Annonaceae**	77	17	14	5
**Cactaceae**	76	18	22	12
**Boraginaceae**	76	19	25	10
**Asparagaceae**	73	19	24	10
**Meliaceae**	72	17	9	6
**Adoxaceae**	71	16	3	2

Families	Number of use-records	Number of studies citing	Number of species	Number of genera
**Passifloraceae**	64	20	19	2
**Crassulaceae**	63	17	15	5
**Nyctaginaceae**	60	20	14	6
**Scrophulariaceae**	59	20	8	3
**Brassicaceae**	59	14	11	7
**Phytolaccaceae**	57	15	6	4
**Melastomataceae**	56	14	27	12
**Plantaginaceae**	56	16	13	9
**Malpighiaceae**	56	18	10	8
**Convolvulaceae**	56	19	25	8
**Aristolochiaceae**	54	13	15	1
**Polypodiaceae**	54	14	19	8
**Bixaceae**	53	14	2	2
**Lythraceae**	52	16	13	6
**Arecaceae**	51	15	14	9
**Araceae**	51	15	27	9
**Salicaceae**	46	14	12	7
**Onagraceae**	43	15	11	5
**Moraceae**	42	15	17	7
**Pinaceae**	42	11	5	2
**Zingiberaceae**	42	13	6	4
**Papaveraceae**	41	16	3	2
**Smilacaceae**	41	13	8	1
**Bromeliaceae**	37	15	16	8
**Musaceae**	37	15	3	1
**Pteridaceae**	37	12	23	10
**Vitaceae**	37	16	10	3
**Sapotaceae**	36	14	7	4
**Oxalidaceae**	35	13	8	3
**Polygonaceae**	35	12	20	9
**Fagaceae**	35	9	11	1
**Begoniaceae**	35	12	7	1
**Sapindaceae**	34	12	19	11
**Equisetaceae**	34	14	3	1

Families	Number of use-records	Number of studies citing	Number of species	Number of genera
**Cyperaceae**	30	13	15	5
**Siparunaceae**	29	7	3	1
**Menispermaceae**	28	15	8	5
**Loranthaceae**	28	10	10	4
**Costaceae**	27	13	5	1
**Caricaceae**	27	15	2	2
**Primulaceae**	26	7	13	5
**Caprifoliaceae**	25	6	5	1
**Combretaceae**	24	9	4	2
**Orchidaceae**	24	10	20	18
**Rhamnaceae**	23	13	11	9
**Dioscoreaceae**	22	13	9	1
**Magnoliaceae**	22	7	3	1
**Celastraceae**	21	7	7	4
**Ranunculaceae**	21	9	6	3
**Cupressaceae**	21	8	4	4
**Marantaceae**	20	9	8	3
**Gesneriaceae**	19	7	10	7
**Ericaceae**	19	8	7	6
**Selaginellaceae**	19	11	6	1
**Muntingiaceae**	18	7	1	1
**Myricaceae**	18	6	1	1
**Lygodiaceae**	18	9	3	1
**Oleaceae**	17	8	9	4
**Simaroubaceae**	17	8	4	4
**Campanulaceae**	17	7	5	3
**Cannabaceae**	17	8	4	4
**Violaceae**	17	7	5	2
**Altingiaceae**	16	7	2	1
**Santalaceae**	16	8	6	1
**Portulacaceae**	15	7	2	1
**Polemoniaceae**	15	5	4	1
**Martyniaceae**	15	6	1	1
**Zamiaceae**	15	6	5	2

Families	Number of use-records	Number of studies citing	Number of species	Number of genera
**Myristicaceae**	15	3	2	2
**Araliaceae**	14	5	3	3
**Phyllanthaceae**	14	5	6	1
**Tectariaceae**	14	8	3	2
**Hypericaceae**	13	7	8	2
**Polygalaceae**	13	8	6	3
**Basellaceae**	13	8	3	1
**Schisandraceae**	12	8	1	1
**Pentaphylacaceae**	12	4	4	1
**Cistaceae**	12	3	1	1
**Betulaceae**	12	4	4	2
**Ebenaceae**	12	6	4	1
**Dryopteridaceae**	11	6	7	4
**Cyatheaceae**	11	3	3	1
**Alstroemeriaceae**	11	5	2	1
**Geraniaceae**	11	5	4	2
**Juglandaceae**	11	4	3	1
**Clusiaceae**	11	5	4	3
**Caryophyllaceae**	11	5	3	2
**Chrysobalanaceae**	10	6	5	4
**Moringaceae**	10	4	1	1
**Cannaceae**	10	6	1	1
**Lentibulariaceae**	8	2	2	1
**Linaceae**	8	4	1	1
**Actinidiaceae**	8	1	1	1
**Talinaceae**	8	4	2	1
**Dilleniaceae**	8	5	3	3
**Capparaceae**	8	4	4	3
**Balsaminaceae**	8	4	2	1
**Nephrolepidaceae**	7	5	3	1
**Plumbaginaceae**	7	4	1	1
**Monimiaceae**	7	4	3	2
**Orobanchaceae**	7	3	2	2
**Loasaceae**	7	5	4	2

Families	Number of use-records	Number of studies citing	Number of species	Number of genera
**Dennstaedtiaceae**	7	3	1	1
**Zygophyllaceae**	6	3	4	3
**Heliconiaceae**	6	5	5	1
**Proteaceae**	6	2	3	3
**Iridaceae**	6	4	5	4
**Picramniaceae**	6	5	4	2
**Pontederiaceae**	5	2	2	2
**Pedaliaceae**	5	3	1	1
**Platanaceae**	5	2	1	1
**Tropaeolaceae**	5	2	1	1
**Gentianaceae**	4	3	2	2
**Cleomaceae**	4	2	2	1
**Loganiaceae**	4	4	2	2
**Olacaceae**	4	3	1	1
**Icanicaceae**	4	1	1	1
**Aspleniaceae**	4	1	1	1
**Ginkgoaceae**	4	1	1	1
**Nymphaeaceae**	4	3	1	1
**Connaraceae**	4	1	1	1
**Krameriaceae**	3	1	1	1
**Clethraceae**	3	2	2	1
**Schoepfiaceae**	3	1	1	1
**Lytrhraceae**	3	1	0	0
**Buxaceae**	3	1	1	1
**Theaceae**	3	1	1	1
**Thelypteridaceae**	3	4	3	2
**Podostemaceae**	3	1	1	1
**Anemiaceae**	2	2	3	1
**Blechnaceae**	2	1	1	1
**Cabombaceae**	2	1	1	1
**Marchantiaceae**	2	1	1	1
**Melanthiaceae**	2	2	1	1
**Stegnospermataceae**	2	1	1	1
**Molluginaceae**	1	1	1	1

Families	Number of use-records	Number of studies citing	Number of species	Number of genera
**Tetrachondraceae**	1	1	1	1
**Winteraceae**	1	1	1	1
**Cytinaceae**	1	1	1	1
**Erythroxylaceae**	1	1	1	1
**Fouquieriaceae**	1	1	1	1
**Hydrangeaceae**	1	1	1	1
**Hypoxidaceae**	1	1	1	1
**Lycopodiaceae**	1	1	1	1
**Phrymaceae**	1	1	1	1
**Osmundaceae**	1	1	1	1
**Hymenophyllacae**	1	1	1	1
**Ulmaceae**	1	1	1	1
**Velloziaceae**	1	1	1	1
**Marattiaceae**		1	1	1
**Dicksoniaceae**		1	1	1
**Marcgraviaceae**		1	1	1
**Haemodoraceae**		1	1	1
